# Gene Expression in the Hippocampus in a Rat Model of Premenstrual Dysphoric Disorder After Treatment With Baixiangdan Capsules

**DOI:** 10.3389/fpsyg.2018.02065

**Published:** 2018-11-13

**Authors:** Sheng Wei, Peng Sun, Yinghui Guo, Jingxuan Chen, Jieqiong Wang, Chunhong Song, Zifa Li, Ling Xue, Mingqi Qiao

**Affiliations:** ^1^Lab of Traditional Chinese Medicine Classical Theory, Ministry of Education, Shandong University of Traditional Chinese Medicine, Jinan, China; ^2^Behavioral Phenotyping Core Facility, Shandong University of Traditional Chinese Medicine, Jinan, China

**Keywords:** premenstrual dysphoric disorder, gene chip, Baixiangdan, differentially expressed genes, gene ontology, KEGG, signal pathways, traditional Chinese medicine

## Abstract

**Objective:** To explore the targets, signal regulatory networks and mechanisms involved in Baixiangdan (BXD) capsule regulation of premenstrual dysphoric disorder (PMDD) at the gene transcription level, since the etiology and pathogenesis of PMDD are not well understood.

**Methods:** The PMDD rat model was prepared using the resident-intruder paradigm. The rats were tested for aggressive behavior, and those with scores in the lowest 30% were used as controls, while rats with scores in the highest 30% were divided into a PMDD model group, BXD administration group and fluoxetine administration group, which were evaluated with open-field tests and aggressive behavior tests. We also analyzed gene expression profiles in the hippocampus for each group, and verified differential expression of genes by real-time PCR.

**Results:** Before and after BXD or fluoxetine administration, scores in the open-field test exhibited no significant differences. The aggressive behavior of the PMDD model rats was improved to a degree after administration of both substances. Gene chip data indicated that 715 genes were differentially expressed in the control and BXD groups. Other group-to-group comparisons exhibited smaller numbers of differentially expressed genes. The effective targets of both drugs included the *Htr2c, Cdh3, Serpinb1a, Ace, Trpv4, Cacna1a, Mapk13, Mapk8, Cyp2c13, and Htr1a* genes. The results of real-time PCR tests were in accordance with the gene chip data. Based on the target genes and signaling pathway network analysis, we have elaborated the impact and likely mechanism of BXD in treating PMDD and premenstrual irritability.

**Conclusion:** Our work contributes to the understanding of PMDD pathogenesis and the mechanisms of BXD treatment. We speculate that the differentially expressed genes could participate in neuroactive ligand-receptor interactions, mitogen-activated protein kinase, calcium, and gamma-aminobutyric acid signal transduction.

## Introduction

Most women of childbearing age experience premenstrual syndrome (PMS), which is caused by an increase or decrease in ovarian steroids during ovulation (Rapkin and Winer, 2008). The symptoms include emotional problems such as irritability, depression, anxiety, emotional instability, anhedonia, and lassitude as well as physical problems such as breast tenderness, weight gain, distension, muscle and joint pain, headache, and limb edema (Baker and O’Brien, 2012). Recent research in China found that 15–20% of women of childbearing age experienced PMS, while 3–8% women in this age group showed the features of premenstrual dysphoric disorder (PMDD) (O’Brien et al., 2007). The ability to work or study, and the quality of life of women can be severely affected by the resulting emotional problems and physical discomfort, and hence, there is a need for fundamental research to clarify the etiology and pathogenesis of PMDD. In this study, we will not differentiate between PMS and PMDD, since PMDD is a serious form of PMS with emotional problems that can be severe and disabling.

The hippocampus is an important part of the limbic system (Tanaka et al., 1992), which participates in regulating physiological functions such as emotion (Guzman-Velez et al., 2016), learning and memory (Zhang et al., 2013; Ferbinteanu, 2016), hormonal responses, and immunity (Lin et al., 2016; Jo et al., 2017). The hippocampus is especially vulnerable to chronic stress, which can deleteriously affect its function and structure. As an important part of the system regulating autonomic nervous activity, the hippocampus probably plays a role in cognitive disorders caused by autonomic dysfunction (Garcia et al., 2010), but that role is not yet well understood. Magnetic resonance imaging studies have found that depression is associated with atrophy and functional impairment in the hippocampus (Czéh et al., 2001). The therapeutic effects of antidepressants are closely related to their effects on the part of the hippocampus called the dentate gyrus (Santarelli et al., 2003). Therefore, the hippocampus is a natural target for investigation of the neural mechanisms involved in PMS/PMDD.

The anti-depressive effects of the widely used selective serotonin reuptake inhibitor, fluoxetine, rely on its ability to inhibit the 5-HT transporter and thereby reduce reuptake of 5-HT at the presynaptic membrane, consequently prolonging and enhancing the effects of 5-HT (Francois et al., 2003; Sarkisova and Folomkina, 2010). Baixiangdan (BXD) is a novel capsule formulation combining several plant extracts that have been used in traditional Chinese medicine to treat PMS/PMDD. Analytical studies have shown that the main active components of BXD are paeoniflorin, paeonol, and alpha-cyperone (Peng et al., 2010; Zhou et al., 2011; Xie et al., 2015), which may have antipyretic, anti-inflammatory, analgesic, and neuroprotective functions (Lee et al., 2008; Nizamutdinova et al., 2008). Both fluoxetine and BXD can stably and effectively treat PMS/PMDD, but the neural effects of BXD that underlie these actions are unclear. To clarify this question, this paper adopts gene chip technology to identify differentially expressed genes in a rat model of PMS/PMDD based on the widely recognized resident-intruder paradigm (Czéh et al., 2001; Schneider and Popik, 2007b). The goal was to identify relevant signal regulatory pathways and quantify the transcription level of differentially expressed genes, screened by real-time fluorescence quantitative polymerase chain reaction (RT-qPCR) technology.

## Materials and Methods

### Animals

This study used 180 SPF female, healthy, non-pregnant Wistar rats, 6–8 weeks old and with a body mass of 120–140 g, supplied by Beijing Vital River Laboratory Animal Technology Co., Ltd., with production license number SCXK (Jing) 2012-0001. Animal experiments were performed in accordance with the Guide for the Care and Use of Laboratory Animals, formulated by the National Institutes of Health, United States, and were approved by the Institutional Committee for Animal Care and Use of Shandong University of Traditional Chinese Medicine (Approval ID: DWSY201404013).

The living environment featured constant temperature and humidity (23 ± 3°C, 60 ± 5% relative humidity), 12 h/12 h light-dark cycle with day-night reversal (lights on at 20:00, lights off at 8:00). Except during the experimental period, they could freely consume food and water. All experimental operations were conducted under dim light (<28 lux) (Rygula et al., 2006).

Spayed female rats (intruders) were kept in another lab (with the same feeding conditions as the experimental rats), to ensure that the intruders and residents were unfamiliar with each other before the aggressive behavior test. In the aggressive behavior test, the intruders were temporarily placed into the cages of the residents.

### Experimental Animal Screening

The estrous cycle of the rat is divided into proestrus, estrus, metestrus and diestrus phases. The proestrus and estrus phases constitute the receptive phases, while metestrus and diestrus constitute the non-receptive phases. The determination of the phase of the estrous cycle in this experiment was made according to the presence of keratinocytes, nuclear epithelial cells, and leucocytes as well as their proportions, examined in vaginal smears under a microscope (Marcondes et al., 2002). Except for the rats excluded from experiments during the feeding period, vaginal smearing was conducted every day from 13:00 to 14:00 to check whether the estrous cycle of the rats was regular.

### Grouping

After 9 days of vaginal smear testing, rats with 4 days estrous cycles were selected as quasi-residents for the aggressive behavior test. Rats with other cycles were removed and spayed for later use as intruders. During the period in which the intruders recovered from the spaying surgery, daily vaginal smearing was conducted on the quasi-residents, and rats with irregular cycles were removed whenever they were detected. After 2 weeks, when the wounds of the intruders had healed, the aggressive behavior test and open field test (baseline phase data collection) were conducted on all residents in diestrus 1 (the 2nd day of non-receptivity). For model creation, the aggressive behavior test scores of all residents were arranged in high-to-low order by analyzing the resident-intruder experiment videos. The rats with scores within the top 30% were divided randomly into three groups, the PMDD model group (PMDD), fluoxetine intervention group (FXT), and BXD intervention group (BXD), while those with scores within the bottom 30% were used as the control group (CTRL).

### Drugs

Baixiangdan capsules were obtained from the Qingdao Haichuan Center for Innovative Biomedical Research (Qingdao, China, batch number: 20071020). Fluoxetine dispersible tablets were obtained from Eli Lilly and Company (Suzhou, China, batch number: H20050463).

Drug administration was performed during the diestrus 1 phase of the estrous cycle, for 5 days at a dosage of 1 mL of fluid per 100 g of body weight. The drugs were administered intragastrically once each afternoon at 14:00. For the FXT and BXD intervention group, fluoxetine capsules and BXD capsules were used with dosages of 2.7 and 0.2 g/kg/day, respectively, while for the PMDD model group and control group, pure water of the same volume was given.

### Behavioral Experiments

Behavioral data were collected at baseline and after the drug administration interventions. Given that only rats with 4 days estrous cycle were selected for the experiment, behavioral data collection was always performed at diestrus 1.

The open field test (Katz et al., 1981) was conducted by placing the rats in an open field box (Xinruan XR-XZ301, Shanghai, China) with dimensions 50 cm × 50 cm × 50 cm. The animal behavior analysis system XR-Xmaze (Xinruan) was used for data collection. The specific steps were as follows: the operator held the front third part of the rat’s tail and gently placed it in the central area of the open field box; then the observer began to observe and record the motion traces, break times, central grid stopping time, and number of fecal pellets dropped within 180 s. To minimize possible olfactory confounds, after each rat completed the test, the open field box was wiped clean with 75% ethyl alcohol. When the box was completely dry, data collection for the next rat commenced. In this experiment, the experimenters made an effort to grasp and hold the rats as gently as possible, to minimize the resulting stress.

The aggressive behavior test was conducted from 14:30 to 17:30 in the home environment of the residents. Because of the day-night reversal, this timeframe corresponded to the active part of the day for the rats. The specific steps were as follows: after removing the rats that were not to be tested from the cage, the rats and the cage were placed under the camera equipment; after a 15 min adaptation phase, intruders (spayed rats) were placed into the cage for 10 min. After the test, a blinded method was used for evaluation of aggressive behaviors. Three people who were trained together observed the video of each rat, and their observations were checked for consistency (Kappa > 0.95). Two types of aggressive behavior were recorded: frontal attack (springing when the intruder attempted to approach) and side attack (pushing the intruder away by springing from the side with arched back). The observers recorded scores codifying the reactions of the residents to the intruders, including the number of attacks, attack duration, number of bites, number of mounting events, mounting duration, and piloerection. A composite aggressive behavior score was calculated as follows:

$$\begin{array}{c} \mathrm{composite}\;\mathrm{aggression}\;(\mathrm{CA})=(\mathrm{number}\;\mathrm{of}\;\mathrm{attacks})+0.2\times \\ (\mathrm{attack}\;\mathrm{duration}\;[s])+(\mathrm{number}\;\mathrm{of}\;\mathrm{bites})+0.2\times \\ (\mathrm{mounting}\;\mathrm{duration}\;[s])+(\mathrm{piloerection})(\mathrm{Albertet}\;\mathrm{al}.,\;1991). \end{array}$$

### Rat Brain Tissue and RNA Extraction

After decapitating each rat, the operators removed the hippocampus onto an ultra-clean work platform, then immediately placed it in liquid nitrogen for quick freezing, and after 30 min transferred it into a -70°C freezer for storage. In this experiment, all operating instruments, watch glasses, and EP tubes used were sterilized at high temperature after submerging them in diethylpyrocarbonate-treated water overnight. The ultra-clean work platform was scrubbed with diethylpyrocarbonate-treated water and 75% ethyl alcohol, and exposed to UV light to prevent contamination.

Suitably sized (50–100 mg) tissue samples were extracted to be frozen and minced with a biological grinding mill. Then 1 ml of TRIzol^TM^ Reagent (Invitrogen, Thermo Fisher Scientific, Waltham, MA, United States) was added, and RNA was extracted after homogenizing with a bead mill homogenizer. The extracted RNA was stored after passing classical RNA integrity detection and purity detection tests (Vermeulen et al., 2011).

### Gene Chip Experiment

Gene chip analysis was performed by Shanghai Kangcheng Biological Co. Ltd. (Shanghai, China). The rat whole genome oligonucleotide chip used in the experiment was synthesized at Agilent (Santa Clara, CA, United States). The genes covered by the rat whole genome expression chip produced by this company exceed 41,000. The probe design is described in various public databases, including Goldenpath, Ensembl, Unigene, Human Genome (Build 33), Refseq, GenBank, etc.

Under standard conditions, marker probes and the high-density genome chip were hybridized. They were cleaned thereafter, and after spin-drying the slide, the next step of scanning was initiated. Chips in different groups were scanned with an Agilent SureScan gene chip-microarray scanner, to obtain 16-bit tiff files. Agilent Feature Extraction (Version 10.7.3.1) image analysis software was used to analyze the chip images, and to convert the images into a digital format. Finally, GeneSpring GX11.5.1 (software) (Silicon Genetics) was used to conduct standardized processing of initial signal intensity, to obtain the standardized ratio (Cy3/Cy5). *T*-tests were used to compare levels of expression for each gene in each group. In this experiment, genes with *P* < 0.05 and ratio > 2.0 were considered to be differentially expressed.

Heat map and gene clustering analysis were performed using TIGR MultiExperiment Viewer (MeV) Version 4.1^1^. In this experiment, genes with *P* < 0.05 and ratio > 2.0 were considered to be differentially expressed.

### GO and KEGG Enrichment Analysis

Gene Ontology-TermFinder (Boyle et al., 2004) was used to identify Gene Ontology (GO) terms that annotate the list of enriched genes with significant *P*-values less than 0.05^2^. We used in-house scripts to locate information on differentially expressed genes in Kyoto Encyclopedia of Genes and Genomes (KEGG), a collection of databases dealing with genomes, biological pathways, diseases, drugs, and chemical substances^3^.

### Real-Time Fluorogenic Quantitative Polymerase Chain Reaction

The primers used in RT-PCR are listed in Table 1. Glyceraldehyde-3-phosphate dehydrogenase (GAPDH) transcription levels were used as an internal reference. A Thermo Scientific RevertAid First Strand cDNA Synthesis Kit (Thermo Fisher Scientific Inc., Waltham, MA, United States) was used for reverse transcription, and 5 μg of RNA was used for each sample. RT-qPCR Master Mix (Toyobo Co., Ltd., Osaka, Japan) was used for RT-qPCR, which was run on an ABI 7500 fast real-time fluorogenic quantitative RT-PCR system (Life Technologies, Thermo Fisher Scientific).

**Table 1:** Primers used in RT-qPCR.

Gene name	Used as	Sequences	Tm
Htr2c	Forward	5′-TTCTTCATCCCGTTGACGATT-3′	54.3°C
Htr2c	Reverse	5′-TCGGTGTGACCTCGAAGTAAC-3′	57.8°C
Htr2c	Probe	5′-CGATCTACGTCCTGCGCCGTC-3′	63.3°C
Cdh3	Forward	5′-GACAGTGACCGATCTGGATTCC-3′	57.2°C
Cdh3	Reverse	5′-GGTGAAATGATCCCCATCGT-3′	54.9°C
Cdh3	Probe	5′-CCAACTCACCGGCATGGCGTG-3′	63.7°C
Serpinb1a	Forward	5′-TGGGTGTGGTGGACAGCAT-3′	59.5°C
Serpinb1a	Reverse	5′-CTCCCACATCCCCTTGAAGTAG-3′	56.9°C
Serpinb1a	Probe	5′-ACCAAACTTGTGCTGGTGAACGCCA-3′	63.5°C
Ace	Forward	5′-GGGAGAACATTTACGACATGGTAGT-3′	56.5°C
Ace	Reverse	5′-TCCAGCCCTTCTGTACCATTG-3′	58.3°C
Ace	Probe	5′-CCCGGACAAACCCAACCTCGATGT-3′	63.3°C
Trpv4	Forward	5′-ATCCGACGGGAGGTGACA-3′	58.4°C
Trpv4	Reverse	5′-CGTAGGCCCAGTCCTTGAAC-3′	57.8°C
Trpv4	Probe	5′-AGGACACACGGCACCTGTCTCGC-3′	65.5°C
Cacna1a	Forward	5′-GGCATGGTGTTCTCCATCTACTT-3′	56.8°C
Cacna1a	Reverse	5′-CCGCGATAGCTAAGAACACGT-3′	57.3°C
Cacna1a	Probe	5′-CGTCCTCACCCTCTTCGGGAACTACAC-3′	63.5°C
Mapk8	Forward	5′-CCGTACATCAACGTCTGGTATGAT-3′	56.5°C
Mapk8	Reverse	5′-CTCCCTTTCATCTAACTGCTTGTC-3′	56.7°C
Mapk8	Probe	5′-TCAGAAGCAGAGGCCCCACCACC-3′	65.4°C
Mapk13	Forward	5′- GGCGGCCAAATCCTACAT-3′	55.7°C
Mapk13	Reverse	5′-GGGAAAAGCTGTGTGAAATCCT-3′	55.6°C
Mapk13	Probe	5′-AGTCCCTGCCCCAGAGCCCCA-3′	67.9°C
Cyp2c13	Forward	5′-GACACCGCAGCCCCTCTAT-3′	59.5°C
Cyp2c13	Reverse	5′-TCTCTGAACCTCGTGGACCAT-3′	57.7°C
Cyp2c13	Probe	5′-AGGAGCCACATGCCCTACACAAATGC-3′	63.3°C
Htr1a	Forward	5′-TGTTGCTCATGCTGGTTCTCTAC-3′	57.1°C
Htr1a	Reverse	5′-CTGACAGTCTTGCGGATTCG-3′	55.9°C
Htr1a	Probe	5′-CGCATCTTCAGAGCCGCACGCT-3′	64.5°C
GAPDH	Forward	5′-ATCAACGGGAAACCCATCAC-3′	55.3°C
GAPDH	Reverse	5′-GACATACTCAGCACCAGCATCAC-3′	57.8°C
GAPDH	Probe	5′-TCCAGGAGCGAGATCCCGCTAACAT-3′	63.3°C


The RT-qPCR experimental data was analyzed using ABI 7900 system SDS software (Life Technologies, Thermo Fisher Scientific), and relative quantitative analysis was conducted by the 2^-ΔΔCT^ method (Livak and Schmittgen, 2001). An index quantifying expression difference (multiple) from the control was calculated by the following formulas.

$$\begin{array}{c} \mathrm{Relative}\;\mathrm{quantity}\;(\mathrm{RQ})=2^{-\Delta \Delta \mathrm{CT}}\;\mathrm{and}\\ \Delta \Delta \mathrm{CT}=(C_{T1}-C_{T2})-(C_{T3}-C_{T4}). \end{array}$$

C_T1_ and C_T2_ were, respectively, the threshold cycle numbers of target genes and reference genes of experimental samples; C_T3_ and C_T4_ were, respectively, the threshold cycle numbers of target genes and reference genes of control samples. We used samples from the control group (CTRL) as the control samples, with the gene expression level set to the control value 1. Three repeated experiments were conducted for every group of samples, and the average was taken as the result. All tested genes were selected considering gene chip data and previous studies.

### Statistics

Graphpad Prism 5.0 software (GPW5-384305-RAG-5235, Graphpad software, Inc., La Jolla, CA, United States) was used to analyze the experimental data. Two-factor analysis of variance was used to compare the groups in the same period of the menstrual cycle before and after drug administration. Single-factor analysis of variance was used for comparing the groups in different periods of the menstrual cycle, with significance level α = 0.05.

## Results

### BXD and Fluoxetine Can Effectively Treat Rats in PMDD Irritability Model

**FIGURE 1:**
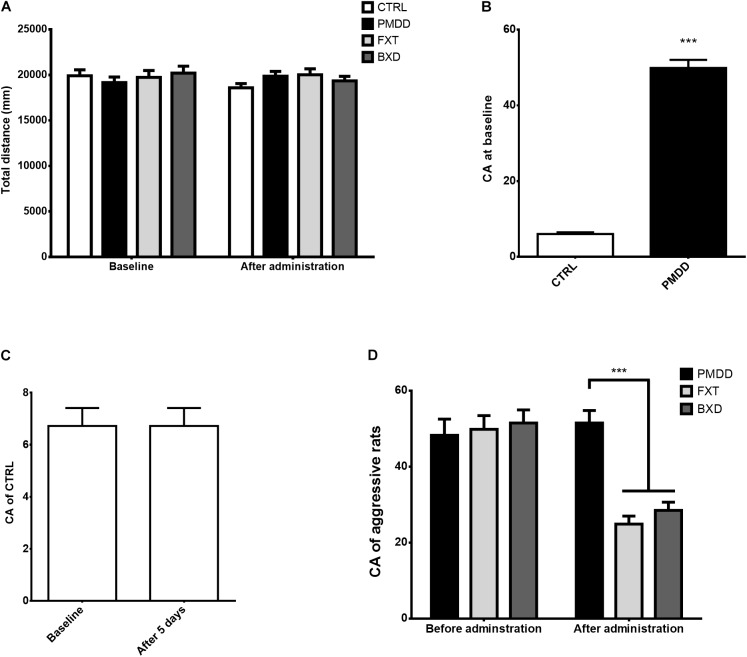
Results of behavioral experiments. **(A)** Total distance (mm) traversed by control group (CTRL, *n* = 8), premenstrual dysphoric disorder model group (PMDD, *n* = 9), fluoxetine administration group (FXT, *n* = 9) and Baixiangdan capsule administration group (BXD, *n* = 9) in open field test at baseline and after administration. **(B)** Composite aggression (CA) scores of non-aggressive rats (control group, CTRL, *n* = 8) and aggressive rats (PMDD, *n* = 27) in aggressive behavior test at baseline. Aggressive rats included those from the PMDD model group (*n* = 9), fluoxetine administration group (*n* = 9) and Baixiangdan capsule administration group (*n* = 9). None of the aggressive rats were given drugs at baseline, and therefore could be treated as equivalent to PMDD model rats for statistical purposes. ^∗∗∗^*P* < 0.001 vs. control group. **(C)** Composite aggression (CA) scores of the control group (CTRL, *n* = 8) in aggressive behavior test at baseline and after 5 days. As the control group was not given drugs, the time points labeled “after administration” were changed as “after 5 days.” **(D)** Composite aggression (CA) scores of PMDD model group (PMDD, *n* = 9), fluoxetine administration group (FXT, *n* = 9) and Baixiangdan capsule administration group (BXD, *n* = 9) in aggressive behavior test before and after drug administration. As there was no baseline testing of aggressive behavior test if rats were given drugs or tap water, the time points labeled “baseline” were changed to “Before administration” ^∗∗∗^*P* < 0.001 vs. PMDD model group.

At baseline, before drug administration, the open field test behavioral scores of rats in different groups did not differ significantly (*p* > 0.05) (Figure 1A). After drug administration, the open field test behavioral scores of rats in different groups again did not differ (*p* > 0.05) (Figure 1A), and moreover, the open field test behavioral scores of rats in different groups before and after the administration did not differ either (*p* > 0.05) (Figure 1A).

Before drug administration (baseline), in the aggressive behavior test, some rats showed very different responses to intruders from other rats, that is, some were aggressive and some were not (*p* < 0.001) (Figure 1B). After the non-aggressive rats were placed into the control group and fed with pure water for 5 days, the composite aggression scores before and after did not differ significantly (*p* > 0.05) (Figure 1C). As described in the Methods section, the aggressive rats were then divided into the PMDD model group, fluoxetine group, and BXD group, and administered pure water, fluoxetine, and BXD, respectively, for 5 days. The composite aggression scores of the PMDD model group before and after administration did not differ significantly (*p* > 0.05) (Figure 1D), but the composite aggression scores of the fluoxetine group and BXD group both showed significant declines after administration (*p* < 0.001 and *p* < 0.001, respectively) (Figure 1D). After administration, when the fluoxetine group and BXD group were compared with the PMDD model group, there were again significant declines in the composite aggression scores for both (*p* < 0.001 and *p* < 0.001, respectively) (Figure 1D).

### Results for Differentially Expressed Genes

**FIGURE 2:**
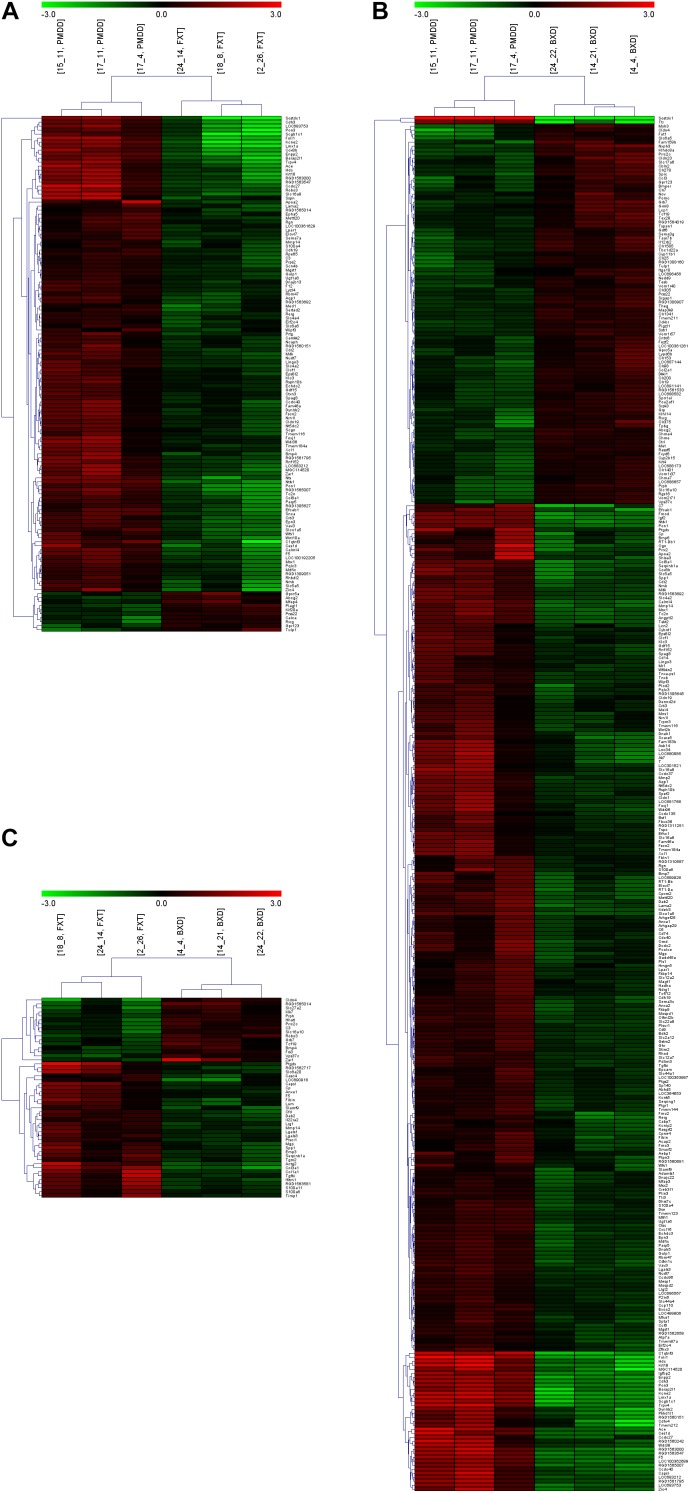
Heat maps and clustering analyses of multiple comparisons. **(A)** Comparison between the PMDD model group (PMDD, samples including 15_11, 17_11 and 17_4) and fluoxetine administration group (FXT, samples including 24_14, 18_8 and 2_26). **(B)** Comparison between the PMDD model group (PMDD, samples including 15_11, 17_11 and 17_4) and Baixiangdan administration group (BXD, samples including 24_22, 14_21 and 4_4). **(C)** Comparison between the fluoxetine administration group (FXT, samples including 24_14, 18_8 and 2_26) and Baixiangdan administration group (BXD, samples including 24_22, 14_21 and 4_4). *P* < 0.05 and ratio > 2.0.

When the PMDD model group was compared with the control group, the number of differentially expressed genes was 137. When the BXD group was compared with the control group, the number of differentially expressed genes was 715. When the fluoxetine group was compared with the BXD group, the number of differentially expressed genes was 199 (Table 2 and Supplementary Materials). The gap in the number of differentially expressed genes between the two drug treatment groups was very large, suggesting that BXD’s mechanism of action against PMS/PMDD is more complex and targets more systems than that of fluoxetine. Besides, the heat maps exhibited a similar tendency, that is, the number of differentially expressed genes between the PMDD model group and fluoxetine group was less than the number of differentially expressed genes between the PMDD model group and BXD group (Figures 2A,B). We also found a significant difference in the number of differentially expressed genes between both drug treatment groups (Figure 2C).

**Table 2:** Numbers of differentially expressed genes in each group.

Groups	Acronyms	Up-regulated genes	Down-regulated genes	Comments
Control group	CTRL	N.A.	N.A.	N.A.
PMDD model group	PMDD	132	5	*P* < 0.05 and ratio > 2.0
Fluoxetine administration group	FXT	112	87	
BXD administration group	BXD	284	431	


The GO analysis indicated that the differentially expressed genes might have participated in several molecular, biological, and cellular processes. As a result, the number of differential genes divided according to functional distribution was larger than that of initial differential genes screened. Since the functions of many of the differentially expressed genes are not yet clear, it may also lead to an opposite situation (Table 3 and Supplementary Materials).

**Table 3:** Functions of differentially expressed genes in each group.

Groups	Acronyms	Molecular function	Biological process	Cellular component	Comments
Control group	CTRL	N.A.	N.A.	N.A.	N.A.
PMDD model group	PMDD	15	111	8	Ratio > 2.0
Fluoxetine administration group	FXT	26	146	5	
BXD administration group	BXD	34	186	25	


The KEGG database was used to conduct an analysis of the signal regulatory pathways that involve the differentially expressed genes. When compared with the BXD group, the signal pathways altered in the fluoxetine group were obviously fewer. In addition, more PMDD irritability-related pathways were affected in the BXD group than in the fluoxetine group, indicating that compared with fluoxetine, the BXD capsules acted on more targets against PMDD. Some genes that were differentially expressed jointly participated in the same signal pathways. For example, when comparing the BXD group and control group, 16 differentially expressed genes were involved in neuroactive ligand-receptor interaction pathways. In addition, 12 differentially expressed genes were involved in the endoplasmic reticulum protein processing pathway and the mitogen activated protein kinase (MAPK) signal pathway. There were also cases of the same differentially expressed gene participating in several signal regulatory pathways. For example, when the fluoxetine group was compared with the control group, the differentially expressed gene *Camk2a* (calcium/calmodulin dependent protein kinase II) participated in a total of 13 pathways. Comparison of the BXD group and the model group showed that the differentially expressed genes were related to 34 signal pathways, including 9 differentially expressed genes involved in neuroactive ligand-receptor interaction pathways and 4 genes involved in cytokine-cytokine receptor interaction pathways. The *Rt1-Bb* (RT1 class II, locus Bb) gene was the gene that was involved in the largest number (15) of signal pathways (Table 4 and Supplementary Materials).

**Table 4:** Regulated and associated signaling pathways in groups.

Groups	Associated signaling pathways
PMDD vs. CTRL	Olfactory transduction; Calcium signaling pathway; Cell adhesion molecules; PI3K-Akt signaling pathway; Ovarian steroidogenesis; Jak-STAT signaling pathway; Neuroactive ligand-receptor interaction; Prolactin signaling; ECM-receptor interaction; Vitamin digestion and absorption
FXT vs. CTRL	Steroid hormone biosynthesis; Calcium signaling pathway; Serotonergic synapse; Vitamin digestion and absorption; Dopaminergic synapse; Neuroactive ligand-receptor interaction; GABAergic synapse; Gap junction; p53 signaling pathway; Cholinergic synapse
BXD vs. CTRL	Calcium signaling pathway; Serotonergic synapse; Glutamatergic synapse; Steroid hormone biosynthesis; Vascular smooth muscle contractio; Axon guidance; Neuroactive ligand-receptor interaction; Long-term potentiation; Adrenergic signaling in cardiomyocytes; Dopaminergic synapse


*PMDD, premenstrual dysphoria disorder; FXT, fluoxetine; BXD, Baixiangdan; CTRL, control.*

Through analysis of the effects of different drugs in the gene chip results, 20 representative differentially expressed genes with obvious intervention effects related to PMS/PMDD irritability were listed (Table 5). Analysis of the signal regulatory pathways affected in the different groups indicated that the pathways related to PMS/PMDD irritability involve olfactory transduction, calcium signaling, cell adhesion, PI3K-Akt signaling, ovarian steroidogenesis, Jak-STAT signaling, neuroactive ligand-receptor interactions, prolactin signaling, extracellular matrix (ECM)-receptor interaction, vitamin digestion and absorption, etc. By linking the differentially expressed genes with obvious intervention effects with the listed signal regulatory pathways, differentially expressed genes for signal regulatory pathways can be identified, namely *Htr2c, Cdh3, Serpinb1a, Ace, Trpv4, Cacna1a, Mapk13, Mapk8, Cyp2c13*, and *Htr1a*.

**Table 5:** Differentially expressed genes obviously affected by fluoxetine or Baixiangdan.

Gene symbol	Gene ID	MvsC (FC)	FvsC (FC)	BvsC (FC)
Msx1	NM_031059	3.54	1.03	1.23
Cldn1	NM_031699	4.67	1.64	1.49
Pon3	NM_001004086	4.73	1.54	2.24
Crb3	NM_001025661	4.08	1.28	2.04
Slc5a5	NM_052983	5.35	1.69	1.29
Htr2c	NM_012765	4.72	1.68	2.31
Cdh3	NM_053938	5.66	1.47	1.30
Serpinb1a	NM_001031642	3.71	2.23	1.44
Ace	NM-012544	5.32	1.59	1.41
Trpv4	NM_023970	5.74	1.29	2.29
Cyp2c23	NM_031839	-8.76	-2.87	-3.35
Hp	NM_012582	-18.45	-3.36	-4.55
C5	XM_345342	-7.23	-1.78	-1.54
Serpinc1	NM_001012027	-12.54	-3.27	-4.36
Aldob	NM_012496	-4.79	-2.23	-1.97
Cacna1a	NM_012918	-2.63	-1.76	-1.71
Mapk13	NM_019231	-2.08	-1.35	-1.24
Mapk8	XM_341399	-1.52	-1.38	-1.33
Cyp2c13	NM_138514	-3.45	-1.57	-1.41
Htr1a	NM_012585	-2.33	-1.17	-1.75


### Comparison of Selected Gene Expression of Hippocampal Tissues in Different Groups

To lower the errors in the RT-PCR experiment, three repetitions were performed for every sample, and in data processing the average CT value for the three amplification curves for every sample was used to calculate the relative quantity. Compared with the control group, the genes *Htr2c, Cdh3, Serpinb1a, Ace*, and *Trpv4* in the hippocampus of rats in the treatment groups showed up-regulated expression; the genes *Cacna1a, Mapk8, Mapk13, Ctp2c13*, and *Htr1a* showed down-regulated expression (Table 6). Among the up-regulated genes, when comparing the PMDD model group with the fluoxetine group and BXD group, the expression levels declined to some degree, indicating that the two drugs had treatment effects on PMS/PMDD irritability.

**Table 6:** Relative quantity (RQ) values of selected genes in each group.

Gene name	Relative quantity (RQ)			
	
	CTRL	PMDD	FXT	BXD
Htr2c	1	4.72	3.16	3.11
Cdh3	1	5.66	2.29	2.08
Serpinb1a	1	3.71	2.16	1.95
Ace	1	5.32	2.68	2.09
Trpv4	1	5.74	1.88	1.78
Cacna1a	1	0.45	0.85	0.75
Mapk8	1	0.66	0.7	0.9
Mapk13	1	0.48	0.78	0.73
Cyp2c13	1	0.29	0.57	0.63
Htr1a	1	0.43	0.89	0.72


*PMDD, premenstrual dysphoria disorder; FXT, fluoxetine; BXD, Baixiangdan; CTRL, control.*

## Discussion

### PMDD Rat Model

Previously used rat models of PMDD include marble burial (Schneider and Popik, 2007a, 2009), progesterone withdrawal (Andréen et al., 2009), emotional stimulation dominated multi-factor continuous modeling induction (Qiao et al., 2017), resident-intruder (Czéh et al., 2001; Schneider and Popik, 2007b), etc. However, according to clinical diagnostic standards for human PMDD patients (American Psychiatric Association, 2000), the phase in which some of these symptoms are manifested corresponds to the luteal phase (non-receptive phase of rats). The resident-intruder method adopted in this study as a PMDD rat model has faced much less controversy, because the expression of animal aggression occurs at the same phase as symptom expression in human PMDD patients. Compared with metestrus and diestrus 2, diestrus 1 yields the most obvious aggressive behaviors of rats. Therefore, in experiments with fluoxetine and BXD intervention, behavioral data during the baseline phase and after drug intervention were collected in diestrus 1.

The estrous cycle of 60–70% of rats is 4–5 days (Marcondes et al., 2002). The selected rats in this experiment were those with 4 days cycles; rats with other cycles were removed. It has been reported that proestrus and estrus last for around 24 h in total, and metestrus, diestrus 1 and diestrus 2 each last for around 24 h (Ho et al., 2001). However, Hubscher et al. (2005) found that proestrus lasted for 12–14 h, estrus lasted for 25–27 h, metestrus lasted for 6–8 h, and diestrus lasted for 55–57 h. For the receptive phase, these two cycles have a gap of almost 12 h. Therefore, in this experiment, rats whose vaginal smear results for leucocytes were (-, +, +, +) or (-, -, +, +) were considered as rats with regular estrous cycles (the ideal status was -, +, +, +). According to research by Ho et al. (2001), about 30–60% of normal injury-free female rats show no aggressive behaviors during any phase of the estrous cycle. These rats were the non-aggressive ones in this experiment, also referred to as the control group in the following drug intervention experiments. After the aggressive behavior test, rats within the top 30% of aggression scores were equally divided into the PMDD model group, fluoxetine group and BXD group (Schneider and Popik, 2007b). Spaying could reduce aggressive behaviors in female rats, and estrogen and progestin could be given with specific therapies to restore cyclic hormone secretion (Melchior et al., 2004), thus recovering cyclic aggressive behavior. Therefore, spaying was conducted in rats with irregular estrous cycle to erase the regularity and enhance stability, and these rats were then used as intruders in resident-intruder experiments.

### Possible Action Targets and Mechanism of BXD Capsule Against PMDD Irritability

Research on the anti-depressive effects of fluoxetine has been thorough, and the drug has also been widely applied clinically for PMDD depression (Francois et al., 2003; Sarkisova and Folomkina, 2010). This paper used fluoxetine as a positive control for the effects of BXD capsules in behavioral experiments and the following chip experiment. On the other hand, because there have been few reports on the use of BXD in PMS/PMDD, this paper focuses on the possible targets of BXD and the mechanism by which BXD capsules affect PMDD irritability. Though our behavioral experiments revealed that fluoxetine and BXD had a similar effect on the PMDD model, some studies have shown different effects. Some studies have shown that the active compounds in BXD could ease most of symptoms of PMDD (Lee et al., 2008; Nizamutdinova et al., 2008; Peng et al., 2010; Zhou et al., 2011; Xie et al., 2015), while other studies showed that fluoxetine was mainly to treat PMDD depression (Francois et al., 2003; Sarkisova and Folomkina, 2010; Li et al., 2016), which is only one subtype of PMDD; these results suggest that BXD could be active on multiple targets. Through analysis of the 6 signal regulatory pathways influenced by BXD capsules, we found that BXD can alter PMDD irritability by influencing various intercellular and intracellular signal transduction pathways. The important targets include 5-Htr (5-Htr1a, 5-Htr2c, 5-Htr3a), mitogen-activated protein kinase (Mapk8, Mapk13), Ca2+ channel proteins (Cacna1a, Cacn2d3, Cacn1i), Drd2, Glul, Gabarapl2, etc.

5-hydroxytryptamine (5-HT), acting mostly on G-protein coupled receptors, serves as a key signal molecule for neuroactive ligand-receptor interaction, and as an important central neurotransmitter, it is closely related to PMDD (Yonkers et al., 2008). 5-Htr1a participates in the stress response involving the hypothalamus-pituitary-adrenal axis system, and is closely associated with negative emotions such as human anxiety and depression, changes in cognitive ability and dietary behaviors, as well as mental disorders such as schizophrenia and Alzheimer’s disease (Nichols and Sanders-Bush, 2001; Müller et al., 2007). 5-Htr2c has regulatory effects on emotion, anxiety, dietary behavior, and reproductive behavior (Heisler et al., 2007). According to our qPCR results, when compared with the control group, the mRNA protein expression level of 5-Htr2c in the hippocampus of rats in the PMDD model group was significantly increased (FCA = 5.64), and the level of 5-Htr1a was significantly decreased. After drug intervention, the mRNA expression levels of the two differential genes basically returned to normal. This is consistent with the chip results, demonstrating the reality and reliability of the chip results as well. Besides, another traditional Chinese medicine, Shuyu capsule, which was found to reduce 5-HT_3A_R and 5-HT_3B_R expression (Li et al., 2016) and has some of the same herbal ingredients as BXD, was seen to effectively treat PMDD depression.

Mitogen activated protein kinase, one of the most important cellular signal transduction pathways, is closely associated with physiological and pathological processes such as cell growth, development, division, apoptosis and intercellular function synchronization (Kumar et al., 2004; Zhang et al., 2015), and also participates in nervous system impairment and restoration (Riddick et al., 2017). There are four main MAPK signal pathways, including ERK, JNK (c-Jun terminal kinase / stress-activated protein kinase), P38, and ERK5 (Large mitogen activated protein kinase 1). MAPK8 (JNK1) and MAPK13 (mitogen-activated protein kinase 1) serve, respectively, as key genes in the JNK pathway and P38 pathway, and JNK and P38 pathway activation are both closely related to cell apoptosis (Xia et al., 1995). JNK pathway activation can promote the apoptosis of various types of cells (Xia et al., 1995). Research has shown that JNK pathway activation can lead to neuronal atrophy or death, and is closely related to neurodegenerative diseases such as Parkinson’s disease (Chen et al., 2012) and Alzheimer’s disease (Yao et al., 2017). This study revealed that when the PMDD model group was compared with the control group, MAPK8 and MAPK13 saw down-regulated expression, but after BXD intervention, the two genes did not see significant up-regulation or down-regulation, indicating that in regulation of the JNK and P38 pathways, the two genes might not have a role in the mRNA expression level, but work through regulation of phosphorylation levels.

Ca^2+^ channels have two common forms, namely, voltage-dependent calcium channels and ligand-gated calcium channels. The types and their physiological functions differ in different tissue cells and different parts of the same cell (Spedding and Paoletti, 1992). The physiological functions include calcium homeostasis maintenance (Nayler and Sturrock, 1986), control of muscle contraction (Held et al., 2007), release of neurotransmitters (Miki et al., 2013), promotion of cell growth and proliferation, regulation of hormone secretion (Sosial and Nussinovitch, 2015), and influences on gene expression (Doran et al., 2007). Cacna1a, a P/Q type of voltage-dependent calcium channel located in nerve cell membranes, is widely distributed in the neuromuscular junction, and mainly participates in mediating the release of certain neurotransmitters (Catterall, 1998). Our chip results showed that when the PMDD model group was compared with the control group, the expression of several calcium ion pathway protein genes (Cacna1a, Cacn2d3, Cacn1i, etc.) was down-regulated, but returned to normal level after BXD intervention. Cacna1a also serves as a key gene in the GABA synapse pathway and dopamine synapse pathway, so it can be deduced that BXD can influence various signal pathways in the central nervous system by acting on genes for calcium ion pathway protein, thus realizing the treatment of PMDD irritability. The signaling pathways through Ca^2+^ channels was described in our previous report, in which we found that paeoniflorin, one of the active compounds of BXD, could inhibit one of the subtypes of the Ca^2+^ channels (Song et al., 2017).

Based on these considerations, it can be predicted that BXD participates in the regulation of pathways involved in the activation of upstream nerve receptor-ligand interaction by regulating the expression or phosphorylation level of important regulatory factors represented by 5-HT and MAPK, thereby realizing the signal transduction of downstream Ca2+ and MAPK and the activation of or inhibition of pathways such as GABAergic synapse and dopaminergic synapses, etc., under the influence of numerous relevant neurotransmitters, hormones, and growth factors. Ultimately, it reduces excitation of the hypothalamic-pituitary-adrenal axis and the hypothalamic-pituitary-gonadal axis of the PMDD rat models, thus resulting in restoration of a normal nerve-internal secretion-immune network functional state.

## Data Availability Statement

LX and MQ are responsible for providing the data supporting the results reported in the current study when required. The initial data obtained by gene chip have been uploaded as Supplementary Materials (in three.zip files).

## Author Contributions

SW contributed to the PMDD modeling. PS, YG, and JC were responsible for data of gene chip and RT-qPCR. JW, CS, and ZL provided essential assistance. LX and MQ directed this project, designed the experiments, and provided key advice.

## Conflict of Interest Statement

The authors declare that the research was conducted in the absence of any commercial or financial relationships that could be construed as a potential conflict of interest.

## Footnotes

1 http://www.sigenae.org/index.php?id=88

2 http://search.cpan.org/dist/GO-TermFinder/

3 http://www.genome.jp/kegg/

## References

[B1] Albert D. Jonik R. Watson N. Moe I. Walsh M. (1991). Aggression by a female rat cohabitating with a sterile male: termination of pseudopregnancy does not abolish aggression. *Physiol. Behav.* 50 519–523. 10.1016/0031-9384(91)90539-Z 1801004

[B2] American Psychiatric Association. (2000). *Diagnostic and Statistical Manual of Mental Disorders: DSM-IV-TR^®^*. Washington, D.C: American Psychiatric Pub.

[B3] Andréen L. Nyberg S. Turkmen S. van Wingen G. Fernández G. Bäckström T. (2009). Sex steroid induced negative mood may be explained by the paradoxical effect mediated by GABAA modulators. *Psychoneuroendocrinology* 34 1121–1132. 10.1016/j.psyneuen.2009.02.003 19272715

[B4] Baker L. O’Brien P. (2012). Premenstrual syndrome (PMS): a peri-menopausal perspective. *Maturitas* 72 121–125. 10.1016/j.maturitas.2012.03.007 22534048

[B5] Boyle E. I. Weng S. Gollub J. Jin H. Botstein D. Cherry J. M. (2004). GO::TermFinder–open source software for accessing gene ontology information and finding significantly enriched gene ontology terms associated with a list of genes. *Bioinformatics* 20 3710–3715. 10.1093/bioinformatics/bth456 15297299 PMC3037731

[B6] Catterall W. A. (1998). Structure and function of neuronal Ca2+ channels and their role in neurotransmitter release. *Cell Calcium* 24 307–323. 10.1016/S0143-4160(98)90055-0 10091001

[B7] Chen C. Y. Weng Y. H. Chien K. Y. Lin K. J. Yeh T. H. Cheng Y. P. (2012). (G2019S) LRRK2 activates MKK4-JNK pathway and causes degeneration of SN dopaminergic neurons in a transgenic mouse model of PD. *Cell Death Differ.* 19 1623–1633. 10.1038/cdd.2012.42 22539006 PMC3438494

[B8] Czéh B. Michaelis T. Watanabe T. Frahm J. de Biurrun G. van Kampen M. (2001). Stress-induced changes in cerebral metabolites, hippocampal volume, and cell proliferation are prevented by antidepressant treatment with tianeptine. *Proc. Natl. Acad. Sci. U.S.A.* 98 12796–12801. 10.1073/pnas.211427898 11675510 PMC60133

[B9] Doran D. E. Weiss D. Zhang Y. Griendling K. K. Taylor W. R. (2007). Differential effects of AT1 receptor and Ca2+ channel blockade on atherosclerosis, inflammatory gene expression, and production of reactive oxygen species. *Atherosclerosis* 195 39–47. 10.1016/j.atherosclerosis.2006.11.030 17224157 PMC2141541

[B10] Ferbinteanu J. (2016). Contributions of hippocampus and striatum to memory-guided behavior depend on past experience. *J. Neurosci.* 36 6459–6470. 10.1523/JNEUROSCI.0840-16.2016 27307234 PMC5015782

[B11] Francois C. Toumi M. Aakhus A. M. Hansen K. (2003). A pharmacoeconomic evaluation of escitalopram, a new selective serotonin reuptake inhibitor. *Eur. J. Health Econ.* 4 12–19. 10.1007/s10198-002-0139-0 15609164

[B12] Garcia T. Esparza J. L. Nogues M. R. Romeu M. Domingo J. L. Gomez M. (2010). Oxidative stress status and RNA expression in hippocampus of an animal model of Alzheimer’s disease after chronic exposure to aluminum. *Hippocampus* 20 218–225. 10.1002/hipo.20612 19405147

[B13] Guzman-Velez E. Warren D. E. Feinstein J. S. Bruss J. Tranel D. (2016). Dissociable contributions of amygdala and hippocampus to emotion and memory in patients with Alzheimer’s disease. *Hippocampus* 26 727–738. 10.1002/hipo.22554 26606553

[B14] Heisler L. Zhou L. Bajwa P. Hsu J. Tecott L. (2007). Serotonin 5-HT2C receptors regulate anxiety-like behavior. *Genes Brain Behav.* 6 491–496. 10.1111/j.1601-183X.2007.00316.x 17451451

[B15] Held B. Tsvilovskyy V. Meissner M. Kaestner L. Ludwig A. Mossmang S. (2007). Ca2+ channel currents and contraction in CaVbeta3-deficient ileum smooth muscle from mouse. *Cell Calcium* 42 477–487. 10.1016/j.ceca.2007.04.013 17580090

[B16] Ho H.-P. Olsson M. Pharm M. Westberg L. Melke J. Eriksson E. (2001). The serotonin reuptake inhibitor fluoxetine reduces sex steroid-related aggression in female rats: an animal model of premenstrual irritability? *Neuropsychopharmacology* 24 502–510. 11282250 10.1016/S0893-133X(00)00219-0

[B17] Hubscher C. Brooks D. Johnson J. (2005). A quantitative method for assessing stages of the rat estrous cycle. *Biotech. Histochem.* 80 79–87. 10.1080/10520290500138422 16195173

[B18] Jo E. Elvitigala D. A. S. Wan Q. Oh M. Oh C. Lee J. (2017). Identification and molecular profiling of DC-SIGN-like from big belly seahorse (*Hippocampus abdominalis*) inferring its potential relevancy in host immunity. *Dev. Comp. Immunol.* 77 270–279. 10.1016/j.dci.2017.08.017 28867209

[B19] Katz R. J. Roth K. A. Carroll B. J. (1981). Acute and chronic stress effects on open field activity in the rat: implications for a model of depression. *Neurosci. Biobehav. Rev.* 5 247–251. 10.1016/0149-7634(81)90005-1 7196554

[B20] Kumar P. Miller A. I. Polverini P. J. (2004). p38 MAPK mediates gamma-irradiation-induced endothelial cell apoptosis, and vascular endothelial growth factor protects endothelial cells through the phosphoinositide 3-kinase-Akt-Bcl-2 pathway. *J. Biol. Chem.* 279 43352–43360. 10.1074/jbc.M405777200 15292252

[B21] Lee B. Shin Y. W. Bae E. A. Han S. J. Kim J. S. Kang S. S. (2008). Antiallergic effect of the root of *Paeonia lactiflora* and its constituents paeoniflorin and paeonol. *Arch. Pharm. Res.* 31 445–450. 10.1007/s12272-001-1177-6 18449501

[B22] Li F. Feng J. Gao D. Wang J. Song C. Wei S. (2016). Shuyu capsules relieve premenstrual syndrome depression by reducing 5-HT3AR and 5-HT3BR expression in the rat brain. *Neural Plast.* 2016:7950781. 10.1155/2016/7950781 27725889 PMC5048033

[B23] Lin T. Zhang D. Liu X. Xiao D. (2016). Parental care improves immunity in the seahorse (*Hippocampus erectus*). *Fish Shellfish Immunol.* 58 554–562. 10.1016/j.fsi.2016.09.065 27702678

[B24] Livak K. J. Schmittgen T. D. (2001). Analysis of relative gene expression data using real-time quantitative PCR and the 2(-Delta Delta C(T)) method. *Methods* 25 402–408. 10.1006/meth.2001.1262 11846609

[B25] Marcondes F. Bianchi F. Tanno A. (2002). Determination of the estrous cycle phases of rats: some helpful considerations. *Braz. J. Biol.* 62 609–614. 10.1590/S1519-69842002000400008 12659010

[B26] Melchior L. K. Ho H.-P. Olsson M. Annerbrink K. Hedner J. Eriksson E. (2004). Association between estrus cycle-related aggression and tidal volume variability in female Wistar rats. *Psychoneuroendocrinology* 29 1097–1100. 10.1016/j.psyneuen.2003.10.008 15219662

[B27] Miki T. Hirai H. Takahashi T. (2013). Activity-dependent neurotrophin signaling underlies developmental switch of Ca2+ channel subtypes mediating neurotransmitter release. *J. Neurosci.* 33 18755–18763. 10.1523/JNEUROSCI.3161-13.2013 24285882 PMC6618703

[B28] Müller C. P. Carey R. J. Huston J. P. De Souza Silva M. A. (2007). Serotonin and psychostimulant addiction: focus on 5-HT1A-receptors. *Prog. Neurobiol.* 81 133–178. 10.1016/j.pneurobio.2007.01.001 17316955

[B29] Nayler W. G. Sturrock W. J. (1986). Calcium channel blockers, beta blockers and the maintenance of calcium homeostasis. *Adv. Exp. Med. Biol.* 194 535–556. 10.1007/978-1-4684-5107-8_41 2875627

[B30] Nichols C. D. Sanders-Bush E. (2001). Serotonin receptor signaling and hallucinogenic drug action. *Heffter Rev. Psychedelic Res.* 2 73–79.

[B31] Nizamutdinova I. T. Jin Y. C. Kim J. S. Yean M. H. Kang S. S. Kim Y. S. (2008). Paeonol and paeoniflorin, the main active principles of *Paeonia albiflora*, protect the heart from myocardial ischemia/reperfusion injury in rats. *Planta Med.* 74 14–18. 10.1055/s-2007-993775 18203054

[B32] O’Brien P. S. Rapkin A. Schmidt P. J. (2007). *The Premenstrual Syndromes: PMS and PMDD*. Boca Raton, FL: CRC Press 10.3109/9781435628168

[B33] Peng S. Sheng W. Zhang H. Y. Qiao M. Q. (2010). Metabolic and behavioral patterns in a pre-menstrual syndrome animal model with liver-qi invasion and their reversal by a Chinese traditional formula. *Chin. Med.* 1 91–97. 10.4236/cm.2010.13017

[B34] Qiao M. Sun P. Wang Y. Wei S. Wei X. Song C. (2017). Profiling proteins in the hypothalamus and hippocampus of a rat model of premenstrual syndrome irritability. *Neural Plast.* 2017:6537230. 10.1155/2017/6537230 28255462 PMC5306999

[B35] Rapkin A. J. Winer S. A. (2008). The pharmacologic management of premenstrual dysphoric disorder. *Expert Opin. Pharmacother.* 9 429–445. 10.1517/14656566.9.3.429 18220493

[B36] Riddick G. Kotliarova S. Rodriguez V. Kim H. S. Linkous A. Storaska A. J. (2017). A core regulatory circuit in glioblastoma stem cells links MAPK activation to a transcriptional program of neural stem cell identity. *Sci. Rep.* 7:43605. 10.1038/srep43605 28256619 PMC5335262

[B37] Rygula R. Abumaria N. Domenici E. Hiemke C. Fuchs E. (2006). Effects of fluoxetine on behavioral deficits evoked by chronic social stress in rats. *Behav. Brain Res.* 174 188–192. 10.1016/j.bbr.2006.07.017 16949682

[B38] Santarelli L. Saxe M. Gross C. Surget A. Battaglia F. Dulawa S. (2003). Requirement of hippocampal neurogenesis for the behavioral effects of antidepressants. *Science* 301 805–809. 10.1126/science.1083328 12907793

[B39] Sarkisova K. Folomkina A. A. (2010). Effect of selective serotonin reuptake inhibitor fluoxetine on symptoms of depression-like behavior in WAG/Rij rats. *Zh Vyssh Nerv Deiat Im I P Pavlova* 60 98–108. 20352689

[B40] Schneider T. Popik P. (2007a). Attenuation of estrous cycle-dependent marble burying in female rats by acute treatment with progesterone and antidepressants. *Psychoneuroendocrinology* 32 651–659. 17561352 10.1016/j.psyneuen.2007.04.003

[B41] Schneider T. Popik P. (2007b). Increased depressive-like traits in an animal model of premenstrual irritability. *Horm. Behav.* 51 142–148. 10.1016/j.yhbeh.2006.09.006 17049520

[B42] Schneider T. Popik P. (2009). An animal model of premenstrual dysphoric disorder sensitive to antidepressants. *Curr. Protoc. Neurosci.* 46 9.31.1–9.31.10. 10.1002/0471142301.ns0931s46 19170024

[B43] Song C. Wang J. Gao D. Yu Y. Li F. Wei S. (2017). Paeoniflorin, the main active ingredient of shuyu capsule, inhibits Cav1.2 and regulates Calmodulin/Calmodulin-dependent protein kinase II signalling. *Biomed. Res. Int.* 2017:8459287. 10.1155/2017/8459287 29362718 PMC5736929

[B44] Sosial E. Nussinovitch I. (2015). Multiple Ca2+ channel-dependent components in growth hormone secretion from rat anterior pituitary somatotrophs. *J. Neuroendocrinol.* 27 166–176. 10.1111/jne.12240 25442738

[B45] Spedding M. Paoletti R. (1992). Classification of calcium channels and the sites of action of drugs modifying channel function. *Pharmacol. Rev.* 44 363–376. 1332082

[B46] Tanaka T. Fujita T. Tanaka S. Takano K. Yonemasu Y. (1992). Effect of anticonvulsants upon experimental limbic seizure status and regional cerebral blood flow in the hippocampus. *No To Shinkei* 44 234–240. 1591100

[B47] Vermeulen J. De Preter K. Lefever S. Nuytens J. De Vloed F. Derveaux S. (2011). Measurable impact of RNA quality on gene expression results from quantitative PCR. *Nucleic Acids Res.* 39:e63. 10.1093/nar/gkr065 21317187 PMC3089491

[B48] Xia Z. Dickens M. Raingeaud J. Davis R. J. Greenberg M. E. (1995). Opposing effects of ERK and JNK-p38 MAP kinases on apoptosis. *Science* 270 1326–1331. 10.1126/science.270.5240.1326 7481820

[B49] Xie Y. Li L. Shao Q. Wang Y. Liang Q. L. Zhang H. Y. (2015). Urinary metabolomics study on an induced-stress rat model using UPLC-QTOF/MS. *RSC Adv.* 5 75111–75120. 10.1039/C5RA10992B

[B50] Yao Z. Yang W. Gao Z. Jia P. (2017). Nicotinamide mononucleotide inhibits JNK activation to reverse Alzheimer disease. *Neurosci. Lett.* 647 133–140. 10.1016/j.neulet.2017.03.027 28330719

[B51] Yonkers K. A. O’Brien P. Eriksson E. (2008). Premenstrual syndrome. *Lancet* 371 1200–1210. 10.1016/S0140-6736(08)60527-9 18395582 PMC3118460

[B52] Zhang C. Spevak W. Zhang Y. Burton E. A. Ma Y. Habets G. (2015). RAF inhibitors that evade paradoxical MAPK pathway activation. *Nature* 526 583–586. 10.1038/nature14982 26466569

[B53] Zhang H. Li X. Nie J. Niu Q. (2013). Lactation exposure to BDE-153 damages learning and memory, disrupts spontaneous behavior and induces hippocampus neuron death in adult rats. *Brain Res.* 1517 44–56. 10.1016/j.brainres.2013.04.014 23624224

[B54] Zhou J. Xie G. Yan X. (2011). *Encyclopedia of Traditional Chinese Medicines - Molecular Structures, Pharmacological Activities, Natural Sources and Applications*. Berlin: Springer.

