# Supplementary material for: Gene Expression in the Hippocampus in a Rat Model of Premenstrual Dysphoric Disorder After Treatment With Baixiangdan Capsules
Source: Front Psychol. 2018 Nov 13;9:2065. doi: 10.3389/fpsyg.2018.02065 (PMC6242977; doi:10.3389/fpsyg.2018.02065)
Supplement: Supplementary file 1 [file Data_Sheet_1.ZIP › File 1. Summary & QC/Labeling_Report.pdf]

Labeling Efficiency-QC

In the experiment, 1μg RNA is used for labeling. The specific activity (pmol dyes per μg cRNA) of the labeled RNA can be obtained by the following calculation:

$$\text{Specific Activity} = \frac{(\text{pmol per μl dye})}{(\text{μg per μl cRNA})}$$

Sample ID	Dye Name	Dye pmol/μl	cRNA Concentration (μg/μl)	Specific Activity*(pmol Dye/μg cRNA)	Volume (μl)	Total Amount (μg)
3_1	Cy3	16.78	0.71886	23.34251	20	14.3772
6_25	Cy3	16.31	0.70795	23.03835	20	14.159
21_20	Cy3	16.32	0.71884	22.70324	20	14.3768
15_11	Cy3	16.15	0.70825	22.80268	20	14.165
17_4	Cy3	16.54	0.7394	22.36949	20	14.788
17_11	Cy3	16.67	0.71212	23.40898	20	14.2424
2_26	Cy3	16.57	0.73789	22.45592	20	14.7578
18_8	Cy3	16.95	0.7583	22.35263	20	15.166
24_14	Cy3	16.02	0.74489	21.50653	20	14.8978
14_21	Cy3	16.69	0.76526	21.80958	20	15.3052
4_4	Cy3	16.72	0.78584	21.2766	20	15.7168
24_22	Cy3	16.97	0.78848	21.52242	20	15.7696

*For two-color, if the yield is <825 ng and the specific activity is <8.0 pmol Cy3 or Cy5 per μg cRNA do not proceed to the hybridization step. Repeat cRNA preparation.

*For one-color, if the yield is <1.65μg and the specific activity is <9.0 pmol Cy3 or Cy5 per μg cRNA do not proceed to the hybridization step. Repeat cRNA preparation.

KangChen Bio-tech 421 Hong Cao Rd. Building 63, 2nd floor Shanghai P.R. China 200233
Tel: 86-21-64451989 Fax: 86-21-64452021
Email: ping@kangchen.com.cn Website: www.kangchen.com.cn
