# Supplementary material for: Gene Expression in the Hippocampus in a Rat Model of Premenstrual Dysphoric Disorder After Treatment With Baixiangdan Capsules
Source: Front Psychol. 2018 Nov 13;9:2065. doi: 10.3389/fpsyg.2018.02065 (PMC6242977; doi:10.3389/fpsyg.2018.02065)
Supplement: Supplementary file 1 [file Data_Sheet_1.ZIP › File 1. Summary & QC/RNA_QC.pdf]

RNA QC EUK

1. RNA Quantification and Quality Assurance by NanoDrop ND-1000

Sample ID	OD260/280 Ratio	OD260/230 Ratio	Conc. (ng/µl)	Volume (µl)	Quantity (ng)	QC result Pass or Fail
3_1	1.98	2.32	566.80	40	22672.00	pass
6_25	2.02	2.17	672.79	50	33639.50	pass
21_20	1.96	1.86	654.33	40	26173.20	pass
15_11	2.01	2.36	876.47	30	26294.10	pass
17_4	2.00	2.33	717.13	50	35856.50	pass
17_11	2.01	2.32	660.81	50	33040.50	pass
28_18	2.00	2.34	639.66	50	31983.00	pass
2_26	1.96	2.33	577.20	30	17316.00	pass
18_8	1.97	2.31	537.93	50	26896.50	pass
24_14	2.01	2.33	620.48	50	31024.00	pass
27_30	2.01	2.34	728.05	50	36402.50	pass
4_4	1.98	2.32	654.06	30	19621.80	pass
14_21	1.91	2.43	468.02	30	14040.60	pass
24_22	1.90	2.42	471.89	50	23594.50	pass
15_11	1.95	2.25	518.80	50	25940.00	pass

*For spectrophotometer, the O.D. A260/A280 ratio should be close to 2.0 for pure RNA (ratios between 1.8 and 2.1 are acceptable). The O.D. A260/A230 ratio should be more than 1.8.

KangChen Bio-tech 421 Hong Cao Rd. Tel: 86-21-64451989 Email: ping@kangchen.com.cn
Building 63, 2nd floor Shanghai P.R. China 200233 Fax: 86-21-64452021 Website: www.kangchen.com.cn

2. RNA Integrity and gDNA contamination test by Denaturing Agarose Gel Electrophoresis

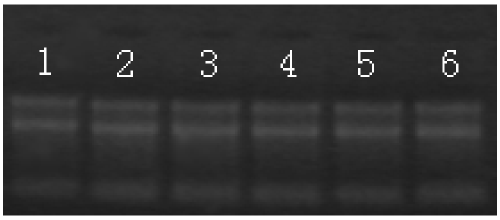

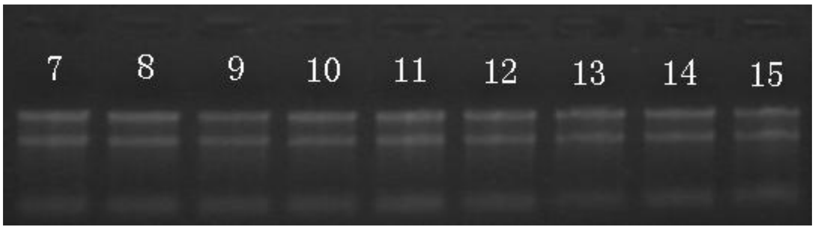

Lane 1:	Total RNA of sample	3_1
Lane 2:	Total RNA of sample	6_25
Lane 3:	Total RNA of sample	21_20
Lane 4:	Total RNA of sample	15_11
Lane 5:	Total RNA of sample	17_4
Lane 6:	Total RNA of sample	17_11
Lane 7:	Total RNA of sample	28_18
Lane 8:	Total RNA of sample	2_26
Lane 9:	Total RNA of sample	18_8
Lane 10:	Total RNA of sample	24_14
Lane 11:	Total RNA of sample	27_30
Lane 12:	Total RNA of sample	4_4
Lane 13:	Total RNA of sample	14_21
Lane 14:	Total RNA of sample	24_22
Lane 15:	Total RNA of sample	15_11

*The 28S and 18S ribosomal RNA bands should be fairly sharp, intense bands. The intensity of the upper band should be about twice that of the lower band. Smaller, more diffuse bands representing low molecular weight RNAs (tRNA and 5S ribosomal RNA) may be present. It is normal to see a diffuse smear of ethidium bromide staining material migrating between the 18S and 28S ribosomal bands, probably comprised of mRNA and other heterogeneous RNA species. DNA contamination of the RNA preparation will be evident as a high molecular weight smear or band migrating above the 28S ribosomal RNA band. Degradation of the RNA will be reflected by smearing of ribosomal RNA bands.
