# Supplementary material for: Gene Expression in the Hippocampus in a Rat Model of Premenstrual Dysphoric Disorder After Treatment With Baixiangdan Capsules
Source: Front Psychol. 2018 Nov 13;9:2065. doi: 10.3389/fpsyg.2018.02065 (PMC6242977; doi:10.3389/fpsyg.2018.02065)
Supplement: Supplementary file 3 [file Data_Sheet_3.ZIP › Data Analysis Folder/GO Analysis Report/BXD vs blank (down)/BP_result(Rat).html]

| GO.ID | Term | Ontology | Count | Pop.Hits | List.Total | Pop.Total | Fold.Enrichment | Pvalue | FDR | Enrichment.Score | GENES |
| --- | --- | --- | --- | --- | --- | --- | --- | --- | --- | --- | --- |
| GO:0016192 | vesicle-mediated transport | Biological process | 41 | 723 | 295 | 13692 | 2.63202756874604 | 1.21577037060562e-08 | 5.32993730473504e-05 | 7.91514844496151 | YWHAZ//NSF//RGD1304592//TMED2//EXOC4//GCGR//SNAP25//SCAMP1//GOSR1//KLHL12//SEC24C//ASGR2//LRP4//PIK3CB//CAP1//PICALM//ATP5B//CDC42SE2//NAPA//PPP3CB//RAPGEF4//KRT18//SPTBN1//ANK3//KIT//ITGB1//CD14//PPP3CA//PRKCI//TMED9//HNRNPK//CANX//CALR//RGD1560691//MLC1//PLCB1//VAMP1//ARF1//ARF4//GSN//ARCN1 |
| GO:0033036 | macromolecule localization | Biological process | 60 | 1394 | 295 | 13692 | 1.99771417454952 | 1.15449910060472e-07 | 0.000224165549396363 | 6.9376064010773 | UGT8//KEAP1//PPP3CA//KPNB1//PTTG1IP//LRP4//NRCAM//YWHAZ//CALR//HSPA9//AHCYL1//EXOC4//MGEA5//SRPR//KDELR3//MAPK9//MAL//VAMP1//TMED2//NAPA//VPS16//ARCN1//SEC24C//SPTBN1//SCAMP1//NSF//ARF1//GABARAPL2//SENP2//ARF4//GOSR1//MLC1//TMED9//PDCD6IP//ZFP828//ITGB1//PICALM//HSP90AB1//SIDT1//MESDC2//PTPRK//CLASP2//PRKCI//TCF7L2//ZDHHC17//KRT18//ANK3//PLEKHA1//PSAP//CD14//FMR1//HNRNPA1//HNRNPA3//ARL2BP//TNKS2//DNAJA3//HNRNPK//RBPJ//GPR158//RGD1304592 |
| GO:0008104 | protein localization | Biological process | 54 | 1204 | 295 | 13692 | 2.08167126527395 | 1.53397958072329e-07 | 0.000224165549396363 | 6.81418042137675 | UGT8//KEAP1//PPP3CA//KPNB1//PTTG1IP//YWHAZ//CALR//HSPA9//AHCYL1//EXOC4//MGEA5//SRPR//KDELR3//MAPK9//MAL//VAMP1//TMED2//NAPA//VPS16//ARCN1//SEC24C//SPTBN1//SCAMP1//NSF//ARF1//GABARAPL2//SENP2//ARF4//GOSR1//MLC1//TMED9//PDCD6IP//ZFP828//ITGB1//PICALM//HSP90AB1//MESDC2//PTPRK//CLASP2//PRKCI//TCF7L2//ZDHHC17//KRT18//ANK3//LRP4//PLEKHA1//CD14//ARL2BP//TNKS2//DNAJA3//RBPJ//GPR158//RGD1304592//NRCAM |
| GO:0034613 | cellular protein localization | Biological process | 38 | 717 | 295 | 13692 | 2.45985391107014 | 2.52219238140054e-07 | 0.000237245344456949 | 6.59822179048531 | UGT8//KEAP1//PPP3CA//KPNB1//PTTG1IP//YWHAZ//CALR//HSPA9//AHCYL1//EXOC4//MGEA5//SRPR//KDELR3//MAPK9//MAL//VAMP1//TMED2//NAPA//VPS16//ARCN1//SEC24C//SPTBN1//ZFP828//ITGB1//HSP90AB1//MESDC2//PTPRK//CLASP2//TCF7L2//KRT18//ANK3//LRP4//ARL2BP//TNKS2//DNAJA3//GPR158//RGD1304592//PRKCI |
| GO:0070727 | cellular macromolecule localization | Biological process | 38 | 719 | 295 | 13692 | 2.45301148016313 | 2.70580912929914e-07 | 0.000237245344456949 | 6.56770284226012 | UGT8//KEAP1//PPP3CA//KPNB1//PTTG1IP//YWHAZ//CALR//HSPA9//AHCYL1//EXOC4//MGEA5//SRPR//KDELR3//MAPK9//MAL//VAMP1//TMED2//NAPA//VPS16//ARCN1//SEC24C//SPTBN1//ZFP828//ITGB1//HSP90AB1//MESDC2//PTPRK//CLASP2//PRKCI//TCF7L2//KRT18//ANK3//LRP4//ARL2BP//TNKS2//DNAJA3//GPR158//RGD1304592 |
| GO:0051641 | cellular localization | Biological process | 62 | 1510 | 295 | 13692 | 1.90572230328881 | 3.62172297589279e-07 | 0.000264627225438566 | 6.44108477175863 | UGT8//YWHAZ//KEAP1//PPP3CA//KPNB1//PTTG1IP//TMED2//EXOC4//CALR//HSPA9//AHCYL1//MGEA5//SRPR//KDELR3//MAPK9//MAL//VAMP1//NAPA//VPS16//ARCN1//SEC24C//GCGR//SNAP25//SCAMP1//GOSR1//KLHL12//ANP32A//SPTBN1//NSF//PPP3CB//PICALM//RAPGEF4//HIF1A//ZFP828//GLUL//TCF7L2//MLC1//ITGB1//HSP90AB1//RTN4//MESDC2//PTPRK//CLASP2//PRKCI//KRT18//ANK3//LRP4//KIT//SLC1A3//SLC25A13//CNP//TMED9//CD14//HNRNPA1//ARL2BP//PRIMA1//TNKS2//DNAJA3//RBPJ//GPR158//RGD1304592//PLCB1 |
| GO:0046907 | intracellular transport | Biological process | 39 | 774 | 295 | 13692 | 2.3386677177769 | 6.25779053389847e-07 | 0.000385343064675862 | 6.20357897802181 | KEAP1//PPP3CA//KPNB1//PTTG1IP//YWHAZ//CALR//HSPA9//AHCYL1//EXOC4//MGEA5//SRPR//MAPK9//MAL//VAMP1//TMED2//NAPA//VPS16//ARCN1//SEC24C//GOSR1//KLHL12//ANP32A//SPTBN1//UGT8//SNAP25//PICALM//HIF1A//MLC1//ITGB1//HSP90AB1//TCF7L2//KRT18//ANK3//SLC1A3//SLC25A13//CNP//PRKCI//TMED9//HNRNPA1 |
| GO:0006457 | protein folding | Biological process | 15 | 148 | 295 | 13692 | 4.70407695831425 | 7.03180774955952e-07 | 0.000385343064675862 | 6.15293301143354 | HSPA8//CCT4//HSPH1//DNAJC7//CANX//CALR//HSPA9//CCT5//FKBP9//CCT2//DNAJA4//HSP90AB1//MESDC2//DNAJA3//DNAJB2 |
| GO:0051234 | establishment of localization | Biological process | 92 | 2726 | 295 | 13692 | 1.56641506149197 | 2.31689919689951e-06 | 0.00112858734213416 | 5.63509286098026 | YWHAZ//KEAP1//VAMP1//NSF//ARF1//ARF4//PICALM//GSN//ARCN1//MLC1//PPP3CA//KPNB1//PTTG1IP//RGD1304592//TMED2//EXOC4//CALR//HSPA9//AHCYL1//MGEA5//SRPR//MAPK9//SLC44A1//LAPTM4A//ACBD5//SCN4B//ITPR1//SLC24A2//CACNA2D3//CLCN2//CLCC1//SLC1A3//SLC25A13//MAL//NAPA//VPS16//SEC24C//GCGR//SNAP25//SCAMP1//GOSR1//KLHL12//ASGR2//LRP4//PIK3CB//CAP1//ATP5B//CDC42SE2//ANP32A//SPTBN1//DDR1//HIF1A//PPP3CB//UGT8//CNKSR3//GABARAPL2//SENP2//KDELR3//TMED9//PDCD6IP//MAGT1//ZDHHC17//SERINC1//PRKCI//RAPGEF4//ZFP828//GLUL//TCF7L2//STIM2//ITGB1//HSP90AB1//SIDT1//RTN4//KRT18//ANK3//KIT//PLEKHA1//CD14//PSAP//CNP//HNRNPK//CANX//RGD1560691//FMR1//HNRNPA1//HNRNPA3//GFAP//PRIMA1//SLC25A5//HIATL1//RBPJ//PLCB1 |
| GO:0051179 | localization | Biological process | 108 | 3407 | 295 | 13692 | 1.47128394680941 | 4.04196571287996e-06 | 0.00161264884381356 | 5.39340737483115 | CAP1//HIF1A//SEMA3C//ITGB1//ROBO1//UGT8//KIT//YWHAZ//KEAP1//VAMP1//NSF//ARF1//ARF4//PICALM//GSN//ARCN1//MLC1//PPP3CA//KPNB1//PTTG1IP//LRP4//NRCAM//RGD1304592//TMED2//EXOC4//CALR//HSPA9//AHCYL1//MGEA5//SRPR//KDELR3//MAPK9//SLC44A1//LAPTM4A//ACBD5//SCN4B//ITPR1//SLC24A2//CACNA2D3//CLCN2//CLCC1//SLC1A3//SLC25A13//MAL//NAPA//VPS16//SEC24C//GCGR//SNAP25//SCAMP1//GOSR1//KLHL12//ASGR2//PIK3CB//ATP5B//CDC42SE2//ANP32A//SPTBN1//DDR1//PPP3CB//CNKSR3//ADAM10//RAPGEF4//RBPJ//GABARAPL2//SENP2//TMED9//PDCD6IP//MAGT1//ZDHHC17//SERINC1//PRKCI//CLASP2//NRP1//PTPRK//RTN4//PTP4A1//ZFP828//GLUL//TCF7L2//STIM2//HSP90AB1//SIDT1//MESDC2//KRT18//ANK3//PLEKHA1//CD14//PSAP//CNP//HNRNPK//CANX//RGD1560691//FMR1//HNRNPA1//HNRNPA3//ARL2BP//GFAP//PRIMA1//SLC25A5//HIATL1//CCL6//ELMO2//TNKS2//DNAJA3//LPAR1//GPR158//PLCB1 |
| GO:0045184 | establishment of protein localization | Biological process | 42 | 932 | 295 | 13692 | 2.09159816687277 | 4.04633605883877e-06 | 0.00161264884381356 | 5.39293805076976 | KEAP1//PPP3CA//KPNB1//PTTG1IP//YWHAZ//CALR//HSPA9//AHCYL1//EXOC4//MGEA5//SRPR//MAPK9//MAL//VAMP1//TMED2//NAPA//VPS16//ARCN1//SEC24C//SPTBN1//SCAMP1//NSF//ARF1//GABARAPL2//SENP2//ARF4//GOSR1//KDELR3//MLC1//TMED9//PDCD6IP//ITGB1//HSP90AB1//TCF7L2//ZDHHC17//KRT18//ANK3//CD14//PICALM//RBPJ//PRKCI//PLEKHA1 |
| GO:0006810 | transport | Biological process | 89 | 2679 | 295 | 13692 | 1.54192115702166 | 7.15983580118855e-06 | 0.00255106597905597 | 5.14509693739262 | YWHAZ//KEAP1//VAMP1//NSF//ARF1//ARF4//PICALM//GSN//ARCN1//MLC1//PPP3CA//KPNB1//PTTG1IP//RGD1304592//TMED2//EXOC4//CALR//HSPA9//AHCYL1//MGEA5//SRPR//MAPK9//SCN4B//ITPR1//SLC24A2//CACNA2D3//CLCN2//CLCC1//SLC1A3//SLC25A13//MAL//NAPA//VPS16//SEC24C//GCGR//SNAP25//SCAMP1//GOSR1//KLHL12//ASGR2//LRP4//PIK3CB//CAP1//ATP5B//CDC42SE2//ANP32A//SPTBN1//DDR1//HIF1A//PPP3CB//UGT8//CNKSR3//GABARAPL2//SENP2//KDELR3//TMED9//PDCD6IP//MAGT1//ZDHHC17//SERINC1//SLC44A1//PRKCI//RAPGEF4//GLUL//TCF7L2//STIM2//ITGB1//HSP90AB1//SIDT1//RTN4//KRT18//ANK3//KIT//CD14//PSAP//CNP//HNRNPK//CANX//RGD1560691//FMR1//HNRNPA1//HNRNPA3//GFAP//SLC25A5//HIATL1//RBPJ//PLCB1//LAPTM4A//ACBD5 |
| GO:0051649 | establishment of localization in cell | Biological process | 52 | 1299 | 295 | 13692 | 1.85797158179043 | 8.07268970249588e-06 | 0.00255106597905597 | 5.09298174057786 | YWHAZ//KEAP1//PPP3CA//KPNB1//PTTG1IP//TMED2//EXOC4//CALR//HSPA9//AHCYL1//MGEA5//SRPR//MAPK9//MAL//VAMP1//NAPA//VPS16//ARCN1//SEC24C//GCGR//SNAP25//SCAMP1//GOSR1//KLHL12//ANP32A//SPTBN1//NSF//PPP3CB//UGT8//PICALM//RAPGEF4//HIF1A//ZFP828//GLUL//TCF7L2//MLC1//ITGB1//HSP90AB1//RTN4//KRT18//ANK3//KIT//SLC1A3//SLC25A13//CNP//PRKCI//TMED9//CD14//HNRNPA1//RBPJ//PLCB1//PRIMA1 |
| GO:0015031 | protein transport | Biological process | 40 | 893 | 295 | 13692 | 2.07899481845617 | 8.14665230537945e-06 | 0.00255106597905597 | 5.08902081874243 | KEAP1//PPP3CA//KPNB1//PTTG1IP//YWHAZ//CALR//HSPA9//AHCYL1//EXOC4//MGEA5//SRPR//MAPK9//MAL//VAMP1//TMED2//NAPA//VPS16//ARCN1//SEC24C//SPTBN1//ITGB1//HSP90AB1//TCF7L2//ZDHHC17//KRT18//ANK3//CD14//PICALM//RBPJ//SCAMP1//NSF//ARF1//GABARAPL2//SENP2//ARF4//GOSR1//KDELR3//MLC1//TMED9//PDCD6IP |
| GO:0035637 | multicellular organismal signaling | Biological process | 29 | 558 | 295 | 13692 | 2.41217422999818 | 1.07441740424888e-05 | 0.00314016393348473 | 4.96882696524251 | EXOC4//SNAP25//NSF//PLP1//CLDN11//NAPA//NRCAM//MRF//CD9//UGT8//TCF7L2//NDRG1//GLUL//PRIMA1//MAL//MPDZ//LPAR1//PLK2//EGR1//NPTN//KIT//CANX//PPP3CA//RAPGEF4//SLC1A3//GFAP//SLC24A2//MGLL//SCN4B |
| GO:0022008 | neurogenesis | Biological process | 43 | 1009 | 295 | 13692 | 1.97798121986864 | 1.24280464289787e-05 | 0.00340528472154016 | 4.90559713296682 | HIF1A//SNAP25//CNP//ACTB//PICALM//ROBO1//NRP1//SEMA3C//ANK3//NRCAM//RTN4//CHN1//RBPJ//PLP1//GFAP//ITGB1//NPTN//PRKCI//RGD1560691//LPAR1//CD9//MRF//GSN//TCF7L2//NAPA//MAPK9//PRICKLE2//PTPRK//NDRG1//PLS3//S1PR5//CALR//ZEB1//SEMA7A//KIT//SLC1A3//EGR1//EXOC4//CLCF1//UGT8//FAIM//RAPGEF4//NPTX1 |
| GO:0016044 | cellular membrane organization | Biological process | 19 | 293 | 295 | 13692 | 3.00975299357899 | 2.00001425989882e-05 | 0.00492973627558975 | 4.69896690784937 | NSF//RGD1304592//NAPA//SPTA1//SPTBN1//ANK3//CD9//NDRG1//ITGB1//LRP4//NRCAM//CNP//PRKCI//TMED9//KLHL12//PICALM//DNAJA3//GPR158//TMED2 |
| GO:0019226 | transmission of nerve impulse | Biological process | 28 | 547 | 295 | 13692 | 2.37583119015896 | 2.02407055110893e-05 | 0.00492973627558975 | 4.69377435377725 | EXOC4//SNAP25//NSF//PLP1//CLDN11//NAPA//NRCAM//MRF//CD9//UGT8//TCF7L2//NDRG1//GLUL//PRIMA1//MAL//MPDZ//LPAR1//PLK2//EGR1//NPTN//KIT//CANX//PPP3CA//RAPGEF4//SLC1A3//GFAP//SLC24A2//MGLL |
| GO:0061024 | membrane organization | Biological process | 19 | 298 | 295 | 13692 | 2.95925378227733 | 2.52932953345343e-05 | 0.00583609509192623 | 4.59699458495485 | NSF//RGD1304592//NAPA//SPTA1//SPTBN1//ANK3//CD9//NDRG1//ITGB1//LRP4//NRCAM//CNP//PRKCI//TMED9//KLHL12//PICALM//DNAJA3//GPR158//TMED2 |
| GO:0007272 | ensheathment of neurons | Biological process | 10 | 92 | 295 | 13692 | 5.04495210022108 | 2.80634586236984e-05 | 0.00585858107649018 | 4.55185880627187 | PLP1//CLDN11//MRF//CD9//UGT8//TCF7L2//NDRG1//MAL//MPDZ//LPAR1 |
| GO:0008366 | axon ensheathment | Biological process | 10 | 92 | 295 | 13692 | 5.04495210022108 | 2.80634586236984e-05 | 0.00585858107649018 | 4.55185880627187 | PLP1//MRF//CD9//UGT8//TCF7L2//NDRG1//MAL//MPDZ//LPAR1//CLDN11 |
| GO:0007399 | nervous system development | Biological process | 55 | 1503 | 295 | 13692 | 1.69843364119219 | 5.49086934216069e-05 | 0.0109418050891057 | 4.26035889041077 | TMED2//APAF1//HIF1A//SNAP25//CNP//ACTB//PICALM//ROBO1//NRP1//SEMA3C//ANK3//NRCAM//RTN4//FMR1//ZEB1//MAPK9//CD9//NT5E//ARF4//NAPA//TCF7L2//CHN1//PLP1//CLDN11//RBPJ//GFAP//ITGB1//NPTN//PRKCI//RGD1560691//LPAR1//MRF//GSN//LUZP1//SLC1A3//PLCB1//UGT8//PRICKLE2//PTPRK//NDRG1//PLS3//MAL//MPDZ//S1PR5//CALR//SEMA7A//KIT//EGR1//EXOC4//CLCF1//FAIM//RAPGEF4//LRP4//NPTX1//MOBP |
| GO:0006886 | intracellular protein transport | Biological process | 25 | 492 | 295 | 13692 | 2.35841256717652 | 6.42896797318862e-05 | 0.0122541719975908 | 4.19185873773238 | KEAP1//PPP3CA//KPNB1//PTTG1IP//YWHAZ//CALR//HSPA9//AHCYL1//EXOC4//MGEA5//SRPR//MAPK9//SPTBN1//ITGB1//HSP90AB1//TCF7L2//KRT18//ANK3//TMED2//MAL//VAMP1//NAPA//VPS16//ARCN1//SEC24C |
| GO:0048709 | oligodendrocyte differentiation | Biological process | 8 | 67 | 295 | 13692 | 5.54191753098912 | 9.14118971316176e-05 | 0.0166931463383916 | 4.03899727776324 | CD9//MRF//GSN//TCF7L2//PLP1//EGR1//RTN4//EXOC4 |
| GO:0048518 | positive regulation of biological process | Biological process | 95 | 3107 | 295 | 13692 | 1.41914648715585 | 9.51935808530541e-05 | 0.0166931463383916 | 4.0213923362173 | RGD1306565//KIT//MADD//LPAR1//PPP3CB//NSF//MAPK9//NPTN//SENP2//SQSTM1//EIF4G3//HIF1A//CLCF1//MAL//APAF1//PRUNE2//EI24//CR1L//PPP2R5C//RBPJ//ZFP91//ITGB1//RPS4X//ADAM10//CALR//CNBP//MGEA5//GFAP//EGR1//PLP1//TMED2//MKX//SPRY2//TMED9//ANK3//GADD45A//CNKSR3//AHCYL1//HSD17B12//TCF7L2//PRKCI//RGD1560691//CAT//RAPGEF4//NT5E//PLCB1//NFIA//ELF1//PICALM//MRF//PIAS1//RRN3//ILF2//ATMIN//SNAP25//PTP4A1//DSTN//INSIG1//INSIG2//DNAJA3//DNAJB2//GLUL//CAMKK1//STIM2//MLC1//TAF1//CD14//HSP90AB1//RTN4//SPTA1//ARL2BP//PDIA3//PLK2//ZDHHC17//MAP3K4//ZEB1//HNRNPK//CUL4A//SEMA7A//SDC4//PPP3CA//PAK2//NRP1//FAIM//ROBO1//SLC1A3//EXOC4//PLEKHA1//ITPR1//SLC24A2//SCN4B//NDRG1//TNKS2//FNDC5//RGD1304592 |
| GO:0051960 | regulation of nervous system development | Biological process | 24 | 480 | 295 | 13692 | 2.32067796610169 | 0.00011565000187848 | 0.0194862277772027 | 3.9368543558392 | HIF1A//GFAP//NRCAM//ITGB1//NPTN//PRKCI//RGD1560691//RTN4//LPAR1//RBPJ//NRP1//TCF7L2//MRF//S1PR5//CALR//ZEB1//SEMA7A//KIT//CLCF1//ROBO1//FAIM//CHN1//RAPGEF4//SNAP25 |
| GO:0060284 | regulation of cell development | Biological process | 25 | 512 | 295 | 13692 | 2.26628707627119 | 0.000120984831138131 | 0.0194862277772027 | 3.91726907733619 | HIF1A//GFAP//MAPK9//NRCAM//ITGB1//NPTN//PRKCI//RGD1560691//RTN4//LPAR1//RBPJ//NRP1//TCF7L2//MKX//PLCB1//S1PR5//CALR//ZEB1//SEMA7A//KIT//CLCF1//ROBO1//FAIM//CHN1//RAPGEF4 |
| GO:0042552 | myelination | Biological process | 9 | 89 | 295 | 13692 | 4.69350599885736 | 0.000124455834343448 | 0.0194862277772027 | 3.90498473936256 | PLP1//MRF//CD9//UGT8//TCF7L2//NDRG1//MAL//MPDZ//LPAR1 |
| GO:0019228 | regulation of action potential in neuron | Biological process | 10 | 111 | 295 | 13692 | 4.18140174072377 | 0.000140047250551179 | 0.0211712809109093 | 3.85372541295375 | PLP1//CLDN11//MRF//CD9//UGT8//TCF7L2//NDRG1//MAL//MPDZ//LPAR1 |
| GO:0050767 | regulation of neurogenesis | Biological process | 22 | 428 | 295 | 13692 | 2.38574370346903 | 0.000150917568746278 | 0.0220540873794561 | 3.82126019981732 | HIF1A//GFAP//NRCAM//ITGB1//NPTN//PRKCI//RGD1560691//RTN4//LPAR1//RBPJ//NRP1//S1PR5//CALR//ZEB1//SEMA7A//KIT//CLCF1//TCF7L2//ROBO1//FAIM//CHN1//RAPGEF4 |
| GO:0051050 | positive regulation of transport | Biological process | 24 | 492 | 295 | 13692 | 2.26407606448946 | 0.000168110493197299 | 0.0237740774895793 | 3.77440517772378 | MGEA5//CNKSR3//AHCYL1//PRKCI//SNAP25//GLUL//TCF7L2//STIM2//MAPK9//MLC1//HSP90AB1//RTN4//PPP3CB//HIF1A//ITGB1//CD14//HNRNPK//CALR//RGD1560691//ITPR1//SCN4B//ANK3//PLCB1//RGD1304592 |
| GO:0010720 | positive regulation of cell development | Biological process | 12 | 162 | 295 | 13692 | 3.43804143126177 | 0.00020260319878847 | 0.0270726110024698 | 3.69335370208919 | HIF1A//GFAP//MAPK9//TCF7L2//RTN4//SEMA7A//NPTN//KIT//CLCF1//FAIM//ROBO1//PRKCI |
| GO:0031646 | positive regulation of neurological system process | Biological process | 8 | 75 | 295 | 13692 | 4.95077966101695 | 0.000203785621140854 | 0.0270726110024698 | 3.6908264625253 | MRF//SLC1A3//GLUL//EGR1//GFAP//NPTN//PLK2//SLC24A2 |
| GO:0006633 | fatty acid biosynthetic process | Biological process | 10 | 117 | 295 | 13692 | 3.96697088222512 | 0.000216413452591089 | 0.0279046051811569 | 3.66471574631737 | FADS1//ELOVL7//MGST2//MAPK9//PLP1//INSIG1//INSIG2//MGLL//HSD17B12//SC5DL |
| GO:0016053 | organic acid biosynthetic process | Biological process | 15 | 241 | 295 | 13692 | 2.88881074618468 | 0.000234504082699522 | 0.0283055946273683 | 3.62984959184681 | SLC1A3//GLUL//MAT2B//GATM//MGLL//HSD17B12//SC5DL//FADS1//MGST2//ELOVL7//RGN//MAPK9//PLP1//INSIG1//INSIG2 |
| GO:0046394 | carboxylic acid biosynthetic process | Biological process | 15 | 241 | 295 | 13692 | 2.88881074618468 | 0.000234504082699522 | 0.0283055946273683 | 3.62984959184681 | SLC1A3//GLUL//MAT2B//GATM//MGLL//HSD17B12//SC5DL//FADS1//MGST2//ELOVL7//RGN//MAPK9//PLP1//INSIG1//INSIG2 |
| GO:0006903 | vesicle targeting | Biological process | 4 | 15 | 295 | 13692 | 12.3769491525424 | 0.000238893020349596 | 0.0283055946273683 | 3.62179653868853 | TMED9//KLHL12//TMED2//EXOC4 |
| GO:0048468 | cell development | Biological process | 48 | 1333 | 295 | 13692 | 1.67130596208374 | 0.000258625159696543 | 0.0298371763186749 | 3.58732922815683 | CHN2//HIF1A//SEMA3C//RBPJ//KIT//GOPC//SNAP25//CNP//ACTB//PICALM//ROBO1//NRP1//ANK3//NRCAM//RTN4//DNAJA3//CHN1//GFAP//MAPK9//ITGB1//NPTN//PRKCI//RGD1560691//LPAR1//CD9//MRF//GSN//TCF7L2//PLP1//PRICKLE2//PTPRK//NDRG1//ACTG1//MKX//PLCB1//S1PR5//CALR//ZEB1//SEMA7A//ARIH2//SLC1A3//CLCF1//PPP3CA//UGT8//FAIM//RAPGEF4//LRP4//NPTX1 |
| GO:0044237 | cellular metabolic process | Biological process | 175 | 6739 | 295 | 13692 | 1.20527865875589 | 0.000272811661032869 | 0.0301050884446319 | 3.56413707018128 | NFIA//POLA1//EGR1//ZEB1//CALR//MKX//PIAS1//DNAJA3//TCF7L2//PARN//UPF2//RGD1306565//KIT//MADD//LPAR1//TNKS2//ARIH2//HNRNPA1//HNRNPM//SRSF1//FKBP9//PHYH//EIF3D//NSF//LRP4//MAPK9//NPTN//SENP2//SQSTM1//EIF4G3//HSPA8//CANX//CCT4//HSPA9//CCT5//CCT2//DNAJA4//HSP90AB1//MESDC2//DNAJB2//PJA2//KLHL20//UAP1//GPCPD1//HIF1A//GLYR1//FADS1//GSTM2//MGST1//NT5E//ATP5B//PAICS//CTPS//SMC2//DDX1//APAF1//ANP32A//CNBP//DMTF1//HDGF//KEAP1//CNOT6//ZFP445//HNRPDL//ILF2//HDAC8//TSC22D1//HMGN5//RRN3//TAF1//ELF1//RNF141//RBPJ//GAR1//RGD1564051//AHCYL1//HNRPH1//HNRNPK//DDX46//HNRNPA3//CPSF3//AUH//RPS4X//EIF5//RPL5//EIF5B//EEF2//FMR1//PPP3CB//MAPK10//PAK2//ADAM10//CAMKK1//PLK2//PRKCI//TLK2//OXSR1//NEK7//GADD45A//PPM1B//PPP3CA//PTPRK//PTPN3//ST6GALNAC3//GALNT1//USP32//CUL4A//KLHL12//ADAMTS4//GLUL//SLC1A3//MAT2B//GATM//ACADSB//FAAH//SC5DL//MGLL//HSD17B12//HADHA//CAT//INSIG1//INSIG2//ETNK1//SERINC1//PSAP//ORMDL3//UGT8//MGST2//SLC25A13//SPTA1//LAMP1//PIK3CB//EI24//SPTBN1//ZFP91//PLEKHA1//ASGR2//CNP//SPRY2//RLF//NBR1//TMED2//PLCB1//PICALM//MRF//ATMIN//PDCD4//FOXN3//MGEA5//DDR1//ZDHHC17//ELOVL7//TSTA3//RGN//TRIM37//ACAP2//RGD1560691//CHN2//CNKSR3//UXS1//PTP4A1//PRIMA1//CLCF1//ARL2BP//PLP1//PPP2R5C//MAP3K4//CHN1//GLA//SDC4//CES1D//ITGB1//NRP1//HSPH1//DNAJC7 |
| GO:0048193 | Golgi vesicle transport | Biological process | 9 | 99 | 295 | 13692 | 4.21941448382126 | 0.000281019316164888 | 0.0301050884446319 | 3.5512638273707 | GOSR1//KLHL12//SEC24C//EXOC4//KRT18//SPTBN1//ANK3//PRKCI//TMED9 |
| GO:0060291 | long-term synaptic potentiation | Biological process | 5 | 28 | 295 | 13692 | 8.28813559322034 | 0.000293586292074776 | 0.0301050884446319 | 3.53226422605591 | EGR1//GFAP//NPTN//PLK2//SLC24A2 |
| GO:0035556 | intracellular signal transduction | Biological process | 53 | 1527 | 295 | 13692 | 1.61094868635743 | 0.000295059403059044 | 0.0301050884446319 | 3.53009054055189 | RGD1306565//PPP2R5C//PPP3CA//RCAN2//KIT//MAP3K4//OXSR1//FAIM//ZFP91//MAPK10//MAPK9//CLCF1//ARF1//ARF4//RASGRF2//RAPGEF4//DNAJA3//PLK2//PJA2//PLEKHA1//CAT//PIK3CB//GSN//ITPR1//NDRG1//TRIAP1//HIF1A//NBR1//ARHGEF26//LPAR1//ARL2BP//GADD45A//SQSTM1//ZDHHC17//PSAP//ITGB1//PDCD4//PLCB1//SPRY2//RGN//EXOC4//TCF7L2//CNKSR3//SEMA7A//MPDZ//PRKCI//CHN1//CHN2//PLCL1//TLK2//DCLK3//DDHD2 |
| GO:0031175 | neuron projection development | Biological process | 25 | 543 | 295 | 13692 | 2.13690420451353 | 0.000298148819005874 | 0.0301050884446319 | 3.5255669065941 | SNAP25//CNP//ACTB//PICALM//ROBO1//NRP1//SEMA3C//ANK3//NRCAM//RTN4//CHN1//ITGB1//NPTN//PRKCI//RGD1560691//GFAP//LPAR1//RBPJ//SEMA7A//UGT8//RAPGEF4//NPTX1//MAPK9//PRICKLE2//PTPRK |
| GO:0001508 | regulation of action potential | Biological process | 11 | 145 | 295 | 13692 | 3.52102863822326 | 0.000302149610301963 | 0.0301050884446319 | 3.5197779615473 | PLP1//CLDN11//MRF//CD9//UGT8//TCF7L2//NDRG1//MAL//MPDZ//LPAR1//SCN4B |
| GO:0031344 | regulation of cell projection organization | Biological process | 16 | 275 | 295 | 13692 | 2.70042526964561 | 0.000313752785916799 | 0.0305664936324277 | 3.50341240924661 | NRCAM//ITGB1//NPTN//PRKCI//RGD1560691//GFAP//RTN4//LPAR1//RBPJ//NRP1//KIT//SEMA7A//ROBO1//CHN1//RAPGEF4//ERMN |
| GO:0032502 | developmental process | Biological process | 111 | 3887 | 295 | 13692 | 1.32541936834211 | 0.000326932797966402 | 0.0311581170931458 | 3.48554150872118 | CAP1//NCL//HIF1A//RTN4//ATP5B//NRP1//NRCAM//RBPJ//KIT//PLEKHA1//TCF7L2//SDC4//CHN2//ITGB1//ADAM10//CR1L//TMED2//KEAP1//RRN3//TAPT1//SRSF1//SEMA3C//IFITM1//ERMN//LRP4//TET2//APAF1//UPF2//HSP90AB1//ROBO1//MKX//LUZP1//RPS4X//PTP4A1//DDX1//WIPF3//GOPC//GATM//FOXN3//DNAJA3//ZEB1//MOBP//SNAP25//CNP//ACTB//PICALM//ANK3//FMR1//MAPK9//CD9//NT5E//ARF4//NAPA//PPP3CB//RAPGEF4//TNXA-PS1//DDR1//CAT//GSTM2//CANX//FADS1//MGEA5//GSN//PDCD4//CHN1//SPTA1//CDC42SE2//PLP1//CLDN11//ITPR1//SENP2//GFAP//NPTN//PRKCI//RGD1560691//LPAR1//MRF//MAP3K4//SLC1A3//PLCB1//SQSTM1//CLCF1//EGR1//ASGR2//CLCN2//SPRY2//NBR1//UGT8//PRICKLE2//PTPRK//NDRG1//MGST1//PIK3CB//INSIG1//INSIG2//PLS3//MAL//MPDZ//ACTG1//S1PR5//CALR//RGD1309676//SEMA7A//ARIH2//PSAP//EXOC4//PPP3CA//FAIM//PIAS1//NPTX1//FNDC5 |
| GO:0010001 | glial cell differentiation | Biological process | 11 | 148 | 295 | 13692 | 3.44965643609711 | 0.00036046656918433 | 0.0336230944532788 | 3.44313500692794 | GFAP//CD9//MRF//GSN//TCF7L2//PLP1//NDRG1//RTN4//EGR1//EXOC4//CLCF1 |
| GO:0010975 | regulation of neuron projection development | Biological process | 14 | 228 | 295 | 13692 | 2.84995539696699 | 0.000431857962954319 | 0.038835984588275 | 3.36465906811154 | ITGB1//NPTN//PRKCI//RGD1560691//GFAP//RTN4//LPAR1//RBPJ//NRP1//SEMA7A//ROBO1//CHN1//RAPGEF4//NRCAM |
| GO:0048699 | generation of neurons | Biological process | 36 | 929 | 295 | 13692 | 1.79858787469669 | 0.000436880076974117 | 0.038835984588275 | 3.35963775995395 | HIF1A//SNAP25//CNP//ACTB//PICALM//ROBO1//NRP1//SEMA3C//ANK3//NRCAM//RTN4//CHN1//RBPJ//GFAP//ITGB1//NPTN//PRKCI//RGD1560691//LPAR1//NAPA//MAPK9//PRICKLE2//PTPRK//PLS3//S1PR5//CALR//ZEB1//SEMA7A//KIT//SLC1A3//CLCF1//TCF7L2//UGT8//FAIM//RAPGEF4//NPTX1 |
| GO:0023051 | regulation of signaling | Biological process | 58 | 1744 | 295 | 13692 | 1.54357020681076 | 0.000442928656344377 | 0.038835984588275 | 3.35366622107077 | KIT//MADD//LPAR1//RGD1306565//NSF//ZFP91//PPP3CB//ITGB1//PLCB1//GPR158//MGLL//CDC42SE2//PJA2//NAPA//CAT//ZEB1//SNAP25//LRP4//TCF7L2//HIF1A//MRF//GLUL//MGEA5//ACAP2//CHN2//NBR1//CALR//RTN4//SPRY2//RASGRF2//ARHGEF26//CLCF1//ARL2BP//SQSTM1//PLK2//ZDHHC17//DNAJA3//PSAP//MAP3K4//PDCD4//NPTN//MAPK9//PPP3CA//EGR1//RAPGEF4//SLC1A3//RGN//EXOC4//ELF1//GFAP//PLEKHA1//SLC24A2//HSP90AB1//ROBO1//CNKSR3//SEMA7A//TNKS2//PAK2 |
| GO:0032024 | positive regulation of insulin secretion | Biological process | 6 | 47 | 295 | 13692 | 5.92513523260007 | 0.000485602315504184 | 0.0417427559053008 | 3.31371925107552 | PPP3CB//HIF1A//GLUL//SNAP25//MGEA5//TCF7L2 |
| GO:0007009 | plasma membrane organization | Biological process | 8 | 86 | 295 | 13692 | 4.31754040204966 | 0.000522663764697956 | 0.0440645758545354 | 3.28177760768342 | NDRG1//GPR158//ANK3//RGD1304592//TMED2//SPTBN1//PRKCI//SPTA1 |
| GO:0014003 | oligodendrocyte development | Biological process | 5 | 32 | 295 | 13692 | 7.2521186440678 | 0.000560654222241893 | 0.0463756247228011 | 3.25130490286239 | PLP1//MRF//CD9//GSN//TCF7L2 |
| GO:0050806 | positive regulation of synaptic transmission | Biological process | 7 | 67 | 295 | 13692 | 4.84917783961548 | 0.000577712133819985 | 0.0465793264866614 | 3.23828851077653 | GLUL//EGR1//GFAP//NPTN//PLK2//SLC24A2//SLC1A3 |
| GO:0042063 | gliogenesis | Biological process | 12 | 182 | 295 | 13692 | 3.06023468057366 | 0.000586555200491136 | 0.0465793264866614 | 3.23169111033344 | PLP1//GFAP//CD9//MRF//GSN//TCF7L2//NDRG1//RTN4//EGR1//EXOC4//CLCF1//PRKCI |
| GO:0006468 | protein phosphorylation | Biological process | 36 | 945 | 295 | 13692 | 1.76813559322034 | 0.000599112413102636 | 0.0465793264866614 | 3.22249168210196 | RGD1306565//KIT//MADD//LPAR1//LRP4//MAPK9//NPTN//SENP2//SQSTM1//EIF4G3//GADD45A//DNAJA3//SPTBN1//ZFP91//MAPK10//SPRY2//PAK2//TAF1//DDR1//CAMKK1//CNKSR3//CLCF1//ARL2BP//MAP3K4//PDCD4//SDC4//ITGB1//NRP1//HSP90AB1//PPP3CB//ADAM10//PLK2//PRKCI//TLK2//OXSR1//NEK7 |
| GO:0050769 | positive regulation of neurogenesis | Biological process | 10 | 133 | 295 | 13692 | 3.48974130240856 | 0.000605616243097559 | 0.0465793264866614 | 3.21780248523679 | HIF1A//GFAP//RTN4//SEMA7A//NPTN//KIT//CLCF1//ROBO1//PRKCI//FAIM |
| GO:0032270 | positive regulation of cellular protein metabolic process | Biological process | 26 | 604 | 295 | 13692 | 1.99793467280278 | 0.000627944044759989 | 0.0469858911973704 | 3.20207905392945 | RGD1306565//KIT//MADD//LPAR1//MAPK9//NPTN//SENP2//SQSTM1//EIF4G3//ZFP91//RPS4X//DNAJA3//DNAJB2//CAMKK1//TAF1//SPRY2//PIAS1//CLCF1//ARL2BP//MAP3K4//SDC4//MGEA5//ITGB1//PAK2//NRP1//HSP90AB1 |
| GO:0021782 | glial cell development | Biological process | 7 | 68 | 295 | 13692 | 4.77786640079761 | 0.000632337495585049 | 0.0469858911973704 | 3.19905106515361 | GFAP//CD9//MRF//GSN//TCF7L2//PLP1//NDRG1 |
| GO:0044238 | primary metabolic process | Biological process | 177 | 6937 | 295 | 13692 | 1.18425832492432 | 0.000702480255443449 | 0.0513278906644013 | 3.15336587793173 | NFIA//POLA1//EGR1//ZEB1//CALR//MKX//PIAS1//DNAJA3//TCF7L2//PARN//UPF2//RGD1306565//KIT//MADD//LPAR1//TNKS2//ARIH2//HNRNPA1//HNRNPM//SRSF1//FKBP9//PHYH//EIF3D//LRP4//MAPK9//NPTN//SENP2//SQSTM1//EIF4G3//HSPA8//CANX//CCT4//HSPA9//CCT5//CCT2//DNAJA4//HSP90AB1//MESDC2//DNAJB2//PJA2//KLHL20//MRFAP1//UAP1//GPCPD1//GLYR1//HIF1A//GSTM2//MGST1//NT5E//ATP5B//PAICS//NSF//CTPS//SMC2//DDX1//APAF1//ANP32A//CNBP//DMTF1//HDGF//KEAP1//CNOT6//ZFP445//HNRPDL//ILF2//HDAC8//TSC22D1//HMGN5//RRN3//TAF1//ELF1//RNF141//RBPJ//GAR1//RGD1564051//AHCYL1//HNRPH1//HNRNPK//DDX46//HNRNPA3//CPSF3//AUH//RPS4X//EIF5//RPL5//EIF5B//EEF2//FMR1//PPP3CB//MAPK10//PAK2//ADAM10//CAMKK1//PLK2//PRKCI//TLK2//OXSR1//NEK7//GADD45A//PPM1B//PPP3CA//PTPRK//PTPN3//ST6GALNAC3//GALNT1//ADAMTS4//BLMH//LONRF1//SPPL2A//PPP2R5C//USP32//CUL4A//KLHL12//GLUL//SLC1A3//MAT2B//GATM//PLCL1//DDHD2//ACADSB//FAAH//SC5DL//MGLL//HSD17B12//HADHA//FADS1//CAT//INSIG1//INSIG2//ETNK1//SERINC1//PSAP//ORMDL3//UGT8//MGST2//SLC25A13//CR1L//SPTBN1//ZFP91//PLEKHA1//ASGR2//CNP//GLA//SPRY2//RLF//PLCB1//TMED2//PICALM//MRF//ATMIN//PDCD4//FOXN3//MGEA5//DDR1//ZDHHC17//ELOVL7//TSTA3//TRIM37//ACAP2//RGN//RGD1560691//CHN2//CNKSR3//UXS1//PTP4A1//PIK3CB//CLCF1//ARL2BP//PLP1//MAP3K4//CHN1//SDC4//CES1D//ITGB1//NRP1//HSPH1//DNAJC7 |
| GO:0006612 | protein targeting to membrane | Biological process | 6 | 51 | 295 | 13692 | 5.46041874376869 | 0.000757919136644588 | 0.054470778607375 | 3.12037712732625 | SRPR//TMED2//SPTBN1//ANK3//EXOC4//MGEA5 |
| GO:0006900 | membrane budding | Biological process | 4 | 20 | 295 | 13692 | 9.28271186440678 | 0.000778836832593155 | 0.0550713011949741 | 3.10855351809999 | PRKCI//TMED9//KLHL12//PICALM |
| GO:0051971 | positive regulation of transmission of nerve impulse | Biological process | 7 | 71 | 295 | 13692 | 4.57598472189067 | 0.000821168283049498 | 0.0565843877104022 | 3.08556783324518 | SLC1A3//GLUL//EGR1//GFAP//NPTN//PLK2//SLC24A2 |
| GO:0006605 | protein targeting | Biological process | 17 | 330 | 295 | 13692 | 2.39100154083205 | 0.00082604945562631 | 0.0565843877104022 | 3.08299395066674 | KEAP1//PPP3CA//KPNB1//PTTG1IP//CALR//HSPA9//AHCYL1//EXOC4//MGEA5//SRPR//YWHAZ//MAPK9//SPTBN1//HSP90AB1//TCF7L2//TMED2//ANK3 |
| GO:0070887 | cellular response to chemical stimulus | Biological process | 42 | 1182 | 295 | 13692 | 1.64921276778801 | 0.000855412689813724 | 0.0568977248887472 | 3.06782431139134 | KIT//GSTM2//GCGR//ADAM10//ZEB1//HIF1A//EGR1//INSIG1//PRKCI//KRT18//PTPRK//PPP3CA//PPP3CB//PLCB1//NRP1//SPRY2//TCF7L2//CAT//NPTN//CCL6//ELMO2//HSP90AB1//DNAJA3//ROBO1//PLEKHA1//RGD1306565//RPL5//GSN//CALR//ITGB1//PSAP//MAPK9//HSPA9//MLC1//MGST1//LPAR1//ITPR1//HIGD1A//NDRG1//APAF1//RGD1560691//RGD1304592 |
| GO:0008152 | metabolic process | Biological process | 190 | 7576 | 295 | 13692 | 1.16401481932239 | 0.000856580712284971 | 0.0568977248887472 | 3.06723170888238 | NFIA//POLA1//EGR1//ZEB1//CALR//MKX//PIAS1//DNAJA3//TCF7L2//PARN//UPF2//RGD1306565//KIT//MADD//LPAR1//TNKS2//ARIH2//HNRNPA1//HNRNPM//SRSF1//FKBP9//PHYH//EIF3D//NSF//LRP4//MAPK9//NPTN//SENP2//SQSTM1//EIF4G3//HSPA8//CANX//CCT4//HSPA9//CCT5//CCT2//DNAJA4//HSP90AB1//MESDC2//DNAJB2//PJA2//KLHL20//MRFAP1//MGEA5//UAP1//GPCPD1//HIF1A//GLYR1//FADS1//MGST1//RGD1309676//GSTM2//NT5E//ATP5B//PAICS//CTPS//SMC2//DDX1//APAF1//ANP32A//CNBP//DMTF1//HDGF//KEAP1//CNOT6//ZFP445//HNRPDL//ILF2//HDAC8//TSC22D1//HMGN5//RRN3//TAF1//ELF1//RNF141//RBPJ//GAR1//RGD1564051//AHCYL1//HNRPH1//HNRNPK//DDX46//HNRNPA3//CPSF3//AUH//RPS4X//EIF5//RPL5//EIF5B//EEF2//FMR1//PPP3CB//MAPK10//PAK2//ADAM10//CAMKK1//PLK2//PRKCI//TLK2//OXSR1//NEK7//GADD45A//PPM1B//PPP3CA//PTPRK//PTPN3//ST6GALNAC3//GALNT1//ADAMTS4//BLMH//LONRF1//SPPL2A//PPP2R5C//USP32//CUL4A//KLHL12//GLUL//SLC1A3//MAT2B//GATM//PLCL1//DDHD2//ACADSB//FAAH//SC5DL//MGLL//HSD17B12//HADHA//CAT//INSIG1//INSIG2//ETNK1//SERINC1//PDIA3//PDIA4//PSAP//ORMDL3//UGT8//MGST2//SLC25A13//SPTA1//LAMP1//PIK3CB//EI24//ROBO1//CR1L//SPTBN1//ZFP91//PLEKHA1//RGD1308874//ASGR2//CNP//GLA//PLP1//TMED2//SPRY2//RLF//PLCB1//NBR1//SIDT1//PICALM//MRF//ATMIN//PDCD4//FOXN3//DDR1//ZDHHC17//ELOVL7//TSTA3//RGN//TRIM37//ACAP2//RGD1560691//CHN2//CNKSR3//UXS1//PTP4A1//PRIMA1//CLCF1//ARL2BP//OAZ2//TRIAP1//MAP3K4//CHN1//SDC4//CES1D//ITGB1//NRP1//HSPH1//DNAJC7 |
| GO:0072657 | protein localization to membrane | Biological process | 8 | 93 | 295 | 13692 | 3.9925642427556 | 0.000881319320928556 | 0.0576672224321013 | 3.05486670885135 | LRP4//DNAJA3//GPR158//ANK3//RGD1304592//TMED2//SPTBN1//PRKCI |
| GO:0051325 | interphase | Biological process | 12 | 192 | 295 | 13692 | 2.90084745762712 | 0.000939904645088784 | 0.060099446158956 | 3.02691620407302 | TAF1//ITGB1//PPP3CA//PLK2//PAICS//POLA1//ANP32B//PLCB1//GADD45A//CUL4A//PRUNE2 |
| GO:0051650 | establishment of vesicle localization | Biological process | 4 | 21 | 295 | 13692 | 8.84067796610169 | 0.000945908253870429 | 0.060099446158956 | 3.02415098491964 | TMED2//EXOC4//TMED9//KLHL12 |
| GO:0006887 | exocytosis | Biological process | 12 | 193 | 295 | 13692 | 2.88581715991921 | 0.000983364079951607 | 0.0615866875215406 | 3.00728565954364 | YWHAZ//EXOC4//NAPA//PPP3CB//RAPGEF4//NSF//KIT//PPP3CA//PLCB1//GCGR//SNAP25//SCAMP1 |
| GO:0044283 | small molecule biosynthetic process | Biological process | 16 | 307 | 295 | 13692 | 2.41894771710926 | 0.00103393228939871 | 0.0638416782637175 | 2.98550790157416 | SLC1A3//GLUL//MAT2B//GATM//MGLL//HSD17B12//SC5DL//FADS1//ETNK1//MGST2//ELOVL7//RGN//MAPK9//PLP1//INSIG1//INSIG2 |
| GO:0006677 | glycosylceramide metabolic process | Biological process | 3 | 10 | 295 | 13692 | 13.9240677966102 | 0.00106178586377274 | 0.0646509614830513 | 2.9739630609937 | UGT8//GLA//ST6GALNAC3 |
| GO:0031644 | regulation of neurological system process | Biological process | 15 | 279 | 295 | 13692 | 2.49535265172225 | 0.00108084016270184 | 0.0649096338806146 | 2.96623852583307 | NAPA//TCF7L2//MRF//PLK2//EGR1//NPTN//KIT//PPP3CA//RAPGEF4//SLC1A3//GFAP//MGLL//RTN4//GLUL//SLC24A2 |
| GO:0061077 | chaperone-mediated protein folding | Biological process | 4 | 22 | 295 | 13692 | 8.43882896764253 | 0.00113667592895917 | 0.0673403685480676 | 2.94436333683027 | HSPA8//CCT4//HSPH1//DNAJC7 |
| GO:0090277 | positive regulation of peptide hormone secretion | Biological process | 6 | 56 | 295 | 13692 | 4.9728813559322 | 0.00124907351161711 | 0.0725507076008872 | 2.90341200137752 | GLUL//SNAP25//MGEA5//TCF7L2//PPP3CB//HIF1A |
| GO:0051648 | vesicle localization | Biological process | 5 | 38 | 295 | 13692 | 6.10704727921499 | 0.0012577221208183 | 0.0725507076008872 | 2.90041530064421 | TMED2//EXOC4//TCF7L2//TMED9//KLHL12 |
| GO:0051969 | regulation of transmission of nerve impulse | Biological process | 14 | 256 | 295 | 13692 | 2.53824152542373 | 0.00133832967411133 | 0.0744044117081984 | 2.87343689253866 | NAPA//TCF7L2//MRF//PLK2//EGR1//NPTN//KIT//PPP3CA//RAPGEF4//SLC1A3//GFAP//GLUL//SLC24A2//MGLL |
| GO:0044255 | cellular lipid metabolic process | Biological process | 26 | 637 | 295 | 13692 | 1.89443099273608 | 0.00135702165040289 | 0.0744044117081984 | 2.86741322339737 | PHYH//ACADSB//FAAH//SC5DL//MGLL//HSD17B12//HADHA//FADS1//CAT//INSIG1//INSIG2//ETNK1//SERINC1//PSAP//ORMDL3//ST6GALNAC3//UGT8//KIT//MGST2//ELOVL7//MAPK9//PIK3CB//PLP1//CES1D//GLA//PLCB1 |
| GO:0030182 | neuron differentiation | Biological process | 32 | 846 | 295 | 13692 | 1.7555956244741 | 0.00136774067993256 | 0.0744044117081984 | 2.86399623591269 | SNAP25//CNP//ACTB//PICALM//ROBO1//NRP1//SEMA3C//ANK3//NRCAM//RTN4//CHN1//RBPJ//ITGB1//NPTN//PRKCI//RGD1560691//GFAP//LPAR1//MAPK9//PRICKLE2//PTPRK//PLS3//S1PR5//CALR//ZEB1//SEMA7A//SLC1A3//UGT8//RAPGEF4//NPTX1//HIF1A//NAPA |
| GO:0007275 | multicellular organismal development | Biological process | 97 | 3429 | 295 | 13692 | 1.31295283004879 | 0.00137214428919325 | 0.0744044117081984 | 2.86260021756107 | NCL//HIF1A//RTN4//ATP5B//NRP1//NRCAM//RBPJ//KIT//PLEKHA1//TCF7L2//SDC4//ITGB1//ADAM10//CR1L//TMED2//KEAP1//RRN3//TAPT1//SRSF1//SEMA3C//IFITM1//LRP4//TET2//APAF1//UPF2//HSP90AB1//ROBO1//LUZP1//GATM//FOXN3//DNAJA3//ZEB1//MOBP//SNAP25//CNP//ACTB//PICALM//ANK3//FMR1//MAPK9//CD9//NT5E//ARF4//NAPA//PPP3CB//RAPGEF4//TNXA-PS1//MKX//DDR1//CHN1//PLP1//CLDN11//ITPR1//SENP2//GFAP//NPTN//PRKCI//RGD1560691//LPAR1//MRF//GSN//MAP3K4//SLC1A3//PLCB1//SPTA1//CLCF1//EGR1//ASGR2//CLCN2//SPRY2//NBR1//UGT8//PRICKLE2//PTPRK//NDRG1//MGST1//PIK3CB//INSIG1//INSIG2//PLS3//MAL//MPDZ//CAT//S1PR5//CALR//RGD1309676//SEMA7A//EXOC4//PPP3CA//FAIM//NPTX1//PSAP//ARIH2//RPS4X//PTP4A1//DDX1//WIPF3 |
| GO:0031401 | positive regulation of protein modification process | Biological process | 23 | 537 | 295 | 13692 | 1.98791781081337 | 0.00137471654844071 | 0.0744044117081984 | 2.86178683938885 | RGD1306565//KIT//MADD//LPAR1//MAPK9//NPTN//SENP2//SQSTM1//EIF4G3//ZFP91//DNAJA3//DNAJB2//CAMKK1//SPRY2//PIAS1//CLCF1//ARL2BP//MAP3K4//SDC4//ITGB1//PAK2//NRP1//HSP90AB1 |
| GO:0002793 | positive regulation of peptide secretion | Biological process | 6 | 58 | 295 | 13692 | 4.80140268848626 | 0.00150224658594 | 0.080315232106841 | 2.82325877430697 | GLUL//SNAP25//MGEA5//TCF7L2//PPP3CB//HIF1A |
| GO:0048666 | neuron development | Biological process | 26 | 644 | 295 | 13692 | 1.87383935151069 | 0.00158274775211483 | 0.0829666823595636 | 2.80058829460941 | SNAP25//CNP//ACTB//PICALM//ROBO1//NRP1//SEMA3C//ANK3//NRCAM//RTN4//CHN1//ITGB1//NPTN//PRKCI//RGD1560691//GFAP//LPAR1//RBPJ//MAPK9//PRICKLE2//PTPRK//SEMA7A//SLC1A3//UGT8//RAPGEF4//NPTX1 |
| GO:0048522 | positive regulation of cellular process | Biological process | 82 | 2813 | 295 | 13692 | 1.35297257888616 | 0.00160288556750688 | 0.0829666823595636 | 2.79509748149754 | RGD1306565//KIT//MADD//LPAR1//PPP3CB//NSF//MAPK9//NPTN//SENP2//SQSTM1//EIF4G3//HIF1A//MAL//APAF1//PRUNE2//EI24//PPP2R5C//RBPJ//ZFP91//ITGB1//RPS4X//ADAM10//CALR//CNBP//CLCF1//MGEA5//GFAP//TMED9//ANK3//GADD45A//HSD17B12//TCF7L2//EGR1//PRKCI//RGD1560691//CAT//RAPGEF4//PLCB1//NFIA//ELF1//PICALM//MRF//PIAS1//RRN3//ILF2//ATMIN//SNAP25//PTP4A1//DSTN//INSIG1//INSIG2//DNAJA3//DNAJB2//GLUL//CAMKK1//TAF1//SPRY2//RTN4//SPTA1//ARL2BP//PDIA3//PLK2//ZDHHC17//MAP3K4//ZEB1//HNRNPK//CUL4A//SEMA7A//CD14//SDC4//PPP3CA//PAK2//NRP1//FAIM//ROBO1//SLC1A3//EXOC4//SLC24A2//HSP90AB1//TNKS2//FNDC5//RGD1304592 |
| GO:0048869 | cellular developmental process | Biological process | 73 | 2442 | 295 | 13692 | 1.38746512305834 | 0.00160861496363205 | 0.0829666823595636 | 2.79354789572759 | CAP1//CHN2//ITGB1//HIF1A//SEMA3C//RBPJ//KIT//GOPC//SNAP25//CNP//ACTB//PICALM//ROBO1//NRP1//ANK3//NRCAM//RTN4//DNAJA3//CHN1//SPTA1//ERMN//CDC42SE2//PLP1//GFAP//MAPK9//NPTN//PRKCI//RGD1560691//LPAR1//CD9//MRF//GSN//TCF7L2//TET2//APAF1//SQSTM1//WIPF3//NAPA//CLCF1//EGR1//PPP3CB//ZEB1//UGT8//PRICKLE2//PTPRK//NDRG1//MGST1//PLEKHA1//MKX//PLS3//SPRY2//ACTG1//PLCB1//INSIG1//KEAP1//S1PR5//CALR//NBR1//ADAM10//RGD1309676//SEMA7A//ARIH2//SLC1A3//EXOC4//PPP3CA//FAIM//RAPGEF4//LRP4//PIAS1//NPTX1//CLCN2//PSAP//FNDC5 |
| GO:0051128 | regulation of cellular component organization | Biological process | 39 | 1110 | 295 | 13692 | 1.63074667888227 | 0.00162778398490867 | 0.0829791277888327 | 2.78840322867698 | DDR1//TLK2//CLASP2//SPTA1//ERMN//CDC42SE2//TMED9//MAPK9//NAPA//NRCAM//ITGB1//NPTN//PRKCI//RGD1560691//GFAP//RTN4//LPAR1//RBPJ//PICALM//ADAM10//EI24//DNAJB2//NRP1//DSTN//KIT//MGEA5//TCF7L2//SEMA7A//CD14//PLK2//HNRNPK//ROBO1//CALR//CHN1//RAPGEF4//SDC4//GSN//SNAP25//RRN3 |
| GO:0006665 | sphingolipid metabolic process | Biological process | 7 | 80 | 295 | 13692 | 4.06118644067797 | 0.00166456519166685 | 0.0838787793134192 | 2.77869919129929 | ORMDL3//ST6GALNAC3//UGT8//KIT//GLA//PSAP//SERINC1 |
| GO:0030154 | cell differentiation | Biological process | 69 | 2291 | 295 | 13692 | 1.39787673209094 | 0.00184520121627938 | 0.0908258263864343 | 2.73395626779494 | CHN2//ITGB1//HIF1A//SEMA3C//RBPJ//KIT//GOPC//SNAP25//CNP//ACTB//PICALM//ROBO1//NRP1//ANK3//NRCAM//RTN4//DNAJA3//CHN1//PLP1//GFAP//MAPK9//NPTN//PRKCI//RGD1560691//LPAR1//CD9//MRF//GSN//TCF7L2//TET2//NAPA//CLCF1//EGR1//PPP3CB//ZEB1//PRICKLE2//PTPRK//NDRG1//MGST1//PLEKHA1//MKX//PLS3//SPRY2//ACTG1//PLCB1//INSIG1//KEAP1//S1PR5//CALR//NBR1//ADAM10//RGD1309676//SEMA7A//ARIH2//SLC1A3//EXOC4//PPP3CA//UGT8//FAIM//RAPGEF4//LRP4//PIAS1//NPTX1//CLCN2//PSAP//FNDC5//APAF1//SQSTM1//WIPF3 |
| GO:0030030 | cell projection organization | Biological process | 29 | 756 | 295 | 13692 | 1.7804143126177 | 0.00186489729776768 | 0.0908258263864343 | 2.72934508033836 | SNAP25//CNP//ACTB//PICALM//ROBO1//NRP1//SEMA3C//ANK3//NRCAM//RTN4//CHN1//ITGB1//NPTN//PRKCI//RGD1560691//GFAP//LPAR1//RBPJ//MAPK9//PRICKLE2//PTPRK//KIT//ERMN//PLEKHA1//SEMA7A//UGT8//RAPGEF4//GSN//NPTX1 |
| GO:0001934 | positive regulation of protein phosphorylation | Biological process | 20 | 451 | 295 | 13692 | 2.05825096771769 | 0.0018654061101036 | 0.0908258263864343 | 2.7292266050472 | RGD1306565//KIT//MADD//LPAR1//ZFP91//CAMKK1//SPRY2//CLCF1//ARL2BP//MAP3K4//SDC4//ITGB1//PAK2//NRP1//HSP90AB1//MAPK9//NPTN//SENP2//SQSTM1//EIF4G3 |
| GO:0060292 | long term synaptic depression | Biological process | 3 | 12 | 295 | 13692 | 11.6033898305085 | 0.00188529885975491 | 0.0908258263864343 | 2.72461979513755 | MGLL//PLK2//SLC24A2 |
| GO:0072659 | protein localization to plasma membrane | Biological process | 6 | 61 | 295 | 13692 | 4.56526813003612 | 0.00195299839118879 | 0.0930646189888223 | 2.70929811446848 | ANK3//TMED2//SPTBN1//PRKCI//GPR158//RGD1304592 |
| GO:0045664 | regulation of neuron differentiation | Biological process | 17 | 358 | 295 | 13692 | 2.20399583372787 | 0.00198878296003129 | 0.0937508010406148 | 2.70141260973827 | NRCAM//ITGB1//NPTN//PRKCI//RGD1560691//GFAP//RTN4//LPAR1//RBPJ//NRP1//CALR//ZEB1//SEMA7A//ROBO1//CHN1//RAPGEF4//S1PR5 |
| GO:0071840 | cellular component organization or biogenesis | Biological process | 94 | 3342 | 295 | 13692 | 1.3054681556766 | 0.0020415340246195 | 0.0940702464910065 | 2.69004337764088 | RRN3//GAR1//RLF//CNP//SRSF1//CAP1//DDR1//TLK2//CHN2//EIF3D//MAPK9//HIF1A//RBPJ//CALR//SPTA1//SSX2IP//UGT8//NSF//RGD1304592//DNAJA3//APAF1//RGD1564051//CCT2//YWHAZ//NAPA//SPTBN1//ANK3//PRKCI//ERMN//CLASP2//TMED2//TMED9//PLK2//SMC2//GADD45A//GOPC//CD9//SNAP25//ACTB//PICALM//ROBO1//NRP1//SEMA3C//NRCAM//RTN4//CHN1//GSN//CDC42SE2//ITGB1//NPTN//RGD1560691//GFAP//LPAR1//HMGN5//MGEA5//TAF1//HSD17B12//OLFML2B//MKX//ADAM10//EI24//DNAJB2//DSTN//ZFP828//PRICKLE2//PTPRK//KIT//PLEKHA1//NDRG1//LSM14A//GLUL//LRP4//TCF7L2//EXOC4//ACTG1//SEMA7A//HSP90AB1//CD14//KLHL12//HNRNPK//SLC1A3//RAPGEF4//CAT//HAUS7//NBR1//MLC1//SQSTM1//SDC4//NPTX1//PIAS1//NELL2//MGST1//TRIM37//GPR158 |
| GO:0006796 | phosphate-containing compound metabolic process | Biological process | 43 | 1275 | 295 | 13692 | 1.56532003988036 | 0.00205850186032062 | 0.0940702464910065 | 2.68644873618332 | RGD1306565//KIT//MADD//LPAR1//LRP4//MAPK9//NPTN//SENP2//SQSTM1//EIF4G3//PPP3CB//MAPK10//PAK2//ADAM10//CAMKK1//PLK2//PRKCI//TLK2//OXSR1//NEK7//GADD45A//DNAJA3//PPM1B//PPP3CA//PTPRK//PTPN3//SPTBN1//ZFP91//SPRY2//RPL5//TAF1//DDR1//CNKSR3//PTP4A1//CLCF1//ARL2BP//MAP3K4//PDCD4//SDC4//PIK3CB//ITGB1//NRP1//HSP90AB1 |
| GO:0006643 | membrane lipid metabolic process | Biological process | 7 | 83 | 295 | 13692 | 3.91439656932816 | 0.00205993240491255 | 0.0940702464910065 | 2.68614703043431 | PSAP//SERINC1//ORMDL3//ST6GALNAC3//UGT8//KIT//GLA |
| GO:0032268 | regulation of cellular protein metabolic process | Biological process | 35 | 978 | 295 | 13692 | 1.66101694915254 | 0.00209912629188359 | 0.0941556531442924 | 2.67796143172814 | RGD1306565//KIT//MADD//LPAR1//LRP4//MAPK9//NPTN//SENP2//SQSTM1//EIF4G3//FMR1//EIF5B//GADD45A//DNAJA3//ZFP91//SPRY2//RPS4X//CALR//DNAJB2//CAMKK1//HSP90AB1//TAF1//CNKSR3//EGR1//PIAS1//CLCF1//ARL2BP//MAP3K4//PDCD4//SDC4//MGEA5//ITGB1//PAK2//NRP1//PTPN3 |
| GO:0006793 | phosphorus metabolic process | Biological process | 43 | 1277 | 295 | 13692 | 1.56286848147804 | 0.0021205190691125 | 0.0941556531442924 | 2.67355781772297 | RGD1306565//KIT//MADD//LPAR1//LRP4//MAPK9//NPTN//SENP2//SQSTM1//EIF4G3//PPP3CB//MAPK10//PAK2//ADAM10//CAMKK1//PLK2//PRKCI//TLK2//OXSR1//NEK7//GADD45A//DNAJA3//PPM1B//PPP3CA//PTPRK//PTPN3//SPTBN1//ZFP91//SPRY2//RPL5//TAF1//DDR1//CNKSR3//PTP4A1//CLCF1//ARL2BP//MAP3K4//PDCD4//SDC4//PIK3CB//ITGB1//NRP1//HSP90AB1 |
| GO:0006631 | fatty acid metabolic process | Biological process | 14 | 269 | 295 | 13692 | 2.41557557809842 | 0.00212623395558507 | 0.0941556531442924 | 2.67238895052325 | PHYH//MGLL//HSD17B12//SC5DL//ACADSB//HADHA//FADS1//MGST2//FAAH//ELOVL7//MAPK9//PLP1//INSIG1//INSIG2 |
| GO:0051329 | interphase of mitotic cell cycle | Biological process | 11 | 184 | 295 | 13692 | 2.77472365512159 | 0.00216004184852597 | 0.0946962346393785 | 2.66553783477136 | TAF1//ITGB1//PPP3CA//PLK2//PAICS//POLA1//ANP32B//PLCB1//GADD45A//CUL4A |
| GO:0019725 | cellular homeostasis | Biological process | 27 | 696 | 295 | 13692 | 1.80052600818235 | 0.00227957536236925 | 0.0989471127586811 | 2.64214604552384 | PDIA3//PDIA4//TXNDC15//ITGB1//RGN//SLC24A2//PIK3CB//STIM2//ARF1//HIF1A//ITPR1//NPTN//PLP1//CLDN11//MGEA5//MRF//CD9//UGT8//TCF7L2//NDRG1//PPP3CB//MAL//MPDZ//LPAR1//ATP5B//PPP3CA//SCN4B |
| GO:0071841 | cellular component organization or biogenesis at cellular level | Biological process | 77 | 2640 | 295 | 13692 | 1.35372881355932 | 0.00230750600610712 | 0.0991775130468001 | 2.63685715989434 | RRN3//GAR1//RLF//CNP//SRSF1//TLK2//CHN2//EIF3D//MAPK9//CALR//CAP1//SPTA1//SSX2IP//UGT8//NSF//RGD1304592//DNAJA3//APAF1//RGD1564051//CCT2//YWHAZ//PRKCI//ERMN//CLASP2//TMED2//TMED9//PLK2//SMC2//GADD45A//GOPC//SNAP25//ACTB//PICALM//ROBO1//NRP1//SEMA3C//ANK3//NRCAM//RTN4//CHN1//GSN//DDR1//NAPA//ITGB1//NPTN//RGD1560691//GFAP//LPAR1//RBPJ//HMGN5//MGEA5//TAF1//HSD17B12//OLFML2B//MKX//DSTN//CD9//ZFP828//PRICKLE2//PTPRK//KIT//PLEKHA1//LSM14A//TCF7L2//EXOC4//ACTG1//SEMA7A//KLHL12//RAPGEF4//LRP4//HAUS7//SDC4//NPTX1//PIAS1//TRIM37//SPTBN1//DNAJB2 |
| GO:0051085 | chaperone mediated protein folding requiring cofactor | Biological process | 3 | 13 | 295 | 13692 | 10.7108213820078 | 0.00241204650189037 | 0.102664192857159 | 2.6176143236796 | HSPA8//CCT4//HSPH1 |
| GO:0016043 | cellular component organization | Biological process | 91 | 3233 | 295 | 13692 | 1.30641320702292 | 0.00243912261879703 | 0.102818399623136 | 2.61276636642331 | RLF//CNP//SRSF1//CAP1//DDR1//TLK2//CHN2//EIF3D//MAPK9//HIF1A//RBPJ//CALR//SPTA1//SSX2IP//UGT8//NSF//RGD1304592//DNAJA3//APAF1//CCT2//YWHAZ//NAPA//RRN3//SPTBN1//ANK3//PRKCI//ERMN//CLASP2//TMED2//TMED9//PLK2//SMC2//GADD45A//GOPC//CD9//SNAP25//ACTB//PICALM//ROBO1//NRP1//SEMA3C//NRCAM//RTN4//CHN1//GSN//CDC42SE2//ITGB1//NPTN//RGD1560691//GFAP//LPAR1//HMGN5//MGEA5//TAF1//HSD17B12//OLFML2B//MKX//ADAM10//EI24//DNAJB2//DSTN//ZFP828//PRICKLE2//PTPRK//KIT//PLEKHA1//NDRG1//LSM14A//GLUL//LRP4//TCF7L2//ACTG1//SEMA7A//HSP90AB1//CD14//KLHL12//HNRNPK//SLC1A3//RAPGEF4//CAT//HAUS7//NBR1//MLC1//SQSTM1//SDC4//NPTX1//PIAS1//NELL2//MGST1//TRIM37//GPR158 |
| GO:0016310 | phosphorylation | Biological process | 37 | 1068 | 295 | 13692 | 1.60796038849743 | 0.00273339118658183 | 0.113535444254057 | 2.56329821023326 | RGD1306565//KIT//MADD//LPAR1//LRP4//MAPK9//NPTN//SENP2//SQSTM1//EIF4G3//PPP3CB//MAPK10//PAK2//ADAM10//CAMKK1//PLK2//PRKCI//TLK2//OXSR1//NEK7//GADD45A//DNAJA3//SPTBN1//ZFP91//SPRY2//TAF1//DDR1//CNKSR3//CLCF1//ARL2BP//MAP3K4//PDCD4//SDC4//PIK3CB//ITGB1//NRP1//HSP90AB1 |
| GO:0008219 | cell death | Biological process | 45 | 1372 | 295 | 13692 | 1.52231061916292 | 0.00276890010659157 | 0.113535444254057 | 2.55769271198235 | MAPK9//PDCD4//MADD//SQSTM1//FAIM//CCL6//GHITM//PRUNE2//KRT18//GSN//EI24//DNAJA3//PDCD6IP//APAF1//TCF7L2//TRIAP1//MAL//ROBO1//RGD1306565//LAMP1//TSTA3//MGEA5//EGR1//LPAR1//HIF1A//RGD1560691//PRKCI//RBPJ//KIT//PPP2R5C//ITGB1//PDIA3//CAT//PAK2//PLK2//HIGD1A//RRN3//KLHL20//SPRY2//HSP90AB1//CLCF1//DBC1 |
| GO:0006873 | cellular ion homeostasis | Biological process | 23 | 568 | 295 | 13692 | 1.87942229649081 | 0.00281368844683309 | 0.113535444254057 | 2.55072399265535 | ITGB1//RGN//SLC24A2//PIK3CB//STIM2//ARF1//HIF1A//ITPR1//NPTN//PLP1//CLDN11//MGEA5//MRF//CD9//UGT8//TCF7L2//NDRG1//MAL//MPDZ//LPAR1//ATP5B//PPP3CA//SCN4B |
| GO:0006897 | endocytosis | Biological process | 15 | 308 | 295 | 13692 | 2.26040061633282 | 0.00283544857954666 | 0.113535444254057 | 2.54737822417471 | CAP1//PICALM//ATP5B//CDC42SE2//ITGB1//CD14//HNRNPK//CANX//CALR//RGD1560691//MLC1//ASGR2//SCAMP1//LRP4//PIK3CB |
| GO:0016071 | mRNA metabolic process | Biological process | 13 | 248 | 295 | 13692 | 2.4329688354292 | 0.00284873802079562 | 0.113535444254057 | 2.54534748805581 | PARN//UPF2//HNRNPA1//HNRNPM//SRSF1//AHCYL1//DDX1//HNRNPK//HNRPH1//DDX46//HNRNPA3//CPSF3//AUH |
| GO:0042327 | positive regulation of phosphorylation | Biological process | 20 | 468 | 295 | 13692 | 1.98348544111256 | 0.00285971349550348 | 0.113535444254057 | 2.54367747510684 | RGD1306565//KIT//MADD//LPAR1//MAPK9//NPTN//SENP2//SQSTM1//EIF4G3//ZFP91//CAMKK1//SPRY2//CLCF1//ARL2BP//MAP3K4//SDC4//ITGB1//PAK2//NRP1//HSP90AB1 |
| GO:0006664 | glycolipid metabolic process | Biological process | 4 | 28 | 295 | 13692 | 6.63050847457627 | 0.00287464286318439 | 0.113535444254057 | 2.54141610303788 | ST6GALNAC3//UGT8//KIT//GLA |
| GO:0016265 | death | Biological process | 45 | 1376 | 295 | 13692 | 1.51788529759559 | 0.00292675218140989 | 0.114183463113505 | 2.53361404927875 | MAPK9//PDCD4//MADD//SQSTM1//FAIM//CCL6//GHITM//PRUNE2//KRT18//GSN//EI24//DNAJA3//PDCD6IP//APAF1//TCF7L2//TRIAP1//MAL//ROBO1//RGD1306565//LAMP1//DBC1//TSTA3//MGEA5//EGR1//LPAR1//HIF1A//RGD1560691//PRKCI//RBPJ//KIT//PPP2R5C//ITGB1//PDIA3//CAT//PAK2//PLK2//HIGD1A//RRN3//KLHL20//SPRY2//HSP90AB1//CLCF1 |
| GO:0051049 | regulation of transport | Biological process | 33 | 926 | 295 | 13692 | 1.65404693048285 | 0.00300485106158354 | 0.114183463113505 | 2.52217704936355 | KEAP1//PPP3CB//MGEA5//CNKSR3//AHCYL1//NAPA//PRKCI//SNAP25//NSF//RAPGEF4//PICALM//GLUL//TCF7L2//STIM2//MAPK9//MLC1//HSP90AB1//RTN4//SCN4B//HIF1A//ITGB1//CD14//PPP3CA//HNRNPK//CALR//RGD1560691//EXOC4//GFAP//CACNA2D3//ITPR1//ANK3//PLCB1//RGD1304592 |
| GO:0051247 | positive regulation of protein metabolic process | Biological process | 26 | 675 | 295 | 13692 | 1.78778154425612 | 0.00301388560106724 | 0.114183463113505 | 2.52087323635056 | RGD1306565//KIT//MADD//LPAR1//MAPK9//NPTN//SENP2//SQSTM1//EIF4G3//ZFP91//RPS4X//DNAJA3//DNAJB2//CAMKK1//TAF1//SPRY2//PIAS1//CLCF1//ARL2BP//MAP3K4//SDC4//MGEA5//ITGB1//PAK2//NRP1//HSP90AB1 |
| GO:0006901 | vesicle coating | Biological process | 3 | 14 | 295 | 13692 | 9.94576271186441 | 0.00302127776486464 | 0.114183463113505 | 2.51980934549713 | TMED9//KLHL12//PICALM |
| GO:0051318 | G1 phase | Biological process | 3 | 14 | 295 | 13692 | 9.94576271186441 | 0.00302127776486464 | 0.114183463113505 | 2.51980934549713 | TAF1//PLCB1//PRUNE2 |
| GO:0071842 | cellular component organization at cellular level | Biological process | 74 | 2544 | 295 | 13692 | 1.35007994883275 | 0.00309788715054339 | 0.115574692917746 | 2.50893440671527 | RLF//CNP//SRSF1//TLK2//CHN2//EIF3D//MAPK9//CALR//CAP1//SPTA1//SSX2IP//UGT8//NSF//RGD1304592//DNAJA3//APAF1//CCT2//YWHAZ//RRN3//PRKCI//ERMN//CLASP2//TMED2//TMED9//PLK2//SMC2//GADD45A//GOPC//SNAP25//ACTB//PICALM//ROBO1//NRP1//SEMA3C//ANK3//NRCAM//RTN4//CHN1//GSN//DDR1//NAPA//ITGB1//NPTN//RGD1560691//GFAP//LPAR1//RBPJ//HMGN5//MGEA5//TAF1//HSD17B12//OLFML2B//MKX//DSTN//CD9//ZFP828//PRICKLE2//PTPRK//KIT//PLEKHA1//LSM14A//TCF7L2//ACTG1//SEMA7A//KLHL12//RAPGEF4//LRP4//HAUS7//SDC4//NPTX1//PIAS1//TRIM37//SPTBN1//DNAJB2 |
| GO:0032989 | cellular component morphogenesis | Biological process | 29 | 783 | 295 | 13692 | 1.71902071563089 | 0.00311081518346123 | 0.115574692917746 | 2.50712578998008 | CAP1//CHN2//HIF1A//RBPJ//SNAP25//CNP//ACTB//PICALM//ROBO1//NRP1//SEMA3C//ANK3//NRCAM//RTN4//CHN1//SPTA1//ERMN//CDC42SE2//MAPK9//CD9//UGT8//GFAP//ITGB1//ACTG1//SEMA7A//SLC1A3//GSN//NPTX1 |
| GO:0010562 | positive regulation of phosphorus metabolic process | Biological process | 20 | 475 | 295 | 13692 | 1.9542551293488 | 0.00338245628876425 | 0.123572403082854 | 2.47076780704997 | RGD1306565//KIT//MADD//LPAR1//MAPK9//NPTN//SENP2//SQSTM1//EIF4G3//ZFP91//CAMKK1//SPRY2//CLCF1//ARL2BP//MAP3K4//SDC4//ITGB1//PAK2//NRP1//HSP90AB1 |
| GO:0045937 | positive regulation of phosphate metabolic process | Biological process | 20 | 475 | 295 | 13692 | 1.9542551293488 | 0.00338245628876425 | 0.123572403082854 | 2.47076780704997 | RGD1306565//KIT//MADD//LPAR1//MAPK9//NPTN//SENP2//SQSTM1//EIF4G3//ZFP91//CAMKK1//SPRY2//CLCF1//ARL2BP//MAP3K4//SDC4//ITGB1//PAK2//NRP1//HSP90AB1 |
| GO:0031399 | regulation of protein modification process | Biological process | 29 | 789 | 295 | 13692 | 1.70594831475156 | 0.00346825020415237 | 0.124829092043155 | 2.45988957948789 | RGD1306565//KIT//MADD//LPAR1//LRP4//MAPK9//NPTN//SENP2//SQSTM1//EIF4G3//GADD45A//DNAJA3//ZFP91//SPRY2//DNAJB2//CAMKK1//CNKSR3//EGR1//PIAS1//CLCF1//ARL2BP//MAP3K4//PDCD4//SDC4//ITGB1//PAK2//NRP1//MGEA5//HSP90AB1 |
| GO:0006629 | lipid metabolic process | Biological process | 32 | 898 | 295 | 13692 | 1.65393529878072 | 0.0034738022876973 | 0.124829092043155 | 2.45919490316167 | PHYH//ACADSB//FAAH//SC5DL//MGLL//HSD17B12//HADHA//FADS1//CAT//INSIG1//INSIG2//ETNK1//SERINC1//PSAP//ORMDL3//ST6GALNAC3//UGT8//KIT//MGST2//PLEKHA1//PLCB1//NT5E//ELOVL7//MAPK9//PIK3CB//PLP1//CES1D//GLA//PLCL1//ATP5B//GPCPD1//DDHD2 |
| GO:0043001 | Golgi to plasma membrane protein transport | Biological process | 3 | 15 | 295 | 13692 | 9.28271186440678 | 0.0037168739509934 | 0.132477848789879 | 2.42982216672677 | KRT18//SPTBN1//ANK3 |
| GO:0001932 | regulation of protein phosphorylation | Biological process | 25 | 655 | 295 | 13692 | 1.77150989778755 | 0.00405684026374593 | 0.143428933195663 | 2.39181209210821 | RGD1306565//KIT//MADD//LPAR1//MAPK9//NPTN//SENP2//SQSTM1//EIF4G3//GADD45A//DNAJA3//ZFP91//SPRY2//CAMKK1//CNKSR3//CLCF1//ARL2BP//MAP3K4//PDCD4//SDC4//ITGB1//PAK2//NRP1//HSP90AB1//LRP4 |
| GO:0048856 | anatomical structure development | Biological process | 97 | 3546 | 295 | 13692 | 1.26963205139235 | 0.00409484247076402 | 0.143614315134636 | 2.38776280095945 | CAP1//NCL//HIF1A//RTN4//ATP5B//NRP1//NRCAM//RBPJ//KIT//PLEKHA1//TCF7L2//SDC4//CHN2//ITGB1//ADAM10//CR1L//TMED2//KEAP1//RRN3//TAPT1//SRSF1//SEMA3C//IFITM1//ERMN//LRP4//TET2//APAF1//UPF2//HSP90AB1//ROBO1//MKX//LUZP1//GOPC//GATM//FOXN3//DNAJA3//MOBP//SNAP25//CNP//ACTB//PICALM//ANK3//FMR1//ZEB1//MAPK9//CD9//NT5E//ARF4//NAPA//PPP3CB//RAPGEF4//TNXA-PS1//DDR1//CHN1//SPTA1//CDC42SE2//PLP1//CLDN11//GFAP//NPTN//PRKCI//RGD1560691//LPAR1//MRF//GSN//SLC1A3//PLCB1//CLCF1//EGR1//ASGR2//CLCN2//SPRY2//NBR1//UGT8//PRICKLE2//PTPRK//NDRG1//MGST1//PIK3CB//INSIG1//INSIG2//PLS3//MAL//MPDZ//CAT//ACTG1//S1PR5//CALR//RGD1309676//SEMA7A//ARIH2//EXOC4//PPP3CA//FAIM//PIAS1//NPTX1//PSAP |
| GO:0009987 | cellular process | Biological process | 268 | 11723 | 295 | 13692 | 1.06106234737739 | 0.00433043009890545 | 0.150671472647631 | 2.36346896731753 | RRN3//GAR1//NFIA//POLA1//SLC25A5//RLF//TAF1//ITGB1//PPP3CA//PLK2//PAICS//ANP32B//PLCB1//GADD45A//EGR1//ZEB1//CALR//MKX//PIAS1//DNAJA3//TCF7L2//RGD1306565//PARN//UPF2//KIT//MADD//LPAR1//TNKS2//ARIH2//CNP//SPTBN1//ANK3//HNRNPA1//HNRNPM//SRSF1//FKBP9//CAP1//DSTN//DDR1//PHYH//TLK2//CHN2//EIF3D//HIF1A//SEMA3C//MAPK9//RBPJ//NSF//LRP4//NPTN//SENP2//SQSTM1//EIF4G3//PIK3CB//ROBO1//UGT8//YWHAZ//KEAP1//HSPA8//CANX//CCT4//HSPA9//CCT5//CCT2//DNAJA4//HSP90AB1//MESDC2//DNAJB2//GSN//SPTA1//SSX2IP//KPNB1//PTTG1IP//PDCD4//FAIM//CCL6//GHITM//PRUNE2//KRT18//EI24//PDCD6IP//SDC4//CD9//MPDZ//CLDN11//NRP1//PTPRK//NRCAM//KLHL12//CHN1//RAPGEF4//RIN2//ZDHHC17//PPP2R5C//PRKCI//PLCL1//DCLK3//DDHD2//RGD1304592//TMED2//EXOC4//PJA2//KLHL20//UAP1//GPCPD1//GLYR1//PDIA3//PDIA4//TXNDC15//FADS1//GSTM2//MGST1//NT5E//ATP5B//CTPS//SMC2//DDX1//APAF1//ANP32A//CNBP//DMTF1//HDGF//CNOT6//ZFP445//HNRPDL//ILF2//HDAC8//TSC22D1//HMGN5//ELF1//RNF141//RGD1564051//AHCYL1//HNRPH1//HNRNPK//DDX46//HNRNPA3//CPSF3//AUH//RPS4X//EIF5//RPL5//EIF5B//EEF2//FMR1//PPP3CB//MAPK10//PAK2//ADAM10//CAMKK1//OXSR1//NEK7//PPM1B//PTPN3//ST6GALNAC3//GALNT1//USP32//CUL4A//ADAMTS4//GLUL//SLC1A3//MAT2B//GATM//MGEA5//SRPR//KDELR3//ACADSB//FAAH//SC5DL//MGLL//HSD17B12//HADHA//CAT//INSIG1//INSIG2//ETNK1//SERINC1//PSAP//ORMDL3//MGST2//SLC25A13//RGN//SLC24A2//STIM2//ARF1//MAL//VAMP1//NAPA//VPS16//ARCN1//SEC24C//GCGR//SNAP25//SCAMP1//GOSR1//LAMP1//TRIAP1//ERMN//ACTR10//CLASP2//TMED9//PTP4A1//DBC1//SESN1//CDH19//CLCF1//ARF4//S1PR5//ITPR1//PLP1//SEMA7A//RCAN2//MAP3K4//ZFP91//RASGRF2//GOPC//ACTB//PICALM//RTN4//PLEKHA1//TSTA3//GPR158//SPRY2//CDC42SE2//ASGR2//GFAP//RGD1560691//MRF//NBR1//SIDT1//ATMIN//FOXN3//ELOVL7//HABP4//TET2//WIPF3//OLFML2B//NDRG1//ZFP828//PRICKLE2//TRIM37//ACAP2//MLC1//CNKSR3//UXS1//LSM14A//SCN4B//ARHGEF26//FCGRT//PRIMA1//HIGD1A//PLS3//ARL2BP//GLA//ACTG1//RGD1309676//CD14//CES1D//HSPH1//HAUS7//CACNA2D3//HIATL1//ELMO2//NPTX1//CLCN2//DNAJC7//FNDC5 |
| GO:0055082 | cellular chemical homeostasis | Biological process | 24 | 624 | 295 | 13692 | 1.7851368970013 | 0.00439835316946704 | 0.151829766101917 | 2.35670990153442 | ITGB1//RGN//SLC24A2//PIK3CB//STIM2//ARF1//HIF1A//ITPR1//NPTN//PLP1//CLDN11//MGEA5//MRF//CD9//UGT8//TCF7L2//NDRG1//PPP3CB//MAL//MPDZ//LPAR1//ATP5B//PPP3CA//SCN4B |
| GO:0072661 | protein targeting to plasma membrane | Biological process | 3 | 16 | 295 | 13692 | 8.70254237288136 | 0.00450234942227872 | 0.15419705615546 | 2.34656080292086 | TMED2//SPTBN1//ANK3 |
| GO:0042391 | regulation of membrane potential | Biological process | 13 | 262 | 295 | 13692 | 2.30296286712382 | 0.00453727651552335 | 0.15419705615546 | 2.34320475269198 | PLP1//CLDN11//MRF//CD9//UGT8//TCF7L2//NDRG1//MAL//MPDZ//LPAR1//MGEA5//PPP3CA//SCN4B |
| GO:0006397 | mRNA processing | Biological process | 11 | 203 | 295 | 13692 | 2.51502045587376 | 0.00458400128830147 | 0.154586628060874 | 2.33875526898523 | PARN//HNRNPA1//HNRNPM//SRSF1//AHCYL1//CPSF3//DDX1//HNRNPK//HNRPH1//DDX46//HNRNPA3 |
| GO:0055002 | striated muscle cell development | Biological process | 8 | 121 | 295 | 13692 | 3.06866507914274 | 0.00464938382588076 | 0.154797963523988 | 2.33260459952971 | DNAJA3//ANK3//ITGB1//ACTG1//MKX//PLCB1//PPP3CA//LRP4 |
| GO:0071347 | cellular response to interleukin-1 | Biological process | 5 | 51 | 295 | 13692 | 4.55034895314058 | 0.00469020008633025 | 0.154797963523988 | 2.32880862966539 | EGR1//PLCB1//HIF1A//MAPK9//HSPA9 |
| GO:0050804 | regulation of synaptic transmission | Biological process | 12 | 233 | 295 | 13692 | 2.39039790499745 | 0.00469619734231077 | 0.154797963523988 | 2.32825366157393 | NAPA//PLK2//EGR1//NPTN//KIT//RAPGEF4//SLC1A3//GFAP//GLUL//SLC24A2//MGLL//PPP3CA |
| GO:0044267 | cellular protein metabolic process | Biological process | 79 | 2799 | 295 | 13692 | 1.30999327847112 | 0.00489225414131514 | 0.160057031011385 | 2.31049099045342 | RGD1306565//KIT//MADD//LPAR1//TNKS2//ARIH2//FKBP9//EIF3D//LRP4//MAPK9//NPTN//SENP2//SQSTM1//EIF4G3//HSPA8//CANX//CCT4//CALR//HSPA9//CCT5//CCT2//DNAJA4//HSP90AB1//MESDC2//DNAJA3//DNAJB2//PJA2//KLHL20//RPS4X//EIF5//RPL5//RGD1564051//EIF5B//EEF2//FMR1//PPP3CB//MAPK10//PAK2//ADAM10//CAMKK1//PLK2//PRKCI//TLK2//OXSR1//NEK7//GADD45A//PPM1B//PPP3CA//PTPRK//PTPN3//ST6GALNAC3//GALNT1//USP32//CUL4A//KLHL12//SPTBN1//ZFP91//SPRY2//TCF7L2//MGEA5//TAF1//DDR1//ZDHHC17//CNKSR3//EGR1//PIAS1//PTP4A1//PLCB1//CLCF1//ARL2BP//PPP2R5C//MAP3K4//PDCD4//SDC4//ITGB1//NRP1//HSPH1//TRIM37//DNAJC7 |
| GO:0031345 | negative regulation of cell projection organization | Biological process | 6 | 74 | 295 | 13692 | 3.7632615666514 | 0.00516620704532716 | 0.166534203578781 | 2.28682819265724 | GFAP//RTN4//LPAR1//RBPJ//NRP1//ITGB1 |
| GO:0071453 | cellular response to oxygen levels | Biological process | 6 | 74 | 295 | 13692 | 3.7632615666514 | 0.00516620704532716 | 0.166534203578781 | 2.28682819265724 | EGR1//ITPR1//HIF1A//HIGD1A//NDRG1//LPAR1 |
| GO:0048583 | regulation of response to stimulus | Biological process | 57 | 1898 | 295 | 13692 | 1.39387401546677 | 0.00527294073245369 | 0.168498067113649 | 2.27794711008575 | KIT//MADD//LPAR1//RGD1306565//PPP3CB//CR1L//ZFP91//ITGB1//PLCB1//GPR158//MGLL//CDC42SE2//PJA2//ADAM10//CAT//ZEB1//LRP4//TCF7L2//HIF1A//ACAP2//CHN2//NBR1//CALR//SPRY2//RASGRF2//ARHGEF26//FCGRT//CLCF1//ARL2BP//SQSTM1//PLK2//ZDHHC17//DNAJA3//PSAP//MAP3K4//PDCD4//NPTN//MAPK9//MLC1//RTN4//NRP1//SEMA7A//NT5E//SPPL2A//RGN//EXOC4//PLEKHA1//ELF1//ROBO1//RAPGEF4//HSP90AB1//CNKSR3//RGD1560691//EGR1//TNKS2//PAK2//RGD1304592 |
| GO:0006458 | 'de novo' protein folding | Biological process | 3 | 17 | 295 | 13692 | 8.19062811565304 | 0.005380868931549 | 0.168498067113649 | 2.26914758647019 | HSPA8//CCT4//HSPH1 |
| GO:0006893 | Golgi to plasma membrane transport | Biological process | 3 | 17 | 295 | 13692 | 8.19062811565304 | 0.005380868931549 | 0.168498067113649 | 2.26914758647019 | KRT18//SPTBN1//ANK3 |
| GO:0051084 | 'de novo' posttranslational protein folding | Biological process | 3 | 17 | 295 | 13692 | 8.19062811565304 | 0.005380868931549 | 0.168498067113649 | 2.26914758647019 | HSPA8//CCT4//HSPH1 |
| GO:0032507 | maintenance of protein location in cell | Biological process | 6 | 75 | 295 | 13692 | 3.71308474576271 | 0.00551637521068571 | 0.171274095927765 | 2.25834620181663 | KEAP1//KDELR3//ZFP828//CLASP2//ARL2BP//ANK3 |
| GO:0065008 | regulation of biological quality | Biological process | 62 | 2106 | 295 | 13692 | 1.36640108165532 | 0.00554765547941209 | 0.171274095927765 | 2.25589051732408 | ITGB1//HIF1A//SPTA1//YWHAZ//KEAP1//PDIA3//PDIA4//TXNDC15//KDELR3//HSD17B12//RGN//SLC24A2//PIK3CB//STIM2//ARF1//DNAJA3//ITPR1//NPTN//SNAP25//NSF//DDR1//PPP3CB//PLEKHA1//ERMN//CDC42SE2//PLP1//CLDN11//MGEA5//NAPA//CALR//MRF//RAPGEF4//KIT//RTN4//NRP1//DSTN//CD9//UGT8//ZFP828//TCF7L2//ASGR2//GLUL//NDRG1//CLASP2//PRIMA1//MAL//MPDZ//LPAR1//SEMA7A//HSP90AB1//PPP3CA//CNP//PLK2//EGR1//RRN3//ATP5B//ARL2BP//GFAP//GSN//MGLL//SCN4B//ANK3 |
| GO:0044248 | cellular catabolic process | Biological process | 40 | 1232 | 295 | 13692 | 1.50693374422188 | 0.00569480882253321 | 0.174587705440459 | 2.24452085081447 | PARN//UPF2//ARIH2//PHYH//HIF1A//NT5E//NSF//ATP5B//APAF1//AUH//USP32//CUL4A//ACADSB//HADHA//LAMP1//PIK3CB//EI24//FAAH//CNP//SQSTM1//NBR1//TMED2//MGLL//DNAJB2//ACAP2//HSP90AB1//TAF1//RGN//CHN2//SPRY2//PRIMA1//GSTM2//CAT//KLHL20//PPP2R5C//PLCB1//CHN1//CES1D//GLA//PTPN3 |
| GO:0012501 | programmed cell death | Biological process | 42 | 1310 | 295 | 13692 | 1.48806831414154 | 0.00574073954978855 | 0.174773626293562 | 2.24103215608578 | MAPK9//PDCD4//MADD//SQSTM1//FAIM//CCL6//GHITM//PRUNE2//KRT18//GSN//EI24//DNAJA3//PDCD6IP//APAF1//TCF7L2//TRIAP1//MAL//ROBO1//RGD1306565//LAMP1//HIF1A//RGD1560691//PRKCI//RBPJ//KIT//PPP2R5C//ITGB1//PDIA3//LPAR1//CAT//PAK2//PLK2//HIGD1A//RRN3//KLHL20//SPRY2//HSP90AB1//CLCF1//EGR1 |
| GO:0033365 | protein localization to organelle | Biological process | 15 | 334 | 295 | 13692 | 2.08444128691769 | 0.00597997273164591 | 0.18080138245197 | 2.22330079636656 | KEAP1//PPP3CA//KPNB1//PTTG1IP//SRPR//KDELR3//YWHAZ//MAPK9//SPTBN1//HSP90AB1//ZFP828//CALR//TCF7L2//ARL2BP//TNKS2 |
| GO:0016050 | vesicle organization | Biological process | 6 | 77 | 295 | 13692 | 3.61664098613251 | 0.00626832761414305 | 0.188221563427419 | 2.20284831320914 | NSF//RGD1304592//PRKCI//TMED9//KLHL12//PICALM |
| GO:0009966 | regulation of signal transduction | Biological process | 46 | 1477 | 295 | 13692 | 1.44551369587919 | 0.00654141742643708 | 0.19508553739796 | 2.18432813641858 | KIT//MADD//LPAR1//RGD1306565//ZFP91//ITGB1//PLCB1//GPR158//PJA2//CAT//ZEB1//LRP4//TCF7L2//HIF1A//ACAP2//CHN2//NBR1//CALR//SPRY2//RASGRF2//ARHGEF26//CLCF1//ARL2BP//SQSTM1//PLK2//ZDHHC17//DNAJA3//PSAP//MAP3K4//PDCD4//NPTN//MAPK9//RGN//EXOC4//ELF1//RAPGEF4//PLEKHA1//HSP90AB1//ROBO1//CNKSR3//SEMA7A//EGR1//TNKS2//MGLL//PAK2//CDC42SE2 |
| GO:0010627 | regulation of intracellular protein kinase cascade | Biological process | 21 | 540 | 295 | 13692 | 1.80497175141243 | 0.00666603668343909 | 0.197458816352682 | 2.17613230073079 | PJA2//NBR1//KIT//CLCF1//ARL2BP//SQSTM1//PLK2//LPAR1//ZDHHC17//DNAJA3//PSAP//ITGB1//MAP3K4//PDCD4//MAPK9//PLCB1//SPRY2//TCF7L2//PLEKHA1//CNKSR3//SEMA7A |
| GO:0010604 | positive regulation of macromolecule metabolic process | Biological process | 49 | 1599 | 295 | 13692 | 1.42230419435876 | 0.00677944348189347 | 0.19859005690728 | 2.16880595549321 | RGD1306565//KIT//MADD//LPAR1//NSF//MAPK9//NPTN//SENP2//SQSTM1//EIF4G3//RBPJ//ZFP91//EGR1//PLP1//CALR//TMED2//MKX//SPRY2//HIF1A//TCF7L2//RPS4X//PLCB1//NFIA//CNBP//ELF1//PICALM//MRF//PIAS1//RRN3//ILF2//ATMIN//INSIG1//INSIG2//DNAJA3//DNAJB2//CAMKK1//TAF1//CLCF1//ARL2BP//MAP3K4//HNRNPK//SDC4//MGEA5//PPP3CA//ZEB1//ITGB1//PAK2//NRP1//HSP90AB1 |
| GO:0030073 | insulin secretion | Biological process | 8 | 129 | 295 | 13692 | 2.87836026803311 | 0.00680247124264211 | 0.19859005690728 | 2.16733328551855 | PPP3CB//GLUL//SNAP25//MGEA5//TCF7L2//HIF1A//PPP3CA//RAPGEF4 |
| GO:0050796 | regulation of insulin secretion | Biological process | 7 | 103 | 295 | 13692 | 3.15431956557512 | 0.00684012285424254 | 0.19859005690728 | 2.16493609792309 | GLUL//SNAP25//MGEA5//TCF7L2//PPP3CB//HIF1A//PPP3CA |
| GO:0044260 | cellular macromolecule metabolic process | Biological process | 130 | 5065 | 295 | 13692 | 1.19126608329011 | 0.0069135553635438 | 0.199401491538 | 2.1602985549887 | NFIA//POLA1//EGR1//ZEB1//CALR//MKX//PIAS1//DNAJA3//TCF7L2//PARN//UPF2//RGD1306565//KIT//MADD//LPAR1//TNKS2//ARIH2//HNRNPA1//HNRNPM//SRSF1//FKBP9//EIF3D//NSF//LRP4//MAPK9//NPTN//SENP2//SQSTM1//EIF4G3//HSPA8//CANX//CCT4//HSPA9//CCT5//CCT2//DNAJA4//HSP90AB1//MESDC2//DNAJB2//PJA2//KLHL20//SMC2//DDX1//APAF1//ANP32A//HIF1A//CNBP//DMTF1//HDGF//KEAP1//CNOT6//ZFP445//HNRPDL//ILF2//HDAC8//TSC22D1//HMGN5//RRN3//TAF1//ELF1//RNF141//RBPJ//GAR1//RGD1564051//AHCYL1//HNRPH1//HNRNPK//DDX46//HNRNPA3//CPSF3//AUH//RPS4X//EIF5//RPL5//EIF5B//EEF2//FMR1//PPP3CB//MAPK10//PAK2//ADAM10//CAMKK1//PLK2//PRKCI//TLK2//OXSR1//NEK7//GADD45A//PPM1B//PPP3CA//PTPRK//PTPN3//ST6GALNAC3//GALNT1//USP32//CUL4A//KLHL12//ADAMTS4//SPTBN1//ZFP91//ASGR2//SPRY2//RLF//CNP//PLCB1//PICALM//MRF//ATMIN//PDCD4//FOXN3//MGEA5//DDR1//ZDHHC17//INSIG1//INSIG2//CAT//TRIM37//RGD1560691//CNKSR3//PTP4A1//CLCF1//ARL2BP//PPP2R5C//MAP3K4//MAT2B//SDC4//ITGB1//NRP1//HSPH1//DNAJC7 |
| GO:0070555 | response to interleukin-1 | Biological process | 6 | 79 | 295 | 13692 | 3.52508045483802 | 0.00709221420591139 | 0.202706522756206 | 2.14921815589114 | EGR1//PLCB1//HIF1A//MAPK9//HSPA9//PRKCI |
| GO:0051246 | regulation of protein metabolic process | Biological process | 37 | 1133 | 295 | 13692 | 1.51571199904259 | 0.00712062146543243 | 0.202706522756206 | 2.14748210085336 | RGD1306565//KIT//MADD//LPAR1//LRP4//MAPK9//NPTN//SENP2//SQSTM1//EIF4G3//FMR1//EIF5B//GADD45A//DNAJA3//ZFP91//SPRY2//RPS4X//CALR//CR1L//DNAJB2//CAMKK1//HSP90AB1//TAF1//CNKSR3//EGR1//PIAS1//CLCF1//ARL2BP//MAP3K4//PDCD4//SDC4//MGEA5//ITGB1//PAK2//NRP1//PTPN3//CUL4A |
| GO:0010977 | negative regulation of neuron projection development | Biological process | 4 | 36 | 295 | 13692 | 5.15706214689266 | 0.00722885537187487 | 0.204460012582577 | 2.14093046412079 | RTN4//GFAP//LPAR1//RBPJ |
| GO:0032879 | regulation of localization | Biological process | 40 | 1252 | 295 | 13692 | 1.48286132019278 | 0.00742334833797276 | 0.208615122523542 | 2.12940015985551 | KEAP1//PPP3CB//MGEA5//HIF1A//CNKSR3//AHCYL1//NAPA//ADAM10//RAPGEF4//RBPJ//PRKCI//SNAP25//NSF//PICALM//RTN4//ITGB1//PTP4A1//KIT//PTPRK//GLUL//TCF7L2//STIM2//MAPK9//MLC1//HSP90AB1//SCN4B//CD14//PPP3CA//HNRNPK//NRP1//CALR//RGD1560691//ITPR1//EXOC4//GFAP//CACNA2D3//LPAR1//ANK3//PLCB1//RGD1304592 |
| GO:0045595 | regulation of cell differentiation | Biological process | 32 | 947 | 295 | 13692 | 1.56835680919228 | 0.00754812079676782 | 0.210770455879173 | 2.12216115819578 | HIF1A//GFAP//MAPK9//NRCAM//ITGB1//NPTN//PRKCI//RGD1560691//RTN4//LPAR1//RBPJ//NRP1//ZEB1//KIT//TCF7L2//INSIG1//KEAP1//MKX//PLCB1//S1PR5//CALR//NBR1//ADAM10//RGD1309676//SEMA7A//CLCF1//ROBO1//FAIM//CHN1//RAPGEF4//PIAS1//FNDC5 |
| GO:0009893 | positive regulation of metabolic process | Biological process | 52 | 1731 | 295 | 13692 | 1.39428370002644 | 0.00774930024774709 | 0.215018558772932 | 2.11073751197918 | RGD1306565//KIT//MADD//LPAR1//NSF//MAPK9//NPTN//SENP2//SQSTM1//EIF4G3//RBPJ//ZFP91//EGR1//PLP1//CALR//TMED2//MKX//SPRY2//GADD45A//HIF1A//TCF7L2//RPS4X//NT5E//PLCB1//NFIA//CNBP//ELF1//PICALM//MRF//PIAS1//RRN3//ILF2//ATMIN//INSIG1//INSIG2//DNAJA3//DNAJB2//CAMKK1//TAF1//CLCF1//ARL2BP//MAP3K4//HNRNPK//SDC4//MGEA5//PPP3CA//ZEB1//ITGB1//PAK2//NRP1//RGD1560691//HSP90AB1 |
| GO:0006611 | protein export from nucleus | Biological process | 4 | 37 | 295 | 13692 | 5.01768208886853 | 0.00797034629185793 | 0.218973835028057 | 2.09852280917153 | TCF7L2//CALR//HSPA9//AHCYL1 |
| GO:0046887 | positive regulation of hormone secretion | Biological process | 6 | 81 | 295 | 13692 | 3.43804143126177 | 0.0079917458039437 | 0.218973835028057 | 2.09735833830813 | GLUL//SNAP25//MGEA5//TCF7L2//PPP3CB//HIF1A |
| GO:0014015 | positive regulation of gliogenesis | Biological process | 4 | 38 | 295 | 13692 | 4.88563782337199 | 0.00876006678256848 | 0.238534986178759 | 2.05749258296543 | GFAP//RTN4//CLCF1//PRKCI |
| GO:0050801 | ion homeostasis | Biological process | 23 | 626 | 295 | 13692 | 1.70529051822169 | 0.00893623044592346 | 0.241829841203262 | 2.04884564023908 | ITGB1//RGN//SLC24A2//PIK3CB//STIM2//ARF1//HIF1A//ITPR1//NPTN//PLP1//CLDN11//MGEA5//MRF//CD9//UGT8//TCF7L2//NDRG1//MAL//MPDZ//LPAR1//ATP5B//PPP3CA//SCN4B |
| GO:0007049 | cell cycle | Biological process | 31 | 923 | 295 | 13692 | 1.55885193822649 | 0.00924165736653695 | 0.248560895060724 | 2.03425013694053 | TAF1//ITGB1//PPP3CA//PLK2//PAICS//POLA1//ANP32B//PLCB1//GADD45A//SPTBN1//ANK3//HSPA8//MADD//PPP2R5C//CALR//DBC1//SESN1//TCF7L2//SMC2//CLASP2//EIF4G3//CUL4A//ZFP828//PTPRK//PTPN3//HAUS7//PRUNE2//PTP4A1//DMTF1//PDCD6IP |
| GO:0048741 | skeletal muscle fiber development | Biological process | 6 | 84 | 295 | 13692 | 3.31525423728814 | 0.00949089395008188 | 0.253707799250969 | 2.0226928793216 | DNAJA3//ANK3//MKX//PLCB1//LRP4//PPP3CA |
| GO:0050805 | negative regulation of synaptic transmission | Biological process | 4 | 39 | 295 | 13692 | 4.76036505867014 | 0.00959920408793137 | 0.255047943766613 | 2.01776477472597 | MGLL//PLK2//SLC24A2//RAPGEF4 |
| GO:0060627 | regulation of vesicle-mediated transport | Biological process | 11 | 225 | 295 | 13692 | 2.26910734463277 | 0.00967250511603992 | 0.255447364028428 | 2.01446103189699 | NAPA//NSF//RAPGEF4//PICALM//ITGB1//CD14//PPP3CA//HNRNPK//CALR//RGD1560691//PLCB1 |
| GO:0022402 | cell cycle process | Biological process | 24 | 667 | 295 | 13692 | 1.6700531090387 | 0.00974574933640308 | 0.255840509525695 | 2.01118476299545 | TAF1//ITGB1//PPP3CA//PLK2//PAICS//POLA1//ANP32B//PLCB1//GADD45A//SPTBN1//ANK3//PPP2R5C//CALR//DBC1//SESN1//TCF7L2//SMC2//CLASP2//EIF4G3//ZFP828//CUL4A//HAUS7//PRUNE2 |
| GO:0009225 | nucleotide-sugar metabolic process | Biological process | 3 | 21 | 295 | 13692 | 6.63050847457627 | 0.00987736334338492 | 0.257752148198806 | 2.00535897021391 | UAP1//TSTA3//UXS1 |
| GO:0014823 | response to activity | Biological process | 5 | 61 | 295 | 13692 | 3.80439010836343 | 0.00998548232053119 | 0.258717263163048 | 2.00063095291784 | HIF1A//FNDC5//ITGB1//ZEB1//RTN4 |
| GO:0051651 | maintenance of location in cell | Biological process | 6 | 85 | 295 | 13692 | 3.27625124626122 | 0.0100323756244795 | 0.258717263163048 | 1.99859621568952 | KEAP1//KDELR3//ZFP828//CLASP2//ARL2BP//ANK3 |
| GO:0032787 | monocarboxylic acid metabolic process | Biological process | 15 | 356 | 295 | 13692 | 1.9556274995239 | 0.0104433827336846 | 0.267344825078113 | 1.98115880548917 | PHYH//HIF1A//ACADSB//FAAH//SC5DL//MGLL//HSD17B12//HADHA//FADS1//MGST2//ELOVL7//MAPK9//PLP1//INSIG1//INSIG2 |
| GO:0090002 | establishment of protein localization to plasma membrane | Biological process | 4 | 40 | 295 | 13692 | 4.64135593220339 | 0.010488893684634 | 0.267344825078113 | 1.97927031657254 | TMED2//SPTBN1//ANK3//PRKCI |
| GO:0045185 | maintenance of protein location | Biological process | 6 | 87 | 295 | 13692 | 3.20093512565751 | 0.0111806460249604 | 0.278389319375669 | 1.95153310190848 | KEAP1//KDELR3//ZFP828//CLASP2//ARL2BP//ANK3 |
| GO:0010559 | regulation of glycoprotein biosynthetic process | Biological process | 3 | 22 | 295 | 13692 | 6.32912172573189 | 0.0112574718825048 | 0.278389319375669 | 1.94855912908973 | TCF7L2//MGEA5//PLCB1 |
| GO:0010765 | positive regulation of sodium ion transport | Biological process | 3 | 22 | 295 | 13692 | 6.32912172573189 | 0.0112574718825048 | 0.278389319375669 | 1.94855912908973 | SCN4B//CNKSR3//AHCYL1 |
| GO:0040036 | regulation of fibroblast growth factor receptor signaling pathway | Biological process | 3 | 22 | 295 | 13692 | 6.32912172573189 | 0.0112574718825048 | 0.278389319375669 | 1.94855912908973 | SPRY2//TCF7L2//NPTN |
| GO:0042517 | positive regulation of tyrosine phosphorylation of Stat3 protein | Biological process | 3 | 22 | 295 | 13692 | 6.32912172573189 | 0.0112574718825048 | 0.278389319375669 | 1.94855912908973 | KIT//CLCF1//ARL2BP |
| GO:0007243 | intracellular protein kinase cascade | Biological process | 25 | 713 | 295 | 13692 | 1.62740390329712 | 0.0114196631372785 | 0.278389319375669 | 1.94234670692895 | RGD1306565//FAIM//MAPK10//MAPK9//CLCF1//PJA2//NBR1//KIT//ARL2BP//SQSTM1//PLK2//LPAR1//ZDHHC17//DNAJA3//PSAP//ITGB1//MAP3K4//PDCD4//PLCB1//SPRY2//TCF7L2//PLEKHA1//CNKSR3//SEMA7A//OXSR1 |
| GO:0014909 | smooth muscle cell migration | Biological process | 4 | 41 | 295 | 13692 | 4.52815212897892 | 0.0114302184050229 | 0.278389319375669 | 1.94194547116289 | RAPGEF4//RBPJ//LPAR1//DDR1 |
| GO:0090150 | establishment of protein localization to membrane | Biological process | 4 | 41 | 295 | 13692 | 4.52815212897892 | 0.0114302184050229 | 0.278389319375669 | 1.94194547116289 | TMED2//SPTBN1//ANK3//PRKCI |
| GO:0090276 | regulation of peptide hormone secretion | Biological process | 7 | 114 | 295 | 13692 | 2.84995539696699 | 0.0116491815250024 | 0.282154761356964 | 1.93370458722698 | PPP3CB//GLUL//SNAP25//MGEA5//TCF7L2//HIF1A//PPP3CA |
| GO:0042325 | regulation of phosphorylation | Biological process | 25 | 716 | 295 | 13692 | 1.62058517185873 | 0.0119892016979036 | 0.287936243268799 | 1.92120973347643 | RGD1306565//KIT//MADD//LPAR1//LRP4//MAPK9//NPTN//SENP2//SQSTM1//EIF4G3//GADD45A//DNAJA3//ZFP91//SPRY2//CAMKK1//CNKSR3//CLCF1//ARL2BP//MAP3K4//PDCD4//SDC4//ITGB1//PAK2//NRP1//HSP90AB1 |
| GO:0006839 | mitochondrial transport | Biological process | 5 | 64 | 295 | 13692 | 3.6260593220339 | 0.0121505942072828 | 0.287936243268799 | 1.91540248300115 | YWHAZ//MAPK9//SLC1A3//SLC25A13//CNP |
| GO:0043241 | protein complex disassembly | Biological process | 5 | 64 | 295 | 13692 | 3.6260593220339 | 0.0121505942072828 | 0.287936243268799 | 1.91540248300115 | CLASP2//DSTN//NAPA//MGEA5//GSN |
| GO:0071478 | cellular response to radiation | Biological process | 5 | 64 | 295 | 13692 | 3.6260593220339 | 0.0121505942072828 | 0.287936243268799 | 1.91540248300115 | MAPK9//PTPRK//ITGB1//GADD45A//EGR1 |
| GO:0006915 | apoptotic process | Biological process | 40 | 1295 | 295 | 13692 | 1.43362345396244 | 0.0126661720116977 | 0.298540312361735 | 1.89735461837534 | MAPK9//APAF1//TCF7L2//TRIAP1//MAL//PRUNE2//EI24//ROBO1//DNAJA3//RGD1306565//HIF1A//MADD//RGD1560691//PRKCI//RBPJ//PPP2R5C//ITGB1//PDIA3//LPAR1//CAT//PAK2//PLK2//FAIM//HIGD1A//KRT18//RRN3//KLHL20//SPRY2//HSP90AB1//CLCF1//EGR1//PDCD4//SQSTM1//CCL6//GHITM//GSN//PDCD6IP |
| GO:0042692 | muscle cell differentiation | Biological process | 12 | 267 | 295 | 13692 | 2.08600266615883 | 0.013172889529931 | 0.306958362414457 | 1.88031895022731 | DNAJA3//ANK3//ITGB1//ACTG1//MKX//PLCB1//TCF7L2//PPP3CA//LRP4//ZEB1//PIAS1//CALR |
| GO:0055001 | muscle cell development | Biological process | 8 | 145 | 295 | 13692 | 2.56074810052601 | 0.0132691214684768 | 0.306958362414457 | 1.87715783026466 | DNAJA3//ANK3//ITGB1//ACTG1//MKX//PLCB1//PPP3CA//LRP4 |
| GO:0002791 | regulation of peptide secretion | Biological process | 7 | 117 | 295 | 13692 | 2.77687961755758 | 0.0133033961812835 | 0.306958362414457 | 1.8760374752353 | PPP3CB//GLUL//SNAP25//MGEA5//TCF7L2//HIF1A//PPP3CA |
| GO:0090087 | regulation of peptide transport | Biological process | 7 | 117 | 295 | 13692 | 2.77687961755758 | 0.0133033961812835 | 0.306958362414457 | 1.8760374752353 | PPP3CB//GLUL//SNAP25//MGEA5//TCF7L2//HIF1A//PPP3CA |
| GO:0051970 | negative regulation of transmission of nerve impulse | Biological process | 4 | 43 | 295 | 13692 | 4.31754040204966 | 0.0134718371667124 | 0.309022523894101 | 1.87057317510405 | RAPGEF4//MGLL//PLK2//SLC24A2 |
| GO:0007568 | aging | Biological process | 12 | 268 | 295 | 13692 | 2.07821907412092 | 0.0135338331632453 | 0.309022523894101 | 1.86857918153428 | PDCD4//DNAJA3//CAT//CALR//GSTM2//CNP//CANX//APAF1//RTN4//FADS1//MGEA5//GSN |
| GO:0014013 | regulation of gliogenesis | Biological process | 5 | 66 | 295 | 13692 | 3.51617873651772 | 0.0137586864267671 | 0.312286335944367 | 1.86142302720793 | GFAP//RTN4//CLCF1//TCF7L2//PRKCI |
| GO:0000902 | cell morphogenesis | Biological process | 25 | 725 | 295 | 13692 | 1.60046756282876 | 0.0138369747857391 | 0.312286335944367 | 1.85895885044813 | HIF1A//RBPJ//SNAP25//CNP//ACTB//PICALM//ROBO1//NRP1//SEMA3C//ANK3//NRCAM//RTN4//CHN1//SPTA1//ERMN//CDC42SE2//MAPK9//GFAP//SEMA7A//SLC1A3//UGT8//GSN//NPTX1//CAP1 |
| GO:0048167 | regulation of synaptic plasticity | Biological process | 7 | 118 | 295 | 13692 | 2.75334673944269 | 0.0138904734281824 | 0.312286335944367 | 1.85728295197705 | EGR1//NPTN//KIT//GFAP//PLK2//SLC24A2//MGLL |
| GO:0006687 | glycosphingolipid metabolic process | Biological process | 3 | 24 | 295 | 13692 | 5.80169491525424 | 0.0143355887921371 | 0.317410208407722 | 1.84358446498294 | UGT8//ST6GALNAC3//KIT |
| GO:0008038 | neuron recognition | Biological process | 3 | 24 | 295 | 13692 | 5.80169491525424 | 0.0143355887921371 | 0.317410208407722 | 1.84358446498294 | RTN4//NRP1//ROBO1 |
| GO:0042304 | regulation of fatty acid biosynthetic process | Biological process | 3 | 24 | 295 | 13692 | 5.80169491525424 | 0.0143355887921371 | 0.317410208407722 | 1.84358446498294 | MAPK9//INSIG1//INSIG2 |
| GO:0036294 | cellular response to decreased oxygen levels | Biological process | 5 | 67 | 295 | 13692 | 3.4636984568682 | 0.0146139802700009 | 0.32033844751842 | 1.83523148332141 | EGR1//ITPR1//HIF1A//HIGD1A//NDRG1 |
| GO:0071456 | cellular response to hypoxia | Biological process | 5 | 67 | 295 | 13692 | 3.4636984568682 | 0.0146139802700009 | 0.32033844751842 | 1.83523148332141 | EGR1//ITPR1//HIF1A//HIGD1A//NDRG1 |
| GO:0006913 | nucleocytoplasmic transport | Biological process | 12 | 273 | 295 | 13692 | 2.04015645371578 | 0.0154531800373544 | 0.334830013371754 | 1.81098213562016 | KEAP1//PPP3CA//KPNB1//PTTG1IP//CALR//HSPA9//AHCYL1//SPTBN1//HSP90AB1//TCF7L2//HNRNPA1//ANP32A |
| GO:0006919 | activation of cysteine-type endopeptidase activity involved in apoptotic process | Biological process | 5 | 68 | 295 | 13692 | 3.41276171485543 | 0.0155042182286647 | 0.334830013371754 | 1.80955012736517 | APAF1//MAPK9//ROBO1//DNAJA3//RGD1306565 |
| GO:0097202 | activation of cysteine-type endopeptidase activity | Biological process | 5 | 68 | 295 | 13692 | 3.41276171485543 | 0.0155042182286647 | 0.334830013371754 | 1.80955012736517 | MAPK9//ROBO1//APAF1//DNAJA3//RGD1306565 |
| GO:0014812 | muscle cell migration | Biological process | 4 | 45 | 295 | 13692 | 4.12564971751412 | 0.015731646536899 | 0.338076168714535 | 1.8032258200044 | DDR1//RAPGEF4//RBPJ//LPAR1 |
| GO:0035773 | insulin secretion involved in cellular response to glucose stimulus | Biological process | 3 | 25 | 295 | 13692 | 5.56962711864407 | 0.0160356673148127 | 0.341263910233684 | 1.79491296245144 | PPP3CB//HIF1A//TCF7L2 |
| GO:0061178 | regulation of insulin secretion involved in cellular response to glucose stimulus | Biological process | 3 | 25 | 295 | 13692 | 5.56962711864407 | 0.0160356673148127 | 0.341263910233684 | 1.79491296245144 | PPP3CB//HIF1A//TCF7L2 |
| GO:0044093 | positive regulation of molecular function | Biological process | 28 | 848 | 295 | 13692 | 1.5325231851615 | 0.0161259835306333 | 0.341528076320272 | 1.79247378807869 | RGD1306565//KIT//MADD//LPAR1//MAPK9//ROBO1//APAF1//DNAJA3//ZFP91//RPL5//SPTA1//HSP90AB1//TCF7L2//CAMKK1//STIM2//RGN//RGD1560691//CHN2//MAP3K4//PLCB1//CHN1//SDC4//CAT//PRKCI//SERINC1//PAK2//SPRY2//CNOT6 |
| GO:0043067 | regulation of programmed cell death | Biological process | 34 | 1080 | 295 | 13692 | 1.46116760828625 | 0.0162902912012716 | 0.343349214549878 | 1.78807115227989 | PPP3CB//TCF7L2//TRIAP1//MAL//APAF1//PRUNE2//EI24//RGD1306565//HIF1A//MADD//RGD1560691//RTN4//MAPK9//PRKCI//RBPJ//PPP2R5C//ITGB1//PDIA3//LPAR1//DNAJA3//CAT//PAK2//PLK2//FAIM//HIGD1A//KRT18//RRN3//KLHL20//SPRY2//KIT//HSP90AB1//CLCF1//EGR1//SERBP1 |
| GO:0048731 | system development | Biological process | 82 | 3069 | 295 | 13692 | 1.24011465115894 | 0.0166051808108049 | 0.343861359216937 | 1.77975639108593 | NCL//HIF1A//RTN4//ATP5B//NRP1//NRCAM//RBPJ//KIT//PLEKHA1//TCF7L2//SEMA3C//LRP4//TET2//TMED2//APAF1//UPF2//HSP90AB1//ITGB1//ROBO1//LUZP1//MOBP//SNAP25//CNP//ACTB//PICALM//ANK3//FMR1//ZEB1//MAPK9//CD9//NT5E//ARF4//NAPA//PPP3CB//RAPGEF4//TNXA-PS1//MKX//DNAJA3//DDR1//CHN1//PLP1//CLDN11//GFAP//NPTN//PRKCI//RGD1560691//LPAR1//MRF//GSN//SLC1A3//PLCB1//SPTA1//CLCF1//EGR1//ASGR2//CLCN2//SPRY2//NBR1//UGT8//PRICKLE2//PTPRK//NDRG1//MGST1//INSIG1//INSIG2//PLS3//MAL//MPDZ//CAT//KEAP1//S1PR5//CALR//ADAM10//RGD1309676//SEMA7A//TAPT1//EXOC4//PPP3CA//FAIM//NPTX1//PSAP//ARIH2 |
| GO:0006464 | cellular protein modification process | Biological process | 55 | 1928 | 295 | 13692 | 1.32403825866798 | 0.0166188827253956 | 0.343861359216937 | 1.77939817685189 | RGD1306565//KIT//MADD//LPAR1//TNKS2//ARIH2//FKBP9//LRP4//MAPK9//NPTN//SENP2//SQSTM1//EIF4G3//PJA2//KLHL20//PPP3CB//MAPK10//PAK2//ADAM10//CAMKK1//PLK2//PRKCI//TLK2//OXSR1//NEK7//GADD45A//DNAJA3//PPM1B//PPP3CA//PTPRK//PTPN3//ST6GALNAC3//GALNT1//KLHL12//SPTBN1//ZFP91//SPRY2//MGEA5//TAF1//DDR1//ZDHHC17//DNAJB2//CNKSR3//EGR1//PIAS1//PTP4A1//CLCF1//ARL2BP//MAP3K4//PDCD4//SDC4//ITGB1//NRP1//TRIM37//HSP90AB1 |
| GO:0036211 | protein modification process | Biological process | 55 | 1928 | 295 | 13692 | 1.32403825866798 | 0.0166188827253956 | 0.343861359216937 | 1.77939817685189 | RGD1306565//KIT//MADD//LPAR1//TNKS2//ARIH2//FKBP9//LRP4//MAPK9//NPTN//SENP2//SQSTM1//EIF4G3//PJA2//KLHL20//PPP3CB//MAPK10//PAK2//ADAM10//CAMKK1//PLK2//PRKCI//TLK2//OXSR1//NEK7//GADD45A//DNAJA3//PPM1B//PPP3CA//PTPRK//PTPN3//ST6GALNAC3//GALNT1//KLHL12//SPTBN1//ZFP91//SPRY2//MGEA5//TAF1//DDR1//ZDHHC17//DNAJB2//CNKSR3//EGR1//PIAS1//PTP4A1//CLCF1//ARL2BP//MAP3K4//PDCD4//SDC4//ITGB1//NRP1//TRIM37//HSP90AB1 |
| GO:0007268 | synaptic transmission | Biological process | 17 | 446 | 295 | 13692 | 1.76912670061564 | 0.016671676615095 | 0.343861359216937 | 1.77802072242339 | SNAP25//NSF//NAPA//GLUL//PRIMA1//PLK2//EGR1//NPTN//KIT//CANX//PPP3CA//RAPGEF4//SLC1A3//GFAP//SLC24A2//MGLL//EXOC4 |
| GO:0048747 | muscle fiber development | Biological process | 6 | 95 | 295 | 13692 | 2.93138269402319 | 0.0167067676809324 | 0.343861359216937 | 1.77710756651134 | DNAJA3//ANK3//MKX//PLCB1//PPP3CA//LRP4 |
| GO:0051169 | nuclear transport | Biological process | 12 | 277 | 295 | 13692 | 2.01069571070183 | 0.0171319607564277 | 0.350965027832612 | 1.76619292909723 | KEAP1//PPP3CA//KPNB1//PTTG1IP//CALR//HSPA9//AHCYL1//ANP32A//SPTBN1//HSP90AB1//TCF7L2//HNRNPA1 |
| GO:0006892 | post-Golgi vesicle-mediated transport | Biological process | 3 | 26 | 295 | 13692 | 5.35541069100391 | 0.0178440975101091 | 0.358678251078214 | 1.74850541219647 | KRT18//SPTBN1//ANK3 |
| GO:0031124 | mRNA 3'-end processing | Biological process | 3 | 26 | 295 | 13692 | 5.35541069100391 | 0.0178440975101091 | 0.358678251078214 | 1.74850541219647 | PARN//AHCYL1//CPSF3 |
| GO:0042516 | regulation of tyrosine phosphorylation of Stat3 protein | Biological process | 3 | 26 | 295 | 13692 | 5.35541069100391 | 0.0178440975101091 | 0.358678251078214 | 1.74850541219647 | KIT//CLCF1//ARL2BP |
| GO:0031645 | negative regulation of neurological system process | Biological process | 4 | 47 | 295 | 13692 | 3.95009015506671 | 0.0182163592279526 | 0.358678251078214 | 1.73953841798913 | RAPGEF4//MGLL//PLK2//SLC24A2 |
| GO:0006639 | acylglycerol metabolic process | Biological process | 5 | 71 | 295 | 13692 | 3.26856051563619 | 0.0183901224554932 | 0.358678251078214 | 1.73541537888765 | CAT//INSIG1//INSIG2//MGLL//CES1D |
| GO:0050657 | nucleic acid transport | Biological process | 5 | 71 | 295 | 13692 | 3.26856051563619 | 0.0183901224554932 | 0.358678251078214 | 1.73541537888765 | SIDT1//FMR1//HNRNPA1//SENP2//HNRNPA3 |
| GO:0050658 | RNA transport | Biological process | 5 | 71 | 295 | 13692 | 3.26856051563619 | 0.0183901224554932 | 0.358678251078214 | 1.73541537888765 | SIDT1//FMR1//HNRNPA1//SENP2//HNRNPA3 |
| GO:0051236 | establishment of RNA localization | Biological process | 5 | 71 | 295 | 13692 | 3.26856051563619 | 0.0183901224554932 | 0.358678251078214 | 1.73541537888765 | SIDT1//FMR1//HNRNPA1//SENP2//HNRNPA3 |
| GO:0030072 | peptide hormone secretion | Biological process | 8 | 154 | 295 | 13692 | 2.41109399075501 | 0.0184530042141222 | 0.358678251078214 | 1.73393291907652 | PPP3CB//RAPGEF4//SNAP25//GLUL//MGEA5//TCF7L2//HIF1A//PPP3CA |
| GO:0022411 | cellular component disassembly | Biological process | 7 | 125 | 295 | 13692 | 2.5991593220339 | 0.01852919540614 | 0.358678251078214 | 1.73214343865521 | APAF1//CLASP2//DDR1//DSTN//NAPA//MGEA5//GSN |
| GO:0044242 | cellular lipid catabolic process | Biological process | 7 | 125 | 295 | 13692 | 2.5991593220339 | 0.01852919540614 | 0.358678251078214 | 1.73214343865521 | PHYH//ACADSB//HADHA//FAAH//MGLL//CES1D//GLA |
| GO:0015693 | magnesium ion transport | Biological process | 2 | 10 | 295 | 13692 | 9.28271186440678 | 0.0185720718509933 | 0.358678251078214 | 1.73113964481905 | MAGT1//ZDHHC17 |
| GO:0040037 | negative regulation of fibroblast growth factor receptor signaling pathway | Biological process | 2 | 10 | 295 | 13692 | 9.28271186440678 | 0.0185720718509933 | 0.358678251078214 | 1.73113964481905 | SPRY2//TCF7L2 |
| GO:0070848 | response to growth factor stimulus | Biological process | 11 | 249 | 295 | 13692 | 2.05039820298142 | 0.0193065038546223 | 0.371226810959053 | 1.71429636392718 | HIF1A//NRP1//SPRY2//TCF7L2//NPTN//CAT//EGR1//MAPK9//ITGB1//ZEB1//APAF1 |
| GO:0060326 | cell chemotaxis | Biological process | 7 | 127 | 295 | 13692 | 2.55822767916722 | 0.0200330052397917 | 0.383513951839506 | 1.69825389538156 | KIT//ADAM10//NRP1//RGD1560691//LPAR1//CCL6//ELMO2 |
| GO:0071363 | cellular response to growth factor stimulus | Biological process | 10 | 219 | 295 | 13692 | 2.11934060831205 | 0.020475120181366 | 0.38607765555404 | 1.68877354041243 | HIF1A//NRP1//SPRY2//TCF7L2//NPTN//ZEB1//APAF1//CAT//EGR1//MAPK9 |
| GO:0006638 | neutral lipid metabolic process | Biological process | 5 | 73 | 295 | 13692 | 3.17901091246808 | 0.020498579609812 | 0.38607765555404 | 1.68827623109021 | CAT//INSIG1//INSIG2//MGLL//CES1D |
| GO:0051656 | establishment of organelle localization | Biological process | 5 | 73 | 295 | 13692 | 3.17901091246808 | 0.020498579609812 | 0.38607765555404 | 1.68827623109021 | TMED2//EXOC4//ZFP828//TMED9//KLHL12 |
| GO:0031325 | positive regulation of cellular metabolic process | Biological process | 47 | 1621 | 295 | 13692 | 1.34573552630203 | 0.0205191819671741 | 0.38607765555404 | 1.68783995711889 | RGD1306565//KIT//MADD//LPAR1//NSF//MAPK9//NPTN//SENP2//SQSTM1//EIF4G3//RBPJ//ZFP91//GADD45A//HIF1A//TCF7L2//RPS4X//EGR1//PLCB1//NFIA//CNBP//ELF1//PICALM//MRF//PIAS1//RRN3//ILF2//ATMIN//INSIG1//INSIG2//DNAJA3//DNAJB2//CAMKK1//TAF1//SPRY2//CLCF1//ARL2BP//MAP3K4//HNRNPK//CALR//SDC4//MGEA5//PPP3CA//ZEB1//ITGB1//PAK2//NRP1//HSP90AB1 |
| GO:0043244 | regulation of protein complex disassembly | Biological process | 4 | 49 | 295 | 13692 | 3.78886198547215 | 0.0209318107668789 | 0.391337607306918 | 1.67919320013492 | CLASP2//DSTN//MGEA5//GSN |
| GO:0007267 | cell-cell signaling | Biological process | 24 | 715 | 295 | 13692 | 1.55793765556477 | 0.0210074135702494 | 0.391337607306918 | 1.67762741457711 | YWHAZ//EXOC4//SNAP25//NSF//PPP3CB//NAPA//RAPGEF4//GLUL//MGEA5//TCF7L2//RTN4//HIF1A//PRIMA1//PPP3CA//PLK2//EGR1//NPTN//KIT//CANX//SLC1A3//GFAP//SLC24A2//MGLL//SDC4 |
| GO:0022403 | cell cycle phase | Biological process | 18 | 494 | 295 | 13692 | 1.69118232347492 | 0.0210665317802082 | 0.391337607306918 | 1.67640695716349 | TAF1//ITGB1//PPP3CA//PLK2//PAICS//POLA1//ANP32B//PLCB1//GADD45A//CLASP2//SMC2//EIF4G3//ZFP828//CALR//CUL4A//HAUS7//PRUNE2 |
| GO:0043412 | macromolecule modification | Biological process | 56 | 1997 | 295 | 13692 | 1.30153195895538 | 0.0214078538510774 | 0.392819149215925 | 1.66942686877995 | RGD1306565//KIT//MADD//LPAR1//TNKS2//ARIH2//FKBP9//LRP4//MAPK9//NPTN//SENP2//SQSTM1//EIF4G3//PJA2//KLHL20//PPP3CB//MAPK10//PAK2//ADAM10//CAMKK1//PLK2//PRKCI//TLK2//OXSR1//NEK7//GADD45A//DNAJA3//PPM1B//PPP3CA//PTPRK//PTPN3//ST6GALNAC3//GALNT1//KLHL12//SPTBN1//ZFP91//SPRY2//MGEA5//TAF1//DDR1//ZDHHC17//GAR1//DNAJB2//CNKSR3//EGR1//PIAS1//PTP4A1//CLCF1//ARL2BP//MAP3K4//PDCD4//SDC4//ITGB1//NRP1//TRIM37//HSP90AB1 |
| GO:0000904 | cell morphogenesis involved in differentiation | Biological process | 18 | 495 | 295 | 13692 | 1.68776579352851 | 0.0214474673838568 | 0.392819149215925 | 1.66862398395263 | HIF1A//RBPJ//SNAP25//CNP//ACTB//PICALM//ROBO1//NRP1//SEMA3C//ANK3//NRCAM//RTN4//CHN1//MAPK9//SEMA7A//SLC1A3//NPTX1 |
| GO:0044057 | regulation of system process | Biological process | 18 | 495 | 295 | 13692 | 1.68776579352851 | 0.0214474673838568 | 0.392819149215925 | 1.66862398395263 | MGEA5//NAPA//PPP3CA//TCF7L2//MRF//HIF1A//PLK2//EGR1//NPTN//KIT//RAPGEF4//SLC1A3//GFAP//MGLL//RTN4//GLUL//SLC24A2//SCN4B |
| GO:0042503 | tyrosine phosphorylation of Stat3 protein | Biological process | 3 | 28 | 295 | 13692 | 4.9728813559322 | 0.0217877768497317 | 0.392819149215925 | 1.66178708141738 | KIT//CLCF1//ARL2BP |
| GO:0007409 | axonogenesis | Biological process | 13 | 321 | 295 | 13692 | 1.87967685727863 | 0.0220744541141461 | 0.392819149215925 | 1.65611002741723 | ROBO1//NRP1//SEMA3C//ANK3//NRCAM//RTN4//CHN1//SEMA7A//NPTX1//SNAP25//CNP//ACTB//PICALM |
| GO:0048812 | neuron projection morphogenesis | Biological process | 15 | 390 | 295 | 13692 | 1.7851368970013 | 0.0220824468843535 | 0.392819149215925 | 1.65595280551281 | SNAP25//CNP//ACTB//PICALM//ROBO1//NRP1//SEMA3C//ANK3//NRCAM//RTN4//CHN1//GFAP//SEMA7A//NPTX1//UGT8 |
| GO:0019220 | regulation of phosphate metabolic process | Biological process | 26 | 795 | 295 | 13692 | 1.5179277262552 | 0.0223106326399881 | 0.392819149215925 | 1.65148811469328 | RGD1306565//KIT//MADD//LPAR1//LRP4//MAPK9//NPTN//SENP2//SQSTM1//EIF4G3//GADD45A//DNAJA3//ZFP91//SPRY2//RPL5//CAMKK1//CNKSR3//CLCF1//ARL2BP//MAP3K4//PDCD4//SDC4//ITGB1//PAK2//NRP1//HSP90AB1 |
| GO:0051174 | regulation of phosphorus metabolic process | Biological process | 26 | 795 | 295 | 13692 | 1.5179277262552 | 0.0223106326399881 | 0.392819149215925 | 1.65148811469328 | RGD1306565//KIT//MADD//LPAR1//LRP4//MAPK9//NPTN//SENP2//SQSTM1//EIF4G3//GADD45A//DNAJA3//ZFP91//SPRY2//RPL5//CAMKK1//CNKSR3//CLCF1//ARL2BP//MAP3K4//PDCD4//SDC4//ITGB1//PAK2//NRP1//HSP90AB1 |
| GO:0002260 | lymphocyte homeostasis | Biological process | 4 | 50 | 295 | 13692 | 3.71308474576271 | 0.022377648020854 | 0.392819149215925 | 1.65018556147353 | HIF1A//DNAJA3//PPP3CB//SPTA1 |
| GO:0010560 | positive regulation of glycoprotein biosynthetic process | Biological process | 2 | 11 | 295 | 13692 | 8.43882896764253 | 0.0223799170179262 | 0.392819149215925 | 1.65014152811703 | TCF7L2//PLCB1 |
| GO:0035774 | positive regulation of insulin secretion involved in cellular response to glucose stimulus | Biological process | 2 | 11 | 295 | 13692 | 8.43882896764253 | 0.0223799170179262 | 0.392819149215925 | 1.65014152811703 | PPP3CB//HIF1A |
| GO:0060333 | interferon-gamma-mediated signaling pathway | Biological process | 2 | 11 | 295 | 13692 | 8.43882896764253 | 0.0223799170179262 | 0.392819149215925 | 1.65014152811703 | HSP90AB1//DNAJA3 |
| GO:0060363 | cranial suture morphogenesis | Biological process | 2 | 11 | 295 | 13692 | 8.43882896764253 | 0.0223799170179262 | 0.392819149215925 | 1.65014152811703 | INSIG1//INSIG2 |
| GO:0002790 | peptide secretion | Biological process | 8 | 160 | 295 | 13692 | 2.32067796610169 | 0.0226287113644077 | 0.392819149215925 | 1.64534017707761 | PPP3CB//RAPGEF4//SNAP25//GLUL//MGEA5//TCF7L2//HIF1A//PPP3CA |
| GO:0050890 | cognition | Biological process | 9 | 191 | 295 | 13692 | 2.18702635548851 | 0.0227297257175542 | 0.392819149215925 | 1.64340580492929 | EGR1//PLCB1//SLC24A2//PLK2//SNAP25//PJA2//HIF1A//KIT//MAGT1 |
| GO:0071843 | cellular component biogenesis at cellular level | Biological process | 9 | 191 | 295 | 13692 | 2.18702635548851 | 0.0227297257175542 | 0.392819149215925 | 1.64340580492929 | RRN3//GAR1//SRSF1//EIF3D//RGD1564051//LSM14A//EXOC4//SPTBN1//ANK3 |
| GO:0006403 | RNA localization | Biological process | 5 | 75 | 295 | 13692 | 3.09423728813559 | 0.0227591386635139 | 0.392819149215925 | 1.64284417816279 | SIDT1//FMR1//HNRNPA1//SENP2//HNRNPA3 |
| GO:0045807 | positive regulation of endocytosis | Biological process | 5 | 75 | 295 | 13692 | 3.09423728813559 | 0.0227591386635139 | 0.392819149215925 | 1.64284417816279 | HNRNPK//CALR//RGD1560691//ITGB1//CD14 |
| GO:0042981 | regulation of apoptotic process | Biological process | 33 | 1070 | 295 | 13692 | 1.43144622208142 | 0.0232594582889228 | 0.399880255445637 | 1.63340040417641 | PPP3CB//TCF7L2//TRIAP1//MAL//APAF1//PRUNE2//EI24//RGD1306565//RTN4//MAPK9//PRKCI//RBPJ//PPP2R5C//ITGB1//PDIA3//HIF1A//LPAR1//DNAJA3//CAT//PAK2//PLK2//FAIM//HIGD1A//KRT18//RRN3//KLHL20//SPRY2//HSP90AB1//CLCF1//EGR1//SERBP1//MADD//RGD1560691 |
| GO:0043270 | positive regulation of ion transport | Biological process | 6 | 103 | 295 | 13692 | 2.70370248477867 | 0.0238765892594386 | 0.405825227311622 | 1.6220277115292 | MGEA5//CNKSR3//AHCYL1//STIM2//ITPR1//SCN4B |
| GO:0006413 | translational initiation | Biological process | 4 | 51 | 295 | 13692 | 3.64027916251246 | 0.0238829627386858 | 0.405825227311622 | 1.62191179893086 | EIF3D//EIF5B//FMR1//EIF5 |
| GO:0006672 | ceramide metabolic process | Biological process | 4 | 51 | 295 | 13692 | 3.64027916251246 | 0.0238829627386858 | 0.405825227311622 | 1.62191179893086 | ST6GALNAC3//UGT8//GLA//ORMDL3 |
| GO:0033554 | cellular response to stress | Biological process | 29 | 919 | 295 | 13692 | 1.46462809612512 | 0.0249272483629049 | 0.421934582328089 | 1.60332565919223 | POLA1//SMC2//DDX1//GADD45A//PPP2R5C//MAPK10//MAPK9//RGD1306565//FADS1//SQSTM1//NBR1//NDRG1//TRIAP1//INSIG1//INSIG2//GFAP//PTPRK//HIGD1A//CAT//MAP3K4//PDCD4//HIF1A//PLCB1//RTN4//PLEKHA1//MGST1//EGR1//ITPR1//CALR |
| GO:0051168 | nuclear export | Biological process | 5 | 77 | 295 | 13692 | 3.01386748844376 | 0.0251752816788634 | 0.422972200645932 | 1.59902566155323 | CALR//HSPA9//AHCYL1//TCF7L2//HNRNPA1 |
| GO:0044281 | small molecule metabolic process | Biological process | 46 | 1601 | 295 | 13692 | 1.33355635778486 | 0.0251815110329809 | 0.422972200645932 | 1.59891821332282 | PHYH//UAP1//GPCPD1//HIF1A//GLYR1//GSTM2//MGST1//NT5E//ATP5B//PAICS//NSF//CTPS//GLUL//SLC1A3//MAT2B//GATM//ACADSB//FAAH//SC5DL//MGLL//HSD17B12//HADHA//FADS1//ETNK1//PDIA3//PDIA4//MGST2//INSIG1//INSIG2//AHCYL1//SLC25A13//CAT//CNP//TMED2//ELOVL7//TSTA3//RGN//MAPK9//ACAP2//CHN2//UXS1//SPRY2//PLP1//PLCB1//CHN1//MGEA5 |
| GO:0046326 | positive regulation of glucose import | Biological process | 3 | 30 | 295 | 13692 | 4.64135593220339 | 0.026168248400723 | 0.433146408237587 | 1.58222534634596 | RGD1304592//PRKCI//MGEA5 |
| GO:0008354 | germ cell migration | Biological process | 2 | 12 | 295 | 13692 | 7.73559322033898 | 0.0264788406495605 | 0.433146408237587 | 1.57710103398011 | KIT//ITGB1 |
| GO:0032288 | myelin assembly | Biological process | 2 | 12 | 295 | 13692 | 7.73559322033898 | 0.0264788406495605 | 0.433146408237587 | 1.57710103398011 | CD9//UGT8 |
| GO:0033233 | regulation of protein sumoylation | Biological process | 2 | 12 | 295 | 13692 | 7.73559322033898 | 0.0264788406495605 | 0.433146408237587 | 1.57710103398011 | PIAS1//EGR1 |
| GO:0034260 | negative regulation of GTPase activity | Biological process | 2 | 12 | 295 | 13692 | 7.73559322033898 | 0.0264788406495605 | 0.433146408237587 | 1.57710103398011 | SPRY2//TMED2 |
| GO:0070841 | inclusion body assembly | Biological process | 2 | 12 | 295 | 13692 | 7.73559322033898 | 0.0264788406495605 | 0.433146408237587 | 1.57710103398011 | TRIM37//DNAJB2 |
| GO:0097094 | craniofacial suture morphogenesis | Biological process | 2 | 12 | 295 | 13692 | 7.73559322033898 | 0.0264788406495605 | 0.433146408237587 | 1.57710103398011 | INSIG1//INSIG2 |
| GO:0010941 | regulation of cell death | Biological process | 34 | 1124 | 295 | 13692 | 1.40396887628928 | 0.0274661002593189 | 0.447625961103547 | 1.56120299897359 | PPP3CB//TCF7L2//TRIAP1//MAL//APAF1//PRUNE2//EI24//RGD1306565//EGR1//LPAR1//HIF1A//MADD//RGD1560691//RTN4//MAPK9//PRKCI//RBPJ//PPP2R5C//ITGB1//PDIA3//DNAJA3//CAT//PAK2//PLK2//FAIM//HIGD1A//KRT18//RRN3//KLHL20//SPRY2//KIT//HSP90AB1//CLCF1//SERBP1 |
| GO:0032984 | macromolecular complex disassembly | Biological process | 5 | 79 | 295 | 13692 | 2.93756704569835 | 0.0277501599741064 | 0.45036220060518 | 1.55673450891351 | CLASP2//DSTN//NAPA//MGEA5//GSN |
| GO:0046879 | hormone secretion | Biological process | 9 | 198 | 295 | 13692 | 2.10970724191063 | 0.0278394517253658 | 0.45036220060518 | 1.55533932204254 | YWHAZ//PPP3CB//SNAP25//RAPGEF4//GLUL//MGEA5//TCF7L2//HIF1A//PPP3CA |
| GO:0045597 | positive regulation of cell differentiation | Biological process | 17 | 474 | 295 | 13692 | 1.66462132589573 | 0.0280311643178464 | 0.451788043889436 | 1.55235886277078 | HIF1A//GFAP//MAPK9//TCF7L2//PLCB1//ITGB1//ZEB1//RTN4//SEMA7A//NPTN//KIT//CLCF1//FAIM//ROBO1//PIAS1//PRKCI//FNDC5 |
| GO:0034599 | cellular response to oxidative stress | Biological process | 6 | 107 | 295 | 13692 | 2.60262949469349 | 0.0281336989009617 | 0.451788043889436 | 1.5507731648604 | PTPRK//CAT//HIF1A//PLEKHA1//RGD1306565//MGST1 |
| GO:0071704 | organic substance metabolic process | Biological process | 26 | 813 | 295 | 13692 | 1.48432047032335 | 0.028451239668113 | 0.455219834689808 | 1.54589880591947 | MGEA5//UAP1//NT5E//NSF//ATP5B//TNKS2//ST6GALNAC3//GALNT1//ADAMTS4//MAT2B//ETNK1//UGT8//KIT//MGST2//ASGR2//TCF7L2//GLA//TMED2//TSTA3//ACAP2//CHN2//UXS1//SPRY2//PLCB1//CHN1//HIF1A |
| GO:0065009 | regulation of molecular function | Biological process | 43 | 1493 | 295 | 13692 | 1.3367602483908 | 0.029085148446476 | 0.463670148324912 | 1.53632871528247 | RGD1306565//KIT//MADD//LPAR1//GADD45A//DNAJA3//MAPK9//ROBO1//APAF1//ZFP91//RPL5//TMED2//CAT//TRIM37//SENP2//SPTA1//HSP90AB1//TCF7L2//CAMKK1//STIM2//ACAP2//AHCYL1//RGN//RGD1560691//CHN2//SPRY2//OAZ2//TRIAP1//HABP4//MAP3K4//PDCD4//PLCB1//CHN1//SDC4//HIF1A//GLA//FOXN3//PRKCI//SERINC1//CACNA2D3//PAK2//CNOT6//SCN4B |
| GO:0051047 | positive regulation of secretion | Biological process | 10 | 233 | 295 | 13692 | 1.99199825416455 | 0.0297637651448699 | 0.467015082415319 | 1.526312130982 | SNAP25//GLUL//MGEA5//TCF7L2//MAPK9//RTN4//PPP3CB//HIF1A//CD14//PLCB1 |
| GO:0008610 | lipid biosynthetic process | Biological process | 15 | 406 | 295 | 13692 | 1.71478667445938 | 0.0301396179279243 | 0.467015082415319 | 1.52086225742437 | MGLL//HSD17B12//SC5DL//FADS1//ETNK1//UGT8//INSIG1//INSIG2//SERINC1//NT5E//ELOVL7//MGST2//MAPK9//PIK3CB//PLP1 |
| GO:0006898 | receptor-mediated endocytosis | Biological process | 6 | 109 | 295 | 13692 | 2.55487482506609 | 0.0304386621565631 | 0.467015082415319 | 1.51657443964097 | PICALM//HNRNPK//CANX//MLC1//CAP1//ATP5B |
| GO:0019222 | regulation of metabolic process | Biological process | 93 | 3634 | 295 | 13692 | 1.18779884891281 | 0.0307250974555545 | 0.467015082415319 | 1.51250673092019 | EGR1//ZEB1//CALR//MKX//PIAS1//DNAJA3//TCF7L2//RGD1306565//KIT//MADD//LPAR1//NSF//LRP4//MAPK9//NPTN//SENP2//SQSTM1//EIF4G3//HIF1A//ANP32A//DDX1//ELF1//DMTF1//HDGF//KEAP1//CNOT6//HNRPDL//RNF141//TAF1//HDAC8//HMGN5//RBPJ//FMR1//EIF5B//GADD45A//ROBO1//APAF1//ZFP91//PPP3CB//PLP1//TMED2//SPRY2//INSIG1//INSIG2//RPL5//RPS4X//NT5E//SIDT1//PLCB1//NFIA//CNBP//PICALM//MRF//RRN3//ILF2//ATMIN//HSPA8//PDCD4//FOXN3//PTPRK//PSAP//CR1L//DNAJB2//AHCYL1//ASGR2//CAT//TRIM37//CAMKK1//ACAP2//HSP90AB1//RGN//RGD1560691//CHN2//CNKSR3//CLCF1//ARL2BP//OAZ2//TRIAP1//HNRPH1//MAP3K4//CHN1//GLA//HNRNPK//SDC4//MGEA5//PPP3CA//ITGB1//PAK2//NRP1//PTPN3//PRKCI//CUL4A//SERINC1 |
| GO:0014850 | response to muscle activity | Biological process | 2 | 13 | 295 | 13692 | 7.14054758800521 | 0.0308546214036824 | 0.467015082415319 | 1.51067977815254 | HIF1A//FNDC5 |
| GO:0014888 | striated muscle adaptation | Biological process | 2 | 13 | 295 | 13692 | 7.14054758800521 | 0.0308546214036824 | 0.467015082415319 | 1.51067977815254 | MGEA5//PPP3CA |
| GO:0022010 | central nervous system myelination | Biological process | 2 | 13 | 295 | 13692 | 7.14054758800521 | 0.0308546214036824 | 0.467015082415319 | 1.51067977815254 | PLP1//MRF |
| GO:0032291 | axon ensheathment in central nervous system | Biological process | 2 | 13 | 295 | 13692 | 7.14054758800521 | 0.0308546214036824 | 0.467015082415319 | 1.51067977815254 | PLP1//MRF |
| GO:0034349 | glial cell apoptotic process | Biological process | 2 | 13 | 295 | 13692 | 7.14054758800521 | 0.0308546214036824 | 0.467015082415319 | 1.51067977815254 | PRKCI//APAF1 |
| GO:0043243 | positive regulation of protein complex disassembly | Biological process | 2 | 13 | 295 | 13692 | 7.14054758800521 | 0.0308546214036824 | 0.467015082415319 | 1.51067977815254 | DSTN//MGEA5 |
| GO:0060600 | dichotomous subdivision of an epithelial terminal unit | Biological process | 2 | 13 | 295 | 13692 | 7.14054758800521 | 0.0308546214036824 | 0.467015082415319 | 1.51067977815254 | NRP1//SEMA3C |
| GO:0070498 | interleukin-1-mediated signaling pathway | Biological process | 2 | 13 | 295 | 13692 | 7.14054758800521 | 0.0308546214036824 | 0.467015082415319 | 1.51067977815254 | EGR1//PLCB1 |
| GO:0000084 | S phase of mitotic cell cycle | Biological process | 3 | 32 | 295 | 13692 | 4.35127118644068 | 0.0309843176255918 | 0.467015082415319 | 1.50885806397141 | CUL4A//POLA1//ANP32B |
| GO:0001975 | response to amphetamine | Biological process | 3 | 32 | 295 | 13692 | 4.35127118644068 | 0.0309843176255918 | 0.467015082415319 | 1.50885806397141 | EGR1//PPP3CA//PPP3CB |
| GO:0071479 | cellular response to ionizing radiation | Biological process | 3 | 32 | 295 | 13692 | 4.35127118644068 | 0.0309843176255918 | 0.467015082415319 | 1.50885806397141 | EGR1//ITGB1//GADD45A |
| GO:0009612 | response to mechanical stimulus | Biological process | 8 | 170 | 295 | 13692 | 2.18416749750748 | 0.0309994044212723 | 0.467015082415319 | 1.50864665000572 | EGR1//ITGB1//GADD45A//HABP4//SLC1A3//KIT//HIF1A//MAPK9 |
| GO:0006914 | autophagy | Biological process | 5 | 82 | 295 | 13692 | 2.83009508061182 | 0.0319161700963411 | 0.477026895516148 | 1.49598922903242 | SQSTM1//NBR1//LAMP1//PIK3CB//EI24 |
| GO:0051146 | striated muscle cell differentiation | Biological process | 9 | 203 | 295 | 13692 | 2.05774400935126 | 0.0319519091580696 | 0.477026895516148 | 1.49550318720688 | DNAJA3//ANK3//ITGB1//ACTG1//MKX//PLCB1//PPP3CA//LRP4//CALR |
| GO:1901137 | carbohydrate derivative biosynthetic process | Biological process | 10 | 236 | 295 | 13692 | 1.96667624245906 | 0.0320893241012624 | 0.477026895516148 | 1.49363943037048 | TNKS2//ST6GALNAC3//GALNT1//MAT2B//UGT8//TCF7L2//PLCB1//TSTA3//NT5E//MGEA5 |
| GO:0060255 | regulation of macromolecule metabolic process | Biological process | 80 | 3072 | 295 | 13692 | 1.20868644067797 | 0.0321414654363133 | 0.477026895516148 | 1.49293432619135 | EGR1//ZEB1//CALR//MKX//PIAS1//DNAJA3//TCF7L2//RGD1306565//KIT//MADD//LPAR1//NSF//LRP4//MAPK9//NPTN//SENP2//SQSTM1//EIF4G3//HIF1A//ANP32A//DDX1//ELF1//DMTF1//HDGF//KEAP1//CNOT6//HNRPDL//RNF141//TAF1//HDAC8//HMGN5//RBPJ//FMR1//EIF5B//GADD45A//ZFP91//PPP3CB//PLP1//TMED2//SPRY2//RPS4X//SIDT1//PLCB1//NFIA//CNBP//PICALM//MRF//RRN3//ILF2//ATMIN//HSPA8//PDCD4//FOXN3//PTPRK//CR1L//INSIG1//INSIG2//DNAJB2//AHCYL1//ASGR2//CAT//TRIM37//CAMKK1//HSP90AB1//RGD1560691//CNKSR3//CLCF1//ARL2BP//HNRPH1//MAP3K4//HNRNPK//SDC4//MGEA5//PPP3CA//ITGB1//PAK2//NRP1//PTPN3//PRKCI//CUL4A |
| GO:0031023 | microtubule organizing center organization | Biological process | 4 | 56 | 295 | 13692 | 3.31525423728814 | 0.0323168311971478 | 0.477026895516148 | 1.49057123023228 | GADD45A//PLK2//HAUS7//CLASP2 |
| GO:0051028 | mRNA transport | Biological process | 4 | 56 | 295 | 13692 | 3.31525423728814 | 0.0323168311971478 | 0.477026895516148 | 1.49057123023228 | FMR1//HNRNPA1//SENP2//HNRNPA3 |
| GO:0007613 | memory | Biological process | 5 | 83 | 295 | 13692 | 2.79599754952011 | 0.033387040394705 | 0.491170419766398 | 1.47642207741811 | SNAP25//PJA2//PLCB1//PLK2//SLC24A2 |
| GO:0007528 | neuromuscular junction development | Biological process | 3 | 33 | 295 | 13692 | 4.21941448382126 | 0.0335546359560645 | 0.491985030205307 | 1.47424746860838 | LRP4//DNAJA3//ANK3 |
| GO:0017156 | calcium ion-dependent exocytosis | Biological process | 4 | 57 | 295 | 13692 | 3.25709188224799 | 0.0341873599592524 | 0.494644838486345 | 1.46613443530746 | PPP3CA//PLCB1//PPP3CB//RAPGEF4 |
| GO:0071326 | cellular response to monosaccharide stimulus | Biological process | 4 | 57 | 295 | 13692 | 3.25709188224799 | 0.0341873599592524 | 0.494644838486345 | 1.46613443530746 | PPP3CB//HIF1A//TCF7L2//PPP3CA |
| GO:0071331 | cellular response to hexose stimulus | Biological process | 4 | 57 | 295 | 13692 | 3.25709188224799 | 0.0341873599592524 | 0.494644838486345 | 1.46613443530746 | PPP3CB//HIF1A//TCF7L2//PPP3CA |
| GO:0071333 | cellular response to glucose stimulus | Biological process | 4 | 57 | 295 | 13692 | 3.25709188224799 | 0.0341873599592524 | 0.494644838486345 | 1.46613443530746 | PPP3CB//HIF1A//TCF7L2//PPP3CA |
| GO:0007417 | central nervous system development | Biological process | 23 | 712 | 295 | 13692 | 1.49931441630166 | 0.0345039776197668 | 0.497583677253479 | 1.46213083655741 | CD9//NT5E//APAF1//ARF4//NAPA//TCF7L2//GFAP//MRF//GSN//ROBO1//RTN4//RBPJ//PLCB1//HIF1A//PLP1//CNP//ZEB1//EGR1//EXOC4//CLCF1//FMR1//MAPK9//NRCAM |
| GO:0048667 | cell morphogenesis involved in neuron differentiation | Biological process | 14 | 378 | 295 | 13692 | 1.71902071563089 | 0.0348259074309548 | 0.499640517619816 | 1.45809755869087 | SNAP25//CNP//ACTB//PICALM//ROBO1//NRP1//SEMA3C//ANK3//NRCAM//RTN4//CHN1//SEMA7A//NPTX1//SLC1A3 |
| GO:0015833 | peptide transport | Biological process | 8 | 174 | 295 | 13692 | 2.13395675043834 | 0.0348745434287554 | 0.499640517619816 | 1.45749146938631 | PPP3CB//RAPGEF4//SNAP25//GLUL//MGEA5//TCF7L2//HIF1A//PPP3CA |
| GO:0043069 | negative regulation of programmed cell death | Biological process | 20 | 599 | 295 | 13692 | 1.54970147986758 | 0.0350537197223004 | 0.500332979590053 | 1.45526589013229 | TCF7L2//TRIAP1//RTN4//PRKCI//RBPJ//CAT//PAK2//HIF1A//PLK2//FAIM//HIGD1A//KRT18//RRN3//KLHL20//SPRY2//DNAJA3//HSP90AB1//CLCF1//SERBP1//KIT |
| GO:0007413 | axonal fasciculation | Biological process | 2 | 14 | 295 | 13692 | 6.63050847457627 | 0.035493512010152 | 0.500332979590053 | 1.44985102597878 | RTN4//NRP1 |
| GO:0020027 | hemoglobin metabolic process | Biological process | 2 | 14 | 295 | 13692 | 6.63050847457627 | 0.035493512010152 | 0.500332979590053 | 1.44985102597878 | HIF1A//CAT |
| GO:0045922 | negative regulation of fatty acid metabolic process | Biological process | 2 | 14 | 295 | 13692 | 6.63050847457627 | 0.035493512010152 | 0.500332979590053 | 1.44985102597878 | INSIG1//INSIG2 |
| GO:0046716 | muscle cell homeostasis | Biological process | 2 | 14 | 295 | 13692 | 6.63050847457627 | 0.035493512010152 | 0.500332979590053 | 1.44985102597878 | PPP3CB//HIF1A |
| GO:0043408 | regulation of MAPK cascade | Biological process | 12 | 309 | 295 | 13692 | 1.80246832318578 | 0.0358798804640936 | 0.504158320367264 | 1.44514901250283 | NBR1//ITGB1//KIT//LPAR1//MAP3K4//PDCD4//MAPK9//PLCB1//SPRY2//CNKSR3//SEMA7A//PSAP |
| GO:0006509 | membrane protein ectodomain proteolysis | Biological process | 3 | 34 | 295 | 13692 | 4.09531405782652 | 0.0362322883820276 | 0.506041932470232 | 1.44090423565075 | PTPN3//ADAM10//SPPL2A |
| GO:0030155 | regulation of cell adhesion | Biological process | 10 | 241 | 295 | 13692 | 1.92587383078979 | 0.0362447916960887 | 0.506041932470232 | 1.44075439185599 | DDR1//PIK3CB//ATP5B//ADAM10//ITGB1//HSD17B12//ANK3//SDC4//NPTN//GSN |
| GO:0009914 | hormone transport | Biological process | 9 | 208 | 295 | 13692 | 2.00827900912647 | 0.0364690652867006 | 0.507556768942525 | 1.438075367644 | YWHAZ//PPP3CB//SNAP25//RAPGEF4//GLUL//MGEA5//TCF7L2//HIF1A//PPP3CA |
| GO:0034330 | cell junction organization | Biological process | 6 | 114 | 295 | 13692 | 2.44281891168599 | 0.0367316520622725 | 0.509593552661401 | 1.43495953815638 | CD9//UGT8//PRKCI//PTPRK//SDC4//ITGB1 |
| GO:0009743 | response to carbohydrate stimulus | Biological process | 8 | 177 | 295 | 13692 | 2.09778799195633 | 0.0379878026231659 | 0.521463081286445 | 1.42035582716741 | FADS1//EGR1//GLUL//HIF1A//TCF7L2//PLCB1//PPP3CB//PPP3CA |
| GO:0071902 | positive regulation of protein serine/threonine kinase activity | Biological process | 8 | 177 | 295 | 13692 | 2.09778799195633 | 0.0379878026231659 | 0.521463081286445 | 1.42035582716741 | KIT//MADD//LPAR1//RGD1306565//ZFP91//MAP3K4//HSP90AB1//SPRY2 |
| GO:0043624 | cellular protein complex disassembly | Biological process | 4 | 59 | 295 | 13692 | 3.1466819879345 | 0.0381138826096334 | 0.521463081286445 | 1.41891680798325 | CLASP2//DSTN//NAPA//GSN |
| GO:0071260 | cellular response to mechanical stimulus | Biological process | 4 | 59 | 295 | 13692 | 3.1466819879345 | 0.0381138826096334 | 0.521463081286445 | 1.41891680798325 | EGR1//ITGB1//GADD45A//HABP4 |
| GO:0006935 | chemotaxis | Biological process | 12 | 312 | 295 | 13692 | 1.7851368970013 | 0.0381819455047785 | 0.521463081286445 | 1.4181419465431 | KIT//ROBO1//NRP1//SEMA3C//ANK3//NRCAM//CHN1//ADAM10//CCL6//ELMO2//RGD1560691//LPAR1 |
| GO:0042330 | taxis | Biological process | 12 | 313 | 295 | 13692 | 1.77943358423133 | 0.0389719722579511 | 0.527926550778337 | 1.40924761527297 | KIT//ROBO1//NRP1//SEMA3C//ANK3//NRCAM//CHN1//ADAM10//CCL6//ELMO2//RGD1560691//LPAR1 |
| GO:0010828 | positive regulation of glucose transport | Biological process | 3 | 35 | 295 | 13692 | 3.97830508474576 | 0.0390164695374501 | 0.527926550778337 | 1.4087520309377 | PRKCI//MGEA5//RGD1304592 |
| GO:0048169 | regulation of long-term neuronal synaptic plasticity | Biological process | 3 | 35 | 295 | 13692 | 3.97830508474576 | 0.0390164695374501 | 0.527926550778337 | 1.4087520309377 | NPTN//KIT//EGR1 |
| GO:0034329 | cell junction assembly | Biological process | 5 | 87 | 295 | 13692 | 2.66744593804792 | 0.0396881951896534 | 0.535363223727509 | 1.40133865007233 | CD9//UGT8//PTPRK//SDC4//ITGB1 |
| GO:0007611 | learning or memory | Biological process | 8 | 179 | 295 | 13692 | 2.07434901997917 | 0.0401639172040923 | 0.537931725176901 | 1.39616393687004 | PLCB1//SLC24A2//PLK2//SNAP25//PJA2//HIF1A//KIT//EGR1 |
| GO:0010894 | negative regulation of steroid biosynthetic process | Biological process | 2 | 15 | 295 | 13692 | 6.18847457627119 | 0.0403822255577148 | 0.537931725176901 | 1.39380974976088 | INSIG1//INSIG2 |
| GO:0030252 | growth hormone secretion | Biological process | 2 | 15 | 295 | 13692 | 6.18847457627119 | 0.0403822255577148 | 0.537931725176901 | 1.39380974976088 | MGEA5//SNAP25 |
| GO:0051150 | regulation of smooth muscle cell differentiation | Biological process | 2 | 15 | 295 | 13692 | 6.18847457627119 | 0.0403822255577148 | 0.537931725176901 | 1.39380974976088 | PIAS1//ZEB1 |
| GO:0009056 | catabolic process | Biological process | 42 | 1487 | 295 | 13692 | 1.31094115099222 | 0.0404921234736262 | 0.537931725176901 | 1.39262944751668 | PARN//UPF2//ARIH2//PHYH//GLYR1//HIF1A//NT5E//NSF//ATP5B//APAF1//AUH//USP32//CUL4A//ADAMTS4//ACADSB//HADHA//LAMP1//PIK3CB//EI24//FAAH//CNP//PLCB1//GLA//SQSTM1//NBR1//TMED2//MGLL//DNAJB2//ACAP2//HSP90AB1//TAF1//RGN//CHN2//SPRY2//PRIMA1//GSTM2//CAT//KLHL20//PPP2R5C//CHN1//CES1D//PTPN3 |
| GO:0015931 | nucleobase-containing compound transport | Biological process | 5 | 88 | 295 | 13692 | 2.63713405238829 | 0.0413689115152773 | 0.546760927598049 | 1.38332590596278 | SIDT1//FMR1//HNRNPA1//SENP2//HNRNPA3 |
| GO:0034504 | protein localization to nucleus | Biological process | 9 | 213 | 295 | 13692 | 1.96113630938171 | 0.0414061651374435 | 0.546760927598049 | 1.38293499013922 | KEAP1//PPP3CA//KPNB1//PTTG1IP//SPTBN1//HSP90AB1//TCF7L2//ARL2BP//CALR |
| GO:0048519 | negative regulation of biological process | Biological process | 71 | 2719 | 295 | 13692 | 1.2119759881811 | 0.0416517436321934 | 0.547140477335953 | 1.38036681336908 | EGR1//ZEB1//CALR//MKX//PIAS1//DNAJA3//TCF7L2//PPP3CB//KEAP1//GADD45A//TRIAP1//ATP5B//PPP2R5C//CLASP2//DBC1//SESN1//PLK2//ADAM10//ITGB1//CD9//DDR1//ROBO1//SPRY2//PTPRK//RBPJ//DNAJB2//MGEA5//INSIG1//INSIG2//GFAP//RTN4//LPAR1//SIDT1//HSPA8//PLCB1//PDCD4//FOXN3//LRP4//EI24//HIF1A//NRP1//STIM2//HSP90AB1//CNKSR3//PRKCI//KIT//CAT//PAK2//FAIM//HIGD1A//KRT18//RRN3//KLHL20//CLCF1//GLA//NBR1//SERBP1//CR1L//PTPN3//FMR1//PPP3CA//PICALM//NT5E//RAPGEF4//ELF1//ITPR1//GSN//PLEKHA1//MGLL//SLC24A2 |
| GO:0006695 | cholesterol biosynthetic process | Biological process | 3 | 36 | 295 | 13692 | 3.86779661016949 | 0.0419062526434115 | 0.547140477335953 | 1.37772117307337 | SC5DL//INSIG1//INSIG2 |
| GO:0001776 | leukocyte homeostasis | Biological process | 4 | 61 | 295 | 13692 | 3.04351208669075 | 0.0422883002205001 | 0.547140477335953 | 1.37377977098205 | HIF1A//SPTA1//DNAJA3//PPP3CB |
| GO:0071322 | cellular response to carbohydrate stimulus | Biological process | 4 | 61 | 295 | 13692 | 3.04351208669075 | 0.0422883002205001 | 0.547140477335953 | 1.37377977098205 | PPP3CB//HIF1A//TCF7L2//PPP3CA |
| GO:0031346 | positive regulation of cell projection organization | Biological process | 7 | 149 | 295 | 13692 | 2.18050278694119 | 0.0424725068729069 | 0.547140477335953 | 1.37189210470417 | ITGB1//NPTN//PRKCI//RGD1560691//KIT//SEMA7A//ROBO1 |
| GO:0010646 | regulation of cell communication | Biological process | 39 | 1369 | 295 | 13692 | 1.32222703693157 | 0.0426128304652168 | 0.547140477335953 | 1.3704596177686 | PPP3CB//ANK3//NAPA//CAT//SNAP25//LRP4//TCF7L2//HIF1A//MRF//GLUL//MGEA5//CALR//RTN4//LPAR1//SPRY2//KIT//CLCF1//ARL2BP//PLK2//ZDHHC17//DNAJA3//ITGB1//NPTN//PLCB1//PPP3CA//EGR1//RAPGEF4//SLC1A3//EXOC4//ELF1//GFAP//PLEKHA1//SLC24A2//MGLL//ROBO1//CNKSR3//SEMA7A//TNKS2//PAK2 |
| GO:0080090 | regulation of primary metabolic process | Biological process | 81 | 3158 | 295 | 13692 | 1.19046811433969 | 0.0428094981922031 | 0.547140477335953 | 1.36845986288362 | EGR1//ZEB1//CALR//MKX//PIAS1//DNAJA3//TCF7L2//RGD1306565//KIT//MADD//LPAR1//LRP4//MAPK9//NPTN//SENP2//SQSTM1//EIF4G3//HIF1A//ANP32A//DDX1//ELF1//DMTF1//HDGF//KEAP1//CNOT6//HNRPDL//RNF141//TAF1//HDAC8//HMGN5//RBPJ//FMR1//EIF5B//GADD45A//ZFP91//SPRY2//INSIG1//INSIG2//RPS4X//NT5E//TMED2//PLCB1//NFIA//CNBP//PICALM//MRF//RRN3//ILF2//ATMIN//HSPA8//PDCD4//FOXN3//PTPRK//PSAP//CR1L//DNAJB2//AHCYL1//CAT//TRIM37//CAMKK1//ACAP2//HSP90AB1//RGN//RGD1560691//CHN2//CNKSR3//CLCF1//ARL2BP//HNRPH1//MAP3K4//CHN1//SDC4//MGEA5//PPP3CA//HNRNPK//ITGB1//PAK2//NRP1//PTPN3//PRKCI//CUL4A |
| GO:0048878 | chemical homeostasis | Biological process | 25 | 807 | 295 | 13692 | 1.43784260601096 | 0.0431442395255031 | 0.547140477335953 | 1.36507718162413 | ITGB1//RGN//SLC24A2//PIK3CB//STIM2//ARF1//HIF1A//ITPR1//NPTN//PLP1//CLDN11//MGEA5//MRF//CD9//UGT8//TCF7L2//NDRG1//PPP3CB//MAL//MPDZ//LPAR1//ATP5B//ASGR2//PPP3CA//SCN4B |
| GO:0006470 | protein dephosphorylation | Biological process | 6 | 119 | 295 | 13692 | 2.34017946161515 | 0.043803316984445 | 0.547140477335953 | 1.35849300151651 | PTP4A1//PTPRK//PTPN3//PPM1B//PPP3CA//PPP3CB |
| GO:0009605 | response to external stimulus | Biological process | 33 | 1128 | 295 | 13692 | 1.35784349080418 | 0.0441383104603245 | 0.547140477335953 | 1.3551842949125 | KIT//HIF1A//MAPK9//ROBO1//NRP1//SEMA3C//ANK3//NRCAM//APAF1//GATM//FADS1//CHN1//ADAM10//SQSTM1//NBR1//EGR1//ITGB1//GADD45A//HABP4//CR1L//SLC1A3//ZEB1//CAT//HIGD1A//RTN4//MGLL//SEMA7A//NT5E//GSN//CCL6//ELMO2//RGD1560691//LPAR1 |
| GO:0032940 | secretion by cell | Biological process | 19 | 577 | 295 | 13692 | 1.52834944041359 | 0.0441496792846464 | 0.547140477335953 | 1.35507244690893 | YWHAZ//GCGR//SNAP25//SCAMP1//EXOC4//NSF//PPP3CB//NAPA//RAPGEF4//GLUL//MGEA5//TCF7L2//RTN4//HIF1A//KIT//PPP3CA//CD14//RBPJ//PLCB1 |
| GO:0019752 | carboxylic acid metabolic process | Biological process | 22 | 692 | 295 | 13692 | 1.47557558538258 | 0.0441622052802718 | 0.547140477335953 | 1.35494924784363 | PHYH//HIF1A//GSTM2//MGST1//GLUL//SLC1A3//MAT2B//GATM//ACADSB//FAAH//SC5DL//MGLL//HSD17B12//HADHA//FADS1//MGST2//ELOVL7//RGN//MAPK9//PLP1//INSIG1//INSIG2 |
| GO:0043436 | oxoacid metabolic process | Biological process | 22 | 692 | 295 | 13692 | 1.47557558538258 | 0.0441622052802718 | 0.547140477335953 | 1.35494924784363 | PHYH//HIF1A//GSTM2//MGST1//GLUL//SLC1A3//MAT2B//GATM//ACADSB//FAAH//SC5DL//MGLL//HSD17B12//HADHA//FADS1//MGST2//ELOVL7//RGN//MAPK9//PLP1//INSIG1//INSIG2 |
| GO:1901135 | carbohydrate derivative metabolic process | Biological process | 24 | 770 | 295 | 13692 | 1.446656394453 | 0.0442970385923158 | 0.547140477335953 | 1.35362530686961 | MGEA5//UAP1//NT5E//NSF//ATP5B//TNKS2//ST6GALNAC3//GALNT1//ADAMTS4//MAT2B//UGT8//KIT//ASGR2//TCF7L2//GLA//TMED2//TSTA3//ACAP2//CHN2//UXS1//SPRY2//PLCB1//CHN1//HIF1A |
| GO:0001678 | cellular glucose homeostasis | Biological process | 4 | 62 | 295 | 13692 | 2.9944231820667 | 0.0444685212217452 | 0.547140477335953 | 1.35194731255499 | PPP3CB//HIF1A//TCF7L2//PPP3CA |
| GO:0050793 | regulation of developmental process | Biological process | 38 | 1333 | 295 | 13692 | 1.32311721998296 | 0.0446879724779926 | 0.547140477335953 | 1.34980934911218 | HIF1A//SPTA1//ERMN//CDC42SE2//GFAP//MAPK9//NRCAM//ITGB1//NPTN//PRKCI//RGD1560691//RTN4//LPAR1//RBPJ//NBR1//NRP1//ZEB1//TCF7L2//MRF//ROBO1//KIT//PLCB1//INSIG1//KEAP1//MKX//S1PR5//CALR//ADAM10//RGD1309676//SEMA7A//CLCF1//FAIM//CHN1//RAPGEF4//PIAS1//SNAP25//EGR1//FNDC5 |
| GO:0007043 | cell-cell junction assembly | Biological process | 3 | 37 | 295 | 13692 | 3.7632615666514 | 0.0449005973214897 | 0.547140477335953 | 1.34774788145322 | CD9//UGT8//ITGB1 |
| GO:0000165 | MAPK cascade | Biological process | 14 | 392 | 295 | 13692 | 1.65762711864407 | 0.0449352199002317 | 0.547140477335953 | 1.34741312865402 | MAPK10//MAPK9//RGD1306565//NBR1//PSAP//ITGB1//KIT//LPAR1//MAP3K4//PDCD4//PLCB1//SPRY2//CNKSR3//SEMA7A |
| GO:0043085 | positive regulation of catalytic activity | Biological process | 21 | 655 | 295 | 13692 | 1.48806831414154 | 0.0450990164014506 | 0.547140477335953 | 1.34583292987549 | RGD1306565//KIT//MADD//LPAR1//MAPK9//ROBO1//APAF1//DNAJA3//ZFP91//RPL5//CAMKK1//RGN//CHN2//MAP3K4//PLCB1//CHN1//SDC4//SERINC1//PAK2//HSP90AB1//SPRY2 |
| GO:0048523 | negative regulation of cellular process | Biological process | 65 | 2472 | 295 | 13692 | 1.22042126049037 | 0.0452556418444312 | 0.547140477335953 | 1.34432727128379 | EGR1//ZEB1//CALR//MKX//PIAS1//DNAJA3//TCF7L2//KEAP1//GADD45A//TRIAP1//ATP5B//PPP2R5C//CLASP2//DBC1//SESN1//PLK2//ADAM10//ITGB1//CD9//DDR1//ROBO1//SPRY2//PTPRK//RBPJ//DNAJB2//GFAP//RTN4//LPAR1//HSPA8//PLCB1//PDCD4//FOXN3//LRP4//EI24//NRP1//HIF1A//HSP90AB1//CNKSR3//PRKCI//KIT//CAT//PAK2//FAIM//HIGD1A//KRT18//RRN3//KLHL20//CLCF1//GLA//INSIG1//NBR1//INSIG2//SERBP1//PTPN3//FMR1//PPP3CA//PICALM//RAPGEF4//ELF1//GSN//PLEKHA1//MGEA5//MGLL//SLC24A2 |
| GO:0010647 | positive regulation of cell communication | Biological process | 23 | 733 | 295 | 13692 | 1.45635997872685 | 0.04542579726973 | 0.547140477335953 | 1.34269744163565 | ANK3//CAT//HIF1A//LPAR1//KIT//CLCF1//ARL2BP//PLK2//ZDHHC17//ITGB1//NPTN//PLCB1//SLC1A3//EXOC4//SPRY2//TCF7L2//GLUL//EGR1//GFAP//SLC24A2//SEMA7A//TNKS2//PAK2 |
| GO:0006929 | substrate-dependent cell migration | Biological process | 2 | 16 | 295 | 13692 | 5.80169491525424 | 0.0455079221484914 | 0.547140477335953 | 1.34191299354317 | ATP5B//ROBO1 |
| GO:0009084 | glutamine family amino acid biosynthetic process | Biological process | 2 | 16 | 295 | 13692 | 5.80169491525424 | 0.0455079221484914 | 0.547140477335953 | 1.34191299354317 | SLC1A3//GLUL |
| GO:0010869 | regulation of receptor biosynthetic process | Biological process | 2 | 16 | 295 | 13692 | 5.80169491525424 | 0.0455079221484914 | 0.547140477335953 | 1.34191299354317 | HIF1A//HNRNPK |
| GO:0033327 | Leydig cell differentiation | Biological process | 2 | 16 | 295 | 13692 | 5.80169491525424 | 0.0455079221484914 | 0.547140477335953 | 1.34191299354317 | MGST1//PLEKHA1 |
| GO:0035088 | establishment or maintenance of apical/basal cell polarity | Biological process | 2 | 16 | 295 | 13692 | 5.80169491525424 | 0.0455079221484914 | 0.547140477335953 | 1.34191299354317 | PRKCI//PRICKLE2 |
| GO:0045939 | negative regulation of steroid metabolic process | Biological process | 2 | 16 | 295 | 13692 | 5.80169491525424 | 0.0455079221484914 | 0.547140477335953 | 1.34191299354317 | INSIG1//INSIG2 |
| GO:0048016 | inositol phosphate-mediated signaling | Biological process | 2 | 16 | 295 | 13692 | 5.80169491525424 | 0.0455079221484914 | 0.547140477335953 | 1.34191299354317 | PPP3CA//ITPR1 |
| GO:0060251 | regulation of glial cell proliferation | Biological process | 2 | 16 | 295 | 13692 | 5.80169491525424 | 0.0455079221484914 | 0.547140477335953 | 1.34191299354317 | GFAP//PRKCI |
| GO:0061082 | myeloid leukocyte cytokine production | Biological process | 2 | 16 | 295 | 13692 | 5.80169491525424 | 0.0455079221484914 | 0.547140477335953 | 1.34191299354317 | KIT//SEMA7A |
| GO:0061245 | establishment or maintenance of bipolar cell polarity | Biological process | 2 | 16 | 295 | 13692 | 5.80169491525424 | 0.0455079221484914 | 0.547140477335953 | 1.34191299354317 | PRKCI//PRICKLE2 |
| GO:0070972 | protein localization to endoplasmic reticulum | Biological process | 2 | 16 | 295 | 13692 | 5.80169491525424 | 0.0455079221484914 | 0.547140477335953 | 1.34191299354317 | SRPR//KDELR3 |
| GO:0019538 | protein metabolic process | Biological process | 86 | 3387 | 295 | 13692 | 1.1784960441969 | 0.04555343846433 | 0.547140477335953 | 1.34147883604061 | RGD1306565//KIT//MADD//LPAR1//TNKS2//ARIH2//FKBP9//EIF3D//LRP4//MAPK9//NPTN//SENP2//SQSTM1//EIF4G3//HSPA8//CANX//CCT4//CALR//HSPA9//CCT5//CCT2//DNAJA4//HSP90AB1//MESDC2//DNAJA3//DNAJB2//PJA2//KLHL20//RPS4X//EIF5//RPL5//RGD1564051//EIF5B//EEF2//FMR1//PPP3CB//MAPK10//PAK2//ADAM10//CAMKK1//PLK2//PRKCI//TLK2//OXSR1//NEK7//GADD45A//PPM1B//PPP3CA//PTPRK//PTPN3//ST6GALNAC3//GALNT1//ADAMTS4//BLMH//LONRF1//SPPL2A//PPP2R5C//USP32//CUL4A//KLHL12//CR1L//SPTBN1//ZFP91//ASGR2//SPRY2//TCF7L2//MGEA5//TAF1//DDR1//ZDHHC17//CNKSR3//EGR1//PIAS1//PTP4A1//PLCB1//CLCF1//ARL2BP//MAP3K4//PDCD4//SDC4//ITGB1//NRP1//HSPH1//HIF1A//TRIM37//DNAJC7 |
| GO:0023056 | positive regulation of signaling | Biological process | 23 | 734 | 295 | 13692 | 1.45437583706646 | 0.0460025368164006 | 0.551024921866394 | 1.33721821842742 | NSF//CAT//HIF1A//LPAR1//KIT//CLCF1//ARL2BP//PLK2//ZDHHC17//ITGB1//NPTN//PLCB1//SLC1A3//EXOC4//SPRY2//TCF7L2//GLUL//EGR1//GFAP//SLC24A2//SEMA7A//TNKS2//PAK2 |
| GO:0019221 | cytokine-mediated signaling pathway | Biological process | 7 | 152 | 295 | 13692 | 2.13746654772525 | 0.0464366430941254 | 0.551374624900153 | 1.33313918295568 | KRT18//PLCB1//KIT//HSP90AB1//DNAJA3//ROBO1//EGR1 |
| GO:0043280 | positive regulation of cysteine-type endopeptidase activity involved in apoptotic process | Biological process | 5 | 91 | 295 | 13692 | 2.55019556714472 | 0.0466665459273909 | 0.551374624900153 | 1.33099434268005 | MAPK9//ROBO1//APAF1//DNAJA3//RGD1306565 |
| GO:2001056 | positive regulation of cysteine-type endopeptidase activity | Biological process | 5 | 91 | 295 | 13692 | 2.55019556714472 | 0.0466665459273909 | 0.551374624900153 | 1.33099434268005 | MAPK9//ROBO1//APAF1//DNAJA3//RGD1306565 |
| GO:0006690 | icosanoid metabolic process | Biological process | 4 | 63 | 295 | 13692 | 2.94689265536723 | 0.0467106864504999 | 0.551374624900153 | 1.33058375036425 | MGST2//MGLL//FADS1//MAPK9 |
| GO:0046486 | glycerolipid metabolic process | Biological process | 9 | 218 | 295 | 13692 | 1.91615611879956 | 0.0467767819622469 | 0.551374624900153 | 1.32996965905767 | CAT//INSIG1//INSIG2//ETNK1//SERINC1//MGLL//PIK3CB//CES1D//PLCB1 |
| GO:0071845 | cellular component disassembly at cellular level | Biological process | 6 | 121 | 295 | 13692 | 2.30149880935705 | 0.0468547171725189 | 0.551374624900153 | 1.32924667929346 | APAF1//CLASP2//DDR1//DSTN//NAPA//GSN |
| GO:0045860 | positive regulation of protein kinase activity | Biological process | 11 | 287 | 295 | 13692 | 1.77891690781315 | 0.0469121202298716 | 0.551374624900153 | 1.32871493833135 | RGD1306565//KIT//MADD//LPAR1//ZFP91//CAMKK1//MAP3K4//PAK2//HSP90AB1//SPRY2//SDC4 |
| GO:0051347 | positive regulation of transferase activity | Biological process | 12 | 323 | 295 | 13692 | 1.72434276119011 | 0.0475135830677266 | 0.556950663553244 | 1.3231822175735 | RGD1306565//KIT//MADD//LPAR1//ZFP91//CAMKK1//MAP3K4//SDC4//PAK2//HSP90AB1//SPRY2//SERINC1 |
| GO:0014910 | regulation of smooth muscle cell migration | Biological process | 3 | 38 | 295 | 13692 | 3.66422836752899 | 0.0479983570819687 | 0.559640418742954 | 1.31877362767538 | RAPGEF4//RBPJ//LPAR1 |
| GO:0042398 | cellular modified amino acid biosynthetic process | Biological process | 3 | 38 | 295 | 13692 | 3.66422836752899 | 0.0479983570819687 | 0.559640418742954 | 1.31877362767538 | MAT2B//GATM//MGST2 |
| GO:0051170 | nuclear import | Biological process | 8 | 186 | 295 | 13692 | 1.9962821213778 | 0.0484295664086861 | 0.56317034253496 | 1.31488941918716 | KEAP1//PPP3CA//KPNB1//PTTG1IP//SPTBN1//HSP90AB1//TCF7L2//HNRNPA1 |
| GO:0009062 | fatty acid catabolic process | Biological process | 4 | 64 | 295 | 13692 | 2.90084745762712 | 0.0490146926000477 | 0.566536939813484 | 1.3096737167335 | PHYH//ACADSB//HADHA//FAAH |
| GO:0021675 | nerve development | Biological process | 4 | 64 | 295 | 13692 | 2.90084745762712 | 0.0490146926000477 | 0.566536939813484 | 1.3096737167335 | SLC1A3//RTN4//NRP1//NPTX1 |
| GO:0046883 | regulation of hormone secretion | Biological process | 7 | 154 | 295 | 13692 | 2.10970724191063 | 0.0492068152719177 | 0.566536939813484 | 1.30797474215124 | PPP3CB//SNAP25//GLUL//MGEA5//TCF7L2//HIF1A//PPP3CA |
| GO:0003001 | generation of a signal involved in cell-cell signaling | Biological process | 12 | 325 | 295 | 13692 | 1.71373142112125 | 0.0493652169271786 | 0.566536939813484 | 1.30657895021138 | YWHAZ//SNAP25//NSF//PPP3CB//NAPA//RAPGEF4//GLUL//MGEA5//TCF7L2//RTN4//HIF1A//PPP3CA |
| GO:0023061 | signal release | Biological process | 12 | 325 | 295 | 13692 | 1.71373142112125 | 0.0493652169271786 | 0.566536939813484 | 1.30657895021138 | YWHAZ//SNAP25//NSF//PPP3CB//NAPA//RAPGEF4//GLUL//MGEA5//TCF7L2//RTN4//HIF1A//PPP3CA |
