# Supplementary material for: Gene Expression in the Hippocampus in a Rat Model of Premenstrual Dysphoric Disorder After Treatment With Baixiangdan Capsules
Source: Front Psychol. 2018 Nov 13;9:2065. doi: 10.3389/fpsyg.2018.02065 (PMC6242977; doi:10.3389/fpsyg.2018.02065)
Supplement: Supplementary file 3 [file Data_Sheet_3.ZIP › Data Analysis Folder/GO Analysis Report/BXD vs blank (down)/CC_result(Rat).html]

| GO.ID | Term | Ontology | Count | Pop.Hits | List.Total | Pop.Total | Fold.Enrichment | Pvalue | FDR | Enrichment.Score | GENES |
| --- | --- | --- | --- | --- | --- | --- | --- | --- | --- | --- | --- |
| GO:0044464 | cell part | Cellular component | 305 | 11835 | 329 | 15288 | 1.19752986543699 | 5.64823901089653e-14 | 1.94744532645545e-11 | 13.248086933961 | CAT//GSTM2//ITGB1//PPP3CB//GCGR//ITPR1//MAPK10//CNP//DDR1//SLC1A3//NPTN//NT5E//ROBO1//CD14//NSF//S1PR5//CAP1//ARF4//LRP4//RTN4//PRKCI//PICALM//MADD//RASGRF2//CLASP2//LPAR1//MAGT1//STIM2//ATP5B//PJA2//SERBP1//NRP1//RAPGEF4//FNDC5//GPR158//HAUS7//SERINC1//MGST2//DNAJB4//NDRG1//SPTBN1//GPC5//HIATL1//MESDC2//GOPC//MLC1//SCN4B//MAGEE1//CDH19//PLEKHA1//ANK3//DDX50//RGD1304592//PTTG1IP//MAP4//NRCAM//UAP1//LRRC4//CDC42SE2//FAAH//GABARAPL2//GOSR1//ZDHHC17//EXOC4//PPP2R5C//RGD1564051//RPS4X//SMC2//ZFP828//TNKS2//HSPA8//PLCB1//KPNB1//GLUL//MAL//VAMP1//MPDZ//UGT8//KIT//TMED2//PLK2//CHN1//CHN2//PLCL1//NAPA//RLBP1//HSP90AB1//ZFP644//ARHGEF26//RLF//ZFP280D//ZIC4//DCLK3//FUS//DNAJA3//TRIM37//TBC1D14//AHCYL1//CLCF1//DSTN//LOC502894//NCALD//ZMAT2//SQSTM1//NBR1//DNAJB2//POLA1//HMGN5//HNRNPK//NEK7//LSM14A//CALR//GFAP//KRT18//TLK2//GSN//PTPRK//NCL//EEF2//HNRNPA1//RPL5//ACTB//DDX1//HNRNPH2//ILF2//HNRNPH3//FCGRT//LAMP1//PSAP//GLA//PPP3CA//MOBP//SLC25A5//YWHAZ//ACADSB//MAPK9//GATM//PHYH//HIGD1A//MGEA5//HADHA//CLCC1//MGST1//UXS1//GHITM//NARS//HSPA9//TIMMDC1//RGD1309676//AUH//SLC25A13//COA5//MAT2B//TRIAP1//PDCD4//ARF1//CNBP//APAF1//PIK3CB//CES1D//ACTG1//GPCPD1//ELMO2//PDCD6IP//TCF7L2//ANP32A//CANX//PTP4A1//PDIA3//INSIG1//PDIA4//KEAP1//INSIG2//SEC62//FKBP9//KDELR3//ORMDL3//TMED9//ELOVL7//SUMF1//ACBD5//PLP1//LRP12//EGR1//FMR1//RGN//GADD45A//NFIA//ZEB1//CCT4//PAK2//HIF1A//ADAM10//CAMKK1//LUZP1//FADS1//ELF1//DMTF1//HDGF//HNRNPM//HNRPH1//ANP32B//DDX46//ZFP91//MRFAP1//CNOT6//BLMH//HSPH1//MKX//IK//MRF//SESN1//TTC37//CBX3//CCT2//PIAS1//ZFP445//RGD1562291//DNAJC7//KLHL20//HNRPDL//RGD1560691//ZBTB2//VPRBP//ARIH2//TAF1//WDR13//PARN//GLYR1//FLYWCH1//HABP4//UPF2//ANP32E//HNRNPA3//HDAC8//TSC22D1//ARL2BP//RGD1564943//RBPJ//HMG1L1//SEC24C//SRSF1//SENP2//SNAP25//KCNAB1//NELL2//PLS3//DBC1//FAIM//BCAS1//WIPF3//PRUNE2//CCT5//ERMN//NDRG3//KLHL24//EHBP1//MTMR6//HARS//MAP3K4//CNKSR3//PRICKLE2//PTPN3//MAGI1//DDHD2//FOXN3//SPPL2A//VPS16//SRPR//HSD17B12//EI24//AZI1//SCAMP1//GALNT1//LAPTM4A//USP32//OPALIN//KLHL12//NPTX1//MGLL//SLMAP//CCT6A//CPSF3//EIF3D//ACTR10//CACNA2D3//CLDN11//CLDN12//SDC4//SPTA1//PPM1B//CD9//CR1L//SEMA7A//RGD1308874//DOCK9//GAR1//CLCN2//ATP6V1D//MED20//ARCN1//ST6GALNAC3//PRIMA1//CUL4A//CDC40 |
| GO:0005623 | cell | Cellular component | 305 | 11848 | 329 | 15288 | 1.19621589782637 | 7.25305521957337e-14 | 1.94744532645545e-11 | 13.1394790161182 | CAT//GSTM2//ITGB1//PPP3CB//GCGR//ITPR1//MAPK10//CNP//DDR1//SLC1A3//NPTN//NT5E//ROBO1//CD14//NSF//S1PR5//CAP1//ARF4//LRP4//RTN4//PRKCI//PICALM//MADD//RASGRF2//CLASP2//LPAR1//MAGT1//STIM2//ATP5B//PJA2//SERBP1//NRP1//RAPGEF4//FNDC5//GPR158//HAUS7//SERINC1//MGST2//DNAJB4//NDRG1//SPTBN1//GPC5//HIATL1//MESDC2//GOPC//MLC1//SCN4B//MAGEE1//CDH19//PLEKHA1//ANK3//DDX50//RGD1304592//PTTG1IP//MAP4//NRCAM//UAP1//LRRC4//CDC42SE2//FAAH//GABARAPL2//GOSR1//ZDHHC17//EXOC4//PPP2R5C//RGD1564051//RPS4X//SMC2//ZFP828//TNKS2//HSPA8//PLCB1//KPNB1//GLUL//MAL//VAMP1//MPDZ//UGT8//KIT//TMED2//PLK2//CHN1//CHN2//PLCL1//NAPA//RLBP1//HSP90AB1//ZFP644//ARHGEF26//RLF//ZFP280D//ZIC4//DCLK3//FUS//DNAJA3//TRIM37//TBC1D14//AHCYL1//CLCF1//DSTN//LOC502894//NCALD//ZMAT2//SQSTM1//NBR1//DNAJB2//POLA1//HMGN5//HNRNPK//NEK7//LSM14A//CALR//GFAP//KRT18//TLK2//GSN//PTPRK//NCL//EEF2//HNRNPA1//RPL5//ACTB//DDX1//HNRNPH2//ILF2//HNRNPH3//FCGRT//LAMP1//PSAP//GLA//PPP3CA//MOBP//SLC25A5//YWHAZ//ACADSB//MAPK9//GATM//PHYH//HIGD1A//MGEA5//HADHA//CLCC1//MGST1//UXS1//GHITM//NARS//HSPA9//TIMMDC1//RGD1309676//AUH//SLC25A13//COA5//MAT2B//TRIAP1//PDCD4//ARF1//CNBP//APAF1//PIK3CB//CES1D//ACTG1//GPCPD1//ELMO2//PDCD6IP//TCF7L2//ANP32A//CANX//PTP4A1//PDIA3//INSIG1//PDIA4//KEAP1//INSIG2//SEC62//FKBP9//KDELR3//ORMDL3//TMED9//ELOVL7//SUMF1//ACBD5//PLP1//LRP12//EGR1//FMR1//RGN//GADD45A//NFIA//ZEB1//CCT4//PAK2//HIF1A//ADAM10//CAMKK1//LUZP1//FADS1//ELF1//DMTF1//HDGF//HNRNPM//HNRPH1//ANP32B//DDX46//ZFP91//MRFAP1//CNOT6//BLMH//HSPH1//MKX//IK//MRF//SESN1//TTC37//CBX3//CCT2//PIAS1//ZFP445//RGD1562291//DNAJC7//KLHL20//HNRPDL//RGD1560691//ZBTB2//VPRBP//ARIH2//TAF1//WDR13//PARN//GLYR1//FLYWCH1//HABP4//UPF2//ANP32E//HNRNPA3//HDAC8//TSC22D1//ARL2BP//RGD1564943//RBPJ//HMG1L1//SEC24C//SRSF1//SENP2//SNAP25//KCNAB1//NELL2//PLS3//DBC1//FAIM//BCAS1//WIPF3//PRUNE2//CCT5//ERMN//NDRG3//KLHL24//EHBP1//MTMR6//HARS//MAP3K4//CNKSR3//PRICKLE2//PTPN3//MAGI1//DDHD2//FOXN3//SPPL2A//VPS16//SRPR//HSD17B12//EI24//AZI1//SCAMP1//GALNT1//LAPTM4A//USP32//OPALIN//KLHL12//NPTX1//MGLL//SLMAP//CCT6A//CPSF3//EIF3D//ACTR10//CACNA2D3//CLDN11//CLDN12//SDC4//SPTA1//PPM1B//CD9//CR1L//SEMA7A//RGD1308874//DOCK9//GAR1//CLCN2//ATP6V1D//MED20//ARCN1//ST6GALNAC3//PRIMA1//CUL4A//CDC40 |
| GO:0005737 | cytoplasm | Cellular component | 224 | 7544 | 329 | 15288 | 1.37975226190745 | 2.88898402459908e-12 | 5.17128140403235e-10 | 11.5392548596953 | FAAH//GABARAPL2//GOSR1//PJA2//ZDHHC17//EXOC4//RGD1564051//RPS4X//TNKS2//ATP5B//SQSTM1//NBR1//LSM14A//ITGB1//KIT//CALR//CAT//LAMP1//PSAP//MLC1//GLA//PPP3CA//GLUL//MOBP//SLC25A5//MAPK10//YWHAZ//ACADSB//VAMP1//MAPK9//GATM//PHYH//HIGD1A//MGEA5//HADHA//CLCC1//MGST1//UXS1//GHITM//NARS//HSPA9//HSP90AB1//TIMMDC1//DNAJA3//RGD1309676//AUH//SLC25A13//COA5//MAT2B//TRIAP1//HSPA8//PLCB1//PPP3CB//PDCD4//ARF1//CNBP//APAF1//ACTB//PRKCI//PIK3CB//CES1D//NRP1//RAPGEF4//ACTG1//GSN//NDRG1//GPCPD1//ELMO2//UAP1//PDCD6IP//NCALD//TCF7L2//MAL//ANP32A//CANX//PTP4A1//PDIA3//INSIG1//TMED2//RTN4//PDIA4//STIM2//KEAP1//FNDC5//INSIG2//SEC62//MGST2//FKBP9//MESDC2//KDELR3//ORMDL3//TMED9//ELOVL7//SUMF1//TRIM37//ACBD5//RPL5//CNP//SLC1A3//ARL2BP//SPPL2A//GCGR//SNAP25//PICALM//VPS16//SRPR//ITPR1//HSD17B12//FADS1//RASGRF2//SERINC1//EI24//SCAMP1//ADAM10//ARF4//GALNT1//CLASP2//LAPTM4A//USP32//KLHL20//GOPC//OPALIN//NSF//KLHL12//NPTX1//LPAR1//MGLL//CCT4//PLK2//HAUS7//CCT5//AZI1//DDHD2//SLMAP//KRT18//NEK7//CCT6A//CCT2//EIF3D//NCL//SPTA1//MAGT1//FMR1//DDX1//SENP2//ANP32E//ATP6V1D//ANK3//HABP4//SEC24C//ARCN1//ST6GALNAC3//SPTBN1//CAP1//DSTN//MPDZ//SDC4//PAK2//SERBP1//MRFAP1//TLK2//MAGEE1//TTC37//EGR1//GFAP//GSTM2//KPNB1//RGN//ZEB1//HIF1A//EEF2//HNRNPA1//KCNAB1//NT5E//ROBO1//CAMKK1//NELL2//PLS3//PLCL1//POLA1//ELF1//DMTF1//HDGF//HNRNPK//DBC1//FAIM//BCAS1//WIPF3//CNOT6//BLMH//HSPH1//PRUNE2//DNAJB4//ERMN//NDRG3//DNAJC7//KLHL24//HNRPDL//EHBP1//MTMR6//RGD1560691//HARS//MAP3K4//CNKSR3//HNRNPH2//ILF2//PRICKLE2//ARIH2//WDR13//FUS//PARN//FLYWCH1//UPF2//PLEKHA1//HNRNPA3//SMC2//PTPN3//HDAC8//PTTG1IP//MAP4//TSC22D1//MAGI1//RBPJ//CDC42SE2 |
| GO:0005622 | intracellular | Cellular component | 277 | 10529 | 329 | 15288 | 1.22249592311407 | 9.39783284634172e-11 | 1.26165905962138e-08 | 10.0269722837818 | FAAH//GABARAPL2//GOSR1//PJA2//ZDHHC17//EXOC4//PPP2R5C//RGD1564051//RPS4X//SMC2//ZFP828//TNKS2//ATP5B//SQSTM1//NBR1//DNAJB2//CLASP2//POLA1//HMGN5//PLCB1//HNRNPK//NEK7//LSM14A//ITGB1//KIT//CALR//GFAP//KRT18//TLK2//HSPA8//NCL//EEF2//HNRNPA1//RPL5//ACTB//DDX1//HNRNPH2//ILF2//HNRNPH3//CAT//LAMP1//PSAP//MLC1//GLA//PPP3CA//GLUL//MOBP//SLC25A5//MAPK10//YWHAZ//ACADSB//VAMP1//MAPK9//GATM//PHYH//HIGD1A//MGEA5//HADHA//CLCC1//MGST1//UXS1//GHITM//NARS//HSPA9//HSP90AB1//TIMMDC1//DNAJA3//RGD1309676//AUH//SLC25A13//COA5//MAT2B//TRIAP1//PPP3CB//PDCD4//ARF1//CNBP//APAF1//PRKCI//PIK3CB//CES1D//NRP1//RAPGEF4//ACTG1//GSN//NDRG1//GPCPD1//ELMO2//UAP1//PDCD6IP//NCALD//TCF7L2//MAL//ANP32A//CANX//PTP4A1//PDIA3//INSIG1//TMED2//RTN4//PDIA4//STIM2//KEAP1//FNDC5//INSIG2//SEC62//MGST2//FKBP9//MESDC2//KDELR3//ORMDL3//TMED9//ELOVL7//SUMF1//TRIM37//ACBD5//EGR1//FMR1//RGN//GADD45A//NFIA//ZEB1//CCT4//PAK2//HIF1A//ADAM10//CAMKK1//LUZP1//FADS1//ELF1//PICALM//DMTF1//HDGF//HNRNPM//HNRPH1//ANP32B//DDX46//ZFP91//SERBP1//MRFAP1//CNOT6//BLMH//HSPH1//MKX//IK//MRF//SESN1//TTC37//DNAJB4//CBX3//CCT2//PIAS1//ZFP445//RGD1562291//DNAJC7//KLHL20//HNRPDL//SPTBN1//RGD1560691//ZBTB2//VPRBP//ARIH2//MAGEE1//TAF1//WDR13//FUS//PARN//GLYR1//FLYWCH1//HABP4//UPF2//PLEKHA1//DDX50//ANP32E//HNRNPA3//HDAC8//PTTG1IP//TSC22D1//ARL2BP//RGD1564943//RBPJ//HMG1L1//SEC24C//SRSF1//CDC42SE2//ITPR1//SENP2//GSTM2//KPNB1//SNAP25//CNP//MPDZ//KCNAB1//NT5E//ROBO1//NSF//CAP1//ARF4//NELL2//PLS3//PLCL1//RASGRF2//LPAR1//DBC1//FAIM//BCAS1//WIPF3//PRUNE2//HAUS7//CCT5//ERMN//NDRG3//KLHL24//EHBP1//MTMR6//HARS//MAP3K4//CNKSR3//GOPC//PRICKLE2//PTPN3//MAP4//MAGI1//DSTN//DDHD2//FOXN3//SLC1A3//SPPL2A//GCGR//VPS16//SRPR//HSD17B12//SERINC1//EI24//AZI1//SCAMP1//GALNT1//LAPTM4A//USP32//OPALIN//KLHL12//NPTX1//MGLL//PLK2//SLMAP//CCT6A//CPSF3//EIF3D//ACTR10//SPTA1//MAGT1//GAR1//CLCN2//ATP6V1D//ANK3//MED20//ARCN1//ST6GALNAC3//CUL4A//SDC4//CDC40//UGT8//CHN1//CHN2//NAPA//RLBP1//ZFP644//ARHGEF26//RLF//ZFP280D//ZIC4//DCLK3//TBC1D14//AHCYL1//CLCF1//LOC502894//ZMAT2 |
| GO:0044424 | intracellular part | Cellular component | 261 | 9903 | 329 | 15288 | 1.22469657808401 | 4.27347156061975e-09 | 4.58970845610561e-07 | 8.36921918186677 | FAAH//GABARAPL2//GOSR1//PJA2//ZDHHC17//EXOC4//PPP2R5C//RGD1564051//RPS4X//SMC2//ZFP828//TNKS2//ATP5B//SQSTM1//NBR1//DNAJB2//CLASP2//POLA1//HMGN5//PLCB1//HNRNPK//NEK7//LSM14A//ITGB1//KIT//CALR//GFAP//KRT18//TLK2//HSPA8//NCL//EEF2//HNRNPA1//RPL5//ACTB//DDX1//HNRNPH2//ILF2//HNRNPH3//CAT//LAMP1//PSAP//MLC1//GLA//PPP3CA//GLUL//MOBP//SLC25A5//MAPK10//YWHAZ//ACADSB//VAMP1//MAPK9//GATM//PHYH//HIGD1A//MGEA5//HADHA//CLCC1//MGST1//UXS1//GHITM//NARS//HSPA9//HSP90AB1//TIMMDC1//DNAJA3//RGD1309676//AUH//SLC25A13//COA5//MAT2B//TRIAP1//PPP3CB//PDCD4//ARF1//CNBP//APAF1//PRKCI//PIK3CB//CES1D//NRP1//RAPGEF4//ACTG1//GSN//NDRG1//GPCPD1//ELMO2//UAP1//PDCD6IP//NCALD//TCF7L2//MAL//ANP32A//CANX//PTP4A1//PDIA3//INSIG1//TMED2//RTN4//PDIA4//STIM2//KEAP1//FNDC5//INSIG2//SEC62//MGST2//FKBP9//MESDC2//KDELR3//ORMDL3//TMED9//ELOVL7//SUMF1//TRIM37//ACBD5//EGR1//FMR1//RGN//GADD45A//NFIA//ZEB1//CCT4//PAK2//HIF1A//ADAM10//CAMKK1//LUZP1//FADS1//ELF1//PICALM//DMTF1//HDGF//HNRNPM//HNRPH1//ANP32B//DDX46//ZFP91//SERBP1//MRFAP1//CNOT6//BLMH//HSPH1//MKX//IK//MRF//SESN1//TTC37//DNAJB4//CBX3//CCT2//PIAS1//ZFP445//RGD1562291//DNAJC7//KLHL20//HNRPDL//SPTBN1//RGD1560691//ZBTB2//VPRBP//ARIH2//MAGEE1//TAF1//WDR13//FUS//PARN//GLYR1//FLYWCH1//HABP4//UPF2//PLEKHA1//DDX50//ANP32E//HNRNPA3//HDAC8//PTTG1IP//TSC22D1//ARL2BP//RGD1564943//RBPJ//HMG1L1//SEC24C//SRSF1//CDC42SE2//ITPR1//SENP2//GSTM2//KPNB1//SNAP25//CNP//MPDZ//KCNAB1//NT5E//ROBO1//NSF//CAP1//ARF4//NELL2//PLS3//PLCL1//RASGRF2//LPAR1//DBC1//FAIM//BCAS1//WIPF3//PRUNE2//HAUS7//CCT5//ERMN//NDRG3//KLHL24//EHBP1//MTMR6//HARS//MAP3K4//CNKSR3//GOPC//PRICKLE2//PTPN3//MAP4//MAGI1//DSTN//DDHD2//FOXN3//SLC1A3//SPPL2A//GCGR//VPS16//SRPR//HSD17B12//SERINC1//EI24//AZI1//SCAMP1//GALNT1//LAPTM4A//USP32//OPALIN//KLHL12//NPTX1//MGLL//PLK2//SLMAP//CCT6A//CPSF3//EIF3D//ACTR10//SPTA1//MAGT1//GAR1//CLCN2//ATP6V1D//ANK3//MED20//ARCN1//ST6GALNAC3//CUL4A//SDC4//CDC40 |
| GO:0043231 | intracellular membrane-bounded organelle | Cellular component | 212 | 7493 | 329 | 15288 | 1.31472494895945 | 8.80584165363461e-09 | 7.88122828000298e-07 | 8.05522912827588 | FAAH//GABARAPL2//GOSR1//PJA2//ZDHHC17//SMC2//ATP5B//PLCB1//HNRNPK//ITGB1//KIT//CALR//CAT//LAMP1//PSAP//NBR1//MLC1//GLA//PPP3CA//GLUL//MOBP//SLC25A5//MAPK10//YWHAZ//ACADSB//VAMP1//MAPK9//GATM//PHYH//HIGD1A//MGEA5//HADHA//CLCC1//MGST1//UXS1//GHITM//NARS//HSPA9//HSP90AB1//TIMMDC1//DNAJA3//RGD1309676//AUH//SLC25A13//COA5//MAT2B//TRIAP1//MAL//ANP32A//CANX//PTP4A1//PDIA3//INSIG1//CNBP//TMED2//RTN4//SQSTM1//PDIA4//STIM2//KEAP1//FNDC5//INSIG2//SEC62//MGST2//FKBP9//MESDC2//KDELR3//ORMDL3//TMED9//ELOVL7//SUMF1//TRIM37//ACBD5//EGR1//HSPA8//FMR1//RGN//GADD45A//NCL//NFIA//ZEB1//CCT4//PAK2//HIF1A//HNRNPA1//ADAM10//CAMKK1//PDCD4//APAF1//LUZP1//PRKCI//DDX1//FADS1//POLA1//PIK3CB//ELF1//PICALM//DMTF1//HDGF//HNRNPM//HNRPH1//ANP32B//DDX46//ZFP91//SERBP1//MRFAP1//CNOT6//BLMH//HSPH1//MKX//IK//MRF//SESN1//TTC37//DNAJB4//CBX3//CCT2//NDRG1//PIAS1//ZFP445//RGD1562291//DNAJC7//TLK2//KLHL20//HNRPDL//SPTBN1//ZFP828//RGD1560691//ZBTB2//HNRNPH2//TNKS2//ILF2//VPRBP//ARIH2//MAGEE1//TAF1//WDR13//FUS//PARN//GLYR1//FLYWCH1//NEK7//HABP4//UPF2//PLEKHA1//HNRNPH3//DDX50//ANP32E//HNRNPA3//HDAC8//PTTG1IP//UAP1//TSC22D1//ARL2BP//RGD1564943//RGD1564051//RBPJ//HMG1L1//TCF7L2//HMGN5//SEC24C//SRSF1//CDC42SE2//PPP2R5C//ITPR1//SENP2//FOXN3//RPL5//WIPF3//HAUS7//KRT18//CCT5//CNP//SLC1A3//SPPL2A//GCGR//SNAP25//EXOC4//VPS16//SRPR//CES1D//HSD17B12//RASGRF2//SERINC1//EI24//SCAMP1//ARF1//ARF4//GALNT1//CLASP2//LAPTM4A//USP32//GOPC//OPALIN//NSF//KLHL12//NPTX1//LPAR1//CPSF3//MAGT1//GAR1//ATP6V1D//ANK3//MED20//ST6GALNAC3//ARCN1//KPNB1//ACTB//PDCD6IP//CDC40//AZI1//LSM14A |
| GO:0043227 | membrane-bounded organelle | Cellular component | 212 | 7508 | 329 | 15288 | 1.31209830081955 | 1.08325074971541e-08 | 8.31008075138822e-07 | 7.96527100171925 | FAAH//GABARAPL2//GOSR1//PJA2//ZDHHC17//SMC2//ATP5B//PLCB1//HNRNPK//ITGB1//KIT//CALR//CAT//LAMP1//PSAP//NBR1//MLC1//GLA//PPP3CA//GLUL//MOBP//SLC25A5//MAPK10//YWHAZ//ACADSB//VAMP1//MAPK9//GATM//PHYH//HIGD1A//MGEA5//HADHA//CLCC1//MGST1//UXS1//GHITM//NARS//HSPA9//HSP90AB1//TIMMDC1//DNAJA3//RGD1309676//AUH//SLC25A13//COA5//MAT2B//TRIAP1//MAL//ANP32A//CANX//PTP4A1//PDIA3//INSIG1//CNBP//TMED2//RTN4//SQSTM1//PDIA4//STIM2//KEAP1//FNDC5//INSIG2//SEC62//MGST2//FKBP9//MESDC2//KDELR3//ORMDL3//TMED9//ELOVL7//SUMF1//TRIM37//ACBD5//EGR1//HSPA8//FMR1//RGN//GADD45A//NCL//NFIA//ZEB1//CCT4//PAK2//HIF1A//HNRNPA1//ADAM10//CAMKK1//PDCD4//APAF1//LUZP1//PRKCI//DDX1//FADS1//POLA1//PIK3CB//ELF1//PICALM//DMTF1//HDGF//HNRNPM//HNRPH1//ANP32B//DDX46//ZFP91//SERBP1//MRFAP1//CNOT6//BLMH//HSPH1//MKX//IK//MRF//SESN1//TTC37//DNAJB4//CBX3//CCT2//NDRG1//PIAS1//ZFP445//RGD1562291//DNAJC7//TLK2//KLHL20//HNRPDL//SPTBN1//ZFP828//RGD1560691//ZBTB2//HNRNPH2//TNKS2//ILF2//VPRBP//ARIH2//MAGEE1//TAF1//WDR13//FUS//PARN//GLYR1//FLYWCH1//NEK7//HABP4//UPF2//PLEKHA1//HNRNPH3//DDX50//ANP32E//HNRNPA3//HDAC8//PTTG1IP//UAP1//TSC22D1//ARL2BP//RGD1564943//RGD1564051//RBPJ//HMG1L1//TCF7L2//HMGN5//SEC24C//SRSF1//CDC42SE2//PPP2R5C//ITPR1//SENP2//FOXN3//RPL5//WIPF3//HAUS7//KRT18//CCT5//CNP//SLC1A3//SPPL2A//GCGR//SNAP25//EXOC4//VPS16//SRPR//CES1D//HSD17B12//RASGRF2//SERINC1//EI24//AZI1//LSM14A//SCAMP1//ARF1//ARF4//GALNT1//CLASP2//LAPTM4A//USP32//GOPC//OPALIN//NSF//KLHL12//NPTX1//LPAR1//CPSF3//MAGT1//GAR1//ATP6V1D//ANK3//MED20//ST6GALNAC3//ARCN1//KPNB1//ACTB//PDCD6IP//CDC40 |
| GO:0043229 | intracellular organelle | Cellular component | 233 | 8629 | 329 | 15288 | 1.25472984468504 | 4.09795299438261e-08 | 2.75075094747933e-06 | 7.38743302748615 | FAAH//GABARAPL2//GOSR1//PJA2//ZDHHC17//RGD1564051//RPS4X//SMC2//ZFP828//TNKS2//ATP5B//PPP2R5C//CLASP2//POLA1//HMGN5//PLCB1//HNRNPK//NEK7//LSM14A//ITGB1//KIT//CALR//GFAP//KRT18//TLK2//CAT//LAMP1//PSAP//NBR1//MLC1//GLA//PPP3CA//GLUL//MOBP//SLC25A5//MAPK10//YWHAZ//ACADSB//VAMP1//MAPK9//GATM//PHYH//HIGD1A//MGEA5//HADHA//CLCC1//MGST1//UXS1//GHITM//NARS//HSPA9//HSP90AB1//TIMMDC1//DNAJA3//RGD1309676//AUH//SLC25A13//COA5//MAT2B//TRIAP1//MAL//ANP32A//CANX//PTP4A1//PDIA3//INSIG1//CNBP//TMED2//RTN4//SQSTM1//PDIA4//STIM2//KEAP1//FNDC5//INSIG2//SEC62//MGST2//FKBP9//MESDC2//KDELR3//ORMDL3//TMED9//ELOVL7//SUMF1//TRIM37//ACBD5//RPL5//EGR1//HSPA8//FMR1//RGN//GADD45A//NCL//NFIA//ZEB1//CCT4//PAK2//HIF1A//HNRNPA1//ADAM10//CAMKK1//PDCD4//APAF1//LUZP1//PRKCI//DDX1//FADS1//PIK3CB//ELF1//PICALM//DMTF1//HDGF//HNRNPM//HNRPH1//ANP32B//DDX46//ZFP91//SERBP1//MRFAP1//CNOT6//BLMH//HSPH1//MKX//IK//MRF//SESN1//TTC37//DNAJB4//CBX3//CCT2//NDRG1//PIAS1//ZFP445//RGD1562291//DNAJC7//KLHL20//HNRPDL//SPTBN1//RGD1560691//ZBTB2//HNRNPH2//ILF2//VPRBP//ARIH2//MAGEE1//TAF1//WDR13//FUS//PARN//GLYR1//FLYWCH1//HABP4//UPF2//PLEKHA1//HNRNPH3//DDX50//ANP32E//HNRNPA3//HDAC8//PTTG1IP//UAP1//TSC22D1//ARL2BP//RGD1564943//RBPJ//HMG1L1//TCF7L2//SEC24C//SRSF1//CDC42SE2//ITPR1//SENP2//FOXN3//WIPF3//HAUS7//CCT5//CNP//SLC1A3//SPPL2A//GCGR//SNAP25//EXOC4//VPS16//SRPR//CES1D//HSD17B12//RASGRF2//SERINC1//EI24//AZI1//SCAMP1//ARF1//ARF4//GALNT1//LAPTM4A//USP32//GOPC//OPALIN//NSF//KLHL12//NPTX1//LPAR1//PLK2//DDHD2//SLMAP//PDCD6IP//CPSF3//ERMN//ACTR10//CCT6A//MAP4//NRP1//SPTA1//MAGT1//MPDZ//ACTB//BCAS1//GAR1//CLCN2//ACTG1//GSN//DBC1//ATP6V1D//ANK3//MED20//ST6GALNAC3//ARCN1//CAP1//DSTN//KPNB1//PLS3//SDC4//CDC40 |
| GO:0043226 | organelle | Cellular component | 233 | 8654 | 329 | 15288 | 1.25110513401748 | 5.68122038242965e-08 | 3.38979482818302e-06 | 7.2455583635098 | FAAH//GABARAPL2//GOSR1//PJA2//ZDHHC17//RGD1564051//RPS4X//SMC2//ZFP828//TNKS2//ATP5B//PPP2R5C//CLASP2//POLA1//HMGN5//PLCB1//HNRNPK//NEK7//LSM14A//ITGB1//KIT//CALR//GFAP//KRT18//TLK2//CAT//LAMP1//PSAP//NBR1//MLC1//GLA//PPP3CA//GLUL//MOBP//SLC25A5//MAPK10//YWHAZ//ACADSB//VAMP1//MAPK9//GATM//PHYH//HIGD1A//MGEA5//HADHA//CLCC1//MGST1//UXS1//GHITM//NARS//HSPA9//HSP90AB1//TIMMDC1//DNAJA3//RGD1309676//AUH//SLC25A13//COA5//MAT2B//TRIAP1//MAL//ANP32A//CANX//PTP4A1//PDIA3//INSIG1//CNBP//TMED2//RTN4//SQSTM1//PDIA4//STIM2//KEAP1//FNDC5//INSIG2//SEC62//MGST2//FKBP9//MESDC2//KDELR3//ORMDL3//TMED9//ELOVL7//SUMF1//TRIM37//ACBD5//RPL5//EGR1//HSPA8//FMR1//RGN//GADD45A//NCL//NFIA//ZEB1//CCT4//PAK2//HIF1A//HNRNPA1//ADAM10//CAMKK1//PDCD4//APAF1//LUZP1//PRKCI//DDX1//FADS1//PIK3CB//ELF1//PICALM//DMTF1//HDGF//HNRNPM//HNRPH1//ANP32B//DDX46//ZFP91//SERBP1//MRFAP1//CNOT6//BLMH//HSPH1//MKX//IK//MRF//SESN1//TTC37//DNAJB4//CBX3//CCT2//NDRG1//PIAS1//ZFP445//RGD1562291//DNAJC7//KLHL20//HNRPDL//SPTBN1//RGD1560691//ZBTB2//HNRNPH2//ILF2//VPRBP//ARIH2//MAGEE1//TAF1//WDR13//FUS//PARN//GLYR1//FLYWCH1//HABP4//UPF2//PLEKHA1//HNRNPH3//DDX50//ANP32E//HNRNPA3//HDAC8//PTTG1IP//UAP1//TSC22D1//ARL2BP//RGD1564943//RBPJ//HMG1L1//TCF7L2//SEC24C//SRSF1//CDC42SE2//ITPR1//SENP2//FOXN3//WIPF3//HAUS7//CCT5//CNP//SLC1A3//SPPL2A//GCGR//SNAP25//EXOC4//VPS16//SRPR//CES1D//HSD17B12//RASGRF2//SERINC1//EI24//AZI1//SCAMP1//ARF1//ARF4//GALNT1//LAPTM4A//USP32//GOPC//OPALIN//NSF//KLHL12//NPTX1//LPAR1//PLK2//DDHD2//SLMAP//PDCD6IP//CPSF3//ERMN//ACTR10//CCT6A//MAP4//NRP1//SPTA1//MAGT1//MPDZ//ACTB//BCAS1//GAR1//CLCN2//ACTG1//GSN//DBC1//ATP6V1D//ANK3//MED20//ST6GALNAC3//ARCN1//CAP1//DSTN//KPNB1//PLS3//SDC4//CDC40 |
| GO:0044444 | cytoplasmic part | Cellular component | 163 | 5449 | 329 | 15288 | 1.39003447831537 | 1.22834547553978e-07 | 6.59621520364862e-06 | 6.91067946949838 | FAAH//GABARAPL2//GOSR1//PJA2//ZDHHC17//EXOC4//RGD1564051//RPS4X//TNKS2//ATP5B//SQSTM1//NBR1//LSM14A//ITGB1//KIT//CALR//CAT//LAMP1//PSAP//MLC1//GLA//PPP3CA//GLUL//MOBP//SLC25A5//MAPK10//YWHAZ//ACADSB//VAMP1//MAPK9//GATM//PHYH//HIGD1A//MGEA5//HADHA//CLCC1//MGST1//UXS1//GHITM//NARS//HSPA9//HSP90AB1//TIMMDC1//DNAJA3//RGD1309676//AUH//SLC25A13//COA5//MAT2B//TRIAP1//HSPA8//PLCB1//PPP3CB//PDCD4//ARF1//CNBP//APAF1//ACTB//PRKCI//PIK3CB//CES1D//NRP1//RAPGEF4//ACTG1//GSN//NDRG1//GPCPD1//ELMO2//UAP1//PDCD6IP//NCALD//TCF7L2//MAL//ANP32A//CANX//PTP4A1//PDIA3//INSIG1//TMED2//RTN4//PDIA4//STIM2//KEAP1//FNDC5//INSIG2//SEC62//MGST2//FKBP9//MESDC2//KDELR3//ORMDL3//TMED9//ELOVL7//SUMF1//TRIM37//ACBD5//RPL5//CNP//SLC1A3//ARL2BP//SPPL2A//GCGR//SNAP25//PICALM//VPS16//SRPR//ITPR1//HSD17B12//FADS1//RASGRF2//SERINC1//EI24//SCAMP1//ADAM10//ARF4//GALNT1//CLASP2//LAPTM4A//USP32//KLHL20//GOPC//OPALIN//NSF//KLHL12//NPTX1//LPAR1//MGLL//CCT4//PLK2//HAUS7//CCT5//AZI1//DDHD2//SLMAP//KRT18//NEK7//CCT6A//CCT2//EIF3D//NCL//SPTA1//MAGT1//FMR1//DDX1//SENP2//ANP32E//ATP6V1D//ANK3//HABP4//SEC24C//ARCN1//ST6GALNAC3//SPTBN1//CAP1//DSTN//MPDZ//SDC4//PAK2//SERBP1//MRFAP1//TLK2//MAGEE1//TTC37 |
| GO:0042470 | melanosome | Cellular component | 11 | 78 | 329 | 15288 | 6.5531914893617 | 8.34406979409434e-07 | 3.73397123285722e-05 | 6.07862207190376 | HSPA8//ITGB1//CNP//LAMP1//YWHAZ//CANX//CCT4//PDIA3//PDIA4//HSP90AB1//PDCD6IP |
| GO:0048770 | pigment granule | Cellular component | 11 | 78 | 329 | 15288 | 6.5531914893617 | 8.34406979409434e-07 | 3.73397123285722e-05 | 6.07862207190376 | HSPA8//ITGB1//CNP//LAMP1//YWHAZ//CANX//CCT4//PDIA3//PDIA4//HSP90AB1//PDCD6IP |
| GO:0044422 | organelle part | Cellular component | 141 | 4657 | 329 | 15288 | 1.40691432252523 | 1.05366515405603e-06 | 4.35244759790837e-05 | 5.97729738233999 | FAAH//GABARAPL2//GOSR1//PJA2//ZDHHC17//RGD1564051//RPS4X//SMC2//TNKS2//ATP5B//PPP2R5C//CLASP2//ZFP828//POLA1//HMGN5//PLCB1//HNRNPK//NEK7//GFAP//KRT18//TLK2//CAT//MGST1//FNDC5//ACBD5//ITPR1//RTN4//SENP2//NCL//HNRNPA1//HNRNPM//HNRPH1//HNRNPH2//TCF7L2//SRSF1//ZEB1//HIF1A//FOXN3//RBPJ//TAF1//HNRNPA3//HSPA8//FMR1//MOBP//RPL5//HADHA//DDX46//WIPF3//HSPA9//HAUS7//CCT5//SPTBN1//ILF2//DDX50//CNP//VAMP1//SLC25A5//SLC1A3//GATM//HIGD1A//GHITM//SLC25A13//ARL2BP//ACADSB//DNAJA3//LAMP1//SPPL2A//SRPR//PDIA3//CALR//CES1D//PDIA4//FKBP9//CANX//INSIG1//TMED2//HSD17B12//FADS1//RASGRF2//SERINC1//EI24//TMED9//ELOVL7//NSF//ADAM10//CCT4//PLK2//KEAP1//NDRG1//AZI1//DDHD2//SLMAP//PDCD6IP//PTP4A1//CPSF3//ACTR10//HSPH1//CCT6A//CCT2//MAP4//NRP1//VPS16//PICALM//SPTA1//HSP90AB1//VPRBP//MAGT1//YWHAZ//MPDZ//ACTB//EXOC4//BCAS1//GAR1//ANP32A//ATP6V1D//MED20//KLHL20//ARF1//HABP4//PPP3CA//ANK3//SEC24C//GOPC//ST6GALNAC3//ARCN1//SCAMP1//CAP1//DSTN//NBR1//ACTG1//KPNB1//PLS3//GALNT1//UXS1//INSIG2//UPF2//TTC37//ORMDL3//SDC4//CDC40//DDX1 |
| GO:0043209 | myelin sheath | Cellular component | 8 | 41 | 329 | 15288 | 9.0669434353918 | 2.1736866924348e-06 | 8.3376410988392e-05 | 5.6628030534201 | ITGB1//CNP//EXOC4//MPDZ//PRKCI//PLP1//RTN4//ERMN |
| GO:0005783 | endoplasmic reticulum | Cellular component | 43 | 974 | 329 | 15288 | 2.05146576958364 | 5.56114440097797e-06 | 0.000199088969555011 | 5.25483582786255 | SRPR//PDIA3//CALR//CES1D//PDIA4//FKBP9//ITPR1//CANX//FAAH//INSIG1//TMED2//HSD17B12//FADS1//RASGRF2//MGST1//PJA2//SERINC1//EI24//TMED9//ELOVL7//GLUL//MAGT1//ANK3//RTN4//INSIG2//ORMDL3//SPPL2A//CAT//MAL//ANP32A//PTP4A1//CNBP//SQSTM1//STIM2//KEAP1//CLCC1//FNDC5//SEC62//MGST2//MESDC2//KDELR3//MLC1//SUMF1 |
| GO:0045202 | synapse | Cellular component | 28 | 527 | 329 | 15288 | 2.46889256732206 | 1.0571757764654e-05 | 0.0003548146199762 | 4.97585279660724 | HSPA8//PICALM//ITPR1//YWHAZ//MPDZ//ADAM10//NSF//ACTB//EXOC4//PJA2//BCAS1//VAMP1//SCAMP1//ITGB1//DNAJA3//ANK3//NPTN//MAGEE1//LRRC4//RAPGEF4//NT5E//FMR1//SNAP25//MGLL//SLC1A3//GOPC//NRCAM//PRIMA1 |
| GO:0044446 | intracellular organelle part | Cellular component | 134 | 4569 | 329 | 15288 | 1.36281974266914 | 1.50304622614876e-05 | 0.000474785778495226 | 4.82302766249186 | FAAH//GABARAPL2//GOSR1//PJA2//ZDHHC17//RGD1564051//RPS4X//SMC2//TNKS2//ATP5B//PPP2R5C//CLASP2//ZFP828//POLA1//HMGN5//PLCB1//HNRNPK//NEK7//GFAP//KRT18//TLK2//CAT//MGST1//FNDC5//ACBD5//ITPR1//RTN4//SENP2//NCL//HNRNPA1//HNRNPM//HNRPH1//HNRNPH2//TCF7L2//SRSF1//ZEB1//HIF1A//FOXN3//RBPJ//TAF1//HNRNPA3//HSPA8//FMR1//MOBP//RPL5//HADHA//DDX46//WIPF3//HSPA9//HAUS7//CCT5//SPTBN1//ILF2//DDX50//CNP//VAMP1//SLC25A5//SLC1A3//GATM//HIGD1A//GHITM//SLC25A13//ARL2BP//ACADSB//DNAJA3//LAMP1//SPPL2A//SRPR//PDIA3//CALR//CES1D//PDIA4//FKBP9//CANX//INSIG1//TMED2//HSD17B12//FADS1//RASGRF2//SERINC1//EI24//TMED9//ELOVL7//NSF//ADAM10//CCT4//PLK2//KEAP1//NDRG1//AZI1//DDHD2//SLMAP//PDCD6IP//PTP4A1//CPSF3//ACTR10//HSPH1//CCT6A//CCT2//MAP4//NRP1//VPS16//PICALM//SPTA1//HSP90AB1//VPRBP//MAGT1//YWHAZ//MPDZ//ACTB//EXOC4//BCAS1//GAR1//ANP32A//ATP6V1D//MED20//KLHL20//SEC24C//GOPC//ST6GALNAC3//ARCN1//SCAMP1//CAP1//DSTN//ACTG1//KPNB1//GALNT1//UXS1//INSIG2//UPF2//TTC37//ORMDL3//CDC40//DDX1 |
| GO:0043197 | dendritic spine | Cellular component | 15 | 192 | 329 | 15288 | 3.63031914893617 | 1.78579528392732e-05 | 0.000504722140773143 | 4.74816832828985 | ITPR1//YWHAZ//MPDZ//ADAM10//NSF//ACTB//PICALM//EXOC4//PJA2//BCAS1//FMR1//CANX//SLC1A3//LPAR1//RAPGEF4 |
| GO:0044309 | neuron spine | Cellular component | 15 | 192 | 329 | 15288 | 3.63031914893617 | 1.78579528392732e-05 | 0.000504722140773143 | 4.74816832828985 | ITPR1//YWHAZ//MPDZ//ADAM10//NSF//ACTB//PICALM//EXOC4//PJA2//BCAS1//FMR1//CANX//SLC1A3//LPAR1//RAPGEF4 |
| GO:0005634 | nucleus | Cellular component | 130 | 4445 | 329 | 15288 | 1.35902161166024 | 2.61719692435678e-05 | 0.000702505047915868 | 4.58216359874188 | SMC2//PLCB1//HNRNPK//ITPR1//RTN4//POLA1//TNKS2//SENP2//NCL//HNRNPA1//HNRNPM//HNRPH1//HNRNPH2//TCF7L2//HMGN5//SRSF1//ZEB1//HIF1A//FOXN3//RBPJ//TAF1//HNRNPA3//HSPA8//FMR1//MOBP//RPL5//HADHA//DDX46//WIPF3//HSPA9//HAUS7//KRT18//CCT5//SPTBN1//ILF2//DDX50//CPSF3//HSP90AB1//VPRBP//GAR1//ANP32A//MED20//KLHL20//KPNB1//EI24//UPF2//ACTB//CDC40//DDX1//EGR1//PPP3CA//RGN//GADD45A//MAPK10//NFIA//YWHAZ//CCT4//PAK2//PTP4A1//ADAM10//MAPK9//CAMKK1//KIT//PDCD4//CALR//CNBP//APAF1//LUZP1//PRKCI//FADS1//PIK3CB//ELF1//PICALM//SQSTM1//DMTF1//HDGF//KEAP1//MGEA5//ANP32B//CLCC1//MGST1//ZFP91//SERBP1//MRFAP1//CNOT6//BLMH//HSPH1//MKX//IK//MRF//SESN1//TTC37//DNAJB4//CBX3//CCT2//NDRG1//PIAS1//ZFP445//RGD1562291//DNAJC7//TLK2//HNRPDL//ZFP828//RGD1560691//ZBTB2//ARIH2//MAGEE1//WDR13//FUS//PARN//GLYR1//DNAJA3//FLYWCH1//NEK7//HABP4//PLEKHA1//HNRNPH3//ANP32E//HDAC8//PTTG1IP//UAP1//TSC22D1//ARL2BP//RGD1564943//RGD1564051//HMG1L1//MAT2B//SEC24C//CDC42SE2//PPP2R5C |
| GO:0044430 | cytoskeletal part | Cellular component | 44 | 1074 | 329 | 15288 | 1.90372043266373 | 2.74722644436373e-05 | 0.000702505047915868 | 4.5611055417136 | TNKS2//NEK7//GFAP//KRT18//TLK2//CCT4//PLK2//KEAP1//HAUS7//CCT5//NDRG1//AZI1//ARL2BP//DDHD2//CLASP2//SLMAP//PDCD6IP//PTP4A1//ZFP828//ACTR10//CNP//HSPH1//CCT6A//CCT2//MAP4//NRP1//VPS16//DNAJA3//SPTA1//ITPR1//YWHAZ//MPDZ//ADAM10//NSF//ACTB//PICALM//EXOC4//PJA2//BCAS1//CAP1//DSTN//ACTG1//SPTBN1//SLC25A5 |
| GO:0043005 | neuron projection | Cellular component | 33 | 742 | 329 | 15288 | 2.06663990365315 | 6.43390999501573e-05 | 0.00157045893969248 | 4.19152501880573 | NRP1//ITPR1//YWHAZ//MPDZ//ADAM10//NSF//ACTB//PICALM//EXOC4//PJA2//BCAS1//SNAP25//CANX//ROBO1//RAPGEF4//VPS16//KLHL24//PTPRK//ANK3//NRCAM//FMR1//LAMP1//CLCN2//NELL2//PLK2//MAGEE1//ERMN//SLC1A3//LPAR1//GLUL//HSPA8//RTN4//HNRNPA3 |
| GO:0031410 | cytoplasmic vesicle | Cellular component | 33 | 749 | 329 | 15288 | 2.04732551203022 | 7.70382265281751e-05 | 0.00179867511502739 | 4.11329372351972 | ITGB1//KIT//CALR//ADAM10//TMED2//KLHL12//NPTX1//ITPR1//LPAR1//PICALM//HSPA8//SENP2//ANP32E//SEC24C//MLC1//GOPC//SPPL2A//ZDHHC17//ARCN1//VAMP1//SCAMP1//CNP//LAMP1//YWHAZ//CANX//CCT4//PDIA3//PDIA4//HSP90AB1//PDCD6IP//MPDZ//GABARAPL2//NBR1 |
| GO:0005856 | cytoskeleton | Cellular component | 52 | 1416 | 329 | 15288 | 1.70645510277678 | 8.99257773023073e-05 | 0.00201208926713913 | 4.04611579952954 | TNKS2//NEK7//GFAP//KRT18//TLK2//CCT4//PLK2//KEAP1//HAUS7//CCT5//NDRG1//AZI1//ARL2BP//DDHD2//CLASP2//SLMAP//PDCD6IP//PTP4A1//ZFP828//ACTR10//CNP//HSPH1//CCT6A//CCT2//MAP4//NRP1//VPS16//DNAJA3//SPTA1//ITPR1//YWHAZ//MPDZ//ADAM10//NSF//ACTB//PICALM//EXOC4//PJA2//BCAS1//SNAP25//CLCN2//ACTG1//GSN//HNRNPH2//DBC1//SPTBN1//CAP1//DSTN//SEC62//SLC25A5//ERMN//CDC42SE2 |
| GO:0030425 | dendrite | Cellular component | 22 | 426 | 329 | 15288 | 2.39976026370992 | 0.000143210662253242 | 0.00297618610095906 | 3.84402464693482 | ITPR1//YWHAZ//MPDZ//ADAM10//NSF//ACTB//PICALM//EXOC4//PJA2//BCAS1//CANX//FMR1//SLC1A3//LPAR1//RAPGEF4//LAMP1//CLCN2//NELL2//PLK2//MAGEE1//PTPRK//ANK3 |
| GO:0043228 | non-membrane-bounded organelle | Cellular component | 88 | 2842 | 329 | 15288 | 1.4388428885861 | 0.000149640641947662 | 0.00297618610095906 | 3.82495043737754 | RGD1564051//RPS4X//SMC2//ZFP828//TNKS2//PPP2R5C//CLASP2//POLA1//HMGN5//PLCB1//HNRNPK//NEK7//LSM14A//GFAP//KRT18//TLK2//CANX//RPL5//HSPA8//FMR1//MOBP//NCL//ITPR1//HIF1A//HNRNPA1//HADHA//DDX46//WIPF3//HSPA9//HAUS7//CCT5//SPTBN1//ILF2//DDX50//HNRNPA3//RBPJ//CCT4//PLK2//KEAP1//NDRG1//AZI1//ARL2BP//DDHD2//SLMAP//PDCD6IP//PTP4A1//ERMN//CDC42SE2//ACTR10//CNP//HSPH1//CCT6A//CCT2//MAP4//NRP1//VPS16//DNAJA3//SPTA1//DDX1//YWHAZ//MPDZ//ADAM10//NSF//ACTB//PICALM//EXOC4//PJA2//BCAS1//SNAP25//CLCN2//ACTG1//GSN//HNRNPH2//DBC1//ARF1//HABP4//PPP3CA//ANK3//CAP1//DSTN//GAR1//NBR1//PLS3//TTC37//SLC25A5//ATP5B//SDC4//SEC62 |
| GO:0043232 | intracellular non-membrane-bounded organelle | Cellular component | 88 | 2842 | 329 | 15288 | 1.4388428885861 | 0.000149640641947662 | 0.00297618610095906 | 3.82495043737754 | RGD1564051//RPS4X//SMC2//ZFP828//TNKS2//PPP2R5C//CLASP2//POLA1//HMGN5//PLCB1//HNRNPK//NEK7//LSM14A//GFAP//KRT18//TLK2//CANX//RPL5//HSPA8//FMR1//MOBP//NCL//ITPR1//HIF1A//HNRNPA1//HADHA//DDX46//WIPF3//HSPA9//HAUS7//CCT5//SPTBN1//ILF2//DDX50//HNRNPA3//RBPJ//CCT4//PLK2//KEAP1//NDRG1//AZI1//ARL2BP//DDHD2//SLMAP//PDCD6IP//PTP4A1//ERMN//CDC42SE2//ACTR10//CNP//HSPH1//CCT6A//CCT2//MAP4//NRP1//VPS16//DNAJA3//SPTA1//DDX1//YWHAZ//MPDZ//ADAM10//NSF//ACTB//PICALM//EXOC4//PJA2//BCAS1//SNAP25//CLCN2//ACTG1//GSN//HNRNPH2//DBC1//ARF1//HABP4//PPP3CA//ANK3//CAP1//DSTN//GAR1//NBR1//PLS3//TTC37//SLC25A5//ATP5B//SDC4//SEC62 |
| GO:0016023 | cytoplasmic membrane-bounded vesicle | Cellular component | 30 | 680 | 329 | 15288 | 2.05006257822278 | 0.000161021646804023 | 0.00308816515477716 | 3.79311573604519 | ITGB1//KIT//CALR//ADAM10//TMED2//KLHL12//NPTX1//ITPR1//LPAR1//PICALM//HSPA8//SEC24C//MLC1//GOPC//SPPL2A//ZDHHC17//ARCN1//VAMP1//SCAMP1//CNP//LAMP1//YWHAZ//CANX//CCT4//PDIA3//PDIA4//HSP90AB1//PDCD6IP//SENP2//ANP32E |
| GO:0042995 | cell projection | Cellular component | 45 | 1211 | 329 | 15288 | 1.72672488008855 | 0.000217812137672552 | 0.00403327992862622 | 3.6619179226641 | GSN//RAPGEF4//PTPRK//NRP1//CNP//HIF1A//ITPR1//YWHAZ//MPDZ//ADAM10//NSF//ACTB//PICALM//EXOC4//PJA2//BCAS1//HSPA8//SNAP25//SLC1A3//RTN4//HNRNPA3//ERMN//CANX//ROBO1//VPS16//KLHL24//ANK3//NRCAM//FMR1//LAMP1//CLCN2//NELL2//PLK2//MAGEE1//PIK3CB//HSP90AB1//PLS3//ITGB1//CLASP2//PLEKHA1//LPAR1//GLUL//GFAP//MAGI1//CDC42SE2 |
| GO:0031982 | vesicle | Cellular component | 33 | 807 | 329 | 15288 | 1.90018191884837 | 0.000306583798471606 | 0.00548784999264175 | 3.51345079931952 | ITGB1//KIT//CALR//ADAM10//TMED2//KLHL12//NPTX1//ITPR1//LPAR1//PICALM//HSPA8//SENP2//ANP32E//SEC24C//MLC1//GOPC//SPPL2A//ZDHHC17//ARCN1//VAMP1//SCAMP1//LAMP1//MPDZ//GABARAPL2//NBR1//CNP//YWHAZ//CANX//CCT4//PDIA3//PDIA4//HSP90AB1//PDCD6IP |
| GO:0031988 | membrane-bounded vesicle | Cellular component | 30 | 715 | 329 | 15288 | 1.94970986460348 | 0.000378402800627611 | 0.00655491303022668 | 3.42204565796629 | ITGB1//KIT//CALR//ADAM10//TMED2//KLHL12//NPTX1//ITPR1//LPAR1//PICALM//HSPA8//SENP2//ANP32E//SEC24C//MLC1//GOPC//SPPL2A//ZDHHC17//ARCN1//VAMP1//SCAMP1//CNP//LAMP1//YWHAZ//CANX//CCT4//PDIA3//PDIA4//HSP90AB1//PDCD6IP |
| GO:0005730 | nucleolus | Cellular component | 23 | 492 | 329 | 15288 | 2.17228853139595 | 0.000426229593162576 | 0.00715266536025948 | 3.37035640046641 | GAR1//HSPA8//FMR1//MOBP//NCL//ITPR1//HIF1A//HNRNPA1//RPL5//POLA1//HADHA//DDX46//WIPF3//HSPA9//HAUS7//KRT18//CCT5//SPTBN1//ILF2//DDX50//HNRNPA3//RBPJ//HMGN5 |
| GO:0043234 | protein complex | Cellular component | 85 | 2819 | 329 | 15288 | 1.40113062576891 | 0.000492473137156188 | 0.00801388105008706 | 3.30761745390345 | EXOC4//PPP2R5C//ATP5B//DNAJB2//CLASP2//ZFP828//SMC2//HSPA8//SCN4B//GFAP//KRT18//TLK2//RBPJ//FCGRT//ITGB1//SENP2//POLA1//ZEB1//HIF1A//FOXN3//TCF7L2//TAF1//SRPR//CCT4//CCT6A//CCT5//CCT2//FMR1//CPSF3//EIF3D//ACTR10//CNP//HSPH1//NDRG1//MAP4//NRP1//VPS16//DNAJA3//CACNA2D3//PICALM//PIK3CB//RAPGEF4//PPP3CA//PPP3CB//ITPR1//SNAP25//HSP90AB1//VPRBP//MAGT1//PPM1B//MAGEE1//ATP6V1D//HADHA//MED20//RPL5//SEC24C//ARCN1//CUL4A//KLHL12//KLHL20//ACTG1//INSIG2//CLCN2//UPF2//ACTB//ORMDL3//CALR//APAF1//CD14//MAT2B//TTC37//NAPA//HAUS7//SLC25A5//DDX1//CLCF1//KPNB1//GLUL//YWHAZ//RTN4//PRKCI//HIGD1A//GSN//SPTBN1//SSX2IP |
| GO:0044445 | cytosolic part | Cellular component | 12 | 183 | 329 | 15288 | 3.04708754795954 | 0.000616450200489238 | 0.00927075042588815 | 3.21010200183595 | RGD1564051//RPS4X//CCT4//CCT6A//CCT5//CCT2//PIK3CB//DNAJA3//APAF1//TCF7L2//MAPK9//TRIM37 |
| GO:0044432 | endoplasmic reticulum part | Cellular component | 25 | 570 | 329 | 15288 | 2.03807390817469 | 0.00061892310917264 | 0.00927075042588815 | 3.20836330144228 | SRPR//PDIA3//CALR//CES1D//PDIA4//FKBP9//ITPR1//CANX//FAAH//INSIG1//TMED2//HSD17B12//FADS1//RASGRF2//MGST1//PJA2//SERINC1//EI24//TMED9//ELOVL7//MAGT1//RTN4//INSIG2//ORMDL3//SPPL2A |
| GO:0044463 | cell projection part | Cellular component | 27 | 636 | 329 | 15288 | 1.97270172621437 | 0.00062150282184725 | 0.00927075042588815 | 3.20655689516408 | NRP1//ITPR1//YWHAZ//MPDZ//ADAM10//NSF//ACTB//PICALM//EXOC4//PJA2//BCAS1//SNAP25//ROBO1//PIK3CB//HSP90AB1//ITGB1//CLASP2//PLEKHA1//CANX//ANK3//ERMN//NRCAM//FMR1//SLC1A3//LPAR1//RAPGEF4//GLUL |
| GO:0042645 | mitochondrial nucleoid | Cellular component | 5 | 34 | 329 | 15288 | 6.83354192740926 | 0.000745952099470652 | 0.0108263858761011 | 3.12728905939527 | SLC25A5//HADHA//ATP5B//HSPA9//DNAJA3 |
| GO:0014069 | postsynaptic density | Cellular component | 10 | 138 | 329 | 15288 | 3.3672525439408 | 0.00080709892443757 | 0.0111131313441788 | 3.09307323144354 | ITPR1//YWHAZ//MPDZ//ADAM10//NSF//ACTB//PICALM//EXOC4//PJA2//BCAS1 |
| GO:0044327 | dendritic spine head | Cellular component | 10 | 138 | 329 | 15288 | 3.3672525439408 | 0.00080709892443757 | 0.0111131313441788 | 3.09307323144354 | ITPR1//YWHAZ//MPDZ//ADAM10//NSF//ACTB//PICALM//EXOC4//PJA2//BCAS1 |
| GO:0044456 | synapse part | Cellular component | 19 | 391 | 329 | 15288 | 2.25803994123089 | 0.000845057781313951 | 0.0113449007141398 | 3.07311359489898 | HSPA8//PICALM//ITPR1//YWHAZ//MPDZ//ADAM10//NSF//ACTB//EXOC4//PJA2//BCAS1//VAMP1//SCAMP1//NPTN//MAGEE1//DNAJA3//ANK3//LRRC4//NT5E |
| GO:0071944 | cell periphery | Cellular component | 90 | 3081 | 329 | 15288 | 1.35739294371128 | 0.000913205058103105 | 0.0119607589317407 | 3.03943169168445 | CAT//GSTM2//ITGB1//PPP3CB//GCGR//ITPR1//MAPK10//CNP//DDR1//SLC1A3//NPTN//NT5E//ROBO1//CD14//NSF//S1PR5//CAP1//ARF4//LRP4//RTN4//PRKCI//PICALM//MADD//RASGRF2//CLASP2//LPAR1//MAGT1//STIM2//ATP5B//PJA2//SERBP1//NRP1//RAPGEF4//FNDC5//GPR158//HAUS7//SERINC1//MGST2//DNAJB4//NDRG1//SPTBN1//GPC5//HIATL1//MESDC2//GOPC//MLC1//SCN4B//MAGEE1//CDH19//PLEKHA1//ANK3//DDX50//RGD1304592//PTTG1IP//MAP4//NRCAM//UAP1//LRRC4//CDC42SE2//EXOC4//FCGRT//PLP1//LRP12//CACNA2D3//MPDZ//CLDN11//CLDN12//MAGI1//SDC4//KEAP1//NCL//SNAP25//SPTA1//CD9//LAMP1//CR1L//KIT//CALR//SEMA7A//PTP4A1//PTPN3//HSP90AB1//PRICKLE2//DNAJA3//ACTB//DSTN//PRIMA1//PTPRK//PIK3CB//SLMAP |
| GO:0009295 | nucleoid | Cellular component | 5 | 36 | 329 | 15288 | 6.45390070921986 | 0.000975754843329632 | 0.0124757226397146 | 3.01065928434483 | SLC25A5//HADHA//ATP5B//HSPA9//DNAJA3 |
| GO:0005798 | Golgi-associated vesicle | Cellular component | 6 | 55 | 329 | 15288 | 5.06924564796905 | 0.00113257845262766 | 0.0141440611409547 | 2.94593170510921 | TMED2//GOPC//SPPL2A//ZDHHC17//ARCN1//ADAM10 |
| GO:0032991 | macromolecular complex | Cellular component | 102 | 3615 | 329 | 15288 | 1.31113269179836 | 0.00123441123855707 | 0.0150654280705715 | 2.90854013294207 | EXOC4//PPP2R5C//RGD1564051//RPS4X//ATP5B//DNAJB2//CLASP2//ZFP828//SMC2//LSM14A//HSPA8//SCN4B//GFAP//KRT18//TLK2//RBPJ//NCL//EEF2//HNRNPA1//RPL5//ACTB//DDX1//HNRNPH2//ILF2//HNRNPH3//FCGRT//CANX//ITGB1//SENP2//POLA1//ZEB1//HIF1A//FOXN3//TCF7L2//TAF1//HNRNPM//HNRNPK//HNRPH1//HNRNPA3//SRPR//CCT4//CCT6A//CCT5//CCT2//FMR1//CALR//CPSF3//EIF3D//ACTR10//CNP//HSPH1//NDRG1//MAP4//NRP1//VPS16//DNAJA3//CACNA2D3//PICALM//PIK3CB//RAPGEF4//PPP3CA//PPP3CB//ITPR1//SNAP25//HSP90AB1//VPRBP//MAGT1//PPM1B//MAGEE1//ATP6V1D//HADHA//MED20//SEC24C//ARCN1//GAR1//CUL4A//KLHL12//KLHL20//ACTG1//INSIG2//CLCN2//UPF2//ORMDL3//KPNB1//GLUL//YWHAZ//RTN4//PRKCI//HIGD1A//GSN//SPTBN1//SSX2IP//APAF1//CD14//MAT2B//TTC37//NAPA//HAUS7//CDC40//SRSF1//SLC25A5//CLCF1 |
| GO:0005794 | Golgi apparatus | Cellular component | 32 | 852 | 329 | 15288 | 1.74528019178903 | 0.00156340372483633 | 0.018583898623454 | 2.80592885766358 | FAAH//GABARAPL2//GOSR1//PJA2//ZDHHC17//NSF//ADAM10//TMED2//GOPC//ST6GALNAC3//SPPL2A//ARCN1//GALNT1//UXS1//CAT//GLUL//PSAP//SCAMP1//CALR//ARF1//APAF1//ARF4//PICALM//CLASP2//EXOC4//CLCC1//LAPTM4A//USP32//KLHL20//TMED9//OPALIN//GLA |
| GO:0005844 | polysome | Cellular component | 4 | 24 | 329 | 15288 | 7.74468085106383 | 0.00159191682807986 | 0.018583898623454 | 2.79807962632797 | FMR1//RPS4X//EEF2//CALR |
| GO:0009986 | cell surface | Cellular component | 22 | 511 | 329 | 15288 | 2.00058292043136 | 0.00164502200006673 | 0.0187952513624646 | 2.78382828954155 | ITGB1//CD9//LAMP1//CR1L//KIT//CALR//SEMA7A//HSPA8//SDC4//SLC1A3//ADAM10//NT5E//ROBO1//CD14//LPAR1//ATP5B//NRP1//HSPA9//HSP90AB1//KLHL20//PTPRK//RGD1308874 |
| GO:0008287 | protein serine/threonine phosphatase complex | Cellular component | 5 | 41 | 329 | 15288 | 5.66683964711988 | 0.00177683849202455 | 0.0198783806295247 | 2.75035204614532 | PPP2R5C//PPP3CA//PPP3CB//ITPR1//PPM1B |
| GO:0005886 | plasma membrane | Cellular component | 86 | 2986 | 329 | 15288 | 1.33833064941358 | 0.00190658372184297 | 0.020894601196524 | 2.71974411924628 | SCN4B//CDC42SE2//FCGRT//PLP1//LRP12//ITGB1//CACNA2D3//MLC1//PICALM//MPDZ//PRKCI//CLDN11//ANK3//CLDN12//MAGI1//SDC4//KEAP1//SNAP25//CD9//LAMP1//CR1L//KIT//CALR//SEMA7A//PTP4A1//PTPN3//MAGEE1//DDR1//SLC1A3//RAPGEF4//HSP90AB1//PRICKLE2//DNAJA3//ROBO1//PRIMA1//PTPRK//PIK3CB//EXOC4//CLASP2//PLEKHA1//SLMAP//CAT//GSTM2//PPP3CB//GCGR//ITPR1//MAPK10//CNP//NPTN//NT5E//CD14//NSF//S1PR5//CAP1//ARF4//LRP4//RTN4//MADD//RASGRF2//LPAR1//MAGT1//STIM2//ATP5B//PJA2//SERBP1//NRP1//FNDC5//GPR158//HAUS7//SERINC1//MGST2//DNAJB4//NDRG1//SPTBN1//GPC5//HIATL1//MESDC2//GOPC//CDH19//DDX50//RGD1304592//PTTG1IP//MAP4//NRCAM//UAP1//LRRC4 |
| GO:0012505 | endomembrane system | Cellular component | 40 | 1158 | 329 | 15288 | 1.60511520229302 | 0.00199141713994521 | 0.0213878200830116 | 2.70083775925878 | FAAH//GABARAPL2//GOSR1//PJA2//ZDHHC17//ITPR1//RTN4//POLA1//TNKS2//SENP2//SRPR//CANX//INSIG1//TMED2//HSD17B12//FADS1//RASGRF2//MGST1//SERINC1//EI24//TMED9//ELOVL7//PICALM//MAGT1//SEC24C//ST6GALNAC3//SPPL2A//ARCN1//VAMP1//SCAMP1//KPNB1//GALNT1//UXS1//INSIG2//ORMDL3//CALR//MPDZ//ARF1//DOCK9//LAPTM4A |
| GO:0048471 | perinuclear region of cytoplasm | Cellular component | 19 | 426 | 329 | 15288 | 2.07252022774948 | 0.0022697034178277 | 0.0238986418700681 | 2.64403088836454 | SNAP25//MOBP//CNP//ANP32A//YWHAZ//PAK2//CALR//ARF1//GALNT1//PICALM//SERBP1//MRFAP1//GSN//NDRG1//TLK2//KLHL20//TNKS2//MLC1//MAGEE1 |
| GO:0031974 | membrane-enclosed lumen | Cellular component | 53 | 1694 | 329 | 15288 | 1.45384209600844 | 0.00328243935164071 | 0.0338974986890589 | 2.48380328945512 | SMC2//PLCB1//HNRNPK//NCL//HNRNPA1//POLA1//HNRNPM//HNRPH1//HNRNPH2//TCF7L2//HMGN5//SRSF1//ZEB1//HIF1A//FOXN3//RBPJ//TAF1//HSPA8//FMR1//MOBP//ITPR1//RPL5//HADHA//DDX46//WIPF3//HSPA9//HAUS7//KRT18//CCT5//SPTBN1//ILF2//DDX50//HNRNPA3//CAT//GATM//ARL2BP//ACADSB//DNAJA3//PDIA3//CALR//CES1D//PDIA4//FKBP9//CPSF3//GAR1//ANP32A//MED20//SENP2//KLHL20//ACTB//SLC25A5//ATP5B//DDX1 |
| GO:0005789 | endoplasmic reticulum membrane | Cellular component | 21 | 508 | 329 | 15288 | 1.92092477801977 | 0.00336435191614477 | 0.034087867527731 | 2.47309858265828 | SRPR//MAGT1//RTN4//INSIG2//ORMDL3//CALR//SPPL2A//ITPR1//CANX//FAAH//INSIG1//TMED2//HSD17B12//FADS1//RASGRF2//MGST1//PJA2//SERINC1//EI24//TMED9//ELOVL7 |
| GO:0005829 | cytosol | Cellular component | 36 | 1056 | 329 | 15288 | 1.58413926499033 | 0.00413121154550545 | 0.0410826037025264 | 2.38392256568829 | RGD1564051//RPS4X//CCT4//CCT6A//CCT5//CCT2//PIK3CB//DNAJA3//APAF1//MAPK9//TRIM37//TCF7L2//CAT//HSPA8//PLCB1//PPP3CA//PPP3CB//PDCD4//CALR//ARF1//CNBP//ACTB//PRKCI//CES1D//NRP1//RAPGEF4//ACTG1//GSN//NDRG1//HSP90AB1//NBR1//GPCPD1//ELMO2//UAP1//PDCD6IP//NCALD |
| GO:0042175 | nuclear outer membrane-endoplasmic reticulum membrane network | Cellular component | 21 | 522 | 329 | 15288 | 1.86940572267058 | 0.00459124243725908 | 0.0448272216146932 | 2.33806977402032 | SRPR//ITPR1//CANX//FAAH//INSIG1//TMED2//HSD17B12//FADS1//RASGRF2//MGST1//PJA2//SERINC1//EI24//TMED9//ELOVL7//MAGT1//RTN4//INSIG2//ORMDL3//CALR//SPPL2A |
| GO:0030660 | Golgi-associated vesicle membrane | Cellular component | 4 | 32 | 329 | 15288 | 5.80851063829787 | 0.00470703289619323 | 0.0451370833081386 | 2.32725276651489 | TMED2//ARCN1//SPPL2A//ZDHHC17 |
| GO:0005911 | cell-cell junction | Cellular component | 13 | 264 | 329 | 15288 | 2.28820116054159 | 0.00483253644269786 | 0.0455275801706798 | 2.31582486222379 | KEAP1//NDRG1//SSX2IP//MPDZ//PRKCI//CLDN11//ANK3//CLDN12//MAGI1//ITGB1//SCN4B//MLC1//PTPRK |
| GO:0016234 | inclusion body | Cellular component | 4 | 33 | 329 | 15288 | 5.63249516441006 | 0.00526696328333158 | 0.0487648152267079 | 2.27843970915383 | SEC62//TRIM37//HSP90AB1//DNAJB2 |
| GO:0071013 | catalytic step 2 spliceosome | Cellular component | 6 | 77 | 329 | 15288 | 3.62088974854932 | 0.00625126893041528 | 0.0568971426378476 | 2.20403181728931 | HNRNPA1//HNRNPM//HNRNPK//CDC40//HNRNPA3//SRSF1 |
| GO:0030864 | cortical actin cytoskeleton | Cellular component | 4 | 35 | 329 | 15288 | 5.31063829787234 | 0.00651681578112967 | 0.0583255012411105 | 2.18596455556872 | SPTA1//SPTBN1//CAP1//DSTN |
| GO:0016327 | apicolateral plasma membrane | Cellular component | 7 | 104 | 329 | 15288 | 3.12765957446809 | 0.00718361462557057 | 0.0628663748293799 | 2.14365697401299 | MPDZ//PRKCI//CLDN11//ANK3//CLDN12//MAGI1//PRICKLE2 |
| GO:0044297 | cell body | Cellular component | 18 | 441 | 329 | 15288 | 1.89665653495441 | 0.00727598601905911 | 0.0628663748293799 | 2.13810814411258 | HSPA8//SNAP25//ITPR1//LAMP1//CANX//SLC1A3//ROBO1//RTN4//EXOC4//NRP1//ERMN//VPS16//PTPRK//GLUL//CLCN2//NELL2//KLHL24//GFAP |
| GO:0070013 | intracellular organelle lumen | Cellular component | 50 | 1644 | 329 | 15288 | 1.41326292902625 | 0.00740819935833407 | 0.0628663748293799 | 2.13028733908808 | SMC2//PLCB1//HNRNPK//NCL//HNRNPA1//POLA1//HNRNPM//HNRPH1//HNRNPH2//TCF7L2//HMGN5//SRSF1//ZEB1//HIF1A//FOXN3//RBPJ//TAF1//HSPA8//FMR1//MOBP//ITPR1//RPL5//HADHA//DDX46//WIPF3//HSPA9//HAUS7//KRT18//CCT5//SPTBN1//ILF2//DDX50//HNRNPA3//ACADSB//DNAJA3//PDIA3//CALR//CES1D//PDIA4//FKBP9//CPSF3//GAR1//ANP32A//MED20//SENP2//KLHL20//ACTB//SLC25A5//ATP5B//DDX1 |
| GO:0031090 | organelle membrane | Cellular component | 50 | 1645 | 329 | 15288 | 1.41240380262562 | 0.00749245435582926 | 0.0628663748293799 | 2.12537589410429 | FAAH//GABARAPL2//GOSR1//PJA2//ZDHHC17//ATP5B//CAT//MGST1//FNDC5//ACBD5//ITPR1//CNP//VAMP1//SLC25A5//SLC1A3//GATM//HIGD1A//HADHA//GHITM//SLC25A13//LAMP1//SPPL2A//SRPR//CANX//INSIG1//TMED2//HSD17B12//FADS1//RASGRF2//SERINC1//EI24//TMED9//ELOVL7//PICALM//MAGT1//ATP6V1D//SEC24C//ST6GALNAC3//RTN4//ARCN1//SCAMP1//VPS16//KPNB1//SENP2//GALNT1//UXS1//INSIG2//ORMDL3//CALR//NDRG1 |
| GO:0043233 | organelle lumen | Cellular component | 50 | 1651 | 329 | 15288 | 1.40727089964818 | 0.00801522576178336 | 0.0662180959088872 | 2.09608424053181 | SMC2//PLCB1//HNRNPK//NCL//HNRNPA1//POLA1//HNRNPM//HNRPH1//HNRNPH2//TCF7L2//HMGN5//SRSF1//ZEB1//HIF1A//FOXN3//RBPJ//TAF1//HSPA8//FMR1//MOBP//ITPR1//RPL5//HADHA//DDX46//WIPF3//HSPA9//HAUS7//KRT18//CCT5//SPTBN1//ILF2//DDX50//HNRNPA3//ACADSB//DNAJA3//PDIA3//CALR//CES1D//PDIA4//FKBP9//CPSF3//GAR1//ANP32A//MED20//SENP2//KLHL20//ACTB//SLC25A5//ATP5B//DDX1 |
| GO:0043025 | neuronal cell body | Cellular component | 17 | 413 | 329 | 15288 | 1.91272989542012 | 0.00828461908127413 | 0.0667115933231242 | 2.08172745522338 | GLUL//CLCN2//NELL2//KLHL24//HSPA8//SNAP25//ITPR1//LAMP1//CANX//SLC1A3//ROBO1//RTN4//EXOC4//NRP1//ERMN//VPS16//PTPRK |
| GO:0005923 | tight junction | Cellular component | 6 | 82 | 329 | 15288 | 3.40010378827193 | 0.00844765055115911 | 0.0667115933231242 | 2.07326405963534 | MPDZ//PRKCI//CLDN11//ANK3//CLDN12//MAGI1 |
| GO:0070160 | occluding junction | Cellular component | 6 | 82 | 329 | 15288 | 3.40010378827193 | 0.00844765055115911 | 0.0667115933231242 | 2.07326405963534 | MPDZ//PRKCI//CLDN11//ANK3//CLDN12//MAGI1 |
| GO:0030863 | cortical cytoskeleton | Cellular component | 5 | 60 | 329 | 15288 | 3.87234042553192 | 0.009301105943667 | 0.0723868679963649 | 2.03146540879278 | SPTA1//CAP1//DSTN//SPTBN1//ACTB |
| GO:0005788 | endoplasmic reticulum lumen | Cellular component | 5 | 61 | 329 | 15288 | 3.80885943494942 | 0.00995763663222181 | 0.0763892981643302 | 2.00184372577097 | CALR//PDIA3//CES1D//PDIA4//FKBP9 |
| GO:0030016 | myofibril | Cellular component | 8 | 143 | 329 | 15288 | 2.59961315280464 | 0.0122480735664835 | 0.0926368381014315 | 1.91193221376784 | ARF1//HABP4//PPP3CA//ANK3//NBR1//SPTBN1//SDC4//ACTG1 |
| GO:0015630 | microtubule cytoskeleton | Cellular component | 25 | 721 | 329 | 15288 | 1.61123734765544 | 0.0130526722549153 | 0.0973511805679099 | 1.88430056673509 | TNKS2//NEK7//CCT4//PLK2//KEAP1//HAUS7//CCT5//NDRG1//AZI1//ARL2BP//DDHD2//CLASP2//SLMAP//KRT18//PDCD6IP//PTP4A1//ZFP828//ACTR10//CNP//HSPH1//CCT6A//CCT2//MAP4//SLC25A5//DBC1 |
| GO:0043198 | dendritic shaft | Cellular component | 4 | 43 | 329 | 15288 | 4.32261256803563 | 0.0134356763138212 | 0.0988350435687943 | 1.87174046752869 | FMR1//NSF//EXOC4//LPAR1 |
| GO:0030135 | coated vesicle | Cellular component | 10 | 206 | 329 | 15288 | 2.25573228671762 | 0.0139060900054915 | 0.100913112607418 | 1.85679496402262 | TMED2//KLHL12//PICALM//HSPA8//SEC24C//MLC1//GOPC//ARCN1//VAMP1//SCAMP1 |
| GO:0005681 | spliceosomal complex | Cellular component | 7 | 119 | 329 | 15288 | 2.7334167709637 | 0.0144557693663523 | 0.103503308663082 | 1.83995878931538 | HNRNPA1//HNRNPM//HNRNPK//CDC40//HNRNPA3//SRSF1//HNRPH1 |
| GO:0044448 | cell cortex part | Cellular component | 6 | 95 | 329 | 15288 | 2.93482642777156 | 0.0166592330477877 | 0.11771063350871 | 1.77834499637345 | EXOC4//SPTA1//ACTB//SPTBN1//CAP1//DSTN |
| GO:0043292 | contractile fiber | Cellular component | 8 | 152 | 329 | 15288 | 2.44568868980963 | 0.0171465917930565 | 0.119580776530797 | 1.76582219122263 | ACTG1//ARF1//HABP4//PPP3CA//ANK3//NBR1//SPTBN1//SDC4 |
| GO:0010494 | cytoplasmic stress granule | Cellular component | 3 | 26 | 329 | 15288 | 5.36170212765957 | 0.0178010559941851 | 0.122497820485506 | 1.74955423371109 | FMR1//DDX1//LSM14A |
| GO:0044431 | Golgi apparatus part | Cellular component | 14 | 346 | 329 | 15288 | 1.88021153609642 | 0.0180210946338082 | 0.122497820485506 | 1.7442188327252 | FAAH//GABARAPL2//GOSR1//PJA2//ZDHHC17//NSF//ADAM10//TMED2//GOPC//ST6GALNAC3//SPPL2A//ARCN1//GALNT1//UXS1 |
| GO:0043194 | axon initial segment | Cellular component | 2 | 10 | 329 | 15288 | 9.2936170212766 | 0.0185359515845681 | 0.124422575011413 | 1.73198511358269 | ANK3//NRCAM |
| GO:0005778 | peroxisomal membrane | Cellular component | 4 | 48 | 329 | 15288 | 3.87234042553191 | 0.0194925149088492 | 0.127652201293317 | 1.71013212493704 | CAT//MGST1//FNDC5//ACBD5 |
| GO:0031903 | microbody membrane | Cellular component | 4 | 48 | 329 | 15288 | 3.87234042553191 | 0.0194925149088492 | 0.127652201293317 | 1.71013212493704 | CAT//MGST1//FNDC5//ACBD5 |
| GO:0044449 | contractile fiber part | Cellular component | 7 | 127 | 329 | 15288 | 2.56123303735969 | 0.0199761656180745 | 0.127965373578717 | 1.69948787000004 | ARF1//HABP4//PPP3CA//ANK3//NBR1//SPTBN1//SDC4 |
| GO:0030662 | coated vesicle membrane | Cellular component | 6 | 99 | 329 | 15288 | 2.81624758220503 | 0.0200169299452742 | 0.127965373578717 | 1.6986025307562 | PICALM//SEC24C//TMED2//ARCN1//VAMP1//SCAMP1 |
| GO:0043296 | apical junction complex | Cellular component | 6 | 100 | 329 | 15288 | 2.78808510638298 | 0.0209231661183595 | 0.13218517888893 | 1.67937259687494 | MPDZ//PRKCI//CLDN11//ANK3//CLDN12//MAGI1 |
| GO:0031430 | M band | Cellular component | 2 | 11 | 329 | 15288 | 8.44874274661509 | 0.0223365571305187 | 0.137870473322857 | 1.6509837665134 | NBR1//SPTBN1 |
| GO:0043220 | Schmidt-Lanterman incisure | Cellular component | 2 | 11 | 329 | 15288 | 8.44874274661509 | 0.0223365571305187 | 0.137870473322857 | 1.6509837665134 | MPDZ//PRKCI |
| GO:0044304 | main axon | Cellular component | 4 | 51 | 329 | 15288 | 3.64455569461827 | 0.0238190111519012 | 0.145350102142852 | 1.62307627224816 | ROBO1//ANK3//ERMN//NRCAM |
| GO:0031981 | nuclear lumen | Cellular component | 41 | 1397 | 329 | 15288 | 1.36377343547724 | 0.0254364093407756 | 0.153475863101084 | 1.59454419465675 | SMC2//PLCB1//HNRNPK//NCL//HNRNPA1//POLA1//HNRNPM//HNRPH1//HNRNPH2//TCF7L2//HMGN5//SRSF1//ZEB1//HIF1A//FOXN3//RBPJ//TAF1//HSPA8//FMR1//MOBP//ITPR1//RPL5//HADHA//DDX46//WIPF3//HSPA9//HAUS7//KRT18//CCT5//SPTBN1//ILF2//DDX50//HNRNPA3//CPSF3//GAR1//ANP32A//MED20//SENP2//KLHL20//ACTB//DDX1 |
| GO:0031463 | Cul3-RING ubiquitin ligase complex | Cellular component | 2 | 12 | 329 | 15288 | 7.74468085106383 | 0.026427737218439 | 0.155952691058261 | 1.57794002020598 | KLHL12//KLHL20 |
| GO:0042589 | zymogen granule membrane | Cellular component | 2 | 12 | 329 | 15288 | 7.74468085106383 | 0.026427737218439 | 0.155952691058261 | 1.57794002020598 | SCAMP1//TMED2 |
| GO:0005815 | microtubule organizing center | Cellular component | 15 | 402 | 329 | 15288 | 1.73388377262623 | 0.0279071458628262 | 0.162892797047149 | 1.55428457767863 | TNKS2//CCT4//PLK2//KEAP1//HAUS7//CCT5//NDRG1//AZI1//ARL2BP//DDHD2//KRT18//CLASP2//SLMAP//NEK7//PDCD6IP |
| GO:0030424 | axon | Cellular component | 13 | 334 | 329 | 15288 | 1.80863804306281 | 0.0291955629834178 | 0.16858083142038 | 1.53468314575147 | NRP1//ROBO1//ANK3//ERMN//NRCAM//GLUL//SNAP25//CANX//ACTB//RAPGEF4//VPS16//KLHL24//PTPRK |
| GO:0015629 | actin cytoskeleton | Cellular component | 12 | 301 | 329 | 15288 | 1.85254824344384 | 0.030156935185607 | 0.172279512709266 | 1.52061279740479 | ACTR10//VPS16//DNAJA3//SPTA1//CAP1//DSTN//ACTG1//SPTBN1//SNAP25//CLCN2//GSN//HNRNPH2 |
| GO:0000407 | pre-autophagosomal structure | Cellular component | 2 | 13 | 329 | 15288 | 7.14893617021277 | 0.0307953049635166 | 0.174074513320089 | 1.51151549076786 | SQSTM1//NBR1 |
| GO:0097060 | synaptic membrane | Cellular component | 9 | 203 | 329 | 15288 | 2.06016140865737 | 0.0318620398126023 | 0.178228285201744 | 1.49672642404651 | NPTN//PICALM//MPDZ//PJA2//MAGEE1//DNAJA3//ANK3//LRRC4//NT5E |
| GO:0045211 | postsynaptic membrane | Cellular component | 8 | 172 | 329 | 15288 | 2.16130628401781 | 0.0328024568476471 | 0.18159710646584 | 1.48409362715976 | MPDZ//NPTN//PICALM//PJA2//MAGEE1//DNAJA3//ANK3//LRRC4 |
| GO:0030663 | COPI coated vesicle membrane | Cellular component | 2 | 14 | 329 | 15288 | 6.63829787234043 | 0.0354255462864541 | 0.194117534243121 | 1.45068344390723 | TMED2//ARCN1 |
| GO:0014704 | intercalated disc | Cellular component | 3 | 34 | 329 | 15288 | 4.10012515644556 | 0.0361461943908628 | 0.196065721089832 | 1.44193742014123 | ITGB1//SCN4B//ANK3 |
| GO:0031256 | leading edge membrane | Cellular component | 5 | 86 | 329 | 15288 | 2.70163285502227 | 0.0379416337241343 | 0.203746573098601 | 1.42088397281212 | ROBO1//ITGB1//CLASP2//PLEKHA1//PTPRK |
| GO:0005938 | cell cortex | Cellular component | 8 | 178 | 329 | 15288 | 2.08845326320822 | 0.0389501154026146 | 0.206132340460341 | 1.4094912512483 | EXOC4//SPTA1//ACTB//SPTBN1//CAP1//DSTN//NCL//CLASP2 |
| GO:0044438 | microbody part | Cellular component | 4 | 60 | 329 | 15288 | 3.09787234042553 | 0.0400631038677056 | 0.206132340460341 | 1.39725540700454 | CAT//MGST1//FNDC5//ACBD5 |
| GO:0044439 | peroxisomal part | Cellular component | 4 | 60 | 329 | 15288 | 3.09787234042553 | 0.0400631038677056 | 0.206132340460341 | 1.39725540700454 | CAT//MGST1//FNDC5//ACBD5 |
| GO:0030134 | ER to Golgi transport vesicle | Cellular component | 2 | 15 | 329 | 15288 | 6.19574468085106 | 0.0403052062352623 | 0.206132340460341 | 1.3946388522904 | SEC24C//KLHL12 |
| GO:0043218 | compact myelin | Cellular component | 2 | 15 | 329 | 15288 | 6.19574468085106 | 0.0403052062352623 | 0.206132340460341 | 1.3946388522904 | MPDZ//PRKCI |
| GO:0030017 | sarcomere | Cellular component | 6 | 117 | 329 | 15288 | 2.38297872340426 | 0.0407593397866977 | 0.206488353447704 | 1.38977285924157 | PPP3CA//ANK3//NBR1//SPTBN1//ARF1//HABP4 |
| GO:0044291 | cell-cell contact zone | Cellular component | 3 | 36 | 329 | 15288 | 3.87234042553191 | 0.0418070850207227 | 0.209816865945122 | 1.37875011237181 | ITGB1//SCN4B//ANK3 |
| GO:0030659 | cytoplasmic vesicle membrane | Cellular component | 9 | 214 | 329 | 15288 | 1.9542652614834 | 0.0423206112678028 | 0.210427483803797 | 1.37344806809205 | ITPR1//PICALM//SEC24C//SPPL2A//ZDHHC17//TMED2//ARCN1//VAMP1//SCAMP1 |
| GO:0044428 | nuclear part | Cellular component | 49 | 1784 | 329 | 15288 | 1.2763095124511 | 0.0430680596823172 | 0.212179339902792 | 1.36584469378754 | SMC2//PLCB1//HNRNPK//ITPR1//RTN4//POLA1//TNKS2//SENP2//NCL//HNRNPA1//HNRNPM//HNRPH1//HNRNPH2//TCF7L2//HMGN5//SRSF1//ZEB1//HIF1A//FOXN3//RBPJ//TAF1//HNRNPA3//HSPA8//FMR1//MOBP//RPL5//HADHA//DDX46//WIPF3//HSPA9//HAUS7//KRT18//CCT5//SPTBN1//ILF2//DDX50//CPSF3//HSP90AB1//VPRBP//GAR1//ANP32A//MED20//KLHL20//KPNB1//EI24//UPF2//ACTB//CDC40//DDX1 |
| GO:0055037 | recycling endosome | Cellular component | 4 | 62 | 329 | 15288 | 2.99794097460535 | 0.0443502839983788 | 0.216510022792086 | 1.35310359480556 | SCAMP1//NDRG1//VPS16//MLC1 |
| GO:0005777 | peroxisome | Cellular component | 6 | 121 | 329 | 15288 | 2.30420256725866 | 0.0467160876570093 | 0.223986955998339 | 1.33053353533311 | CAT//MGST1//FNDC5//ACBD5//PHYH//TRIM37 |
| GO:0042579 | microbody | Cellular component | 6 | 121 | 329 | 15288 | 2.30420256725866 | 0.0467160876570093 | 0.223986955998339 | 1.33053353533311 | CAT//PHYH//TRIM37//MGST1//FNDC5//ACBD5 |
| GO:0044459 | plasma membrane part | Cellular component | 40 | 1426 | 329 | 15288 | 1.3034525976545 | 0.0495599467050735 | 0.235197463747839 | 1.30486916931988 | SCN4B//CDC42SE2//FCGRT//PLP1//LRP12//ITGB1//CACNA2D3//MLC1//PICALM//MPDZ//PRKCI//CLDN11//ANK3//CLDN12//MAGI1//SDC4//KEAP1//SNAP25//CD9//LAMP1//CR1L//KIT//CALR//SEMA7A//PTP4A1//PTPN3//MAGEE1//DDR1//SLC1A3//RAPGEF4//HSP90AB1//PRICKLE2//DNAJA3//ROBO1//PRIMA1//PTPRK//PIK3CB//EXOC4//CLASP2//PLEKHA1 |
