# Supplementary material for: Gene Expression in the Hippocampus in a Rat Model of Premenstrual Dysphoric Disorder After Treatment With Baixiangdan Capsules
Source: Front Psychol. 2018 Nov 13;9:2065. doi: 10.3389/fpsyg.2018.02065 (PMC6242977; doi:10.3389/fpsyg.2018.02065)
Supplement: Supplementary file 3 [file Data_Sheet_3.ZIP › Data Analysis Folder/GO Analysis Report/BXD vs blank (down)/MF_result(Rat).html]

| GO.ID | Term | Ontology | Count | Pop.Hits | List.Total | Pop.Total | Fold.Enrichment | Pvalue | FDR | Enrichment.Score | GENES |
| --- | --- | --- | --- | --- | --- | --- | --- | --- | --- | --- | --- |
| GO:0005488 | binding | Molecular function | 246 | 9220 | 302 | 14392 | 1.27150594015314 | 1.7205570531225e-11 | 1.30920017212958e-08 | 10.7643309217379 | HSPA8//CANX//CCT4//CALR//HSPA9//CCT5//CCT2//DNAJA4//HSP90AB1//DNAJA3//DNAJB2//HADHA//ACBD5//SNAP25//NAPA//NCL//HNRNPA1//NT5E//HSD17B12//POLA1//HDGF//HNRNPM//RCAN2//HNRPH1//UXS1//TSTA3//HNRPDL//HNRNPH2//FUS//PARN//GLYR1//HNRNPH3//HNRNPA3//MAT2B//SRSF1//ITGB1//PLCB1//PPP3CA//PPP3CB//SDC4//KPNB1//CD9//SLC25A5//ITPR1//NFIA//YWHAZ//ZEB1//KCNAB1//MAPK9//ROBO1//CD14//NSF//TMED2//PLK2//RTN4//PICALM//SQSTM1//PDIA4//EXOC4//KEAP1//NRP1//RAPGEF4//MRFAP1//VPS16//PIAS1//EIF5B//HABP4//TMED9//ANK3//NCALD//PPM1B//GLUL//OXSR1//RGD1306565//EGR1//HNRNPK//RBPJ//MKX//RGN//NELL2//PLS3//LRP4//PLCL1//STIM2//ATP5B//SPTA1//FKBP9//USP32//CDH19//SLC25A13//RGD1304592//GLA//PTPN3//LPAR1//KIT//ADAMTS4//TAF1//DDX46//ZFP445//ZFP644//ZFP828//ZBTB2//RLF//ZIC4//ARIH2//DDX3X//ZMAT2//HIF1A//DDX1//DMTF1//MRF//EIF4G3//RNF141//ILF2//UPF2//HMG1L1//TCF7L2//HMGN5//FOXN3//CNBP//FMR1//CNP//PDCD4//CNOT6//CPSF3//AUH//DDX50//PUM1//SERBP1//EIF5//EIF3D//EEF2//CAP1//WIPF3//GSN//KLHL20//DSTN//SSX2IP//ERMN//CAT//CNPY4//CLCF1//ANP32A//NPTN//ASGR2//GALNT1//UAP1//SRPR//NBR1//GOPC//TRIM37//GFAP//SEMA7A//FNDC5//GOSR1//RLBP1//ADAM10//CHN1//CHN2//PHYH//NPTX1//CACNA2D3//NEK7//HDAC8//DDHD2//SC5DL//CAMKK1//RASGRF2//DDR1//MAPK10//PAK2//APAF1//ACTB//PRKCI//PIK3CB//PAICS//ACTG1//HSPH1//TLK2//RGD1560691//MAP3K4//DCLK3//SMC2//MAGI1//ARF1//ARF4//DOCK9//FAAH//ARHGEF26//ACAP2//MGLL//FABP12//ELMO2//CCL6//CCL9//PTPRK//CLASP2//NDRG1//MPDZ//RPL5//RGD1564051//INSIG2//PJA2//LIMCH1//LONRF1//PRICKLE2//ZFP280D//SEC24C//SLC1A3//CLDN11//MGST1//MAP4//GCGR//DNAJC7//PDCD6IP//TNKS2//PRIMA1//RPS4X//FCGRT//SPRY2//SCAMP1//SENP2//LOC681140//FADS1//OLFML2B//PLEKHA1//SEMA3C//SPTBN1//NRCAM//GAR1//CUL4A//HAUS7//MLC1//GSTM2//SPPL2A//SUMF1//GPC5//SCN4B//ATMIN//KDELR3//SLC24A2//RGD1564943//ACADSB//MESDC2//RRN3//GPCPD1 |
| GO:0005515 | protein binding | Molecular function | 151 | 4586 | 302 | 14392 | 1.56912341910161 | 2.91906392893997e-11 | 1.30920017212958e-08 | 10.5347563936445 | HSPA8//CANX//CCT4//CALR//HSPA9//CCT5//CCT2//DNAJA4//HSP90AB1//DNAJA3//DNAJB2//SNAP25//NAPA//MKX//RBPJ//PTPN3//ITGB1//LPAR1//SDC4//HSD17B12//KIT//ADAMTS4//TAF1//PLCB1//CAP1//PLS3//WIPF3//VPS16//GSN//KLHL20//DSTN//NCALD//SSX2IP//ERMN//CAT//GLA//CNPY4//CLCF1//ANP32A//NPTN//NRP1//NBR1//NELL2//SQSTM1//GOPC//TRIM37//GFAP//CD9//SEMA7A//FNDC5//GOSR1//PPP3CA//PPP3CB//CAMKK1//RASGRF2//DDR1//LRP4//ELMO2//CCL6//CCL9//PTPRK//TCF7L2//CLASP2//NDRG1//NCL//MPDZ//NSF//FOXN3//HDGF//EGR1//NFIA//YWHAZ//ZEB1//HIF1A//MAPK9//INSIG2//GLUL//PAK2//ROBO1//APAF1//CLDN11//PICALM//PAICS//MGST1//ACTG1//MAP4//RAPGEF4//DOCK9//DNAJC7//ADAM10//PDCD6IP//PIAS1//TNKS2//PRIMA1//FCGRT//ACTB//SPRY2//PARN//RGD1306565//ITPR1//KPNB1//SCAMP1//SENP2//PRKCI//HNRNPM//LOC681140//EXOC4//PLEKHA1//SEMA3C//SPTBN1//NRCAM//CUL4A//HAUS7//RTN4//HADHA//MLC1//PJA2//GLYR1//ATP5B//GSTM2//MGLL//FAAH//OLFML2B//SPPL2A//SUMF1//HSPH1//GPC5//PIK3CB//SCN4B//ATMIN//POLA1//SPTA1//SMC2//SLC24A2//RGD1564943//SRSF1//MESDC2//MAGI1//RRN3//SLC25A5//KCNAB1//CD14//TMED2//PLK2//PDIA4//KEAP1//MRFAP1//EIF5B//HABP4//TMED9//ANK3 |
| GO:0003723 | RNA binding | Molecular function | 34 | 624 | 302 | 14392 | 2.59662081847512 | 3.41960666442875e-07 | 0.00010224623926642 | 6.46602384519887 | DDX1//ILF2//CNBP//CALR//HNRNPA3//SERBP1//AUH//EIF5//EIF5B//EIF3D//EEF2//RPL5//RGD1564051//RPS4X//GAR1//FMR1//NCL//CNP//HNRNPA1//PDCD4//HNRNPM//HNRNPK//HNRPH1//DDX46//CNOT6//EIF4G3//CPSF3//HNRPDL//HNRNPH2//PARN//UPF2//DDX50//PUM1//SRSF1 |
| GO:0051082 | unfolded protein binding | Molecular function | 11 | 84 | 302 | 14392 | 6.24061810154525 | 1.37837963002493e-06 | 0.000309101632033091 | 5.86063115360496 | HSPA8//CANX//CCT4//CALR//HSPA9//CCT5//CCT2//DNAJA4//HSP90AB1//DNAJA3//DNAJB2 |
| GO:0000166 | nucleotide binding | Molecular function | 67 | 2016 | 302 | 14392 | 1.58379323031641 | 6.74757640960601e-05 | 0.00877975143675055 | 4.17085218878962 | HSP90AB1//HSPA8//GLUL//MAPK10//DDR1//CCT4//PAK2//MAPK9//CAMKK1//NSF//APAF1//ACTB//PLK2//PRKCI//DDX1//PIK3CB//PAICS//ATP5B//DDX46//ACTG1//HSPH1//HSPA9//CCT5//CCT2//DNAJA4//TLK2//RGD1560691//MAP3K4//ILF2//DCLK3//OXSR1//DDX3X//NEK7//DDX50//SMC2//RGD1306565//MAGI1//EEF2//EIF5//ARF1//ARF4//DOCK9//SRPR//POLA1//CNP//RAPGEF4//CAT//HADHA//NCL//HNRNPA1//NT5E//HSD17B12//HDGF//HNRNPM//RCAN2//HNRPH1//UXS1//TSTA3//HNRPDL//HNRNPH2//FUS//PARN//GLYR1//HNRNPH3//HNRNPA3//MAT2B//SRSF1 |
| GO:1901265 | nucleoside phosphate binding | Molecular function | 67 | 2016 | 302 | 14392 | 1.58379323031641 | 6.74757640960601e-05 | 0.00877975143675055 | 4.17085218878962 | NCL//HNRNPA1//NT5E//HSD17B12//POLA1//HDGF//HNRNPM//RCAN2//HNRPH1//UXS1//TSTA3//HNRPDL//HNRNPH2//FUS//PARN//GLYR1//HNRNPH3//HNRNPA3//MAT2B//SRSF1//HSP90AB1//HSPA8//GLUL//MAPK10//DDR1//CCT4//PAK2//MAPK9//CAMKK1//NSF//APAF1//ACTB//PLK2//PRKCI//DDX1//PIK3CB//PAICS//ATP5B//DDX46//ACTG1//HSPH1//HSPA9//CCT5//CCT2//DNAJA4//TLK2//RGD1560691//MAP3K4//ILF2//DCLK3//OXSR1//DDX3X//NEK7//DDX50//SMC2//RGD1306565//MAGI1//EEF2//EIF5//ARF1//ARF4//DOCK9//SRPR//CNP//RAPGEF4//CAT//HADHA |
| GO:0097159 | organic cyclic compound binding | Molecular function | 67 | 2017 | 302 | 14392 | 1.58300800809017 | 6.85153400861247e-05 | 0.00877975143675055 | 4.16421218224625 | NCL//HNRNPA1//NT5E//HSD17B12//POLA1//HDGF//HNRNPM//RCAN2//HNRPH1//UXS1//TSTA3//HNRPDL//HNRNPH2//FUS//PARN//GLYR1//HNRNPH3//HNRNPA3//MAT2B//SRSF1//HSP90AB1//HSPA8//GLUL//MAPK10//DDR1//CCT4//PAK2//MAPK9//CAMKK1//NSF//APAF1//ACTB//PLK2//PRKCI//DDX1//PIK3CB//PAICS//ATP5B//DDX46//ACTG1//HSPH1//HSPA9//CCT5//CCT2//DNAJA4//TLK2//RGD1560691//MAP3K4//ILF2//DCLK3//OXSR1//DDX3X//NEK7//DDX50//SMC2//RGD1306565//MAGI1//EEF2//EIF5//ARF1//ARF4//DOCK9//SRPR//CNP//RAPGEF4//CAT//HADHA |
| GO:0019899 | enzyme binding | Molecular function | 41 | 1076 | 302 | 14392 | 1.81587434451857 | 0.000141304085581567 | 0.0158437205958332 | 3.84984528103977 | ITGB1//KIT//ADAMTS4//NBR1//SDC4//NELL2//SQSTM1//RAPGEF4//DOCK9//NSF//NDRG1//GFAP//PAK2//HIF1A//ADAM10//ACTB//HSP90AB1//SPRY2//PTPRK//PARN//DNAJA3//RGD1306565//TCF7L2//ITPR1//PPP3CB//MAPK9//CALR//PIAS1//TRIM37//CUL4A//HAUS7//PJA2//EGR1//PTPN3//RRN3//CAT//PLCB1//PPP3CA//HSPA9//TNKS2//PRIMA1 |
| GO:0036094 | small molecule binding | Molecular function | 69 | 2182 | 302 | 14392 | 1.50698368954905 | 0.000234321041681205 | 0.0233539971542268 | 3.63018871065305 | NCL//HNRNPA1//NT5E//HSD17B12//POLA1//HDGF//HNRNPM//RCAN2//HNRPH1//UXS1//TSTA3//HNRPDL//HNRNPH2//FUS//PARN//GLYR1//HNRNPH3//HNRNPA3//MAT2B//SRSF1//HSP90AB1//RLBP1//HSPA8//GLUL//MAPK10//DDR1//CCT4//PAK2//MAPK9//CAMKK1//NSF//APAF1//ACTB//PLK2//PRKCI//DDX1//PIK3CB//PAICS//ATP5B//DDX46//ACTG1//HSPH1//HSPA9//CCT5//CCT2//DNAJA4//TLK2//RGD1560691//MAP3K4//ILF2//DCLK3//OXSR1//DDX3X//NEK7//DDX50//SMC2//RGD1306565//MAGI1//EEF2//EIF5//ARF1//ARF4//DOCK9//SRPR//CNP//RAPGEF4//PHYH//CAT//HADHA |
| GO:0019901 | protein kinase binding | Molecular function | 18 | 378 | 302 | 14392 | 2.26931567328918 | 0.00107511283578031 | 0.0964376213694938 | 2.96854595306455 | NBR1//SDC4//NELL2//SQSTM1//MAPK9//PJA2//ITGB1//PAK2//HIF1A//ADAM10//ACTB//HSP90AB1//SPRY2//PTPRK//PARN//DNAJA3//RGD1306565//TCF7L2 |
| GO:0019900 | kinase binding | Molecular function | 19 | 419 | 302 | 14392 | 2.16099511609161 | 0.00139614310430441 | 0.113849124051005 | 2.85507006436008 | NBR1//SDC4//NELL2//SQSTM1//ITGB1//PAK2//HIF1A//ADAM10//ACTB//HSP90AB1//SPRY2//PTPRK//PARN//DNAJA3//RGD1306565//TCF7L2//MAPK9//PJA2//GFAP |
| GO:0005200 | structural constituent of cytoskeleton | Molecular function | 4 | 25 | 302 | 14392 | 7.62490066225166 | 0.00169741524275332 | 0.126881789395811 | 2.77021190218651 | GFAP//ACTG1//SPTBN1//ANK3 |
| GO:0019904 | protein domain specific binding | Molecular function | 23 | 564 | 302 | 14392 | 1.9434033159551 | 0.00185904765361472 | 0.128274288099416 | 2.730709477664 | ADAM10//PICALM//WIPF3//PDCD6IP//NSF//EXOC4//LPAR1//PLEKHA1//ROBO1//SQSTM1//SRSF1//TCF7L2//ITGB1//KPNB1//SNAP25//YWHAZ//SCAMP1//SENP2//PRKCI//HNRNPM//PIAS1//SSX2IP//LOC681140 |
| GO:0016788 | hydrolase activity, acting on ester bonds | Molecular function | 25 | 639 | 302 | 14392 | 1.86446123392304 | 0.00209594221505591 | 0.133839623530595 | 2.67862069498425 | RGD1308874//MGLL//CES1D//ABHD3//CNP//USP32//RGN//PLCB1//PLCL1//DDX1//CPSF3//CNOT6//PARN//DDHD2//PPP3CA//PPM1B//PPP3CB//PTP4A1//MTMR6//PTPRK//PTPN3//NT5E//POLA1//GPCPD1//FAAH |
| GO:0016505 | apoptotic protease activator activity | Molecular function | 3 | 13 | 302 | 14392 | 10.9974528782476 | 0.00223812079482601 | 0.133839623530595 | 2.65011647763311 | MAPK9//RGD1306565//APAF1 |
| GO:0032403 | protein complex binding | Molecular function | 18 | 410 | 302 | 14392 | 2.0921983524471 | 0.00262873790947503 | 0.140767546629425 | 2.58025271177984 | GFAP//ITGB1//CD9//CALR//SEMA7A//FCGRT//DNAJB2//ITPR1//YWHAZ//HIF1A//NSF//RTN4//EXOC4//HADHA//RAPGEF4//SPTBN1//MLC1//RBPJ |
| GO:0004527 | exonuclease activity | Molecular function | 5 | 46 | 302 | 14392 | 5.17995968902966 | 0.0026678353318843 | 0.140767546629425 | 2.57384098010069 | PARN//POLA1//CPSF3//DDX1//CNOT6 |
| GO:0008092 | cytoskeletal protein binding | Molecular function | 21 | 529 | 302 | 14392 | 1.89181136468909 | 0.00396218741071056 | 0.197449005967076 | 2.40206498626529 | ITGB1//CAP1//PLS3//WIPF3//VPS16//GSN//KLHL20//DSTN//NCALD//ERMN//CLASP2//NDRG1//MAP4//SNAP25//ACTB//SPTBN1//NRCAM//HSPH1//MAGI1//SDC4//ANP32A |
| GO:0031072 | heat shock protein binding | Molecular function | 7 | 102 | 302 | 14392 | 3.27048435268147 | 0.00564978586669272 | 0.266729364338072 | 2.24796801212323 | HIF1A//DNAJA3//DNAJB2//APAF1//HSPA9//DNAJA4//DNAJC7 |
| GO:0001968 | fibronectin binding | Molecular function | 3 | 20 | 302 | 14392 | 7.14834437086093 | 0.00800408043631793 | 0.358130670586726 | 2.09668855560885 | ITGB1//SDC4//HSD17B12 |
| GO:0005178 | integrin binding | Molecular function | 5 | 60 | 302 | 14392 | 3.97130242825607 | 0.0083843300806257 | 0.358130670586726 | 2.0765316323913 | GFAP//ITGB1//CD9//CALR//SEMA7A |
| GO:0046983 | protein dimerization activity | Molecular function | 28 | 839 | 302 | 14392 | 1.59041432168539 | 0.0102893418475912 | 0.41783063167975 | 1.9876124037702 | CAT//GSTM2//PLCB1//MGLL//FAAH//ADAM10//KIT//LRP4//SQSTM1//MGST1//OLFML2B//GOPC//SPPL2A//TRIM37//SUMF1//GLA//RGD1306565//ITGB1//PPP3CA//PPP3CB//HIF1A//ROBO1//POLA1//SPTA1//SMC2//CLCF1//SLC24A2//PDCD6IP |
| GO:0005083 | small GTPase regulator activity | Molecular function | 10 | 203 | 302 | 14392 | 2.34756793788536 | 0.0107136059405064 | 0.41783063167975 | 1.97006433155014 | RAPGEF4//RASGRF2//DOCK9//ARHGEF26//TBC1D14//ACAP2//MADD//CHN2//GOPC//DNAJA3 |
| GO:0016831 | carboxy-lyase activity | Molecular function | 3 | 23 | 302 | 14392 | 6.21595162683559 | 0.0118725473645445 | 0.427844024217468 | 1.92545608916062 | GLUL//PAICS//UXS1 |
| GO:0032559 | adenyl ribonucleotide binding | Molecular function | 38 | 1243 | 302 | 14392 | 1.4568897081937 | 0.0119243039079562 | 0.427844024217468 | 1.92356696388174 | HSPA8//GLUL//MAPK10//DDR1//CCT4//PAK2//MAPK9//CAMKK1//NSF//APAF1//ACTB//PLK2//PRKCI//DDX1//PIK3CB//PAICS//ATP5B//DDX46//ACTG1//HSPH1//HSPA9//CCT5//CCT2//DNAJA4//HSP90AB1//TLK2//RGD1560691//MAP3K4//ILF2//DCLK3//OXSR1//DDX3X//NEK7//DDX50//SMC2//RGD1306565//MAGI1//RAPGEF4 |
| GO:0016298 | lipase activity | Molecular function | 6 | 92 | 302 | 14392 | 3.10797581341779 | 0.012844171978835 | 0.435879378933744 | 1.89129388801997 | PLCB1//PLCL1//DDHD2//CES1D//MGLL//FAAH |
| GO:0030554 | adenyl nucleotide binding | Molecular function | 38 | 1251 | 302 | 14392 | 1.44757306737392 | 0.0131201150849622 | 0.435879378933744 | 1.88206235546761 | HSPA8//GLUL//MAPK10//DDR1//CCT4//PAK2//MAPK9//CAMKK1//NSF//APAF1//ACTB//PLK2//PRKCI//DDX1//PIK3CB//PAICS//ATP5B//DDX46//ACTG1//HSPH1//HSPA9//CCT5//CCT2//DNAJA4//HSP90AB1//TLK2//RGD1560691//MAP3K4//ILF2//DCLK3//OXSR1//DDX3X//NEK7//DDX50//SMC2//RGD1306565//MAGI1//RAPGEF4 |
| GO:0042578 | phosphoric ester hydrolase activity | Molecular function | 13 | 309 | 302 | 14392 | 2.00492938125549 | 0.0137414437735862 | 0.440216966603815 | 1.86196763481936 | CNP//PLCB1//PLCL1//DDHD2//PPP3CA//PPM1B//PPP3CB//PTP4A1//MTMR6//PTPRK//PTPN3//NT5E//GPCPD1 |
| GO:0005524 | ATP binding | Molecular function | 37 | 1218 | 302 | 14392 | 1.44766689502931 | 0.0143063979762145 | 0.442511689126359 | 1.84446969789428 | HSPA8//GLUL//MAPK10//DDR1//CCT4//PAK2//MAPK9//CAMKK1//NSF//APAF1//ACTB//PLK2//PRKCI//DDX1//PIK3CB//PAICS//ATP5B//DDX46//ACTG1//HSPH1//HSPA9//CCT5//CCT2//DNAJA4//HSP90AB1//TLK2//RGD1560691//MAP3K4//ILF2//DCLK3//OXSR1//DDX3X//NEK7//DDX50//SMC2//RGD1306565//MAGI1 |
| GO:0004629 | phospholipase C activity | Molecular function | 3 | 25 | 302 | 14392 | 5.71867549668874 | 0.0149521145662388 | 0.44706822553054 | 1.82529738396104 | PLCB1//PLCL1//DDHD2 |
| GO:0017076 | purine nucleotide binding | Molecular function | 45 | 1559 | 302 | 14392 | 1.37556338117914 | 0.0166484849704094 | 0.474908775454174 | 1.7786252816128 | HSPA8//GLUL//MAPK10//DDR1//CCT4//PAK2//MAPK9//CAMKK1//NSF//APAF1//ACTB//PLK2//PRKCI//DDX1//PIK3CB//PAICS//ATP5B//DDX46//ACTG1//HSPH1//HSPA9//CCT5//CCT2//DNAJA4//HSP90AB1//TLK2//RGD1560691//MAP3K4//ILF2//DCLK3//OXSR1//DDX3X//NEK7//DDX50//SMC2//RGD1306565//MAGI1//EEF2//EIF5//ARF1//ARF4//DOCK9//SRPR//RAPGEF4//POLA1 |
| GO:0015172 | acidic amino acid transmembrane transporter activity | Molecular function | 2 | 10 | 302 | 14392 | 9.53112582781457 | 0.0176702023238525 | 0.474908775454174 | 1.75275847779207 | SLC1A3//SLC25A13 |
| GO:0051393 | alpha-actinin binding | Molecular function | 2 | 10 | 302 | 14392 | 9.53112582781457 | 0.0176702023238525 | 0.474908775454174 | 1.75275847779207 | ITGB1//MAGI1 |
| GO:0016504 | peptidase activator activity | Molecular function | 3 | 27 | 302 | 14392 | 5.29506990434143 | 0.0184408050268506 | 0.474908775454174 | 1.73422012389586 | MAPK9//RGD1306565//APAF1 |
| GO:0005088 | Ras guanyl-nucleotide exchange factor activity | Molecular function | 5 | 73 | 302 | 14392 | 3.26408418760773 | 0.0185304427434739 | 0.474908775454174 | 1.73211420406208 | RASGRF2//DOCK9//ARHGEF26//MADD//RAPGEF4 |
| GO:0030234 | enzyme regulator activity | Molecular function | 25 | 766 | 302 | 14392 | 1.55534037660159 | 0.0191534575476088 | 0.477240317227919 | 1.71775281656185 | RGD1310507//GOPC//DNAJA3//RAPGEF4//DOCK9//RASGRF2//ARHGEF26//PLCB1//CHN1//TBC1D14//MGST2//ACAP2//OAZ2//PPP2R5C//MAPK9//RGD1306565//APAF1//MADD//ANP32E//PAK2//CHN2//TRIAP1//SPRY2//MAT2B//RGN |
| GO:0008656 | cysteine-type endopeptidase activator activity involved in apoptotic process | Molecular function | 2 | 11 | 302 | 14392 | 8.66465984346779 | 0.0213008989792895 | 0.50281332590586 | 1.67160206728868 | MAPK9//RGD1306565 |
| GO:0045295 | gamma-catenin binding | Molecular function | 2 | 11 | 302 | 14392 | 8.66465984346779 | 0.0213008989792895 | 0.50281332590586 | 1.67160206728868 | PTPRK//TCF7L2 |
| GO:0052689 | carboxylic ester hydrolase activity | Molecular function | 6 | 104 | 302 | 14392 | 2.7493632195619 | 0.0222433807382204 | 0.505161722612986 | 1.65279920430353 | RGD1308874//MGLL//CES1D//ABHD3//RGN//FAAH |
| GO:0032555 | purine ribonucleotide binding | Molecular function | 44 | 1549 | 302 | 14392 | 1.35367829704274 | 0.0228649442937563 | 0.505161722612986 | 1.64082985236087 | HSPA8//GLUL//MAPK10//DDR1//CCT4//PAK2//MAPK9//CAMKK1//NSF//APAF1//ACTB//PLK2//PRKCI//DDX1//PIK3CB//PAICS//ATP5B//DDX46//ACTG1//HSPH1//HSPA9//CCT5//CCT2//DNAJA4//HSP90AB1//TLK2//RGD1560691//MAP3K4//ILF2//DCLK3//OXSR1//DDX3X//NEK7//DDX50//SMC2//RGD1306565//MAGI1//EEF2//EIF5//ARF1//ARF4//DOCK9//SRPR//RAPGEF4 |
| GO:0032553 | ribonucleotide binding | Molecular function | 44 | 1550 | 302 | 14392 | 1.35280495620594 | 0.0230898892164241 | 0.505161722612986 | 1.63657815079054 | HSP90AB1//HSPA8//GLUL//MAPK10//DDR1//CCT4//PAK2//MAPK9//CAMKK1//NSF//APAF1//ACTB//PLK2//PRKCI//DDX1//PIK3CB//PAICS//ATP5B//DDX46//ACTG1//HSPH1//HSPA9//CCT5//CCT2//DNAJA4//TLK2//RGD1560691//MAP3K4//ILF2//DCLK3//OXSR1//DDX3X//NEK7//DDX50//SMC2//RGD1306565//MAGI1//EEF2//EIF5//ARF1//ARF4//DOCK9//SRPR//RAPGEF4 |
| GO:0035639 | purine ribonucleoside triphosphate binding | Molecular function | 43 | 1518 | 302 | 14392 | 1.34992888865621 | 0.0254004406880768 | 0.516888388398579 | 1.59515874846905 | HSPA8//GLUL//MAPK10//DDR1//CCT4//PAK2//MAPK9//CAMKK1//NSF//APAF1//ACTB//PLK2//PRKCI//DDX1//PIK3CB//PAICS//ATP5B//DDX46//ACTG1//HSPH1//HSPA9//CCT5//CCT2//DNAJA4//HSP90AB1//TLK2//RGD1560691//MAP3K4//ILF2//DCLK3//OXSR1//DDX3X//NEK7//DDX50//SMC2//RGD1306565//MAGI1//EEF2//EIF5//ARF1//ARF4//DOCK9//SRPR |
| GO:0016829 | lyase activity | Molecular function | 7 | 137 | 302 | 14392 | 2.43495915309131 | 0.025494634714303 | 0.516888388398579 | 1.59355120619892 | HADHA//AUH//GLUL//MGST2//PAICS//RGD1308874//UXS1 |
| GO:0042802 | identical protein binding | Molecular function | 26 | 827 | 302 | 14392 | 1.49824227039407 | 0.0258589503780321 | 0.516888388398579 | 1.58738910723134 | CAT//GSTM2//PLCB1//MGLL//FAAH//ADAM10//KIT//LRP4//SQSTM1//MGST1//OLFML2B//GOPC//SPPL2A//TRIM37//SUMF1//GLA//RGD1306565//GLUL//PAK2//ROBO1//APAF1//NELL2//CLDN11//PICALM//PAICS//ACTG1 |
| GO:0004364 | glutathione transferase activity | Molecular function | 3 | 31 | 302 | 14392 | 4.61183507797479 | 0.0266582975219929 | 0.516888388398579 | 1.57416758937126 | GSTM2//MGST1//MGST2 |
| GO:0008081 | phosphoric diester hydrolase activity | Molecular function | 5 | 81 | 302 | 14392 | 2.9417055024119 | 0.027653502084721 | 0.516888388398579 | 1.55824986111304 | CNP//PLCB1//PLCL1//DDHD2//GPCPD1 |
| GO:0004721 | phosphoprotein phosphatase activity | Molecular function | 7 | 140 | 302 | 14392 | 2.38278145695364 | 0.0282572145824292 | 0.516888388398579 | 1.54887065038204 | PPM1B//PPP3CA//PPP3CB//PTP4A1//MTMR6//PTPRK//PTPN3 |
| GO:0008134 | transcription factor binding | Molecular function | 14 | 378 | 302 | 14392 | 1.76502330144714 | 0.0288586725433512 | 0.516888388398579 | 1.53972364969286 | MKX//RBPJ//TAF1//ACTB//DNAJA3//EGR1//NFIA//YWHAZ//ZEB1//HIF1A//MAPK9//INSIG2//FOXN3//TCF7L2 |
| GO:0030971 | receptor tyrosine kinase binding | Molecular function | 3 | 32 | 302 | 14392 | 4.46771523178808 | 0.0289711363855859 | 0.516888388398579 | 1.53803446936959 | LRP4//DNAJA3//ELMO2 |
| GO:0030695 | GTPase regulator activity | Molecular function | 12 | 307 | 302 | 14392 | 1.86276074810708 | 0.0289941718888312 | 0.516888388398579 | 1.53768929075253 | GOPC//DNAJA3//RAPGEF4//DOCK9//RASGRF2//ARHGEF26//PLCB1//CHN1//TBC1D14//ACAP2//MADD//CHN2 |
| GO:0045502 | dynein binding | Molecular function | 2 | 13 | 302 | 14392 | 7.33163525216505 | 0.0293883030193172 | 0.516888388398579 | 1.53182549084553 | GLUL//ATMIN |
| GO:0005509 | calcium ion binding | Molecular function | 18 | 531 | 302 | 14392 | 1.61544505556179 | 0.031424517467127 | 0.540591283717622 | 1.50273138231259 | PLCB1//PPP3CB//RGN//CANX//CALR//NELL2//PLS3//LRP4//PLCL1//STIM2//ATP5B//SPTA1//FKBP9//USP32//CDH19//SLC25A13//RGD1304592//NCALD |
| GO:0016595 | glutamate binding | Molecular function | 2 | 14 | 302 | 14392 | 6.80794701986755 | 0.0338189242440038 | 0.540591283717622 | 1.47084021111804 | GLUL//SLC1A3 |
| GO:0060589 | nucleoside-triphosphatase regulator activity | Molecular function | 12 | 318 | 302 | 14392 | 1.79832562788954 | 0.036502404467143 | 0.540591283717622 | 1.43767852698412 | GOPC//DNAJA3//RAPGEF4//DOCK9//RASGRF2//ARHGEF26//PLCB1//CHN1//TBC1D14//ACAP2//MADD//CHN2 |
| GO:0004091 | carboxylesterase activity | Molecular function | 3 | 35 | 302 | 14392 | 4.08476821192053 | 0.0365243813019134 | 0.540591283717622 | 1.43741713198342 | MGLL//CES1D//ABHD3 |
| GO:0030145 | manganese ion binding | Molecular function | 3 | 35 | 302 | 14392 | 4.08476821192053 | 0.0365243813019134 | 0.540591283717622 | 1.43741713198342 | PPM1B//GLUL//GALNT1 |
| GO:0003729 | mRNA binding | Molecular function | 5 | 88 | 302 | 14392 | 2.70770620108368 | 0.0376352959392411 | 0.540591283717622 | 1.4244046646098 | SERBP1//AUH//DDX1//CALR//HNRNPA3 |
| GO:0008047 | enzyme activator activity | Molecular function | 11 | 284 | 302 | 14392 | 1.84581662158381 | 0.0376831878396252 | 0.540591283717622 | 1.42385236483359 | PLCB1//CHN1//TBC1D14//ACAP2//MAPK9//RGD1306565//APAF1//PAK2//CHN2//SPRY2//MGST2 |
| GO:0043566 | structure-specific DNA binding | Molecular function | 9 | 215 | 302 | 14392 | 1.99488680117049 | 0.0378789288525715 | 0.540591283717622 | 1.42160231055564 | EGR1//ZEB1//HIF1A//POLA1//FOXN3//HNRNPA1//CNBP//HNRNPK//HMGN5 |
| GO:0004602 | glutathione peroxidase activity | Molecular function | 2 | 15 | 302 | 14392 | 6.35408388520971 | 0.038490805421529 | 0.540591283717622 | 1.41464300118221 | MGST1//MGST2 |
| GO:0035035 | histone acetyltransferase binding | Molecular function | 2 | 15 | 302 | 14392 | 6.35408388520971 | 0.038490805421529 | 0.540591283717622 | 1.41464300118221 | EGR1//HIF1A |
| GO:0004722 | protein serine/threonine phosphatase activity | Molecular function | 3 | 36 | 302 | 14392 | 3.97130242825607 | 0.0392449055631736 | 0.540591283717622 | 1.40621671164227 | PPP3CA//PPP3CB//PPM1B |
| GO:0043028 | cysteine-type endopeptidase regulator activity involved in apoptotic process | Molecular function | 3 | 36 | 302 | 14392 | 3.97130242825607 | 0.0392449055631736 | 0.540591283717622 | 1.40621671164227 | MAPK9//RGD1306565//TRIAP1 |
| GO:0043169 | cation binding | Molecular function | 72 | 2827 | 302 | 14392 | 1.21372667068031 | 0.0396267388376923 | 0.540591283717622 | 1.40201166733455 | PPM1B//GLUL//OXSR1//RGD1306565//PLCB1//PPP3CB//RGN//CANX//CALR//NELL2//PLS3//LRP4//PLCL1//STIM2//ATP5B//SPTA1//FKBP9//USP32//CDH19//SLC25A13//RGD1304592//NCALD//PPP3CA//ADAM10//NT5E//NSF//CHN1//CHN2//POLA1//PHYH//NRP1//NPTX1//CNOT6//GSN//DNAJA4//CACNA2D3//PARN//NEK7//HDAC8//DDHD2//SC5DL//EGR1//ZEB1//CNBP//ADAMTS4//PRKCI//SQSTM1//PJA2//MRFAP1//PIAS1//ZFP445//NBR1//ZFP644//LIMCH1//LONRF1//ZFP828//ZBTB2//RNF141//PRICKLE2//RLF//ZFP280D//ZIC4//ARIH2//FUS//TRIM37//ACAP2//ZMAT2//SEC24C//CAT//FADS1//GALNT1//GLA |
| GO:0046872 | metal ion binding | Molecular function | 71 | 2783 | 302 | 14392 | 1.21579219147473 | 0.0397177162335826 | 0.540591283717622 | 1.40101573136667 | PPM1B//GLUL//OXSR1//RGD1306565//PLCB1//PPP3CB//RGN//CANX//CALR//NELL2//PLS3//LRP4//PLCL1//STIM2//ATP5B//SPTA1//FKBP9//USP32//CDH19//SLC25A13//RGD1304592//NCALD//SC5DL//NT5E//EGR1//ZEB1//ADAM10//CNBP//ADAMTS4//PRKCI//SQSTM1//PJA2//MRFAP1//PIAS1//ZFP445//NBR1//ZFP644//LIMCH1//LONRF1//ZFP828//ZBTB2//RNF141//PRICKLE2//RLF//ZFP280D//ZIC4//ARIH2//FUS//TRIM37//ACAP2//ZMAT2//SEC24C//CAT//FADS1//GALNT1//PPP3CA//NSF//CHN1//CHN2//POLA1//PHYH//NRP1//NPTX1//CNOT6//GSN//DNAJA4//CACNA2D3//PARN//NEK7//HDAC8//DDHD2 |
| GO:0003676 | nucleic acid binding | Molecular function | 63 | 2436 | 302 | 14392 | 1.23247316738982 | 0.0414052912223487 | 0.540591283717622 | 1.38294415641935 | EGR1//HNRNPK//RBPJ//NFIA//HIF1A//DDX1//POLA1//DMTF1//HDGF//MRF//EIF4G3//HNRPDL//RNF141//ILF2//TAF1//GLYR1//UPF2//HMG1L1//ZEB1//FOXN3//HNRNPA1//CNBP//FMR1//NCL//CNP//PDCD4//HNRNPM//HNRPH1//DDX46//CNOT6//CPSF3//HNRNPH2//PARN//AUH//DDX50//PUM1//SRSF1//CALR//HNRNPA3//SERBP1//EIF5//EIF5B//EIF3D//EEF2//RPL5//RGD1564051//TCF7L2//RPS4X//GAR1//HMGN5//MKX//PIAS1//ZFP445//ZFP644//ZFP828//ZBTB2//RLF//ZIC4//ARIH2//DDX3X//FUS//HNRNPH3//ZMAT2 |
| GO:0016830 | carbon-carbon lyase activity | Molecular function | 3 | 37 | 302 | 14392 | 3.8639699301951 | 0.0420654658197159 | 0.540591283717622 | 1.37607429748147 | GLUL//PAICS//UXS1 |
| GO:0019843 | rRNA binding | Molecular function | 3 | 37 | 302 | 14392 | 3.8639699301951 | 0.0420654658197159 | 0.540591283717622 | 1.37607429748147 | RPL5//RGD1564051//RPS4X |
| GO:0016209 | antioxidant activity | Molecular function | 4 | 63 | 302 | 14392 | 3.02575423105224 | 0.0431199114724866 | 0.540591283717622 | 1.36532213945049 | CAT//MGST1//MGST2//RGD1309676 |
| GO:0043167 | ion binding | Molecular function | 72 | 2840 | 302 | 14392 | 1.20817087958213 | 0.0432556077693059 | 0.540591283717622 | 1.36395758148492 | PPM1B//GLUL//OXSR1//RGD1306565//PLCB1//PPP3CB//RGN//CANX//CALR//NELL2//PLS3//LRP4//PLCL1//STIM2//ATP5B//SPTA1//FKBP9//USP32//CDH19//SLC25A13//RGD1304592//NCALD//PPP3CA//ADAM10//NT5E//NSF//CHN1//CHN2//POLA1//PHYH//NRP1//NPTX1//CNOT6//GSN//DNAJA4//CACNA2D3//PARN//NEK7//HDAC8//DDHD2//SC5DL//EGR1//ZEB1//CNBP//ADAMTS4//PRKCI//SQSTM1//PJA2//MRFAP1//PIAS1//ZFP445//NBR1//ZFP644//LIMCH1//LONRF1//ZFP828//ZBTB2//RNF141//PRICKLE2//RLF//ZFP280D//ZIC4//ARIH2//FUS//TRIM37//ACAP2//ZMAT2//SEC24C//CAT//FADS1//GALNT1//GLA |
| GO:0004707 | MAP kinase activity | Molecular function | 2 | 16 | 302 | 14392 | 5.95695364238411 | 0.0433919425057623 | 0.540591283717622 | 1.36259090758799 | MAPK10//MAPK9 |
| GO:0042805 | actinin binding | Molecular function | 2 | 16 | 302 | 14392 | 5.95695364238411 | 0.0433919425057623 | 0.540591283717622 | 1.36259090758799 | ITGB1//MAGI1 |
| GO:0015179 | L-amino acid transmembrane transporter activity | Molecular function | 3 | 38 | 302 | 14392 | 3.76228651097944 | 0.0449850944483479 | 0.548686919662488 | 1.34693136336189 | SLC1A3//SLC25A13//SERINC1 |
| GO:0001077 | RNA polymerase II core promoter proximal region sequence-specific DNA binding transcription factor activity involved in positive regulation of transcription | Molecular function | 4 | 64 | 302 | 14392 | 2.97847682119205 | 0.045265141644397 | 0.548686919662488 | 1.3442361162902 | EGR1//HIF1A//HNRNPK//RBPJ |
| GO:0001228 | RNA polymerase II transcription regulatory region sequence-specific DNA binding transcription factor activity involved in positive regulation of transcription | Molecular function | 4 | 66 | 302 | 14392 | 2.8882199478226 | 0.049731566682049 | 0.587127854510613 | 1.3033678590736 | EGR1//HIF1A//HNRNPK//RBPJ |
