# Supplementary material for: Gene Expression in the Hippocampus in a Rat Model of Premenstrual Dysphoric Disorder After Treatment With Baixiangdan Capsules
Source: Front Psychol. 2018 Nov 13;9:2065. doi: 10.3389/fpsyg.2018.02065 (PMC6242977; doi:10.3389/fpsyg.2018.02065)
Supplement: Supplementary file 3 [file Data_Sheet_3.ZIP › Data Analysis Folder/GO Analysis Report/BXD vs blank (up)/BP_result(Rat).html]

| GO.ID | Term | Ontology | Count | Pop.Hits | List.Total | Pop.Total | Fold.Enrichment | Pvalue | FDR | Enrichment.Score | GENES |
| --- | --- | --- | --- | --- | --- | --- | --- | --- | --- | --- | --- |
| GO:0008217 | regulation of blood pressure | Biological process | 12 | 148 | 147 | 13692 | 7.55212355212355 | 6.23845343752455e-08 | 0.000155563137808086 | 7.20492306204795 | CALCA//DRD2//CHRNA7//PCSK5//P2RX2//TACR1//NPY//HRH3//NPPA//POMC//TAC1//MYH6 |
| GO:0007218 | neuropeptide signaling pathway | Biological process | 9 | 72 | 147 | 13692 | 11.6428571428571 | 7.09685847664627e-08 | 0.000155563137808086 | 7.14893385525081 | CALCA//NPY//POMC//TAC1//TACR1//SSTR1//NMBR//GRP//NPW |
| GO:0048265 | response to pain | Biological process | 6 | 32 | 147 | 13692 | 17.4642857142857 | 9.95222830254788e-07 | 0.00145435229594566 | 6.00207966989205 | TACR1//SCN9A//P2RX2//CALCA//TAC1//KCNIP3 |
| GO:0001976 | neurological system process involved in regulation of systemic arterial blood pressure | Biological process | 5 | 19 | 147 | 13692 | 24.5112781954887 | 1.37244275921847e-06 | 0.00150419726410344 | 5.86250575971864 | CALCA//CHRNA7//P2RX2//TACR1//DRD2 |
| GO:0007610 | behavior | Biological process | 19 | 552 | 147 | 13692 | 3.20600414078675 | 7.04170639326467e-06 | 0.00504836223039161 | 5.15232208696866 | TACR1//FOSL1//HRH3//CHRNA7//ITGA8//DRD2//TAC1//FEZF2//CALCA//NPY//POMC//HTR2C//NPW//NTSR1//SCN9A//P2RX2//CDH13//CCL3//KCNIP3 |
| GO:0033555 | multicellular organismal response to stress | Biological process | 7 | 67 | 147 | 13692 | 9.73134328358209 | 7.232094867739e-06 | 0.00504836223039161 | 5.14073588559335 | CALCA//TAC1//TACR1//KCNIP3//SCN9A//P2RX2//ACP5 |
| GO:0007631 | feeding behavior | Biological process | 8 | 95 | 147 | 13692 | 7.84360902255639 | 8.06079735692091e-06 | 0.00504836223039161 | 5.09362199658408 | TACR1//NPY//HRH3//CALCA//DRD2//POMC//HTR2C//NPW |
| GO:0008015 | blood circulation | Biological process | 13 | 312 | 147 | 13692 | 3.88095238095238 | 3.13892305176476e-05 | 0.0158070443022374 | 4.50321933055786 | CALCA//DRD2//CHRNA7//PCSK5//MYH6//P2RX2//TACR1//NPPA//NPY//POMC//TAC1//HRH3//HTR2C |
| GO:0003013 | circulatory system process | Biological process | 13 | 313 | 147 | 13692 | 3.86855317206755 | 3.24505927737537e-05 | 0.0158070443022374 | 4.48877736555024 | CALCA//DRD2//CHRNA7//PCSK5//MYH6//P2RX2//TACR1//NPPA//NPY//POMC//TAC1//HRH3//HTR2C |
| GO:0006874 | cellular calcium ion homeostasis | Biological process | 11 | 246 | 147 | 13692 | 4.16492450638792 | 6.94201316951033e-05 | 0.0304337857351333 | 4.1585145667752 | CALCA//EPOR//TAC1//TACR1//CCL3//TRHR//HRH3//DRD2//HTR2C//CHRNA7//SYPL2 |
| GO:0055074 | calcium ion homeostasis | Biological process | 11 | 254 | 147 | 13692 | 4.03374578177728 | 9.24241124904187e-05 | 0.0309007801835257 | 4.03421471108068 | CALCA//CHRNA7//CCL3//SYPL2//EPOR//TAC1//TACR1//TRHR//HRH3//DRD2//HTR2C |
| GO:0006875 | cellular metal ion homeostasis | Biological process | 12 | 300 | 147 | 13692 | 3.72571428571429 | 9.43881835322061e-05 | 0.0309007801835257 | 4.02508237167621 | CALCA//CHRNA7//CCL3//SYPL2//EPOR//TAC1//TACR1//TRHR//HRH3//DRD2//HTR2C//MT4 |
| GO:0050886 | endocrine process | Biological process | 6 | 69 | 147 | 13692 | 8.09937888198758 | 9.50009749897367e-05 | 0.0309007801835257 | 4.0222719375481 | PCSK5//TACR1//INHA//TBX3//NKX3-1//TAC1 |
| GO:0007204 | elevation of cytosolic calcium ion concentration | Biological process | 9 | 172 | 147 | 13692 | 4.87375415282392 | 9.86794987612592e-05 | 0.0309007801835257 | 4.00577306515891 | CALCA//CCL3//DRD2//HTR2C//EPOR//TAC1//TACR1//TRHR//HRH3 |
| GO:0072503 | cellular divalent inorganic cation homeostasis | Biological process | 11 | 262 | 147 | 13692 | 3.91057797164667 | 0.000121662108155345 | 0.0335872288894067 | 3.91484466237333 | CALCA//CHRNA7//CCL3//SYPL2//EPOR//TAC1//TACR1//TRHR//HRH3//DRD2//HTR2C |
| GO:0002027 | regulation of heart rate | Biological process | 5 | 46 | 147 | 13692 | 10.1242236024845 | 0.000128134098209503 | 0.0335872288894067 | 3.89233528326341 | CHRNA7//TAC1//CALCA//DRD2//MYH6 |
| GO:0003073 | regulation of systemic arterial blood pressure | Biological process | 6 | 73 | 147 | 13692 | 7.65557729941292 | 0.000130242447791951 | 0.0335872288894067 | 3.88524745019637 | CALCA//DRD2//CHRNA7//PCSK5//P2RX2//TACR1 |
| GO:0072507 | divalent inorganic cation homeostasis | Biological process | 11 | 271 | 147 | 13692 | 3.78070637849236 | 0.00016365539130712 | 0.0392097364415439 | 3.78606968313712 | CALCA//CHRNA7//CCL3//SYPL2//EPOR//TAC1//TACR1//TRHR//HRH3//DRD2//HTR2C |
| GO:0055065 | metal ion homeostasis | Biological process | 12 | 321 | 147 | 13692 | 3.48197596795728 | 0.000178022323841344 | 0.0392097364415439 | 3.74952553413377 | DRD2//TAC1//TACR1//CALCA//CHRNA7//CCL3//SYPL2//MT4//EPOR//TRHR//HRH3//HTR2C |
| GO:0051480 | cytosolic calcium ion homeostasis | Biological process | 9 | 187 | 147 | 13692 | 4.48281130634072 | 0.000186030570603005 | 0.0392097364415439 | 3.73041568183987 | CALCA//EPOR//TAC1//TACR1//CCL3//TRHR//HRH3//DRD2//HTR2C |
| GO:0035815 | positive regulation of renal sodium excretion | Biological process | 3 | 11 | 147 | 13692 | 25.4025974025974 | 0.000187820361604111 | 0.0392097364415439 | 3.72625732765585 | DRD2//TAC1//TACR1 |
| GO:0007613 | memory | Biological process | 6 | 83 | 147 | 13692 | 6.73321858864028 | 0.000264498698389455 | 0.0527073769881532 | 3.57757646080712 | DRD2//TAC1//TACR1//CHRNA7//HRH3//ITGA8 |
| GO:0030003 | cellular cation homeostasis | Biological process | 12 | 337 | 147 | 13692 | 3.31665960152607 | 0.000278449814541405 | 0.0530749559543269 | 3.55525306703144 | CALCA//CHRNA7//CCL3//SYPL2//MT4//EPOR//TAC1//TACR1//TRHR//HRH3//DRD2//HTR2C |
| GO:0032652 | regulation of interleukin-1 production | Biological process | 4 | 32 | 147 | 13692 | 11.6428571428571 | 0.000362990485985915 | 0.0663062621067605 | 3.4401047577131 | CHRNA7//ACP5//CALCA//CCL3 |
| GO:0060986 | endocrine hormone secretion | Biological process | 4 | 33 | 147 | 13692 | 11.2900432900433 | 0.000409634972294542 | 0.0718335887415709 | 3.38760297286676 | INHA//TBX3//NKX3-1//TAC1 |
| GO:0032940 | secretion by cell | Biological process | 16 | 577 | 147 | 13692 | 2.58281752909136 | 0.000469725444224178 | 0.0792029364414922 | 3.32815591419005 | PCSK5//CCL3//RAB3C//CBLN4//HRH3//HTR2C//DRD2//CHRNA7//INHA//TACR1//RAB26//TBX3//LRP2//TNFSF13B//NKX3-1//TAC1 |
| GO:0048266 | behavioral response to pain | Biological process | 3 | 15 | 147 | 13692 | 18.6285714285714 | 0.000501848656573105 | 0.0814853522376479 | 3.29942723409995 | TACR1//SCN9A//P2RX2 |
| GO:0007267 | cell-cell signaling | Biological process | 18 | 715 | 147 | 13692 | 2.34485514485514 | 0.000650533497850012 | 0.0997299528657983 | 3.18673033544851 | DRD2//NPY//TAC1//HTR3A//P2RX2//RAB3C//CHRNA7//HRH3//HTR2C//INHA//TACR1//TBX3//SCN9A//LRP2//NKX3-1//POMC//BHLHA15//CCL3 |
| GO:0065008 | regulation of biological quality | Biological process | 38 | 2106 | 147 | 13692 | 1.68064034730701 | 0.000667375544576466 | 0.0997299528657983 | 3.17562971175088 | DRD2//TNFSF13B//COL2A1//SCX//CALCA//CHRNA7//PCSK5//MYH6//P2RX2//TACR1//TAC1//TMSB10//CCL3//SYPL2//MT4//EPOR//TRHR//HRH3//RAB3C//NPPA//NPY//POMC//HTR2C//INHA//WNT10B//SCN9A//HPSE//TBX3//CHRNE//DIO3//CHST8//AFP//BHLHA15//ACP5//TRPV2//LRP2//CAMP//NKX3-1 |
| GO:0007612 | learning | Biological process | 6 | 99 | 147 | 13692 | 5.64502164502164 | 0.000682458619063401 | 0.0997299528657983 | 3.16592367697184 | DRD2//TAC1//TACR1//CHRNA7//FOSL1//HRH3 |
| GO:0032612 | interleukin-1 production | Biological process | 4 | 39 | 147 | 13692 | 9.55311355311355 | 0.000783324441185337 | 0.110777237101823 | 3.10605832244715 | CHRNA7//ACP5//CALCA//CCL3 |
| GO:0007271 | synaptic transmission, cholinergic | Biological process | 3 | 18 | 147 | 13692 | 15.5238095238095 | 0.000879014498746281 | 0.117516973696468 | 3.05600396147506 | TAC1//TACR1//CHRNA7 |
| GO:0019233 | sensory perception of pain | Biological process | 6 | 104 | 147 | 13692 | 5.37362637362637 | 0.000884594008207904 | 0.117516973696468 | 3.05325600664746 | CALCA//HTR2C//CCL3//TAC1//TACR1//KCNIP3 |
| GO:0055080 | cation homeostasis | Biological process | 12 | 390 | 147 | 13692 | 2.86593406593407 | 0.00101709368572628 | 0.119955195456249 | 2.99263904184616 | DRD2//TAC1//TACR1//CALCA//CHRNA7//CCL3//SYPL2//MT4//EPOR//TRHR//HRH3//HTR2C |
| GO:0009190 | cyclic nucleotide biosynthetic process | Biological process | 6 | 107 | 147 | 13692 | 5.22296395193591 | 0.00102629313760749 | 0.119955195456249 | 2.98872857503093 | NPPA//HTR2C//CALCA//DRD2//HRH3//RAMP1 |
| GO:0035812 | renal sodium excretion | Biological process | 3 | 19 | 147 | 13692 | 14.7067669172932 | 0.00103565360039129 | 0.119955195456249 | 2.98478548068621 | DRD2//TAC1//TACR1 |
| GO:0035813 | regulation of renal sodium excretion | Biological process | 3 | 19 | 147 | 13692 | 14.7067669172932 | 0.00103565360039129 | 0.119955195456249 | 2.98478548068621 | DRD2//TAC1//TACR1 |
| GO:0045778 | positive regulation of ossification | Biological process | 4 | 42 | 147 | 13692 | 8.87074829931973 | 0.00103975762484887 | 0.119955195456249 | 2.98306788614202 | WNT10B//CALCA//TAC1//TACR1 |
| GO:0046903 | secretion | Biological process | 17 | 688 | 147 | 13692 | 2.3014950166113 | 0.00113453853314037 | 0.124368150809622 | 2.9451807492757 | ATP4B//PCSK5//DRD2//TAC1//TACR1//CCL3//RAB3C//CBLN4//HRH3//HTR2C//CHRNA7//INHA//RAB26//TBX3//LRP2//TNFSF13B//NKX3-1 |
| GO:0045776 | negative regulation of blood pressure | Biological process | 4 | 43 | 147 | 13692 | 8.66445182724252 | 0.00113692895818713 | 0.124368150809622 | 2.94426667166339 | CALCA//DRD2//NPY//HRH3 |
| GO:0006873 | cellular ion homeostasis | Biological process | 15 | 568 | 147 | 13692 | 2.45975855130785 | 0.00116311454908634 | 0.124368150809622 | 2.93437751176655 | CALCA//CHRNA7//CCL3//SYPL2//MT4//EPOR//TAC1//TACR1//TRHR//HRH3//SCN9A//P2RX2//DRD2//CHRNE//HTR2C |
| GO:0007186 | G-protein coupled receptor signaling pathway | Biological process | 32 | 1738 | 147 | 13692 | 1.71494328456354 | 0.00142148609466443 | 0.148376072357354 | 2.84725738433094 | HTR2C//DRD2//CALCA//HTR3A//RGS9//TAC1//TACR1//NPY//POMC//SSTR1//NMBR//GRP//NPW//RAMP1//RGS16//BHLHA15//OLR1401//OLR1513//OLR19//OLR56//OLR98//OLR200//OLR305//OLR375//OLR202//OLR857//OLR278//OLR1450//OLR606//OLR1138//OLR1585//GPR84 |
| GO:0010817 | regulation of hormone levels | Biological process | 11 | 354 | 147 | 13692 | 2.89426957223567 | 0.00152355153343494 | 0.155331393548344 | 2.81714285138032 | PCSK5//INHA//TACR1//TBX3//DIO3//CHST8//AFP//DRD2//LRP2//NKX3-1//TAC1 |
| GO:0044062 | regulation of excretion | Biological process | 3 | 22 | 147 | 13692 | 12.7012987012987 | 0.00160759327247502 | 0.160174747875693 | 2.79382381993029 | DRD2//TAC1//TACR1 |
| GO:0014070 | response to organic cyclic compound | Biological process | 11 | 358 | 147 | 13692 | 2.86193136472466 | 0.00166530197079142 | 0.162237418665546 | 2.77850700398503 | DRD2//TACR1//CHRNA7//HTR2C//HTR3A//TAC1//CCL3//WNT10B//FOSL1//ACP5//HRH3 |
| GO:0035094 | response to nicotine | Biological process | 4 | 50 | 147 | 13692 | 7.45142857142857 | 0.00200212566093807 | 0.189967904571557 | 2.69850866804585 | HTR2C//CHRNA7//DRD2//TACR1 |
| GO:0007166 | cell surface receptor signaling pathway | Biological process | 46 | 2870 | 147 | 13692 | 1.49288203086113 | 0.00206145115803737 | 0.189967904571557 | 2.68582695047996 | DRD2//WNT10B//NPPA//SSTR1//BHLHA15//RGS9//RAMP1//NPW//OLR1401//OLR1513//OLR19//OLR56//OLR98//OLR200//OLR305//OLR375//OLR202//OLR857//OLR278//OLR1450//OLR606//OLR1138//OLR1585//GPR84//HTR2C//CALCA//HTR3A//TAC1//TACR1//NPY//POMC//NMBR//GRP//GPC2//FRS3//MYH6//SCX//ITGA8//CCL3//P2RX2//RGS16//EPOR//CDH13//NKX3-1//EFNB1//AFP |
| GO:0045761 | regulation of adenylate cyclase activity | Biological process | 3 | 24 | 147 | 13692 | 11.6428571428571 | 0.00207994056100245 | 0.189967904571557 | 2.68194907580526 | CALCA//DRD2//HRH3 |
| GO:0032502 | developmental process | Biological process | 58 | 3887 | 147 | 13692 | 1.38983424602154 | 0.00238303433386191 | 0.210347008512488 | 2.622869700457 | COL2A1//TBX3//RAMP1//INHA//AFP//TEAD2//CCL3//NKX3-1//ITGA8//MYH6//CML1//SCX//EFNB1//KIRREL3//PCSK5//SPIC//RBBP8//CALCA//FREM2//CDH13//DRD2//SMARCD3//WNT10B//SEMA5B//SEMA4G//TMSB10//EPOR//RGS9//NTNG1//FEZF2//HRH3//NRL//NPPA//SRPK3//P2RX2//LRP2//ERCC5//SCN9A//MEGF11//SSTR1//NPY//GPC2//ACP5//KRT4//UPK2//HTR2C//NNMT//LCP1//RSPH9//RAB26//DKKL1//LOC680430//CHRNA7//CAMP//TRPV2//BHLHA15//HPSE//PRTN3 |
| GO:0007616 | long-term memory | Biological process | 3 | 26 | 147 | 13692 | 10.7472527472527 | 0.00263030153819258 | 0.210347008512488 | 2.57999446106674 | DRD2//TAC1//TACR1 |
| GO:0032720 | negative regulation of tumor necrosis factor production | Biological process | 3 | 26 | 147 | 13692 | 10.7472527472527 | 0.00263030153819258 | 0.210347008512488 | 2.57999446106674 | POMC//CHRNA7//ACP5 |
| GO:0008284 | positive regulation of cell proliferation | Biological process | 15 | 618 | 147 | 13692 | 2.26074895977809 | 0.00264568599105457 | 0.210347008512488 | 2.57746170226672 | CDH13//DRD2//SMARCD3//TNFSF13B//HPSE//EFNB1//TAC1//TACR1//HRH3//TBX3//CHRNA7//NKX3-1//WNT10B//CAMP//SCX |
| GO:2000021 | regulation of ion homeostasis | Biological process | 6 | 129 | 147 | 13692 | 4.33222591362126 | 0.00266587117946817 | 0.210347008512488 | 2.57416084044272 | DRD2//TAC1//TACR1//CALCA//CCL3//HTR2C |
| GO:0003001 | generation of a signal involved in cell-cell signaling | Biological process | 10 | 325 | 147 | 13692 | 2.86593406593407 | 0.00266730869921324 | 0.210347008512488 | 2.57392671861588 | RAB3C//HRH3//HTR2C//DRD2//INHA//TACR1//TBX3//LRP2//NKX3-1//TAC1 |
| GO:0023061 | signal release | Biological process | 10 | 325 | 147 | 13692 | 2.86593406593407 | 0.00266730869921324 | 0.210347008512488 | 2.57392671861588 | RAB3C//HRH3//HTR2C//DRD2//INHA//TACR1//TBX3//LRP2//NKX3-1//TAC1 |
| GO:0003008 | system process | Biological process | 42 | 2589 | 147 | 13692 | 1.51100811123986 | 0.00277101784604304 | 0.210347008512488 | 2.55736067724445 | ATP4B//DRD2//CALCA//CHRNA7//PCSK5//MYH6//P2RX2//TACR1//TAC1//NPY//HTR3A//RAB3C//COL2A1//RGS9//WNT10B//FOSL1//HRH3//ITGA8//NPPA//POMC//HTR2C//INHA//SCN9A//KCNIP3//TBX3//OLR1401//OLR1513//OLR19//OLR56//OLR98//OLR200//OLR305//OLR375//OLR202//OLR857//OLR278//OLR1450//OLR606//OLR1138//OLR1585//CCL3//NKX3-1 |
| GO:0055082 | cellular chemical homeostasis | Biological process | 15 | 624 | 147 | 13692 | 2.23901098901099 | 0.00289928395841708 | 0.210347008512488 | 2.53770924737894 | CALCA//CHRNA7//CCL3//SYPL2//MT4//EPOR//TAC1//TACR1//TRHR//HRH3//SCN9A//P2RX2//DRD2//CHRNE//HTR2C |
| GO:0032228 | regulation of synaptic transmission, GABAergic | Biological process | 3 | 27 | 147 | 13692 | 10.3492063492063 | 0.00293600313556293 | 0.210347008512488 | 2.53224348494229 | TAC1//TACR1//DRD2 |
| GO:0009653 | anatomical structure morphogenesis | Biological process | 32 | 1819 | 147 | 13692 | 1.63857692609754 | 0.00296334738149851 | 0.210347008512488 | 2.52821743491048 | COL2A1//RAMP1//TEAD2//CML1//SCX//NKX3-1//TBX3//FREM2//CDH13//SMARCD3//DRD2//NTNG1//EFNB1//FEZF2//CCL3//MEGF11//WNT10B//MYH6//AFP//HTR2C//LRP2//NNMT//LCP1//RSPH9//PCSK5//ITGA8//CHRNA7//CAMP//TRPV2//NRL//HPSE//ACP5 |
| GO:0050801 | ion homeostasis | Biological process | 15 | 626 | 147 | 13692 | 2.23185759926974 | 0.00298816346409923 | 0.210347008512488 | 2.52459564861886 | DRD2//TAC1//TACR1//CALCA//CHRNA7//CCL3//SYPL2//MT4//EPOR//TRHR//HRH3//SCN9A//P2RX2//CHRNE//HTR2C |
| GO:0009187 | cyclic nucleotide metabolic process | Biological process | 6 | 132 | 147 | 13692 | 4.23376623376623 | 0.00298936392624833 | 0.210347008512488 | 2.52442121057448 | NPPA//HTR2C//CALCA//DRD2//HRH3//RAMP1 |
| GO:0031100 | organ regeneration | Biological process | 5 | 92 | 147 | 13692 | 5.06211180124224 | 0.00309922879749308 | 0.210347008512488 | 2.50874636121769 | AFP//HTR2C//LRP2//NNMT//LCP1 |
| GO:0007611 | learning or memory | Biological process | 7 | 179 | 147 | 13692 | 3.64245810055866 | 0.00318713354177943 | 0.210347008512488 | 2.49659973908577 | FOSL1//HRH3//CHRNA7//ITGA8//DRD2//TAC1//TACR1 |
| GO:0043279 | response to alkaloid | Biological process | 6 | 134 | 147 | 13692 | 4.17057569296375 | 0.00322077510543044 | 0.210347008512488 | 2.49203959926614 | DRD2//TACR1//CHRNA7//HTR2C//HTR3A//TAC1 |
| GO:0007275 | multicellular organismal development | Biological process | 52 | 3429 | 147 | 13692 | 1.41249010540349 | 0.00323510279605921 | 0.210347008512488 | 2.49011191497921 | COL2A1//TBX3//RAMP1//INHA//AFP//TEAD2//NKX3-1//ITGA8//MYH6//CML1//SCX//EFNB1//KIRREL3//PCSK5//SPIC//RBBP8//CALCA//CDH13//DRD2//SMARCD3//WNT10B//EPOR//RGS9//NTNG1//FEZF2//HRH3//NRL//NPPA//SRPK3//P2RX2//ERCC5//SCN9A//MEGF11//SSTR1//NPY//GPC2//ACP5//CCL3//LRP2//HTR2C//NNMT//LCP1//RAB26//LOC680430//CHRNA7//CAMP//TRPV2//FREM2//HPSE//PRTN3//SEMA5B//SEMA4G |
| GO:0045744 | negative regulation of G-protein coupled receptor protein signaling pathway | Biological process | 4 | 57 | 147 | 13692 | 6.53634085213033 | 0.00324099303435178 | 0.210347008512488 | 2.48932190236669 | CALCA//DRD2//RGS9//RGS16 |
| GO:0032651 | regulation of interleukin-1 beta production | Biological process | 3 | 28 | 147 | 13692 | 9.97959183673469 | 0.00326268170137984 | 0.210347008512488 | 2.4864252927696 | CHRNA7//ACP5//CCL3 |
| GO:0042417 | dopamine metabolic process | Biological process | 3 | 28 | 147 | 13692 | 9.97959183673469 | 0.00326268170137984 | 0.210347008512488 | 2.4864252927696 | CHRNA7//HTR2C//DRD2 |
| GO:0051952 | regulation of amine transport | Biological process | 4 | 58 | 147 | 13692 | 6.42364532019704 | 0.00345245091294287 | 0.217147552997073 | 2.46187248751822 | HRH3//HTR2C//DRD2//CHRNA7 |
| GO:0072358 | cardiovascular system development | Biological process | 16 | 701 | 147 | 13692 | 2.12594253107805 | 0.00351676009643983 | 0.217147552997073 | 2.45385725703024 | RAMP1//TBX3//TEAD2//CALCA//CDH13//COL2A1//SMARCD3//SCX//EPOR//NPPA//PCSK5//NKX3-1//MYH6//CHRNA7//CAMP//HPSE |
| GO:0072359 | circulatory system development | Biological process | 16 | 701 | 147 | 13692 | 2.12594253107805 | 0.00351676009643983 | 0.217147552997073 | 2.45385725703024 | RAMP1//TBX3//TEAD2//CALCA//CDH13//COL2A1//SMARCD3//SCX//EPOR//NPPA//PCSK5//NKX3-1//MYH6//CHRNA7//CAMP//HPSE |
| GO:0044060 | regulation of endocrine process | Biological process | 3 | 29 | 147 | 13692 | 9.63546798029557 | 0.00361078181249623 | 0.219856492583104 | 2.44239875374758 | INHA//NKX3-1//TAC1 |
| GO:0008277 | regulation of G-protein coupled receptor protein signaling pathway | Biological process | 5 | 96 | 147 | 13692 | 4.85119047619048 | 0.00372305881882745 | 0.223587532352596 | 2.42910010251639 | CALCA//DRD2//RGS9//RGS16//RAMP1 |
| GO:0006816 | calcium ion transport | Biological process | 8 | 236 | 147 | 13692 | 3.15738498789346 | 0.00396034637753131 | 0.227422128256707 | 2.40226682839976 | BHLHA15//CALCA//CCL3//DRD2//HTR2C//TRPV2//CHRNA7//RAMP1 |
| GO:0043278 | response to morphine | Biological process | 3 | 30 | 147 | 13692 | 9.31428571428571 | 0.00398072658538458 | 0.227422128256707 | 2.40003765073455 | DRD2//TAC1//TACR1 |
| GO:0055078 | sodium ion homeostasis | Biological process | 3 | 30 | 147 | 13692 | 9.31428571428571 | 0.00398072658538458 | 0.227422128256707 | 2.40003765073455 | DRD2//TAC1//TACR1 |
| GO:0048856 | anatomical structure development | Biological process | 53 | 3546 | 147 | 13692 | 1.39215212311659 | 0.00399441238042117 | 0.227422128256707 | 2.39854710088338 | COL2A1//TBX3//RAMP1//INHA//AFP//TEAD2//NKX3-1//ITGA8//MYH6//CML1//SCX//EFNB1//KIRREL3//PCSK5//SPIC//RBBP8//CALCA//FREM2//CDH13//DRD2//SMARCD3//WNT10B//TMSB10//EPOR//RGS9//NTNG1//FEZF2//HRH3//NRL//NPPA//SRPK3//P2RX2//CCL3//MEGF11//SSTR1//NPY//GPC2//ACP5//KRT4//UPK2//LRP2//HTR2C//NNMT//LCP1//RSPH9//RAB26//LOC680430//CHRNA7//CAMP//TRPV2//BHLHA15//HPSE//PRTN3 |
| GO:0008306 | associative learning | Biological process | 4 | 61 | 147 | 13692 | 6.10772833723653 | 0.00414274169634414 | 0.232843328163753 | 2.38271214450754 | DRD2//TAC1//TACR1//CHRNA7 |
| GO:0008016 | regulation of heart contraction | Biological process | 5 | 99 | 147 | 13692 | 4.7041847041847 | 0.00424660187638744 | 0.235659526912437 | 2.37195845268785 | CHRNA7//MYH6//CALCA//DRD2//TAC1 |
| GO:0014072 | response to isoquinoline alkaloid | Biological process | 3 | 31 | 147 | 13692 | 9.01382488479263 | 0.00437291817832523 | 0.236677448071331 | 2.35922864866344 | DRD2//TAC1//TACR1 |
| GO:0031279 | regulation of cyclase activity | Biological process | 3 | 31 | 147 | 13692 | 9.01382488479263 | 0.00437291817832523 | 0.236677448071331 | 2.35922864866344 | CALCA//DRD2//HRH3 |
| GO:0050890 | cognition | Biological process | 7 | 191 | 147 | 13692 | 3.41361256544503 | 0.00454940147162187 | 0.24152807667131 | 2.34204573623741 | TACR1//FOSL1//HRH3//CHRNA7//ITGA8//DRD2//TAC1 |
| GO:0030432 | peristalsis | Biological process | 2 | 10 | 147 | 13692 | 18.6285714285714 | 0.00486892623495687 | 0.24152807667131 | 2.31256680503798 | DRD2//P2RX2 |
| GO:0032692 | negative regulation of interleukin-1 production | Biological process | 2 | 10 | 147 | 13692 | 18.6285714285714 | 0.00486892623495687 | 0.24152807667131 | 2.31256680503798 | CHRNA7//ACP5 |
| GO:0046541 | saliva secretion | Biological process | 2 | 10 | 147 | 13692 | 18.6285714285714 | 0.00486892623495687 | 0.24152807667131 | 2.31256680503798 | TAC1//TACR1 |
| GO:0046884 | follicle-stimulating hormone secretion | Biological process | 2 | 10 | 147 | 13692 | 18.6285714285714 | 0.00486892623495687 | 0.24152807667131 | 2.31256680503798 | INHA//TBX3 |
| GO:0051350 | negative regulation of lyase activity | Biological process | 2 | 10 | 147 | 13692 | 18.6285714285714 | 0.00486892623495687 | 0.24152807667131 | 2.31256680503798 | DRD2//HRH3 |
| GO:0060456 | positive regulation of digestive system process | Biological process | 2 | 10 | 147 | 13692 | 18.6285714285714 | 0.00486892623495687 | 0.24152807667131 | 2.31256680503798 | TAC1//TACR1 |
| GO:0009887 | organ morphogenesis | Biological process | 17 | 792 | 147 | 13692 | 1.9992784992785 | 0.00490328440322687 | 0.24152807667131 | 2.30951291581564 | COL2A1//TBX3//SCX//SMARCD3//TEAD2//MEGF11//AFP//HTR2C//LRP2//NNMT//LCP1//ITGA8//NRL//MYH6//WNT10B//ACP5//NKX3-1 |
| GO:0032501 | multicellular organismal process | Biological process | 78 | 5780 | 147 | 13692 | 1.25694513099357 | 0.00497956905263143 | 0.24256034140818 | 2.30280824080706 | COL2A1//TBX3//ACP5//RAMP1//INHA//AFP//TEAD2//CCL3//NKX3-1//ITGA8//DRD2//ATP4B//MYH6//CML1//SCX//EFNB1//KIRREL3//PCSK5//SPIC//RBBP8//CALCA//CHRNA7//CDH13//SMARCD3//WNT10B//P2RX2//TACR1//TAC1//NPY//HTR3A//RAB3C//SEMA5B//SEMA4G//SSTR1//TMSB10//EPOR//RGS9//NTNG1//FEZF2//HRH3//NRL//NPPA//SRPK3//FOSL1//POMC//ERCC5//NTSR1//SCN9A//HPSE//MEGF11//HTR2C//KCNIP3//GPC2//LRP2//NNMT//LCP1//RAB26//DIO3//LOC680430//CAMP//TRPV2//FREM2//OLR1401//OLR1513//OLR19//OLR56//OLR98//OLR200//OLR305//OLR375//OLR202//OLR857//OLR278//OLR1450//OLR606//OLR1138//OLR1585//PRTN3 |
| GO:0023052 | signaling | Biological process | 65 | 4621 | 147 | 13692 | 1.31016786719016 | 0.00512067928923763 | 0.246692945099096 | 2.29067242339934 | CCL3//CHRNA7//DRD2//P2RX2//WNT10B//EPOR//POMC//GRB7//SMOC2//ARHGAP8//RGD1564053//RGS9//PLEK2//NPPA//SSTR1//BHLHA15//RAMP1//NPW//OLR1401//OLR1513//OLR19//OLR56//OLR98//OLR200//OLR305//OLR375//OLR202//OLR857//OLR278//OLR1450//OLR606//OLR1138//OLR1585//GPR84//HTR2C//CALCA//HTR3A//TAC1//TACR1//NPY//NMBR//GRP//GPC2//RAB3C//RAB26//DOK3//CDH13//FRS3//HRH3//NKX3-1//INHA//SCN9A//MYH6//SCX//ITGA8//SLC7A3//TBX3//TEAD2//RGS16//TPD52L1//LRP2//EFNB1//HPSE//AFP//HRK |
| GO:0032640 | tumor necrosis factor production | Biological process | 4 | 66 | 147 | 13692 | 5.64502164502164 | 0.00549056494813863 | 0.256493168123229 | 2.26038296680019 | POMC//CHRNA7//ACP5//CCL3 |
| GO:0032680 | regulation of tumor necrosis factor production | Biological process | 4 | 66 | 147 | 13692 | 5.64502164502164 | 0.00549056494813863 | 0.256493168123229 | 2.26038296680019 | POMC//CHRNA7//ACP5//CCL3 |
| GO:0046879 | hormone secretion | Biological process | 7 | 198 | 147 | 13692 | 3.29292929292929 | 0.0055247622203637 | 0.256493168123229 | 2.25768640879329 | INHA//TACR1//TBX3//DRD2//NKX3-1//TAC1//LRP2 |
| GO:0032611 | interleukin-1 beta production | Biological process | 3 | 34 | 147 | 13692 | 8.21848739495798 | 0.00568669137787577 | 0.256493168123229 | 2.24514034068093 | CHRNA7//ACP5//CCL3 |
| GO:0051339 | regulation of lyase activity | Biological process | 3 | 34 | 147 | 13692 | 8.21848739495798 | 0.00568669137787577 | 0.256493168123229 | 2.24514034068093 | CALCA//DRD2//HRH3 |
| GO:0051932 | synaptic transmission, GABAergic | Biological process | 3 | 34 | 147 | 13692 | 8.21848739495798 | 0.00568669137787577 | 0.256493168123229 | 2.24514034068093 | DRD2//TAC1//TACR1 |
| GO:0048878 | chemical homeostasis | Biological process | 17 | 807 | 147 | 13692 | 1.96211718888299 | 0.00589575718831932 | 0.256493168123229 | 2.2294604108248 | DRD2//TAC1//TACR1//CALCA//CHRNA7//CCL3//SYPL2//MT4//EPOR//TRHR//HRH3//SCN9A//P2RX2//CHRNE//BHLHA15//NPPA//HTR2C |
| GO:0002031 | G-protein coupled receptor internalization | Biological process | 2 | 11 | 147 | 13692 | 16.9350649350649 | 0.00590917198459081 | 0.256493168123229 | 2.22847336983218 | CALCA//DRD2 |
| GO:0032230 | positive regulation of synaptic transmission, GABAergic | Biological process | 2 | 11 | 147 | 13692 | 16.9350649350649 | 0.00590917198459081 | 0.256493168123229 | 2.22847336983218 | TAC1//TACR1 |
| GO:0051797 | regulation of hair follicle development | Biological process | 2 | 11 | 147 | 13692 | 16.9350649350649 | 0.00590917198459081 | 0.256493168123229 | 2.22847336983218 | HPSE//WNT10B |
| GO:0007507 | heart development | Biological process | 10 | 365 | 147 | 13692 | 2.55185909980431 | 0.00602334843165504 | 0.257259266931166 | 2.22016201354065 | TBX3//COL2A1//SMARCD3//TEAD2//SCX//MYH6//EPOR//NPPA//PCSK5//NKX3-1 |
| GO:0018958 | phenol-containing compound metabolic process | Biological process | 4 | 68 | 147 | 13692 | 5.47899159663865 | 0.00610286582135977 | 0.257259266931166 | 2.21446617841038 | DIO3//CHRNA7//DRD2//HTR2C |
| GO:0071706 | tumor necrosis factor superfamily cytokine production | Biological process | 4 | 68 | 147 | 13692 | 5.47899159663865 | 0.00610286582135977 | 0.257259266931166 | 2.21446617841038 | POMC//CHRNA7//ACP5//CCL3 |
| GO:0007154 | cell communication | Biological process | 66 | 4745 | 147 | 13692 | 1.29555923528526 | 0.00623209481783479 | 0.260204796965597 | 2.20536594773774 | CCL3//CHRNA7//DRD2//P2RX2//WNT10B//EPOR//POMC//GRB7//SMOC2//ARHGAP8//RGD1564053//RGS9//PLEK2//NPPA//SSTR1//BHLHA15//RAMP1//NPW//OLR1401//OLR1513//OLR19//OLR56//OLR98//OLR200//OLR305//OLR375//OLR202//OLR857//OLR278//OLR1450//OLR606//OLR1138//OLR1585//GPR84//HTR2C//CALCA//HTR3A//TAC1//TACR1//NPY//NMBR//GRP//GPC2//RAB3C//RAB26//DOK3//CDH13//FRS3//HRH3//NKX3-1//INHA//SCN9A//MYH6//SCX//ITGA8//FOSL1//SLC7A3//TBX3//TEAD2//RGS16//TPD52L1//LRP2//EFNB1//HRK//HPSE//AFP |
| GO:0030817 | regulation of cAMP biosynthetic process | Biological process | 4 | 70 | 147 | 13692 | 5.32244897959184 | 0.00675899638949936 | 0.279541888410992 | 2.17011778554658 | CALCA//DRD2//HRH3//RAMP1 |
| GO:0007165 | signal transduction | Biological process | 60 | 4238 | 147 | 13692 | 1.31868131868132 | 0.00689540697299348 | 0.279848700357896 | 2.1614400962888 | CCL3//CHRNA7//P2RX2//DRD2//WNT10B//RGS9//PLEK2//NPPA//SSTR1//BHLHA15//RAMP1//NPW//OLR1401//OLR1513//OLR19//OLR56//OLR98//OLR200//OLR305//OLR375//OLR202//OLR857//OLR278//OLR1450//OLR606//OLR1138//OLR1585//GPR84//HTR2C//CALCA//HTR3A//TAC1//TACR1//NPY//POMC//NMBR//GRP//GPC2//RAB3C//RAB26//DOK3//CDH13//FRS3//NKX3-1//MYH6//SCX//ITGA8//SLC7A3//TEAD2//RGS16//EPOR//TPD52L1//EFNB1//HPSE//AFP//ARHGAP8//HRK//GRB7//SMOC2//RGD1564053 |
| GO:0051046 | regulation of secretion | Biological process | 11 | 432 | 147 | 13692 | 2.37169312169312 | 0.0068987271005119 | 0.279848700357896 | 2.16123103451216 | DRD2//TAC1//TACR1//HRH3//HTR2C//CHRNA7//INHA//RAB3C//RAB26//CCL3//NKX3-1 |
| GO:0032225 | regulation of synaptic transmission, dopaminergic | Biological process | 2 | 12 | 147 | 13692 | 15.5238095238095 | 0.00704131459943984 | 0.279848700357896 | 2.1523462513715 | DRD2//CHRNA7 |
| GO:0044057 | regulation of system process | Biological process | 12 | 495 | 147 | 13692 | 2.25800865800866 | 0.0070767997251382 | 0.279848700357896 | 2.1501630948424 | CHRNA7//MYH6//CALCA//DRD2//TAC1//TACR1//HTR2C//INHA//NPPA//SCN9A//CCL3//NKX3-1 |
| GO:0060402 | calcium ion transport into cytosol | Biological process | 4 | 71 | 147 | 13692 | 5.24748490945674 | 0.00710390105913002 | 0.279848700357896 | 2.14850309590247 | CALCA//CCL3//DRD2//HTR2C |
| GO:0009914 | hormone transport | Biological process | 7 | 208 | 147 | 13692 | 3.13461538461538 | 0.00718022904605319 | 0.279848700357896 | 2.14386170173954 | INHA//TACR1//TBX3//DRD2//LRP2//NKX3-1//TAC1 |
| GO:0050433 | regulation of catecholamine secretion | Biological process | 3 | 37 | 147 | 13692 | 7.55212355212355 | 0.00721325345356804 | 0.279848700357896 | 2.14186880764743 | DRD2//CHRNA7//HRH3 |
| GO:0045787 | positive regulation of cell cycle | Biological process | 5 | 113 | 147 | 13692 | 4.12136536030341 | 0.00739936418995197 | 0.284397375821914 | 2.13080559657424 | WNT10B//DRD2//NKX3-1//FOSL1//TBX3 |
| GO:0060401 | cytosolic calcium ion transport | Biological process | 4 | 72 | 147 | 13692 | 5.17460317460317 | 0.00746024138218981 | 0.284397375821914 | 2.1272471203481 | CALCA//CCL3//DRD2//HTR2C |
| GO:0030814 | regulation of cAMP metabolic process | Biological process | 4 | 73 | 147 | 13692 | 5.10371819960861 | 0.00782817078528322 | 0.293921820392408 | 2.10633970801442 | CALCA//DRD2//HRH3//RAMP1 |
| GO:0019725 | cellular homeostasis | Biological process | 15 | 696 | 147 | 13692 | 2.00738916256158 | 0.00784417267014409 | 0.293921820392408 | 2.10545285497552 | CALCA//CHRNA7//CCL3//SYPL2//MT4//EPOR//TAC1//TACR1//TRHR//HRH3//SCN9A//P2RX2//DRD2//CHRNE//HTR2C |
| GO:0042127 | regulation of cell proliferation | Biological process | 20 | 1042 | 147 | 13692 | 1.7877707704963 | 0.00791828334400081 | 0.294184357458471 | 2.1013689617232 | CDH13//DRD2//SMARCD3//CHRNA7//NKX3-1//WNT10B//CAMP//TBX3//SCX//FOSL1//FEZF2//BNIPL//TNFSF13B//HPSE//EFNB1//TACR1//TAC1//HRH3//KRT4//INHA |
| GO:0070838 | divalent metal ion transport | Biological process | 8 | 266 | 147 | 13692 | 2.80128893662728 | 0.00800643963093554 | 0.294522405996751 | 2.09656056661071 | CHRNA7//CCL3//RAMP1//BHLHA15//CALCA//DRD2//HTR2C//TRPV2 |
| GO:0030534 | adult behavior | Biological process | 5 | 116 | 147 | 13692 | 4.01477832512315 | 0.00824265113397378 | 0.294522405996751 | 2.08393308106087 | DRD2//NPY//NTSR1//HTR2C//CHRNA7 |
| GO:0007210 | serotonin receptor signaling pathway | Biological process | 2 | 13 | 147 | 13692 | 14.3296703296703 | 0.00826328830693439 | 0.294522405996751 | 2.08284709440241 | HTR2C//HTR3A |
| GO:0014051 | gamma-aminobutyric acid secretion | Biological process | 2 | 13 | 147 | 13692 | 14.3296703296703 | 0.00826328830693439 | 0.294522405996751 | 2.08284709440241 | HRH3//HTR2C |
| GO:0015812 | gamma-aminobutyric acid transport | Biological process | 2 | 13 | 147 | 13692 | 14.3296703296703 | 0.00826328830693439 | 0.294522405996751 | 2.08284709440241 | HRH3//HTR2C |
| GO:0072511 | divalent inorganic cation transport | Biological process | 8 | 269 | 147 | 13692 | 2.7700477960701 | 0.00853783435169791 | 0.301853756434223 | 2.06865227549148 | CHRNA7//CCL3//RAMP1//BHLHA15//CALCA//DRD2//HTR2C//TRPV2 |
| GO:0042592 | homeostatic process | Biological process | 21 | 1125 | 147 | 13692 | 1.73866666666667 | 0.00884888831188629 | 0.310348210874476 | 2.05311128640266 | DRD2//TNFSF13B//COL2A1//SCX//TAC1//TACR1//CALCA//CHRNA7//CCL3//SYPL2//MT4//EPOR//TRHR//HRH3//SCN9A//P2RX2//CHRNE//BHLHA15//ACP5//NPPA//HTR2C |
| GO:0071705 | nitrogen compound transport | Biological process | 6 | 166 | 147 | 13692 | 3.36660929432014 | 0.00901542855580801 | 0.311556549344769 | 2.04501362390565 | HRH3//HTR2C//DRD2//CHRNA7//SLC7A3//SLC25A42 |
| GO:0006939 | smooth muscle contraction | Biological process | 4 | 77 | 147 | 13692 | 4.83858998144712 | 0.00941874390771033 | 0.311556549344769 | 2.02600701125422 | P2RX2//DRD2//CALCA//TACR1 |
| GO:0032274 | gonadotropin secretion | Biological process | 2 | 14 | 147 | 13692 | 13.3061224489796 | 0.00957306057369369 | 0.311556549344769 | 2.01894919307286 | INHA//TBX3 |
| GO:0042634 | regulation of hair cycle | Biological process | 2 | 14 | 147 | 13692 | 13.3061224489796 | 0.00957306057369369 | 0.311556549344769 | 2.01894919307286 | HPSE//WNT10B |
| GO:0051955 | regulation of amino acid transport | Biological process | 2 | 14 | 147 | 13692 | 13.3061224489796 | 0.00957306057369369 | 0.311556549344769 | 2.01894919307286 | HRH3//HTR2C |
| GO:0006584 | catecholamine metabolic process | Biological process | 3 | 41 | 147 | 13692 | 6.81533101045296 | 0.00959285936139354 | 0.311556549344769 | 2.01805192257042 | CHRNA7//DRD2//HTR2C |
| GO:0009712 | catechol-containing compound metabolic process | Biological process | 3 | 41 | 147 | 13692 | 6.81533101045296 | 0.00959285936139354 | 0.311556549344769 | 2.01805192257042 | CHRNA7//DRD2//HTR2C |
| GO:0034311 | diol metabolic process | Biological process | 3 | 41 | 147 | 13692 | 6.81533101045296 | 0.00959285936139354 | 0.311556549344769 | 2.01805192257042 | CHRNA7//DRD2//HTR2C |
| GO:0050432 | catecholamine secretion | Biological process | 3 | 41 | 147 | 13692 | 6.81533101045296 | 0.00959285936139354 | 0.311556549344769 | 2.01805192257042 | DRD2//CHRNA7//HRH3 |
| GO:0008283 | cell proliferation | Biological process | 23 | 1280 | 147 | 13692 | 1.67366071428571 | 0.0095940087047317 | 0.311556549344769 | 2.01799989183059 | CALCA//CDH13//DRD2//SMARCD3//CHRNA7//NKX3-1//WNT10B//CAMP//TBX3//SCX//FOSL1//FEZF2//BNIPL//TNFSF13B//HPSE//EFNB1//INHA//TACR1//TAC1//HRH3//KRT4//DIO3//LRP2 |
| GO:0003015 | heart process | Biological process | 5 | 121 | 147 | 13692 | 3.84887839433294 | 0.00979264519314481 | 0.313364646180634 | 2.00909998055476 | CHRNA7//MYH6//CALCA//DRD2//TAC1 |
| GO:0060047 | heart contraction | Biological process | 5 | 121 | 147 | 13692 | 3.84887839433294 | 0.00979264519314481 | 0.313364646180634 | 2.00909998055476 | CHRNA7//MYH6//CALCA//DRD2//TAC1 |
| GO:0001503 | ossification | Biological process | 8 | 279 | 147 | 13692 | 2.67076292882745 | 0.0105015909576325 | 0.331215645742884 | 1.9787449017129 | CCL3//COL2A1//SCX//CALCA//WNT10B//TAC1//TACR1//ACP5 |
| GO:0031644 | regulation of neurological system process | Biological process | 8 | 279 | 147 | 13692 | 2.67076292882745 | 0.0105015909576325 | 0.331215645742884 | 1.9787449017129 | HTR2C//CALCA//TAC1//TACR1//CHRNA7//DRD2//SCN9A//CCL3 |
| GO:0014061 | regulation of norepinephrine secretion | Biological process | 2 | 15 | 147 | 13692 | 12.4190476190476 | 0.0109686316378661 | 0.336269098604231 | 1.9598475482859 | CHRNA7//HRH3 |
| GO:0042756 | drinking behavior | Biological process | 2 | 15 | 147 | 13692 | 12.4190476190476 | 0.0109686316378661 | 0.336269098604231 | 1.9598475482859 | TACR1//HRH3 |
| GO:0045671 | negative regulation of osteoclast differentiation | Biological process | 2 | 15 | 147 | 13692 | 12.4190476190476 | 0.0109686316378661 | 0.336269098604231 | 1.9598475482859 | CALCA//CCL3 |
| GO:0045885 | positive regulation of survival gene product expression | Biological process | 2 | 15 | 147 | 13692 | 12.4190476190476 | 0.0109686316378661 | 0.336269098604231 | 1.9598475482859 | CDH13//TNFSF13B |
| GO:0006171 | cAMP biosynthetic process | Biological process | 4 | 81 | 147 | 13692 | 4.59964726631393 | 0.0112062364453811 | 0.338814762596902 | 1.9505402183988 | CALCA//DRD2//HRH3//RAMP1 |
| GO:0046887 | positive regulation of hormone secretion | Biological process | 4 | 81 | 147 | 13692 | 4.59964726631393 | 0.0112062364453811 | 0.338814762596902 | 1.9505402183988 | DRD2//NKX3-1//TAC1//TACR1 |
| GO:0032101 | regulation of response to external stimulus | Biological process | 9 | 341 | 147 | 13692 | 2.45831587767072 | 0.011333410542564 | 0.340312820675346 | 1.94563937902194 | TAC1//HPSE//NPY//CALCA//CHRNA7//ACP5//CCL3//CDH13//DRD2 |
| GO:0007588 | excretion | Biological process | 3 | 44 | 147 | 13692 | 6.35064935064935 | 0.0116436495793591 | 0.346124942169702 | 1.93391087331573 | DRD2//TAC1//TACR1 |
| GO:0003014 | renal system process | Biological process | 4 | 82 | 147 | 13692 | 4.54355400696864 | 0.0116848748725173 | 0.346124942169702 | 1.93237593388932 | PCSK5//DRD2//TAC1//TACR1 |
| GO:0048243 | norepinephrine secretion | Biological process | 2 | 16 | 147 | 13692 | 11.6428571428571 | 0.0124480340475722 | 0.366256250097695 | 1.90489923248063 | CHRNA7//HRH3 |
| GO:0048731 | system development | Biological process | 45 | 3069 | 147 | 13692 | 1.3657310431504 | 0.0127872153148865 | 0.373318850062912 | 1.89322402198361 | COL2A1//TBX3//RAMP1//INHA//AFP//TEAD2//NKX3-1//ITGA8//EFNB1//KIRREL3//PCSK5//CALCA//SCX//CDH13//DRD2//SMARCD3//WNT10B//RGS9//NTNG1//FEZF2//EPOR//HRH3//NRL//NPPA//MYH6//SRPK3//P2RX2//MEGF11//SSTR1//NPY//GPC2//ACP5//CCL3//LRP2//HTR2C//NNMT//LCP1//RAB26//LOC680430//CHRNA7//CAMP//TRPV2//FREM2//HPSE//PRTN3 |
| GO:0031099 | regeneration | Biological process | 6 | 180 | 147 | 13692 | 3.1047619047619 | 0.0130785140528369 | 0.373318850062912 | 1.883441596632 | WNT10B//AFP//HTR2C//LRP2//NNMT//LCP1 |
| GO:0006029 | proteoglycan metabolic process | Biological process | 3 | 46 | 147 | 13692 | 6.07453416149068 | 0.0131402413161618 | 0.373318850062912 | 1.88139665902927 | CHST8//HPSE//COL2A1 |
| GO:0009408 | response to heat | Biological process | 4 | 85 | 147 | 13692 | 4.38319327731092 | 0.0131990013138119 | 0.373318850062912 | 1.87945892790846 | CALCA//TACR1//TRPV2//HSPB7 |
| GO:0030802 | regulation of cyclic nucleotide biosynthetic process | Biological process | 4 | 85 | 147 | 13692 | 4.38319327731092 | 0.0131990013138119 | 0.373318850062912 | 1.87945892790846 | CALCA//DRD2//HRH3//RAMP1 |
| GO:1900371 | regulation of purine nucleotide biosynthetic process | Biological process | 4 | 85 | 147 | 13692 | 4.38319327731092 | 0.0131990013138119 | 0.373318850062912 | 1.87945892790846 | CALCA//DRD2//HRH3//RAMP1 |
| GO:0030808 | regulation of nucleotide biosynthetic process | Biological process | 4 | 86 | 147 | 13692 | 4.33222591362126 | 0.0137301678127856 | 0.3814715055178 | 1.86232415469905 | CALCA//DRD2//HRH3//RAMP1 |
| GO:1900542 | regulation of purine nucleotide metabolic process | Biological process | 8 | 293 | 147 | 13692 | 2.54314968308142 | 0.0137904602989713 | 0.3814715055178 | 1.86042123766991 | CALCA//DRD2//HRH3//RAMP1//ARHGAP8//MYH6//RGS9//RGS16 |
| GO:0048513 | organ development | Biological process | 36 | 2341 | 147 | 13692 | 1.43235491548178 | 0.0138746686921467 | 0.3814715055178 | 1.85777737843162 | COL2A1//INHA//AFP//NKX3-1//ITGA8//EFNB1//PCSK5//TBX3//SCX//WNT10B//SMARCD3//TEAD2//EPOR//HRH3//NPPA//MYH6//SRPK3//P2RX2//MEGF11//FEZF2//SSTR1//KIRREL3//DRD2//NPY//ACP5//CCL3//LRP2//HTR2C//NNMT//LCP1//CALCA//LOC680430//NRL//FREM2//HPSE//PRTN3 |
| GO:0014821 | phasic smooth muscle contraction | Biological process | 2 | 17 | 147 | 13692 | 10.9579831932773 | 0.0140093322053754 | 0.3814715055178 | 1.85358256610128 | DRD2//P2RX2 |
| GO:0032098 | regulation of appetite | Biological process | 2 | 17 | 147 | 13692 | 10.9579831932773 | 0.0140093322053754 | 0.3814715055178 | 1.85358256610128 | NPY//POMC |
| GO:0051482 | elevation of cytosolic calcium ion concentration involved in phospholipase C-activating G-protein coupled signaling pathway | Biological process | 2 | 17 | 147 | 13692 | 10.9579831932773 | 0.0140093322053754 | 0.3814715055178 | 1.85358256610128 | CALCA//DRD2 |
| GO:0050673 | epithelial cell proliferation | Biological process | 7 | 239 | 147 | 13692 | 2.72803347280335 | 0.0146603485313114 | 0.396486999786832 | 1.83385570476815 | CALCA//CDH13//TACR1//HRH3//NKX3-1//WNT10B//KRT4 |
| GO:0007200 | phospholipase C-activating G-protein coupled receptor signaling pathway | Biological process | 3 | 48 | 147 | 13692 | 5.82142857142857 | 0.0147416471179867 | 0.396486999786832 | 1.831453989049 | HTR2C//CALCA//DRD2 |
| GO:0006140 | regulation of nucleotide metabolic process | Biological process | 8 | 299 | 147 | 13692 | 2.4921165790731 | 0.0154092612047955 | 0.405990097583425 | 1.81221818297997 | CALCA//DRD2//HRH3//RAMP1//ARHGAP8//MYH6//RGS9//RGS16 |
| GO:0015837 | amine transport | Biological process | 5 | 136 | 147 | 13692 | 3.42436974789916 | 0.0156203846180557 | 0.405990097583425 | 1.8063082767684 | HRH3//HTR2C//DRD2//CHRNA7//SLC7A3 |
| GO:0030803 | negative regulation of cyclic nucleotide biosynthetic process | Biological process | 2 | 18 | 147 | 13692 | 10.3492063492063 | 0.0156506219187041 | 0.405990097583425 | 1.80546839993895 | DRD2//HRH3 |
| GO:0030818 | negative regulation of cAMP biosynthetic process | Biological process | 2 | 18 | 147 | 13692 | 10.3492063492063 | 0.0156506219187041 | 0.405990097583425 | 1.80546839993895 | DRD2//HRH3 |
| GO:0032732 | positive regulation of interleukin-1 production | Biological process | 2 | 18 | 147 | 13692 | 10.3492063492063 | 0.0156506219187041 | 0.405990097583425 | 1.80546839993895 | CALCA//CCL3 |
| GO:0045684 | positive regulation of epidermis development | Biological process | 2 | 18 | 147 | 13692 | 10.3492063492063 | 0.0156506219187041 | 0.405990097583425 | 1.80546839993895 | HPSE//WNT10B |
| GO:0071347 | cellular response to interleukin-1 | Biological process | 3 | 51 | 147 | 13692 | 5.47899159663866 | 0.0173421111119025 | 0.427810175998725 | 1.76089803555402 | CCL3//NKX3-1//CAMP |
| GO:0002029 | desensitization of G-protein coupled receptor protein signaling pathway | Biological process | 2 | 19 | 147 | 13692 | 9.80451127819549 | 0.0173700299561526 | 0.427810175998725 | 1.76019943257302 | CALCA//DRD2 |
| GO:0010634 | positive regulation of epithelial cell migration | Biological process | 2 | 19 | 147 | 13692 | 9.80451127819549 | 0.0173700299561526 | 0.427810175998725 | 1.76019943257302 | TAC1//TACR1 |
| GO:0015874 | norepinephrine transport | Biological process | 2 | 19 | 147 | 13692 | 9.80451127819549 | 0.0173700299561526 | 0.427810175998725 | 1.76019943257302 | CHRNA7//HRH3 |
| GO:0022401 | negative adaptation of signaling pathway | Biological process | 2 | 19 | 147 | 13692 | 9.80451127819549 | 0.0173700299561526 | 0.427810175998725 | 1.76019943257302 | CALCA//DRD2 |
| GO:0030800 | negative regulation of cyclic nucleotide metabolic process | Biological process | 2 | 19 | 147 | 13692 | 9.80451127819549 | 0.0173700299561526 | 0.427810175998725 | 1.76019943257302 | DRD2//HRH3 |
| GO:0030809 | negative regulation of nucleotide biosynthetic process | Biological process | 2 | 19 | 147 | 13692 | 9.80451127819549 | 0.0173700299561526 | 0.427810175998725 | 1.76019943257302 | DRD2//HRH3 |
| GO:0030815 | negative regulation of cAMP metabolic process | Biological process | 2 | 19 | 147 | 13692 | 9.80451127819549 | 0.0173700299561526 | 0.427810175998725 | 1.76019943257302 | DRD2//HRH3 |
| GO:1900372 | negative regulation of purine nucleotide biosynthetic process | Biological process | 2 | 19 | 147 | 13692 | 9.80451127819549 | 0.0173700299561526 | 0.427810175998725 | 1.76019943257302 | DRD2//HRH3 |
| GO:0007600 | sensory perception | Biological process | 27 | 1660 | 147 | 13692 | 1.51497418244406 | 0.0175737337599436 | 0.430409211193256 | 1.75513595739665 | COL2A1//RGS9//NPY//P2RX2//WNT10B//DRD2//TAC1//TACR1//KCNIP3//OLR1401//OLR1513//OLR19//OLR56//OLR98//OLR200//OLR305//OLR375//OLR202//OLR857//OLR278//OLR1450//OLR606//OLR1138//OLR1585//CALCA//HTR2C//CCL3 |
| GO:0030799 | regulation of cyclic nucleotide metabolic process | Biological process | 4 | 93 | 147 | 13692 | 4.00614439324117 | 0.0178296713292862 | 0.434251550597726 | 1.74885666250008 | CALCA//DRD2//HRH3//RAMP1 |
| GO:0045638 | negative regulation of myeloid cell differentiation | Biological process | 3 | 52 | 147 | 13692 | 5.37362637362637 | 0.0182621417408468 | 0.44232723420924 | 1.73844829078383 | LOC680430//CALCA//CCL3 |
| GO:0050877 | neurological system process | Biological process | 34 | 2227 | 147 | 13692 | 1.42202835332606 | 0.0187579595945316 | 0.450329922355968 | 1.7268144039649 | DRD2//CALCA//CHRNA7//P2RX2//TACR1//NPY//TAC1//HTR3A//RAB3C//COL2A1//RGS9//WNT10B//FOSL1//HRH3//ITGA8//HTR2C//SCN9A//KCNIP3//OLR1401//OLR1513//OLR19//OLR56//OLR98//OLR200//OLR305//OLR375//OLR202//OLR857//OLR278//OLR1450//OLR606//OLR1138//OLR1585//CCL3 |
| GO:0048747 | muscle fiber development | Biological process | 4 | 95 | 147 | 13692 | 3.9218045112782 | 0.0191264073792559 | 0.450329922355968 | 1.71836659828903 | P2RX2//TBX3//MYH6//WNT10B |
| GO:0050671 | positive regulation of lymphocyte proliferation | Biological process | 4 | 95 | 147 | 13692 | 3.9218045112782 | 0.0191264073792559 | 0.450329922355968 | 1.71836659828903 | TNFSF13B//EFNB1//TAC1//TACR1 |
| GO:0002052 | positive regulation of neuroblast proliferation | Biological process | 2 | 20 | 147 | 13692 | 9.31428571428571 | 0.0191657136095875 | 0.450329922355968 | 1.71747500573724 | DRD2//SMARCD3 |
| GO:0031623 | receptor internalization | Biological process | 3 | 53 | 147 | 13692 | 5.2722371967655 | 0.0192088721442897 | 0.450329922355968 | 1.71649813414357 | CALCA//DRD2//RAMP1 |
| GO:0051937 | catecholamine transport | Biological process | 3 | 53 | 147 | 13692 | 5.2722371967655 | 0.0192088721442897 | 0.450329922355968 | 1.71649813414357 | DRD2//CHRNA7//HRH3 |
| GO:0051240 | positive regulation of multicellular organismal process | Biological process | 11 | 502 | 147 | 13692 | 2.04097894137735 | 0.0194509151193965 | 0.45181604757553 | 1.71105996133288 | CHRNA7//DRD2//TAC1//TACR1//CALCA//HPSE//WNT10B//CCL3//SCX//DIO3//HTR2C |
| GO:0051234 | establishment of localization | Biological process | 40 | 2726 | 147 | 13692 | 1.36673304685044 | 0.0194783834379049 | 0.45181604757553 | 1.71044708919918 | GOLGA1//ATP4B//PCSK5//DRD2//CALCA//TAC1//TACR1//RAB3C//RAB26//AP1S2//AFP//LOC360919//CHRNA7//P2RX2//KCNN3//SCN11A//CCL3//RAMP1//CLCNKB//BHLHA15//KCNIP3//LRP2//CBLN4//HRH3//HTR2C//SLC2A5//SLC7A3//SLC6A5//SLC25A42//INHA//CDH13//TBX3//LCP1//ITGA8//TNFSF13B//PRTN3//SLC16A10//SLC24A4//TRPV2//NKX3-1 |
| GO:0032946 | positive regulation of mononuclear cell proliferation | Biological process | 4 | 96 | 147 | 13692 | 3.88095238095238 | 0.019796118186231 | 0.456769379623351 | 1.70341996204038 | TNFSF13B//EFNB1//TAC1//TACR1 |
| GO:0001508 | regulation of action potential | Biological process | 5 | 145 | 147 | 13692 | 3.21182266009852 | 0.0200424414351727 | 0.460031744773807 | 1.69804937675482 | CHRNA7//SCN9A//P2RX2//TAC1//TACR1 |
| GO:0046058 | cAMP metabolic process | Biological process | 4 | 97 | 147 | 13692 | 3.84094256259205 | 0.0204801614616715 | 0.462012059805751 | 1.68866662378817 | CALCA//DRD2//HRH3//RAMP1 |
| GO:0051049 | regulation of transport | Biological process | 17 | 926 | 147 | 13692 | 1.70996605985807 | 0.0209318884870454 | 0.462012059805751 | 1.67919158759518 | DRD2//TAC1//TACR1//CALCA//HRH3//HTR2C//CHRNA7//INHA//RAB3C//RAB26//CDH13//LCP1//SCN11A//CCL3//PRTN3//TRPV2//NKX3-1 |
| GO:0002548 | monocyte chemotaxis | Biological process | 2 | 21 | 147 | 13692 | 8.87074829931973 | 0.0210358602619861 | 0.462012059805751 | 1.67703972280106 | CALCA//CCL3 |
| GO:0023058 | adaptation of signaling pathway | Biological process | 2 | 21 | 147 | 13692 | 8.87074829931973 | 0.0210358602619861 | 0.462012059805751 | 1.67703972280106 | CALCA//DRD2 |
| GO:1900543 | negative regulation of purine nucleotide metabolic process | Biological process | 2 | 21 | 147 | 13692 | 8.87074829931973 | 0.0210358602619861 | 0.462012059805751 | 1.67703972280106 | DRD2//HRH3 |
| GO:0051649 | establishment of localization in cell | Biological process | 22 | 1299 | 147 | 13692 | 1.57747718024854 | 0.0210873465339418 | 0.462012059805751 | 1.67597806502901 | GOLGA1//PCSK5//BHLHA15//RAMP1//KCNIP3//AP1S2//CCL3//RAB3C//CBLN4//HRH3//HTR2C//DRD2//CHRNA7//LRP2//INHA//TACR1//RAB26//TBX3//LCP1//TNFSF13B//NKX3-1//TAC1 |
| GO:0030316 | osteoclast differentiation | Biological process | 3 | 55 | 147 | 13692 | 5.08051948051948 | 0.0211825784719334 | 0.462012059805751 | 1.67402117605222 | CALCA//CCL3//ACP5 |
| GO:0051209 | release of sequestered calcium ion into cytosol | Biological process | 3 | 55 | 147 | 13692 | 5.08051948051948 | 0.0211825784719334 | 0.462012059805751 | 1.67402117605222 | CCL3//DRD2//HTR2C |
| GO:0051282 | regulation of sequestering of calcium ion | Biological process | 3 | 55 | 147 | 13692 | 5.08051948051948 | 0.0211825784719334 | 0.462012059805751 | 1.67402117605222 | CCL3//DRD2//HTR2C |
| GO:0051283 | negative regulation of sequestering of calcium ion | Biological process | 3 | 55 | 147 | 13692 | 5.08051948051948 | 0.0211825784719334 | 0.462012059805751 | 1.67402117605222 | CCL3//DRD2//HTR2C |
| GO:0009306 | protein secretion | Biological process | 5 | 148 | 147 | 13692 | 3.14671814671815 | 0.0216813532248151 | 0.470549765037571 | 1.66391361513507 | PCSK5//TNFSF13B//DRD2//CCL3//CBLN4 |
| GO:0043491 | protein kinase B signaling cascade | Biological process | 4 | 99 | 147 | 13692 | 3.76334776334776 | 0.0218915378183329 | 0.472770944805771 | 1.65972372940705 | CCL3//HPSE//DRD2//NKX3-1 |
| GO:0007268 | synaptic transmission | Biological process | 10 | 446 | 147 | 13692 | 2.08840486867393 | 0.0220557497014503 | 0.473982385740971 | 1.65647817544308 | DRD2//RAB3C//CHRNA7//P2RX2//HTR2C//TAC1//TACR1//SCN9A//NPY//HTR3A |
| GO:0051208 | sequestering of calcium ion | Biological process | 3 | 56 | 147 | 13692 | 4.98979591836735 | 0.0222095994461256 | 0.474354375382814 | 1.65345927395517 | CCL3//DRD2//HTR2C |
| GO:0006164 | purine nucleotide biosynthetic process | Biological process | 6 | 203 | 147 | 13692 | 2.75299085151302 | 0.0222894619819479 | 0.474354375382814 | 1.6519004142964 | NPPA//HTR2C//CALCA//DRD2//HRH3//RAMP1 |
| GO:0070665 | positive regulation of leukocyte proliferation | Biological process | 4 | 100 | 147 | 13692 | 3.72571428571429 | 0.0226190090302899 | 0.479042200912034 | 1.64552642602879 | TNFSF13B//EFNB1//TAC1//TACR1 |
| GO:0051496 | positive regulation of stress fiber assembly | Biological process | 2 | 22 | 147 | 13692 | 8.46753246753247 | 0.0229786869609372 | 0.484320017484369 | 1.63867479122265 | TAC1//TACR1 |
| GO:0050678 | regulation of epithelial cell proliferation | Biological process | 6 | 205 | 147 | 13692 | 2.72613240418118 | 0.0232545722748255 | 0.487690543552349 | 1.63349164412887 | CDH13//TACR1//HRH3//NKX3-1//WNT10B//KRT4 |
| GO:0006865 | amino acid transport | Biological process | 4 | 101 | 147 | 13692 | 3.68882602545969 | 0.0233610889931554 | 0.487690543552349 | 1.63150691615318 | HRH3//HTR2C//SLC7A3//SLC6A5 |
| GO:0006810 | transport | Biological process | 39 | 2679 | 147 | 13692 | 1.35594304911214 | 0.0238347375938608 | 0.495220329912255 | 1.62278962503191 | GOLGA1//ATP4B//PCSK5//DRD2//CALCA//TAC1//TACR1//RAB3C//RAB26//AP1S2//CHRNA7//P2RX2//KCNN3//SCN11A//CCL3//RAMP1//CLCNKB//BHLHA15//KCNIP3//LRP2//CBLN4//HRH3//HTR2C//SLC2A5//SLC7A3//SLC6A5//SLC25A42//INHA//CDH13//TBX3//LCP1//TNFSF13B//PRTN3//SLC16A10//SLC24A4//TRPV2//NKX3-1//AFP//LOC360919 |
| GO:0060341 | regulation of cellular localization | Biological process | 12 | 587 | 147 | 13692 | 1.90411292285228 | 0.0242439482507055 | 0.501346552505155 | 1.61539665167312 | HRH3//HTR2C//DRD2//CHRNA7//INHA//TACR1//RAB3C//RAB26//LCP1//CCL3//NKX3-1//TAC1 |
| GO:0001958 | endochondral ossification | Biological process | 2 | 23 | 147 | 13692 | 8.09937888198758 | 0.0249924399977328 | 0.507253967361392 | 1.60219134187989 | COL2A1//SCX |
| GO:0036075 | replacement ossification | Biological process | 2 | 23 | 147 | 13692 | 8.09937888198758 | 0.0249924399977328 | 0.507253967361392 | 1.60219134187989 | COL2A1//SCX |
| GO:0051954 | positive regulation of amine transport | Biological process | 2 | 23 | 147 | 13692 | 8.09937888198758 | 0.0249924399977328 | 0.507253967361392 | 1.60219134187989 | HTR2C//DRD2 |
| GO:0070723 | response to cholesterol | Biological process | 2 | 23 | 147 | 13692 | 8.09937888198758 | 0.0249924399977328 | 0.507253967361392 | 1.60219134187989 | CCL3//ACP5 |
| GO:0046883 | regulation of hormone secretion | Biological process | 5 | 154 | 147 | 13692 | 3.02411873840445 | 0.0252156438636363 | 0.508149838999522 | 1.59832993796076 | INHA//TACR1//DRD2//NKX3-1//TAC1 |
| GO:0072522 | purine-containing compound biosynthetic process | Biological process | 6 | 209 | 147 | 13692 | 2.67395762132604 | 0.0252683998407609 | 0.508149838999522 | 1.59742225955738 | NPPA//HTR2C//CALCA//DRD2//HRH3//RAMP1 |
| GO:0022600 | digestive system process | Biological process | 3 | 59 | 147 | 13692 | 4.73607748184019 | 0.0254513786274562 | 0.509355506316403 | 1.59428868821599 | ATP4B//TAC1//TACR1 |
| GO:0040008 | regulation of growth | Biological process | 10 | 457 | 147 | 13692 | 2.03813691778681 | 0.0255607234009144 | 0.509355506316403 | 1.59242685925465 | CDH13//IGFBPL1//NPPA//CAMP//BNIPL//DRD2//DIO3//TRPV2//WNT10B//MYH6 |
| GO:0065007 | biological regulation | Biological process | 98 | 8017 | 147 | 13692 | 1.13858051640264 | 0.0262017304941233 | 0.519766454688853 | 1.58167002474017 | KCNIP3//RBBP8//FEZF2//WNT10B//TBX3//CCL3//CHRNA7//CDH13//IGFBPL1//DRD2//TNFSF13B//KRT1//COL2A1//SCX//NKX3-1//CAMP//CALCA//PCSK5//MYH6//SMARCD3//TAC1//TACR1//P2RX2//INHA//TMSB10//EPOR//POMC//GRB7//SMOC2//ARHGAP8//RGD1564053//RGS9//PLEK2//NRL//RFX2//TEAD2//SYPL2//MT4//BNIPL//TPD52L1//ACP5//NPPA//SSTR1//BHLHA15//RAMP1//NPW//OLR1401//OLR1513//OLR19//OLR56//OLR98//OLR200//OLR305//OLR375//OLR202//OLR857//OLR278//OLR1450//OLR606//OLR1138//OLR1585//GPR84//HTR2C//HRH3//TRHR//HTR3A//NPY//NMBR//GRP//GPC2//RAB3C//RAB26//DOK3//FOSL1//FRS3//PLAGL1//HPSE//LRP2//LOC680319//SCN9A//ITGA8//SLC7A3//HRK//LCP1//SCN11A//RGS16//DIO3//EFNB1//CHRNE//CHST8//AFP//ERCC5//DKKL1//LOC680430//TRPV2//SPIC//KRT4//PRTN3 |
| GO:0045884 | regulation of survival gene product expression | Biological process | 2 | 24 | 147 | 13692 | 7.76190476190476 | 0.027075394491981 | 0.52829579571526 | 1.56742520693007 | CDH13//TNFSF13B |
| GO:0045980 | negative regulation of nucleotide metabolic process | Biological process | 2 | 24 | 147 | 13692 | 7.76190476190476 | 0.027075394491981 | 0.52829579571526 | 1.56742520693007 | DRD2//HRH3 |
| GO:0030278 | regulation of ossification | Biological process | 5 | 157 | 147 | 13692 | 2.9663330300273 | 0.0271137212673206 | 0.52829579571526 | 1.56681087286754 | CALCA//WNT10B//CCL3//TAC1//TACR1 |
| GO:0048871 | multicellular organismal homeostasis | Biological process | 5 | 157 | 147 | 13692 | 2.9663330300273 | 0.0271137212673206 | 0.52829579571526 | 1.56681087286754 | DRD2//COL2A1//SCX//ACP5//CALCA |
| GO:0007565 | female pregnancy | Biological process | 5 | 158 | 147 | 13692 | 2.94755877034358 | 0.0277661172866966 | 0.538613531791495 | 1.55648484617181 | CALCA//PCSK5//EPOR//NPPA//FOSL1 |
| GO:0001963 | synaptic transmission, dopaminergic | Biological process | 2 | 25 | 147 | 13692 | 7.45142857142857 | 0.0292258539816721 | 0.561956771296713 | 1.53423278986298 | CHRNA7//DRD2 |
| GO:0010632 | regulation of epithelial cell migration | Biological process | 2 | 25 | 147 | 13692 | 7.45142857142857 | 0.0292258539816721 | 0.561956771296713 | 1.53423278986298 | TAC1//TACR1 |
| GO:0006898 | receptor-mediated endocytosis | Biological process | 4 | 109 | 147 | 13692 | 3.41808650065531 | 0.0298303041049021 | 0.571074468104327 | 1.52534231920221 | CALCA//DRD2//RAMP1//LRP2 |
| GO:0051238 | sequestering of metal ion | Biological process | 3 | 63 | 147 | 13692 | 4.43537414965986 | 0.0301479549374283 | 0.574646236720372 | 1.52074214254582 | CCL3//DRD2//HTR2C |
| GO:0001894 | tissue homeostasis | Biological process | 4 | 110 | 147 | 13692 | 3.38701298701299 | 0.0307062309166024 | 0.581613441694803 | 1.51277348842762 | ACP5//CALCA//COL2A1//SCX |
| GO:0048646 | anatomical structure formation involved in morphogenesis | Biological process | 14 | 750 | 147 | 13692 | 1.73866666666667 | 0.0308285298863237 | 0.581613441694803 | 1.51104718489011 | RAMP1//SCX//NKX3-1//TEAD2//CDH13//SMARCD3//MEGF11//MYH6//RSPH9//CHRNA7//CAMP//HPSE//WNT10B//TBX3 |
| GO:0003007 | heart morphogenesis | Biological process | 5 | 163 | 147 | 13692 | 2.85714285714286 | 0.0311777279677251 | 0.581613441694803 | 1.50615553658246 | TBX3//SMARCD3//TEAD2//MYH6//COL2A1 |
| GO:0050909 | sensory perception of taste | Biological process | 3 | 64 | 147 | 13692 | 4.36607142857143 | 0.031388636421611 | 0.581613441694803 | 1.5032275504176 | NPY//P2RX2//WNT10B |
| GO:0032233 | positive regulation of actin filament bundle assembly | Biological process | 2 | 26 | 147 | 13692 | 7.16483516483517 | 0.0314421500186287 | 0.581613441694803 | 1.50248776449965 | TAC1//TACR1 |
| GO:0044058 | regulation of digestive system process | Biological process | 2 | 26 | 147 | 13692 | 7.16483516483517 | 0.0314421500186287 | 0.581613441694803 | 1.50248776449965 | TAC1//TACR1 |
| GO:0050850 | positive regulation of calcium-mediated signaling | Biological process | 2 | 26 | 147 | 13692 | 7.16483516483517 | 0.0314421500186287 | 0.581613441694803 | 1.50248776449965 | CCL3//CDH13 |
| GO:0030001 | metal ion transport | Biological process | 10 | 474 | 147 | 13692 | 1.96503918022905 | 0.0317425151647781 | 0.584702464211711 | 1.49835866425706 | DRD2//KCNN3//SCN11A//CHRNA7//CCL3//RAMP1//BHLHA15//CALCA//HTR2C//TRPV2 |
| GO:0050794 | regulation of cellular process | Biological process | 89 | 7207 | 147 | 13692 | 1.15023092628199 | 0.031903058245707 | 0.585200867569789 | 1.4961676832258 | KCNIP3//RBBP8//FEZF2//WNT10B//TBX3//CCL3//CHRNA7//CDH13//IGFBPL1//NKX3-1//CAMP//CALCA//DRD2//SMARCD3//TACR1//P2RX2//INHA//TMSB10//EPOR//POMC//GRB7//SMOC2//ARHGAP8//RGD1564053//RGS9//PLEK2//NRL//RFX2//TEAD2//BNIPL//TPD52L1//ACP5//NPPA//SSTR1//BHLHA15//RAMP1//NPW//OLR1401//OLR1513//OLR19//OLR56//OLR98//OLR200//OLR305//OLR375//OLR202//OLR857//OLR278//OLR1450//OLR606//OLR1138//OLR1585//GPR84//HTR2C//HRH3//HTR3A//TAC1//NPY//NMBR//GRP//GPC2//RAB3C//RAB26//DOK3//SCX//FOSL1//FRS3//LOC680319//MYH6//ITGA8//TNFSF13B//SLC7A3//LCP1//HPSE//SCN11A//RGS16//EFNB1//HRK//COL2A1//ERCC5//SCN9A//DKKL1//LOC680430//TRPV2//PLAGL1//SPIC//KRT4//PRTN3//AFP |
| GO:0007270 | neuron-neuron synaptic transmission | Biological process | 4 | 112 | 147 | 13692 | 3.3265306122449 | 0.0325033479198482 | 0.58673385028201 | 1.48807190338323 | DRD2//CHRNA7//TAC1//TACR1 |
| GO:0050896 | response to stimulus | Biological process | 82 | 6556 | 147 | 13692 | 1.16499607774776 | 0.0325531204682363 | 0.58673385028201 | 1.48740737461712 | CCL3//CHRNA7//RBBP8//DRD2//DIO3//CLDN3//P2RX2//KRT1//TACR1//LCP1//CALCA//TNFSF13B//TAC1//WNT10B//EPOR//POMC//GRB7//SMOC2//ARHGAP8//RGD1564053//RGS9//PLEK2//ERCC5//FOSL1//HSPB7//SCN9A//NPPA//SSTR1//BHLHA15//RAMP1//NPW//OLR1401//OLR1513//OLR19//OLR56//OLR98//OLR200//OLR305//OLR375//OLR202//OLR857//OLR278//OLR1450//OLR606//OLR1138//OLR1585//GPR84//HTR2C//HTR3A//NPY//NMBR//GRP//GPC2//RAB3C//RAB26//DOK3//CDH13//EFNB1//FEZF2//KCNIP3//HRH3//ITGA8//ACP5//CAMP//NTSR1//FRS3//TRPV2//COX8B//LRP2//NNMT//AFP//ATP4B//NKX3-1//HPSE//MYH6//SCX//SLC7A3//TEAD2//RGS16//TPD52L1//HRK//COL2A1 |
| GO:0030335 | positive regulation of cell migration | Biological process | 6 | 222 | 147 | 13692 | 2.51737451737452 | 0.032608125879027 | 0.58673385028201 | 1.48667416113842 | TACR1//TAC1//CCL3//ACP5//GRB7//CDH13 |
| GO:0015844 | monoamine transport | Biological process | 3 | 65 | 147 | 13692 | 4.2989010989011 | 0.0326558073605863 | 0.58673385028201 | 1.48603957456092 | DRD2//CHRNA7//HRH3 |
| GO:0090305 | nucleic acid phosphodiester bond hydrolysis | Biological process | 3 | 65 | 147 | 13692 | 4.2989010989011 | 0.0326558073605863 | 0.58673385028201 | 1.48603957456092 | ERCC5//TPD52L1//RBBP8 |
| GO:0003081 | regulation of systemic arterial blood pressure by renin-angiotensin | Biological process | 2 | 27 | 147 | 13692 | 6.8994708994709 | 0.033722641769274 | 0.598542759176102 | 1.47207841088958 | PCSK5//TACR1 |
| GO:0006182 | cGMP biosynthetic process | Biological process | 2 | 27 | 147 | 13692 | 6.8994708994709 | 0.033722641769274 | 0.598542759176102 | 1.47207841088958 | NPPA//HTR2C |
| GO:0045907 | positive regulation of vasoconstriction | Biological process | 2 | 27 | 147 | 13692 | 6.8994708994709 | 0.033722641769274 | 0.598542759176102 | 1.47207841088958 | TACR1//HTR2C |
| GO:0015850 | organic alcohol transport | Biological process | 3 | 66 | 147 | 13692 | 4.23376623376623 | 0.0339493903326076 | 0.600137609750612 | 1.46916802042801 | DRD2//CHRNA7//HRH3 |
| GO:0050727 | regulation of inflammatory response | Biological process | 5 | 167 | 147 | 13692 | 2.78870829769033 | 0.0340887964281625 | 0.6001818616107 | 1.46738833211916 | TAC1//CHRNA7//ACP5//CCL3//CALCA |
| GO:0007420 | brain development | Biological process | 11 | 549 | 147 | 13692 | 1.86625032526672 | 0.0344096536277556 | 0.603407686016322 | 1.46331969902428 | FEZF2//SSTR1//KIRREL3//TBX3//DRD2//NPY//LRP2//WNT10B//EPOR//HRH3//ITGA8 |
| GO:0006936 | muscle contraction | Biological process | 5 | 168 | 147 | 13692 | 2.77210884353742 | 0.0348420901547364 | 0.608556666288304 | 1.45789579983428 | MYH6//P2RX2//DRD2//CALCA//TACR1 |
| GO:0051017 | actin filament bundle assembly | Biological process | 3 | 67 | 147 | 13692 | 4.17057569296375 | 0.0352692999395862 | 0.613573852917246 | 1.45260316055911 | TAC1//TACR1//LCP1 |
| GO:2000147 | positive regulation of cell motility | Biological process | 6 | 227 | 147 | 13692 | 2.46192573945878 | 0.0357652982941574 | 0.615222168408293 | 1.44653814844798 | TACR1//TAC1//CCL3//ACP5//GRB7//CDH13 |
| GO:0002762 | negative regulation of myeloid leukocyte differentiation | Biological process | 2 | 28 | 147 | 13692 | 6.65306122448979 | 0.0360657156206504 | 0.615222168408293 | 1.44290544606046 | CALCA//CCL3 |
| GO:0007212 | dopamine receptor signaling pathway | Biological process | 2 | 28 | 147 | 13692 | 6.65306122448979 | 0.0360657156206504 | 0.615222168408293 | 1.44290544606046 | DRD2//RGS9 |
| GO:0051851 | modification by host of symbiont morphology or physiology | Biological process | 2 | 28 | 147 | 13692 | 6.65306122448979 | 0.0360657156206504 | 0.615222168408293 | 1.44290544606046 | CCL3//CAMP |
| GO:0051926 | negative regulation of calcium ion transport | Biological process | 2 | 28 | 147 | 13692 | 6.65306122448979 | 0.0360657156206504 | 0.615222168408293 | 1.44290544606046 | CALCA//DRD2 |
| GO:0002062 | chondrocyte differentiation | Biological process | 3 | 68 | 147 | 13692 | 4.10924369747899 | 0.0366154430527352 | 0.622178691252679 | 1.43633570646243 | COL2A1//WNT10B//SCX |
| GO:0006163 | purine nucleotide metabolic process | Biological process | 11 | 555 | 147 | 13692 | 1.84607464607465 | 0.0367833187197198 | 0.622618028058887 | 1.43434908976244 | NPPA//HTR2C//RAB26//MYH6//CALCA//DRD2//HRH3//RAMP1//ARHGAP8//RGS9//RGS16 |
| GO:0030279 | negative regulation of ossification | Biological process | 2 | 29 | 147 | 13692 | 6.42364532019704 | 0.0384697847916248 | 0.648659755871089 | 1.41488024322454 | CCL3//CALCA |
| GO:0060349 | bone morphogenesis | Biological process | 3 | 70 | 147 | 13692 | 3.99183673469388 | 0.0393860200680331 | 0.661564413709797 | 1.40465790215147 | COL2A1//SCX//ACP5 |
| GO:0050804 | regulation of synaptic transmission | Biological process | 6 | 233 | 147 | 13692 | 2.39852851011649 | 0.0398062345457069 | 0.663538145431099 | 1.40004890237985 | HTR2C//TAC1//TACR1//CHRNA7//DRD2//SCN9A |
| GO:0051047 | positive regulation of secretion | Biological process | 6 | 233 | 147 | 13692 | 2.39852851011649 | 0.0398062345457069 | 0.663538145431099 | 1.40004890237985 | DRD2//TAC1//TACR1//HTR2C//CCL3//NKX3-1 |
| GO:0051272 | positive regulation of cellular component movement | Biological process | 6 | 234 | 147 | 13692 | 2.38827838827839 | 0.0405068130752873 | 0.672658592886589 | 1.39247192413959 | TACR1//TAC1//CCL3//ACP5//GRB7//CDH13 |
| GO:0006308 | DNA catabolic process | Biological process | 2 | 30 | 147 | 13692 | 6.20952380952381 | 0.0409332889492134 | 0.674629844937412 | 1.38792335882141 | TPD52L1//RBBP8 |
| GO:0040018 | positive regulation of multicellular organism growth | Biological process | 2 | 30 | 147 | 13692 | 6.20952380952381 | 0.0409332889492134 | 0.674629844937412 | 1.38792335882141 | DRD2//DIO3 |
| GO:0050880 | regulation of blood vessel size | Biological process | 4 | 121 | 147 | 13692 | 3.07910271546635 | 0.0413416864131854 | 0.676275944908227 | 1.38361181157418 | CALCA//NPPA//TACR1//HTR2C |
| GO:0055002 | striated muscle cell development | Biological process | 4 | 121 | 147 | 13692 | 3.07910271546635 | 0.0413416864131854 | 0.676275944908227 | 1.38361181157418 | P2RX2//MYH6//TBX3//WNT10B |
| GO:0040017 | positive regulation of locomotion | Biological process | 6 | 236 | 147 | 13692 | 2.3680387409201 | 0.0419313784582924 | 0.683372353758936 | 1.37746086030119 | TACR1//TAC1//CCL3//ACP5//GRB7//CDH13 |
| GO:0035150 | regulation of tube size | Biological process | 4 | 122 | 147 | 13692 | 3.05386416861827 | 0.0423998763608497 | 0.688448362836908 | 1.37263540981951 | CALCA//NPPA//TACR1//HTR2C |
| GO:0051716 | cellular response to stimulus | Biological process | 65 | 5068 | 147 | 13692 | 1.19461044086143 | 0.0426709977947342 | 0.690293927424778 | 1.36986720172281 | CCL3//CHRNA7//RBBP8//CALCA//P2RX2//DRD2//WNT10B//EPOR//POMC//GRB7//SMOC2//ARHGAP8//RGD1564053//RGS9//PLEK2//ERCC5//NPPA//SSTR1//BHLHA15//RAMP1//NPW//OLR1401//OLR1513//OLR19//OLR56//OLR98//OLR200//OLR305//OLR375//OLR202//OLR857//OLR278//OLR1450//OLR606//OLR1138//OLR1585//GPR84//HTR2C//HTR3A//TAC1//TACR1//NPY//NMBR//GRP//GPC2//RAB3C//RAB26//DOK3//CDH13//FRS3//NKX3-1//MYH6//SCX//ITGA8//FOSL1//SLC7A3//TEAD2//RGS16//TPD52L1//EFNB1//HRK//HPSE//AFP//CAMP//COL2A1 |
| GO:0042312 | regulation of vasodilation | Biological process | 2 | 31 | 147 | 13692 | 6.00921658986175 | 0.0434546938299643 | 0.69722961391226 | 1.36196330565139 | CALCA//NPPA |
| GO:0051492 | regulation of stress fiber assembly | Biological process | 2 | 31 | 147 | 13692 | 6.00921658986175 | 0.0434546938299643 | 0.69722961391226 | 1.36196330565139 | TAC1//TACR1 |
| GO:0055067 | monovalent inorganic cation homeostasis | Biological process | 3 | 73 | 147 | 13692 | 3.82778864970646 | 0.0437358904712298 | 0.69722961391226 | 1.35916202672854 | DRD2//TAC1//TACR1 |
| GO:0071356 | cellular response to tumor necrosis factor | Biological process | 3 | 73 | 147 | 13692 | 3.82778864970646 | 0.0437358904712298 | 0.69722961391226 | 1.35916202672854 | CCL3//NKX3-1//CAMP |
| GO:0006812 | cation transport | Biological process | 11 | 575 | 147 | 13692 | 1.78186335403727 | 0.0455268270375028 | 0.718172384192189 | 1.34173261650758 | DRD2//KCNN3//SCN11A//CHRNA7//CCL3//RAMP1//BHLHA15//CALCA//HTR2C//TRPV2//P2RX2 |
| GO:0010631 | epithelial cell migration | Biological process | 2 | 32 | 147 | 13692 | 5.82142857142857 | 0.0460324908663333 | 0.718172384192189 | 1.33693552434054 | TAC1//TACR1 |
| GO:0030199 | collagen fibril organization | Biological process | 2 | 32 | 147 | 13692 | 5.82142857142857 | 0.0460324908663333 | 0.718172384192189 | 1.33693552434054 | COL2A1//SCX |
| GO:0051930 | regulation of sensory perception of pain | Biological process | 2 | 32 | 147 | 13692 | 5.82142857142857 | 0.0460324908663333 | 0.718172384192189 | 1.33693552434054 | HTR2C//CCL3 |
| GO:0051931 | regulation of sensory perception | Biological process | 2 | 32 | 147 | 13692 | 5.82142857142857 | 0.0460324908663333 | 0.718172384192189 | 1.33693552434054 | HTR2C//CCL3 |
| GO:0090132 | epithelium migration | Biological process | 2 | 32 | 147 | 13692 | 5.82142857142857 | 0.0460324908663333 | 0.718172384192189 | 1.33693552434054 | TAC1//TACR1 |
| GO:0046545 | development of primary female sexual characteristics | Biological process | 4 | 127 | 147 | 13692 | 2.93363329583802 | 0.0479193704450514 | 0.743373598780736 | 1.31948889625389 | INHA//AFP//WNT10B//TBX3 |
| GO:0006897 | endocytosis | Biological process | 7 | 308 | 147 | 13692 | 2.11688311688312 | 0.0484301371850549 | 0.743373598780736 | 1.31488430075259 | CALCA//DRD2//LRP2//CDH13//RAMP1//PRTN3//CHRNA7 |
| GO:0038032 | termination of G-protein coupled receptor signaling pathway | Biological process | 2 | 33 | 147 | 13692 | 5.64502164502164 | 0.0486651968179907 | 0.743373598780736 | 1.31278151585596 | RGS9//RGS16 |
| GO:0042755 | eating behavior | Biological process | 2 | 33 | 147 | 13692 | 5.64502164502164 | 0.0486651968179907 | 0.743373598780736 | 1.31278151585596 | TACR1//HRH3 |
| GO:0046717 | acid secretion | Biological process | 2 | 33 | 147 | 13692 | 5.64502164502164 | 0.0486651968179907 | 0.743373598780736 | 1.31278151585596 | ATP4B//DRD2 |
| GO:2000179 | positive regulation of neural precursor cell proliferation | Biological process | 2 | 33 | 147 | 13692 | 5.64502164502164 | 0.0486651968179907 | 0.743373598780736 | 1.31278151585596 | DRD2//SMARCD3 |
| GO:0006357 | regulation of transcription from RNA polymerase II promoter | Biological process | 17 | 1027 | 147 | 13692 | 1.54179997217972 | 0.048886369213904 | 0.74415917581165 | 1.31081221654923 | KCNIP3//RBBP8//FEZF2//WNT10B//TBX3//DRD2//POMC//PLAGL1//BHLHA15//CCL3//NRL//NKX3-1//TEAD2//SPIC//SCX//ITGA8//SMARCD3 |
| GO:0046942 | carboxylic acid transport | Biological process | 5 | 185 | 147 | 13692 | 2.51737451737452 | 0.0492381989247134 | 0.746921329017106 | 1.30769784143049 | HRH3//HTR2C//SLC7A3//SLC6A5//DRD2 |
