# Supplementary material for: Gene Expression in the Hippocampus in a Rat Model of Premenstrual Dysphoric Disorder After Treatment With Baixiangdan Capsules
Source: Front Psychol. 2018 Nov 13;9:2065. doi: 10.3389/fpsyg.2018.02065 (PMC6242977; doi:10.3389/fpsyg.2018.02065)
Supplement: Supplementary file 3 [file Data_Sheet_3.ZIP › Data Analysis Folder/GO Analysis Report/BXD vs blank (up)/CC_result(Rat).html]

| GO.ID | Term | Ontology | Count | Pop.Hits | List.Total | Pop.Total | Fold.Enrichment | Pvalue | FDR | Enrichment.Score | GENES |
| --- | --- | --- | --- | --- | --- | --- | --- | --- | --- | --- | --- |
| GO:0044425 | membrane part | Cellular component | 97 | 5668 | 174 | 15288 | 1.50363808921227 | 3.72240604047039e-07 | 0.00019989320437326 | 6.42917625573032 | RGS9//ATP4B//DRD2//EPOR//TACR1//SSTR1//EFNB1//HTR2C//COX8B//NMBR//TRHR//ACP5//LRP2//CHRNE//TRPV2//DIO3//SLC7A3//KCNJ16//KCNN3//RAMP1//CML1//FXYD6//KCNK12//KCNK13//CLDN3//SLC2A5//TPBG//HRH3//P2RX2//EMB//PCSK5//HRK//SLC16A10//SLC6A5//OLR1401//OLR1513//CLDN23//OLR19//OLR56//OLR98//OLR200//OLR305//OLR375//TAAR7B//SLC2A6//SEMA5B//SLC10A4//CHST8//SLC35D3//GPR123//TMEM79//GALNT14//SLC24A4//KIRREL3//VSTM2B//SEMA4G//SYPL2//OLR202//NTSR1//OLR857//OLR278//OLR1450//OLR606//OLR1138//OLR1585//VOM1R37//LOC500413//LOC688657//GPR84//VOM2R71//SLC25A42//FRMD3//SCN11A//SCN9A//LCP1//NTNG1//HTR3A//CYP2A1//METTL7B//CHRNA7//CDH13//MYH6//GRB7//PDCD1//SCUBE1//GPC2//PDZD11//UPK2//AP1S2//PCSK7//RAB26//ITGA8//INHA//CLCNKB//HPSE//RGS16//RFTN1 |
| GO:0016020 | membrane | Cellular component | 111 | 7097 | 174 | 15288 | 1.37419890871811 | 2.66130857740838e-06 | 0.00071456135303415 | 5.57490476629942 | DRD2//TAC1//TACR1//SSTR1//HTR2C//NMBR//CHRNA7//TRHR//CHRNE//TRPV2//DIO3//RGS9//SLC7A3//SCN11A//KCNJ16//KCNN3//RAMP1//FXYD6//KCNIP3//HTR3A//CLCNKB//HRH3//P2RX2//RAB3C//GPC2//CDH13//TAAR7B//NTNG1//KRT1//LCP1//DOK3//GPR123//GPRC5A//PRTN3//WNT10B//KIRREL3//FRS3//RGS16//NTSR1//VOM2R71//ATP4B//EPOR//EFNB1//COX8B//ACP5//LRP2//CML1//KCNK12//KCNK13//CLDN3//SLC2A5//TPBG//EMB//PCSK5//HRK//SLC16A10//SLC6A5//OLR1401//OLR1513//CLDN23//OLR19//OLR56//OLR98//OLR200//OLR305//OLR375//SLC2A6//SEMA5B//SLC10A4//CHST8//SLC35D3//TMEM79//GALNT14//SLC24A4//VSTM2B//SEMA4G//SYPL2//OLR202//OLR857//OLR278//OLR1450//OLR606//OLR1138//OLR1585//VOM1R37//LOC500413//LOC688657//GPR84//SLC25A42//FRMD3//SCN9A//HPSE//CYP2A1//METTL7B//MYH6//GRB7//PDCD1//SCUBE1//PDZD11//UPK2//AP1S2//PCSK7//N4BP3//RAB26//ITGA8//INHA//PRPH//FOSL1//RFTN1//SYT17//TNFSF13B |
| GO:0031224 | intrinsic to membrane | Cellular component | 80 | 4750 | 174 | 15288 | 1.47978221415608 | 2.39856032195149e-05 | 0.00429342297629317 | 4.62004935488779 | ATP4B//DRD2//EPOR//TACR1//SSTR1//EFNB1//HTR2C//COX8B//NMBR//TRHR//ACP5//LRP2//CHRNE//TRPV2//DIO3//SLC7A3//KCNJ16//KCNN3//RAMP1//CML1//FXYD6//KCNK12//KCNK13//CLDN3//SLC2A5//TPBG//HRH3//P2RX2//EMB//PCSK5//HRK//SLC16A10//SLC6A5//OLR1401//OLR1513//CLDN23//OLR19//OLR56//OLR98//OLR200//OLR305//OLR375//TAAR7B//SLC2A6//SEMA5B//SLC10A4//CHST8//SLC35D3//GPR123//TMEM79//GALNT14//SLC24A4//KIRREL3//VSTM2B//SEMA4G//SYPL2//OLR202//NTSR1//OLR857//OLR278//OLR1450//OLR606//OLR1138//OLR1585//VOM1R37//LOC500413//LOC688657//GPR84//VOM2R71//SLC25A42//SCN11A//SCN9A//NTNG1//HTR3A//CHRNA7//GPC2//PCSK7//UPK2//RAB26//CLCNKB |
| GO:0016021 | integral to membrane | Cellular component | 77 | 4653 | 174 | 15288 | 1.45398222874378 | 7.65253054630705e-05 | 0.0102735222584172 | 4.11619492817086 | SCN11A//SCN9A//DRD2//HTR3A//CHRNA7//PCSK7//UPK2//CLCNKB//ATP4B//EPOR//TACR1//SSTR1//EFNB1//HTR2C//COX8B//NMBR//TRHR//ACP5//LRP2//CHRNE//TRPV2//DIO3//SLC7A3//KCNJ16//KCNN3//RAMP1//CML1//FXYD6//KCNK12//KCNK13//CLDN3//SLC2A5//TPBG//HRH3//P2RX2//EMB//PCSK5//HRK//SLC16A10//SLC6A5//OLR1401//OLR1513//CLDN23//OLR19//OLR56//OLR98//OLR200//OLR305//OLR375//TAAR7B//SLC2A6//SEMA5B//SLC10A4//CHST8//SLC35D3//GPR123//TMEM79//GALNT14//SLC24A4//KIRREL3//VSTM2B//SEMA4G//SYPL2//OLR202//NTSR1//OLR857//OLR278//OLR1450//OLR606//OLR1138//OLR1585//VOM1R37//LOC500413//LOC688657//GPR84//VOM2R71//SLC25A42 |
| GO:0005615 | extracellular space | Cellular component | 20 | 707 | 174 | 15288 | 2.48548992830318 | 0.000160832657395782 | 0.0167538582385586 | 3.79362576226202 | INHA//AFP//CALCA//NPY//POMC//TAC1//COL2A1//CCL3//ACP5//LRP2//RAMP1//PCSK5//GRP//GPC2//CDH13//SCUBE1//WNT10B//CAMP//LOC360919//CBLN4 |
| GO:0005886 | plasma membrane | Cellular component | 54 | 2986 | 174 | 15288 | 1.58893225858605 | 0.000187193946799537 | 0.0167538582385586 | 3.72770819894227 | RGS9//SCN11A//SCN9A//LCP1//NTNG1//DRD2//HTR3A//CHRNA7//CDH13//CLDN3//CLDN23//MYH6//GRB7//HTR2C//PDCD1//SCUBE1//SLC16A10//PDZD11//LRP2//UPK2//RAB26//TRPV2//ITGA8//INHA//TAC1//TACR1//SSTR1//NMBR//TRHR//CHRNE//DIO3//SLC7A3//KCNJ16//KCNN3//RAMP1//FXYD6//KCNIP3//CLCNKB//HRH3//P2RX2//RAB3C//GPC2//TAAR7B//KRT1//DOK3//GPR123//GPRC5A//PRTN3//WNT10B//KIRREL3//FRS3//RGS16//NTSR1//VOM2R71 |
| GO:0071944 | cell periphery | Cellular component | 54 | 3081 | 174 | 15288 | 1.53993889131384 | 0.000421870447327003 | 0.031735230371298 | 3.37482089654829 | DRD2//TAC1//TACR1//SSTR1//HTR2C//NMBR//CHRNA7//TRHR//CHRNE//TRPV2//DIO3//RGS9//SLC7A3//SCN11A//KCNJ16//KCNN3//RAMP1//FXYD6//KCNIP3//HTR3A//CLCNKB//HRH3//P2RX2//RAB3C//GPC2//CDH13//TAAR7B//NTNG1//KRT1//LCP1//DOK3//GPR123//GPRC5A//PRTN3//WNT10B//KIRREL3//FRS3//RGS16//NTSR1//VOM2R71//SCN9A//CLDN3//CLDN23//MYH6//GRB7//PDCD1//SCUBE1//SLC16A10//PDZD11//LRP2//UPK2//RAB26//ITGA8//INHA |
| GO:0044421 | extracellular region part | Cellular component | 22 | 887 | 174 | 15288 | 2.17921704311317 | 0.0004727781060901 | 0.031735230371298 | 3.32534264343056 | COL2A1//GPC2//WNT10B//SMOC2//FREM2//AFP//CALCA//INHA//NPY//POMC//TAC1//CCL3//ACP5//LRP2//RAMP1//PCSK5//GRP//CDH13//SCUBE1//CAMP//LOC360919//CBLN4 |
| GO:0005576 | extracellular region | Cellular component | 29 | 1410 | 174 | 15288 | 1.80709219858156 | 0.00124446334083405 | 0.0742529793364316 | 2.90501789223175 | COL2A1//GPC2//WNT10B//SMOC2//FREM2//AFP//CALCA//INHA//NPY//POMC//TAC1//CCL3//ACP5//LRP2//RAMP1//PCSK5//GRP//CDH13//SCUBE1//CAMP//LOC360919//CBLN4//NPPA//NXPH3//HPSE//NPW//KIRREL3//IGFBPL1//LOC499602 |
| GO:0044297 | cell body | Cellular component | 12 | 441 | 174 | 15288 | 2.39080459770115 | 0.00464925178289664 | 0.232880991319418 | 2.3326169337133 | CALCA//INHA//PRPH//TAC1//CHRNA7//HTR3A//P2RX2//KLHL14//DRD2//ITGA8//TACR1//TRPV2 |
| GO:0030424 | axon | Cellular component | 10 | 334 | 174 | 15288 | 2.63060086723106 | 0.00496935699417725 | 0.232880991319418 | 2.30369980280526 | PRPH//CALCA//P2RX2//DRD2//TRPV2//SCN11A//TAC1//CHRNA7//HTR3A//KIRREL3 |
| GO:0044456 | synapse part | Cellular component | 11 | 391 | 174 | 15288 | 2.47182291207338 | 0.00520404449875795 | 0.232880991319418 | 2.28365899852813 | RAB3C//SYT17//SYPL2//DRD2//CHRNA7//P2RX2//ITGA8//FOSL1//CALCA//CHRNE//HTR3A |
| GO:0032982 | myosin filament | Cellular component | 2 | 12 | 174 | 15288 | 14.6436781609195 | 0.00788675351215918 | 0.325783587386883 | 2.10310173213986 | MYH3//MYH6 |
| GO:0001518 | voltage-gated sodium channel complex | Cellular component | 2 | 13 | 174 | 15288 | 13.5172413793103 | 0.00925142166232513 | 0.354858102333471 | 2.03379152427057 | SCN11A//SCN9A |
| GO:0034706 | sodium channel complex | Cellular component | 2 | 16 | 174 | 15288 | 10.9827586206897 | 0.013918477222834 | 0.498281484577457 | 1.85640827693065 | SCN11A//SCN9A |
| GO:0045202 | synapse | Cellular component | 12 | 527 | 174 | 15288 | 2.00065432179546 | 0.01752303807793 | 0.588116965490526 | 1.75639059530858 | RAB3C//SYT17//SYPL2//DRD2//CHRNA7//P2RX2//ITGA8//FOSL1//CALCA//CHRNE//HTR3A//EFNB1 |
| GO:0043025 | neuronal cell body | Cellular component | 10 | 413 | 174 | 15288 | 2.12741087083577 | 0.0199423112759158 | 0.629942420892164 | 1.70022450920358 | DRD2//ITGA8//CALCA//INHA//PRPH//TAC1//CHRNA7//HTR3A//P2RX2//KLHL14 |
| GO:0042734 | presynaptic membrane | Cellular component | 3 | 54 | 174 | 15288 | 4.88122605363985 | 0.0235256724475301 | 0.701849228017981 | 1.6284579540398 | CHRNA7//FOSL1//P2RX2 |
| GO:0044459 | plasma membrane part | Cellular component | 24 | 1426 | 174 | 15288 | 1.47874449871838 | 0.0335037109986849 | 0.946920674015463 | 1.47490708618731 | RGS9//SCN11A//SCN9A//LCP1//NTNG1//DRD2//HTR3A//CHRNA7//CDH13//CLDN3//CLDN23//MYH6//GRB7//HTR2C//PDCD1//SCUBE1//SLC16A10//PDZD11//LRP2//UPK2//RAB26//TRPV2//ITGA8//INHA |
| GO:0045095 | keratin filament | Cellular component | 3 | 65 | 174 | 15288 | 4.0551724137931 | 0.0378690155446262 | 0.966692400764311 | 1.42171598479451 | KRT1//KRT4//LOC689927 |
| GO:0008021 | synaptic vesicle | Cellular component | 4 | 111 | 174 | 15288 | 3.16620068344206 | 0.0379856414102094 | 0.966692400764311 | 1.42038053587645 | DRD2//RAB3C//SYT17//SYPL2 |
| GO:0005882 | intermediate filament | Cellular component | 4 | 115 | 174 | 15288 | 3.05607196401799 | 0.0423686330548764 | 0.966692400764311 | 1.37295554747649 | PRPH//KRT1//KRT4//LOC689927 |
| GO:0045121 | membrane raft | Cellular component | 6 | 224 | 174 | 15288 | 2.35344827586207 | 0.0431700406102475 | 0.966692400764311 | 1.36481754280081 | CDH13//EFNB1//CHRNA7//HPSE//RGS16//RFTN1 |
| GO:0043005 | neuron projection | Cellular component | 14 | 742 | 174 | 15288 | 1.65777488614183 | 0.044028635454767 | 0.966692400764311 | 1.35626477408279 | PRPH//DRD2//CHRNA7//P2RX2//ITGA8//CALCA//TAC1//TRPV2//SCN11A//HTR3A//KIRREL3//TACR1//CDH13//KLHL14 |
| GO:0033267 | axon part | Cellular component | 5 | 170 | 174 | 15288 | 2.5841784989858 | 0.0450043017115601 | 0.966692400764311 | 1.34674597244011 | PRPH//CALCA//P2RX2//DRD2//TRPV2 |
