# Supplementary material for: Gene Expression in the Hippocampus in a Rat Model of Premenstrual Dysphoric Disorder After Treatment With Baixiangdan Capsules
Source: Front Psychol. 2018 Nov 13;9:2065. doi: 10.3389/fpsyg.2018.02065 (PMC6242977; doi:10.3389/fpsyg.2018.02065)
Supplement: Supplementary file 3 [file Data_Sheet_3.ZIP › Data Analysis Folder/GO Analysis Report/BXD vs blank (up)/MF_result(Rat).html]

| GO.ID | Term | Ontology | Count | Pop.Hits | List.Total | Pop.Total | Fold.Enrichment | Pvalue | FDR | Enrichment.Score | GENES |
| --- | --- | --- | --- | --- | --- | --- | --- | --- | --- | --- | --- |
| GO:0030594 | neurotransmitter receptor activity | Molecular function | 7 | 65 | 158 | 14392 | 9.8095423563778 | 6.86685167512867e-06 | 0.00320633688514694 | 5.16324233332064 | DRD2//NMBR//NPY//SSTR1//CHRNA7//NTSR1//TACR1 |
| GO:0005215 | transporter activity | Molecular function | 28 | 1052 | 158 | 14392 | 2.42441160899071 | 1.00399483335148e-05 | 0.00320633688514694 | 4.99826852210406 | SLC7A3//COX8B//CHRNA7//CHRNE//P2RX2//TRPV2//FXYD6//HTR3A//KCNJ16//KCNK12//KCNK13//KCNIP3//CLCNKB//SCN11A//SCN9A//SLC6A5//SLC25A42//SLC2A5//ATP4B//SLC10A4//RAMP1//AP1S2//KCNN3//SLC2A6//LRP2//SYT17//SYPL2//PMP2 |
| GO:0022892 | substrate-specific transporter activity | Molecular function | 25 | 880 | 158 | 14392 | 2.58774453394707 | 1.07235347329329e-05 | 0.00320633688514694 | 4.96966203722024 | SLC7A3//COX8B//CHRNA7//CHRNE//P2RX2//TRPV2//FXYD6//HTR3A//KCNJ16//KCNK12//KCNK13//KCNIP3//CLCNKB//SCN11A//SCN9A//SLC6A5//SLC25A42//SLC2A5//ATP4B//SLC10A4//RAMP1//AP1S2//KCNN3//SLC2A6//LRP2 |
| GO:0005261 | cation channel activity | Molecular function | 12 | 254 | 158 | 14392 | 4.30339878401276 | 2.37536011203953e-05 | 0.00446409266429273 | 4.62427054064287 | CHRNA7//CHRNE//P2RX2//HTR3A//KCNJ16//SCN11A//SCN9A//TRPV2//KCNK12//KCNK13//KCNIP3//KCNN3 |
| GO:0005216 | ion channel activity | Molecular function | 14 | 348 | 158 | 14392 | 3.66448421358941 | 2.97346045313953e-05 | 0.00446409266429273 | 4.52673783321901 | CHRNA7//CHRNE//P2RX2//HTR3A//KCNJ16//KCNK12//KCNK13//KCNIP3//CLCNKB//SCN11A//SCN9A//TRPV2//KCNN3//FXYD6 |
| GO:0022891 | substrate-specific transmembrane transporter activity | Molecular function | 22 | 772 | 158 | 14392 | 2.595789335607 | 3.67781406163642e-05 | 0.00446409266429273 | 4.43441023106491 | SLC7A3//COX8B//CHRNA7//CHRNE//P2RX2//TRPV2//FXYD6//HTR3A//KCNJ16//KCNK12//KCNK13//KCNIP3//CLCNKB//SCN11A//SCN9A//SLC6A5//SLC25A42//SLC2A5//ATP4B//SLC10A4//KCNN3//SLC2A6 |
| GO:0022838 | substrate-specific channel activity | Molecular function | 14 | 357 | 158 | 14392 | 3.57210225862497 | 3.9317129816969e-05 | 0.00446409266429273 | 4.40541819353953 | CHRNA7//CHRNE//P2RX2//TRPV2//FXYD6//HTR3A//KCNJ16//KCNK12//KCNK13//KCNIP3//CLCNKB//SCN11A//SCN9A//KCNN3 |
| GO:0008324 | cation transmembrane transporter activity | Molecular function | 17 | 507 | 158 | 14392 | 3.05425311462312 | 4.22854134370359e-05 | 0.00446409266429273 | 4.37380941882593 | COX8B//CHRNA7//CHRNE//P2RX2//HTR3A//KCNJ16//SCN11A//SCN9A//TRPV2//KCNK12//KCNK13//KCNIP3//SLC6A5//ATP4B//SLC10A4//KCNN3//SLC7A3 |
| GO:0022836 | gated channel activity | Molecular function | 12 | 271 | 158 | 14392 | 4.03344387874259 | 4.47902274009304e-05 | 0.00446409266429273 | 4.34881673262746 | CHRNA7//CHRNE//P2RX2//HTR3A//KCNJ16//KCNK12//KCNK13//KCNIP3//CLCNKB//SCN11A//SCN9A//KCNN3 |
| GO:0015267 | channel activity | Molecular function | 14 | 375 | 158 | 14392 | 3.40064135021097 | 6.6893725511869e-05 | 0.00539797072694168 | 4.17461461621374 | CHRNA7//CHRNE//P2RX2//TRPV2//FXYD6//HTR3A//KCNJ16//KCNK12//KCNK13//KCNIP3//CLCNKB//SCN11A//SCN9A//KCNN3 |
| GO:0022803 | passive transmembrane transporter activity | Molecular function | 14 | 375 | 158 | 14392 | 3.40064135021097 | 6.6893725511869e-05 | 0.00539797072694168 | 4.17461461621374 | CHRNA7//CHRNE//P2RX2//TRPV2//FXYD6//HTR3A//KCNJ16//KCNK12//KCNK13//KCNIP3//CLCNKB//SCN11A//SCN9A//KCNN3 |
| GO:0008188 | neuropeptide receptor activity | Molecular function | 5 | 40 | 158 | 14392 | 11.3860759493671 | 7.22136552099221e-05 | 0.00539797072694168 | 4.14138067193754 | NMBR//NPY//SSTR1//NTSR1//TACR1 |
| GO:0015075 | ion transmembrane transporter activity | Molecular function | 19 | 648 | 158 | 14392 | 2.67080793874043 | 8.98772700220117e-05 | 0.00620153163151881 | 4.04635012753442 | COX8B//CHRNA7//CHRNE//P2RX2//TRPV2//FXYD6//HTR3A//KCNJ16//KCNK12//KCNK13//KCNIP3//CLCNKB//SCN11A//SCN9A//SLC6A5//ATP4B//SLC10A4//KCNN3//SLC7A3 |
| GO:0022857 | transmembrane transporter activity | Molecular function | 22 | 839 | 158 | 14392 | 2.38849745779333 | 0.00012528100014967 | 0.00802693265244671 | 3.90211478819043 | SLC7A3//COX8B//CHRNA7//CHRNE//P2RX2//TRPV2//FXYD6//HTR3A//KCNJ16//KCNK12//KCNK13//KCNIP3//CLCNKB//SCN11A//SCN9A//SLC6A5//SLC25A42//SLC2A5//ATP4B//SLC10A4//KCNN3//SLC2A6 |
| GO:0015077 | monovalent inorganic cation transmembrane transporter activity | Molecular function | 12 | 310 | 158 | 14392 | 3.5260106165782 | 0.000160797984163108 | 0.00961571945295386 | 3.79371940006721 | COX8B//KCNJ16//SCN11A//SCN9A//HTR3A//KCNK12//KCNK13//KCNIP3//SLC6A5//ATP4B//SLC10A4//KCNN3 |
| GO:0046873 | metal ion transmembrane transporter activity | Molecular function | 12 | 337 | 158 | 14392 | 3.24351124967134 | 0.000345856450188283 | 0.0192073104162049 | 3.46110412036102 | KCNJ16//SCN11A//SCN9A//HTR3A//TRPV2//KCNK12//KCNK13//KCNIP3//SLC6A5//ATP4B//SLC10A4//KCNN3 |
| GO:0008227 | G-protein coupled amine receptor activity | Molecular function | 5 | 56 | 158 | 14392 | 8.13291139240506 | 0.000364018146126515 | 0.0192073104162049 | 3.43887696644384 | DRD2//TAAR7B//HRH3//HTR2C//HTR3A |
| GO:0005244 | voltage-gated ion channel activity | Molecular function | 8 | 165 | 158 | 14392 | 4.41641733793633 | 0.0004728553478054 | 0.0213784821348925 | 3.32527169490253 | KCNJ16//CLCNKB//SCN11A//SCN9A//HTR3A//KCNK12//KCNK13//KCNIP3 |
| GO:0022832 | voltage-gated channel activity | Molecular function | 8 | 165 | 158 | 14392 | 4.41641733793633 | 0.0004728553478054 | 0.0213784821348925 | 3.32527169490253 | KCNJ16//KCNK12//KCNK13//KCNIP3//CLCNKB//SCN11A//SCN9A//HTR3A |
| GO:0015079 | potassium ion transmembrane transporter activity | Molecular function | 7 | 126 | 158 | 14392 | 5.06047819971871 | 0.00047666626833651 | 0.0213784821348925 | 3.32178558017627 | KCNJ16//HTR3A//KCNK12//KCNK13//KCNIP3//ATP4B//KCNN3 |
| GO:0022890 | inorganic cation transmembrane transporter activity | Molecular function | 13 | 429 | 158 | 14392 | 2.7602608362102 | 0.000901907475493246 | 0.0385243335960687 | 3.04483801339352 | COX8B//KCNJ16//SCN11A//SCN9A//HTR3A//TRPV2//KCNK12//KCNK13//KCNIP3//SLC6A5//ATP4B//SLC10A4//KCNN3 |
| GO:0005179 | hormone activity | Molecular function | 6 | 108 | 158 | 14392 | 5.06047819971871 | 0.00121255686042141 | 0.0494392501726366 | 2.91629788687971 | CALCA//GRP//INHA//NPPA//NPY//POMC |
| GO:0005267 | potassium channel activity | Molecular function | 6 | 110 | 158 | 14392 | 4.96846950517837 | 0.00133312433074083 | 0.0519918488988924 | 2.87512934523229 | KCNJ16//HTR3A//KCNN3//KCNK12//KCNK13//KCNIP3 |
| GO:0001664 | G-protein coupled receptor binding | Molecular function | 8 | 195 | 158 | 14392 | 3.73696851671535 | 0.00140286462103685 | 0.0524320652112523 | 2.85298423714944 | CCL3//CALCA//RAMP1//POMC//TAC1//NPY//NPW//WNT10B |
| GO:0005231 | excitatory extracellular ligand-gated ion channel activity | Molecular function | 4 | 47 | 158 | 14392 | 7.75222192297334 | 0.00172776304233369 | 0.0619921379589328 | 2.7625158199957 | CHRNA7//CHRNE//P2RX2//HTR3A |
| GO:0071855 | neuropeptide receptor binding | Molecular function | 3 | 27 | 158 | 14392 | 10.1209563994374 | 0.00312944576926177 | 0.107965879039531 | 2.504532570014 | POMC//TAC1//NPY |
| GO:0051378 | serotonin binding | Molecular function | 2 | 10 | 158 | 14392 | 18.2177215189873 | 0.00508665371288961 | 0.16898994001711 | 2.29356782708266 | HTR2C//HTR3A |
| GO:0005230 | extracellular ligand-gated ion channel activity | Molecular function | 4 | 66 | 158 | 14392 | 5.52052167242041 | 0.00594531879717835 | 0.190462534323892 | 2.22582485287988 | CHRNA7//CHRNE//P2RX2//HTR3A |
| GO:0008656 | cysteine-type endopeptidase activator activity involved in apoptotic process | Molecular function | 2 | 11 | 158 | 14392 | 16.5615650172612 | 0.00617239964658085 | 0.190918706309759 | 2.20954596227743 | NKX3-1//TPD52L1 |
| GO:0008144 | drug binding | Molecular function | 5 | 112 | 158 | 14392 | 4.06645569620253 | 0.00783283063908221 | 0.226646744621185 | 2.10608126364088 | DRD2//HTR2C//CHRNA7//HRH3//P2RX2 |
| GO:0015081 | sodium ion transmembrane transporter activity | Molecular function | 5 | 112 | 158 | 14392 | 4.06645569620253 | 0.00783283063908221 | 0.226646744621185 | 2.10608126364088 | SCN11A//SCN9A//SLC6A5//ATP4B//SLC10A4 |
| GO:0008528 | G-protein coupled peptide receptor activity | Molecular function | 5 | 114 | 158 | 14392 | 3.99511436819898 | 0.00842380869424286 | 0.231810867777646 | 2.074491504593 | NMBR//NPY//SSTR1//NTSR1//TACR1 |
| GO:0016505 | apoptotic protease activator activity | Molecular function | 2 | 13 | 158 | 14392 | 14.0136319376826 | 0.00862854537828374 | 0.231810867777646 | 2.06406241256188 | NKX3-1//TPD52L1 |
| GO:0043176 | amine binding | Molecular function | 3 | 39 | 158 | 14392 | 7.00681596884129 | 0.00888373656840868 | 0.231810867777646 | 2.05140432817205 | DRD2//HTR2C//HTR3A |
| GO:0001653 | peptide receptor activity | Molecular function | 5 | 116 | 158 | 14392 | 3.92623308598865 | 0.00904501713736634 | 0.231810867777646 | 2.04359060595656 | NMBR//NPY//SSTR1//NTSR1//TACR1 |
| GO:0015276 | ligand-gated ion channel activity | Molecular function | 5 | 118 | 158 | 14392 | 3.85968676249732 | 0.00969718240518691 | 0.235091151823045 | 2.0133544351807 | CHRNA7//CHRNE//P2RX2//HTR3A//KCNJ16 |
| GO:0022834 | ligand-gated channel activity | Molecular function | 5 | 118 | 158 | 14392 | 3.85968676249732 | 0.00969718240518691 | 0.235091151823045 | 2.0133544351807 | CHRNA7//CHRNE//P2RX2//HTR3A//KCNJ16 |
| GO:0004993 | serotonin receptor activity | Molecular function | 2 | 14 | 158 | 14392 | 13.0126582278481 | 0.00999457506166709 | 0.235924574481984 | 2.00023566600772 | HTR2C//HTR3A |
| GO:0005184 | neuropeptide hormone activity | Molecular function | 2 | 15 | 158 | 14392 | 12.1451476793249 | 0.0114497225530219 | 0.256760028251516 | 1.94120503691811 | CALCA//GRP |
| GO:0005248 | voltage-gated sodium channel activity | Molecular function | 2 | 15 | 158 | 14392 | 12.1451476793249 | 0.0114497225530219 | 0.256760028251516 | 1.94120503691811 | SCN11A//SCN9A |
| GO:0004872 | receptor activity | Molecular function | 40 | 2579 | 158 | 14392 | 1.41277406118552 | 0.012143267678524 | 0.265671002625269 | 1.91566443156436 | DRD2//TAAR7B//P2RX2//NKX3-1//EPOR//OLR1401//OLR19//OLR98//OLR305//OLR375//GPR123//OLR202//OLR278//OLR1450//OLR1138//OLR1585//LOC688657//VOM2R71//NMBR//RAMP1//HRH3//NPY//OLR1513//OLR56//OLR200//OLR857//OLR606//HTR2C//HTR3A//SSTR1//TRHR//CHRNA7//NTSR1//TACR1//VOM1R37//LRP2//CHRNE//SEMA5B//FRS3//SEMA4G |
| GO:0004889 | acetylcholine-activated cation-selective channel activity | Molecular function | 2 | 16 | 158 | 14392 | 11.3860759493671 | 0.0129918920256834 | 0.277469693977095 | 1.88632759746595 | CHRNA7//CHRNE |
| GO:0004888 | transmembrane signaling receptor activity | Molecular function | 34 | 2149 | 158 | 14392 | 1.44114130210696 | 0.0161783337125046 | 0.337487566049224 | 1.79106621058097 | DRD2//TAAR7B//P2RX2//EPOR//OLR1401//OLR19//OLR98//OLR305//OLR375//GPR123//OLR202//OLR278//OLR1450//OLR1138//OLR1585//LOC688657//VOM2R71//NMBR//RAMP1//HRH3//NPY//OLR1513//OLR56//OLR200//OLR857//OLR606//HTR2C//HTR3A//SSTR1//TRHR//CHRNA7//NTSR1//TACR1//VOM1R37 |
| GO:0038023 | signaling receptor activity | Molecular function | 35 | 2232 | 158 | 14392 | 1.42836078217867 | 0.016627422338749 | 0.338972678133133 | 1.77917507193654 | DRD2//TAAR7B//P2RX2//NKX3-1//EPOR//OLR1401//OLR19//OLR98//OLR305//OLR375//GPR123//OLR202//OLR278//OLR1450//OLR1138//OLR1585//LOC688657//VOM2R71//NMBR//RAMP1//HRH3//NPY//OLR1513//OLR56//OLR200//OLR857//OLR606//HTR2C//HTR3A//SSTR1//TRHR//CHRNA7//NTSR1//TACR1//VOM1R37 |
| GO:0004930 | G-protein coupled receptor activity | Molecular function | 26 | 1569 | 158 | 14392 | 1.50943518003082 | 0.0211298729935962 | 0.421188801672351 | 1.6751031133758 | DRD2//TAAR7B//NMBR//RAMP1//HRH3//NPY//HTR2C//HTR3A//SSTR1//TRHR//NTSR1//TACR1//VOM1R37//OLR1401//OLR19//OLR98//OLR305//OLR375//GPR123//OLR202//OLR278//OLR1450//OLR1138//OLR1585//LOC688657//VOM2R71 |
| GO:0005102 | receptor binding | Molecular function | 19 | 1052 | 158 | 14392 | 1.64513644895798 | 0.0218014128326294 | 0.425127550236273 | 1.66151536118747 | NPY//POMC//RAMP1//NPW//WNT10B//FRS3//EFNB1//DOK3//TNFSF13B//INHA//NPPA//CALCA//GRP//CCL3//SMARCD3//NKX3-1//TAC1//DRD2//NXPH3 |
| GO:0004520 | endodeoxyribonuclease activity | Molecular function | 2 | 24 | 158 | 14392 | 7.59071729957806 | 0.0282217841040075 | 0.538615751942441 | 1.54941553480443 | RBBP8//ERCC5 |
| GO:0004871 | signal transducer activity | Molecular function | 36 | 2433 | 158 | 14392 | 1.34779690646022 | 0.0338660659509873 | 0.619956350163992 | 1.47023525021129 | DRD2//TAAR7B//P2RX2//NKX3-1//EPOR//OLR1401//OLR19//OLR98//OLR305//OLR375//GPR123//OLR202//OLR278//OLR1450//OLR1138//OLR1585//LOC688657//VOM2R71//NMBR//RAMP1//HRH3//NPY//OLR1513//OLR56//OLR200//OLR857//OLR606//HTR2C//HTR3A//SSTR1//TRHR//CHRNA7//NTSR1//TACR1//VOM1R37//RGS9 |
| GO:0060089 | molecular transducer activity | Molecular function | 36 | 2433 | 158 | 14392 | 1.34779690646022 | 0.0338660659509873 | 0.619956350163992 | 1.47023525021129 | DRD2//TAAR7B//P2RX2//RGS9//NKX3-1//EPOR//OLR1401//OLR19//OLR98//OLR305//OLR375//GPR123//OLR202//OLR278//OLR1450//OLR1138//OLR1585//LOC688657//VOM2R71//NMBR//RAMP1//HRH3//NPY//OLR1513//OLR56//OLR200//OLR857//OLR606//HTR2C//HTR3A//SSTR1//TRHR//CHRNA7//NTSR1//TACR1//VOM1R37 |
| GO:0016504 | peptidase activator activity | Molecular function | 2 | 27 | 158 | 14392 | 6.74730426629161 | 0.0351336164524808 | 0.630297079157506 | 1.45427714413 | NKX3-1//TPD52L1 |
| GO:0004536 | deoxyribonuclease activity | Molecular function | 2 | 28 | 158 | 14392 | 6.50632911392405 | 0.0375687406123665 | 0.64977939886362 | 1.4251733632083 | RBBP8//ERCC5 |
| GO:0005516 | calmodulin binding | Molecular function | 4 | 115 | 158 | 14392 | 3.16829939460649 | 0.037880582637048 | 0.64977939886362 | 1.42158334978204 | MYH3//MYH6//PNCK//KCNN3 |
| GO:0042802 | identical protein binding | Molecular function | 15 | 827 | 158 | 14392 | 1.65215128648616 | 0.0383927626976275 | 0.64977939886362 | 1.41575063544277 | CHRNA7//BHLHA15//TRHR//MYH6//CDH13//ERCC5//TPD52L1//EPOR//KCNN3//CLDN3//GRB7//P2RX2//LCP1//SCUBE1//BNIPL |
| GO:0005158 | insulin receptor binding | Molecular function | 2 | 30 | 158 | 14392 | 6.07257383966245 | 0.0426256274173418 | 0.708059033210289 | 1.37032921546258 | DOK3//FRS3 |
| GO:0005272 | sodium channel activity | Molecular function | 2 | 31 | 158 | 14392 | 5.87668436096366 | 0.0452441134399698 | 0.737890359193689 | 1.34443791738342 | SCN11A//SCN9A |
| GO:0022843 | voltage-gated cation channel activity | Molecular function | 4 | 125 | 158 | 14392 | 2.91483544303797 | 0.0488961091789171 | 0.78321089166944 | 1.3107256977139 | KCNJ16//SCN11A//SCN9A//HTR3A |
