# Supplementary material for: Gene Expression in the Hippocampus in a Rat Model of Premenstrual Dysphoric Disorder After Treatment With Baixiangdan Capsules
Source: Front Psychol. 2018 Nov 13;9:2065. doi: 10.3389/fpsyg.2018.02065 (PMC6242977; doi:10.3389/fpsyg.2018.02065)
Supplement: Supplementary file 3 [file Data_Sheet_3.ZIP › Data Analysis Folder/GO Analysis Report/BXD vs fluoxetine (down)/BP_result(Rat).html]

| GO.ID | Term | Ontology | Count | Pop.Hits | List.Total | Pop.Total | Fold.Enrichment | Pvalue | FDR | Enrichment.Score | GENES |
| --- | --- | --- | --- | --- | --- | --- | --- | --- | --- | --- | --- |
| GO:0031589 | cell-substrate adhesion | Biological process | 6 | 180 | 26 | 13692 | 17.5538461538462 | 8.78791305088824e-07 | 0.00195744356030682 | 6.056114248714 | COL3A1//SPP1//RGD1562717//COL1A1//LGALS1//MMP14 |
| GO:0010810 | regulation of cell-substrate adhesion | Biological process | 5 | 96 | 26 | 13692 | 27.4278846153846 | 8.92994324957493e-07 | 0.00195744356030682 | 6.04915130107503 | SPP1//RGD1562717//COL1A1//LGALS1//MMP14 |
| GO:0001501 | skeletal system development | Biological process | 7 | 333 | 26 | 13692 | 11.0699930699931 | 2.08767825042307e-06 | 0.00305079381661825 | 5.68033643327181 | COL1A1//TGFBI//MGP//LUM//TIMP1//LGALS3//COL3A1 |
| GO:0009611 | response to wounding | Biological process | 9 | 688 | 26 | 13692 | 6.88886404293381 | 2.79930414025851e-06 | 0.00306803733772333 | 5.5529499135166 | ANXA1//PLSCR1//F5//SPP1//COL1A1//COL3A1//TIMP1//LGALS1//TGM2 |
| GO:0030155 | regulation of cell adhesion | Biological process | 6 | 241 | 26 | 13692 | 13.1107564634536 | 4.78877341984961e-06 | 0.00373348347965505 | 5.31977571105448 | SPP1//RGD1562717//COL1A1//LGALS1//TGM2//MMP14 |
| GO:0030198 | extracellular matrix organization | Biological process | 5 | 140 | 26 | 13692 | 18.8076923076923 | 5.75730247335309e-06 | 0.00373348347965505 | 5.23978095326896 | COL1A1//COL3A1//LGALS3//TGFBI//RGD1562717 |
| GO:0043062 | extracellular structure organization | Biological process | 5 | 141 | 26 | 13692 | 18.6743044189853 | 5.96131030054411e-06 | 0.00373348347965505 | 5.22465827150945 | LGALS3//TGFBI//RGD1562717//COL1A1//COL3A1 |
| GO:0051216 | cartilage development | Biological process | 5 | 150 | 26 | 13692 | 17.5538461538462 | 8.06438501796186e-06 | 0.0044192829898431 | 5.09342874588062 | TGFBI//COL1A1//MGP//LUM//TIMP1 |
| GO:0018149 | peptide cross-linking | Biological process | 3 | 28 | 26 | 13692 | 56.4230769230769 | 1.92964260288162e-05 | 0.00745949095727062 | 4.71452312102825 | TGM2//ANXA1//COL3A1 |
| GO:0010812 | negative regulation of cell-substrate adhesion | Biological process | 3 | 29 | 26 | 13692 | 54.4774535809019 | 2.14958326567746e-05 | 0.00745949095727062 | 4.66764572750726 | MMP14//COL1A1//LGALS1 |
| GO:0048513 | organ development | Biological process | 14 | 2341 | 26 | 13692 | 3.14934446160418 | 2.16439277989453e-05 | 0.00745949095727062 | 4.66466392348146 | MMP14//CP//COL1A1//TGFBI//LGALS1//COL3A1//PLSCR1//ANXA1//MGP//SPP1//TIMP1//LUM//TGM2 |
| GO:0061448 | connective tissue development | Biological process | 5 | 184 | 26 | 13692 | 14.3102006688963 | 2.17112812437071e-05 | 0.00745949095727062 | 4.66331454685174 | TGFBI//MGP//LUM//TIMP1//COL1A1 |
| GO:0007155 | cell adhesion | Biological process | 8 | 679 | 26 | 13692 | 6.20459952418715 | 2.47235298473843e-05 | 0.00745949095727062 | 4.60688952372073 | COL3A1//SPP1//RGD1562717//COL1A1//LGALS1//TGM2//MMP14//TGFBI |
| GO:0009888 | tissue development | Biological process | 10 | 1149 | 26 | 13692 | 4.5832496485238 | 2.53564519994154e-05 | 0.00745949095727062 | 4.59591151517809 | TGFBI//DAB2//COL1A1//ANXA1//MGP//SPP1//COL3A1//LUM//TIMP1//TGM2 |
| GO:0022610 | biological adhesion | Biological process | 8 | 682 | 26 | 13692 | 6.17730656440334 | 2.55228933300774e-05 | 0.00745949095727062 | 4.59307009460365 | SPP1//TGFBI//COL3A1//RGD1562717//COL1A1//LGALS1//TGM2//MMP14 |
| GO:0006950 | response to stress | Biological process | 13 | 2176 | 26 | 13692 | 3.14613970588235 | 5.34611876745313e-05 | 0.0146483654228216 | 4.27196139734219 | MMP14//ANXA1//PLSCR1//F5//SPP1//CFD//LGALS1//COL1A1//COL3A1//TIMP1//LGALS3//TGM2//IFITM1 |
| GO:0045785 | positive regulation of cell adhesion | Biological process | 4 | 120 | 26 | 13692 | 17.5538461538462 | 7.22634603720695e-05 | 0.0186354711924207 | 4.14108124586082 | SPP1//RGD1562717//LGALS1//TGM2 |
| GO:0007275 | multicellular organismal development | Biological process | 16 | 3429 | 26 | 13692 | 2.45723130762501 | 8.64672427986032e-05 | 0.0210595773571709 | 4.06314838925432 | COL1A1//LGALS3//COL3A1//MMP14//TGFBI//DAB2//IFITM1//CP//LGALS1//PLSCR1//ANXA1//MGP//SPP1//TIMP1//LUM//TGM2 |
| GO:0032502 | developmental process | Biological process | 17 | 3887 | 26 | 13692 | 2.30318022599988 | 9.51591896770143e-05 | 0.02195673092337 | 4.02154926483044 | DAB2//COL1A1//LGALS3//COL3A1//MMP14//TGFBI//SPP1//IFITM1//CP//LGALS1//TIMP1//PLSCR1//MGP//ANXA1//LRG1//LUM//TGM2 |
| GO:0048731 | system development | Biological process | 15 | 3069 | 26 | 13692 | 2.57387773516806 | 0.000104464436506094 | 0.0228986044821358 | 3.98103153403032 | COL1A1//LGALS3//COL3A1//MMP14//TGFBI//CP//LGALS1//PLSCR1//ANXA1//MGP//SPP1//TIMP1//LUM//TGM2 |
| GO:0048856 | anatomical structure development | Biological process | 16 | 3546 | 26 | 13692 | 2.37615514772875 | 0.000133413571325683 | 0.0278516712710378 | 3.8747999901191 | DAB2//COL1A1//LGALS3//COL3A1//MMP14//TGFBI//IFITM1//CP//LGALS1//PLSCR1//ANXA1//MGP//SPP1//TIMP1//LUM//TGM2 |
| GO:0010033 | response to organic substance | Biological process | 11 | 1714 | 26 | 13692 | 3.37967866439278 | 0.000146137461841477 | 0.0291212105778652 | 3.8352384398799 | MGP//ANXA1//MMP14//LUM//COL1A1//COL3A1//TIMP1//PLSCR1//SPP1//PTGDS//LGALS1 |
| GO:0048545 | response to steroid hormone stimulus | Biological process | 6 | 477 | 26 | 13692 | 6.62409288824383 | 0.000220114557673387 | 0.0419557487321795 | 3.65735123364771 | ANXA1//COL1A1//MMP14//MGP//PTGDS//SPP1 |
| GO:0002376 | immune system process | Biological process | 9 | 1218 | 26 | 13692 | 3.89124668435013 | 0.000262020477478649 | 0.0478624072194332 | 3.58166476628156 | ANXA1//LGALS1//PLSCR1//CFD//SPP1//TIMP1//LGALS3//COL3A1//IFITM1 |
| GO:0006952 | defense response | Biological process | 7 | 711 | 26 | 13692 | 5.18468029860435 | 0.00027301530412875 | 0.0478759637320176 | 3.56381300749267 | ANXA1//PLSCR1//SPP1//CFD//LGALS3//TGM2//IFITM1 |
| GO:0009719 | response to endogenous stimulus | Biological process | 8 | 987 | 26 | 13692 | 4.26841243862521 | 0.000342942932193148 | 0.0578254544128754 | 3.46477814320977 | MGP//ANXA1//MMP14//COL1A1//COL3A1//TIMP1//SPP1//PTGDS |
| GO:0048583 | regulation of response to stimulus | Biological process | 11 | 1898 | 26 | 13692 | 3.05203858312394 | 0.000365996225491597 | 0.0594269426872282 | 3.43652339344882 | ANXA1//CFD//IL22RA2//TGM2//LGALS1//PLSCR1//PTGDS//SPP1//COL3A1//DAB2//COL1A1 |
| GO:0007162 | negative regulation of cell adhesion | Biological process | 3 | 77 | 26 | 13692 | 20.5174825174825 | 0.000405088016229438 | 0.0634252093982092 | 3.39245060441555 | COL1A1//LGALS1//MMP14 |
| GO:0030154 | cell differentiation | Biological process | 12 | 2291 | 26 | 13692 | 2.75835208004566 | 0.000446616492136353 | 0.0675160931560611 | 3.35006524384504 | DAB2//SPP1//COL1A1//TGFBI//LGALS1//PLSCR1//ANXA1//TIMP1//MMP14//LRG1//MGP//LGALS3 |
| GO:0009725 | response to hormone stimulus | Biological process | 7 | 801 | 26 | 13692 | 4.60213195044656 | 0.000563489891381035 | 0.0823446561271486 | 3.24911387049066 | ANXA1//COL1A1//TIMP1//MMP14//SPP1//MGP//PTGDS |
| GO:0001516 | prostaglandin biosynthetic process | Biological process | 2 | 20 | 26 | 13692 | 52.6615384615385 | 0.000645104887910455 | 0.0857012069272556 | 3.19036966747737 | ANXA1//PTGDS |
| GO:0043277 | apoptotic cell clearance | Biological process | 2 | 20 | 26 | 13692 | 52.6615384615385 | 0.000645104887910455 | 0.0857012069272556 | 3.19036966747737 | ANXA1//TGM2 |
| GO:0046457 | prostanoid biosynthetic process | Biological process | 2 | 20 | 26 | 13692 | 52.6615384615385 | 0.000645104887910455 | 0.0857012069272556 | 3.19036966747737 | PTGDS//ANXA1 |
| GO:0048869 | cellular developmental process | Biological process | 12 | 2442 | 26 | 13692 | 2.58779058779059 | 0.000814966326429622 | 0.105082716913749 | 3.08886033549061 | DAB2//SPP1//COL1A1//TGFBI//LGALS1//PLSCR1//MGP//LGALS3//ANXA1//TIMP1//MMP14//LRG1 |
| GO:0031960 | response to corticosteroid stimulus | Biological process | 4 | 232 | 26 | 13692 | 9.07957559681698 | 0.000895672546305831 | 0.11218938408585 | 3.04785073735259 | MGP//ANXA1//PTGDS//COL1A1 |
| GO:0031214 | biomineral tissue development | Biological process | 3 | 104 | 26 | 13692 | 15.1908284023669 | 0.000974774888559221 | 0.118705919762323 | 3.01109566732137 | MGP//COL1A1//SPP1 |
| GO:0006692 | prostanoid metabolic process | Biological process | 2 | 27 | 26 | 13692 | 39.008547008547 | 0.00118204939667612 | 0.13637117250074 | 2.92736437433834 | PTGDS//ANXA1 |
| GO:0006693 | prostaglandin metabolic process | Biological process | 2 | 27 | 26 | 13692 | 39.008547008547 | 0.00118204939667612 | 0.13637117250074 | 2.92736437433834 | PTGDS//ANXA1 |
| GO:0032964 | collagen biosynthetic process | Biological process | 2 | 29 | 26 | 13692 | 36.3183023872679 | 0.00136408423659998 | 0.153337058801393 | 2.86515880976682 | COL1A1//COL3A1 |
| GO:0030199 | collagen fibril organization | Biological process | 2 | 32 | 26 | 13692 | 32.9134615384615 | 0.00166064636712433 | 0.182006841836827 | 2.77972284025047 | COL1A1//COL3A1 |
| GO:0042060 | wound healing | Biological process | 4 | 276 | 26 | 13692 | 7.63210702341137 | 0.00170227543240872 | 0.182018914528776 | 2.76897016863603 | F5//COL1A1//COL3A1//TIMP1 |
| GO:0001503 | ossification | Biological process | 4 | 279 | 26 | 13692 | 7.55004135649297 | 0.00177106557552078 | 0.184838363040185 | 2.75176535831327 | SPP1//COL1A1//MGP//MMP14 |
| GO:0048771 | tissue remodeling | Biological process | 3 | 129 | 26 | 13692 | 12.2468694096601 | 0.00181296752069525 | 0.184838363040185 | 2.74160997621843 | TGM2//SPP1//MMP14 |
| GO:0032091 | negative regulation of protein binding | Biological process | 2 | 35 | 26 | 13692 | 30.0923076923077 | 0.00198514942778159 | 0.197793070258966 | 2.70220679710314 | DAB2//PLSCR1 |
| GO:0010977 | negative regulation of neuron projection development | Biological process | 2 | 36 | 26 | 13692 | 29.2564102564103 | 0.00209947383414627 | 0.200088984541245 | 2.67788953362747 | SPP1//LGALS1 |
| GO:0071230 | cellular response to amino acid stimulus | Biological process | 2 | 36 | 26 | 13692 | 29.2564102564103 | 0.00209947383414627 | 0.200088984541245 | 2.67788953362747 | COL1A1//COL3A1 |
| GO:0042176 | regulation of protein catabolic process | Biological process | 3 | 140 | 26 | 13692 | 11.2846153846154 | 0.00228985094860821 | 0.213589501248902 | 2.64019278591501 | DAB2//TIMP1//SERPINB1A |
| GO:0046456 | icosanoid biosynthetic process | Biological process | 2 | 39 | 26 | 13692 | 27.0059171597633 | 0.00246076108395463 | 0.220162787593002 | 2.6089305500343 | PTGDS//ANXA1 |
| GO:0072376 | protein activation cascade | Biological process | 2 | 39 | 26 | 13692 | 27.0059171597633 | 0.00246076108395463 | 0.220162787593002 | 2.6089305500343 | CFD//F5 |
| GO:0006636 | unsaturated fatty acid biosynthetic process | Biological process | 2 | 42 | 26 | 13692 | 25.0769230769231 | 0.0028492866807337 | 0.249825456166731 | 2.54526385206027 | PTGDS//ANXA1 |
| GO:0030162 | regulation of proteolysis | Biological process | 3 | 153 | 26 | 13692 | 10.3257918552036 | 0.00294582854044593 | 0.250960026671028 | 2.53079253451485 | DAB2//TIMP1//SERPINB1A |
| GO:0071229 | cellular response to acid | Biological process | 2 | 43 | 26 | 13692 | 24.4937388193202 | 0.00298479673214625 | 0.250960026671028 | 2.52508523944687 | COL1A1//COL3A1 |
| GO:0016043 | cellular component organization | Biological process | 13 | 3233 | 26 | 13692 | 2.11753789050418 | 0.00303396017645175 | 0.250960026671028 | 2.51799012403075 | DAB2//MGP//PLSCR1//COL1A1//LGALS1//LGALS3//TGFBI//RGD1562717//COL3A1//ANXA1//TGM2//SPP1//MMP14 |
| GO:0007584 | response to nutrient | Biological process | 4 | 327 | 26 | 13692 | 6.44177840508116 | 0.00315167818753115 | 0.255869577298825 | 2.50145813398533 | SPP1//COL1A1//CP//MGP |
| GO:0002252 | immune effector process | Biological process | 4 | 332 | 26 | 13692 | 6.34476367006488 | 0.00332833771369014 | 0.265298773396683 | 2.47777261394653 | LGALS1//CFD//PLSCR1//IFITM1 |
| GO:0032963 | collagen metabolic process | Biological process | 2 | 47 | 26 | 13692 | 22.4091653027823 | 0.00355655261571058 | 0.278427261915628 | 2.44897076195735 | COL1A1//COL3A1 |
| GO:0071418 | cellular response to amine stimulus | Biological process | 2 | 48 | 26 | 13692 | 21.9423076923077 | 0.00370686608649296 | 0.28510352496816 | 2.43099310292313 | COL1A1//COL3A1 |
| GO:0009612 | response to mechanical stimulus | Biological process | 3 | 170 | 26 | 13692 | 9.29321266968326 | 0.00396345215923972 | 0.289444529131391 | 2.40192637958074 | COL1A1//MGP//MMP14 |
| GO:0044259 | multicellular organismal macromolecule metabolic process | Biological process | 2 | 50 | 26 | 13692 | 21.0646153846154 | 0.0040162666306532 | 0.289444529131391 | 2.39617746309969 | COL1A1//COL3A1 |
| GO:0009605 | response to external stimulus | Biological process | 7 | 1128 | 26 | 13692 | 3.26800327332242 | 0.00412165814274269 | 0.289444529131391 | 2.3849280321777 | ANXA1//MGP//COL1A1//MMP14//CP//SPP1//TGM2 |
| GO:0071840 | cellular component organization or biogenesis | Biological process | 13 | 3342 | 26 | 13692 | 2.04847396768402 | 0.00414261401870195 | 0.289444529131391 | 2.38272552949604 | DAB2//MGP//PLSCR1//COL1A1//LGALS1//LGALS3//TGFBI//RGD1562717//COL3A1//ANXA1//TGM2//SPP1//MMP14 |
| GO:0030324 | lung development | Biological process | 3 | 173 | 26 | 13692 | 9.13205869275233 | 0.00416259916537305 | 0.289444529131391 | 2.38063540718861 | CP//MGP//MMP14 |
| GO:2000242 | negative regulation of reproductive process | Biological process | 2 | 51 | 26 | 13692 | 20.6515837104072 | 0.00417533215870381 | 0.289444529131391 | 2.37930896953269 | PLSCR1//TIMP1 |
| GO:0030323 | respiratory tube development | Biological process | 3 | 175 | 26 | 13692 | 9.02769230769231 | 0.00429870427187995 | 0.289444529131391 | 2.36666243101892 | CP//MGP//MMP14 |
| GO:0043588 | skin development | Biological process | 2 | 52 | 26 | 13692 | 20.2544378698225 | 0.004337293539935 | 0.289444529131391 | 2.36278118461628 | COL1A1//COL3A1 |
| GO:0014070 | response to organic cyclic compound | Biological process | 4 | 358 | 26 | 13692 | 5.88397077782553 | 0.00435751344039047 | 0.289444529131391 | 2.36076126467043 | LGALS1//ANXA1//LUM//MMP14 |
| GO:0071417 | cellular response to organic nitrogen | Biological process | 2 | 53 | 26 | 13692 | 19.8722786647315 | 0.00450214007072086 | 0.294587792090153 | 2.34658099734198 | COL1A1//COL3A1 |
| GO:0010811 | positive regulation of cell-substrate adhesion | Biological process | 2 | 54 | 26 | 13692 | 19.5042735042735 | 0.00466986107494295 | 0.301068690478675 | 2.33069603919473 | SPP1//RGD1562717 |
| GO:0032501 | multicellular organismal process | Biological process | 18 | 5780 | 26 | 13692 | 1.63997870641469 | 0.00491377065986795 | 0.309713822970756 | 2.30858511718401 | COL1A1//LGALS3//COL3A1//MGP//MMP14//TGFBI//SPP1//DAB2//IFITM1//CP//TGM2//LGALS1//F5//PLSCR1//ANXA1//TIMP1//PTGDS//LUM |
| GO:0006954 | inflammatory response | Biological process | 4 | 371 | 26 | 13692 | 5.677793904209 | 0.00494524808575568 | 0.309713822970756 | 2.30581191648901 | ANXA1//PLSCR1//TGM2//SPP1 |
| GO:0009628 | response to abiotic stimulus | Biological process | 6 | 873 | 26 | 13692 | 3.61934972244251 | 0.00504970415120888 | 0.311801450688729 | 2.2967340652994 | MMP14//MGP//COL1A1//COL3A1//ANXA1//PLSCR1 |
| GO:0008544 | epidermis development | Biological process | 3 | 195 | 26 | 13692 | 8.10177514792899 | 0.00581037537394012 | 0.346249953486104 | 2.23579580950902 | ANXA1//COL1A1//COL3A1 |
| GO:0060541 | respiratory system development | Biological process | 3 | 195 | 26 | 13692 | 8.10177514792899 | 0.00581037537394012 | 0.346249953486104 | 2.23579580950902 | CP//MGP//MMP14 |
| GO:0034097 | response to cytokine stimulus | Biological process | 4 | 389 | 26 | 13692 | 5.41506822226617 | 0.00584454757253004 | 0.346249953486104 | 2.23324910201429 | PLSCR1//ANXA1//COL3A1//TIMP1 |
| GO:0001763 | morphogenesis of a branching structure | Biological process | 3 | 197 | 26 | 13692 | 8.01952362358454 | 0.00597693442536881 | 0.349371740277558 | 2.22352150890728 | MGP//MMP14//TGM2 |
| GO:0006690 | icosanoid metabolic process | Biological process | 2 | 63 | 26 | 13692 | 16.7179487179487 | 0.00630695339766212 | 0.359086801238321 | 2.2001803780146 | PTGDS//ANXA1 |
| GO:0044236 | multicellular organismal metabolic process | Biological process | 2 | 63 | 26 | 13692 | 16.7179487179487 | 0.00630695339766212 | 0.359086801238321 | 2.2001803780146 | COL1A1//COL3A1 |
| GO:0006955 | immune response | Biological process | 5 | 641 | 26 | 13692 | 4.10776431057242 | 0.0064213149913817 | 0.36091083233612 | 2.19237602566619 | LGALS1//CFD//LGALS3//PLSCR1//COL3A1 |
| GO:0030177 | positive regulation of Wnt receptor signaling pathway | Biological process | 2 | 65 | 26 | 13692 | 16.203550295858 | 0.00670148135475053 | 0.3718898007497 | 2.1738291863912 | COL1A1//DAB2 |
| GO:0008610 | lipid biosynthetic process | Biological process | 4 | 406 | 26 | 13692 | 5.18832891246684 | 0.00678940304810708 | 0.372059287036268 | 2.16816840897576 | PTGDS//PLSCR1//TIMP1//ANXA1 |
| GO:0033559 | unsaturated fatty acid metabolic process | Biological process | 2 | 66 | 26 | 13692 | 15.958041958042 | 0.0069028732346254 | 0.373607361241948 | 2.16097010198591 | PTGDS//ANXA1 |
| GO:0051100 | negative regulation of binding | Biological process | 2 | 69 | 26 | 13692 | 15.2642140468227 | 0.00752343667257164 | 0.401538159205933 | 2.12358373028708 | DAB2//PLSCR1 |
| GO:0048584 | positive regulation of response to stimulus | Biological process | 6 | 950 | 26 | 13692 | 3.32599190283401 | 0.00760211387182766 | 0.401538159205933 | 2.11906562940063 | CFD//TGM2//LGALS1//PLSCR1//COL1A1//DAB2 |
| GO:0048678 | response to axon injury | Biological process | 2 | 71 | 26 | 13692 | 14.8342361863489 | 0.00795069949003629 | 0.411610555335496 | 2.09959466111721 | SPP1//LGALS1 |
| GO:0051384 | response to glucocorticoid stimulus | Biological process | 3 | 219 | 26 | 13692 | 7.21390937829294 | 0.00800033807433786 | 0.411610555335496 | 2.09689166041841 | ANXA1//MGP//PTGDS |
| GO:0050896 | response to stimulus | Biological process | 19 | 6556 | 26 | 13692 | 1.52618857652415 | 0.00816508419904751 | 0.411610555335496 | 2.08803933242233 | MMP14//LGALS1//ANXA1//PLSCR1//F5//MGP//COL1A1//SPP1//CFD//COL3A1//TGM2//CP//IFITM1//TIMP1//LUM//IL22RA2//LGALS3//PTGDS//DAB2 |
| GO:0051607 | defense response to virus | Biological process | 2 | 72 | 26 | 13692 | 14.6282051282051 | 0.00816836640378379 | 0.411610555335496 | 2.0878647895827 | PLSCR1//IFITM1 |
| GO:0031345 | negative regulation of cell projection organization | Biological process | 2 | 74 | 26 | 13692 | 14.2328482328482 | 0.00861172041787011 | 0.429020253544802 | 2.0649100781225 | LGALS1//SPP1 |
| GO:0035295 | tube development | Biological process | 4 | 438 | 26 | 13692 | 4.80927291886196 | 0.00883489081915198 | 0.435192824170363 | 2.05379881309924 | CP//MGP//MMP14//TGM2 |
| GO:0009894 | regulation of catabolic process | Biological process | 4 | 442 | 26 | 13692 | 4.76575008701706 | 0.00911614746231293 | 0.442609320961124 | 2.04018865831057 | DAB2//SERPINB1A//TIMP1//PLSCR1 |
| GO:0001568 | blood vessel development | Biological process | 4 | 443 | 26 | 13692 | 4.75499218614343 | 0.00918737413491385 | 0.442609320961124 | 2.03680859760971 | MMP14//TGFBI//COL1A1//COL3A1 |
| GO:0048511 | rhythmic process | Biological process | 3 | 240 | 26 | 13692 | 6.58269230769231 | 0.0102686511968773 | 0.476042496000268 | 1.98848659790448 | MMP14//PTGDS//ANXA1 |
| GO:0016053 | organic acid biosynthetic process | Biological process | 3 | 241 | 26 | 13692 | 6.55537823172678 | 0.0103850803098358 | 0.476042496000268 | 1.98359014063262 | PTGDS//PLSCR1//ANXA1 |
| GO:0046394 | carboxylic acid biosynthetic process | Biological process | 3 | 241 | 26 | 13692 | 6.55537823172678 | 0.0103850803098358 | 0.476042496000268 | 1.98359014063262 | PTGDS//PLSCR1//ANXA1 |
| GO:0043200 | response to amino acid stimulus | Biological process | 2 | 82 | 26 | 13692 | 12.844277673546 | 0.0104905939846538 | 0.476042496000268 | 1.9791999210577 | COL1A1//COL3A1 |
| GO:0045087 | innate immune response | Biological process | 3 | 242 | 26 | 13692 | 6.52828989192626 | 0.010502282975716 | 0.476042496000268 | 1.97871628416967 | CFD//PLSCR1//LGALS3 |
| GO:0010035 | response to inorganic substance | Biological process | 4 | 461 | 26 | 13692 | 4.56933088603371 | 0.010532874569349 | 0.476042496000268 | 1.97745308757649 | COL1A1//CP//MGP//ANXA1 |
| GO:0001944 | vasculature development | Biological process | 4 | 465 | 26 | 13692 | 4.53002481389578 | 0.0108484462100353 | 0.485301920253008 | 1.96463246003945 | MMP14//TGFBI//COL1A1//COL3A1 |
| GO:0006909 | phagocytosis | Biological process | 2 | 85 | 26 | 13692 | 12.3909502262443 | 0.0112379586408836 | 0.497648592743775 | 1.94931257057437 | TGM2//ANXA1 |
| GO:0030097 | hemopoiesis | Biological process | 4 | 477 | 26 | 13692 | 4.41606192549589 | 0.011832108961594 | 0.518719656876281 | 1.92693783958578 | LGALS1//PLSCR1//TIMP1//ANXA1 |
| GO:0048646 | anatomical structure formation involved in morphogenesis | Biological process | 5 | 750 | 26 | 13692 | 3.51076923076923 | 0.0122519380875699 | 0.531806896791153 | 1.91179520646999 | MMP14//TGFBI//IFITM1//COL1A1//TGM2 |
| GO:0031667 | response to nutrient levels | Biological process | 4 | 490 | 26 | 13692 | 4.2989010989011 | 0.0129613815955028 | 0.557085263869454 | 1.8873487031473 | CP//MGP//COL1A1//SPP1 |
| GO:2000026 | regulation of multicellular organismal development | Biological process | 6 | 1078 | 26 | 13692 | 2.93106893106893 | 0.0137758811540177 | 0.585654013192808 | 1.86088061257864 | COL1A1//LGALS1//MGP//SPP1//TIMP1//DAB2 |
| GO:0043393 | regulation of protein binding | Biological process | 2 | 95 | 26 | 13692 | 11.0866396761134 | 0.0138932521377856 | 0.585654013192808 | 1.85719608254346 | DAB2//PLSCR1 |
| GO:0006979 | response to oxidative stress | Biological process | 3 | 274 | 26 | 13692 | 5.76586187535093 | 0.014666863262901 | 0.60203907200602 | 1.83366275686387 | COL1A1//ANXA1//MMP14 |
| GO:0031347 | regulation of defense response | Biological process | 3 | 275 | 26 | 13692 | 5.74489510489511 | 0.0148101032969394 | 0.60203907200602 | 1.82944191236776 | ANXA1//PLSCR1//TGM2 |
| GO:0051179 | localization | Biological process | 12 | 3407 | 26 | 13692 | 1.85482377909733 | 0.0148179970431318 | 0.60203907200602 | 1.82921049621521 | SLC6A20//PTGDS//DAB2//COL1A1//CP//PLSCR1//MMP14//ANXA1//SPP1//S100A6//TGM2//TIMP1 |
| GO:0048534 | hemopoietic or lymphoid organ development | Biological process | 4 | 510 | 26 | 13692 | 4.13031674208145 | 0.0148312545110972 | 0.60203907200602 | 1.82882211234289 | LGALS1//PLSCR1//TIMP1//ANXA1 |
| GO:0009991 | response to extracellular stimulus | Biological process | 4 | 515 | 26 | 13692 | 4.09021657953697 | 0.0153243242969884 | 0.616347135027497 | 1.81461866594879 | CP//MGP//COL1A1//SPP1 |
| GO:0051128 | regulation of cellular component organization | Biological process | 6 | 1110 | 26 | 13692 | 2.84656964656965 | 0.0157572957325447 | 0.627999859013418 | 1.80251831408042 | COL1A1//LGALS1//DAB2//ANXA1//SPP1//MMP14 |
| GO:0006508 | proteolysis | Biological process | 5 | 804 | 26 | 13692 | 3.27497129735936 | 0.0161847482242264 | 0.632818425598771 | 1.79089405225026 | SERPINB1A//DAB2//TIMP1//CFD//MMP14 |
| GO:0051129 | negative regulation of cellular component organization | Biological process | 3 | 285 | 26 | 13692 | 5.54331983805668 | 0.0162867150993716 | 0.632818425598771 | 1.78816650059482 | LGALS1//SPP1//MMP14 |
| GO:0009653 | anatomical structure morphogenesis | Biological process | 8 | 1819 | 26 | 13692 | 2.31606546284941 | 0.016311241353253 | 0.632818425598771 | 1.78751298608707 | DAB2//MMP14//TGFBI//IFITM1//COL1A1//SPP1//MGP//TGM2 |
| GO:0042592 | homeostatic process | Biological process | 6 | 1125 | 26 | 13692 | 2.80861538461538 | 0.0167524107695645 | 0.640200492401648 | 1.77592268662225 | ANXA1//CP//TIMP1//SPP1//TGM2//LGALS1 |
| GO:0002526 | acute inflammatory response | Biological process | 2 | 105 | 26 | 13692 | 10.0307692307692 | 0.016793580434806 | 0.640200492401648 | 1.77485670128267 | ANXA1//PLSCR1 |
| GO:0035239 | tube morphogenesis | Biological process | 3 | 291 | 26 | 13692 | 5.42902458366376 | 0.0172114197763644 | 0.650472968099841 | 1.76418330308278 | MGP//MMP14//TGM2 |
| GO:0022602 | ovulation cycle process | Biological process | 2 | 108 | 26 | 13692 | 9.75213675213675 | 0.0177100434415352 | 0.6516439936735 | 1.75178037351371 | MMP14//ANXA1 |
| GO:0042542 | response to hydrogen peroxide | Biological process | 2 | 108 | 26 | 13692 | 9.75213675213675 | 0.0177100434415352 | 0.6516439936735 | 1.75178037351371 | ANXA1//COL1A1 |
| GO:2000241 | regulation of reproductive process | Biological process | 2 | 108 | 26 | 13692 | 9.75213675213675 | 0.0177100434415352 | 0.6516439936735 | 1.75178037351371 | PLSCR1//TIMP1 |
| GO:0002520 | immune system development | Biological process | 4 | 539 | 26 | 13692 | 3.90809190809191 | 0.0178369706297491 | 0.6516439936735 | 1.74867890279056 | LGALS1//PLSCR1//TIMP1//ANXA1 |
| GO:0002335 | mature B cell differentiation | Biological process | 1 | 10 | 26 | 13692 | 52.6615384615385 | 0.0188338824446621 | 0.655299528868243 | 1.72506014464127 | LGALS1 |
| GO:0002674 | negative regulation of acute inflammatory response | Biological process | 1 | 10 | 26 | 13692 | 52.6615384615385 | 0.0188338824446621 | 0.655299528868243 | 1.72506014464127 | ANXA1 |
| GO:0043249 | erythrocyte maturation | Biological process | 1 | 10 | 26 | 13692 | 52.6615384615385 | 0.0188338824446621 | 0.655299528868243 | 1.72506014464127 | TIMP1 |
| GO:0043589 | skin morphogenesis | Biological process | 1 | 10 | 26 | 13692 | 52.6615384615385 | 0.0188338824446621 | 0.655299528868243 | 1.72506014464127 | COL1A1 |
| GO:0048668 | collateral sprouting | Biological process | 1 | 10 | 26 | 13692 | 52.6615384615385 | 0.0188338824446621 | 0.655299528868243 | 1.72506014464127 | SPP1 |
| GO:2000050 | regulation of non-canonical Wnt receptor signaling pathway | Biological process | 1 | 10 | 26 | 13692 | 52.6615384615385 | 0.0188338824446621 | 0.655299528868243 | 1.72506014464127 | DAB2 |
| GO:0007160 | cell-matrix adhesion | Biological process | 2 | 113 | 26 | 13692 | 9.32062627637849 | 0.0192839182850792 | 0.663916993948235 | 1.71480471749262 | MMP14//COL3A1 |
| GO:0080134 | regulation of response to stress | Biological process | 4 | 554 | 26 | 13692 | 3.80227714523743 | 0.0195323669212973 | 0.663916993948235 | 1.70924512596082 | ANXA1//PLSCR1//SPP1//TGM2 |
| GO:0001775 | cell activation | Biological process | 4 | 555 | 26 | 13692 | 3.7954261954262 | 0.0196488671196916 | 0.663916993948235 | 1.70666248436488 | LGALS1//PLSCR1//ANXA1//TIMP1 |
| GO:0044283 | small molecule biosynthetic process | Biological process | 3 | 307 | 26 | 13692 | 5.1460786770233 | 0.0198202235515909 | 0.663916993948235 | 1.70289145143123 | PTGDS//PLSCR1//ANXA1 |
| GO:0001101 | response to acid | Biological process | 2 | 115 | 26 | 13692 | 9.15852842809365 | 0.0199295066761422 | 0.663916993948235 | 1.70050345144908 | COL1A1//COL3A1 |
| GO:0006897 | endocytosis | Biological process | 3 | 308 | 26 | 13692 | 5.12937062937063 | 0.0199902014601202 | 0.663916993948235 | 1.69918282906459 | DAB2//TGM2//ANXA1 |
| GO:0006633 | fatty acid biosynthetic process | Biological process | 2 | 117 | 26 | 13692 | 9.00197238658777 | 0.0205841505449711 | 0.665005523006235 | 1.68646705051462 | PTGDS//ANXA1 |
| GO:0010225 | response to UV-C | Biological process | 1 | 11 | 26 | 13692 | 47.8741258741259 | 0.0206983992591949 | 0.665005523006235 | 1.68406324003969 | PLSCR1 |
| GO:0070208 | protein heterotrimerization | Biological process | 1 | 11 | 26 | 13692 | 47.8741258741259 | 0.0206983992591949 | 0.665005523006235 | 1.68406324003969 | COL1A1 |
| GO:0070887 | cellular response to chemical stimulus | Biological process | 6 | 1182 | 26 | 13692 | 2.67317454119485 | 0.0209397083768428 | 0.665005523006235 | 1.679029370948 | COL1A1//COL3A1//SPP1//ANXA1//LGALS1//PLSCR1 |
| GO:0009314 | response to radiation | Biological process | 3 | 317 | 26 | 13692 | 4.98374181024023 | 0.0215567965014712 | 0.665005523006235 | 1.66641577804488 | ANXA1//PLSCR1//COL3A1 |
| GO:0042698 | ovulation cycle | Biological process | 2 | 120 | 26 | 13692 | 8.77692307692308 | 0.0215829371555039 | 0.665005523006235 | 1.66588945383159 | MMP14//ANXA1 |
| GO:0043123 | positive regulation of I-kappaB kinase/NF-kappaB cascade | Biological process | 2 | 120 | 26 | 13692 | 8.77692307692308 | 0.0215829371555039 | 0.665005523006235 | 1.66588945383159 | TGM2//LGALS1 |
| GO:0060828 | regulation of canonical Wnt receptor signaling pathway | Biological process | 2 | 120 | 26 | 13692 | 8.77692307692308 | 0.0215829371555039 | 0.665005523006235 | 1.66588945383159 | DAB2//COL1A1 |
| GO:0001889 | liver development | Biological process | 2 | 121 | 26 | 13692 | 8.70438652256834 | 0.0219203156020192 | 0.665005523006235 | 1.65915319730315 | ANXA1//CP |
| GO:0016049 | cell growth | Biological process | 3 | 320 | 26 | 13692 | 4.93701923076923 | 0.022093730348739 | 0.665005523006235 | 1.65573095080592 | DAB2//SPP1//EMP3 |
| GO:0032102 | negative regulation of response to external stimulus | Biological process | 2 | 122 | 26 | 13692 | 8.63303909205549 | 0.02225990391755 | 0.665005523006235 | 1.65247671458208 | ANXA1//SPP1 |
| GO:0006825 | copper ion transport | Biological process | 1 | 12 | 26 | 13692 | 43.8846153846154 | 0.0225595089455672 | 0.665005523006235 | 1.64667035790463 | CP |
| GO:0034505 | tooth mineralization | Biological process | 1 | 12 | 26 | 13692 | 43.8846153846154 | 0.0225595089455672 | 0.665005523006235 | 1.64667035790463 | COL1A1 |
| GO:0035456 | response to interferon-beta | Biological process | 1 | 12 | 26 | 13692 | 43.8846153846154 | 0.0225595089455672 | 0.665005523006235 | 1.64667035790463 | PLSCR1 |
| GO:0060206 | estrous cycle phase | Biological process | 1 | 12 | 26 | 13692 | 43.8846153846154 | 0.0225595089455672 | 0.665005523006235 | 1.64667035790463 | ANXA1 |
| GO:0070365 | hepatocyte differentiation | Biological process | 1 | 12 | 26 | 13692 | 43.8846153846154 | 0.0225595089455672 | 0.665005523006235 | 1.64667035790463 | ANXA1 |
| GO:0061008 | hepaticobiliary system development | Biological process | 2 | 123 | 26 | 13692 | 8.56285178236398 | 0.0226016931861152 | 0.665005523006235 | 1.64585902484188 | CP//ANXA1 |
| GO:0071310 | cellular response to organic substance | Biological process | 5 | 877 | 26 | 13692 | 3.00236821331462 | 0.0227542822158626 | 0.665031821562278 | 1.64293685976676 | COL1A1//COL3A1//LGALS1//PLSCR1//ANXA1 |
| GO:0030308 | negative regulation of cell growth | Biological process | 2 | 125 | 26 | 13692 | 8.42584615384616 | 0.0232918390363777 | 0.676234585003178 | 1.63279621984576 | SPP1//DAB2 |
| GO:0042221 | response to chemical stimulus | Biological process | 12 | 3628 | 26 | 13692 | 1.7418369943177 | 0.0242483508989182 | 0.686186419471576 | 1.61531779190102 | CP//MGP//COL1A1//ANXA1//LGALS1//MMP14//SPP1//TIMP1//LUM//COL3A1//PLSCR1//PTGDS |
| GO:0003333 | amino acid transmembrane transport | Biological process | 1 | 13 | 26 | 13692 | 40.508875739645 | 0.0244172174811966 | 0.686186419471576 | 1.61230382857354 | SLC6A20 |
| GO:0032781 | positive regulation of ATPase activity | Biological process | 1 | 13 | 26 | 13692 | 40.508875739645 | 0.0244172174811966 | 0.686186419471576 | 1.61230382857354 | PLSCR1 |
| GO:0048679 | regulation of axon regeneration | Biological process | 1 | 13 | 26 | 13692 | 40.508875739645 | 0.0244172174811966 | 0.686186419471576 | 1.61230382857354 | SPP1 |
| GO:0070570 | regulation of neuron projection regeneration | Biological process | 1 | 13 | 26 | 13692 | 40.508875739645 | 0.0244172174811966 | 0.686186419471576 | 1.61230382857354 | SPP1 |
| GO:0043434 | response to peptide hormone stimulus | Biological process | 3 | 334 | 26 | 13692 | 4.7300783049286 | 0.0246970102070233 | 0.689628616226689 | 1.60735561877204 | ANXA1//COL1A1//TIMP1 |
| GO:0014075 | response to amine stimulus | Biological process | 2 | 132 | 26 | 13692 | 7.97902097902098 | 0.0257753799942677 | 0.706591356894796 | 1.58879492339858 | COL1A1//COL3A1 |
| GO:0050776 | regulation of immune response | Biological process | 3 | 340 | 26 | 13692 | 4.64660633484163 | 0.0258619476224457 | 0.706591356894796 | 1.58733877219226 | CFD//PLSCR1//COL3A1 |
| GO:0032101 | regulation of response to external stimulus | Biological process | 3 | 341 | 26 | 13692 | 4.6329799233025 | 0.0260589774343871 | 0.706591356894796 | 1.58404263019351 | ANXA1//SPP1//TGM2 |
| GO:0045723 | positive regulation of fatty acid biosynthetic process | Biological process | 1 | 14 | 26 | 13692 | 37.6153846153846 | 0.0262715308334516 | 0.706591356894796 | 1.58051462028014 | ANXA1 |
| GO:0045780 | positive regulation of bone resorption | Biological process | 1 | 14 | 26 | 13692 | 37.6153846153846 | 0.0262715308334516 | 0.706591356894796 | 1.58051462028014 | SPP1 |
| GO:0046852 | positive regulation of bone remodeling | Biological process | 1 | 14 | 26 | 13692 | 37.6153846153846 | 0.0262715308334516 | 0.706591356894796 | 1.58051462028014 | SPP1 |
| GO:0001649 | osteoblast differentiation | Biological process | 2 | 136 | 26 | 13692 | 7.74434389140271 | 0.0272412263596126 | 0.728204490003303 | 1.56477334502563 | SPP1//COL1A1 |
| GO:0031349 | positive regulation of defense response | Biological process | 2 | 139 | 26 | 13692 | 7.57719977863863 | 0.0283624665305842 | 0.753582141030795 | 1.54725600360031 | PLSCR1//TGM2 |
| GO:0031295 | T cell costimulation | Biological process | 1 | 16 | 26 | 13692 | 32.9134615384615 | 0.029969995807148 | 0.777446518452881 | 1.52331331781287 | LGALS1 |
| GO:0034110 | regulation of homotypic cell-cell adhesion | Biological process | 1 | 16 | 26 | 13692 | 32.9134615384615 | 0.029969995807148 | 0.777446518452881 | 1.52331331781287 | LGALS1 |
| GO:0045071 | negative regulation of viral genome replication | Biological process | 1 | 16 | 26 | 13692 | 32.9134615384615 | 0.029969995807148 | 0.777446518452881 | 1.52331331781287 | PLSCR1 |
| GO:0051043 | regulation of membrane protein ectodomain proteolysis | Biological process | 1 | 16 | 26 | 13692 | 32.9134615384615 | 0.029969995807148 | 0.777446518452881 | 1.52331331781287 | TIMP1 |
| GO:0000302 | response to reactive oxygen species | Biological process | 2 | 144 | 26 | 13692 | 7.31410256410256 | 0.030272146361016 | 0.779912361992564 | 1.51895678552843 | COL1A1//ANXA1 |
| GO:0002682 | regulation of immune system process | Biological process | 4 | 635 | 26 | 13692 | 3.31726226529376 | 0.0304208517109326 | 0.779912361992564 | 1.51682863090826 | CFD//LGALS1//PLSCR1//COL3A1 |
| GO:0008347 | glial cell migration | Biological process | 1 | 17 | 26 | 13692 | 30.9773755656109 | 0.0318141593132185 | 0.788528867067513 | 1.49737954847535 | MMP14 |
| GO:0031294 | lymphocyte costimulation | Biological process | 1 | 17 | 26 | 13692 | 30.9773755656109 | 0.0318141593132185 | 0.788528867067513 | 1.49737954847535 | LGALS1 |
| GO:0031638 | zymogen activation | Biological process | 1 | 17 | 26 | 13692 | 30.9773755656109 | 0.0318141593132185 | 0.788528867067513 | 1.49737954847535 | MMP14 |
| GO:0048525 | negative regulation of viral reproduction | Biological process | 1 | 17 | 26 | 13692 | 30.9773755656109 | 0.0318141593132185 | 0.788528867067513 | 1.49737954847535 | PLSCR1 |
| GO:0051482 | elevation of cytosolic calcium ion concentration involved in phospholipase C-activating G-protein coupled signaling pathway | Biological process | 1 | 17 | 26 | 13692 | 30.9773755656109 | 0.0318141593132185 | 0.788528867067513 | 1.49737954847535 | TGM2 |
| GO:0043122 | regulation of I-kappaB kinase/NF-kappaB cascade | Biological process | 2 | 148 | 26 | 13692 | 7.11642411642412 | 0.0318361335472057 | 0.788528867067513 | 1.49707968217419 | TGM2//LGALS1 |
| GO:0009615 | response to virus | Biological process | 2 | 152 | 26 | 13692 | 6.92914979757085 | 0.0334317575160511 | 0.819685038668886 | 1.4758407918352 | PLSCR1//IFITM1 |
| GO:0001953 | negative regulation of cell-matrix adhesion | Biological process | 1 | 18 | 26 | 13692 | 29.2564102564103 | 0.0336549514052006 | 0.819685038668886 | 1.47295103218994 | MMP14 |
| GO:0050482 | arachidonic acid secretion | Biological process | 1 | 18 | 26 | 13692 | 29.2564102564103 | 0.0336549514052006 | 0.819685038668886 | 1.47295103218994 | ANXA1 |
| GO:0031329 | regulation of cellular catabolic process | Biological process | 3 | 379 | 26 | 13692 | 4.1684595088289 | 0.0341534845867699 | 0.827231361482869 | 1.46656497984182 | DAB2//TIMP1//PLSCR1 |
| GO:0050793 | regulation of developmental process | Biological process | 6 | 1333 | 26 | 13692 | 2.37036182122454 | 0.0354348016014302 | 0.849728717395577 | 1.45056999449962 | COL1A1//LGALS1//MGP//SPP1//TIMP1//DAB2 |
| GO:0048754 | branching morphogenesis of a tube | Biological process | 2 | 157 | 26 | 13692 | 6.70847623713866 | 0.0354699715518683 | 0.849728717395577 | 1.45013915984696 | MGP//MMP14 |
| GO:0065008 | regulation of biological quality | Biological process | 8 | 2106 | 26 | 13692 | 2.00043830813062 | 0.0365146103162063 | 0.870000280577437 | 1.43753332978699 | ANXA1//F5//CP//PLSCR1//TIMP1//SPP1//TGM2//LGALS1 |
| GO:0009967 | positive regulation of signal transduction | Biological process | 4 | 677 | 26 | 13692 | 3.11146460629474 | 0.0372600156084182 | 0.871051564158222 | 1.42875696751148 | TGM2//LGALS1//COL1A1//DAB2 |
| GO:0034105 | positive regulation of tissue remodeling | Biological process | 1 | 20 | 26 | 13692 | 26.3307692307692 | 0.0373264450063752 | 0.871051564158222 | 1.42798337057822 | SPP1 |
| GO:0060351 | cartilage development involved in endochondral bone morphogenesis | Biological process | 1 | 20 | 26 | 13692 | 26.3307692307692 | 0.0373264450063752 | 0.871051564158222 | 1.42798337057822 | COL1A1 |
| GO:0048585 | negative regulation of response to stimulus | Biological process | 4 | 678 | 26 | 13692 | 3.1068754254595 | 0.0374330135070386 | 0.871051564158222 | 1.42674520903848 | ANXA1//SPP1//COL3A1//DAB2 |
| GO:0006869 | lipid transport | Biological process | 2 | 164 | 26 | 13692 | 6.42213883677298 | 0.0384031897049349 | 0.871051564158222 | 1.41563270235848 | PLSCR1//ANXA1 |
| GO:0071842 | cellular component organization at cellular level | Biological process | 9 | 2544 | 26 | 13692 | 1.86302612481858 | 0.0390354008311902 | 0.871051564158222 | 1.40854135676605 | LGALS1//LGALS3//TGFBI//RGD1562717//COL1A1//COL3A1//ANXA1//SPP1//MMP14 |
| GO:0010718 | positive regulation of epithelial to mesenchymal transition | Biological process | 1 | 21 | 26 | 13692 | 25.0769230769231 | 0.0391571583204354 | 0.871051564158222 | 1.40718883297038 | COL1A1 |
| GO:0042749 | regulation of circadian sleep/wake cycle | Biological process | 1 | 21 | 26 | 13692 | 25.0769230769231 | 0.0391571583204354 | 0.871051564158222 | 1.40718883297038 | PTGDS |
| GO:0043462 | regulation of ATPase activity | Biological process | 1 | 21 | 26 | 13692 | 25.0769230769231 | 0.0391571583204354 | 0.871051564158222 | 1.40718883297038 | PLSCR1 |
| GO:0045187 | regulation of circadian sleep/wake cycle, sleep | Biological process | 1 | 21 | 26 | 13692 | 25.0769230769231 | 0.0391571583204354 | 0.871051564158222 | 1.40718883297038 | PTGDS |
| GO:0051893 | regulation of focal adhesion assembly | Biological process | 1 | 21 | 26 | 13692 | 25.0769230769231 | 0.0391571583204354 | 0.871051564158222 | 1.40718883297038 | MMP14 |
| GO:0090109 | regulation of cell-substrate junction assembly | Biological process | 1 | 21 | 26 | 13692 | 25.0769230769231 | 0.0391571583204354 | 0.871051564158222 | 1.40718883297038 | MMP14 |
| GO:0030111 | regulation of Wnt receptor signaling pathway | Biological process | 2 | 166 | 26 | 13692 | 6.34476367006488 | 0.0392579964878844 | 0.871051564158222 | 1.40607186817103 | DAB2//COL1A1 |
| GO:0002253 | activation of immune response | Biological process | 2 | 167 | 26 | 13692 | 6.30677107323814 | 0.0396881507793317 | 0.871051564158222 | 1.40133913603972 | CFD//PLSCR1 |
| GO:0050727 | regulation of inflammatory response | Biological process | 2 | 167 | 26 | 13692 | 6.30677107323814 | 0.0396881507793317 | 0.871051564158222 | 1.40133913603972 | ANXA1//TGM2 |
| GO:0002684 | positive regulation of immune system process | Biological process | 3 | 403 | 26 | 13692 | 3.92021378125597 | 0.0398720840239183 | 0.871051564158222 | 1.39933106415114 | CFD//LGALS1//PLSCR1 |
| GO:0051239 | regulation of multicellular organismal process | Biological process | 7 | 1747 | 26 | 13692 | 2.11007881643257 | 0.0399364426085316 | 0.871051564158222 | 1.39863062309807 | COL1A1//LGALS1//MGP//PTGDS//SPP1//TIMP1//DAB2 |
| GO:0051098 | regulation of binding | Biological process | 2 | 169 | 26 | 13692 | 6.23213472917615 | 0.0405539216419843 | 0.880140556824055 | 1.39196714231294 | DAB2//PLSCR1 |
| GO:0072358 | cardiovascular system development | Biological process | 4 | 701 | 26 | 13692 | 3.0049380006584 | 0.0415433641192001 | 0.89277504067928 | 1.38149833786236 | MMP14//TGFBI//COL1A1//COL3A1 |
| GO:0072359 | circulatory system development | Biological process | 4 | 701 | 26 | 13692 | 3.0049380006584 | 0.0415433641192001 | 0.89277504067928 | 1.38149833786236 | MMP14//TGFBI//COL1A1//COL3A1 |
| GO:0010769 | regulation of cell morphogenesis involved in differentiation | Biological process | 2 | 173 | 26 | 13692 | 6.08803912850156 | 0.0423070925164232 | 0.893399844590795 | 1.37358681979896 | COL1A1//SPP1 |
| GO:0001958 | endochondral ossification | Biological process | 1 | 23 | 26 | 13692 | 22.8963210702341 | 0.042808547413235 | 0.893399844590795 | 1.36846950846841 | COL1A1 |
| GO:0036075 | replacement ossification | Biological process | 1 | 23 | 26 | 13692 | 22.8963210702341 | 0.042808547413235 | 0.893399844590795 | 1.36846950846841 | COL1A1 |
| GO:0048821 | erythrocyte development | Biological process | 1 | 23 | 26 | 13692 | 22.8963210702341 | 0.042808547413235 | 0.893399844590795 | 1.36846950846841 | TIMP1 |
| GO:0060343 | trabecula formation | Biological process | 1 | 23 | 26 | 13692 | 22.8963210702341 | 0.042808547413235 | 0.893399844590795 | 1.36846950846841 | COL1A1 |
| GO:0030163 | protein catabolic process | Biological process | 3 | 415 | 26 | 13692 | 3.80685820203893 | 0.0429054511446174 | 0.893399844590795 | 1.36748752712456 | DAB2//SERPINB1A//TIMP1 |
| GO:0007249 | I-kappaB kinase/NF-kappaB cascade | Biological process | 2 | 177 | 26 | 13692 | 5.95045632333768 | 0.0440886863749081 | 0.893399844590795 | 1.35567284081141 | TGM2//LGALS1 |
| GO:0060070 | canonical Wnt receptor signaling pathway | Biological process | 2 | 177 | 26 | 13692 | 5.95045632333768 | 0.0440886863749081 | 0.893399844590795 | 1.35567284081141 | DAB2//COL1A1 |
| GO:0045926 | negative regulation of growth | Biological process | 2 | 178 | 26 | 13692 | 5.91702679343129 | 0.0445384678822946 | 0.893399844590795 | 1.3512647267207 | DAB2//SPP1 |
| GO:0048872 | homeostasis of number of cells | Biological process | 2 | 178 | 26 | 13692 | 5.91702679343129 | 0.0445384678822946 | 0.893399844590795 | 1.3512647267207 | ANXA1//TIMP1 |
| GO:0022410 | circadian sleep/wake cycle process | Biological process | 1 | 24 | 26 | 13692 | 21.9423076923077 | 0.0446292349373595 | 0.893399844590795 | 1.35038055804701 | PTGDS |
| GO:0042304 | regulation of fatty acid biosynthetic process | Biological process | 1 | 24 | 26 | 13692 | 21.9423076923077 | 0.0446292349373595 | 0.893399844590795 | 1.35038055804701 | ANXA1 |
| GO:0045069 | regulation of viral genome replication | Biological process | 1 | 24 | 26 | 13692 | 21.9423076923077 | 0.0446292349373595 | 0.893399844590795 | 1.35038055804701 | PLSCR1 |
| GO:0050802 | circadian sleep/wake cycle, sleep | Biological process | 1 | 24 | 26 | 13692 | 21.9423076923077 | 0.0446292349373595 | 0.893399844590795 | 1.35038055804701 | PTGDS |
| GO:0060445 | branching involved in salivary gland morphogenesis | Biological process | 1 | 24 | 26 | 13692 | 21.9423076923077 | 0.0446292349373595 | 0.893399844590795 | 1.35038055804701 | TGM2 |
| GO:0016477 | cell migration | Biological process | 4 | 725 | 26 | 13692 | 2.90546419098143 | 0.0461018447108919 | 0.907838544770637 | 1.33628169648167 | COL1A1//SPP1//MMP14//TIMP1 |
| GO:0042063 | gliogenesis | Biological process | 2 | 182 | 26 | 13692 | 5.78698224852071 | 0.0463548959307943 | 0.907838544770637 | 1.33390438960067 | MMP14//ANXA1 |
| GO:0015914 | phospholipid transport | Biological process | 1 | 25 | 26 | 13692 | 21.0646153846154 | 0.0464465922604228 | 0.907838544770637 | 1.33304614424912 | PLSCR1 |
| GO:0045124 | regulation of bone resorption | Biological process | 1 | 25 | 26 | 13692 | 21.0646153846154 | 0.0464465922604228 | 0.907838544770637 | 1.33304614424912 | SPP1 |
| GO:0048640 | negative regulation of developmental growth | Biological process | 1 | 25 | 26 | 13692 | 21.0646153846154 | 0.0464465922604228 | 0.907838544770637 | 1.33304614424912 | SPP1 |
| GO:0010647 | positive regulation of cell communication | Biological process | 4 | 733 | 26 | 13692 | 2.87375380417672 | 0.0476826360805658 | 0.907838544770637 | 1.32163974312525 | TGM2//LGALS1//COL1A1//DAB2 |
| GO:0046942 | carboxylic acid transport | Biological process | 2 | 185 | 26 | 13692 | 5.69313929313929 | 0.047735171025023 | 0.907838544770637 | 1.321161517111 | SLC6A20//ANXA1 |
| GO:0023056 | positive regulation of signaling | Biological process | 4 | 734 | 26 | 13692 | 2.86983860825823 | 0.0478823915052927 | 0.907838544770637 | 1.31982416671144 | TGM2//LGALS1//COL1A1//DAB2 |
| GO:0071841 | cellular component organization or biogenesis at cellular level | Biological process | 9 | 2640 | 26 | 13692 | 1.79527972027972 | 0.0482019676470052 | 0.907838544770637 | 1.31693523311395 | LGALS1//LGALS3//TGFBI//RGD1562717//COL1A1//COL3A1//ANXA1//SPP1//MMP14 |
| GO:0010770 | positive regulation of cell morphogenesis involved in differentiation | Biological process | 1 | 26 | 26 | 13692 | 20.2544378698225 | 0.0482606252304404 | 0.907838544770637 | 1.31640705597942 | COL1A1 |
| GO:0019079 | viral genome replication | Biological process | 1 | 26 | 26 | 13692 | 20.2544378698225 | 0.0482606252304404 | 0.907838544770637 | 1.31640705597942 | PLSCR1 |
| GO:0042516 | regulation of tyrosine phosphorylation of Stat3 protein | Biological process | 1 | 26 | 26 | 13692 | 20.2544378698225 | 0.0482606252304404 | 0.907838544770637 | 1.31640705597942 | IL22RA2 |
| GO:0060071 | Wnt receptor signaling pathway, planar cell polarity pathway | Biological process | 1 | 26 | 26 | 13692 | 20.2544378698225 | 0.0482606252304404 | 0.907838544770637 | 1.31640705597942 | DAB2 |
| GO:0090175 | regulation of establishment of planar polarity | Biological process | 1 | 26 | 26 | 13692 | 20.2544378698225 | 0.0482606252304404 | 0.907838544770637 | 1.31640705597942 | DAB2 |
| GO:0010876 | lipid localization | Biological process | 2 | 187 | 26 | 13692 | 5.63225010283834 | 0.0486637906070027 | 0.907838544770637 | 1.31279406524518 | PLSCR1//ANXA1 |
| GO:0015849 | organic acid transport | Biological process | 2 | 187 | 26 | 13692 | 5.63225010283834 | 0.0486637906070027 | 0.907838544770637 | 1.31279406524518 | SLC6A20//ANXA1 |
