# Supplementary material for: Gene Expression in the Hippocampus in a Rat Model of Premenstrual Dysphoric Disorder After Treatment With Baixiangdan Capsules
Source: Front Psychol. 2018 Nov 13;9:2065. doi: 10.3389/fpsyg.2018.02065 (PMC6242977; doi:10.3389/fpsyg.2018.02065)
Supplement: Supplementary file 3 [file Data_Sheet_3.ZIP › Data Analysis Folder/GO Analysis Report/BXD vs fluoxetine (down)/CC_result(Rat).html]

| GO.ID | Term | Ontology | Count | Pop.Hits | List.Total | Pop.Total | Fold.Enrichment | Pvalue | FDR | Enrichment.Score | GENES |
| --- | --- | --- | --- | --- | --- | --- | --- | --- | --- | --- | --- |
| GO:0044421 | extracellular region part | Cellular component | 17 | 887 | 30 | 15288 | 9.76685456595265 | 4.82927919633456e-14 | 2.59332292843166e-11 | 13.3161176858976 | COL1A1//COL3A1//LGALS1//LUM//LGALS3//TGFBI//TIMP1//RGD1562717//CP//MGP//SPP1//ANXA1//PTGDS//CFD//SERPINB1A//F5//LRG1 |
| GO:0005576 | extracellular region | Cellular component | 19 | 1410 | 30 | 15288 | 6.86695035460993 | 3.88901465745677e-13 | 1.04420043552714e-10 | 12.4101604200203 | COL1A1//COL3A1//LGALS1//LUM//LGALS3//TGFBI//TIMP1//RGD1562717//CP//MGP//SPP1//ANXA1//PTGDS//CFD//SERPINB1A//F5//LRG1//IL22RA2//FIBIN |
| GO:0005615 | extracellular space | Cellular component | 15 | 707 | 30 | 15288 | 10.8118811881188 | 6.64056422088618e-13 | 1.18866099553863e-10 | 12.1777950188839 | CP//MGP//SPP1//ANXA1//PTGDS//COL1A1//CFD//LGALS1//LUM//COL3A1//TGFBI//SERPINB1A//F5//RGD1562717//LRG1 |
| GO:0031012 | extracellular matrix | Cellular component | 11 | 350 | 30 | 15288 | 16.016 | 2.86736243514197e-11 | 3.84943406917809e-09 | 10.542517408681 | COL1A1//COL3A1//LGALS1//LUM//LGALS3//TGFBI//TIMP1//RGD1562717//MGP//MMP14//PLSCR1 |
| GO:0005578 | proteinaceous extracellular matrix | Cellular component | 8 | 262 | 30 | 15288 | 15.5603053435115 | 2.82409294912548e-08 | 3.03307582736077e-06 | 7.54912101348851 | COL1A1//COL3A1//LUM//TGFBI//TIMP1//RGD1562717//LGALS1//LGALS3 |
| GO:0005583 | fibrillar collagen | Cellular component | 3 | 12 | 30 | 15288 | 127.4 | 1.48235491444459e-06 | 0.000132670764842791 | 5.82904780247605 | COL1A1//COL3A1//LUM |
| GO:0044420 | extracellular matrix part | Cellular component | 5 | 154 | 30 | 15288 | 16.5454545454545 | 1.13008373437925e-05 | 0.000866935664802367 | 4.94688937595768 | COL1A1//COL3A1//LUM//TGFBI//TIMP1 |
| GO:0005581 | collagen | Cellular component | 3 | 55 | 30 | 15288 | 27.7963636363636 | 0.000166986307015568 | 0.01120895585842 | 3.77731913981884 | LUM//COL1A1//COL3A1 |
| GO:0005604 | basement membrane | Cellular component | 2 | 86 | 30 | 15288 | 11.8511627906977 | 0.0122843787387328 | 0.732967931411057 | 1.9106468023229 | TGFBI//TIMP1 |
| GO:0045177 | apical part of cell | Cellular component | 3 | 306 | 30 | 15288 | 4.99607843137255 | 0.0216308295271965 | 1 | 1.66492682535166 | DAB2//SLC6A20//SPP1 |
| GO:0001533 | cornified envelope | Cellular component | 1 | 14 | 30 | 15288 | 36.4 | 0.0271362409608572 | 1 | 1.5664503130147 | ANXA1 |
| GO:0005614 | interstitial matrix | Cellular component | 1 | 14 | 30 | 15288 | 36.4 | 0.0271362409608572 | 1 | 1.5664503130147 | RGD1562717 |
| GO:0031091 | platelet alpha granule | Cellular component | 1 | 14 | 30 | 15288 | 36.4 | 0.0271362409608572 | 1 | 1.5664503130147 | F5 |
| GO:0031967 | organelle envelope | Cellular component | 4 | 699 | 30 | 15288 | 2.91616595135908 | 0.0462385391127033 | 1 | 1.33499589572322 | PTGDS//S100A6//LGALS3//ANXA1 |
| GO:0031975 | envelope | Cellular component | 4 | 714 | 30 | 15288 | 2.85490196078431 | 0.0493372183936062 | 1 | 1.30682533945028 | PTGDS//S100A6//LGALS3//ANXA1 |
| GO:0031988 | membrane-bounded vesicle | Cellular component | 4 | 715 | 30 | 15288 | 2.85090909090909 | 0.0495478795944376 | 1 | 1.30497492644893 | DAB2//F5//MMP14//SPP1 |
