# Supplementary material for: Gene Expression in the Hippocampus in a Rat Model of Premenstrual Dysphoric Disorder After Treatment With Baixiangdan Capsules
Source: Front Psychol. 2018 Nov 13;9:2065. doi: 10.3389/fpsyg.2018.02065 (PMC6242977; doi:10.3389/fpsyg.2018.02065)
Supplement: Supplementary file 3 [file Data_Sheet_3.ZIP › Data Analysis Folder/GO Analysis Report/BXD vs fluoxetine (down)/MF_result(Rat).html]

| GO.ID | Term | Ontology | Count | Pop.Hits | List.Total | Pop.Total | Fold.Enrichment | Pvalue | FDR | Enrichment.Score | GENES |
| --- | --- | --- | --- | --- | --- | --- | --- | --- | --- | --- | --- |
| GO:0005509 | calcium ion binding | Molecular function | 8 | 531 | 28 | 14392 | 7.74387947269303 | 5.26955103929204e-06 | 0.00472678728224496 | 5.27822638468573 | MGP//ANXA1//MMP14//S100A6//PLSCR1//CAPSL//S100A11//RGD1563581 |
| GO:0050840 | extracellular matrix binding | Molecular function | 3 | 39 | 28 | 14392 | 39.5384615384615 | 5.75103337606769e-05 | 0.0257933846916636 | 4.24025411197105 | LGALS1//SPP1//TGFBI |
| GO:0048407 | platelet-derived growth factor binding | Molecular function | 2 | 11 | 28 | 14392 | 93.4545454545455 | 0.000198592690875508 | 0.0593792145717769 | 3.70203673958106 | COL1A1//COL3A1 |
| GO:0005201 | extracellular matrix structural constituent | Molecular function | 2 | 29 | 28 | 14392 | 35.448275862069 | 0.00143456772016188 | 0.321701811246302 | 2.84327894564145 | COL1A1//COL3A1 |
| GO:0048306 | calcium-dependent protein binding | Molecular function | 2 | 40 | 28 | 14392 | 25.7 | 0.00271988263327125 | 0.447772125843005 | 2.56544983597403 | MGP//S100A6 |
| GO:0005518 | collagen binding | Molecular function | 2 | 42 | 28 | 14392 | 24.4761904761905 | 0.00299513127654184 | 0.447772125843005 | 2.52358413773935 | LUM//RGD1562717 |
| GO:0005515 | protein binding | Molecular function | 16 | 4586 | 28 | 14392 | 1.7932839075447 | 0.00496299147621597 | 0.600564756601918 | 2.30425647068935 | LGALS1//CP//ANXA1//SPP1//PLSCR1//MMP14//COL3A1//LUM//RGD1562717//S100A6//DAB2//COL1A1//TGM2//LGALS3//IL22RA2//MGP |
| GO:0061134 | peptidase regulator activity | Molecular function | 3 | 192 | 28 | 14392 | 8.03125 | 0.00599065378636813 | 0.600564756601918 | 2.22252577855929 | SERPINB1A//TIMP1//MMP14 |
| GO:0005178 | integrin binding | Molecular function | 2 | 60 | 28 | 14392 | 17.1333333333333 | 0.00602573334383195 | 0.600564756601918 | 2.2199900910272 | MMP14//COL3A1 |
| GO:0046872 | metal ion binding | Molecular function | 11 | 2783 | 28 | 14392 | 2.03162055335968 | 0.0115077727040278 | 1 | 1.93900872467199 | MGP//ANXA1//MMP14//S100A6//PLSCR1//CAPSL//S100A11//RGD1563581//CP//TGM2//TIMP1 |
| GO:0043169 | cation binding | Molecular function | 11 | 2827 | 28 | 14392 | 2 | 0.0129352059722573 | 1 | 1.88822665145439 | MGP//ANXA1//MMP14//S100A6//PLSCR1//CAPSL//S100A11//RGD1563581//TGM2//TIMP1//CP |
| GO:0043167 | ion binding | Molecular function | 11 | 2840 | 28 | 14392 | 1.99084507042254 | 0.0133826468661912 | 1 | 1.87345798180768 | MGP//ANXA1//MMP14//S100A6//PLSCR1//CAPSL//S100A11//RGD1563581//TGM2//TIMP1//CP |
| GO:0004857 | enzyme inhibitor activity | Molecular function | 3 | 274 | 28 | 14392 | 5.62773722627737 | 0.0157333764380686 | 1 | 1.80317806624725 | ANXA1//SERPINB1A//TIMP1 |
| GO:0019838 | growth factor binding | Molecular function | 2 | 108 | 28 | 14392 | 9.51851851851852 | 0.0185736381167416 | 1 | 1.73110302037048 | COL1A1//COL3A1 |
| GO:0005523 | tropomyosin binding | Molecular function | 1 | 11 | 28 | 14392 | 46.7272727272727 | 0.0212011048618761 | 1 | 1.67364150591498 | S100A6 |
| GO:0055102 | lipase inhibitor activity | Molecular function | 1 | 12 | 28 | 14392 | 42.8333333333333 | 0.0231068394466664 | 1 | 1.63625945325482 | ANXA1 |
| GO:0019865 | immunoglobulin binding | Molecular function | 1 | 14 | 28 | 14392 | 36.7142857142857 | 0.0269075804200629 | 1 | 1.57012535305227 | LGALS3 |
| GO:0004175 | endopeptidase activity | Molecular function | 3 | 346 | 28 | 14392 | 4.45664739884393 | 0.0289400472851186 | 1 | 1.53850076362302 | CFD//F5//MMP14 |
| GO:0005488 | binding | Molecular function | 23 | 9220 | 28 | 14392 | 1.2822125813449 | 0.031419376017524 | 1 | 1.50280244421129 | TGM2//DAB2//MGP//ANXA1//MMP14//S100A6//PLSCR1//CAPSL//S100A11//RGD1563581//LGALS1//CP//LGALS3//SPP1//COL3A1//PTGDS//TIMP1//LUM//RGD1562717//ACTG2//COL1A1//TGFBI//IL22RA2 |
| GO:0030246 | carbohydrate binding | Molecular function | 3 | 376 | 28 | 14392 | 4.10106382978723 | 0.0357591590285404 | 1 | 1.44661270334253 | LGALS1//RGD1562717//LGALS3 |
| GO:0004866 | endopeptidase inhibitor activity | Molecular function | 2 | 154 | 28 | 14392 | 6.67532467532468 | 0.035854395745593 | 1 | 1.44545759226883 | SERPINB1A//TIMP1 |
| GO:0016755 | transferase activity, transferring amino-acyl groups | Molecular function | 1 | 19 | 28 | 14392 | 27.0526315789474 | 0.036347152861368 | 1 | 1.43952960254977 | TGM2 |
| GO:0005328 | neurotransmitter:sodium symporter activity | Molecular function | 1 | 20 | 28 | 14392 | 25.7 | 0.0382244422038776 | 1 | 1.41765884337923 | SLC6A20 |
| GO:0005548 | phospholipid transporter activity | Molecular function | 1 | 20 | 28 | 14392 | 25.7 | 0.0382244422038776 | 1 | 1.41765884337923 | PLSCR1 |
| GO:0061135 | endopeptidase regulator activity | Molecular function | 2 | 161 | 28 | 14392 | 6.38509316770186 | 0.0388750613895746 | 1 | 1.41032891214819 | SERPINB1A//TIMP1 |
| GO:0004252 | serine-type endopeptidase activity | Molecular function | 2 | 163 | 28 | 14392 | 6.30674846625767 | 0.0397556039870585 | 1 | 1.40060164413672 | CFD//F5 |
| GO:0030414 | peptidase inhibitor activity | Molecular function | 2 | 166 | 28 | 14392 | 6.19277108433735 | 0.0410907398959164 | 1 | 1.38625603859161 | SERPINB1A//TIMP1 |
| GO:0043236 | laminin binding | Molecular function | 1 | 23 | 28 | 14392 | 22.3478260869565 | 0.0438351750920438 | 1 | 1.3581772543918 | LGALS1 |
| GO:0032403 | protein complex binding | Molecular function | 3 | 410 | 28 | 14392 | 3.7609756097561 | 0.0444131589642539 | 1 | 1.35248833576682 | MMP14//COL3A1//LGALS3 |
| GO:0005501 | retinoid binding | Molecular function | 1 | 24 | 28 | 14392 | 21.4166666666667 | 0.0456983955734571 | 1 | 1.3400990473224 | PTGDS |
| GO:0005544 | calcium-dependent phospholipid binding | Molecular function | 1 | 24 | 28 | 14392 | 21.4166666666667 | 0.0456983955734571 | 1 | 1.3400990473224 | ANXA1 |
| GO:0005326 | neurotransmitter transporter activity | Molecular function | 1 | 25 | 28 | 14392 | 20.56 | 0.0475581147357581 | 1 | 1.32277536969854 | SLC6A20 |
| GO:0008236 | serine-type peptidase activity | Molecular function | 2 | 183 | 28 | 14392 | 5.61748633879781 | 0.0489706612071512 | 1 | 1.31006403205124 | CFD//F5 |
| GO:0005154 | epidermal growth factor receptor binding | Molecular function | 1 | 26 | 28 | 14392 | 19.7692307692308 | 0.0494143389152942 | 1 | 1.30614701042475 | PLSCR1 |
