# Supplementary material for: Gene Expression in the Hippocampus in a Rat Model of Premenstrual Dysphoric Disorder After Treatment With Baixiangdan Capsules
Source: Front Psychol. 2018 Nov 13;9:2065. doi: 10.3389/fpsyg.2018.02065 (PMC6242977; doi:10.3389/fpsyg.2018.02065)
Supplement: Supplementary file 3 [file Data_Sheet_3.ZIP › Data Analysis Folder/GO Analysis Report/BXD vs fluoxetine (up)/BP_result(Rat).html]

| GO.ID | Term | Ontology | Count | Pop.Hits | List.Total | Pop.Total | Fold.Enrichment | Pvalue | FDR | Enrichment.Score | GENES |
| --- | --- | --- | --- | --- | --- | --- | --- | --- | --- | --- | --- |
| GO:0001501 | skeletal system development | Biological process | 8 | 333 | 38 | 13692 | 8.65623518255097 | 2.9115257893381e-06 | 0.00656505662150305 | 5.5358793587012 | BMP4//COL1A1//TGFBI//MGP//LUM//TIMP1//LGALS3//COL3A1 |
| GO:0051216 | cartilage development | Biological process | 6 | 150 | 38 | 13692 | 14.4126315789474 | 3.23309338745125e-06 | 0.00656505662150305 | 5.49038175067177 | BMP4//TGFBI//COL1A1//MGP//LUM//TIMP1 |
| GO:0048545 | response to steroid hormone stimulus | Biological process | 9 | 477 | 38 | 13692 | 6.79841112214499 | 4.6405999097096e-06 | 0.00656505662150305 | 5.33342587275645 | ANXA1//COL1A1//C3//BMP4//CLDN4//MMP14//MGP//PTGDS//SPP1 |
| GO:0010810 | regulation of cell-substrate adhesion | Biological process | 5 | 96 | 38 | 13692 | 18.7664473684211 | 6.37573069343059e-06 | 0.00656505662150305 | 5.19547003553088 | SPP1//RGD1562717//COL1A1//LGALS1//MMP14 |
| GO:0009719 | response to endogenous stimulus | Biological process | 12 | 987 | 38 | 13692 | 4.38073908174692 | 8.61158016395662e-06 | 0.00656505662150305 | 5.06491715127324 | FRS3//MGP//ANXA1//MMP14//COL1A1//COL3A1//C3//BMP4//CLDN4//TIMP1//SPP1//PTGDS |
| GO:0031589 | cell-substrate adhesion | Biological process | 6 | 180 | 38 | 13692 | 12.0105263157895 | 9.24460628739857e-06 | 0.00656505662150305 | 5.0341115799998 | COL3A1//SPP1//RGD1562717//COL1A1//LGALS1//MMP14 |
| GO:0061448 | connective tissue development | Biological process | 6 | 184 | 38 | 13692 | 11.7494279176201 | 1.04825265398087e-05 | 0.00656505662150305 | 4.9795340293701 | BMP4//TGFBI//MGP//LUM//TIMP1//COL1A1 |
| GO:0009611 | response to wounding | Biological process | 10 | 688 | 38 | 13692 | 5.23714810281518 | 1.26155890723873e-05 | 0.00691334281166824 | 4.89909246572395 | C3//ANXA1//PLSCR1//F5//SPP1//COL1A1//COL3A1//TIMP1//LGALS1//TGM2 |
| GO:0010033 | response to organic substance | Biological process | 15 | 1714 | 38 | 13692 | 3.15328870601241 | 2.51097890625803e-05 | 0.0122312572500391 | 4.60015693560815 | FRS3//MGP//ANXA1//MMP14//BMP4//LUM//COL1A1//COL3A1//C3//CLDN4//TIMP1//PLSCR1//SPP1//PTGDS//LGALS1 |
| GO:0031960 | response to corticosteroid stimulus | Biological process | 6 | 232 | 38 | 13692 | 9.31851179673321 | 3.89007800094979e-05 | 0.0150476172762966 | 4.41004169043716 | C3//BMP4//MGP//ANXA1//PTGDS//COL1A1 |
| GO:0030198 | extracellular matrix organization | Biological process | 5 | 140 | 38 | 13692 | 12.8684210526316 | 3.98081521862965e-05 | 0.0150476172762966 | 4.40002798101725 | COL1A1//COL3A1//LGALS3//TGFBI//RGD1562717 |
| GO:0043062 | extracellular structure organization | Biological process | 5 | 141 | 38 | 13692 | 12.7771556550952 | 4.11887334205199e-05 | 0.0150476172762966 | 4.38522156266695 | LGALS3//TGFBI//RGD1562717//COL1A1//COL3A1 |
| GO:0009725 | response to hormone stimulus | Biological process | 10 | 801 | 38 | 13692 | 4.4983244628425 | 4.68379015011322e-05 | 0.0150682873837576 | 4.32940257104832 | ANXA1//COL1A1//C3//BMP4//CLDN4//TIMP1//MMP14//SPP1//MGP//PTGDS |
| GO:0030155 | regulation of cell adhesion | Biological process | 6 | 241 | 38 | 13692 | 8.9705175802577 | 4.81195308787879e-05 | 0.0150682873837576 | 4.31767861528371 | SPP1//RGD1562717//COL1A1//LGALS1//TGM2//MMP14 |
| GO:0018149 | peptide cross-linking | Biological process | 3 | 28 | 38 | 13692 | 38.6052631578947 | 6.1589087032221e-05 | 0.0176814514727681 | 4.21049623364001 | TGM2//ANXA1//COL3A1 |
| GO:0010812 | negative regulation of cell-substrate adhesion | Biological process | 3 | 29 | 38 | 13692 | 37.2740471869328 | 6.85640225905699e-05 | 0.0176814514727681 | 4.16390371066421 | MMP14//COL1A1//LGALS1 |
| GO:0032964 | collagen biosynthetic process | Biological process | 3 | 29 | 38 | 13692 | 37.2740471869328 | 6.85640225905699e-05 | 0.0176814514727681 | 4.16390371066421 | BMP4//COL1A1//COL3A1 |
| GO:0072376 | protein activation cascade | Biological process | 3 | 39 | 38 | 13692 | 27.7165991902834 | 0.000168231943296788 | 0.0409738244118399 | 3.77409153838123 | C3//CFD//F5 |
| GO:0031214 | biomineral tissue development | Biological process | 4 | 104 | 38 | 13692 | 13.8582995951417 | 0.000190097070778276 | 0.0438145184440823 | 3.72102457516313 | MGP//BMP4//COL1A1//SPP1 |
| GO:0009888 | tissue development | Biological process | 11 | 1149 | 38 | 13692 | 3.44949841967844 | 0.000199883752025923 | 0.0438145184440823 | 3.69922250699381 | BMP4//TGFBI//DAB2//COL1A1//ANXA1//MGP//SPP1//COL3A1//LUM//TIMP1//TGM2 |
| GO:0007275 | multicellular organismal development | Biological process | 20 | 3429 | 38 | 13692 | 2.10157940783718 | 0.000238850002113677 | 0.0498627813936362 | 3.62187475053739 | BMP4//COL1A1//LGALS3//COL3A1//MMP14//TGFBI//DAB2//IFITM1//ROBO3//CP//LGALS1//C3//PLSCR1//ANXA1//MGP//SPP1//TIMP1//LUM//TGM2//ZAR1 |
| GO:0032963 | collagen metabolic process | Biological process | 3 | 47 | 38 | 13692 | 22.9988801791713 | 0.000293952210408583 | 0.0584450242803108 | 3.53172326973365 | COL1A1//COL3A1//BMP4 |
| GO:0051384 | response to glucocorticoid stimulus | Biological process | 5 | 219 | 38 | 13692 | 8.22638788752704 | 0.00032652472704367 | 0.0584450242803108 | 3.48608392491878 | ANXA1//BMP4//C3//MGP//PTGDS |
| GO:0045785 | positive regulation of cell adhesion | Biological process | 4 | 120 | 38 | 13692 | 12.0105263157895 | 0.000328999184923819 | 0.0584450242803108 | 3.4828051779878 | SPP1//RGD1562717//LGALS1//TGM2 |
| GO:0002376 | immune system process | Biological process | 11 | 1218 | 38 | 13692 | 3.2540834845735 | 0.000333285950503597 | 0.0584450242803108 | 3.47718299341227 | ANXA1//C3//BMP4//LGALS1//PLSCR1//CFD//SPP1//TIMP1//LGALS3//COL3A1//IFITM1 |
| GO:0044259 | multicellular organismal macromolecule metabolic process | Biological process | 3 | 50 | 38 | 13692 | 21.6189473684211 | 0.000353283296423082 | 0.0595689989045689 | 3.45187689594408 | COL1A1//COL3A1//BMP4 |
| GO:2000242 | negative regulation of reproductive process | Biological process | 3 | 51 | 38 | 13692 | 21.1950464396285 | 0.000374646129905057 | 0.06083143087051 | 3.42637874930519 | PLSCR1//BMP4//TIMP1 |
| GO:0032502 | developmental process | Biological process | 21 | 3887 | 38 | 13692 | 1.94665077925067 | 0.000450319226153132 | 0.0687202991481539 | 3.34647951065651 | DAB2//BMP4//COL1A1//LGALS3//COL3A1//MMP14//TGFBI//SPP1//IFITM1//ROBO3//CP//LGALS1//C3//ZAR1//TIMP1//PLSCR1//MGP//ANXA1//LRG1//LUM//TGM2 |
| GO:0007155 | cell adhesion | Biological process | 8 | 679 | 38 | 13692 | 4.24525230602279 | 0.000456610685559628 | 0.0687202991481539 | 3.3404539294089 | COL3A1//SPP1//RGD1562717//COL1A1//LGALS1//TGM2//MMP14//TGFBI |
| GO:0022610 | biological adhesion | Biological process | 8 | 682 | 38 | 13692 | 4.22657817564439 | 0.000470257521543024 | 0.0687202991481539 | 3.32766424939054 | SPP1//TGFBI//COL3A1//RGD1562717//COL1A1//LGALS1//TGM2//MMP14 |
| GO:0048511 | rhythmic process | Biological process | 5 | 240 | 38 | 13692 | 7.50657894736842 | 0.000496778567651158 | 0.070254104534925 | 3.30383714904649 | BMP4//MMP14//CLDN4//PTGDS//ANXA1 |
| GO:0048731 | system development | Biological process | 18 | 3069 | 38 | 13692 | 2.11328908782219 | 0.000593984523616176 | 0.0813758797354161 | 3.22622487049971 | BMP4//COL1A1//LGALS3//COL3A1//MMP14//TGFBI//ROBO3//CP//LGALS1//C3//PLSCR1//ANXA1//MGP//SPP1//TIMP1//LUM//TGM2 |
| GO:0006952 | defense response | Biological process | 8 | 711 | 38 | 13692 | 4.05418609815678 | 0.000620212908733771 | 0.0823943452087531 | 3.20745919886376 | C3//ANXA1//PLSCR1//SPP1//CFD//LGALS3//TGM2//IFITM1 |
| GO:0044236 | multicellular organismal metabolic process | Biological process | 3 | 63 | 38 | 13692 | 17.1578947368421 | 0.000698204071947695 | 0.090027254453491 | 3.15601762269563 | COL1A1//COL3A1//BMP4 |
| GO:0009605 | response to external stimulus | Biological process | 10 | 1128 | 38 | 13692 | 3.1942889137738 | 0.000771497517737844 | 0.0966355747932202 | 3.11266546692357 | C3//ANXA1//BMP4//MGP//COL1A1//MMP14//ROBO3//CP//SPP1//TGM2 |
| GO:0048513 | organ development | Biological process | 15 | 2341 | 38 | 13692 | 2.30872996245419 | 0.000889167527295869 | 0.108280845546252 | 3.05101640626739 | BMP4//MMP14//CP//COL1A1//TGFBI//LGALS1//COL3A1//PLSCR1//ANXA1//MGP//SPP1//TIMP1//LUM//TGM2 |
| GO:0042060 | wound healing | Biological process | 5 | 276 | 38 | 13692 | 6.52745995423341 | 0.000934526285782785 | 0.110728736131668 | 3.02940847854425 | C3//F5//COL1A1//COL3A1//TIMP1 |
| GO:0001503 | ossification | Biological process | 5 | 279 | 38 | 13692 | 6.45727221279004 | 0.000980884072391949 | 0.113163046667534 | 3.00838231748806 | BMP4//SPP1//COL1A1//MGP//MMP14 |
| GO:0016043 | cellular component organization | Biological process | 18 | 3233 | 38 | 13692 | 2.00608852784606 | 0.00114461298831317 | 0.121108706992769 | 2.94134133031236 | DAB2//BMP4//MGP//ROBO3//PLSCR1//COL1A1//LGALS1//LGALS3//TGFBI//RGD1562717//COL3A1//ANXA1//GRB7//TGM2//PRPH//SPP1//C3//MMP14 |
| GO:0009612 | response to mechanical stimulus | Biological process | 4 | 170 | 38 | 13692 | 8.47801857585139 | 0.0012182179017074 | 0.121108706992769 | 2.91427502283196 | COL1A1//BMP4//MGP//MMP14 |
| GO:0048856 | anatomical structure development | Biological process | 19 | 3546 | 38 | 13692 | 1.93062605752961 | 0.00122138513037935 | 0.121108706992769 | 2.91314737157898 | DAB2//BMP4//COL1A1//LGALS3//COL3A1//MMP14//TGFBI//IFITM1//ROBO3//CP//LGALS1//C3//PLSCR1//ANXA1//MGP//SPP1//TIMP1//LUM//TGM2 |
| GO:0048583 | regulation of response to stimulus | Biological process | 13 | 1898 | 38 | 13692 | 2.46791636625811 | 0.00123194198847766 | 0.121108706992769 | 2.90940974239661 | C3//ANXA1//CFD//BMP4//IL22RA2//TGM2//LGALS1//PLSCR1//PTGDS//SPP1//COL3A1//DAB2//COL1A1 |
| GO:0007162 | negative regulation of cell adhesion | Biological process | 3 | 77 | 38 | 13692 | 14.0382775119617 | 0.00125219445800878 | 0.121108706992769 | 2.90232822265722 | COL1A1//LGALS1//MMP14 |
| GO:0001568 | blood vessel development | Biological process | 6 | 443 | 38 | 13692 | 4.88012355946299 | 0.00127074173742625 | 0.121108706992769 | 2.8959427054709 | BMP4//MMP14//TGFBI//C3//COL1A1//COL3A1 |
| GO:0030324 | lung development | Biological process | 4 | 173 | 38 | 13692 | 8.33100091268634 | 0.00129958045090266 | 0.121108706992769 | 2.8861968302056 | BMP4//CP//MGP//MMP14 |
| GO:0030323 | respiratory tube development | Biological process | 4 | 175 | 38 | 13692 | 8.23578947368421 | 0.00135589345631559 | 0.121108706992769 | 2.86777443521242 | CP//BMP4//MGP//MMP14 |
| GO:0001516 | prostaglandin biosynthetic process | Biological process | 2 | 20 | 38 | 13692 | 36.0315789473684 | 0.00138082804167918 | 0.121108706992769 | 2.85986040194254 | ANXA1//PTGDS |
| GO:0043277 | apoptotic cell clearance | Biological process | 2 | 20 | 38 | 13692 | 36.0315789473684 | 0.00138082804167918 | 0.121108706992769 | 2.85986040194254 | ANXA1//TGM2 |
| GO:0046457 | prostanoid biosynthetic process | Biological process | 2 | 20 | 38 | 13692 | 36.0315789473684 | 0.00138082804167918 | 0.121108706992769 | 2.85986040194254 | PTGDS//ANXA1 |
| GO:0006950 | response to stress | Biological process | 14 | 2176 | 38 | 13692 | 2.31820820433436 | 0.00138125806332994 | 0.121108706992769 | 2.85972517369517 | MMP14//C3//ANXA1//PLSCR1//F5//SPP1//CFD//LGALS1//COL1A1//COL3A1//TIMP1//LGALS3//TGM2//IFITM1 |
| GO:0001944 | vasculature development | Biological process | 6 | 465 | 38 | 13692 | 4.64923599320883 | 0.00162833475025375 | 0.139972932257107 | 2.78825630875671 | BMP4//MMP14//TGFBI//COL1A1//COL3A1//C3 |
| GO:0006909 | phagocytosis | Biological process | 3 | 85 | 38 | 13692 | 12.7170278637771 | 0.00166514260953517 | 0.140384330773119 | 2.77854856583216 | TGM2//C3//ANXA1 |
| GO:0071840 | cellular component organization or biogenesis | Biological process | 18 | 3342 | 38 | 13692 | 1.94065954833223 | 0.00172124583868836 | 0.142376259562449 | 2.76415709669108 | DAB2//BMP4//MGP//ROBO3//PLSCR1//COL1A1//LGALS1//LGALS3//TGFBI//RGD1562717//COL3A1//ANXA1//GRB7//TGM2//PRPH//SPP1//C3//MMP14 |
| GO:0001958 | endochondral ossification | Biological process | 2 | 23 | 38 | 13692 | 31.3318077803204 | 0.00182905867272051 | 0.14579260402194 | 2.73777236295986 | BMP4//COL1A1 |
| GO:0036075 | replacement ossification | Biological process | 2 | 23 | 38 | 13692 | 31.3318077803204 | 0.00182905867272051 | 0.14579260402194 | 2.73777236295986 | BMP4//COL1A1 |
| GO:0007584 | response to nutrient | Biological process | 5 | 327 | 38 | 13692 | 5.50941574118783 | 0.00198025789704227 | 0.155025903939881 | 2.7032782461179 | BMP4//SPP1//COL1A1//CP//MGP |
| GO:0060541 | respiratory system development | Biological process | 4 | 195 | 38 | 13692 | 7.39109311740891 | 0.002016350387789 | 0.155082107018719 | 2.69543399689871 | CP//BMP4//MGP//MMP14 |
| GO:0001763 | morphogenesis of a branching structure | Biological process | 4 | 197 | 38 | 13692 | 7.31605663905958 | 0.00209271999354208 | 0.156210134765463 | 2.67928887648435 | BMP4//MGP//MMP14//TGM2 |
| GO:0002252 | immune effector process | Biological process | 5 | 332 | 38 | 13692 | 5.42644261255549 | 0.00211596850619471 | 0.156210134765463 | 2.67449080057241 | C3//LGALS1//CFD//PLSCR1//IFITM1 |
| GO:0048640 | negative regulation of developmental growth | Biological process | 2 | 25 | 38 | 13692 | 28.8252631578947 | 0.00216127362912696 | 0.156210134765463 | 2.66529024556609 | SPP1//BMP4 |
| GO:0051179 | localization | Biological process | 18 | 3407 | 38 | 13692 | 1.90363493117884 | 0.00217354430216543 | 0.156210134765463 | 2.6628315033959 | ROBO3//SLC6A20//PTGDS//DAB2//BMP4//COL1A1//CP//SLC27A2//PLSCR1//MMP14//ANXA1//GRB7//SPP1//S100A6//TGM2//C3//SLC16A10//TIMP1 |
| GO:0030154 | cell differentiation | Biological process | 14 | 2291 | 38 | 13692 | 2.20184244986101 | 0.00229322082667823 | 0.162152904905764 | 2.63955412268388 | DAB2//BMP4//SPP1//COL1A1//ROBO3//TGFBI//LGALS1//PLSCR1//ANXA1//TIMP1//MMP14//LRG1//MGP//LGALS3 |
| GO:0006692 | prostanoid metabolic process | Biological process | 2 | 27 | 38 | 13692 | 26.6900584795322 | 0.00251986637493032 | 0.172610846682727 | 2.59862248865063 | PTGDS//ANXA1 |
| GO:0006693 | prostaglandin metabolic process | Biological process | 2 | 27 | 38 | 13692 | 26.6900584795322 | 0.00251986637493032 | 0.172610846682727 | 2.59862248865063 | PTGDS//ANXA1 |
| GO:0014070 | response to organic cyclic compound | Biological process | 5 | 358 | 38 | 13692 | 5.03234342840341 | 0.00293421196876834 | 0.19522213424853 | 2.53250851573431 | LGALS1//BMP4//ANXA1//LUM//MMP14 |
| GO:0040011 | locomotion | Biological process | 8 | 906 | 38 | 13692 | 3.18159637504357 | 0.00293901935684374 | 0.19522213424853 | 2.5317975535862 | ROBO3//C3//BMP4//MMP14//COL1A1//GRB7//SPP1//TIMP1 |
| GO:0002526 | acute inflammatory response | Biological process | 3 | 105 | 38 | 13692 | 10.2947368421053 | 0.00304211287453737 | 0.196126806499586 | 2.51682467592428 | C3//ANXA1//PLSCR1 |
| GO:0048638 | regulation of developmental growth | Biological process | 3 | 105 | 38 | 13692 | 10.2947368421053 | 0.00304211287453737 | 0.196126806499586 | 2.51682467592428 | C3//SPP1//BMP4 |
| GO:0022602 | ovulation cycle process | Biological process | 3 | 108 | 38 | 13692 | 10.0087719298246 | 0.00329417868555234 | 0.205387332380587 | 2.48225284716849 | BMP4//MMP14//ANXA1 |
| GO:2000241 | regulation of reproductive process | Biological process | 3 | 108 | 38 | 13692 | 10.0087719298246 | 0.00329417868555234 | 0.205387332380587 | 2.48225284716849 | PLSCR1//BMP4//TIMP1 |
| GO:0016477 | cell migration | Biological process | 7 | 725 | 38 | 13692 | 3.4789110707804 | 0.00339590759247091 | 0.205387332380587 | 2.46904413604469 | ROBO3//BMP4//COL1A1//GRB7//SPP1//MMP14//TIMP1 |
| GO:0002520 | immune system development | Biological process | 6 | 539 | 38 | 13692 | 4.01093643198906 | 0.00341596573652947 | 0.205387332380587 | 2.46648649410446 | BMP4//LGALS1//C3//PLSCR1//TIMP1//ANXA1 |
| GO:0006954 | inflammatory response | Biological process | 5 | 371 | 38 | 13692 | 4.85600794438928 | 0.0034199989196585 | 0.205387332380587 | 2.46597403113288 | C3//ANXA1//PLSCR1//TGM2//SPP1 |
| GO:0030199 | collagen fibril organization | Biological process | 2 | 32 | 38 | 13692 | 22.5197368421053 | 0.00352987276507118 | 0.209121110838812 | 2.45224094855647 | COL1A1//COL3A1 |
| GO:0048584 | positive regulation of response to stimulus | Biological process | 8 | 950 | 38 | 13692 | 3.03423822714681 | 0.00393612484938935 | 0.221806206593941 | 2.40493113469676 | C3//CFD//BMP4//TGM2//LGALS1//PLSCR1//COL1A1//DAB2 |
| GO:0031018 | endocrine pancreas development | Biological process | 2 | 34 | 38 | 13692 | 21.1950464396285 | 0.00397854181712161 | 0.221806206593941 | 2.40027607285495 | BMP4//ANXA1 |
| GO:0050766 | positive regulation of phagocytosis | Biological process | 2 | 34 | 38 | 13692 | 21.1950464396285 | 0.00397854181712161 | 0.221806206593941 | 2.40027607285495 | ANXA1//C3 |
| GO:0043627 | response to estrogen stimulus | Biological process | 4 | 237 | 38 | 13692 | 6.08127914723518 | 0.00407144288892603 | 0.221806206593941 | 2.39025165277787 | C3//BMP4//ANXA1//MMP14 |
| GO:0048646 | anatomical structure formation involved in morphogenesis | Biological process | 7 | 750 | 38 | 13692 | 3.36294736842105 | 0.00409703075100345 | 0.221806206593941 | 2.38753077635904 | BMP4//MMP14//TGFBI//IFITM1//C3//COL1A1//TGM2 |
| GO:0006956 | complement activation | Biological process | 2 | 35 | 38 | 13692 | 20.5894736842105 | 0.00421230752687179 | 0.221806206593941 | 2.37547992990154 | C3//CFD |
| GO:0032091 | negative regulation of protein binding | Biological process | 2 | 35 | 38 | 13692 | 20.5894736842105 | 0.00421230752687179 | 0.221806206593941 | 2.37547992990154 | DAB2//PLSCR1 |
| GO:0048869 | cellular developmental process | Biological process | 14 | 2442 | 38 | 13692 | 2.06569248674512 | 0.00422048110722781 | 0.221806206593941 | 2.37463803948743 | DAB2//BMP4//SPP1//COL1A1//ROBO3//TGFBI//LGALS1//PLSCR1//MGP//LGALS3//ANXA1//TIMP1//MMP14//LRG1 |
| GO:0016053 | organic acid biosynthetic process | Biological process | 4 | 241 | 38 | 13692 | 5.98034505350513 | 0.00432091026215762 | 0.221806206593941 | 2.36442475314132 | PTGDS//PLSCR1//SLC27A2//ANXA1 |
| GO:0046394 | carboxylic acid biosynthetic process | Biological process | 4 | 241 | 38 | 13692 | 5.98034505350513 | 0.00432091026215762 | 0.221806206593941 | 2.36442475314132 | PTGDS//PLSCR1//SLC27A2//ANXA1 |
| GO:0045087 | innate immune response | Biological process | 4 | 242 | 38 | 13692 | 5.95563288386255 | 0.00438485957311673 | 0.221806206593941 | 2.35804431052988 | C3//CFD//PLSCR1//LGALS3 |
| GO:0042698 | ovulation cycle | Biological process | 3 | 120 | 38 | 13692 | 9.0078947368421 | 0.00442917738261954 | 0.221806206593941 | 2.35367692645486 | BMP4//MMP14//ANXA1 |
| GO:0010977 | negative regulation of neuron projection development | Biological process | 2 | 36 | 38 | 13692 | 20.0175438596491 | 0.00445231436593678 | 0.221806206593941 | 2.35141417888998 | SPP1//LGALS1 |
| GO:0071230 | cellular response to amino acid stimulus | Biological process | 2 | 36 | 38 | 13692 | 20.0175438596491 | 0.00445231436593678 | 0.221806206593941 | 2.35141417888998 | COL1A1//COL3A1 |
| GO:0070848 | response to growth factor stimulus | Biological process | 4 | 249 | 38 | 13692 | 5.78820545339252 | 0.00485056504598763 | 0.238931204063031 | 2.31420766715842 | FRS3//BMP4//COL1A1//LUM |
| GO:0008610 | lipid biosynthetic process | Biological process | 5 | 406 | 38 | 13692 | 4.43738656987296 | 0.00501483691499678 | 0.240348203290809 | 2.29974318588756 | PTGDS//PLSCR1//TIMP1//SLC27A2//ANXA1 |
| GO:0048870 | cell motility | Biological process | 7 | 779 | 38 | 13692 | 3.23775420579691 | 0.00504380353621223 | 0.240348203290809 | 2.29724183819742 | ROBO3//BMP4//MMP14//COL1A1//GRB7//SPP1//TIMP1 |
| GO:0051674 | localization of cell | Biological process | 7 | 779 | 38 | 13692 | 3.23775420579691 | 0.00504380353621223 | 0.240348203290809 | 2.29724183819742 | ROBO3//BMP4//MMP14//COL1A1//GRB7//SPP1//TIMP1 |
| GO:0046456 | icosanoid biosynthetic process | Biological process | 2 | 39 | 38 | 13692 | 18.4777327935223 | 0.00520943658038879 | 0.245571720090586 | 2.28320924469658 | PTGDS//ANXA1 |
| GO:0048771 | tissue remodeling | Biological process | 3 | 129 | 38 | 13692 | 8.37943696450428 | 0.00541853485675002 | 0.252711242680767 | 2.26611812852572 | TGM2//SPP1//MMP14 |
| GO:0015909 | long-chain fatty acid transport | Biological process | 2 | 41 | 38 | 13692 | 17.5763799743261 | 0.00574476008165184 | 0.265105559978544 | 2.24072810403234 | SLC27A2//ANXA1 |
| GO:0006636 | unsaturated fatty acid biosynthetic process | Biological process | 2 | 42 | 38 | 13692 | 17.1578947368421 | 0.00602149232592833 | 0.27498148288406 | 2.2202958627957 | PTGDS//ANXA1 |
| GO:0001649 | osteoblast differentiation | Biological process | 3 | 136 | 38 | 13692 | 7.94814241486068 | 0.00627303060338694 | 0.279168979236769 | 2.20252259372368 | BMP4//SPP1//COL1A1 |
| GO:0045445 | myoblast differentiation | Biological process | 2 | 43 | 38 | 13692 | 16.7588739290086 | 0.00630422649280113 | 0.279168979236769 | 2.20036819228139 | BMP4//LGALS1 |
| GO:0071229 | cellular response to acid | Biological process | 2 | 43 | 38 | 13692 | 16.7588739290086 | 0.00630422649280113 | 0.279168979236769 | 2.20036819228139 | COL1A1//COL3A1 |
| GO:0060350 | endochondral bone morphogenesis | Biological process | 2 | 44 | 38 | 13692 | 16.377990430622 | 0.00659292894298324 | 0.289034004860385 | 2.18092160495349 | BMP4//COL1A1 |
| GO:0031349 | positive regulation of defense response | Biological process | 3 | 139 | 38 | 13692 | 7.77659977281333 | 0.00666252547783204 | 0.289193185097185 | 2.17636111719829 | C3//PLSCR1//TGM2 |
| GO:0042176 | regulation of protein catabolic process | Biological process | 3 | 140 | 38 | 13692 | 7.72105263157895 | 0.00679549848267473 | 0.290203769717893 | 2.16777868016261 | DAB2//TIMP1//SERPINB1A |
| GO:0031347 | regulation of defense response | Biological process | 4 | 275 | 38 | 13692 | 5.24095693779904 | 0.00687121852832015 | 0.290203769717893 | 2.16296623918513 | C3//ANXA1//PLSCR1//TGM2 |
| GO:0035295 | tube development | Biological process | 5 | 438 | 38 | 13692 | 4.11319394376352 | 0.00688439599695732 | 0.290203769717893 | 2.16213415659737 | BMP4//CP//MGP//MMP14//TGM2 |
| GO:0050764 | regulation of phagocytosis | Biological process | 2 | 46 | 38 | 13692 | 15.6659038901602 | 0.00718810479537343 | 0.300120489742068 | 2.14338559993804 | C3//ANXA1 |
| GO:0002673 | regulation of acute inflammatory response | Biological process | 2 | 47 | 38 | 13692 | 15.3325867861142 | 0.00749451157692742 | 0.306586966760451 | 2.12525666519824 | C3//ANXA1 |
| GO:0032570 | response to progesterone stimulus | Biological process | 2 | 47 | 38 | 13692 | 15.3325867861142 | 0.00749451157692742 | 0.306586966760451 | 2.12525666519824 | C3//CLDN4 |
| GO:0002682 | regulation of immune system process | Biological process | 6 | 635 | 38 | 13692 | 3.40455864069623 | 0.00755278111544906 | 0.306586966760451 | 2.12189310125942 | C3//CFD//LGALS1//PLSCR1//BMP4//COL3A1 |
| GO:0051129 | negative regulation of cellular component organization | Biological process | 4 | 285 | 38 | 13692 | 5.05706371191136 | 0.00777768507177936 | 0.31113460821685 | 2.10914964596066 | LGALS1//BMP4//SPP1//MMP14 |
| GO:0071418 | cellular response to amine stimulus | Biological process | 2 | 48 | 38 | 13692 | 15.0131578947368 | 0.00780675339960161 | 0.31113460821685 | 2.10752953895012 | COL1A1//COL3A1 |
| GO:0006955 | immune response | Biological process | 6 | 641 | 38 | 13692 | 3.37269069710157 | 0.00789651820288687 | 0.311876899112217 | 2.10256435916445 | C3//LGALS1//CFD//LGALS3//PLSCR1//COL3A1 |
| GO:0035239 | tube morphogenesis | Biological process | 4 | 291 | 38 | 13692 | 4.95279435702659 | 0.00835784124246461 | 0.322398518557592 | 2.07790588255545 | BMP4//MGP//MMP14//TGM2 |
| GO:0032355 | response to estradiol stimulus | Biological process | 3 | 151 | 38 | 13692 | 7.15859184384803 | 0.00836340319169326 | 0.322398518557592 | 2.07761696578765 | C3//BMP4//ANXA1 |
| GO:2000026 | regulation of multicellular organismal development | Biological process | 8 | 1078 | 38 | 13692 | 2.67395762132604 | 0.00838353811942643 | 0.322398518557592 | 2.07657265661393 | BMP4//COL1A1//LGALS1//MGP//C3//SPP1//TIMP1//DAB2 |
| GO:0010035 | response to inorganic substance | Biological process | 5 | 461 | 38 | 13692 | 3.90798036305514 | 0.00850037745369832 | 0.324049171800117 | 2.07056178929526 | C3//COL1A1//CP//MGP//ANXA1 |
| GO:0009653 | anatomical structure morphogenesis | Biological process | 11 | 1819 | 38 | 13692 | 2.17893000781227 | 0.00859222911474528 | 0.324727003784856 | 2.06589415087835 | DAB2//BMP4//MMP14//TGFBI//IFITM1//COL1A1//ROBO3//C3//SPP1//MGP//TGM2 |
| GO:0030162 | regulation of proteolysis | Biological process | 3 | 153 | 38 | 13692 | 7.06501547987616 | 0.00866946254762375 | 0.324845502639167 | 2.06200782522693 | DAB2//TIMP1//SERPINB1A |
| GO:0043588 | skin development | Biological process | 2 | 52 | 38 | 13692 | 13.8582995951417 | 0.00911341336476824 | 0.336996554296616 | 2.04031893061903 | COL1A1//COL3A1 |
| GO:0071495 | cellular response to endogenous stimulus | Biological process | 5 | 470 | 38 | 13692 | 3.83314669652855 | 0.00919933904234727 | 0.336996554296616 | 2.03624337489066 | FRS3//COL1A1//COL3A1//BMP4//ANXA1 |
| GO:0009628 | response to abiotic stimulus | Biological process | 7 | 873 | 38 | 13692 | 2.88913004159884 | 0.00928151270760979 | 0.336996554296616 | 2.03238123638579 | MMP14//BMP4//MGP//COL1A1//COL3A1//ANXA1//PLSCR1 |
| GO:0048754 | branching morphogenesis of a tube | Biological process | 3 | 157 | 38 | 13692 | 6.88501508548441 | 0.00930122789002977 | 0.336996554296616 | 2.03145971481558 | BMP4//MGP//MMP14 |
| GO:0071417 | cellular response to organic nitrogen | Biological process | 2 | 53 | 38 | 13692 | 13.59682224429 | 0.00945433822113869 | 0.338848559878785 | 2.02436886522892 | COL1A1//COL3A1 |
| GO:0071310 | cellular response to organic substance | Biological process | 7 | 877 | 38 | 13692 | 2.87595270959611 | 0.00950692811703707 | 0.338848559878785 | 2.02195978982751 | FRS3//COL1A1//COL3A1//BMP4//LGALS1//PLSCR1//ANXA1 |
| GO:0030097 | hemopoiesis | Biological process | 5 | 477 | 38 | 13692 | 3.77689506785833 | 0.00976995380458232 | 0.343737240273317 | 2.01010748975742 | BMP4//LGALS1//PLSCR1//TIMP1//ANXA1 |
| GO:0010811 | positive regulation of cell-substrate adhesion | Biological process | 2 | 54 | 38 | 13692 | 13.3450292397661 | 0.00980090215195361 | 0.343737240273317 | 2.008733946595 | SPP1//RGD1562717 |
| GO:0051128 | regulation of cellular component organization | Biological process | 8 | 1110 | 38 | 13692 | 2.59687055476529 | 0.00993679389336606 | 0.34573733673426 | 2.00275371812013 | COL1A1//LGALS1//DAB2//ANXA1//BMP4//SPP1//C3//MMP14 |
| GO:0044283 | small molecule biosynthetic process | Biological process | 4 | 307 | 38 | 13692 | 4.6946682667581 | 0.010042653661958 | 0.346669241370267 | 1.99815151443524 | PTGDS//PLSCR1//SLC27A2//ANXA1 |
| GO:0006897 | endocytosis | Biological process | 4 | 308 | 38 | 13692 | 4.67942583732057 | 0.0101547635018702 | 0.3467362048382 | 1.99333018658992 | DAB2//TGM2//C3//ANXA1 |
| GO:0009967 | positive regulation of signal transduction | Biological process | 6 | 677 | 38 | 13692 | 3.19334525382881 | 0.0102027761003941 | 0.3467362048382 | 1.99128164382201 | BMP4//TGM2//LGALS1//C3//COL1A1//DAB2 |
| GO:0006869 | lipid transport | Biological process | 3 | 164 | 38 | 13692 | 6.59114249037227 | 0.0104704584735886 | 0.346827693436697 | 1.98003430130203 | SLC27A2//PLSCR1//ANXA1 |
| GO:0006935 | chemotaxis | Biological process | 4 | 312 | 38 | 13692 | 4.61943319838057 | 0.0106113768479134 | 0.346827693436697 | 1.97422826184263 | ROBO3//SPP1//BMP4//C3 |
| GO:0032269 | negative regulation of cellular protein metabolic process | Biological process | 4 | 312 | 38 | 13692 | 4.61943319838057 | 0.0106113768479134 | 0.346827693436697 | 1.97422826184263 | GRB7//BMP4//TIMP1//HHATL |
| GO:0042330 | taxis | Biological process | 4 | 313 | 38 | 13692 | 4.60467462586178 | 0.010727585009346 | 0.346827693436697 | 1.96949803527177 | C3//ROBO3//SPP1//BMP4 |
| GO:0001525 | angiogenesis | Biological process | 4 | 314 | 38 | 13692 | 4.59001005698961 | 0.0108446195886172 | 0.346827693436697 | 1.96478567771729 | BMP4//C3//MMP14//TGFBI |
| GO:0030500 | regulation of bone mineralization | Biological process | 2 | 57 | 38 | 13692 | 12.6426592797784 | 0.0108741069054705 | 0.346827693436697 | 1.96360640167938 | BMP4//MGP |
| GO:0071385 | cellular response to glucocorticoid stimulus | Biological process | 2 | 57 | 38 | 13692 | 12.6426592797784 | 0.0108741069054705 | 0.346827693436697 | 1.96360640167938 | BMP4//ANXA1 |
| GO:0031667 | response to nutrient levels | Biological process | 5 | 490 | 38 | 13692 | 3.67669172932331 | 0.0108940958498314 | 0.346827693436697 | 1.96280880798066 | CP//MGP//COL1A1//BMP4//SPP1 |
| GO:0002253 | activation of immune response | Biological process | 3 | 167 | 38 | 13692 | 6.47273873306019 | 0.010996589732596 | 0.346827693436697 | 1.95874197756406 | C3//CFD//PLSCR1 |
| GO:0050727 | regulation of inflammatory response | Biological process | 3 | 167 | 38 | 13692 | 6.47273873306019 | 0.010996589732596 | 0.346827693436697 | 1.95874197756406 | C3//ANXA1//TGM2 |
| GO:0051246 | regulation of protein metabolic process | Biological process | 8 | 1133 | 38 | 13692 | 2.54415385330051 | 0.011181100819933 | 0.349566674768637 | 1.95151543647756 | BMP4//DAB2//C3//GRB7//SERPINB1A//IL22RA2//TIMP1//HHATL |
| GO:0071384 | cellular response to corticosteroid stimulus | Biological process | 2 | 58 | 38 | 13692 | 12.4246823956443 | 0.0112429062824767 | 0.349566674768637 | 1.94912140947053 | ANXA1//BMP4 |
| GO:0000904 | cell morphogenesis involved in differentiation | Biological process | 5 | 495 | 38 | 13692 | 3.63955342902711 | 0.0113492883806467 | 0.350389297610952 | 1.94503136860718 | BMP4//ROBO3//COL1A1//SPP1//DAB2 |
| GO:0042221 | response to chemical stimulus | Biological process | 17 | 3628 | 38 | 13692 | 1.68835954273777 | 0.0115251047577837 | 0.353329085721145 | 1.93835511838772 | C3//ROBO3//CP//MGP//COL1A1//FRS3//ANXA1//LGALS1//MMP14//SPP1//TIMP1//BMP4//LUM//COL3A1//CLDN4//PLSCR1//PTGDS |
| GO:0048661 | positive regulation of smooth muscle cell proliferation | Biological process | 2 | 59 | 38 | 13692 | 12.21409455843 | 0.0116171849406491 | 0.353678741526428 | 1.93489909680416 | BMP4//TGM2 |
| GO:0072358 | cardiovascular system development | Biological process | 6 | 701 | 38 | 13692 | 3.0840153164652 | 0.0119874643836391 | 0.359952355190917 | 1.92127267016639 | BMP4//MMP14//TGFBI//COL1A1//COL3A1//C3 |
| GO:0072359 | circulatory system development | Biological process | 6 | 701 | 38 | 13692 | 3.0840153164652 | 0.0119874643836391 | 0.359952355190917 | 1.92127267016639 | BMP4//MMP14//TGFBI//COL1A1//COL3A1//C3 |
| GO:0015908 | fatty acid transport | Biological process | 2 | 61 | 38 | 13692 | 11.8136324417601 | 0.012382053902965 | 0.364314928259051 | 1.90720730969888 | SLC27A2//ANXA1 |
| GO:0050729 | positive regulation of inflammatory response | Biological process | 2 | 61 | 38 | 13692 | 11.8136324417601 | 0.012382053902965 | 0.364314928259051 | 1.90720730969888 | C3//TGM2 |
| GO:0070167 | regulation of biomineral tissue development | Biological process | 2 | 61 | 38 | 13692 | 11.8136324417601 | 0.012382053902965 | 0.364314928259051 | 1.90720730969888 | MGP//BMP4 |
| GO:0001541 | ovarian follicle development | Biological process | 2 | 62 | 38 | 13692 | 11.6230899830221 | 0.0127725814204663 | 0.371427448424964 | 1.89372132017073 | BMP4//MMP14 |
| GO:0048534 | hemopoietic or lymphoid organ development | Biological process | 5 | 510 | 38 | 13692 | 3.53250773993808 | 0.0127932355639073 | 0.371427448424964 | 1.89301960330545 | BMP4//LGALS1//PLSCR1//TIMP1//ANXA1 |
| GO:0006928 | cellular component movement | Biological process | 7 | 932 | 38 | 13692 | 2.70623447029591 | 0.0130345483503398 | 0.371667921890145 | 1.88490401262403 | ROBO3//BMP4//MMP14//COL1A1//GRB7//SPP1//TIMP1 |
| GO:0045926 | negative regulation of growth | Biological process | 3 | 178 | 38 | 13692 | 6.07273802483737 | 0.0130558530955936 | 0.371667921890145 | 1.88419474524248 | DAB2//SPP1//BMP4 |
| GO:0048872 | homeostasis of number of cells | Biological process | 3 | 178 | 38 | 13692 | 6.07273802483737 | 0.0130558530955936 | 0.371667921890145 | 1.88419474524248 | ANXA1//BMP4//TIMP1 |
| GO:0006690 | icosanoid metabolic process | Biological process | 2 | 63 | 38 | 13692 | 11.4385964912281 | 0.0131684626450079 | 0.372455098294933 | 1.88046492388116 | PTGDS//ANXA1 |
| GO:0009991 | response to extracellular stimulus | Biological process | 5 | 515 | 38 | 13692 | 3.4982115482882 | 0.0133012337695715 | 0.373798774652573 | 1.87610807373473 | CP//MGP//COL1A1//BMP4//SPP1 |
| GO:0031016 | pancreas development | Biological process | 2 | 64 | 38 | 13692 | 11.2598684210526 | 0.0135696664934989 | 0.378913489856683 | 1.86743082601845 | BMP4//ANXA1 |
| GO:0042063 | gliogenesis | Biological process | 3 | 182 | 38 | 13692 | 5.93927125506073 | 0.0138558421305232 | 0.38445577152034 | 1.85836707352485 | MMP14//BMP4//ANXA1 |
| GO:0030177 | positive regulation of Wnt receptor signaling pathway | Biological process | 2 | 65 | 38 | 13692 | 11.0866396761134 | 0.0139761620061877 | 0.385355309654886 | 1.85461207382467 | COL1A1//DAB2 |
| GO:0050776 | regulation of immune response | Biological process | 4 | 340 | 38 | 13692 | 4.2390092879257 | 0.0141846125509529 | 0.386911733070842 | 1.8481825223483 | C3//CFD//PLSCR1//COL3A1 |
| GO:0070887 | cellular response to chemical stimulus | Biological process | 8 | 1182 | 38 | 13692 | 2.43868554635319 | 0.0142191399526867 | 0.386911733070842 | 1.84712667119403 | FRS3//COL1A1//COL3A1//SPP1//BMP4//ANXA1//LGALS1//PLSCR1 |
| GO:0032101 | regulation of response to external stimulus | Biological process | 4 | 341 | 38 | 13692 | 4.22657817564439 | 0.0143247589188687 | 0.386911733070842 | 1.84391267833691 | C3//ANXA1//SPP1//TGM2 |
| GO:0033559 | unsaturated fatty acid metabolic process | Biological process | 2 | 66 | 38 | 13692 | 10.9186602870813 | 0.0143879183462456 | 0.386911733070842 | 1.84200203553768 | PTGDS//ANXA1 |
| GO:0046942 | carboxylic acid transport | Biological process | 3 | 185 | 38 | 13692 | 5.84295874822191 | 0.0144738878247304 | 0.386911733070842 | 1.83941479755834 | SLC6A20//SLC27A2//ANXA1 |
| GO:0010647 | positive regulation of cell communication | Biological process | 6 | 733 | 38 | 13692 | 2.94937890428664 | 0.0147000293438128 | 0.388676720676267 | 1.83268179832371 | BMP4//TGM2//LGALS1//C3//COL1A1//DAB2 |
| GO:0023056 | positive regulation of signaling | Biological process | 6 | 734 | 38 | 13692 | 2.9453606768966 | 0.0147912105255543 | 0.388676720676267 | 1.82999628150987 | BMP4//TGM2//LGALS1//C3//COL1A1//DAB2 |
| GO:0010876 | lipid localization | Biological process | 3 | 187 | 38 | 13692 | 5.78046721080777 | 0.0148945458653314 | 0.388676720676267 | 1.82697273388481 | SLC27A2//PLSCR1//ANXA1 |
| GO:0015849 | organic acid transport | Biological process | 3 | 187 | 38 | 13692 | 5.78046721080777 | 0.0148945458653314 | 0.388676720676267 | 1.82697273388481 | SLC6A20//SLC27A2//ANXA1 |
| GO:0002062 | chondrocyte differentiation | Biological process | 2 | 68 | 38 | 13692 | 10.5975232198142 | 0.0152270907732791 | 0.395003348816897 | 1.81738306329559 | BMP4//TGFBI |
| GO:0051100 | negative regulation of binding | Biological process | 2 | 69 | 38 | 13692 | 10.4439359267735 | 0.0156544457974829 | 0.4037005316245 | 1.80536230277434 | DAB2//PLSCR1 |
| GO:0010627 | regulation of intracellular protein kinase cascade | Biological process | 5 | 540 | 38 | 13692 | 3.33625730994152 | 0.016048632916939 | 0.405540712510793 | 1.79456195652493 | IL22RA2//TGM2//LGALS1//C3//BMP4 |
| GO:0006959 | humoral immune response | Biological process | 2 | 70 | 38 | 13692 | 10.2947368421053 | 0.0160869395226883 | 0.405540712510793 | 1.79352657086884 | C3//CFD |
| GO:0060349 | bone morphogenesis | Biological process | 2 | 70 | 38 | 13692 | 10.2947368421053 | 0.0160869395226883 | 0.405540712510793 | 1.79352657086884 | BMP4//COL1A1 |
| GO:0071842 | cellular component organization at cellular level | Biological process | 13 | 2544 | 38 | 13692 | 1.84123634558093 | 0.0160958220750178 | 0.405540712510793 | 1.79328683733646 | ROBO3//LGALS1//LGALS3//TGFBI//RGD1562717//COL1A1//COL3A1//ANXA1//GRB7//PRPH//BMP4//SPP1//MMP14 |
| GO:0051248 | negative regulation of protein metabolic process | Biological process | 4 | 355 | 38 | 13692 | 4.05989621942179 | 0.0163804408377354 | 0.408562086741676 | 1.78567441449137 | GRB7//BMP4//TIMP1//HHATL |
| GO:0022603 | regulation of anatomical structure morphogenesis | Biological process | 5 | 543 | 38 | 13692 | 3.31782494911311 | 0.0164021275699213 | 0.408562086741676 | 1.78509981463845 | BMP4//COL1A1//C3//SPP1//DAB2 |
| GO:0048678 | response to axon injury | Biological process | 2 | 71 | 38 | 13692 | 10.1497405485545 | 0.0165245417204809 | 0.409285824308408 | 1.78187057607991 | SPP1//LGALS1 |
| GO:0008544 | epidermis development | Biological process | 3 | 195 | 38 | 13692 | 5.54331983805668 | 0.0166465105436877 | 0.409990461929926 | 1.77867678981204 | ANXA1//COL1A1//COL3A1 |
| GO:0055085 | transmembrane transport | Biological process | 5 | 546 | 38 | 13692 | 3.2995951417004 | 0.016760836406306 | 0.410500038018131 | 1.77570431281912 | SLC6A20//CP//S100A6//BMP4//SLC16A10 |
| GO:0051607 | defense response to virus | Biological process | 2 | 72 | 38 | 13692 | 10.0087719298246 | 0.016967222282898 | 0.413246124934582 | 1.77038925054917 | PLSCR1//IFITM1 |
| GO:0010740 | positive regulation of intracellular protein kinase cascade | Biological process | 4 | 360 | 38 | 13692 | 4.00350877192982 | 0.017157529700912 | 0.415572432092808 | 1.76554524064181 | TGM2//LGALS1//C3//BMP4 |
| GO:0080134 | regulation of response to stress | Biological process | 5 | 554 | 38 | 13692 | 3.25194755842675 | 0.0177431252953879 | 0.425737956547899 | 1.75096988061499 | C3//ANXA1//PLSCR1//SPP1//TGM2 |
| GO:0031345 | negative regulation of cell projection organization | Biological process | 2 | 74 | 38 | 13692 | 9.73826458036984 | 0.0178676986695701 | 0.425737956547899 | 1.74793138031294 | LGALS1//SPP1 |
| GO:0001775 | cell activation | Biological process | 5 | 555 | 38 | 13692 | 3.24608819345661 | 0.0178685638697111 | 0.425737956547899 | 1.74791035116102 | LGALS1//PLSCR1//BMP4//ANXA1//TIMP1 |
| GO:0045807 | positive regulation of endocytosis | Biological process | 2 | 75 | 38 | 13692 | 9.60842105263158 | 0.0183254348764971 | 0.434263278370612 | 1.73694571042642 | C3//ANXA1 |
| GO:0035282 | segmentation | Biological process | 2 | 76 | 38 | 13692 | 9.48199445983379 | 0.018788130212584 | 0.44283420888155 | 1.72611643856699 | IFITM1//BMP4 |
| GO:0030334 | regulation of cell migration | Biological process | 4 | 375 | 38 | 13692 | 3.84336842105263 | 0.0196271283553836 | 0.460135458342255 | 1.70714323736591 | BMP4//COL1A1//GRB7//TIMP1 |
| GO:0050896 | response to stimulus | Biological process | 25 | 6556 | 38 | 13692 | 1.37399248579044 | 0.0198255054432594 | 0.462313914166219 | 1.70277573170669 | MMP14//C3//LGALS1//ANXA1//BMP4//PLSCR1//F5//GRB7//MGP//COL1A1//SPP1//CFD//COL3A1//TGM2//ROBO3//CP//FRS3//IFITM1//TIMP1//LUM//CLDN4//IL22RA2//LGALS3//PTGDS//DAB2 |
| GO:0015718 | monocarboxylic acid transport | Biological process | 2 | 79 | 38 | 13692 | 9.12191872085277 | 0.0202056764676165 | 0.468686167375824 | 1.69452660519485 | SLC27A2//ANXA1 |
| GO:0030098 | lymphocyte differentiation | Biological process | 3 | 212 | 38 | 13692 | 5.09880834160874 | 0.0207404551422237 | 0.476053169337742 | 1.68318171739792 | LGALS1//BMP4//ANXA1 |
| GO:0033273 | response to vitamin | Biological process | 3 | 212 | 38 | 13692 | 5.09880834160874 | 0.0207404551422237 | 0.476053169337742 | 1.68318171739792 | BMP4//SPP1//COL1A1 |
| GO:0048514 | blood vessel morphogenesis | Biological process | 4 | 383 | 38 | 13692 | 3.76308918510375 | 0.0210303892411352 | 0.48010933042173 | 1.67715268909624 | BMP4//MMP14//TGFBI//C3 |
| GO:0030282 | bone mineralization | Biological process | 2 | 81 | 38 | 13692 | 8.89668615984405 | 0.0211749650402133 | 0.48010933042173 | 1.6741772980152 | MGP//BMP4 |
| GO:0000003 | reproduction | Biological process | 7 | 1027 | 38 | 13692 | 2.45590119407574 | 0.0212457139830784 | 0.48010933042173 | 1.67272866943675 | BMP4//MMP14//CLDN4//PLSCR1//ANXA1//TIMP1//ZAR1 |
| GO:0071841 | cellular component organization or biogenesis at cellular level | Biological process | 13 | 2640 | 38 | 13692 | 1.77428229665072 | 0.0215833565113669 | 0.482168777147738 | 1.66588101558398 | ROBO3//LGALS1//LGALS3//TGFBI//RGD1562717//COL1A1//COL3A1//ANXA1//GRB7//PRPH//BMP4//SPP1//MMP14 |
| GO:0030218 | erythrocyte differentiation | Biological process | 2 | 82 | 38 | 13692 | 8.78818998716303 | 0.021666799520553 | 0.482168777147738 | 1.66420523511375 | TIMP1//BMP4 |
| GO:0043200 | response to amino acid stimulus | Biological process | 2 | 82 | 38 | 13692 | 8.78818998716303 | 0.021666799520553 | 0.482168777147738 | 1.66420523511375 | COL1A1//COL3A1 |
| GO:0034097 | response to cytokine stimulus | Biological process | 4 | 389 | 38 | 13692 | 3.70504667839264 | 0.0221227284559468 | 0.486899400904473 | 1.65516131135075 | PLSCR1//ANXA1//COL3A1//TIMP1 |
| GO:2000145 | regulation of cell motility | Biological process | 4 | 389 | 38 | 13692 | 3.70504667839264 | 0.0221227284559468 | 0.486899400904473 | 1.65516131135075 | BMP4//COL1A1//GRB7//TIMP1 |
| GO:0006508 | proteolysis | Biological process | 6 | 804 | 38 | 13692 | 2.68892380204242 | 0.0222125639098756 | 0.486899400904473 | 1.65340130963498 | SERPINB1A//DAB2//TIMP1//CFD//MMP14//KLK7 |
| GO:0071363 | cellular response to growth factor stimulus | Biological process | 3 | 219 | 38 | 13692 | 4.93583273251622 | 0.0225737394402075 | 0.491891415517762 | 1.64639649215276 | FRS3//COL1A1//BMP4 |
| GO:0070374 | positive regulation of ERK1 and ERK2 cascade | Biological process | 2 | 84 | 38 | 13692 | 8.57894736842105 | 0.0226647048208458 | 0.491891415517762 | 1.64464993270556 | C3//BMP4 |
| GO:0030335 | positive regulation of cell migration | Biological process | 3 | 222 | 38 | 13692 | 4.86913229018492 | 0.0233858560761859 | 0.505042330236448 | 1.63104672742926 | BMP4//COL1A1//GRB7 |
| GO:0001935 | endothelial cell proliferation | Biological process | 2 | 86 | 38 | 13692 | 8.37943696450428 | 0.0236814011900075 | 0.506435428375575 | 1.62559260469045 | BMP4//MMP14 |
| GO:0050768 | negative regulation of neurogenesis | Biological process | 2 | 86 | 38 | 13692 | 8.37943696450428 | 0.0236814011900075 | 0.506435428375575 | 1.62559260469045 | SPP1//BMP4 |
| GO:0006810 | transport | Biological process | 13 | 2679 | 38 | 13692 | 1.74845287911829 | 0.0241872829889743 | 0.51245624214731 | 1.61641291415851 | SLC6A20//DAB2//COL1A1//CP//SLC27A2//PLSCR1//ANXA1//S100A6//TGM2//C3//SLC16A10//BMP4//PTGDS |
| GO:0061039 | ovum-producing ovary development | Biological process | 2 | 87 | 38 | 13692 | 8.28312159709619 | 0.0241967249371563 | 0.51245624214731 | 1.61624341244827 | BMP4//MMP14 |
| GO:2000147 | positive regulation of cell motility | Biological process | 3 | 227 | 38 | 13692 | 4.76188268026895 | 0.0247746355708879 | 0.520335512201312 | 1.60599272516626 | BMP4//COL1A1//GRB7 |
| GO:0002684 | positive regulation of immune system process | Biological process | 4 | 403 | 38 | 13692 | 3.57633537939141 | 0.0248061409785753 | 0.520335512201312 | 1.60544079244142 | C3//CFD//LGALS1//PLSCR1 |
| GO:0034101 | erythrocyte homeostasis | Biological process | 2 | 91 | 38 | 13692 | 7.91902834008097 | 0.0263038661750179 | 0.530020628216285 | 1.57998041367468 | BMP4//TIMP1 |
| GO:0051270 | regulation of cellular component movement | Biological process | 4 | 412 | 38 | 13692 | 3.4982115482882 | 0.0266318510992553 | 0.530020628216285 | 1.57459864601873 | BMP4//COL1A1//GRB7//TIMP1 |
| GO:0030099 | myeloid cell differentiation | Biological process | 3 | 234 | 38 | 13692 | 4.61943319838057 | 0.0267929494902846 | 0.530020628216285 | 1.57197947463941 | BMP4//TIMP1//PLSCR1 |
| GO:0051272 | positive regulation of cellular component movement | Biological process | 3 | 234 | 38 | 13692 | 4.61943319838057 | 0.0267929494902846 | 0.530020628216285 | 1.57197947463941 | BMP4//COL1A1//GRB7 |
| GO:0040017 | positive regulation of locomotion | Biological process | 3 | 236 | 38 | 13692 | 4.58028545941124 | 0.0273854640006962 | 0.530020628216285 | 1.56247989628222 | BMP4//COL1A1//GRB7 |
| GO:0002335 | mature B cell differentiation | Biological process | 1 | 10 | 38 | 13692 | 36.0315789473684 | 0.0274182718194458 | 0.530020628216285 | 1.56195992237405 | LGALS1 |
| GO:0002524 | hypersensitivity | Biological process | 1 | 10 | 38 | 13692 | 36.0315789473684 | 0.0274182718194458 | 0.530020628216285 | 1.56195992237405 | C3 |
| GO:0002674 | negative regulation of acute inflammatory response | Biological process | 1 | 10 | 38 | 13692 | 36.0315789473684 | 0.0274182718194458 | 0.530020628216285 | 1.56195992237405 | ANXA1 |
| GO:0043249 | erythrocyte maturation | Biological process | 1 | 10 | 38 | 13692 | 36.0315789473684 | 0.0274182718194458 | 0.530020628216285 | 1.56195992237405 | TIMP1 |
| GO:0043587 | tongue morphogenesis | Biological process | 1 | 10 | 38 | 13692 | 36.0315789473684 | 0.0274182718194458 | 0.530020628216285 | 1.56195992237405 | BMP4 |
| GO:0043589 | skin morphogenesis | Biological process | 1 | 10 | 38 | 13692 | 36.0315789473684 | 0.0274182718194458 | 0.530020628216285 | 1.56195992237405 | COL1A1 |
| GO:0045662 | negative regulation of myoblast differentiation | Biological process | 1 | 10 | 38 | 13692 | 36.0315789473684 | 0.0274182718194458 | 0.530020628216285 | 1.56195992237405 | BMP4 |
| GO:0048668 | collateral sprouting | Biological process | 1 | 10 | 38 | 13692 | 36.0315789473684 | 0.0274182718194458 | 0.530020628216285 | 1.56195992237405 | SPP1 |
| GO:0060390 | regulation of SMAD protein import into nucleus | Biological process | 1 | 10 | 38 | 13692 | 36.0315789473684 | 0.0274182718194458 | 0.530020628216285 | 1.56195992237405 | BMP4 |
| GO:0060513 | prostatic bud formation | Biological process | 1 | 10 | 38 | 13692 | 36.0315789473684 | 0.0274182718194458 | 0.530020628216285 | 1.56195992237405 | BMP4 |
| GO:0072044 | collecting duct development | Biological process | 1 | 10 | 38 | 13692 | 36.0315789473684 | 0.0274182718194458 | 0.530020628216285 | 1.56195992237405 | BMP4 |
| GO:2000050 | regulation of non-canonical Wnt receptor signaling pathway | Biological process | 1 | 10 | 38 | 13692 | 36.0315789473684 | 0.0274182718194458 | 0.530020628216285 | 1.56195992237405 | DAB2 |
| GO:0050793 | regulation of developmental process | Biological process | 8 | 1333 | 38 | 13692 | 2.16243534567852 | 0.0274440425650312 | 0.530020628216285 | 1.5615519157651 | BMP4//COL1A1//LGALS1//MGP//C3//SPP1//TIMP1//DAB2 |
| GO:0051234 | establishment of localization | Biological process | 13 | 2726 | 38 | 13692 | 1.71830713982315 | 0.0276388557315281 | 0.531441857574646 | 1.55847994102903 | SLC6A20//PTGDS//DAB2//COL1A1//CP//SLC27A2//PLSCR1//ANXA1//S100A6//TGM2//C3//SLC16A10//BMP4 |
| GO:0040012 | regulation of locomotion | Biological process | 4 | 418 | 38 | 13692 | 3.44799798539411 | 0.0278932000343281 | 0.532400195207502 | 1.55450165864439 | BMP4//COL1A1//GRB7//TIMP1 |
| GO:0071383 | cellular response to steroid hormone stimulus | Biological process | 2 | 94 | 38 | 13692 | 7.66629339305711 | 0.0279315795843352 | 0.532400195207502 | 1.55390450341956 | BMP4//ANXA1 |
| GO:0050778 | positive regulation of immune response | Biological process | 3 | 239 | 38 | 13692 | 4.52279233648976 | 0.028287435651693 | 0.535373240763244 | 1.54840642096992 | C3//CFD//PLSCR1 |
| GO:0043393 | regulation of protein binding | Biological process | 2 | 95 | 38 | 13692 | 7.58559556786704 | 0.0284830237637056 | 0.535373240763244 | 1.54541390779556 | DAB2//PLSCR1 |
| GO:0048660 | regulation of smooth muscle cell proliferation | Biological process | 2 | 95 | 38 | 13692 | 7.58559556786704 | 0.0284830237637056 | 0.535373240763244 | 1.54541390779556 | BMP4//TGM2 |
| GO:0002863 | positive regulation of inflammatory response to antigenic stimulus | Biological process | 1 | 11 | 38 | 13692 | 32.755980861244 | 0.030119492815708 | 0.535373240763244 | 1.52115234552558 | C3 |
| GO:0003128 | heart field specification | Biological process | 1 | 11 | 38 | 13692 | 32.755980861244 | 0.030119492815708 | 0.535373240763244 | 1.52115234552558 | BMP4 |
| GO:0003323 | type B pancreatic cell development | Biological process | 1 | 11 | 38 | 13692 | 32.755980861244 | 0.030119492815708 | 0.535373240763244 | 1.52115234552558 | BMP4 |
| GO:0010225 | response to UV-C | Biological process | 1 | 11 | 38 | 13692 | 32.755980861244 | 0.030119492815708 | 0.535373240763244 | 1.52115234552558 | PLSCR1 |
| GO:0030449 | regulation of complement activation | Biological process | 1 | 11 | 38 | 13692 | 32.755980861244 | 0.030119492815708 | 0.535373240763244 | 1.52115234552558 | C3 |
| GO:0042487 | regulation of odontogenesis of dentin-containing tooth | Biological process | 1 | 11 | 38 | 13692 | 32.755980861244 | 0.030119492815708 | 0.535373240763244 | 1.52115234552558 | BMP4 |
| GO:0060363 | cranial suture morphogenesis | Biological process | 1 | 11 | 38 | 13692 | 32.755980861244 | 0.030119492815708 | 0.535373240763244 | 1.52115234552558 | BMP4 |
| GO:0061036 | positive regulation of cartilage development | Biological process | 1 | 11 | 38 | 13692 | 32.755980861244 | 0.030119492815708 | 0.535373240763244 | 1.52115234552558 | BMP4 |
| GO:0070208 | protein heterotrimerization | Biological process | 1 | 11 | 38 | 13692 | 32.755980861244 | 0.030119492815708 | 0.535373240763244 | 1.52115234552558 | COL1A1 |
| GO:0070233 | negative regulation of T cell apoptotic process | Biological process | 1 | 11 | 38 | 13692 | 32.755980861244 | 0.030119492815708 | 0.535373240763244 | 1.52115234552558 | BMP4 |
| GO:0070243 | regulation of thymocyte apoptotic process | Biological process | 1 | 11 | 38 | 13692 | 32.755980861244 | 0.030119492815708 | 0.535373240763244 | 1.52115234552558 | BMP4 |
| GO:0072498 | embryonic skeletal joint development | Biological process | 1 | 11 | 38 | 13692 | 32.755980861244 | 0.030119492815708 | 0.535373240763244 | 1.52115234552558 | BMP4 |
| GO:2000257 | regulation of protein activation cascade | Biological process | 1 | 11 | 38 | 13692 | 32.755980861244 | 0.030119492815708 | 0.535373240763244 | 1.52115234552558 | C3 |
| GO:0048659 | smooth muscle cell proliferation | Biological process | 2 | 98 | 38 | 13692 | 7.35338345864662 | 0.0301635927163598 | 0.535373240763244 | 1.52051693190158 | BMP4//TGM2 |
| GO:0019538 | protein metabolic process | Biological process | 15 | 3387 | 38 | 13692 | 1.5957298028064 | 0.0316966638049098 | 0.542548184556926 | 1.49898644653197 | BMP4//DAB2//C3//CFD//MMP14//KLK7//F5//GRB7//ANXA1//COL3A1//TGM2//SERPINB1A//IL22RA2//TIMP1//HHATL |
| GO:0016485 | protein processing | Biological process | 2 | 102 | 38 | 13692 | 7.06501547987616 | 0.0324645603686199 | 0.542548184556926 | 1.48859047391783 | C3//MMP14 |
| GO:0060348 | bone development | Biological process | 2 | 102 | 38 | 13692 | 7.06501547987616 | 0.0324645603686199 | 0.542548184556926 | 1.48859047391783 | BMP4//COL1A1 |
| GO:0002864 | regulation of acute inflammatory response to antigenic stimulus | Biological process | 1 | 12 | 38 | 13692 | 30.0263157894737 | 0.03281340841201 | 0.542548184556926 | 1.48394865600083 | C3 |
| GO:0003337 | mesenchymal to epithelial transition involved in metanephros morphogenesis | Biological process | 1 | 12 | 38 | 13692 | 30.0263157894737 | 0.03281340841201 | 0.542548184556926 | 1.48394865600083 | BMP4 |
| GO:0006825 | copper ion transport | Biological process | 1 | 12 | 38 | 13692 | 30.0263157894737 | 0.03281340841201 | 0.542548184556926 | 1.48394865600083 | CP |
| GO:0032331 | negative regulation of chondrocyte differentiation | Biological process | 1 | 12 | 38 | 13692 | 30.0263157894737 | 0.03281340841201 | 0.542548184556926 | 1.48394865600083 | BMP4 |
| GO:0034505 | tooth mineralization | Biological process | 1 | 12 | 38 | 13692 | 30.0263157894737 | 0.03281340841201 | 0.542548184556926 | 1.48394865600083 | COL1A1 |
| GO:0035456 | response to interferon-beta | Biological process | 1 | 12 | 38 | 13692 | 30.0263157894737 | 0.03281340841201 | 0.542548184556926 | 1.48394865600083 | PLSCR1 |
| GO:0048715 | negative regulation of oligodendrocyte differentiation | Biological process | 1 | 12 | 38 | 13692 | 30.0263157894737 | 0.03281340841201 | 0.542548184556926 | 1.48394865600083 | BMP4 |
| GO:0060206 | estrous cycle phase | Biological process | 1 | 12 | 38 | 13692 | 30.0263157894737 | 0.03281340841201 | 0.542548184556926 | 1.48394865600083 | ANXA1 |
| GO:0070242 | thymocyte apoptotic process | Biological process | 1 | 12 | 38 | 13692 | 30.0263157894737 | 0.03281340841201 | 0.542548184556926 | 1.48394865600083 | BMP4 |
| GO:0070365 | hepatocyte differentiation | Biological process | 1 | 12 | 38 | 13692 | 30.0263157894737 | 0.03281340841201 | 0.542548184556926 | 1.48394865600083 | ANXA1 |
| GO:0072176 | nephric duct development | Biological process | 1 | 12 | 38 | 13692 | 30.0263157894737 | 0.03281340841201 | 0.542548184556926 | 1.48394865600083 | BMP4 |
| GO:0072189 | ureter development | Biological process | 1 | 12 | 38 | 13692 | 30.0263157894737 | 0.03281340841201 | 0.542548184556926 | 1.48394865600083 | BMP4 |
| GO:0090192 | regulation of glomerulus development | Biological process | 1 | 12 | 38 | 13692 | 30.0263157894737 | 0.03281340841201 | 0.542548184556926 | 1.48394865600083 | BMP4 |
| GO:0097094 | craniofacial suture morphogenesis | Biological process | 1 | 12 | 38 | 13692 | 30.0263157894737 | 0.03281340841201 | 0.542548184556926 | 1.48394865600083 | BMP4 |
| GO:0042592 | homeostatic process | Biological process | 7 | 1125 | 38 | 13692 | 2.2419649122807 | 0.0330040287855854 | 0.542548184556926 | 1.48143304277202 | ANXA1//CP//BMP4//TIMP1//SPP1//TGM2//LGALS1 |
| GO:0032526 | response to retinoic acid | Biological process | 2 | 103 | 38 | 13692 | 6.99642309657639 | 0.0330503753142788 | 0.542548184556926 | 1.4808236043782 | COL1A1//BMP4 |
| GO:0009894 | regulation of catabolic process | Biological process | 4 | 442 | 38 | 13692 | 3.26077637532746 | 0.0332962391481286 | 0.542548184556926 | 1.47760481782864 | DAB2//SERPINB1A//TIMP1//PLSCR1 |
| GO:0010721 | negative regulation of cell development | Biological process | 2 | 104 | 38 | 13692 | 6.92914979757085 | 0.0336403663867344 | 0.542548184556926 | 1.47313928282447 | SPP1//BMP4 |
| GO:0048729 | tissue morphogenesis | Biological process | 4 | 449 | 38 | 13692 | 3.20994021802837 | 0.0349808542157077 | 0.542548184556926 | 1.45616958946459 | BMP4//COL1A1//TGM2 |
| GO:0048706 | embryonic skeletal system development | Biological process | 2 | 107 | 38 | 13692 | 6.73487456960157 | 0.0354351333573414 | 0.542548184556926 | 1.45056592846621 | BMP4//COL1A1 |
| GO:0002922 | positive regulation of humoral immune response | Biological process | 1 | 13 | 38 | 13692 | 27.7165991902834 | 0.0355000378330872 | 0.542548184556926 | 1.4497711841085 | C3 |
| GO:0003333 | amino acid transmembrane transport | Biological process | 1 | 13 | 38 | 13692 | 27.7165991902834 | 0.0355000378330872 | 0.542548184556926 | 1.4497711841085 | SLC6A20 |
| GO:0018345 | protein palmitoylation | Biological process | 1 | 13 | 38 | 13692 | 27.7165991902834 | 0.0355000378330872 | 0.542548184556926 | 1.4497711841085 | HHATL |
| GO:0032781 | positive regulation of ATPase activity | Biological process | 1 | 13 | 38 | 13692 | 27.7165991902834 | 0.0355000378330872 | 0.542548184556926 | 1.4497711841085 | PLSCR1 |
| GO:0048679 | regulation of axon regeneration | Biological process | 1 | 13 | 38 | 13692 | 27.7165991902834 | 0.0355000378330872 | 0.542548184556926 | 1.4497711841085 | SPP1 |
| GO:0060601 | lateral sprouting from an epithelium | Biological process | 1 | 13 | 38 | 13692 | 27.7165991902834 | 0.0355000378330872 | 0.542548184556926 | 1.4497711841085 | BMP4 |
| GO:0061318 | renal filtration cell differentiation | Biological process | 1 | 13 | 38 | 13692 | 27.7165991902834 | 0.0355000378330872 | 0.542548184556926 | 1.4497711841085 | BMP4 |
| GO:0070570 | regulation of neuron projection regeneration | Biological process | 1 | 13 | 38 | 13692 | 27.7165991902834 | 0.0355000378330872 | 0.542548184556926 | 1.4497711841085 | SPP1 |
| GO:0072010 | glomerular epithelium development | Biological process | 1 | 13 | 38 | 13692 | 27.7165991902834 | 0.0355000378330872 | 0.542548184556926 | 1.4497711841085 | BMP4 |
| GO:0072112 | glomerular visceral epithelial cell differentiation | Biological process | 1 | 13 | 38 | 13692 | 27.7165991902834 | 0.0355000378330872 | 0.542548184556926 | 1.4497711841085 | BMP4 |
| GO:0072311 | glomerular epithelial cell differentiation | Biological process | 1 | 13 | 38 | 13692 | 27.7165991902834 | 0.0355000378330872 | 0.542548184556926 | 1.4497711841085 | BMP4 |
| GO:0090184 | positive regulation of kidney development | Biological process | 1 | 13 | 38 | 13692 | 27.7165991902834 | 0.0355000378330872 | 0.542548184556926 | 1.4497711841085 | BMP4 |
| GO:0008585 | female gonad development | Biological process | 2 | 108 | 38 | 13692 | 6.67251461988304 | 0.0360415664643563 | 0.542548184556926 | 1.44319634166518 | BMP4//MMP14 |
| GO:0042542 | response to hydrogen peroxide | Biological process | 2 | 108 | 38 | 13692 | 6.67251461988304 | 0.0360415664643563 | 0.542548184556926 | 1.44319634166518 | ANXA1//COL1A1 |
| GO:0051604 | protein maturation | Biological process | 2 | 108 | 38 | 13692 | 6.67251461988304 | 0.0360415664643563 | 0.542548184556926 | 1.44319634166518 | C3//MMP14 |
| GO:0042476 | odontogenesis | Biological process | 2 | 109 | 38 | 13692 | 6.6112988894254 | 0.0366520449954859 | 0.542548184556926 | 1.43590178894978 | COL1A1//BMP4 |
| GO:0040008 | regulation of growth | Biological process | 4 | 457 | 38 | 13692 | 3.15374870436485 | 0.0369666915862556 | 0.542548184556926 | 1.43218941580847 | DAB2//C3//SPP1//BMP4 |
| GO:0032501 | multicellular organismal process | Biological process | 22 | 5780 | 38 | 13692 | 1.37144418138773 | 0.0371033768037618 | 0.542548184556926 | 1.43058656314767 | BMP4//COL1A1//LGALS3//COL3A1//MGP//MMP14//TGFBI//SPP1//DAB2//IFITM1//ROBO3//CP//TGM2//LGALS1//C3//F5//ZAR1//PLSCR1//ANXA1//TIMP1//PTGDS//LUM |
| GO:0007623 | circadian rhythm | Biological process | 2 | 111 | 38 | 13692 | 6.49217638691323 | 0.0378850351490015 | 0.542548184556926 | 1.42153230548436 | PTGDS//CLDN4 |
| GO:0048565 | digestive tract development | Biological process | 2 | 111 | 38 | 13692 | 6.49217638691323 | 0.0378850351490015 | 0.542548184556926 | 1.42153230548436 | BMP4//COL3A1 |
| GO:0006631 | fatty acid metabolic process | Biological process | 3 | 269 | 38 | 13692 | 4.01839170416748 | 0.0381738896160022 | 0.542548184556926 | 1.41823358663975 | SLC27A2//PTGDS//ANXA1 |
| GO:0002320 | lymphoid progenitor cell differentiation | Biological process | 1 | 14 | 38 | 13692 | 25.7368421052632 | 0.0381794002544885 | 0.542548184556926 | 1.41817089805372 | BMP4 |
| GO:0002714 | positive regulation of B cell mediated immunity | Biological process | 1 | 14 | 38 | 13692 | 25.7368421052632 | 0.0381794002544885 | 0.542548184556926 | 1.41817089805372 | C3 |
| GO:0002891 | positive regulation of immunoglobulin mediated immune response | Biological process | 1 | 14 | 38 | 13692 | 25.7368421052632 | 0.0381794002544885 | 0.542548184556926 | 1.41817089805372 | C3 |
| GO:0003309 | type B pancreatic cell differentiation | Biological process | 1 | 14 | 38 | 13692 | 25.7368421052632 | 0.0381794002544885 | 0.542548184556926 | 1.41817089805372 | BMP4 |
| GO:0006699 | bile acid biosynthetic process | Biological process | 1 | 14 | 38 | 13692 | 25.7368421052632 | 0.0381794002544885 | 0.542548184556926 | 1.41817089805372 | SLC27A2 |
| GO:0031365 | N-terminal protein amino acid modification | Biological process | 1 | 14 | 38 | 13692 | 25.7368421052632 | 0.0381794002544885 | 0.542548184556926 | 1.41817089805372 | HHATL |
| GO:0045601 | regulation of endothelial cell differentiation | Biological process | 1 | 14 | 38 | 13692 | 25.7368421052632 | 0.0381794002544885 | 0.542548184556926 | 1.41817089805372 | BMP4 |
| GO:0045723 | positive regulation of fatty acid biosynthetic process | Biological process | 1 | 14 | 38 | 13692 | 25.7368421052632 | 0.0381794002544885 | 0.542548184556926 | 1.41817089805372 | ANXA1 |
| GO:0045780 | positive regulation of bone resorption | Biological process | 1 | 14 | 38 | 13692 | 25.7368421052632 | 0.0381794002544885 | 0.542548184556926 | 1.41817089805372 | SPP1 |
| GO:0045844 | positive regulation of striated muscle tissue development | Biological process | 1 | 14 | 38 | 13692 | 25.7368421052632 | 0.0381794002544885 | 0.542548184556926 | 1.41817089805372 | BMP4 |
| GO:0046852 | positive regulation of bone remodeling | Biological process | 1 | 14 | 38 | 13692 | 25.7368421052632 | 0.0381794002544885 | 0.542548184556926 | 1.41817089805372 | SPP1 |
| GO:0048636 | positive regulation of muscle organ development | Biological process | 1 | 14 | 38 | 13692 | 25.7368421052632 | 0.0381794002544885 | 0.542548184556926 | 1.41817089805372 | BMP4 |
| GO:0060438 | trachea development | Biological process | 1 | 14 | 38 | 13692 | 25.7368421052632 | 0.0381794002544885 | 0.542548184556926 | 1.41817089805372 | BMP4 |
| GO:0061311 | cell surface receptor signaling pathway involved in heart development | Biological process | 1 | 14 | 38 | 13692 | 25.7368421052632 | 0.0381794002544885 | 0.542548184556926 | 1.41817089805372 | BMP4 |
| GO:0072074 | kidney mesenchyme development | Biological process | 1 | 14 | 38 | 13692 | 25.7368421052632 | 0.0381794002544885 | 0.542548184556926 | 1.41817089805372 | BMP4 |
| GO:0072111 | cell proliferation involved in kidney development | Biological process | 1 | 14 | 38 | 13692 | 25.7368421052632 | 0.0381794002544885 | 0.542548184556926 | 1.41817089805372 | BMP4 |
| GO:0072283 | metanephric renal vesicle morphogenesis | Biological process | 1 | 14 | 38 | 13692 | 25.7368421052632 | 0.0381794002544885 | 0.542548184556926 | 1.41817089805372 | BMP4 |
| GO:0071702 | organic substance transport | Biological process | 4 | 462 | 38 | 13692 | 3.11961722488038 | 0.0382407365483782 | 0.542548184556926 | 1.4174737514426 | SLC6A20//SLC27A2//PLSCR1//ANXA1 |
| GO:0070372 | regulation of ERK1 and ERK2 cascade | Biological process | 2 | 112 | 38 | 13692 | 6.43421052631579 | 0.0385074954441123 | 0.544570516216091 | 1.41445472729193 | C3//BMP4 |
| GO:0007160 | cell-matrix adhesion | Biological process | 2 | 113 | 38 | 13692 | 6.3772706101537 | 0.0391338985080656 | 0.551649553245529 | 1.40744688564891 | MMP14//COL3A1 |
| GO:0006979 | response to oxidative stress | Biological process | 3 | 274 | 38 | 13692 | 3.945063388398 | 0.0399735976735145 | 0.561680295515024 | 1.39822676293725 | COL1A1//ANXA1//MMP14 |
| GO:0001101 | response to acid | Biological process | 2 | 115 | 38 | 13692 | 6.26636155606407 | 0.0403984314385483 | 0.565125246034974 | 1.3936354970381 | COL1A1//COL3A1 |
| GO:0043401 | steroid hormone mediated signaling pathway | Biological process | 1 | 15 | 38 | 13692 | 24.0210526315789 | 0.040851514802692 | 0.565125246034974 | 1.38879183489037 | BMP4 |
| GO:0051150 | regulation of smooth muscle cell differentiation | Biological process | 1 | 15 | 38 | 13692 | 24.0210526315789 | 0.040851514802692 | 0.565125246034974 | 1.38879183489037 | BMP4 |
| GO:0060442 | branching involved in prostate gland morphogenesis | Biological process | 1 | 15 | 38 | 13692 | 24.0210526315789 | 0.040851514802692 | 0.565125246034974 | 1.38879183489037 | BMP4 |
| GO:0007596 | blood coagulation | Biological process | 2 | 116 | 38 | 13692 | 6.21234119782214 | 0.0410365108127778 | 0.565125246034974 | 1.38682957282745 | F5//C3 |
| GO:0022612 | gland morphogenesis | Biological process | 2 | 116 | 38 | 13692 | 6.21234119782214 | 0.0410365108127778 | 0.565125246034974 | 1.38682957282745 | BMP4//TGM2 |
| GO:0006633 | fatty acid biosynthetic process | Biological process | 2 | 117 | 38 | 13692 | 6.15924426450742 | 0.0416784319705369 | 0.565125246034974 | 1.38008862846461 | PTGDS//ANXA1 |
| GO:0006694 | steroid biosynthetic process | Biological process | 2 | 117 | 38 | 13692 | 6.15924426450742 | 0.0416784319705369 | 0.565125246034974 | 1.38008862846461 | SLC27A2//TIMP1 |
| GO:0007599 | hemostasis | Biological process | 2 | 117 | 38 | 13692 | 6.15924426450742 | 0.0416784319705369 | 0.565125246034974 | 1.38008862846461 | C3//F5 |
| GO:0002694 | regulation of leukocyte activation | Biological process | 3 | 279 | 38 | 13692 | 3.87436332767402 | 0.0418162317876917 | 0.565125246034974 | 1.37865510560601 | LGALS1//PLSCR1//BMP4 |
| GO:0035148 | tube formation | Biological process | 2 | 118 | 38 | 13692 | 6.10704727921499 | 0.0423241699237492 | 0.565125246034974 | 1.37341155066901 | BMP4//TGM2 |
| GO:0042110 | T cell activation | Biological process | 3 | 281 | 38 | 13692 | 3.84678778797528 | 0.0425652542844223 | 0.565125246034974 | 1.37094476779278 | LGALS1//BMP4//ANXA1 |
| GO:0050817 | coagulation | Biological process | 2 | 119 | 38 | 13692 | 6.05572755417957 | 0.04297369978693 | 0.565125246034974 | 1.36679725446415 | C3//F5 |
| GO:0002507 | tolerance induction | Biological process | 1 | 16 | 38 | 13692 | 22.5197368421053 | 0.0435164005552324 | 0.565125246034974 | 1.36134703434961 | C3 |
| GO:0007184 | SMAD protein import into nucleus | Biological process | 1 | 16 | 38 | 13692 | 22.5197368421053 | 0.0435164005552324 | 0.565125246034974 | 1.36134703434961 | BMP4 |
| GO:0010575 | positive regulation vascular endothelial growth factor production | Biological process | 1 | 16 | 38 | 13692 | 22.5197368421053 | 0.0435164005552324 | 0.565125246034974 | 1.36134703434961 | C3 |
| GO:0021904 | dorsal/ventral neural tube patterning | Biological process | 1 | 16 | 38 | 13692 | 22.5197368421053 | 0.0435164005552324 | 0.565125246034974 | 1.36134703434961 | BMP4 |
| GO:0031295 | T cell costimulation | Biological process | 1 | 16 | 38 | 13692 | 22.5197368421053 | 0.0435164005552324 | 0.565125246034974 | 1.36134703434961 | LGALS1 |
| GO:0034110 | regulation of homotypic cell-cell adhesion | Biological process | 1 | 16 | 38 | 13692 | 22.5197368421053 | 0.0435164005552324 | 0.565125246034974 | 1.36134703434961 | LGALS1 |
| GO:0045071 | negative regulation of viral genome replication | Biological process | 1 | 16 | 38 | 13692 | 22.5197368421053 | 0.0435164005552324 | 0.565125246034974 | 1.36134703434961 | PLSCR1 |
| GO:0051043 | regulation of membrane protein ectodomain proteolysis | Biological process | 1 | 16 | 38 | 13692 | 22.5197368421053 | 0.0435164005552324 | 0.565125246034974 | 1.36134703434961 | TIMP1 |
| GO:0072215 | regulation of metanephros development | Biological process | 1 | 16 | 38 | 13692 | 22.5197368421053 | 0.0435164005552324 | 0.565125246034974 | 1.36134703434961 | BMP4 |
| GO:0043123 | positive regulation of I-kappaB kinase/NF-kappaB cascade | Biological process | 2 | 120 | 38 | 13692 | 6.00526315789474 | 0.0436269967768335 | 0.565125246034974 | 1.36024468222612 | TGM2//LGALS1 |
| GO:0055123 | digestive system development | Biological process | 2 | 120 | 38 | 13692 | 6.00526315789474 | 0.0436269967768335 | 0.565125246034974 | 1.36024468222612 | COL3A1//BMP4 |
| GO:0060828 | regulation of canonical Wnt receptor signaling pathway | Biological process | 2 | 120 | 38 | 13692 | 6.00526315789474 | 0.0436269967768335 | 0.565125246034974 | 1.36024468222612 | DAB2//COL1A1 |
| GO:0045321 | leukocyte activation | Biological process | 4 | 483 | 38 | 13692 | 2.98398169336384 | 0.0438689800950048 | 0.565125246034974 | 1.35784246234136 | LGALS1//PLSCR1//BMP4//ANXA1 |
| GO:0001889 | liver development | Biological process | 2 | 121 | 38 | 13692 | 5.95563288386255 | 0.044284036212102 | 0.565125246034974 | 1.35375280277168 | ANXA1//CP |
| GO:0032102 | negative regulation of response to external stimulus | Biological process | 2 | 122 | 38 | 13692 | 5.90681622088007 | 0.0449447935129146 | 0.565125246034974 | 1.34732061048412 | ANXA1//SPP1 |
| GO:0048762 | mesenchymal cell differentiation | Biological process | 2 | 122 | 38 | 13692 | 5.90681622088007 | 0.0449447935129146 | 0.565125246034974 | 1.34732061048412 | COL1A1//BMP4 |
| GO:0061008 | hepaticobiliary system development | Biological process | 2 | 123 | 38 | 13692 | 5.85879332477535 | 0.0456092442006386 | 0.565125246034974 | 1.34094712447495 | CP//ANXA1 |
| GO:0007243 | intracellular protein kinase cascade | Biological process | 5 | 713 | 38 | 13692 | 2.52675869196132 | 0.0457571634673216 | 0.565125246034974 | 1.33954090573322 | IL22RA2//TGM2//LGALS1//C3//BMP4 |
| GO:0051239 | regulation of multicellular organismal process | Biological process | 9 | 1747 | 38 | 13692 | 1.8562347482903 | 0.0459101956655806 | 0.565125246034974 | 1.3380908563017 | BMP4//C3//COL1A1//LGALS1//MGP//PTGDS//SPP1//TIMP1//DAB2 |
| GO:0008347 | glial cell migration | Biological process | 1 | 17 | 38 | 13692 | 21.1950464396285 | 0.0461740765408206 | 0.565125246034974 | 1.33560178148028 | MMP14 |
| GO:0031294 | lymphocyte costimulation | Biological process | 1 | 17 | 38 | 13692 | 21.1950464396285 | 0.0461740765408206 | 0.565125246034974 | 1.33560178148028 | LGALS1 |
| GO:0031638 | zymogen activation | Biological process | 1 | 17 | 38 | 13692 | 21.1950464396285 | 0.0461740765408206 | 0.565125246034974 | 1.33560178148028 | MMP14 |
| GO:0048525 | negative regulation of viral reproduction | Biological process | 1 | 17 | 38 | 13692 | 21.1950464396285 | 0.0461740765408206 | 0.565125246034974 | 1.33560178148028 | PLSCR1 |
| GO:0051482 | elevation of cytosolic calcium ion concentration involved in phospholipase C-activating G-protein coupled signaling pathway | Biological process | 1 | 17 | 38 | 13692 | 21.1950464396285 | 0.0461740765408206 | 0.565125246034974 | 1.33560178148028 | TGM2 |
| GO:0060395 | SMAD protein signal transduction | Biological process | 1 | 17 | 38 | 13692 | 21.1950464396285 | 0.0461740765408206 | 0.565125246034974 | 1.33560178148028 | BMP4 |
| GO:0060572 | morphogenesis of an epithelial bud | Biological process | 1 | 17 | 38 | 13692 | 21.1950464396285 | 0.0461740765408206 | 0.565125246034974 | 1.33560178148028 | BMP4 |
| GO:0061437 | renal system vasculature development | Biological process | 1 | 17 | 38 | 13692 | 21.1950464396285 | 0.0461740765408206 | 0.565125246034974 | 1.33560178148028 | BMP4 |
| GO:0061440 | kidney vasculature development | Biological process | 1 | 17 | 38 | 13692 | 21.1950464396285 | 0.0461740765408206 | 0.565125246034974 | 1.33560178148028 | BMP4 |
| GO:0070232 | regulation of T cell apoptotic process | Biological process | 1 | 17 | 38 | 13692 | 21.1950464396285 | 0.0461740765408206 | 0.565125246034974 | 1.33560178148028 | BMP4 |
| GO:0072012 | glomerulus vasculature development | Biological process | 1 | 17 | 38 | 13692 | 21.1950464396285 | 0.0461740765408206 | 0.565125246034974 | 1.33560178148028 | BMP4 |
| GO:0072077 | renal vesicle morphogenesis | Biological process | 1 | 17 | 38 | 13692 | 21.1950464396285 | 0.0461740765408206 | 0.565125246034974 | 1.33560178148028 | BMP4 |
| GO:0030100 | regulation of endocytosis | Biological process | 2 | 124 | 38 | 13692 | 5.81154499151104 | 0.0462773638974808 | 0.565125246034974 | 1.33463138777958 | C3//ANXA1 |
| GO:0033189 | response to vitamin A | Biological process | 2 | 124 | 38 | 13692 | 5.81154499151104 | 0.0462773638974808 | 0.565125246034974 | 1.33463138777958 | BMP4//COL1A1 |
| GO:0035270 | endocrine system development | Biological process | 2 | 124 | 38 | 13692 | 5.81154499151104 | 0.0462773638974808 | 0.565125246034974 | 1.33463138777958 | BMP4//ANXA1 |
| GO:0048589 | developmental growth | Biological process | 3 | 291 | 38 | 13692 | 3.71459576776994 | 0.046412291262644 | 0.565198569153976 | 1.33336699098282 | C3//SPP1//BMP4 |
| GO:0009966 | regulation of signal transduction | Biological process | 8 | 1477 | 38 | 13692 | 1.95160888001995 | 0.0465865911122472 | 0.565749627246791 | 1.33173906708567 | BMP4//IL22RA2//TGM2//LGALS1//PLSCR1//C3//DAB2//COL1A1 |
| GO:0030308 | negative regulation of cell growth | Biological process | 2 | 125 | 38 | 13692 | 5.76505263157895 | 0.0469491283261407 | 0.567010960280443 | 1.32837246658555 | SPP1//DAB2 |
| GO:0060560 | developmental growth involved in morphogenesis | Biological process | 2 | 125 | 38 | 13692 | 5.76505263157895 | 0.0469491283261407 | 0.567010960280443 | 1.32837246658555 | SPP1//BMP4 |
| GO:0046545 | development of primary female sexual characteristics | Biological process | 2 | 127 | 38 | 13692 | 5.67426440116038 | 0.0483034947700977 | 0.567763603888092 | 1.31602144679585 | BMP4//MMP14 |
| GO:0000902 | cell morphogenesis | Biological process | 5 | 725 | 38 | 13692 | 2.48493647912886 | 0.0485726246774932 | 0.567763603888092 | 1.31360842829237 | DAB2//BMP4//ROBO3//COL1A1//SPP1 |
| GO:0001710 | mesodermal cell fate commitment | Biological process | 1 | 18 | 38 | 13692 | 20.0175438596491 | 0.0488245617394641 | 0.567763603888092 | 1.31136164635013 | BMP4 |
| GO:0001953 | negative regulation of cell-matrix adhesion | Biological process | 1 | 18 | 38 | 13692 | 20.0175438596491 | 0.0488245617394641 | 0.567763603888092 | 1.31136164635013 | MMP14 |
| GO:0010714 | positive regulation of collagen metabolic process | Biological process | 1 | 18 | 38 | 13692 | 20.0175438596491 | 0.0488245617394641 | 0.567763603888092 | 1.31136164635013 | BMP4 |
| GO:0032967 | positive regulation of collagen biosynthetic process | Biological process | 1 | 18 | 38 | 13692 | 20.0175438596491 | 0.0488245617394641 | 0.567763603888092 | 1.31136164635013 | BMP4 |
| GO:0045843 | negative regulation of striated muscle tissue development | Biological process | 1 | 18 | 38 | 13692 | 20.0175438596491 | 0.0488245617394641 | 0.567763603888092 | 1.31136164635013 | BMP4 |
| GO:0050482 | arachidonic acid secretion | Biological process | 1 | 18 | 38 | 13692 | 20.0175438596491 | 0.0488245617394641 | 0.567763603888092 | 1.31136164635013 | ANXA1 |
| GO:0060231 | mesenchymal to epithelial transition | Biological process | 1 | 18 | 38 | 13692 | 20.0175438596491 | 0.0488245617394641 | 0.567763603888092 | 1.31136164635013 | BMP4 |
| GO:0060602 | branch elongation of an epithelium | Biological process | 1 | 18 | 38 | 13692 | 20.0175438596491 | 0.0488245617394641 | 0.567763603888092 | 1.31136164635013 | BMP4 |
| GO:0060914 | heart formation | Biological process | 1 | 18 | 38 | 13692 | 20.0175438596491 | 0.0488245617394641 | 0.567763603888092 | 1.31136164635013 | BMP4 |
| GO:0070229 | negative regulation of lymphocyte apoptotic process | Biological process | 1 | 18 | 38 | 13692 | 20.0175438596491 | 0.0488245617394641 | 0.567763603888092 | 1.31136164635013 | BMP4 |
| GO:0072087 | renal vesicle development | Biological process | 1 | 18 | 38 | 13692 | 20.0175438596491 | 0.0488245617394641 | 0.567763603888092 | 1.31136164635013 | BMP4 |
| GO:2000826 | regulation of heart morphogenesis | Biological process | 1 | 18 | 38 | 13692 | 20.0175438596491 | 0.0488245617394641 | 0.567763603888092 | 1.31136164635013 | BMP4 |
| GO:0033002 | muscle cell proliferation | Biological process | 2 | 128 | 38 | 13692 | 5.62993421052632 | 0.0489860487301467 | 0.568134491092495 | 1.3099275898129 | BMP4//TGM2 |
