# Supplementary material for: Gene Expression in the Hippocampus in a Rat Model of Premenstrual Dysphoric Disorder After Treatment With Baixiangdan Capsules
Source: Front Psychol. 2018 Nov 13;9:2065. doi: 10.3389/fpsyg.2018.02065 (PMC6242977; doi:10.3389/fpsyg.2018.02065)
Supplement: Supplementary file 3 [file Data_Sheet_3.ZIP › Data Analysis Folder/GO Analysis Report/BXD vs fluoxetine (up)/CC_result(Rat).html]

| GO.ID | Term | Ontology | Count | Pop.Hits | List.Total | Pop.Total | Fold.Enrichment | Pvalue | FDR | Enrichment.Score | GENES |
| --- | --- | --- | --- | --- | --- | --- | --- | --- | --- | --- | --- |
| GO:0044421 | extracellular region part | Cellular component | 19 | 887 | 42 | 15288 | 7.79706877113867 | 3.34585791247062e-13 | 1.79672569899672e-10 | 12.4754925060336 | COL1A1//COL3A1//BMP4//LGALS1//LUM//LGALS3//TGFBI//TIMP1//RGD1562717//C3//CP//MGP//SPP1//ANXA1//PTGDS//CFD//SERPINB1A//F5//LRG1 |
| GO:0005615 | extracellular space | Cellular component | 17 | 707 | 42 | 15288 | 8.75247524752475 | 1.44135490363231e-12 | 3.87003791625275e-10 | 11.8412290700454 | C3//CP//BMP4//MGP//SPP1//ANXA1//PTGDS//COL1A1//CFD//LGALS1//LUM//COL3A1//TGFBI//SERPINB1A//F5//RGD1562717//LRG1 |
| GO:0005576 | extracellular region | Cellular component | 21 | 1410 | 42 | 15288 | 5.42127659574468 | 1.27905038708862e-11 | 2.28950019288863e-09 | 10.8931123465272 | COL1A1//COL3A1//BMP4//LGALS1//LUM//LGALS3//TGFBI//TIMP1//RGD1562717//C3//CP//MGP//SPP1//ANXA1//PTGDS//CFD//SERPINB1A//F5//LRG1//IL22RA2//FIBIN |
| GO:0031012 | extracellular matrix | Cellular component | 12 | 350 | 42 | 15288 | 12.48 | 1.02509007112391e-10 | 1.37618342048385e-08 | 9.98923797297556 | COL1A1//COL3A1//BMP4//LGALS1//LUM//LGALS3//TGFBI//TIMP1//RGD1562717//MGP//MMP14//PLSCR1 |
| GO:0005578 | proteinaceous extracellular matrix | Cellular component | 9 | 262 | 42 | 15288 | 12.5038167938931 | 3.02430585636005e-08 | 3.24810448973069e-06 | 7.51937428955563 | COL1A1//COL3A1//LUM//TGFBI//TIMP1//RGD1562717//BMP4//LGALS1//LGALS3 |
| GO:0005583 | fibrillar collagen | Cellular component | 3 | 12 | 42 | 15288 | 91 | 4.16931180087766e-06 | 0.000373153406178551 | 5.3799356250615 | COL1A1//COL3A1//LUM |
| GO:0044420 | extracellular matrix part | Cellular component | 5 | 154 | 42 | 15288 | 11.8181818181818 | 6.12017931464659e-05 | 0.0046950518456646 | 4.21323585330857 | COL1A1//COL3A1//LUM//TGFBI//TIMP1 |
| GO:0005581 | collagen | Cellular component | 3 | 55 | 42 | 15288 | 19.8545454545455 | 0.000457967388689364 | 0.0307410609657736 | 3.33916544648632 | LUM//COL1A1//COL3A1 |
| GO:0016323 | basolateral plasma membrane | Cellular component | 4 | 273 | 42 | 15288 | 5.33333333333333 | 0.0065346697803246 | 0.389901963559368 | 2.18477635394516 | GRB7//CLDN4//ANXA1//SLC16A10 |
| GO:0045177 | apical part of cell | Cellular component | 4 | 306 | 42 | 15288 | 4.75816993464052 | 0.00968855356724933 | 0.520275326561289 | 2.01374105521456 | DAB2//SLC6A20//CLDN4//SPP1 |
| GO:0031988 | membrane-bounded vesicle | Cellular component | 6 | 715 | 42 | 15288 | 3.05454545454545 | 0.0127778009647869 | 0.616404946271189 | 1.89354388100025 | DAB2//F5//MMP14//KLK7//BMP4//SPP1 |
| GO:0044459 | plasma membrane part | Cellular component | 9 | 1426 | 42 | 15288 | 2.29733520336606 | 0.0137744122071774 | 0.616404946271189 | 1.86092692464503 | CP//SLC6A20//PLSCR1//CLDN4//GRB7//ANXA1//SLC16A10//DAB2//S100A6 |
| GO:0031982 | vesicle | Cellular component | 6 | 807 | 42 | 15288 | 2.70631970260223 | 0.0219706335647877 | 0.882501208482344 | 1.65815741919583 | DAB2//F5//BMP4//SPP1//MMP14//KLK7 |
| GO:0005604 | basement membrane | Cellular component | 2 | 86 | 42 | 15288 | 8.46511627906977 | 0.0232813343231765 | 0.882501208482344 | 1.63299213258803 | TGFBI//TIMP1 |
| GO:0016324 | apical plasma membrane | Cellular component | 3 | 230 | 42 | 15288 | 4.74782608695652 | 0.0250832512087534 | 0.882501208482344 | 1.60061617236648 | DAB2//SLC6A20//CLDN4 |
| GO:0048471 | perinuclear region of cytoplasm | Cellular component | 4 | 426 | 42 | 15288 | 3.41784037558685 | 0.0289149660655483 | 0.882501208482344 | 1.5388773130458 | SPP1//PTGDS//S100A6//HHATL |
| GO:0060053 | neurofilament cytoskeleton | Cellular component | 1 | 12 | 42 | 15288 | 30.3333333333333 | 0.0324849506421736 | 0.882501208482344 | 1.48831778876772 | PRPH |
| GO:0001533 | cornified envelope | Cellular component | 1 | 14 | 42 | 15288 | 26 | 0.0377980033428192 | 0.882501208482344 | 1.42253114090612 | ANXA1 |
| GO:0005614 | interstitial matrix | Cellular component | 1 | 14 | 42 | 15288 | 26 | 0.0377980033428192 | 0.882501208482344 | 1.42253114090612 | RGD1562717 |
| GO:0005779 | integral to peroxisomal membrane | Cellular component | 1 | 14 | 42 | 15288 | 26 | 0.0377980033428192 | 0.882501208482344 | 1.42253114090612 | SLC27A2 |
| GO:0031091 | platelet alpha granule | Cellular component | 1 | 14 | 42 | 15288 | 26 | 0.0377980033428192 | 0.882501208482344 | 1.42253114090612 | F5 |
| GO:0031231 | intrinsic to peroxisomal membrane | Cellular component | 1 | 14 | 42 | 15288 | 26 | 0.0377980033428192 | 0.882501208482344 | 1.42253114090612 | SLC27A2 |
| GO:0042599 | lamellar body | Cellular component | 1 | 14 | 42 | 15288 | 26 | 0.0377980033428192 | 0.882501208482344 | 1.42253114090612 | KLK7 |
