# Supplementary material for: Gene Expression in the Hippocampus in a Rat Model of Premenstrual Dysphoric Disorder After Treatment With Baixiangdan Capsules
Source: Front Psychol. 2018 Nov 13;9:2065. doi: 10.3389/fpsyg.2018.02065 (PMC6242977; doi:10.3389/fpsyg.2018.02065)
Supplement: Supplementary file 3 [file Data_Sheet_3.ZIP › Data Analysis Folder/GO Analysis Report/BXD vs fluoxetine (up)/MF_result(Rat).html]

| GO.ID | Term | Ontology | Count | Pop.Hits | List.Total | Pop.Total | Fold.Enrichment | Pvalue | FDR | Enrichment.Score | GENES |
| --- | --- | --- | --- | --- | --- | --- | --- | --- | --- | --- | --- |
| GO:0005509 | calcium ion binding | Molecular function | 8 | 531 | 38 | 14392 | 5.70601645356329 | 5.98828176254815e-05 | 0.0316948444646773 | 4.2226977732854 | MGP//ANXA1//MMP14//S100A6//PLSCR1//CAPSL//S100A11//RGD1563581 |
| GO:0005515 | protein binding | Molecular function | 24 | 4586 | 38 | 14392 | 1.98205063465467 | 7.06685495310531e-05 | 0.0316948444646773 | 4.15077382246941 | LGALS1//CP//ANXA1//SLC27A2//FRS3//BMP4//SPP1//PLSCR1//MMP14//COL3A1//LUM//RGD1562717//S100A6//DAB2//PRRC2C//COL1A1//GRB7//TGM2//LGALS3//IL22RA2//ROBO3//MGP//C3//PRPH |
| GO:0050840 | extracellular matrix binding | Molecular function | 3 | 39 | 38 | 14392 | 29.1336032388664 | 0.000145344548736467 | 0.0434580200722036 | 3.83760125214995 | LGALS1//SPP1//TGFBI |
| GO:0048407 | platelet-derived growth factor binding | Molecular function | 2 | 11 | 38 | 14392 | 68.8612440191388 | 0.000367803282897653 | 0.0824798861897987 | 3.43438439870324 | COL1A1//COL3A1 |
| GO:0061134 | peptidase regulator activity | Molecular function | 4 | 192 | 38 | 14392 | 7.89035087719298 | 0.00158943374210547 | 0.285144413333721 | 2.79875757157918 | C3//SERPINB1A//TIMP1//MMP14 |
| GO:0005201 | extracellular matrix structural constituent | Molecular function | 2 | 29 | 38 | 14392 | 26.1197822141561 | 0.00263491272583037 | 0.39391945251164 | 2.57923376501234 | COL1A1//COL3A1 |
| GO:0048306 | calcium-dependent protein binding | Molecular function | 2 | 40 | 38 | 14392 | 18.9368421052632 | 0.00497051111168395 | 0.511638728843284 | 2.30359895099082 | MGP//S100A6 |
| GO:0042802 | identical protein binding | Molecular function | 7 | 827 | 38 | 14392 | 3.20575319798893 | 0.00532739284929153 | 0.511638728843284 | 2.27348527656062 | BMP4//ANXA1//LGALS1//S100A6//ROBO3//COL1A1//GRB7 |
| GO:0005518 | collagen binding | Molecular function | 2 | 42 | 38 | 14392 | 18.0350877192982 | 0.00546850498628074 | 0.511638728843284 | 2.26213138755179 | LUM//RGD1562717 |
| GO:0004857 | enzyme inhibitor activity | Molecular function | 4 | 274 | 38 | 14392 | 5.52900499423742 | 0.00570388772400539 | 0.511638728843284 | 2.24382903177555 | ANXA1//C3//SERPINB1A//TIMP1 |
| GO:0005319 | lipid transporter activity | Molecular function | 2 | 47 | 38 | 14392 | 16.1164613661814 | 0.00680911612238342 | 0.555252469252539 | 2.16690925931845 | SLC27A2//PLSCR1 |
| GO:0004866 | endopeptidase inhibitor activity | Molecular function | 3 | 154 | 38 | 14392 | 7.37799043062201 | 0.00770557883522533 | 0.565559563660911 | 2.1131947319615 | SERPINB1A//TIMP1//C3 |
| GO:0061135 | endopeptidase regulator activity | Molecular function | 3 | 161 | 38 | 14392 | 7.05720823798627 | 0.00870168339081426 | 0.565559563660911 | 2.06039672248248 | C3//SERPINB1A//TIMP1 |
| GO:0004252 | serine-type endopeptidase activity | Molecular function | 3 | 163 | 38 | 14392 | 6.97061672586374 | 0.00899955423101226 | 0.565559563660911 | 2.04577900165024 | CFD//KLK7//F5 |
| GO:0030414 | peptidase inhibitor activity | Molecular function | 3 | 166 | 38 | 14392 | 6.84464172479391 | 0.00945751778697175 | 0.565559563660911 | 2.02422283324681 | C3//SERPINB1A//TIMP1 |
| GO:0005178 | integrin binding | Molecular function | 2 | 60 | 38 | 14392 | 12.6245614035088 | 0.0109116644096812 | 0.607047082132017 | 1.96210899929178 | MMP14//COL3A1 |
| GO:0008236 | serine-type peptidase activity | Molecular function | 3 | 183 | 38 | 14392 | 6.20880069025022 | 0.0123091908664981 | 0.607047082132017 | 1.90977049408447 | CFD//KLK7//F5 |
| GO:0004175 | endopeptidase activity | Molecular function | 4 | 346 | 38 | 14392 | 4.37846060237298 | 0.0127298003087148 | 0.607047082132017 | 1.89517840903318 | CFD//KLK7//F5//MMP14 |
| GO:0017171 | serine hydrolase activity | Molecular function | 3 | 186 | 38 | 14392 | 6.10865874363328 | 0.01285829939856 | 0.607047082132017 | 1.89081646614424 | CFD//KLK7//F5 |
| GO:0042803 | protein homodimerization activity | Molecular function | 5 | 558 | 38 | 14392 | 3.39369930201849 | 0.0150104548626489 | 0.652727773092556 | 1.82360614710747 | BMP4//ANXA1//LGALS1//S100A6//ROBO3 |
| GO:0005488 | binding | Molecular function | 31 | 9220 | 38 | 14392 | 1.27341020664459 | 0.0152812522128692 | 0.652727773092556 | 1.81584105630635 | C3//PRPH//TGM2//SLC27A2//DAB2//MGP//ANXA1//MMP14//S100A6//PLSCR1//CAPSL//S100A11//RGD1563581//LGALS1//GRB7//CP//LGALS3//FRS3//BMP4//SPP1//COL3A1//PTGDS//TIMP1//LUM//RGD1562717//ACTG2//PRRC2C//COL1A1//TGFBI//IL22RA2//ROBO3 |
| GO:0030246 | carbohydrate binding | Molecular function | 4 | 376 | 38 | 14392 | 4.02911534154535 | 0.0168068440282 | 0.685260867877064 | 1.77451383027359 | LGALS1//RGD1562717//BMP4//LGALS3 |
| GO:0005102 | receptor binding | Molecular function | 7 | 1052 | 38 | 14392 | 2.52011206724034 | 0.0187001885374734 | 0.729307352961463 | 1.72815401483401 | FRS3//BMP4//SPP1//PLSCR1//MMP14//COL3A1//SLC27A2 |
| GO:0032403 | protein complex binding | Molecular function | 4 | 410 | 38 | 14392 | 3.69499358151476 | 0.0223278245491015 | 0.834502442522669 | 1.65115358916975 | FRS3//MMP14//COL3A1//LGALS3 |
| GO:0005523 | tropomyosin binding | Molecular function | 1 | 11 | 38 | 14392 | 34.4306220095694 | 0.0286733349336057 | 0.918744103231459 | 1.54252179226341 | S100A6 |
| GO:0070696 | transmembrane receptor protein serine/threonine kinase binding | Molecular function | 1 | 11 | 38 | 14392 | 34.4306220095694 | 0.0286733349336057 | 0.918744103231459 | 1.54252179226341 | BMP4 |
| GO:0005198 | structural molecule activity | Molecular function | 5 | 672 | 38 | 14392 | 2.81798245614035 | 0.030691609647824 | 0.918744103231459 | 1.51298033402383 | COL1A1//COL3A1//PRPH//ANXA1//CLDN4 |
| GO:0033612 | receptor serine/threonine kinase binding | Molecular function | 1 | 12 | 38 | 14392 | 31.5614035087719 | 0.0312399445763648 | 0.918744103231459 | 1.5052897452877 | BMP4 |
| GO:0055102 | lipase inhibitor activity | Molecular function | 1 | 12 | 38 | 14392 | 31.5614035087719 | 0.0312399445763648 | 0.918744103231459 | 1.5052897452877 | ANXA1 |
| GO:0070851 | growth factor receptor binding | Molecular function | 2 | 106 | 38 | 14392 | 7.14597815292949 | 0.0318031300801683 | 0.918744103231459 | 1.49753013442917 | FRS3//PLSCR1 |
| GO:0008201 | heparin binding | Molecular function | 2 | 107 | 38 | 14392 | 7.07919331037875 | 0.0323557662457727 | 0.918744103231459 | 1.49004831080483 | BMP4//RGD1562717 |
| GO:0019838 | growth factor binding | Molecular function | 2 | 108 | 38 | 14392 | 7.01364522417154 | 0.0329122101085365 | 0.918744103231459 | 1.48264295314429 | COL1A1//COL3A1 |
| GO:0015645 | fatty acid ligase activity | Molecular function | 1 | 13 | 38 | 14392 | 29.1336032388664 | 0.0337999502861072 | 0.918744103231459 | 1.4710839384941 | SLC27A2 |
| GO:0019865 | immunoglobulin binding | Molecular function | 1 | 14 | 38 | 14392 | 27.0526315789474 | 0.036353368596778 | 0.959087400920878 | 1.43945534009492 | LGALS3 |
| GO:0042056 | chemoattractant activity | Molecular function | 1 | 15 | 38 | 14392 | 25.2491228070175 | 0.0389002160020723 | 0.996956964395967 | 1.41004798715122 | BMP4 |
| GO:0016878 | acid-thiol ligase activity | Molecular function | 1 | 17 | 38 | 14392 | 22.2786377708978 | 0.0439742638706488 | 1 | 1.356801421901 | SLC27A2 |
| GO:0005125 | cytokine activity | Molecular function | 2 | 130 | 38 | 14392 | 5.82672064777328 | 0.0460720993695733 | 1 | 1.33656199777884 | BMP4//SPP1 |
| GO:0070011 | peptidase activity, acting on L-amino acid peptides | Molecular function | 4 | 517 | 38 | 14392 | 2.93026570294207 | 0.0463882067134374 | 1 | 1.3335924162183 | CFD//KLK7//F5//MMP14 |
| GO:0016755 | transferase activity, transferring amino-acyl groups | Molecular function | 1 | 19 | 38 | 14392 | 19.9335180055402 | 0.0490222250400567 | 1 | 1.3096069807076 | TGM2 |
| GO:0030234 | enzyme regulator activity | Molecular function | 5 | 766 | 38 | 14392 | 2.47217259859832 | 0.0494838201767699 | 1 | 1.30553677998777 | ANXA1//C3//SERPINB1A//TIMP1//MMP14 |
