# Supplementary material for: Gene Expression in the Hippocampus in a Rat Model of Premenstrual Dysphoric Disorder After Treatment With Baixiangdan Capsules
Source: Front Psychol. 2018 Nov 13;9:2065. doi: 10.3389/fpsyg.2018.02065 (PMC6242977; doi:10.3389/fpsyg.2018.02065)
Supplement: Supplementary file 3 [file Data_Sheet_3.ZIP › Data Analysis Folder/GO Analysis Report/BXD vs model (down)/BP_result(Rat).html]

| GO.ID | Term | Ontology | Count | Pop.Hits | List.Total | Pop.Total | Fold.Enrichment | Pvalue | FDR | Enrichment.Score | GENES |
| --- | --- | --- | --- | --- | --- | --- | --- | --- | --- | --- | --- |
| GO:0070887 | cellular response to chemical stimulus | Biological process | 38 | 1182 | 172 | 13692 | 2.55920198323693 | 4.90722207336105e-08 | 0.000215132615696148 | 7.30916428784078 | CD74//XCL1//GSTM2//UGT1A6//FMO2//GHR//PON3//ATP7A//MMP2//SPP1//WFS1//IGFBP2//PLOD2//KRT18//TLR3//IGF2//TCF7L2//BMP7//CCL6//AQP1//ANXA1//LCN2//ANXA2//TSPO//T//NTRK1//MSX1//PTGS2//SLC5A5//PLSCR1//RT1-BB//MSX2//FOXJ1//MTUS1//MGST1//LPAR1//NDRG1//RGD1560691 |
| GO:0009605 | response to external stimulus | Biological process | 34 | 1128 | 172 | 13692 | 2.39943097476497 | 1.28363744997517e-06 | 0.00281373329034557 | 5.89155762090007 | SERPING1//C6//ANXA1//XCL1//ENPP2//CXCL16//MGP//IGFBP2//MMP2//MMP14//NTRK1//BMP7//LMX1A//SEMA3C//LAMA2//CP//TSPO//IGF2//GADD45A//AQP1//PTGS2//TLR3//SPP1//ACE//LCN2//GHR//BMP6//ANXA2//SLC12A2//CCL6//T//RGD1560691//LPAR1//CD74 |
| GO:0048545 | response to steroid hormone stimulus | Biological process | 20 | 477 | 172 | 13692 | 3.33772122275852 | 2.31452742371633e-06 | 0.0033822960751908 | 5.63553766910536 | ANXA1//IGF2//GHR//IGFBP2//PTGS2//BMP7//FOXJ1//TSPO//AQP1//APOA2//MMP2//MMP14//MGP//PTGDS//BMP6//MDK//UGT1A6//RT1-BB//MSX2//SPP1 |
| GO:0051179 | localization | Biological process | 70 | 3407 | 172 | 13692 | 1.63555197575443 | 3.27854077656646e-06 | 0.00359328069111684 | 5.48431941046394 | DNAH5//TEKT2//SEMA3C//DCDC2//XCL1//APOA2//CD74//AQP1//MESP1//VAV3//KDELR3//TTR//PTGDS//SLC44A1//TSPO//P2RX6//TRPM3//SLC12A2//SLC12A7//SLC5A5//TRPV4//SLC4A2//ATP7A//GULP1//EXOC2//SCARA5//DAB2//CXCL16//ANXA2//SLC2A12//PON1//DHRS7C//ABHD5//ACE//CP//MAGT1//SLC22A8//SLC16A8//SLCO1A5//FOLR1//LCN2//PLSCR1//MDK//MMP2//MMP14//NMB//ANXA1//ENPP2//LAMA2//SPP1//GHR//TCF7L2//STIM2//S100A6//MSX1//BMP7//MSX2//MDFIC//PTGS2//TLR3//KRT18//CD14//RGD1560691//FOXJ1//WFS1//SLC16A6//CCDC40//CCL6//LPAR1//CRB3 |
| GO:0010033 | response to organic substance | Biological process | 43 | 1714 | 172 | 13692 | 1.99708284714119 | 5.20164416383426e-06 | 0.003734283951252 | 5.28385936051557 | CD74//CD14//CREB3L1//GHR//AQP1//MGP//ANXA1//MDK//MMP14//RERG//APOA2//TCF7L2//PTGS2//MGST1//GSTM2//IGF2//UGT1A6//FOXJ1//MMP2//WFS1//SLC22A8//IGFBP2//BMP7//ACE//TSPO//HADHA//PLOD2//KRT18//CXCL16//NTRK1//PLSCR1//TLR3//SPP1//PTGDS//BMP6//LCN2//MSX1//SLC5A5//RT1-BB//XCL1//MSX2//MTUS1//CTSC |
| GO:0031214 | biomineral tissue development | Biological process | 9 | 104 | 172 | 13692 | 6.88886404293381 | 6.24473781983667e-06 | 0.003734283951252 | 5.20448579040248 | PTGS2//TCF7L2//MGP//OMD//CREB3L1//BMP6//BMP7//MSX2//SPP1 |
| GO:0048513 | organ development | Biological process | 53 | 2341 | 172 | 13692 | 1.80224114123362 | 6.31265671055551e-06 | 0.003734283951252 | 5.19978782745788 | MMP2//MMP14//ADAMTS1//BMP6//BMP7//SEMA3C//ACE//WFS1//MSX1//CP//UGT1A6//CDKN1C//FOXJ1//MESP1//WNT2B//TGFBI//MSX2//RT1-BB//T//IGF2//CD9//LMX1A//ZFHX3//TCF7L2//PDLIM3//TNXA-PS1//ATP7A//TLR3//MDK//AQP1//SPTA1//PLSCR1//NTRK1//CLCF1//ANXA1//PTGS2//MGP//TSPO//OMD//CREB3L1//APOA2//SLCO1A5//SPP1//MGST1//FSCN2//PRRX2//SOSTDC1//FMOD//CD74//COL8A1//GHR//SLC12A2 |
| GO:0016064 | immunoglobulin mediated immune response | Biological process | 8 | 80 | 172 | 13692 | 7.96046511627907 | 7.07687823444078e-06 | 0.003734283951252 | 5.15015827684994 | RT1-BB//RT1-DB1//XCL1//FOXJ1//C6//SERPING1//CLCF1//CD74 |
| GO:0040011 | locomotion | Biological process | 28 | 906 | 172 | 13692 | 2.46018789465578 | 8.15430157687756e-06 | 0.003734283951252 | 5.08861323073623 | DNAH5//TEKT2//SEMA3C//DCDC2//XCL1//MESP1//ENPP2//CXCL16//NTRK1//BMP7//LMX1A//LAMA2//TSPO//AQP1//ACE//MDK//MMP2//MMP14//VAV3//SPP1//MSX2//FOXJ1//CCDC40//CCL6//RGD1560691//LPAR1//CD74//PTGS2 |
| GO:0019724 | B cell mediated immunity | Biological process | 8 | 82 | 172 | 13692 | 7.76630743051617 | 8.51798346544707e-06 | 0.003734283951252 | 5.06966320728552 | RT1-BB//RT1-DB1//XCL1//FOXJ1//C6//SERPING1//CD74//CLCF1 |
| GO:0009612 | response to mechanical stimulus | Biological process | 11 | 170 | 172 | 13692 | 5.15088919288646 | 9.81591503092939e-06 | 0.00391208831778131 | 5.00806920962887 | IGF2//GADD45A//AQP1//PTGS2//TLR3//SLC12A2//NTRK1//MGP//IGFBP2//MMP2//MMP14 |
| GO:0048870 | cell motility | Biological process | 25 | 779 | 172 | 13692 | 2.55470639161716 | 1.40456217822007e-05 | 0.00465093585827915 | 4.85245903036048 | DNAH5//TEKT2//SEMA3C//DCDC2//XCL1//MESP1//TSPO//AQP1//ACE//MDK//MMP2//MMP14//VAV3//ENPP2//LAMA2//CXCL16//SPP1//MSX2//FOXJ1//CCDC40//CCL6//RGD1560691//LPAR1//CD74//PTGS2 |
| GO:0051674 | localization of cell | Biological process | 25 | 779 | 172 | 13692 | 2.55470639161716 | 1.40456217822007e-05 | 0.00465093585827915 | 4.85245903036048 | DNAH5//TEKT2//SEMA3C//DCDC2//XCL1//MESP1//TSPO//AQP1//ACE//MDK//MMP2//MMP14//VAV3//ENPP2//LAMA2//CXCL16//SPP1//MSX2//FOXJ1//CCDC40//CCL6//RGD1560691//LPAR1//CD74//PTGS2 |
| GO:0048731 | system development | Biological process | 63 | 3069 | 172 | 13692 | 1.63411307372298 | 1.48524411532637e-05 | 0.00465093585827915 | 4.8282021596625 | LGALS3//PTGS2//ANXA2//MMP2//MMP14//TGFBI//ADAMTS1//ATP7A//TCF7L2//T//BMP6//BMP7//SEMA3C//DCDC2//ACE//WFS1//MSX1//CP//UGT1A6//CDKN1C//FOXJ1//MESP1//WNT2B//MSX2//RT1-BB//IGF2//NTRK1//LMX1A//LAMA2//CD9//ZFHX3//PDLIM3//TNXA-PS1//TSPO//TLR3//RGD1560691//TRPV4//LPAR1//MDK//AQP1//SPTA1//PLSCR1//CLCF1//ANXA1//MGP//OMD//CREB3L1//APOA2//SLCO1A5//SPP1//NDRG1//MGST1//FSCN2//PRRX2//SOSTDC1//FMOD//CD74//KCNIP2//C6//COL8A1//GHR//SLC12A2 |
| GO:0006955 | immune response | Biological process | 22 | 641 | 172 | 13692 | 2.73214091354352 | 1.74793001429907e-05 | 0.00476593500714469 | 4.75747596015253 | SERPING1//CTSC//XCL1//C6//TLR3//RT1-BB//RT1-DB1//FOXJ1//APOA2//CD74//CXCL16//ATP7A//CD14//LGALS3//LCN2//PLSCR1//CLCF1//VAV3//MR1//BMP6//ENPP2//CCL6 |
| GO:0007275 | multicellular organismal development | Biological process | 68 | 3429 | 172 | 13692 | 1.57862825286374 | 1.79818580730377e-05 | 0.00476593500714469 | 4.7451654344507 | LGALS3//PTGS2//ANXA2//MMP2//MMP14//TGFBI//ADAMTS1//ATP7A//TCF7L2//T//BMP6//BMP7//EPCAM//C6//DAB2//MSX1//SEMA3C//DCDC2//ACE//WFS1//CP//UGT1A6//CDKN1C//FOXJ1//MESP1//WNT2B//MSX2//RT1-BB//SOSTDC1//IGF2//NTRK1//LMX1A//LAMA2//CD9//ZFHX3//PDLIM3//TNXA-PS1//FBLN1//TSPO//TLR3//RGD1560691//TRPV4//LPAR1//MDK//AQP1//SPTA1//PLSCR1//CLCF1//ANXA1//MGP//PRRX2//OMD//CREB3L1//APOA2//SLCO1A5//SPP1//NDRG1//MGST1//FSCN2//FMOD//CD74//KCNIP2//COL8A1//CCDC40//GHR//SLC12A2//IGFBP2//WIPF3 |
| GO:0006811 | ion transport | Biological process | 24 | 742 | 172 | 13692 | 2.57481351469943 | 1.87046159150644e-05 | 0.00476593500714469 | 4.72805120527816 | P2RX6//TRPM3//AQP1//SLC12A2//SLC12A7//SLC5A5//TRPV4//TSPO//SLC4A2//ATP7A//DHRS7C//CP//MAGT1//SLC22A8//SLC16A8//SLCO1A5//SLC44A1//LCN2//STIM2//S100A6//SCARA5//XCL1//PTGS2//WFS1 |
| GO:0048584 | positive regulation of response to stimulus | Biological process | 28 | 950 | 172 | 13692 | 2.3462423500612 | 1.95681638067072e-05 | 0.00476593500714469 | 4.70844992477778 | SERPING1//XCL1//CD74//C6//TLR3//RT1-BB//BMP6//BMP7//CDKN1C//MSX2//MSX1//PTGS2//LPAR1//MESP1//CLCF1//GHR//S100A4//IGF2//PLSCR1//PRRX2//NTRK1//ACE//VAV3//C1QTNF3//TCF7L2//RGD1560691//WNT2B//DAB2 |
| GO:0051384 | response to glucocorticoid stimulus | Biological process | 12 | 219 | 172 | 13692 | 4.36189869385155 | 2.07372360511032e-05 | 0.00478484436042297 | 4.68324912874475 | ANXA1//UGT1A6//RT1-BB//AQP1//GHR//MGP//PTGDS//BMP6//APOA2//IGFBP2//PTGS2//MDK |
| GO:0009725 | response to hormone stimulus | Biological process | 25 | 801 | 172 | 13692 | 2.4845396742444 | 2.24655754721903e-05 | 0.00492445414350411 | 4.64848245215684 | GHR//ANXA1//IGF2//IGFBP2//PTGS2//BMP7//FOXJ1//TSPO//HADHA//UGT1A6//PLOD2//AQP1//APOA2//MMP2//MMP14//SPP1//MGP//PTGDS//BMP6//MDK//SLC5A5//MTUS1//RT1-BB//MSX2//RERG |
| GO:0006812 | cation transport | Biological process | 20 | 575 | 172 | 13692 | 2.76885743174924 | 3.59741288348732e-05 | 0.00710687684856528 | 4.4440097143278 | AQP1//SLC12A2//SLC12A7//SLC5A5//TRPV4//ATP7A//DHRS7C//CP//MAGT1//SLC22A8//SLC44A1//LCN2//STIM2//SCARA5//XCL1//PTGS2//TSPO//WFS1//P2RX6//TRPM3 |
| GO:0031960 | response to corticosteroid stimulus | Biological process | 12 | 232 | 172 | 13692 | 4.11748195669607 | 3.66196302710392e-05 | 0.00710687684856528 | 4.43628604485295 | GHR//MGP//ANXA1//PTGDS//BMP6//APOA2//IGFBP2//PTGS2//MDK//UGT1A6//RT1-BB//AQP1 |
| GO:0006928 | cellular component movement | Biological process | 27 | 932 | 172 | 13692 | 2.30614332767741 | 3.79704123436403e-05 | 0.00710687684856528 | 4.42055468656852 | DNAH5//TEKT2//SEMA3C//DCDC2//XCL1//MESP1//DNAH1//TSPO//AQP1//ACE//MDK//MMP2//MMP14//VAV3//ENPP2//LAMA2//CXCL16//SPP1//MSX2//FOXJ1//CCDC40//CCL6//RGD1560691//LPAR1//CD74//PTGS2//CD9 |
| GO:0009719 | response to endogenous stimulus | Biological process | 28 | 987 | 172 | 13692 | 2.25828797624938 | 3.89062601198829e-05 | 0.00710687684856528 | 4.40998051392407 | GHR//AQP1//MGP//ANXA1//MDK//MMP14//RERG//PTGS2//MGST1//MMP2//IGF2//IGFBP2//BMP7//FOXJ1//TSPO//HADHA//UGT1A6//PLOD2//TCF7L2//APOA2//SPP1//PTGDS//BMP6//SLC5A5//MTUS1//RT1-BB//MSX2//XCL1 |
| GO:0043627 | response to estrogen stimulus | Biological process | 12 | 237 | 172 | 13692 | 4.0306152487489 | 4.50834130521309e-05 | 0.00770711733774848 | 4.34598321299865 | IGF2//GHR//ANXA1//IGFBP2//PTGS2//BMP7//FOXJ1//MSX2//MMP2//AQP1//APOA2//MMP14 |
| GO:0002376 | immune system process | Biological process | 32 | 1218 | 172 | 13692 | 2.09141940657578 | 4.57082688826324e-05 | 0.00770711733774848 | 4.34000522651108 | ANXA1//SERPING1//CTSC//XCL1//C6//TLR3//SPTA1//RT1-BB//RT1-DB1//ACE//MR1//FOXJ1//CD74//CLCF1//APOA2//BMP6//ENPP2//PLSCR1//CCL6//NTRK1//SPP1//VAV3//CXCL16//ATP7A//IGF2//IGFBP2//CD14//LGALS3//LCN2//NDRG1//WNT2B//RGD1560691 |
| GO:0009887 | organ morphogenesis | Biological process | 24 | 792 | 172 | 13692 | 2.4122621564482 | 5.3360388016052e-05 | 0.00866414596527304 | 4.27278102105894 | FOXJ1//MESP1//BMP6//MSX1//T//MSX2//SEMA3C//TCF7L2//ATP7A//APOA2//SLCO1A5//FSCN2//PRRX2//BMP7//SOSTDC1//AQP1//FMOD//COL8A1//MMP2//ADAMTS1//GHR//WNT2B//IGF2 |
| GO:0002455 | humoral immune response mediated by circulating immunoglobulin | Biological process | 5 | 34 | 172 | 13692 | 11.7065663474692 | 6.11381525029931e-05 | 0.00941283251289144 | 4.21368768909502 | FOXJ1//C6//SERPING1//RT1-DB1//RT1-BB |
| GO:0023019 | signal transduction involved in regulation of gene expression | Biological process | 4 | 18 | 172 | 13692 | 17.6899224806202 | 6.41282538755771e-05 | 0.00941283251289144 | 4.19295058516503 | MSX2//MSX1//T//MESP1 |
| GO:0048583 | regulation of response to stimulus | Biological process | 43 | 1898 | 172 | 13692 | 1.80347734457324 | 6.44126312469761e-05 | 0.00941283251289144 | 4.19102895961289 | GHR//CD74//LPAR1//SERPING1//XCL1//C6//TLR3//FOXJ1//ANXA1//APOA2//RT1-BB//MDFIC//BMP6//BMP7//CDKN1C//MSX2//MSX1//SOSTDC1//TCF7L2//PTGS2//ACAP2//NTRK1//RASGRF2//VAV3//ARHGEF26//MESP1//CLCF1//ANXA2//S100A4//IGF2//IGFBP2//PLSCR1//PTGDS//PRRX2//TRPV4//SPP1//ACE//MDK//RGN//C1QTNF3//RGD1560691//DAB2//WNT2B |
| GO:0030282 | bone mineralization | Biological process | 7 | 81 | 172 | 13692 | 6.87941429801895 | 6.95330065129326e-05 | 0.00966481748012382 | 4.15780899190379 | MGP//OMD//CREB3L1//BMP6//BMP7//PTGS2//TCF7L2 |
| GO:0048522 | positive regulation of cellular process | Biological process | 57 | 2813 | 172 | 13692 | 1.61303416860258 | 7.05461129936045e-05 | 0.00966481748012382 | 4.15152691035661 | GHR//CD74//LPAR1//CTSC//XCL1//IGF2//DAB2//TLR3//BMP6//PRRX2//T//C6//C7//MDFIC//PTGS2//EPCAM//CLCF1//NMB//AQP1//TSPO//GADD45A//BMP7//SPP1//COL8A1//APOA2//ABHD5//TCF7L2//NTRK1//RGD1560691//MDK//CDKN1C//MESP1//CXCL16//MSX2//MSX1//BAIAP2L1//VAV3//TRPV4//ANXA1//ANXA2//LCN2//WFS1//LAMA2//SPTA1//IGFBP2//ACE//MMP2//PLSCR1//S100A4//ZFHX3//CD14//FOXJ1//MOSPD1//CREB3L1//RT1-BB//C1QTNF3//WNT2B |
| GO:0002449 | lymphocyte mediated immunity | Biological process | 9 | 142 | 172 | 13692 | 5.04536521454307 | 7.55593992653682e-05 | 0.00969589085021114 | 4.12171150359262 | CTSC//XCL1//RT1-BB//RT1-DB1//FOXJ1//C6//SERPING1//CD74//CLCF1 |
| GO:0006082 | organic acid metabolic process | Biological process | 22 | 707 | 172 | 13692 | 2.47708956942206 | 7.63865382781641e-05 | 0.00969589085021114 | 4.11698317109232 | PTGDS//CD74//PTGS2//GHR//GSTM2//MGST1//HDC//ATP7A//SLC5A5//ABHD5//HADHA//BDH2//PLSCR1//ELOVL7//UGT1A6//RGN//ANXA1//TTR//PON1//PON3//FOLR1//FMO2 |
| GO:0002252 | immune effector process | Biological process | 14 | 332 | 172 | 13692 | 3.35682263939479 | 7.74078877183827e-05 | 0.00969589085021114 | 4.11121478327019 | SERPING1//CTSC//XCL1//C6//TLR3//RT1-BB//RT1-DB1//ACE//CLCF1//APOA2//FOXJ1//CD74//ATP7A//PLSCR1 |
| GO:0065008 | regulation of biological quality | Biological process | 46 | 2106 | 172 | 13692 | 1.73875306433446 | 8.11148296613386e-05 | 0.00987798370098079 | 4.09089973948402 | ANXA1//SPTA1//CD74//ACE//WFS1//AQP1//SERPING1//F5//SLC5A5//KDELR3//RGN//TRPV4//STIM2//ATP7A//CP//SCARA5//C7//SLC12A7//NMB//ANXA2//PTGS2//PLSCR1//DHRS7C//ABHD5//LCN2//MSX1//LAMA2//BAIAP2L1//CD9//TCF7L2//APOA2//NDRG1//BMP6//TTR//LPAR1//C1QTNF3//SPP1//SLC12A2//MMP2//XCL1//TSPO//TLR3//BDH2//TMEM123//SLCO1A5//IGF2 |
| GO:0048518 | positive regulation of biological process | Biological process | 61 | 3107 | 172 | 13692 | 1.56288500834575 | 8.83909272390325e-05 | 0.0101507482686819 | 4.05359231023848 | GHR//CD74//LPAR1//SERPING1//CTSC//XCL1//IGF2//DAB2//TLR3//BMP6//C6//PRRX2//RT1-BB//CLCF1//FOXJ1//T//C7//MDFIC//PTGS2//EPCAM//NMB//AQP1//PLSCR1//LCN2//TSPO//GADD45A//BMP7//SPP1//COL8A1//APOA2//PON1//ABHD5//TCF7L2//NTRK1//RGD1560691//MDK//CDKN1C//MESP1//CXCL16//MSX2//MSX1//BAIAP2L1//VAV3//TRPV4//ANXA1//ANXA2//WFS1//LAMA2//STIM2//CD14//SPTA1//IGFBP2//ACE//MMP2//S100A4//ZFHX3//MOSPD1//CREB3L1//C1QTNF3//WNT2B//NDRG1 |
| GO:0050670 | regulation of lymphocyte proliferation | Biological process | 9 | 145 | 172 | 13692 | 4.94097834803529 | 8.88063036709644e-05 | 0.0101507482686819 | 4.05155620592916 | CD74//VAV3//CLCF1//SPTA1//IGF2//IGFBP2//XCL1//FOXJ1//RT1-BB |
| GO:0032944 | regulation of mononuclear cell proliferation | Biological process | 9 | 146 | 172 | 13692 | 4.90713603058299 | 9.36337045098419e-05 | 0.0101507482686819 | 4.02856779391335 | CD74//VAV3//CLCF1//SPTA1//IGF2//IGFBP2//XCL1//FOXJ1//RT1-BB |
| GO:0032502 | developmental process | Biological process | 72 | 3887 | 172 | 13692 | 1.47453946069486 | 9.58618746725957e-05 | 0.0101507482686819 | 4.01835408220838 | MSX1//DAB2//LGALS3//PTGS2//ANXA2//MMP2//MMP14//TGFBI//ADAMTS1//ATP7A//TCF7L2//T//IGF2//SPP1//MSX2//BMP6//BMP7//EPCAM//C6//SEMA3C//DCDC2//ACE//WFS1//CP//UGT1A6//CDKN1C//FOXJ1//MESP1//WNT2B//RT1-BB//IGFBP2//WIPF3//SOSTDC1//NTRK1//LMX1A//LAMA2//CD9//ZFHX3//PDLIM3//TNXA-PS1//FBLN1//TSPO//GSTM2//CTSC//SPTA1//TLR3//RGD1560691//TRPV4//LPAR1//MDK//AQP1//PLSCR1//MGP//ABHD5//CLCF1//ANXA1//PRRX2//OMD//CREB3L1//APOA2//SLCO1A5//NDRG1//MGST1//FSCN2//FMOD//CD74//KCNIP2//GHR//COL8A1//CCDC40//SLC12A2//RGD1305645 |
| GO:0071310 | cellular response to organic substance | Biological process | 25 | 877 | 172 | 13692 | 2.26923178913314 | 9.84665812223987e-05 | 0.0101507482686819 | 4.00671114060213 | CD74//GHR//MMP2//WFS1//IGFBP2//UGT1A6//PLOD2//KRT18//TLR3//IGF2//TCF7L2//BMP7//NTRK1//MSX1//PTGS2//AQP1//SLC5A5//PLSCR1//RT1-BB//LCN2//XCL1//MSX2//FOXJ1//MTUS1//ANXA1 |
| GO:0001516 | prostaglandin biosynthetic process | Biological process | 4 | 20 | 172 | 13692 | 15.9209302325581 | 9.9562540044097e-05 | 0.0101507482686819 | 4.00190403218133 | PTGS2//ANXA1//PTGDS//CD74 |
| GO:0046457 | prostanoid biosynthetic process | Biological process | 4 | 20 | 172 | 13692 | 15.9209302325581 | 9.9562540044097e-05 | 0.0101507482686819 | 4.00190403218133 | PTGDS//CD74//PTGS2//ANXA1 |
| GO:0042180 | cellular ketone metabolic process | Biological process | 22 | 723 | 172 | 13692 | 2.42227154298948 | 0.00010582933092211 | 0.0104971583383 | 3.9753939495765 | PTGDS//CD74//PTGS2//GHR//GSTM2//MGST1//HDC//ATP7A//SLC5A5//ABHD5//HADHA//BDH2//PLSCR1//ELOVL7//UGT1A6//RGN//ANXA1//TTR//C1QTNF3//PON1//PON3//FOLR1 |
| GO:0061448 | connective tissue development | Biological process | 10 | 184 | 172 | 13692 | 4.32633973710819 | 0.000109949349989444 | 0.0104971583383 | 3.95880733380507 | WNT2B//TGFBI//MSX2//ATP7A//MGP//BMP6//MSX1//PRRX2//GHR//BMP7 |
| GO:0016477 | cell migration | Biological process | 22 | 725 | 172 | 13692 | 2.41558941459503 | 0.00011014354095844 | 0.0104971583383 | 3.9580409656794 | SEMA3C//DCDC2//XCL1//MESP1//TSPO//AQP1//ACE//ENPP2//LAMA2//CXCL16//SPP1//MSX2//MMP14//FOXJ1//CCL6//RGD1560691//LPAR1//CD74//PTGS2//MDK//MMP2//VAV3 |
| GO:0002443 | leukocyte mediated immunity | Biological process | 10 | 185 | 172 | 13692 | 4.30295411690761 | 0.000114989671873692 | 0.0105221656275218 | 3.93934116529591 | CTSC//XCL1//RT1-BB//RT1-DB1//ACE//FOXJ1//C6//SERPING1//CD74//CLCF1 |
| GO:0051216 | cartilage development | Biological process | 9 | 150 | 172 | 13692 | 4.77627906976744 | 0.000115206193002063 | 0.0105221656275218 | 3.93852417443424 | WNT2B//TGFBI//MSX2//GHR//MSX1//ATP7A//MGP//BMP6//PRRX2 |
| GO:0032355 | response to estradiol stimulus | Biological process | 9 | 151 | 172 | 13692 | 4.74464808255044 | 0.000121205506312589 | 0.0106272987934878 | 3.91647764991517 | MSX2//MMP2//IGF2//GHR//ANXA1//IGFBP2//PTGS2//BMP7//FOXJ1 |
| GO:0070663 | regulation of leukocyte proliferation | Biological process | 9 | 151 | 172 | 13692 | 4.74464808255044 | 0.000121205506312589 | 0.0106272987934878 | 3.91647764991517 | CD74//VAV3//CLCF1//SPTA1//IGF2//IGFBP2//XCL1//FOXJ1//RT1-BB |
| GO:0002460 | adaptive immune response based on somatic recombination of immune receptors built from immunoglobulin superfamily domains | Biological process | 9 | 153 | 172 | 13692 | 4.68262653898769 | 0.000133991045125047 | 0.0115179753299648 | 3.87292422538692 | CTSC//XCL1//RT1-BB//RT1-DB1//FOXJ1//C6//SERPING1//CD74//CLCF1 |
| GO:0009611 | response to wounding | Biological process | 21 | 688 | 172 | 13692 | 2.42979313142239 | 0.000147153612489316 | 0.0124061814837146 | 3.83222907189316 | SERPING1//C6//APOA2//ANXA1//PLSCR1//F5//SPP1//PTGS2//CD14//TSPO//MMP2//MSX2//IGF2//AQP1//FMOD//CDH3//ANXA2//NTRK1//XCL1//ACE//MDK |
| GO:0042592 | homeostatic process | Biological process | 29 | 1125 | 172 | 13692 | 2.05203100775194 | 0.000153142168934792 | 0.0124802133718431 | 3.8149052063361 | ANXA1//SPTA1//CD74//WFS1//AQP1//RGN//TRPV4//STIM2//ATP7A//CP//SCARA5//C7//SLC12A7//NMB//DHRS7C//LCN2//LAMA2//CD9//PTGS2//TCF7L2//NDRG1//LPAR1//C1QTNF3//APOA2//SPP1//SLC12A2//XCL1//TSPO//BDH2 |
| GO:0006935 | chemotaxis | Biological process | 13 | 312 | 172 | 13692 | 3.31686046511628 | 0.000157874775825178 | 0.0124802133718431 | 3.80168725311409 | XCL1//NTRK1//BMP7//LMX1A//SEMA3C//LAMA2//SPP1//CCL6//RGD1560691//LPAR1//CD74//ENPP2//CXCL16 |
| GO:0019752 | carboxylic acid metabolic process | Biological process | 21 | 692 | 172 | 13692 | 2.41574808441995 | 0.000159418783946901 | 0.0124802133718431 | 3.79746050800977 | PTGDS//CD74//PTGS2//GHR//GSTM2//MGST1//HDC//ATP7A//SLC5A5//ABHD5//HADHA//BDH2//PLSCR1//ELOVL7//UGT1A6//RGN//ANXA1//TTR//PON1//PON3//FOLR1 |
| GO:0043436 | oxoacid metabolic process | Biological process | 21 | 692 | 172 | 13692 | 2.41574808441995 | 0.000159418783946901 | 0.0124802133718431 | 3.79746050800977 | PTGDS//CD74//PTGS2//GHR//GSTM2//MGST1//HDC//ATP7A//SLC5A5//ABHD5//HADHA//BDH2//PLSCR1//ELOVL7//UGT1A6//RGN//ANXA1//TTR//PON1//PON3//FOLR1 |
| GO:0042330 | taxis | Biological process | 13 | 313 | 172 | 13692 | 3.30626346682517 | 0.00016293978555551 | 0.0125320705241291 | 3.78797285959847 | XCL1//ENPP2//CXCL16//NTRK1//BMP7//LMX1A//SEMA3C//LAMA2//SPP1//CCL6//RGD1560691//LPAR1//CD74 |
| GO:0019725 | cellular homeostasis | Biological process | 21 | 696 | 172 | 13692 | 2.40186447473937 | 0.000172580830055041 | 0.013044730326919 | 3.76300744653322 | RGN//TRPV4//STIM2//ATP7A//CP//SCARA5//C7//AQP1//SLC12A7//NMB//DHRS7C//LCN2//LAMA2//CD9//TCF7L2//NDRG1//WFS1//LPAR1//SLC12A2//XCL1//TSPO |
| GO:0071383 | cellular response to steroid hormone stimulus | Biological process | 7 | 94 | 172 | 13692 | 5.92800593765463 | 0.000178558375072982 | 0.0132677951918636 | 3.74821977491573 | BMP7//ANXA1//UGT1A6//RT1-BB//MSX2//MMP2//AQP1 |
| GO:0065009 | regulation of molecular function | Biological process | 35 | 1493 | 172 | 13692 | 1.86615056309288 | 0.000190677703177383 | 0.0134878467060556 | 3.71970008804034 | GHR//CD74//LPAR1//GADD45A//C6//MDFIC//SERPINB1A//SERPING1//PCOLCE//MMP14//FOXJ1//DAB2//PLSCR1//SPTA1//TCF7L2//STIM2//ACAP2//NTRK1//PON1//RGN//MSX2//RGD1560691//CDKN1C//IGF2//ATP7A//VAV3//AQP1//MSX1//BMP7//WFS1//XCL1//ABHD5//TLR3//ANXA2//APOA2 |
| GO:0030509 | BMP signaling pathway | Biological process | 7 | 95 | 172 | 13692 | 5.865605875153 | 0.000190749656883085 | 0.0134878467060556 | 3.71953623457317 | MSX2//MSX1//SOSTDC1//TCF7L2//BMP6//BMP7//T |
| GO:0050671 | positive regulation of lymphocyte proliferation | Biological process | 7 | 95 | 172 | 13692 | 5.865605875153 | 0.000190749656883085 | 0.0134878467060556 | 3.71953623457317 | CD74//VAV3//CLCF1//SPTA1//IGF2//IGFBP2//XCL1 |
| GO:0032946 | positive regulation of mononuclear cell proliferation | Biological process | 7 | 96 | 172 | 13692 | 5.80450581395349 | 0.000203604907703419 | 0.0141683161170125 | 3.69121175795205 | CD74//VAV3//CLCF1//SPTA1//IGF2//IGFBP2//XCL1 |
| GO:0042493 | response to drug | Biological process | 17 | 505 | 172 | 13692 | 2.67976053419295 | 0.000210646964454209 | 0.0142760584806849 | 3.67644479492918 | TLR3//TSPO//IGF2//AQP1//COX8B//ANXA1//APOA2//IGFBP2//PTGS2//NTRK1//MDK//MMP2//CDH3//LCN2//HADHA//MGST1//VAV3 |
| GO:0001503 | ossification | Biological process | 12 | 279 | 172 | 13692 | 3.423855963991 | 0.0002116660130576 | 0.0142760584806849 | 3.67434887049501 | IGF2//SPP1//MSX2//BMP6//MMP2//PTGS2//TCF7L2//MGP//OMD//CREB3L1//BMP7//MMP14 |
| GO:0007178 | transmembrane receptor protein serine/threonine kinase signaling pathway | Biological process | 10 | 200 | 172 | 13692 | 3.98023255813954 | 0.00021749850880042 | 0.0144471736754703 | 3.66254371628162 | WFIKKN2//SMURF2//BMP6//BMP7//MSX2//MSX1//T//CDKN1C//SOSTDC1//TCF7L2 |
| GO:0002250 | adaptive immune response | Biological process | 9 | 164 | 172 | 13692 | 4.36854792966534 | 0.000226217801781923 | 0.0148020722837605 | 3.64547322208319 | CTSC//XCL1//RT1-BB//RT1-DB1//FOXJ1//C6//SERPING1//CD74//CLCF1 |
| GO:0032870 | cellular response to hormone stimulus | Biological process | 13 | 326 | 172 | 13692 | 3.17441860465116 | 0.000242665535230034 | 0.0155735804253847 | 3.61499190031527 | GHR//IGF2//BMP7//SLC5A5//MTUS1//ANXA1//UGT1A6//RT1-BB//MSX2//MMP2//AQP1//IGFBP2//PLOD2 |
| GO:0042127 | regulation of cell proliferation | Biological process | 27 | 1042 | 172 | 13692 | 2.06269249654064 | 0.000245113378045517 | 0.0155735804253847 | 3.61063298473817 | BMP6//PRRX2//PTGS2//EPCAM//T//CLCF1//NMB//CD9//MSX2//NTRK1//MSX1//BMP7//RERG//CD74//VAV3//SPTA1//IGF2//IGFBP2//XCL1//FOXJ1//RT1-BB//AQP1//TCF7L2//OGN//CDKN1C//TSPO//ANXA1 |
| GO:0070665 | positive regulation of leukocyte proliferation | Biological process | 7 | 100 | 172 | 13692 | 5.57232558139535 | 0.00026220806188303 | 0.0164217163327886 | 3.58135395956627 | CD74//VAV3//CLCF1//SPTA1//IGF2//IGFBP2//XCL1 |
| GO:0006873 | cellular ion homeostasis | Biological process | 18 | 568 | 172 | 13692 | 2.52268260727154 | 0.000286308933707831 | 0.0176785685264103 | 3.54316510046083 | RGN//TRPV4//STIM2//ATP7A//CP//SCARA5//C7//NMB//DHRS7C//LCN2//LAMA2//CD9//TCF7L2//NDRG1//WFS1//LPAR1//XCL1//TSPO |
| GO:0001501 | skeletal system development | Biological process | 13 | 333 | 172 | 13692 | 3.10768908443327 | 0.000298021592946696 | 0.018146203659421 | 3.52575226827991 | BMP6//WNT2B//TGFBI//MSX2//PRRX2//MMP2//T//ATP7A//MGP//MSX1//BMP7//GHR//LGALS3 |
| GO:0030001 | metal ion transport | Biological process | 16 | 474 | 172 | 13692 | 2.68707683249926 | 0.000315394322741348 | 0.0186866970792183 | 3.50114612845891 | AQP1//SLC12A2//SLC12A7//SLC5A5//TRPV4//ATP7A//DHRS7C//CP//MAGT1//LCN2//STIM2//SCARA5//XCL1//PTGS2//TSPO//WFS1 |
| GO:0043068 | positive regulation of programmed cell death | Biological process | 17 | 524 | 172 | 13692 | 2.58259364459435 | 0.000324164626228624 | 0.0186866970792183 | 3.48923437835823 | CTSC//XCL1//C6//C7//TSPO//ACE//ANXA1//MSX2//PTGS2//MMP2//MSX1//BMP7//LPAR1//PLSCR1//TLR3//TCF7L2//NTRK1 |
| GO:0050801 | ion homeostasis | Biological process | 19 | 626 | 172 | 13692 | 2.41611561037224 | 0.000332427802582348 | 0.0186866970792183 | 3.47830266117205 | RGN//TRPV4//STIM2//ATP7A//CP//SCARA5//C7//NMB//DHRS7C//LCN2//LAMA2//CD9//TCF7L2//NDRG1//WFS1//LPAR1//XCL1//TSPO//BDH2 |
| GO:0030003 | cellular cation homeostasis | Biological process | 13 | 337 | 172 | 13692 | 3.07080256711062 | 0.000334252781149091 | 0.0186866970792183 | 3.47592497050523 | RGN//TRPV4//STIM2//ATP7A//CP//SCARA5//C7//NMB//DHRS7C//LCN2//WFS1//XCL1//LPAR1 |
| GO:0006692 | prostanoid metabolic process | Biological process | 4 | 27 | 172 | 13692 | 11.7932816537468 | 0.000336735645360002 | 0.0186866970792183 | 3.47271090866753 | PTGDS//CD74//PTGS2//ANXA1 |
| GO:0006693 | prostaglandin metabolic process | Biological process | 4 | 27 | 172 | 13692 | 11.7932816537468 | 0.000336735645360002 | 0.0186866970792183 | 3.47271090866753 | PTGDS//CD74//PTGS2//ANXA1 |
| GO:0046503 | glycerolipid catabolic process | Biological process | 4 | 27 | 172 | 13692 | 11.7932816537468 | 0.000336735645360002 | 0.0186866970792183 | 3.47271090866753 | ABHD5//ENPP2//APOA2//CES1D |
| GO:0010647 | positive regulation of cell communication | Biological process | 21 | 733 | 172 | 13692 | 2.28062438529141 | 0.000347821592098833 | 0.0189362697586018 | 3.4586434613261 | CD74//BMP6//BMP7//CDKN1C//MSX2//MSX1//LAMA2//TLR3//LPAR1//MESP1//CLCF1//GHR//S100A4//IGF2//PRRX2//NTRK1//C1QTNF3//TCF7L2//PTGS2//WNT2B//DAB2 |
| GO:0022008 | neurogenesis | Biological process | 26 | 1009 | 172 | 13692 | 2.05125959388757 | 0.000352463298942618 | 0.0189362697586018 | 3.45288609823717 | DCDC2//NTRK1//BMP7//LMX1A//SEMA3C//LAMA2//TSPO//RGD1560691//TRPV4//LPAR1//CD9//TCF7L2//MMP2//ATP7A//NDRG1//ANXA1//FSCN2//CDKN1C//MMP14//KCNIP2//ZFHX3//BMP6//SPP1//CLCF1//ACE//MESP1 |
| GO:0023056 | positive regulation of signaling | Biological process | 21 | 734 | 172 | 13692 | 2.27751726760028 | 0.00035419117705414 | 0.0189362697586018 | 3.45076226134026 | CD74//BMP6//BMP7//CDKN1C//MSX2//MSX1//LAMA2//TLR3//LPAR1//MESP1//CLCF1//GHR//S100A4//IGF2//PRRX2//NTRK1//C1QTNF3//TCF7L2//PTGS2//WNT2B//DAB2 |
| GO:0071470 | cellular response to osmotic stress | Biological process | 3 | 12 | 172 | 13692 | 19.9011627906977 | 0.000394288285241547 | 0.0208260221987824 | 3.40418612612366 | TRPV4//AQP1//TSPO |
| GO:0022414 | reproductive process | Biological process | 26 | 1018 | 172 | 13692 | 2.0331246858866 | 0.000403889900510573 | 0.0208936362640948 | 3.39373700646697 | MMP2//MMP14//ADAMTS1//MNS1//MEI4//ACE//FOXJ1//WIPF3//T//CD9//IGF2//IGFBP2//PTGS2//SOSTDC1//FBLN1//ATP7A//WNT2B//TLR3//TCF7L2//BMP6//MGST1//PLSCR1//MDFIC//NTRK1//ANXA1//BMP7 |
| GO:0055080 | cation homeostasis | Biological process | 14 | 390 | 172 | 13692 | 2.85760286225403 | 0.000409644889394135 | 0.0208936362640948 | 3.38759245889752 | RGN//TRPV4//STIM2//ATP7A//CP//SCARA5//C7//NMB//DHRS7C//LCN2//WFS1//XCL1//LPAR1//BDH2 |
| GO:0006875 | cellular metal ion homeostasis | Biological process | 12 | 300 | 172 | 13692 | 3.18418604651163 | 0.000409866039852225 | 0.0208936362640948 | 3.3873580644007 | RGN//TRPV4//STIM2//CP//SCARA5//C7//NMB//DHRS7C//LCN2//WFS1//XCL1//LPAR1 |
| GO:2000026 | regulation of multicellular organismal development | Biological process | 27 | 1078 | 172 | 13692 | 1.99380851706433 | 0.000420071059555105 | 0.0211677186791906 | 3.37667723778709 | FOXJ1//NTRK1//RGD1560691//TRPV4//LPAR1//MGP//OMD//CREB3L1//BMP6//BMP7//TCF7L2//MESP1//MSX1//CD74//MSX2//ZFHX3//LMX1A//C6//AQP1//LAMA2//CLCF1//SPP1//ACE//TSPO//WNT2B//PTGS2//DAB2 |
| GO:0042476 | odontogenesis | Biological process | 7 | 109 | 172 | 13692 | 5.11222530403243 | 0.000444152716215824 | 0.0221268807714792 | 3.3524676776191 | MSX1//BMP7//SOSTDC1//TCF7L2//MSX2//AQP1//FMOD |
| GO:0031667 | response to nutrient levels | Biological process | 16 | 490 | 172 | 13692 | 2.59933554817276 | 0.000454591748936608 | 0.0223924744644729 | 3.34237845119754 | CP//MGP//IGFBP2//TSPO//LCN2//GHR//BMP6//MMP2//SPP1//PTGS2//BMP7//AQP1//T//ACE//IGF2//NTRK1 |
| GO:0000003 | reproduction | Biological process | 26 | 1027 | 172 | 13692 | 2.01530762437445 | 0.000461843361097856 | 0.0224969032783667 | 3.33550529488899 | MMP2//MMP14//ADAMTS1//MNS1//MEI4//ACE//FOXJ1//WIPF3//T//CD9//IGF2//IGFBP2//PTGS2//SOSTDC1//FBLN1//ATP7A//WNT2B//TLR3//TCF7L2//BMP6//MGST1//PLSCR1//MDFIC//NTRK1//ANXA1//BMP7 |
| GO:0001568 | blood vessel development | Biological process | 15 | 443 | 172 | 13692 | 2.69541708226154 | 0.000471646413484838 | 0.0227219546892036 | 3.3263834636946 | PTGS2//ANXA2//MMP2//MMP14//TGFBI//T//SEMA3C//C6//AQP1//NTRK1//PRRX2//MESP1//BMP7//ATP7A//TCF7L2 |
| GO:0009653 | anatomical structure morphogenesis | Biological process | 39 | 1819 | 172 | 13692 | 1.70675172916374 | 0.000484425589748968 | 0.0228480681595986 | 3.31477292334643 | MSX1//DAB2//PTGS2//ANXA2//MMP2//MMP14//TGFBI//T//BMP7//SEMA3C//FOXJ1//MESP1//BMP6//MSX2//IGF2//NTRK1//LMX1A//LAMA2//SPTA1//TSPO//ATP7A//PRRX2//TCF7L2//CD9//APOA2//SLCO1A5//FSCN2//SOSTDC1//AQP1//FMOD//C6//COL8A1//SPP1//MGP//DCDC2//ADAMTS1//GHR//SLC12A2//WNT2B |
| GO:0010942 | positive regulation of cell death | Biological process | 17 | 543 | 172 | 13692 | 2.49222664782218 | 0.0004874738654771 | 0.0228480681595986 | 3.31204866280304 | CTSC//XCL1//C6//C7//TSPO//ACE//ANXA1//MSX2//PTGS2//MMP2//MSX1//BMP7//LPAR1//PLSCR1//TLR3//TCF7L2//NTRK1 |
| GO:0048878 | chemical homeostasis | Biological process | 22 | 807 | 172 | 13692 | 2.17013918907236 | 0.000493158081862742 | 0.0228480681595986 | 3.30701384527456 | WFS1//AQP1//RGN//TRPV4//STIM2//ATP7A//CP//SCARA5//C7//NMB//DHRS7C//LCN2//LAMA2//CD9//TCF7L2//NDRG1//LPAR1//C1QTNF3//APOA2//XCL1//TSPO//BDH2 |
| GO:0046651 | lymphocyte proliferation | Biological process | 9 | 183 | 172 | 13692 | 3.91498284407167 | 0.000507215851128865 | 0.0228480681595986 | 3.29480718266925 | CD74//VAV3//CLCF1//SPTA1//IGF2//IGFBP2//XCL1//FOXJ1//RT1-BB |
| GO:0019886 | antigen processing and presentation of exogenous peptide antigen via MHC class II | Biological process | 3 | 13 | 172 | 13692 | 18.3703041144902 | 0.000507853083102211 | 0.0228480681595986 | 3.29426190666534 | CD74//RT1-DB1//RT1-BB |
| GO:0072358 | cardiovascular system development | Biological process | 20 | 701 | 172 | 13692 | 2.27117407026507 | 0.000510746049188108 | 0.0228480681595986 | 3.29179498411292 | PTGS2//ANXA2//MMP2//MMP14//TGFBI//ATP7A//TCF7L2//T//FOXJ1//MESP1//MSX1//MSX2//SEMA3C//PDLIM3//C6//AQP1//NTRK1//PRRX2//ADAMTS1//BMP7 |
| GO:0072359 | circulatory system development | Biological process | 20 | 701 | 172 | 13692 | 2.27117407026507 | 0.000510746049188108 | 0.0228480681595986 | 3.29179498411292 | PTGS2//ANXA2//MMP2//MMP14//TGFBI//ATP7A//TCF7L2//T//FOXJ1//MESP1//MSX1//MSX2//SEMA3C//PDLIM3//C6//AQP1//NTRK1//PRRX2//ADAMTS1//BMP7 |
| GO:0032943 | mononuclear cell proliferation | Biological process | 9 | 184 | 172 | 13692 | 3.89370576339737 | 0.000527646699758892 | 0.0233656881994241 | 3.27765677387235 | CD74//VAV3//CLCF1//SPTA1//IGF2//IGFBP2//XCL1//FOXJ1//RT1-BB |
| GO:0002684 | positive regulation of immune system process | Biological process | 14 | 403 | 172 | 13692 | 2.76542212476196 | 0.000567348113882156 | 0.0248725413125937 | 3.24615038458711 | SERPING1//XCL1//C6//TLR3//RT1-BB//CD74//CLCF1//FOXJ1//VAV3//SPTA1//IGF2//IGFBP2//PLSCR1//RGD1560691 |
| GO:0048002 | antigen processing and presentation of peptide antigen | Biological process | 4 | 31 | 172 | 13692 | 10.271567891973 | 0.000580566711954401 | 0.0252000442099811 | 3.23614786900205 | MR1//CD74//RT1-DB1//RT1-BB |
| GO:0008283 | cell proliferation | Biological process | 30 | 1280 | 172 | 13692 | 1.86573401162791 | 0.000595017725183089 | 0.0255740951686536 | 3.2254700967344 | MMP14//BMP6//PRRX2//PTGS2//EPCAM//T//CLCF1//NMB//CD9//MSX2//NTRK1//MSX1//BMP7//RERG//CD74//VAV3//SPTA1//IGF2//IGFBP2//XCL1//ANXA1//FOXJ1//RT1-BB//AQP1//TCF7L2//OGN//COL8A1//CDKN1C//TSPO//WNT2B |
| GO:0061311 | cell surface receptor signaling pathway involved in heart development | Biological process | 3 | 14 | 172 | 13692 | 17.0581395348837 | 0.000640407864806538 | 0.0272577483428336 | 3.19354334317824 | MESP1//MSX2//MSX1 |
| GO:0048856 | anatomical structure development | Biological process | 64 | 3546 | 172 | 13692 | 1.43674497304529 | 0.000664927482731177 | 0.0280292508105142 | 3.17722571645178 | MSX1//DAB2//LGALS3//PTGS2//ANXA2//MMP2//MMP14//TGFBI//ADAMTS1//ATP7A//TCF7L2//T//BMP6//BMP7//EPCAM//C6//SEMA3C//DCDC2//ACE//WFS1//CP//UGT1A6//CDKN1C//FOXJ1//MESP1//WNT2B//MSX2//RT1-BB//IGF2//NTRK1//LMX1A//LAMA2//CD9//ZFHX3//PDLIM3//TNXA-PS1//TSPO//SPTA1//TLR3//RGD1560691//TRPV4//LPAR1//MDK//AQP1//PLSCR1//CLCF1//ANXA1//MGP//PRRX2//OMD//CREB3L1//APOA2//SLCO1A5//SPP1//NDRG1//MGST1//FSCN2//SOSTDC1//FMOD//CD74//KCNIP2//COL8A1//GHR//SLC12A2 |
| GO:0070661 | leukocyte proliferation | Biological process | 9 | 191 | 172 | 13692 | 3.75100450505296 | 0.000690399801792702 | 0.0288258355338972 | 3.1608993419747 | CD74//VAV3//CLCF1//SPTA1//IGF2//IGFBP2//XCL1//FOXJ1//RT1-BB |
| GO:0044092 | negative regulation of molecular function | Biological process | 17 | 562 | 172 | 13692 | 2.40796987503104 | 0.000717489179524811 | 0.0296742694626111 | 3.1441846441523 | GADD45A//SERPINB1A//SERPING1//FOXJ1//DAB2//PLSCR1//STIM2//MSX2//CDKN1C//AQP1//MSX1//BMP7//WFS1//XCL1//TCF7L2//ATP7A//APOA2 |
| GO:0030500 | regulation of bone mineralization | Biological process | 5 | 57 | 172 | 13692 | 6.9828641370869 | 0.000728961163041265 | 0.0298669695212421 | 3.13729560902931 | BMP6//BMP7//MGP//OMD//CREB3L1 |
| GO:0055065 | metal ion homeostasis | Biological process | 12 | 321 | 172 | 13692 | 2.97587480982395 | 0.000746676989482915 | 0.0303095548323435 | 3.12686723221879 | RGN//TRPV4//STIM2//CP//SCARA5//C7//NMB//DHRS7C//LCN2//WFS1//XCL1//LPAR1 |
| GO:0051251 | positive regulation of lymphocyte activation | Biological process | 8 | 155 | 172 | 13692 | 4.1086271567892 | 0.000757571440631643 | 0.0304696623461387 | 3.12057640598191 | CD74//VAV3//CLCF1//SPTA1//IGF2//IGFBP2//XCL1//RT1-BB |
| GO:0001944 | vasculature development | Biological process | 15 | 465 | 172 | 13692 | 2.56789197299325 | 0.000777172003801152 | 0.0306993344961882 | 3.10948285245422 | PTGS2//ANXA2//MMP2//MMP14//TGFBI//ATP7A//TCF7L2//T//SEMA3C//C6//AQP1//NTRK1//PRRX2//MESP1//BMP7 |
| GO:0009991 | response to extracellular stimulus | Biological process | 16 | 515 | 172 | 13692 | 2.47315421088282 | 0.000777286981997466 | 0.0306993344961882 | 3.10941860579768 | CP//MGP//IGFBP2//TSPO//ACE//IGF2//NTRK1//LCN2//GHR//BMP6//MMP2//SPP1//PTGS2//BMP7//AQP1//T |
| GO:0002495 | antigen processing and presentation of peptide antigen via MHC class II | Biological process | 3 | 15 | 172 | 13692 | 15.9209302325581 | 0.000793144349457148 | 0.0307711931683198 | 3.1006477654344 | CD74//RT1-DB1//RT1-BB |
| GO:0090023 | positive regulation of neutrophil chemotaxis | Biological process | 3 | 15 | 172 | 13692 | 15.9209302325581 | 0.000793144349457148 | 0.0307711931683198 | 3.1006477654344 | CD74//XCL1//RGD1560691 |
| GO:0071260 | cellular response to mechanical stimulus | Biological process | 5 | 59 | 172 | 13692 | 6.7461568782026 | 0.00085419048806499 | 0.0321958038481637 | 3.0684452690177 | IGF2//GADD45A//AQP1//PTGS2//TLR3 |
| GO:0043065 | positive regulation of apoptotic process | Biological process | 16 | 520 | 172 | 13692 | 2.44937388193202 | 0.000861184887897832 | 0.0321958038481637 | 3.06490359979626 | CTSC//XCL1//C6//C7//TSPO//ACE//ANXA1//MSX2//PTGS2//MMP2//MSX1//BMP7//LPAR1//PLSCR1//TLR3//TCF7L2 |
| GO:0009967 | positive regulation of signal transduction | Biological process | 19 | 677 | 172 | 13692 | 2.23410394696163 | 0.000862780131655785 | 0.0321958038481637 | 3.06409986451016 | CD74//BMP6//BMP7//CDKN1C//MSX2//MSX1//TLR3//LPAR1//MESP1//CLCF1//GHR//S100A4//IGF2//PRRX2//NTRK1//C1QTNF3//TCF7L2//WNT2B//DAB2 |
| GO:0071495 | cellular response to endogenous stimulus | Biological process | 15 | 470 | 172 | 13692 | 2.54057397328055 | 0.000866295753396892 | 0.0321958038481637 | 3.06233381451802 | GHR//MMP2//IGFBP2//UGT1A6//PLOD2//IGF2//TCF7L2//BMP7//SLC5A5//MTUS1//ANXA1//RT1-BB//MSX2//AQP1//XCL1 |
| GO:0055082 | cellular chemical homeostasis | Biological process | 18 | 624 | 172 | 13692 | 2.29628801431127 | 0.000866584136424113 | 0.0321958038481637 | 3.06218926536688 | RGN//TRPV4//STIM2//ATP7A//CP//SCARA5//C7//NMB//DHRS7C//LCN2//LAMA2//CD9//TCF7L2//NDRG1//WFS1//LPAR1//XCL1//TSPO |
| GO:0016043 | cellular component organization | Biological process | 59 | 3233 | 172 | 13692 | 1.45272948302031 | 0.000925115578546585 | 0.0340815688768759 | 3.03380400565088 | TEKT2//MTFR1//MSX1//DAB2//IGFBP2//LMX1A//ATP7A//MSX2//FOXJ1//SPTA1//VAV3//MGP//CD74//GULP1//TRPV4//PDLIM3//GADD45A//CCP110//MEI4//CD9//NTRK1//BMP7//SEMA3C//LAMA2//PLSCR1//AQP1//APOA2//RGD1560691//LPAR1//TSPO//MMP2//HMGN5//LGALS3//TGFBI//OLFML2B//FBLN1//ANXA2//CXCL16//RERG//BAIAP2L1//ANXA1//LCN2//NDRG1//P2RX6//CLDN19//TCF7L2//KCNIP2//CLDN1//CD14//IGF2//SPP1//DCDC2//C1QTNF3//MMP14//MGST1//SCARA5//TMEM123//CCDC40//CRB3 |
| GO:0044093 | positive regulation of molecular function | Biological process | 22 | 848 | 172 | 13692 | 2.06521500658183 | 0.000952324630926166 | 0.0347915931831693 | 3.02121498293126 | GHR//CD74//LPAR1//C6//MDFIC//PCOLCE//MMP14//SPTA1//TCF7L2//STIM2//NTRK1//PON1//RGN//RGD1560691//FOXJ1//IGF2//ATP7A//VAV3//ABHD5//TLR3//ANXA2//PLSCR1 |
| GO:0006884 | cell volume homeostasis | Biological process | 3 | 16 | 172 | 13692 | 14.9258720930233 | 0.000967201112466954 | 0.0350430551822738 | 3.01448321262497 | SLC12A2//AQP1//SLC12A7 |
| GO:0070167 | regulation of biomineral tissue development | Biological process | 5 | 61 | 172 | 13692 | 6.52497140678612 | 0.000994786477094993 | 0.0357470812752824 | 3.00227012706409 | MGP//OMD//CREB3L1//BMP6//BMP7 |
| GO:0030154 | cell differentiation | Biological process | 45 | 2291 | 172 | 13692 | 1.56360074304914 | 0.00109036046865408 | 0.0388629292242235 | 2.9624299023647 | DAB2//T//IGF2//SPP1//MSX2//BMP6//SEMA3C//DCDC2//MSX1//WNT2B//TGFBI//RT1-BB//NTRK1//BMP7//LMX1A//LAMA2//TSPO//RGD1560691//TRPV4//LPAR1//CD9//TCF7L2//MMP2//ATP7A//MESP1//PLSCR1//CLCF1//ANXA1//FOXJ1//NDRG1//MGST1//FSCN2//CDKN1C//MMP14//CD74//KCNIP2//GHR//ZFHX3//ACE//PTGS2//MGP//MDK//LGALS3//WIPF3//ABHD5 |
| GO:0045321 | leukocyte activation | Biological process | 15 | 483 | 172 | 13692 | 2.47219413549039 | 0.00113958196058834 | 0.0401693481022946 | 2.94325443419471 | TLR3//RT1-BB//NTRK1//CLCF1//CD74//VAV3//PLSCR1//FOXJ1//ATP7A//SPTA1//IGF2//IGFBP2//XCL1//NDRG1//ANXA1 |
| GO:0002381 | immunoglobulin production involved in immunoglobulin mediated immune response | Biological process | 4 | 37 | 172 | 13692 | 8.60590823381521 | 0.0011492963066852 | 0.0401693481022946 | 2.93956798891965 | RT1-BB//XCL1//CLCF1//RT1-DB1 |
| GO:0060395 | SMAD protein signal transduction | Biological process | 3 | 17 | 172 | 13692 | 14.0478796169631 | 0.00116366496555461 | 0.0401693481022946 | 2.93417204078155 | BMP6//BMP7//T |
| GO:0072132 | mesenchyme morphogenesis | Biological process | 3 | 17 | 172 | 13692 | 14.0478796169631 | 0.00116366496555461 | 0.0401693481022946 | 2.93417204078155 | MSX2//MSX1//BMP7 |
| GO:0090100 | positive regulation of transmembrane receptor protein serine/threonine kinase signaling pathway | Biological process | 5 | 64 | 172 | 13692 | 6.21911337209302 | 0.00123698223018715 | 0.0423666413839099 | 2.90763653916401 | BMP6//BMP7//CDKN1C//MSX2//MSX1 |
| GO:0006805 | xenobiotic metabolic process | Biological process | 4 | 38 | 172 | 13692 | 8.37943696450428 | 0.00127204872627676 | 0.0425699359999795 | 2.89549625256511 | PON3//UGT1A6//GSTM2//FMO2 |
| GO:0007566 | embryo implantation | Biological process | 4 | 38 | 172 | 13692 | 8.37943696450428 | 0.00127204872627676 | 0.0425699359999795 | 2.89549625256511 | PTGS2//MMP2//SOSTDC1//FBLN1 |
| GO:0071466 | cellular response to xenobiotic stimulus | Biological process | 4 | 38 | 172 | 13692 | 8.37943696450428 | 0.00127204872627676 | 0.0425699359999795 | 2.89549625256511 | GSTM2//UGT1A6//FMO2//PON3 |
| GO:0006950 | response to stress | Biological process | 43 | 2176 | 172 | 13692 | 1.57306985294118 | 0.00129002835676125 | 0.0428445781518282 | 2.88940074315069 | MDK//ACE//MMP2//MMP14//PLOD2//SERPING1//C6//TLR3//APOA2//ANXA1//CREB3L1//CD74//PLSCR1//F5//SPP1//PTGS2//CD14//TRPV4//AQP1//SLC12A2//GADD45A//VAV3//LCN2//MDFIC//CD9//TSPO//ATP7A//NDRG1//WFS1//CXCL16//SCARA5//MSX2//IGF2//FMOD//CDH3//ANXA2//LGALS3//NTRK1//XCL1//RT1-BB//MGST1//RGD1305645//IGFBP2 |
| GO:0051098 | regulation of binding | Biological process | 8 | 169 | 172 | 13692 | 3.76826751066465 | 0.00132548801039578 | 0.0436912739667301 | 2.87762419629189 | DAB2//PLSCR1//SPTA1//TCF7L2//MSX1//ANXA2//PON1//MSX2 |
| GO:0001539 | ciliary or flagellar motility | Biological process | 3 | 18 | 172 | 13692 | 13.2674418604651 | 0.00138357230789042 | 0.0436958802796498 | 2.85899813894644 | CCDC40//DNAH5//TEKT2 |
| GO:0002478 | antigen processing and presentation of exogenous peptide antigen | Biological process | 3 | 18 | 172 | 13692 | 13.2674418604651 | 0.00138357230789042 | 0.0436958802796498 | 2.85899813894644 | CD74//RT1-DB1//RT1-BB |
| GO:0042104 | positive regulation of activated T cell proliferation | Biological process | 3 | 18 | 172 | 13692 | 13.2674418604651 | 0.00138357230789042 | 0.0436958802796498 | 2.85899813894644 | IGF2//IGFBP2//XCL1 |
| GO:0050482 | arachidonic acid secretion | Biological process | 3 | 18 | 172 | 13692 | 13.2674418604651 | 0.00138357230789042 | 0.0436958802796498 | 2.85899813894644 | ACE//ANXA1//NMB |
| GO:0090022 | regulation of neutrophil chemotaxis | Biological process | 3 | 18 | 172 | 13692 | 13.2674418604651 | 0.00138357230789042 | 0.0436958802796498 | 2.85899813894644 | CD74//XCL1//RGD1560691 |
| GO:0009628 | response to abiotic stimulus | Biological process | 22 | 873 | 172 | 13692 | 2.0060736833693 | 0.00138543051069145 | 0.0436958802796498 | 2.85841525232858 | ACE//MMP2//MMP14//PLOD2//MGP//IGFBP2//TRPV4//AQP1//SLC12A2//IGF2//PTGS2//NTRK1//CD14//CD9//ANXA1//PLSCR1//GADD45A//TLR3//SCARA5//LPAR1//NDRG1//TSPO |
| GO:0046456 | icosanoid biosynthetic process | Biological process | 4 | 39 | 172 | 13692 | 8.16457960644007 | 0.00140368296629694 | 0.0439553294588985 | 2.85273097022289 | PTGDS//CD74//PTGS2//ANXA1 |
| GO:0033273 | response to vitamin | Biological process | 9 | 212 | 172 | 13692 | 3.379442738043 | 0.00143916531267442 | 0.0447468136933664 | 2.84188931707192 | TSPO//BMP6//IGFBP2//MMP2//SPP1//PTGS2//BMP7//AQP1//T |
| GO:0002440 | production of molecular mediator of immune response | Biological process | 6 | 98 | 172 | 13692 | 4.87375415282392 | 0.00146057176401263 | 0.0447772490449746 | 2.83547709945798 | RT1-BB//RT1-DB1//XCL1//CLCF1//APOA2//CD74 |
| GO:0045834 | positive regulation of lipid metabolic process | Biological process | 6 | 98 | 172 | 13692 | 4.87375415282392 | 0.00146057176401263 | 0.0447772490449746 | 2.83547709945798 | APOA2//ABHD5//ANXA1//BMP6//VAV3//IGF2 |
| GO:0006575 | cellular modified amino acid metabolic process | Biological process | 7 | 134 | 172 | 13692 | 4.15845192641444 | 0.00150659268001067 | 0.0455510504080467 | 2.82200414697824 | GSTM2//MGST1//SLC5A5//GHR//PLSCR1//TTR//FOLR1 |
| GO:0090092 | regulation of transmembrane receptor protein serine/threonine kinase signaling pathway | Biological process | 7 | 134 | 172 | 13692 | 4.15845192641444 | 0.00150659268001067 | 0.0455510504080467 | 2.82200414697824 | BMP6//BMP7//CDKN1C//MSX2//MSX1//SOSTDC1//TCF7L2 |
| GO:0046461 | neutral lipid catabolic process | Biological process | 3 | 19 | 172 | 13692 | 12.5691554467564 | 0.00162791044892638 | 0.0482213473519814 | 2.78836948925444 | ABHD5//APOA2//CES1D |
| GO:0046464 | acylglycerol catabolic process | Biological process | 3 | 19 | 172 | 13692 | 12.5691554467564 | 0.00162791044892638 | 0.0482213473519814 | 2.78836948925444 | ABHD5//APOA2//CES1D |
| GO:0070207 | protein homotrimerization | Biological process | 3 | 19 | 172 | 13692 | 12.5691554467564 | 0.00162791044892638 | 0.0482213473519814 | 2.78836948925444 | LCN2//MGST1//SCARA5 |
| GO:0002696 | positive regulation of leukocyte activation | Biological process | 8 | 175 | 172 | 13692 | 3.63906976744186 | 0.00165443259190929 | 0.0484730178832924 | 2.78135092300894 | CD74//VAV3//CLCF1//SPTA1//IGF2//IGFBP2//XCL1//RT1-BB |
| GO:0051240 | positive regulation of multicellular organismal process | Biological process | 15 | 502 | 172 | 13692 | 2.37862503474474 | 0.00166776310224047 | 0.0484730178832924 | 2.77786563876809 | XCL1//PTGS2//AQP1//BMP6//BMP7//LAMA2//TLR3//CD14//GHR//SPP1//MSX2//NTRK1//CD74//MESP1//WNT2B |
| GO:0006810 | transport | Biological process | 50 | 2679 | 172 | 13692 | 1.48571577384828 | 0.00166957703019552 | 0.0484730178832924 | 2.77739353886352 | APOA2//CD74//AQP1//VAV3//TSPO//P2RX6//TRPM3//SLC12A2//SLC12A7//SLC5A5//TRPV4//SLC4A2//ATP7A//GULP1//EXOC2//SCARA5//DAB2//CXCL16//ANXA2//SLC2A12//PON1//DHRS7C//KDELR3//CP//MAGT1//SLC22A8//SLC16A8//SLCO1A5//SLC44A1//FOLR1//LCN2//PLSCR1//NMB//ANXA1//GHR//TCF7L2//STIM2//S100A6//MDFIC//PTGS2//TLR3//KRT18//CD14//ACE//RGD1560691//XCL1//WFS1//SLC16A6//TTR//PTGDS |
| GO:0051239 | regulation of multicellular organismal process | Biological process | 36 | 1747 | 172 | 13692 | 1.6403934984891 | 0.00171015326805433 | 0.0493244205733565 | 2.76696496534623 | XCL1//APOA2//FOXJ1//PTGS2//AQP1//NTRK1//RGD1560691//TRPV4//LPAR1//MGP//OMD//CREB3L1//BMP6//BMP7//TCF7L2//LAMA2//TLR3//TSPO//CD14//MESP1//GHR//MSX1//ANXA2//SERPING1//PTGDS//CD74//MSX2//ZFHX3//LMX1A//C6//SPP1//MMP2//CLCF1//ACE//WNT2B//DAB2 |
| GO:0006820 | anion transport | Biological process | 7 | 138 | 172 | 13692 | 4.03791708796764 | 0.00178376645528537 | 0.0511113211762814 | 2.74866200748253 | TSPO//SLC4A2//SLC12A2//SLC5A5//SLC16A8//SLCO1A5//SLC22A8 |
| GO:0006959 | humoral immune response | Biological process | 5 | 70 | 172 | 13692 | 5.68604651162791 | 0.00184854671443951 | 0.052009868781906 | 2.73316956993969 | SERPING1//C6//RT1-DB1//RT1-BB//FOXJ1 |
| GO:0060349 | bone morphogenesis | Biological process | 5 | 70 | 172 | 13692 | 5.68604651162791 | 0.00184854671443951 | 0.052009868781906 | 2.73316956993969 | BMP6//MSX2//GHR//MSX1//T |
| GO:0006636 | unsaturated fatty acid biosynthetic process | Biological process | 4 | 42 | 172 | 13692 | 7.58139534883721 | 0.00185519911382028 | 0.052009868781906 | 2.73160947182105 | PTGDS//CD74//PTGS2//ANXA1 |
| GO:0042981 | regulation of apoptotic process | Biological process | 25 | 1070 | 172 | 13692 | 1.85992175613997 | 0.00186257969862209 | 0.052009868781906 | 2.72988513499242 | CTSC//XCL1//CD74//TCF7L2//C6//C7//TSPO//ACE//ANXA1//MSX2//PTGS2//MMP2//MSX1//BMP7//LPAR1//PLSCR1//TLR3//AQP1//WFS1//KRT18//ATP7A//NTRK1//CLCF1//LCN2//RGD1560691 |
| GO:0042129 | regulation of T cell proliferation | Biological process | 6 | 103 | 172 | 13692 | 4.63716414540528 | 0.00188464951205223 | 0.052264940738839 | 2.72476940361973 | SPTA1//IGF2//IGFBP2//XCL1//FOXJ1//RT1-BB |
| GO:0014070 | response to organic cyclic compound | Biological process | 12 | 358 | 172 | 13692 | 2.66831232947902 | 0.00189555784157742 | 0.052264940738839 | 2.72226295885315 | SLC22A8//IGF2//NTRK1//MMP2//GHR//MSX1//GSTM2//ANXA1//PTGS2//MMP14//UGT1A6//FOXJ1 |
| GO:0030198 | extracellular matrix organization | Biological process | 7 | 140 | 172 | 13692 | 3.98023255813954 | 0.00193642962005189 | 0.0530581715894218 | 2.71299828291676 | ATP7A//ANXA2//LGALS3//TGFBI//OLFML2B//LAMA2//FBLN1 |
| GO:0043062 | extracellular structure organization | Biological process | 7 | 141 | 172 | 13692 | 3.95200395843642 | 0.00201646351067176 | 0.0549079256570497 | 2.69540963244996 | ATP7A//LGALS3//TGFBI//OLFML2B//LAMA2//FBLN1//ANXA2 |
| GO:0048705 | skeletal system morphogenesis | Biological process | 8 | 181 | 172 | 13692 | 3.51843762045484 | 0.00204496374825313 | 0.0553402535329736 | 2.68931438646993 | BMP6//MSX2//PRRX2//BMP7//MMP2//MSX1//T//GHR |
| GO:0002526 | acute inflammatory response | Biological process | 6 | 105 | 172 | 13692 | 4.54883720930233 | 0.0020781409874002 | 0.0558930680292176 | 2.68232499192183 | SERPING1//C6//ANXA1//PLSCR1//PTGS2//APOA2 |
| GO:0043067 | regulation of programmed cell death | Biological process | 25 | 1080 | 172 | 13692 | 1.84270025839793 | 0.00211336161954603 | 0.0560875884279382 | 2.67502618400013 | CTSC//XCL1//CD74//TCF7L2//C6//C7//MSX2//MSX1//WFS1//LCN2//RGD1560691//TSPO//ACE//ANXA1//PTGS2//MMP2//BMP7//LPAR1//PLSCR1//TLR3//AQP1//KRT18//NTRK1//ATP7A//CLCF1 |
| GO:0042063 | gliogenesis | Biological process | 8 | 182 | 172 | 13692 | 3.49910554561717 | 0.00211657405701424 | 0.0560875884279382 | 2.67436653135252 | TSPO//CD9//TCF7L2//LAMA2//NDRG1//MMP14//CLCF1//ANXA1 |
| GO:0042221 | response to chemical stimulus | Biological process | 63 | 3628 | 172 | 13692 | 1.38232994025794 | 0.0021237544888316 | 0.0560875884279382 | 2.672895690184 | CD74//CD14//XCL1//CREB3L1//GSTM2//UGT1A6//FMO2//ENPP2//CXCL16//NTRK1//BMP7//LMX1A//SEMA3C//LAMA2//CP//MGP//IGFBP2//TSPO//IGF2//AQP1//COX8B//ANXA1//APOA2//PTGS2//MDK//MMP2//CDH3//LCN2//HADHA//MGST1//VAV3//SLC22A8//PON1//PTGR1//GHR//MMP14//RERG//TCF7L2//SPP1//CTSC//CYBRD1//ATP7A//PON3//FOXJ1//WFS1//ACE//BMP6//PLOD2//KRT18//PLSCR1//TLR3//PTGDS//CCL6//ANXA2//T//MSX1//SLC5A5//RT1-BB//MSX2//MTUS1//LPAR1//NDRG1//RGD1560691 |
| GO:0071840 | cellular component organization or biogenesis | Biological process | 59 | 3342 | 172 | 13692 | 1.4053484196902 | 0.0021414960210402 | 0.0562174763846721 | 2.66928272819802 | TEKT2//MTFR1//MSX1//DAB2//IGFBP2//LMX1A//ATP7A//MSX2//FOXJ1//SPTA1//VAV3//MGP//CD74//GULP1//TRPV4//PDLIM3//GADD45A//CCP110//MEI4//CD9//NTRK1//BMP7//SEMA3C//LAMA2//PLSCR1//AQP1//APOA2//RGD1560691//LPAR1//TSPO//MMP2//HMGN5//LGALS3//TGFBI//OLFML2B//FBLN1//ANXA2//CXCL16//RERG//BAIAP2L1//ANXA1//LCN2//NDRG1//P2RX6//CLDN19//TCF7L2//KCNIP2//CLDN1//CD14//IGF2//SPP1//DCDC2//C1QTNF3//MMP14//MGST1//SCARA5//TMEM123//CCDC40//CRB3 |
| GO:0002504 | antigen processing and presentation of peptide or polysaccharide antigen via MHC class II | Biological process | 3 | 21 | 172 | 13692 | 11.3720930232558 | 0.00219359065927258 | 0.0572422705372083 | 2.65884441185936 | CD74//RT1-DB1//RT1-BB |
| GO:0009410 | response to xenobiotic stimulus | Biological process | 4 | 44 | 172 | 13692 | 7.23678646934461 | 0.0022066811238696 | 0.0572431363730434 | 2.65626041996445 | GSTM2//UGT1A6//FMO2//PON3 |
| GO:0050867 | positive regulation of cell activation | Biological process | 8 | 184 | 172 | 13692 | 3.46107178968655 | 0.00226571280958635 | 0.0581590820612959 | 2.64479513997851 | CD74//VAV3//CLCF1//SPTA1//IGF2//IGFBP2//XCL1//RT1-BB |
| GO:0048869 | cellular developmental process | Biological process | 46 | 2442 | 172 | 13692 | 1.4995143134678 | 0.00226852258952591 | 0.0581590820612959 | 2.64425689175278 | MSX1//DAB2//T//IGF2//SPP1//MSX2//BMP6//SEMA3C//DCDC2//WNT2B//TGFBI//RT1-BB//NTRK1//BMP7//LMX1A//LAMA2//TSPO//SPTA1//RGD1560691//TRPV4//LPAR1//CD9//TCF7L2//MMP2//ATP7A//MESP1//PLSCR1//MGP//MDK//LGALS3//WIPF3//ABHD5//CLCF1//ANXA1//FOXJ1//NDRG1//MGST1//FSCN2//CDKN1C//MMP14//CD74//KCNIP2//GHR//ZFHX3//ACE//PTGS2 |
| GO:0050793 | regulation of developmental process | Biological process | 29 | 1333 | 172 | 13692 | 1.73183412132103 | 0.0023372903275054 | 0.0595737255568818 | 2.6312873381845 | FOXJ1//SPTA1//NTRK1//RGD1560691//TRPV4//LPAR1//MGP//OMD//CREB3L1//BMP6//BMP7//TCF7L2//MESP1//MSX1//CD74//GHR//MSX2//ZFHX3//LMX1A//C6//AQP1//LAMA2//CLCF1//SPP1//ACE//TSPO//WNT2B//PTGS2//DAB2 |
| GO:0051234 | establishment of localization | Biological process | 50 | 2726 | 172 | 13692 | 1.460099984644 | 0.00244069990458217 | 0.0618498750386603 | 2.61248561582902 | APOA2//CD74//AQP1//VAV3//TTR//PTGDS//SLC44A1//TSPO//P2RX6//TRPM3//SLC12A2//SLC12A7//SLC5A5//TRPV4//SLC4A2//ATP7A//GULP1//EXOC2//SCARA5//DAB2//CXCL16//ANXA2//SLC2A12//PON1//DHRS7C//KDELR3//CP//MAGT1//SLC22A8//SLC16A8//SLCO1A5//FOLR1//LCN2//PLSCR1//NMB//ANXA1//GHR//TCF7L2//STIM2//S100A6//MDFIC//PTGS2//TLR3//KRT18//CD14//ACE//RGD1560691//XCL1//WFS1//SLC16A6 |
| GO:0010876 | lipid localization | Biological process | 8 | 187 | 172 | 13692 | 3.40554657380923 | 0.00250477702176417 | 0.0628034460090972 | 2.60123092948839 | APOA2//GULP1//PON1//ABHD5//PLSCR1//ACE//ANXA1//NMB |
| GO:0042475 | odontogenesis of dentin-containing tooth | Biological process | 5 | 75 | 172 | 13692 | 5.30697674418605 | 0.00250698062308212 | 0.0628034460090972 | 2.60084902277135 | MSX2//MSX1//BMP7//SOSTDC1//TCF7L2 |
| GO:0030593 | neutrophil chemotaxis | Biological process | 4 | 46 | 172 | 13692 | 6.9221435793731 | 0.00260157323901733 | 0.0645627635893439 | 2.58476394341647 | CD74//XCL1//RGD1560691//SPP1 |
| GO:0050870 | positive regulation of T cell activation | Biological process | 6 | 110 | 172 | 13692 | 4.34207188160677 | 0.00262748220835784 | 0.0645627635893439 | 2.5804602160564 | SPTA1//IGF2//IGFBP2//XCL1//CD74//RT1-BB |
| GO:0048608 | reproductive structure development | Biological process | 10 | 277 | 172 | 13692 | 2.87381412140039 | 0.0026409989225887 | 0.0645627635893439 | 2.57823177596607 | MMP2//MMP14//ADAMTS1//WNT2B//TLR3//TCF7L2//BMP6//MGST1//NTRK1//BMP7 |
| GO:0061458 | reproductive system development | Biological process | 10 | 277 | 172 | 13692 | 2.87381412140039 | 0.0026409989225887 | 0.0645627635893439 | 2.57823177596607 | MMP2//MMP14//ADAMTS1//WNT2B//TLR3//TCF7L2//BMP6//MGST1//NTRK1//BMP7 |
| GO:0071214 | cellular response to abiotic stimulus | Biological process | 7 | 148 | 172 | 13692 | 3.76508485229415 | 0.00265084339554788 | 0.0645627635893439 | 2.57661592841868 | TRPV4//IGF2//GADD45A//AQP1//PTGS2//TLR3//TSPO |
| GO:0002694 | regulation of leukocyte activation | Biological process | 10 | 279 | 172 | 13692 | 2.85321330332583 | 0.00278135483250349 | 0.0665178245370461 | 2.55574360230159 | CD74//VAV3//CLCF1//PLSCR1//FOXJ1//SPTA1//IGF2//IGFBP2//XCL1//RT1-BB |
| GO:0048646 | anatomical structure formation involved in morphogenesis | Biological process | 19 | 750 | 172 | 13692 | 2.0166511627907 | 0.00279496631568914 | 0.0665178245370461 | 2.55362342176628 | PTGS2//ANXA2//MMP2//MMP14//TGFBI//BMP7//T//SEMA3C//MESP1//MSX2//MSX1//ATP7A//CD9//FOXJ1//C6//AQP1//NTRK1//ADAMTS1//WNT2B |
| GO:0051099 | positive regulation of binding | Biological process | 5 | 77 | 172 | 13692 | 5.16913319238901 | 0.00281286366643398 | 0.0665178245370461 | 2.55085131666177 | SPTA1//TCF7L2//PLSCR1//ANXA2//PON1 |
| GO:0002673 | regulation of acute inflammatory response | Biological process | 4 | 47 | 172 | 13692 | 6.77486392874814 | 0.00281609722369008 | 0.0665178245370461 | 2.55035235557281 | SERPING1//C6//ANXA1//PTGS2 |
| GO:0007584 | response to nutrient | Biological process | 11 | 327 | 172 | 13692 | 2.67783230211223 | 0.0028314891581212 | 0.0665178245370461 | 2.54798509696422 | TSPO//BMP6//IGFBP2//MMP2//SPP1//PTGS2//BMP7//AQP1//T//CP//MGP |
| GO:0006972 | hyperosmotic response | Biological process | 3 | 23 | 172 | 13692 | 10.3832153690597 | 0.00286767081147393 | 0.0665178245370461 | 2.54247070410805 | AQP1//TRPV4//SLC12A2 |
| GO:0019884 | antigen processing and presentation of exogenous antigen | Biological process | 3 | 23 | 172 | 13692 | 10.3832153690597 | 0.00286767081147393 | 0.0665178245370461 | 2.54247070410805 | CD74//RT1-DB1//RT1-BB |
| GO:0045940 | positive regulation of steroid metabolic process | Biological process | 3 | 23 | 172 | 13692 | 10.3832153690597 | 0.00286767081147393 | 0.0665178245370461 | 2.54247070410805 | APOA2//BMP6//IGF2 |
| GO:0060343 | trabecula formation | Biological process | 3 | 23 | 172 | 13692 | 10.3832153690597 | 0.00286767081147393 | 0.0665178245370461 | 2.54247070410805 | MSX2//MMP2//ADAMTS1 |
| GO:0050767 | regulation of neurogenesis | Biological process | 13 | 428 | 172 | 13692 | 2.41789828298196 | 0.00293679548428827 | 0.0677626915953672 | 2.5321262962888 | NTRK1//RGD1560691//TRPV4//LPAR1//ZFHX3//BMP7//LMX1A//BMP6//SPP1//CLCF1//TCF7L2//ACE//TSPO |
| GO:0051249 | regulation of lymphocyte activation | Biological process | 9 | 236 | 172 | 13692 | 3.03577059519117 | 0.00298066402611221 | 0.0684148224632248 | 2.52568697392666 | CD74//VAV3//CLCF1//FOXJ1//SPTA1//IGF2//IGFBP2//XCL1//RT1-BB |
| GO:0006915 | apoptotic process | Biological process | 28 | 1295 | 172 | 13692 | 1.72118164676304 | 0.00305551734898401 | 0.0697676461351349 | 2.5149152460477 | ATP7A//CTSC//CD74//TCF7L2//C6//C7//MSX2//MSX1//WFS1//LCN2//RGD1560691//TSPO//ACE//ANXA1//PTGS2//MMP2//BMP7//LPAR1//PLSCR1//TLR3//AQP1//KRT18//NTRK1//CLCF1//CCL6//RNF152//GULP1 |
| GO:0045087 | innate immune response | Biological process | 9 | 242 | 172 | 13692 | 2.96050355564098 | 0.00351964712719314 | 0.0791989367884894 | 2.4535008758386 | SERPING1//TLR3//CXCL16//PLSCR1//RT1-BB//C6//CD14//LGALS3//LCN2 |
| GO:0035094 | response to nicotine | Biological process | 4 | 50 | 172 | 13692 | 6.36837209302326 | 0.00353174013611999 | 0.0791989367884894 | 2.45201125911515 | NTRK1//MSX1//IGF2//MMP2 |
| GO:0006725 | cellular aromatic compound metabolic process | Biological process | 8 | 198 | 172 | 13692 | 3.21634954193094 | 0.00355485849496243 | 0.0791989367884894 | 2.44917768215787 | ATP7A//SLC5A5//PON3//UGT1A6//PON1//TTR//HDC//FOLR1 |
| GO:0008219 | cell death | Biological process | 29 | 1372 | 172 | 13692 | 1.68260560037969 | 0.00356100262993114 | 0.0791989367884894 | 2.44842770558321 | ATP7A//CTSC//CD74//PLSCR1//LCN2//CCL6//RNF152//KRT18//GULP1//TCF7L2//C6//C7//NTRK1//TSPO//PTGS2//BMP7//LPAR1//MSX2//MSX1//WFS1//RGD1560691//ACE//ANXA1//MMP2//TLR3//AQP1//CLCF1//TMEM123 |
| GO:0035295 | tube development | Biological process | 13 | 438 | 172 | 13692 | 2.36269512583625 | 0.00357441651985783 | 0.0791989367884894 | 2.44679484138383 | BMP7//EPCAM//T//FOXJ1//MESP1//CP//MGP//MMP14//ATP7A//MSX2//SLC12A2//WNT2B//AQP1 |
| GO:0010941 | regulation of cell death | Biological process | 25 | 1124 | 172 | 13692 | 1.77056608458164 | 0.00359044077170283 | 0.0791989367884894 | 2.44485223304621 | CTSC//XCL1//CD74//TCF7L2//C6//C7//TSPO//PTGS2//BMP7//LPAR1//MSX2//MSX1//WFS1//LCN2//RGD1560691//ACE//ANXA1//MMP2//PLSCR1//TLR3//AQP1//KRT18//NTRK1//ATP7A//CLCF1 |
| GO:0012501 | programmed cell death | Biological process | 28 | 1310 | 172 | 13692 | 1.70147345996805 | 0.00359502473104685 | 0.0791989367884894 | 2.44429811765377 | ATP7A//CTSC//CD74//PLSCR1//LCN2//CCL6//RNF152//KRT18//GULP1//TCF7L2//C6//C7//NTRK1//MSX2//MSX1//WFS1//RGD1560691//TSPO//ACE//ANXA1//PTGS2//MMP2//BMP7//LPAR1//TLR3//AQP1//CLCF1 |
| GO:0002920 | regulation of humoral immune response | Biological process | 3 | 25 | 172 | 13692 | 9.55255813953488 | 0.00365641077498393 | 0.0797497753110923 | 2.43694501991645 | SERPING1//C6//FOXJ1 |
| GO:0046006 | regulation of activated T cell proliferation | Biological process | 3 | 25 | 172 | 13692 | 9.55255813953488 | 0.00365641077498393 | 0.0797497753110923 | 2.43694501991645 | IGF2//IGFBP2//XCL1 |
| GO:0051050 | positive regulation of transport | Biological process | 14 | 492 | 172 | 13692 | 2.26517300056722 | 0.00368342324449293 | 0.0797916066562458 | 2.43374837558254 | AQP1//PON1//NMB//ANXA1//ANXA2//TCF7L2//STIM2//PTGS2//TLR3//CD14//RGD1560691//XCL1//TSPO//WFS1 |
| GO:0007399 | nervous system development | Biological process | 31 | 1503 | 172 | 13692 | 1.64187903263241 | 0.00370343212881591 | 0.0797916066562458 | 2.43139561006486 | DCDC2//T//NTRK1//BMP7//LMX1A//SEMA3C//LAMA2//CD9//ZFHX3//TCF7L2//TSPO//RGD1560691//TRPV4//LPAR1//MMP2//MDK//AQP1//ATP7A//WNT2B//MSX1//MESP1//NDRG1//ANXA1//FSCN2//CDKN1C//MMP14//KCNIP2//BMP6//SPP1//CLCF1//ACE |
| GO:0016265 | death | Biological process | 29 | 1376 | 172 | 13692 | 1.67771430502975 | 0.00371293060170487 | 0.0797916066562458 | 2.43028316808553 | ATP7A//CTSC//CD74//PLSCR1//LCN2//CCL6//RNF152//KRT18//GULP1//TCF7L2//C6//C7//NTRK1//TSPO//PTGS2//BMP7//LPAR1//MSX2//MSX1//WFS1//RGD1560691//ACE//ANXA1//MMP2//TLR3//AQP1//CLCF1//TMEM123 |
| GO:0034097 | response to cytokine stimulus | Biological process | 12 | 389 | 172 | 13692 | 2.45567047288815 | 0.00373277555677734 | 0.0798267709312774 | 2.42796812241859 | CD74//KRT18//CXCL16//PTGS2//PLSCR1//TLR3//GHR//ANXA1//MMP2//RT1-BB//LCN2//XCL1 |
| GO:0051094 | positive regulation of developmental process | Biological process | 17 | 657 | 172 | 13692 | 2.05978549431879 | 0.00376223137825854 | 0.0800661279722594 | 2.4245544987349 | BMP6//BMP7//MESP1//TCF7L2//CD74//GHR//ZFHX3//MSX2//C6//AQP1//NTRK1//CLCF1//ACE//TSPO//WNT2B//LPAR1//PTGS2 |
| GO:0050776 | regulation of immune response | Biological process | 11 | 340 | 172 | 13692 | 2.57544459644323 | 0.00380686057986026 | 0.080624525517427 | 2.41943302823058 | SERPING1//XCL1//C6//TLR3//FOXJ1//APOA2//RT1-BB//CD74//PLSCR1//CLCF1//VAV3 |
| GO:0000904 | cell morphogenesis involved in differentiation | Biological process | 14 | 495 | 172 | 13692 | 2.25144467935166 | 0.0038869256163744 | 0.0816350598992912 | 2.41039377033021 | MSX1//MSX2//NTRK1//BMP7//LMX1A//SEMA3C//LAMA2//TSPO//MMP2//SPP1//ATP7A//DCDC2//DAB2 |
| GO:0032101 | regulation of response to external stimulus | Biological process | 11 | 341 | 172 | 13692 | 2.56789197299325 | 0.00389181740852004 | 0.0816350598992912 | 2.40984754361755 | SERPING1//C6//ANXA1//XCL1//PTGS2//ANXA2//SPP1//ACE//RGD1560691//LPAR1//CD74 |
| GO:0016044 | cellular membrane organization | Biological process | 10 | 293 | 172 | 13692 | 2.71688229224542 | 0.00393950252308122 | 0.0822418050532765 | 2.40455861703776 | VAV3//GULP1//SPTA1//CD9//PLSCR1//ANXA1//ANXA2//NDRG1//KCNIP2//CRB3 |
| GO:0008285 | negative regulation of cell proliferation | Biological process | 13 | 445 | 172 | 13692 | 2.32552913509276 | 0.00408451261493656 | 0.0846929000861271 | 2.38885975829235 | FOXJ1//RT1-BB//OGN//CDKN1C//TSPO//BMP7//XCL1//CD9//MSX2//PTGS2//NTRK1//MSX1//RERG |
| GO:0002702 | positive regulation of production of molecular mediator of immune response | Biological process | 3 | 26 | 172 | 13692 | 9.18515205724508 | 0.00409555082533279 | 0.0846929000861271 | 2.38768768015143 | CLCF1//XCL1//CD74 |
| GO:0001775 | cell activation | Biological process | 15 | 555 | 172 | 13692 | 2.1514770584538 | 0.00431567298244122 | 0.0888258702113723 | 2.36495147114898 | TLR3//RT1-BB//NTRK1//CLCF1//CD74//VAV3//PLSCR1//FOXJ1//ATP7A//SPTA1//IGF2//IGFBP2//XCL1//NDRG1//ANXA1 |
| GO:0061024 | membrane organization | Biological process | 10 | 298 | 172 | 13692 | 2.67129701888559 | 0.00443528994932688 | 0.0908612669992946 | 2.35307798366166 | VAV3//GULP1//SPTA1//CD9//PLSCR1//ANXA1//ANXA2//NDRG1//KCNIP2//CRB3 |
| GO:0003007 | heart morphogenesis | Biological process | 7 | 163 | 172 | 13692 | 3.41860465116279 | 0.0045184037391799 | 0.0921334046165799 | 2.34501496556618 | FOXJ1//MESP1//MSX2//SEMA3C//MSX1//ADAMTS1//T |
| GO:0006906 | vesicle fusion | Biological process | 3 | 27 | 172 | 13692 | 8.84496124031008 | 0.00456540272282939 | 0.0922337582344887 | 2.34052090650029 | ANXA1//ANXA2//VAV3 |
| GO:0060325 | face morphogenesis | Biological process | 3 | 27 | 172 | 13692 | 8.84496124031008 | 0.00456540272282939 | 0.0922337582344887 | 2.34052090650029 | MMP2//MSX1//TCF7L2 |
| GO:0050865 | regulation of cell activation | Biological process | 10 | 300 | 172 | 13692 | 2.65348837209302 | 0.00464688256854101 | 0.0930827105338787 | 2.33283830244668 | CD74//VAV3//CLCF1//PLSCR1//FOXJ1//SPTA1//IGF2//IGFBP2//XCL1//RT1-BB |
| GO:0046649 | lymphocyte activation | Biological process | 12 | 400 | 172 | 13692 | 2.38813953488372 | 0.00465611604385612 | 0.0930827105338787 | 2.33197620435079 | RT1-BB//NTRK1//CLCF1//CD74//VAV3//FOXJ1//ATP7A//SPTA1//IGF2//IGFBP2//XCL1//ANXA1 |
| GO:0006869 | lipid transport | Biological process | 7 | 164 | 172 | 13692 | 3.39775950085082 | 0.00467112142277676 | 0.0930827105338787 | 2.33057884336164 | PON1//PLSCR1//APOA2//ACE//ANXA1//NMB//GULP1 |
| GO:0035270 | endocrine system development | Biological process | 6 | 124 | 172 | 13692 | 3.85183795948987 | 0.00474652331505694 | 0.0941229370730201 | 2.32362438151851 | BMP6//MSX1//TCF7L2//TSPO//MDK//ANXA1 |
| GO:0009888 | tissue development | Biological process | 25 | 1149 | 172 | 13692 | 1.73204201833748 | 0.00476626186820494 | 0.0941229370730201 | 2.32182210030427 | BMP7//SEMA3C//MSX1//T//FOXJ1//MESP1//WNT2B//TGFBI//MSX2//DAB2//ANXA1//PTGS2//TCF7L2//MGP//OMD//CREB3L1//BMP6//ATP7A//SPP1//ZFHX3//PRRX2//GHR//SLC12A2//MMP2//AQP1 |
| GO:0008284 | positive regulation of cell proliferation | Biological process | 16 | 618 | 172 | 13692 | 2.06096184240235 | 0.00488051180259829 | 0.0959469226125153 | 2.31153463263062 | BMP6//PRRX2//CD74//VAV3//CLCF1//SPTA1//IGF2//IGFBP2//XCL1//AQP1//PTGS2//TCF7L2//TSPO//EPCAM//T//NMB |
| GO:0044242 | cellular lipid catabolic process | Biological process | 6 | 125 | 172 | 13692 | 3.82102325581395 | 0.00493514841992038 | 0.0965879047898703 | 2.30669978180222 | HADHA//BDH2//APOA2//ABHD5//ENPP2//CES1D |
| GO:0048732 | gland development | Biological process | 10 | 303 | 172 | 13692 | 2.62721620999309 | 0.00497911377081657 | 0.0970152656500437 | 2.30284795015086 | BMP7//ATP7A//MSX1//TCF7L2//TSPO//MDK//MSX2//SLC12A2//SEMA3C//MMP2 |
| GO:0032309 | icosanoid secretion | Biological process | 3 | 28 | 172 | 13692 | 8.52906976744186 | 0.00506656707740701 | 0.0978494716623451 | 2.29528620325338 | ACE//ANXA1//NMB |
| GO:0032350 | regulation of hormone metabolic process | Biological process | 3 | 28 | 172 | 13692 | 8.52906976744186 | 0.00506656707740701 | 0.0978494716623451 | 2.29528620325338 | BMP6//IGF2//TCF7L2 |
| GO:0010817 | regulation of hormone levels | Biological process | 11 | 354 | 172 | 13692 | 2.47359085534095 | 0.00514080926830701 | 0.0988478413695523 | 2.28896850880723 | SLC5A5//NMB//ANXA1//TRPV4//TCF7L2//BMP6//TTR//ACE//AQP1//SLCO1A5//IGF2 |
| GO:0042552 | myelination | Biological process | 5 | 89 | 172 | 13692 | 4.47217141363993 | 0.00524023104423615 | 0.100264827899598 | 2.28064956434743 | LAMA2//CD9//TCF7L2//NDRG1//LPAR1 |
| GO:0050863 | regulation of T cell activation | Biological process | 7 | 168 | 172 | 13692 | 3.31686046511628 | 0.00532126052801893 | 0.100264827899598 | 2.27398547757008 | FOXJ1//SPTA1//IGF2//IGFBP2//XCL1//RT1-BB//CD74 |
| GO:0035107 | appendage morphogenesis | Biological process | 6 | 127 | 172 | 13692 | 3.76084966123421 | 0.00532885604484632 | 0.100264827899598 | 2.27336601174883 | MSX2//MSX1//BMP7//PRRX2//ATP7A//SEMA3C |
| GO:0035108 | limb morphogenesis | Biological process | 6 | 127 | 172 | 13692 | 3.76084966123421 | 0.00532885604484632 | 0.100264827899598 | 2.27336601174883 | MSX2//MSX1//BMP7//PRRX2//ATP7A//SEMA3C |
| GO:0060326 | cell chemotaxis | Biological process | 6 | 127 | 172 | 13692 | 3.76084966123421 | 0.00532885604484632 | 0.100264827899598 | 2.27336601174883 | XCL1//SPP1//RGD1560691//LPAR1//CD74//CCL6 |
| GO:0044283 | small molecule biosynthetic process | Biological process | 10 | 307 | 172 | 13692 | 2.59298537989546 | 0.00545084701966049 | 0.102052331122027 | 2.26353600646058 | PTGDS//CD74//PTGS2//ABHD5//PLSCR1//ELOVL7//RGN//ANXA1//ATP7A//HDC |
| GO:0007548 | sex differentiation | Biological process | 9 | 259 | 172 | 13692 | 2.7661847894406 | 0.00547041464728018 | 0.102052331122027 | 2.261979753703 | MMP2//MMP14//ADAMTS1//WNT2B//TLR3//TCF7L2//BMP6//MGST1//NTRK1 |
| GO:0071702 | organic substance transport | Biological process | 13 | 462 | 172 | 13692 | 2.2399577167019 | 0.00557177659426075 | 0.103502833005251 | 2.25400630535376 | APOA2//GULP1//SLC2A12//PON1//SLC22A8//SLCO1A5//AQP1//SLC44A1//FOLR1//PLSCR1//ACE//ANXA1//NMB |
| GO:0055072 | iron ion homeostasis | Biological process | 4 | 57 | 172 | 13692 | 5.58629130966952 | 0.00566059921634892 | 0.10426918892636 | 2.247137593091 | CP//SCARA5//LCN2//BDH2 |
| GO:0071385 | cellular response to glucocorticoid stimulus | Biological process | 4 | 57 | 172 | 13692 | 5.58629130966952 | 0.00566059921634892 | 0.10426918892636 | 2.247137593091 | AQP1//ANXA1//UGT1A6//RT1-BB |
| GO:0042098 | T cell proliferation | Biological process | 6 | 129 | 172 | 13692 | 3.7025419145484 | 0.00574511726522164 | 0.105383238873354 | 2.2407011023771 | SPTA1//IGF2//IGFBP2//XCL1//FOXJ1//RT1-BB |
| GO:0071384 | cellular response to corticosteroid stimulus | Biological process | 4 | 58 | 172 | 13692 | 5.48997594226143 | 0.00602140471235671 | 0.109149822291784 | 2.22030218188829 | ANXA1//UGT1A6//RT1-BB//AQP1 |
| GO:0007272 | ensheathment of neurons | Biological process | 5 | 92 | 172 | 13692 | 4.32633973710819 | 0.00602514986190963 | 0.109149822291784 | 2.22003214653052 | LAMA2//CD9//TCF7L2//NDRG1//LPAR1 |
| GO:0008366 | axon ensheathment | Biological process | 5 | 92 | 172 | 13692 | 4.32633973710819 | 0.00602514986190963 | 0.109149822291784 | 2.22003214653052 | LAMA2//CD9//TCF7L2//NDRG1//LPAR1 |
| GO:0048699 | generation of neurons | Biological process | 21 | 929 | 172 | 13692 | 1.7994592835507 | 0.00633464254583867 | 0.114284250703526 | 2.19827788666052 | DCDC2//NTRK1//BMP7//LMX1A//SEMA3C//LAMA2//RGD1560691//TRPV4//LPAR1//TSPO//MMP2//ATP7A//FSCN2//CDKN1C//KCNIP2//ZFHX3//BMP6//SPP1//CLCF1//TCF7L2//ACE |
| GO:0050896 | response to stimulus | Biological process | 99 | 6556 | 172 | 13692 | 1.20208365849852 | 0.00656517165174811 | 0.117704198174842 | 2.18275391444389 | GHR//CD74//LPAR1//MDK//ACE//MMP2//MMP14//PLOD2//ATP7A//SERPING1//CTSC//XCL1//C6//TLR3//CD14//RT1-BB//RT1-DB1//APOA2//FOXJ1//ANXA1//CREB3L1//PLSCR1//LCN2//F5//WNT2B//SOSTDC1//TCF7L2//IGFBP2//BAIAP2L1//DCDC2//ARHGAP29//ASB14//GSTM2//UGT1A6//FMO2//ENPP2//CXCL16//MGP//TSPO//SPP1//PTGS2//MR1//BMP6//CCL6//TRPV4//AQP1//SLC12A2//GADD45A//VAV3//CLCF1//WFIKKN2//SMURF2//NMB//MDFIC//RASGRF2//RHOD//RERG//NTRK1//BMP7//LMX1A//SEMA3C//LAMA2//CP//IGF2//COX8B//CDH3//HADHA//MGST1//SLC22A8//PON1//PTGR1//CD9//CYBRD1//PON3//MSX2//MSX1//T//MESP1//NDRG1//CDKN1C//WFS1//KRT18//SCARA5//ARHGEF26//P2RX6//FMOD//ANXA2//S100A4//LGALS3//PTGDS//PRRX2//RGN//C1QTNF3//SLC5A5//MTUS1//RGD1560691//DAB2//RGD1305645 |
| GO:0007167 | enzyme linked receptor protein signaling pathway | Biological process | 14 | 526 | 172 | 13692 | 2.11875497391458 | 0.00657790341077469 | 0.117704198174842 | 2.18191250793995 | WFIKKN2//SMURF2//BMP6//BMP7//MSX2//MSX1//T//CDKN1C//SOSTDC1//TCF7L2//IGF2//IGFBP2//GHR//NTRK1 |
| GO:0002921 | negative regulation of humoral immune response | Biological process | 2 | 10 | 172 | 13692 | 15.9209302325581 | 0.00660774769229877 | 0.117757584890398 | 2.17994654830486 | SERPING1//FOXJ1 |
| GO:0046470 | phosphatidylcholine metabolic process | Biological process | 3 | 31 | 172 | 13692 | 7.70367591897975 | 0.0067633876092954 | 0.118333671863822 | 2.16983572247347 | APOA2//ENPP2//PON1 |
| GO:0050798 | activated T cell proliferation | Biological process | 3 | 31 | 172 | 13692 | 7.70367591897975 | 0.0067633876092954 | 0.118333671863822 | 2.16983572247347 | IGF2//IGFBP2//XCL1 |
| GO:0060323 | head morphogenesis | Biological process | 3 | 31 | 172 | 13692 | 7.70367591897975 | 0.0067633876092954 | 0.118333671863822 | 2.16983572247347 | MMP2//MSX1//TCF7L2 |
| GO:0061383 | trabecula morphogenesis | Biological process | 3 | 31 | 172 | 13692 | 7.70367591897975 | 0.0067633876092954 | 0.118333671863822 | 2.16983572247347 | MSX2//MMP2//ADAMTS1 |
| GO:0030510 | regulation of BMP signaling pathway | Biological process | 4 | 60 | 172 | 13692 | 5.30697674418605 | 0.00678825245836095 | 0.118333671863822 | 2.16824201443417 | MSX2//MSX1//SOSTDC1//TCF7L2 |
| GO:0030030 | cell projection organization | Biological process | 18 | 756 | 172 | 13692 | 1.8953488372093 | 0.00680202675859561 | 0.118333671863822 | 2.16736166392233 | NTRK1//BMP7//LMX1A//SEMA3C//LAMA2//AQP1//RGD1560691//TRPV4//LPAR1//TSPO//MMP2//VAV3//LCN2//FOXJ1//BAIAP2L1//SPP1//ATP7A//DCDC2 |
| GO:0006631 | fatty acid metabolic process | Biological process | 9 | 269 | 172 | 13692 | 2.66335264113426 | 0.00695776423687745 | 0.120564578713323 | 2.15753029136277 | PTGDS//CD74//PTGS2//HADHA//BDH2//ELOVL7//ANXA1//GHR//ABHD5 |
| GO:0002697 | regulation of immune effector process | Biological process | 7 | 177 | 172 | 13692 | 3.14820654316121 | 0.00703050442723198 | 0.121345399247972 | 2.15301351394117 | SERPING1//XCL1//C6//CLCF1//APOA2//FOXJ1//CD74 |
| GO:0048736 | appendage development | Biological process | 6 | 135 | 172 | 13692 | 3.53798449612403 | 0.00713652992787723 | 0.122213075014898 | 2.1465129086028 | MSX2//MSX1//BMP7//PRRX2//ATP7A//SEMA3C |
| GO:0060173 | limb development | Biological process | 6 | 135 | 172 | 13692 | 3.53798449612403 | 0.00713652992787723 | 0.122213075014898 | 2.1465129086028 | MSX2//MSX1//BMP7//PRRX2//ATP7A//SEMA3C |
| GO:0048666 | neuron development | Biological process | 16 | 644 | 172 | 13692 | 1.97775530839232 | 0.00717290120937826 | 0.122357972380989 | 2.14430515055552 | NTRK1//BMP7//LMX1A//SEMA3C//LAMA2//RGD1560691//TRPV4//LPAR1//TSPO//MMP2//ATP7A//FSCN2//CDKN1C//KCNIP2//SPP1//DCDC2 |
| GO:0003156 | regulation of organ formation | Biological process | 3 | 32 | 172 | 13692 | 7.46293604651163 | 0.00739507726356106 | 0.124692379705583 | 2.13105728414675 | MESP1//WNT2B//BMP7 |
| GO:0030728 | ovulation | Biological process | 3 | 32 | 172 | 13692 | 7.46293604651163 | 0.00739507726356106 | 0.124692379705583 | 2.13105728414675 | ADAMTS1//MMP2//PTGS2 |
| GO:0071715 | icosanoid transport | Biological process | 3 | 32 | 172 | 13692 | 7.46293604651163 | 0.00739507726356106 | 0.124692379705583 | 2.13105728414675 | ACE//ANXA1//NMB |
| GO:0051960 | regulation of nervous system development | Biological process | 13 | 480 | 172 | 13692 | 2.15595930232558 | 0.0075910803946565 | 0.127456089716435 | 2.11969640907869 | NTRK1//RGD1560691//TRPV4//LPAR1//TCF7L2//ZFHX3//BMP7//LMX1A//BMP6//SPP1//CLCF1//ACE//TSPO |
| GO:0002700 | regulation of production of molecular mediator of immune response | Biological process | 4 | 62 | 172 | 13692 | 5.1357839459865 | 0.00761712944929423 | 0.127456089716435 | 2.11820866372702 | CLCF1//XCL1//APOA2//CD74 |
| GO:0015697 | quaternary ammonium group transport | Biological process | 2 | 11 | 172 | 13692 | 14.4735729386892 | 0.00800981002413311 | 0.128968693670265 | 2.09637778434636 | SLC44A1//SLC22A8 |
| GO:0030449 | regulation of complement activation | Biological process | 2 | 11 | 172 | 13692 | 14.4735729386892 | 0.00800981002413311 | 0.128968693670265 | 2.09637778434636 | SERPING1//C6 |
| GO:0042640 | anagen | Biological process | 2 | 11 | 172 | 13692 | 14.4735729386892 | 0.00800981002413311 | 0.128968693670265 | 2.09637778434636 | MSX2//PTGS2 |
| GO:0044241 | lipid digestion | Biological process | 2 | 11 | 172 | 13692 | 14.4735729386892 | 0.00800981002413311 | 0.128968693670265 | 2.09637778434636 | APOA2//AQP1 |
| GO:2000257 | regulation of protein activation cascade | Biological process | 2 | 11 | 172 | 13692 | 14.4735729386892 | 0.00800981002413311 | 0.128968693670265 | 2.09637778434636 | SERPING1//C6 |
| GO:0006690 | icosanoid metabolic process | Biological process | 4 | 63 | 172 | 13692 | 5.05426356589147 | 0.00805545115201745 | 0.128968693670265 | 2.09391013155204 | PTGDS//CD74//PTGS2//ANXA1 |
| GO:0006970 | response to osmotic stress | Biological process | 4 | 63 | 172 | 13692 | 5.05426356589147 | 0.00805545115201745 | 0.128968693670265 | 2.09391013155204 | TRPV4//AQP1//SLC12A2//TSPO |
| GO:0032846 | positive regulation of homeostatic process | Biological process | 4 | 63 | 172 | 13692 | 5.05426356589147 | 0.00805545115201745 | 0.128968693670265 | 2.09391013155204 | PTGS2//SPP1//XCL1//TSPO |
| GO:0002712 | regulation of B cell mediated immunity | Biological process | 3 | 33 | 172 | 13692 | 7.23678646934461 | 0.00806054335439155 | 0.128968693670265 | 2.09363568178544 | FOXJ1//CLCF1//XCL1 |
| GO:0002889 | regulation of immunoglobulin mediated immune response | Biological process | 3 | 33 | 172 | 13692 | 7.23678646934461 | 0.00806054335439155 | 0.128968693670265 | 2.09363568178544 | FOXJ1//CLCF1//XCL1 |
| GO:0046688 | response to copper ion | Biological process | 3 | 33 | 172 | 13692 | 7.23678646934461 | 0.00806054335439155 | 0.128968693670265 | 2.09363568178544 | ATP7A//AQP1//CP |
| GO:0046717 | acid secretion | Biological process | 3 | 33 | 172 | 13692 | 7.23678646934461 | 0.00806054335439155 | 0.128968693670265 | 2.09363568178544 | ACE//ANXA1//NMB |
| GO:0006952 | defense response | Biological process | 17 | 711 | 172 | 13692 | 1.90334608968698 | 0.0081390447059416 | 0.129751170875811 | 2.0894265660303 | MDK//SERPING1//C6//TLR3//APOA2//ANXA1//PLSCR1//SPP1//PTGS2//CD14//CXCL16//LGALS3//LCN2//XCL1//ACE//RT1-BB//CD74 |
| GO:0032103 | positive regulation of response to external stimulus | Biological process | 6 | 139 | 172 | 13692 | 3.43617199263845 | 0.0081902585478141 | 0.130094541571076 | 2.08670238833523 | XCL1//PTGS2//ACE//LPAR1//CD74//RGD1560691 |
| GO:0050868 | negative regulation of T cell activation | Biological process | 4 | 64 | 172 | 13692 | 4.97529069767442 | 0.00851001574680613 | 0.134685592180498 | 2.07006963630264 | FOXJ1//RT1-BB//CD74//XCL1 |
| GO:0070206 | protein trimerization | Biological process | 3 | 34 | 172 | 13692 | 7.02393980848153 | 0.00876017786136915 | 0.138146114187922 | 2.05748707608911 | LCN2//MGST1//SCARA5 |
| GO:0060429 | epithelium development | Biological process | 14 | 545 | 172 | 13692 | 2.04489012161297 | 0.00885789130935631 | 0.139186363800065 | 2.05266965302353 | T//FOXJ1//MESP1//ANXA1//BMP7//BMP6//MSX2//SLC12A2//WNT2B//SEMA3C//MMP2//MSX1//AQP1 |
| GO:0006944 | cellular membrane fusion | Biological process | 4 | 65 | 172 | 13692 | 4.89874776386404 | 0.00898105671421504 | 0.139307655354866 | 2.0466725610885 | VAV3//CD9//ANXA1//ANXA2 |
| GO:0042102 | positive regulation of T cell proliferation | Biological process | 4 | 65 | 172 | 13692 | 4.89874776386404 | 0.00898105671421504 | 0.139307655354866 | 2.0466725610885 | IGF2//IGFBP2//XCL1//SPTA1 |
| GO:0050795 | regulation of behavior | Biological process | 6 | 142 | 172 | 13692 | 3.36357680969538 | 0.00905085590185896 | 0.139307655354866 | 2.04331034942705 | XCL1//PTGDS//RGD1560691//LPAR1//CD74//MDK |
| GO:0042110 | T cell activation | Biological process | 9 | 281 | 172 | 13692 | 2.54961516179757 | 0.00913191816071419 | 0.139307655354866 | 2.03943798926563 | FOXJ1//ATP7A//SPTA1//IGF2//IGFBP2//XCL1//RT1-BB//CD74//ANXA1 |
| GO:0044281 | small molecule metabolic process | Biological process | 31 | 1601 | 172 | 13692 | 1.54137675580669 | 0.00920794701421753 | 0.139307655354866 | 2.03583718845506 | PTGDS//CD74//PTGS2//FMO2//GHR//GSTM2//MGST1//AQP1//RERG//HDC//ATP7A//SLC5A5//ABHD5//HADHA//BDH2//APOA2//PLSCR1//PON1//ELOVL7//UGT1A6//RGN//ANXA1//ACAP2//NTRK1//FOXJ1//ENPP2//TTR//VAV3//C1QTNF3//PON3//FOLR1 |
| GO:0043433 | negative regulation of sequence-specific DNA binding transcription factor activity | Biological process | 5 | 102 | 172 | 13692 | 3.90218878248974 | 0.00923634597717329 | 0.139307655354866 | 2.03449980754752 | FOXJ1//MSX2//WFS1//XCL1//TCF7L2 |
| GO:0060348 | bone development | Biological process | 5 | 102 | 172 | 13692 | 3.90218878248974 | 0.00923634597717329 | 0.139307655354866 | 2.03449980754752 | BMP6//MSX2//MSX1//T//GHR |
| GO:0033559 | unsaturated fatty acid metabolic process | Biological process | 4 | 66 | 172 | 13692 | 4.82452431289641 | 0.0094688017800156 | 0.139307655354866 | 2.02370497487756 | PTGDS//CD74//PTGS2//ANXA1 |
| GO:0045137 | development of primary sexual characteristics | Biological process | 8 | 234 | 172 | 13692 | 2.72152653548002 | 0.00948419534775037 | 0.139307655354866 | 2.02299950935683 | MMP2//MMP14//ADAMTS1//WNT2B//TLR3//BMP6//MGST1//NTRK1 |
| GO:0030890 | positive regulation of B cell proliferation | Biological process | 3 | 35 | 172 | 13692 | 6.82325581395349 | 0.00949434139052125 | 0.139307655354866 | 2.02253515629641 | CD74//VAV3//CLCF1 |
| GO:0033280 | response to vitamin D | Biological process | 3 | 35 | 172 | 13692 | 6.82325581395349 | 0.00949434139052125 | 0.139307655354866 | 2.02253515629641 | SPP1//PTGS2//BMP7 |
| GO:0060324 | face development | Biological process | 3 | 35 | 172 | 13692 | 6.82325581395349 | 0.00949434139052125 | 0.139307655354866 | 2.02253515629641 | MMP2//MSX1//TCF7L2 |
| GO:0003091 | renal water homeostasis | Biological process | 2 | 12 | 172 | 13692 | 13.2674418604651 | 0.00953291437191142 | 0.139307655354866 | 2.02077430795061 | AQP1//WFS1 |
| GO:0006825 | copper ion transport | Biological process | 2 | 12 | 172 | 13692 | 13.2674418604651 | 0.00953291437191142 | 0.139307655354866 | 2.02077430795061 | ATP7A//CP |
| GO:0014012 | peripheral nervous system axon regeneration | Biological process | 2 | 12 | 172 | 13692 | 13.2674418604651 | 0.00953291437191142 | 0.139307655354866 | 2.02077430795061 | TSPO//MMP2 |
| GO:0035456 | response to interferon-beta | Biological process | 2 | 12 | 172 | 13692 | 13.2674418604651 | 0.00953291437191142 | 0.139307655354866 | 2.02077430795061 | TLR3//PLSCR1 |
| GO:0042178 | xenobiotic catabolic process | Biological process | 2 | 12 | 172 | 13692 | 13.2674418604651 | 0.00953291437191142 | 0.139307655354866 | 2.02077430795061 | PON3//GSTM2 |
| GO:0042538 | hyperosmotic salinity response | Biological process | 2 | 12 | 172 | 13692 | 13.2674418604651 | 0.00953291437191142 | 0.139307655354866 | 2.02077430795061 | AQP1//TRPV4 |
| GO:0046886 | positive regulation of hormone biosynthetic process | Biological process | 2 | 12 | 172 | 13692 | 13.2674418604651 | 0.00953291437191142 | 0.139307655354866 | 2.02077430795061 | BMP6//IGF2 |
| GO:0070365 | hepatocyte differentiation | Biological process | 2 | 12 | 172 | 13692 | 13.2674418604651 | 0.00953291437191142 | 0.139307655354866 | 2.02077430795061 | MESP1//ANXA1 |
| GO:0071636 | positive regulation of transforming growth factor beta production | Biological process | 2 | 12 | 172 | 13692 | 13.2674418604651 | 0.00953291437191142 | 0.139307655354866 | 2.02077430795061 | PTGS2//XCL1 |
| GO:0032526 | response to retinoic acid | Biological process | 5 | 103 | 172 | 13692 | 3.8643034545044 | 0.00961143253151621 | 0.139988439263013 | 2.01721187828702 | AQP1//T//BMP6//IGFBP2//MMP2 |
| GO:0009966 | regulation of signal transduction | Biological process | 29 | 1477 | 172 | 13692 | 1.56298908850435 | 0.00983499372174282 | 0.142381978670503 | 2.00722591298037 | GHR//CD74//LPAR1//MDFIC//BMP6//BMP7//CDKN1C//MSX2//MSX1//SOSTDC1//TCF7L2//ACAP2//NTRK1//FOXJ1//TLR3//RASGRF2//VAV3//ARHGEF26//MESP1//CLCF1//S100A4//IGF2//IGFBP2//PRRX2//RGN//C1QTNF3//PLSCR1//DAB2//WNT2B |
| GO:0007610 | behavior | Biological process | 14 | 552 | 172 | 13692 | 2.01895854398382 | 0.00984072525938923 | 0.142381978670503 | 2.00697289297683 | MDK//XCL1//NTRK1//PTGS2//IGF2//ATP7A//NMB//WFS1//ACE//PTGDS//TSPO//RGD1560691//LPAR1//CD74 |
| GO:0002377 | immunoglobulin production | Biological process | 4 | 67 | 172 | 13692 | 4.75251648733079 | 0.00997347285038513 | 0.143827976895028 | 2.00115359022023 | RT1-BB//RT1-DB1//XCL1//CLCF1 |
| GO:0002718 | regulation of cytokine production involved in immune response | Biological process | 3 | 36 | 172 | 13692 | 6.63372093023256 | 0.0102633640891532 | 0.146086325217038 | 1.98871026438131 | XCL1//APOA2//CD74 |
| GO:0010977 | negative regulation of neuron projection development | Biological process | 3 | 36 | 172 | 13692 | 6.63372093023256 | 0.0102633640891532 | 0.146086325217038 | 1.98871026438131 | SPP1//TRPV4//LPAR1 |
| GO:0031103 | axon regeneration | Biological process | 3 | 36 | 172 | 13692 | 6.63372093023256 | 0.0102633640891532 | 0.146086325217038 | 1.98871026438131 | TSPO//MMP2//SPP1 |
| GO:0090130 | tissue migration | Biological process | 3 | 36 | 172 | 13692 | 6.63372093023256 | 0.0102633640891532 | 0.146086325217038 | 1.98871026438131 | T//MESP1//AQP1 |
| GO:0018958 | phenol-containing compound metabolic process | Biological process | 4 | 68 | 172 | 13692 | 4.68262653898769 | 0.0104952859533616 | 0.148423656837217 | 1.97900572418256 | ATP7A//SLC5A5//TTR//HDC |
| GO:0021782 | glial cell development | Biological process | 4 | 68 | 172 | 13692 | 4.68262653898769 | 0.0104952859533616 | 0.148423656837217 | 1.97900572418256 | CD9//TCF7L2//LAMA2//NDRG1 |
| GO:0050778 | positive regulation of immune response | Biological process | 8 | 239 | 172 | 13692 | 2.66459083390094 | 0.0106873931595761 | 0.15065444248097 | 1.97112821382527 | SERPING1//XCL1//C6//TLR3//RT1-BB//CD74//PLSCR1//VAV3 |
| GO:0045596 | negative regulation of cell differentiation | Biological process | 11 | 393 | 172 | 13692 | 2.22812000710101 | 0.0108654081515945 | 0.151304408465539 | 1.96395395506311 | FOXJ1//TCF7L2//MESP1//CD74//MSX2//ZFHX3//BMP7//LMX1A//SPP1//MSX1//TSPO |
| GO:0019218 | regulation of steroid metabolic process | Biological process | 4 | 69 | 172 | 13692 | 4.61476238624874 | 0.0110344511863536 | 0.151304408465539 | 1.95724926220014 | TSPO//APOA2//BMP6//IGF2 |
| GO:0045216 | cell-cell junction organization | Biological process | 4 | 69 | 172 | 13692 | 4.61476238624874 | 0.0110344511863536 | 0.151304408465539 | 1.95724926220014 | TRPV4//CD9//CLDN19//CLDN1 |
| GO:0051100 | negative regulation of binding | Biological process | 4 | 69 | 172 | 13692 | 4.61476238624874 | 0.0110344511863536 | 0.151304408465539 | 1.95724926220014 | DAB2//PLSCR1//MSX1//MSX2 |
| GO:0051928 | positive regulation of calcium ion transport | Biological process | 4 | 69 | 172 | 13692 | 4.61476238624874 | 0.0110344511863536 | 0.151304408465539 | 1.95724926220014 | STIM2//XCL1//TSPO//WFS1 |
| GO:0061025 | membrane fusion | Biological process | 4 | 69 | 172 | 13692 | 4.61476238624874 | 0.0110344511863536 | 0.151304408465539 | 1.95724926220014 | VAV3//CD9//ANXA1//ANXA2 |
| GO:0007043 | cell-cell junction assembly | Biological process | 3 | 37 | 172 | 13692 | 6.45443117536141 | 0.0110675465405474 | 0.151304408465539 | 1.95594864307756 | CD9//CLDN19//TRPV4 |
| GO:0003203 | endocardial cushion morphogenesis | Biological process | 2 | 13 | 172 | 13692 | 12.2468694096601 | 0.0111738346062404 | 0.151304408465539 | 1.95179776130311 | MSX2//MSX1 |
| GO:0021542 | dentate gyrus development | Biological process | 2 | 13 | 172 | 13692 | 12.2468694096601 | 0.0111738346062404 | 0.151304408465539 | 1.95179776130311 | MDK//LMX1A |
| GO:0032781 | positive regulation of ATPase activity | Biological process | 2 | 13 | 172 | 13692 | 12.2468694096601 | 0.0111738346062404 | 0.151304408465539 | 1.95179776130311 | PLSCR1//RGN |
| GO:0016053 | organic acid biosynthetic process | Biological process | 8 | 241 | 172 | 13692 | 2.64247804689762 | 0.0111990897816553 | 0.151304408465539 | 1.95081727365735 | PTGDS//CD74//PTGS2//ABHD5//PLSCR1//ELOVL7//RGN//ANXA1 |
| GO:0046394 | carboxylic acid biosynthetic process | Biological process | 8 | 241 | 172 | 13692 | 2.64247804689762 | 0.0111990897816553 | 0.151304408465539 | 1.95081727365735 | PTGDS//CD74//PTGS2//ABHD5//PLSCR1//ELOVL7//RGN//ANXA1 |
| GO:0048729 | tissue morphogenesis | Biological process | 12 | 449 | 172 | 13692 | 2.1275185166002 | 0.0112120977699046 | 0.151304408465539 | 1.9503131238314 | BMP7//T//FOXJ1//MESP1//MSX2//MSX1//ATP7A//SLC12A2//WNT2B//SEMA3C//MMP2 |
| GO:0034599 | cellular response to oxidative stress | Biological process | 5 | 107 | 172 | 13692 | 3.71984351227994 | 0.0112166817407163 | 0.151304408465539 | 1.95013560251282 | ATP7A//AQP1//ANXA1//LCN2//MGST1 |
| GO:0051924 | regulation of calcium ion transport | Biological process | 6 | 149 | 172 | 13692 | 3.20555642266271 | 0.0113076283662453 | 0.151607279659038 | 1.94662847336444 | DHRS7C//STIM2//XCL1//PTGS2//TSPO//WFS1 |
| GO:0035239 | tube morphogenesis | Biological process | 9 | 291 | 172 | 13692 | 2.46199952049868 | 0.0113082984599693 | 0.151607279659038 | 1.94660273769515 | T//FOXJ1//MESP1//MGP//MMP14//BMP7//MSX2//SLC12A2//WNT2B |
| GO:0007155 | cell adhesion | Biological process | 16 | 679 | 172 | 13692 | 1.87580915847519 | 0.0115552453968518 | 0.153608576217182 | 1.93722082704623 | CDH19//CDHR4//MMP2//SPP1//COL8A1//CDH3//CLDN1//LAMA2//BMP7//VAV3//MMP14//CD9//OMD//TGFBI//CPXM2//AEBP1 |
| GO:0000041 | transition metal ion transport | Biological process | 4 | 70 | 172 | 13692 | 4.54883720930233 | 0.0115911726694999 | 0.153608576217182 | 1.93587262459463 | ATP7A//CP//LCN2//SCARA5 |
| GO:0007368 | determination of left/right symmetry | Biological process | 4 | 70 | 172 | 13692 | 4.54883720930233 | 0.0115911726694999 | 0.153608576217182 | 1.93587262459463 | FOXJ1//MESP1//CCDC40//T |
| GO:0050790 | regulation of catalytic activity | Biological process | 23 | 1108 | 172 | 13692 | 1.65244311980522 | 0.0115977278120181 | 0.153608576217182 | 1.93562708795939 | GHR//CD74//LPAR1//GADD45A//C6//MDFIC//SERPINB1A//SERPING1//PCOLCE//MMP14//ACAP2//NTRK1//RGN//FOXJ1//CDKN1C//IGF2//ATP7A//VAV3//AQP1//BMP7//ABHD5//APOA2//PLSCR1 |
| GO:0051128 | regulation of cellular component organization | Biological process | 23 | 1110 | 172 | 13692 | 1.64946574481458 | 0.0118327839178696 | 0.156249773180543 | 1.92691306619783 | IGFBP2//LMX1A//SPTA1//AQP1//APOA2//NTRK1//RGD1560691//TRPV4//LPAR1//CXCL16//DAB2//MSX1//RERG//BAIAP2L1//ANXA1//ANXA2//LCN2//TCF7L2//CD14//BMP7//IGF2//SPP1//MMP14 |
| GO:0019216 | regulation of lipid metabolic process | Biological process | 7 | 196 | 172 | 13692 | 2.84302325581395 | 0.0119339779119228 | 0.157112790287897 | 1.92321477030782 | TSPO//APOA2//ABHD5//ANXA1//BMP6//VAV3//IGF2 |
| GO:0022610 | biological adhesion | Biological process | 16 | 682 | 172 | 13692 | 1.86755779854054 | 0.01201239215245 | 0.157671638312398 | 1.92037049841236 | CD9//SPP1//OMD//TGFBI//CPXM2//AEBP1//LAMA2//CDH19//CDHR4//MMP2//COL8A1//CDH3//CLDN1//BMP7//VAV3//MMP14 |
| GO:0048678 | response to axon injury | Biological process | 4 | 71 | 172 | 13692 | 4.48476907959384 | 0.0121656485046804 | 0.159206576252295 | 1.91486473552088 | TSPO//MMP2//SPP1//NTRK1 |
| GO:0001763 | morphogenesis of a branching structure | Biological process | 7 | 197 | 172 | 13692 | 2.82859166568292 | 0.012246646071376 | 0.159789572550334 | 1.91198283310605 | MGP//MMP14//BMP7//MSX2//SLC12A2//WNT2B//SEMA3C |
| GO:0042113 | B cell activation | Biological process | 6 | 152 | 172 | 13692 | 3.14228886168911 | 0.012386995712165 | 0.161141214249648 | 1.90703401273932 | RT1-BB//NTRK1//CLCF1//CD74//VAV3//FOXJ1 |
| GO:0006576 | cellular biogenic amine metabolic process | Biological process | 5 | 110 | 172 | 13692 | 3.6183932346723 | 0.0125343674218783 | 0.16167767177583 | 1.90189757890719 | HDC//ATP7A//APOA2//ENPP2//PON1 |
| GO:0006874 | cellular calcium ion homeostasis | Biological process | 8 | 246 | 172 | 13692 | 2.58876914350539 | 0.0125574969548462 | 0.16167767177583 | 1.90109691848346 | TRPV4//NMB//DHRS7C//WFS1//XCL1//LPAR1//RGN//STIM2 |
| GO:0002009 | morphogenesis of an epithelium | Biological process | 10 | 348 | 172 | 13692 | 2.28748997594226 | 0.0125680446954656 | 0.16167767177583 | 1.90073228349326 | T//FOXJ1//MESP1//MSX2//SLC12A2//BMP7//WNT2B//SEMA3C//MMP2 |
| GO:0060284 | regulation of cell development | Biological process | 13 | 512 | 172 | 13692 | 2.02121184593023 | 0.0125757495610306 | 0.16167767177583 | 1.90046611994855 | NTRK1//RGD1560691//TRPV4//LPAR1//TCF7L2//ZFHX3//BMP7//LMX1A//BMP6//SPP1//CLCF1//ACE//TSPO |
| GO:0010171 | body morphogenesis | Biological process | 3 | 39 | 172 | 13692 | 6.12343470483005 | 0.0127824504449648 | 0.162880789423847 | 1.89338588227207 | MMP2//MSX1//TCF7L2 |
| GO:0035137 | hindlimb morphogenesis | Biological process | 3 | 39 | 172 | 13692 | 6.12343470483005 | 0.0127824504449648 | 0.162880789423847 | 1.89338588227207 | MSX2//MSX1//ATP7A |
| GO:0072376 | protein activation cascade | Biological process | 3 | 39 | 172 | 13692 | 6.12343470483005 | 0.0127824504449648 | 0.162880789423847 | 1.89338588227207 | SERPING1//C6//F5 |
| GO:0010042 | response to manganese ion | Biological process | 2 | 14 | 172 | 13692 | 11.3720930232558 | 0.0129294057298127 | 0.162880789423847 | 1.88842143600083 | TSPO//PTGS2 |
| GO:0022011 | myelination in peripheral nervous system | Biological process | 2 | 14 | 172 | 13692 | 11.3720930232558 | 0.0129294057298127 | 0.162880789423847 | 1.88842143600083 | NDRG1//LAMA2 |
| GO:0032292 | peripheral nervous system axon ensheathment | Biological process | 2 | 14 | 172 | 13692 | 11.3720930232558 | 0.0129294057298127 | 0.162880789423847 | 1.88842143600083 | LAMA2//NDRG1 |
| GO:0071392 | cellular response to estradiol stimulus | Biological process | 2 | 14 | 172 | 13692 | 11.3720930232558 | 0.0129294057298127 | 0.162880789423847 | 1.88842143600083 | MSX2//MMP2 |
| GO:0019228 | regulation of action potential in neuron | Biological process | 5 | 111 | 172 | 13692 | 3.585795097423 | 0.0129959274817361 | 0.163249702234759 | 1.88619272071654 | LAMA2//CD9//TCF7L2//NDRG1//LPAR1 |
| GO:0006518 | peptide metabolic process | Biological process | 4 | 73 | 172 | 13692 | 4.36189869385155 | 0.0133686253394277 | 0.165559473130088 | 1.87391324780117 | GSTM2//MGST1//BDH2//ACE |
| GO:0019882 | antigen processing and presentation | Biological process | 4 | 73 | 172 | 13692 | 4.36189869385155 | 0.0133686253394277 | 0.165559473130088 | 1.87391324780117 | MR1//RT1-BB//CD74//RT1-DB1 |
| GO:0034614 | cellular response to reactive oxygen species | Biological process | 4 | 73 | 172 | 13692 | 4.36189869385155 | 0.0133686253394277 | 0.165559473130088 | 1.87391324780117 | ATP7A//AQP1//ANXA1//LCN2 |
| GO:0045995 | regulation of embryonic development | Biological process | 4 | 73 | 172 | 13692 | 4.36189869385155 | 0.0133686253394277 | 0.165559473130088 | 1.87391324780117 | BMP7//MESP1//WNT2B//LAMA2 |
| GO:0050921 | positive regulation of chemotaxis | Biological process | 4 | 73 | 172 | 13692 | 4.36189869385155 | 0.0133686253394277 | 0.165559473130088 | 1.87391324780117 | XCL1//LPAR1//CD74//RGD1560691 |
| GO:0008610 | lipid biosynthetic process | Biological process | 11 | 406 | 172 | 13692 | 2.15677626303128 | 0.0135912871763768 | 0.167842825299256 | 1.86673941102866 | PTGDS//CD74//PTGS2//ABHD5//APOA2//PLSCR1//TSPO//ELOVL7//ANXA1//BMP6//IGF2 |
| GO:0071453 | cellular response to oxygen levels | Biological process | 4 | 74 | 172 | 13692 | 4.30295411690761 | 0.0139974921577928 | 0.171155557364815 | 1.85394976714988 | AQP1//PTGS2//NDRG1//LPAR1 |
| GO:0002682 | regulation of immune system process | Biological process | 15 | 635 | 172 | 13692 | 1.8804248306171 | 0.0140123890044304 | 0.171155557364815 | 1.85348781453644 | SERPING1//XCL1//C6//TLR3//RT1-BB//CD74//FOXJ1//CLCF1//APOA2//VAV3//PLSCR1//SPTA1//IGF2//IGFBP2//RGD1560691 |
| GO:0001822 | kidney development | Biological process | 7 | 203 | 172 | 13692 | 2.74498797113071 | 0.014248161233466 | 0.171155557364815 | 1.84624117900183 | BMP7//AQP1//WNT2B//ACE//BMP6//ADAMTS1//WFS1 |
| GO:0030278 | regulation of ossification | Biological process | 6 | 157 | 172 | 13692 | 3.04221596800474 | 0.0143438481373167 | 0.171155557364815 | 1.84333432142932 | MGP//OMD//CREB3L1//BMP6//BMP7//MSX2 |
| GO:0042445 | hormone metabolic process | Biological process | 6 | 157 | 172 | 13692 | 3.04221596800474 | 0.0143438481373167 | 0.171155557364815 | 1.84333432142932 | SLC5A5//BMP6//TCF7L2//TTR//ACE//IGF2 |
| GO:0048754 | branching morphogenesis of a tube | Biological process | 6 | 157 | 172 | 13692 | 3.04221596800474 | 0.0143438481373167 | 0.171155557364815 | 1.84333432142932 | MSX2//SLC12A2//WNT2B//MGP//MMP14//BMP7 |
| GO:0044255 | cellular lipid metabolic process | Biological process | 15 | 637 | 172 | 13692 | 1.8745208280092 | 0.014382492446069 | 0.171155557364815 | 1.84216584540129 | PTGDS//CD74//PTGS2//GHR//ABHD5//HADHA//BDH2//APOA2//PLSCR1//ELOVL7//ANXA1//ENPP2//TTR//CES1D//PON1 |
| GO:0034330 | cell junction organization | Biological process | 5 | 114 | 172 | 13692 | 3.49143206854345 | 0.0144492776850675 | 0.171155557364815 | 1.84015386261194 | TRPV4//CD9//CLDN19//CLDN1//MMP14 |
| GO:0051336 | regulation of hydrolase activity | Biological process | 14 | 579 | 172 | 13692 | 1.92481021809857 | 0.014461976919774 | 0.171155557364815 | 1.83977233590287 | C6//SERPINB1A//SERPING1//PCOLCE//MMP14//ACAP2//NTRK1//RGN//FOXJ1//VAV3//AQP1//ABHD5//APOA2//PLSCR1 |
| GO:0032787 | monocarboxylic acid metabolic process | Biological process | 10 | 356 | 172 | 13692 | 2.23608570681996 | 0.0145353348604633 | 0.171155557364815 | 1.83757495863891 | PTGDS//CD74//PTGS2//GHR//ABHD5//HADHA//BDH2//ELOVL7//UGT1A6//ANXA1 |
| GO:0006879 | cellular iron ion homeostasis | Biological process | 3 | 41 | 172 | 13692 | 5.82473057288712 | 0.0146408992563073 | 0.171155557364815 | 1.83443224772721 | LCN2//CP//SCARA5 |
| GO:0015909 | long-chain fatty acid transport | Biological process | 3 | 41 | 172 | 13692 | 5.82473057288712 | 0.0146408992563073 | 0.171155557364815 | 1.83443224772721 | ACE//ANXA1//NMB |
| GO:0042130 | negative regulation of T cell proliferation | Biological process | 3 | 41 | 172 | 13692 | 5.82473057288712 | 0.0146408992563073 | 0.171155557364815 | 1.83443224772721 | XCL1//FOXJ1//RT1-BB |
| GO:0061371 | determination of heart left/right asymmetry | Biological process | 3 | 41 | 172 | 13692 | 5.82473057288712 | 0.0146408992563073 | 0.171155557364815 | 1.83443224772721 | FOXJ1//MESP1//T |
| GO:0071695 | anatomical structure maturation | Biological process | 3 | 41 | 172 | 13692 | 5.82473057288712 | 0.0146408992563073 | 0.171155557364815 | 1.83443224772721 | MMP2//MSX2//PTGS2 |
| GO:0007565 | female pregnancy | Biological process | 6 | 158 | 172 | 13692 | 3.02296143656167 | 0.0147596306896149 | 0.171155557364815 | 1.83092450914366 | PTGS2//MMP2//SOSTDC1//FBLN1//IGF2//IGFBP2 |
| GO:0001542 | ovulation from ovarian follicle | Biological process | 2 | 15 | 172 | 13692 | 10.6139534883721 | 0.014796522865252 | 0.171155557364815 | 1.82984033040601 | ADAMTS1//MMP2 |
| GO:0015695 | organic cation transport | Biological process | 2 | 15 | 172 | 13692 | 10.6139534883721 | 0.014796522865252 | 0.171155557364815 | 1.82984033040601 | SLC22A8//SLC44A1 |
| GO:0032352 | positive regulation of hormone metabolic process | Biological process | 2 | 15 | 172 | 13692 | 10.6139534883721 | 0.014796522865252 | 0.171155557364815 | 1.82984033040601 | BMP6//IGF2 |
| GO:0048820 | hair follicle maturation | Biological process | 2 | 15 | 172 | 13692 | 10.6139534883721 | 0.014796522865252 | 0.171155557364815 | 1.82984033040601 | MSX2//PTGS2 |
| GO:0070633 | transepithelial transport | Biological process | 2 | 15 | 172 | 13692 | 10.6139534883721 | 0.014796522865252 | 0.171155557364815 | 1.82984033040601 | SLC12A2//AQP1 |
| GO:0072337 | modified amino acid transport | Biological process | 2 | 15 | 172 | 13692 | 10.6139534883721 | 0.014796522865252 | 0.171155557364815 | 1.82984033040601 | SLC22A8//SLCO1A5 |
| GO:0090030 | regulation of steroid hormone biosynthetic process | Biological process | 2 | 15 | 172 | 13692 | 10.6139534883721 | 0.014796522865252 | 0.171155557364815 | 1.82984033040601 | BMP6//IGF2 |
| GO:2000677 | regulation of transcription regulatory region DNA binding | Biological process | 2 | 15 | 172 | 13692 | 10.6139534883721 | 0.014796522865252 | 0.171155557364815 | 1.82984033040601 | MSX2//MSX1 |
| GO:0055074 | calcium ion homeostasis | Biological process | 8 | 254 | 172 | 13692 | 2.50723310748947 | 0.0149776551979275 | 0.172794843125564 | 1.82455617157039 | RGN//TRPV4//STIM2//NMB//DHRS7C//WFS1//XCL1//LPAR1 |
| GO:0008406 | gonad development | Biological process | 7 | 206 | 172 | 13692 | 2.70501241815308 | 0.0153322051460556 | 0.176420964200283 | 1.81439537848605 | MMP2//MMP14//ADAMTS1//WNT2B//TLR3//MGST1//NTRK1 |
| GO:0045595 | regulation of cell differentiation | Biological process | 20 | 947 | 172 | 13692 | 1.68119643427224 | 0.0154069317558155 | 0.176546464298292 | 1.81228384111244 | NTRK1//RGD1560691//TRPV4//LPAR1//FOXJ1//TCF7L2//MESP1//CD74//GHR//BMP7//MSX2//BMP6//ZFHX3//LMX1A//SPP1//CLCF1//ACE//MSX1//TSPO//PTGS2 |
| GO:0022612 | gland morphogenesis | Biological process | 5 | 116 | 172 | 13692 | 3.43123496391339 | 0.0154765564482192 | 0.176546464298292 | 1.81032566393098 | BMP7//MSX2//SLC12A2//SEMA3C//MMP2 |
| GO:0044106 | cellular amine metabolic process | Biological process | 5 | 116 | 172 | 13692 | 3.43123496391339 | 0.0154765564482192 | 0.176546464298292 | 1.81032566393098 | HDC//ATP7A//APOA2//ENPP2//PON1 |
| GO:0010740 | positive regulation of intracellular protein kinase cascade | Biological process | 10 | 360 | 172 | 13692 | 2.21124031007752 | 0.0156014614416266 | 0.176546464298292 | 1.80683471790797 | CLCF1//GHR//S100A4//LPAR1//TLR3//IGF2//C1QTNF3//TCF7L2//CD74//NTRK1 |
| GO:0002367 | cytokine production involved in immune response | Biological process | 3 | 42 | 172 | 13692 | 5.68604651162791 | 0.0156244146518833 | 0.176546464298292 | 1.8061962439499 | XCL1//APOA2//CD74 |
| GO:0031102 | neuron projection regeneration | Biological process | 3 | 42 | 172 | 13692 | 5.68604651162791 | 0.0156244146518833 | 0.176546464298292 | 1.8061962439499 | TSPO//MMP2//SPP1 |
| GO:0048468 | cell development | Biological process | 26 | 1333 | 172 | 13692 | 1.55267886739127 | 0.0156250064205605 | 0.176546464298292 | 1.80617979552543 | DAB2//SEMA3C//MSX1//MSX2//BMP6//NTRK1//BMP7//LMX1A//LAMA2//RGD1560691//TRPV4//LPAR1//CD9//TCF7L2//TSPO//MMP2//ATP7A//NDRG1//FSCN2//CDKN1C//KCNIP2//ZFHX3//SPP1//CLCF1//DCDC2//ACE |
| GO:0001655 | urogenital system development | Biological process | 8 | 257 | 172 | 13692 | 2.47796579495068 | 0.0159673280269662 | 0.179028748959801 | 1.79676775262993 | BMP7//ACE//BMP6//ADAMTS1//WFS1//MMP2//AQP1//WNT2B |
| GO:0009855 | determination of bilateral symmetry | Biological process | 4 | 77 | 172 | 13692 | 4.1353065539112 | 0.0159956724422218 | 0.179028748959801 | 1.79599749788636 | FOXJ1//MESP1//CCDC40//T |
| GO:0016050 | vesicle organization | Biological process | 4 | 77 | 172 | 13692 | 4.1353065539112 | 0.0159956724422218 | 0.179028748959801 | 1.79599749788636 | VAV3//ANXA1//ANXA2//AQP1 |
| GO:0006633 | fatty acid biosynthetic process | Biological process | 5 | 117 | 172 | 13692 | 3.40190816935003 | 0.0160080450712231 | 0.179028748959801 | 1.79566170159849 | PTGDS//CD74//PTGS2//ELOVL7//ANXA1 |
| GO:0055114 | oxidation-reduction process | Biological process | 10 | 363 | 172 | 13692 | 2.19296559677109 | 0.0164385753100391 | 0.18245425035557 | 1.78413582437503 | COX8B//CYBRD1//HADHA//BDH2//MTFR1//APOA2//IGF2//FMO2//MGST1//DHRS7C |
| GO:0045597 | positive regulation of cell differentiation | Biological process | 12 | 474 | 172 | 13692 | 2.01530762437445 | 0.016564930183508 | 0.18245425035557 | 1.78081039020648 | TCF7L2//CD74//BMP6//ZFHX3//BMP7//MSX2//CLCF1//ACE//MESP1//TSPO//PTGS2//GHR |
| GO:0042439 | ethanolamine-containing compound metabolic process | Biological process | 3 | 43 | 172 | 13692 | 5.55381287182261 | 0.0166443201170955 | 0.18245425035557 | 1.77873394009423 | APOA2//ENPP2//PON1 |
| GO:0009799 | specification of symmetry | Biological process | 4 | 78 | 172 | 13692 | 4.08228980322004 | 0.0166994638869043 | 0.18245425035557 | 1.77729747104981 | FOXJ1//MESP1//CCDC40//T |
| GO:0015698 | inorganic anion transport | Biological process | 4 | 78 | 172 | 13692 | 4.08228980322004 | 0.0166994638869043 | 0.18245425035557 | 1.77729747104981 | TSPO//SLC4A2//SLC12A2//SLC5A5 |
| GO:0002823 | negative regulation of adaptive immune response based on somatic recombination of immune receptors built from immunoglobulin superfamily domains | Biological process | 2 | 16 | 172 | 13692 | 9.95058139534884 | 0.016772140258507 | 0.18245425035557 | 1.77541151442725 | FOXJ1//XCL1 |
| GO:0009713 | catechol-containing compound biosynthetic process | Biological process | 2 | 16 | 172 | 13692 | 9.95058139534884 | 0.016772140258507 | 0.18245425035557 | 1.77541151442725 | ATP7A//HDC |
| GO:0034312 | diol biosynthetic process | Biological process | 2 | 16 | 172 | 13692 | 9.95058139534884 | 0.016772140258507 | 0.18245425035557 | 1.77541151442725 | ATP7A//HDC |
| GO:0042423 | catecholamine biosynthetic process | Biological process | 2 | 16 | 172 | 13692 | 9.95058139534884 | 0.016772140258507 | 0.18245425035557 | 1.77541151442725 | ATP7A//HDC |
| GO:0042730 | fibrinolysis | Biological process | 2 | 16 | 172 | 13692 | 9.95058139534884 | 0.016772140258507 | 0.18245425035557 | 1.77541151442725 | ANXA2//SERPING1 |
| GO:0060317 | cardiac epithelial to mesenchymal transition | Biological process | 2 | 16 | 172 | 13692 | 9.95058139534884 | 0.016772140258507 | 0.18245425035557 | 1.77541151442725 | MSX2//MSX1 |
| GO:0048519 | negative regulation of biological process | Biological process | 46 | 2719 | 172 | 13692 | 1.34675025873055 | 0.0169353235749473 | 0.183773412258834 | 1.77120650108775 | MSX2//MSX1//FOXJ1//CDKN1C//ZFHX3//MOSPD1//TCF7L2//SERPING1//ANXA1//XCL1//APOA2//CD74//GADD45A//MMP2//CD9//PTGS2//NTRK1//BMP7//RERG//DHRS7C//ABHD5//TRPV4//LPAR1//DAB2//MESP1//MDFIC//SOSTDC1//STIM2//TSPO//RT1-BB//ANXA2//AQP1//WFS1//KRT18//ATP7A//CLCF1//PLSCR1//LMX1A//C1QTNF3//PTPN3//NMB//OGN//SPP1//MMP14//RGD1305645 |
| GO:0023051 | regulation of signaling | Biological process | 32 | 1744 | 172 | 13692 | 1.46063580115212 | 0.0170598528907856 | 0.184667642156059 | 1.7680247181272 | GHR//CD74//LPAR1//MDFIC//BMP6//BMP7//NMB//CDKN1C//MSX2//MSX1//SOSTDC1//TCF7L2//PTGS2//LAMA2//ACAP2//NTRK1//FOXJ1//TLR3//RASGRF2//VAV3//ARHGEF26//MESP1//CLCF1//S100A4//IGF2//IGFBP2//PRRX2//RGN//C1QTNF3//PLSCR1//DAB2//WNT2B |
| GO:0071345 | cellular response to cytokine stimulus | Biological process | 8 | 261 | 172 | 13692 | 2.43998930767175 | 0.0173595551145425 | 0.187205123553672 | 1.76046140898528 | CD74//KRT18//TLR3//RT1-BB//MMP2//LCN2//XCL1//PLSCR1 |
| GO:0015718 | monocarboxylic acid transport | Biological process | 4 | 79 | 172 | 13692 | 4.0306152487489 | 0.0174223746372943 | 0.187205123553672 | 1.75889265176299 | SLCO1A5//ACE//ANXA1//NMB |
| GO:0070555 | response to interleukin-1 | Biological process | 4 | 79 | 172 | 13692 | 4.0306152487489 | 0.0174223746372943 | 0.187205123553672 | 1.75889265176299 | MMP2//LCN2//GHR//ANXA1 |
| GO:0051130 | positive regulation of cellular component organization | Biological process | 12 | 478 | 172 | 13692 | 1.99844312542571 | 0.0175755965080899 | 0.187881947240433 | 1.75508992629246 | AQP1//NTRK1//RGD1560691//BAIAP2L1//TRPV4//ANXA1//ANXA2//LCN2//TCF7L2//CD14//IGF2//BMP7 |
| GO:0030098 | lymphocyte differentiation | Biological process | 7 | 212 | 172 | 13692 | 2.62845546292233 | 0.0176744936002771 | 0.187881947240433 | 1.75265302051584 | RT1-BB//NTRK1//CLCF1//FOXJ1//ATP7A//CD74//ANXA1 |
| GO:0072001 | renal system development | Biological process | 7 | 212 | 172 | 13692 | 2.62845546292233 | 0.0176744936002771 | 0.187881947240433 | 1.75265302051584 | BMP7//ACE//BMP6//ADAMTS1//WFS1//AQP1//WNT2B |
| GO:0060350 | endochondral bone morphogenesis | Biological process | 3 | 44 | 172 | 13692 | 5.42758985200846 | 0.0177007327263559 | 0.187881947240433 | 1.75200875553499 | BMP6//MSX2//GHR |
| GO:0072503 | cellular divalent inorganic cation homeostasis | Biological process | 8 | 262 | 172 | 13692 | 2.43067637138292 | 0.0177208672464272 | 0.187881947240433 | 1.75151502787101 | RGN//TRPV4//STIM2//NMB//DHRS7C//WFS1//XCL1//LPAR1 |
| GO:0006629 | lipid metabolic process | Biological process | 19 | 898 | 172 | 13692 | 1.68428549230849 | 0.0177425014045482 | 0.187881947240433 | 1.75098515171906 | PTGDS//CD74//PTGS2//GHR//ABHD5//HADHA//BDH2//APOA2//PLSCR1//TSPO//PON1//ELOVL7//ANXA1//BMP6//ENPP2//TTR//VAV3//CES1D//IGF2 |
| GO:0043085 | positive regulation of catalytic activity | Biological process | 15 | 655 | 172 | 13692 | 1.82300727853719 | 0.018066013533964 | 0.190846755019032 | 1.74313766875633 | GHR//CD74//LPAR1//C6//MDFIC//PCOLCE//MMP14//NTRK1//RGN//FOXJ1//VAV3//ATP7A//ABHD5//PLSCR1//IGF2 |
| GO:0007292 | female gamete generation | Biological process | 4 | 80 | 172 | 13692 | 3.98023255813953 | 0.01816454762604 | 0.191426386520575 | 1.74077541343358 | ADAMTS1//MMP2//PTGS2//MEI4 |
| GO:0006954 | inflammatory response | Biological process | 10 | 371 | 172 | 13692 | 2.14567792891619 | 0.0188340434467522 | 0.192958391740983 | 1.72505643207587 | SERPING1//C6//APOA2//ANXA1//PLSCR1//PTGS2//XCL1//ACE//SPP1//CD14 |
| GO:0048762 | mesenchymal cell differentiation | Biological process | 5 | 122 | 172 | 13692 | 3.26248570339306 | 0.0188482294942727 | 0.192958391740983 | 1.72472943893196 | SEMA3C//MSX1//MSX2//BMP7 |
| GO:0008347 | glial cell migration | Biological process | 2 | 17 | 172 | 13692 | 9.36525307797537 | 0.0188532702975673 | 0.192958391740983 | 1.72461330598869 | MMP14//TSPO |
| GO:0009415 | response to water stimulus | Biological process | 2 | 17 | 172 | 13692 | 9.36525307797537 | 0.0188532702975673 | 0.192958391740983 | 1.72461330598869 | CD9//NTRK1 |
| GO:0010893 | positive regulation of steroid biosynthetic process | Biological process | 2 | 17 | 172 | 13692 | 9.36525307797537 | 0.0188532702975673 | 0.192958391740983 | 1.72461330598869 | BMP6//IGF2 |
| GO:0014044 | Schwann cell development | Biological process | 2 | 17 | 172 | 13692 | 9.36525307797537 | 0.0188532702975673 | 0.192958391740983 | 1.72461330598869 | LAMA2//NDRG1 |
| GO:0042346 | positive regulation of NF-kappaB import into nucleus | Biological process | 2 | 17 | 172 | 13692 | 9.36525307797537 | 0.0188532702975673 | 0.192958391740983 | 1.72461330598869 | PTGS2//TLR3 |
| GO:0050891 | multicellular organismal water homeostasis | Biological process | 2 | 17 | 172 | 13692 | 9.36525307797537 | 0.0188532702975673 | 0.192958391740983 | 1.72461330598869 | WFS1//AQP1 |
| GO:0060572 | morphogenesis of an epithelial bud | Biological process | 2 | 17 | 172 | 13692 | 9.36525307797537 | 0.0188532702975673 | 0.192958391740983 | 1.72461330598869 | WNT2B//BMP7 |
| GO:0061437 | renal system vasculature development | Biological process | 2 | 17 | 172 | 13692 | 9.36525307797537 | 0.0188532702975673 | 0.192958391740983 | 1.72461330598869 | BMP7//AQP1 |
| GO:0061440 | kidney vasculature development | Biological process | 2 | 17 | 172 | 13692 | 9.36525307797537 | 0.0188532702975673 | 0.192958391740983 | 1.72461330598869 | BMP7//AQP1 |
| GO:0072012 | glomerulus vasculature development | Biological process | 2 | 17 | 172 | 13692 | 9.36525307797537 | 0.0188532702975673 | 0.192958391740983 | 1.72461330598869 | BMP7//AQP1 |
| GO:0050727 | regulation of inflammatory response | Biological process | 6 | 167 | 172 | 13692 | 2.86004734716613 | 0.0188852232610349 | 0.192958391740983 | 1.72387787657846 | SERPING1//C6//ANXA1//PTGS2//ACE//XCL1 |
| GO:0050864 | regulation of B cell activation | Biological process | 4 | 81 | 172 | 13692 | 3.93109388458226 | 0.0189261196278793 | 0.192958391740983 | 1.7229384191554 | CD74//VAV3//CLCF1//FOXJ1 |
| GO:0070838 | divalent metal ion transport | Biological process | 8 | 266 | 172 | 13692 | 2.39412484700122 | 0.0192203763733447 | 0.19550378195068 | 1.71623811223114 | TRPV4//DHRS7C//MAGT1//STIM2//XCL1//PTGS2//TSPO//WFS1 |
| GO:0031175 | neuron projection development | Biological process | 13 | 543 | 172 | 13692 | 1.90582037774637 | 0.0195244815557279 | 0.19785323911057 | 1.70942048935724 | NTRK1//BMP7//LMX1A//SEMA3C//LAMA2//RGD1560691//TRPV4//LPAR1//TSPO//MMP2//SPP1//ATP7A//DCDC2 |
| GO:0032989 | cellular component morphogenesis | Biological process | 17 | 783 | 172 | 13692 | 1.72832575960082 | 0.0195416178227365 | 0.19785323911057 | 1.70903948450659 | MSX1//DAB2//MSX2//NTRK1//BMP7//LMX1A//SEMA3C//LAMA2//SPTA1//TSPO//MMP2//CD9//FOXJ1//SPP1//ATP7A//DCDC2 |
| GO:0045786 | negative regulation of cell cycle | Biological process | 8 | 267 | 172 | 13692 | 2.38515808727463 | 0.0196090223581144 | 0.198078235064455 | 1.70754405829857 | GADD45A//ZFHX3//TCF7L2//BMP7//PTPN3//RGD1305645//PTGS2 |
| GO:0030182 | neuron differentiation | Biological process | 18 | 846 | 172 | 13692 | 1.69371598218704 | 0.0197848800078934 | 0.199395204493344 | 1.70366657931596 | NTRK1//BMP7//LMX1A//SEMA3C//LAMA2//RGD1560691//TRPV4//LPAR1//TSPO//MMP2//ATP7A//FSCN2//CDKN1C//KCNIP2//ZFHX3//BMP6//SPP1//DCDC2 |
| GO:0007568 | aging | Biological process | 8 | 268 | 172 | 13692 | 2.37625824366539 | 0.0200032563606574 | 0.201133660286977 | 1.69889929911868 | RGD1305645//TSPO//CP//GSTM2//CTSC//IGFBP2//NTRK1//MMP2 |
| GO:0033189 | response to vitamin A | Biological process | 5 | 124 | 172 | 13692 | 3.20986496624156 | 0.0200714659664777 | 0.201357681457753 | 1.6974209066405 | BMP6//IGFBP2//MMP2//AQP1//T |
| GO:0030334 | regulation of cell migration | Biological process | 10 | 375 | 172 | 13692 | 2.12279069767442 | 0.0201239790650252 | 0.201423571280983 | 1.69628614314259 | XCL1//AQP1//ACE//CXCL16//RGD1560691//LPAR1//CD74//PTGS2//ENPP2//LAMA2 |
| GO:0000902 | cell morphogenesis | Biological process | 16 | 725 | 172 | 13692 | 1.75679230152366 | 0.0202660908177156 | 0.202383922881242 | 1.69323001549174 | DAB2//MSX1//MSX2//NTRK1//BMP7//LMX1A//SEMA3C//LAMA2//SPTA1//TSPO//MMP2//FOXJ1//SPP1//ATP7A//DCDC2 |
| GO:0072511 | divalent inorganic cation transport | Biological process | 8 | 269 | 172 | 13692 | 2.36742456989712 | 0.0204031181882062 | 0.203289250311582 | 1.69030345470916 | TRPV4//DHRS7C//MAGT1//STIM2//XCL1//PTGS2//TSPO//WFS1 |
| GO:0000187 | activation of MAPK activity | Biological process | 4 | 83 | 172 | 13692 | 3.8363687307369 | 0.0205079770398438 | 0.203870683316724 | 1.68807717745529 | MDFIC//GHR//CD74//LPAR1 |
| GO:0000413 | protein peptidyl-prolyl isomerization | Biological process | 2 | 18 | 172 | 13692 | 8.84496124031008 | 0.0210369825462769 | 0.206342391098066 | 1.67701655337193 | FKBP9//FKBP14 |
| GO:0002820 | negative regulation of adaptive immune response | Biological process | 2 | 18 | 172 | 13692 | 8.84496124031008 | 0.0210369825462769 | 0.206342391098066 | 1.67701655337193 | FOXJ1//XCL1 |
| GO:0002825 | regulation of T-helper 1 type immune response | Biological process | 2 | 18 | 172 | 13692 | 8.84496124031008 | 0.0210369825462769 | 0.206342391098066 | 1.67701655337193 | RT1-BB//XCL1 |
| GO:0050892 | intestinal absorption | Biological process | 2 | 18 | 172 | 13692 | 8.84496124031008 | 0.0210369825462769 | 0.206342391098066 | 1.67701655337193 | APOA2//SLCO1A5 |
| GO:0002690 | positive regulation of leukocyte chemotaxis | Biological process | 3 | 47 | 172 | 13692 | 5.08114794656111 | 0.0210898422273787 | 0.206342391098066 | 1.67592666919532 | XCL1//CD74//RGD1560691 |
| GO:0048284 | organelle fusion | Biological process | 3 | 47 | 172 | 13692 | 5.08114794656111 | 0.0210898422273787 | 0.206342391098066 | 1.67592666919532 | VAV3//ANXA1//ANXA2 |
| GO:0060322 | head development | Biological process | 3 | 47 | 172 | 13692 | 5.08114794656111 | 0.0210898422273787 | 0.206342391098066 | 1.67592666919532 | MMP2//MSX1//TCF7L2 |
| GO:0048667 | cell morphogenesis involved in neuron differentiation | Biological process | 10 | 378 | 172 | 13692 | 2.10594315245478 | 0.0211331509131003 | 0.206342391098066 | 1.67503574562859 | NTRK1//BMP7//LMX1A//SEMA3C//LAMA2//TSPO//MMP2//SPP1//ATP7A//DCDC2 |
| GO:0072507 | divalent inorganic cation homeostasis | Biological process | 8 | 271 | 172 | 13692 | 2.3499528018536 | 0.0212198831707288 | 0.206728817378833 | 1.67325701150195 | RGN//TRPV4//STIM2//NMB//DHRS7C//WFS1//XCL1//LPAR1 |
| GO:0006520 | cellular amino acid metabolic process | Biological process | 9 | 325 | 172 | 13692 | 2.20443649373882 | 0.0216658340173708 | 0.210605357720961 | 1.66422458832034 | GSTM2//MGST1//HDC//GHR//ATP7A//SLC5A5//PLSCR1//TTR//FOLR1 |
| GO:0033554 | cellular response to stress | Biological process | 19 | 919 | 172 | 13692 | 1.64579801098262 | 0.0220012863287367 | 0.213132758938182 | 1.65755192694411 | GADD45A//VAV3//TRPV4//MDFIC//TSPO//MMP2//ATP7A//NDRG1//WFS1//SCARA5//CD74//TLR3//SPP1//AQP1//ANXA1//LCN2//MGST1//PTGS2//RGD1305645 |
| GO:0030324 | lung development | Biological process | 6 | 173 | 172 | 13692 | 2.7608549536228 | 0.0220366063579811 | 0.213132758938182 | 1.65685528611256 | ATP7A//WNT2B//CP//MGP//MMP14//FOXJ1 |
| GO:0030335 | positive regulation of cell migration | Biological process | 7 | 222 | 172 | 13692 | 2.5100565681961 | 0.0221218226815417 | 0.213132758938182 | 1.65517909310312 | XCL1//AQP1//LPAR1//CD74//RGD1560691//PTGS2//CXCL16 |
| GO:0003006 | developmental process involved in reproduction | Biological process | 11 | 437 | 172 | 13692 | 2.00377840455537 | 0.0221648043056079 | 0.213132758938182 | 1.65433609875986 | MMP2//MMP14//ADAMTS1//WNT2B//TLR3//TCF7L2//BMP6//MGST1//PTGS2//NTRK1//BMP7 |
| GO:0002822 | regulation of adaptive immune response based on somatic recombination of immune receptors built from immunoglobulin superfamily domains | Biological process | 4 | 85 | 172 | 13692 | 3.74610123119015 | 0.0221689183567087 | 0.213132758938182 | 1.65425549602274 | XCL1//FOXJ1//RT1-BB//CLCF1 |
| GO:0040007 | growth | Biological process | 16 | 734 | 172 | 13692 | 1.73525125150497 | 0.0224433658478498 | 0.215258405053194 | 1.64891201107313 | MMP2//IGFBP2//LMX1A//MESP1//MSX2//CXCL16//DAB2//MSX1//RERG//SLC12A2//GHR//PTGS2//WFS1//TCF7L2//SPP1//BMP6 |
| GO:0006979 | response to oxidative stress | Biological process | 8 | 274 | 172 | 13692 | 2.32422339161433 | 0.0224882184111229 | 0.215258405053194 | 1.64804494940503 | ATP7A//MMP2//AQP1//ANXA1//LCN2//MGST1//PTGS2//MMP14 |
| GO:0080134 | regulation of response to stress | Biological process | 13 | 554 | 172 | 13692 | 1.86797917891025 | 0.0225839005923561 | 0.215333565459463 | 1.64620104649266 | SERPING1//C6//TLR3//ANXA1//MDFIC//PTGS2//ANXA2//CD74//PLSCR1//TRPV4//SPP1//XCL1//ACE |
| GO:0015833 | peptide transport | Biological process | 6 | 174 | 172 | 13692 | 2.74498797113071 | 0.0225943065947429 | 0.215333565459463 | 1.64600098235658 | CD74//ANXA1//TRPV4//TCF7L2//SLC22A8//AQP1 |
| GO:0051704 | multi-organism process | Biological process | 17 | 797 | 172 | 13692 | 1.69796621049867 | 0.022761701417872 | 0.216083241632006 | 1.6427952779195 | CD14//LCN2//XCL1//TLR3//IGF2//IGFBP2//MMP2//PTGS2//SOSTDC1//FBLN1//BAIAP2L1//ACE//UGT1A6//MGST1//PLSCR1//TSPO//AQP1 |
| GO:0048514 | blood vessel morphogenesis | Biological process | 10 | 383 | 172 | 13692 | 2.07845042200498 | 0.0228966384184264 | 0.216083241632006 | 1.64022827414447 | PTGS2//ANXA2//MMP2//MMP14//TGFBI//T//C6//AQP1//NTRK1//PRRX2 |
| GO:0031344 | regulation of cell projection organization | Biological process | 8 | 275 | 172 | 13692 | 2.31577167019028 | 0.0229226673440953 | 0.216083241632006 | 1.6397348480737 | AQP1//NTRK1//RGD1560691//TRPV4//LPAR1//LCN2//SPP1//BMP7 |
| GO:0031347 | regulation of defense response | Biological process | 8 | 275 | 172 | 13692 | 2.31577167019028 | 0.0229226673440953 | 0.216083241632006 | 1.6397348480737 | SERPING1//C6//TLR3//ANXA1//PTGS2//PLSCR1//XCL1//ACE |
| GO:0007009 | plasma membrane organization | Biological process | 4 | 86 | 172 | 13692 | 3.7025419145484 | 0.0230293223698747 | 0.216083241632006 | 1.63771884081796 | PLSCR1//NDRG1//CRB3//SPTA1 |
| GO:0046631 | alpha-beta T cell activation | Biological process | 4 | 86 | 172 | 13692 | 3.7025419145484 | 0.0230293223698747 | 0.216083241632006 | 1.63771884081796 | ATP7A//ANXA1//RT1-BB//XCL1 |
| GO:0030323 | respiratory tube development | Biological process | 6 | 175 | 172 | 13692 | 2.7293023255814 | 0.0231614557036917 | 0.216083241632006 | 1.63523414856234 | CP//MGP//MMP14//FOXJ1//ATP7A//WNT2B |
| GO:0006958 | complement activation, classical pathway | Biological process | 2 | 19 | 172 | 13692 | 8.37943696450428 | 0.023320402793024 | 0.216083241632006 | 1.63226395265758 | C6//SERPING1 |
| GO:0070265 | necrotic cell death | Biological process | 2 | 19 | 172 | 13692 | 8.37943696450428 | 0.023320402793024 | 0.216083241632006 | 1.63226395265758 | TSPO//TMEM123 |
| GO:0071604 | transforming growth factor beta production | Biological process | 2 | 19 | 172 | 13692 | 8.37943696450428 | 0.023320402793024 | 0.216083241632006 | 1.63226395265758 | PTGS2//XCL1 |
| GO:0071634 | regulation of transforming growth factor beta production | Biological process | 2 | 19 | 172 | 13692 | 8.37943696450428 | 0.023320402793024 | 0.216083241632006 | 1.63226395265758 | PTGS2//XCL1 |
| GO:0016358 | dendrite development | Biological process | 5 | 129 | 172 | 13692 | 3.085451595457 | 0.0233540261003848 | 0.216083241632006 | 1.63163823876146 | ATP7A//DCDC2//RGD1560691//LPAR1//BMP7 |
| GO:0048771 | tissue remodeling | Biological process | 5 | 129 | 172 | 13692 | 3.085451595457 | 0.0233540261003848 | 0.216083241632006 | 1.63163823876146 | ATP7A//SEMA3C//SPP1//MMP2//MMP14 |
| GO:0042060 | wound healing | Biological process | 8 | 276 | 172 | 13692 | 2.30738119312437 | 0.0233630147202488 | 0.216083241632006 | 1.6314711173862 | SERPING1//F5//MSX2//ANXA2//IGF2//AQP1//FMOD//CDH3 |
| GO:0010463 | mesenchymal cell proliferation | Biological process | 3 | 49 | 172 | 13692 | 4.87375415282392 | 0.0235329248954997 | 0.2162858338404 | 1.62832409122466 | PRRX2//BMP7//MSX1 |
| GO:0035272 | exocrine system development | Biological process | 3 | 49 | 172 | 13692 | 4.87375415282392 | 0.0235329248954997 | 0.2162858338404 | 1.62832409122466 | BMP7//IGF2//SEMA3C |
| GO:0045669 | positive regulation of osteoblast differentiation | Biological process | 3 | 49 | 172 | 13692 | 4.87375415282392 | 0.0235329248954997 | 0.2162858338404 | 1.62832409122466 | MSX2//BMP6//BMP7 |
| GO:0051090 | regulation of sequence-specific DNA binding transcription factor activity | Biological process | 8 | 277 | 172 | 13692 | 2.29905129712031 | 0.0238092979758702 | 0.217922328280718 | 1.62325340970644 | FOXJ1//MSX2//RGD1560691//WFS1//XCL1//TCF7L2//NTRK1//TLR3 |
| GO:0009894 | regulation of catabolic process | Biological process | 11 | 442 | 172 | 13692 | 1.98111122803325 | 0.0238539440091601 | 0.217922328280718 | 1.62243980447272 | ABHD5//ACAP2//NTRK1//DAB2//RGN//FOXJ1//SERPINB1A//VAV3//APOA2//PTPN3//PLSCR1 |
| GO:0034329 | cell junction assembly | Biological process | 4 | 87 | 172 | 13692 | 3.65998396150762 | 0.023909817496128 | 0.217922328280718 | 1.62142373884562 | TRPV4//CD9//CLDN19//MMP14 |
| GO:0051250 | negative regulation of lymphocyte activation | Biological process | 4 | 87 | 172 | 13692 | 3.65998396150762 | 0.023909817496128 | 0.217922328280718 | 1.62142373884562 | FOXJ1//RT1-BB//CD74//XCL1 |
| GO:0032879 | regulation of localization | Biological process | 24 | 1252 | 172 | 13692 | 1.52596775391931 | 0.0240908216572677 | 0.219116518973987 | 1.61814838741834 | XCL1//APOA2//CD74//AQP1//PON1//DHRS7C//ABHD5//ACE//NMB//ENPP2//LAMA2//CXCL16//ANXA1//ANXA2//TCF7L2//STIM2//MDFIC//PTGS2//TLR3//CD14//RGD1560691//TSPO//WFS1//LPAR1 |
| GO:0007389 | pattern specification process | Biological process | 10 | 387 | 172 | 13692 | 2.05696773030467 | 0.024382638281432 | 0.221311565684882 | 1.61291930408777 | T//SEMA3C//FOXJ1//MESP1//BMP7//MSX2//MSX1//WNT2B//CCDC40//SOSTDC1 |
| GO:2000147 | positive regulation of cell motility | Biological process | 7 | 227 | 172 | 13692 | 2.45476897858826 | 0.0246137071277305 | 0.222947297619774 | 1.60882297126797 | XCL1//AQP1//CXCL16//LPAR1//CD74//RGD1560691//PTGS2 |
| GO:0046889 | positive regulation of lipid biosynthetic process | Biological process | 3 | 50 | 172 | 13692 | 4.77627906976744 | 0.0248095962631197 | 0.223796851887895 | 1.60538030312505 | ANXA1//BMP6//IGF2 |
| GO:0048645 | organ formation | Biological process | 3 | 50 | 172 | 13692 | 4.77627906976744 | 0.0248095962631197 | 0.223796851887895 | 1.60538030312505 | MESP1//WNT2B//BMP7 |
| GO:2000145 | regulation of cell motility | Biological process | 10 | 389 | 172 | 13692 | 2.04639206074012 | 0.0251512576217686 | 0.225613367551507 | 1.59944029432419 | XCL1//AQP1//ACE//ENPP2//LAMA2//CXCL16//RGD1560691//LPAR1//CD74//PTGS2 |
| GO:0046546 | development of primary male sexual characteristics | Biological process | 5 | 132 | 172 | 13692 | 3.01532769556025 | 0.0254808938360633 | 0.225613367551507 | 1.59378534159331 | WNT2B//TLR3//BMP6//MGST1//NTRK1 |
| GO:0048812 | neuron projection morphogenesis | Biological process | 10 | 390 | 172 | 13692 | 2.04114490161002 | 0.0255420581854349 | 0.225613367551507 | 1.59274411010461 | NTRK1//BMP7//LMX1A//SEMA3C//LAMA2//TSPO//MMP2//SPP1//ATP7A//DCDC2 |
| GO:0009893 | positive regulation of metabolic process | Biological process | 31 | 1731 | 172 | 13692 | 1.42561766958204 | 0.025612026685886 | 0.225613367551507 | 1.59155605434408 | GHR//CD74//LPAR1//IGF2//DAB2//TLR3//C6//T//MDFIC//PLSCR1//LCN2//TSPO//GADD45A//BMP7//BMP6//APOA2//ABHD5//TCF7L2//MDK//CDKN1C//MESP1//ANXA1//WFS1//CLCF1//VAV3//PTGS2//MSX1//FOXJ1//MOSPD1//CREB3L1//RGD1560691 |
| GO:0002720 | positive regulation of cytokine production involved in immune response | Biological process | 2 | 20 | 172 | 13692 | 7.96046511627907 | 0.0257007121140895 | 0.225613367551507 | 1.59005484309147 | XCL1//CD74 |
| GO:0002828 | regulation of type 2 immune response | Biological process | 2 | 20 | 172 | 13692 | 7.96046511627907 | 0.0257007121140895 | 0.225613367551507 | 1.59005484309147 | CD74//XCL1 |
| GO:0003197 | endocardial cushion development | Biological process | 2 | 20 | 172 | 13692 | 7.96046511627907 | 0.0257007121140895 | 0.225613367551507 | 1.59005484309147 | MSX2//MSX1 |
| GO:0050996 | positive regulation of lipid catabolic process | Biological process | 2 | 20 | 172 | 13692 | 7.96046511627907 | 0.0257007121140895 | 0.225613367551507 | 1.59005484309147 | ABHD5//APOA2 |
| GO:0051968 | positive regulation of synaptic transmission, glutamatergic | Biological process | 2 | 20 | 172 | 13692 | 7.96046511627907 | 0.0257007121140895 | 0.225613367551507 | 1.59005484309147 | PTGS2//NTRK1 |
| GO:0071391 | cellular response to estrogen stimulus | Biological process | 2 | 20 | 172 | 13692 | 7.96046511627907 | 0.0257007121140895 | 0.225613367551507 | 1.59005484309147 | MSX2//MMP2 |
| GO:0002819 | regulation of adaptive immune response | Biological process | 4 | 89 | 172 | 13692 | 3.57773713091194 | 0.0257314515911846 | 0.225613367551507 | 1.5895357131966 | XCL1//FOXJ1//RT1-BB//CLCF1 |
| GO:0003206 | cardiac chamber morphogenesis | Biological process | 4 | 89 | 172 | 13692 | 3.57773713091194 | 0.0257314515911846 | 0.225613367551507 | 1.5895357131966 | MSX2//MESP1//SEMA3C//ADAMTS1 |
| GO:0045766 | positive regulation of angiogenesis | Biological process | 4 | 89 | 172 | 13692 | 3.57773713091194 | 0.0257314515911846 | 0.225613367551507 | 1.5895357131966 | PTGS2//C6//AQP1//NTRK1 |
| GO:0048520 | positive regulation of behavior | Biological process | 4 | 89 | 172 | 13692 | 3.57773713091194 | 0.0257314515911846 | 0.225613367551507 | 1.5895357131966 | XCL1//LPAR1//CD74//RGD1560691 |
| GO:0030888 | regulation of B cell proliferation | Biological process | 3 | 51 | 172 | 13692 | 4.68262653898769 | 0.0261229876078596 | 0.227680273703492 | 1.58297715559723 | CD74//VAV3//CLCF1 |
| GO:0050810 | regulation of steroid biosynthetic process | Biological process | 3 | 51 | 172 | 13692 | 4.68262653898769 | 0.0261229876078596 | 0.227680273703492 | 1.58297715559723 | BMP6//IGF2//TSPO |
| GO:0070301 | cellular response to hydrogen peroxide | Biological process | 3 | 51 | 172 | 13692 | 4.68262653898769 | 0.0261229876078596 | 0.227680273703492 | 1.58297715559723 | AQP1//ANXA1//LCN2 |
| GO:0032496 | response to lipopolysaccharide | Biological process | 8 | 283 | 172 | 13692 | 2.25030816007889 | 0.0266137057857193 | 0.23149699635832 | 1.57489464855141 | TSPO//CD14//PLSCR1//LCN2//ACE//PTGS2//UGT1A6//MGST1 |
| GO:0046578 | regulation of Ras protein signal transduction | Biological process | 7 | 231 | 172 | 13692 | 2.4122621564482 | 0.0267414298479702 | 0.23214738307624 | 1.5728153749917 | ACAP2//NTRK1//FOXJ1//RASGRF2//VAV3//ARHGEF26//LPAR1 |
| GO:0043279 | response to alkaloid | Biological process | 5 | 134 | 172 | 13692 | 2.97032280458174 | 0.0269656653074761 | 0.233631376893232 | 1.56918886015975 | IGF2//NTRK1//MMP2//GHR//MSX1 |
| GO:0032945 | negative regulation of mononuclear cell proliferation | Biological process | 3 | 52 | 172 | 13692 | 4.59257602862254 | 0.0274730508446101 | 0.237090265556635 | 1.56109311009495 | FOXJ1//RT1-BB//XCL1 |
| GO:0050672 | negative regulation of lymphocyte proliferation | Biological process | 3 | 52 | 172 | 13692 | 4.59257602862254 | 0.0274730508446101 | 0.237090265556635 | 1.56109311009495 | FOXJ1//RT1-BB//XCL1 |
| GO:0009636 | response to toxin | Biological process | 5 | 135 | 172 | 13692 | 2.94832041343669 | 0.0277283243605376 | 0.238823131623962 | 1.55707637430818 | LCN2//ATP7A//SLC22A8//PON1//PTGR1 |
| GO:0001759 | organ induction | Biological process | 2 | 21 | 172 | 13692 | 7.58139534883721 | 0.0281751459514386 | 0.23984434922545 | 1.55013382556614 | MESP1//WNT2B |
| GO:0002704 | negative regulation of leukocyte mediated immunity | Biological process | 2 | 21 | 172 | 13692 | 7.58139534883721 | 0.0281751459514386 | 0.23984434922545 | 1.55013382556614 | XCL1//FOXJ1 |
| GO:0002707 | negative regulation of lymphocyte mediated immunity | Biological process | 2 | 21 | 172 | 13692 | 7.58139534883721 | 0.0281751459514386 | 0.23984434922545 | 1.55013382556614 | XCL1//FOXJ1 |
| GO:0043462 | regulation of ATPase activity | Biological process | 2 | 21 | 172 | 13692 | 7.58139534883721 | 0.0281751459514386 | 0.23984434922545 | 1.55013382556614 | RGN//PLSCR1 |
| GO:0045581 | negative regulation of T cell differentiation | Biological process | 2 | 21 | 172 | 13692 | 7.58139534883721 | 0.0281751459514386 | 0.23984434922545 | 1.55013382556614 | FOXJ1//CD74 |
| GO:0046885 | regulation of hormone biosynthetic process | Biological process | 2 | 21 | 172 | 13692 | 7.58139534883721 | 0.0281751459514386 | 0.23984434922545 | 1.55013382556614 | BMP6//IGF2 |
| GO:0051272 | positive regulation of cellular component movement | Biological process | 7 | 234 | 172 | 13692 | 2.38133571854502 | 0.0284173971417288 | 0.241243472594168 | 1.5464157032823 | XCL1//AQP1//CXCL16//LPAR1//CD74//RGD1560691//PTGS2 |
| GO:0001649 | osteoblast differentiation | Biological process | 5 | 136 | 172 | 13692 | 2.9266415868673 | 0.0285045891431978 | 0.241243472594168 | 1.54508521440824 | MSX2//BMP6//BMP7//IGF2//SPP1 |
| GO:2000027 | regulation of organ morphogenesis | Biological process | 5 | 136 | 172 | 13692 | 2.9266415868673 | 0.0285045891431978 | 0.241243472594168 | 1.54508521440824 | MESP1//MSX1//WNT2B//BMP7//DAB2 |
| GO:0043410 | positive regulation of MAPK cascade | Biological process | 6 | 184 | 172 | 13692 | 2.59580384226491 | 0.0287008769631666 | 0.242436694810255 | 1.54210483307627 | TLR3//CD74//NTRK1//C1QTNF3//IGF2//LPAR1 |
| GO:0046942 | carboxylic acid transport | Biological process | 6 | 185 | 172 | 13692 | 2.58177247014456 | 0.0293657468150722 | 0.24757583468707 | 1.53215894991925 | SLCO1A5//FOLR1//SLC22A8//ACE//ANXA1//NMB |
| GO:0006816 | calcium ion transport | Biological process | 7 | 236 | 172 | 13692 | 2.36115490737091 | 0.0295735277408507 | 0.248372309608984 | 1.52909686664727 | DHRS7C//STIM2//XCL1//PTGS2//TSPO//WFS1//TRPV4 |
| GO:0040017 | positive regulation of locomotion | Biological process | 7 | 236 | 172 | 13692 | 2.36115490737091 | 0.0295735277408507 | 0.248372309608984 | 1.52909686664727 | XCL1//AQP1//CXCL16//LPAR1//CD74//RGD1560691//PTGS2 |
| GO:0002688 | regulation of leukocyte chemotaxis | Biological process | 3 | 54 | 172 | 13692 | 4.42248062015504 | 0.0302829127924563 | 0.253359331454444 | 1.51880235411085 | XCL1//RGD1560691//CD74 |
| GO:0070664 | negative regulation of leukocyte proliferation | Biological process | 3 | 54 | 172 | 13692 | 4.42248062015504 | 0.0302829127924563 | 0.253359331454444 | 1.51880235411085 | FOXJ1//RT1-BB//XCL1 |
| GO:0015849 | organic acid transport | Biological process | 6 | 187 | 172 | 13692 | 2.55415993035692 | 0.0307257473985378 | 0.255894959258794 | 1.5124975441742 | SLCO1A5//FOLR1//SLC22A8//ACE//ANXA1//NMB |
| GO:0042474 | middle ear morphogenesis | Biological process | 2 | 22 | 172 | 13692 | 7.23678646934461 | 0.0307409932047452 | 0.255894959258794 | 1.51228210507401 | MSX1//PRRX2 |
| GO:0071496 | cellular response to external stimulus | Biological process | 7 | 238 | 172 | 13692 | 2.34131326949384 | 0.0307610956955713 | 0.255894959258794 | 1.51199819923259 | IGF2//GADD45A//AQP1//PTGS2//TLR3//LCN2//T |
| GO:0009308 | amine metabolic process | Biological process | 5 | 139 | 172 | 13692 | 2.86347666053204 | 0.0309156522714609 | 0.256208354552145 | 1.50982158614043 | HDC//ATP7A//APOA2//ENPP2//PON1 |
| GO:0046661 | male sex differentiation | Biological process | 5 | 139 | 172 | 13692 | 2.86347666053204 | 0.0309156522714609 | 0.256208354552145 | 1.50982158614043 | WNT2B//TLR3//BMP6//MGST1//NTRK1 |
| GO:0010035 | response to inorganic substance | Biological process | 11 | 461 | 172 | 13692 | 1.89946022297331 | 0.0311376715821954 | 0.257561419276122 | 1.50671386629621 | CYBRD1//ATP7A//TSPO//PTGS2//IGFBP2//MMP2//CP//MGP//AQP1//ANXA1//LCN2 |
| GO:0043393 | regulation of protein binding | Biological process | 4 | 95 | 172 | 13692 | 3.35177478580171 | 0.031687104724826 | 0.260634237541151 | 1.49911744081837 | DAB2//PLSCR1//SPTA1//TCF7L2 |
| GO:0050920 | regulation of chemotaxis | Biological process | 4 | 95 | 172 | 13692 | 3.35177478580171 | 0.031687104724826 | 0.260634237541151 | 1.49911744081837 | XCL1//RGD1560691//LPAR1//CD74 |
| GO:0050871 | positive regulation of B cell activation | Biological process | 3 | 55 | 172 | 13692 | 4.34207188160677 | 0.0317425292759163 | 0.260634237541151 | 1.49835847119145 | CD74//VAV3//CLCF1 |
| GO:0035023 | regulation of Rho protein signal transduction | Biological process | 5 | 140 | 172 | 13692 | 2.84302325581395 | 0.0317469623282332 | 0.260634237541151 | 1.49829782335466 | FOXJ1//LPAR1//RASGRF2//VAV3//ARHGEF26 |
| GO:0030155 | regulation of cell adhesion | Biological process | 7 | 241 | 172 | 13692 | 2.31216829103541 | 0.032602080332325 | 0.267154243321332 | 1.48675468680251 | MMP2//SPP1//COL8A1//BMP7//VAV3//MMP14//LAMA2 |
| GO:0034612 | response to tumor necrosis factor | Biological process | 4 | 96 | 172 | 13692 | 3.31686046511628 | 0.0327519445029444 | 0.26788157593453 | 1.48476291057817 | KRT18//LCN2//PTGS2//CXCL16 |
| GO:0006821 | chloride transport | Biological process | 3 | 56 | 172 | 13692 | 4.26453488372093 | 0.033238454365895 | 0.270074984123118 | 1.47835917971253 | SLC12A2//TSPO//SLC4A2 |
| GO:0018208 | peptidyl-proline modification | Biological process | 2 | 23 | 172 | 13692 | 6.9221435793731 | 0.0333955953374376 | 0.270074984123118 | 1.47631081003846 | FKBP9//FKBP14 |
| GO:0030104 | water homeostasis | Biological process | 2 | 23 | 172 | 13692 | 6.9221435793731 | 0.0333955953374376 | 0.270074984123118 | 1.47631081003846 | WFS1//AQP1 |
| GO:0042092 | type 2 immune response | Biological process | 2 | 23 | 172 | 13692 | 6.9221435793731 | 0.0333955953374376 | 0.270074984123118 | 1.47631081003846 | CD74//XCL1 |
| GO:0042403 | thyroid hormone metabolic process | Biological process | 2 | 23 | 172 | 13692 | 6.9221435793731 | 0.0333955953374376 | 0.270074984123118 | 1.47631081003846 | SLC5A5//TTR |
| GO:0071312 | cellular response to alkaloid | Biological process | 2 | 23 | 172 | 13692 | 6.9221435793731 | 0.0333955953374376 | 0.270074984123118 | 1.47631081003846 | NTRK1//MSX1 |
| GO:0007411 | axon guidance | Biological process | 5 | 142 | 172 | 13692 | 2.80298067474615 | 0.033451349538972 | 0.270074984123118 | 1.47558635665532 | NTRK1//BMP7//LMX1A//SEMA3C//LAMA2 |
| GO:0002237 | response to molecule of bacterial origin | Biological process | 8 | 297 | 172 | 13692 | 2.14423302795396 | 0.0340360818870844 | 0.274290777560621 | 1.46806044014905 | ACE//PTGS2//CD14//UGT1A6//MGST1//TSPO//PLSCR1//LCN2 |
| GO:0060485 | mesenchyme development | Biological process | 5 | 143 | 172 | 13692 | 2.78337941128639 | 0.0343245294051292 | 0.276107774150617 | 1.46439540829825 | SEMA3C//MSX1//MSX2//BMP7 |
| GO:0001707 | mesoderm formation | Biological process | 3 | 57 | 172 | 13692 | 4.18971848225214 | 0.0347705574297586 | 0.278672986786219 | 1.45878834679941 | T//MESP1//BMP7 |
| GO:0034341 | response to interferon-gamma | Biological process | 3 | 57 | 172 | 13692 | 4.18971848225214 | 0.0347705574297586 | 0.278672986786219 | 1.45878834679941 | RT1-BB//TLR3//CXCL16 |
| GO:0044248 | cellular catabolic process | Biological process | 23 | 1232 | 172 | 13692 | 1.48612579281184 | 0.0351767283382114 | 0.281181012306166 | 1.45374455517799 | SMURF2//RERG//HDC//HADHA//BDH2//RGD1305645//APOA2//PON3//ABHD5//PON1//WFS1//ACAP2//NTRK1//DAB2//RGN//FOXJ1//ENPP2//GSTM2//VAV3//ACE//CES1D//PTPN3//PLSCR1 |
| GO:0000302 | response to reactive oxygen species | Biological process | 5 | 144 | 172 | 13692 | 2.7640503875969 | 0.0352117645429026 | 0.281181012306166 | 1.45331221088426 | ATP7A//MMP2//AQP1//ANXA1//LCN2 |
| GO:0051270 | regulation of cellular component movement | Biological process | 10 | 412 | 172 | 13692 | 1.9321517272522 | 0.0352839968846501 | 0.281245531531466 | 1.4524222249396 | XCL1//AQP1//ACE//ENPP2//LAMA2//CXCL16//RGD1560691//LPAR1//CD74//PTGS2 |
| GO:0046890 | regulation of lipid biosynthetic process | Biological process | 4 | 99 | 172 | 13692 | 3.21634954193094 | 0.0360712072592766 | 0.284931184634773 | 1.4428393221322 | TSPO//ANXA1//BMP6//IGF2 |
| GO:0001508 | regulation of action potential | Biological process | 5 | 145 | 172 | 13692 | 2.74498797113071 | 0.0361131002416406 | 0.284931184634773 | 1.4423352266094 | LAMA2//CD9//TCF7L2//NDRG1//LPAR1 |
| GO:0006767 | water-soluble vitamin metabolic process | Biological process | 2 | 24 | 172 | 13692 | 6.63372093023256 | 0.0361363454965634 | 0.284931184634773 | 1.44205577017376 | RGN//FOLR1 |
| GO:0010862 | positive regulation of pathway-restricted SMAD protein phosphorylation | Biological process | 2 | 24 | 172 | 13692 | 6.63372093023256 | 0.0361363454965634 | 0.284931184634773 | 1.44205577017376 | BMP6//BMP7 |
| GO:0014037 | Schwann cell differentiation | Biological process | 2 | 24 | 172 | 13692 | 6.63372093023256 | 0.0361363454965634 | 0.284931184634773 | 1.44205577017376 | LAMA2//NDRG1 |
| GO:0060445 | branching involved in salivary gland morphogenesis | Biological process | 2 | 24 | 172 | 13692 | 6.63372093023256 | 0.0361363454965634 | 0.284931184634773 | 1.44205577017376 | SEMA3C//BMP7 |
| GO:0032844 | regulation of homeostatic process | Biological process | 7 | 247 | 172 | 13692 | 2.25600225967423 | 0.0365023990155781 | 0.287300749163904 | 1.43767859184518 | CD74//DHRS7C//PTGS2//TCF7L2//SPP1//XCL1//TSPO |
| GO:0060541 | respiratory system development | Biological process | 6 | 195 | 172 | 13692 | 2.44937388193202 | 0.0365761035941357 | 0.287364942933138 | 1.43680256123511 | CP//MGP//MMP14//FOXJ1//ATP7A//WNT2B |
| GO:0045664 | regulation of neuron differentiation | Biological process | 9 | 358 | 172 | 13692 | 2.00123424710926 | 0.0370077955573526 | 0.290236450310257 | 1.4317067837455 | NTRK1//RGD1560691//TRPV4//LPAR1//BMP7//LMX1A//BMP6//SPP1//ZFHX3 |
| GO:0002695 | negative regulation of leukocyte activation | Biological process | 4 | 100 | 172 | 13692 | 3.18418604651163 | 0.0372193111052498 | 0.290854652202166 | 1.42923166951684 | FOXJ1//RT1-BB//CD74//XCL1 |
| GO:0003205 | cardiac chamber development | Biological process | 4 | 100 | 172 | 13692 | 3.18418604651163 | 0.0372193111052498 | 0.290854652202166 | 1.42923166951684 | MSX2//MESP1//SEMA3C//ADAMTS1 |
| GO:0022600 | digestive system process | Biological process | 3 | 59 | 172 | 13692 | 4.04769412692156 | 0.0379427014747781 | 0.294930502243665 | 1.42087175110098 | AQP1//APOA2//SLCO1A5 |
| GO:0050777 | negative regulation of immune response | Biological process | 3 | 59 | 172 | 13692 | 4.04769412692156 | 0.0379427014747781 | 0.294930502243665 | 1.42087175110098 | SERPING1//FOXJ1//XCL1 |
| GO:0051897 | positive regulation of protein kinase B signaling cascade | Biological process | 3 | 59 | 172 | 13692 | 4.04769412692156 | 0.0379427014747781 | 0.294930502243665 | 1.42087175110098 | IGF2//C1QTNF3//TCF7L2 |
| GO:0040012 | regulation of locomotion | Biological process | 10 | 418 | 172 | 13692 | 1.90441749193279 | 0.0383388286744423 | 0.297482167980097 | 1.41636115979382 | XCL1//AQP1//ACE//ENPP2//LAMA2//CXCL16//RGD1560691//LPAR1//CD74//PTGS2 |
| GO:0006457 | protein folding | Biological process | 5 | 148 | 172 | 13692 | 2.68934632306725 | 0.0389021272013784 | 0.300182169843989 | 1.41002665043362 | CD74//APOA2//FKBP9//FKBP14//DNAJC22 |
| GO:0010001 | glial cell differentiation | Biological process | 5 | 148 | 172 | 13692 | 2.68934632306725 | 0.0389021272013784 | 0.300182169843989 | 1.41002665043362 | CD9//TCF7L2//LAMA2//NDRG1//CLCF1 |
| GO:0015914 | phospholipid transport | Biological process | 2 | 25 | 172 | 13692 | 6.36837209302326 | 0.0389606876462659 | 0.300182169843989 | 1.4093733865382 | APOA2//PLSCR1 |
| GO:0042993 | positive regulation of transcription factor import into nucleus | Biological process | 2 | 25 | 172 | 13692 | 6.36837209302326 | 0.0389606876462659 | 0.300182169843989 | 1.4093733865382 | PTGS2//TLR3 |
| GO:0030595 | leukocyte chemotaxis | Biological process | 4 | 102 | 172 | 13692 | 3.12175102599179 | 0.0395781690338409 | 0.303372871414344 | 1.40254430100614 | XCL1//SPP1//RGD1560691//CD74 |
| GO:0002698 | negative regulation of immune effector process | Biological process | 3 | 60 | 172 | 13692 | 3.98023255813953 | 0.0395824093177474 | 0.303372871414344 | 1.40249777451676 | SERPING1//XCL1//FOXJ1 |
| GO:0042100 | B cell proliferation | Biological process | 3 | 60 | 172 | 13692 | 3.98023255813953 | 0.0395824093177474 | 0.303372871414344 | 1.40249777451676 | CD74//VAV3//CLCF1 |
| GO:0031346 | positive regulation of cell projection organization | Biological process | 5 | 149 | 172 | 13692 | 2.67129701888559 | 0.0398602695220428 | 0.304681004641393 | 1.39945976906904 | AQP1//NTRK1//RGD1560691//BMP7//LCN2 |
| GO:0048858 | cell projection morphogenesis | Biological process | 11 | 480 | 172 | 13692 | 1.82427325581395 | 0.0398920840931021 | 0.304681004641393 | 1.39911327413161 | NTRK1//BMP7//LMX1A//SEMA3C//LAMA2//TSPO//MMP2//FOXJ1//SPP1//ATP7A//DCDC2 |
| GO:0051345 | positive regulation of hydrolase activity | Biological process | 8 | 307 | 172 | 13692 | 2.07438830391637 | 0.0401290408947524 | 0.30595776570886 | 1.39654122000935 | C6//PCOLCE//MMP14//NTRK1//RGN//FOXJ1//ABHD5//PLSCR1 |
| GO:0001558 | regulation of cell growth | Biological process | 7 | 253 | 172 | 13692 | 2.20250022980053 | 0.0406999886652114 | 0.30703072912157 | 1.39040571172409 | CXCL16//DAB2//MSX1//RERG//SPP1//IGFBP2//LMX1A |
| GO:0006897 | endocytosis | Biological process | 8 | 308 | 172 | 13692 | 2.0676532769556 | 0.0407759058581155 | 0.30703072912157 | 1.38959638210103 | DAB2//SCARA5//CXCL16//GULP1//GHR//CD14//RGD1560691//ANXA1 |
| GO:0043270 | positive regulation of ion transport | Biological process | 4 | 103 | 172 | 13692 | 3.09144276360352 | 0.0407889462662581 | 0.30703072912157 | 1.38945751401756 | STIM2//XCL1//TSPO//WFS1 |
| GO:0043524 | negative regulation of neuron apoptotic process | Biological process | 4 | 103 | 172 | 13692 | 3.09144276360352 | 0.0407889462662581 | 0.30703072912157 | 1.38945751401756 | ATP7A//NTRK1//WFS1//CLCF1 |
| GO:1901215 | negative regulation of neuron death | Biological process | 4 | 103 | 172 | 13692 | 3.09144276360352 | 0.0407889462662581 | 0.30703072912157 | 1.38945751401756 | ATP7A//NTRK1//WFS1//CLCF1 |
| GO:0016054 | organic acid catabolic process | Biological process | 5 | 150 | 172 | 13692 | 2.65348837209302 | 0.0408327024829564 | 0.30703072912157 | 1.3889918756588 | HDC//HADHA//BDH2//PON1//PON3 |
| GO:0046395 | carboxylic acid catabolic process | Biological process | 5 | 150 | 172 | 13692 | 2.65348837209302 | 0.0408327024829564 | 0.30703072912157 | 1.3889918756588 | HDC//HADHA//BDH2//PON1//PON3 |
| GO:0022603 | regulation of anatomical structure morphogenesis | Biological process | 12 | 543 | 172 | 13692 | 1.75921881022742 | 0.0412545621613783 | 0.30703072912157 | 1.38452801773332 | SPTA1//BMP7//MESP1//MSX1//TCF7L2//C6//AQP1//NTRK1//SPP1//WNT2B//PTGS2//DAB2 |
| GO:0001776 | leukocyte homeostasis | Biological process | 3 | 61 | 172 | 13692 | 3.91498284407167 | 0.0412576298958418 | 0.30703072912157 | 1.38449572432027 | SPTA1//CD74//ANXA1 |
| GO:0015908 | fatty acid transport | Biological process | 3 | 61 | 172 | 13692 | 3.91498284407167 | 0.0412576298958418 | 0.30703072912157 | 1.38449572432027 | ACE//ANXA1//NMB |
| GO:0045598 | regulation of fat cell differentiation | Biological process | 3 | 61 | 172 | 13692 | 3.91498284407167 | 0.0412576298958418 | 0.30703072912157 | 1.38449572432027 | MSX2//TCF7L2//PTGS2 |
| GO:0048332 | mesoderm morphogenesis | Biological process | 3 | 61 | 172 | 13692 | 3.91498284407167 | 0.0412576298958418 | 0.30703072912157 | 1.38449572432027 | BMP7//T//MESP1 |
| GO:0051153 | regulation of striated muscle cell differentiation | Biological process | 3 | 61 | 172 | 13692 | 3.91498284407167 | 0.0412576298958418 | 0.30703072912157 | 1.38449572432027 | ZFHX3//MSX1//MESP1 |
| GO:0010959 | regulation of metal ion transport | Biological process | 6 | 201 | 172 | 13692 | 2.37625824366539 | 0.0414040744497907 | 0.30703072912157 | 1.38295691916915 | DHRS7C//STIM2//XCL1//PTGS2//TSPO//WFS1 |
| GO:0051223 | regulation of protein transport | Biological process | 7 | 254 | 172 | 13692 | 2.19382896905329 | 0.0414289616784543 | 0.30703072912157 | 1.38269595115666 | APOA2//MDFIC//PTGS2//TLR3//TCF7L2//ANXA1//CD14 |
| GO:0001913 | T cell mediated cytotoxicity | Biological process | 2 | 26 | 172 | 13692 | 6.12343470483005 | 0.041866115714677 | 0.30703072912157 | 1.37813733053305 | XCL1//CTSC |
| GO:0019439 | aromatic compound catabolic process | Biological process | 2 | 26 | 172 | 13692 | 6.12343470483005 | 0.041866115714677 | 0.30703072912157 | 1.37813733053305 | PON3//PON1 |
| GO:0030166 | proteoglycan biosynthetic process | Biological process | 2 | 26 | 172 | 13692 | 6.12343470483005 | 0.041866115714677 | 0.30703072912157 | 1.37813733053305 | TCF7L2//DSE |
| GO:0035115 | embryonic forelimb morphogenesis | Biological process | 2 | 26 | 172 | 13692 | 6.12343470483005 | 0.041866115714677 | 0.30703072912157 | 1.37813733053305 | MSX2//MSX1 |
| GO:0044058 | regulation of digestive system process | Biological process | 2 | 26 | 172 | 13692 | 6.12343470483005 | 0.041866115714677 | 0.30703072912157 | 1.37813733053305 | AQP1//APOA2 |
| GO:0050974 | detection of mechanical stimulus involved in sensory perception | Biological process | 2 | 26 | 172 | 13692 | 6.12343470483005 | 0.041866115714677 | 0.30703072912157 | 1.37813733053305 | SLC12A2//NTRK1 |
| GO:0060571 | morphogenesis of an epithelial fold | Biological process | 2 | 26 | 172 | 13692 | 6.12343470483005 | 0.041866115714677 | 0.30703072912157 | 1.37813733053305 | WNT2B//BMP7 |
| GO:0019233 | sensory perception of pain | Biological process | 4 | 104 | 172 | 13692 | 3.06171735241503 | 0.0420206289856163 | 0.30703072912157 | 1.3765374511462 | NTRK1//ACE//AQP1//PTGS2 |
| GO:0030326 | embryonic limb morphogenesis | Biological process | 4 | 104 | 172 | 13692 | 3.06171735241503 | 0.0420206289856163 | 0.30703072912157 | 1.3765374511462 | MSX2//MSX1//BMP7//PRRX2 |
| GO:0035113 | embryonic appendage morphogenesis | Biological process | 4 | 104 | 172 | 13692 | 3.06171735241503 | 0.0420206289856163 | 0.30703072912157 | 1.3765374511462 | MSX2//MSX1//BMP7//PRRX2 |
| GO:0055085 | transmembrane transport | Biological process | 12 | 546 | 172 | 13692 | 1.74955277280859 | 0.0427190760850264 | 0.311614691442189 | 1.36937814866475 | ATP7A//CP//STIM2//S100A6//SCARA5//AQP1//MDFIC//PTGS2//TLR3//APOA2//TRPV4//SLC16A6 |
| GO:0051049 | regulation of transport | Biological process | 18 | 926 | 172 | 13692 | 1.54739062735446 | 0.042902644758573 | 0.31187488439252 | 1.36751593465326 | APOA2//CD74//AQP1//PON1//DHRS7C//NMB//ANXA1//ANXA2//TCF7L2//STIM2//MDFIC//PTGS2//TLR3//CD14//RGD1560691//XCL1//TSPO//WFS1 |
| GO:0015711 | organic anion transport | Biological process | 3 | 62 | 172 | 13692 | 3.85183795948987 | 0.0429681638168526 | 0.31187488439252 | 1.36685320487433 | SLC16A8//SLCO1A5//SLC22A8 |
| GO:0030856 | regulation of epithelial cell differentiation | Biological process | 3 | 62 | 172 | 13692 | 3.85183795948987 | 0.0429681638168526 | 0.31187488439252 | 1.36685320487433 | BMP6//MSX2//FOXJ1 |
| GO:0016042 | lipid catabolic process | Biological process | 6 | 203 | 172 | 13692 | 2.35284683239775 | 0.0430987837763823 | 0.312305897645719 | 1.36553498521418 | HADHA//BDH2//APOA2//ABHD5//ENPP2//CES1D |
| GO:0002699 | positive regulation of immune effector process | Biological process | 4 | 105 | 172 | 13692 | 3.03255813953488 | 0.0432732156722923 | 0.313052438130907 | 1.36378083078137 | XCL1//C6//CLCF1//CD74 |
| GO:0006650 | glycerophospholipid metabolic process | Biological process | 5 | 153 | 172 | 13692 | 2.60145918832649 | 0.0438360407963683 | 0.315561909443807 | 1.35816867756057 | ABHD5//APOA2//PLSCR1//ENPP2//PON1 |
| GO:0006790 | sulfur compound metabolic process | Biological process | 5 | 153 | 172 | 13692 | 2.60145918832649 | 0.0438360407963683 | 0.315561909443807 | 1.35816867756057 | GSTM2//MGST1//TCF7L2//GHR//DSE |
| GO:0030162 | regulation of proteolysis | Biological process | 5 | 153 | 172 | 13692 | 2.60145918832649 | 0.0438360407963683 | 0.315561909443807 | 1.35816867756057 | DAB2//WFS1//PTPN3//SERPINB1A//SERPING1 |
| GO:0050866 | negative regulation of cell activation | Biological process | 4 | 106 | 172 | 13692 | 3.00394910048267 | 0.0445466996820891 | 0.316114400890104 | 1.35118446587964 | FOXJ1//RT1-BB//CD74//XCL1 |
| GO:0042310 | vasoconstriction | Biological process | 3 | 63 | 172 | 13692 | 3.7906976744186 | 0.0447137994500944 | 0.316114400890104 | 1.34955842541037 | MMP2//PTGS2//ACE |
| GO:0001525 | angiogenesis | Biological process | 8 | 314 | 172 | 13692 | 2.02814397866983 | 0.0448035043341917 | 0.316114400890104 | 1.34868801605806 | C6//AQP1//NTRK1//PTGS2//ANXA2//MMP2//MMP14//TGFBI |
| GO:0030811 | regulation of nucleotide catabolic process | Biological process | 6 | 205 | 172 | 13692 | 2.32989222915485 | 0.0448365901633157 | 0.316114400890104 | 1.3483674230522 | ACAP2//NTRK1//RGN//FOXJ1//VAV3//PLSCR1 |
| GO:0033121 | regulation of purine nucleotide catabolic process | Biological process | 6 | 205 | 172 | 13692 | 2.32989222915485 | 0.0448365901633157 | 0.316114400890104 | 1.3483674230522 | ACAP2//NTRK1//RGN//FOXJ1//VAV3//PLSCR1 |
| GO:0010470 | regulation of gastrulation | Biological process | 2 | 27 | 172 | 13692 | 5.89664082687339 | 0.0448501727540248 | 0.316114400890104 | 1.34823587979993 | BMP7//MESP1 |
| GO:0030195 | negative regulation of blood coagulation | Biological process | 2 | 27 | 172 | 13692 | 5.89664082687339 | 0.0448501727540248 | 0.316114400890104 | 1.34823587979993 | ANXA2//SERPING1 |
| GO:0030513 | positive regulation of BMP signaling pathway | Biological process | 2 | 27 | 172 | 13692 | 5.89664082687339 | 0.0448501727540248 | 0.316114400890104 | 1.34823587979993 | MSX2//MSX1 |
| GO:0045599 | negative regulation of fat cell differentiation | Biological process | 2 | 27 | 172 | 13692 | 5.89664082687339 | 0.0448501727540248 | 0.316114400890104 | 1.34823587979993 | MSX2//TCF7L2 |
| GO:0046579 | positive regulation of Ras protein signal transduction | Biological process | 2 | 27 | 172 | 13692 | 5.89664082687339 | 0.0448501727540248 | 0.316114400890104 | 1.34823587979993 | LPAR1//NTRK1 |
| GO:0060444 | branching involved in mammary gland duct morphogenesis | Biological process | 2 | 27 | 172 | 13692 | 5.89664082687339 | 0.0448501727540248 | 0.316114400890104 | 1.34823587979993 | MSX2//SLC12A2 |
| GO:0070613 | regulation of protein processing | Biological process | 2 | 27 | 172 | 13692 | 5.89664082687339 | 0.0448501727540248 | 0.316114400890104 | 1.34823587979993 | SERPING1//C6 |
| GO:1900047 | negative regulation of hemostasis | Biological process | 2 | 27 | 172 | 13692 | 5.89664082687339 | 0.0448501727540248 | 0.316114400890104 | 1.34823587979993 | ANXA2//SERPING1 |
| GO:0071222 | cellular response to lipopolysaccharide | Biological process | 4 | 107 | 172 | 13692 | 2.97587480982395 | 0.0458410693108802 | 0.322579852100961 | 1.33874526036008 | TSPO//CD14//PLSCR1//LCN2 |
| GO:0010038 | response to metal ion | Biological process | 8 | 316 | 172 | 13692 | 2.01530762437445 | 0.0462023547503992 | 0.324601159015625 | 1.33533588961609 | CYBRD1//ATP7A//TSPO//PTGS2//IGFBP2//CP//MGP//AQP1 |
| GO:0031016 | pancreas development | Biological process | 3 | 64 | 172 | 13692 | 3.73146802325581 | 0.0464943134027151 | 0.325089425769542 | 1.33260016127874 | BMP6//IGF2//ANXA1 |
| GO:0046824 | positive regulation of nucleocytoplasmic transport | Biological process | 3 | 64 | 172 | 13692 | 3.73146802325581 | 0.0464943134027151 | 0.325089425769542 | 1.33260016127874 | PTGS2//TLR3//TCF7L2 |
| GO:0071478 | cellular response to radiation | Biological process | 3 | 64 | 172 | 13692 | 3.73146802325581 | 0.0464943134027151 | 0.325089425769542 | 1.33260016127874 | AQP1//PTGS2//GADD45A |
| GO:0032990 | cell part morphogenesis | Biological process | 11 | 493 | 172 | 13692 | 1.77616868720223 | 0.0467919253447131 | 0.325642501238363 | 1.32982908454961 | NTRK1//BMP7//LMX1A//SEMA3C//LAMA2//TSPO//MMP2//FOXJ1//SPP1//ATP7A//DCDC2 |
| GO:0051093 | negative regulation of developmental process | Biological process | 11 | 493 | 172 | 13692 | 1.77616868720223 | 0.0467919253447131 | 0.325642501238363 | 1.32982908454961 | FOXJ1//TCF7L2//MESP1//CD74//MSX2//ZFHX3//BMP7//LMX1A//SPP1//MSX1//TSPO |
| GO:0009314 | response to radiation | Biological process | 8 | 317 | 172 | 13692 | 2.00895018707358 | 0.0469124357350535 | 0.325642501238363 | 1.32871201751477 | ANXA1//PLSCR1//AQP1//PTGS2//GADD45A//IGF2//NTRK1//MMP2 |
| GO:0070482 | response to oxygen levels | Biological process | 8 | 317 | 172 | 13692 | 2.00895018707358 | 0.0469124357350535 | 0.325642501238363 | 1.32871201751477 | ACE//MMP2//MMP14//PLOD2//LPAR1//AQP1//PTGS2//NDRG1 |
| GO:0022602 | ovulation cycle process | Biological process | 4 | 108 | 172 | 13692 | 2.94832041343669 | 0.047156307860267 | 0.325642501238363 | 1.32646020566947 | MMP2//MMP14//ADAMTS1//ANXA1 |
| GO:0042542 | response to hydrogen peroxide | Biological process | 4 | 108 | 172 | 13692 | 2.94832041343669 | 0.047156307860267 | 0.325642501238363 | 1.32646020566947 | AQP1//ANXA1//LCN2//MMP2 |
| GO:0009914 | hormone transport | Biological process | 6 | 208 | 172 | 13692 | 2.29628801431127 | 0.0475245641553577 | 0.325642501238363 | 1.3230818573303 | NMB//ANXA1//TRPV4//TCF7L2//AQP1//SLCO1A5 |
| GO:0051056 | regulation of small GTPase mediated signal transduction | Biological process | 7 | 262 | 172 | 13692 | 2.12684182496006 | 0.0475677997786804 | 0.325642501238363 | 1.32268693615792 | ACAP2//NTRK1//FOXJ1//RASGRF2//VAV3//ARHGEF26//LPAR1 |
| GO:0010623 | developmental programmed cell death | Biological process | 2 | 28 | 172 | 13692 | 5.68604651162791 | 0.0479104501137647 | 0.325642501238363 | 1.31956974897058 | BMP7//NTRK1 |
| GO:0030501 | positive regulation of bone mineralization | Biological process | 2 | 28 | 172 | 13692 | 5.68604651162791 | 0.0479104501137647 | 0.325642501238363 | 1.31956974897058 | BMP6//BMP7 |
| GO:0032371 | regulation of sterol transport | Biological process | 2 | 28 | 172 | 13692 | 5.68604651162791 | 0.0479104501137647 | 0.325642501238363 | 1.31956974897058 | PON1//APOA2 |
| GO:0032374 | regulation of cholesterol transport | Biological process | 2 | 28 | 172 | 13692 | 5.68604651162791 | 0.0479104501137647 | 0.325642501238363 | 1.31956974897058 | PON1//APOA2 |
| GO:0032410 | negative regulation of transporter activity | Biological process | 2 | 28 | 172 | 13692 | 5.68604651162791 | 0.0479104501137647 | 0.325642501238363 | 1.31956974897058 | STIM2//APOA2 |
| GO:0032722 | positive regulation of chemokine production | Biological process | 2 | 28 | 172 | 13692 | 5.68604651162791 | 0.0479104501137647 | 0.325642501238363 | 1.31956974897058 | CD74//TLR3 |
| GO:0042088 | T-helper 1 type immune response | Biological process | 2 | 28 | 172 | 13692 | 5.68604651162791 | 0.0479104501137647 | 0.325642501238363 | 1.31956974897058 | RT1-BB//XCL1 |
| GO:0042345 | regulation of NF-kappaB import into nucleus | Biological process | 2 | 28 | 172 | 13692 | 5.68604651162791 | 0.0479104501137647 | 0.325642501238363 | 1.31956974897058 | PTGS2//TLR3 |
| GO:0045620 | negative regulation of lymphocyte differentiation | Biological process | 2 | 28 | 172 | 13692 | 5.68604651162791 | 0.0479104501137647 | 0.325642501238363 | 1.31956974897058 | FOXJ1//CD74 |
| GO:0051926 | negative regulation of calcium ion transport | Biological process | 2 | 28 | 172 | 13692 | 5.68604651162791 | 0.0479104501137647 | 0.325642501238363 | 1.31956974897058 | STIM2//PTGS2 |
| GO:0051101 | regulation of DNA binding | Biological process | 3 | 65 | 172 | 13692 | 3.67406082289803 | 0.0483094709844167 | 0.327846317021181 | 1.31596771825363 | MSX1//PLSCR1//MSX2 |
| GO:0006898 | receptor-mediated endocytosis | Biological process | 4 | 109 | 172 | 13692 | 2.92127160230425 | 0.048492393704177 | 0.328579063368025 | 1.31432637750838 | GHR//DAB2//SCARA5//CXCL16 |
| GO:0001817 | regulation of cytokine production | Biological process | 8 | 320 | 172 | 13692 | 1.99011627906977 | 0.0490855403413693 | 0.331573203168818 | 1.30904642386093 | XCL1//APOA2//PTGS2//TLR3//TSPO//CD14//FOXJ1//CD74 |
| GO:0016049 | cell growth | Biological process | 8 | 320 | 172 | 13692 | 1.99011627906977 | 0.0490855403413693 | 0.331573203168818 | 1.30904642386093 | IGFBP2//LMX1A//CXCL16//DAB2//MSX1//RERG//SPP1//MMP2 |
| GO:0048523 | negative regulation of cellular process | Biological process | 40 | 2472 | 172 | 13692 | 1.28810115150147 | 0.0492555846443129 | 0.332209973970258 | 1.30754452165962 | MSX2//MSX1//FOXJ1//CDKN1C//ZFHX3//MOSPD1//TCF7L2//APOA2//CD74//GADD45A//MMP2//CD9//PTGS2//NTRK1//BMP7//RERG//TRPV4//LPAR1//DAB2//XCL1//MESP1//MDFIC//SOSTDC1//RT1-BB//AQP1//ANXA1//WFS1//KRT18//ATP7A//CLCF1//TSPO//LMX1A//C1QTNF3//PTPN3//NMB//OGN//SPP1//MMP14//RGD1305645 |
| GO:0007409 | axonogenesis | Biological process | 8 | 321 | 172 | 13692 | 1.98391653988263 | 0.0498242590541665 | 0.334676173961593 | 1.30255915105356 | NTRK1//BMP7//LMX1A//SEMA3C//LAMA2//TSPO//MMP2//SPP1 |
| GO:0046483 | heterocycle metabolic process | Biological process | 16 | 813 | 172 | 13692 | 1.56663520123573 | 0.0498637862260141 | 0.334676173961593 | 1.30221474800162 | GHR//HDC//AQP1//RERG//ATP7A//FMO2//SPTA1//ACAP2//NTRK1//RGN//FOXJ1//BDH2//VAV3//PON3//FOLR1//PLSCR1 |
| GO:0031329 | regulation of cellular catabolic process | Biological process | 9 | 379 | 172 | 13692 | 1.8903479167945 | 0.0499463018512942 | 0.334676173961593 | 1.30149666249373 | ABHD5//ACAP2//NTRK1//DAB2//RGN//FOXJ1//VAV3//PTPN3//PLSCR1 |
