# Supplementary material for: Gene Expression in the Hippocampus in a Rat Model of Premenstrual Dysphoric Disorder After Treatment With Baixiangdan Capsules
Source: Front Psychol. 2018 Nov 13;9:2065. doi: 10.3389/fpsyg.2018.02065 (PMC6242977; doi:10.3389/fpsyg.2018.02065)
Supplement: Supplementary file 3 [file Data_Sheet_3.ZIP › Data Analysis Folder/GO Analysis Report/BXD vs model (down)/CC_result(Rat).html]

| GO.ID | Term | Ontology | Count | Pop.Hits | List.Total | Pop.Total | Fold.Enrichment | Pvalue | FDR | Enrichment.Score | GENES |
| --- | --- | --- | --- | --- | --- | --- | --- | --- | --- | --- | --- |
| GO:0044421 | extracellular region part | Cellular component | 46 | 887 | 196 | 15288 | 4.04509582863585 | 1.66109874240094e-16 | 8.92010024669305e-14 | 15.7796045505677 | C1QTNF3//COL8A1//FMOD//MMP2//OMD//LGALS3//TGFBI//ANXA2//ADAMTS1//LAMA2//FBLN1//C6//CP//ACE//IGF2//TTR//GHR//MGP//SPP1//ANXA1//PTGDS//BMP6//APOA2//IGFBP2//GDF15//PCOLCE//CD14//PON1//ENPP2//BMP7//LCN2//XCL1//SOSTDC1//CCL6//OGN//SERPINB1A//CPXM2//SERPING1//SEMA3C//F5//AEBP1//PON3//RGD1305645//CXCL16//AQP1//CLCF1 |
| GO:0005615 | extracellular space | Cellular component | 39 | 707 | 196 | 15288 | 4.3026874115983 | 6.88895582463657e-15 | 1.84968463891492e-12 | 14.1618466001481 | APOA2//PON1//CLCF1//C6//CP//ACE//IGF2//TTR//GHR//MGP//SPP1//ANXA1//PTGDS//BMP6//IGFBP2//GDF15//PCOLCE//CD14//FMOD//MMP2//ENPP2//BMP7//TGFBI//LCN2//XCL1//SOSTDC1//CCL6//OGN//SERPINB1A//CPXM2//C1QTNF3//SERPING1//SEMA3C//F5//AEBP1//PON3//FBLN1//RGD1305645//CXCL16 |
| GO:0005576 | extracellular region | Cellular component | 51 | 1410 | 196 | 15288 | 2.82127659574468 | 4.81798197046383e-12 | 8.62418772713026e-10 | 11.317134829523 | C1QTNF3//COL8A1//FMOD//MMP2//OMD//LGALS3//TGFBI//ANXA2//ADAMTS1//LAMA2//FBLN1//C6//CP//ACE//IGF2//TTR//GHR//MGP//SPP1//ANXA1//PTGDS//BMP6//APOA2//IGFBP2//GDF15//PCOLCE//CD14//PON1//ENPP2//BMP7//LCN2//XCL1//SOSTDC1//CCL6//OGN//SERPINB1A//CPXM2//SERPING1//SEMA3C//F5//AEBP1//PON3//RGD1305645//CXCL16//AQP1//CLCF1//MDK//WFIKKN2//SCGB1C1//FIBIN//RGD1563000 |
| GO:0031012 | extracellular matrix | Cellular component | 20 | 350 | 196 | 15288 | 4.45714285714286 | 2.4646097595719e-08 | 3.30873860222528e-06 | 7.60825183609519 | C1QTNF3//COL8A1//FMOD//MMP2//OMD//LGALS3//TGFBI//ANXA2//ADAMTS1//LAMA2//FBLN1//MGP//PCOLCE//MMP14//BMP7//PLSCR1//OGN//CPXM2//OLFML2B//AEBP1 |
| GO:0044459 | plasma membrane part | Cellular component | 36 | 1426 | 196 | 15288 | 1.96914446002805 | 5.81076609982298e-05 | 0.00624076279120988 | 4.23576660582102 | MR1//CD74//RT1-DB1//RT1-BB//CP//FOLR1//GHR//NTRK1//SLCO1A5//PLSCR1//SCARA5//C6//C7//PTGS2//CLDN1//EPCAM//CLDN19//TRPV4//KCNIP2//KCNE2//ACE//CD9//TMEM123//PTPN3//AQP1//SLC16A8//SLC4A2//ATP7A//ANXA1//SLC22A8//SLC12A2//IGFBP2//DAB2//ANXA2//S100A6//CYBRD1 |
| GO:0071944 | cell periphery | Cellular component | 61 | 3081 | 196 | 15288 | 1.54430379746835 | 0.000170526810873166 | 0.0152621495731484 | 3.76820732987453 | ACE//GSTM2//ATP7A//GHR//AQP1//ANXA1//CD74//ANXA2//NTRK1//CD14//CLDN1//TRPV4//DAB2//BST1//MMP2//SLC12A2//LGALS3//RASGRF2//SLC5A5//LPAR1//CDH3//MAGT1//STIM2//PLSCR1//KCNE2//EPCAM//MFAP3//RT1-DA//VAV3//NDRG1//BAIAP2L1//MTUS1//RT1-BB//MTFR1//CDH19//CABP7//EPS8L2//EXOC2//MR1//RT1-DB1//CP//FOLR1//SLCO1A5//SCARA5//C6//C7//PTGS2//CLDN19//EPN3//KCNIP2//SPTA1//CD9//TMEM123//PTPN3//SLC16A8//SLC4A2//SLC22A8//IGFBP2//S100A6//CYBRD1//LAMA2 |
| GO:0042613 | MHC class II protein complex | Cellular component | 3 | 11 | 196 | 15288 | 21.2727272727273 | 0.00031740866142257 | 0.02434977874056 | 3.49838122642583 | CD74//RT1-DB1//RT1-BB |
| GO:0005886 | plasma membrane | Cellular component | 58 | 2986 | 196 | 15288 | 1.51507032819826 | 0.000443427395881402 | 0.0297650639485391 | 3.35317747876196 | MR1//CD74//RT1-DB1//RT1-BB//CP//FOLR1//GHR//NTRK1//SLCO1A5//PLSCR1//SCARA5//C6//C7//PTGS2//CLDN1//EPCAM//CLDN19//TRPV4//KCNIP2//KCNE2//ACE//CD9//TMEM123//PTPN3//AQP1//SLC16A8//SLC4A2//ATP7A//ANXA1//SLC22A8//SLC12A2//IGFBP2//DAB2//ANXA2//S100A6//CYBRD1//LAMA2//GSTM2//CD14//BST1//MMP2//LGALS3//RASGRF2//SLC5A5//LPAR1//CDH3//MAGT1//STIM2//MFAP3//RT1-DA//VAV3//NDRG1//BAIAP2L1//MTUS1//MTFR1//CDH19//CABP7//EPS8L2 |
| GO:0005578 | proteinaceous extracellular matrix | Cellular component | 11 | 262 | 196 | 15288 | 3.27480916030534 | 0.000575528694421691 | 0.0343398787671609 | 3.23993301865741 | C1QTNF3//COL8A1//ANXA2//ADAMTS1//TGFBI//LAMA2//FBLN1//FMOD//MMP2//OMD//LGALS3 |
| GO:0042611 | MHC protein complex | Cellular component | 4 | 32 | 196 | 15288 | 9.75 | 0.000711280842568995 | 0.038195781245955 | 3.14795888830569 | MR1//CD74//RT1-DB1//RT1-BB |
| GO:0005903 | brush border | Cellular component | 5 | 61 | 196 | 15288 | 6.39344262295082 | 0.00109429598310329 | 0.0534215402660424 | 2.96086519496531 | ATP7A//AQP1//SLCO1A5//CYBRD1//FOLR1 |
| GO:0005783 | endoplasmic reticulum | Cellular component | 24 | 974 | 196 | 15288 | 1.92197125256673 | 0.00157690305806372 | 0.0689422422351896 | 2.8021950046052 | CES1D//FKBP9//PTGS2//UGT1A6//RASGRF2//MGST1//ELOVL7//CREB3L1//PTGDS//MAGT1//DHRS7C//WFS1//PLOD2//FMO3//FMO2//ATP7A//MR1//MGP//CTSC//CD74//STIM2//KDELR3//DSE//FIBIN |
| GO:0005811 | lipid particle | Cellular component | 4 | 40 | 196 | 15288 | 7.8 | 0.00166899282878485 | 0.0689422422351896 | 2.77754552936381 | ANXA2//CES1D//ABHD5//PLIN3 |
| GO:0031526 | brush border membrane | Cellular component | 4 | 42 | 196 | 15288 | 7.42857142857143 | 0.0020037967735792 | 0.0768599191008593 | 2.69814632701072 | ATP7A//AQP1//SLCO1A5//CYBRD1 |
| GO:0016323 | basolateral plasma membrane | Cellular component | 10 | 273 | 196 | 15288 | 2.85714285714286 | 0.00279021926236295 | 0.0998898495925936 | 2.55436166744585 | TRPV4//AQP1//SLC16A8//SLC4A2//ATP7A//ANXA1//SLC22A8//SLC12A2//EPCAM//CLDN19 |
| GO:0005930 | axoneme | Cellular component | 4 | 47 | 196 | 15288 | 6.63829787234043 | 0.00303827295721841 | 0.101972036126643 | 2.51737321187921 | DNAH5//DNAH1//TEKT2//EFHC1 |
| GO:0044420 | extracellular matrix part | Cellular component | 7 | 154 | 196 | 15288 | 3.54545454545454 | 0.00372596533756515 | 0.117696669780734 | 2.42876118963683 | C1QTNF3//COL8A1//ANXA2//ADAMTS1//TGFBI//LAMA2//FBLN1 |
| GO:0005604 | basement membrane | Cellular component | 5 | 86 | 196 | 15288 | 4.53488372093023 | 0.00495469615898732 | 0.147815102076455 | 2.30498297296905 | LAMA2//ANXA2//ADAMTS1//TGFBI//FBLN1 |
| GO:0030286 | dynein complex | Cellular component | 3 | 28 | 196 | 15288 | 8.35714285714286 | 0.00536911799687057 | 0.151748229701026 | 2.27009705146111 | DNAH5//DYNLRB2//DNAH1 |
| GO:0005929 | cilium | Cellular component | 7 | 190 | 196 | 15288 | 2.87368421052632 | 0.011362835274342 | 0.302963781938522 | 1.94451328919145 | DNAH1//TEKT2//KLC3//ANXA1//TRPV4//CCDC40//EFHC1 |
| GO:0005875 | microtubule associated complex | Cellular component | 4 | 69 | 196 | 15288 | 4.52173913043478 | 0.0118477456624003 | 0.302963781938522 | 1.92636427746133 | DNAH5//DYNLRB2//KLC3//DNAH1 |
| GO:0044432 | endoplasmic reticulum part | Cellular component | 14 | 570 | 196 | 15288 | 1.91578947368421 | 0.0152537308327023 | 0.372329702598233 | 1.81662392143908 | CES1D//FKBP9//PTGS2//UGT1A6//RASGRF2//MGST1//ELOVL7//CREB3L1//MAGT1//DHRS7C//WFS1//PLOD2//FMO3//FMO2 |
| GO:0031225 | anchored to membrane | Cellular component | 4 | 80 | 196 | 15288 | 3.9 | 0.0194573974142661 | 0.454287930933082 | 1.71091525061506 | CP//FOLR1//CD14//BST1 |
| GO:0042995 | cell projection | Cellular component | 24 | 1211 | 196 | 15288 | 1.5458298926507 | 0.021664975207421 | 0.484753820266045 | 1.66424180361952 | DNAH5//S100A6//EFHC1//AQP1//FOLR1//ANXA1//TRPV4//CCDC40//KLC3//P2RX6//S100A4//ATP7A//PTGS2//NTRK1//HDC//KCNIP2//SLCO1A5//CYBRD1//FSCN2//DNAH1//TEKT2//LPAR1//LAMA2//SPP1 |
| GO:0005770 | late endosome | Cellular component | 5 | 127 | 196 | 15288 | 3.07086614173228 | 0.0238389045049239 | 0.500495514005826 | 1.62271370607937 | CD74//RT1-BB//RT1-DA//RT1-DB1//ATP7A |
| GO:0034364 | high-density lipoprotein particle | Cellular component | 2 | 19 | 196 | 15288 | 8.21052631578947 | 0.0242325574751424 | 0.500495514005826 | 1.61560074852328 | APOA2//PON1 |
| GO:0005789 | endoplasmic reticulum membrane | Cellular component | 12 | 508 | 196 | 15288 | 1.84251968503937 | 0.0308995617759516 | 0.61455795087726 | 1.51004767978667 | MAGT1//WFS1//PLOD2//FMO3//FMO2//DHRS7C//PTGS2//UGT1A6//RASGRF2//MGST1//ELOVL7//CREB3L1 |
| GO:0009986 | cell surface | Cellular component | 12 | 511 | 196 | 15288 | 1.83170254403131 | 0.0321168927083422 | 0.615956120870706 | 1.49326647911251 | ACE//CD9//CD74//RT1-BB//TMEM123//GHR//NTRK1//CD14//LPAR1//EPCAM//SCARA5//TLR3 |
| GO:0019861 | flagellum | Cellular component | 3 | 56 | 196 | 15288 | 4.17857142857143 | 0.0350265378126932 | 0.648594855359181 | 1.45560278826473 | KLC3//TEKT2//EFHC1 |
| GO:0042175 | nuclear outer membrane-endoplasmic reticulum membrane network | Cellular component | 12 | 522 | 196 | 15288 | 1.79310344827586 | 0.0368763314405756 | 0.660086332786303 | 1.43325229028548 | PTGS2//UGT1A6//RASGRF2//MGST1//ELOVL7//CREB3L1//MAGT1//WFS1//PLOD2//FMO3//FMO2//DHRS7C |
| GO:0042383 | sarcolemma | Cellular component | 4 | 101 | 196 | 15288 | 3.08910891089109 | 0.0409397580715073 | 0.702427207915649 | 1.38785472807605 | AQP1//ANXA1//ANXA2//LAMA2 |
| GO:0005771 | multivesicular body | Cellular component | 2 | 26 | 196 | 15288 | 6 | 0.0434503655819339 | 0.702427207915649 | 1.36200656514055 | CD74//RT1-BB |
| GO:0045177 | apical part of cell | Cellular component | 8 | 306 | 196 | 15288 | 2.03921568627451 | 0.0438530188477215 | 0.702427207915649 | 1.35800050437824 | CD9//AQP1//IGFBP2//DAB2//SLC12A2//EPCAM//SPP1//MGST1 |
| GO:0031226 | intrinsic to plasma membrane | Cellular component | 11 | 480 | 196 | 15288 | 1.7875 | 0.0454280344975317 | 0.702427207915649 | 1.34267605308945 | CP//FOLR1//GHR//NTRK1//SLCO1A5//PLSCR1//SCARA5//C6//C7//KCNIP2//KCNE2 |
| GO:0034358 | plasma lipoprotein particle | Cellular component | 2 | 27 | 196 | 15288 | 5.77777777777778 | 0.0465392733539039 | 0.702427207915649 | 1.33218040190132 | APOA2//PON1 |
| GO:0048471 | perinuclear region of cytoplasm | Cellular component | 10 | 426 | 196 | 15288 | 1.83098591549296 | 0.048162743960615 | 0.702427207915649 | 1.31728877812469 | S100A4//ATP7A//SPP1//PTGDS//ANXA2//S100A6//NDRG1//MOSPD1//EPN3//CABP7 |
| GO:0032994 | protein-lipid complex | Cellular component | 2 | 28 | 196 | 15288 | 5.57142857142857 | 0.0497062083813681 | 0.702427207915649 | 1.3035893638345 | APOA2//PON1 |
| GO:0046658 | anchored to plasma membrane | Cellular component | 2 | 28 | 196 | 15288 | 5.57142857142857 | 0.0497062083813681 | 0.702427207915649 | 1.3035893638345 | CP//FOLR1 |
