# Supplementary material for: Gene Expression in the Hippocampus in a Rat Model of Premenstrual Dysphoric Disorder After Treatment With Baixiangdan Capsules
Source: Front Psychol. 2018 Nov 13;9:2065. doi: 10.3389/fpsyg.2018.02065 (PMC6242977; doi:10.3389/fpsyg.2018.02065)
Supplement: Supplementary file 3 [file Data_Sheet_3.ZIP › Data Analysis Folder/GO Analysis Report/BXD vs model (down)/MF_result(Rat).html]

| GO.ID | Term | Ontology | Count | Pop.Hits | List.Total | Pop.Total | Fold.Enrichment | Pvalue | FDR | Enrichment.Score | GENES |
| --- | --- | --- | --- | --- | --- | --- | --- | --- | --- | --- | --- |
| GO:0005509 | calcium ion binding | Molecular function | 23 | 531 | 172 | 14392 | 3.62431568344063 | 8.79819216444845e-08 | 7.89197837151026e-05 | 7.05560655667512 | S100A4//RGN//MGP//ANXA1//ANXA2//KCNIP2//MMP14//PON1//ENPP2//S100A6//STIM2//PLSCR1//SPTA1//CAPSL//FKBP9//EFHC1//FBLN1//PLS1//CDH19//CABP7//FKBP14//CDHR4//CALML4 |
| GO:0005515 | protein binding | Molecular function | 85 | 4586 | 172 | 14392 | 1.55087779794927 | 1.11446727943586e-06 | 0.000499838574826983 | 5.95293267780649 | DNAJC22//CD74//PTPN3//SERPING1//LPAR1//MMP2//LCN2//MSX1//CP//ACE//TRPV4//WIPF3//BAIAP2L1//PLS1//ANXA1//FSCN2//ANXA2//LAMA2//CLCF1//GHR//NTRK1//SLC22A8//AQP1//SPP1//BMP6//GDF15//PLSCR1//VAV3//IGF2//SMURF2//CD9//MMP14//TTR//RASGRF2//AEBP1//PCOLCE//IGFBP2//S100A6//XCL1//CCL6//CXCL16//TCF7L2//KLC3//NDRG1//DAB2//EFHC1//MDK//PDLIM3//MSX2//FOXJ1//RT1-BB//MDFIC//S100A4//P2RX6//CTSC//KCNIP2//CLDN1//MGST1//C1QTNF3//CRB3//PTGS2//UGT1A6//ZFHX3//EXOC2//LGALS3//LLGL2//SEMA3C//WFS1//NMB//HADHA//ATP7A//APOA2//GSTM2//HDC//PON1//OLFML2B//PON3//SPTA1//CREB3L1//MGP//PRRX2//TSPO//CD14//CDKN1C//ABHD5 |
| GO:0005488 | binding | Molecular function | 132 | 9220 | 172 | 14392 | 1.19794178479544 | 0.000223026696051901 | 0.062906172726495 | 3.65164314926525 | DNAJC22//HADHA//BST1//PTGR1//DHRS7C//RBM47//TSPO//IGF2//S100A4//CD9//AQP1//PTGS2//KCNIP2//NTRK1//CD14//TRPV4//DAB2//EXOC2//CDKN1C//ABHD5//MSX2//T//RGN//MGP//ANXA1//ANXA2//MMP14//PON1//ENPP2//S100A6//STIM2//PLSCR1//SPTA1//CAPSL//FKBP9//EFHC1//FBLN1//PLS1//CDH19//CABP7//FKBP14//CDHR4//CALML4//CD74//PTPN3//SERPING1//LPAR1//MMP2//LCN2//MSX1//EIF2C4//ZIC4//AEBP1//SP140//CREB3L1//TCF7L2//HMGN5//FOXJ1//TLR3//CP//ACE//WIPF3//BAIAP2L1//FSCN2//LAMA2//CLCF1//RT1-BB//LGALS3//GHR//SLC22A8//SPP1//BMP6//GDF15//VAV3//SMURF2//TTR//PTGDS//NT5DC2//CYBRD1//ARHGAP29//PON3//PLOD2//ATP7A//RASGRF2//PCOLCE//IGFBP2//P2RX6//SPEF2//DNAH1//RGD1560691//AK7//RHOD//RERG//FOLR1//APOA2//ARHGEF26//ACAP2//EPN3//XCL1//CCL6//CXCL16//KLC3//NDRG1//MDK//PDLIM3//MDFIC//ADAMTS1//BMP7//LMX1A//RNF152//CPXM2//ZFHX3//CTSC//CLDN1//MGST1//C1QTNF3//MESP1//HDC//FMO3//CRB3//UGT1A6//TGFBI//OLFML2B//LLGL2//SEMA3C//WFS1//NMB//GSTM2//PRRX2//KDELR3//FMO2//BDH2 |
| GO:0055102 | lipase inhibitor activity | Molecular function | 3 | 12 | 172 | 14392 | 20.9186046511628 | 0.000340890720719565 | 0.062906172726495 | 3.46738482040542 | ANXA1//ANXA2//APOA2 |
| GO:0042802 | identical protein binding | Molecular function | 22 | 827 | 172 | 14392 | 2.22592165574646 | 0.00035064756257801 | 0.062906172726495 | 3.45512917562332 | GSTM2//HDC//S100A4//GHR//ANXA1//APOA2//PTGS2//NTRK1//PON1//S100A6//UGT1A6//LCN2//MGST1//XCL1//OLFML2B//PON3//P2RX6//CTSC//KCNIP2//CLDN1//C1QTNF3//SMURF2 |
| GO:0046983 | protein dimerization activity | Molecular function | 22 | 839 | 172 | 14392 | 2.19408487401946 | 0.000426941989662978 | 0.0638278274546152 | 3.36963113031634 | GSTM2//HDC//S100A4//GHR//ANXA1//APOA2//PTGS2//NTRK1//PON1//S100A6//UGT1A6//LCN2//MGST1//XCL1//OLFML2B//PON3//TTR//BMP6//SPTA1//RT1-BB//CLCF1//CREB3L1 |
| GO:0005102 | receptor binding | Molecular function | 25 | 1052 | 172 | 14392 | 1.98846051817137 | 0.000732006159074407 | 0.0820761905862179 | 3.13548526478754 | AQP1//NTRK1//SPP1//BMP6//GDF15//CLCF1//PLSCR1//VAV3//IGF2//SMURF2//CD9//MMP14//TTR//XCL1//CCL6//CXCL16//MDK//SEMA3C//NMB//ACE//APOA2//TCF7L2//CD74//S100A4//LAMA2 |
| GO:0005215 | transporter activity | Molecular function | 25 | 1052 | 172 | 14392 | 1.98846051817137 | 0.000732006159074407 | 0.0820761905862179 | 3.13548526478754 | ATP7A//COX8B//P2RX6//TRPM3//AQP1//KCNIP2//KCNE2//TRPV4//APOA2//SLC4A2//SLC22A8//SLCO1A5//SLC5A5//SLC12A2//FOLR1//S100A6//MAGT1//SLC44A1//SLC16A8//SLC12A7//PLSCR1//SLC2A12//PTGDS//WFS1//LCN2 |
| GO:0005125 | cytokine activity | Molecular function | 7 | 130 | 172 | 14392 | 4.50554561717353 | 0.000949506666760809 | 0.0946341644538273 | 3.02250198160915 | XCL1//CCL6//CXCL16//SPP1//BMP6//GDF15//CLCF1 |
| GO:0042803 | protein homodimerization activity | Molecular function | 16 | 558 | 172 | 14392 | 2.39926648328749 | 0.00107471237748466 | 0.096401700260374 | 2.96870774931568 | GSTM2//HDC//S100A4//GHR//ANXA1//APOA2//PTGS2//NTRK1//PON1//S100A6//UGT1A6//LCN2//MGST1//XCL1//OLFML2B//PON3 |
| GO:0052689 | carboxylic ester hydrolase activity | Molecular function | 6 | 104 | 172 | 14392 | 4.82737030411449 | 0.0015417255566511 | 0.125720711301458 | 2.81199292838614 | PON1//PON3//CES1D//RGN//ENPP2//ABHD5 |
| GO:0022892 | substrate-specific transporter activity | Molecular function | 21 | 880 | 172 | 14392 | 1.99677589852008 | 0.00189674450520741 | 0.141781651764254 | 2.72199116539573 | ATP7A//COX8B//P2RX6//TRPM3//AQP1//KCNIP2//KCNE2//TRPV4//APOA2//SLC4A2//SLC22A8//SLCO1A5//SLC5A5//SLC12A2//S100A6//MAGT1//SLC44A1//SLC16A8//SLC12A7//PLSCR1//SLC2A12 |
| GO:0022891 | substrate-specific transmembrane transporter activity | Molecular function | 19 | 772 | 172 | 14392 | 2.05934449933727 | 0.00222021540701027 | 0.153194863083709 | 2.6536048879198 | ATP7A//COX8B//P2RX6//TRPM3//AQP1//KCNIP2//KCNE2//TRPV4//SLC4A2//SLC22A8//SLCO1A5//SLC5A5//SLC12A2//S100A6//MAGT1//SLC44A1//SLC16A8//SLC12A7//SLC2A12 |
| GO:0022857 | transmembrane transporter activity | Molecular function | 20 | 839 | 172 | 14392 | 1.99462261274496 | 0.00246690963325044 | 0.158058424358975 | 2.60784675910349 | ATP7A//COX8B//P2RX6//TRPM3//AQP1//KCNIP2//KCNE2//TRPV4//SLC4A2//SLC22A8//SLCO1A5//SLC5A5//SLC12A2//FOLR1//S100A6//MAGT1//SLC44A1//SLC16A8//SLC12A7//SLC2A12 |
| GO:0015291 | secondary active transmembrane transporter activity | Molecular function | 7 | 158 | 172 | 14392 | 3.70709449514277 | 0.00290224935705943 | 0.173554511552154 | 2.53726527634261 | SLC4A2//SLC22A8//SLC5A5//SLC12A2//SLC16A8//SLCO1A5//SLC12A7 |
| GO:0015296 | anion:cation symporter activity | Molecular function | 3 | 26 | 172 | 14392 | 9.65474060822898 | 0.00356310240645648 | 0.199756428661966 | 2.44817169549354 | SLC5A5//SLC12A2//SLC12A7 |
| GO:0016504 | peptidase activator activity | Molecular function | 3 | 27 | 172 | 14392 | 9.29715762273902 | 0.00397362817382136 | 0.209155504469036 | 2.40081277376687 | PCOLCE//MMP14//FBLN1 |
| GO:0008509 | anion transmembrane transporter activity | Molecular function | 6 | 127 | 172 | 14392 | 3.95312213880242 | 0.00419710042412782 | 0.209155504469036 | 2.37705063928916 | SLC4A2//SLC22A8//SLC5A5//SLC12A2//SLCO1A5//SLC12A7 |
| GO:0015075 | ion transmembrane transporter activity | Molecular function | 16 | 648 | 172 | 14392 | 2.06603502727534 | 0.00478116927562405 | 0.225721517907093 | 2.32046587998795 | ATP7A//COX8B//P2RX6//TRPM3//AQP1//KCNIP2//KCNE2//TRPV4//SLC4A2//SLC22A8//SLC5A5//SLC12A2//SLCO1A5//MAGT1//SLC12A7//S100A6 |
| GO:0043167 | ion binding | Molecular function | 48 | 2840 | 172 | 14392 | 1.41421552571241 | 0.0058456658706822 | 0.251890480270239 | 2.23316601187732 | S100A4//RGN//MGP//ANXA1//ANXA2//KCNIP2//MMP14//PON1//ENPP2//S100A6//STIM2//PLSCR1//SPTA1//CAPSL//FKBP9//EFHC1//FBLN1//PLS1//CDH19//CABP7//FKBP14//CDHR4//CALML4//NT5DC2//VAV3//CYBRD1//ARHGAP29//PON3//LCN2//PLOD2//CP//ATP7A//ACE//ADAMTS1//MMP2//PDLIM3//PTGR1//LMX1A//RNF152//CPXM2//AEBP1//ZFHX3//ZIC4//SP140//ACAP2//PTGS2//APOA2//CTSC |
| GO:0008009 | chemokine activity | Molecular function | 3 | 31 | 172 | 14392 | 8.09752438109527 | 0.00589710154478821 | 0.251890480270239 | 2.22936139384989 | XCL1//CCL6//CXCL16 |
| GO:0004180 | carboxypeptidase activity | Molecular function | 3 | 34 | 172 | 14392 | 7.38303693570451 | 0.00764820217616318 | 0.291947841747394 | 2.11644064023621 | CPXM2//AEBP1//ACE |
| GO:0008324 | cation transmembrane transporter activity | Molecular function | 13 | 507 | 172 | 14392 | 2.14549791293977 | 0.00791078825625861 | 0.291947841747394 | 2.10178023985413 | ATP7A//COX8B//P2RX6//AQP1//KCNE2//TRPV4//TRPM3//KCNIP2//SLC5A5//SLC12A2//MAGT1//SLC12A7//SLC22A8 |
| GO:0005452 | inorganic anion exchanger activity | Molecular function | 2 | 12 | 172 | 14392 | 13.9457364341085 | 0.00866289913298336 | 0.291947841747394 | 2.06233674230696 | SLC4A2//SLC22A8 |
| GO:0005527 | macrolide binding | Molecular function | 2 | 12 | 172 | 14392 | 13.9457364341085 | 0.00866289913298336 | 0.291947841747394 | 2.06233674230696 | FKBP9//FKBP14 |
| GO:0005528 | FK506 binding | Molecular function | 2 | 12 | 172 | 14392 | 13.9457364341085 | 0.00866289913298336 | 0.291947841747394 | 2.06233674230696 | FKBP9//FKBP14 |
| GO:0043169 | cation binding | Molecular function | 47 | 2827 | 172 | 14392 | 1.39112050739958 | 0.0087877276780152 | 0.291947841747394 | 2.05612340984982 | S100A4//RGN//MGP//ANXA1//ANXA2//KCNIP2//MMP14//PON1//ENPP2//S100A6//STIM2//PLSCR1//SPTA1//CAPSL//FKBP9//EFHC1//FBLN1//PLS1//CDH19//CABP7//FKBP14//CDHR4//CALML4//NT5DC2//VAV3//CYBRD1//ARHGAP29//PON3//LCN2//PLOD2//CP//ATP7A//ACE//ADAMTS1//MMP2//PDLIM3//PTGR1//LMX1A//RNF152//CPXM2//AEBP1//ZFHX3//ZIC4//SP140//ACAP2//PTGS2//APOA2 |
| GO:0022804 | active transmembrane transporter activity | Molecular function | 9 | 297 | 172 | 14392 | 2.53558844256519 | 0.00947020616356986 | 0.303384818882934 | 2.02364056643219 | ATP7A//SLC4A2//SLC22A8//SLC5A5//SLC12A2//SLC44A1//SLC16A8//SLCO1A5//SLC12A7 |
| GO:0046872 | metal ion binding | Molecular function | 46 | 2783 | 172 | 14392 | 1.38304824139919 | 0.0106807608072552 | 0.330366980831307 | 1.97139781073214 | S100A4//RGN//MGP//ANXA1//ANXA2//KCNIP2//MMP14//PON1//ENPP2//S100A6//STIM2//PLSCR1//SPTA1//CAPSL//FKBP9//EFHC1//FBLN1//PLS1//CDH19//CABP7//FKBP14//CDHR4//CALML4//LCN2//PLOD2//CP//ATP7A//ACE//ADAMTS1//MMP2//PDLIM3//PTGR1//LMX1A//RNF152//CPXM2//AEBP1//ZFHX3//ZIC4//SP140//ACAP2//PTGS2//NT5DC2//VAV3//CYBRD1//ARHGAP29//PON3 |
| GO:0050840 | extracellular matrix binding | Molecular function | 3 | 39 | 172 | 14392 | 6.43649373881932 | 0.0111843090991448 | 0.33441084206443 | 1.95139083889829 | SPP1//TGFBI//OLFML2B |
| GO:0008514 | organic anion transmembrane transporter activity | Molecular function | 2 | 14 | 172 | 14392 | 11.953488372093 | 0.0117587582928173 | 0.336004560006361 | 1.92963853668245 | SLCO1A5//SLC22A8 |
| GO:0048306 | calcium-dependent protein binding | Molecular function | 3 | 40 | 172 | 14392 | 6.27558139534884 | 0.0119867847493908 | 0.336004560006361 | 1.92129729336957 | S100A4//MGP//S100A6 |
| GO:0008237 | metallopeptidase activity | Molecular function | 6 | 160 | 172 | 14392 | 3.13779069767442 | 0.0124967959063886 | 0.339685634182745 | 1.90320132287577 | CPXM2//AEBP1//ADAMTS1//MMP2//MMP14//ACE |
| GO:0015301 | anion:anion antiporter activity | Molecular function | 2 | 15 | 172 | 14392 | 11.1565891472868 | 0.0134621586821894 | 0.350824070358585 | 1.87088529459987 | SLC4A2//SLC22A8 |
| GO:0042379 | chemokine receptor binding | Molecular function | 3 | 42 | 172 | 14392 | 5.97674418604651 | 0.0136887875836683 | 0.350824070358585 | 1.86363501563811 | XCL1//CCL6//CXCL16 |
| GO:0004806 | triglyceride lipase activity | Molecular function | 2 | 16 | 172 | 14392 | 10.4593023255814 | 0.0152656500720937 | 0.380369114296335 | 1.81628469698525 | CES1D//ABHD5 |
| GO:0043168 | anion binding | Molecular function | 2 | 17 | 172 | 14392 | 9.84404924760602 | 0.0171666256747632 | 0.416174681898989 | 1.76531506272189 | ACE//CTSC |
| GO:0031406 | carboxylic acid binding | Molecular function | 6 | 177 | 172 | 14392 | 2.83642096964919 | 0.0196231485171318 | 0.463209584733348 | 1.70723130919293 | PTGDS//FOLR1//HDC//FMO3//PLOD2//MGST1 |
| GO:0030234 | enzyme regulator activity | Molecular function | 16 | 766 | 172 | 14392 | 1.747768534823 | 0.0211860335793419 | 0.487278772324864 | 1.67395034370665 | ANXA1//ANXA2//WFIKKN2//SERPINB1A//SERPING1//VAV3//RASGRF2//ARHGEF26//ARHGAP29//ACAP2//PCOLCE//MMP14//FBLN1//IGF2//APOA2//RGN |
| GO:0038024 | cargo receptor activity | Molecular function | 3 | 50 | 172 | 14392 | 5.02046511627907 | 0.0218112035209895 | 0.48911623895819 | 1.66132036981315 | CXCL16//ENPP2//SCARA5 |
| GO:0004181 | metallocarboxypeptidase activity | Molecular function | 2 | 20 | 172 | 14392 | 8.36744186046512 | 0.0234290960738223 | 0.49472937335331 | 1.63024446674763 | CPXM2//AEBP1 |
| GO:0000982 | RNA polymerase II core promoter proximal region sequence-specific DNA binding transcription factor activity | Molecular function | 4 | 91 | 172 | 14392 | 3.67799642218247 | 0.0235742809419885 | 0.49472937335331 | 1.62756154515221 | PLSCR1//CREB3L1//MSX2//MSX1 |
| GO:0046982 | protein heterodimerization activity | Molecular function | 9 | 347 | 172 | 14392 | 2.17022987735406 | 0.0237161238062345 | 0.49472937335331 | 1.62495629115621 | GSTM2//S100A4//TTR//BMP6//APOA2//UGT1A6//SPTA1//RT1-BB//CLCF1 |
| GO:0003777 | microtubule motor activity | Molecular function | 3 | 53 | 172 | 14392 | 4.73628784554629 | 0.0254044518684951 | 0.510428938214563 | 1.59509017107669 | DNAH1//KLC3//DNAH5 |
| GO:0015103 | inorganic anion transmembrane transporter activity | Molecular function | 4 | 94 | 172 | 14392 | 3.56061355764473 | 0.0261758429853622 | 0.510428938214563 | 1.58209932309722 | SLC12A2//SLC4A2//SLC5A5//SLC12A7 |
| GO:0016597 | amino acid binding | Molecular function | 4 | 94 | 172 | 14392 | 3.56061355764473 | 0.0261758429853622 | 0.510428938214563 | 1.58209932309722 | FOLR1//MGST1//HDC//FMO3 |
| GO:0017124 | SH3 domain binding | Molecular function | 4 | 95 | 172 | 14392 | 3.52313341493268 | 0.0270798290686231 | 0.516821418607551 | 1.56735408130108 | PLSCR1//WIPF3//CRB3//BAIAP2L1 |
| GO:0061134 | peptidase regulator activity | Molecular function | 6 | 192 | 172 | 14392 | 2.61482558139535 | 0.0278701400814895 | 0.520823242772835 | 1.55486084842107 | WFIKKN2//SERPINB1A//SERPING1//PCOLCE//MMP14//FBLN1 |
| GO:0005126 | cytokine receptor binding | Molecular function | 6 | 193 | 172 | 14392 | 2.60127726232076 | 0.0284901373364126 | 0.521543942668614 | 1.54530545724342 | CLCF1//SMURF2//NTRK1//XCL1//CCL6//CXCL16 |
| GO:0005089 | Rho guanyl-nucleotide exchange factor activity | Molecular function | 3 | 56 | 172 | 14392 | 4.48255813953488 | 0.0292963804988293 | 0.525577066148998 | 1.53318603242698 | VAV3//RASGRF2//ARHGEF26 |
| GO:0008028 | monocarboxylic acid transmembrane transporter activity | Molecular function | 2 | 23 | 172 | 14392 | 7.27603640040445 | 0.03047954383479 | 0.529333850630562 | 1.51599153705409 | SLCO1A5//SLC16A8 |
| GO:0008289 | lipid binding | Molecular function | 13 | 608 | 172 | 14392 | 1.7890911872705 | 0.0306860203264094 | 0.529333850630562 | 1.51305943161869 | TSPO//PTGDS//ANXA1//APOA2//PON1//RASGRF2//LPAR1//VAV3//ARHGEF26//ACAP2//ANXA2//PTGS2//EPN3 |
| GO:0042277 | peptide binding | Molecular function | 5 | 148 | 172 | 14392 | 2.82683846637335 | 0.0324705454105414 | 0.538098975268784 | 1.488510416431 | RT1-BB//GHR//MGST1//KDELR3//ACE |
| GO:0005544 | calcium-dependent phospholipid binding | Molecular function | 2 | 24 | 172 | 14392 | 6.97286821705426 | 0.0329938056184873 | 0.538098975268784 | 1.48156758855323 | ANXA1//ANXA2 |
| GO:0016628 | oxidoreductase activity, acting on the CH-CH group of donors, NAD or NADP as acceptor | Molecular function | 2 | 24 | 172 | 14392 | 6.97286821705426 | 0.0329938056184873 | 0.538098975268784 | 1.48156758855323 | PTGR1//BDH2 |
| GO:0005154 | epidermal growth factor receptor binding | Molecular function | 2 | 26 | 172 | 14392 | 6.43649373881932 | 0.038254944606244 | 0.599479057519441 | 1.41731242256474 | PLSCR1//VAV3 |
| GO:0015293 | symporter activity | Molecular function | 4 | 106 | 172 | 14392 | 3.15752523036419 | 0.0382558139955747 | 0.599479057519441 | 1.41730255281581 | SLC5A5//SLC12A2//SLC12A7//SLC16A8 |
| GO:0008201 | heparin binding | Molecular function | 4 | 107 | 172 | 14392 | 3.12801564877201 | 0.0393847410026554 | 0.599479057519441 | 1.40467200614204 | PCOLCE//ADAMTS1//MDK//BMP7 |
| GO:0003779 | actin binding | Molecular function | 7 | 264 | 172 | 14392 | 2.21863988724454 | 0.0394306180531182 | 0.599479057519441 | 1.40416641554775 | ANXA2//TRPV4//FSCN2//ACE//WIPF3//BAIAP2L1//PLS1 |
| GO:0051117 | ATPase binding | Molecular function | 2 | 27 | 172 | 14392 | 6.19810508182601 | 0.0409974499208378 | 0.612911876316525 | 1.38724315595712 | WFS1//PTPN3 |
| GO:0035326 | enhancer binding | Molecular function | 2 | 28 | 172 | 14392 | 5.97674418604651 | 0.0438117322293274 | 0.631327327525568 | 1.35840957533664 | T//MESP1 |
| GO:0051015 | actin filament binding | Molecular function | 3 | 66 | 172 | 14392 | 3.80338266384778 | 0.0443972910726886 | 0.631327327525568 | 1.35264352781498 | ANXA2//TRPV4//FSCN2 |
| GO:0001871 | pattern binding | Molecular function | 5 | 162 | 172 | 14392 | 2.58254378409417 | 0.0450445361891152 | 0.631327327525568 | 1.34635788053011 | PCOLCE//ADAMTS1//MDK//BMP7//ENPP2 |
| GO:0030247 | polysaccharide binding | Molecular function | 5 | 162 | 172 | 14392 | 2.58254378409417 | 0.0450445361891152 | 0.631327327525568 | 1.34635788053011 | PCOLCE//ADAMTS1//MDK//BMP7//ENPP2 |
| GO:0016709 | oxidoreductase activity, acting on paired donors, with incorporation or reduction of molecular oxygen, NADH or NADPH as one donor, and incorporation of one atom of oxygen | Molecular function | 2 | 29 | 172 | 14392 | 5.77064955894146 | 0.0466957061724869 | 0.634637097526072 | 1.3307230524388 | FMO3//FMO2 |
| GO:0042169 | SH2 domain binding | Molecular function | 2 | 29 | 172 | 14392 | 5.77064955894146 | 0.0466957061724869 | 0.634637097526072 | 1.3307230524388 | GHR//TRPV4 |
| GO:0000981 | sequence-specific DNA binding RNA polymerase II transcription factor activity | Molecular function | 6 | 219 | 172 | 14392 | 2.29244982478496 | 0.0478973023264726 | 0.641251943087253 | 1.31968894624555 | MSX2//MSX1//PLSCR1//CREB3L1//FOXJ1//T |
| GO:0042562 | hormone binding | Molecular function | 3 | 69 | 172 | 14392 | 3.63801820020222 | 0.0495511026924024 | 0.643012928888934 | 1.3049466764381 | TSPO//GHR//TTR |
