# Supplementary material for: Gene Expression in the Hippocampus in a Rat Model of Premenstrual Dysphoric Disorder After Treatment With Baixiangdan Capsules
Source: Front Psychol. 2018 Nov 13;9:2065. doi: 10.3389/fpsyg.2018.02065 (PMC6242977; doi:10.3389/fpsyg.2018.02065)
Supplement: Supplementary file 3 [file Data_Sheet_3.ZIP › Data Analysis Folder/GO Analysis Report/BXD vs model (up)/BP_result(Rat).html]

| GO.ID | Term | Ontology | Count | Pop.Hits | List.Total | Pop.Total | Fold.Enrichment | Pvalue | FDR | Enrichment.Score | GENES |
| --- | --- | --- | --- | --- | --- | --- | --- | --- | --- | --- | --- |
| GO:0050877 | neurological system process | Biological process | 22 | 2227 | 55 | 13692 | 2.45927256398743 | 2.16906078770864e-05 | 0.0478682073674058 | 4.66372827675644 | SLC17A6//CHRNA7//GRM8//CHRNA4//COL2A1//RXRG//CCKBR//TULP1//OLR1401//OLR19//OLR98//OLR153//OLR200//OLR305//OLR375//OLR1341//OLR25//OLR278//OLR7//OLR1585//CCL3//CBLN2 |
| GO:0003008 | system process | Biological process | 24 | 2589 | 55 | 13692 | 2.30772147898452 | 2.18376858427946e-05 | 0.0478682073674058 | 4.66079338606594 | SLC17A6//CHRNA7//GRM8//CHRNA4//COL2A1//POMC//CYP11B1//RXRG//CCKBR//TULP1//OLR1401//OLR19//OLR98//OLR153//OLR200//OLR305//OLR375//OLR1341//OLR25//OLR278//OLR7//OLR1585//CCL3//CBLN2 |
| GO:0007600 | sensory perception | Biological process | 18 | 1660 | 55 | 13692 | 2.69940854326396 | 5.0230765299368e-05 | 0.06388092038989 | 4.2990302050226 | COL2A1//GRM8//CHRNA4//TULP1//OLR1401//OLR19//OLR98//OLR153//OLR200//OLR305//OLR375//OLR1341//OLR25//OLR278//OLR7//OLR1585//CCL3//CCKBR |
| GO:0023052 | signaling | Biological process | 33 | 4621 | 55 | 13692 | 1.77779701363341 | 5.82855113046442e-05 | 0.06388092038989 | 4.23443938936552 | CCL3//MET//CHRNA7//SLC17A6//POMC//CHRNA4//RASSF5//GRB7//SRGAP1//SSTR1//OLR1401//OLR19//OLR98//OLR153//OLR200//OLR305//OLR375//OLR1341//OLR25//OLR278//OLR7//OLR1585//GRM8//GRP//CNTN6//ITGA10//TULP1//GDF6//BMPER//RXRG//CCKBR//RGS16//CBLN2 |
| GO:0007165 | signal transduction | Biological process | 31 | 4238 | 55 | 13692 | 1.82097902097902 | 8.01214285882523e-05 | 0.0702504685861796 | 4.09625131571316 | CCL3//MET//CHRNA7//RASSF5//SSTR1//OLR1401//OLR19//OLR98//OLR153//OLR200//OLR305//OLR375//OLR1341//OLR25//OLR278//OLR7//OLR1585//GRM8//POMC//GRP//CNTN6//ITGA10//SRGAP1//TULP1//GDF6//BMPER//RGS16//RXRG//CCKBR//CHRNA4//GRB7 |
| GO:0007154 | cell communication | Biological process | 33 | 4745 | 55 | 13692 | 1.73133825079031 | 0.000105378378667867 | 0.0769964686799881 | 3.97724848768594 | CCL3//MET//CHRNA7//SLC17A6//POMC//CHRNA4//RASSF5//GRB7//SRGAP1//SSTR1//OLR1401//OLR19//OLR98//OLR153//OLR200//OLR305//OLR375//OLR1341//OLR25//OLR278//OLR7//OLR1585//GRM8//GRP//CNTN6//ITGA10//TULP1//GDF6//BMPER//RXRG//CCKBR//RGS16//CBLN2 |
| GO:0007186 | G-protein coupled receptor signaling pathway | Biological process | 17 | 1738 | 55 | 13692 | 2.43502458416152 | 0.000312209937582414 | 0.1955326237659 | 3.50555327754356 | GRM8//POMC//SSTR1//GRP//RGS16//OLR1401//OLR19//OLR98//OLR153//OLR200//OLR305//OLR375//OLR1341//OLR25//OLR278//OLR7//OLR1585 |
| GO:0007166 | cell surface receptor signaling pathway | Biological process | 23 | 2870 | 55 | 13692 | 1.9950332594235 | 0.00036623920651739 | 0.20069908517153 | 3.43623516559759 | SSTR1//OLR1401//OLR19//OLR98//OLR153//OLR200//OLR305//OLR375//OLR1341//OLR25//OLR278//OLR7//OLR1585//GRM8//POMC//GRP//CNTN6//ITGA10//GDF6//BMPER//CCL3//RGS16//MET |
| GO:0051716 | cellular response to stimulus | Biological process | 33 | 5068 | 55 | 13692 | 1.62099447513812 | 0.000437061802833957 | 0.212897660402674 | 3.35945714716697 | CCL3//MET//CHRNA7//POMC//CHRNA4//RASSF5//GRB7//SRGAP1//SSTR1//OLR1401//OLR19//OLR98//OLR153//OLR200//OLR305//OLR375//OLR1341//OLR25//OLR278//OLR7//OLR1585//GRM8//GRP//CNTN6//ITGA10//TULP1//GDF6//BMPER//RXRG//CYP11B1//RGS16//CCKBR//COL2A1 |
| GO:0065007 | biological regulation | Biological process | 44 | 8017 | 55 | 13692 | 1.36629661968317 | 0.00063970644875794 | 0.280447307135481 | 3.19401927122105 | MET//FEZF2//CCL3//CHRNA7//SLC17A6//CHRNA4//NOV//COL2A1//POMC//RASSF5//GRB7//SRGAP1//RXRG//CYP11B1//SSTR1//OLR1401//OLR19//OLR98//OLR153//OLR200//OLR305//OLR375//OLR1341//OLR25//OLR278//OLR7//OLR1585//GRM8//CCKBR//GRP//CNTN6//ITGA10//TULP1//BMPER//GDF6//IL12RB2//LCP1//RGS16//CHRNE//MAP3K9//DKKL1//SPIC//KRT4//CBLN2 |
| GO:0050794 | regulation of cellular process | Biological process | 41 | 7207 | 55 | 13692 | 1.41622917113412 | 0.000704308934846561 | 0.280699124578848 | 3.15223680212315 | MET//FEZF2//CCL3//CHRNA7//NOV//POMC//CHRNA4//RASSF5//GRB7//SRGAP1//RXRG//SSTR1//OLR1401//OLR19//OLR98//OLR153//OLR200//OLR305//OLR375//OLR1341//OLR25//OLR278//OLR7//OLR1585//GRM8//GRP//CNTN6//ITGA10//TULP1//CCKBR//BMPER//GDF6//LCP1//RGS16//MAP3K9//COL2A1//DKKL1//IL12RB2//SPIC//KRT4//CBLN2 |
| GO:0019233 | sensory perception of pain | Biological process | 4 | 104 | 55 | 13692 | 9.57482517482518 | 0.000795661983043018 | 0.290681844471716 | 3.09927139215768 | GRM8//CCL3//CCKBR//CHRNA4 |
| GO:0051606 | detection of stimulus | Biological process | 14 | 1397 | 55 | 13692 | 2.49480054662589 | 0.00097340509687927 | 0.328262149593748 | 3.01170638406257 | TULP1//OLR1401//OLR19//OLR98//OLR153//OLR200//OLR305//OLR375//OLR1341//OLR25//OLR278//OLR7//OLR1585//GRM8 |
| GO:0032501 | multicellular organismal process | Biological process | 35 | 5780 | 55 | 13692 | 1.5074551745832 | 0.00108416298077836 | 0.339497893409452 | 2.96490542597828 | COL2A1//SLC17A6//CCL3//BMPER//MET//SPIC//CHRNA7//GRM8//CHRNA4//THEG//SSTR1//FEZF2//RXRG//SRPK3//CCKBR//POMC//CYP11B1//TULP1//LCP1//IL12RB2//GDF6//OLR1401//OLR19//OLR98//OLR153//OLR200//OLR305//OLR375//OLR1341//OLR25//OLR278//OLR7//OLR1585//CBLN2//ABCG2 |
| GO:0050896 | response to stimulus | Biological process | 38 | 6556 | 55 | 13692 | 1.44294192689556 | 0.00117394489561816 | 0.343104961492668 | 2.93035228817411 | CCL3//MET//CHRNA7//CHRNA4//CCKBR//LCP1//POMC//RASSF5//GRB7//SRGAP1//ABCG2//SSTR1//OLR1401//OLR19//OLR98//OLR153//OLR200//OLR305//OLR375//OLR1341//OLR25//OLR278//OLR7//OLR1585//GRM8//GRP//CNTN6//ITGA10//FEZF2//TULP1//CYP11B1//GDF6//RXRG//BMPER//IL12RB2//CLDN4//RGS16//COL2A1 |
| GO:0007608 | sensory perception of smell | Biological process | 13 | 1296 | 55 | 13692 | 2.49713804713805 | 0.001526594843792 | 0.418286987199008 | 2.81627620881965 | OLR1401//OLR19//OLR98//OLR153//OLR200//OLR305//OLR375//OLR1341//OLR25//OLR278//OLR7//OLR1585//GRM8 |
| GO:0042221 | response to chemical stimulus | Biological process | 25 | 3628 | 55 | 13692 | 1.71544552470683 | 0.00186484477725292 | 0.442066771740475 | 2.72935731140919 | MET//CCL3//ABCG2//FEZF2//CYP11B1//RXRG//CHRNA7//IL12RB2//CLDN4//CCKBR//CHRNA4//SSTR1//OLR1401//OLR19//OLR98//OLR153//OLR200//OLR305//OLR375//OLR1341//OLR25//OLR278//OLR7//OLR1585//COL2A1 |
| GO:0050906 | detection of stimulus involved in sensory perception | Biological process | 13 | 1333 | 55 | 13692 | 2.42782513810271 | 0.00197006561746125 | 0.442066771740475 | 2.70551930844412 | OLR1401//OLR19//OLR98//OLR153//OLR200//OLR305//OLR375//OLR1341//OLR25//OLR278//OLR7//OLR1585//GRM8 |
| GO:0007267 | cell-cell signaling | Biological process | 9 | 715 | 55 | 13692 | 3.1335791481246 | 0.00201269853413085 | 0.442066771740475 | 2.69622126970551 | SLC17A6//GRM8//CHRNA7//CHRNA4//CCKBR//CBLN2//MET//POMC//CCL3 |
| GO:0015837 | amine transport | Biological process | 4 | 136 | 55 | 13692 | 7.32192513368984 | 0.00214593699546288 | 0.442066771740475 | 2.66838303303385 | CCKBR//CHRNA4//CHRNA7//SLC17A6 |
| GO:0007271 | synaptic transmission, cholinergic | Biological process | 2 | 18 | 55 | 13692 | 27.6606060606061 | 0.00232608021866643 | 0.442066771740475 | 2.63337531199646 | CHRNA7//CHRNA4 |
| GO:0032640 | tumor necrosis factor production | Biological process | 3 | 66 | 55 | 13692 | 11.3157024793388 | 0.00234681997509593 | 0.442066771740475 | 2.62952022391701 | POMC//CHRNA7//CCL3 |
| GO:0032680 | regulation of tumor necrosis factor production | Biological process | 3 | 66 | 55 | 13692 | 11.3157024793388 | 0.00234681997509593 | 0.442066771740475 | 2.62952022391701 | POMC//CHRNA7//CCL3 |
| GO:0007606 | sensory perception of chemical stimulus | Biological process | 13 | 1364 | 55 | 13692 | 2.37264729405492 | 0.00242007356792231 | 0.442066771740475 | 2.61617143168194 | GRM8//OLR1401//OLR19//OLR98//OLR153//OLR200//OLR305//OLR375//OLR1341//OLR25//OLR278//OLR7//OLR1585 |
| GO:0071706 | tumor necrosis factor superfamily cytokine production | Biological process | 3 | 68 | 55 | 13692 | 10.9828877005348 | 0.00255572236411743 | 0.448171473771633 | 2.59248632667983 | POMC//CHRNA7//CCL3 |
| GO:0050789 | regulation of biological process | Biological process | 41 | 7619 | 55 | 13692 | 1.33964610006085 | 0.00295124761346401 | 0.488282147416071 | 2.52999435110672 | MET//FEZF2//CCL3//CHRNA7//NOV//POMC//CHRNA4//RASSF5//GRB7//SRGAP1//RXRG//SSTR1//OLR1401//OLR19//OLR98//OLR153//OLR200//OLR305//OLR375//OLR1341//OLR25//OLR278//OLR7//OLR1585//GRM8//GRP//CNTN6//ITGA10//TULP1//CCKBR//COL2A1//BMPER//GDF6//IL12RB2//LCP1//RGS16//MAP3K9//DKKL1//SPIC//KRT4//CBLN2 |
| GO:0007218 | neuropeptide signaling pathway | Biological process | 3 | 72 | 55 | 13692 | 10.3727272727273 | 0.00300721213052781 | 0.488282147416071 | 2.5218359354285 | POMC//SSTR1//GRP |
| GO:0050911 | detection of chemical stimulus involved in sensory perception of smell | Biological process | 12 | 1265 | 55 | 13692 | 2.36153790873158 | 0.00380609423726741 | 0.59592561200644 | 2.41952046297003 | OLR1401//OLR19//OLR98//OLR153//OLR200//OLR305//OLR375//OLR1341//OLR25//OLR278//OLR7//OLR1585 |
| GO:0071705 | nitrogen compound transport | Biological process | 4 | 166 | 55 | 13692 | 5.9986856516977 | 0.0043947055420323 | 0.656168036275759 | 2.35707021860037 | CCKBR//CHRNA4//CHRNA7//SLC17A6 |
| GO:0050907 | detection of chemical stimulus involved in sensory perception | Biological process | 12 | 1291 | 55 | 13692 | 2.31397788888106 | 0.00449020097816441 | 0.656168036275759 | 2.34773421985316 | OLR1401//OLR19//OLR98//OLR153//OLR200//OLR305//OLR375//OLR1341//OLR25//OLR278//OLR7//OLR1585 |
| GO:0032720 | negative regulation of tumor necrosis factor production | Biological process | 2 | 26 | 55 | 13692 | 19.1496503496504 | 0.00484044590393062 | 0.684532736865543 | 2.31511462912077 | POMC//CHRNA7 |
| GO:0031644 | regulation of neurological system process | Biological process | 5 | 279 | 55 | 13692 | 4.4613880742913 | 0.00513506000520242 | 0.695995461350571 | 2.28945447713466 | RXRG//CHRNA7//CCKBR//GRM8//CCL3 |
| GO:0009593 | detection of chemical stimulus | Biological process | 12 | 1316 | 55 | 13692 | 2.27001934235977 | 0.00523901693078669 | 0.695995461350571 | 2.28075019805227 | OLR1401//OLR19//OLR98//OLR153//OLR200//OLR305//OLR375//OLR1341//OLR25//OLR278//OLR7//OLR1585 |
| GO:0032651 | regulation of interleukin-1 beta production | Biological process | 2 | 28 | 55 | 13692 | 17.7818181818182 | 0.00560098069364782 | 0.722197040028001 | 2.25173592432406 | CHRNA7//CCL3 |
| GO:0019226 | transmission of nerve impulse | Biological process | 7 | 547 | 55 | 13692 | 3.18577364134951 | 0.00601598752140886 | 0.75001217691199 | 2.22069307324569 | SLC17A6//GRM8//CHRNA7//CHRNA4//RXRG//CCKBR//CBLN2 |
| GO:0007610 | behavior | Biological process | 7 | 552 | 55 | 13692 | 3.15691699604743 | 0.00631581212228101 | 0.75001217691199 | 2.1995707975159 | CCKBR//CHRNA7//CHRNA4//FEZF2//POMC//MET//CCL3 |
| GO:0060393 | regulation of pathway-restricted SMAD protein phosphorylation | Biological process | 2 | 30 | 55 | 13692 | 16.5963636363636 | 0.00641258351457913 | 0.75001217691199 | 2.19296696578895 | GDF6//BMPER |
| GO:0001775 | cell activation | Biological process | 7 | 555 | 55 | 13692 | 3.13985257985258 | 0.00650101795681013 | 0.75001217691199 | 2.1870186343708 | LCP1//SSTR1//CHRNA7//CHRNA4//BMPER//CCL3//IL12RB2 |
| GO:0035637 | multicellular organismal signaling | Biological process | 7 | 558 | 55 | 13692 | 3.12297165200391 | 0.00669027293863143 | 0.752055296486159 | 2.17455616424659 | SLC17A6//GRM8//CHRNA7//CHRNA4//RXRG//CCKBR//CBLN2 |
| GO:0032652 | regulation of interleukin-1 production | Biological process | 2 | 32 | 55 | 13692 | 15.5590909090909 | 0.00727441699698704 | 0.759310574161695 | 2.13820180717192 | CHRNA7//CCL3 |
| GO:0051930 | regulation of sensory perception of pain | Biological process | 2 | 32 | 55 | 13692 | 15.5590909090909 | 0.00727441699698704 | 0.759310574161695 | 2.13820180717192 | CCL3//CCKBR |
| GO:0051931 | regulation of sensory perception | Biological process | 2 | 32 | 55 | 13692 | 15.5590909090909 | 0.00727441699698704 | 0.759310574161695 | 2.13820180717192 | CCL3//CCKBR |
| GO:0030317 | sperm motility | Biological process | 2 | 33 | 55 | 13692 | 15.0876033057851 | 0.00772391121893112 | 0.76958242690441 | 2.11216272678155 | MET//CHRNA7 |
| GO:0060389 | pathway-restricted SMAD protein phosphorylation | Biological process | 2 | 33 | 55 | 13692 | 15.0876033057851 | 0.00772391121893112 | 0.76958242690441 | 2.11216272678155 | GDF6//BMPER |
| GO:0032611 | interleukin-1 beta production | Biological process | 2 | 34 | 55 | 13692 | 14.6438502673797 | 0.00818565349203048 | 0.797464553534703 | 2.08694664347821 | CHRNA7//CCL3 |
| GO:0007268 | synaptic transmission | Biological process | 6 | 446 | 55 | 13692 | 3.34904198940073 | 0.00868994568739989 | 0.828189606381763 | 2.06098293790533 | SLC17A6//CHRNA7//CHRNA4//CCKBR//GRM8//CBLN2 |
| GO:0071346 | cellular response to interferon-gamma | Biological process | 2 | 36 | 55 | 13692 | 13.830303030303 | 0.00914547497427936 | 0.853058772068951 | 2.03879373432623 | CCL3//CYP11B1 |
| GO:0050433 | regulation of catecholamine secretion | Biological process | 2 | 37 | 55 | 13692 | 13.4565110565111 | 0.00964335206027192 | 0.880759488171502 | 2.01577197769129 | CHRNA4//CHRNA7 |
| GO:0071702 | organic substance transport | Biological process | 6 | 462 | 55 | 13692 | 3.23305785123967 | 0.0102361898932755 | 0.899945668550427 | 1.98986166604035 | CCKBR//SLC17A6//MET//CHRNA4//CHRNA7//SLC6A5 |
| GO:0007270 | neuron-neuron synaptic transmission | Biological process | 3 | 112 | 55 | 13692 | 6.66818181818182 | 0.0102673819155838 | 0.899945668550427 | 1.98854028323499 | CHRNA7//CCKBR//GRM8 |
| GO:0002286 | T cell activation involved in immune response | Biological process | 2 | 39 | 55 | 13692 | 12.7664335664336 | 0.0106745380393755 | 0.899945668550427 | 1.97165091080329 | IL12RB2//LCP1 |
| GO:0032612 | interleukin-1 production | Biological process | 2 | 39 | 55 | 13692 | 12.7664335664336 | 0.0106745380393755 | 0.899945668550427 | 1.97165091080329 | CHRNA7//CCL3 |
| GO:0030534 | adult behavior | Biological process | 3 | 116 | 55 | 13692 | 6.43824451410658 | 0.0112903662607023 | 0.933905012960734 | 1.94729196928656 | CHRNA7//CHRNA4//MET |
| GO:0050432 | catecholamine secretion | Biological process | 2 | 41 | 55 | 13692 | 12.1436807095344 | 0.0117523054128425 | 0.954113091294473 | 1.92987693085048 | CHRNA4//CHRNA7 |
| GO:0055082 | cellular chemical homeostasis | Biological process | 7 | 624 | 55 | 13692 | 2.79265734265734 | 0.0119877408423102 | 0.955531924594326 | 1.92126265444605 | CHRNA4//CHRNA7//CCL3//CCKBR//RXRG//CHRNE//MET |
| GO:0045321 | leukocyte activation | Biological process | 6 | 483 | 55 | 13692 | 3.09249011857708 | 0.0125526609013478 | 0.982694024848371 | 1.90126420308489 | LCP1//SSTR1//CHRNA7//CHRNA4//CCL3//IL12RB2 |
| GO:0014070 | response to organic cyclic compound | Biological process | 5 | 358 | 55 | 13692 | 3.47689182326054 | 0.0141914866925815 | 1 | 1.84797210570083 | CHRNA7//CHRNA4//CCL3//MET//RXRG |
| GO:0002263 | cell activation involved in immune response | Biological process | 3 | 129 | 55 | 13692 | 5.78942917547569 | 0.0150112163535081 | 1 | 1.82358411560391 | LCP1//CCL3//IL12RB2 |
| GO:0002366 | leukocyte activation involved in immune response | Biological process | 3 | 129 | 55 | 13692 | 5.78942917547569 | 0.0150112163535081 | 1 | 1.82358411560391 | LCP1//CCL3//IL12RB2 |
| GO:0016358 | dendrite development | Biological process | 3 | 129 | 55 | 13692 | 5.78942917547569 | 0.0150112163535081 | 1 | 1.82358411560391 | MET//FEZF2//TULP1 |
| GO:0070371 | ERK1 and ERK2 cascade | Biological process | 3 | 130 | 55 | 13692 | 5.7448951048951 | 0.0153228227856296 | 1 | 1.81466122117238 | CCL3//BMPER//CCKBR |
| GO:0051094 | positive regulation of developmental process | Biological process | 7 | 657 | 55 | 13692 | 2.65238688252387 | 0.0155603109468498 | 1 | 1.80798172860907 | DKKL1//GDF6//FEZF2//CCL3//CHRNA7//MET//CBLN2 |
| GO:0035094 | response to nicotine | Biological process | 2 | 50 | 55 | 13692 | 9.95781818181818 | 0.0171578675549289 | 1 | 1.76553668890428 | CHRNA7//CHRNA4 |
| GO:0007215 | glutamate receptor signaling pathway | Biological process | 2 | 53 | 55 | 13692 | 9.39416809605489 | 0.0191537919625563 | 1 | 1.71774523394664 | GRM8//SSTR1 |
| GO:0051937 | catecholamine transport | Biological process | 2 | 53 | 55 | 13692 | 9.39416809605489 | 0.0191537919625563 | 1 | 1.71774523394664 | CHRNA4//CHRNA7 |
| GO:0051969 | regulation of transmission of nerve impulse | Biological process | 4 | 256 | 55 | 13692 | 3.88977272727273 | 0.0193282951505349 | 1 | 1.71380645116379 | RXRG//CHRNA7//CCKBR//GRM8 |
| GO:0001508 | regulation of action potential | Biological process | 3 | 145 | 55 | 13692 | 5.15059561128527 | 0.0204378061372174 | 1 | 1.68956572466434 | CHRNA7//RXRG//CHRNA4 |
| GO:0019725 | cellular homeostasis | Biological process | 7 | 696 | 55 | 13692 | 2.50376175548589 | 0.0207009560944439 | 1 | 1.68400959575312 | CHRNA4//CHRNA7//CCL3//CCKBR//RXRG//CHRNE//MET |
| GO:0042391 | regulation of membrane potential | Biological process | 4 | 262 | 55 | 13692 | 3.80069396252602 | 0.0208473793552122 | 1 | 1.68094853076708 | CHRNA4//CHRNA7//RXRG//CHRNE |
| GO:0008217 | regulation of blood pressure | Biological process | 3 | 148 | 55 | 13692 | 5.04619164619165 | 0.0215605220858529 | 1 | 1.66634072696119 | CHRNA7//POMC//CYP11B1 |
| GO:0046649 | lymphocyte activation | Biological process | 5 | 400 | 55 | 13692 | 3.11181818181818 | 0.0218482304825198 | 1 | 1.66058373136162 | LCP1//SSTR1//CHRNA7//CHRNA4//IL12RB2 |
| GO:0034341 | response to interferon-gamma | Biological process | 2 | 57 | 55 | 13692 | 8.73492822966507 | 0.0219590707232371 | 1 | 1.65838604256027 | CCL3//CYP11B1 |
| GO:0051952 | regulation of amine transport | Biological process | 2 | 58 | 55 | 13692 | 8.58432601880878 | 0.0226855125299327 | 1 | 1.64425140434563 | CHRNA4//CHRNA7 |
| GO:0006873 | cellular ion homeostasis | Biological process | 6 | 568 | 55 | 13692 | 2.62970550576184 | 0.025741593986035 | 1 | 1.58936456396211 | CHRNA4//CHRNA7//CCL3//CCKBR//RXRG//CHRNE |
| GO:0032879 | regulation of localization | Biological process | 10 | 1252 | 55 | 13692 | 1.98838222480395 | 0.0258579860941046 | 1 | 1.58740530243614 | BMPER//MET//CHRNA4//CHRNA7//CCL3//GRB7//LCP1//GRM8//TULP1//CCKBR |
| GO:0042110 | T cell activation | Biological process | 4 | 281 | 55 | 13692 | 3.54370753801359 | 0.0261361203763561 | 1 | 1.58275887827754 | LCP1//SSTR1//IL12RB2//CHRNA7 |
| GO:0035249 | synaptic transmission, glutamatergic | Biological process | 2 | 63 | 55 | 13692 | 7.9030303030303 | 0.0264640375535708 | 1 | 1.57734389584516 | CCKBR//GRM8 |
| GO:0015844 | monoamine transport | Biological process | 2 | 65 | 55 | 13692 | 7.65986013986014 | 0.0280421087961133 | 1 | 1.5521893300749 | CHRNA4//CHRNA7 |
| GO:0015850 | organic alcohol transport | Biological process | 2 | 66 | 55 | 13692 | 7.54380165289256 | 0.0288450865717701 | 1 | 1.53992815327073 | CHRNA4//CHRNA7 |
| GO:0051046 | regulation of secretion | Biological process | 5 | 432 | 55 | 13692 | 2.88131313131313 | 0.0292213295756516 | 1 | 1.5343000274766 | CHRNA4//CHRNA7//GRM8//CCL3//CCKBR |
| GO:0060341 | regulation of cellular localization | Biological process | 6 | 587 | 55 | 13692 | 2.54458726962986 | 0.029623603840155 | 1 | 1.5283621087896 | CHRNA4//CHRNA7//LCP1//GRM8//CCL3//CCKBR |
| GO:0051049 | regulation of transport | Biological process | 8 | 926 | 55 | 13692 | 2.15071666993913 | 0.0305599043190001 | 1 | 1.51484800984096 | MET//CHRNA4//CHRNA7//LCP1//GRM8//CCL3//TULP1//CCKBR |
| GO:0009582 | detection of abiotic stimulus | Biological process | 2 | 69 | 55 | 13692 | 7.21581027667984 | 0.0313087781844281 | 1 | 1.50433388026282 | TULP1//GRM8 |
| GO:0006468 | protein phosphorylation | Biological process | 8 | 945 | 55 | 13692 | 2.10747474747475 | 0.0339211522577521 | 1 | 1.46952940374185 | MET//CHRNA7//GDF6//IL12RB2//MAP3K9//BMPER//CCL3//SRPK3 |
| GO:0009605 | response to external stimulus | Biological process | 9 | 1128 | 55 | 13692 | 1.9862669245648 | 0.0345134017199405 | 1 | 1.46201223353983 | CCL3//FEZF2//TULP1//CHRNA7//POMC//RXRG//SSTR1//GRM8//ABCG2 |
| GO:0007519 | skeletal muscle tissue development | Biological process | 3 | 181 | 55 | 13692 | 4.12616775489704 | 0.0361080891674565 | 1 | 1.44239549373842 | MET//RXRG//SRPK3 |
| GO:0032504 | multicellular organism reproduction | Biological process | 6 | 618 | 55 | 13692 | 2.41694616063548 | 0.0367596889221245 | 1 | 1.43462817247571 | SSTR1//THEG//MET//CHRNA7//ABCG2//CCKBR |
| GO:0048609 | multicellular organismal reproductive process | Biological process | 6 | 618 | 55 | 13692 | 2.41694616063548 | 0.0367596889221245 | 1 | 1.43462817247571 | SSTR1//THEG//MET//CHRNA7//ABCG2//CCKBR |
| GO:0010033 | response to organic substance | Biological process | 12 | 1714 | 55 | 13692 | 1.74290866659595 | 0.0368355099303973 | 1 | 1.43373331363522 | MET//RXRG//CCL3//IL12RB2//CLDN4//CCKBR//CYP11B1//CHRNA7//CHRNA4//SSTR1//ABCG2//COL2A1 |
| GO:0002285 | lymphocyte activation involved in immune response | Biological process | 2 | 76 | 55 | 13692 | 6.5511961722488 | 0.0373662194827024 | 1 | 1.42752083955618 | LCP1//IL12RB2 |
| GO:0045666 | positive regulation of neuron differentiation | Biological process | 2 | 76 | 55 | 13692 | 6.5511961722488 | 0.0373662194827024 | 1 | 1.42752083955618 | GDF6//FEZF2 |
| GO:0050801 | ion homeostasis | Biological process | 6 | 626 | 55 | 13692 | 2.38605866976474 | 0.0387675833910736 | 1 | 1.41153127024975 | CHRNA4//CHRNA7//CCL3//CCKBR//RXRG//CHRNE |
| GO:0001817 | regulation of cytokine production | Biological process | 4 | 320 | 55 | 13692 | 3.11181818181818 | 0.0393380516215149 | 1 | 1.40518715410096 | CHRNA7//POMC//IL12RB2//CCL3 |
| GO:0018894 | dibenzo-p-dioxin metabolic process | Biological process | 1 | 10 | 55 | 13692 | 24.8945454545455 | 0.0394637909581559 | 1 | 1.40380119802128 | CYP11B1 |
| GO:0032342 | aldosterone biosynthetic process | Biological process | 1 | 10 | 55 | 13692 | 24.8945454545455 | 0.0394637909581559 | 1 | 1.40380119802128 | CYP11B1 |
| GO:0032692 | negative regulation of interleukin-1 production | Biological process | 1 | 10 | 55 | 13692 | 24.8945454545455 | 0.0394637909581559 | 1 | 1.40380119802128 | CHRNA7 |
| GO:0046415 | urate metabolic process | Biological process | 1 | 10 | 55 | 13692 | 24.8945454545455 | 0.0394637909581559 | 1 | 1.40380119802128 | ABCG2 |
| GO:0071621 | granulocyte chemotaxis | Biological process | 1 | 10 | 55 | 13692 | 24.8945454545455 | 0.0394637909581559 | 1 | 1.40380119802128 | CCL3 |
| GO:0071772 | response to BMP stimulus | Biological process | 1 | 10 | 55 | 13692 | 24.8945454545455 | 0.0394637909581559 | 1 | 1.40380119802128 | COL2A1 |
| GO:0071773 | cellular response to BMP stimulus | Biological process | 1 | 10 | 55 | 13692 | 24.8945454545455 | 0.0394637909581559 | 1 | 1.40380119802128 | COL2A1 |
| GO:0060538 | skeletal muscle organ development | Biological process | 3 | 188 | 55 | 13692 | 3.97253384912959 | 0.0397074044294442 | 1 | 1.40112850071782 | MET//RXRG//SRPK3 |
| GO:0071495 | cellular response to endogenous stimulus | Biological process | 5 | 470 | 55 | 13692 | 2.64835589941973 | 0.0398332308059889 | 1 | 1.39975446727109 | RXRG//CYP11B1//SSTR1//MET//COL2A1 |
| GO:0009725 | response to hormone stimulus | Biological process | 7 | 801 | 55 | 13692 | 2.17555328566564 | 0.0402490108617074 | 1 | 1.39524478815526 | CLDN4//CCKBR//RXRG//CYP11B1//SSTR1//MET//ABCG2 |
| GO:0045597 | positive regulation of cell differentiation | Biological process | 5 | 474 | 55 | 13692 | 2.62600690448792 | 0.0410714143842254 | 1 | 1.38646034100591 | DKKL1//GDF6//FEZF2//CCL3//MET |
| GO:0048878 | chemical homeostasis | Biological process | 7 | 807 | 55 | 13692 | 2.1593781683001 | 0.0416418669603822 | 1 | 1.38046980768177 | CHRNA4//CHRNA7//CCL3//CCKBR//RXRG//CHRNE//MET |
| GO:0032870 | cellular response to hormone stimulus | Biological process | 4 | 326 | 55 | 13692 | 3.05454545454545 | 0.041654408488752 | 1 | 1.38033902832634 | SSTR1//MET//RXRG//CYP11B1 |
| GO:0009719 | response to endogenous stimulus | Biological process | 8 | 987 | 55 | 13692 | 2.01779497098646 | 0.0422398310601512 | 1 | 1.37427782744846 | CLDN4//CCKBR//RXRG//CYP11B1//SSTR1//MET//ABCG2//COL2A1 |
| GO:0009581 | detection of external stimulus | Biological process | 2 | 82 | 55 | 13692 | 6.07184035476718 | 0.0428856954206239 | 1 | 1.36768754314979 | TULP1//GRM8 |
| GO:0051960 | regulation of nervous system development | Biological process | 5 | 480 | 55 | 13692 | 2.59318181818182 | 0.0429726264382397 | 1 | 1.36680810191823 | MET//RXRG//GDF6//FEZF2//CBLN2 |
| GO:0001821 | histamine secretion | Biological process | 1 | 11 | 55 | 13692 | 22.6314049586777 | 0.0433250313833352 | 1 | 1.36326111402422 | CCKBR |
| GO:0006705 | mineralocorticoid biosynthetic process | Biological process | 1 | 11 | 55 | 13692 | 22.6314049586777 | 0.0433250313833352 | 1 | 1.36326111402422 | CYP11B1 |
| GO:0032230 | positive regulation of synaptic transmission, GABAergic | Biological process | 1 | 11 | 55 | 13692 | 22.6314049586777 | 0.0433250313833352 | 1 | 1.36326111402422 | CCKBR |
| GO:0032341 | aldosterone metabolic process | Biological process | 1 | 11 | 55 | 13692 | 22.6314049586777 | 0.0433250313833352 | 1 | 1.36326111402422 | CYP11B1 |
| GO:0046085 | adenosine metabolic process | Biological process | 1 | 11 | 55 | 13692 | 22.6314049586777 | 0.0433250313833352 | 1 | 1.36326111402422 | CYP11B1 |
| GO:0060080 | regulation of inhibitory postsynaptic membrane potential | Biological process | 1 | 11 | 55 | 13692 | 22.6314049586777 | 0.0433250313833352 | 1 | 1.36326111402422 | CHRNA4 |
| GO:0072498 | embryonic skeletal joint development | Biological process | 1 | 11 | 55 | 13692 | 22.6314049586777 | 0.0433250313833352 | 1 | 1.36326111402422 | COL2A1 |
| GO:0000187 | activation of MAPK activity | Biological process | 2 | 83 | 55 | 13692 | 5.9986856516977 | 0.0438337077326407 | 1 | 1.35819179241422 | MET//CHRNA7 |
| GO:0070374 | positive regulation of ERK1 and ERK2 cascade | Biological process | 2 | 84 | 55 | 13692 | 5.92727272727273 | 0.0447895441293231 | 1 | 1.3488233578082 | CCL3//BMPER |
| GO:0031667 | response to nutrient levels | Biological process | 5 | 490 | 55 | 13692 | 2.54025974025974 | 0.0462590369828295 | 1 | 1.33480341258132 | CHRNA7//POMC//RXRG//SSTR1//ABCG2 |
| GO:0006855 | drug transmembrane transport | Biological process | 1 | 12 | 55 | 13692 | 20.7454545454545 | 0.0471710311840745 | 1 | 1.32632462973992 | ABCG2 |
| GO:0008212 | mineralocorticoid metabolic process | Biological process | 1 | 12 | 55 | 13692 | 20.7454545454545 | 0.0471710311840745 | 1 | 1.32632462973992 | CYP11B1 |
| GO:0014808 | release of sequestered calcium ion into cytosol by sarcoplasmic reticulum | Biological process | 1 | 12 | 55 | 13692 | 20.7454545454545 | 0.0471710311840745 | 1 | 1.32632462973992 | CCL3 |
| GO:0032225 | regulation of synaptic transmission, dopaminergic | Biological process | 1 | 12 | 55 | 13692 | 20.7454545454545 | 0.0471710311840745 | 1 | 1.32632462973992 | CHRNA7 |
| GO:0043922 | negative regulation by host of viral transcription | Biological process | 1 | 12 | 55 | 13692 | 20.7454545454545 | 0.0471710311840745 | 1 | 1.32632462973992 | CCL3 |
| GO:0050716 | positive regulation of interleukin-1 secretion | Biological process | 1 | 12 | 55 | 13692 | 20.7454545454545 | 0.0471710311840745 | 1 | 1.32632462973992 | CCL3 |
| GO:0050718 | positive regulation of interleukin-1 beta secretion | Biological process | 1 | 12 | 55 | 13692 | 20.7454545454545 | 0.0471710311840745 | 1 | 1.32632462973992 | CCL3 |
| GO:0060206 | estrous cycle phase | Biological process | 1 | 12 | 55 | 13692 | 20.7454545454545 | 0.0471710311840745 | 1 | 1.32632462973992 | CCKBR |
| GO:0070296 | sarcoplasmic reticulum calcium ion transport | Biological process | 1 | 12 | 55 | 13692 | 20.7454545454545 | 0.0471710311840745 | 1 | 1.32632462973992 | CCL3 |
| GO:0044057 | regulation of system process | Biological process | 5 | 495 | 55 | 13692 | 2.51460055096419 | 0.0479576609358322 | 1 | 1.31914200712935 | CHRNA7//RXRG//CCKBR//GRM8//CCL3 |
| GO:0022414 | reproductive process | Biological process | 8 | 1018 | 55 | 13692 | 1.95634934809787 | 0.0491978981166726 | 1 | 1.30805345121292 | SSTR1//THEG//CLDN4//MET//CHRNA7//CCL3//ABCG2//CCKBR |
