# Supplementary material for: Gene Expression in the Hippocampus in a Rat Model of Premenstrual Dysphoric Disorder After Treatment With Baixiangdan Capsules
Source: Front Psychol. 2018 Nov 13;9:2065. doi: 10.3389/fpsyg.2018.02065 (PMC6242977; doi:10.3389/fpsyg.2018.02065)
Supplement: Supplementary file 3 [file Data_Sheet_3.ZIP › Data Analysis Folder/GO Analysis Report/BXD vs model (up)/CC_result(Rat).html]

| GO.ID | Term | Ontology | Count | Pop.Hits | List.Total | Pop.Total | Fold.Enrichment | Pvalue | FDR | Enrichment.Score | GENES |
| --- | --- | --- | --- | --- | --- | --- | --- | --- | --- | --- | --- |
| GO:0031224 | intrinsic to membrane | Cellular component | 41 | 4750 | 68 | 15288 | 1.94058204334365 | 5.95453646353599e-07 | 0.000274218765012145 | 6.22515204092577 | MET//SSTR1//CHRNA4//CCKBR//CHRNE//FXYD6//FUT1//TPBG//SLC17A6//SLC16A10//SLC6A5//OLR1401//CLDN23//CBLN2//OLR19//OLR98//OLR153//OLR200//OLR305//OLR375//TAAR7B//TSPAN1//OLR1341//CLDN4//GPR123//ABCG2//OLR25//OLR278//OLR7//OLR1585//VOM1R40//VOM1R37//VOM1R57//LOC688657//VOM2R71//CNTN6//GRM8//ITGA10//CHRNA7//CYP11B1//PIGZL1 |
| GO:0044425 | membrane part | Cellular component | 45 | 5668 | 68 | 15288 | 1.78494333513222 | 1.02129893859272e-06 | 0.000274218765012145 | 5.9908471194421 | MET//SSTR1//CHRNA4//CCKBR//CHRNE//FXYD6//FUT1//TPBG//SLC17A6//SLC16A10//SLC6A5//OLR1401//CLDN23//CBLN2//OLR19//OLR98//OLR153//OLR200//OLR305//OLR375//TAAR7B//TSPAN1//OLR1341//CLDN4//GPR123//ABCG2//OLR25//OLR278//OLR7//OLR1585//VOM1R40//VOM1R37//VOM1R57//LOC688657//VOM2R71//LCP1//CNTN6//GRM8//ITGA10//CHRNA7//GRB7//IL12RB2//CYP11B1//PIGZL1//RGS16 |
| GO:0016021 | integral to membrane | Cellular component | 39 | 4653 | 68 | 15288 | 1.88440095574013 | 3.61389659263457e-06 | 0.000646887490081588 | 5.44202427841889 | GRM8//ITGA10//CHRNA7//CHRNA4//CYP11B1//MET//SSTR1//CCKBR//CHRNE//FXYD6//FUT1//TPBG//SLC17A6//SLC16A10//SLC6A5//OLR1401//CLDN23//CBLN2//OLR19//OLR98//OLR153//OLR200//OLR305//OLR375//TAAR7B//TSPAN1//OLR1341//CLDN4//GPR123//ABCG2//OLR25//OLR278//OLR7//OLR1585//VOM1R40//VOM1R37//VOM1R57//LOC688657//VOM2R71 |
| GO:0016020 | membrane | Cellular component | 48 | 7097 | 68 | 15288 | 1.52057621695994 | 4.60356079022324e-05 | 0.0061802803608747 | 4.33690611759685 | MET//SSTR1//CHRNA7//CHRNA4//CCKBR//CHRNE//FXYD6//TAAR7B//CLDN4//LCP1//GPR123//TULP1//GPRC5A//RGS16//VOM2R71//FUT1//TPBG//SLC17A6//SLC16A10//SLC6A5//OLR1401//CLDN23//CBLN2//OLR19//OLR98//OLR153//OLR200//OLR305//OLR375//TSPAN1//OLR1341//ABCG2//OLR25//OLR278//OLR7//OLR1585//VOM1R40//VOM1R37//VOM1R57//LOC688657//CNTN6//GRM8//ITGA10//GRB7//IL12RB2//CYP11B1//PIGZL1//PRPH |
| GO:0045211 | postsynaptic membrane | Cellular component | 5 | 172 | 68 | 15288 | 6.53556771545828 | 0.00100035494506227 | 0.107438121099688 | 2.99984587666912 | MET//CHRNA7//CHRNA4//CHRNE//GRM8 |
| GO:0005892 | acetylcholine-gated channel complex | Cellular component | 2 | 15 | 68 | 15288 | 29.9764705882353 | 0.00197176884178108 | 0.157235595734693 | 2.70514400046139 | CHRNA7//CHRNA4 |
| GO:0097060 | synaptic membrane | Cellular component | 5 | 203 | 68 | 15288 | 5.53752535496957 | 0.00207976739213382 | 0.157235595734693 | 2.68198523521115 | CHRNA7//GRM8//MET//CHRNA4//CHRNE |
| GO:0043025 | neuronal cell body | Cellular component | 7 | 413 | 68 | 15288 | 3.81056829511466 | 0.00234242973161554 | 0.157235595734693 | 2.63033342824353 | MET//PRPH//CHRNA7//CHRNA4//GRM8//NOV//KLHL14 |
| GO:0044297 | cell body | Cellular component | 7 | 441 | 68 | 15288 | 3.56862745098039 | 0.00337364432874156 | 0.201294111614913 | 2.47190070544785 | MET//PRPH//CHRNA7//CHRNA4//GRM8//NOV//KLHL14 |
| GO:0005886 | plasma membrane | Cellular component | 23 | 2986 | 68 | 15288 | 1.73172845829558 | 0.00384089386145203 | 0.206256000359974 | 2.4155676938821 | LCP1//CNTN6//GRM8//ITGA10//CHRNA7//CHRNA4//CLDN23//CLDN4//GRB7//IL12RB2//SLC16A10//ABCG2//MET//SSTR1//CCKBR//CHRNE//FXYD6//TAAR7B//GPR123//TULP1//GPRC5A//RGS16//VOM2R71 |
| GO:0071944 | cell periphery | Cellular component | 23 | 3081 | 68 | 15288 | 1.6783320923306 | 0.00574481366551141 | 0.280451358034512 | 2.24072405319894 | MET//SSTR1//CHRNA7//CHRNA4//CCKBR//CHRNE//FXYD6//TAAR7B//CLDN4//LCP1//GPR123//TULP1//GPRC5A//RGS16//VOM2R71//CNTN6//GRM8//ITGA10//CLDN23//GRB7//IL12RB2//SLC16A10//ABCG2 |
| GO:0044456 | synapse part | Cellular component | 6 | 391 | 68 | 15288 | 3.44997743342861 | 0.00776474312115086 | 0.347472254671501 | 2.109872907342 | SLC17A6//MET//CHRNA7//GRM8//CHRNA4//CHRNE |
| GO:0045202 | synapse | Cellular component | 7 | 527 | 68 | 15288 | 2.98627078915057 | 0.00877077317221713 | 0.362300399498508 | 2.05696212047355 | SLC17A6//MET//CHRNA7//GRM8//CHRNA4//CHRNE//TULP1 |
| GO:0005615 | extracellular space | Cellular component | 8 | 707 | 68 | 15288 | 2.54397204426325 | 0.0128550406505295 | 0.488195930577023 | 1.89092654568213 | MET//POMC//COL2A1//CCL3//GRP//GDF6//CBLN2//BMPER |
| GO:0001750 | photoreceptor outer segment | Cellular component | 2 | 40 | 68 | 15288 | 11.2411764705882 | 0.013636757837347 | 0.488195930577023 | 1.8652888716678 | PRPH//TULP1 |
| GO:0030054 | cell junction | Cellular component | 7 | 627 | 68 | 15288 | 2.50999155643118 | 0.0210456364370244 | 0.705255191542743 | 1.67683793630187 | CLDN23//CLDN4//GRB7//CHRNA7//CHRNA4//CHRNE//SLC17A6 |
| GO:0044459 | plasma membrane part | Cellular component | 12 | 1426 | 68 | 15288 | 1.8919231086544 | 0.022326514443625 | 0.705255191542743 | 1.65117907254235 | LCP1//CNTN6//GRM8//ITGA10//CHRNA7//CHRNA4//CLDN23//CLDN4//GRB7//IL12RB2//SLC16A10//ABCG2 |
| GO:0042734 | presynaptic membrane | Cellular component | 2 | 54 | 68 | 15288 | 8.32679738562091 | 0.0240424123186813 | 0.717265300840659 | 1.6190219591264 | CHRNA7//GRM8 |
| GO:0043235 | receptor complex | Cellular component | 3 | 145 | 68 | 15288 | 4.65152129817444 | 0.0267574403401222 | 0.756249761191875 | 1.57255543422745 | ITGA10//CHRNA7//CHRNA4 |
| GO:0042995 | cell projection | Cellular component | 10 | 1211 | 68 | 15288 | 1.85651139068344 | 0.0407032808492906 | 0.94522709287952 | 1.3903705834697 | LCP1//TULP1//PRPH//MET//CHRNA7//GRM8//KLHL14//NOV//CHRNA4//GRB7 |
| GO:0044421 | extracellular region part | Cellular component | 8 | 887 | 68 | 15288 | 2.02772067113204 | 0.0424221893825049 | 0.94522709287952 | 1.37240692157608 | COL2A1//MET//POMC//CCL3//GRP//GDF6//CBLN2//BMPER |
| GO:0043005 | neuron projection | Cellular component | 7 | 742 | 68 | 15288 | 2.12097669256382 | 0.0461915510576819 | 0.94522709287952 | 1.33543745441328 | PRPH//MET//CHRNA7//NOV//CHRNA4//GRM8//KLHL14 |
| GO:0031513 | nonmotile primary cilium | Cellular component | 2 | 78 | 68 | 15288 | 5.76470588235294 | 0.0471477713004824 | 0.94522709287952 | 1.32653883176608 | TULP1//PRPH |
