# Supplementary material for: Gene Expression in the Hippocampus in a Rat Model of Premenstrual Dysphoric Disorder After Treatment With Baixiangdan Capsules
Source: Front Psychol. 2018 Nov 13;9:2065. doi: 10.3389/fpsyg.2018.02065 (PMC6242977; doi:10.3389/fpsyg.2018.02065)
Supplement: Supplementary file 3 [file Data_Sheet_3.ZIP › Data Analysis Folder/GO Analysis Report/BXD vs model (up)/MF_result(Rat).html]

| GO.ID | Term | Ontology | Count | Pop.Hits | List.Total | Pop.Total | Fold.Enrichment | Pvalue | FDR | Enrichment.Score | GENES |
| --- | --- | --- | --- | --- | --- | --- | --- | --- | --- | --- | --- |
| GO:0038023 | signaling receptor activity | Molecular function | 28 | 2232 | 65 | 14392 | 2.7776123518059 | 9.97992310037915e-08 | 5.62267514371501e-05 | 7.00087280512642 | TAAR7B//GRM8//RXRG//IL12RB2//OLR1401//OLR19//OLR98//OLR153//OLR305//OLR375//OLR1341//GPR123//OLR278//OLR1585//LOC688657//VOM2R71//OLR200//OLR25//OLR7//SSTR1//MET//TULP1//CCKBR//CHRNA7//CHRNA4//VOM1R40//VOM1R37//VOM1R57 |
| GO:0004872 | receptor activity | Molecular function | 30 | 2579 | 65 | 14392 | 2.575595788469 | 1.45641120716165e-07 | 5.62267514371501e-05 | 6.83671598780611 | TAAR7B//GRM8//RXRG//IL12RB2//OLR1401//OLR19//OLR98//OLR153//OLR305//OLR375//OLR1341//GPR123//OLR278//OLR1585//LOC688657//VOM2R71//OLR200//OLR25//OLR7//SSTR1//MET//TULP1//CCKBR//CHRNA7//CHRNA4//VOM1R40//VOM1R37//VOM1R57//CHRNE//ITGA10 |
| GO:0004888 | transmembrane signaling receptor activity | Molecular function | 27 | 2149 | 65 | 14392 | 2.78185918316212 | 1.88049335910201e-07 | 5.62267514371501e-05 | 6.72572819593498 | TAAR7B//GRM8//IL12RB2//OLR1401//OLR19//OLR98//OLR153//OLR305//OLR375//OLR1341//GPR123//OLR278//OLR1585//LOC688657//VOM2R71//OLR200//OLR25//OLR7//SSTR1//MET//TULP1//CCKBR//CHRNA7//CHRNA4//VOM1R40//VOM1R37//VOM1R57 |
| GO:0004871 | signal transducer activity | Molecular function | 28 | 2433 | 65 | 14392 | 2.54814252742736 | 6.30234524712238e-07 | 0.000113064073733376 | 6.20049780953822 | TAAR7B//GRM8//RXRG//IL12RB2//OLR1401//OLR19//OLR98//OLR153//OLR305//OLR375//OLR1341//GPR123//OLR278//OLR1585//LOC688657//VOM2R71//OLR200//OLR25//OLR7//SSTR1//MET//TULP1//CCKBR//CHRNA7//CHRNA4//VOM1R40//VOM1R37//VOM1R57 |
| GO:0060089 | molecular transducer activity | Molecular function | 28 | 2433 | 65 | 14392 | 2.54814252742736 | 6.30234524712238e-07 | 0.000113064073733376 | 6.20049780953822 | TAAR7B//GRM8//RXRG//IL12RB2//OLR1401//OLR19//OLR98//OLR153//OLR305//OLR375//OLR1341//GPR123//OLR278//OLR1585//LOC688657//VOM2R71//OLR200//OLR25//OLR7//SSTR1//MET//TULP1//CCKBR//CHRNA7//CHRNA4//VOM1R40//VOM1R37//VOM1R57 |
| GO:0004930 | G-protein coupled receptor activity | Molecular function | 20 | 1569 | 65 | 14392 | 2.82237583958425 | 1.12281852137151e-05 | 0.00167861368945041 | 4.94969043210537 | TAAR7B//GRM8//SSTR1//TULP1//CCKBR//VOM1R40//VOM1R37//VOM1R57//OLR1401//OLR19//OLR98//OLR153//OLR305//OLR375//OLR1341//GPR123//OLR278//OLR1585//LOC688657//VOM2R71 |
| GO:0004889 | acetylcholine-activated cation-selective channel activity | Molecular function | 3 | 16 | 65 | 14392 | 41.5153846153846 | 4.72162954252746e-05 | 0.0060504309994959 | 4.32590809052022 | CHRNA7//CHRNA4//CHRNE |
| GO:0030594 | neurotransmitter receptor activity | Molecular function | 4 | 65 | 65 | 14392 | 13.6255621301775 | 0.000208624311231458 | 0.0233920008968272 | 3.68063508412394 | SSTR1//CCKBR//CHRNA7//CHRNA4 |
| GO:0005231 | excitatory extracellular ligand-gated ion channel activity | Molecular function | 3 | 47 | 65 | 14392 | 14.1328968903437 | 0.00123720758161402 | 0.123308355634197 | 2.90755742730203 | CHRNA7//CHRNA4//CHRNE |
| GO:0042166 | acetylcholine binding | Molecular function | 2 | 14 | 65 | 14392 | 31.6307692307692 | 0.00176488294329956 | 0.158310000013971 | 2.75328409410898 | CHRNA7//CHRNA4 |
| GO:0015464 | acetylcholine receptor activity | Molecular function | 2 | 15 | 65 | 14392 | 29.5220512820513 | 0.00203048226138006 | 0.165576598950719 | 2.69240080022389 | CHRNA7//CHRNA4 |
| GO:0005230 | extracellular ligand-gated ion channel activity | Molecular function | 3 | 66 | 65 | 14392 | 10.0643356643357 | 0.0032850188953069 | 0.245555162424191 | 2.48346212805126 | CHRNA7//CHRNA4//CHRNE |
| GO:0042165 | neurotransmitter binding | Molecular function | 2 | 24 | 65 | 14392 | 18.4512820512821 | 0.00519942923853606 | 0.347324650375235 | 2.28404432793425 | CHRNA7//CHRNA4 |
| GO:0004984 | olfactory receptor activity | Molecular function | 13 | 1318 | 65 | 14392 | 2.18391502276176 | 0.00542089755323667 | 0.347324650375235 | 2.26592880015058 | OLR1401//OLR19//OLR98//OLR153//OLR200//OLR305//OLR375//OLR1341//OLR25//OLR278//OLR7//OLR1585//LOC688657 |
| GO:0071855 | neuropeptide receptor binding | Molecular function | 2 | 27 | 65 | 14392 | 16.4011396011396 | 0.0065550083259147 | 0.391989497889699 | 2.18342675234971 | CCKBR//POMC |
| GO:0016503 | pheromone receptor activity | Molecular function | 3 | 96 | 65 | 14392 | 6.91923076923077 | 0.0093197278851865 | 0.522487244563268 | 2.03059676786997 | VOM1R40//VOM1R37//VOM1R57 |
| GO:0008188 | neuropeptide receptor activity | Molecular function | 2 | 40 | 65 | 14392 | 11.0707692307692 | 0.0140288255503246 | 0.740226854037716 | 1.85297868523414 | SSTR1//CCKBR |
| GO:0015276 | ligand-gated ion channel activity | Molecular function | 3 | 118 | 65 | 14392 | 5.62920469361147 | 0.016234154809641 | 0.766422992855157 | 1.78957031689717 | CHRNA7//CHRNA4//CHRNE |
| GO:0022834 | ligand-gated channel activity | Molecular function | 3 | 118 | 65 | 14392 | 5.62920469361147 | 0.016234154809641 | 0.766422992855157 | 1.78957031689717 | CHRNA7//CHRNA4//CHRNE |
| GO:0015171 | amino acid transmembrane transporter activity | Molecular function | 2 | 66 | 65 | 14392 | 6.70955710955711 | 0.0358001905170262 | 1 | 1.44611466217531 | SLC17A6//SLC6A5 |
| GO:0051015 | actin filament binding | Molecular function | 2 | 66 | 65 | 14392 | 6.70955710955711 | 0.0358001905170262 | 1 | 1.44611466217531 | LCP1//TULP1 |
| GO:0005096 | GTPase activator activity | Molecular function | 3 | 161 | 65 | 14392 | 4.1257525083612 | 0.0362444167922506 | 1 | 1.44075888407412 | TBC1D22A//SRGAP1//RGS16 |
| GO:0008047 | enzyme activator activity | Molecular function | 4 | 284 | 65 | 14392 | 3.11852654387866 | 0.0393419406193082 | 1 | 1.40514422145099 | RGS16//TBC1D22A//SRGAP1//CCL3 |
| GO:0008417 | fucosyltransferase activity | Molecular function | 1 | 10 | 65 | 14392 | 22.1415384615385 | 0.0442706085016012 | 1 | 1.353884508582 | FUT1 |
| GO:0009881 | photoreceptor activity | Molecular function | 1 | 10 | 65 | 14392 | 22.1415384615385 | 0.0442706085016012 | 1 | 1.353884508582 | TULP1 |
| GO:0015172 | acidic amino acid transmembrane transporter activity | Molecular function | 1 | 10 | 65 | 14392 | 22.1415384615385 | 0.0442706085016012 | 1 | 1.353884508582 | SLC17A6 |
| GO:0005099 | Ras GTPase activator activity | Molecular function | 2 | 78 | 65 | 14392 | 5.67731755424063 | 0.0484310378892288 | 1 | 1.3148762238146 | TBC1D22A//SRGAP1 |
| GO:0000030 | mannosyltransferase activity | Molecular function | 1 | 11 | 65 | 14392 | 20.1286713286713 | 0.0485900641021716 | 1 | 1.31345252799604 | PIGZL1 |
| GO:0048407 | platelet-derived growth factor binding | Molecular function | 1 | 11 | 65 | 14392 | 20.1286713286713 | 0.0485900641021716 | 1 | 1.31345252799604 | COL2A1 |
| GO:0060229 | lipase activator activity | Molecular function | 1 | 11 | 65 | 14392 | 20.1286713286713 | 0.0485900641021716 | 1 | 1.31345252799604 | CCL3 |
| GO:0005275 | amine transmembrane transporter activity | Molecular function | 2 | 79 | 65 | 14392 | 5.60545277507303 | 0.0495475413313471 | 1 | 1.30497789138499 | SLC17A6//SLC6A5 |
