# Supplementary material for: Gene Expression in the Hippocampus in a Rat Model of Premenstrual Dysphoric Disorder After Treatment With Baixiangdan Capsules
Source: Front Psychol. 2018 Nov 13;9:2065. doi: 10.3389/fpsyg.2018.02065 (PMC6242977; doi:10.3389/fpsyg.2018.02065)
Supplement: Supplementary file 3 [file Data_Sheet_3.ZIP › Data Analysis Folder/GO Analysis Report/fluoxetine vs blank (down)/BP_result(Rat).html]

| GO.ID | Term | Ontology | Count | Pop.Hits | List.Total | Pop.Total | Fold.Enrichment | Pvalue | FDR | Enrichment.Score | GENES |
| --- | --- | --- | --- | --- | --- | --- | --- | --- | --- | --- | --- |
| GO:0007272 | ensheathment of neurons | Biological process | 8 | 92 | 63 | 13692 | 18.8985507246377 | 8.7275636325748e-09 | 1.9130819482604e-05 | 8.05910697606573 | PLP1//CLDN11//CD9//UGT8//FA2H//NDRG1//MAL//LPAR1 |
| GO:0008366 | axon ensheathment | Biological process | 8 | 92 | 63 | 13692 | 18.8985507246377 | 8.7275636325748e-09 | 1.9130819482604e-05 | 8.05910697606573 | PLP1//CD9//UGT8//FA2H//NDRG1//MAL//LPAR1//CLDN11 |
| GO:0001508 | regulation of action potential | Biological process | 9 | 145 | 63 | 13692 | 13.4896551724138 | 1.90035780990208e-08 | 2.77705621287024e-05 | 7.72116461997569 | PLP1//CLDN11//CD9//UGT8//FA2H//NDRG1//MAL//LPAR1//SCN4B |
| GO:0019228 | regulation of action potential in neuron | Biological process | 8 | 111 | 63 | 13692 | 15.6636636636637 | 3.86726235749588e-08 | 4.23851954381548e-05 | 7.41259636411836 | PLP1//CLDN11//CD9//UGT8//FA2H//NDRG1//MAL//LPAR1 |
| GO:0007399 | nervous system development | Biological process | 23 | 1503 | 63 | 13692 | 3.32579285872699 | 8.75065036133399e-08 | 7.67257023681764e-05 | 7.05795966836713 | ROBO3//SATB2//BMP4//CNP//RTN4//CD9//NT5E//CHN1//PLP1//CLDN11//GFAP//P2RY2//TNR//LPAR1//UGT8//FA2H//NDRG1//MAL//S1PR5//SEMA7A//EGR1//MOBP//BHLHE40 |
| GO:0042552 | myelination | Biological process | 7 | 89 | 63 | 13692 | 17.0936329588015 | 1.58937969949452e-07 | 0.0001098117390072 | 6.79877233835185 | PLP1//CD9//UGT8//FA2H//NDRG1//MAL//LPAR1 |
| GO:0035637 | multicellular organismal signaling | Biological process | 14 | 558 | 63 | 13692 | 5.45280764635603 | 1.7533808691843e-07 | 0.0001098117390072 | 6.75612373626293 | PLP1//CLDN11//CD9//UGT8//FA2H//NDRG1//PRIMA1//MAL//LPAR1//BHLHE40//EGR1//TNR//GFAP//SCN4B |
| GO:0010001 | glial cell differentiation | Biological process | 8 | 148 | 63 | 13692 | 11.7477477477477 | 3.6123328582782e-07 | 0.000185298704764013 | 6.44221223846565 | GFAP//CD9//PLP1//FA2H//NDRG1//RTN4//EGR1//BMP4 |
| GO:0022008 | neurogenesis | Biological process | 18 | 1009 | 63 | 13692 | 3.87710604558969 | 3.80403362882326e-07 | 0.000185298704764013 | 6.41975565242132 | ROBO3//SATB2//CNP//RTN4//CHN1//PLP1//GFAP//P2RY2//TNR//LPAR1//CD9//FA2H//NDRG1//S1PR5//BMP4//SEMA7A//EGR1//UGT8 |
| GO:0048709 | oligodendrocyte differentiation | Biological process | 6 | 67 | 63 | 13692 | 19.4626865671642 | 5.96447368475568e-07 | 0.000261482526339689 | 6.22442787318241 | CD9//PLP1//FA2H//BMP4//EGR1//RTN4 |
| GO:0007275 | multicellular organismal development | Biological process | 34 | 3429 | 63 | 13692 | 2.15495285311558 | 8.22894406887486e-07 | 0.000327960825435885 | 6.08465588951219 | BMP4//RTN4//PLEKHA1//RPE65//ROBO3//SATB2//WFS1//WNT10A//FA2H//P2RY2//FOXN3//MOBP//TNR//BHLHE40//CNP//CD9//NT5E//MKX//CHN1//PLP1//CLDN11//ITPR1//PNLIP//GFAP//LPAR1//EGR1//CLCN2//UGT8//NDRG1//MGST1//MAL//S1PR5//SEMA7A//ZAR1 |
| GO:0019226 | transmission of nerve impulse | Biological process | 13 | 547 | 63 | 13692 | 5.16514320536258 | 9.56652231039495e-07 | 0.000349496948406429 | 6.0192459113242 | PLP1//CLDN11//CD9//UGT8//FA2H//NDRG1//PRIMA1//MAL//LPAR1//BHLHE40//EGR1//TNR//GFAP |
| GO:0006873 | cellular ion homeostasis | Biological process | 13 | 568 | 63 | 13692 | 4.97417840375587 | 1.45859322721513e-06 | 0.000491882516008549 | 5.83606580735737 | RGN//ITPR1//P2RY2//PLP1//CLDN11//CD9//UGT8//FA2H//NDRG1//WFS1//MAL//LPAR1//SCN4B |
| GO:0032502 | developmental process | Biological process | 36 | 3887 | 63 | 13692 | 2.01286339078981 | 1.60864357596006e-06 | 0.000503735245500636 | 5.79354017102906 | BMP4//RTN4//PLEKHA1//RPE65//CHN2//ERMN//ROBO3//SATB2//WFS1//WNT10A//FA2H//MKX//ZAR1//P2RY2//FOXN3//MOBP//TNR//BHLHE40//CNP//CD9//NT5E//CHN1//PLP1//CLDN11//ITPR1//PNLIP//GFAP//LPAR1//EGR1//CLCN2//UGT8//NDRG1//MGST1//MAL//S1PR5//SEMA7A |
| GO:0042063 | gliogenesis | Biological process | 8 | 182 | 63 | 13692 | 9.55311355311355 | 1.73420922168555e-06 | 0.00050685154852463 | 5.76089850873857 | PLP1//GFAP//CD9//FA2H//NDRG1//RTN4//EGR1//BMP4 |
| GO:0065008 | regulation of biological quality | Biological process | 25 | 2106 | 63 | 13692 | 2.57993035770814 | 2.56840379736366e-06 | 0.000703742640477643 | 5.59033669665429 | WFS1//RGN//ITPR1//P2RY2//PLEKHA1//ERMN//PLP1//CLDN11//BMP4//TNR//RTN4//CD9//UGT8//FA2H//NDRG1//PRIMA1//MAL//LPAR1//SEMA7A//CNP//BHLHE40//EGR1//NTS//GFAP//SCN4B |
| GO:0042391 | regulation of membrane potential | Biological process | 9 | 262 | 63 | 13692 | 7.46564885496183 | 2.8781969127767e-06 | 0.000742236192094885 | 5.54087949698493 | PLP1//CLDN11//CD9//UGT8//FA2H//NDRG1//MAL//LPAR1//SCN4B |
| GO:0055082 | cellular chemical homeostasis | Biological process | 13 | 624 | 63 | 13692 | 4.52777777777778 | 4.12273523689173e-06 | 0.000985152415165925 | 5.38481455480549 | RGN//ITPR1//P2RY2//PLP1//CLDN11//CD9//UGT8//FA2H//NDRG1//WFS1//MAL//LPAR1//SCN4B |
| GO:0050801 | ion homeostasis | Biological process | 13 | 626 | 63 | 13692 | 4.51331203407881 | 4.26959304018079e-06 | 0.000985152415165925 | 5.36961351814281 | RGN//ITPR1//P2RY2//PLP1//CLDN11//CD9//UGT8//FA2H//NDRG1//WFS1//MAL//LPAR1//SCN4B |
| GO:0048468 | cell development | Biological process | 19 | 1333 | 63 | 13692 | 3.0977744436109 | 4.96006543160158e-06 | 0.00108724634260707 | 5.30451259439769 | CHN2//SATB2//BMP4//CNP//ROBO3//RTN4//CHN1//GFAP//P2RY2//TNR//LPAR1//CD9//PLP1//FA2H//NDRG1//MKX//S1PR5//SEMA7A//UGT8 |
| GO:0048856 | anatomical structure development | Biological process | 33 | 3546 | 63 | 13692 | 2.02256063169769 | 6.31535294525609e-06 | 0.00131840511009537 | 5.19960237307479 | BMP4//RTN4//PLEKHA1//RPE65//CHN2//ERMN//ROBO3//SATB2//WFS1//WNT10A//FA2H//MKX//P2RY2//FOXN3//MOBP//TNR//BHLHE40//CNP//CD9//NT5E//CHN1//PLP1//CLDN11//GFAP//LPAR1//EGR1//CLCN2//UGT8//NDRG1//MGST1//MAL//S1PR5//SEMA7A |
| GO:0048731 | system development | Biological process | 30 | 3069 | 63 | 13692 | 2.12447051156729 | 8.82801471576926e-06 | 0.00175918256881511 | 5.05413695156701 | BMP4//RTN4//PLEKHA1//RPE65//ROBO3//SATB2//WFS1//WNT10A//FA2H//MOBP//TNR//BHLHE40//CNP//CD9//NT5E//MKX//CHN1//PLP1//CLDN11//GFAP//P2RY2//LPAR1//EGR1//CLCN2//UGT8//NDRG1//MGST1//MAL//S1PR5//SEMA7A |
| GO:0019725 | cellular homeostasis | Biological process | 13 | 696 | 63 | 13692 | 4.05938697318008 | 1.34193730686687e-05 | 0.00255784919708885 | 4.87226777322647 | RGN//ITPR1//P2RY2//PLP1//CLDN11//CD9//UGT8//FA2H//NDRG1//WFS1//MAL//LPAR1//SCN4B |
| GO:0021782 | glial cell development | Biological process | 5 | 68 | 63 | 13692 | 15.9803921568627 | 1.46359160715893e-05 | 0.00267349400241031 | 4.8345800896029 | GFAP//CD9//PLP1//FA2H//NDRG1 |
| GO:0010977 | negative regulation of neuron projection development | Biological process | 4 | 36 | 63 | 13692 | 24.1481481481481 | 2.14657709390918e-05 | 0.00376423759187914 | 4.6682535093005 | TNR//RTN4//GFAP//LPAR1 |
| GO:0045664 | regulation of neuron differentiation | Biological process | 9 | 358 | 63 | 13692 | 5.46368715083799 | 3.51669794870778e-05 | 0.00577731802613683 | 4.45386493189427 | P2RY2//GFAP//TNR//RTN4//LPAR1//BMP4//SEMA7A//CHN1//S1PR5 |
| GO:0031344 | regulation of cell projection organization | Biological process | 8 | 275 | 63 | 13692 | 6.32242424242424 | 3.55811101062259e-05 | 0.00577731802613683 | 4.44878050635082 | P2RY2//GFAP//TNR//RTN4//LPAR1//SEMA7A//CHN1//ERMN |
| GO:0048869 | cellular developmental process | Biological process | 25 | 2442 | 63 | 13692 | 2.22495222495222 | 3.70321585900948e-05 | 0.00579817797353484 | 4.4314209723566 | BMP4//CHN2//ROBO3//SATB2//FA2H//CNP//RTN4//CHN1//ERMN//PLP1//GFAP//P2RY2//TNR//LPAR1//CD9//WNT10A//EGR1//UGT8//NDRG1//MGST1//PLEKHA1//MKX//S1PR5//SEMA7A//CLCN2 |
| GO:0030154 | cell differentiation | Biological process | 24 | 2291 | 63 | 13692 | 2.27673505019642 | 3.95409128617657e-05 | 0.00597749524089589 | 4.40295330856177 | BMP4//CHN2//ROBO3//SATB2//FA2H//CNP//RTN4//CHN1//PLP1//GFAP//P2RY2//TNR//LPAR1//CD9//WNT10A//EGR1//NDRG1//MGST1//PLEKHA1//MKX//S1PR5//SEMA7A//UGT8//CLCN2 |
| GO:0048878 | chemical homeostasis | Biological process | 13 | 807 | 63 | 13692 | 3.50103263114416 | 6.31248373229831e-05 | 0.00922464289413193 | 4.19979972807838 | WFS1//RGN//ITPR1//P2RY2//PLP1//CLDN11//CD9//UGT8//FA2H//NDRG1//MAL//LPAR1//SCN4B |
| GO:0010975 | regulation of neuron projection development | Biological process | 7 | 228 | 63 | 13692 | 6.67251461988304 | 8.11064095648819e-05 | 0.0114700161139498 | 4.09094482361047 | P2RY2//GFAP//TNR//RTN4//LPAR1//SEMA7A//CHN1 |
| GO:0060284 | regulation of cell development | Biological process | 10 | 512 | 63 | 13692 | 4.24479166666667 | 0.000105163351664647 | 0.0144073791780566 | 3.97813558093135 | GFAP//P2RY2//TNR//RTN4//LPAR1//BMP4//MKX//S1PR5//SEMA7A//CHN1 |
| GO:0050767 | regulation of neurogenesis | Biological process | 9 | 428 | 63 | 13692 | 4.57009345794393 | 0.000138743237824786 | 0.0184318289279958 | 3.85778817466977 | GFAP//P2RY2//TNR//RTN4//LPAR1//S1PR5//BMP4//SEMA7A//CHN1 |
| GO:0030030 | cell projection organization | Biological process | 12 | 756 | 63 | 13692 | 3.44973544973545 | 0.000145359276632811 | 0.0187427961399483 | 3.83755724692154 | CNP//ROBO3//RTN4//CHN1//P2RY2//GFAP//TNR//LPAR1//ERMN//PLEKHA1//SEMA7A//UGT8 |
| GO:0031175 | neuron projection development | Biological process | 10 | 543 | 63 | 13692 | 4.0024554941682 | 0.000170247271418311 | 0.0213246867970822 | 3.7689198398702 | CNP//ROBO3//RTN4//CHN1//P2RY2//GFAP//TNR//LPAR1//SEMA7A//UGT8 |
| GO:0048640 | negative regulation of developmental growth | Biological process | 3 | 25 | 63 | 13692 | 26.08 | 0.000198645182695393 | 0.0239092355984302 | 3.70192196246956 | TNR//RTN4//BMP4 |
| GO:0032989 | cellular component morphogenesis | Biological process | 12 | 783 | 63 | 13692 | 3.33077905491699 | 0.000201788712851715 | 0.0239092355984302 | 3.69510312988955 | CHN2//BMP4//CNP//ROBO3//RTN4//CHN1//ERMN//TNR//CD9//UGT8//GFAP//SEMA7A |
| GO:0048699 | generation of neurons | Biological process | 13 | 929 | 63 | 13692 | 3.04126300681737 | 0.0002589051297252 | 0.0298694760188231 | 3.5868593447398 | ROBO3//SATB2//CNP//RTN4//CHN1//GFAP//P2RY2//TNR//LPAR1//S1PR5//BMP4//SEMA7A//UGT8 |
| GO:0060291 | long-term synaptic potentiation | Biological process | 3 | 28 | 63 | 13692 | 23.2857142857143 | 0.000280169113295724 | 0.0314938818638065 | 3.55257974437002 | EGR1//GFAP//TNR |
| GO:0051960 | regulation of nervous system development | Biological process | 9 | 480 | 63 | 13692 | 4.075 | 0.000325967394364777 | 0.0357260264223796 | 3.48682583905982 | GFAP//P2RY2//TNR//RTN4//LPAR1//S1PR5//BMP4//SEMA7A//CHN1 |
| GO:0031345 | negative regulation of cell projection organization | Biological process | 4 | 74 | 63 | 13692 | 11.7477477477477 | 0.000367937382853778 | 0.0384056544388324 | 3.43422608511715 | GFAP//TNR//RTN4//LPAR1 |
| GO:0071453 | cellular response to oxygen levels | Biological process | 4 | 74 | 63 | 13692 | 11.7477477477477 | 0.000367937382853778 | 0.0384056544388324 | 3.43422608511715 | EGR1//ITPR1//NDRG1//LPAR1 |
| GO:0048812 | neuron projection morphogenesis | Biological process | 8 | 390 | 63 | 13692 | 4.45811965811966 | 0.000396921513997602 | 0.0404675329619881 | 3.40129536076222 | CNP//ROBO3//RTN4//CHN1//TNR//GFAP//SEMA7A//UGT8 |
| GO:0030182 | neuron differentiation | Biological process | 12 | 846 | 63 | 13692 | 3.08274231678487 | 0.000410570641292197 | 0.0407869249934747 | 3.38661210898553 | CNP//ROBO3//RTN4//CHN1//P2RY2//GFAP//TNR//LPAR1//S1PR5//BMP4//SEMA7A//UGT8 |
| GO:0014003 | oligodendrocyte development | Biological process | 3 | 32 | 63 | 13692 | 20.375 | 0.000418661410745064 | 0.0407869249934747 | 3.37813706746517 | PLP1//FA2H//CD9 |
| GO:0042592 | homeostatic process | Biological process | 14 | 1125 | 63 | 13692 | 2.70459259259259 | 0.000482259378074485 | 0.0459614155104031 | 3.31671931825868 | WFS1//RGN//ITPR1//P2RY2//PLP1//CLDN11//BMP4//CD9//UGT8//FA2H//NDRG1//MAL//LPAR1//SCN4B |
| GO:0032501 | multicellular organismal process | Biological process | 40 | 5780 | 63 | 13692 | 1.5040369088812 | 0.000523460808993733 | 0.048826642268692 | 3.2811158279773 | BMP4//RTN4//PLEKHA1//RPE65//CHN2//ERMN//ROBO3//SATB2//WFS1//WNT10A//FA2H//ZAR1//CLDN11//EIF4G3//P2RY2//FOXN3//MOBP//TNR//BHLHE40//CNP//CD9//NT5E//MKX//EGR1//CHN1//PLP1//ITPR1//PNLIP//GFAP//LPAR1//CLCN2//UGT8//NDRG1//MGST1//PRIMA1//MAL//S1PR5//SEMA7A//NTS//SCN4B |
| GO:0048666 | neuron development | Biological process | 10 | 644 | 63 | 13692 | 3.37474120082816 | 0.000660795514774118 | 0.0603526570160361 | 3.17993291350163 | CNP//ROBO3//RTN4//CHN1//P2RY2//GFAP//TNR//LPAR1//SEMA7A//UGT8 |
| GO:0023052 | signaling | Biological process | 34 | 4621 | 63 | 13692 | 1.59907667892952 | 0.000742633733718977 | 0.0664429854821224 | 3.12922532707962 | RGD1306565//LPAR1//BMP4//TNR//CHN1//CHN2//PLCL1//SPTBN1//GCGR//S1PR5//PLP1//SEMA7A//GJC2//CLDN11//PLEKHA1//ITPR1//NDRG1//EGR1//CD9//UGT8//WFS1//FA2H//RTN4//MOG//P2RY2//PRIMA1//MAL//OTUD7B//BHLHE40//RGN//GFAP//WNT10A//SCN4B |
| GO:0070887 | cellular response to chemical stimulus | Biological process | 14 | 1182 | 63 | 13692 | 2.57416807670615 | 0.000790347171523307 | 0.0692976399991636 | 3.10218209661208 | GCGR//WFS1//EGR1//BMP4//PLEKHA1//RGD1306565//RPL5//SATB2//P2RY2//MGST1//LPAR1//ITPR1//NDRG1//WNT10A |
| GO:0030516 | regulation of axon extension | Biological process | 3 | 40 | 63 | 13692 | 16.3 | 0.000812380581203682 | 0.0698328719215087 | 3.09024046633183 | TNR//RTN4//SEMA7A |
| GO:0045662 | negative regulation of myoblast differentiation | Biological process | 2 | 10 | 63 | 13692 | 43.4666666666667 | 0.000915626090803142 | 0.0771943227323264 | 3.03828184058204 | BMP4//MKX |
| GO:0031102 | neuron projection regeneration | Biological process | 3 | 42 | 63 | 13692 | 15.5238095238095 | 0.000937782729995026 | 0.0775705563829848 | 3.02789776939726 | RTN4//TNR//GFAP |
| GO:0050770 | regulation of axonogenesis | Biological process | 4 | 97 | 63 | 13692 | 8.96219931271478 | 0.00102392772941216 | 0.0831277623285724 | 2.98973069553318 | TNR//RTN4//SEMA7A//CHN1 |
| GO:0042487 | regulation of odontogenesis of dentin-containing tooth | Biological process | 2 | 11 | 63 | 13692 | 39.5151515151515 | 0.00111578270915376 | 0.0889380253987288 | 2.95242037296445 | BMP4//WNT10A |
| GO:0009791 | post-embryonic development | Biological process | 4 | 101 | 63 | 13692 | 8.60726072607261 | 0.00119016773814383 | 0.0931731315004027 | 2.92439182632463 | ITPR1//BMP4//PNLIP//PLEKHA1 |
| GO:0061387 | regulation of extent of cell growth | Biological process | 3 | 46 | 63 | 13692 | 14.1739130434783 | 0.00122391298672058 | 0.0941339391891758 | 2.91224945697018 | TNR//RTN4//SEMA7A |
| GO:0007154 | cell communication | Biological process | 34 | 4745 | 63 | 13692 | 1.55728837372673 | 0.00126222039059551 | 0.0954064515925986 | 2.89886480827503 | RGD1306565//LPAR1//BMP4//TNR//CHN1//CHN2//PLCL1//SPTBN1//GCGR//S1PR5//PLP1//SEMA7A//GJC2//CLDN11//PLEKHA1//ITPR1//NDRG1//EGR1//CD9//UGT8//WFS1//FA2H//RTN4//MOG//P2RY2//PRIMA1//MAL//OTUD7B//BHLHE40//RGN//GFAP//WNT10A//SCN4B |
| GO:0032288 | myelin assembly | Biological process | 2 | 12 | 63 | 13692 | 36.2222222222222 | 0.00133497329873998 | 0.0975420490279345 | 2.87452742068581 | CD9//UGT8 |
| GO:0090192 | regulation of glomerulus development | Biological process | 2 | 12 | 63 | 13692 | 36.2222222222222 | 0.00133497329873998 | 0.0975420490279345 | 2.87452742068581 | BMP4//EGR1 |
| GO:0048638 | regulation of developmental growth | Biological process | 4 | 105 | 63 | 13692 | 8.27936507936508 | 0.00137448044567663 | 0.0987823323581368 | 2.86186143435463 | TNR//RTN4//SEMA7A//BMP4 |
| GO:0007417 | central nervous system development | Biological process | 10 | 712 | 63 | 13692 | 3.05243445692884 | 0.00142116021177594 | 0.100489780135899 | 2.84735695995561 | CD9//BMP4//NT5E//GFAP//RTN4//PLP1//TNR//CNP//FA2H//EGR1 |
| GO:0048858 | cell projection morphogenesis | Biological process | 8 | 480 | 63 | 13692 | 3.62222222222222 | 0.00153243623496323 | 0.101413786936524 | 2.81461758753043 | CNP//ROBO3//RTN4//CHN1//TNR//GFAP//SEMA7A//UGT8 |
| GO:0022010 | central nervous system myelination | Biological process | 2 | 13 | 63 | 13692 | 33.4358974358974 | 0.00157302406744609 | 0.101413786936524 | 2.8032646325712 | FA2H//PLP1 |
| GO:0032291 | axon ensheathment in central nervous system | Biological process | 2 | 13 | 63 | 13692 | 33.4358974358974 | 0.00157302406744609 | 0.101413786936524 | 2.8032646325712 | PLP1//FA2H |
| GO:0032469 | endoplasmic reticulum calcium ion homeostasis | Biological process | 2 | 13 | 63 | 13692 | 33.4358974358974 | 0.00157302406744609 | 0.101413786936524 | 2.8032646325712 | ITPR1//WFS1 |
| GO:0048679 | regulation of axon regeneration | Biological process | 2 | 13 | 63 | 13692 | 33.4358974358974 | 0.00157302406744609 | 0.101413786936524 | 2.8032646325712 | TNR//RTN4 |
| GO:0070570 | regulation of neuron projection regeneration | Biological process | 2 | 13 | 63 | 13692 | 33.4358974358974 | 0.00157302406744609 | 0.101413786936524 | 2.8032646325712 | RTN4//TNR |
| GO:0000902 | cell morphogenesis | Biological process | 10 | 725 | 63 | 13692 | 2.99770114942529 | 0.00162698276981869 | 0.103372354534567 | 2.78861704633314 | BMP4//CNP//ROBO3//RTN4//CHN1//ERMN//TNR//GFAP//SEMA7A//UGT8 |
| GO:0032990 | cell part morphogenesis | Biological process | 8 | 493 | 63 | 13692 | 3.52670723461798 | 0.00181377980345669 | 0.10988600348087 | 2.74141543830586 | CNP//ROBO3//RTN4//CHN1//TNR//GFAP//SEMA7A//UGT8 |
| GO:0022011 | myelination in peripheral nervous system | Biological process | 2 | 14 | 63 | 13692 | 31.047619047619 | 0.00182976237547981 | 0.10988600348087 | 2.73760530683713 | NDRG1//FA2H |
| GO:0032292 | peripheral nervous system axon ensheathment | Biological process | 2 | 14 | 63 | 13692 | 31.047619047619 | 0.00182976237547981 | 0.10988600348087 | 2.73760530683713 | NDRG1//FA2H |
| GO:0072111 | cell proliferation involved in kidney development | Biological process | 2 | 14 | 63 | 13692 | 31.047619047619 | 0.00182976237547981 | 0.10988600348087 | 2.73760530683713 | EGR1//BMP4 |
| GO:0048167 | regulation of synaptic plasticity | Biological process | 4 | 118 | 63 | 13692 | 7.36723163841808 | 0.00211052690721529 | 0.125034458935565 | 2.67560910663176 | BHLHE40//EGR1//GFAP//TNR |
| GO:0007184 | SMAD protein import into nucleus | Biological process | 2 | 16 | 63 | 13692 | 27.1666666666667 | 0.00239861677209579 | 0.13656540167361 | 2.62003913388252 | BMP4//SPTBN1 |
| GO:0033327 | Leydig cell differentiation | Biological process | 2 | 16 | 63 | 13692 | 27.1666666666667 | 0.00239861677209579 | 0.13656540167361 | 2.62003913388252 | MGST1//PLEKHA1 |
| GO:0072215 | regulation of metanephros development | Biological process | 2 | 16 | 63 | 13692 | 27.1666666666667 | 0.00239861677209579 | 0.13656540167361 | 2.62003913388252 | EGR1//BMP4 |
| GO:0060560 | developmental growth involved in morphogenesis | Biological process | 4 | 125 | 63 | 13692 | 6.95466666666667 | 0.00260241138714595 | 0.143161013888191 | 2.58462404941732 | TNR//RTN4//SEMA7A//BMP4 |
| GO:0014044 | Schwann cell development | Biological process | 2 | 17 | 63 | 13692 | 25.5686274509804 | 0.00271039328301092 | 0.143161013888191 | 2.56696768763145 | NDRG1//FA2H |
| GO:0030517 | negative regulation of axon extension | Biological process | 2 | 17 | 63 | 13692 | 25.5686274509804 | 0.00271039328301092 | 0.143161013888191 | 2.56696768763145 | TNR//RTN4 |
| GO:0061437 | renal system vasculature development | Biological process | 2 | 17 | 63 | 13692 | 25.5686274509804 | 0.00271039328301092 | 0.143161013888191 | 2.56696768763145 | BMP4//EGR1 |
| GO:0061440 | kidney vasculature development | Biological process | 2 | 17 | 63 | 13692 | 25.5686274509804 | 0.00271039328301092 | 0.143161013888191 | 2.56696768763145 | BMP4//EGR1 |
| GO:0072012 | glomerulus vasculature development | Biological process | 2 | 17 | 63 | 13692 | 25.5686274509804 | 0.00271039328301092 | 0.143161013888191 | 2.56696768763145 | BMP4//EGR1 |
| GO:0010714 | positive regulation of collagen metabolic process | Biological process | 2 | 18 | 63 | 13692 | 24.1481481481481 | 0.0030401781647896 | 0.156801659699266 | 2.51710096451042 | BMP4//MKX |
| GO:0032967 | positive regulation of collagen biosynthetic process | Biological process | 2 | 18 | 63 | 13692 | 24.1481481481481 | 0.0030401781647896 | 0.156801659699266 | 2.51710096451042 | BMP4//MKX |
| GO:0048675 | axon extension | Biological process | 3 | 64 | 63 | 13692 | 10.1875 | 0.00316753470237536 | 0.161470606223414 | 2.49927861844371 | TNR//RTN4//SEMA7A |
| GO:2000026 | regulation of multicellular organismal development | Biological process | 12 | 1078 | 63 | 13692 | 2.41929499072356 | 0.00333173738851195 | 0.167888927715361 | 2.4773292375218 | BMP4//GFAP//P2RY2//TNR//RTN4//LPAR1//WNT10A//MKX//S1PR5//SEMA7A//CHN1//EGR1 |
| GO:0014013 | regulation of gliogenesis | Biological process | 3 | 66 | 63 | 13692 | 9.87878787878788 | 0.00345633663244396 | 0.171861562991699 | 2.46138396570324 | GFAP//RTN4//BMP4 |
| GO:0007409 | axonogenesis | Biological process | 6 | 321 | 63 | 13692 | 4.06230529595016 | 0.00350445516376116 | 0.171861562991699 | 2.45537949203817 | ROBO3//RTN4//CHN1//TNR//SEMA7A//CNP |
| GO:0036294 | cellular response to decreased oxygen levels | Biological process | 3 | 67 | 63 | 13692 | 9.73134328358209 | 0.0036065838948988 | 0.171861562991699 | 2.44290396097348 | EGR1//ITPR1//NDRG1 |
| GO:0050806 | positive regulation of synaptic transmission | Biological process | 3 | 67 | 63 | 13692 | 9.73134328358209 | 0.0036065838948988 | 0.171861562991699 | 2.44290396097348 | TNR//EGR1//GFAP |
| GO:0071456 | cellular response to hypoxia | Biological process | 3 | 67 | 63 | 13692 | 9.73134328358209 | 0.0036065838948988 | 0.171861562991699 | 2.44290396097348 | EGR1//ITPR1//NDRG1 |
| GO:0043586 | tongue development | Biological process | 2 | 20 | 63 | 13692 | 21.7333333333333 | 0.0037531062448123 | 0.176920621260829 | 2.42560914118708 | BMP4//WNT10A |
| GO:0030198 | extracellular matrix organization | Biological process | 4 | 140 | 63 | 13692 | 6.20952380952381 | 0.00391099551573222 | 0.182402173840107 | 2.40771268200079 | MKX//GFAP//TNR//OLFML2B |
| GO:0043062 | extracellular structure organization | Biological process | 4 | 141 | 63 | 13692 | 6.16548463356974 | 0.00401148160067653 | 0.185119319340694 | 2.39669519542035 | GFAP//TNR//OLFML2B//MKX |
| GO:0051971 | positive regulation of transmission of nerve impulse | Biological process | 3 | 71 | 63 | 13692 | 9.1830985915493 | 0.0042472971237165 | 0.193959901983053 | 2.37188735646237 | TNR//EGR1//GFAP |
| GO:0022412 | cellular process involved in reproduction in multicellular organism | Biological process | 2 | 22 | 63 | 13692 | 19.7575757575758 | 0.00453607851061369 | 0.200620547477638 | 2.34331943726523 | CHN2//CD9 |
| GO:0032965 | regulation of collagen biosynthetic process | Biological process | 2 | 22 | 63 | 13692 | 19.7575757575758 | 0.00453607851061369 | 0.200620547477638 | 2.34331943726523 | BMP4//MKX |
| GO:0045661 | regulation of myoblast differentiation | Biological process | 2 | 22 | 63 | 13692 | 19.7575757575758 | 0.00453607851061369 | 0.200620547477638 | 2.34331943726523 | BMP4//MKX |
| GO:0003008 | system process | Biological process | 21 | 2589 | 63 | 13692 | 1.7628427964465 | 0.00457619861947167 | 0.200620547477638 | 2.33949513424375 | BMP4//WFS1//RPE65//EGR1//TNR//PLP1//CLDN11//P2RY2//PNLIP//CD9//UGT8//FA2H//NDRG1//PRIMA1//MAL//LPAR1//BHLHE40//NTS//GFAP//RTN4//SCN4B |
| GO:0008361 | regulation of cell size | Biological process | 3 | 75 | 63 | 13692 | 8.69333333333333 | 0.00495302212583995 | 0.203571904775617 | 2.30512973195925 | TNR//RTN4//SEMA7A |
| GO:0031646 | positive regulation of neurological system process | Biological process | 3 | 75 | 63 | 13692 | 8.69333333333333 | 0.00495302212583995 | 0.203571904775617 | 2.30512973195925 | TNR//EGR1//GFAP |
| GO:0010712 | regulation of collagen metabolic process | Biological process | 2 | 23 | 63 | 13692 | 18.8985507246377 | 0.00495342274995013 | 0.203571904775617 | 2.30509460556575 | BMP4//MKX |
| GO:0014911 | positive regulation of smooth muscle cell migration | Biological process | 2 | 23 | 63 | 13692 | 18.8985507246377 | 0.00495342274995013 | 0.203571904775617 | 2.30509460556575 | LPAR1//P2RY2 |
| GO:0042481 | regulation of odontogenesis | Biological process | 2 | 23 | 63 | 13692 | 18.8985507246377 | 0.00495342274995013 | 0.203571904775617 | 2.30509460556575 | BMP4//WNT10A |
| GO:0016053 | organic acid biosynthetic process | Biological process | 5 | 241 | 63 | 13692 | 4.50899031811895 | 0.00496856610652167 | 0.203571904775617 | 2.30376892753924 | SC5DL//FA2H//SLC27A2//RGN//PLP1 |
| GO:0046394 | carboxylic acid biosynthetic process | Biological process | 5 | 241 | 63 | 13692 | 4.50899031811895 | 0.00496856610652167 | 0.203571904775617 | 2.30376892753924 | SC5DL//FA2H//SLC27A2//RGN//PLP1 |
| GO:0014037 | Schwann cell differentiation | Biological process | 2 | 24 | 63 | 13692 | 18.1111111111111 | 0.00538779006219773 | 0.217795068403219 | 2.26858933511041 | NDRG1//FA2H |
| GO:0006874 | cellular calcium ion homeostasis | Biological process | 5 | 246 | 63 | 13692 | 4.41734417344173 | 0.0054150689908647 | 0.217795068403219 | 2.26639600586027 | ITPR1//P2RY2//WFS1//LPAR1//RGN |
| GO:0055074 | calcium ion homeostasis | Biological process | 5 | 254 | 63 | 13692 | 4.27821522309711 | 0.00618762289782712 | 0.246604898037037 | 2.20847616206757 | RGN//ITPR1//P2RY2//WFS1//LPAR1 |
| GO:0044253 | positive regulation of multicellular organismal metabolic process | Biological process | 2 | 26 | 63 | 13692 | 16.7179487179487 | 0.00630695339766212 | 0.24854973085203 | 2.2001803780146 | BMP4//MKX |
| GO:0048588 | developmental cell growth | Biological process | 3 | 82 | 63 | 13692 | 7.95121951219512 | 0.00634981064220514 | 0.24854973085203 | 2.19723922561694 | TNR//RTN4//SEMA7A |
| GO:0010720 | positive regulation of cell development | Biological process | 4 | 162 | 63 | 13692 | 5.36625514403292 | 0.00654419174335993 | 0.253891474361858 | 2.18414398441876 | GFAP//RTN4//SEMA7A//BMP4 |
| GO:0050793 | regulation of developmental process | Biological process | 13 | 1333 | 63 | 13692 | 2.11952988247062 | 0.00685188752365336 | 0.261365912047123 | 2.16418977475755 | BMP4//ERMN//GFAP//P2RY2//TNR//RTN4//LPAR1//WNT10A//MKX//S1PR5//SEMA7A//CHN1//EGR1 |
| GO:0022604 | regulation of cell morphogenesis | Biological process | 5 | 261 | 63 | 13692 | 4.16347381864623 | 0.00692481338825416 | 0.261365912047123 | 2.15959192559109 | ERMN//TNR//RTN4//SEMA7A//CHN1 |
| GO:0009166 | nucleotide catabolic process | Biological process | 6 | 370 | 63 | 13692 | 3.52432432432432 | 0.0069505400725103 | 0.261365912047123 | 2.15798144844611 | NT5E//CNP//ACAP2//RGN//CHN2//CHN1 |
| GO:0009653 | anatomical structure morphogenesis | Biological process | 16 | 1819 | 63 | 13692 | 1.91167308044713 | 0.00700570944092377 | 0.261365912047123 | 2.15454787883966 | BMP4//RTN4//CHN2//ERMN//CNP//ROBO3//CHN1//TNR//CD9//UGT8//WNT10A//GFAP//MKX//SEMA7A//SATB2//PLEKHA1 |
| GO:0072503 | cellular divalent inorganic cation homeostasis | Biological process | 5 | 262 | 63 | 13692 | 4.14758269720102 | 0.0070349401509034 | 0.261365912047123 | 2.15273959293328 | RGN//ITPR1//P2RY2//WFS1//LPAR1 |
| GO:0007009 | plasma membrane organization | Biological process | 3 | 86 | 63 | 13692 | 7.58139534883721 | 0.00724314297877221 | 0.264616156824478 | 2.14007294179377 | FA2H//NDRG1//SPTBN1 |
| GO:0050768 | negative regulation of neurogenesis | Biological process | 3 | 86 | 63 | 13692 | 7.58139534883721 | 0.00724314297877221 | 0.264616156824478 | 2.14007294179377 | TNR//RTN4//BMP4 |
| GO:0000904 | cell morphogenesis involved in differentiation | Biological process | 7 | 495 | 63 | 13692 | 3.07340067340067 | 0.00744720715857872 | 0.267244129415604 | 2.12800656524132 | BMP4//CNP//ROBO3//RTN4//CHN1//TNR//SEMA7A |
| GO:0044057 | regulation of system process | Biological process | 7 | 495 | 63 | 13692 | 3.07340067340067 | 0.00744720715857872 | 0.267244129415604 | 2.12800656524132 | P2RY2//BHLHE40//EGR1//TNR//GFAP//RTN4//SCN4B |
| GO:0006629 | lipid metabolic process | Biological process | 10 | 898 | 63 | 13692 | 2.42019302152932 | 0.00749795344847612 | 0.267244129415604 | 2.12505726025119 | SLC27A2//SC5DL//FA2H//UGT8//PLEKHA1//PNLIP//NT5E//PLP1//CES1D//PLCL1 |
| GO:0048667 | cell morphogenesis involved in neuron differentiation | Biological process | 6 | 378 | 63 | 13692 | 3.44973544973545 | 0.00768816484852475 | 0.271813828193004 | 2.11417731315987 | CNP//ROBO3//RTN4//CHN1//TNR//SEMA7A |
| GO:0032964 | collagen biosynthetic process | Biological process | 2 | 29 | 63 | 13692 | 14.9885057471264 | 0.00780939635640687 | 0.273891149011902 | 2.1073825345239 | BMP4//MKX |
| GO:1901292 | nucleoside phosphate catabolic process | Biological process | 6 | 380 | 63 | 13692 | 3.43157894736842 | 0.0078811125318892 | 0.274212677300018 | 2.10341247129777 | NT5E//CNP//ACAP2//RGN//CHN2//CHN1 |
| GO:0051240 | positive regulation of multicellular organismal process | Biological process | 7 | 502 | 63 | 13692 | 3.03054448871182 | 0.0080180456726023 | 0.274662985279001 | 2.09593147423882 | BMP4//P2RY2//MKX//TNR//EGR1//GFAP//SEMA7A |
| GO:0048610 | cellular process involved in reproduction | Biological process | 6 | 382 | 63 | 13692 | 3.41361256544503 | 0.0080775467054769 | 0.274662985279001 | 2.09272052215312 | CHN2//EIF4G3//BMP4//CD9//MGST1//PLEKHA1 |
| GO:0072507 | divalent inorganic cation homeostasis | Biological process | 5 | 271 | 63 | 13692 | 4.00984009840098 | 0.00808200846281733 | 0.274662985279001 | 2.09248069913552 | RGN//ITPR1//P2RY2//WFS1//LPAR1 |
| GO:0010769 | regulation of cell morphogenesis involved in differentiation | Biological process | 4 | 173 | 63 | 13692 | 5.02504816955684 | 0.00821813612967163 | 0.277140836865234 | 2.08522666912482 | TNR//RTN4//SEMA7A//CHN1 |
| GO:0071842 | cellular component organization at cellular level | Biological process | 20 | 2544 | 63 | 13692 | 1.70859538784067 | 0.00833549962891795 | 0.278952903612033 | 2.07906836349298 | CNP//CHN2//UGT8//SATB2//ERMN//ROBO3//RTN4//CHN1//P2RY2//GFAP//TNR//LPAR1//OLFML2B//MKX//CD9//PLEKHA1//LSM14A//SEMA7A//BMP4//SPTBN1 |
| GO:0034655 | nucleobase-containing compound catabolic process | Biological process | 6 | 387 | 63 | 13692 | 3.36950904392765 | 0.00858411386689839 | 0.28509663024608 | 2.06630453015701 | NT5E//CNP//ACAP2//RGN//CHN2//CHN1 |
| GO:0033554 | cellular response to stress | Biological process | 10 | 919 | 63 | 13692 | 2.36488937250635 | 0.00876773982501096 | 0.286625555584096 | 2.05711234597633 | RGD1306565//NDRG1//WFS1//GFAP//RTN4//TNR//PLEKHA1//MGST1//EGR1//ITPR1 |
| GO:0090066 | regulation of anatomical structure size | Biological process | 5 | 277 | 63 | 13692 | 3.92298435619735 | 0.00883762212895047 | 0.286625555584096 | 2.0536645715472 | TNR//RTN4//P2RY2//SEMA7A//NTS |
| GO:0044246 | regulation of multicellular organismal metabolic process | Biological process | 2 | 31 | 63 | 13692 | 14.0215053763441 | 0.00889166869512706 | 0.286625555584096 | 2.05101672754735 | BMP4//MKX |
| GO:0051148 | negative regulation of muscle cell differentiation | Biological process | 2 | 31 | 63 | 13692 | 14.0215053763441 | 0.00889166869512706 | 0.286625555584096 | 2.05101672754735 | BMP4//MKX |
| GO:0048518 | positive regulation of biological process | Biological process | 23 | 3107 | 63 | 13692 | 1.60884025319172 | 0.00895793353246726 | 0.286653873038952 | 2.04779216432824 | BMP4//RGD1306565//LPAR1//EIF4G3//MAL//PRUNE2//GFAP//EGR1//PLP1//MKX//WNT10A//P2RY2//NT5E//WFS1//RTN4//MOG//SEMA7A//SATB2//PLEKHA1//ITPR1//TNR//SCN4B//NDRG1 |
| GO:0031644 | regulation of neurological system process | Biological process | 5 | 279 | 63 | 13692 | 3.89486260454002 | 0.00910003326691797 | 0.289090911899771 | 2.04095702002918 | BHLHE40//EGR1//TNR//GFAP//RTN4 |
| GO:0046434 | organophosphate catabolic process | Biological process | 6 | 396 | 63 | 13692 | 3.29292929292929 | 0.00955312220460742 | 0.301301350683446 | 2.01985466667514 | NT5E//CNP//ACAP2//RGN//CHN2//CHN1 |
| GO:0006928 | cellular component movement | Biological process | 10 | 932 | 63 | 13692 | 2.33190271816881 | 0.0096336386639904 | 0.301670513592385 | 2.0162096471204 | ROBO3//SATB2//UGT8//BMP4//P2RY2//RTN4//TNR//LPAR1//CD9 |
| GO:0051129 | negative regulation of cellular component organization | Biological process | 5 | 285 | 63 | 13692 | 3.81286549707602 | 0.00991962674345628 | 0.308423004562499 | 2.00350466920742 | GFAP//TNR//RTN4//LPAR1//BMP4 |
| GO:0061448 | connective tissue development | Biological process | 4 | 184 | 63 | 13692 | 4.72463768115942 | 0.0101519206081758 | 0.313422675677766 | 1.9934517872457 | BMP4//MKX//SATB2//EGR1 |
| GO:0050771 | negative regulation of axonogenesis | Biological process | 2 | 34 | 63 | 13692 | 12.7843137254902 | 0.0106329722606192 | 0.323588980352282 | 1.9733453191123 | TNR//RTN4 |
| GO:0045595 | regulation of cell differentiation | Biological process | 10 | 947 | 63 | 13692 | 2.29496656107005 | 0.0107133077292417 | 0.323588980352282 | 1.97007642022597 | GFAP//P2RY2//TNR//RTN4//LPAR1//BMP4//MKX//S1PR5//SEMA7A//CHN1 |
| GO:0008610 | lipid biosynthetic process | Biological process | 6 | 406 | 63 | 13692 | 3.21182266009852 | 0.0107192893924746 | 0.323588980352282 | 1.96983400411678 | SC5DL//FA2H//UGT8//SLC27A2//NT5E//PLP1 |
| GO:0071840 | cellular component organization or biogenesis | Biological process | 24 | 3342 | 63 | 13692 | 1.5607420706164 | 0.0107764578310751 | 0.323588980352282 | 1.96752396616352 | CNP//CHN2//BMP4//UGT8//SATB2//SPTBN1//ERMN//CD9//ROBO3//RTN4//CHN1//P2RY2//GFAP//TNR//LPAR1//LYZL4//OLFML2B//MKX//PLEKHA1//FA2H//NDRG1//LSM14A//SEMA7A//MGST1 |
| GO:0016044 | cellular membrane organization | Biological process | 5 | 293 | 63 | 13692 | 3.70875995449374 | 0.0110898584858433 | 0.330734283006374 | 1.95507399570923 | SPTBN1//CD9//FA2H//NDRG1//CNP |
| GO:0023051 | regulation of signaling | Biological process | 15 | 1744 | 63 | 13692 | 1.86926605504587 | 0.0112205694627743 | 0.332371463005422 | 1.94998510134504 | LPAR1//RGD1306565//BMP4//ACAP2//CHN2//RTN4//MOG//OTUD7B//BHLHE40//EGR1//TNR//RGN//GFAP//PLEKHA1//SEMA7A |
| GO:0051239 | regulation of multicellular organismal process | Biological process | 15 | 1747 | 63 | 13692 | 1.86605609616485 | 0.0113914596622909 | 0.335168853419351 | 1.94342062336056 | BMP4//GFAP//P2RY2//TNR//RTN4//LPAR1//MKX//WNT10A//FA2H//S1PR5//SEMA7A//BHLHE40//EGR1//CHN1//SCN4B |
| GO:0043270 | positive regulation of ion transport | Biological process | 3 | 103 | 63 | 13692 | 6.33009708737864 | 0.0118456499175778 | 0.340137566946237 | 1.92644110650098 | ITPR1//WFS1//SCN4B |
| GO:0061024 | membrane organization | Biological process | 5 | 298 | 63 | 13692 | 3.64653243847875 | 0.0118675006533128 | 0.340137566946237 | 1.92564073570144 | SPTBN1//CD9//FA2H//NDRG1//CNP |
| GO:0000186 | activation of MAPKK activity | Biological process | 2 | 36 | 63 | 13692 | 12.0740740740741 | 0.0118706769486255 | 0.340137566946237 | 1.92552451384494 | BMP4//RGD1306565 |
| GO:0031103 | axon regeneration | Biological process | 2 | 36 | 63 | 13692 | 12.0740740740741 | 0.0118706769486255 | 0.340137566946237 | 1.92552451384494 | RTN4//TNR |
| GO:0022603 | regulation of anatomical structure morphogenesis | Biological process | 7 | 543 | 63 | 13692 | 2.80171884591774 | 0.0120356937132615 | 0.342029871117536 | 1.91952887280409 | BMP4//ERMN//TNR//RTN4//WNT10A//SEMA7A//CHN1 |
| GO:0010721 | negative regulation of cell development | Biological process | 3 | 104 | 63 | 13692 | 6.26923076923077 | 0.0121580953864089 | 0.342029871117536 | 1.9151344536783 | TNR//RTN4//BMP4 |
| GO:0006875 | cellular metal ion homeostasis | Biological process | 5 | 300 | 63 | 13692 | 3.62222222222222 | 0.0121887351879663 | 0.342029871117536 | 1.91404135831813 | RGN//ITPR1//P2RY2//WFS1//LPAR1 |
| GO:0044270 | cellular nitrogen compound catabolic process | Biological process | 6 | 418 | 63 | 13692 | 3.11961722488038 | 0.0122487887238716 | 0.342029871117536 | 1.91190685632379 | NT5E//CNP//ACAP2//RGN//CHN2//CHN1 |
| GO:0071841 | cellular component organization or biogenesis at cellular level | Biological process | 20 | 2640 | 63 | 13692 | 1.64646464646465 | 0.0125117793979133 | 0.342832450952067 | 1.90268092150238 | CNP//CHN2//UGT8//SATB2//ERMN//ROBO3//RTN4//CHN1//P2RY2//GFAP//TNR//LPAR1//OLFML2B//MKX//CD9//PLEKHA1//LSM14A//SEMA7A//BMP4//SPTBN1 |
| GO:0007043 | cell-cell junction assembly | Biological process | 2 | 37 | 63 | 13692 | 11.7477477477477 | 0.0125121332464258 | 0.342832450952067 | 1.90266863929387 | CD9//UGT8 |
| GO:0072210 | metanephric nephron development | Biological process | 2 | 37 | 63 | 13692 | 11.7477477477477 | 0.0125121332464258 | 0.342832450952067 | 1.90266863929387 | BMP4//EGR1 |
| GO:0034599 | cellular response to oxidative stress | Biological process | 3 | 107 | 63 | 13692 | 6.09345794392523 | 0.0131237937047342 | 0.352015489242162 | 1.88194060498515 | PLEKHA1//RGD1306565//MGST1 |
| GO:0014015 | positive regulation of gliogenesis | Biological process | 2 | 38 | 63 | 13692 | 11.4385964912281 | 0.0131684626450079 | 0.352015489242162 | 1.88046492388116 | GFAP//RTN4 |
| GO:0014910 | regulation of smooth muscle cell migration | Biological process | 2 | 38 | 63 | 13692 | 11.4385964912281 | 0.0131684626450079 | 0.352015489242162 | 1.88046492388116 | P2RY2//LPAR1 |
| GO:0022029 | telencephalon cell migration | Biological process | 2 | 38 | 63 | 13692 | 11.4385964912281 | 0.0131684626450079 | 0.352015489242162 | 1.88046492388116 | RTN4//TNR |
| GO:0044283 | small molecule biosynthetic process | Biological process | 5 | 307 | 63 | 13692 | 3.53963083604777 | 0.0133597422292518 | 0.352825963452048 | 1.87420192131358 | SC5DL//FA2H//SLC27A2//RGN//PLP1 |
| GO:0051345 | positive regulation of hydrolase activity | Biological process | 5 | 307 | 63 | 13692 | 3.53963083604777 | 0.0133597422292518 | 0.352825963452048 | 1.87420192131358 | RGD1306565//RPL5//RGN//CHN2//CHN1 |
| GO:0008585 | female gonad development | Biological process | 3 | 108 | 63 | 13692 | 6.03703703703704 | 0.013455182524374 | 0.353218683753626 | 1.87111040649995 | BMP4//PLEKHA1//WNT10A |
| GO:0016043 | cellular component organization | Biological process | 23 | 3233 | 63 | 13692 | 1.54613877719353 | 0.0144899339749905 | 0.374729305308793 | 1.83893359343584 | CNP//CHN2//BMP4//UGT8//SATB2//SPTBN1//ERMN//CD9//ROBO3//RTN4//CHN1//P2RY2//GFAP//TNR//LPAR1//OLFML2B//MKX//PLEKHA1//FA2H//NDRG1//LSM14A//SEMA7A//MGST1 |
| GO:0009755 | hormone-mediated signaling pathway | Biological process | 2 | 40 | 63 | 13692 | 10.8666666666667 | 0.0145251581165504 | 0.374729305308793 | 1.83787913131136 | BMP4//GCGR |
| GO:0030811 | regulation of nucleotide catabolic process | Biological process | 4 | 205 | 63 | 13692 | 4.24065040650406 | 0.0146164943448457 | 0.374729305308793 | 1.83515677712185 | ACAP2//RGN//CHN2//CHN1 |
| GO:0033121 | regulation of purine nucleotide catabolic process | Biological process | 4 | 205 | 63 | 13692 | 4.24065040650406 | 0.0146164943448457 | 0.374729305308793 | 1.83515677712185 | ACAP2//RGN//CHN2//CHN1 |
| GO:0008406 | gonad development | Biological process | 4 | 206 | 63 | 13692 | 4.22006472491909 | 0.0148555482270442 | 0.378643740856755 | 1.82811131642263 | BMP4//PLEKHA1//WNT10A//MGST1 |
| GO:0001676 | long-chain fatty acid metabolic process | Biological process | 2 | 41 | 63 | 13692 | 10.6016260162602 | 0.0152252353457077 | 0.379246771338537 | 1.81743598548969 | PLP1//SLC27A2 |
| GO:0014909 | smooth muscle cell migration | Biological process | 2 | 41 | 63 | 13692 | 10.6016260162602 | 0.0152252353457077 | 0.379246771338537 | 1.81743598548969 | P2RY2//LPAR1 |
| GO:0021885 | forebrain cell migration | Biological process | 2 | 41 | 63 | 13692 | 10.6016260162602 | 0.0152252353457077 | 0.379246771338537 | 1.81743598548969 | RTN4//TNR |
| GO:0048742 | regulation of skeletal muscle fiber development | Biological process | 2 | 41 | 63 | 13692 | 10.6016260162602 | 0.0152252353457077 | 0.379246771338537 | 1.81743598548969 | BMP4//MKX |
| GO:0055065 | metal ion homeostasis | Biological process | 5 | 321 | 63 | 13692 | 3.3852544132918 | 0.0159261795778208 | 0.39446537440207 | 1.7978883916352 | RGN//ITPR1//P2RY2//WFS1//LPAR1 |
| GO:0007267 | cell-cell signaling | Biological process | 8 | 715 | 63 | 13692 | 2.43170163170163 | 0.0162620613395448 | 0.400521780407665 | 1.78882440513502 | BMP4//RTN4//PRIMA1//BHLHE40//EGR1//TNR//GFAP//GJC2 |
| GO:0009056 | catabolic process | Biological process | 13 | 1487 | 63 | 13692 | 1.90002241649854 | 0.0163867633057596 | 0.401338381745531 | 1.78550681929485 | SLC27A2//NT5E//CNP//PNLIP//LYZL4//WFS1//ACAP2//RGN//CHN2//PRIMA1//CHN1//CES1D//PTPN3 |
| GO:0006633 | fatty acid biosynthetic process | Biological process | 3 | 117 | 63 | 13692 | 5.57264957264957 | 0.016652962596493 | 0.403718782281974 | 1.77850849339764 | PLP1//SC5DL//FA2H |
| GO:0045445 | myoblast differentiation | Biological process | 2 | 43 | 63 | 13692 | 10.1085271317829 | 0.0166681340312585 | 0.403718782281974 | 1.77811301596602 | BMP4//MKX |
| GO:0048519 | negative regulation of biological process | Biological process | 20 | 2719 | 63 | 13692 | 1.59862694618119 | 0.0171074776301068 | 0.412083417199935 | 1.76681401921869 | EGR1//BMP4//BHLHE40//MKX//SATB2//TNR//CD9//GFAP//RTN4//LPAR1//FOXN3//P2RY2//WFS1//OTUD7B//SERBP1//PTPN3//NT5E//ITPR1//PLEKHA1 |
| GO:0000003 | reproduction | Biological process | 10 | 1027 | 63 | 13692 | 2.11619604024667 | 0.0181191672031798 | 0.430510774779859 | 1.74186176733113 | BMP4//PLEKHA1//CHN2//EIF4G3//CLDN11//CD9//WNT10A//MGST1//P2RY2//ZAR1 |
| GO:0014812 | muscle cell migration | Biological process | 2 | 45 | 63 | 13692 | 9.65925925925926 | 0.0181670833335479 | 0.430510774779859 | 1.74071479166951 | P2RY2//LPAR1 |
| GO:0032835 | glomerulus development | Biological process | 2 | 45 | 63 | 13692 | 9.65925925925926 | 0.0181670833335479 | 0.430510774779859 | 1.74071479166951 | BMP4//EGR1 |
| GO:0048762 | mesenchymal cell differentiation | Biological process | 3 | 122 | 63 | 13692 | 5.34426229508197 | 0.0185982016285458 | 0.438357612578198 | 1.73052904828524 | WNT10A//RTN4//BMP4 |
| GO:0030003 | cellular cation homeostasis | Biological process | 5 | 337 | 63 | 13692 | 3.22453016815035 | 0.0192419243105063 | 0.451104792391763 | 1.71575149801315 | RGN//ITPR1//P2RY2//WFS1//LPAR1 |
| GO:0032963 | collagen metabolic process | Biological process | 2 | 47 | 63 | 13692 | 9.24822695035461 | 0.0197209666779336 | 0.459822369771107 | 1.70507180072327 | BMP4//MKX |
| GO:0010927 | cellular component assembly involved in morphogenesis | Biological process | 3 | 125 | 63 | 13692 | 5.216 | 0.0198235465070117 | 0.459822369771107 | 1.70281864598391 | CHN2//CD9//UGT8 |
| GO:0035556 | intracellular signal transduction | Biological process | 13 | 1527 | 63 | 13692 | 1.85025103689151 | 0.020051160516798 | 0.462201457289047 | 1.69786048631283 | RGD1306565//PLEKHA1//ITPR1//NDRG1//LPAR1//OTUD7B//RGN//BMP4//SEMA7A//CHN1//CHN2//PLCL1 |
| GO:0032101 | regulation of response to external stimulus | Biological process | 5 | 341 | 63 | 13692 | 3.18670576735093 | 0.0201369704247737 | 0.462201457289047 | 1.69600586778304 | RTN4//TNR//SEMA7A//NT5E//LPAR1 |
| GO:0046545 | development of primary female sexual characteristics | Biological process | 3 | 127 | 63 | 13692 | 5.13385826771654 | 0.0206647520942694 | 0.471845172819151 | 1.68476980038647 | BMP4//PLEKHA1//WNT10A |
| GO:0050877 | neurological system process | Biological process | 17 | 2227 | 63 | 13692 | 1.65903307888041 | 0.020903272498241 | 0.474818376333101 | 1.6797857178759 | WFS1//RPE65//EGR1//TNR//PLP1//CLDN11//P2RY2//CD9//UGT8//FA2H//NDRG1//PRIMA1//MAL//LPAR1//BHLHE40//GFAP//RTN4 |
| GO:0045685 | regulation of glial cell differentiation | Biological process | 2 | 49 | 63 | 13692 | 8.87074829931973 | 0.021328682698155 | 0.479512538198521 | 1.67103596661287 | RTN4//BMP4 |
| GO:0090183 | regulation of kidney development | Biological process | 2 | 49 | 63 | 13692 | 8.87074829931973 | 0.021328682698155 | 0.479512538198521 | 1.67103596661287 | BMP4//EGR1 |
| GO:0044259 | multicellular organismal macromolecule metabolic process | Biological process | 2 | 50 | 63 | 13692 | 8.69333333333333 | 0.0221523878994608 | 0.495490145669572 | 1.65457945248095 | BMP4//MKX |
| GO:0050804 | regulation of synaptic transmission | Biological process | 4 | 233 | 63 | 13692 | 3.7310443490701 | 0.0222684776556216 | 0.495558406305813 | 1.65230947171143 | BHLHE40//EGR1//TNR//GFAP |
| GO:0045137 | development of primary sexual characteristics | Biological process | 4 | 234 | 63 | 13692 | 3.71509971509971 | 0.0225794354150287 | 0.499940630603464 | 1.64628692154316 | BMP4//PLEKHA1//WNT10A//MGST1 |
| GO:0046660 | female sex differentiation | Biological process | 3 | 132 | 63 | 13692 | 4.93939393939394 | 0.0228529717506513 | 0.501414984756233 | 1.64105731721376 | BMP4//PLEKHA1//WNT10A |
| GO:0070301 | cellular response to hydrogen peroxide | Biological process | 2 | 51 | 63 | 13692 | 8.52287581699346 | 0.0229891450583948 | 0.501414984756233 | 1.63847717937559 | PLEKHA1//RGD1306565 |
| GO:0071347 | cellular response to interleukin-1 | Biological process | 2 | 51 | 63 | 13692 | 8.52287581699346 | 0.0229891450583948 | 0.501414984756233 | 1.63847717937559 | EGR1//P2RY2 |
| GO:0050769 | positive regulation of neurogenesis | Biological process | 3 | 133 | 63 | 13692 | 4.90225563909774 | 0.0233052316296118 | 0.505792749822862 | 1.63254657627732 | GFAP//RTN4//SEMA7A |
| GO:0044248 | cellular catabolic process | Biological process | 11 | 1232 | 63 | 13692 | 1.94047619047619 | 0.0237600700219259 | 0.511940012618698 | 1.62415228380485 | SLC27A2//NT5E//CNP//WFS1//ACAP2//RGN//CHN2//PRIMA1//CHN1//CES1D//PTPN3 |
| GO:0065007 | biological regulation | Biological process | 45 | 8017 | 63 | 13692 | 1.21990769614569 | 0.0238220261346292 | 0.511940012618698 | 1.62302130323665 | EGR1//BMP4//BHLHE40//MKX//SATB2//RGD1306565//LPAR1//EIF4G3//WFS1//TNR//CHN1//CHN2//PLCL1//RGN//MAL//PRUNE2//SPTBN1//GCGR//S1PR5//ITPR1//P2RY2//PLP1//SEMA7A//PLEKHA1//CD9//ERMN//CLDN11//GFAP//WNT10A//RPL5//RTN4//NT5E//FOXN3//NDRG1//UGT8//FA2H//ACAP2//MOG//SCN4B//PRIMA1//OTUD7B//SERBP1//PTPN3//CNP//NTS |
| GO:0048511 | rhythmic process | Biological process | 4 | 240 | 63 | 13692 | 3.62222222222222 | 0.0245011646489503 | 0.523966369858527 | 1.61081327120319 | BMP4//PLEKHA1//EGR1//BHLHE40 |
| GO:0007229 | integrin-mediated signaling pathway | Biological process | 2 | 53 | 63 | 13692 | 8.20125786163522 | 0.024701282277247 | 0.525681657783742 | 1.60728050133637 | PLP1//SEMA7A |
| GO:0006195 | purine nucleotide catabolic process | Biological process | 5 | 361 | 63 | 13692 | 3.01015697137581 | 0.0250237311705951 | 0.529971195419753 | 1.60164793421145 | NT5E//ACAP2//RGN//CHN2//CHN1 |
| GO:0051050 | positive regulation of transport | Biological process | 6 | 492 | 63 | 13692 | 2.65040650406504 | 0.0252180741343259 | 0.53151940867733 | 1.59828808290058 | P2RY2//RTN4//ITPR1//WFS1//BMP4//SCN4B |
| GO:0060688 | regulation of morphogenesis of a branching structure | Biological process | 2 | 54 | 63 | 13692 | 8.04938271604938 | 0.0255763981395718 | 0.536492485377429 | 1.59216061616109 | BMP4//RTN4 |
| GO:0019953 | sexual reproduction | Biological process | 6 | 495 | 63 | 13692 | 2.63434343434343 | 0.025884621241426 | 0.540372283440055 | 1.58695818548074 | CHN2//BMP4//CLDN11//EIF4G3//PLEKHA1//CD9 |
| GO:0072523 | purine-containing compound catabolic process | Biological process | 5 | 366 | 63 | 13692 | 2.96903460837887 | 0.0263551374783059 | 0.544687045234058 | 1.57913471399981 | NT5E//ACAP2//RGN//CHN2//CHN1 |
| GO:0051179 | localization | Biological process | 23 | 3407 | 63 | 13692 | 1.46717542314842 | 0.0264353426106477 | 0.544687045234058 | 1.5778150566245 | ROBO3//SATB2//UGT8//SLC44A1//LMBRD1//SCN4B//ITPR1//CLCN2//MAL//GCGR//SPTBN1//BMP4//P2RY2//SLC27A2//RTN4//TNR//PNLIP//PLEKHA1//CNP//GFAP//PRIMA1//WFS1//LPAR1 |
| GO:0045767 | regulation of anti-apoptosis | Biological process | 2 | 55 | 63 | 13692 | 7.9030303030303 | 0.0264640375535708 | 0.544687045234058 | 1.57734389584516 | RTN4//SERBP1 |
| GO:0070848 | response to growth factor stimulus | Biological process | 4 | 249 | 63 | 13692 | 3.49129852744311 | 0.0275653822612323 | 0.564703905762815 | 1.55963598059111 | EGR1//BMP4//WNT10A//P2RY2 |
| GO:0060485 | mesenchyme development | Biological process | 3 | 143 | 63 | 13692 | 4.55944055944056 | 0.0280956848519542 | 0.572890615771941 | 1.55136037720853 | WNT10A//RTN4//BMP4 |
| GO:0048641 | regulation of skeletal muscle tissue development | Biological process | 2 | 58 | 63 | 13692 | 7.49425287356322 | 0.0292008032345927 | 0.592668154539141 | 1.53460520212852 | BMP4//MKX |
| GO:0031329 | regulation of cellular catabolic process | Biological process | 5 | 379 | 63 | 13692 | 2.86719437115215 | 0.0300278118751746 | 0.601707078067084 | 1.5224763136605 | ACAP2//RGN//CHN2//CHN1//PTPN3 |
| GO:0051969 | regulation of transmission of nerve impulse | Biological process | 4 | 256 | 63 | 13692 | 3.39583333333333 | 0.0301010176345887 | 0.601707078067084 | 1.52141882182756 | BHLHE40//EGR1//TNR//GFAP |
| GO:0048168 | regulation of neuronal synaptic plasticity | Biological process | 2 | 59 | 63 | 13692 | 7.36723163841808 | 0.0301372474435804 | 0.601707078067084 | 1.52089641607758 | EGR1//BHLHE40 |
| GO:0043085 | positive regulation of catalytic activity | Biological process | 7 | 655 | 63 | 13692 | 2.32264631043257 | 0.0301951544650453 | 0.601707078067084 | 1.52006274439164 | BMP4//RGD1306565//LPAR1//RPL5//RGN//CHN2//CHN1 |
| GO:0007548 | sex differentiation | Biological process | 4 | 259 | 63 | 13692 | 3.35649935649936 | 0.0312288552230123 | 0.619490051120751 | 1.50544393570068 | BMP4//PLEKHA1//WNT10A//MGST1 |
| GO:0043065 | positive regulation of apoptotic process | Biological process | 6 | 520 | 63 | 13692 | 2.50769230769231 | 0.0319031961020885 | 0.624391888093888 | 1.49616580659893 | MAL//PRUNE2//RGD1306565//EGR1//BMP4//LPAR1 |
| GO:0007422 | peripheral nervous system development | Biological process | 2 | 61 | 63 | 13692 | 7.12568306010929 | 0.0320456603150376 | 0.624391888093888 | 1.49423077515738 | NDRG1//FA2H |
| GO:0015908 | fatty acid transport | Biological process | 2 | 61 | 63 | 13692 | 7.12568306010929 | 0.0320456603150376 | 0.624391888093888 | 1.49423077515738 | P2RY2//SLC27A2 |
| GO:0051153 | regulation of striated muscle cell differentiation | Biological process | 2 | 61 | 63 | 13692 | 7.12568306010929 | 0.0320456603150376 | 0.624391888093888 | 1.49423077515738 | BMP4//MKX |
| GO:0009605 | response to external stimulus | Biological process | 10 | 1128 | 63 | 13692 | 1.92671394799054 | 0.0322537789201825 | 0.625666224717169 | 1.49141939519808 | BMP4//ROBO3//CHN1//BHLHE40//EGR1//RTN4//TNR//SEMA7A//NT5E//LPAR1 |
| GO:0043068 | positive regulation of programmed cell death | Biological process | 6 | 524 | 63 | 13692 | 2.48854961832061 | 0.0329447066902218 | 0.636253718634063 | 1.48221435466327 | MAL//PRUNE2//RGD1306565//BMP4//LPAR1//EGR1 |
| GO:0055080 | cation homeostasis | Biological process | 5 | 390 | 63 | 13692 | 2.78632478632479 | 0.0333777428902735 | 0.641789582591926 | 1.4765430350457 | RGN//ITPR1//P2RY2//WFS1//LPAR1 |
| GO:0044236 | multicellular organismal metabolic process | Biological process | 2 | 63 | 63 | 13692 | 6.8994708994709 | 0.0340006074241237 | 0.650911191909861 | 1.46851332417573 | BMP4//MKX |
| GO:0051234 | establishment of localization | Biological process | 19 | 2726 | 63 | 13692 | 1.51479579359257 | 0.0343400291679811 | 0.654550816836649 | 1.46419934029077 | SLC44A1//LMBRD1//SCN4B//ITPR1//CLCN2//MAL//GCGR//SPTBN1//UGT8//SLC27A2//PNLIP//P2RY2//RTN4//PLEKHA1//CNP//GFAP//PRIMA1//WFS1//BMP4 |
| GO:0006631 | fatty acid metabolic process | Biological process | 4 | 269 | 63 | 13692 | 3.23172242874845 | 0.035167753523239 | 0.667426110155324 | 1.45385537304278 | SLC27A2//SC5DL//FA2H//PLP1 |
| GO:0071407 | cellular response to organic cyclic compound | Biological process | 2 | 65 | 63 | 13692 | 6.68717948717949 | 0.0360011028235572 | 0.680296701631357 | 1.44368419526467 | EGR1//P2RY2 |
| GO:0032535 | regulation of cellular component size | Biological process | 3 | 158 | 63 | 13692 | 4.12658227848101 | 0.0361900065882903 | 0.680931282759934 | 1.44141133782969 | TNR//RTN4//SEMA7A |
| GO:0015850 | organic alcohol transport | Biological process | 2 | 66 | 63 | 13692 | 6.58585858585859 | 0.0370181267670295 | 0.693536186951527 | 1.43158556168592 | SLC44A1//RTN4 |
| GO:0065009 | regulation of molecular function | Biological process | 12 | 1493 | 63 | 13692 | 1.74681848626926 | 0.0376704461603405 | 0.702754195603969 | 1.42399923607954 | BMP4//RGD1306565//LPAR1//RPL5//ACAP2//RGN//CHN2//BHLHE40//WFS1//CHN1//FOXN3//SCN4B |
| GO:0060021 | palate development | Biological process | 2 | 67 | 63 | 13692 | 6.48756218905473 | 0.0380461742400182 | 0.706548024363881 | 1.41968900748693 | PLEKHA1//SATB2 |
| GO:0010942 | positive regulation of cell death | Biological process | 6 | 543 | 63 | 13692 | 2.40147329650092 | 0.0381961409156569 | 0.706548024363881 | 1.41798051310131 | MAL//PRUNE2//RGD1306565//BMP4//LPAR1//EGR1 |
| GO:0048608 | reproductive structure development | Biological process | 4 | 277 | 63 | 13692 | 3.13838748495788 | 0.0385186652608432 | 0.706551583696806 | 1.41432877035056 | BMP4//PLEKHA1//WNT10A//MGST1 |
| GO:0061458 | reproductive system development | Biological process | 4 | 277 | 63 | 13692 | 3.13838748495788 | 0.0385186652608432 | 0.706551583696806 | 1.41432877035056 | BMP4//PLEKHA1//WNT10A//MGST1 |
| GO:0006869 | lipid transport | Biological process | 3 | 164 | 63 | 13692 | 3.97560975609756 | 0.0397297562688083 | 0.712308042687102 | 1.40088409921437 | SLC27A2//PNLIP//P2RY2 |
| GO:0032147 | activation of protein kinase activity | Biological process | 3 | 164 | 63 | 13692 | 3.97560975609756 | 0.0397297562688083 | 0.712308042687102 | 1.40088409921437 | BMP4//RGD1306565//LPAR1 |
| GO:0007420 | brain development | Biological process | 6 | 549 | 63 | 13692 | 2.3752276867031 | 0.0399604581964346 | 0.712308042687102 | 1.39836954069024 | BMP4//RTN4//TNR//CNP//CD9//NT5E |
| GO:0045216 | cell-cell junction organization | Biological process | 2 | 69 | 63 | 13692 | 6.29951690821256 | 0.0401348629126874 | 0.712308042687102 | 1.39647821608985 | CD9//UGT8 |
| GO:0051928 | positive regulation of calcium ion transport | Biological process | 2 | 69 | 63 | 13692 | 6.29951690821256 | 0.0401348629126874 | 0.712308042687102 | 1.39647821608985 | ITPR1//WFS1 |
| GO:0046700 | heterocycle catabolic process | Biological process | 5 | 412 | 63 | 13692 | 2.63754045307443 | 0.0407593140206728 | 0.712308042687102 | 1.38977313378101 | NT5E//ACAP2//RGN//CHN2//CHN1 |
| GO:0007610 | behavior | Biological process | 6 | 552 | 63 | 13692 | 2.36231884057971 | 0.0408619359965207 | 0.712308042687102 | 1.38868106079002 | EGR1//TNR//CNP//WFS1//LPAR1//PLCL1 |
| GO:0048678 | response to axon injury | Biological process | 2 | 71 | 63 | 13692 | 6.12206572769953 | 0.0422662234335257 | 0.712308042687102 | 1.37400655546535 | RTN4//TNR |
| GO:0022414 | reproductive process | Biological process | 9 | 1018 | 63 | 13692 | 1.92141453831041 | 0.0423316311534114 | 0.712308042687102 | 1.37333499665055 | BMP4//PLEKHA1//CHN2//EIF4G3//CLDN11//CD9//WNT10A//MGST1//P2RY2 |
| GO:0009798 | axis specification | Biological process | 2 | 72 | 63 | 13692 | 6.03703703703704 | 0.0433476138092292 | 0.712308042687102 | 1.36303480448552 | BMP4//WNT10A |
| GO:0034614 | cellular response to reactive oxygen species | Biological process | 2 | 73 | 63 | 13692 | 5.95433789954338 | 0.044439323589275 | 0.712308042687102 | 1.35223256007503 | PLEKHA1//RGD1306565 |
| GO:0007204 | elevation of cytosolic calcium ion concentration | Biological process | 3 | 172 | 63 | 13692 | 3.7906976744186 | 0.0447137994500944 | 0.712308042687102 | 1.34955842541037 | ITPR1//LPAR1//P2RY2 |
| GO:0048589 | developmental growth | Biological process | 4 | 291 | 63 | 13692 | 2.98739977090493 | 0.0448120858797033 | 0.712308042687102 | 1.34860484039935 | TNR//RTN4//SEMA7A//BMP4 |
| GO:0006677 | glycosylceramide metabolic process | Biological process | 1 | 10 | 63 | 13692 | 21.7333333333333 | 0.0450856720952101 | 0.712308042687102 | 1.34596145188303 | UGT8 |
| GO:0010044 | response to aluminum ion | Biological process | 1 | 10 | 63 | 13692 | 21.7333333333333 | 0.0450856720952101 | 0.712308042687102 | 1.34596145188303 | NT5E |
| GO:0010935 | regulation of macrophage cytokine production | Biological process | 1 | 10 | 63 | 13692 | 21.7333333333333 | 0.0450856720952101 | 0.712308042687102 | 1.34596145188303 | SEMA7A |
| GO:0016338 | calcium-independent cell-cell adhesion | Biological process | 1 | 10 | 63 | 13692 | 21.7333333333333 | 0.0450856720952101 | 0.712308042687102 | 1.34596145188303 | CLDN11 |
| GO:0021801 | cerebral cortex radial glia guided migration | Biological process | 1 | 10 | 63 | 13692 | 21.7333333333333 | 0.0450856720952101 | 0.712308042687102 | 1.34596145188303 | RTN4 |
| GO:0033194 | response to hydroperoxide | Biological process | 1 | 10 | 63 | 13692 | 21.7333333333333 | 0.0450856720952101 | 0.712308042687102 | 1.34596145188303 | MGST1 |
| GO:0035590 | purinergic nucleotide receptor signaling pathway | Biological process | 1 | 10 | 63 | 13692 | 21.7333333333333 | 0.0450856720952101 | 0.712308042687102 | 1.34596145188303 | P2RY2 |
| GO:0042135 | neurotransmitter catabolic process | Biological process | 1 | 10 | 63 | 13692 | 21.7333333333333 | 0.0450856720952101 | 0.712308042687102 | 1.34596145188303 | PRIMA1 |
| GO:0042364 | water-soluble vitamin biosynthetic process | Biological process | 1 | 10 | 63 | 13692 | 21.7333333333333 | 0.0450856720952101 | 0.712308042687102 | 1.34596145188303 | RGN |
| GO:0043587 | tongue morphogenesis | Biological process | 1 | 10 | 63 | 13692 | 21.7333333333333 | 0.0450856720952101 | 0.712308042687102 | 1.34596145188303 | BMP4 |
| GO:0060390 | regulation of SMAD protein import into nucleus | Biological process | 1 | 10 | 63 | 13692 | 21.7333333333333 | 0.0450856720952101 | 0.712308042687102 | 1.34596145188303 | BMP4 |
| GO:0060405 | regulation of penile erection | Biological process | 1 | 10 | 63 | 13692 | 21.7333333333333 | 0.0450856720952101 | 0.712308042687102 | 1.34596145188303 | P2RY2 |
| GO:0060513 | prostatic bud formation | Biological process | 1 | 10 | 63 | 13692 | 21.7333333333333 | 0.0450856720952101 | 0.712308042687102 | 1.34596145188303 | BMP4 |
| GO:0072044 | collecting duct development | Biological process | 1 | 10 | 63 | 13692 | 21.7333333333333 | 0.0450856720952101 | 0.712308042687102 | 1.34596145188303 | BMP4 |
| GO:0072203 | cell proliferation involved in metanephros development | Biological process | 1 | 10 | 63 | 13692 | 21.7333333333333 | 0.0450856720952101 | 0.712308042687102 | 1.34596145188303 | EGR1 |
| GO:1900542 | regulation of purine nucleotide metabolic process | Biological process | 4 | 293 | 63 | 13692 | 2.96700796359499 | 0.0457558056182465 | 0.712308042687102 | 1.33955379366302 | ACAP2//RGN//CHN2//CHN1 |
| GO:0010646 | regulation of cell communication | Biological process | 11 | 1369 | 63 | 13692 | 1.74628682736791 | 0.0463320678592165 | 0.712308042687102 | 1.33411831624826 | BMP4//RTN4//MOG//LPAR1//OTUD7B//BHLHE40//EGR1//TNR//GFAP//PLEKHA1//SEMA7A |
| GO:0042475 | odontogenesis of dentin-containing tooth | Biological process | 2 | 75 | 63 | 13692 | 5.79555555555555 | 0.0466532442051634 | 0.712308042687102 | 1.33111815060105 | BMP4//WNT10A |
| GO:0048583 | regulation of response to stimulus | Biological process | 14 | 1898 | 63 | 13692 | 1.60309097295399 | 0.0470738384572144 | 0.712308042687102 | 1.32722038735688 | LPAR1//RGD1306565//BMP4//ACAP2//CHN2//MOG//OTUD7B//RTN4//TNR//SEMA7A//NT5E//RGN//PLEKHA1//EGR1 |
| GO:0001656 | metanephros development | Biological process | 2 | 76 | 63 | 13692 | 5.71929824561404 | 0.0477752284706727 | 0.712308042687102 | 1.32079722738514 | BMP4//EGR1 |
| GO:0016477 | cell migration | Biological process | 7 | 725 | 63 | 13692 | 2.0983908045977 | 0.0480676304248358 | 0.712308042687102 | 1.31814728662491 | ROBO3//SATB2//BMP4//P2RY2//RTN4//TNR//LPAR1 |
| GO:0045926 | negative regulation of growth | Biological process | 3 | 178 | 63 | 13692 | 3.66292134831461 | 0.0486474419280916 | 0.712308042687102 | 1.31293999169003 | RTN4//TNR//BMP4 |
| GO:0006140 | regulation of nucleotide metabolic process | Biological process | 4 | 299 | 63 | 13692 | 2.90746934225195 | 0.0486539108722895 | 0.712308042687102 | 1.3128822447689 | ACAP2//RGN//CHN2//CHN1 |
| GO:0007276 | gamete generation | Biological process | 5 | 434 | 63 | 13692 | 2.50384024577573 | 0.0490681148315388 | 0.712308042687102 | 1.30920062704022 | CHN2//BMP4//CLDN11//EIF4G3//PLEKHA1 |
| GO:0001675 | acrosome assembly | Biological process | 1 | 11 | 63 | 13692 | 19.7575757575758 | 0.0494826610338154 | 0.712308042687102 | 1.30554695331884 | CHN2 |
| GO:0003128 | heart field specification | Biological process | 1 | 11 | 63 | 13692 | 19.7575757575758 | 0.0494826610338154 | 0.712308042687102 | 1.30554695331884 | BMP4 |
| GO:0003323 | type B pancreatic cell development | Biological process | 1 | 11 | 63 | 13692 | 19.7575757575758 | 0.0494826610338154 | 0.712308042687102 | 1.30554695331884 | BMP4 |
| GO:0010934 | macrophage cytokine production | Biological process | 1 | 11 | 63 | 13692 | 19.7575757575758 | 0.0494826610338154 | 0.712308042687102 | 1.30554695331884 | SEMA7A |
| GO:0015697 | quaternary ammonium group transport | Biological process | 1 | 11 | 63 | 13692 | 19.7575757575758 | 0.0494826610338154 | 0.712308042687102 | 1.30554695331884 | SLC44A1 |
| GO:0033962 | cytoplasmic mRNA processing body assembly | Biological process | 1 | 11 | 63 | 13692 | 19.7575757575758 | 0.0494826610338154 | 0.712308042687102 | 1.30554695331884 | LSM14A |
| GO:0034123 | positive regulation of toll-like receptor signaling pathway | Biological process | 1 | 11 | 63 | 13692 | 19.7575757575758 | 0.0494826610338154 | 0.712308042687102 | 1.30554695331884 | MOG |
| GO:0035640 | exploration behavior | Biological process | 1 | 11 | 63 | 13692 | 19.7575757575758 | 0.0494826610338154 | 0.712308042687102 | 1.30554695331884 | TNR |
| GO:0044241 | lipid digestion | Biological process | 1 | 11 | 63 | 13692 | 19.7575757575758 | 0.0494826610338154 | 0.712308042687102 | 1.30554695331884 | PNLIP |
| GO:0046085 | adenosine metabolic process | Biological process | 1 | 11 | 63 | 13692 | 19.7575757575758 | 0.0494826610338154 | 0.712308042687102 | 1.30554695331884 | NT5E |
| GO:0060363 | cranial suture morphogenesis | Biological process | 1 | 11 | 63 | 13692 | 19.7575757575758 | 0.0494826610338154 | 0.712308042687102 | 1.30554695331884 | BMP4 |
| GO:0061036 | positive regulation of cartilage development | Biological process | 1 | 11 | 63 | 13692 | 19.7575757575758 | 0.0494826610338154 | 0.712308042687102 | 1.30554695331884 | BMP4 |
| GO:0070233 | negative regulation of T cell apoptotic process | Biological process | 1 | 11 | 63 | 13692 | 19.7575757575758 | 0.0494826610338154 | 0.712308042687102 | 1.30554695331884 | BMP4 |
| GO:0070243 | regulation of thymocyte apoptotic process | Biological process | 1 | 11 | 63 | 13692 | 19.7575757575758 | 0.0494826610338154 | 0.712308042687102 | 1.30554695331884 | BMP4 |
| GO:0072498 | embryonic skeletal joint development | Biological process | 1 | 11 | 63 | 13692 | 19.7575757575758 | 0.0494826610338154 | 0.712308042687102 | 1.30554695331884 | BMP4 |
| GO:0051336 | regulation of hydrolase activity | Biological process | 6 | 579 | 63 | 13692 | 2.25215889464594 | 0.0495627719372226 | 0.712308042687102 | 1.30484441247807 | RGD1306565//RPL5//ACAP2//RGN//CHN2//CHN1 |
| GO:0031099 | regeneration | Biological process | 3 | 180 | 63 | 13692 | 3.62222222222222 | 0.0499954595468985 | 0.712308042687102 | 1.30106943532929 | GFAP//RTN4//TNR |
