# Supplementary material for: Gene Expression in the Hippocampus in a Rat Model of Premenstrual Dysphoric Disorder After Treatment With Baixiangdan Capsules
Source: Front Psychol. 2018 Nov 13;9:2065. doi: 10.3389/fpsyg.2018.02065 (PMC6242977; doi:10.3389/fpsyg.2018.02065)
Supplement: Supplementary file 3 [file Data_Sheet_3.ZIP › Data Analysis Folder/GO Analysis Report/fluoxetine vs blank (down)/CC_result(Rat).html]

| GO.ID | Term | Ontology | Count | Pop.Hits | List.Total | Pop.Total | Fold.Enrichment | Pvalue | FDR | Enrichment.Score | GENES |
| --- | --- | --- | --- | --- | --- | --- | --- | --- | --- | --- | --- |
| GO:0043209 | myelin sheath | Cellular component | 5 | 41 | 64 | 15288 | 29.1310975609756 | 7.31628180565004e-07 | 0.000392884332963407 | 6.1357095748903 | CNP//PLP1//RTN4//ERMN//GJC2 |
| GO:0005886 | plasma membrane | Cellular component | 23 | 2986 | 64 | 15288 | 1.83996148693905 | 0.00156795933023716 | 0.268821162700105 | 2.8046652062451 | SCN4B//PLP1//GJC2//CLDN11//CLDN12//CD9//SEMA7A//PTPN3//P2RY2//PRIMA1//PLEKHA1//GCGR//ITPR1//CNP//NT5E//S1PR5//RTN4//RPE65//LPAR1//SERBP1//NDRG1//SPTBN1//WNT10A |
| GO:0005788 | endoplasmic reticulum lumen | Cellular component | 3 | 61 | 64 | 15288 | 11.7479508196721 | 0.00211809742098022 | 0.268821162700105 | 2.67405406858117 | SLC27A2//CES1D//FKBP9 |
| GO:0071944 | cell periphery | Cellular component | 23 | 3081 | 64 | 15288 | 1.78322784810127 | 0.00240986675553758 | 0.268821162700105 | 2.61800696943116 | GCGR//ITPR1//CNP//NT5E//S1PR5//RTN4//RPE65//LPAR1//SERBP1//NDRG1//SPTBN1//SCN4B//WNT10A//PLEKHA1//PLP1//GJC2//CLDN11//CLDN12//CD9//SEMA7A//PTPN3//P2RY2//PRIMA1 |
| GO:0044432 | endoplasmic reticulum part | Cellular component | 8 | 570 | 64 | 15288 | 3.35263157894737 | 0.0025029903417142 | 0.268821162700105 | 2.60154082620013 | SLC27A2//CES1D//FKBP9//ITPR1//MGST1//FA2H//WFS1//RTN4 |
| GO:0030176 | integral to endoplasmic reticulum membrane | Cellular component | 3 | 70 | 64 | 15288 | 10.2375 | 0.00313663390336427 | 0.280728734351102 | 2.50353616763537 | SLC27A2//WFS1//RTN4 |
| GO:0005911 | cell-cell junction | Cellular component | 5 | 264 | 64 | 15288 | 4.52414772727273 | 0.00491816684566469 | 0.310883880576339 | 2.30819674218486 | NDRG1//GJC2//CLDN11//CLDN12//SCN4B |
| GO:0044464 | cell part | Cellular component | 58 | 11835 | 64 | 15288 | 1.17065906210393 | 0.00515381884453083 | 0.310883880576339 | 2.28787085086143 | GCGR//ITPR1//CNP//NT5E//S1PR5//RTN4//RPE65//LPAR1//SERBP1//NDRG1//SPTBN1//SCN4B//WNT10A//PLEKHA1//SATB2//MAL//UGT8//CHN1//CHN2//PLCL1//FUS//LSM14A//GFAP//RPL5//P2RY2//MOBP//SLC27A2//MGST1//CES1D//WFS1//FKBP9//FA2H//PLP1//EGR1//RGN//TNR//BHLHE40//MKX//OTUD7B//BMP4//BCAS1//PRUNE2//ERMN//ZAR1//PTPN3//FOXN3//LMBRD1//OPALIN//NTS//GJC2//CLDN11//CLDN12//CD9//SEMA7A//CLCN2//ROBO3//PRIMA1//CDC40 |
| GO:0005623 | cell | Cellular component | 58 | 11848 | 64 | 15288 | 1.16937457798785 | 0.00538170818513549 | 0.310883880576339 | 2.2690798548854 | GCGR//ITPR1//CNP//NT5E//S1PR5//RTN4//RPE65//LPAR1//SERBP1//NDRG1//SPTBN1//SCN4B//WNT10A//PLEKHA1//SATB2//MAL//UGT8//CHN1//CHN2//PLCL1//FUS//LSM14A//GFAP//RPL5//P2RY2//MOBP//SLC27A2//MGST1//CES1D//WFS1//FKBP9//FA2H//PLP1//EGR1//RGN//TNR//BHLHE40//MKX//OTUD7B//BMP4//BCAS1//PRUNE2//ERMN//ZAR1//PTPN3//FOXN3//LMBRD1//OPALIN//NTS//GJC2//CLDN11//CLDN12//CD9//SEMA7A//CLCN2//ROBO3//PRIMA1//CDC40 |
| GO:0031227 | intrinsic to endoplasmic reticulum membrane | Cellular component | 3 | 89 | 64 | 15288 | 8.05196629213483 | 0.0061511432853633 | 0.310883880576339 | 2.21104415635817 | SLC27A2//WFS1//RTN4 |
| GO:0005783 | endoplasmic reticulum | Cellular component | 10 | 974 | 64 | 15288 | 2.45251540041068 | 0.00689329521314764 | 0.310883880576339 | 2.16157312195618 | SLC27A2//CES1D//FKBP9//ITPR1//MGST1//FA2H//WFS1//RTN4//MAL//RPE65 |
| GO:0016020 | membrane | Cellular component | 40 | 7097 | 64 | 15288 | 1.34634352543328 | 0.00694712582293494 | 0.310883880576339 | 2.1581948353258 | GCGR//ITPR1//CNP//NT5E//S1PR5//RTN4//RPE65//LPAR1//SERBP1//NDRG1//SPTBN1//SCN4B//WNT10A//PLEKHA1//MOG//CD9//MAL//P2RY2//UGT8//CLDN11//SLC44A1//SC5DL//MGST1//LMBRD1//FA2H//OPALIN//GJC2//FAM18B2//PRIMA1//SLC27A2//PLP1//CLDN12//SEMA7A//PTPN3//WFS1//CLCN2//TNR//GFAP//CHN2//FKBP9 |
| GO:0005778 | peroxisomal membrane | Cellular component | 2 | 48 | 64 | 15288 | 9.953125 | 0.0171957909708077 | 0.659581410808838 | 1.76457784273487 | SLC27A2//MGST1 |
| GO:0031903 | microbody membrane | Cellular component | 2 | 48 | 64 | 15288 | 9.953125 | 0.0171957909708077 | 0.659581410808838 | 1.76457784273487 | SLC27A2//MGST1 |
| GO:0005789 | endoplasmic reticulum membrane | Cellular component | 6 | 508 | 64 | 15288 | 2.82135826771654 | 0.0192717079057585 | 0.689927143026154 | 1.71507979540275 | SLC27A2//WFS1//RTN4//ITPR1//MGST1//FA2H |
| GO:0042175 | nuclear outer membrane-endoplasmic reticulum membrane network | Cellular component | 6 | 522 | 64 | 15288 | 2.74568965517241 | 0.0217054976184924 | 0.694992063623594 | 1.66343025311079 | ITPR1//SLC27A2//MGST1//FA2H//WFS1//RTN4 |
| GO:0031301 | integral to organelle membrane | Cellular component | 3 | 143 | 64 | 15288 | 5.01136363636364 | 0.0220475753602965 | 0.694992063623594 | 1.65663916427065 | SLC27A2//WFS1//RTN4 |
| GO:0005737 | cytoplasm | Cellular component | 40 | 7544 | 64 | 15288 | 1.26656945917285 | 0.0232958233616847 | 0.694992063623594 | 1.63272193535025 | LSM14A//P2RY2//MOBP//SLC27A2//MGST1//CES1D//NDRG1//MAL//WFS1//RTN4//RPE65//FKBP9//FA2H//RPL5//CNP//LMBRD1//GCGR//ITPR1//BHLHE40//OPALIN//NTS//LPAR1//SPTBN1//SERBP1//EGR1//GFAP//RGN//BMP4//TNR//NT5E//PLCL1//BCAS1//PRUNE2//ERMN//OTUD7B//FUS//ZAR1//PLEKHA1//PTPN3//SATB2 |
| GO:0044438 | microbody part | Cellular component | 2 | 60 | 64 | 15288 | 7.9625 | 0.0261331008252605 | 0.701673757158245 | 1.5828090559672 | SLC27A2//MGST1 |
| GO:0044439 | peroxisomal part | Cellular component | 2 | 60 | 64 | 15288 | 7.9625 | 0.0261331008252605 | 0.701673757158245 | 1.5828090559672 | SLC27A2//MGST1 |
| GO:0042995 | cell projection | Cellular component | 10 | 1211 | 64 | 15288 | 1.97254335260116 | 0.0281290348434973 | 0.719299605283717 | 1.55084516898426 | CNP//ITPR1//BCAS1//RTN4//ERMN//ROBO3//CLCN2//PLEKHA1//LPAR1//GFAP |
| GO:0031300 | intrinsic to organelle membrane | Cellular component | 3 | 163 | 64 | 15288 | 4.39647239263804 | 0.0308772330246549 | 0.753685187919986 | 1.51036162465641 | SLC27A2//WFS1//RTN4 |
| GO:0044459 | plasma membrane part | Cellular component | 11 | 1426 | 64 | 15288 | 1.84265427769986 | 0.0333452815896991 | 0.778539835376888 | 1.47696561075886 | SCN4B//PLP1//GJC2//CLDN11//CLDN12//CD9//SEMA7A//PTPN3//P2RY2//PRIMA1//PLEKHA1 |
| GO:0043005 | neuron projection | Cellular component | 7 | 742 | 64 | 15288 | 2.25353773584906 | 0.0348592312673449 | 0.779975299606842 | 1.45768219439273 | ITPR1//BCAS1//ROBO3//CLCN2//ERMN//LPAR1//RTN4 |
| GO:0044297 | cell body | Cellular component | 5 | 441 | 64 | 15288 | 2.70833333333333 | 0.0371029510285871 | 0.785207937997673 | 1.43059154686844 | ITPR1//RTN4//ERMN//CLCN2//GFAP |
| GO:0044425 | membrane part | Cellular component | 31 | 5668 | 64 | 15288 | 1.30647935779817 | 0.0410402145362742 | 0.785207937997673 | 1.38679037763122 | MOG//CD9//GCGR//ITPR1//MAL//P2RY2//UGT8//NT5E//S1PR5//RTN4//CLDN11//SLC44A1//SC5DL//LPAR1//MGST1//LMBRD1//FA2H//OPALIN//GJC2//FAM18B2//PRIMA1//SCN4B//PLP1//SLC27A2//CLDN12//SEMA7A//PTPN3//WFS1//PLEKHA1//CLCN2//TNR |
| GO:0031430 | M band | Cellular component | 1 | 11 | 64 | 15288 | 21.7159090909091 | 0.0451117654242689 | 0.785207937997673 | 1.34571017669268 | SPTBN1 |
| GO:0005923 | tight junction | Cellular component | 2 | 82 | 64 | 15288 | 5.82621951219512 | 0.0462536602613139 | 0.785207937997673 | 1.33485389387864 | CLDN11//CLDN12 |
| GO:0070160 | occluding junction | Cellular component | 2 | 82 | 64 | 15288 | 5.82621951219512 | 0.0462536602613139 | 0.785207937997673 | 1.33485389387864 | CLDN11//CLDN12 |
| GO:0043197 | dendritic spine | Cellular component | 3 | 192 | 64 | 15288 | 3.732421875 | 0.0465073931520681 | 0.785207937997673 | 1.33247800302559 | ITPR1//BCAS1//LPAR1 |
| GO:0044309 | neuron spine | Cellular component | 3 | 192 | 64 | 15288 | 3.732421875 | 0.0465073931520681 | 0.785207937997673 | 1.33247800302559 | ITPR1//BCAS1//LPAR1 |
| GO:0030054 | cell junction | Cellular component | 6 | 627 | 64 | 15288 | 2.28588516746411 | 0.0467907896013511 | 0.785207937997673 | 1.3298396259637 | NDRG1//GJC2//CLDN11//CLDN12//SCN4B//PRIMA1 |
