# Supplementary material for: Gene Expression in the Hippocampus in a Rat Model of Premenstrual Dysphoric Disorder After Treatment With Baixiangdan Capsules
Source: Front Psychol. 2018 Nov 13;9:2065. doi: 10.3389/fpsyg.2018.02065 (PMC6242977; doi:10.3389/fpsyg.2018.02065)
Supplement: Supplementary file 3 [file Data_Sheet_3.ZIP › Data Analysis Folder/GO Analysis Report/fluoxetine vs blank (down)/MF_result(Rat).html]

| GO.ID | Term | Ontology | Count | Pop.Hits | List.Total | Pop.Total | Fold.Enrichment | Pvalue | FDR | Enrichment.Score | GENES |
| --- | --- | --- | --- | --- | --- | --- | --- | --- | --- | --- | --- |
| GO:0005178 | integrin binding | Molecular function | 4 | 60 | 62 | 14392 | 15.4752688172043 | 0.000127095287748996 | 0.114004473110849 | 3.89587055127563 | GFAP//CD9//TNR//SEMA7A |
| GO:0052689 | carboxylic ester hydrolase activity | Molecular function | 4 | 104 | 62 | 14392 | 8.92803970223325 | 0.00104060075114494 | 0.315093983444106 | 2.9827158649497 | CES1D//RGN//PNLIP//RPE65 |
| GO:0016788 | hydrolase activity, acting on ester bonds | Molecular function | 9 | 639 | 62 | 14392 | 3.2694229895502 | 0.00156394562663632 | 0.315093983444106 | 2.80577835003723 | CES1D//CNP//OTUD7B//RGN//PLCL1//PTPN3//PNLIP//NT5E//RPE65 |
| GO:0050253 | retinyl-palmitate esterase activity | Molecular function | 2 | 14 | 62 | 14392 | 33.1612903225806 | 0.00160719226030453 | 0.315093983444106 | 2.79393216767041 | PNLIP//CES1D |
| GO:0032403 | protein complex binding | Molecular function | 7 | 410 | 62 | 14392 | 3.96317859952793 | 0.00184456059380482 | 0.315093983444106 | 2.73410707363163 | GFAP//CD9//TNR//SEMA7A//ITPR1//RTN4//SPTBN1 |
| GO:0004806 | triglyceride lipase activity | Molecular function | 2 | 16 | 62 | 14392 | 29.0161290322581 | 0.00210765206317128 | 0.315093983444106 | 2.67620108203475 | PNLIP//CES1D |
| GO:0005200 | structural constituent of cytoskeleton | Molecular function | 2 | 25 | 62 | 14392 | 18.5703225806452 | 0.00513945990262489 | 0.658585076093504 | 2.28908251789852 | GFAP//SPTBN1 |
| GO:0051117 | ATPase binding | Molecular function | 2 | 27 | 62 | 14392 | 17.194743130227 | 0.00598001945317184 | 0.670509681186893 | 2.2232974032371 | WFS1//PTPN3 |
| GO:0016298 | lipase activity | Molecular function | 3 | 92 | 62 | 14392 | 7.56942496493689 | 0.00728263024518298 | 0.708604179974695 | 2.13771173955186 | PLCL1//PNLIP//CES1D |
| GO:0005198 | structural molecule activity | Molecular function | 8 | 672 | 62 | 14392 | 2.76344086021505 | 0.00789971215133439 | 0.708604179974695 | 2.1023887331861 | RPL5//GFAP//SPTBN1//PLP1//MOBP//MAL//CLDN11//CLDN12 |
| GO:0005515 | protein binding | Molecular function | 29 | 4586 | 62 | 14392 | 1.46788965012732 | 0.00987748199465481 | 0.80546375901867 | 2.00535375330728 | MKX//BHLHE40//WNT10A//PTPN3//LPAR1//ERMN//TNR//SLC27A2//BMP4//GFAP//CD9//SEMA7A//NTS//NDRG1//FOXN3//EGR1//CLDN11//MGST1//PRIMA1//RGD1306565//ITPR1//PLEKHA1//SPTBN1//WFS1//P2RY2//RTN4//OLFML2B//ROBO3//SCN4B |
| GO:0000981 | sequence-specific DNA binding RNA polymerase II transcription factor activity | Molecular function | 4 | 219 | 62 | 14392 | 4.23979967594638 | 0.0146287118205129 | 1 | 1.83479391542454 | EGR1//BHLHE40//FOXN3//MKX |
| GO:0033613 | activating transcription factor binding | Molecular function | 2 | 44 | 62 | 14392 | 10.5513196480938 | 0.0153794129705022 | 1 | 1.81306024116249 | BHLHE40//WFS1 |
| GO:0005488 | binding | Molecular function | 48 | 9220 | 62 | 14392 | 1.20848086208103 | 0.0170699174473772 | 1 | 1.76776857919196 | NT5E//FUS//CD9//ITPR1//P2RY2//SLC27A2//RTN4//RGD1306565//EGR1//MKX//BHLHE40//RGN//PLCL1//FKBP9//WNT10A//PTPN3//LPAR1//EIF4G3//OTUD7B//SATB2//FOXN3//CNP//SERBP1//ERMN//TNR//BMP4//GFAP//SEMA7A//NTS//CHN1//CHN2//RPE65//SC5DL//ACAP2//NDRG1//RPL5//CLDN11//MGST1//GCGR//PRIMA1//FA2H//OLFML2B//PLEKHA1//SPTBN1//LMBRD1//WFS1//ROBO3//SCN4B |
| GO:0008134 | transcription factor binding | Molecular function | 5 | 378 | 62 | 14392 | 3.07048984468339 | 0.0232016647097721 | 1 | 1.63448085354312 | MKX//BHLHE40//WFS1//EGR1//FOXN3 |
| GO:0001085 | RNA polymerase II transcription factor binding | Molecular function | 2 | 59 | 62 | 14392 | 7.86878075451066 | 0.0266953008932279 | 1 | 1.5735651796747 | BHLHE40//MKX |
| GO:0005543 | phospholipid binding | Molecular function | 5 | 403 | 62 | 14392 | 2.88001280717202 | 0.0295213988596278 | 1 | 1.52986306749183 | ITPR1//PLEKHA1//PLCL1//LPAR1//ACAP2 |
| GO:0005096 | GTPase activator activity | Molecular function | 3 | 161 | 62 | 14392 | 4.32538569424965 | 0.0321446340830875 | 1 | 1.49289151364492 | ACAP2//CHN2//CHN1 |
| GO:0042803 | protein homodimerization activity | Molecular function | 6 | 558 | 62 | 14392 | 2.49601109954908 | 0.0325072802975199 | 1 | 1.48801936397629 | BMP4//BHLHE40//MGST1//OLFML2B//ROBO3//RGD1306565 |
| GO:0008047 | enzyme activator activity | Molecular function | 4 | 284 | 62 | 14392 | 3.2694229895502 | 0.0339140332457454 | 1 | 1.46962055843155 | CHN1//ACAP2//RGD1306565//CHN2 |
| GO:0005102 | receptor binding | Molecular function | 9 | 1052 | 62 | 14392 | 1.98589476266405 | 0.0353139323790722 | 1 | 1.45205391892963 | WNT10A//BMP4//GFAP//CD9//TNR//SEMA7A//NTS//P2RY2//SLC27A2 |
| GO:0019899 | enzyme binding | Molecular function | 9 | 1076 | 62 | 14392 | 1.94159971219571 | 0.0399324989988775 | 1 | 1.39867351055541 | NDRG1//GFAP//RGD1306565//ITPR1//WFS1//EGR1//PTPN3//SLC27A2//PRIMA1 |
| GO:0008253 | 5'-nucleotidase activity | Molecular function | 1 | 10 | 62 | 14392 | 23.2129032258064 | 0.0422668435625908 | 1 | 1.37400018355337 | NT5E |
| GO:0042578 | phosphoric ester hydrolase activity | Molecular function | 4 | 309 | 62 | 14392 | 3.00490656644744 | 0.0440158879120073 | 1 | 1.35639053289902 | CNP//PLCL1//PTPN3//NT5E |
| GO:0008656 | cysteine-type endopeptidase activator activity involved in apoptotic process | Molecular function | 1 | 11 | 62 | 14392 | 21.1026392961877 | 0.0463955777928174 | 1 | 1.33352341236975 | RGD1306565 |
| GO:0070696 | transmembrane receptor protein serine/threonine kinase binding | Molecular function | 1 | 11 | 62 | 14392 | 21.1026392961877 | 0.0463955777928174 | 1 | 1.33352341236975 | BMP4 |
| GO:0019903 | protein phosphatase binding | Molecular function | 2 | 80 | 62 | 14392 | 5.80322580645161 | 0.0465591844706578 | 1 | 1.33199463538126 | ITPR1//RGD1306565 |
| GO:0008081 | phosphoric diester hydrolase activity | Molecular function | 2 | 81 | 62 | 14392 | 5.731581043409 | 0.0476083764248121 | 1 | 1.32231662889659 | CNP//PLCL1 |
