# Supplementary material for: Gene Expression in the Hippocampus in a Rat Model of Premenstrual Dysphoric Disorder After Treatment With Baixiangdan Capsules
Source: Front Psychol. 2018 Nov 13;9:2065. doi: 10.3389/fpsyg.2018.02065 (PMC6242977; doi:10.3389/fpsyg.2018.02065)
Supplement: Supplementary file 3 [file Data_Sheet_3.ZIP › Data Analysis Folder/GO Analysis Report/fluoxetine vs blank (up)/BP_result(Rat).html]

| GO.ID | Term | Ontology | Count | Pop.Hits | List.Total | Pop.Total | Fold.Enrichment | Pvalue | FDR | Enrichment.Score | GENES |
| --- | --- | --- | --- | --- | --- | --- | --- | --- | --- | --- | --- |
| GO:0008015 | blood circulation | Biological process | 14 | 312 | 72 | 13692 | 8.53311965811966 | 6.9237464382302e-10 | 1.58236621607639e-06 | 9.15965884525849 | CALCA//DRD2//HSD11B2//P2RX2//TACR1//PTPRO//NPPA//TAC1//ADRA2A//TACR3//SLIT2//HRH3//HTR2C//HTR2A |
| GO:0003013 | circulatory system process | Biological process | 14 | 313 | 72 | 13692 | 8.50585729499467 | 7.21882397845067e-10 | 1.58236621607639e-06 | 9.14153354775856 | CALCA//DRD2//HSD11B2//P2RX2//TACR1//PTPRO//NPPA//TAC1//ADRA2A//TACR3//SLIT2//HRH3//HTR2C//HTR2A |
| GO:0008217 | regulation of blood pressure | Biological process | 10 | 148 | 72 | 13692 | 12.8490990990991 | 4.85071393106676e-09 | 7.08850995793223e-06 | 8.31419433696382 | CALCA//DRD2//HSD11B2//P2RX2//TACR1//PTPRO//HRH3//TACR3//NPPA//TAC1 |
| GO:0007631 | feeding behavior | Biological process | 8 | 95 | 72 | 13692 | 16.0140350877193 | 3.31131835494209e-08 | 3.62920491701653e-05 | 7.47999906354024 | TACR1//HRH3//TACR3//MC4R//CALCA//DRD2//HTR2C//NPW |
| GO:0007610 | behavior | Biological process | 15 | 552 | 72 | 13692 | 5.16757246376812 | 1.37694922172e-07 | 0.00012073090776041 | 6.86108207509051 | TACR1//SNCG//SLIT2//HRH3//HTR2A//ITGA8//DRD2//TAC1//CALCA//HTR2C//MC4R//NPW//SCN9A//P2RX2//TACR3 |
| GO:0007204 | elevation of cytosolic calcium ion concentration | Biological process | 9 | 172 | 72 | 13692 | 9.95058139534884 | 2.72752431792262e-07 | 0.000199291110162879 | 6.5642313686617 | CALCA//DRD2//HTR2C//HTR2A//EPOR//TAC1//TACR1//TRHR//HRH3 |
| GO:0051480 | cytosolic calcium ion homeostasis | Biological process | 9 | 187 | 72 | 13692 | 9.1524064171123 | 5.53309303048023e-07 | 0.000345081061333496 | 6.25703202772827 | CALCA//EPOR//TAC1//TACR1//TRHR//HRH3//DRD2//HTR2C//HTR2A |
| GO:0048265 | response to pain | Biological process | 5 | 32 | 72 | 13692 | 29.7135416666667 | 6.29709965937036e-07 | 0.000345081061333496 | 6.20085943339762 | TACR1//SCN9A//P2RX2//CALCA//TAC1 |
| GO:0003001 | generation of a signal involved in cell-cell signaling | Biological process | 11 | 325 | 72 | 13692 | 6.43641025641026 | 9.45441474392747e-07 | 0.00041448154237378 | 6.02436535009231 | HRH3//HTR2C//DRD2//HTR2A//SNCG//TACR1//MC4R//NNAT//ADRA2A//LRP2//TAC1 |
| GO:0023061 | signal release | Biological process | 11 | 325 | 72 | 13692 | 6.43641025641026 | 9.45441474392747e-07 | 0.00041448154237378 | 6.02436535009231 | HRH3//HTR2C//DRD2//HTR2A//SNCG//TACR1//MC4R//NNAT//ADRA2A//LRP2//TAC1 |
| GO:0065008 | regulation of biological quality | Biological process | 28 | 2106 | 72 | 13692 | 2.52833175055397 | 1.06009311984761e-06 | 0.000422495294310175 | 5.97465598411286 | DRD2//HTR2A//SCX//CALCA//HSD11B2//P2RX2//TACR1//TAC1//PTPRO//MT4//EPOR//TRHR//HRH3//NPPA//ADRA2A//TACR3//HTR2C//SCN9A//MC4R//SLIT2//NNAT//CHRNE//DIO3//BHLHA15//TRPV2//LRP2//SNCG//CAMP |
| GO:0033555 | multicellular organismal response to stress | Biological process | 6 | 67 | 72 | 13692 | 17.0298507462687 | 1.32512277431091e-06 | 0.000477961124944 | 5.87774388192674 | ADRA2A//CALCA//TAC1//TACR1//SCN9A//P2RX2 |
| GO:0009190 | cyclic nucleotide biosynthetic process | Biological process | 7 | 107 | 72 | 13692 | 12.4408099688474 | 1.41731172998905e-06 | 0.000477961124944 | 5.84853461854247 | NPPA//HTR2C//CALCA//DRD2//HRH3//ADRA2A//MC4R |
| GO:0003073 | regulation of systemic arterial blood pressure | Biological process | 6 | 73 | 72 | 13692 | 15.6301369863014 | 2.20498513689265e-06 | 0.000690475345724098 | 5.65659433362801 | CALCA//DRD2//HSD11B2//P2RX2//TACR1//PTPRO |
| GO:0001976 | neurological system process involved in regulation of systemic arterial blood pressure | Biological process | 4 | 19 | 72 | 13692 | 40.0350877192982 | 2.56638680848471e-06 | 0.000750069317893131 | 5.59067788571241 | CALCA//P2RX2//TACR1//DRD2 |
| GO:0048878 | chemical homeostasis | Biological process | 16 | 807 | 72 | 13692 | 3.77034283353986 | 3.34567068442939e-06 | 0.000857623374897098 | 5.47551680903221 | DRD2//TAC1//TACR1//CALCA//MT4//EPOR//TRHR//HRH3//SCN9A//P2RX2//CHRNE//ADRA2A//BHLHA15//NPPA//HTR2C//HTR2A |
| GO:0006875 | cellular metal ion homeostasis | Biological process | 10 | 300 | 72 | 13692 | 6.33888888888889 | 3.54237907946167e-06 | 0.000857623374897098 | 5.45070496564475 | CALCA//EPOR//TAC1//TACR1//TRHR//HRH3//DRD2//HTR2C//HTR2A//MT4 |
| GO:0007267 | cell-cell signaling | Biological process | 15 | 715 | 72 | 13692 | 3.98951048951049 | 3.61008376460538e-06 | 0.000857623374897098 | 5.44248272106281 | DRD2//TAC1//SNCG//HTR3A//P2RX2//HRH3//HTR2C//HTR2A//TACR1//MC4R//NNAT//SCN9A//ADRA2A//LRP2//BHLHA15 |
| GO:0055082 | cellular chemical homeostasis | Biological process | 14 | 624 | 72 | 13692 | 4.26655982905983 | 3.71688962660695e-06 | 0.000857623374897098 | 5.42982033512899 | CALCA//MT4//EPOR//TAC1//TACR1//TRHR//HRH3//SCN9A//P2RX2//DRD2//CHRNE//HTR2C//HTR2A//ADRA2A |
| GO:0007613 | memory | Biological process | 6 | 83 | 72 | 13692 | 13.7469879518072 | 4.69103724284544e-06 | 0.00102827536363172 | 5.32873111911039 | DRD2//TAC1//TACR1//HTR2A//HRH3//ITGA8 |
| GO:0007200 | phospholipase C-activating G-protein coupled receptor signaling pathway | Biological process | 5 | 48 | 72 | 13692 | 19.8090277777778 | 5.01609215024908e-06 | 0.00104716895174724 | 5.29963449371301 | HTR2C//HTR2A//CALCA//DRD2//ADRA2A |
| GO:0006874 | cellular calcium ion homeostasis | Biological process | 9 | 246 | 72 | 13692 | 6.95731707317073 | 5.35249570917713e-06 | 0.0010666064176833 | 5.27144367218512 | CALCA//EPOR//TAC1//TACR1//TRHR//HRH3//DRD2//HTR2C//HTR2A |
| GO:0009187 | cyclic nucleotide metabolic process | Biological process | 7 | 132 | 72 | 13692 | 10.084595959596 | 5.77250859002206e-06 | 0.00110029033298507 | 5.2386354121403 | NPPA//HTR2C//CALCA//DRD2//HRH3//ADRA2A//MC4R |
| GO:0043279 | response to alkaloid | Biological process | 7 | 134 | 72 | 13692 | 9.93407960199005 | 6.37575797653405e-06 | 0.00113094895981243 | 5.19546817709668 | DRD2//TACR1//HTR2C//TACR3//SNCG//HTR3A//TAC1 |
| GO:0055065 | metal ion homeostasis | Biological process | 10 | 321 | 72 | 13692 | 5.92419522326064 | 6.44929835659462e-06 | 0.00113094895981243 | 5.19048753132356 | DRD2//TAC1//TACR1//CALCA//MT4//EPOR//TRHR//HRH3//HTR2C//HTR2A |
| GO:0055074 | calcium ion homeostasis | Biological process | 9 | 254 | 72 | 13692 | 6.73818897637795 | 6.93737550532508e-06 | 0.00113888931786995 | 5.15880479743114 | CALCA//EPOR//TAC1//TACR1//TRHR//HRH3//DRD2//HTR2C//HTR2A |
| GO:0006873 | cellular ion homeostasis | Biological process | 13 | 568 | 72 | 13692 | 4.35240610328638 | 7.01414497775743e-06 | 0.00113888931786995 | 5.15402526178081 | CALCA//MT4//EPOR//TAC1//TACR1//TRHR//HRH3//SCN9A//P2RX2//DRD2//CHRNE//HTR2C//HTR2A |
| GO:0003018 | vascular process in circulatory system | Biological process | 7 | 138 | 72 | 13692 | 9.64613526570048 | 7.74034221286699e-06 | 0.00121191643790032 | 5.11123983804135 | CALCA//ADRA2A//NPPA//SLIT2//TACR1//HTR2C//HTR2A |
| GO:0032940 | secretion by cell | Biological process | 13 | 577 | 72 | 13692 | 4.28451761987291 | 8.30996326539193e-06 | 0.00125623720536132 | 5.08040089603212 | CBLN4//HRH3//HTR2C//DRD2//HTR2A//SNCG//TACR1//MC4R//NNAT//ADRA2A//LRP2//TGFB3//TAC1 |
| GO:0072503 | cellular divalent inorganic cation homeostasis | Biological process | 9 | 262 | 72 | 13692 | 6.5324427480916 | 8.90895014877649e-06 | 0.0013018945817412 | 5.05017347121082 | CALCA//EPOR//TAC1//TACR1//TRHR//HRH3//DRD2//HTR2C//HTR2A |
| GO:0044057 | regulation of system process | Biological process | 12 | 495 | 72 | 13692 | 4.61010101010101 | 9.29835247888097e-06 | 0.00131496700862626 | 5.03159399474881 | CALCA//DRD2//TAC1//TACR1//PTPRO//ADRA2A//TACR3//HTR2C//NPPA//SCN9A//HTR2A//SNCG |
| GO:0030003 | cellular cation homeostasis | Biological process | 10 | 337 | 72 | 13692 | 5.64292779426311 | 9.88019730594008e-06 | 0.00135358703091379 | 5.0052343825353 | CALCA//MT4//EPOR//TAC1//TACR1//TRHR//HRH3//DRD2//HTR2C//HTR2A |
| GO:0051240 | positive regulation of multicellular organismal process | Biological process | 12 | 502 | 72 | 13692 | 4.54581673306773 | 1.07111314242885e-05 | 0.00142295758072972 | 4.97016465191583 | ADRA2A//MC4R//DRD2//TAC1//TACR1//CALCA//TACR3//TGFB3//SCX//DIO3//HTR2C//HTR2A |
| GO:0072507 | divalent inorganic cation homeostasis | Biological process | 9 | 271 | 72 | 13692 | 6.31549815498155 | 1.16828940834489e-05 | 0.00150640610770118 | 4.93244956058136 | CALCA//EPOR//TAC1//TACR1//TRHR//HRH3//DRD2//HTR2C//HTR2A |
| GO:0051952 | regulation of amine transport | Biological process | 5 | 58 | 72 | 13692 | 16.3936781609195 | 1.28868526002417e-05 | 0.0015793100609766 | 4.88985313891152 | HRH3//HTR2C//DRD2//HTR2A//SNCG |
| GO:0019725 | cellular homeostasis | Biological process | 14 | 696 | 72 | 13692 | 3.82519157088123 | 1.29687869970706e-05 | 0.0015793100609766 | 4.88710064266029 | CALCA//MT4//EPOR//TAC1//TACR1//TRHR//HRH3//SCN9A//P2RX2//DRD2//CHRNE//HTR2C//HTR2A//ADRA2A |
| GO:0042592 | homeostatic process | Biological process | 18 | 1125 | 72 | 13692 | 3.04266666666667 | 1.46544777413094e-05 | 0.00173635757886217 | 4.83402965440801 | DRD2//HTR2A//SCX//TAC1//TACR1//CALCA//MT4//EPOR//TRHR//HRH3//SCN9A//P2RX2//CHRNE//ADRA2A//BHLHA15//MC4R//NPPA//HTR2C |
| GO:0043278 | response to morphine | Biological process | 4 | 30 | 72 | 13692 | 25.3555555555556 | 1.73694117093603e-05 | 0.00200388160352199 | 4.7602148905745 | DRD2//TAC1//TACR1//TACR3 |
| GO:0050801 | ion homeostasis | Biological process | 13 | 626 | 72 | 13692 | 3.94914802981896 | 1.98066523907281e-05 | 0.00217705299340119 | 4.7031889202761 | DRD2//TAC1//TACR1//CALCA//MT4//EPOR//TRHR//HRH3//SCN9A//P2RX2//CHRNE//HTR2C//HTR2A |
| GO:0014072 | response to isoquinoline alkaloid | Biological process | 4 | 31 | 72 | 13692 | 24.5376344086021 | 1.98636222025656e-05 | 0.00217705299340119 | 4.70194155346665 | DRD2//TAC1//TACR1//TACR3 |
| GO:0035815 | positive regulation of renal sodium excretion | Biological process | 3 | 11 | 72 | 13692 | 51.8636363636364 | 2.23211700243461e-05 | 0.00238673193626179 | 4.65128304442087 | DRD2//TAC1//TACR1 |
| GO:0030817 | regulation of cAMP biosynthetic process | Biological process | 5 | 70 | 72 | 13692 | 13.5833333333333 | 3.24148550419131e-05 | 0.00338349344056541 | 4.48925591620632 | CALCA//DRD2//HRH3//ADRA2A//MC4R |
| GO:0055080 | cation homeostasis | Biological process | 10 | 390 | 72 | 13692 | 4.87606837606838 | 3.47685444482172e-05 | 0.00354477439211591 | 4.45881349025833 | DRD2//TAC1//TACR1//CALCA//MT4//EPOR//TRHR//HRH3//HTR2C//HTR2A |
| GO:0007218 | neuropeptide signaling pathway | Biological process | 5 | 72 | 72 | 13692 | 13.2060185185185 | 3.71688711496105e-05 | 0.00370337116181574 | 4.42982062859864 | CALCA//TAC1//TACR1//SSTR1//NPW |
| GO:0007210 | serotonin receptor signaling pathway | Biological process | 3 | 13 | 72 | 13692 | 43.8846153846154 | 3.83985375113098e-05 | 0.00372878560465981 | 4.41568531633204 | HTR2C//HTR2A//HTR3A |
| GO:0030814 | regulation of cAMP metabolic process | Biological process | 5 | 73 | 72 | 13692 | 13.0251141552511 | 3.97397451211339e-05 | 0.00372878560465981 | 4.40077492265248 | CALCA//DRD2//HRH3//ADRA2A//MC4R |
| GO:0050433 | regulation of catecholamine secretion | Biological process | 4 | 37 | 72 | 13692 | 20.5585585585586 | 4.07124395036393e-05 | 0.00372878560465981 | 4.3902728737634 | DRD2//HTR2A//SNCG//HRH3 |
| GO:0050880 | regulation of blood vessel size | Biological process | 6 | 121 | 72 | 13692 | 9.4297520661157 | 4.0826119759049e-05 | 0.00372878560465981 | 4.38906189480913 | CALCA//ADRA2A//NPPA//TACR1//HTR2C//HTR2A |
| GO:0010033 | response to organic substance | Biological process | 22 | 1714 | 72 | 13692 | 2.44087903539479 | 4.2489721261077e-05 | 0.00374927556362579 | 4.37171611793786 | DRD2//TAC1//TACR1//P2RX2//NNAT//SST//NNMT//HRH3//TACR3//NR2F2//TGFB3//NPPA//HSD11B2//MC4R//ADRA2A//SLIT2//HTR2C//EPOR//SSTR1//SNCG//HTR3A//CAMP |
| GO:0035150 | regulation of tube size | Biological process | 6 | 122 | 72 | 13692 | 9.35245901639344 | 4.27608983077759e-05 | 0.00374927556362579 | 4.36895317984277 | CALCA//ADRA2A//NPPA//TACR1//HTR2C//HTR2A |
| GO:0032844 | regulation of homeostatic process | Biological process | 8 | 247 | 72 | 13692 | 6.15924426450742 | 4.42721655582352e-05 | 0.003805670074653 | 4.35386923413162 | DRD2//TAC1//TACR1//CALCA//MC4R//HTR2C//HTR2A//ADRA2A |
| GO:0006939 | smooth muscle contraction | Biological process | 5 | 77 | 72 | 13692 | 12.3484848484848 | 5.14295308918168e-05 | 0.00433590506595625 | 4.28878743702491 | P2RX2//DRD2//CALCA//TACR1//TACR3 |
| GO:0046903 | secretion | Biological process | 13 | 688 | 72 | 13692 | 3.59326550387597 | 5.29444259255919e-05 | 0.00437940308033575 | 4.27617975637208 | DRD2//TAC1//TACR1//CBLN4//HRH3//HTR2C//HTR2A//SNCG//MC4R//NNAT//ADRA2A//LRP2//TGFB3 |
| GO:0014073 | response to tropane | Biological process | 4 | 40 | 72 | 13692 | 19.0166666666667 | 5.56694202052064e-05 | 0.00443735887599318 | 4.25438330182923 | DRD2//TACR3//SNCG//HTR3A |
| GO:0042220 | response to cocaine | Biological process | 4 | 40 | 72 | 13692 | 19.0166666666667 | 5.56694202052064e-05 | 0.00443735887599318 | 4.25438330182923 | DRD2//TACR3//SNCG//HTR3A |
| GO:2000021 | regulation of ion homeostasis | Biological process | 6 | 129 | 72 | 13692 | 8.84496124031008 | 5.8460293790226e-05 | 0.00457660585672055 | 4.23313900644089 | DRD2//TAC1//TACR1//CALCA//HTR2C//HTR2A |
| GO:0048266 | behavioral response to pain | Biological process | 3 | 15 | 72 | 13692 | 38.0333333333333 | 6.06285226646929e-05 | 0.00464428344232116 | 4.21732301409043 | TACR1//SCN9A//P2RX2 |
| GO:0050432 | catecholamine secretion | Biological process | 4 | 41 | 72 | 13692 | 18.5528455284553 | 6.14435309431175e-05 | 0.00464428344232116 | 4.21152383488655 | DRD2//HTR2A//SNCG//HRH3 |
| GO:0006171 | cAMP biosynthetic process | Biological process | 5 | 81 | 72 | 13692 | 11.738683127572 | 6.56160076769929e-05 | 0.00487560301111757 | 4.18299019724466 | CALCA//DRD2//HRH3//ADRA2A//MC4R |
| GO:0045778 | positive regulation of ossification | Biological process | 4 | 42 | 72 | 13692 | 18.1111111111111 | 6.7642459096202e-05 | 0.00494240901129583 | 4.16978061230289 | TGFB3//CALCA//TAC1//TACR1 |
| GO:0003014 | renal system process | Biological process | 5 | 82 | 72 | 13692 | 11.5955284552846 | 6.95930241627862e-05 | 0.00500157078573204 | 4.15743429084119 | HSD11B2//DRD2//TAC1//TACR1//PTPRO |
| GO:0015837 | amine transport | Biological process | 6 | 136 | 72 | 13692 | 8.38970588235294 | 7.84661789496616e-05 | 0.00548979299261937 | 4.10531749561916 | HRH3//HTR2C//DRD2//HTR2A//SNCG//SLC7A3 |
| GO:0046879 | hormone secretion | Biological process | 7 | 198 | 72 | 13692 | 6.72306397306397 | 7.9147016225095e-05 | 0.00548979299261937 | 4.10156545302401 | TACR1//MC4R//NNAT//DRD2//ADRA2A//TAC1//LRP2 |
| GO:0051046 | regulation of secretion | Biological process | 10 | 432 | 72 | 13692 | 4.40200617283951 | 8.20759758933953e-05 | 0.00548979299261937 | 4.0857839447534 | DRD2//TAC1//TACR1//HRH3//HTR2C//HTR2A//SNCG//NNAT//ADRA2A//TGFB3 |
| GO:0030802 | regulation of cyclic nucleotide biosynthetic process | Biological process | 5 | 85 | 72 | 13692 | 11.1862745098039 | 8.26474310020252e-05 | 0.00548979299261937 | 4.08277064141479 | CALCA//DRD2//HRH3//ADRA2A//MC4R |
| GO:1900371 | regulation of purine nucleotide biosynthetic process | Biological process | 5 | 85 | 72 | 13692 | 11.1862745098039 | 8.26474310020252e-05 | 0.00548979299261937 | 4.08277064141479 | CALCA//DRD2//HRH3//ADRA2A//MC4R |
| GO:0030808 | regulation of nucleotide biosynthetic process | Biological process | 5 | 86 | 72 | 13692 | 11.0562015503876 | 8.73928756673569e-05 | 0.0057183636854581 | 4.05852396993385 | CALCA//DRD2//HRH3//ADRA2A//MC4R |
| GO:0006164 | purine nucleotide biosynthetic process | Biological process | 7 | 203 | 72 | 13692 | 6.55747126436782 | 9.25508097147085e-05 | 0.00596680514396003 | 4.03361977730987 | NPPA//HTR2C//CALCA//DRD2//HRH3//ADRA2A//MC4R |
| GO:0010817 | regulation of hormone levels | Biological process | 9 | 354 | 72 | 13692 | 4.83474576271187 | 9.44285432537909e-05 | 0.00599963382064666 | 4.02489671010967 | HSD11B2//TACR1//MC4R//NNAT//DIO3//DRD2//ADRA2A//LRP2//TAC1 |
| GO:0002027 | regulation of heart rate | Biological process | 4 | 46 | 72 | 13692 | 16.536231884058 | 9.7065564983024e-05 | 0.00607907766979396 | 4.01293481324599 | TAC1//TACR3//CALCA//DRD2 |
| GO:0090066 | regulation of anatomical structure size | Biological process | 8 | 277 | 72 | 13692 | 5.49217809867629 | 9.89579779584017e-05 | 0.00611030669534694 | 4.00454918737328 | CALCA//ADRA2A//SLIT2//NPPA//TRPV2//TACR1//HTR2C//HTR2A |
| GO:0031644 | regulation of neurological system process | Biological process | 8 | 279 | 72 | 13692 | 5.45280764635603 | 0.000104025904830762 | 0.00629846400936147 | 3.98285849796906 | HTR2C//CALCA//TAC1//TACR1//DRD2//SCN9A//SNCG//HTR2A |
| GO:0030803 | negative regulation of cyclic nucleotide biosynthetic process | Biological process | 3 | 18 | 72 | 13692 | 31.6944444444444 | 0.000107506222181478 | 0.00629846400936147 | 3.96856639918205 | DRD2//HRH3//ADRA2A |
| GO:0030818 | negative regulation of cAMP biosynthetic process | Biological process | 3 | 18 | 72 | 13692 | 31.6944444444444 | 0.000107506222181478 | 0.00629846400936147 | 3.96856639918205 | DRD2//HRH3//ADRA2A |
| GO:0009914 | hormone transport | Biological process | 7 | 208 | 72 | 13692 | 6.39983974358974 | 0.000107752007459423 | 0.00629846400936147 | 3.96757463002636 | TACR1//MC4R//NNAT//DRD2//ADRA2A//LRP2//TAC1 |
| GO:0072522 | purine-containing compound biosynthetic process | Biological process | 7 | 209 | 72 | 13692 | 6.36921850079745 | 0.000111023399700102 | 0.00640429716164799 | 3.95458547806353 | NPPA//HTR2C//CALCA//DRD2//HRH3//ADRA2A//MC4R |
| GO:0030799 | regulation of cyclic nucleotide metabolic process | Biological process | 5 | 93 | 72 | 13692 | 10.2240143369176 | 0.000126755855448533 | 0.00651415317098682 | 3.89703196942855 | CALCA//DRD2//HRH3//ADRA2A//MC4R |
| GO:0014046 | dopamine secretion | Biological process | 3 | 19 | 72 | 13692 | 30.0263157894737 | 0.000127182441313914 | 0.00651415317098682 | 3.89557284282948 | DRD2//HTR2A//SNCG |
| GO:0014059 | regulation of dopamine secretion | Biological process | 3 | 19 | 72 | 13692 | 30.0263157894737 | 0.000127182441313914 | 0.00651415317098682 | 3.89557284282948 | DRD2//HTR2A//SNCG |
| GO:0030800 | negative regulation of cyclic nucleotide metabolic process | Biological process | 3 | 19 | 72 | 13692 | 30.0263157894737 | 0.000127182441313914 | 0.00651415317098682 | 3.89557284282948 | DRD2//HRH3//ADRA2A |
| GO:0030809 | negative regulation of nucleotide biosynthetic process | Biological process | 3 | 19 | 72 | 13692 | 30.0263157894737 | 0.000127182441313914 | 0.00651415317098682 | 3.89557284282948 | DRD2//HRH3//ADRA2A |
| GO:0030815 | negative regulation of cAMP metabolic process | Biological process | 3 | 19 | 72 | 13692 | 30.0263157894737 | 0.000127182441313914 | 0.00651415317098682 | 3.89557284282948 | DRD2//HRH3//ADRA2A |
| GO:0035812 | renal sodium excretion | Biological process | 3 | 19 | 72 | 13692 | 30.0263157894737 | 0.000127182441313914 | 0.00651415317098682 | 3.89557284282948 | DRD2//TAC1//TACR1 |
| GO:0035813 | regulation of renal sodium excretion | Biological process | 3 | 19 | 72 | 13692 | 30.0263157894737 | 0.000127182441313914 | 0.00651415317098682 | 3.89557284282948 | DRD2//TAC1//TACR1 |
| GO:1900372 | negative regulation of purine nucleotide biosynthetic process | Biological process | 3 | 19 | 72 | 13692 | 30.0263157894737 | 0.000127182441313914 | 0.00651415317098682 | 3.89557284282948 | DRD2//HRH3//ADRA2A |
| GO:0007420 | brain development | Biological process | 11 | 549 | 72 | 13692 | 3.81026108075288 | 0.000127786763846913 | 0.00651415317098682 | 3.89351412807078 | SSTR1//MYO16//KIRREL3//SLIT2//DRD2//LRP2//NR2F2//EPOR//HRH3//NNAT//ITGA8 |
| GO:0046058 | cAMP metabolic process | Biological process | 5 | 97 | 72 | 13692 | 9.80240549828179 | 0.000154645793016507 | 0.00779272593775134 | 3.81066189004534 | CALCA//DRD2//HRH3//ADRA2A//MC4R |
| GO:0051937 | catecholamine transport | Biological process | 4 | 53 | 72 | 13692 | 14.3522012578616 | 0.000169413020664282 | 0.00839702039657764 | 3.77105321392659 | DRD2//HTR2A//SNCG//HRH3 |
| GO:0002548 | monocyte chemotaxis | Biological process | 3 | 21 | 72 | 13692 | 27.1666666666667 | 0.000173251052112691 | 0.00839702039657764 | 3.76132411934041 | SLIT2//CALCA//PTPRO |
| GO:1900543 | negative regulation of purine nucleotide metabolic process | Biological process | 3 | 21 | 72 | 13692 | 27.1666666666667 | 0.000173251052112691 | 0.00839702039657764 | 3.76132411934041 | DRD2//HRH3//ADRA2A |
| GO:0051239 | regulation of multicellular organismal process | Biological process | 21 | 1747 | 72 | 13692 | 2.28591871780195 | 0.000174299465348669 | 0.00839702039657764 | 3.75870394505569 | ADRA2A//MC4R//CALCA//DRD2//SMARCD3//TAC1//TACR1//PTPRO//TACR3//TGFB3//HTR2C//SCX//DIO3//NPPA//SCN9A//CAMP//TRPV2//HTR2A//SNCG//GPR149//SLIT2 |
| GO:0044062 | regulation of excretion | Biological process | 3 | 22 | 72 | 13692 | 25.9318181818182 | 0.000199850796272727 | 0.00952332490064821 | 3.69929411702528 | DRD2//TAC1//TACR1 |
| GO:0032879 | regulation of localization | Biological process | 17 | 1252 | 72 | 13692 | 2.58213525026624 | 0.000206779144422794 | 0.0097475243994573 | 3.68449326595936 | DRD2//TACR1//SLIT2//TAC1//HTR2A//CALCA//NR2F2//HRH3//HTR2C//SNCG//SST//ADRA2A//NNAT//LCP1//SCN11A//TGFB3//TRPV2 |
| GO:0019233 | sensory perception of pain | Biological process | 5 | 104 | 72 | 13692 | 9.14262820512821 | 0.000214485451219167 | 0.0100032363632429 | 3.66860216114603 | CALCA//HTR2C//TAC1//TACR1//HTR2A |
| GO:0050804 | regulation of synaptic transmission | Biological process | 7 | 233 | 72 | 13692 | 5.71316165951359 | 0.000217338918397926 | 0.0100296191395422 | 3.66286249856642 | HTR2C//TAC1//TACR1//DRD2//SCN9A//SNCG//HTR2A |
| GO:0060341 | regulation of cellular localization | Biological process | 11 | 587 | 72 | 13692 | 3.56360022714367 | 0.00022921347798483 | 0.0104674154946406 | 3.63975984899809 | HRH3//HTR2C//DRD2//HTR2A//SNCG//TACR1//NNAT//LCP1//ADRA2A//TGFB3//TAC1 |
| GO:0071705 | nitrogen compound transport | Biological process | 6 | 166 | 72 | 13692 | 6.87349397590361 | 0.000234009989506978 | 0.010576286536068 | 3.63076560286697 | HRH3//HTR2C//DRD2//HTR2A//SNCG//SLC7A3 |
| GO:0045761 | regulation of adenylate cyclase activity | Biological process | 3 | 24 | 72 | 13692 | 23.7708333333333 | 0.000260686476956883 | 0.0115439344947371 | 3.58388149716052 | CALCA//DRD2//HRH3 |
| GO:0045980 | negative regulation of nucleotide metabolic process | Biological process | 3 | 24 | 72 | 13692 | 23.7708333333333 | 0.000260686476956883 | 0.0115439344947371 | 3.58388149716052 | DRD2//HRH3//ADRA2A |
| GO:0023052 | signaling | Biological process | 39 | 4621 | 72 | 13692 | 1.60495563730794 | 0.000282650870048815 | 0.0122937212048661 | 3.54874967336743 | TGFB3//DRD2//P2RX2//EPOR//ADRA2A//ARHGAP8//IGSF1//RGD1564053//ASB2//PLCXD3//NPPA//SSTR1//BHLHA15//HTR2A//NPW//OLR278//HTR2C//CALCA//HTR3A//TAC1//TACR1//TACR3//RAB20//DOK3//SNCG//HRH3//SCN9A//MC4R//SCX//ITGA8//NR2F2//SLC7A3//NNAT//SLIT2//TPD52L1//LRP2//EFNB1//PTPRO//HRK |
| GO:0051049 | regulation of transport | Biological process | 14 | 926 | 72 | 13692 | 2.87508999280058 | 0.000283226697466121 | 0.0122937212048661 | 3.54786581166116 | DRD2//TAC1//TACR1//HTR2A//CALCA//HRH3//HTR2C//SNCG//NNAT//LCP1//SCN11A//ADRA2A//TGFB3//TRPV2 |
| GO:0009165 | nucleotide biosynthetic process | Biological process | 7 | 247 | 72 | 13692 | 5.38933873144399 | 0.000310110729835195 | 0.0133286807803676 | 3.50848320690811 | NPPA//HTR2C//CALCA//DRD2//HRH3//ADRA2A//MC4R |
| GO:0007616 | long-term memory | Biological process | 3 | 26 | 72 | 13692 | 21.9423076923077 | 0.000332357508873456 | 0.0139295400244051 | 3.47839450486797 | DRD2//TAC1//TACR1 |
| GO:0044253 | positive regulation of multicellular organismal metabolic process | Biological process | 3 | 26 | 72 | 13692 | 21.9423076923077 | 0.000332357508873456 | 0.0139295400244051 | 3.47839450486797 | MC4R//TGFB3//SCX |
| GO:1901293 | nucleoside phosphate biosynthetic process | Biological process | 7 | 250 | 72 | 13692 | 5.32466666666667 | 0.000333622651131965 | 0.0139295400244051 | 3.47674447084106 | NPPA//HTR2C//CALCA//DRD2//HRH3//ADRA2A//MC4R |
| GO:0007611 | learning or memory | Biological process | 6 | 179 | 72 | 13692 | 6.37430167597765 | 0.000351033457287052 | 0.0145182139315701 | 3.45465148859778 | HRH3//HTR2A//ITGA8//DRD2//TAC1//TACR1 |
| GO:0032228 | regulation of synaptic transmission, GABAergic | Biological process | 3 | 27 | 72 | 13692 | 21.1296296296296 | 0.000372495122121204 | 0.0150234223745273 | 3.42887941002902 | TAC1//TACR1//DRD2 |
| GO:0045907 | positive regulation of vasoconstriction | Biological process | 3 | 27 | 72 | 13692 | 21.1296296296296 | 0.000372495122121204 | 0.0150234223745273 | 3.42887941002902 | TACR1//HTR2C//HTR2A |
| GO:0015844 | monoamine transport | Biological process | 4 | 65 | 72 | 13692 | 11.7025641025641 | 0.000373529434038202 | 0.0150234223745273 | 3.42767517018939 | DRD2//HTR2A//SNCG//HRH3 |
| GO:0051969 | regulation of transmission of nerve impulse | Biological process | 7 | 256 | 72 | 13692 | 5.19986979166667 | 0.000384945152239051 | 0.0153418140674182 | 3.41460114523646 | HTR2C//TAC1//TACR1//DRD2//SCN9A//SNCG//HTR2A |
| GO:0015850 | organic alcohol transport | Biological process | 4 | 66 | 72 | 13692 | 11.5252525252525 | 0.000396058532152513 | 0.0156425279725821 | 3.40224062641697 | DRD2//HTR2A//SNCG//HRH3 |
| GO:0034654 | nucleobase-containing compound biosynthetic process | Biological process | 7 | 258 | 72 | 13692 | 5.15956072351421 | 0.000403394019941495 | 0.0157899944948528 | 3.39427054421364 | NPPA//HTR2C//CALCA//DRD2//HRH3//ADRA2A//MC4R |
| GO:0042417 | dopamine metabolic process | Biological process | 3 | 28 | 72 | 13692 | 20.375 | 0.000415624860057075 | 0.0161135415917872 | 3.38129848358187 | TACR3//HTR2C//DRD2 |
| GO:0050896 | response to stimulus | Biological process | 49 | 6556 | 72 | 13692 | 1.42131889363433 | 0.000419010889932423 | 0.0161135415917872 | 3.37777468973991 | TGFB3//SST//DRD2//HSD11B2//DIO3//CLDN3//P2RX2//KRT1//MC4R//TACR1//SNCG//LCP1//ADRA2A//CALCA//PTPRO//TAC1//SLIT2//MX1//EPOR//ARHGAP8//IGSF1//RGD1564053//ASB2//PLCXD3//HTR2A//SCN9A//NPPA//SSTR1//BHLHA15//NPW//OLR278//HTR2C//HTR3A//TACR3//RAB20//DOK3//EFNB1//HRH3//ITGA8//CAMP//TRPV2//LRP2//NNMT//NNAT//SCX//NR2F2//SLC7A3//TPD52L1//HRK |
| GO:0018958 | phenol-containing compound metabolic process | Biological process | 4 | 68 | 72 | 13692 | 11.1862745098039 | 0.000444004907316268 | 0.0169262392493436 | 3.35261222986601 | TACR3//DIO3//DRD2//HTR2C |
| GO:0090407 | organophosphate biosynthetic process | Biological process | 7 | 267 | 72 | 13692 | 4.9856429463171 | 0.000495387967504499 | 0.0185772206018803 | 3.30505454621579 | NPPA//HTR2C//CALCA//DRD2//HRH3//ADRA2A//MC4R |
| GO:0050890 | cognition | Biological process | 6 | 191 | 72 | 13692 | 5.97382198952879 | 0.00049578804982208 | 0.0185772206018803 | 3.30470394541587 | TACR1//HRH3//HTR2A//ITGA8//DRD2//TAC1 |
| GO:0015872 | dopamine transport | Biological process | 3 | 30 | 72 | 13692 | 19.0166666666667 | 0.000511223089364011 | 0.0186435159277507 | 3.2913895395192 | DRD2//HTR2A//SNCG |
| GO:0048521 | negative regulation of behavior | Biological process | 3 | 30 | 72 | 13692 | 19.0166666666667 | 0.000511223089364011 | 0.0186435159277507 | 3.2913895395192 | SLIT2//DRD2//MC4R |
| GO:0055078 | sodium ion homeostasis | Biological process | 3 | 30 | 72 | 13692 | 19.0166666666667 | 0.000511223089364011 | 0.0186435159277507 | 3.2913895395192 | DRD2//TAC1//TACR1 |
| GO:0007154 | cell communication | Biological process | 39 | 4745 | 72 | 13692 | 1.56301369863014 | 0.000519567419308072 | 0.0186435159277507 | 3.28435809019784 | TGFB3//DRD2//P2RX2//EPOR//ADRA2A//ARHGAP8//IGSF1//RGD1564053//ASB2//PLCXD3//NPPA//SSTR1//BHLHA15//HTR2A//NPW//OLR278//HTR2C//CALCA//HTR3A//TAC1//TACR1//TACR3//RAB20//DOK3//SNCG//HRH3//SCN9A//MC4R//SCX//ITGA8//NR2F2//SLC7A3//NNAT//SLIT2//TPD52L1//LRP2//EFNB1//HRK//PTPRO |
| GO:0060402 | calcium ion transport into cytosol | Biological process | 4 | 71 | 72 | 13692 | 10.7136150234742 | 0.000523492530762781 | 0.0186435159277507 | 3.28108951049259 | CALCA//DRD2//HTR2C//HTR2A |
| GO:0007268 | synaptic transmission | Biological process | 9 | 446 | 72 | 13692 | 3.83744394618834 | 0.000525196818219998 | 0.0186435159277507 | 3.27967791364441 | DRD2//P2RX2//HTR2C//TAC1//TACR1//SCN9A//SNCG//HTR2A//HTR3A |
| GO:0006950 | response to stress | Biological process | 23 | 2176 | 72 | 13692 | 2.01003370098039 | 0.000527325724233825 | 0.0186435159277507 | 3.27792104220864 | DRD2//HSD11B2//TGFB3//DIO3//CLDN3//P2RX2//KRT1//TACR1//ADRA2A//TAC1//CALCA//SCN9A//SST//CAMP//TRPV2//TACR3//SSTR1//MX1//TPD52L1//SLIT2//HRK//SNCG//HTR2A |
| GO:0009719 | response to endogenous stimulus | Biological process | 14 | 987 | 72 | 13692 | 2.69739952718676 | 0.000540801104575896 | 0.0188702334726604 | 3.26696243002735 | DRD2//TAC1//TACR1//SST//NNMT//TACR3//NR2F2//TGFB3//NPPA//HSD11B2//MC4R//ADRA2A//SLIT2//SSTR1 |
| GO:0060401 | cytosolic calcium ion transport | Biological process | 4 | 72 | 72 | 13692 | 10.5648148148148 | 0.000552099804857674 | 0.0188702334726604 | 3.2579824063633 | CALCA//DRD2//HTR2C//HTR2A |
| GO:0009628 | response to abiotic stimulus | Biological process | 13 | 873 | 72 | 13692 | 2.83180603283696 | 0.000557023464941323 | 0.0188702334726604 | 3.25412650953024 | DRD2//HSD11B2//TGFB3//DIO3//CLDN3//P2RX2//SST//TRPV2//CALCA//TACR1//LRP2//TACR3//SNCG |
| GO:0014070 | response to organic cyclic compound | Biological process | 8 | 358 | 72 | 13692 | 4.24953445065177 | 0.000562291587053856 | 0.0188702334726604 | 3.25003841430923 | DRD2//TACR1//HTR2C//TACR3//SNCG//HTR3A//TAC1//HRH3 |
| GO:0031279 | regulation of cyclase activity | Biological process | 3 | 31 | 72 | 13692 | 18.4032258064516 | 0.00056386874655988 | 0.0188702334726604 | 3.24882197629786 | CALCA//DRD2//HRH3 |
| GO:0042312 | regulation of vasodilation | Biological process | 3 | 31 | 72 | 13692 | 18.4032258064516 | 0.00056386874655988 | 0.0188702334726604 | 3.24882197629786 | CALCA//ADRA2A//NPPA |
| GO:0044246 | regulation of multicellular organismal metabolic process | Biological process | 3 | 31 | 72 | 13692 | 18.4032258064516 | 0.00056386874655988 | 0.0188702334726604 | 3.24882197629786 | MC4R//TGFB3//SCX |
| GO:0010646 | regulation of cell communication | Biological process | 17 | 1369 | 72 | 13692 | 2.36145605064524 | 0.000591281430313434 | 0.0196377105340462 | 3.22820576016721 | CALCA//DRD2//ADRA2A//TGFB3//HRH3//HTR2C//HTR2A//SNCG//TACR1//ITGA8//NNAT//TAC1//SCN9A//TPD52L1//SLIT2//ARHGAP8//PTPRO |
| GO:0048513 | organ development | Biological process | 24 | 2341 | 72 | 13692 | 1.94959419051687 | 0.00059785740470371 | 0.0197068185129403 | 3.22340238747483 | GPR149//SLIT2//ITGA8//EFNB1//SCX//SMARCD3//TGFB3//EPOR//HRH3//NNAT//NPPA//NR2F2//SRPK3//P2RX2//SSTR1//MYO16//KIRREL3//DRD2//LRP2//HTR2C//NNMT//LCP1//PTPRO//CALCA |
| GO:0051339 | regulation of lyase activity | Biological process | 3 | 34 | 72 | 13692 | 16.7794117647059 | 0.000742221646294559 | 0.0241029607211507 | 3.12946638395491 | CALCA//DRD2//HRH3 |
| GO:0051932 | synaptic transmission, GABAergic | Biological process | 3 | 34 | 72 | 13692 | 16.7794117647059 | 0.000742221646294559 | 0.0241029607211507 | 3.12946638395491 | DRD2//TAC1//TACR1 |
| GO:0008283 | cell proliferation | Biological process | 16 | 1280 | 72 | 13692 | 2.37708333333333 | 0.000826231529788417 | 0.0266338163720031 | 3.08289823594882 | CALCA//NR2F2//DRD2//SMARCD3//HTR2A//CAMP//SCX//TGFB3//MYO16//SLIT2//EFNB1//TACR1//TAC1//HRH3//DIO3//LRP2 |
| GO:0046887 | positive regulation of hormone secretion | Biological process | 4 | 81 | 72 | 13692 | 9.39094650205761 | 0.000861662645737743 | 0.0275732046636078 | 3.06466273391019 | NNAT//DRD2//TAC1//TACR1 |
| GO:0009725 | response to hormone stimulus | Biological process | 12 | 801 | 72 | 13692 | 2.84893882646692 | 0.000884852356142499 | 0.0281100922415124 | 3.05312918835507 | TACR1//TACR3//NR2F2//TGFB3//NPPA//HSD11B2//MC4R//ADRA2A//SLIT2//SSTR1//SST//TAC1 |
| GO:0018130 | heterocycle biosynthetic process | Biological process | 7 | 296 | 72 | 13692 | 4.49718468468468 | 0.000910983872259636 | 0.0287320381006205 | 3.0404893115568 | NPPA//HTR2C//CALCA//DRD2//HRH3//ADRA2A//MC4R |
| GO:0051649 | establishment of localization in cell | Biological process | 16 | 1299 | 72 | 13692 | 2.34231460097511 | 0.000968485681946538 | 0.0303274373546687 | 3.0139067954896 | BHLHA15//AP1S2//TGFB3//CBLN4//HRH3//HTR2C//DRD2//HTR2A//SNCG//LRP2//TACR1//MC4R//NNAT//LCP1//ADRA2A//TAC1 |
| GO:0009408 | response to heat | Biological process | 4 | 85 | 72 | 13692 | 8.94901960784314 | 0.00103222486572511 | 0.0320941405059495 | 2.98622568322661 | CALCA//SST//TACR1//TRPV2 |
| GO:0007417 | central nervous system development | Biological process | 11 | 712 | 72 | 13692 | 2.93796816479401 | 0.00115589117331512 | 0.0351347972101551 | 2.93708305264001 | EPOR//HRH3//NNAT//SLIT2//ITGA8//SSTR1//MYO16//KIRREL3//DRD2//LRP2//NR2F2 |
| GO:0048731 | system development | Biological process | 28 | 3069 | 72 | 13692 | 1.73498425111328 | 0.00117195310363108 | 0.0351347972101551 | 2.93108976651053 | GPR149//SLIT2//ITGA8//EFNB1//NR2F2//KIRREL3//CALCA//SCX//DRD2//SMARCD3//TGFB3//PTPRO//NTNG1//EPOR//HRH3//NNAT//SNCG//NPPA//SRPK3//P2RX2//SSTR1//MYO16//LRP2//HTR2C//NNMT//LCP1//CAMP//TRPV2 |
| GO:0032502 | developmental process | Biological process | 33 | 3887 | 72 | 13692 | 1.61448417802933 | 0.00118685455077658 | 0.0351347972101551 | 2.92560250064573 | PTPRO//GPR149//SLIT2//ITGA8//TGFB3//SCX//EFNB1//NR2F2//KIRREL3//CALCA//DRD2//SMARCD3//EPOR//NTNG1//HRH3//NNAT//SNCG//NPPA//SRPK3//P2RX2//TACR3//LRP2//HTR2A//SCN9A//SSTR1//MYO16//HTR2C//NNMT//LCP1//RSPH9//CAMP//TRPV2//BHLHA15 |
| GO:0030432 | peristalsis | Biological process | 2 | 10 | 72 | 13692 | 38.0333333333333 | 0.00119413430299113 | 0.0351347972101551 | 2.9229468258299 | DRD2//P2RX2 |
| GO:0046541 | saliva secretion | Biological process | 2 | 10 | 72 | 13692 | 38.0333333333333 | 0.00119413430299113 | 0.0351347972101551 | 2.9229468258299 | TAC1//TACR1 |
| GO:0051350 | negative regulation of lyase activity | Biological process | 2 | 10 | 72 | 13692 | 38.0333333333333 | 0.00119413430299113 | 0.0351347972101551 | 2.9229468258299 | DRD2//HRH3 |
| GO:0051967 | negative regulation of synaptic transmission, glutamatergic | Biological process | 2 | 10 | 72 | 13692 | 38.0333333333333 | 0.00119413430299113 | 0.0351347972101551 | 2.9229468258299 | DRD2//HTR2A |
| GO:0060456 | positive regulation of digestive system process | Biological process | 2 | 10 | 72 | 13692 | 38.0333333333333 | 0.00119413430299113 | 0.0351347972101551 | 2.9229468258299 | TAC1//TACR1 |
| GO:0051179 | localization | Biological process | 30 | 3407 | 72 | 13692 | 1.67449368946287 | 0.00127948720312245 | 0.0366617144177319 | 2.89296405338062 | EFNB1//NR2F2//KIRREL3//DRD2//CALCA//SLIT2//PTPRO//TACR1//TAC1//AP1S2//HTR2A//P2RX2//KCNN3//SCN11A//BHLHA15//LRP2//TGFB3//CBLN4//HRH3//HTR2C//SNCG//SLC7A3//SLC6A5//MC4R//SST//ADRA2A//NNAT//LCP1//ITGA8//TRPV2 |
| GO:0046883 | regulation of hormone secretion | Biological process | 5 | 154 | 72 | 13692 | 6.17424242424242 | 0.00128722890217133 | 0.0366617144177319 | 2.89034421757854 | TACR1//NNAT//DRD2//ADRA2A//TAC1 |
| GO:0006584 | catecholamine metabolic process | Biological process | 3 | 41 | 72 | 13692 | 13.9146341463415 | 0.00128784307033091 | 0.0366617144177319 | 2.89013705455508 | TACR3//DRD2//HTR2C |
| GO:0009712 | catechol-containing compound metabolic process | Biological process | 3 | 41 | 72 | 13692 | 13.9146341463415 | 0.00128784307033091 | 0.0366617144177319 | 2.89013705455508 | TACR3//DRD2//HTR2C |
| GO:0034311 | diol metabolic process | Biological process | 3 | 41 | 72 | 13692 | 13.9146341463415 | 0.00128784307033091 | 0.0366617144177319 | 2.89013705455508 | TACR3//DRD2//HTR2C |
| GO:0016477 | cell migration | Biological process | 11 | 725 | 72 | 13692 | 2.88528735632184 | 0.00133764542508572 | 0.0378337906037148 | 2.87365899145328 | EFNB1//NR2F2//KIRREL3//SLIT2//CALCA//PTPRO//TACR1//TAC1//DRD2//SST//ADRA2A |
| GO:0031100 | organ regeneration | Biological process | 4 | 92 | 72 | 13692 | 8.26811594202899 | 0.00138565547525214 | 0.0389080255912115 | 2.85834473783135 | HTR2C//LRP2//NNMT//LCP1 |
| GO:0051047 | positive regulation of secretion | Biological process | 6 | 233 | 72 | 13692 | 4.89699570815451 | 0.00139456267793781 | 0.0389080255912115 | 2.8555619618061 | DRD2//TAC1//TACR1//HTR2C//NNAT//TGFB3 |
| GO:0048871 | multicellular organismal homeostasis | Biological process | 5 | 157 | 72 | 13692 | 6.05626326963907 | 0.001402250922311 | 0.0389080255912115 | 2.85317426566721 | DRD2//HTR2A//SCX//CALCA//MC4R |
| GO:0007565 | female pregnancy | Biological process | 5 | 158 | 72 | 13692 | 6.01793248945148 | 0.00144222689304963 | 0.0393622912267974 | 2.84096641045713 | CALCA//EPOR//NPPA//HSD11B2//TGFB3 |
| GO:0002031 | G-protein coupled receptor internalization | Biological process | 2 | 11 | 72 | 13692 | 34.5757575757576 | 0.00145453722142819 | 0.0393622912267974 | 2.83727516073247 | CALCA//DRD2 |
| GO:0032230 | positive regulation of synaptic transmission, GABAergic | Biological process | 2 | 11 | 72 | 13692 | 34.5757575757576 | 0.00145453722142819 | 0.0393622912267974 | 2.83727516073247 | TAC1//TACR1 |
| GO:0060259 | regulation of feeding behavior | Biological process | 2 | 11 | 72 | 13692 | 34.5757575757576 | 0.00145453722142819 | 0.0393622912267974 | 2.83727516073247 | MC4R//TACR3 |
| GO:0045776 | negative regulation of blood pressure | Biological process | 3 | 43 | 72 | 13692 | 13.2674418604651 | 0.00147976113214884 | 0.0395518428002948 | 2.82980838417407 | CALCA//DRD2//HRH3 |
| GO:0045777 | positive regulation of blood pressure | Biological process | 3 | 43 | 72 | 13692 | 13.2674418604651 | 0.00147976113214884 | 0.0395518428002948 | 2.82980838417407 | HSD11B2//TACR1//TACR3 |
| GO:0006816 | calcium ion transport | Biological process | 6 | 236 | 72 | 13692 | 4.83474576271186 | 0.00148860722218263 | 0.0395518428002948 | 2.82721987829964 | BHLHA15//CALCA//DRD2//HTR2C//HTR2A//TRPV2 |
| GO:0040012 | regulation of locomotion | Biological process | 8 | 418 | 72 | 13692 | 3.63955342902711 | 0.00152946527035427 | 0.0403926249712839 | 2.81546038011424 | TACR1//SLIT2//NR2F2//TAC1//SST//ADRA2A//DRD2//HTR2C |
| GO:0007588 | excretion | Biological process | 3 | 44 | 72 | 13692 | 12.9659090909091 | 0.00158208096401896 | 0.0415319936901744 | 2.80077129496797 | DRD2//TAC1//TACR1 |
| GO:0050878 | regulation of body fluid levels | Biological process | 6 | 241 | 72 | 13692 | 4.7344398340249 | 0.00165602867700921 | 0.0432144626190975 | 2.78093214692371 | HSD11B2//PTPRO//DRD2//TAC1//TACR1//NPPA |
| GO:0007399 | nervous system development | Biological process | 17 | 1503 | 72 | 13692 | 2.1509203814593 | 0.00168353645845805 | 0.0436723303779887 | 2.77377747414628 | NR2F2//KIRREL3//DRD2//SMARCD3//NTNG1//SLIT2//EFNB1//PTPRO//EPOR//HRH3//NNAT//ITGA8//SNCG//SSTR1//MYO16//LRP2//TRPV2 |
| GO:0042053 | regulation of dopamine metabolic process | Biological process | 2 | 12 | 72 | 13692 | 31.6944444444444 | 0.00173951482066301 | 0.0445921944573907 | 2.75957186671246 | HTR2C//TACR3 |
| GO:0050951 | sensory perception of temperature stimulus | Biological process | 2 | 12 | 72 | 13692 | 31.6944444444444 | 0.00173951482066301 | 0.0445921944573907 | 2.75957186671246 | ADRA2A//CALCA |
| GO:0007187 | G-protein coupled receptor signaling pathway, coupled to cyclic nucleotide second messenger | Biological process | 4 | 98 | 72 | 13692 | 7.76190476190476 | 0.00174951127889398 | 0.0445921944573907 | 2.75708325332647 | DRD2//CALCA//ADRA2A//HTR2C |
| GO:0006940 | regulation of smooth muscle contraction | Biological process | 3 | 46 | 72 | 13692 | 12.4021739130435 | 0.0017997776810801 | 0.045493568934032 | 2.74478113814292 | CALCA//TACR1//TACR3 |
| GO:0007612 | learning | Biological process | 4 | 99 | 72 | 13692 | 7.68350168350168 | 0.00181600697159115 | 0.045493568934032 | 2.74088248856947 | DRD2//TAC1//TACR1//HRH3 |
| GO:0008016 | regulation of heart contraction | Biological process | 4 | 99 | 72 | 13692 | 7.68350168350168 | 0.00181600697159115 | 0.045493568934032 | 2.74088248856947 | CALCA//DRD2//TAC1//TACR3 |
| GO:0006936 | muscle contraction | Biological process | 5 | 168 | 72 | 13692 | 5.65972222222222 | 0.00188969126979722 | 0.0470704916294944 | 2.72360914332706 | P2RX2//DRD2//CALCA//TACR1//TACR3 |
| GO:0031645 | negative regulation of neurological system process | Biological process | 3 | 47 | 72 | 13692 | 12.1382978723404 | 0.00191528466814382 | 0.0474384631928955 | 2.71776666784886 | DRD2//HTR2A//CALCA |
| GO:0006865 | amino acid transport | Biological process | 4 | 101 | 72 | 13692 | 7.53135313531353 | 0.00195422656240771 | 0.0481310631999742 | 2.70902508795626 | HRH3//HTR2C//SLC7A3//SLC6A5 |
| GO:0014051 | gamma-aminobutyric acid secretion | Biological process | 2 | 13 | 72 | 13692 | 29.2564102564103 | 0.00204880869032696 | 0.0496241839690243 | 2.68849859241104 | HRH3//HTR2C |
| GO:0015812 | gamma-aminobutyric acid transport | Biological process | 2 | 13 | 72 | 13692 | 29.2564102564103 | 0.00204880869032696 | 0.0496241839690243 | 2.68849859241104 | HRH3//HTR2C |
| GO:0042069 | regulation of catecholamine metabolic process | Biological process | 2 | 13 | 72 | 13692 | 29.2564102564103 | 0.00204880869032696 | 0.0496241839690243 | 2.68849859241104 | TACR3//HTR2C |
| GO:0019226 | transmission of nerve impulse | Biological process | 9 | 547 | 72 | 13692 | 3.12888482632541 | 0.00219430313569513 | 0.0528561810268541 | 2.65870337627556 | DRD2//TAC1//SNCG//HTR3A//P2RX2//HTR2C//SCN9A//TACR1//HTR2A |
| GO:0019229 | regulation of vasoconstriction | Biological process | 3 | 50 | 72 | 13692 | 11.41 | 0.00228919275817273 | 0.0539560271603723 | 2.64031763664561 | TACR1//HTR2C//HTR2A |
| GO:0035094 | response to nicotine | Biological process | 3 | 50 | 72 | 13692 | 11.41 | 0.00228919275817273 | 0.0539560271603723 | 2.64031763664561 | HTR2C//DRD2//TACR1 |
| GO:0043271 | negative regulation of ion transport | Biological process | 3 | 50 | 72 | 13692 | 11.41 | 0.00228919275817273 | 0.0539560271603723 | 2.64031763664561 | HTR2A//CALCA//DRD2 |
| GO:0051588 | regulation of neurotransmitter transport | Biological process | 3 | 50 | 72 | 13692 | 11.41 | 0.00228919275817273 | 0.0539560271603723 | 2.64031763664561 | HTR2C//SNCG//DRD2 |
| GO:0048870 | cell motility | Biological process | 11 | 779 | 72 | 13692 | 2.68528027385537 | 0.0023640702414469 | 0.0549652627876617 | 2.62633962380618 | EFNB1//NR2F2//KIRREL3//SLIT2//CALCA//PTPRO//TACR1//TAC1//DRD2//SST//ADRA2A |
| GO:0051674 | localization of cell | Biological process | 11 | 779 | 72 | 13692 | 2.68528027385537 | 0.0023640702414469 | 0.0549652627876617 | 2.62633962380618 | EFNB1//NR2F2//KIRREL3//SLIT2//CALCA//PTPRO//TACR1//TAC1//DRD2//SST//ADRA2A |
| GO:0001977 | renal system process involved in regulation of blood volume | Biological process | 2 | 14 | 72 | 13692 | 27.1666666666667 | 0.00238216239271344 | 0.0549652627876617 | 2.62302863586105 | HSD11B2//PTPRO |
| GO:0051955 | regulation of amino acid transport | Biological process | 2 | 14 | 72 | 13692 | 27.1666666666667 | 0.00238216239271344 | 0.0549652627876617 | 2.62302863586105 | HRH3//HTR2C |
| GO:0048856 | anatomical structure development | Biological process | 30 | 3546 | 72 | 13692 | 1.60885504794134 | 0.00250556326781367 | 0.0573159897070855 | 2.60109462644158 | PTPRO//GPR149//SLIT2//ITGA8//TGFB3//SCX//EFNB1//NR2F2//KIRREL3//CALCA//DRD2//SMARCD3//EPOR//NTNG1//HRH3//NNAT//SNCG//NPPA//SRPK3//P2RX2//SSTR1//MYO16//LRP2//HTR2C//NNMT//LCP1//RSPH9//CAMP//TRPV2//BHLHA15 |
| GO:0035637 | multicellular organismal signaling | Biological process | 9 | 558 | 72 | 13692 | 3.06720430107527 | 0.00251018933023732 | 0.0573159897070855 | 2.60029352075946 | DRD2//TAC1//SNCG//HTR3A//P2RX2//HTR2C//SCN9A//TACR1//HTR2A |
| GO:0040011 | locomotion | Biological process | 12 | 906 | 72 | 13692 | 2.51876379690949 | 0.00252581473412428 | 0.0573739471212479 | 2.59759850765965 | EFNB1//NR2F2//KIRREL3//SLIT2//CALCA//PTPRO//TACR1//TAC1//DRD2//SST//ADRA2A//HTR2C |
| GO:0065007 | biological regulation | Biological process | 54 | 8017 | 72 | 13692 | 1.28090308095297 | 0.00263714548232347 | 0.0595940504871448 | 2.57886591100008 | NR2F2//TGFB3//DRD2//HTR2A//ADRA2A//KRT1//SCX//SLIT2//CAMP//CALCA//HSD11B2//MC4R//SMARCD3//TAC1//TACR1//P2RX2//PTPRO//EPOR//ARHGAP8//IGSF1//RGD1564053//ASB2//PLCXD3//MT4//TPD52L1//NPPA//SSTR1//BHLHA15//NPW//OLR278//HTR2C//HRH3//TRHR//HTR3A//TACR3//RAB20//DOK3//MYO16//PLAGL1//LRP2//SNCG//SCN9A//SST//ITGA8//SLC7A3//NNAT//HRK//LCP1//SCN11A//DIO3//EFNB1//CHRNE//TRPV2//GPR149 |
| GO:0070838 | divalent metal ion transport | Biological process | 6 | 266 | 72 | 13692 | 4.28947368421053 | 0.00271796678168599 | 0.0611054685687763 | 2.56575585541023 | BHLHA15//CALCA//DRD2//HTR2C//HTR2A//TRPV2 |
| GO:0042756 | drinking behavior | Biological process | 2 | 15 | 72 | 13692 | 25.3555555555556 | 0.00273932144964402 | 0.0612713532410173 | 2.5623570018405 | TACR1//HRH3 |
| GO:0042127 | regulation of cell proliferation | Biological process | 13 | 1042 | 72 | 13692 | 2.37252079334613 | 0.00278825664162874 | 0.0620493254664995 | 2.55466725463661 | NR2F2//DRD2//SMARCD3//HTR2A//CAMP//SCX//TGFB3//MYO16//SLIT2//EFNB1//TACR1//TAC1//HRH3 |
| GO:0007270 | neuron-neuron synaptic transmission | Biological process | 4 | 112 | 72 | 13692 | 6.79166666666667 | 0.002846769915356 | 0.0629851847292925 | 2.54564763244182 | DRD2//TAC1//TACR1//HTR2A |
| GO:0046942 | carboxylic acid transport | Biological process | 5 | 185 | 72 | 13692 | 5.13963963963964 | 0.00287264019814272 | 0.0629851847292925 | 2.54171876654083 | HRH3//HTR2C//SLC7A3//SLC6A5//DRD2 |
| GO:0072511 | divalent inorganic cation transport | Biological process | 6 | 269 | 72 | 13692 | 4.24163568773234 | 0.00287341171210276 | 0.0629851847292925 | 2.54160214237425 | BHLHA15//CALCA//DRD2//HTR2C//HTR2A//TRPV2 |
| GO:0042311 | vasodilation | Biological process | 3 | 55 | 72 | 13692 | 10.3727272727273 | 0.00300721213052781 | 0.0643420264181753 | 2.5218359354285 | CALCA//ADRA2A//NPPA |
| GO:0051209 | release of sequestered calcium ion into cytosol | Biological process | 3 | 55 | 72 | 13692 | 10.3727272727273 | 0.00300721213052781 | 0.0643420264181753 | 2.5218359354285 | DRD2//HTR2C//HTR2A |
| GO:0051282 | regulation of sequestering of calcium ion | Biological process | 3 | 55 | 72 | 13692 | 10.3727272727273 | 0.00300721213052781 | 0.0643420264181753 | 2.5218359354285 | DRD2//HTR2C//HTR2A |
| GO:0051283 | negative regulation of sequestering of calcium ion | Biological process | 3 | 55 | 72 | 13692 | 10.3727272727273 | 0.00300721213052781 | 0.0643420264181753 | 2.5218359354285 | DRD2//HTR2C//HTR2A |
| GO:0015849 | organic acid transport | Biological process | 5 | 187 | 72 | 13692 | 5.08467023172906 | 0.0030086942097915 | 0.0643420264181753 | 2.52162194976364 | HRH3//HTR2C//SLC7A3//SLC6A5//DRD2 |
| GO:0006812 | cation transport | Biological process | 9 | 575 | 72 | 13692 | 2.97652173913043 | 0.00306878307762625 | 0.0653084709335606 | 2.51303380935882 | DRD2//HTR2A//KCNN3//SCN11A//BHLHA15//CALCA//HTR2C//TRPV2//P2RX2 |
| GO:0007625 | grooming behavior | Biological process | 2 | 16 | 72 | 13692 | 23.7708333333333 | 0.00312003332941514 | 0.0658748088973424 | 2.50584076665343 | MC4R//DRD2 |
| GO:0007275 | multicellular organismal development | Biological process | 29 | 3429 | 72 | 13692 | 1.60829201905317 | 0.00312544713746515 | 0.0658748088973424 | 2.50508784217834 | GPR149//SLIT2//ITGA8//TGFB3//SCX//EFNB1//NR2F2//KIRREL3//CALCA//DRD2//SMARCD3//EPOR//PTPRO//NTNG1//HRH3//NNAT//SNCG//NPPA//SRPK3//P2RX2//SCN9A//SSTR1//MYO16//LRP2//HTR2C//NNMT//LCP1//CAMP//TRPV2 |
| GO:0051208 | sequestering of calcium ion | Biological process | 3 | 56 | 72 | 13692 | 10.1875 | 0.00316554574908571 | 0.0662312669497173 | 2.49955140558844 | DRD2//HTR2C//HTR2A |
| GO:0048518 | positive regulation of biological process | Biological process | 27 | 3107 | 72 | 13692 | 1.6525587383328 | 0.00317696780034543 | 0.0662312669497173 | 2.49798718687357 | TGFB3//ADRA2A//KRT1//CAMP//MC4R//DRD2//SMARCD3//TAC1//TACR1//TPD52L1//CALCA//HTR2A//SCX//TACR3//HTR2C//NR2F2//ITGA8//NNAT//HRK//DIO3//EFNB1//SLIT2//TRPV2//PLAGL1//BHLHA15//HRH3//ARHGAP8 |
| GO:0006928 | cellular component movement | Biological process | 12 | 932 | 72 | 13692 | 2.44849785407725 | 0.00318768187189561 | 0.0662312669497173 | 2.49652502735567 | EFNB1//NR2F2//KIRREL3//SLIT2//CALCA//PTPRO//TACR1//RSPH9//TAC1//DRD2//SST//ADRA2A |
| GO:0023051 | regulation of signaling | Biological process | 18 | 1744 | 72 | 13692 | 1.96272935779817 | 0.00333625643448578 | 0.0686941806152829 | 2.47674057559965 | TGFB3//CALCA//DRD2//ADRA2A//HRH3//HTR2C//HTR2A//SNCG//TACR1//ITGA8//SLC7A3//NNAT//TAC1//ARHGAP8//SCN9A//TPD52L1//SLIT2//PTPRO |
| GO:0030001 | metal ion transport | Biological process | 8 | 474 | 72 | 13692 | 3.20956399437412 | 0.00333755941401808 | 0.0686941806152829 | 2.47657099440624 | DRD2//HTR2A//KCNN3//SCN11A//BHLHA15//CALCA//HTR2C//TRPV2 |
| GO:0048545 | response to steroid hormone stimulus | Biological process | 8 | 477 | 72 | 13692 | 3.18937805730259 | 0.0034680238715184 | 0.0710458722090498 | 2.4599179217976 | TACR1//TACR3//NR2F2//TGFB3//HSD11B2//SLIT2//SSTR1//SST |
| GO:0030334 | regulation of cell migration | Biological process | 7 | 375 | 72 | 13692 | 3.54977777777778 | 0.00349128704470111 | 0.0711897786231147 | 2.45701444313514 | TACR1//SLIT2//NR2F2//TAC1//ADRA2A//DRD2//SST |
| GO:0014821 | phasic smooth muscle contraction | Biological process | 2 | 17 | 72 | 13692 | 22.3725490196078 | 0.00352404743382508 | 0.0711955020732219 | 2.45295825459885 | DRD2//P2RX2 |
| GO:0051482 | elevation of cytosolic calcium ion concentration involved in phospholipase C-activating G-protein coupled signaling pathway | Biological process | 2 | 17 | 72 | 13692 | 22.3725490196078 | 0.00352404743382508 | 0.0711955020732219 | 2.45295825459885 | CALCA//DRD2 |
| GO:0003015 | heart process | Biological process | 4 | 121 | 72 | 13692 | 6.28650137741047 | 0.0037585952137461 | 0.0752405544158123 | 2.42497444361377 | CALCA//DRD2//TAC1//TACR3 |
| GO:0060047 | heart contraction | Biological process | 4 | 121 | 72 | 13692 | 6.28650137741047 | 0.0037585952137461 | 0.0752405544158123 | 2.42497444361377 | CALCA//DRD2//TAC1//TACR3 |
| GO:0045860 | positive regulation of protein kinase activity | Biological process | 6 | 287 | 72 | 13692 | 3.97560975609756 | 0.00395047429262978 | 0.0776757333357392 | 2.40335075999219 | TGFB3//ADRA2A//CALCA//DRD2//HTR2A//TPD52L1 |
| GO:0007271 | synaptic transmission, cholinergic | Biological process | 2 | 18 | 72 | 13692 | 21.1296296296296 | 0.00395111508528053 | 0.0776757333357392 | 2.40328032031239 | TAC1//TACR1 |
| GO:0010714 | positive regulation of collagen metabolic process | Biological process | 2 | 18 | 72 | 13692 | 21.1296296296296 | 0.00395111508528053 | 0.0776757333357392 | 2.40328032031239 | TGFB3//SCX |
| GO:0032967 | positive regulation of collagen biosynthetic process | Biological process | 2 | 18 | 72 | 13692 | 21.1296296296296 | 0.00395111508528053 | 0.0776757333357392 | 2.40328032031239 | TGFB3//SCX |
| GO:0003012 | muscle system process | Biological process | 5 | 200 | 72 | 13692 | 4.75416666666667 | 0.00400928372997469 | 0.0784674101437904 | 2.39693320840347 | P2RX2//DRD2//CALCA//TACR1//TACR3 |
| GO:0008306 | associative learning | Biological process | 3 | 61 | 72 | 13692 | 9.35245901639344 | 0.00403370618787156 | 0.0785945241227952 | 2.39429573865502 | DRD2//TAC1//TACR1 |
| GO:0001666 | response to hypoxia | Biological process | 6 | 291 | 72 | 13692 | 3.92096219931271 | 0.00422581190097273 | 0.0819732715657719 | 2.37408983813739 | TGFB3//DRD2//HSD11B2//DIO3//CLDN3//P2RX2 |
| GO:2000145 | regulation of cell motility | Biological process | 7 | 389 | 72 | 13692 | 3.42202227934876 | 0.00426695150776247 | 0.0824066758151131 | 2.36988229269983 | TACR1//SLIT2//NR2F2//TAC1//SST//ADRA2A//DRD2 |
| GO:1900542 | regulation of purine nucleotide metabolic process | Biological process | 6 | 293 | 72 | 13692 | 3.89419795221843 | 0.00436870214878907 | 0.0827631807035474 | 2.3596475637928 | CALCA//DRD2//HRH3//ADRA2A//MC4R//ARHGAP8 |
| GO:0002029 | desensitization of G-protein coupled receptor protein signaling pathway | Biological process | 2 | 19 | 72 | 13692 | 20.0175438596491 | 0.00440098951398249 | 0.0827631807035474 | 2.35644966620755 | CALCA//DRD2 |
| GO:0010634 | positive regulation of epithelial cell migration | Biological process | 2 | 19 | 72 | 13692 | 20.0175438596491 | 0.00440098951398249 | 0.0827631807035474 | 2.35644966620755 | TAC1//TACR1 |
| GO:0022401 | negative adaptation of signaling pathway | Biological process | 2 | 19 | 72 | 13692 | 20.0175438596491 | 0.00440098951398249 | 0.0827631807035474 | 2.35644966620755 | CALCA//DRD2 |
| GO:0042310 | vasoconstriction | Biological process | 3 | 63 | 72 | 13692 | 9.05555555555555 | 0.00441756028390285 | 0.0827631807035474 | 2.35481751521391 | TACR1//HTR2C//HTR2A |
| GO:0044236 | multicellular organismal metabolic process | Biological process | 3 | 63 | 72 | 13692 | 9.05555555555555 | 0.00441756028390285 | 0.0827631807035474 | 2.35481751521391 | MC4R//TGFB3//SCX |
| GO:0051238 | sequestering of metal ion | Biological process | 3 | 63 | 72 | 13692 | 9.05555555555555 | 0.00441756028390285 | 0.0827631807035474 | 2.35481751521391 | DRD2//HTR2C//HTR2A |
| GO:0036293 | response to decreased oxygen levels | Biological process | 6 | 294 | 72 | 13692 | 3.88095238095238 | 0.00444147564892822 | 0.0828571457229843 | 2.35247271463463 | DRD2//HSD11B2//TGFB3//DIO3//CLDN3//P2RX2 |
| GO:0051641 | cellular localization | Biological process | 16 | 1510 | 72 | 13692 | 2.01501103752759 | 0.00455291870306449 | 0.0845762525179437 | 2.34171010438667 | BHLHA15//AP1S2//TGFB3//CBLN4//HRH3//HTR2C//DRD2//HTR2A//SNCG//LRP2//TACR1//MC4R//NNAT//LCP1//ADRA2A//TAC1 |
| GO:0030073 | insulin secretion | Biological process | 4 | 129 | 72 | 13692 | 5.89664082687339 | 0.00472023595448893 | 0.0873144068543438 | 2.326036291372 | NNAT//DRD2//ADRA2A//MC4R |
| GO:0044271 | cellular nitrogen compound biosynthetic process | Biological process | 7 | 397 | 72 | 13692 | 3.35306465155332 | 0.00476537639976316 | 0.087779034187234 | 2.32190279032805 | NPPA//HTR2C//CALCA//DRD2//HRH3//ADRA2A//MC4R |
| GO:0006140 | regulation of nucleotide metabolic process | Biological process | 6 | 299 | 72 | 13692 | 3.81605351170569 | 0.00481889048743046 | 0.0883933719535361 | 2.31705294322464 | CALCA//DRD2//HRH3//ADRA2A//MC4R//ARHGAP8 |
| GO:0070371 | ERK1 and ERK2 cascade | Biological process | 4 | 130 | 72 | 13692 | 5.85128205128205 | 0.00485101366442728 | 0.0886118496035383 | 2.31416750204328 | DRD2//ARHGAP8//HTR2C//HTR2A |
| GO:0002052 | positive regulation of neuroblast proliferation | Biological process | 2 | 20 | 72 | 13692 | 19.0166666666667 | 0.00487342584519087 | 0.088651862677663 | 2.31216563784175 | DRD2//SMARCD3 |
| GO:0008284 | positive regulation of cell proliferation | Biological process | 9 | 618 | 72 | 13692 | 2.76941747572816 | 0.00492786970246032 | 0.0892718213867192 | 2.30734078384903 | DRD2//SMARCD3//EFNB1//TAC1//TACR1//HRH3//HTR2A//CAMP//SCX |
| GO:0051234 | establishment of localization | Biological process | 24 | 2726 | 72 | 13692 | 1.67424798239178 | 0.00507242526708223 | 0.0915123965880185 | 2.29478434278311 | DRD2//CALCA//TAC1//TACR1//AP1S2//HTR2A//P2RX2//KCNN3//SCN11A//BHLHA15//LRP2//TGFB3//CBLN4//HRH3//HTR2C//SNCG//SLC7A3//SLC6A5//MC4R//NNAT//LCP1//ITGA8//ADRA2A//TRPV2 |
| GO:0046849 | bone remodeling | Biological process | 3 | 67 | 72 | 13692 | 8.51492537313433 | 0.00524995017648254 | 0.0939419656069366 | 2.2798448181524 | TGFB3//CALCA//MC4R |
| GO:0051017 | actin filament bundle assembly | Biological process | 3 | 67 | 72 | 13692 | 8.51492537313433 | 0.00524995017648254 | 0.0939419656069366 | 2.2798448181524 | TAC1//TACR1//LCP1 |
| GO:0023058 | adaptation of signaling pathway | Biological process | 2 | 21 | 72 | 13692 | 18.1111111111111 | 0.00536818108656753 | 0.0952797809049071 | 2.27017284239746 | CALCA//DRD2 |
| GO:0043114 | regulation of vascular permeability | Biological process | 2 | 21 | 72 | 13692 | 18.1111111111111 | 0.00536818108656753 | 0.0952797809049071 | 2.27017284239746 | SLIT2//TACR1 |
| GO:0007165 | signal transduction | Biological process | 33 | 4238 | 72 | 13692 | 1.48076923076923 | 0.00553065173276714 | 0.0975673206120124 | 2.25722368835772 | TGFB3//P2RX2//DRD2//ASB2//PLCXD3//ADRA2A//NPPA//SSTR1//BHLHA15//HTR2A//NPW//OLR278//HTR2C//CALCA//HTR3A//TAC1//TACR1//TACR3//RAB20//DOK3//SCX//ITGA8//NR2F2//SLC7A3//SLIT2//EPOR//TPD52L1//EFNB1//ARHGAP8//PTPRO//HRK//IGSF1//RGD1564053 |
| GO:0009266 | response to temperature stimulus | Biological process | 4 | 135 | 72 | 13692 | 5.63456790123457 | 0.0055415745511841 | 0.0975673206120124 | 2.25636681979624 | CALCA//SST//TACR1//TRPV2 |
| GO:0033674 | positive regulation of kinase activity | Biological process | 6 | 309 | 72 | 13692 | 3.69255663430421 | 0.00564409805740101 | 0.0989749035345841 | 2.24840544966286 | TGFB3//ADRA2A//CALCA//DRD2//HTR2A//TPD52L1 |
| GO:0050886 | endocrine process | Biological process | 3 | 69 | 72 | 13692 | 8.26811594202898 | 0.00569914006163539 | 0.0995419523115918 | 2.24419066970665 | HSD11B2//TACR1//TAC1 |
| GO:0051270 | regulation of cellular component movement | Biological process | 7 | 412 | 72 | 13692 | 3.23098705501618 | 0.00581739476713471 | 0.101176085814813 | 2.23527146407322 | TACR1//SLIT2//NR2F2//TAC1//SST//ADRA2A//DRD2 |
| GO:0003071 | renal system process involved in regulation of systemic arterial blood pressure | Biological process | 2 | 22 | 72 | 13692 | 17.2878787878788 | 0.00588501411559701 | 0.101176085814813 | 2.23025249113515 | HSD11B2//PTPRO |
| GO:0032965 | regulation of collagen biosynthetic process | Biological process | 2 | 22 | 72 | 13692 | 17.2878787878788 | 0.00588501411559701 | 0.101176085814813 | 2.23025249113515 | TGFB3//SCX |
| GO:0051496 | positive regulation of stress fiber assembly | Biological process | 2 | 22 | 72 | 13692 | 17.2878787878788 | 0.00588501411559701 | 0.101176085814813 | 2.23025249113515 | TAC1//TACR1 |
| GO:0051241 | negative regulation of multicellular organismal process | Biological process | 6 | 312 | 72 | 13692 | 3.65705128205128 | 0.00591073202159037 | 0.101221285869735 | 2.22835873007469 | TAC1//TGFB3//CALCA//DRD2//HTR2A//GPR149 |
| GO:0040013 | negative regulation of locomotion | Biological process | 4 | 138 | 72 | 13692 | 5.51207729468599 | 0.005985963590254 | 0.102110756341142 | 2.22286592909286 | SLIT2//NR2F2//DRD2//HTR2C |
| GO:0043406 | positive regulation of MAP kinase activity | Biological process | 4 | 139 | 72 | 13692 | 5.47242206235012 | 0.00613921898438104 | 0.104319131889638 | 2.21188687516961 | TGFB3//ADRA2A//HTR2A//TPD52L1 |
| GO:0070482 | response to oxygen levels | Biological process | 6 | 317 | 72 | 13692 | 3.5993690851735 | 0.00637547592843281 | 0.107077710891635 | 2.19548738965838 | DRD2//HSD11B2//TGFB3//DIO3//CLDN3//P2RX2 |
| GO:0006972 | hyperosmotic response | Biological process | 2 | 23 | 72 | 13692 | 16.536231884058 | 0.00642368566708486 | 0.107077710891635 | 2.19221571872416 | TACR3//SST |
| GO:0010712 | regulation of collagen metabolic process | Biological process | 2 | 23 | 72 | 13692 | 16.536231884058 | 0.00642368566708486 | 0.107077710891635 | 2.19221571872416 | TGFB3//SCX |
| GO:0045909 | positive regulation of vasodilation | Biological process | 2 | 23 | 72 | 13692 | 16.536231884058 | 0.00642368566708486 | 0.107077710891635 | 2.19221571872416 | CALCA//ADRA2A |
| GO:0051954 | positive regulation of amine transport | Biological process | 2 | 23 | 72 | 13692 | 16.536231884058 | 0.00642368566708486 | 0.107077710891635 | 2.19221571872416 | HTR2C//DRD2 |
| GO:0050795 | regulation of behavior | Biological process | 4 | 142 | 72 | 13692 | 5.35680751173709 | 0.00661462905687498 | 0.109842931005075 | 2.17949450568198 | SLIT2//DRD2//TACR3//MC4R |
| GO:0055067 | monovalent inorganic cation homeostasis | Biological process | 3 | 73 | 72 | 13692 | 7.81506849315068 | 0.00666501791283363 | 0.110262032188161 | 2.17619867904317 | DRD2//TAC1//TACR1 |
| GO:0007409 | axonogenesis | Biological process | 6 | 321 | 72 | 13692 | 3.55451713395639 | 0.00676606484809381 | 0.111512888323471 | 2.16966384411317 | EFNB1//PTPRO//SLIT2//TRPV2//DRD2//NTNG1 |
| GO:0051347 | positive regulation of transferase activity | Biological process | 6 | 323 | 72 | 13692 | 3.53250773993808 | 0.00696776884839255 | 0.11382034675871 | 2.15690626522381 | TGFB3//ADRA2A//CALCA//DRD2//HTR2A//TPD52L1 |
| GO:0010596 | negative regulation of endothelial cell migration | Biological process | 2 | 24 | 72 | 13692 | 15.8472222222222 | 0.00698395832073287 | 0.11382034675871 | 2.15589836110228 | NR2F2//SLIT2 |
| GO:0033238 | regulation of cellular amine metabolic process | Biological process | 2 | 24 | 72 | 13692 | 15.8472222222222 | 0.00698395832073287 | 0.11382034675871 | 2.15589836110228 | TACR3//HTR2C |
| GO:0001508 | regulation of action potential | Biological process | 4 | 145 | 72 | 13692 | 5.24597701149425 | 0.00711389715091224 | 0.115508611517034 | 2.14789241791242 | SCN9A//P2RX2//TAC1//TACR1 |
| GO:0001932 | regulation of protein phosphorylation | Biological process | 9 | 655 | 72 | 13692 | 2.61297709923664 | 0.00714798100332403 | 0.115633759109124 | 2.14581661035813 | TGFB3//SLIT2//CAMP//ADRA2A//CALCA//DRD2//HTR2A//TPD52L1//NR2F2 |
| GO:0042221 | response to chemical stimulus | Biological process | 29 | 3628 | 72 | 13692 | 1.52007533994855 | 0.00740667958580634 | 0.11937824744182 | 2.13037644259373 | SST//DRD2//CALCA//PTPRO//SLIT2//P2RX2//EFNB1//HSD11B2//HTR2C//LRP2//HTR2A//NNMT//SCN9A//TAC1//TACR1//NNAT//HRH3//TACR3//NR2F2//TGFB3//NPPA//MC4R//ADRA2A//EPOR//SSTR1//SNCG//HTR3A//OLR278//CAMP |
| GO:0010632 | regulation of epithelial cell migration | Biological process | 2 | 25 | 72 | 13692 | 15.2133333333333 | 0.00756559648879069 | 0.120609363661303 | 2.12115682552871 | TAC1//TACR1 |
| GO:0045124 | regulation of bone resorption | Biological process | 2 | 25 | 72 | 13692 | 15.2133333333333 | 0.00756559648879069 | 0.120609363661303 | 2.12115682552871 | CALCA//MC4R |
| GO:0045987 | positive regulation of smooth muscle contraction | Biological process | 2 | 25 | 72 | 13692 | 15.2133333333333 | 0.00756559648879069 | 0.120609363661303 | 2.12115682552871 | TACR1//TACR3 |
| GO:0048522 | positive regulation of cellular process | Biological process | 24 | 2813 | 72 | 13692 | 1.62246711695699 | 0.00761096450881316 | 0.120893001473322 | 2.11856030324091 | TGFB3//CAMP//DRD2//SMARCD3//TACR1//ADRA2A//TPD52L1//CALCA//HTR2A//SCX//TAC1//HTR2C//NR2F2//ITGA8//MC4R//NNAT//EFNB1//HRK//SLIT2//TRPV2//PLAGL1//BHLHA15//HRH3//ARHGAP8 |
| GO:0043627 | response to estrogen stimulus | Biological process | 5 | 237 | 72 | 13692 | 4.01195499296765 | 0.00813538422513654 | 0.127712782569733 | 2.08962193099804 | TACR1//TACR3//NR2F2//SSTR1//TGFB3 |
| GO:0032233 | positive regulation of actin filament bundle assembly | Biological process | 2 | 26 | 72 | 13692 | 14.6282051282051 | 0.0081683664037838 | 0.127712782569733 | 2.0878647895827 | TAC1//TACR1 |
| GO:0043266 | regulation of potassium ion transport | Biological process | 2 | 26 | 72 | 13692 | 14.6282051282051 | 0.0081683664037838 | 0.127712782569733 | 2.0878647895827 | HTR2A//DRD2 |
| GO:0044058 | regulation of digestive system process | Biological process | 2 | 26 | 72 | 13692 | 14.6282051282051 | 0.0081683664037838 | 0.127712782569733 | 2.0878647895827 | TAC1//TACR1 |
| GO:0032355 | response to estradiol stimulus | Biological process | 4 | 151 | 72 | 13692 | 5.03752759381898 | 0.0081859698681786 | 0.127712782569733 | 2.08692985828687 | SSTR1//TACR1//TACR3//NR2F2 |
| GO:0050673 | epithelial cell proliferation | Biological process | 5 | 239 | 72 | 13692 | 3.97838214783822 | 0.00841954832304314 | 0.13089113421355 | 2.07471120613355 | CALCA//NR2F2//TGFB3//TACR1//HRH3 |
| GO:0006163 | purine nucleotide metabolic process | Biological process | 8 | 555 | 72 | 13692 | 2.74114114114114 | 0.00848792671273769 | 0.131022525008231 | 2.07119837891716 | NPPA//HTR2C//CALCA//DRD2//HRH3//ADRA2A//MC4R//ARHGAP8 |
| GO:0045859 | regulation of protein kinase activity | Biological process | 7 | 443 | 72 | 13692 | 3.00489089541008 | 0.00853420008762782 | 0.131022525008231 | 2.06883717915494 | TGFB3//ADRA2A//CALCA//DRD2//HTR2A//TPD52L1//NR2F2 |
| GO:0051704 | multi-organism process | Biological process | 10 | 797 | 72 | 13692 | 2.38603094939356 | 0.00854106675164046 | 0.131022525008231 | 2.06848788394584 | TACR1//SNCG//MX1//NPPA//HSD11B2//TGFB3//CALCA//CAMP//TAC1//EPOR |
| GO:0006810 | transport | Biological process | 23 | 2679 | 72 | 13692 | 1.63263655592883 | 0.00855003270090524 | 0.131022525008231 | 2.06803222424311 | DRD2//CALCA//TAC1//TACR1//AP1S2//HTR2A//P2RX2//KCNN3//SCN11A//BHLHA15//LRP2//TGFB3//CBLN4//HRH3//HTR2C//SNCG//SLC7A3//SLC6A5//MC4R//NNAT//LCP1//ADRA2A//TRPV2 |
| GO:0040014 | regulation of multicellular organism growth | Biological process | 3 | 80 | 72 | 13692 | 7.13125 | 0.00857743263625965 | 0.131022525008231 | 2.06664268403432 | MC4R//DRD2//DIO3 |
| GO:0030072 | peptide hormone secretion | Biological process | 4 | 154 | 72 | 13692 | 4.93939393939394 | 0.00875972352298331 | 0.133342458072079 | 2.05750960094808 | MC4R//NNAT//DRD2//ADRA2A |
| GO:0006182 | cGMP biosynthetic process | Biological process | 2 | 27 | 72 | 13692 | 14.0864197530864 | 0.00879203610631681 | 0.133371232837692 | 2.05591053706069 | NPPA//HTR2C |
| GO:0030278 | regulation of ossification | Biological process | 4 | 157 | 72 | 13692 | 4.84501061571125 | 0.00935923274506677 | 0.141110969949107 | 2.02875975256975 | CALCA//TGFB3//TAC1//TACR1 |
| GO:0001934 | positive regulation of protein phosphorylation | Biological process | 7 | 451 | 72 | 13692 | 2.95158906134516 | 0.00936662688302695 | 0.141110969949107 | 2.0284167794251 | TGFB3//ADRA2A//CALCA//DRD2//HTR2A//TPD52L1//CAMP |
| GO:0051926 | negative regulation of calcium ion transport | Biological process | 2 | 28 | 72 | 13692 | 13.5833333333333 | 0.00943637543295217 | 0.141674896911172 | 2.02519478872583 | CALCA//DRD2 |
| GO:0050900 | leukocyte migration | Biological process | 4 | 159 | 72 | 13692 | 4.78406708595388 | 0.0097734326439588 | 0.146234568979916 | 2.00995287573855 | CALCA//PTPRO//TACR1//SLIT2 |
| GO:0002790 | peptide secretion | Biological process | 4 | 160 | 72 | 13692 | 4.75416666666667 | 0.00998493872829278 | 0.148891059132094 | 2.00065459579538 | MC4R//NNAT//DRD2//ADRA2A |
| GO:0040008 | regulation of growth | Biological process | 7 | 457 | 72 | 13692 | 2.91283734500365 | 0.0100291270782134 | 0.149043027494534 | 1.99873686574458 | MC4R//NPPA//SLIT2//CAMP//DRD2//DIO3//TRPV2 |
| GO:0032964 | collagen biosynthetic process | Biological process | 2 | 29 | 72 | 13692 | 13.1149425287356 | 0.0101011560041636 | 0.149606310548153 | 1.99562892151434 | TGFB3//SCX |
| GO:0010720 | positive regulation of cell development | Biological process | 4 | 162 | 72 | 13692 | 4.69547325102881 | 0.0104168414794308 | 0.153191210581377 | 1.98226394475972 | DRD2//SMARCD3//TGFB3//TRPV2 |
| GO:0043269 | regulation of ion transport | Biological process | 5 | 252 | 72 | 13692 | 3.77314814814815 | 0.0104367672541356 | 0.153191210581377 | 1.98143400143949 | DRD2//HTR2A//CALCA//SCN11A//TRPV2 |
| GO:0006937 | regulation of muscle contraction | Biological process | 3 | 86 | 72 | 13692 | 6.63372093023256 | 0.0104480319260565 | 0.153191210581377 | 1.9809655089931 | CALCA//TACR1//TACR3 |
| GO:0071702 | organic substance transport | Biological process | 7 | 462 | 72 | 13692 | 2.88131313131313 | 0.0106069862817615 | 0.155003426197475 | 1.97440799283015 | HRH3//HTR2C//DRD2//HTR2A//SNCG//SLC7A3//SLC6A5 |
| GO:0040018 | positive regulation of multicellular organism growth | Biological process | 2 | 30 | 72 | 13692 | 12.6777777777778 | 0.0107861512123635 | 0.156577771241727 | 1.96713349557811 | DRD2//DIO3 |
| GO:0046850 | regulation of bone remodeling | Biological process | 2 | 30 | 72 | 13692 | 12.6777777777778 | 0.0107861512123635 | 0.156577771241727 | 1.96713349557811 | CALCA//MC4R |
| GO:0032147 | activation of protein kinase activity | Biological process | 4 | 164 | 72 | 13692 | 4.63821138211382 | 0.0108607007538292 | 0.157139643910189 | 1.96414215230676 | TGFB3//ADRA2A//CALCA//DRD2 |
| GO:0042327 | positive regulation of phosphorylation | Biological process | 7 | 468 | 72 | 13692 | 2.84437321937322 | 0.0113321777662346 | 0.163421931997278 | 1.9456866213834 | TGFB3//CAMP//ADRA2A//CALCA//DRD2//HTR2A//TPD52L1 |
| GO:0050789 | regulation of biological process | Biological process | 50 | 7619 | 72 | 13692 | 1.24797654985344 | 0.0114864002735635 | 0.164631180211315 | 1.93981605366769 | NR2F2//TGFB3//ADRA2A//KRT1//SLIT2//CAMP//MC4R//CALCA//DRD2//SMARCD3//TAC1//TACR1//P2RX2//PTPRO//EPOR//ARHGAP8//IGSF1//RGD1564053//ASB2//PLCXD3//HTR2A//TPD52L1//NPPA//SSTR1//BHLHA15//NPW//OLR278//HTR2C//HRH3//HTR3A//TACR3//RAB20//DOK3//SCX//MYO16//PLAGL1//LRP2//SNCG//SST//ITGA8//SLC7A3//NNAT//HRK//LCP1//SCN11A//DIO3//EFNB1//SCN9A//TRPV2//GPR149 |
| GO:0051492 | regulation of stress fiber assembly | Biological process | 2 | 31 | 72 | 13692 | 12.2688172043011 | 0.0114911362100051 | 0.164631180211315 | 1.93963702741796 | TAC1//TACR1 |
| GO:0006813 | potassium ion transport | Biological process | 3 | 90 | 72 | 13692 | 6.33888888888889 | 0.0118164853862114 | 0.167874721114355 | 1.92751167779905 | HTR2A//DRD2//KCNN3 |
| GO:0007188 | adenylate cyclase-modulating G-protein coupled receptor signaling pathway | Biological process | 3 | 90 | 72 | 13692 | 6.33888888888889 | 0.0118164853862114 | 0.167874721114355 | 1.92751167779905 | CALCA//DRD2//ADRA2A |
| GO:0009653 | anatomical structure morphogenesis | Biological process | 17 | 1819 | 72 | 13692 | 1.77725856697819 | 0.0118324107719744 | 0.167874721114355 | 1.92692676185555 | PTPRO//SCX//SLIT2//SMARCD3//TGFB3//DRD2//NTNG1//EFNB1//HTR2C//LRP2//NNMT//LCP1//RSPH9//ITGA8//CAMP//TRPV2//NR2F2 |
| GO:0006952 | defense response | Biological process | 9 | 711 | 72 | 13692 | 2.40717299578059 | 0.0118869535445271 | 0.168104530126474 | 1.92492943455938 | KRT1//TACR1//ADRA2A//TAC1//CALCA//SCN9A//CAMP//MX1//SLIT2 |
| GO:0042391 | regulation of membrane potential | Biological process | 5 | 262 | 72 | 13692 | 3.62913486005089 | 0.0121984202504799 | 0.169577955671203 | 1.91369640874512 | SCN9A//P2RX2//TAC1//TACR1//CHRNE |
| GO:0007193 | adenylate cyclase-inhibiting G-protein coupled receptor signaling pathway | Biological process | 2 | 32 | 72 | 13692 | 11.8854166666667 | 0.0122158878977571 | 0.169577955671203 | 1.91307496134856 | DRD2//ADRA2A |
| GO:0010631 | epithelial cell migration | Biological process | 2 | 32 | 72 | 13692 | 11.8854166666667 | 0.0122158878977571 | 0.169577955671203 | 1.91307496134856 | TAC1//TACR1 |
| GO:0090132 | epithelium migration | Biological process | 2 | 32 | 72 | 13692 | 11.8854166666667 | 0.0122158878977571 | 0.169577955671203 | 1.91307496134856 | TAC1//TACR1 |
| GO:0010562 | positive regulation of phosphorus metabolic process | Biological process | 7 | 475 | 72 | 13692 | 2.80245614035088 | 0.0122232285565922 | 0.169577955671203 | 1.9128140674879 | TGFB3//CAMP//ADRA2A//CALCA//DRD2//HTR2A//TPD52L1 |
| GO:0045937 | positive regulation of phosphate metabolic process | Biological process | 7 | 475 | 72 | 13692 | 2.80245614035088 | 0.0122232285565922 | 0.169577955671203 | 1.9128140674879 | TGFB3//CAMP//ADRA2A//CALCA//DRD2//HTR2A//TPD52L1 |
| GO:0043549 | regulation of kinase activity | Biological process | 7 | 476 | 72 | 13692 | 2.79656862745098 | 0.012354558813056 | 0.170859261313683 | 1.90817275883951 | TGFB3//ADRA2A//CALCA//DRD2//HTR2A//TPD52L1//NR2F2 |
| GO:0042325 | regulation of phosphorylation | Biological process | 9 | 716 | 72 | 13692 | 2.39036312849162 | 0.0124034274575618 | 0.170995679163368 | 1.90645828901326 | TGFB3//SLIT2//CAMP//ADRA2A//CALCA//DRD2//HTR2A//TPD52L1//NR2F2 |
| GO:0072521 | purine-containing compound metabolic process | Biological process | 8 | 597 | 72 | 13692 | 2.54829704075935 | 0.0128209854622156 | 0.175905419992268 | 1.89207859226229 | NPPA//HTR2C//CALCA//DRD2//HRH3//ADRA2A//MC4R//ARHGAP8 |
| GO:0048858 | cell projection morphogenesis | Biological process | 7 | 480 | 72 | 13692 | 2.77326388888889 | 0.0128901471159463 | 0.175905419992268 | 1.8897421259919 | DRD2//NTNG1//SLIT2//EFNB1//PTPRO//RSPH9//TRPV2 |
| GO:0042755 | eating behavior | Biological process | 2 | 33 | 72 | 13692 | 11.5252525252525 | 0.0129601849127515 | 0.175905419992268 | 1.88738880201367 | TACR1//HRH3 |
| GO:0045933 | positive regulation of muscle contraction | Biological process | 2 | 33 | 72 | 13692 | 11.5252525252525 | 0.0129601849127515 | 0.175905419992268 | 1.88738880201367 | TACR1//TACR3 |
| GO:2000179 | positive regulation of neural precursor cell proliferation | Biological process | 2 | 33 | 72 | 13692 | 11.5252525252525 | 0.0129601849127515 | 0.175905419992268 | 1.88738880201367 | DRD2//SMARCD3 |
| GO:0006954 | inflammatory response | Biological process | 6 | 371 | 72 | 13692 | 3.07547169811321 | 0.0132305047917826 | 0.178634686544272 | 1.87842358558548 | TACR1//ADRA2A//TAC1//CALCA//SLIT2//SCN9A |
| GO:0015833 | peptide transport | Biological process | 4 | 174 | 72 | 13692 | 4.37164750957854 | 0.0132634638459082 | 0.178634686544272 | 1.87734304209158 | MC4R//NNAT//DRD2//ADRA2A |
| GO:0043542 | endothelial cell migration | Biological process | 3 | 94 | 72 | 13692 | 6.06914893617021 | 0.0132835099939399 | 0.178634686544272 | 1.87668715316195 | SLIT2//NR2F2//CALCA |
| GO:0007568 | aging | Biological process | 5 | 268 | 72 | 13692 | 3.5478855721393 | 0.0133473546449193 | 0.178944350958184 | 1.87460479998893 | CALCA//TACR3//TGFB3//LRP2//HTR2A |
| GO:0046488 | phosphatidylinositol metabolic process | Biological process | 3 | 95 | 72 | 13692 | 6.00526315789474 | 0.013665791863175 | 0.181220399375036 | 1.8643651980845 | HTR2C//HTR2A//DRD2 |
| GO:0050671 | positive regulation of lymphocyte proliferation | Biological process | 3 | 95 | 72 | 13692 | 6.00526315789474 | 0.013665791863175 | 0.181220399375036 | 1.8643651980845 | EFNB1//TAC1//TACR1 |
| GO:0030032 | lamellipodium assembly | Biological process | 2 | 34 | 72 | 13692 | 11.1862745098039 | 0.0137238076169051 | 0.181220399375036 | 1.86252537860057 | SLIT2//PTPRO |
| GO:0030431 | sleep | Biological process | 2 | 34 | 72 | 13692 | 11.1862745098039 | 0.0137238076169051 | 0.181220399375036 | 1.86252537860057 | DRD2//HTR2A |
| GO:0046676 | negative regulation of insulin secretion | Biological process | 2 | 34 | 72 | 13692 | 11.1862745098039 | 0.0137238076169051 | 0.181220399375036 | 1.86252537860057 | ADRA2A//DRD2 |
| GO:0071902 | positive regulation of protein serine/threonine kinase activity | Biological process | 4 | 177 | 72 | 13692 | 4.29755178907721 | 0.0140452809236718 | 0.184473380214064 | 1.85246956992767 | TGFB3//ADRA2A//HTR2A//TPD52L1 |
| GO:0032946 | positive regulation of mononuclear cell proliferation | Biological process | 3 | 96 | 72 | 13692 | 5.94270833333333 | 0.0140543131823671 | 0.184473380214064 | 1.85219037299473 | EFNB1//TAC1//TACR1 |
| GO:0048667 | cell morphogenesis involved in neuron differentiation | Biological process | 6 | 378 | 72 | 13692 | 3.01851851851852 | 0.0143949565171216 | 0.187930101606852 | 1.84178964265826 | DRD2//NTNG1//SLIT2//EFNB1//PTPRO//TRPV2 |
| GO:0040007 | growth | Biological process | 9 | 734 | 72 | 13692 | 2.33174386920981 | 0.0144034019479704 | 0.187930101606852 | 1.84153491952017 | GPR149//TGFB3//NPPA//SLIT2//CAMP//DRD2//DIO3//TRPV2 |
| GO:0071900 | regulation of protein serine/threonine kinase activity | Biological process | 5 | 274 | 72 | 13692 | 3.47019464720195 | 0.0145675832968437 | 0.188781263257247 | 1.83661248995268 | TGFB3//ADRA2A//HTR2A//TPD52L1//NR2F2 |
| GO:0007015 | actin filament organization | Biological process | 4 | 179 | 72 | 13692 | 4.24953445065177 | 0.0145824605554719 | 0.188781263257247 | 1.83616918963232 | LCP1//SLIT2//TAC1//TACR1 |
| GO:0051050 | positive regulation of transport | Biological process | 7 | 492 | 72 | 13692 | 2.70562330623306 | 0.0145978212235873 | 0.188781263257247 | 1.83571195936515 | DRD2//TAC1//TACR1//HTR2C//NNAT//TGFB3//TRPV2 |
| GO:0032990 | cell part morphogenesis | Biological process | 7 | 493 | 72 | 13692 | 2.70013522650439 | 0.0147471171158848 | 0.190151063047173 | 1.83129287073888 | DRD2//NTNG1//SLIT2//EFNB1//PTPRO//RSPH9//TRPV2 |
| GO:0031099 | regeneration | Biological process | 4 | 180 | 72 | 13692 | 4.22592592592593 | 0.0148558783345324 | 0.190991702693812 | 1.82810166600344 | HTR2C//LRP2//NNMT//LCP1 |
| GO:0000904 | cell morphogenesis involved in differentiation | Biological process | 7 | 495 | 72 | 13692 | 2.68922558922559 | 0.0150489983721509 | 0.192908797846519 | 1.82249240478268 | DRD2//NTNG1//SLIT2//EFNB1//PTPRO//TGFB3//TRPV2 |
| GO:0001990 | regulation of systemic arterial blood pressure by hormone | Biological process | 2 | 36 | 72 | 13692 | 10.5648148148148 | 0.0153081600947061 | 0.194524561899106 | 1.81507700451627 | HSD11B2//TACR1 |
| GO:0032890 | regulation of organic acid transport | Biological process | 2 | 36 | 72 | 13692 | 10.5648148148148 | 0.0153081600947061 | 0.194524561899106 | 1.81507700451627 | HRH3//HTR2C |
| GO:0090130 | tissue migration | Biological process | 2 | 36 | 72 | 13692 | 10.5648148148148 | 0.0153081600947061 | 0.194524561899106 | 1.81507700451627 | TAC1//TACR1 |
| GO:0006811 | ion transport | Biological process | 9 | 742 | 72 | 13692 | 2.30660377358491 | 0.0153656958273117 | 0.194691359846631 | 1.81344776816911 | DRD2//HTR2A//P2RX2//KCNN3//SCN11A//BHLHA15//CALCA//HTR2C//TRPV2 |
| GO:0030031 | cell projection assembly | Biological process | 4 | 182 | 72 | 13692 | 4.17948717948718 | 0.0154124305065522 | 0.194720735852233 | 1.81212886859176 | SLIT2//PTPRO//RSPH9//TGFB3 |
| GO:0001503 | ossification | Biological process | 5 | 279 | 72 | 13692 | 3.40800477897252 | 0.0156402239145813 | 0.19685349533719 | 1.80575703361818 | SCX//CALCA//TGFB3//TAC1//TACR1 |
| GO:0070665 | positive regulation of leukocyte proliferation | Biological process | 3 | 100 | 72 | 13692 | 5.705 | 0.015671046960009 | 0.19685349533719 | 1.8049019879752 | EFNB1//TAC1//TACR1 |
| GO:0051338 | regulation of transferase activity | Biological process | 7 | 500 | 72 | 13692 | 2.66233333333333 | 0.015823085296085 | 0.198195445537248 | 1.80070883079902 | TGFB3//ADRA2A//CALCA//DRD2//HTR2A//TPD52L1//NR2F2 |
| GO:0090278 | negative regulation of peptide hormone secretion | Biological process | 2 | 37 | 72 | 13692 | 10.2792792792793 | 0.0161284591120024 | 0.20144491380917 | 1.79240712245113 | DRD2//ADRA2A |
| GO:0030595 | leukocyte chemotaxis | Biological process | 3 | 102 | 72 | 13692 | 5.59313725490196 | 0.0165171603066767 | 0.205140608909006 | 1.78206461616504 | CALCA//PTPRO//SLIT2 |
| GO:0048812 | neuron projection morphogenesis | Biological process | 6 | 390 | 72 | 13692 | 2.92564102564103 | 0.0165552314896299 | 0.205140608909006 | 1.78106474218669 | DRD2//NTNG1//SLIT2//EFNB1//PTPRO//TRPV2 |
| GO:0010638 | positive regulation of organelle organization | Biological process | 4 | 186 | 72 | 13692 | 4.08960573476702 | 0.0165647298252254 | 0.205140608909006 | 1.78081564317294 | DRD2//TAC1//TACR1//HRK |
| GO:0042493 | response to drug | Biological process | 7 | 505 | 72 | 13692 | 2.63597359735974 | 0.0166252783211357 | 0.205310479323546 | 1.77923107550927 | DRD2//SST//HSD11B2//HTR2C//LRP2//HTR2A//NNMT |
| GO:0050796 | regulation of insulin secretion | Biological process | 3 | 103 | 72 | 13692 | 5.53883495145631 | 0.0169496879384763 | 0.206623062467291 | 1.77083829320399 | NNAT//DRD2//ADRA2A |
| GO:0002792 | negative regulation of peptide secretion | Biological process | 2 | 38 | 72 | 13692 | 10.0087719298246 | 0.016967222282898 | 0.206623062467291 | 1.77038925054917 | DRD2//ADRA2A |
| GO:0014910 | regulation of smooth muscle cell migration | Biological process | 2 | 38 | 72 | 13692 | 10.0087719298246 | 0.016967222282898 | 0.206623062467291 | 1.77038925054917 | SLIT2//TACR1 |
| GO:0022029 | telencephalon cell migration | Biological process | 2 | 38 | 72 | 13692 | 10.0087719298246 | 0.016967222282898 | 0.206623062467291 | 1.77038925054917 | SLIT2//DRD2 |
| GO:0032231 | regulation of actin filament bundle assembly | Biological process | 2 | 38 | 72 | 13692 | 10.0087719298246 | 0.016967222282898 | 0.206623062467291 | 1.77038925054917 | TAC1//TACR1 |
| GO:0030030 | cell projection organization | Biological process | 9 | 756 | 72 | 13692 | 2.26388888888889 | 0.0171636260754673 | 0.208435835775204 | 1.76539095551959 | DRD2//NTNG1//SLIT2//EFNB1//PTPRO//RSPH9//TRPV2//TGFB3//ITGA8 |
| GO:0051051 | negative regulation of transport | Biological process | 5 | 286 | 72 | 13692 | 3.32459207459207 | 0.0172291693225122 | 0.208653807485894 | 1.76373566087732 | HTR2A//CALCA//HRH3//ADRA2A//DRD2 |
| GO:0001659 | temperature homeostasis | Biological process | 2 | 39 | 72 | 13692 | 9.75213675213675 | 0.0178242384205528 | 0.21361490805299 | 1.74898901725828 | DRD2//HTR2A |
| GO:0043149 | stress fiber assembly | Biological process | 2 | 39 | 72 | 13692 | 9.75213675213675 | 0.0178242384205528 | 0.21361490805299 | 1.74898901725828 | TAC1//TACR1 |
| GO:0050805 | negative regulation of synaptic transmission | Biological process | 2 | 39 | 72 | 13692 | 9.75213675213675 | 0.0178242384205528 | 0.21361490805299 | 1.74898901725828 | DRD2//HTR2A |
| GO:0002526 | acute inflammatory response | Biological process | 3 | 105 | 72 | 13692 | 5.43333333333333 | 0.0178337263566137 | 0.21361490805299 | 1.74875790152305 | TAC1//TACR1//ADRA2A |
| GO:0090257 | regulation of muscle system process | Biological process | 3 | 106 | 72 | 13692 | 5.38207547169811 | 0.0182852516044762 | 0.218426547776631 | 1.73789905940667 | CALCA//TACR1//TACR3 |
| GO:0034103 | regulation of tissue remodeling | Biological process | 2 | 40 | 72 | 13692 | 9.50833333333333 | 0.0186992979943362 | 0.222161849341924 | 1.72817469736237 | CALCA//MC4R |
| GO:0051966 | regulation of synaptic transmission, glutamatergic | Biological process | 2 | 40 | 72 | 13692 | 9.50833333333333 | 0.0186992979943362 | 0.222161849341924 | 1.72817469736237 | DRD2//HTR2A |
| GO:0022602 | ovulation cycle process | Biological process | 3 | 108 | 72 | 13692 | 5.28240740740741 | 0.0192073453522992 | 0.227581086552648 | 1.71653265484238 | GPR149//TGFB3//SLIT2 |
| GO:0043405 | regulation of MAP kinase activity | Biological process | 4 | 195 | 72 | 13692 | 3.9008547008547 | 0.0193516997735723 | 0.22867345500631 | 1.71328088233181 | TGFB3//ADRA2A//HTR2A//TPD52L1 |
| GO:0014909 | smooth muscle cell migration | Biological process | 2 | 41 | 72 | 13692 | 9.27642276422764 | 0.0195921931186442 | 0.229658220941541 | 1.70791694705708 | TACR1//SLIT2 |
| GO:0021885 | forebrain cell migration | Biological process | 2 | 41 | 72 | 13692 | 9.27642276422764 | 0.0195921931186442 | 0.229658220941541 | 1.70791694705708 | SLIT2//DRD2 |
| GO:0046068 | cGMP metabolic process | Biological process | 2 | 41 | 72 | 13692 | 9.27642276422764 | 0.0195921931186442 | 0.229658220941541 | 1.70791694705708 | NPPA//HTR2C |
| GO:0006898 | receptor-mediated endocytosis | Biological process | 3 | 109 | 72 | 13692 | 5.23394495412844 | 0.019677924366414 | 0.230048054459624 | 1.70602071300595 | CALCA//DRD2//LRP2 |
| GO:0001894 | tissue homeostasis | Biological process | 3 | 110 | 72 | 13692 | 5.18636363636364 | 0.0201548640834225 | 0.234373804089454 | 1.69562012621441 | CALCA//MC4R//SCX |
| GO:0006576 | cellular biogenic amine metabolic process | Biological process | 3 | 110 | 72 | 13692 | 5.18636363636364 | 0.0201548640834225 | 0.234373804089454 | 1.69562012621441 | TACR3//DRD2//HTR2C |
| GO:0006725 | cellular aromatic compound metabolic process | Biological process | 4 | 198 | 72 | 13692 | 3.84175084175084 | 0.0203414525207871 | 0.235917798547965 | 1.69161803866796 | TACR3//DIO3//DRD2//HTR2C |
| GO:0046928 | regulation of neurotransmitter secretion | Biological process | 2 | 42 | 72 | 13692 | 9.05555555555556 | 0.0205027175417858 | 0.237160722172002 | 1.68818857137437 | HTR2C//SNCG |
| GO:0001667 | ameboidal cell migration | Biological process | 3 | 111 | 72 | 13692 | 5.13963963963964 | 0.0206381681729534 | 0.238099287553231 | 1.68532885295972 | EFNB1//TAC1//TACR1 |
| GO:0010959 | regulation of metal ion transport | Biological process | 4 | 201 | 72 | 13692 | 3.78441127694859 | 0.0213620071517892 | 0.245803252896178 | 1.67035794387226 | DRD2//HTR2A//CALCA//TRPV2 |
| GO:0051970 | negative regulation of transmission of nerve impulse | Biological process | 2 | 43 | 72 | 13692 | 8.84496124031008 | 0.0214306666349389 | 0.24594775530778 | 1.6689643193281 | DRD2//HTR2A |
| GO:0031399 | regulation of protein modification process | Biological process | 9 | 789 | 72 | 13692 | 2.16920152091255 | 0.0220106318422345 | 0.251944151426517 | 1.65736749031093 | TGFB3//SLIT2//CAMP//ADRA2A//CALCA//DRD2//HTR2A//TPD52L1//NR2F2 |
| GO:0090276 | regulation of peptide hormone secretion | Biological process | 3 | 114 | 72 | 13692 | 5.00438596491228 | 0.0221262941220146 | 0.252608524559667 | 1.65509131888489 | NNAT//DRD2//ADRA2A |
| GO:0030900 | forebrain development | Biological process | 5 | 306 | 72 | 13692 | 3.10729847494553 | 0.0223516913182153 | 0.254133862899128 | 1.65068960889102 | SLIT2//DRD2//SSTR1//LRP2//NR2F2 |
| GO:0003044 | regulation of systemic arterial blood pressure mediated by a chemical signal | Biological process | 2 | 44 | 72 | 13692 | 8.64393939393939 | 0.0223758373811732 | 0.254133862899128 | 1.65022070290734 | HSD11B2//TACR1 |
| GO:0031401 | positive regulation of protein modification process | Biological process | 7 | 537 | 72 | 13692 | 2.47889509621353 | 0.0224582169550721 | 0.254410395687432 | 1.64862472702881 | TGFB3//CAMP//ADRA2A//CALCA//DRD2//HTR2A//TPD52L1 |
| GO:0050678 | regulation of epithelial cell proliferation | Biological process | 4 | 205 | 72 | 13692 | 3.71056910569106 | 0.0227710569559462 | 0.257099513490657 | 1.64261681042651 | NR2F2//TACR1//HRH3//TGFB3 |
| GO:0009117 | nucleotide metabolic process | Biological process | 8 | 664 | 72 | 13692 | 2.29116465863454 | 0.0228375669095723 | 0.257099513490657 | 1.64135016724026 | NPPA//HTR2C//CALCA//DRD2//HRH3//ADRA2A//MC4R//ARHGAP8 |
| GO:0048699 | generation of neurons | Biological process | 10 | 929 | 72 | 13692 | 2.0470039468963 | 0.0229266400285462 | 0.257099513490657 | 1.63965958783364 | NR2F2//KIRREL3//DRD2//SMARCD3//NTNG1//SLIT2//EFNB1//PTPRO//NNAT//TRPV2 |
| GO:0019220 | regulation of phosphate metabolic process | Biological process | 9 | 795 | 72 | 13692 | 2.15283018867925 | 0.0229888251113909 | 0.257099513490657 | 1.63848322362755 | TGFB3//SLIT2//CAMP//ADRA2A//CALCA//DRD2//HTR2A//TPD52L1//NR2F2 |
| GO:0051174 | regulation of phosphorus metabolic process | Biological process | 9 | 795 | 72 | 13692 | 2.15283018867925 | 0.0229888251113909 | 0.257099513490657 | 1.63848322362755 | TGFB3//SLIT2//CAMP//ADRA2A//CALCA//DRD2//HTR2A//TPD52L1//NR2F2 |
| GO:0030534 | adult behavior | Biological process | 3 | 116 | 72 | 13692 | 4.91810344827586 | 0.0231502397021601 | 0.2575904844017 | 1.6354445078518 | DRD2//SNCG//HTR2C |
| GO:0044106 | cellular amine metabolic process | Biological process | 3 | 116 | 72 | 13692 | 4.91810344827586 | 0.0231502397021601 | 0.2575904844017 | 1.6354445078518 | TACR3//DRD2//HTR2C |
| GO:0014812 | muscle cell migration | Biological process | 2 | 45 | 72 | 13692 | 8.45185185185185 | 0.0233380283645428 | 0.258368475631706 | 1.63193583666117 | TACR1//SLIT2 |
| GO:0045446 | endothelial cell differentiation | Biological process | 2 | 45 | 72 | 13692 | 8.45185185185185 | 0.0233380283645428 | 0.258368475631706 | 1.63193583666117 | TGFB3//NR2F2 |
| GO:0002791 | regulation of peptide secretion | Biological process | 3 | 117 | 72 | 13692 | 4.87606837606838 | 0.0236717729384927 | 0.26074636322199 | 1.62576921361426 | NNAT//DRD2//ADRA2A |
| GO:0090087 | regulation of peptide transport | Biological process | 3 | 117 | 72 | 13692 | 4.87606837606838 | 0.0236717729384927 | 0.26074636322199 | 1.62576921361426 | NNAT//DRD2//ADRA2A |
| GO:0002376 | immune system process | Biological process | 12 | 1218 | 72 | 13692 | 1.8735632183908 | 0.0242168328196983 | 0.266081691933728 | 1.6158826563569 | KRT1//LCP1//CALCA//PTPRO//TACR1//SLIT2//TGFB3//KIRREL3//SSTR1//EFNB1//MX1//TAC1 |
| GO:0044087 | regulation of cellular component biogenesis | Biological process | 5 | 315 | 72 | 13692 | 3.01851851851852 | 0.0249492832636829 | 0.273444144569965 | 1.60294192615677 | SLIT2//HRK//TGFB3//TAC1//TACR1 |
| GO:0042698 | ovulation cycle | Biological process | 3 | 120 | 72 | 13692 | 4.75416666666667 | 0.0252746067530004 | 0.27468009858871 | 1.59731559286181 | GPR149//TGFB3//SLIT2 |
| GO:0032570 | response to progesterone stimulus | Biological process | 2 | 47 | 72 | 13692 | 8.09219858156028 | 0.0253126733188501 | 0.27468009858871 | 1.5966619857657 | TACR1//TGFB3 |
| GO:0032963 | collagen metabolic process | Biological process | 2 | 47 | 72 | 13692 | 8.09219858156028 | 0.0253126733188501 | 0.27468009858871 | 1.5966619857657 | TGFB3//SCX |
| GO:0045453 | bone resorption | Biological process | 2 | 47 | 72 | 13692 | 8.09219858156028 | 0.0253126733188501 | 0.27468009858871 | 1.5966619857657 | CALCA//MC4R |
| GO:0006468 | protein phosphorylation | Biological process | 10 | 945 | 72 | 13692 | 2.01234567901235 | 0.025453469632491 | 0.275525952762569 | 1.59425300941604 | TGFB3//SLIT2//CAMP//ADRA2A//CALCA//DRD2//HTR2A//TPD52L1//NR2F2//SRPK3 |
| GO:0030336 | negative regulation of cell migration | Biological process | 3 | 122 | 72 | 13692 | 4.67622950819672 | 0.0263750068229907 | 0.284798103231505 | 1.57880741934594 | SLIT2//NR2F2//DRD2 |
| GO:0032501 | multicellular organismal process | Biological process | 39 | 5780 | 72 | 13692 | 1.28313148788927 | 0.026891487250359 | 0.28941655079177 | 1.57038517818716 | GPR149//SLIT2//ITGA8//DRD2//HTR2A//TGFB3//SCX//EFNB1//NR2F2//KIRREL3//ADRA2A//CALCA//HSD11B2//MC4R//SMARCD3//P2RX2//TACR1//TAC1//PTPRO//SNCG//HTR3A//SSTR1//EPOR//NTNG1//HRH3//NNAT//NPPA//SRPK3//SCN9A//TACR3//HTR2C//MYO16//LRP2//NNMT//LCP1//DIO3//CAMP//TRPV2//OLR278 |
| GO:0050808 | synapse organization | Biological process | 3 | 123 | 72 | 13692 | 4.63821138211382 | 0.026934751989745 | 0.28941655079177 | 1.56968701900353 | DRD2//P2RX2//SNCG |
| GO:0006753 | nucleoside phosphate metabolic process | Biological process | 8 | 686 | 72 | 13692 | 2.21768707482993 | 0.0270921830553694 | 0.290396407126502 | 1.56715599863233 | NPPA//HTR2C//CALCA//DRD2//HRH3//ADRA2A//MC4R//ARHGAP8 |
| GO:0003158 | endothelium development | Biological process | 2 | 49 | 72 | 13692 | 7.76190476190476 | 0.0273530217797201 | 0.291765565650348 | 1.56299468871754 | TGFB3//NR2F2 |
| GO:0007405 | neuroblast proliferation | Biological process | 2 | 49 | 72 | 13692 | 7.76190476190476 | 0.0273530217797201 | 0.291765565650348 | 1.56299468871754 | DRD2//SMARCD3 |
| GO:2000146 | negative regulation of cell motility | Biological process | 3 | 124 | 72 | 13692 | 4.6008064516129 | 0.0275008557236423 | 0.291963240747925 | 1.5606537923416 | SLIT2//NR2F2//DRD2 |
| GO:0009611 | response to wounding | Biological process | 8 | 688 | 72 | 13692 | 2.21124031007752 | 0.0275047487292183 | 0.291963240747925 | 1.56059231821149 | TACR1//ADRA2A//TAC1//CALCA//SCN9A//TGFB3//DRD2//SLIT2 |
| GO:0034765 | regulation of ion transmembrane transport | Biological process | 3 | 125 | 72 | 13692 | 4.564 | 0.0280733137333766 | 0.297278761852954 | 1.55170632084686 | DRD2//TRPV2//SCN11A |
| GO:0006661 | phosphatidylinositol biosynthetic process | Biological process | 2 | 50 | 72 | 13692 | 7.60666666666667 | 0.0283973479889512 | 0.298546699241156 | 1.54672221655661 | HTR2C//HTR2A |
| GO:0030819 | positive regulation of cAMP biosynthetic process | Biological process | 2 | 50 | 72 | 13692 | 7.60666666666667 | 0.0283973479889512 | 0.298546699241156 | 1.54672221655661 | CALCA//MC4R |
| GO:0044259 | multicellular organismal macromolecule metabolic process | Biological process | 2 | 50 | 72 | 13692 | 7.60666666666667 | 0.0283973479889512 | 0.298546699241156 | 1.54672221655661 | TGFB3//SCX |
| GO:0051716 | cellular response to stimulus | Biological process | 35 | 5068 | 72 | 13692 | 1.31330570902394 | 0.0287374720120701 | 0.30139970646152 | 1.5415514386895 | TGFB3//CALCA//PTPRO//SLIT2//P2RX2//DRD2//EPOR//ADRA2A//ARHGAP8//IGSF1//RGD1564053//ASB2//PLCXD3//NPPA//SSTR1//BHLHA15//HTR2A//NPW//OLR278//HTR2C//HTR3A//TAC1//TACR1//TACR3//RAB20//DOK3//SCX//ITGA8//NR2F2//SLC7A3//TPD52L1//EFNB1//HRK//CAMP//SNCG |
| GO:0060326 | cell chemotaxis | Biological process | 3 | 127 | 72 | 13692 | 4.49212598425197 | 0.0292372728628053 | 0.305909795299614 | 1.53406313909689 | CALCA//PTPRO//SLIT2 |
| GO:0030816 | positive regulation of cAMP metabolic process | Biological process | 2 | 51 | 72 | 13692 | 7.45751633986928 | 0.0294575189579339 | 0.306023135335503 | 1.53080383414829 | CALCA//MC4R |
| GO:0060491 | regulation of cell projection assembly | Biological process | 2 | 51 | 72 | 13692 | 7.45751633986928 | 0.0294575189579339 | 0.306023135335503 | 1.53080383414829 | SLIT2//TGFB3 |
| GO:2000177 | regulation of neural precursor cell proliferation | Biological process | 2 | 51 | 72 | 13692 | 7.45751633986928 | 0.0294575189579339 | 0.306023135335503 | 1.53080383414829 | DRD2//SMARCD3 |
| GO:0051271 | negative regulation of cellular component movement | Biological process | 3 | 128 | 72 | 13692 | 4.45703125 | 0.0298287628690622 | 0.309147272855718 | 1.52536475838052 | SLIT2//NR2F2//DRD2 |
| GO:0072358 | cardiovascular system development | Biological process | 8 | 701 | 72 | 13692 | 2.17023300047551 | 0.0302943714963421 | 0.312495352094032 | 1.51863805318567 | CALCA//SLIT2//SMARCD3//SCX//EPOR//NPPA//CAMP//NR2F2 |
| GO:0072359 | circulatory system development | Biological process | 8 | 701 | 72 | 13692 | 2.17023300047551 | 0.0302943714963421 | 0.312495352094032 | 1.51863805318567 | CALCA//SLIT2//SMARCD3//SCX//EPOR//NPPA//CAMP//NR2F2 |
| GO:0048771 | tissue remodeling | Biological process | 3 | 129 | 72 | 13692 | 4.42248062015504 | 0.0304265849384568 | 0.313122414014541 | 1.51674678985721 | TGFB3//CALCA//MC4R |
| GO:0031623 | receptor internalization | Biological process | 2 | 53 | 72 | 13692 | 7.17610062893082 | 0.031624634655884 | 0.324689457450575 | 1.49997448298135 | CALCA//DRD2 |
| GO:0006836 | neurotransmitter transport | Biological process | 3 | 132 | 72 | 13692 | 4.3219696969697 | 0.0322579733531509 | 0.330418119579938 | 1.49136292117494 | HTR2C//SNCG//DRD2 |
| GO:0019637 | organophosphate metabolic process | Biological process | 9 | 846 | 72 | 13692 | 2.02304964539007 | 0.0326083719813818 | 0.333228677777104 | 1.48667088341275 | NPPA//HTR2C//HTR2A//CALCA//DRD2//HRH3//ADRA2A//MC4R//ARHGAP8 |
| GO:0009605 | response to external stimulus | Biological process | 11 | 1128 | 72 | 13692 | 1.8544621749409 | 0.0327846340501466 | 0.334250780641495 | 1.48432965966832 | DRD2//CALCA//PTPRO//TAC1//SLIT2//EFNB1//TACR1//HSD11B2//LRP2//SSTR1//HRK |
| GO:0050769 | positive regulation of neurogenesis | Biological process | 3 | 133 | 72 | 13692 | 4.28947368421053 | 0.0328810511469894 | 0.334455981968449 | 1.48305430731081 | DRD2//SMARCD3//TRPV2 |
| GO:0006796 | phosphate-containing compound metabolic process | Biological process | 12 | 1275 | 72 | 13692 | 1.78980392156863 | 0.0330670170555028 | 0.335568987896584 | 1.48060498061593 | TGFB3//SLIT2//CAMP//ADRA2A//CALCA//SRPK3//DUSP26//DRD2//PTPRO//HTR2A//TPD52L1//NR2F2 |
| GO:0006793 | phosphorus metabolic process | Biological process | 12 | 1277 | 72 | 13692 | 1.78700078308536 | 0.0334152261415053 | 0.338319518254871 | 1.47605559539094 | TGFB3//SLIT2//CAMP//ADRA2A//CALCA//SRPK3//DUSP26//DRD2//PTPRO//HTR2A//TPD52L1//NR2F2 |
| GO:0035264 | multicellular organism growth | Biological process | 3 | 134 | 72 | 13692 | 4.25746268656716 | 0.033510422407886 | 0.338501594092563 | 1.47482009773149 | DRD2//DIO3 |
| GO:0021954 | central nervous system neuron development | Biological process | 2 | 56 | 72 | 13692 | 6.79166666666667 | 0.0349894302376047 | 0.351820326058851 | 1.45606312944276 | DRD2//SLIT2 |
| GO:0090277 | positive regulation of peptide hormone secretion | Biological process | 2 | 56 | 72 | 13692 | 6.79166666666667 | 0.0349894302376047 | 0.351820326058851 | 1.45606312944276 | NNAT//DRD2 |
| GO:0050794 | regulation of cellular process | Biological process | 46 | 7207 | 72 | 13692 | 1.21377364599232 | 0.0352727446500013 | 0.353857465779418 | 1.45256074560686 | NR2F2//TGFB3//SLIT2//CAMP//CALCA//DRD2//SMARCD3//TACR1//P2RX2//EPOR//ADRA2A//ARHGAP8//IGSF1//RGD1564053//ASB2//PLCXD3//TPD52L1//NPPA//SSTR1//BHLHA15//HTR2A//NPW//OLR278//HTR2C//HRH3//HTR3A//TAC1//TACR3//RAB20//DOK3//SCX//MYO16//SNCG//SST//ITGA8//MC4R//SLC7A3//NNAT//LCP1//SCN11A//EFNB1//HRK//SCN9A//TRPV2//PLAGL1//PTPRO |
| GO:0007166 | cell surface receptor signaling pathway | Biological process | 22 | 2870 | 72 | 13692 | 1.45772357723577 | 0.0355251832619986 | 0.355576263517356 | 1.44946367302134 | DRD2//ADRA2A//NPPA//TGFB3//SSTR1//BHLHA15//HTR2A//NPW//OLR278//HTR2C//CALCA//HTR3A//TAC1//TACR1//TACR3//SCX//ITGA8//SLIT2//P2RX2//EPOR//EFNB1//PTPRO |
| GO:0040017 | positive regulation of locomotion | Biological process | 4 | 236 | 72 | 13692 | 3.22316384180791 | 0.0356090964188546 | 0.35560427949945 | 1.44843904641236 | TACR1//TAC1//ADRA2A//SLIT2 |
| GO:0000902 | cell morphogenesis | Biological process | 8 | 725 | 72 | 13692 | 2.0983908045977 | 0.0359504290005659 | 0.357654456661222 | 1.44429592276464 | DRD2//NTNG1//SLIT2//EFNB1//PTPRO//TGFB3//RSPH9//TRPV2 |
| GO:0001505 | regulation of neurotransmitter levels | Biological process | 3 | 138 | 72 | 13692 | 4.13405797101449 | 0.0360906536192087 | 0.357654456661222 | 1.44260525258596 | HTR2C//SNCG//DRD2 |
| GO:0045744 | negative regulation of G-protein coupled receptor protein signaling pathway | Biological process | 2 | 57 | 72 | 13692 | 6.67251461988304 | 0.0361407217839693 | 0.357654456661222 | 1.44200317818171 | CALCA//DRD2 |
| GO:0046888 | negative regulation of hormone secretion | Biological process | 2 | 57 | 72 | 13692 | 6.67251461988304 | 0.0361407217839693 | 0.357654456661222 | 1.44200317818171 | DRD2//ADRA2A |
| GO:0009308 | amine metabolic process | Biological process | 3 | 139 | 72 | 13692 | 4.10431654676259 | 0.0367513470302711 | 0.362878165271866 | 1.43472673829155 | TACR3//DRD2//HTR2C |
| GO:0002793 | positive regulation of peptide secretion | Biological process | 2 | 58 | 72 | 13692 | 6.55747126436782 | 0.0373065560080077 | 0.367532452896867 | 1.42821484143772 | NNAT//DRD2 |
| GO:0048511 | rhythmic process | Biological process | 4 | 240 | 72 | 13692 | 3.16944444444444 | 0.0375175066022159 | 0.36878194830519 | 1.42576603236338 | GPR149//TGFB3//DRD2//SLIT2 |
| GO:0022008 | neurogenesis | Biological process | 10 | 1009 | 72 | 13692 | 1.88470432771721 | 0.0376054044554161 | 0.368819000296519 | 1.42474973601653 | NR2F2//KIRREL3//DRD2//SMARCD3//NTNG1//SLIT2//EFNB1//PTPRO//NNAT//TRPV2 |
| GO:0010647 | positive regulation of cell communication | Biological process | 8 | 733 | 72 | 13692 | 2.07548885857208 | 0.0379861765480659 | 0.370893982153053 | 1.42037441762301 | ADRA2A//TGFB3//ITGA8//TAC1//TACR1//DRD2//TPD52L1//ARHGAP8 |
| GO:0055086 | nucleobase-containing small molecule metabolic process | Biological process | 8 | 733 | 72 | 13692 | 2.07548885857208 | 0.0379861765480659 | 0.370893982153053 | 1.42037441762301 | NPPA//HTR2C//CALCA//DRD2//HRH3//ADRA2A//MC4R//ARHGAP8 |
| GO:0051048 | negative regulation of secretion | Biological process | 3 | 141 | 72 | 13692 | 4.04609929078014 | 0.0380914193443297 | 0.371095072012314 | 1.41917284456337 | HRH3//ADRA2A//DRD2 |
| GO:0023056 | positive regulation of signaling | Biological process | 8 | 734 | 72 | 13692 | 2.07266121707539 | 0.0382460418329154 | 0.37177527138692 | 1.41741350427696 | ADRA2A//TGFB3//ITGA8//TAC1//TACR1//DRD2//TPD52L1//ARHGAP8 |
| GO:0022600 | digestive system process | Biological process | 2 | 59 | 72 | 13692 | 6.44632768361582 | 0.0384867527993907 | 0.373287443080816 | 1.41468872961201 | TAC1//TACR1 |
| GO:0007411 | axon guidance | Biological process | 3 | 142 | 72 | 13692 | 4.01760563380282 | 0.0387707745303898 | 0.374385628945438 | 1.41149552293103 | SLIT2//EFNB1//PTPRO |
| GO:0032956 | regulation of actin cytoskeleton organization | Biological process | 3 | 142 | 72 | 13692 | 4.01760563380282 | 0.0387707745303898 | 0.374385628945438 | 1.41149552293103 | SLIT2//TAC1//TACR1 |
| GO:0032270 | positive regulation of cellular protein metabolic process | Biological process | 7 | 604 | 72 | 13692 | 2.20391832229581 | 0.0390040133817851 | 0.375810098166474 | 1.40889070323283 | TGFB3//CAMP//ADRA2A//CALCA//DRD2//HTR2A//TPD52L1 |
| GO:0034762 | regulation of transmembrane transport | Biological process | 4 | 244 | 72 | 13692 | 3.11748633879781 | 0.0394841231051133 | 0.379219748792946 | 1.40357750270193 | SCN11A//DRD2//TGFB3//TRPV2 |
| GO:0051130 | positive regulation of cellular component organization | Biological process | 6 | 478 | 72 | 13692 | 2.38702928870293 | 0.0395308907843012 | 0.379219748792946 | 1.40306339920755 | DRD2//TGFB3//HRK//TRPV2//TAC1//TACR1 |
| GO:0030804 | positive regulation of cyclic nucleotide biosynthetic process | Biological process | 2 | 60 | 72 | 13692 | 6.33888888888889 | 0.039681133493055 | 0.37982988915623 | 1.40141593070263 | CALCA//MC4R |
| GO:0045927 | positive regulation of growth | Biological process | 3 | 144 | 72 | 13692 | 3.96180555555556 | 0.0401480606696692 | 0.383462087093311 | 1.39633542823842 | DRD2//DIO3//TRPV2 |
| GO:0050670 | regulation of lymphocyte proliferation | Biological process | 3 | 145 | 72 | 13692 | 3.93448275862069 | 0.0408459655327416 | 0.386335472946796 | 1.38885083346197 | EFNB1//TAC1//TACR1 |
| GO:0010594 | regulation of endothelial cell migration | Biological process | 2 | 61 | 72 | 13692 | 6.23497267759563 | 0.0408895208593324 | 0.386335472946796 | 1.38838797844951 | NR2F2//SLIT2 |
| GO:0030810 | positive regulation of nucleotide biosynthetic process | Biological process | 2 | 61 | 72 | 13692 | 6.23497267759563 | 0.0408895208593324 | 0.386335472946796 | 1.38838797844951 | CALCA//MC4R |
| GO:0051705 | behavioral interaction between organisms | Biological process | 2 | 61 | 72 | 13692 | 6.23497267759563 | 0.0408895208593324 | 0.386335472946796 | 1.38838797844951 | TACR1//SNCG |
| GO:1900373 | positive regulation of purine nucleotide biosynthetic process | Biological process | 2 | 61 | 72 | 13692 | 6.23497267759563 | 0.0408895208593324 | 0.386335472946796 | 1.38838797844951 | CALCA//MC4R |
| GO:0031325 | positive regulation of cellular metabolic process | Biological process | 14 | 1621 | 72 | 13692 | 1.64240180958256 | 0.041040489372126 | 0.386927968618065 | 1.38678746928155 | TGFB3//CAMP//DRD2//ADRA2A//CALCA//NR2F2//SMARCD3//SCX//MC4R//HTR2A//TPD52L1//PLAGL1//BHLHA15//ITGA8 |
| GO:0032944 | regulation of mononuclear cell proliferation | Biological process | 3 | 146 | 72 | 13692 | 3.90753424657534 | 0.041550026893294 | 0.390891240129186 | 1.38142869078227 | EFNB1//TAC1//TACR1 |
| GO:0034764 | positive regulation of transmembrane transport | Biological process | 2 | 62 | 72 | 13692 | 6.13440860215054 | 0.0421117390941395 | 0.395327332309866 | 1.3755968230919 | TGFB3//TRPV2 |
| GO:0009306 | protein secretion | Biological process | 3 | 148 | 72 | 13692 | 3.85472972972973 | 0.0429765627653335 | 0.402583870006885 | 1.36676832201681 | DRD2//TGFB3//CBLN4 |
| GO:0006970 | response to osmotic stress | Biological process | 2 | 63 | 72 | 13692 | 6.03703703703704 | 0.0433476138092292 | 0.402618514702671 | 1.36303480448552 | SST//TACR3 |
| GO:0017015 | regulation of transforming growth factor beta receptor signaling pathway | Biological process | 2 | 63 | 72 | 13692 | 6.03703703703704 | 0.0433476138092292 | 0.402618514702671 | 1.36303480448552 | ITGA8//TGFB3 |
| GO:0030801 | positive regulation of cyclic nucleotide metabolic process | Biological process | 2 | 63 | 72 | 13692 | 6.03703703703704 | 0.0433476138092292 | 0.402618514702671 | 1.36303480448552 | CALCA//MC4R |
| GO:0035249 | synaptic transmission, glutamatergic | Biological process | 2 | 63 | 72 | 13692 | 6.03703703703704 | 0.0433476138092292 | 0.402618514702671 | 1.36303480448552 | DRD2//HTR2A |
| GO:0051924 | regulation of calcium ion transport | Biological process | 3 | 149 | 72 | 13692 | 3.82885906040268 | 0.0436990081902947 | 0.405024211218292 | 1.35952841983439 | CALCA//DRD2//TRPV2 |
| GO:0048675 | axon extension | Biological process | 2 | 64 | 72 | 13692 | 5.94270833333333 | 0.0445969720225029 | 0.411606579677164 | 1.35069462735423 | TRPV2//SLIT2 |
| GO:0090100 | positive regulation of transmembrane receptor protein serine/threonine kinase signaling pathway | Biological process | 2 | 64 | 72 | 13692 | 5.94270833333333 | 0.0445969720225029 | 0.411606579677164 | 1.35069462735423 | TGFB3//ITGA8 |
| GO:0045471 | response to ethanol | Biological process | 3 | 151 | 72 | 13692 | 3.77814569536424 | 0.0451621786150982 | 0.414489487138103 | 1.34522511595669 | DRD2//TACR1//HTR3A |
| GO:0070663 | regulation of leukocyte proliferation | Biological process | 3 | 151 | 72 | 13692 | 3.77814569536424 | 0.0451621786150982 | 0.414489487138103 | 1.34522511595669 | EFNB1//TAC1//TACR1 |
| GO:0015672 | monovalent inorganic cation transport | Biological process | 4 | 255 | 72 | 13692 | 2.98300653594771 | 0.0451929687162439 | 0.414489487138103 | 1.34492912902208 | DRD2//HTR2A//KCNN3//SCN11A |
| GO:0045981 | positive regulation of nucleotide metabolic process | Biological process | 2 | 65 | 72 | 13692 | 5.85128205128205 | 0.045859642148383 | 0.418851398288565 | 1.33856933847631 | CALCA//MC4R |
| GO:1900544 | positive regulation of purine nucleotide metabolic process | Biological process | 2 | 65 | 72 | 13692 | 5.85128205128205 | 0.045859642148383 | 0.418851398288565 | 1.33856933847631 | CALCA//MC4R |
| GO:0006650 | glycerophospholipid metabolic process | Biological process | 3 | 153 | 72 | 13692 | 3.72875816993464 | 0.0466496171959882 | 0.425180710576325 | 1.3311519156978 | HTR2C//HTR2A//DRD2 |
| GO:0097305 | response to alcohol | Biological process | 3 | 154 | 72 | 13692 | 3.70454545454545 | 0.0474023965404849 | 0.431145449032128 | 1.32419970098419 | DRD2//TACR1//HTR3A |
| GO:0051251 | positive regulation of lymphocyte activation | Biological process | 3 | 155 | 72 | 13692 | 3.68064516129032 | 0.048161193653785 | 0.437140109685701 | 1.31730275782108 | EFNB1//TAC1//TACR1 |
| GO:0050806 | positive regulation of synaptic transmission | Biological process | 2 | 67 | 72 | 13692 | 5.67661691542289 | 0.0484242387209157 | 0.438619550728294 | 1.31493719811333 | TAC1//TACR1 |
| GO:0002682 | regulation of immune system process | Biological process | 7 | 635 | 72 | 13692 | 2.09632545931759 | 0.0488442816004481 | 0.441512021724463 | 1.31118627359802 | KRT1//TACR1//SLIT2//TGFB3//EFNB1//CALCA//TAC1 |
| GO:0032970 | regulation of actin filament-based process | Biological process | 3 | 157 | 72 | 13692 | 3.63375796178344 | 0.0496967724428089 | 0.445113558655328 | 1.3036718156086 | SLIT2//TAC1//TACR1 |
