# Supplementary material for: Gene Expression in the Hippocampus in a Rat Model of Premenstrual Dysphoric Disorder After Treatment With Baixiangdan Capsules
Source: Front Psychol. 2018 Nov 13;9:2065. doi: 10.3389/fpsyg.2018.02065 (PMC6242977; doi:10.3389/fpsyg.2018.02065)
Supplement: Supplementary file 3 [file Data_Sheet_3.ZIP › Data Analysis Folder/GO Analysis Report/fluoxetine vs blank (up)/CC_result(Rat).html]

| GO.ID | Term | Ontology | Count | Pop.Hits | List.Total | Pop.Total | Fold.Enrichment | Pvalue | FDR | Enrichment.Score | GENES |
| --- | --- | --- | --- | --- | --- | --- | --- | --- | --- | --- | --- |
| GO:0005886 | plasma membrane | Cellular component | 37 | 2986 | 77 | 15288 | 2.4602082445351 | 1.50212460844602e-08 | 8.06640914735513e-06 | 7.82329403902571 | SCN11A//SCN9A//LCP1//NTNG1//DRD2//ADRA2A//HTR3A//CLDN3//HTR2C//LRP2//PTPRO//TGFB3//TRPV2//TACR3//ITGA8//TAC1//TACR1//SSTR1//TRHR//MC4R//CHRNE//DIO3//SLC7A3//HTR2A//KCNJ16//KCNN3//FXYD6//HRH3//P2RX2//GPR149//MYO16//KRT1//DOK3//GPR123//GPRC5A//KIRREL3//SLIT2 |
| GO:0071944 | cell periphery | Cellular component | 37 | 3081 | 77 | 15288 | 2.3843498273878 | 3.57943440559873e-08 | 9.61078137903259e-06 | 7.44618559177319 | DRD2//TAC1//TACR1//TACR3//SSTR1//ADRA2A//HTR2C//TRHR//MC4R//CHRNE//TRPV2//DIO3//SLC7A3//HTR2A//SCN11A//KCNJ16//PTPRO//KCNN3//FXYD6//HTR3A//HRH3//P2RX2//GPR149//MYO16//NTNG1//KRT1//LCP1//DOK3//GPR123//GPRC5A//KIRREL3//SLIT2//SCN9A//CLDN3//LRP2//TGFB3//ITGA8 |
| GO:0044297 | cell body | Cellular component | 13 | 441 | 77 | 15288 | 5.85281385281385 | 2.74977174883582e-07 | 4.92209143041612e-05 | 6.56070335429161 | TACR3//CALCA//SST//TAC1//TGFB3//SNCG//HTR3A//P2RX2//KLHL14//DRD2//ITGA8//TACR1//TRPV2 |
| GO:0043005 | neuron projection | Cellular component | 15 | 742 | 77 | 15288 | 4.01372212692967 | 3.5948492993024e-06 | 0.000482608518431347 | 5.44431931108171 | DRD2//P2RX2//ITGA8//CALCA//TAC1//TRPV2//SCN11A//SNCG//HTR3A//KIRREL3//TACR1//HTR2A//TACR3//PTPRO//KLHL14 |
| GO:0042995 | cell projection | Cellular component | 19 | 1211 | 77 | 15288 | 3.11508145034157 | 6.23291254714523e-06 | 0.000589616862874238 | 5.20530896657161 | LCP1//LRP2//DNAH8//DRD2//P2RX2//ITGA8//CALCA//KLHL14//TAC1//TRPV2//SCN11A//SNCG//HTR3A//KIRREL3//TACR1//HTR2A//TACR3//PTPRO//CAMP |
| GO:0043025 | neuronal cell body | Cellular component | 11 | 413 | 77 | 15288 | 5.28813559322034 | 6.58789790920936e-06 | 0.000589616862874238 | 5.18125313958889 | TACR3//DRD2//ITGA8//CALCA//SST//TAC1//TGFB3//SNCG//HTR3A//P2RX2//KLHL14 |
| GO:0030424 | axon | Cellular component | 9 | 334 | 77 | 15288 | 5.35002721829069 | 4.44805277365728e-05 | 0.00341229191350566 | 4.35183006873441 | CALCA//P2RX2//DRD2//SNCG//TRPV2//SCN11A//TAC1//HTR3A//KIRREL3 |
| GO:0044425 | membrane part | Cellular component | 45 | 5668 | 77 | 15288 | 1.57631359466222 | 0.000109121569755461 | 0.00732478536983532 | 3.96208939515065 | DRD2//EPOR//TACR1//TACR3//SSTR1//ADRA2A//EFNB1//HTR2C//TRHR//MC4R//LRP2//CHRNE//TRPV2//DIO3//SLC7A3//HTR2A//KCNJ16//KCNN3//FXYD6//KCNK13//CLDN3//TPBG//HRH3//P2RX2//HRK//SLC6A5//GPR149//IGSF1//GPR123//GALNT14//KIRREL3//SLIT2//OLR278//LOC500413//SCN11A//SCN9A//LCP1//NTNG1//HTR3A//MX1//PTPRO//AP1S2//TGFB3//ITGA8//RFTN1 |
| GO:0044463 | cell projection part | Cellular component | 11 | 636 | 77 | 15288 | 3.43396226415094 | 0.000325239096086376 | 0.0187541269893424 | 3.48779725460926 | DRD2//P2RX2//ITGA8//LRP2//TRPV2//TACR3//CALCA//PTPRO//HTR2A//KIRREL3//SNCG |
| GO:0016020 | membrane | Cellular component | 51 | 7097 | 77 | 15288 | 1.42677443734228 | 0.000349238863861125 | 0.0187541269893424 | 3.45687743329043 | DRD2//TAC1//TACR1//TACR3//SSTR1//ADRA2A//HTR2C//TRHR//MC4R//CHRNE//TRPV2//DIO3//SLC7A3//HTR2A//SCN11A//KCNJ16//PTPRO//KCNN3//FXYD6//HTR3A//HRH3//P2RX2//GPR149//MYO16//NTNG1//KRT1//LCP1//DOK3//GPR123//GPRC5A//KIRREL3//SLIT2//EPOR//EFNB1//LRP2//KCNK13//CLDN3//TPBG//HRK//SLC6A5//IGSF1//GALNT14//OLR278//LOC500413//SCN9A//MX1//AP1S2//TGFB3//ITGA8//RFTN1//SYT17 |
| GO:0031224 | intrinsic to membrane | Cellular component | 38 | 4750 | 77 | 15288 | 1.58836363636364 | 0.000596162789975191 | 0.0291035834742434 | 3.22463513432949 | DRD2//EPOR//TACR1//TACR3//SSTR1//ADRA2A//EFNB1//HTR2C//TRHR//MC4R//LRP2//CHRNE//TRPV2//DIO3//SLC7A3//HTR2A//KCNJ16//KCNN3//FXYD6//KCNK13//CLDN3//TPBG//HRH3//P2RX2//HRK//SLC6A5//GPR149//IGSF1//GPR123//GALNT14//KIRREL3//SLIT2//OLR278//LOC500413//SCN11A//SCN9A//NTNG1//HTR3A |
| GO:0016021 | integral to membrane | Cellular component | 37 | 4653 | 77 | 15288 | 1.5788054627513 | 0.000859967943133854 | 0.03848356545524 | 3.06551773756987 | SCN11A//SCN9A//DRD2//ADRA2A//HTR3A//EPOR//TACR1//TACR3//SSTR1//EFNB1//HTR2C//TRHR//MC4R//LRP2//CHRNE//TRPV2//DIO3//SLC7A3//HTR2A//KCNJ16//KCNN3//FXYD6//KCNK13//CLDN3//TPBG//HRH3//P2RX2//HRK//SLC6A5//GPR149//IGSF1//GPR123//GALNT14//KIRREL3//SLIT2//OLR278//LOC500413 |
| GO:0043679 | axon terminus | Cellular component | 4 | 88 | 77 | 15288 | 9.02479338842975 | 0.00100705404708342 | 0.0415990787141382 | 2.99694722088609 | CALCA//P2RX2//DRD2//SNCG |
| GO:0045202 | synapse | Cellular component | 9 | 527 | 77 | 15288 | 3.39071933758841 | 0.00128865163086184 | 0.0494289946980577 | 2.88986447228411 | SYT17//DRD2//P2RX2//ITGA8//CALCA//CHRNE//HTR3A//ADRA2A//EFNB1 |
| GO:0044306 | neuron projection terminus | Cellular component | 4 | 97 | 77 | 15288 | 8.18744142455483 | 0.0014461500505744 | 0.0517721718105635 | 2.83978664289517 | CALCA//P2RX2//DRD2//SNCG |
| GO:0033267 | axon part | Cellular component | 5 | 170 | 77 | 15288 | 5.83957219251337 | 0.00166081016952112 | 0.0557409413145526 | 2.7796800045389 | CALCA//P2RX2//DRD2//SNCG//TRPV2 |
| GO:0005623 | cell | Cellular component | 70 | 11848 | 77 | 15288 | 1.17304032901602 | 0.00179506144984802 | 0.0562088056277859 | 2.74592067974688 | DRD2//TAC1//TACR1//TACR3//SSTR1//ADRA2A//HTR2C//TRHR//MC4R//CHRNE//TRPV2//DIO3//SLC7A3//HTR2A//SCN11A//KCNJ16//PTPRO//KCNN3//FXYD6//HTR3A//HRH3//P2RX2//GPR149//MYO16//NTNG1//KRT1//LCP1//DOK3//GPR123//GPRC5A//KIRREL3//SLIT2//CALCA//PLAGL1//BHLHA15//ASB2//ARHGAP8//ZFP575//PLCXD3//RGD1565844//SMARCD3//NEDD9//SCN9A//HRK//DUSP26//HSD11B2//LRP2//TPBG//KLHL14//RPS4Y2//MX1//NPPA//EFNB1//TGFB3//PNCK//NR2F2//SCX//SNCG//NNAT//DNAH8//CAMP//TPD52L1//LOC500413//GALNT14//SYT17//CLDN3//ITGA8//AP1S2//SST//CBLN4 |
| GO:0001518 | voltage-gated sodium channel complex | Cellular component | 2 | 13 | 77 | 15288 | 30.5454545454546 | 0.00188409404338947 | 0.0562088056277859 | 2.72489742346152 | SCN11A//SCN9A |
| GO:0034706 | sodium channel complex | Cellular component | 2 | 16 | 77 | 15288 | 24.8181818181818 | 0.00287037858720669 | 0.0811259632278944 | 2.54206081842581 | SCN11A//SCN9A |
| GO:0044456 | synapse part | Cellular component | 7 | 391 | 77 | 15288 | 3.55452220413857 | 0.00350742526711008 | 0.0941743684219057 | 2.4550115737358 | SYT17//DRD2//P2RX2//ITGA8//CALCA//CHRNE//HTR3A |
| GO:0044459 | plasma membrane part | Cellular component | 15 | 1426 | 77 | 15288 | 2.0884865485146 | 0.00453878207268527 | 0.11456253755931 | 2.34306066918541 | SCN11A//SCN9A//LCP1//NTNG1//DRD2//ADRA2A//HTR3A//CLDN3//HTR2C//LRP2//PTPRO//TGFB3//TRPV2//TACR3//ITGA8 |
| GO:0044464 | cell part | Cellular component | 69 | 11835 | 77 | 15288 | 1.15755271344625 | 0.00469343729293263 | 0.11456253755931 | 2.32850898018766 | DRD2//TAC1//TACR1//TACR3//SSTR1//ADRA2A//HTR2C//TRHR//MC4R//CHRNE//TRPV2//DIO3//SLC7A3//HTR2A//SCN11A//KCNJ16//PTPRO//KCNN3//FXYD6//HTR3A//HRH3//P2RX2//GPR149//MYO16//NTNG1//KRT1//LCP1//DOK3//GPR123//GPRC5A//KIRREL3//SLIT2//CALCA//PLAGL1//BHLHA15//ASB2//ARHGAP8//ZFP575//PLCXD3//RGD1565844//SMARCD3//NEDD9//SCN9A//HRK//DUSP26//HSD11B2//LRP2//TPBG//KLHL14//RPS4Y2//MX1//NPPA//EFNB1//TGFB3//PNCK//NR2F2//SCX//SNCG//NNAT//DNAH8//CAMP//TPD52L1//LOC500413//GALNT14//SYT17//CLDN3//ITGA8//AP1S2//SST |
| GO:0030425 | dendrite | Cellular component | 7 | 426 | 77 | 15288 | 3.26248399487836 | 0.00558435037235364 | 0.130382441302344 | 2.25302734110013 | DRD2//P2RX2//ITGA8//PTPRO//HTR2A//KIRREL3//TACR1 |
| GO:0016328 | lateral plasma membrane | Cellular component | 2 | 33 | 77 | 15288 | 12.0330578512397 | 0.0119508104150143 | 0.267399383035945 | 1.92260264309786 | DRD2//PTPRO |
| GO:0032589 | neuron projection membrane | Cellular component | 2 | 36 | 77 | 15288 | 11.030303030303 | 0.0141216515365255 | 0.303333075004568 | 1.85011450928588 | TACR3//ITGA8 |
| GO:0043197 | dendritic spine | Cellular component | 4 | 192 | 77 | 15288 | 4.13636363636364 | 0.0160153762872475 | 0.318528039490811 | 1.79546285296923 | DRD2//P2RX2//ITGA8//PTPRO |
| GO:0044309 | neuron spine | Cellular component | 4 | 192 | 77 | 15288 | 4.13636363636364 | 0.0160153762872475 | 0.318528039490811 | 1.79546285296923 | DRD2//P2RX2//ITGA8//PTPRO |
| GO:0043198 | dendritic shaft | Cellular component | 2 | 43 | 77 | 15288 | 9.23467230443975 | 0.019788285518771 | 0.379511047270715 | 1.70359183196805 | HTR2A//KIRREL3 |
| GO:0005615 | extracellular space | Cellular component | 8 | 707 | 77 | 15288 | 2.24662466246625 | 0.0255616323340423 | 0.473330915978645 | 1.59241141612273 | CALCA//SST//TAC1//TGFB3//LRP2//CAMP//SLIT2//CBLN4 |
| GO:0043195 | terminal button | Cellular component | 2 | 52 | 77 | 15288 | 7.63636363636364 | 0.0282274051640339 | 0.505270552436207 | 1.54932904302689 | CALCA//P2RX2 |
| GO:0019861 | flagellum | Cellular component | 2 | 56 | 77 | 15288 | 7.09090909090909 | 0.0323642298324455 | 0.527796670772578 | 1.48993472337489 | DNAH8//DRD2 |
| GO:0014069 | postsynaptic density | Cellular component | 3 | 138 | 77 | 15288 | 4.31620553359684 | 0.0324344322821137 | 0.527796670772578 | 1.4889936993648 | DRD2//P2RX2//ITGA8 |
| GO:0044327 | dendritic spine head | Cellular component | 3 | 138 | 77 | 15288 | 4.31620553359684 | 0.0324344322821137 | 0.527796670772578 | 1.4889936993648 | DRD2//P2RX2//ITGA8 |
| GO:0031226 | intrinsic to plasma membrane | Cellular component | 6 | 480 | 77 | 15288 | 2.48181818181818 | 0.0338467889609204 | 0.534580166823949 | 1.47048252646779 | SCN11A//SCN9A//NTNG1//DRD2//ADRA2A//HTR3A |
| GO:0009986 | cell surface | Cellular component | 6 | 511 | 77 | 15288 | 2.33125778331258 | 0.0437523853902367 | 0.671286598701632 | 1.35899826415993 | HTR2C//TACR1//TRHR//TGFB3//TRPV2//SLIT2 |
| GO:0030139 | endocytic vesicle | Cellular component | 2 | 69 | 77 | 15288 | 5.75494071146245 | 0.0472920570540519 | 0.67819143577361 | 1.3252117951452 | DRD2//LRP2 |
| GO:0031253 | cell projection membrane | Cellular component | 3 | 161 | 77 | 15288 | 3.699604743083 | 0.04764184781978 | 0.67819143577361 | 1.32201140247114 | LRP2//TACR3//ITGA8 |
| GO:0005576 | extracellular region | Cellular component | 12 | 1410 | 77 | 15288 | 1.68974854932302 | 0.048786882097494 | 0.67819143577361 | 1.31169693616364 | CALCA//SST//TAC1//TGFB3//LRP2//CAMP//SLIT2//CBLN4//NPPA//NPW//IGSF1//KIRREL3 |
| GO:0042581 | specific granule | Cellular component | 1 | 10 | 77 | 15288 | 19.8545454545455 | 0.0492541266204298 | 0.67819143577361 | 1.30755737748305 | CAMP |
