# Supplementary material for: Gene Expression in the Hippocampus in a Rat Model of Premenstrual Dysphoric Disorder After Treatment With Baixiangdan Capsules
Source: Front Psychol. 2018 Nov 13;9:2065. doi: 10.3389/fpsyg.2018.02065 (PMC6242977; doi:10.3389/fpsyg.2018.02065)
Supplement: Supplementary file 3 [file Data_Sheet_3.ZIP › Data Analysis Folder/GO Analysis Report/fluoxetine vs blank (up)/MF_result(Rat).html]

| GO.ID | Term | Ontology | Count | Pop.Hits | List.Total | Pop.Total | Fold.Enrichment | Pvalue | FDR | Enrichment.Score | GENES |
| --- | --- | --- | --- | --- | --- | --- | --- | --- | --- | --- | --- |
| GO:0008227 | G-protein coupled amine receptor activity | Molecular function | 6 | 56 | 76 | 14392 | 20.2894736842105 | 4.67212187709817e-07 | 0.000419089332375706 | 6.33048583673024 | DRD2//ADRA2A//HRH3//HTR2C//HTR2A//HTR3A |
| GO:0043176 | amine binding | Molecular function | 5 | 39 | 76 | 14392 | 24.2780026990553 | 1.79848498097526e-06 | 0.000806620513967404 | 5.74509318459376 | DRD2//HTR2C//HTR2A//HTR3A//ADRA2A |
| GO:0005261 | cation channel activity | Molecular function | 9 | 254 | 76 | 14392 | 6.70990468296726 | 7.32769735624072e-06 | 0.00219098150951598 | 5.1350324759162 | CHRNE//P2RX2//HTR3A//KCNJ16//SCN11A//SCN9A//TRPV2//KCNK13//KCNN3 |
| GO:0005216 | ion channel activity | Molecular function | 10 | 348 | 76 | 14392 | 5.44162129461585 | 1.38900172298394e-05 | 0.00258940801267091 | 4.85729721554248 | CHRNE//P2RX2//HTR3A//KCNJ16//KCNK13//SCN11A//SCN9A//TRPV2//KCNN3//FXYD6 |
| GO:0051378 | serotonin binding | Molecular function | 3 | 10 | 76 | 14392 | 56.8105263157895 | 1.65360733551347e-05 | 0.00258940801267091 | 4.78156760981693 | HTR2C//HTR2A//HTR3A |
| GO:0022838 | substrate-specific channel activity | Molecular function | 10 | 357 | 76 | 14392 | 5.30443756449948 | 1.73204549342536e-05 | 0.00258940801267091 | 4.76144070511075 | CHRNE//P2RX2//TRPV2//FXYD6//HTR3A//KCNJ16//KCNK13//SCN11A//SCN9A//KCNN3 |
| GO:0015267 | channel activity | Molecular function | 10 | 375 | 76 | 14392 | 5.04982456140351 | 2.64183651162168e-05 | 0.00296215918865581 | 4.57809406192871 | CHRNE//P2RX2//TRPV2//FXYD6//HTR3A//KCNJ16//KCNK13//SCN11A//SCN9A//KCNN3 |
| GO:0022803 | passive transmembrane transporter activity | Molecular function | 10 | 375 | 76 | 14392 | 5.04982456140351 | 2.64183651162168e-05 | 0.00296215918865581 | 4.57809406192871 | CHRNE//P2RX2//TRPV2//FXYD6//HTR3A//KCNJ16//KCNK13//SCN11A//SCN9A//KCNN3 |
| GO:0004993 | serotonin receptor activity | Molecular function | 3 | 14 | 76 | 14392 | 40.578947368421 | 4.94016285176185e-05 | 0.00492369564225598 | 4.3062587343849 | HTR2C//HTR2A//HTR3A |
| GO:0008324 | cation transmembrane transporter activity | Molecular function | 11 | 507 | 76 | 14392 | 4.10858507214783 | 6.70749845598689e-05 | 0.00601662611502024 | 4.17343941862717 | CHRNE//P2RX2//HTR3A//KCNJ16//SCN11A//SCN9A//TRPV2//KCNK13//SLC6A5//KCNN3//SLC7A3 |
| GO:0022836 | gated channel activity | Molecular function | 8 | 271 | 76 | 14392 | 5.5902116915906 | 8.88082473982425e-05 | 0.00724190890147487 | 4.05154670051391 | CHRNE//P2RX2//HTR3A//KCNJ16//KCNK13//SCN11A//SCN9A//KCNN3 |
| GO:0015075 | ion transmembrane transporter activity | Molecular function | 12 | 648 | 76 | 14392 | 3.50682261208577 | 0.00013812962920959 | 0.0103251897834169 | 3.85971315399169 | CHRNE//P2RX2//TRPV2//FXYD6//HTR3A//KCNJ16//KCNK13//SCN11A//SCN9A//SLC6A5//KCNN3//SLC7A3 |
| GO:0022892 | substrate-specific transporter activity | Molecular function | 14 | 880 | 76 | 14392 | 3.01267942583732 | 0.000181032866487026 | 0.0124912677876048 | 3.74224257188121 | SLC7A3//CHRNE//P2RX2//TRPV2//FXYD6//HTR3A//KCNJ16//KCNK13//SCN11A//SCN9A//SLC6A5//AP1S2//KCNN3//LRP2 |
| GO:0004629 | phospholipase C activity | Molecular function | 3 | 25 | 76 | 14392 | 22.7242105263158 | 0.000299374267695252 | 0.0185323883005901 | 3.52378553157556 | HTR2C//HTR2A//PLCXD3 |
| GO:0008144 | drug binding | Molecular function | 5 | 112 | 76 | 14392 | 8.45394736842105 | 0.000310599891108198 | 0.0185323883005901 | 3.50779870086476 | DRD2//HTR2C//HTR2A//HRH3//P2RX2 |
| GO:0005215 | transporter activity | Molecular function | 15 | 1052 | 76 | 14392 | 2.70012007204323 | 0.000340546955380533 | 0.0185323883005901 | 3.46782299812207 | SLC7A3//CHRNE//P2RX2//TRPV2//FXYD6//HTR3A//KCNJ16//KCNK13//SCN11A//SCN9A//SLC6A5//AP1S2//KCNN3//LRP2//SYT17 |
| GO:0042802 | identical protein binding | Molecular function | 13 | 827 | 76 | 14392 | 2.976770826704 | 0.000356517866515608 | 0.0185323883005901 | 3.44791870105837 | ADRA2A//BHLHA15//TRHR//PTPRO//NR2F2//SLIT2//TPD52L1//EPOR//TGFB3//KCNN3//CLDN3//P2RX2//LCP1 |
| GO:0030594 | neurotransmitter receptor activity | Molecular function | 4 | 65 | 76 | 14392 | 11.6534412955466 | 0.000380919490237244 | 0.0185323883005901 | 3.41916680553298 | DRD2//SSTR1//TACR3//TACR1 |
| GO:0046873 | metal ion transmembrane transporter activity | Molecular function | 8 | 337 | 76 | 14392 | 4.4953927846322 | 0.000392547801238809 | 0.0185323883005901 | 3.40610745089271 | KCNJ16//SCN11A//SCN9A//HTR3A//TRPV2//KCNK13//SLC6A5//KCNN3 |
| GO:0022891 | substrate-specific transmembrane transporter activity | Molecular function | 12 | 772 | 76 | 14392 | 2.94355058631034 | 0.000680853258843436 | 0.0305362686591281 | 3.16694647948467 | SLC7A3//CHRNE//P2RX2//TRPV2//FXYD6//HTR3A//KCNJ16//KCNK13//SCN11A//SCN9A//SLC6A5//KCNN3 |
| GO:0008188 | neuropeptide receptor activity | Molecular function | 3 | 40 | 76 | 14392 | 14.2026315789474 | 0.00121494210175186 | 0.0502682498180324 | 2.91544441794123 | SSTR1//TACR3//TACR1 |
| GO:0015077 | monovalent inorganic cation transmembrane transporter activity | Molecular function | 7 | 310 | 76 | 14392 | 4.27606112054329 | 0.00123288907023045 | 0.0502682498180324 | 2.90907599749516 | KCNJ16//SCN11A//SCN9A//HTR3A//KCNK13//SLC6A5//KCNN3 |
| GO:0022857 | transmembrane transporter activity | Molecular function | 12 | 839 | 76 | 14392 | 2.70848754783263 | 0.00140247612724443 | 0.0546965689625328 | 2.85310452250977 | SLC7A3//CHRNE//P2RX2//TRPV2//FXYD6//HTR3A//KCNJ16//KCNK13//SCN11A//SCN9A//SLC6A5//KCNN3 |
| GO:0005244 | voltage-gated ion channel activity | Molecular function | 5 | 165 | 76 | 14392 | 5.7384370015949 | 0.00178759760117667 | 0.0641390019302189 | 2.74773023680225 | KCNJ16//SCN11A//SCN9A//HTR3A//KCNK13 |
| GO:0022832 | voltage-gated channel activity | Molecular function | 5 | 165 | 76 | 14392 | 5.7384370015949 | 0.00178759760117667 | 0.0641390019302189 | 2.74773023680225 | KCNJ16//KCNK13//SCN11A//SCN9A//HTR3A |
| GO:0022890 | inorganic cation transmembrane transporter activity | Molecular function | 8 | 429 | 76 | 14392 | 3.53134584713532 | 0.00187115355654065 | 0.0645133860635582 | 2.72789057058562 | KCNJ16//SCN11A//SCN9A//HTR3A//TRPV2//KCNK13//SLC6A5//KCNN3 |
| GO:0005231 | excitatory extracellular ligand-gated ion channel activity | Molecular function | 3 | 47 | 76 | 14392 | 12.0873460246361 | 0.0019418744968964 | 0.0645133860635582 | 2.71177884191857 | CHRNE//P2RX2//HTR3A |
| GO:0005267 | potassium channel activity | Molecular function | 4 | 110 | 76 | 14392 | 6.88612440191388 | 0.00271647841114662 | 0.0854873406150528 | 2.56599374211458 | KCNJ16//HTR3A//KCNN3//KCNK13 |
| GO:0005248 | voltage-gated sodium channel activity | Molecular function | 2 | 15 | 76 | 14392 | 25.2491228070175 | 0.00276380476904853 | 0.0854873406150528 | 2.55849263811386 | SCN11A//SCN9A |
| GO:0008528 | G-protein coupled peptide receptor activity | Molecular function | 4 | 114 | 76 | 14392 | 6.64450600184672 | 0.00309047583515831 | 0.0924052274712335 | 2.50997464786521 | MC4R//SSTR1//TACR3//TACR1 |
| GO:0001653 | peptide receptor activity | Molecular function | 4 | 116 | 76 | 14392 | 6.52994555353902 | 0.00329004855470917 | 0.0950885946665669 | 2.48279769266198 | MC4R//SSTR1//TACR3//TACR1 |
| GO:0015276 | ligand-gated ion channel activity | Molecular function | 4 | 118 | 76 | 14392 | 6.4192685102587 | 0.00349824261315129 | 0.0950885946665669 | 2.45615007424583 | CHRNE//P2RX2//HTR3A//KCNJ16 |
| GO:0022834 | ligand-gated channel activity | Molecular function | 4 | 118 | 76 | 14392 | 6.4192685102587 | 0.00349824261315129 | 0.0950885946665669 | 2.45615007424583 | CHRNE//P2RX2//HTR3A//KCNJ16 |
| GO:0022843 | voltage-gated cation channel activity | Molecular function | 4 | 125 | 76 | 14392 | 6.05978947368421 | 0.0042973505839814 | 0.110629586727026 | 2.36679921450838 | KCNJ16//SCN11A//SCN9A//HTR3A |
| GO:0015079 | potassium ion transmembrane transporter activity | Molecular function | 4 | 126 | 76 | 14392 | 6.01169590643275 | 0.0044207401381867 | 0.110629586727026 | 2.35450501322543 | KCNJ16//HTR3A//KCNK13//KCNN3 |
| GO:0004435 | phosphatidylinositol phospholipase C activity | Molecular function | 2 | 19 | 76 | 14392 | 19.9335180055402 | 0.0044399834137937 | 0.110629586727026 | 2.3526186522528 | HTR2C//HTR2A |
| GO:0005230 | extracellular ligand-gated ion channel activity | Molecular function | 3 | 66 | 76 | 14392 | 8.60765550239234 | 0.00510126719225629 | 0.123671261390646 | 2.29232192855987 | CHRNE//P2RX2//HTR3A |
| GO:0005515 | protein binding | Molecular function | 35 | 4586 | 76 | 14392 | 1.4452452544357 | 0.00661667704701315 | 0.156188402925547 | 2.17936006229202 | NPW//HTR2A//MYO16//LCP1//CALCA//DRD2//NPPA//SMARCD3//EFNB1//TGFB3//DOK3//SST//PNCK//KCNN3//EPOR//CLDN3//P2RX2//TPD52L1//LRP2//PTPRO//ADRA2A//MC4R//TAC1//IGSF1//SLIT2//BHLHA15//TRHR//NR2F2//SNCG//SCX//HTR2C//TRPV2//SCN11A//KCNJ16//HRK |
| GO:0004620 | phospholipase activity | Molecular function | 3 | 76 | 76 | 14392 | 7.47506925207756 | 0.00754787115498198 | 0.173601036564586 | 2.12217552201505 | HTR2C//HTR2A//PLCXD3 |
| GO:0005102 | receptor binding | Molecular function | 12 | 1052 | 76 | 14392 | 2.16009605763458 | 0.00873406532196379 | 0.195861414845038 | 2.05878356431961 | NPW//EFNB1//TGFB3//DOK3//NPPA//SST//CALCA//SMARCD3//ADRA2A//TAC1//DRD2//SLIT2 |
| GO:0008081 | phosphoric diester hydrolase activity | Molecular function | 3 | 81 | 76 | 14392 | 7.01364522417154 | 0.00899025844075101 | 0.196689312715943 | 2.04622782353053 | HTR2C//HTR2A//PLCXD3 |
| GO:0005272 | sodium channel activity | Molecular function | 2 | 31 | 76 | 14392 | 12.2173174872666 | 0.0115903080556915 | 0.247535864903697 | 1.93590502088636 | SCN11A//SCN9A |
| GO:0016298 | lipase activity | Molecular function | 3 | 92 | 76 | 14392 | 6.17505720823799 | 0.0126983982116913 | 0.264894492927607 | 1.89625105791779 | HTR2C//HTR2A//PLCXD3 |
| GO:0004930 | G-protein coupled receptor activity | Molecular function | 15 | 1569 | 76 | 14392 | 1.81040555499648 | 0.0160343596660957 | 0.326882286829269 | 1.7949483789868 | DRD2//ADRA2A//SLIT2//HRH3//MC4R//HTR2C//HTR2A//HTR3A//SSTR1//TACR3//TRHR//TACR1//GPR149//GPR123//OLR278 |
| GO:0005179 | hormone activity | Molecular function | 3 | 108 | 76 | 14392 | 5.26023391812865 | 0.0194466644068465 | 0.383495209517176 | 1.71115488040159 | CALCA//NPPA//SST |
| GO:0001664 | G-protein coupled receptor binding | Molecular function | 4 | 195 | 76 | 14392 | 3.88448043184885 | 0.0196664210008808 | 0.383495209517176 | 1.70627466809251 | ADRA2A//CALCA//TAC1//NPW |
| GO:0038023 | signaling receptor activity | Molecular function | 19 | 2232 | 76 | 14392 | 1.61200716845878 | 0.0210109400902966 | 0.399710305421233 | 1.67755451556671 | DRD2//P2RX2//NR2F2//EPOR//GPR149//GPR123//OLR278//ADRA2A//SLIT2//HRH3//MC4R//HTR2C//HTR2A//HTR3A//SSTR1//TACR3//TRHR//IGSF1//TACR1 |
| GO:0015081 | sodium ion transmembrane transporter activity | Molecular function | 3 | 112 | 76 | 14392 | 5.07236842105263 | 0.0213891802232098 | 0.399710305421233 | 1.66980586018578 | SCN11A//SCN9A//SLC6A5 |
| GO:0043178 | alcohol binding | Molecular function | 2 | 44 | 76 | 14392 | 8.60765550239234 | 0.0225634659272376 | 0.41304957013739 | 1.64659418848922 | DRD2//ADRA2A |
| GO:0042578 | phosphoric ester hydrolase activity | Molecular function | 5 | 309 | 76 | 14392 | 3.06421393289048 | 0.0236503608232708 | 0.417759835702969 | 1.62616222903357 | HTR2C//HTR2A//PLCXD3//PTPRO//DUSP26 |
| GO:0004872 | receptor activity | Molecular function | 21 | 2579 | 76 | 14392 | 1.54196853125446 | 0.0237522314613728 | 0.417759835702969 | 1.62429558326705 | DRD2//P2RX2//NR2F2//EPOR//GPR149//GPR123//OLR278//ADRA2A//SLIT2//HRH3//MC4R//HTR2C//HTR2A//HTR3A//SSTR1//TACR3//TRHR//IGSF1//TACR1//LRP2//CHRNE |
| GO:0042803 | protein homodimerization activity | Molecular function | 7 | 558 | 76 | 14392 | 2.37558951141294 | 0.0276832916733348 | 0.477536781365025 | 1.55778227149231 | ADRA2A//BHLHA15//TRHR//PTPRO//NR2F2//SLIT2//TPD52L1 |
| GO:0046983 | protein dimerization activity | Molecular function | 9 | 839 | 76 | 14392 | 2.03136566087447 | 0.0321328553289148 | 0.543833419434652 | 1.49305068128028 | ADRA2A//BHLHA15//TRHR//PTPRO//NR2F2//SLIT2//TPD52L1//TGFB3//SCX |
| GO:0004871 | signal transducer activity | Molecular function | 19 | 2433 | 76 | 14392 | 1.47883271681052 | 0.0464244935717028 | 0.747682923829868 | 1.33325282512646 | DRD2//P2RX2//NR2F2//EPOR//GPR149//GPR123//OLR278//ADRA2A//SLIT2//HRH3//MC4R//HTR2C//HTR2A//HTR3A//SSTR1//TACR3//TRHR//IGSF1//TACR1 |
| GO:0060089 | molecular transducer activity | Molecular function | 19 | 2433 | 76 | 14392 | 1.47883271681052 | 0.0464244935717028 | 0.747682923829868 | 1.33325282512646 | DRD2//P2RX2//NR2F2//EPOR//GPR149//GPR123//OLR278//ADRA2A//SLIT2//HRH3//MC4R//HTR2C//HTR2A//HTR3A//SSTR1//TACR3//TRHR//IGSF1//TACR1 |
| GO:0015171 | amino acid transmembrane transporter activity | Molecular function | 2 | 66 | 76 | 14392 | 5.7384370015949 | 0.0475116239222993 | 0.747682923829868 | 1.3232001253703 | SLC7A3//SLC6A5 |
| GO:0051015 | actin filament binding | Molecular function | 2 | 66 | 76 | 14392 | 5.7384370015949 | 0.0475116239222993 | 0.747682923829868 | 1.3232001253703 | MYO16//LCP1 |
