# Supplementary material for: Gene Expression in the Hippocampus in a Rat Model of Premenstrual Dysphoric Disorder After Treatment With Baixiangdan Capsules
Source: Front Psychol. 2018 Nov 13;9:2065. doi: 10.3389/fpsyg.2018.02065 (PMC6242977; doi:10.3389/fpsyg.2018.02065)
Supplement: Supplementary file 3 [file Data_Sheet_3.ZIP › Data Analysis Folder/GO Analysis Report/fluoxetine vs model (down)/BP_result(Rat).html]

| GO.ID | Term | Ontology | Count | Pop.Hits | List.Total | Pop.Total | Fold.Enrichment | Pvalue | FDR | Enrichment.Score | GENES |
| --- | --- | --- | --- | --- | --- | --- | --- | --- | --- | --- | --- |
| GO:0040011 | locomotion | Biological process | 18 | 906 | 75 | 13692 | 3.62701986754967 | 1.31161932448682e-06 | 0.00575013911855022 | 5.88219219336272 | ROBO3//XCL1//C3//ENPP2//NTRK1//EPHA5//LMX1A//LAMA2//BMP4//AQP1//ACE//MDK//MMP14//VAV3//SNCA//FOXJ1//CCDC40//LPAR1 |
| GO:0006935 | chemotaxis | Biological process | 10 | 312 | 75 | 13692 | 5.85128205128205 | 7.30244782420802e-06 | 0.01097551095014 | 5.13653153736564 | XCL1//NTRK1//EPHA5//LMX1A//LAMA2//ROBO3//BMP4//LPAR1//C3//ENPP2 |
| GO:0042330 | taxis | Biological process | 10 | 313 | 75 | 13692 | 5.83258785942492 | 7.51061424507753e-06 | 0.01097551095014 | 5.12432454337679 | XCL1//C3//ENPP2//NTRK1//EPHA5//LMX1A//LAMA2//ROBO3//BMP4//LPAR1 |
| GO:0006725 | cellular aromatic compound metabolic process | Biological process | 8 | 198 | 75 | 13692 | 7.37616161616162 | 1.22245663697479e-05 | 0.0133981247412437 | 4.91276653724039 | SLC5A5//MED1//PON3//UGT1A6//PON1//SNCA//HDC//FOLR1 |
| GO:0065008 | regulation of biological quality | Biological process | 26 | 2106 | 75 | 13692 | 2.25382716049383 | 2.85885893041807e-05 | 0.018357676312029 | 4.54380727425116 | SNCA//ACE//WFS1//AQP1//C3//F5//F12//SLC5A5//MED1//RGN//TRPV4//SLC4A4//NMB//MSX1//LAMA2//BMP4//BAIAP2L1//APOA2//LPAR1//C1QTNF3//SEMA7A//NTS//XCL1//EPHA5//SCN4B//SLCO1A5 |
| GO:0002712 | regulation of B cell mediated immunity | Biological process | 4 | 33 | 75 | 13692 | 22.1284848484849 | 3.01244868493239e-05 | 0.018357676312029 | 4.52108034227285 | C3//FOXJ1//CLCF1//XCL1 |
| GO:0002889 | regulation of immunoglobulin mediated immune response | Biological process | 4 | 33 | 75 | 13692 | 22.1284848484849 | 3.01244868493239e-05 | 0.018357676312029 | 4.52108034227285 | C3//FOXJ1//CLCF1//XCL1 |
| GO:0051179 | localization | Biological process | 35 | 3407 | 75 | 13692 | 1.87543293219841 | 3.34994093285201e-05 | 0.018357676312029 | 4.47496285050423 | SLC6A6//ROBO3//SNCA//XCL1//APOA2//AQP1//VAV3//SLC4A4//SLC5A5//SCN4B//TRPV4//SLC4A2//GULP1//MED1//BMP4//PON1//ACE//SLC16A8//SLCO1A5//FOLR1//MDK//MMP14//NMB//ENPP2//LAMA2//MSX1//MDFIC//KRT18//C3//FOXJ1//WFS1//CCDC40//EPHA5//LPAR1//CRB3 |
| GO:0070371 | ERK1 and ERK2 cascade | Biological process | 6 | 130 | 75 | 13692 | 8.42584615384615 | 7.68581208461673e-05 | 0.0320263363273366 | 4.11431023808813 | C3//BMP4//NTRK1//C1QTNF3//SEMA7A//MED1 |
| GO:0010033 | response to organic substance | Biological process | 22 | 1714 | 75 | 13692 | 2.343243873979 | 8.4390437647383e-05 | 0.0320263363273366 | 4.07370676086546 | MED1//SNCA//AQP1//MDK//MMP14//RERG//APOA2//MGST1//BMP4//UGT1A6//FOXJ1//WFS1//C3//ACE//KRT18//NTRK1//F12//EPHA5//MSX1//SLC5A5//XCL1//WNT10A |
| GO:0048518 | positive regulation of biological process | Biological process | 32 | 3107 | 75 | 13692 | 1.88024460894754 | 9.04726710147161e-05 | 0.0320263363273366 | 4.04348258786581 | BMP4//LPAR1//C3//XCL1//SNCA//F12//CLCF1//FOXJ1//MDFIC//NMB//AQP1//WNT10A//MED1//COL8A1//APOA2//PON1//NTRK1//EPHA5//MDK//SERTAD2//MSX1//BAIAP2L1//VAV3//TRPV4//WFS1//CAMKK2//LAMA2//ACE//S100A4//SEMA7A//C1QTNF3//SCN4B |
| GO:0044281 | small molecule metabolic process | Biological process | 21 | 1601 | 75 | 13692 | 2.39460337289194 | 9.46371556693812e-05 | 0.0320263363273366 | 4.02393832095468 | MGST1//PRPS2//AQP1//RERG//HDC//SLC5A5//MED1//SNCA//APOA2//PON1//ELOVL7//UGT1A6//RGN//EPHA5//NTRK1//FOXJ1//ENPP2//VAV3//C1QTNF3//PON3//FOLR1 |
| GO:0070374 | positive regulation of ERK1 and ERK2 cascade | Biological process | 5 | 84 | 75 | 13692 | 10.8666666666667 | 9.49686068100767e-05 | 0.0320263363273366 | 4.02241993305158 | C3//BMP4//NTRK1//C1QTNF3//SEMA7A |
| GO:0048878 | chemical homeostasis | Biological process | 14 | 807 | 75 | 13692 | 3.16708798017348 | 0.00010579970093497 | 0.0331304206356363 | 3.97551555992348 | WFS1//AQP1//RGN//TRPV4//SLC4A4//NMB//LAMA2//LPAR1//C1QTNF3//APOA2//XCL1//SNCA//SCN4B//EPHA5 |
| GO:0048583 | regulation of response to stimulus | Biological process | 23 | 1898 | 75 | 13692 | 2.2122655426765 | 0.000133919318629377 | 0.0391401528580793 | 3.87315676900027 | LPAR1//C3//XCL1//F12//FOXJ1//APOA2//MDFIC//SNCA//BMP4//EPHA5//MSX1//SOSTDC1//NTRK1//MED1//VAV3//CLCF1//S100A4//TRPV4//SEMA7A//ACE//MDK//RGN//C1QTNF3 |
| GO:0010647 | positive regulation of cell communication | Biological process | 13 | 733 | 75 | 13692 | 3.23776261937244 | 0.000155325259615878 | 0.0406011204791259 | 3.8087579118198 | BMP4//EPHA5//MSX1//LAMA2//MED1//LPAR1//CLCF1//S100A4//NTRK1//SNCA//C1QTNF3//C3//SEMA7A |
| GO:0023056 | positive regulation of signaling | Biological process | 13 | 734 | 75 | 13692 | 3.2333514986376 | 0.000157440476310479 | 0.0406011204791259 | 3.80288360502363 | SNCA//BMP4//EPHA5//MSX1//LAMA2//MED1//LPAR1//CLCF1//S100A4//NTRK1//C1QTNF3//C3//SEMA7A |
| GO:0070887 | cellular response to chemical stimulus | Biological process | 17 | 1182 | 75 | 13692 | 2.62565143824027 | 0.000175406104024993 | 0.0410581778190854 | 3.75595529752502 | MED1//XCL1//UGT1A6//PON3//WFS1//KRT18//SNCA//BMP4//EPHA5//AQP1//NTRK1//MSX1//SLC5A5//FOXJ1//MGST1//LPAR1//WNT10A |
| GO:0071363 | cellular response to growth factor stimulus | Biological process | 7 | 219 | 75 | 13692 | 5.83525114155251 | 0.000192038694378082 | 0.0410581778190854 | 3.71661125534983 | MED1//SNCA//XCL1//WNT10A//BMP4//NTRK1//FOXJ1 |
| GO:2000026 | regulation of multicellular organismal development | Biological process | 16 | 1078 | 75 | 13692 | 2.70961038961039 | 0.000197623476812222 | 0.0410581778190854 | 3.70416146438217 | BMP4//FOXJ1//NTRK1//TRPV4//LPAR1//MED1//MSX1//WNT10A//LMX1A//C3//AQP1//SEMA7A//LAMA2//CLCF1//ACE//EPHA5 |
| GO:0071702 | organic substance transport | Biological process | 10 | 462 | 75 | 13692 | 3.95151515151515 | 0.000201567051226684 | 0.0410581778190854 | 3.69558045754352 | SLC6A6//APOA2//GULP1//PON1//SNCA//SLCO1A5//AQP1//FOLR1//ACE//NMB |
| GO:0009719 | response to endogenous stimulus | Biological process | 15 | 987 | 75 | 13692 | 2.77446808510638 | 0.000251293667801166 | 0.0410581778190854 | 3.59981845479644 | MED1//AQP1//MDK//MMP14//RERG//MGST1//C3//BMP4//FOXJ1//UGT1A6//APOA2//SNCA//SLC5A5//XCL1//WNT10A |
| GO:0071310 | cellular response to organic substance | Biological process | 14 | 877 | 75 | 13692 | 2.91429874572406 | 0.000252704884722956 | 0.0410581778190854 | 3.59738636319397 | MED1//WFS1//UGT1A6//KRT18//BMP4//SNCA//EPHA5//NTRK1//MSX1//AQP1//SLC5A5//XCL1//FOXJ1//WNT10A |
| GO:0007275 | multicellular organismal development | Biological process | 33 | 3429 | 75 | 13692 | 1.75692038495188 | 0.000258132468927403 | 0.0410581778190854 | 3.58815736473501 | BMP4//MMP14//MED1//RPE65//MSX1//ROBO3//ACE//WFS1//UGT1A6//WNT10A//FOXJ1//C3//SOSTDC1//NTRK1//EPHA5//LMX1A//LAMA2//TRPV4//LPAR1//MDK//AQP1//CLCF1//PRPS2//APOA2//SLCO1A5//MGST1//FSCN2//SEMA7A//COL8A1//CCDC40//WIPF3//PRTG//ZAR1 |
| GO:0042481 | regulation of odontogenesis | Biological process | 3 | 23 | 75 | 13692 | 23.8121739130435 | 0.00025838367588021 | 0.0410581778190854 | 3.58773492759294 | BMP4//WNT10A//MSX1 |
| GO:0051960 | regulation of nervous system development | Biological process | 10 | 480 | 75 | 13692 | 3.80333333333333 | 0.000274042824771278 | 0.0410581778190854 | 3.56218156452057 | NTRK1//TRPV4//LPAR1//LMX1A//MED1//BMP4//SEMA7A//CLCF1//ACE//EPHA5 |
| GO:0051128 | regulation of cellular component organization | Biological process | 16 | 1110 | 75 | 13692 | 2.6314954954955 | 0.000275928438698205 | 0.0410581778190854 | 3.55920353644284 | LMX1A//AQP1//APOA2//NTRK1//TRPV4//LPAR1//MSX1//SERTAD2//RERG//BAIAP2L1//SNCA//EPHA5//SEMA7A//BMP4//C3//MMP14 |
| GO:0048870 | cell motility | Biological process | 13 | 779 | 75 | 13692 | 3.04657252888318 | 0.000282353916627935 | 0.0410581778190854 | 3.54920618363774 | ROBO3//XCL1//BMP4//AQP1//ACE//MDK//MMP14//VAV3//ENPP2//LAMA2//FOXJ1//CCDC40//LPAR1 |
| GO:0051674 | localization of cell | Biological process | 13 | 779 | 75 | 13692 | 3.04657252888318 | 0.000282353916627935 | 0.0410581778190854 | 3.54920618363774 | ROBO3//XCL1//BMP4//AQP1//ACE//MDK//MMP14//VAV3//ENPP2//LAMA2//FOXJ1//CCDC40//LPAR1 |
| GO:0042592 | homeostatic process | Biological process | 16 | 1125 | 75 | 13692 | 2.59640888888889 | 0.000321129391866054 | 0.0410581778190854 | 3.49331994313774 | WFS1//AQP1//RGN//TRPV4//SLC4A4//NMB//LAMA2//BMP4//LPAR1//C1QTNF3//APOA2//MED1//XCL1//SNCA//SCN4B//EPHA5 |
| GO:0042476 | odontogenesis | Biological process | 5 | 109 | 75 | 13692 | 8.3743119266055 | 0.000323199128406787 | 0.0410581778190854 | 3.49052981908753 | BMP4//MSX1//SOSTDC1//WNT10A//AQP1 |
| GO:0051239 | regulation of multicellular organismal process | Biological process | 21 | 1747 | 75 | 13692 | 2.19448196908987 | 0.000325010693096109 | 0.0410581778190854 | 3.48810235017116 | BMP4//SNCA//XCL1//APOA2//FOXJ1//AQP1//C3//NTRK1//TRPV4//LPAR1//F12//LAMA2//MED1//MSX1//WNT10A//LMX1A//SEMA7A//CLCF1//ACE//EPHA5//SCN4B |
| GO:0009605 | response to external stimulus | Biological process | 16 | 1128 | 75 | 13692 | 2.58950354609929 | 0.00033090494083159 | 0.0410581778190854 | 3.48029674822281 | C3//F12//XCL1//ENPP2//BMP4//MMP14//NTRK1//EPHA5//LMX1A//LAMA2//ROBO3//AQP1//ACE//SEMA7A//MED1//LPAR1 |
| GO:0065009 | regulation of molecular function | Biological process | 19 | 1493 | 75 | 13692 | 2.32326858673811 | 0.000331903948384655 | 0.0410581778190854 | 3.47898758112117 | BMP4//LPAR1//SNCA//MDFIC//MMP14//FOXJ1//CAMKK2//EPHA5//NTRK1//PON1//RGN//VAV3//AQP1//MSX1//WFS1//XCL1//APOA2//MED1//SCN4B |
| GO:0051050 | positive regulation of transport | Biological process | 10 | 492 | 75 | 13692 | 3.71056910569106 | 0.000333624020738137 | 0.0410581778190854 | 3.47674268795497 | SNCA//AQP1//PON1//NMB//MED1//C3//XCL1//WFS1//BMP4//SCN4B |
| GO:0006576 | cellular biogenic amine metabolic process | Biological process | 5 | 110 | 75 | 13692 | 8.29818181818182 | 0.000337156569682271 | 0.0410581778190854 | 3.47216837343667 | HDC//APOA2//ENPP2//SNCA//PON1 |
| GO:0032879 | regulation of localization | Biological process | 17 | 1252 | 75 | 13692 | 2.47884984025559 | 0.000348007219790445 | 0.0410868913051564 | 3.45841174604587 | SNCA//XCL1//APOA2//AQP1//BMP4//PON1//ACE//NMB//ENPP2//LAMA2//MED1//SCN4B//MDFIC//C3//WFS1//EPHA5//LPAR1 |
| GO:0002700 | regulation of production of molecular mediator of immune response | Biological process | 4 | 62 | 75 | 13692 | 11.778064516129 | 0.000364242728945641 | 0.0410868913051564 | 3.43860910890678 | CLCF1//XCL1//APOA2//SEMA7A |
| GO:0070372 | regulation of ERK1 and ERK2 cascade | Biological process | 5 | 112 | 75 | 13692 | 8.15 | 0.000366437170751896 | 0.0410868913051564 | 3.43600047868965 | C3//BMP4//NTRK1//C1QTNF3//SEMA7A |
| GO:0002702 | positive regulation of production of molecular mediator of immune response | Biological process | 3 | 26 | 75 | 13692 | 21.0646153846154 | 0.00037488039512004 | 0.0410868913051564 | 3.426107271005 | CLCF1//XCL1//SEMA7A |
| GO:0006874 | cellular calcium ion homeostasis | Biological process | 7 | 246 | 75 | 13692 | 5.19479674796748 | 0.000389526061317041 | 0.0416507866539977 | 3.40946348046241 | TRPV4//NMB//WFS1//XCL1//SNCA//LPAR1//RGN |
| GO:0002697 | regulation of immune effector process | Biological process | 6 | 177 | 75 | 13692 | 6.18847457627119 | 0.000412622771939214 | 0.0418557190082403 | 3.38444680773795 | C3//XCL1//CLCF1//APOA2//FOXJ1//SEMA7A |
| GO:0070848 | response to growth factor stimulus | Biological process | 7 | 249 | 75 | 13692 | 5.13220883534137 | 0.000418969053297174 | 0.0418557190082403 | 3.37781805454832 | MED1//SNCA//BMP4//NTRK1//FOXJ1//XCL1//WNT10A |
| GO:0046503 | glycerolipid catabolic process | Biological process | 3 | 27 | 75 | 13692 | 20.2844444444444 | 0.000420084771068105 | 0.0418557190082403 | 3.3766630622396 | ENPP2//APOA2//CES1D |
| GO:0044106 | cellular amine metabolic process | Biological process | 5 | 116 | 75 | 13692 | 7.86896551724138 | 0.000430732336809707 | 0.0419629014349723 | 3.3657925227792 | HDC//APOA2//ENPP2//SNCA//PON1 |
| GO:0006928 | cellular component movement | Biological process | 14 | 932 | 75 | 13692 | 2.74231759656652 | 0.000469353611667053 | 0.0440248892359521 | 3.32849983589755 | ROBO3//XCL1//BMP4//AQP1//ACE//MDK//MMP14//VAV3//ENPP2//LAMA2//FOXJ1//CCDC40//LPAR1 |
| GO:0055074 | calcium ion homeostasis | Biological process | 7 | 254 | 75 | 13692 | 5.03118110236221 | 0.000471982161060618 | 0.0440248892359521 | 3.32607441555944 | RGN//TRPV4//NMB//WFS1//XCL1//SNCA//LPAR1 |
| GO:0043410 | positive regulation of MAPK cascade | Biological process | 6 | 184 | 75 | 13692 | 5.95304347826087 | 0.00050694997028621 | 0.0442901762127463 | 3.29503489806453 | C3//BMP4//NTRK1//C1QTNF3//SEMA7A//LPAR1 |
| GO:0018958 | phenol-containing compound metabolic process | Biological process | 4 | 68 | 75 | 13692 | 10.7388235294118 | 0.000518740055100096 | 0.0442901762127463 | 3.285050216172 | SLC5A5//MED1//SNCA//HDC |
| GO:0042180 | cellular ketone metabolic process | Biological process | 12 | 723 | 75 | 13692 | 3.03004149377593 | 0.000522395806101927 | 0.0442901762127463 | 3.282000318322 | MGST1//HDC//SLC5A5//MED1//SNCA//ELOVL7//UGT1A6//RGN//C1QTNF3//PON1//PON3//FOLR1 |
| GO:0050776 | regulation of immune response | Biological process | 8 | 340 | 75 | 13692 | 4.29552941176471 | 0.000528036332749326 | 0.0442901762127463 | 3.2773361938115 | C3//XCL1//FOXJ1//APOA2//CLCF1//VAV3//MED1//SEMA7A |
| GO:0050767 | regulation of neurogenesis | Biological process | 9 | 428 | 75 | 13692 | 3.8388785046729 | 0.000529283329601237 | 0.0442901762127463 | 3.27631178440701 | NTRK1//TRPV4//LPAR1//LMX1A//MED1//BMP4//SEMA7A//CLCF1//ACE |
| GO:0016477 | cell migration | Biological process | 12 | 725 | 75 | 13692 | 3.02168275862069 | 0.000535442367535482 | 0.0442901762127463 | 3.27128726770476 | ROBO3//XCL1//BMP4//AQP1//ACE//ENPP2//LAMA2//MMP14//FOXJ1//LPAR1//MDK//VAV3 |
| GO:0072503 | cellular divalent inorganic cation homeostasis | Biological process | 7 | 262 | 75 | 13692 | 4.8775572519084 | 0.000567841457377374 | 0.0453072924738115 | 3.24577290336888 | RGN//TRPV4//NMB//WFS1//XCL1//SNCA//LPAR1 |
| GO:0048584 | positive regulation of response to stimulus | Biological process | 14 | 950 | 75 | 13692 | 2.69035789473684 | 0.000568408094447909 | 0.0453072924738115 | 3.24533974610518 | C3//XCL1//BMP4//EPHA5//MSX1//MED1//LPAR1//CLCF1//S100A4//NTRK1//ACE//VAV3//C1QTNF3//SEMA7A |
| GO:0048522 | positive regulation of cellular process | Biological process | 28 | 2813 | 75 | 13692 | 1.81716317099182 | 0.000588057780682733 | 0.0460365234020197 | 3.23057999943609 | BMP4//LPAR1//XCL1//SNCA//MDFIC//CLCF1//NMB//AQP1//COL8A1//APOA2//NTRK1//EPHA5//MDK//MED1//SERTAD2//MSX1//BAIAP2L1//VAV3//TRPV4//WFS1//CAMKK2//LAMA2//ACE//S100A4//SEMA7A//FOXJ1//C3//C1QTNF3 |
| GO:0044093 | positive regulation of molecular function | Biological process | 13 | 848 | 75 | 13692 | 2.79867924528302 | 0.000635365859159682 | 0.0472204047249275 | 3.19697612532397 | BMP4//LPAR1//SNCA//MDFIC//MMP14//CAMKK2//NTRK1//PON1//RGN//EPHA5//FOXJ1//VAV3//MED1 |
| GO:0035116 | embryonic hindlimb morphogenesis | Biological process | 3 | 31 | 75 | 13692 | 17.6670967741935 | 0.000635493585486023 | 0.0472204047249275 | 3.19688882874819 | BMP4//MSX1//MED1 |
| GO:0046470 | phosphatidylcholine metabolic process | Biological process | 3 | 31 | 75 | 13692 | 17.6670967741935 | 0.000635493585486023 | 0.0472204047249275 | 3.19688882874819 | APOA2//ENPP2//PON1 |
| GO:0010817 | regulation of hormone levels | Biological process | 8 | 354 | 75 | 13692 | 4.12564971751412 | 0.000688271150726189 | 0.0498482662843762 | 3.16224043376314 | SLC5A5//MED1//NMB//TRPV4//ACE//AQP1//EPHA5//SLCO1A5 |
| GO:0072507 | divalent inorganic cation homeostasis | Biological process | 7 | 271 | 75 | 13692 | 4.71557195571956 | 0.000693600420471476 | 0.0498482662843762 | 3.15889065225563 | RGN//TRPV4//NMB//WFS1//XCL1//SNCA//LPAR1 |
| GO:0042475 | odontogenesis of dentin-containing tooth | Biological process | 4 | 75 | 75 | 13692 | 9.73653333333333 | 0.000752172182822755 | 0.0531392667760446 | 3.1236827318947 | BMP4//WNT10A//MSX1//SOSTDC1 |
| GO:0010740 | positive regulation of intracellular protein kinase cascade | Biological process | 8 | 360 | 75 | 13692 | 4.05688888888889 | 0.000768003923986053 | 0.0531392667760446 | 3.11463656100868 | CLCF1//S100A4//LPAR1//C1QTNF3//C3//BMP4//NTRK1//SEMA7A |
| GO:0030030 | cell projection organization | Biological process | 12 | 756 | 75 | 13692 | 2.89777777777778 | 0.000775755719358315 | 0.0531392667760446 | 3.11027501384035 | NTRK1//EPHA5//LMX1A//LAMA2//ROBO3//AQP1//TRPV4//LPAR1//VAV3//FOXJ1//SEMA7A//BAIAP2L1 |
| GO:0051129 | negative regulation of cellular component organization | Biological process | 7 | 285 | 75 | 13692 | 4.4839298245614 | 0.000932243481896519 | 0.0627360179354124 | 3.03047064447647 | APOA2//TRPV4//LPAR1//SNCA//BMP4//MMP14//EPHA5 |
| GO:0006820 | anion transport | Biological process | 5 | 138 | 75 | 13692 | 6.61449275362319 | 0.00094832957446782 | 0.0627360179354124 | 3.02304070538518 | SLC4A2//SLC4A4//SLC5A5//SLC16A8//SLCO1A5 |
| GO:0016064 | immunoglobulin mediated immune response | Biological process | 4 | 80 | 75 | 13692 | 9.128 | 0.000958784945637005 | 0.0627360179354124 | 3.01827879366019 | C3//XCL1//FOXJ1//CLCF1 |
| GO:0009308 | amine metabolic process | Biological process | 5 | 139 | 75 | 13692 | 6.56690647482014 | 0.000979561035138257 | 0.0628886731168105 | 3.00896849851772 | HDC//APOA2//ENPP2//SNCA//PON1 |
| GO:0002718 | regulation of cytokine production involved in immune response | Biological process | 3 | 36 | 75 | 13692 | 15.2133333333333 | 0.000989808039475348 | 0.0628886731168105 | 3.00444902305911 | XCL1//APOA2//SEMA7A |
| GO:0002706 | regulation of lymphocyte mediated immunity | Biological process | 4 | 81 | 75 | 13692 | 9.01530864197531 | 0.00100444502031727 | 0.062906956701013 | 2.99807382997227 | C3//XCL1//FOXJ1//CLCF1 |
| GO:0022008 | neurogenesis | Biological process | 14 | 1009 | 75 | 13692 | 2.53304261645193 | 0.00102802492906966 | 0.0634195171269591 | 2.98799635379782 | ROBO3//NTRK1//EPHA5//LMX1A//LAMA2//TRPV4//LPAR1//FSCN2//MMP14//MED1//BMP4//SEMA7A//CLCF1//ACE |
| GO:0019724 | B cell mediated immunity | Biological process | 4 | 82 | 75 | 13692 | 8.90536585365854 | 0.00105161391115108 | 0.0634195171269591 | 2.97814367751414 | C3//XCL1//FOXJ1//CLCF1 |
| GO:0048731 | system development | Biological process | 29 | 3069 | 75 | 13692 | 1.72507005539264 | 0.00107422218290187 | 0.0634195171269591 | 2.96890588360043 | BMP4//MMP14//MED1//RPE65//ROBO3//ACE//WFS1//MSX1//UGT1A6//WNT10A//FOXJ1//C3//NTRK1//EPHA5//LMX1A//LAMA2//TRPV4//LPAR1//MDK//AQP1//CLCF1//PRPS2//APOA2//SLCO1A5//MGST1//FSCN2//SOSTDC1//SEMA7A//COL8A1 |
| GO:0007411 | axon guidance | Biological process | 5 | 142 | 75 | 13692 | 6.42816901408451 | 0.00107785725657589 | 0.0634195171269591 | 2.96743875008264 | NTRK1//EPHA5//LMX1A//LAMA2//ROBO3 |
| GO:0009967 | positive regulation of signal transduction | Biological process | 11 | 677 | 75 | 13692 | 2.96626292466765 | 0.00108495980486358 | 0.0634195171269591 | 2.96458635107748 | BMP4//EPHA5//MSX1//MED1//LPAR1//CLCF1//S100A4//NTRK1//C1QTNF3//C3//SEMA7A |
| GO:0048545 | response to steroid hormone stimulus | Biological process | 9 | 477 | 75 | 13692 | 3.44452830188679 | 0.00114300907400462 | 0.0638004494780843 | 2.94195032185801 | C3//BMP4//FOXJ1//AQP1//APOA2//MMP14//MDK//MED1//UGT1A6 |
| GO:0009887 | organ morphogenesis | Biological process | 12 | 792 | 75 | 13692 | 2.76606060606061 | 0.00116286766661487 | 0.0638004494780843 | 2.93446970481491 | BMP4//FOXJ1//MSX1//MED1//WNT10A//PRPS2//APOA2//SLCO1A5//FSCN2//SOSTDC1//AQP1//COL8A1 |
| GO:0050670 | regulation of lymphocyte proliferation | Biological process | 5 | 145 | 75 | 13692 | 6.2951724137931 | 0.00118329953119775 | 0.0638004494780843 | 2.92690530754398 | VAV3//CLCF1//BMP4//XCL1//FOXJ1 |
| GO:0002822 | regulation of adaptive immune response based on somatic recombination of immune receptors built from immunoglobulin superfamily domains | Biological process | 4 | 85 | 75 | 13692 | 8.59105882352941 | 0.001202443387364 | 0.0638004494780843 | 2.91993536163537 | C3//XCL1//FOXJ1//CLCF1 |
| GO:0032944 | regulation of mononuclear cell proliferation | Biological process | 5 | 146 | 75 | 13692 | 6.25205479452055 | 0.00122008946620773 | 0.0638004494780843 | 2.91360832239432 | VAV3//CLCF1//BMP4//XCL1//FOXJ1 |
| GO:0009611 | response to wounding | Biological process | 11 | 688 | 75 | 13692 | 2.91883720930233 | 0.00123610745440985 | 0.0638004494780843 | 2.90794377453132 | C3//F12//APOA2//F5//AQP1//CDH3//NTRK1//XCL1//SEMA7A//ACE//MDK |
| GO:0035137 | hindlimb morphogenesis | Biological process | 3 | 39 | 75 | 13692 | 14.0430769230769 | 0.00125209896867968 | 0.0638004494780843 | 2.90236134216972 | BMP4//MSX1//MED1 |
| GO:0072376 | protein activation cascade | Biological process | 3 | 39 | 75 | 13692 | 14.0430769230769 | 0.00125209896867968 | 0.0638004494780843 | 2.90236134216972 | C3//F12//F5 |
| GO:0006875 | cellular metal ion homeostasis | Biological process | 7 | 300 | 75 | 13692 | 4.25973333333333 | 0.0012555266738373 | 0.0638004494780843 | 2.9011740562069 | RGN//TRPV4//NMB//WFS1//XCL1//SNCA//LPAR1 |
| GO:0051384 | response to glucocorticoid stimulus | Biological process | 6 | 219 | 75 | 13692 | 5.00164383561644 | 0.00125639876183128 | 0.0638004494780843 | 2.9008725002663 | UGT1A6//AQP1//BMP4//C3//APOA2//MDK |
| GO:0032502 | developmental process | Biological process | 34 | 3887 | 75 | 13692 | 1.59687162335992 | 0.00127565153204779 | 0.0638004494780843 | 2.89426794503275 | MSX1//MED1//BMP4//MMP14//RPE65//ROBO3//ACE//WFS1//UGT1A6//WNT10A//FOXJ1//C3//WIPF3//PRTG//ZAR1//SOSTDC1//NTRK1//EPHA5//LMX1A//LAMA2//SNCA//TRPV4//LPAR1//MDK//AQP1//CLCF1//PRPS2//APOA2//SLCO1A5//MGST1//FSCN2//SEMA7A//COL8A1//CCDC40 |
| GO:0055080 | cation homeostasis | Biological process | 8 | 390 | 75 | 13692 | 3.74482051282051 | 0.00128603096550869 | 0.0638004494780843 | 2.89074857418968 | RGN//TRPV4//SLC4A4//NMB//WFS1//XCL1//SNCA//LPAR1 |
| GO:0019752 | carboxylic acid metabolic process | Biological process | 11 | 692 | 75 | 13692 | 2.90196531791908 | 0.00129521897891184 | 0.0638004494780843 | 2.88765680046817 | MGST1//HDC//SLC5A5//MED1//SNCA//ELOVL7//UGT1A6//RGN//PON1//PON3//FOLR1 |
| GO:0043436 | oxoacid metabolic process | Biological process | 11 | 692 | 75 | 13692 | 2.90196531791908 | 0.00129521897891184 | 0.0638004494780843 | 2.88765680046817 | MGST1//HDC//SLC5A5//MED1//SNCA//ELOVL7//UGT1A6//RGN//PON1//PON3//FOLR1 |
| GO:0000165 | MAPK cascade | Biological process | 8 | 392 | 75 | 13692 | 3.72571428571429 | 0.00132860373762872 | 0.0647177642862701 | 2.87660453013529 | MDFIC//LPAR1//MED1//C3//BMP4//NTRK1//C1QTNF3//SEMA7A |
| GO:0019725 | cellular homeostasis | Biological process | 11 | 696 | 75 | 13692 | 2.88528735632184 | 0.00135664966147465 | 0.0653577155593941 | 2.86753228920993 | RGN//TRPV4//AQP1//NMB//LAMA2//WFS1//LPAR1//XCL1//SNCA//SCN4B//EPHA5 |
| GO:0070663 | regulation of leukocyte proliferation | Biological process | 5 | 151 | 75 | 13692 | 6.04503311258278 | 0.00141688931854601 | 0.0665504710418575 | 2.84866407369193 | VAV3//CLCF1//BMP4//XCL1//FOXJ1 |
| GO:0002819 | regulation of adaptive immune response | Biological process | 4 | 89 | 75 | 13692 | 8.20494382022472 | 0.00142630900987065 | 0.0665504710418575 | 2.84578637438368 | C3//XCL1//FOXJ1//CLCF1 |
| GO:0051049 | regulation of transport | Biological process | 13 | 926 | 75 | 13692 | 2.5629373650108 | 0.00143009492411369 | 0.0665504710418575 | 2.84463513480727 | SNCA//APOA2//AQP1//PON1//NMB//MED1//SCN4B//MDFIC//C3//XCL1//WFS1//BMP4//EPHA5 |
| GO:0042130 | negative regulation of T cell proliferation | Biological process | 3 | 41 | 75 | 13692 | 13.3580487804878 | 0.00144907385730967 | 0.0665504710418575 | 2.83890947856982 | BMP4//XCL1//FOXJ1 |
| GO:0009653 | anatomical structure morphogenesis | Biological process | 20 | 1819 | 75 | 13692 | 2.00725673446949 | 0.00146612583073168 | 0.0665504710418575 | 2.83382875462853 | MSX1//MED1//BMP4//MMP14//FOXJ1//NTRK1//EPHA5//LMX1A//LAMA2//ROBO3//WNT10A//PRPS2//APOA2//SLCO1A5//FSCN2//SOSTDC1//AQP1//C3//SEMA7A//COL8A1 |
| GO:0048699 | generation of neurons | Biological process | 13 | 929 | 75 | 13692 | 2.55466092572659 | 0.0014724898930338 | 0.0665504710418575 | 2.83194767746766 | ROBO3//NTRK1//EPHA5//LMX1A//LAMA2//TRPV4//LPAR1//FSCN2//MED1//BMP4//SEMA7A//CLCF1//ACE |
| GO:0043408 | regulation of MAPK cascade | Biological process | 7 | 309 | 75 | 13692 | 4.13566343042071 | 0.00148810323780356 | 0.0665698428013348 | 2.82736693837809 | MDFIC//LPAR1//C3//BMP4//NTRK1//C1QTNF3//SEMA7A |
| GO:0006082 | organic acid metabolic process | Biological process | 11 | 707 | 75 | 13692 | 2.84039603960396 | 0.00153811076877824 | 0.0677838484972646 | 2.8130123872024 | MGST1//HDC//SLC5A5//MED1//SNCA//ELOVL7//UGT1A6//RGN//PON1//PON3//FOLR1 |
| GO:0002367 | cytokine production involved in immune response | Biological process | 3 | 42 | 75 | 13692 | 13.04 | 0.00155443452014556 | 0.0677838484972646 | 2.80842756769503 | XCL1//APOA2//SEMA7A |
| GO:0042487 | regulation of odontogenesis of dentin-containing tooth | Biological process | 2 | 11 | 75 | 13692 | 33.1927272727273 | 0.00157708771594913 | 0.0677838484972646 | 2.80214415100003 | BMP4//WNT10A |
| GO:0044241 | lipid digestion | Biological process | 2 | 11 | 75 | 13692 | 33.1927272727273 | 0.00157708771594913 | 0.0677838484972646 | 2.80214415100003 | APOA2//AQP1 |
| GO:0031100 | organ regeneration | Biological process | 4 | 92 | 75 | 13692 | 7.93739130434783 | 0.0016122189635966 | 0.0679612301577644 | 2.79257597479904 | PRPS2//APOA2//SLCO1A5//MED1 |
| GO:0031647 | regulation of protein stability | Biological process | 4 | 92 | 75 | 13692 | 7.93739130434783 | 0.0016122189635966 | 0.0679612301577644 | 2.79257597479904 | MSX1//WFS1//SNCA//APOA2 |
| GO:0051240 | positive regulation of multicellular organismal process | Biological process | 9 | 502 | 75 | 13692 | 3.27298804780876 | 0.00162878265345024 | 0.0680055538354843 | 2.7881368645588 | XCL1//AQP1//C3//F12//BMP4//LAMA2//SNCA//NTRK1//SEMA7A |
| GO:0042439 | ethanolamine-containing compound metabolic process | Biological process | 3 | 43 | 75 | 13692 | 12.7367441860465 | 0.00166447968014446 | 0.0688403671486162 | 2.77872150231266 | APOA2//ENPP2//PON1 |
| GO:0031960 | response to corticosteroid stimulus | Biological process | 6 | 232 | 75 | 13692 | 4.72137931034483 | 0.00168592110135949 | 0.0688787671134732 | 2.77316275358196 | C3//BMP4//APOA2//MDK//UGT1A6//AQP1 |
| GO:0042493 | response to drug | Biological process | 9 | 505 | 75 | 13692 | 3.25354455445545 | 0.00169683094166403 | 0.0688787671134732 | 2.77036142507175 | AQP1//COX8B//APOA2//SNCA//NTRK1//MDK//CDH3//MGST1//VAV3 |
| GO:0042221 | response to chemical stimulus | Biological process | 32 | 3628 | 75 | 13692 | 1.61023153252481 | 0.00172027539749725 | 0.0691897921342013 | 2.76440202167428 | MED1//XCL1//UGT1A6//C3//ENPP2//NTRK1//EPHA5//LMX1A//LAMA2//ROBO3//SNCA//AQP1//COX8B//APOA2//MDK//CDH3//MGST1//VAV3//PON1//MMP14//RERG//PON3//BMP4//FOXJ1//WFS1//ACE//KRT18//F12//MSX1//SLC5A5//LPAR1//WNT10A |
| GO:0071383 | cellular response to steroid hormone stimulus | Biological process | 4 | 94 | 75 | 13692 | 7.76851063829787 | 0.00174513700237488 | 0.0695516419855588 | 2.75817047297996 | BMP4//UGT1A6//AQP1//MED1 |
| GO:0046700 | heterocycle catabolic process | Biological process | 8 | 412 | 75 | 13692 | 3.54485436893204 | 0.00181939577321781 | 0.0706611386675071 | 2.74007281856793 | RERG//HDC//EPHA5//NTRK1//RGN//FOXJ1//VAV3//PON3 |
| GO:0055065 | metal ion homeostasis | Biological process | 7 | 321 | 75 | 13692 | 3.98105919003115 | 0.00184919338660902 | 0.0706611386675071 | 2.73301766842687 | RGN//TRPV4//NMB//WFS1//XCL1//SNCA//LPAR1 |
| GO:0060284 | regulation of cell development | Biological process | 9 | 512 | 75 | 13692 | 3.2090625 | 0.00186454348144345 | 0.0706611386675071 | 2.72942748436984 | NTRK1//TRPV4//LPAR1//BMP4//LMX1A//MED1//SEMA7A//CLCF1//ACE |
| GO:0043627 | response to estrogen stimulus | Biological process | 6 | 237 | 75 | 13692 | 4.62177215189873 | 0.00187793000138986 | 0.0706611386675071 | 2.72632059981042 | C3//BMP4//FOXJ1//AQP1//APOA2//MMP14 |
| GO:0003091 | renal water homeostasis | Biological process | 2 | 12 | 75 | 13692 | 30.4266666666667 | 0.00188580137411002 | 0.0706611386675071 | 2.72450405214519 | AQP1//WFS1 |
| GO:0042538 | hyperosmotic salinity response | Biological process | 2 | 12 | 75 | 13692 | 30.4266666666667 | 0.00188580137411002 | 0.0706611386675071 | 2.72450405214519 | AQP1//TRPV4 |
| GO:0071470 | cellular response to osmotic stress | Biological process | 2 | 12 | 75 | 13692 | 30.4266666666667 | 0.00188580137411002 | 0.0706611386675071 | 2.72450405214519 | TRPV4//AQP1 |
| GO:0040012 | regulation of locomotion | Biological process | 8 | 418 | 75 | 13692 | 3.49397129186603 | 0.00199170896269624 | 0.0726654267731948 | 2.70077412230256 | XCL1//BMP4//AQP1//ACE//ENPP2//LAMA2//LPAR1//SNCA |
| GO:0050793 | regulation of developmental process | Biological process | 16 | 1333 | 75 | 13692 | 2.19126781695424 | 0.00200643365475618 | 0.0726654267731948 | 2.69757519618415 | BMP4//FOXJ1//NTRK1//TRPV4//LPAR1//MED1//MSX1//WNT10A//LMX1A//C3//AQP1//SEMA7A//LAMA2//CLCF1//ACE//EPHA5 |
| GO:0002440 | production of molecular mediator of immune response | Biological process | 4 | 98 | 75 | 13692 | 7.45142857142857 | 0.00203347783315379 | 0.0726654267731948 | 2.69176055745731 | XCL1//CLCF1//APOA2//SEMA7A |
| GO:0019637 | organophosphate metabolic process | Biological process | 12 | 846 | 75 | 13692 | 2.58950354609929 | 0.00203673749457061 | 0.0726654267731948 | 2.69106494154765 | PRPS2//AQP1//RERG//SNCA//APOA2//EPHA5//NTRK1//RGN//FOXJ1//ENPP2//VAV3//PON1 |
| GO:0006869 | lipid transport | Biological process | 5 | 164 | 75 | 13692 | 5.56585365853658 | 0.00203770094389431 | 0.0726654267731948 | 2.69085955337503 | PON1//APOA2//ACE//NMB//GULP1 |
| GO:0050878 | regulation of body fluid levels | Biological process | 6 | 241 | 75 | 13692 | 4.5450622406639 | 0.00204314502885797 | 0.0726654267731948 | 2.6897008046856 | WFS1//AQP1//C3//F5//F12//MED1 |
| GO:0055082 | cellular chemical homeostasis | Biological process | 10 | 624 | 75 | 13692 | 2.92564102564103 | 0.00205531772807394 | 0.0726654267731948 | 2.68712103175235 | RGN//TRPV4//NMB//LAMA2//WFS1//LPAR1//XCL1//SNCA//SCN4B//EPHA5 |
| GO:0050801 | ion homeostasis | Biological process | 10 | 626 | 75 | 13692 | 2.91629392971246 | 0.00210412253692842 | 0.0737957856151536 | 2.6769289719507 | RGN//TRPV4//SLC4A4//NMB//LAMA2//WFS1//LPAR1//XCL1//SNCA//SCN4B |
| GO:0050727 | regulation of inflammatory response | Biological process | 5 | 167 | 75 | 13692 | 5.46586826347305 | 0.00220539776686927 | 0.0754053513809609 | 2.65651306951723 | C3//F12//ACE//XCL1//SEMA7A |
| GO:0023051 | regulation of signaling | Biological process | 19 | 1744 | 75 | 13692 | 1.98889908256881 | 0.00221388633988326 | 0.0754053513809609 | 2.65484467940517 | LPAR1//SNCA//MDFIC//BMP4//EPHA5//NMB//MSX1//SOSTDC1//LAMA2//NTRK1//FOXJ1//MED1//VAV3//CLCF1//S100A4//RGN//C1QTNF3//C3//SEMA7A |
| GO:0021542 | dentate gyrus development | Biological process | 2 | 13 | 75 | 13692 | 28.0861538461538 | 0.00222078277336004 | 0.0754053513809609 | 2.65349392003994 | MDK//LMX1A |
| GO:0009966 | regulation of signal transduction | Biological process | 17 | 1477 | 75 | 13692 | 2.10123222748815 | 0.00222943533461646 | 0.0754053513809609 | 2.65180511996229 | LPAR1//MDFIC//SNCA//BMP4//EPHA5//MSX1//SOSTDC1//NTRK1//FOXJ1//MED1//VAV3//CLCF1//S100A4//RGN//C1QTNF3//C3//SEMA7A |
| GO:0002252 | immune effector process | Biological process | 7 | 332 | 75 | 13692 | 3.84915662650602 | 0.00223698183471945 | 0.0754053513809609 | 2.65033754255207 | C3//XCL1//ACE//CLCF1//APOA2//FOXJ1//SEMA7A |
| GO:0006811 | ion transport | Biological process | 11 | 742 | 75 | 13692 | 2.70641509433962 | 0.0022532164760278 | 0.0754053513809609 | 2.64719708176573 | AQP1//SLC4A4//SLC5A5//SCN4B//TRPV4//SLC4A2//SLC16A8//SLCO1A5//XCL1//SNCA//WFS1 |
| GO:0002682 | regulation of immune system process | Biological process | 10 | 635 | 75 | 13692 | 2.87496062992126 | 0.0023355126392939 | 0.0775673288686701 | 2.63161777806117 | C3//XCL1//FOXJ1//CLCF1//APOA2//VAV3//BMP4//SNCA//MED1//SEMA7A |
| GO:0030003 | cellular cation homeostasis | Biological process | 7 | 337 | 75 | 13692 | 3.79204747774481 | 0.00243281428243303 | 0.0785984901275529 | 2.61389104322415 | RGN//TRPV4//NMB//WFS1//XCL1//SNCA//LPAR1 |
| GO:0043270 | positive regulation of ion transport | Biological process | 4 | 103 | 75 | 13692 | 7.08970873786408 | 0.00243827432877445 | 0.0785984901275529 | 2.61291743375842 | SNCA//XCL1//WFS1//SCN4B |
| GO:0043524 | negative regulation of neuron apoptotic process | Biological process | 4 | 103 | 75 | 13692 | 7.08970873786408 | 0.00243827432877445 | 0.0785984901275529 | 2.61291743375842 | SNCA//NTRK1//WFS1//CLCF1 |
| GO:1901215 | negative regulation of neuron death | Biological process | 4 | 103 | 75 | 13692 | 7.08970873786408 | 0.00243827432877445 | 0.0785984901275529 | 2.61291743375842 | SNCA//NTRK1//WFS1//CLCF1 |
| GO:0006955 | immune response | Biological process | 10 | 641 | 75 | 13692 | 2.84804992199688 | 0.00250089756486291 | 0.0796420510984929 | 2.60190409632462 | C3//XCL1//FOXJ1//APOA2//SNCA//CLCF1//VAV3//MED1//SEMA7A//ENPP2 |
| GO:0007204 | elevation of cytosolic calcium ion concentration | Biological process | 5 | 172 | 75 | 13692 | 5.30697674418605 | 0.00250698062308212 | 0.0796420510984929 | 2.60084902277135 | XCL1//SNCA//LPAR1//TRPV4//NMB |
| GO:0006590 | thyroid hormone generation | Biological process | 2 | 14 | 75 | 13692 | 26.08 | 0.0025817426384607 | 0.0801587297937728 | 2.58808705264556 | SLC5A5//MED1 |
| GO:0061311 | cell surface receptor signaling pathway involved in heart development | Biological process | 2 | 14 | 75 | 13692 | 26.08 | 0.0025817426384607 | 0.0801587297937728 | 2.58808705264556 | BMP4//MSX1 |
| GO:0032101 | regulation of response to external stimulus | Biological process | 7 | 341 | 75 | 13692 | 3.74756598240469 | 0.00259881655893292 | 0.0801587297937728 | 2.58522437469477 | C3//F12//XCL1//SEMA7A//ACE//MED1//LPAR1 |
| GO:0002699 | positive regulation of immune effector process | Biological process | 4 | 105 | 75 | 13692 | 6.95466666666667 | 0.00261466659683155 | 0.0801587297937728 | 2.58258368132348 | C3//XCL1//CLCF1//SEMA7A |
| GO:0002703 | regulation of leukocyte mediated immunity | Biological process | 4 | 105 | 75 | 13692 | 6.95466666666667 | 0.00261466659683155 | 0.0801587297937728 | 2.58258368132348 | C3//XCL1//FOXJ1//CLCF1 |
| GO:0010627 | regulation of intracellular protein kinase cascade | Biological process | 9 | 540 | 75 | 13692 | 3.04266666666667 | 0.00267352619038138 | 0.080917925035487 | 2.57291555710978 | MDFIC//CLCF1//S100A4//LPAR1//C1QTNF3//C3//BMP4//NTRK1//SEMA7A |
| GO:0007399 | nervous system development | Biological process | 17 | 1503 | 75 | 13692 | 2.06488356620093 | 0.00268430121984141 | 0.080917925035487 | 2.57116875125461 | ROBO3//BMP4//NTRK1//EPHA5//LMX1A//LAMA2//MED1//TRPV4//LPAR1//MDK//AQP1//MSX1//FSCN2//MMP14//SEMA7A//CLCF1//ACE |
| GO:0048513 | organ development | Biological process | 23 | 2341 | 75 | 13692 | 1.79362665527552 | 0.00269480316039715 | 0.080917925035487 | 2.56947295198945 | BMP4//MMP14//RPE65//ACE//WFS1//MSX1//UGT1A6//MED1//WNT10A//FOXJ1//LMX1A//MDK//AQP1//EPHA5//NTRK1//CLCF1//PRPS2//APOA2//SLCO1A5//MGST1//FSCN2//SOSTDC1//COL8A1 |
| GO:0032945 | negative regulation of mononuclear cell proliferation | Biological process | 3 | 52 | 75 | 13692 | 10.5323076923077 | 0.00287750263282854 | 0.085236294204867 | 2.54098427033157 | BMP4//FOXJ1//XCL1 |
| GO:0050672 | negative regulation of lymphocyte proliferation | Biological process | 3 | 52 | 75 | 13692 | 10.5323076923077 | 0.00287750263282854 | 0.085236294204867 | 2.54098427033157 | BMP4//FOXJ1//XCL1 |
| GO:0043085 | positive regulation of catalytic activity | Biological process | 10 | 655 | 75 | 13692 | 2.78717557251908 | 0.00292373325511659 | 0.085536270353887 | 2.53406225247866 | BMP4//LPAR1//SNCA//MDFIC//MMP14//CAMKK2//NTRK1//RGN//FOXJ1//VAV3 |
| GO:0007548 | sex differentiation | Biological process | 6 | 259 | 75 | 13692 | 4.22918918918919 | 0.00292665158601347 | 0.085536270353887 | 2.53362897667051 | BMP4//MMP14//WNT10A//MGST1//NTRK1//MED1 |
| GO:0051281 | positive regulation of release of sequestered calcium ion into cytosol | Biological process | 2 | 15 | 75 | 13692 | 24.3413333333333 | 0.00296839400171515 | 0.0861817172418491 | 2.52747845466857 | SNCA//XCL1 |
| GO:0021537 | telencephalon development | Biological process | 5 | 181 | 75 | 13692 | 5.04309392265193 | 0.00312385579958756 | 0.0900298273816615 | 2.5053090218142 | MDK//LMX1A//AQP1//EPHA5//BMP4 |
| GO:0030031 | cell projection assembly | Biological process | 5 | 182 | 75 | 13692 | 5.01538461538462 | 0.00319858505804975 | 0.0900298273816615 | 2.49504209586076 | AQP1//VAV3//LPAR1//FOXJ1//BAIAP2L1 |
| GO:0070664 | negative regulation of leukocyte proliferation | Biological process | 3 | 54 | 75 | 13692 | 10.1422222222222 | 0.00320441156940677 | 0.0900298273816615 | 2.4942517089289 | BMP4//FOXJ1//XCL1 |
| GO:0046651 | lymphocyte proliferation | Biological process | 5 | 183 | 75 | 13692 | 4.9879781420765 | 0.00327459772637668 | 0.0900298273816615 | 2.48484204405246 | VAV3//CLCF1//BMP4//XCL1//FOXJ1 |
| GO:0032943 | mononuclear cell proliferation | Biological process | 5 | 184 | 75 | 13692 | 4.96086956521739 | 0.00335190632291551 | 0.0900298273816615 | 2.47470812727606 | VAV3//CLCF1//BMP4//XCL1//FOXJ1 |
| GO:0006810 | transport | Biological process | 25 | 2679 | 75 | 13692 | 1.70362075401269 | 0.00337549378775309 | 0.0900298273816615 | 2.47166268694846 | SLC6A6//SNCA//APOA2//AQP1//VAV3//SLC4A4//SLC5A5//SCN4B//TRPV4//SLC4A2//GULP1//MED1//PON1//SLC16A8//SLCO1A5//FOLR1//NMB//MDFIC//KRT18//ACE//C3//XCL1//WFS1//BMP4//EPHA5 |
| GO:0002507 | tolerance induction | Biological process | 2 | 16 | 75 | 13692 | 22.82 | 0.00338045218691502 | 0.0900298273816615 | 2.47102520234162 | FOXJ1//C3 |
| GO:0002823 | negative regulation of adaptive immune response based on somatic recombination of immune receptors built from immunoglobulin superfamily domains | Biological process | 2 | 16 | 75 | 13692 | 22.82 | 0.00338045218691502 | 0.0900298273816615 | 2.47102520234162 | FOXJ1//XCL1 |
| GO:0009713 | catechol-containing compound biosynthetic process | Biological process | 2 | 16 | 75 | 13692 | 22.82 | 0.00338045218691502 | 0.0900298273816615 | 2.47102520234162 | SNCA//HDC |
| GO:0034312 | diol biosynthetic process | Biological process | 2 | 16 | 75 | 13692 | 22.82 | 0.00338045218691502 | 0.0900298273816615 | 2.47102520234162 | SNCA//HDC |
| GO:0042423 | catecholamine biosynthetic process | Biological process | 2 | 16 | 75 | 13692 | 22.82 | 0.00338045218691502 | 0.0900298273816615 | 2.47102520234162 | SNCA//HDC |
| GO:0014070 | response to organic cyclic compound | Biological process | 7 | 358 | 75 | 13692 | 3.56960893854749 | 0.00340397647502061 | 0.0900298273816615 | 2.46801344999874 | SNCA//NTRK1//MSX1//BMP4//MMP14//UGT1A6//FOXJ1 |
| GO:0045664 | regulation of neuron differentiation | Biological process | 7 | 358 | 75 | 13692 | 3.56960893854749 | 0.00340397647502061 | 0.0900298273816615 | 2.46801344999874 | NTRK1//TRPV4//LPAR1//LMX1A//MED1//BMP4//SEMA7A |
| GO:0007423 | sensory organ development | Biological process | 8 | 456 | 75 | 13692 | 3.20280701754386 | 0.00340445665235641 | 0.0900298273816615 | 2.46795219115586 | RPE65//MED1//FSCN2//MSX1//BMP4//WNT10A//AQP1//COL8A1 |
| GO:0002443 | leukocyte mediated immunity | Biological process | 5 | 185 | 75 | 13692 | 4.93405405405405 | 0.00343052336357174 | 0.0900298273816615 | 2.46463961856589 | C3//XCL1//ACE//FOXJ1//CLCF1 |
| GO:0046942 | carboxylic acid transport | Biological process | 5 | 185 | 75 | 13692 | 4.93405405405405 | 0.00343052336357174 | 0.0900298273816615 | 2.46463961856589 | SLC6A6//SLCO1A5//FOLR1//ACE//NMB |
| GO:0040008 | regulation of growth | Biological process | 8 | 457 | 75 | 13692 | 3.19579868708972 | 0.00345004812958922 | 0.0900298273816615 | 2.46217484629797 | LMX1A//MSX1//SERTAD2//RERG//SEMA7A//WFS1//C3//BMP4 |
| GO:0000003 | reproduction | Biological process | 13 | 1027 | 75 | 13692 | 2.31088607594937 | 0.00356078979853245 | 0.0915474226261832 | 2.44845366295363 | BMP4//MMP14//ACE//FOXJ1//WIPF3//DNAJB13//SOSTDC1//MED1//WNT10A//MGST1//MDFIC//NTRK1//ZAR1 |
| GO:0010876 | lipid localization | Biological process | 5 | 187 | 75 | 13692 | 4.88128342245989 | 0.00359173282201266 | 0.0915474226261832 | 2.44469597663758 | APOA2//GULP1//PON1//ACE//NMB |
| GO:0015849 | organic acid transport | Biological process | 5 | 187 | 75 | 13692 | 4.88128342245989 | 0.00359173282201266 | 0.0915474226261832 | 2.44469597663758 | SLC6A6//SLCO1A5//FOLR1//ACE//NMB |
| GO:0051480 | cytosolic calcium ion homeostasis | Biological process | 5 | 187 | 75 | 13692 | 4.88128342245989 | 0.00359173282201266 | 0.0915474226261832 | 2.44469597663758 | TRPV4//NMB//XCL1//SNCA//LPAR1 |
| GO:0051247 | positive regulation of protein metabolic process | Biological process | 10 | 675 | 75 | 13692 | 2.70459259259259 | 0.0036256166801562 | 0.0918768989930912 | 2.44061811377965 | BMP4//LPAR1//C3//F12//MDFIC//WFS1//CAMKK2//SNCA//CLCF1//APOA2 |
| GO:0071385 | cellular response to glucocorticoid stimulus | Biological process | 3 | 57 | 75 | 13692 | 9.60842105263158 | 0.0037360132685094 | 0.0929806163012442 | 2.42759159003558 | AQP1//BMP4//UGT1A6 |
| GO:0006873 | cellular ion homeostasis | Biological process | 9 | 568 | 75 | 13692 | 2.89267605633803 | 0.0037404242224523 | 0.0929806163012442 | 2.42707913923748 | RGN//TRPV4//NMB//LAMA2//WFS1//LPAR1//XCL1//SNCA//SCN4B |
| GO:0031638 | zymogen activation | Biological process | 2 | 17 | 75 | 13692 | 21.4776470588235 | 0.0038176347933905 | 0.0929806163012442 | 2.41820561989244 | F12//MMP14 |
| GO:0050891 | multicellular organismal water homeostasis | Biological process | 2 | 17 | 75 | 13692 | 21.4776470588235 | 0.0038176347933905 | 0.0929806163012442 | 2.41820561989244 | WFS1//AQP1 |
| GO:0061437 | renal system vasculature development | Biological process | 2 | 17 | 75 | 13692 | 21.4776470588235 | 0.0038176347933905 | 0.0929806163012442 | 2.41820561989244 | BMP4//AQP1 |
| GO:0061440 | kidney vasculature development | Biological process | 2 | 17 | 75 | 13692 | 21.4776470588235 | 0.0038176347933905 | 0.0929806163012442 | 2.41820561989244 | BMP4//AQP1 |
| GO:0072012 | glomerulus vasculature development | Biological process | 2 | 17 | 75 | 13692 | 21.4776470588235 | 0.0038176347933905 | 0.0929806163012442 | 2.41820561989244 | BMP4//AQP1 |
| GO:0071384 | cellular response to corticosteroid stimulus | Biological process | 3 | 58 | 75 | 13692 | 9.44275862068966 | 0.00392443357905551 | 0.0937934503177303 | 2.4062230169422 | UGT1A6//AQP1//BMP4 |
| GO:0031347 | regulation of defense response | Biological process | 6 | 275 | 75 | 13692 | 3.98312727272727 | 0.00392893390880189 | 0.0937934503177303 | 2.40572527668172 | C3//F12//XCL1//SEMA7A//ACE//MED1 |
| GO:0070661 | leukocyte proliferation | Biological process | 5 | 191 | 75 | 13692 | 4.77905759162304 | 0.00393040323926271 | 0.0937934503177303 | 2.40556289094698 | VAV3//CLCF1//BMP4//XCL1//FOXJ1 |
| GO:0006915 | apoptotic process | Biological process | 15 | 1295 | 75 | 13692 | 2.11459459459459 | 0.00397831594572242 | 0.0937934503177303 | 2.40030072949837 | SNCA//MSX1//WFS1//ACE//BMP4//LPAR1//AQP1//KRT18//MED1//NTRK1//CLCF1//RNF152//DNAJB13//GULP1 |
| GO:0033500 | carbohydrate homeostasis | Biological process | 4 | 118 | 75 | 13692 | 6.18847457627119 | 0.00397937540125407 | 0.0937934503177303 | 2.40018508900068 | WFS1//C1QTNF3//NMB//EPHA5 |
| GO:0042593 | glucose homeostasis | Biological process | 4 | 118 | 75 | 13692 | 6.18847457627119 | 0.00397937540125407 | 0.0937934503177303 | 2.40018508900068 | EPHA5//WFS1//C1QTNF3//NMB |
| GO:0009725 | response to hormone stimulus | Biological process | 11 | 801 | 75 | 13692 | 2.50706616729089 | 0.0040581698521773 | 0.0941888588785399 | 2.39166977979661 | MED1//C3//BMP4//FOXJ1//UGT1A6//AQP1//APOA2//MMP14//MDK//SLC5A5//RERG |
| GO:0071495 | cellular response to endogenous stimulus | Biological process | 8 | 470 | 75 | 13692 | 3.10740425531915 | 0.00408717172199094 | 0.0941888588785399 | 2.38857711507863 | MED1//UGT1A6//BMP4//SNCA//SLC5A5//AQP1//XCL1//WNT10A |
| GO:0022600 | digestive system process | Biological process | 3 | 59 | 75 | 13692 | 9.28271186440678 | 0.00411855261784813 | 0.0941888588785399 | 2.38525538118081 | AQP1//APOA2//SLCO1A5 |
| GO:0002694 | regulation of leukocyte activation | Biological process | 6 | 279 | 75 | 13692 | 3.92602150537634 | 0.00421509863122543 | 0.0941888588785399 | 2.37519225864403 | VAV3//CLCF1//BMP4//FOXJ1//XCL1//SNCA |
| GO:0071840 | cellular component organization or biogenesis | Biological process | 29 | 3342 | 75 | 13692 | 1.58415320167564 | 0.00421680113234383 | 0.0941888588785399 | 2.37501688017135 | MSX1//MED1//LMX1A//BMP4//FOXJ1//VAV3//GULP1//SNCA//TRPV4//NCAPH//NTRK1//EPHA5//LAMA2//ROBO3//AQP1//APOA2//LPAR1//LYZL4//SERTAD2//RERG//BAIAP2L1//CLDN19//SEMA7A//C3//C1QTNF3//MMP14//MGST1//CCDC40//CRB3 |
| GO:0032409 | regulation of transporter activity | Biological process | 4 | 120 | 75 | 13692 | 6.08533333333333 | 0.00422500396223202 | 0.0941888588785399 | 2.37417287943034 | SNCA//PON1//APOA2//SCN4B |
| GO:0048592 | eye morphogenesis | Biological process | 4 | 120 | 75 | 13692 | 6.08533333333333 | 0.00422500396223202 | 0.0941888588785399 | 2.37417287943034 | FSCN2//AQP1//BMP4//COL8A1 |
| GO:0051336 | regulation of hydrolase activity | Biological process | 9 | 579 | 75 | 13692 | 2.83772020725389 | 0.00424155027756795 | 0.0941888588785399 | 2.37247538068615 | SNCA//MMP14//EPHA5//NTRK1//RGN//FOXJ1//VAV3//AQP1//APOA2 |
| GO:0002820 | negative regulation of adaptive immune response | Biological process | 2 | 18 | 75 | 13692 | 20.2844444444444 | 0.00427966168016268 | 0.0941888588785399 | 2.36859056188542 | FOXJ1//XCL1 |
| GO:0050482 | arachidonic acid secretion | Biological process | 2 | 18 | 75 | 13692 | 20.2844444444444 | 0.00427966168016268 | 0.0941888588785399 | 2.36859056188542 | ACE//NMB |
| GO:0050892 | intestinal absorption | Biological process | 2 | 18 | 75 | 13692 | 20.2844444444444 | 0.00427966168016268 | 0.0941888588785399 | 2.36859056188542 | APOA2//SLCO1A5 |
| GO:0060602 | branch elongation of an epithelium | Biological process | 2 | 18 | 75 | 13692 | 20.2844444444444 | 0.00427966168016268 | 0.0941888588785399 | 2.36859056188542 | BMP4//MED1 |
| GO:0051234 | establishment of localization | Biological process | 25 | 2726 | 75 | 13692 | 1.67424798239178 | 0.00428767773474232 | 0.0941888588785399 | 2.36777786400621 | SLC6A6//SNCA//APOA2//AQP1//VAV3//SLC4A4//SLC5A5//SCN4B//TRPV4//SLC4A2//GULP1//MED1//PON1//SLC16A8//SLCO1A5//FOLR1//NMB//MDFIC//KRT18//ACE//C3//XCL1//WFS1//BMP4//EPHA5 |
| GO:0030001 | metal ion transport | Biological process | 8 | 474 | 75 | 13692 | 3.08118143459916 | 0.00430050578722007 | 0.0941888588785399 | 2.36648046356438 | AQP1//SLC4A4//SLC5A5//SCN4B//TRPV4//XCL1//SNCA//WFS1 |
| GO:0030510 | regulation of BMP signaling pathway | Biological process | 3 | 60 | 75 | 13692 | 9.128 | 0.00431842167759729 | 0.0941888588785399 | 2.36467495271224 | BMP4//MSX1//SOSTDC1 |
| GO:0001654 | eye development | Biological process | 6 | 281 | 75 | 13692 | 3.89807829181495 | 0.00436384547282928 | 0.0947084086776414 | 2.36013063651764 | MED1//FSCN2//BMP4//AQP1//COL8A1//RPE65 |
| GO:0030334 | regulation of cell migration | Biological process | 7 | 375 | 75 | 13692 | 3.40778666666667 | 0.00438768805802932 | 0.0947567706719238 | 2.35776425609555 | XCL1//BMP4//AQP1//ACE//LPAR1//ENPP2//LAMA2 |
| GO:0012501 | programmed cell death | Biological process | 15 | 1310 | 75 | 13692 | 2.09038167938931 | 0.00443279513874904 | 0.0952616367072343 | 2.3533223391195 | RNF152//KRT18//DNAJB13//GULP1//SNCA//NTRK1//MSX1//WFS1//ACE//BMP4//LPAR1//AQP1//MED1//CLCF1 |
| GO:0046483 | heterocycle metabolic process | Biological process | 11 | 813 | 75 | 13692 | 2.47006150061501 | 0.00453884940468049 | 0.0970649550737525 | 2.34305422655463 | HDC//PRPS2//AQP1//RERG//EPHA5//NTRK1//RGN//FOXJ1//VAV3//PON3//FOLR1 |
| GO:0015711 | organic anion transport | Biological process | 3 | 62 | 75 | 13692 | 8.83354838709677 | 0.00473560842060336 | 0.0985625551965403 | 2.32462421587171 | SLC4A4//SLC16A8//SLCO1A5 |
| GO:0021766 | hippocampus development | Biological process | 3 | 62 | 75 | 13692 | 8.83354838709677 | 0.00473560842060336 | 0.0985625551965403 | 2.32462421587171 | MDK//LMX1A//EPHA5 |
| GO:0030856 | regulation of epithelial cell differentiation | Biological process | 3 | 62 | 75 | 13692 | 8.83354838709677 | 0.00473560842060336 | 0.0985625551965403 | 2.32462421587171 | BMP4//MED1//FOXJ1 |
| GO:0033081 | regulation of T cell differentiation in thymus | Biological process | 2 | 19 | 75 | 13692 | 19.2168421052632 | 0.00476625495019766 | 0.0985625551965403 | 2.32182273066295 | BMP4//FOXJ1 |
| GO:0042775 | mitochondrial ATP synthesis coupled electron transport | Biological process | 2 | 19 | 75 | 13692 | 19.2168421052632 | 0.00476625495019766 | 0.0985625551965403 | 2.32182273066295 | COX8B//SNCA |
| GO:0046461 | neutral lipid catabolic process | Biological process | 2 | 19 | 75 | 13692 | 19.2168421052632 | 0.00476625495019766 | 0.0985625551965403 | 2.32182273066295 | APOA2//CES1D |
| GO:0046464 | acylglycerol catabolic process | Biological process | 2 | 19 | 75 | 13692 | 19.2168421052632 | 0.00476625495019766 | 0.0985625551965403 | 2.32182273066295 | APOA2//CES1D |
| GO:0043066 | negative regulation of apoptotic process | Biological process | 9 | 594 | 75 | 13692 | 2.76606060606061 | 0.00500831300545816 | 0.103081897727364 | 2.3003085367674 | SNCA//NTRK1//WFS1//CLCF1//BMP4//AQP1//MSX1//KRT18//MED1 |
| GO:0050868 | negative regulation of T cell activation | Biological process | 3 | 64 | 75 | 13692 | 8.5575 | 0.00517637792683713 | 0.105074047117561 | 2.28597402338492 | BMP4//FOXJ1//XCL1 |
| GO:0016043 | cellular component organization | Biological process | 28 | 3233 | 75 | 13692 | 1.58109495824312 | 0.00526653238175988 | 0.105074047117561 | 2.2784752411711 | MSX1//MED1//LMX1A//BMP4//FOXJ1//VAV3//GULP1//SNCA//TRPV4//NCAPH//NTRK1//EPHA5//LAMA2//ROBO3//AQP1//APOA2//LPAR1//SERTAD2//RERG//BAIAP2L1//CLDN19//SEMA7A//C3//C1QTNF3//MMP14//MGST1//CCDC40//CRB3 |
| GO:0002720 | positive regulation of cytokine production involved in immune response | Biological process | 2 | 20 | 75 | 13692 | 18.256 | 0.00527713893476188 | 0.105074047117561 | 2.27760147171142 | XCL1//SEMA7A |
| GO:0032026 | response to magnesium ion | Biological process | 2 | 20 | 75 | 13692 | 18.256 | 0.00527713893476188 | 0.105074047117561 | 2.27760147171142 | C3//SNCA |
| GO:0043586 | tongue development | Biological process | 2 | 20 | 75 | 13692 | 18.256 | 0.00527713893476188 | 0.105074047117561 | 2.27760147171142 | BMP4//WNT10A |
| GO:0043069 | negative regulation of programmed cell death | Biological process | 9 | 599 | 75 | 13692 | 2.74297161936561 | 0.00528664136477887 | 0.105074047117561 | 2.27682015026832 | SNCA//AQP1//BMP4//MSX1//WFS1//KRT18//MED1//NTRK1//CLCF1 |
| GO:0030811 | regulation of nucleotide catabolic process | Biological process | 5 | 205 | 75 | 13692 | 4.45268292682927 | 0.00529684407230406 | 0.105074047117561 | 2.27598281157161 | EPHA5//NTRK1//RGN//FOXJ1//VAV3 |
| GO:0033121 | regulation of purine nucleotide catabolic process | Biological process | 5 | 205 | 75 | 13692 | 4.45268292682927 | 0.00529684407230406 | 0.105074047117561 | 2.27598281157161 | EPHA5//NTRK1//RGN//FOXJ1//VAV3 |
| GO:0007243 | intracellular protein kinase cascade | Biological process | 10 | 713 | 75 | 13692 | 2.56044880785414 | 0.00532660211595795 | 0.105150629525067 | 2.27354974273039 | MDFIC//CLCF1//S100A4//LPAR1//C1QTNF3//MED1//C3//BMP4//NTRK1//SEMA7A |
| GO:2000145 | regulation of cell motility | Biological process | 7 | 389 | 75 | 13692 | 3.28514138817481 | 0.00534867481388914 | 0.105150629525067 | 2.2717538053255 | XCL1//BMP4//AQP1//ACE//ENPP2//LAMA2//LPAR1 |
| GO:0008406 | gonad development | Biological process | 5 | 206 | 75 | 13692 | 4.43106796116505 | 0.00540581378457581 | 0.105799498355269 | 2.26713891863065 | BMP4//MMP14//WNT10A//MGST1//NTRK1 |
| GO:0048523 | negative regulation of cellular process | Biological process | 23 | 2472 | 75 | 13692 | 1.69857605177994 | 0.00543898029365786 | 0.105975509366205 | 2.26448251470407 | BMP4//MSX1//FOXJ1//MED1//SNCA//APOA2//NTRK1//RERG//TRPV4//LPAR1//XCL1//MDFIC//SERTAD2//SOSTDC1//AQP1//WFS1//KRT18//CLCF1//LMX1A//C1QTNF3//NMB//MMP14//EPHA5 |
| GO:0000904 | cell morphogenesis involved in differentiation | Biological process | 8 | 495 | 75 | 13692 | 2.95046464646465 | 0.00556560622808401 | 0.107962910194338 | 2.25448752366129 | MSX1//BMP4//NTRK1//EPHA5//LMX1A//LAMA2//ROBO3//SEMA7A |
| GO:0009914 | hormone transport | Biological process | 5 | 208 | 75 | 13692 | 4.38846153846154 | 0.00562850837928025 | 0.108702117774293 | 2.24960668303102 | NMB//TRPV4//AQP1//EPHA5//SLCO1A5 |
| GO:0002704 | negative regulation of leukocyte mediated immunity | Biological process | 2 | 21 | 75 | 13692 | 17.3866666666667 | 0.00581204017787796 | 0.110302961644229 | 2.23567139214236 | XCL1//FOXJ1 |
| GO:0002707 | negative regulation of lymphocyte mediated immunity | Biological process | 2 | 21 | 75 | 13692 | 17.3866666666667 | 0.00581204017787796 | 0.110302961644229 | 2.23567139214236 | XCL1//FOXJ1 |
| GO:0030224 | monocyte differentiation | Biological process | 2 | 21 | 75 | 13692 | 17.3866666666667 | 0.00581204017787796 | 0.110302961644229 | 2.23567139214236 | BMP4//MED1 |
| GO:0045581 | negative regulation of T cell differentiation | Biological process | 2 | 21 | 75 | 13692 | 17.3866666666667 | 0.00581204017787796 | 0.110302961644229 | 2.23567139214236 | BMP4//FOXJ1 |
| GO:0050806 | positive regulation of synaptic transmission | Biological process | 3 | 67 | 75 | 13692 | 8.17432835820895 | 0.00588253989537949 | 0.110726723741879 | 2.23043511875723 | LAMA2//NTRK1//SNCA |
| GO:0046434 | organophosphate catabolic process | Biological process | 7 | 396 | 75 | 13692 | 3.22707070707071 | 0.00588488289960262 | 0.110726723741879 | 2.23026217454738 | RERG//EPHA5//NTRK1//RGN//FOXJ1//VAV3//PON1 |
| GO:0046660 | female sex differentiation | Biological process | 4 | 132 | 75 | 13692 | 5.53212121212121 | 0.00591656015159811 | 0.110847007284642 | 2.22793071580566 | BMP4//MMP14//WNT10A//MED1 |
| GO:0050865 | regulation of cell activation | Biological process | 6 | 300 | 75 | 13692 | 3.6512 | 0.00597768133387287 | 0.111046214370785 | 2.22346724062317 | VAV3//CLCF1//BMP4//FOXJ1//XCL1//SNCA |
| GO:0000902 | cell morphogenesis | Biological process | 10 | 725 | 75 | 13692 | 2.51806896551724 | 0.00597785278090907 | 0.111046214370785 | 2.22345478471763 | MSX1//BMP4//NTRK1//EPHA5//LMX1A//LAMA2//ROBO3//FOXJ1//SEMA7A//MED1 |
| GO:0030182 | neuron differentiation | Biological process | 11 | 846 | 75 | 13692 | 2.37371158392435 | 0.00609931908376694 | 0.112824535287908 | 2.21471864608247 | NTRK1//EPHA5//LMX1A//LAMA2//ROBO3//TRPV4//LPAR1//FSCN2//MED1//BMP4//SEMA7A |
| GO:0006575 | cellular modified amino acid metabolic process | Biological process | 4 | 134 | 75 | 13692 | 5.44955223880597 | 0.00623647964904075 | 0.114877003283171 | 2.2050604905132 | MGST1//SLC5A5//MED1//FOLR1 |
| GO:0051928 | positive regulation of calcium ion transport | Biological process | 3 | 69 | 75 | 13692 | 7.93739130434783 | 0.00638381375009462 | 0.117098909959895 | 2.19491979215954 | SNCA//XCL1//WFS1 |
| GO:0044248 | cellular catabolic process | Biological process | 14 | 1232 | 75 | 13692 | 2.07454545454545 | 0.00645753720173313 | 0.117957679551659 | 2.18993308318626 | RERG//HDC//APOA2//PON3//PON1//WFS1//EPHA5//NTRK1//RGN//FOXJ1//ENPP2//VAV3//ACE//CES1D |
| GO:2000027 | regulation of organ morphogenesis | Biological process | 4 | 136 | 75 | 13692 | 5.36941176470588 | 0.00656772293442192 | 0.11898075793074 | 2.18258517662396 | BMP4//MSX1//WNT10A//MED1 |
| GO:0030900 | forebrain development | Biological process | 6 | 306 | 75 | 13692 | 3.57960784313726 | 0.00656782468504539 | 0.11898075793074 | 2.18257844835718 | BMP4//MDK//LMX1A//AQP1//EPHA5//MSX1 |
| GO:0010646 | regulation of cell communication | Biological process | 15 | 1369 | 75 | 13692 | 2.00029218407597 | 0.00665476324139195 | 0.12005959691466 | 2.17686739094604 | SNCA//BMP4//EPHA5//NMB//MSX1//SOSTDC1//LAMA2//MED1//LPAR1//CLCF1//S100A4//NTRK1//C1QTNF3//C3//SEMA7A |
| GO:0050790 | regulation of catalytic activity | Biological process | 13 | 1108 | 75 | 13692 | 2.14194945848375 | 0.00673473116891515 | 0.120298037875277 | 2.17167973538077 | BMP4//LPAR1//SNCA//MDFIC//MMP14//CAMKK2//EPHA5//NTRK1//RGN//FOXJ1//VAV3//AQP1//APOA2 |
| GO:0008219 | cell death | Biological process | 15 | 1372 | 75 | 13692 | 1.99591836734694 | 0.0067883392967168 | 0.120298037875277 | 2.16823645878082 | RNF152//KRT18//DNAJB13//GULP1//SNCA//NTRK1//BMP4//LPAR1//MSX1//WFS1//ACE//AQP1//MED1//CLCF1 |
| GO:0033674 | positive regulation of kinase activity | Biological process | 6 | 309 | 75 | 13692 | 3.54485436893204 | 0.00687832931412673 | 0.120298037875277 | 2.16251703527697 | BMP4//LPAR1//MDFIC//CAMKK2//VAV3//SNCA |
| GO:0051971 | positive regulation of transmission of nerve impulse | Biological process | 3 | 71 | 75 | 13692 | 7.71380281690141 | 0.00690985681879576 | 0.120298037875277 | 2.16053095167807 | LAMA2//SNCA//NTRK1 |
| GO:0006972 | hyperosmotic response | Biological process | 2 | 23 | 75 | 13692 | 15.8747826086957 | 0.00695281158707094 | 0.120298037875277 | 2.15783953931913 | AQP1//TRPV4 |
| GO:0030104 | water homeostasis | Biological process | 2 | 23 | 75 | 13692 | 15.8747826086957 | 0.00695281158707094 | 0.120298037875277 | 2.15783953931913 | WFS1//AQP1 |
| GO:0042403 | thyroid hormone metabolic process | Biological process | 2 | 23 | 75 | 13692 | 15.8747826086957 | 0.00695281158707094 | 0.120298037875277 | 2.15783953931913 | SLC5A5//MED1 |
| GO:0042773 | ATP synthesis coupled electron transport | Biological process | 2 | 23 | 75 | 13692 | 15.8747826086957 | 0.00695281158707094 | 0.120298037875277 | 2.15783953931913 | COX8B//SNCA |
| GO:0071312 | cellular response to alkaloid | Biological process | 2 | 23 | 75 | 13692 | 15.8747826086957 | 0.00695281158707094 | 0.120298037875277 | 2.15783953931913 | NTRK1//MSX1 |
| GO:0071549 | cellular response to dexamethasone stimulus | Biological process | 2 | 23 | 75 | 13692 | 15.8747826086957 | 0.00695281158707094 | 0.120298037875277 | 2.15783953931913 | AQP1//BMP4 |
| GO:0016265 | death | Biological process | 15 | 1376 | 75 | 13692 | 1.99011627906977 | 0.0069698224498906 | 0.120298037875277 | 2.15677828503182 | RNF152//KRT18//DNAJB13//GULP1//SNCA//NTRK1//BMP4//LPAR1//MSX1//WFS1//ACE//AQP1//MED1//CLCF1 |
| GO:0060548 | negative regulation of cell death | Biological process | 9 | 626 | 75 | 13692 | 2.62466453674121 | 0.0070043527364051 | 0.120419930966274 | 2.15463199114129 | SNCA//AQP1//BMP4//MSX1//WFS1//KRT18//MED1//NTRK1//CLCF1 |
| GO:0032103 | positive regulation of response to external stimulus | Biological process | 4 | 139 | 75 | 13692 | 5.25352517985612 | 0.00708618972648009 | 0.121350999065972 | 2.14958722399546 | C3//XCL1//ACE//LPAR1 |
| GO:0051270 | regulation of cellular component movement | Biological process | 7 | 412 | 75 | 13692 | 3.10174757281553 | 0.00726139594650433 | 0.12344622761107 | 2.13897988156071 | XCL1//BMP4//AQP1//ACE//ENPP2//LAMA2//LPAR1 |
| GO:0035023 | regulation of Rho protein signal transduction | Biological process | 4 | 140 | 75 | 13692 | 5.216 | 0.0072648555482792 | 0.12344622761107 | 2.13877301663216 | EPHA5//FOXJ1//LPAR1//VAV3 |
| GO:0001525 | angiogenesis | Biological process | 6 | 314 | 75 | 13692 | 3.4884076433121 | 0.00741939492751882 | 0.125585433831052 | 2.12963151121082 | BMP4//C3//AQP1//NTRK1//MMP14//MED1 |
| GO:0006638 | neutral lipid metabolic process | Biological process | 3 | 73 | 75 | 13692 | 7.50246575342466 | 0.00746096490854029 | 0.125803346765541 | 2.1272050026405 | APOA2//CES1D//SNCA |
| GO:0006767 | water-soluble vitamin metabolic process | Biological process | 2 | 24 | 75 | 13692 | 15.2133333333333 | 0.00755814576647191 | 0.126953682146409 | 2.12158473653068 | RGN//FOLR1 |
| GO:0002449 | lymphocyte mediated immunity | Biological process | 4 | 142 | 75 | 13692 | 5.14253521126761 | 0.00763107489870114 | 0.127689436472923 | 2.11741428384131 | C3//XCL1//FOXJ1//CLCF1 |
| GO:0031646 | positive regulation of neurological system process | Biological process | 3 | 75 | 75 | 13692 | 7.3024 | 0.00803741547861922 | 0.133977298320406 | 2.09488358108014 | LAMA2//SNCA//NTRK1 |
| GO:0002920 | regulation of humoral immune response | Biological process | 2 | 25 | 75 | 13692 | 14.6048 | 0.00818642520067858 | 0.135431275772735 | 2.0869057019687 | C3//FOXJ1 |
| GO:0010524 | positive regulation of calcium ion transport into cytosol | Biological process | 2 | 25 | 75 | 13692 | 14.6048 | 0.00818642520067858 | 0.135431275772735 | 2.0869057019687 | SNCA//XCL1 |
| GO:0007409 | axonogenesis | Biological process | 6 | 321 | 75 | 13692 | 3.41233644859813 | 0.00822791293626629 | 0.135605903430795 | 2.08471031244391 | NTRK1//EPHA5//LMX1A//LAMA2//ROBO3//SEMA7A |
| GO:0048666 | neuron development | Biological process | 9 | 644 | 75 | 13692 | 2.55130434782609 | 0.00836950871010781 | 0.13742294451353 | 2.07730003433067 | NTRK1//EPHA5//LMX1A//LAMA2//ROBO3//TRPV4//LPAR1//FSCN2//SEMA7A |
| GO:0051347 | positive regulation of transferase activity | Biological process | 6 | 323 | 75 | 13692 | 3.39120743034056 | 0.0084701616230294 | 0.138556673714033 | 2.07210830261851 | BMP4//LPAR1//MDFIC//CAMKK2//VAV3//SNCA |
| GO:0006066 | alcohol metabolic process | Biological process | 5 | 230 | 75 | 13692 | 3.96869565217391 | 0.00852297761481648 | 0.138902356369351 | 2.0694086522091 | APOA2//PON1//ENPP2//SNCA//HDC |
| GO:0048519 | negative regulation of biological process | Biological process | 24 | 2719 | 75 | 13692 | 1.61141596175064 | 0.00862811239356854 | 0.140094980494091 | 2.06408420622263 | BMP4//MSX1//FOXJ1//MED1//SNCA//XCL1//APOA2//NTRK1//RERG//TRPV4//LPAR1//MDFIC//SERTAD2//SOSTDC1//F12//AQP1//WFS1//KRT18//CLCF1//LMX1A//C1QTNF3//NMB//MMP14//EPHA5 |
| GO:0046578 | regulation of Ras protein signal transduction | Biological process | 5 | 231 | 75 | 13692 | 3.95151515151515 | 0.00867504822139562 | 0.140337311448703 | 2.06172810244743 | EPHA5//NTRK1//FOXJ1//VAV3//LPAR1 |
| GO:0019439 | aromatic compound catabolic process | Biological process | 2 | 26 | 75 | 13692 | 14.0430769230769 | 0.00883738726780846 | 0.141398196284935 | 2.05367611314197 | PON3//PON1 |
| GO:0044058 | regulation of digestive system process | Biological process | 2 | 26 | 75 | 13692 | 14.0430769230769 | 0.00883738726780846 | 0.141398196284935 | 2.05367611314197 | AQP1//APOA2 |
| GO:0046785 | microtubule polymerization | Biological process | 2 | 26 | 75 | 13692 | 14.0430769230769 | 0.00883738726780846 | 0.141398196284935 | 2.05367611314197 | SNCA//TRPV4 |
| GO:0022414 | reproductive process | Biological process | 12 | 1018 | 75 | 13692 | 2.15198428290766 | 0.00894336658051499 | 0.142165042633053 | 2.04849896754448 | BMP4//MMP14//ACE//FOXJ1//WIPF3//DNAJB13//SOSTDC1//MED1//WNT10A//MGST1//MDFIC//NTRK1 |
| GO:0009799 | specification of symmetry | Biological process | 3 | 78 | 75 | 13692 | 7.02153846153846 | 0.00895017147963563 | 0.142165042633053 | 2.04816864379626 | FOXJ1//CCDC40//BMP4 |
| GO:0045137 | development of primary sexual characteristics | Biological process | 5 | 234 | 75 | 13692 | 3.9008547008547 | 0.00914255442109189 | 0.144696601379303 | 2.03893244584258 | BMP4//MMP14//WNT10A//MGST1//NTRK1 |
| GO:0015718 | monocarboxylic acid transport | Biological process | 3 | 79 | 75 | 13692 | 6.9326582278481 | 0.00926736482657118 | 0.145895792614408 | 2.0330437398549 | SLCO1A5//ACE//NMB |
| GO:0051249 | regulation of lymphocyte activation | Biological process | 5 | 236 | 75 | 13692 | 3.86779661016949 | 0.00946374879373658 | 0.145895792614408 | 2.02393679616351 | VAV3//CLCF1//BMP4//FOXJ1//XCL1 |
| GO:0051094 | positive regulation of developmental process | Biological process | 9 | 657 | 75 | 13692 | 2.50082191780822 | 0.00947592457494775 | 0.145895792614408 | 2.02337840476801 | BMP4//MED1//C3//AQP1//NTRK1//SEMA7A//CLCF1//ACE//LPAR1 |
| GO:0003401 | axis elongation | Biological process | 2 | 27 | 75 | 13692 | 13.522962962963 | 0.00951077146754769 | 0.145895792614408 | 2.02178425377657 | BMP4//MED1 |
| GO:0030513 | positive regulation of BMP signaling pathway | Biological process | 2 | 27 | 75 | 13692 | 13.522962962963 | 0.00951077146754769 | 0.145895792614408 | 2.02178425377657 | BMP4//MSX1 |
| GO:0046579 | positive regulation of Ras protein signal transduction | Biological process | 2 | 27 | 75 | 13692 | 13.522962962963 | 0.00951077146754769 | 0.145895792614408 | 2.02178425377657 | LPAR1//NTRK1 |
| GO:0070613 | regulation of protein processing | Biological process | 2 | 27 | 75 | 13692 | 13.522962962963 | 0.00951077146754769 | 0.145895792614408 | 2.02178425377657 | C3//F12 |
| GO:0022603 | regulation of anatomical structure morphogenesis | Biological process | 8 | 543 | 75 | 13692 | 2.68965009208103 | 0.00951783683570271 | 0.145895792614408 | 2.02146174458468 | BMP4//MSX1//WNT10A//C3//AQP1//NTRK1//SEMA7A//MED1 |
| GO:0031175 | neuron projection development | Biological process | 8 | 543 | 75 | 13692 | 2.68965009208103 | 0.00951783683570271 | 0.145895792614408 | 2.02146174458468 | NTRK1//EPHA5//LMX1A//LAMA2//ROBO3//TRPV4//LPAR1//SEMA7A |
| GO:0042113 | B cell activation | Biological process | 4 | 152 | 75 | 13692 | 4.80421052631579 | 0.00964503087891536 | 0.147330367153885 | 2.01569637760837 | NTRK1//CLCF1//VAV3//FOXJ1 |
| GO:0002460 | adaptive immune response based on somatic recombination of immune receptors built from immunoglobulin superfamily domains | Biological process | 4 | 153 | 75 | 13692 | 4.77281045751634 | 0.00986364033976625 | 0.14914834759064 | 2.00596277190529 | C3//XCL1//FOXJ1//CLCF1 |
| GO:0007586 | digestion | Biological process | 3 | 81 | 75 | 13692 | 6.76148148148148 | 0.00992133075835799 | 0.14914834759064 | 2.00343007157062 | AQP1//APOA2//SLCO1A5 |
| GO:0050864 | regulation of B cell activation | Biological process | 3 | 81 | 75 | 13692 | 6.76148148148148 | 0.00992133075835799 | 0.14914834759064 | 2.00343007157062 | VAV3//CLCF1//FOXJ1 |
| GO:0032989 | cellular component morphogenesis | Biological process | 10 | 783 | 75 | 13692 | 2.33154533844189 | 0.0100546446548971 | 0.14914834759064 | 1.99763327336603 | MSX1//MED1//BMP4//NTRK1//EPHA5//LMX1A//LAMA2//ROBO3//FOXJ1//SEMA7A |
| GO:0048856 | anatomical structure development | Biological process | 29 | 3546 | 75 | 13692 | 1.49301748448957 | 0.0101502354208485 | 0.14914834759064 | 1.99352388476656 | MSX1//MED1//BMP4//MMP14//RPE65//ROBO3//ACE//WFS1//UGT1A6//WNT10A//FOXJ1//C3//NTRK1//EPHA5//LMX1A//LAMA2//TRPV4//LPAR1//MDK//AQP1//CLCF1//PRPS2//APOA2//SLCO1A5//MGST1//FSCN2//SOSTDC1//SEMA7A//COL8A1 |
| GO:0030858 | positive regulation of epithelial cell differentiation | Biological process | 2 | 28 | 75 | 13692 | 13.04 | 0.0102063194062938 | 0.14914834759064 | 1.99113084456539 | BMP4//MED1 |
| GO:0032309 | icosanoid secretion | Biological process | 2 | 28 | 75 | 13692 | 13.04 | 0.0102063194062938 | 0.14914834759064 | 1.99113084456539 | ACE//NMB |
| GO:0032371 | regulation of sterol transport | Biological process | 2 | 28 | 75 | 13692 | 13.04 | 0.0102063194062938 | 0.14914834759064 | 1.99113084456539 | PON1//APOA2 |
| GO:0032374 | regulation of cholesterol transport | Biological process | 2 | 28 | 75 | 13692 | 13.04 | 0.0102063194062938 | 0.14914834759064 | 1.99113084456539 | PON1//APOA2 |
| GO:0032410 | negative regulation of transporter activity | Biological process | 2 | 28 | 75 | 13692 | 13.04 | 0.0102063194062938 | 0.14914834759064 | 1.99113084456539 | APOA2//SNCA |
| GO:0045620 | negative regulation of lymphocyte differentiation | Biological process | 2 | 28 | 75 | 13692 | 13.04 | 0.0102063194062938 | 0.14914834759064 | 1.99113084456539 | BMP4//FOXJ1 |
| GO:0051414 | response to cortisol stimulus | Biological process | 2 | 28 | 75 | 13692 | 13.04 | 0.0102063194062938 | 0.14914834759064 | 1.99113084456539 | AQP1//BMP4 |
| GO:0071548 | response to dexamethasone stimulus | Biological process | 2 | 28 | 75 | 13692 | 13.04 | 0.0102063194062938 | 0.14914834759064 | 1.99113084456539 | AQP1//BMP4 |
| GO:0003014 | renal system process | Biological process | 3 | 82 | 75 | 13692 | 6.6790243902439 | 0.0102581554487583 | 0.149178772556609 | 1.98893072406278 | WFS1//AQP1//BMP4 |
| GO:0043523 | regulation of neuron apoptotic process | Biological process | 4 | 155 | 75 | 13692 | 4.71122580645161 | 0.0103104854207693 | 0.149178772556609 | 1.9867208875197 | SNCA//NTRK1//WFS1//CLCF1 |
| GO:1901214 | regulation of neuron death | Biological process | 4 | 155 | 75 | 13692 | 4.71122580645161 | 0.0103104854207693 | 0.149178772556609 | 1.9867208875197 | SNCA//NTRK1//WFS1//CLCF1 |
| GO:0007610 | behavior | Biological process | 8 | 552 | 75 | 13692 | 2.64579710144928 | 0.0104477234045545 | 0.150259672713048 | 1.98097833352936 | MDK//XCL1//NTRK1//SNCA//NMB//WFS1//ACE//LPAR1 |
| GO:0009116 | nucleoside metabolic process | Biological process | 6 | 338 | 75 | 13692 | 3.2407100591716 | 0.0104537409163959 | 0.150259672713048 | 1.98072826757995 | RERG//EPHA5//NTRK1//FOXJ1//VAV3//PRPS2 |
| GO:0021761 | limbic system development | Biological process | 3 | 83 | 75 | 13692 | 6.59855421686747 | 0.0106015743301032 | 0.151886607395988 | 1.97462963736658 | MDK//LMX1A//EPHA5 |
| GO:0080134 | regulation of response to stress | Biological process | 8 | 554 | 75 | 13692 | 2.63624548736462 | 0.0106632694593144 | 0.152272877230079 | 1.97210961609386 | C3//F12//MDFIC//TRPV4//XCL1//SEMA7A//ACE//MED1 |
| GO:0006163 | purine nucleotide metabolic process | Biological process | 8 | 555 | 75 | 13692 | 2.6314954954955 | 0.0107722791300774 | 0.153298732727514 | 1.96769240172527 | PRPS2//AQP1//RERG//EPHA5//NTRK1//RGN//FOXJ1//VAV3 |
| GO:0034762 | regulation of transmembrane transport | Biological process | 5 | 244 | 75 | 13692 | 3.74098360655738 | 0.0108266183025333 | 0.153298732727514 | 1.965507174205 | MED1//SCN4B//MDFIC//BMP4//APOA2 |
| GO:0008285 | negative regulation of cell proliferation | Biological process | 7 | 445 | 75 | 13692 | 2.87173033707865 | 0.0108400107539985 | 0.153298732727514 | 1.96497028694893 | MED1//BMP4//FOXJ1//XCL1//NTRK1//MSX1//RERG |
| GO:0009651 | response to salt stress | Biological process | 2 | 29 | 75 | 13692 | 12.5903448275862 | 0.0109237747823941 | 0.153466307235258 | 1.96162726237446 | AQP1//TRPV4 |
| GO:0048593 | camera-type eye morphogenesis | Biological process | 3 | 84 | 75 | 13692 | 6.52 | 0.0109516109307293 | 0.153466307235258 | 1.9605219934386 | BMP4//AQP1//COL8A1 |
| GO:0032501 | multicellular organismal process | Biological process | 42 | 5780 | 75 | 13692 | 1.32656055363322 | 0.0109568782309844 | 0.153466307235258 | 1.96031316484595 | BMP4//MMP14//MED1//RPE65//MDK//MSX1//ROBO3//ACE//WFS1//UGT1A6//WNT10A//FOXJ1//SNCA//C3//XCL1//APOA2//AQP1//F5//F12//WIPF3//PRTG//ZAR1//DNAJB13//SOSTDC1//NTRK1//EPHA5//LMX1A//LAMA2//NMB//TRPV4//LPAR1//CLDN19//CLCF1//PRPS2//SLCO1A5//MGST1//FSCN2//SEMA7A//COL8A1//NTS//SCN4B//CCDC40 |
| GO:0009893 | positive regulation of metabolic process | Biological process | 17 | 1731 | 75 | 13692 | 1.79290583477759 | 0.0111133231847073 | 0.155161811597952 | 1.95415605582284 | BMP4//LPAR1//SNCA//C3//F12//MDFIC//WNT10A//MED1//APOA2//MDK//SERTAD2//WFS1//CAMKK2//CLCF1//VAV3//MSX1//FOXJ1 |
| GO:0071844 | cellular component assembly at cellular level | Biological process | 11 | 923 | 75 | 13692 | 2.17568797399783 | 0.0114017600231563 | 0.158683542671483 | 1.94302810398403 | TRPV4//AQP1//VAV3//BAIAP2L1//SNCA//LPAR1//FOXJ1//CLDN19//MMP14//EPHA5//CCDC40 |
| GO:0044092 | negative regulation of molecular function | Biological process | 8 | 562 | 75 | 13692 | 2.59871886120996 | 0.0115587784189253 | 0.160359761356229 | 1.93708806158617 | FOXJ1//SNCA//AQP1//MSX1//BMP4//WFS1//XCL1//APOA2 |
| GO:0010604 | positive regulation of macromolecule metabolic process | Biological process | 16 | 1599 | 75 | 13692 | 1.82674171357098 | 0.0117343280865525 | 0.162281685588158 | 1.93054177327092 | BMP4//LPAR1//SNCA//C3//F12//MDFIC//WNT10A//MED1//MDK//SERTAD2//WFS1//CAMKK2//CLCF1//APOA2//MSX1//FOXJ1 |
| GO:0010720 | positive regulation of cell development | Biological process | 4 | 162 | 75 | 13692 | 4.50765432098765 | 0.0119773243441537 | 0.16497057216336 | 1.92164018965572 | SEMA7A//CLCF1//ACE//BMP4 |
| GO:0034329 | cell junction assembly | Biological process | 3 | 87 | 75 | 13692 | 6.2951724137931 | 0.012041647603165 | 0.16497057216336 | 1.91931408649943 | TRPV4//CLDN19//MMP14 |
| GO:0051250 | negative regulation of lymphocyte activation | Biological process | 3 | 87 | 75 | 13692 | 6.2951724137931 | 0.012041647603165 | 0.16497057216336 | 1.91931408649943 | BMP4//FOXJ1//XCL1 |
| GO:0003007 | heart morphogenesis | Biological process | 4 | 163 | 75 | 13692 | 4.48 | 0.0122287303298984 | 0.166251564103444 | 1.91261863203514 | FOXJ1//BMP4//MSX1//MED1 |
| GO:0006184 | GTP catabolic process | Biological process | 5 | 252 | 75 | 13692 | 3.62222222222222 | 0.0123185433395637 | 0.166251564103444 | 1.90944064420012 | EPHA5//NTRK1//FOXJ1//VAV3//RERG |
| GO:0022607 | cellular component assembly | Biological process | 14 | 1330 | 75 | 13692 | 1.92168421052632 | 0.0123319839512928 | 0.166251564103444 | 1.90896704913119 | TRPV4//AQP1//VAV3//BAIAP2L1//SNCA//LPAR1//APOA2//FOXJ1//CLDN19//C1QTNF3//MMP14//EPHA5//MGST1//CCDC40 |
| GO:0009395 | phospholipid catabolic process | Biological process | 2 | 31 | 75 | 13692 | 11.778064516129 | 0.012423393011893 | 0.166251564103444 | 1.90575977572969 | ENPP2//APOA2 |
| GO:0051148 | negative regulation of muscle cell differentiation | Biological process | 2 | 31 | 75 | 13692 | 11.778064516129 | 0.012423393011893 | 0.166251564103444 | 1.90575977572969 | BMP4//MSX1 |
| GO:0060996 | dendritic spine development | Biological process | 2 | 31 | 75 | 13692 | 11.778064516129 | 0.012423393011893 | 0.166251564103444 | 1.90575977572969 | EPHA5//LPAR1 |
| GO:0071320 | cellular response to cAMP | Biological process | 2 | 31 | 75 | 13692 | 11.778064516129 | 0.012423393011893 | 0.166251564103444 | 1.90575977572969 | AQP1//SLC5A5 |
| GO:0002250 | adaptive immune response | Biological process | 4 | 164 | 75 | 13692 | 4.45268292682927 | 0.0124835060645485 | 0.166251564103444 | 1.90366342341625 | C3//XCL1//FOXJ1//CLCF1 |
| GO:0032147 | activation of protein kinase activity | Biological process | 4 | 164 | 75 | 13692 | 4.45268292682927 | 0.0124835060645485 | 0.166251564103444 | 1.90366342341625 | BMP4//LPAR1//MDFIC//CAMKK2 |
| GO:0001558 | regulation of cell growth | Biological process | 5 | 253 | 75 | 13692 | 3.60790513833992 | 0.0125143741227501 | 0.166251564103444 | 1.90259086573878 | MSX1//SERTAD2//RERG//SEMA7A//LMX1A |
| GO:0048468 | cell development | Biological process | 14 | 1333 | 75 | 13692 | 1.91735933983496 | 0.0125624558297647 | 0.166386121926551 | 1.9009254522343 | MSX1//BMP4//NTRK1//EPHA5//LMX1A//LAMA2//ROBO3//TRPV4//LPAR1//MED1//FSCN2//SEMA7A//CLCF1//ACE |
| GO:1901069 | guanosine-containing compound catabolic process | Biological process | 5 | 254 | 75 | 13692 | 3.59370078740157 | 0.0127123143680907 | 0.167863813824427 | 1.89577537581964 | RERG//EPHA5//NTRK1//FOXJ1//VAV3 |
| GO:0045766 | positive regulation of angiogenesis | Biological process | 3 | 89 | 75 | 13692 | 6.15370786516854 | 0.0128018148298079 | 0.168538006648282 | 1.89272845889107 | C3//AQP1//NTRK1 |
| GO:0006152 | purine nucleoside catabolic process | Biological process | 5 | 255 | 75 | 13692 | 3.57960784313725 | 0.0129123740676671 | 0.16897865048553 | 1.88899390105675 | RERG//EPHA5//NTRK1//FOXJ1//VAV3 |
| GO:0046130 | purine ribonucleoside catabolic process | Biological process | 5 | 255 | 75 | 13692 | 3.57960784313725 | 0.0129123740676671 | 0.16897865048553 | 1.88899390105675 | RERG//EPHA5//NTRK1//FOXJ1//VAV3 |
| GO:0042981 | regulation of apoptotic process | Biological process | 12 | 1070 | 75 | 13692 | 2.04740186915888 | 0.0129795416589342 | 0.169352114978475 | 1.88674064332226 | XCL1//SNCA//ACE//BMP4//MSX1//LPAR1//AQP1//WFS1//KRT18//MED1//NTRK1//CLCF1 |
| GO:0006812 | cation transport | Biological process | 8 | 575 | 75 | 13692 | 2.5399652173913 | 0.0131315880278538 | 0.170267514527896 | 1.88168275067894 | AQP1//SLC4A4//SLC5A5//SCN4B//TRPV4//XCL1//SNCA//WFS1 |
| GO:0051057 | positive regulation of small GTPase mediated signal transduction | Biological process | 2 | 32 | 75 | 13692 | 11.41 | 0.0132050535902109 | 0.170267514527896 | 1.87925983195152 | LPAR1//NTRK1 |
| GO:0051279 | regulation of release of sequestered calcium ion into cytosol | Biological process | 2 | 32 | 75 | 13692 | 11.41 | 0.0132050535902109 | 0.170267514527896 | 1.87925983195152 | SNCA//XCL1 |
| GO:0071715 | icosanoid transport | Biological process | 2 | 32 | 75 | 13692 | 11.41 | 0.0132050535902109 | 0.170267514527896 | 1.87925983195152 | ACE//NMB |
| GO:0007266 | Rho protein signal transduction | Biological process | 4 | 168 | 75 | 13692 | 4.34666666666667 | 0.0135366143643166 | 0.174030842736551 | 1.86848994323991 | VAV3//LPAR1 |
| GO:0045595 | regulation of cell differentiation | Biological process | 11 | 947 | 75 | 13692 | 2.12054910242872 | 0.0136296850536645 | 0.174715027120658 | 1.86551417945776 | NTRK1//TRPV4//LPAR1//FOXJ1//BMP4//MED1//LMX1A//SEMA7A//CLCF1//ACE//MSX1 |
| GO:0042454 | ribonucleoside catabolic process | Biological process | 5 | 259 | 75 | 13692 | 3.52432432432432 | 0.0137340050295918 | 0.175539003060439 | 1.86220279789685 | RERG//EPHA5//NTRK1//FOXJ1//VAV3 |
| GO:0009056 | catabolic process | Biological process | 15 | 1487 | 75 | 13692 | 1.84156018829859 | 0.0137954718706499 | 0.17581206011898 | 1.86026343999708 | RERG//HDC//APOA2//PON3//LYZL4//PON1//WFS1//EPHA5//NTRK1//RGN//FOXJ1//ENPP2//ACE//VAV3//CES1D |
| GO:0043067 | regulation of programmed cell death | Biological process | 12 | 1080 | 75 | 13692 | 2.02844444444444 | 0.0138961699777201 | 0.176463773119982 | 1.85710488224559 | XCL1//SNCA//MSX1//WFS1//ACE//BMP4//LPAR1//AQP1//KRT18//MED1//NTRK1//CLCF1 |
| GO:0006119 | oxidative phosphorylation | Biological process | 2 | 33 | 75 | 13692 | 11.0642424242424 | 0.0140076170268599 | 0.176463773119982 | 1.85363574052506 | COX8B//SNCA |
| GO:0046688 | response to copper ion | Biological process | 2 | 33 | 75 | 13692 | 11.0642424242424 | 0.0140076170268599 | 0.176463773119982 | 1.85363574052506 | AQP1//SNCA |
| GO:0046717 | acid secretion | Biological process | 2 | 33 | 75 | 13692 | 11.0642424242424 | 0.0140076170268599 | 0.176463773119982 | 1.85363574052506 | ACE//NMB |
| GO:0009612 | response to mechanical stimulus | Biological process | 4 | 170 | 75 | 13692 | 4.29552941176471 | 0.0140837813622892 | 0.176713380394326 | 1.85128072557064 | AQP1//NTRK1//BMP4//MMP14 |
| GO:0006195 | purine nucleotide catabolic process | Biological process | 6 | 361 | 75 | 13692 | 3.03423822714681 | 0.0141080481610434 | 0.176713380394326 | 1.85053306643865 | RERG//EPHA5//NTRK1//RGN//FOXJ1//VAV3 |
| GO:0009164 | nucleoside catabolic process | Biological process | 5 | 261 | 75 | 13692 | 3.49731800766284 | 0.0141577923391632 | 0.176831229672055 | 1.84900446201825 | RERG//EPHA5//NTRK1//FOXJ1//VAV3 |
| GO:0002376 | immune system process | Biological process | 13 | 1218 | 75 | 13692 | 1.94850574712644 | 0.0143087644834055 | 0.178209157656959 | 1.84439786457737 | SNCA//C3//XCL1//BMP4//ACE//FOXJ1//CLCF1//APOA2//ENPP2//NTRK1//MED1//VAV3//SEMA7A |
| GO:0051056 | regulation of small GTPase mediated signal transduction | Biological process | 5 | 262 | 75 | 13692 | 3.48396946564886 | 0.0143729626257327 | 0.178501609493519 | 1.8424537037243 | EPHA5//NTRK1//FOXJ1//VAV3//LPAR1 |
| GO:0030029 | actin filament-based process | Biological process | 6 | 363 | 75 | 13692 | 3.01752066115702 | 0.0144631763637053 | 0.179114590899672 | 1.83973631798616 | FOXJ1//TRPV4//BAIAP2L1//AQP1//EPHA5 |
| GO:0051402 | neuron apoptotic process | Biological process | 4 | 172 | 75 | 13692 | 4.24558139534884 | 0.0146448449203117 | 0.180853521494779 | 1.83431522286319 | SNCA//NTRK1//WFS1//CLCF1 |
| GO:0002455 | humoral immune response mediated by circulating immunoglobulin | Biological process | 2 | 34 | 75 | 13692 | 10.7388235294118 | 0.0148308372618248 | 0.181615616077765 | 1.82883433056795 | FOXJ1//C3 |
| GO:0030032 | lamellipodium assembly | Biological process | 2 | 34 | 75 | 13692 | 10.7388235294118 | 0.0148308372618248 | 0.181615616077765 | 1.82883433056795 | AQP1//VAV3 |
| GO:0033344 | cholesterol efflux | Biological process | 2 | 34 | 75 | 13692 | 10.7388235294118 | 0.0148308372618248 | 0.181615616077765 | 1.82883433056795 | PON1//APOA2 |
| GO:0072523 | purine-containing compound catabolic process | Biological process | 6 | 366 | 75 | 13692 | 2.9927868852459 | 0.0150075448707902 | 0.183052622710951 | 1.82369034948084 | RERG//EPHA5//NTRK1//RGN//FOXJ1//VAV3 |
| GO:0046039 | GTP metabolic process | Biological process | 5 | 265 | 75 | 13692 | 3.44452830188679 | 0.0150316934707898 | 0.183052622710951 | 1.82299208903458 | RERG//EPHA5//NTRK1//FOXJ1//VAV3 |
| GO:0030509 | BMP signaling pathway | Biological process | 3 | 95 | 75 | 13692 | 5.76505263157895 | 0.0152445489495356 | 0.184619067941337 | 1.81688542086408 | BMP4//MSX1//SOSTDC1 |
| GO:0050671 | positive regulation of lymphocyte proliferation | Biological process | 3 | 95 | 75 | 13692 | 5.76505263157895 | 0.0152445489495356 | 0.184619067941337 | 1.81688542086408 | VAV3//CLCF1//XCL1 |
| GO:0006952 | defense response | Biological process | 9 | 711 | 75 | 13692 | 2.31088607594937 | 0.0153009291987003 | 0.184791387347389 | 1.81528219443635 | MDK//C3//F12//APOA2//SNCA//XCL1//SEMA7A//ACE//MED1 |
| GO:0051130 | positive regulation of cellular component organization | Biological process | 7 | 478 | 75 | 13692 | 2.67347280334728 | 0.0155644268111449 | 0.185733331507617 | 1.80786686838593 | AQP1//NTRK1//BAIAP2L1//TRPV4//SEMA7A//SNCA//C3 |
| GO:0002637 | regulation of immunoglobulin production | Biological process | 2 | 35 | 75 | 13692 | 10.432 | 0.0156744702404497 | 0.185733331507617 | 1.80480712836247 | CLCF1//XCL1 |
| GO:0022904 | respiratory electron transport chain | Biological process | 2 | 35 | 75 | 13692 | 10.432 | 0.0156744702404497 | 0.185733331507617 | 1.80480712836247 | COX8B//SNCA |
| GO:0030890 | positive regulation of B cell proliferation | Biological process | 2 | 35 | 75 | 13692 | 10.432 | 0.0156744702404497 | 0.185733331507617 | 1.80480712836247 | VAV3//CLCF1 |
| GO:0030901 | midbrain development | Biological process | 2 | 35 | 75 | 13692 | 10.432 | 0.0156744702404497 | 0.185733331507617 | 1.80480712836247 | MSX1//LMX1A |
| GO:0071560 | cellular response to transforming growth factor beta stimulus | Biological process | 2 | 35 | 75 | 13692 | 10.432 | 0.0156744702404497 | 0.185733331507617 | 1.80480712836247 | XCL1//WNT10A |
| GO:0032946 | positive regulation of mononuclear cell proliferation | Biological process | 3 | 96 | 75 | 13692 | 5.705 | 0.0156754864639184 | 0.185733331507617 | 1.80477897264607 | VAV3//CLCF1//XCL1 |
| GO:0009166 | nucleotide catabolic process | Biological process | 6 | 370 | 75 | 13692 | 2.96043243243243 | 0.0157554372801275 | 0.186177458318272 | 1.80256953881108 | RERG//EPHA5//NTRK1//RGN//FOXJ1//VAV3 |
| GO:0007267 | cell-cell signaling | Biological process | 9 | 715 | 75 | 13692 | 2.29795804195804 | 0.0158191520287969 | 0.186427856167327 | 1.80081680017365 | BMP4//SNCA//NMB//TRPV4//LAMA2//AQP1//EPHA5//NTRK1//XCL1 |
| GO:0048858 | cell projection morphogenesis | Biological process | 7 | 480 | 75 | 13692 | 2.66233333333333 | 0.0158914776771764 | 0.186778118329065 | 1.79883571782055 | NTRK1//EPHA5//LMX1A//LAMA2//ROBO3//FOXJ1//SEMA7A |
| GO:0006954 | inflammatory response | Biological process | 6 | 371 | 75 | 13692 | 2.95245283018868 | 0.0159463937999001 | 0.186922434274765 | 1.79733751510709 | C3//F12//APOA2//XCL1//SEMA7A//ACE |
| GO:0072521 | purine-containing compound metabolic process | Biological process | 8 | 597 | 75 | 13692 | 2.44636515912898 | 0.0161452921759886 | 0.188749229065424 | 1.79195409129275 | PRPS2//AQP1//RERG//EPHA5//NTRK1//RGN//FOXJ1//VAV3 |
| GO:0045321 | leukocyte activation | Biological process | 7 | 483 | 75 | 13692 | 2.64579710144928 | 0.0163912319449257 | 0.190855354390603 | 1.78538840416124 | SNCA//NTRK1//CLCF1//VAV3//BMP4//FOXJ1//XCL1 |
| GO:0045926 | negative regulation of growth | Biological process | 4 | 178 | 75 | 13692 | 4.10247191011236 | 0.0164125156490094 | 0.190855354390603 | 1.78482484681106 | MSX1//SERTAD2//RERG//BMP4 |
| GO:0010977 | negative regulation of neuron projection development | Biological process | 2 | 36 | 75 | 13692 | 10.1422222222222 | 0.0165382738993305 | 0.191302883310462 | 1.78150981976016 | TRPV4//LPAR1 |
| GO:0042401 | cellular biogenic amine biosynthetic process | Biological process | 2 | 36 | 75 | 13692 | 10.1422222222222 | 0.0165382738993305 | 0.191302883310462 | 1.78150981976016 | HDC//SNCA |
| GO:0031099 | regeneration | Biological process | 4 | 180 | 75 | 13692 | 4.05688888888889 | 0.0170302491512064 | 0.196355119286138 | 1.76877899829679 | PRPS2//APOA2//SLCO1A5//MED1 |
| GO:0035556 | intracellular signal transduction | Biological process | 15 | 1527 | 75 | 13692 | 1.79332023575639 | 0.0172640066452622 | 0.196355119286138 | 1.76285840549248 | MDFIC//CLCF1//VAV3//RERG//EPHA5//LPAR1//S100A4//NTRK1//RGN//C1QTNF3//MED1//C3//BMP4//SEMA7A |
| GO:0048667 | cell morphogenesis involved in neuron differentiation | Biological process | 6 | 378 | 75 | 13692 | 2.89777777777778 | 0.0173284102047547 | 0.196355119286138 | 1.76124127981197 | NTRK1//EPHA5//LMX1A//LAMA2//ROBO3//SEMA7A |
| GO:0043087 | regulation of GTPase activity | Biological process | 4 | 181 | 75 | 13692 | 4.03447513812155 | 0.0173445179275141 | 0.196355119286138 | 1.76083776640883 | EPHA5//NTRK1//FOXJ1//VAV3 |
| GO:0070997 | neuron death | Biological process | 4 | 181 | 75 | 13692 | 4.03447513812155 | 0.0173445179275141 | 0.196355119286138 | 1.76083776640883 | SNCA//NTRK1//WFS1//CLCF1 |
| GO:0031344 | regulation of cell projection organization | Biological process | 5 | 275 | 75 | 13692 | 3.31927272727273 | 0.0173734642084633 | 0.196355119286138 | 1.76011357610586 | AQP1//NTRK1//TRPV4//LPAR1//SEMA7A |
| GO:0002381 | immunoglobulin production involved in immunoglobulin mediated immune response | Biological process | 2 | 37 | 75 | 13692 | 9.86810810810811 | 0.017422008152299 | 0.196355119286138 | 1.75890178737673 | XCL1//CLCF1 |
| GO:0007043 | cell-cell junction assembly | Biological process | 2 | 37 | 75 | 13692 | 9.86810810810811 | 0.017422008152299 | 0.196355119286138 | 1.75890178737673 | CLDN19//TRPV4 |
| GO:0072210 | metanephric nephron development | Biological process | 2 | 37 | 75 | 13692 | 9.86810810810811 | 0.017422008152299 | 0.196355119286138 | 1.75890178737673 | BMP4//AQP1 |
| GO:0002695 | negative regulation of leukocyte activation | Biological process | 3 | 100 | 75 | 13692 | 5.4768 | 0.0174677227467139 | 0.196355119286138 | 1.75776370995701 | BMP4//FOXJ1//XCL1 |
| GO:0070665 | positive regulation of leukocyte proliferation | Biological process | 3 | 100 | 75 | 13692 | 5.4768 | 0.0174677227467139 | 0.196355119286138 | 1.75776370995701 | VAV3//CLCF1//XCL1 |
| GO:0042060 | wound healing | Biological process | 5 | 276 | 75 | 13692 | 3.30724637681159 | 0.0176202241100663 | 0.19702793739558 | 1.75398857213588 | C3//F5//F12//AQP1//CDH3 |
| GO:1901068 | guanosine-containing compound metabolic process | Biological process | 5 | 276 | 75 | 13692 | 3.30724637681159 | 0.0176202241100663 | 0.19702793739558 | 1.75398857213588 | RERG//EPHA5//NTRK1//FOXJ1//VAV3 |
| GO:0042063 | gliogenesis | Biological process | 4 | 182 | 75 | 13692 | 4.01230769230769 | 0.0176624040594122 | 0.19702793739558 | 1.75295018418548 | LAMA2//MMP14//CLCF1//BMP4 |
| GO:1901292 | nucleoside phosphate catabolic process | Biological process | 6 | 380 | 75 | 13692 | 2.88252631578947 | 0.0177380256282476 | 0.197327554726476 | 1.75109472196068 | RERG//EPHA5//NTRK1//RGN//FOXJ1//VAV3 |
| GO:0048608 | reproductive structure development | Biological process | 5 | 277 | 75 | 13692 | 3.29530685920578 | 0.0178693063928857 | 0.197327554726476 | 1.74789230455023 | BMP4//MMP14//WNT10A//MGST1//NTRK1 |
| GO:0051090 | regulation of sequence-specific DNA binding transcription factor activity | Biological process | 5 | 277 | 75 | 13692 | 3.29530685920578 | 0.0178693063928857 | 0.197327554726476 | 1.74789230455023 | FOXJ1//EPHA5//WFS1//XCL1//NTRK1 |
| GO:0061458 | reproductive system development | Biological process | 5 | 277 | 75 | 13692 | 3.29530685920578 | 0.0178693063928857 | 0.197327554726476 | 1.74789230455023 | BMP4//MMP14//WNT10A//MGST1//NTRK1 |
| GO:0006865 | amino acid transport | Biological process | 3 | 101 | 75 | 13692 | 5.42257425742574 | 0.0179329513068708 | 0.19753281037518 | 1.74634823074947 | SLC6A6//FOLR1//SLCO1A5 |
| GO:0032990 | cell part morphogenesis | Biological process | 7 | 493 | 75 | 13692 | 2.59212981744422 | 0.01813812078827 | 0.198250124567655 | 1.74140771030575 | NTRK1//EPHA5//LMX1A//LAMA2//ROBO3//FOXJ1//SEMA7A |
| GO:0033124 | regulation of GTP catabolic process | Biological process | 4 | 184 | 75 | 13692 | 3.96869565217391 | 0.0183090751283273 | 0.198250124567655 | 1.73733359326295 | EPHA5//NTRK1//FOXJ1//VAV3 |
| GO:0006805 | xenobiotic metabolic process | Biological process | 2 | 38 | 75 | 13692 | 9.60842105263158 | 0.0183254348764971 | 0.198250124567655 | 1.73694571042642 | PON3//UGT1A6 |
| GO:0009309 | amine biosynthetic process | Biological process | 2 | 38 | 75 | 13692 | 9.60842105263158 | 0.0183254348764971 | 0.198250124567655 | 1.73694571042642 | HDC//SNCA |
| GO:0014910 | regulation of smooth muscle cell migration | Biological process | 2 | 38 | 75 | 13692 | 9.60842105263158 | 0.0183254348764971 | 0.198250124567655 | 1.73694571042642 | LPAR1//ACE |
| GO:0071466 | cellular response to xenobiotic stimulus | Biological process | 2 | 38 | 75 | 13692 | 9.60842105263158 | 0.0183254348764971 | 0.198250124567655 | 1.73694571042642 | UGT1A6//PON3 |
| GO:0048514 | blood vessel morphogenesis | Biological process | 6 | 383 | 75 | 13692 | 2.85994778067885 | 0.0183649267175627 | 0.198250124567655 | 1.73601080030108 | BMP4//MMP14//MED1//C3//AQP1//NTRK1 |
| GO:0016485 | protein processing | Biological process | 3 | 102 | 75 | 13692 | 5.36941176470588 | 0.0184050640280647 | 0.198250124567655 | 1.73506266737858 | C3//F12//MMP14 |
| GO:0043433 | negative regulation of sequence-specific DNA binding transcription factor activity | Biological process | 3 | 102 | 75 | 13692 | 5.36941176470588 | 0.0184050640280647 | 0.198250124567655 | 1.73506266737858 | FOXJ1//WFS1//XCL1 |
| GO:0040007 | growth | Biological process | 9 | 734 | 75 | 13692 | 2.23847411444142 | 0.0184601319696516 | 0.198355927830766 | 1.73376519857085 | LMX1A//MSX1//SERTAD2//RERG//SEMA7A//WFS1//C3//BMP4//MED1 |
| GO:0010941 | regulation of cell death | Biological process | 12 | 1124 | 75 | 13692 | 1.94903914590747 | 0.0185360495688819 | 0.198684697579409 | 1.73198281783142 | XCL1//SNCA//BMP4//LPAR1//MSX1//WFS1//ACE//AQP1//KRT18//MED1//NTRK1//CLCF1 |
| GO:0042129 | regulation of T cell proliferation | Biological process | 3 | 103 | 75 | 13692 | 5.31728155339806 | 0.0188840677190434 | 0.201482948780741 | 1.72390445084012 | BMP4//XCL1//FOXJ1 |
| GO:0097285 | cell-type specific apoptotic process | Biological process | 5 | 281 | 75 | 13692 | 3.24839857651246 | 0.0188890264481945 | 0.201482948780741 | 1.72379042530065 | SNCA//NTRK1//WFS1//CLCF1//BMP4 |
| GO:0007389 | pattern specification process | Biological process | 6 | 387 | 75 | 13692 | 2.83038759689922 | 0.0192243384536111 | 0.204066585425257 | 1.71614859617618 | BMP4//FOXJ1//WNT10A//MSX1//CCDC40//SOSTDC1 |
| GO:0034655 | nucleobase-containing compound catabolic process | Biological process | 6 | 387 | 75 | 13692 | 2.83038759689922 | 0.0192243384536111 | 0.204066585425257 | 1.71614859617618 | RERG//EPHA5//NTRK1//RGN//FOXJ1//VAV3 |
| GO:0019233 | sensory perception of pain | Biological process | 3 | 104 | 75 | 13692 | 5.26615384615385 | 0.019369968420447 | 0.204129667200095 | 1.71287108732615 | NTRK1//ACE//AQP1 |
| GO:0030326 | embryonic limb morphogenesis | Biological process | 3 | 104 | 75 | 13692 | 5.26615384615385 | 0.019369968420447 | 0.204129667200095 | 1.71287108732615 | MSX1//BMP4//MED1 |
| GO:0035113 | embryonic appendage morphogenesis | Biological process | 3 | 104 | 75 | 13692 | 5.26615384615385 | 0.019369968420447 | 0.204129667200095 | 1.71287108732615 | BMP4//MSX1//MED1 |
| GO:0030154 | cell differentiation | Biological process | 20 | 2291 | 75 | 13692 | 1.59371453513749 | 0.019736177535471 | 0.207490173418477 | 1.70473695683365 | BMP4//ROBO3//MSX1//NTRK1//EPHA5//LMX1A//LAMA2//TRPV4//LPAR1//WNT10A//CLCF1//MED1//FOXJ1//MGST1//FSCN2//MMP14//SEMA7A//ACE//MDK//WIPF3 |
| GO:0002526 | acute inflammatory response | Biological process | 3 | 105 | 75 | 13692 | 5.216 | 0.0198627714152593 | 0.207579906945298 | 1.7019601553179 | C3//F12//APOA2 |
| GO:0048638 | regulation of developmental growth | Biological process | 3 | 105 | 75 | 13692 | 5.216 | 0.0198627714152593 | 0.207579906945298 | 1.7019601553179 | SEMA7A//C3//BMP4 |
| GO:0048812 | neuron projection morphogenesis | Biological process | 6 | 390 | 75 | 13692 | 2.80861538461538 | 0.0198867611580806 | 0.207579906945298 | 1.7014359421501 | NTRK1//EPHA5//LMX1A//LAMA2//ROBO3//SEMA7A |
| GO:0009755 | hormone-mediated signaling pathway | Biological process | 2 | 40 | 75 | 13692 | 9.128 | 0.020190422980777 | 0.209254880254672 | 1.69485458267631 | MED1//BMP4 |
| GO:0048639 | positive regulation of developmental growth | Biological process | 2 | 40 | 75 | 13692 | 9.128 | 0.020190422980777 | 0.209254880254672 | 1.69485458267631 | SEMA7A//C3 |
| GO:0071559 | response to transforming growth factor beta stimulus | Biological process | 2 | 40 | 75 | 13692 | 9.128 | 0.020190422980777 | 0.209254880254672 | 1.69485458267631 | XCL1//WNT10A |
| GO:0050866 | negative regulation of cell activation | Biological process | 3 | 106 | 75 | 13692 | 5.16679245283019 | 0.020362481239158 | 0.210540372057709 | 1.6911693028177 | BMP4//FOXJ1//XCL1 |
| GO:0045860 | positive regulation of protein kinase activity | Biological process | 5 | 287 | 75 | 13692 | 3.18048780487805 | 0.0204896736785901 | 0.211357010369268 | 1.68846495817841 | BMP4//LPAR1//MDFIC//CAMKK2//SNCA |
| GO:0008283 | cell proliferation | Biological process | 13 | 1280 | 75 | 13692 | 1.854125 | 0.0208350771719409 | 0.214262627195132 | 1.68120488660166 | MMP14//BMP4//CLCF1//NMB//NTRK1//MSX1//RERG//MED1//VAV3//XCL1//FOXJ1//AQP1//COL8A1 |
| GO:0034599 | cellular response to oxidative stress | Biological process | 3 | 107 | 75 | 13692 | 5.1185046728972 | 0.0208691016907667 | 0.214262627195132 | 1.68049624471219 | AQP1//MGST1//SNCA |
| GO:0006584 | catecholamine metabolic process | Biological process | 2 | 41 | 75 | 13692 | 8.90536585365854 | 0.0211515178076214 | 0.214648736269936 | 1.6746584627181 | SNCA//HDC |
| GO:0009712 | catechol-containing compound metabolic process | Biological process | 2 | 41 | 75 | 13692 | 8.90536585365854 | 0.0211515178076214 | 0.214648736269936 | 1.6746584627181 | SNCA//HDC |
| GO:0014909 | smooth muscle cell migration | Biological process | 2 | 41 | 75 | 13692 | 8.90536585365854 | 0.0211515178076214 | 0.214648736269936 | 1.6746584627181 | ACE//LPAR1 |
| GO:0015909 | long-chain fatty acid transport | Biological process | 2 | 41 | 75 | 13692 | 8.90536585365854 | 0.0211515178076214 | 0.214648736269936 | 1.6746584627181 | ACE//NMB |
| GO:0034311 | diol metabolic process | Biological process | 2 | 41 | 75 | 13692 | 8.90536585365854 | 0.0211515178076214 | 0.214648736269936 | 1.6746584627181 | SNCA//HDC |
| GO:0008585 | female gonad development | Biological process | 3 | 108 | 75 | 13692 | 5.07111111111111 | 0.0213826358418065 | 0.215994183249953 | 1.66993876025946 | BMP4//MMP14//WNT10A |
| GO:0051604 | protein maturation | Biological process | 3 | 108 | 75 | 13692 | 5.07111111111111 | 0.0213826358418065 | 0.215994183249953 | 1.66993876025946 | C3//F12//MMP14 |
| GO:0042306 | regulation of protein import into nucleus | Biological process | 3 | 109 | 75 | 13692 | 5.0245871559633 | 0.0219030860471505 | 0.220236534932816 | 1.65949469069294 | MED1//MDFIC//BMP4 |
| GO:1900180 | regulation of protein localization to nucleus | Biological process | 3 | 109 | 75 | 13692 | 5.0245871559633 | 0.0219030860471505 | 0.220236534932816 | 1.65949469069294 | MED1//MDFIC//BMP4 |
| GO:0072009 | nephron epithelium development | Biological process | 2 | 42 | 75 | 13692 | 8.69333333333333 | 0.0221313719719952 | 0.221771801635872 | 1.6549916623765 | BMP4//AQP1 |
| GO:1900542 | regulation of purine nucleotide metabolic process | Biological process | 5 | 293 | 75 | 13692 | 3.11535836177474 | 0.0221771003567658 | 0.221771801635872 | 1.65409523823099 | EPHA5//NTRK1//RGN//FOXJ1//VAV3 |
| GO:0046649 | lymphocyte activation | Biological process | 6 | 400 | 75 | 13692 | 2.7384 | 0.0222075321437381 | 0.221771801635872 | 1.65349970060984 | NTRK1//CLCF1//VAV3//BMP4//FOXJ1//XCL1 |
| GO:0002684 | positive regulation of immune system process | Biological process | 6 | 403 | 75 | 13692 | 2.71801488833747 | 0.0229381498661187 | 0.227867088810592 | 1.63944161413043 | C3//XCL1//CLCF1//FOXJ1//VAV3//SEMA7A |
| GO:0007265 | Ras protein signal transduction | Biological process | 5 | 296 | 75 | 13692 | 3.08378378378378 | 0.0230538580646311 | 0.227867088810592 | 1.63725638498272 | VAV3//LPAR1//NTRK1 |
| GO:0031110 | regulation of microtubule polymerization or depolymerization | Biological process | 2 | 43 | 75 | 13692 | 8.49116279069768 | 0.0231297569618416 | 0.227867088810592 | 1.63582893059668 | SNCA//TRPV4 |
| GO:0031532 | actin cytoskeleton reorganization | Biological process | 2 | 43 | 75 | 13692 | 8.49116279069768 | 0.0231297569618416 | 0.227867088810592 | 1.63582893059668 | BAIAP2L1//TRPV4 |
| GO:0042733 | embryonic digit morphogenesis | Biological process | 2 | 43 | 75 | 13692 | 8.49116279069768 | 0.0231297569618416 | 0.227867088810592 | 1.63582893059668 | MSX1//BMP4 |
| GO:0046165 | alcohol biosynthetic process | Biological process | 2 | 43 | 75 | 13692 | 8.49116279069768 | 0.0231297569618416 | 0.227867088810592 | 1.63582893059668 | SNCA//HDC |
| GO:0006950 | response to stress | Biological process | 19 | 2176 | 75 | 13692 | 1.59404411764706 | 0.0232310436283907 | 0.228021645564932 | 1.63393127957254 | MDK//ACE//MMP14//C3//F12//APOA2//F5//TRPV4//AQP1//VAV3//MDFIC//WFS1//SNCA//CDH3//NTRK1//XCL1//SEMA7A//MED1//MGST1 |
| GO:0046879 | hormone secretion | Biological process | 4 | 198 | 75 | 13692 | 3.68808080808081 | 0.0232494697918624 | 0.228021645564932 | 1.63358694682108 | NMB//TRPV4//AQP1//EPHA5 |
| GO:0044085 | cellular component biogenesis | Biological process | 14 | 1442 | 75 | 13692 | 1.77242718446602 | 0.0234949352757678 | 0.229914723770013 | 1.62902574704093 | TRPV4//AQP1//VAV3//BAIAP2L1//SNCA//LPAR1//APOA2//FOXJ1//CLDN19//C1QTNF3//MMP14//EPHA5//MGST1//CCDC40 |
| GO:0006140 | regulation of nucleotide metabolic process | Biological process | 5 | 299 | 75 | 13692 | 3.05284280936455 | 0.0239529003440597 | 0.233874198459594 | 1.62064189238896 | EPHA5//NTRK1//RGN//FOXJ1//VAV3 |
| GO:0006814 | sodium ion transport | Biological process | 3 | 113 | 75 | 13692 | 4.84672566371681 | 0.0240540699742514 | 0.23434009503804 | 1.61881142998398 | SCN4B//SLC4A4//SLC5A5 |
| GO:0009410 | response to xenobiotic stimulus | Biological process | 2 | 44 | 75 | 13692 | 8.29818181818182 | 0.0241464461467333 | 0.23471844768798 | 1.6171467792991 | UGT1A6//PON3 |
| GO:0010959 | regulation of metal ion transport | Biological process | 4 | 201 | 75 | 13692 | 3.63303482587065 | 0.0244038771432033 | 0.236599681764236 | 1.61254117004923 | SNCA//XCL1//WFS1//SCN4B |
| GO:0009150 | purine ribonucleotide metabolic process | Biological process | 6 | 409 | 75 | 13692 | 2.67814180929095 | 0.0244479141968976 | 0.236599681764236 | 1.61175818731413 | PRPS2//RERG//EPHA5//NTRK1//FOXJ1//VAV3 |
| GO:0034330 | cell junction organization | Biological process | 3 | 114 | 75 | 13692 | 4.80421052631579 | 0.0246091113761458 | 0.237635119544104 | 1.6089040681909 | TRPV4//CLDN19//MMP14 |
| GO:0010522 | regulation of calcium ion transport into cytosol | Biological process | 2 | 45 | 75 | 13692 | 8.11377777777778 | 0.0251812147645661 | 0.240618828771707 | 1.59892332296435 | SNCA//XCL1 |
| GO:0014812 | muscle cell migration | Biological process | 2 | 45 | 75 | 13692 | 8.11377777777778 | 0.0251812147645661 | 0.240618828771707 | 1.59892332296435 | ACE//LPAR1 |
| GO:0032835 | glomerulus development | Biological process | 2 | 45 | 75 | 13692 | 8.11377777777778 | 0.0251812147645661 | 0.240618828771707 | 1.59892332296435 | BMP4//AQP1 |
| GO:0048732 | gland development | Biological process | 5 | 303 | 75 | 13692 | 3.01254125412541 | 0.0251866038683937 | 0.240618828771707 | 1.5988303883038 | MED1//BMP4//MSX1//MDK//WNT10A |
| GO:0001822 | kidney development | Biological process | 4 | 203 | 75 | 13692 | 3.59724137931035 | 0.0251925279211253 | 0.240618828771707 | 1.59872825143354 | BMP4//AQP1//ACE//WFS1 |
| GO:0007596 | blood coagulation | Biological process | 3 | 116 | 75 | 13692 | 4.72137931034483 | 0.0257399388321807 | 0.245312808348435 | 1.58939248947817 | F5//F12//C3 |
| GO:0042127 | regulation of cell proliferation | Biological process | 11 | 1042 | 75 | 13692 | 1.92721689059501 | 0.0258387640240481 | 0.245720480436935 | 1.58772826429822 | BMP4//CLCF1//NMB//NTRK1//MSX1//RERG//MED1//VAV3//XCL1//FOXJ1//AQP1 |
| GO:0034763 | negative regulation of transmembrane transport | Biological process | 2 | 46 | 75 | 13692 | 7.93739130434783 | 0.0262338399083386 | 0.248638177035352 | 1.58113813606052 | MDFIC//APOA2 |
| GO:0007599 | hemostasis | Biological process | 3 | 117 | 75 | 13692 | 4.68102564102564 | 0.0263157194672453 | 0.248638177035352 | 1.57978475194793 | C3//F5//F12 |
| GO:0021543 | pallium development | Biological process | 3 | 117 | 75 | 13692 | 4.68102564102564 | 0.0263157194672453 | 0.248638177035352 | 1.57978475194793 | MDK//LMX1A//EPHA5 |
| GO:0051345 | positive regulation of hydrolase activity | Biological process | 5 | 307 | 75 | 13692 | 2.97328990228013 | 0.0264606651976073 | 0.249470013389915 | 1.57739924223397 | SNCA//MMP14//NTRK1//RGN//FOXJ1 |
| GO:0044270 | cellular nitrogen compound catabolic process | Biological process | 6 | 418 | 75 | 13692 | 2.62047846889952 | 0.0268359506501896 | 0.252465252468737 | 1.57128301543867 | RERG//EPHA5//NTRK1//RGN//FOXJ1//VAV3 |
| GO:0009259 | ribonucleotide metabolic process | Biological process | 6 | 419 | 75 | 13692 | 2.61422434367542 | 0.0271105593850114 | 0.254502553198908 | 1.56686152132334 | PRPS2//RERG//EPHA5//NTRK1//FOXJ1//VAV3 |
| GO:0002673 | regulation of acute inflammatory response | Biological process | 2 | 47 | 75 | 13692 | 7.76851063829787 | 0.027304100513018 | 0.255226389443648 | 1.56377212598313 | C3//F12 |
| GO:0021983 | pituitary gland development | Biological process | 2 | 47 | 75 | 13692 | 7.76851063829787 | 0.027304100513018 | 0.255226389443648 | 1.56377212598313 | BMP4//MSX1 |
| GO:0031401 | positive regulation of protein modification process | Biological process | 7 | 537 | 75 | 13692 | 2.37973929236499 | 0.0274047020763255 | 0.255621731707683 | 1.56217491489487 | BMP4//LPAR1//MDFIC//WFS1//CAMKK2//SNCA//CLCF1 |
| GO:0050817 | coagulation | Biological process | 3 | 119 | 75 | 13692 | 4.60235294117647 | 0.0274879962600633 | 0.255854300645685 | 1.56085691693769 | C3//F5//F12 |
| GO:0009117 | nucleotide metabolic process | Biological process | 8 | 664 | 75 | 13692 | 2.19951807228916 | 0.028407430249895 | 0.263852064015974 | 1.54656805099256 | PRPS2//AQP1//RERG//EPHA5//NTRK1//RGN//FOXJ1//VAV3 |
| GO:0046128 | purine ribonucleoside metabolic process | Biological process | 5 | 314 | 75 | 13692 | 2.90700636942675 | 0.0287885597532987 | 0.266826735641568 | 1.54078006165338 | RERG//EPHA5//NTRK1//FOXJ1//VAV3 |
| GO:0030098 | lymphocyte differentiation | Biological process | 4 | 212 | 75 | 13692 | 3.44452830188679 | 0.0289316507931228 | 0.267023909635896 | 1.53862678550549 | NTRK1//CLCF1//BMP4//FOXJ1 |
| GO:0072001 | renal system development | Biological process | 4 | 212 | 75 | 13692 | 3.44452830188679 | 0.0289316507931228 | 0.267023909635896 | 1.53862678550549 | BMP4//ACE//WFS1//AQP1 |
| GO:0044087 | regulation of cellular component biogenesis | Biological process | 5 | 315 | 75 | 13692 | 2.89777777777778 | 0.0291314202579084 | 0.268302828593845 | 1.53563834149192 | AQP1//BAIAP2L1//SNCA//MMP14//EPHA5 |
| GO:0048762 | mesenchymal cell differentiation | Biological process | 3 | 122 | 75 | 13692 | 4.48918032786885 | 0.0292981268976353 | 0.268687176963197 | 1.53316014428836 | MSX1//WNT10A//BMP4 |
| GO:0034504 | protein localization to nucleus | Biological process | 4 | 213 | 75 | 13692 | 3.42835680751174 | 0.0293664469961054 | 0.268687176963197 | 1.53214859495924 | MED1//MDFIC//BMP4//MSX1 |
| GO:0002444 | myeloid leukocyte mediated immunity | Biological process | 2 | 49 | 75 | 13692 | 7.45142857142857 | 0.0294966529765994 | 0.268687176963197 | 1.53022726118323 | C3//ACE |
| GO:0010463 | mesenchymal cell proliferation | Biological process | 2 | 49 | 75 | 13692 | 7.45142857142857 | 0.0294966529765994 | 0.268687176963197 | 1.53022726118323 | BMP4//MSX1 |
| GO:0045685 | regulation of glial cell differentiation | Biological process | 2 | 49 | 75 | 13692 | 7.45142857142857 | 0.0294966529765994 | 0.268687176963197 | 1.53022726118323 | CLCF1//BMP4 |
| GO:0051704 | multi-organism process | Biological process | 9 | 797 | 75 | 13692 | 2.06153074027604 | 0.0295408803139281 | 0.268687176963197 | 1.52957656691896 | XCL1//SOSTDC1//BAIAP2L1//ACE//SNCA//UGT1A6//MGST1//MED1//AQP1 |
| GO:0055085 | transmembrane transport | Biological process | 7 | 546 | 75 | 13692 | 2.34051282051282 | 0.0296379523187314 | 0.269011973013082 | 1.52815180499081 | MED1//SCN4B//AQP1//MDFIC//BMP4//APOA2//TRPV4 |
| GO:0002521 | leukocyte differentiation | Biological process | 5 | 317 | 75 | 13692 | 2.8794952681388 | 0.0298249242592212 | 0.269592717427682 | 1.52542065055264 | NTRK1//CLCF1//BMP4//MED1//FOXJ1 |
| GO:0042278 | purine nucleoside metabolic process | Biological process | 5 | 317 | 75 | 13692 | 2.8794952681388 | 0.0298249242592212 | 0.269592717427682 | 1.52542065055264 | RERG//EPHA5//NTRK1//FOXJ1//VAV3 |
| GO:0007420 | brain development | Biological process | 7 | 549 | 75 | 13692 | 2.32772313296903 | 0.0304091368980245 | 0.27430793448753 | 1.51699590625326 | BMP4//MDK//LMX1A//AQP1//EPHA5//MSX1//MED1 |
| GO:0035270 | endocrine system development | Biological process | 3 | 124 | 75 | 13692 | 4.41677419354839 | 0.0305392866049508 | 0.274502158943906 | 1.51514111224201 | BMP4//MSX1//MDK |
| GO:0031109 | microtubule polymerization or depolymerization | Biological process | 2 | 50 | 75 | 13692 | 7.3024 | 0.0306185117982596 | 0.274502158943906 | 1.51401592185169 | SNCA//TRPV4 |
| GO:0035094 | response to nicotine | Biological process | 2 | 50 | 75 | 13692 | 7.3024 | 0.0306185117982596 | 0.274502158943906 | 1.51401592185169 | NTRK1//MSX1 |
| GO:0006644 | phospholipid metabolic process | Biological process | 4 | 216 | 75 | 13692 | 3.38074074074074 | 0.0306941859512175 | 0.274619002469668 | 1.51294388017201 | APOA2//ENPP2//PON1//SNCA |
| GO:0001817 | regulation of cytokine production | Biological process | 5 | 320 | 75 | 13692 | 2.8525 | 0.0308847159935536 | 0.275200396170201 | 1.51025638794777 | XCL1//APOA2//C3//FOXJ1//SEMA7A |
| GO:0016049 | cell growth | Biological process | 5 | 320 | 75 | 13692 | 2.8525 | 0.0308847159935536 | 0.275200396170201 | 1.51025638794777 | LMX1A//MSX1//SERTAD2//RERG//SEMA7A |
| GO:0030308 | negative regulation of cell growth | Biological process | 3 | 125 | 75 | 13692 | 4.38144 | 0.0311701664867694 | 0.276060626016156 | 1.50626087805386 | MSX1//SERTAD2//RERG |
| GO:0044242 | cellular lipid catabolic process | Biological process | 3 | 125 | 75 | 13692 | 4.38144 | 0.0311701664867694 | 0.276060626016156 | 1.50626087805386 | APOA2//ENPP2//CES1D |
| GO:0060560 | developmental growth involved in morphogenesis | Biological process | 3 | 125 | 75 | 13692 | 4.38144 | 0.0311701664867694 | 0.276060626016156 | 1.50626087805386 | SEMA7A//BMP4//MED1 |
| GO:0046486 | glycerolipid metabolic process | Biological process | 4 | 218 | 75 | 13692 | 3.3497247706422 | 0.0315988519204656 | 0.27929307826476 | 1.50032869629503 | APOA2//ENPP2//CES1D//PON1 |
| GO:0030888 | regulation of B cell proliferation | Biological process | 2 | 51 | 75 | 13692 | 7.15921568627451 | 0.0317571399806664 | 0.280127367555818 | 1.49815861665249 | VAV3//CLCF1 |
| GO:0009119 | ribonucleoside metabolic process | Biological process | 5 | 323 | 75 | 13692 | 2.82600619195046 | 0.0319680712715422 | 0.281069906817741 | 1.49528356521077 | RERG//EPHA5//NTRK1//FOXJ1//VAV3 |
| GO:0001775 | cell activation | Biological process | 7 | 555 | 75 | 13692 | 2.30255855855856 | 0.0319922179521106 | 0.281069906817741 | 1.49495565016372 | SNCA//NTRK1//CLCF1//VAV3//BMP4//FOXJ1//XCL1 |
| GO:0009203 | ribonucleoside triphosphate catabolic process | Biological process | 5 | 324 | 75 | 13692 | 2.81728395061728 | 0.0323344486185835 | 0.281169350944668 | 1.49033454043662 | RERG//EPHA5//NTRK1//FOXJ1//VAV3 |
| GO:0009207 | purine ribonucleoside triphosphate catabolic process | Biological process | 5 | 324 | 75 | 13692 | 2.81728395061728 | 0.0323344486185835 | 0.281169350944668 | 1.49033454043662 | RERG//EPHA5//NTRK1//FOXJ1//VAV3 |
| GO:0003006 | developmental process involved in reproduction | Biological process | 6 | 437 | 75 | 13692 | 2.50654462242563 | 0.0323779262364976 | 0.281169350944668 | 1.48975097068107 | BMP4//MMP14//WNT10A//MGST1//NTRK1//MED1 |
| GO:0032318 | regulation of Ras GTPase activity | Biological process | 3 | 127 | 75 | 13692 | 4.31244094488189 | 0.0324524843927924 | 0.281169350944668 | 1.48875205027255 | EPHA5//NTRK1//FOXJ1 |
| GO:0035107 | appendage morphogenesis | Biological process | 3 | 127 | 75 | 13692 | 4.31244094488189 | 0.0324524843927924 | 0.281169350944668 | 1.48875205027255 | BMP4//MSX1//MED1 |
| GO:0035108 | limb morphogenesis | Biological process | 3 | 127 | 75 | 13692 | 4.31244094488189 | 0.0324524843927924 | 0.281169350944668 | 1.48875205027255 | BMP4//MSX1//MED1 |
| GO:0046545 | development of primary female sexual characteristics | Biological process | 3 | 127 | 75 | 13692 | 4.31244094488189 | 0.0324524843927924 | 0.281169350944668 | 1.48875205027255 | BMP4//MMP14//WNT10A |
| GO:0003001 | generation of a signal involved in cell-cell signaling | Biological process | 5 | 325 | 75 | 13692 | 2.80861538461538 | 0.0327034632083103 | 0.281673836356056 | 1.48540625429328 | SNCA//NMB//TRPV4//AQP1//EPHA5 |
| GO:0006520 | cellular amino acid metabolic process | Biological process | 5 | 325 | 75 | 13692 | 2.80861538461538 | 0.0327034632083103 | 0.281673836356056 | 1.48540625429328 | MGST1//HDC//SLC5A5//MED1//FOLR1 |
| GO:0023061 | signal release | Biological process | 5 | 325 | 75 | 13692 | 2.80861538461538 | 0.0327034632083103 | 0.281673836356056 | 1.48540625429328 | SNCA//NMB//TRPV4//AQP1//EPHA5 |
| GO:0035637 | multicellular organismal signaling | Biological process | 7 | 558 | 75 | 13692 | 2.29017921146953 | 0.0328042946568858 | 0.28181178687609 | 1.48406929580992 | SNCA//CLDN19//LAMA2//LPAR1//EPHA5//NTRK1//SCN4B |
| GO:0002705 | positive regulation of leukocyte mediated immunity | Biological process | 2 | 52 | 75 | 13692 | 7.02153846153846 | 0.0329123254745799 | 0.28181178687609 | 1.48264143082937 | C3//XCL1 |
| GO:0002708 | positive regulation of lymphocyte mediated immunity | Biological process | 2 | 52 | 75 | 13692 | 7.02153846153846 | 0.0329123254745799 | 0.28181178687609 | 1.48264143082937 | C3//XCL1 |
| GO:0032870 | cellular response to hormone stimulus | Biological process | 5 | 326 | 75 | 13692 | 2.8 | 0.0330751195831761 | 0.282653653513926 | 1.48049857694486 | MED1//BMP4//SLC5A5//UGT1A6//AQP1 |
| GO:0030335 | positive regulation of cell migration | Biological process | 4 | 222 | 75 | 13692 | 3.28936936936937 | 0.0334551548464535 | 0.285159605826573 | 1.47553695565737 | XCL1//BMP4//AQP1//LPAR1 |
| GO:0006753 | nucleoside phosphate metabolic process | Biological process | 8 | 686 | 75 | 13692 | 2.12897959183673 | 0.0335650194823711 | 0.285159605826573 | 1.47411309639851 | PRPS2//AQP1//RERG//EPHA5//NTRK1//RGN//FOXJ1//VAV3 |
| GO:0042098 | T cell proliferation | Biological process | 3 | 129 | 75 | 13692 | 4.24558139534884 | 0.0337621508716458 | 0.285159605826573 | 1.47156989374271 | BMP4//XCL1//FOXJ1 |
| GO:0009146 | purine nucleoside triphosphate catabolic process | Biological process | 5 | 328 | 75 | 13692 | 2.78292682926829 | 0.0338263754347952 | 0.285159605826573 | 1.47074453525962 | RERG//EPHA5//NTRK1//FOXJ1//VAV3 |
| GO:0009154 | purine ribonucleotide catabolic process | Biological process | 5 | 328 | 75 | 13692 | 2.78292682926829 | 0.0338263754347952 | 0.285159605826573 | 1.47074453525962 | RERG//EPHA5//NTRK1//FOXJ1//VAV3 |
| GO:0009894 | regulation of catabolic process | Biological process | 6 | 442 | 75 | 13692 | 2.47819004524887 | 0.0339521274067318 | 0.285159605826573 | 1.46913300806808 | EPHA5//NTRK1//RGN//FOXJ1//VAV3//APOA2 |
| GO:0046903 | secretion | Biological process | 8 | 688 | 75 | 13692 | 2.12279069767442 | 0.0340637816832526 | 0.285159605826573 | 1.46770713931529 | SNCA//APOA2//MED1//AQP1//NMB//TRPV4//ACE//EPHA5 |
| GO:0002821 | positive regulation of adaptive immune response | Biological process | 2 | 53 | 75 | 13692 | 6.88905660377358 | 0.0340838579956944 | 0.285159605826573 | 1.46745125277238 | C3//XCL1 |
| GO:0002824 | positive regulation of adaptive immune response based on somatic recombination of immune receptors built from immunoglobulin superfamily domains | Biological process | 2 | 53 | 75 | 13692 | 6.88905660377358 | 0.0340838579956944 | 0.285159605826573 | 1.46745125277238 | C3//XCL1 |
| GO:0007229 | integrin-mediated signaling pathway | Biological process | 2 | 53 | 75 | 13692 | 6.88905660377358 | 0.0340838579956944 | 0.285159605826573 | 1.46745125277238 | VAV3//SEMA7A |
| GO:0042490 | mechanoreceptor differentiation | Biological process | 2 | 53 | 75 | 13692 | 6.88905660377358 | 0.0340838579956944 | 0.285159605826573 | 1.46745125277238 | BMP4//NTRK1 |
| GO:0009261 | ribonucleotide catabolic process | Biological process | 5 | 329 | 75 | 13692 | 2.77446808510638 | 0.0342059835729388 | 0.285636251397645 | 1.4658979171775 | RERG//EPHA5//NTRK1//FOXJ1//VAV3 |
| GO:0001568 | blood vessel development | Biological process | 6 | 443 | 75 | 13692 | 2.47259593679458 | 0.0342728571750779 | 0.285650581474414 | 1.46504968886904 | BMP4//MMP14//MED1//C3//AQP1//NTRK1 |
| GO:0051258 | protein polymerization | Biological process | 3 | 130 | 75 | 13692 | 4.21292307692308 | 0.0344272144625496 | 0.286392615187509 | 1.46309811503809 | BAIAP2L1//SNCA//TRPV4 |
| GO:0002088 | lens development in camera-type eye | Biological process | 2 | 54 | 75 | 13692 | 6.76148148148148 | 0.0352715290120901 | 0.291755439979251 | 1.45257571336568 | BMP4//MED1 |
| GO:0015918 | sterol transport | Biological process | 2 | 54 | 75 | 13692 | 6.76148148148148 | 0.0352715290120901 | 0.291755439979251 | 1.45257571336568 | PON1//APOA2 |
| GO:0030301 | cholesterol transport | Biological process | 2 | 54 | 75 | 13692 | 6.76148148148148 | 0.0352715290120901 | 0.291755439979251 | 1.45257571336568 | PON1//APOA2 |
| GO:0009143 | nucleoside triphosphate catabolic process | Biological process | 5 | 333 | 75 | 13692 | 2.74114114114114 | 0.0357510479655152 | 0.294830156155905 | 1.44671122326234 | RERG//EPHA5//NTRK1//FOXJ1//VAV3 |
| GO:0046822 | regulation of nucleocytoplasmic transport | Biological process | 3 | 132 | 75 | 13692 | 4.14909090909091 | 0.0357777470517658 | 0.294830156155905 | 1.44638701071644 | MED1//MDFIC//BMP4 |
| GO:2000147 | positive regulation of cell motility | Biological process | 4 | 227 | 75 | 13692 | 3.21691629955947 | 0.0358639022411413 | 0.294985642448712 | 1.44534245795307 | XCL1//BMP4//AQP1//LPAR1 |
| GO:0010975 | regulation of neuron projection development | Biological process | 4 | 228 | 75 | 13692 | 3.20280701754386 | 0.0363574644944241 | 0.295031323822995 | 1.43940641132675 | NTRK1//TRPV4//LPAR1//SEMA7A |
| GO:0050769 | positive regulation of neurogenesis | Biological process | 3 | 133 | 75 | 13692 | 4.11789473684211 | 0.0364631924038922 | 0.295031323822995 | 1.4381453109224 | SEMA7A//CLCF1//ACE |
| GO:0035050 | embryonic heart tube development | Biological process | 2 | 55 | 75 | 13692 | 6.63854545454546 | 0.0364751317317663 | 0.295031323822995 | 1.43800313095539 | FOXJ1//MED1 |
| GO:0035966 | response to topologically incorrect protein | Biological process | 2 | 55 | 75 | 13692 | 6.63854545454546 | 0.0364751317317663 | 0.295031323822995 | 1.43800313095539 | WFS1//F12 |
| GO:0050871 | positive regulation of B cell activation | Biological process | 2 | 55 | 75 | 13692 | 6.63854545454546 | 0.0364751317317663 | 0.295031323822995 | 1.43800313095539 | VAV3//CLCF1 |
| GO:0051209 | release of sequestered calcium ion into cytosol | Biological process | 2 | 55 | 75 | 13692 | 6.63854545454546 | 0.0364751317317663 | 0.295031323822995 | 1.43800313095539 | SNCA//XCL1 |
| GO:0051282 | regulation of sequestering of calcium ion | Biological process | 2 | 55 | 75 | 13692 | 6.63854545454546 | 0.0364751317317663 | 0.295031323822995 | 1.43800313095539 | XCL1//SNCA |
| GO:0051283 | negative regulation of sequestering of calcium ion | Biological process | 2 | 55 | 75 | 13692 | 6.63854545454546 | 0.0364751317317663 | 0.295031323822995 | 1.43800313095539 | XCL1//SNCA |
| GO:0060443 | mammary gland morphogenesis | Biological process | 2 | 55 | 75 | 13692 | 6.63854545454546 | 0.0364751317317663 | 0.295031323822995 | 1.43800313095539 | BMP4//MED1 |
| GO:0048869 | cellular developmental process | Biological process | 20 | 2442 | 75 | 13692 | 1.4951678951679 | 0.036736310025322 | 0.296596654053428 | 1.43490446850167 | MSX1//MED1//BMP4//ROBO3//NTRK1//EPHA5//LMX1A//LAMA2//TRPV4//LPAR1//WNT10A//MDK//WIPF3//CLCF1//FOXJ1//MGST1//FSCN2//MMP14//SEMA7A//ACE |
| GO:0051493 | regulation of cytoskeleton organization | Biological process | 4 | 229 | 75 | 13692 | 3.18882096069869 | 0.0368549701625013 | 0.296905267992371 | 1.43350393606555 | BAIAP2L1//SNCA//TRPV4//EPHA5 |
| GO:0001934 | positive regulation of protein phosphorylation | Biological process | 6 | 451 | 75 | 13692 | 2.42873614190687 | 0.0369099842736866 | 0.296905267992371 | 1.43285613984505 | BMP4//LPAR1//MDFIC//CAMKK2//SNCA//CLCF1 |
| GO:0043279 | response to alkaloid | Biological process | 3 | 134 | 75 | 13692 | 4.08716417910448 | 0.0371554071998048 | 0.297786663919459 | 1.42997797473907 | SNCA//NTRK1//MSX1 |
| GO:0090092 | regulation of transmembrane receptor protein serine/threonine kinase signaling pathway | Biological process | 3 | 134 | 75 | 13692 | 4.08716417910448 | 0.0371554071998048 | 0.297786663919459 | 1.42997797473907 | BMP4//MSX1//SOSTDC1 |
| GO:0072358 | cardiovascular system development | Biological process | 8 | 701 | 75 | 13692 | 2.08342368045649 | 0.0374303190462196 | 0.29889711966963 | 1.42677647105758 | BMP4//MMP14//MED1//FOXJ1//MSX1//C3//AQP1//NTRK1 |
| GO:0072359 | circulatory system development | Biological process | 8 | 701 | 75 | 13692 | 2.08342368045649 | 0.0374303190462196 | 0.29889711966963 | 1.42677647105758 | BMP4//MMP14//MED1//FOXJ1//MSX1//C3//AQP1//NTRK1 |
| GO:0051208 | sequestering of calcium ion | Biological process | 2 | 56 | 75 | 13692 | 6.52 | 0.0376944610902556 | 0.300459122581237 | 1.42372246132379 | XCL1 |
| GO:0048736 | appendage development | Biological process | 3 | 135 | 75 | 13692 | 4.05688888888889 | 0.0378543783824504 | 0.30064057034178 | 1.4218838810948 | BMP4//MSX1//MED1 |
| GO:0060173 | limb development | Biological process | 3 | 135 | 75 | 13692 | 4.05688888888889 | 0.0378543783824504 | 0.30064057034178 | 1.4218838810948 | BMP4//MSX1//MED1 |
| GO:0030036 | actin cytoskeleton organization | Biological process | 5 | 339 | 75 | 13692 | 2.69262536873156 | 0.0381489674315262 | 0.302432320469821 | 1.41851721248916 | TRPV4//BAIAP2L1//AQP1//EPHA5//FOXJ1 |
| GO:0034341 | response to interferon-gamma | Biological process | 2 | 57 | 75 | 13692 | 6.40561403508772 | 0.0389293137383191 | 0.306402354450253 | 1.40972325213306 | MED1//SNCA |
| GO:0043255 | regulation of carbohydrate biosynthetic process | Biological process | 2 | 57 | 75 | 13692 | 6.40561403508772 | 0.0389293137383191 | 0.306402354450253 | 1.40972325213306 | C1QTNF3//SNCA |
| GO:0071295 | cellular response to vitamin | Biological process | 2 | 57 | 75 | 13692 | 6.40561403508772 | 0.0389293137383191 | 0.306402354450253 | 1.40972325213306 | MED1//AQP1 |
| GO:0072073 | kidney epithelium development | Biological process | 2 | 57 | 75 | 13692 | 6.40561403508772 | 0.0389293137383191 | 0.306402354450253 | 1.40972325213306 | BMP4//AQP1 |
| GO:0050896 | response to stimulus | Biological process | 44 | 6556 | 75 | 13692 | 1.22523489932886 | 0.039243365343294 | 0.308320633808245 | 1.4062337564564 | LPAR1//MDK//ACE//MMP14//C3//XCL1//MED1//F12//APOA2//FOXJ1//BMP4//F5//SOSTDC1//BAIAP2L1//UGT1A6//ENPP2//TRPV4//AQP1//VAV3//CLCF1//NMB//SEMA7A//MDFIC//RERG//NTRK1//EPHA5//LMX1A//LAMA2//ROBO3//SNCA//COX8B//CDH3//MGST1//PON1//PON3//MSX1//WFS1//KRT18//S100A4//RGN//C1QTNF3//WNT10A//SLC5A5//RPE65 |
| GO:0051272 | positive regulation of cellular component movement | Biological process | 4 | 234 | 75 | 13692 | 3.12068376068376 | 0.0394017170327864 | 0.309010961487899 | 1.40448485224508 | XCL1//BMP4//AQP1//LPAR1 |
| GO:0032368 | regulation of lipid transport | Biological process | 2 | 58 | 75 | 13692 | 6.2951724137931 | 0.0401794880297213 | 0.313987300396253 | 1.39599560136785 | PON1//APOA2 |
| GO:0045665 | negative regulation of neuron differentiation | Biological process | 2 | 58 | 75 | 13692 | 6.2951724137931 | 0.0401794880297213 | 0.313987300396253 | 1.39599560136785 | LMX1A//MED1 |
| GO:0006816 | calcium ion transport | Biological process | 4 | 236 | 75 | 13692 | 3.09423728813559 | 0.0404480790723358 | 0.314422859696106 | 1.39310209872647 | XCL1//SNCA//WFS1//TRPV4 |
| GO:0040017 | positive regulation of locomotion | Biological process | 4 | 236 | 75 | 13692 | 3.09423728813559 | 0.0404480790723358 | 0.314422859696106 | 1.39310209872647 | XCL1//BMP4//AQP1//LPAR1 |
| GO:0007417 | central nervous system development | Biological process | 8 | 712 | 75 | 13692 | 2.05123595505618 | 0.0404503861470355 | 0.314422859696106 | 1.39307732817467 | BMP4//LMX1A//MED1//MDK//AQP1//EPHA5//MSX1//CLCF1 |
| GO:0033157 | regulation of intracellular protein transport | Biological process | 3 | 139 | 75 | 13692 | 3.94014388489209 | 0.0407175492104142 | 0.315939355289302 | 1.39021837007269 | MED1//MDFIC//BMP4 |
| GO:0060341 | regulation of cellular localization | Biological process | 7 | 587 | 75 | 13692 | 2.17703577512777 | 0.0413783781280893 | 0.320447853784533 | 1.38322653599569 | SNCA//APOA2//NMB//MED1//MDFIC//BMP4//EPHA5 |
| GO:0050777 | negative regulation of immune response | Biological process | 2 | 59 | 75 | 13692 | 6.18847457627119 | 0.0414447840090853 | 0.320447853784533 | 1.38253011936251 | FOXJ1//XCL1 |
| GO:0001944 | vasculature development | Biological process | 6 | 465 | 75 | 13692 | 2.35561290322581 | 0.0418338737854473 | 0.322886800484861 | 1.37847191821285 | BMP4//MMP14//MED1//C3//AQP1//NTRK1 |
| GO:0043010 | camera-type eye development | Biological process | 4 | 239 | 75 | 13692 | 3.05539748953975 | 0.0420472860181297 | 0.323395266497334 | 1.37626203090979 | MED1//AQP1//BMP4//COL8A1 |
| GO:0050673 | epithelial cell proliferation | Biological process | 4 | 239 | 75 | 13692 | 3.05539748953975 | 0.0420472860181297 | 0.323395266497334 | 1.37626203090979 | MMP14//BMP4//MED1//COL8A1 |
| GO:0051048 | negative regulation of secretion | Biological process | 3 | 141 | 75 | 13692 | 3.88425531914894 | 0.0421892989199086 | 0.323919240744097 | 1.3747976914407 | APOA2//SNCA//NMB |
| GO:0002698 | negative regulation of immune effector process | Biological process | 2 | 60 | 75 | 13692 | 6.08533333333333 | 0.0427250033998263 | 0.326317796001461 | 1.3693178940484 | XCL1//FOXJ1 |
| GO:0042100 | B cell proliferation | Biological process | 2 | 60 | 75 | 13692 | 6.08533333333333 | 0.0427250033998263 | 0.326317796001461 | 1.3693178940484 | VAV3//CLCF1 |
| GO:0050821 | protein stabilization | Biological process | 2 | 60 | 75 | 13692 | 6.08533333333333 | 0.0427250033998263 | 0.326317796001461 | 1.3693178940484 | MSX1//WFS1 |
| GO:0050795 | regulation of behavior | Biological process | 3 | 142 | 75 | 13692 | 3.8569014084507 | 0.0429351598700066 | 0.326827172051077 | 1.36718691565725 | XCL1//LPAR1//MDK |
| GO:0042327 | positive regulation of phosphorylation | Biological process | 6 | 468 | 75 | 13692 | 2.34051282051282 | 0.0429407963278788 | 0.326827172051077 | 1.36712990592686 | BMP4//LPAR1//MDFIC//CAMKK2//SNCA//CLCF1 |
| GO:0030155 | regulation of cell adhesion | Biological process | 4 | 241 | 75 | 13692 | 3.03004149377593 | 0.0431332049581927 | 0.327722652576632 | 1.3651882709534 | COL8A1//VAV3//MMP14//LAMA2 |
| GO:0060485 | mesenchyme development | Biological process | 3 | 143 | 75 | 13692 | 3.82993006993007 | 0.0436876561123523 | 0.330937452406441 | 1.35964125501068 | MSX1//WNT10A//BMP4 |
| GO:0051246 | regulation of protein metabolic process | Biological process | 11 | 1133 | 75 | 13692 | 1.77242718446602 | 0.0437072958356134 | 0.330937452406441 | 1.35944606241908 | BMP4//LPAR1//SNCA//C3//F12//MDFIC//WFS1//CAMKK2//CLCF1//FOXJ1//APOA2 |
| GO:0015908 | fatty acid transport | Biological process | 2 | 61 | 75 | 13692 | 5.9855737704918 | 0.0440199495921646 | 0.331017939986363 | 1.35635045909883 | ACE//NMB |
| GO:0043154 | negative regulation of cysteine-type endopeptidase activity involved in apoptotic process | Biological process | 2 | 61 | 75 | 13692 | 5.9855737704918 | 0.0440199495921646 | 0.331017939986363 | 1.35635045909883 | AQP1//SNCA |
| GO:0050729 | positive regulation of inflammatory response | Biological process | 2 | 61 | 75 | 13692 | 5.9855737704918 | 0.0440199495921646 | 0.331017939986363 | 1.35635045909883 | C3//ACE |
| GO:0051153 | regulation of striated muscle cell differentiation | Biological process | 2 | 61 | 75 | 13692 | 5.9855737704918 | 0.0440199495921646 | 0.331017939986363 | 1.35635045909883 | BMP4//MSX1 |
| GO:0045927 | positive regulation of growth | Biological process | 3 | 144 | 75 | 13692 | 3.80333333333333 | 0.0444467705405754 | 0.333655208989525 | 1.35215978896508 | SEMA7A//C3//WFS1 |
| GO:0007264 | small GTPase mediated signal transduction | Biological process | 6 | 474 | 75 | 13692 | 2.31088607594937 | 0.0452100549608036 | 0.3376687172425 | 1.34476496500377 | VAV3//LPAR1//NTRK1//RERG |
| GO:0001508 | regulation of action potential | Biological process | 3 | 145 | 75 | 13692 | 3.77710344827586 | 0.0452124856344315 | 0.3376687172425 | 1.34474161622066 | LAMA2//LPAR1//SCN4B |
| GO:0030879 | mammary gland development | Biological process | 3 | 145 | 75 | 13692 | 3.77710344827586 | 0.0452124856344315 | 0.3376687172425 | 1.34474161622066 | MED1//BMP4//MSX1 |
| GO:0001541 | ovarian follicle development | Biological process | 2 | 62 | 75 | 13692 | 5.88903225806452 | 0.0453294276312166 | 0.337966344787846 | 1.34361976468659 | BMP4//MMP14 |
| GO:0010562 | positive regulation of phosphorus metabolic process | Biological process | 6 | 475 | 75 | 13692 | 2.30602105263158 | 0.0455954765371799 | 0.338797574811859 | 1.34107824094054 | BMP4//LPAR1//MDFIC//CAMKK2//SNCA//CLCF1 |
| GO:0045937 | positive regulation of phosphate metabolic process | Biological process | 6 | 475 | 75 | 13692 | 2.30602105263158 | 0.0455954765371799 | 0.338797574811859 | 1.34107824094054 | BMP4//LPAR1//MDFIC//CAMKK2//SNCA//CLCF1 |
| GO:0043549 | regulation of kinase activity | Biological process | 6 | 476 | 75 | 13692 | 2.30117647058823 | 0.0459829648253039 | 0.341098676470613 | 1.33740303033168 | BMP4//LPAR1//MDFIC//CAMKK2//VAV3//SNCA |
| GO:0006970 | response to osmotic stress | Biological process | 2 | 63 | 75 | 13692 | 5.79555555555556 | 0.0466532442051634 | 0.343244818732598 | 1.33111815060105 | TRPV4//AQP1 |
| GO:0032846 | positive regulation of homeostatic process | Biological process | 2 | 63 | 75 | 13692 | 5.79555555555556 | 0.0466532442051634 | 0.343244818732598 | 1.33111815060105 | SNCA//XCL1 |
| GO:0051238 | sequestering of metal ion | Biological process | 2 | 63 | 75 | 13692 | 5.79555555555556 | 0.0466532442051634 | 0.343244818732598 | 1.33111815060105 | XCL1 |
| GO:0060191 | regulation of lipase activity | Biological process | 2 | 63 | 75 | 13692 | 5.79555555555556 | 0.0466532442051634 | 0.343244818732598 | 1.33111815060105 | SNCA//APOA2 |
| GO:0055086 | nucleobase-containing small molecule metabolic process | Biological process | 8 | 733 | 75 | 13692 | 1.9924693042292 | 0.0466637572912018 | 0.343244818732598 | 1.33102029543639 | PRPS2//AQP1//RERG//EPHA5//NTRK1//RGN//FOXJ1//VAV3 |
| GO:0032270 | positive regulation of cellular protein metabolic process | Biological process | 7 | 604 | 75 | 13692 | 2.11576158940397 | 0.0470322112546063 | 0.345375568074027 | 1.32760460209386 | BMP4//LPAR1//MDFIC//WFS1//CAMKK2//SNCA//CLCF1 |
| GO:0010001 | glial cell differentiation | Biological process | 3 | 148 | 75 | 13692 | 3.70054054054054 | 0.0475490536445007 | 0.348587042102828 | 1.32285812228201 | LAMA2//CLCF1//BMP4 |
| GO:0090100 | positive regulation of transmembrane receptor protein serine/threonine kinase signaling pathway | Biological process | 2 | 64 | 75 | 13692 | 5.705 | 0.0479912076334965 | 0.350655757108748 | 1.31883832149986 | BMP4//MSX1 |
| GO:2000117 | negative regulation of cysteine-type endopeptidase activity | Biological process | 2 | 64 | 75 | 13692 | 5.705 | 0.0479912076334965 | 0.350655757108748 | 1.31883832149986 | AQP1//SNCA |
| GO:0001816 | cytokine production | Biological process | 5 | 362 | 75 | 13692 | 2.52154696132597 | 0.0482455192117597 | 0.351454215761111 | 1.31654301541803 | XCL1//APOA2//C3//FOXJ1//SEMA7A |
| GO:0031346 | positive regulation of cell projection organization | Biological process | 3 | 149 | 75 | 13692 | 3.67570469798658 | 0.0483409881623974 | 0.351454215761111 | 1.31568447620619 | AQP1//NTRK1//SEMA7A |
| GO:0051924 | regulation of calcium ion transport | Biological process | 3 | 149 | 75 | 13692 | 3.67570469798658 | 0.0483409881623974 | 0.351454215761111 | 1.31568447620619 | SNCA//XCL1//WFS1 |
| GO:0016054 | organic acid catabolic process | Biological process | 3 | 150 | 75 | 13692 | 3.6512 | 0.0491394297770365 | 0.356078115938063 | 1.30856988745959 | HDC//PON1//PON3 |
| GO:0046395 | carboxylic acid catabolic process | Biological process | 3 | 150 | 75 | 13692 | 3.6512 | 0.0491394297770365 | 0.356078115938063 | 1.30856988745959 | HDC//PON1//PON3 |
| GO:0071407 | cellular response to organic cyclic compound | Biological process | 2 | 65 | 75 | 13692 | 5.61723076923077 | 0.0493431278553408 | 0.356203191946829 | 1.30677332409519 | NTRK1//MSX1 |
| GO:0043269 | regulation of ion transport | Biological process | 4 | 252 | 75 | 13692 | 2.89777777777778 | 0.0493884831427328 | 0.356203191946829 | 1.30637431202143 | SCN4B//SNCA//XCL1//WFS1 |
| GO:0032355 | response to estradiol stimulus | Biological process | 3 | 151 | 75 | 13692 | 3.62701986754967 | 0.0499443586282074 | 0.356203191946829 | 1.30151355959022 | C3//BMP4//FOXJ1 |
| GO:0045765 | regulation of angiogenesis | Biological process | 3 | 151 | 75 | 13692 | 3.62701986754967 | 0.0499443586282074 | 0.356203191946829 | 1.30151355959022 | C3//AQP1//NTRK1 |
