# Supplementary material for: Gene Expression in the Hippocampus in a Rat Model of Premenstrual Dysphoric Disorder After Treatment With Baixiangdan Capsules
Source: Front Psychol. 2018 Nov 13;9:2065. doi: 10.3389/fpsyg.2018.02065 (PMC6242977; doi:10.3389/fpsyg.2018.02065)
Supplement: Supplementary file 3 [file Data_Sheet_3.ZIP › Data Analysis Folder/GO Analysis Report/fluoxetine vs model (down)/CC_result(Rat).html]

| GO.ID | Term | Ontology | Count | Pop.Hits | List.Total | Pop.Total | Fold.Enrichment | Pvalue | FDR | Enrichment.Score | GENES |
| --- | --- | --- | --- | --- | --- | --- | --- | --- | --- | --- | --- |
| GO:0044421 | extracellular region part | Cellular component | 19 | 887 | 90 | 15288 | 3.63863209319805 | 7.58404080730685e-07 | 0.000352927889095735 | 6.12009933886349 | C1QTNF3//COL8A1//BMP4//WNT10A//LAMA2//C3//ACE//APOA2//GDF15//PON1//ENPP2//XCL1//SOSTDC1//F5//F12//PON3//AQP1//SNCA//CLCF1 |
| GO:0005576 | extracellular region | Cellular component | 24 | 1410 | 90 | 15288 | 2.8913475177305 | 1.31444278992825e-06 | 0.000352927889095735 | 5.88125831146645 | C1QTNF3//COL8A1//BMP4//WNT10A//LAMA2//C3//ACE//APOA2//GDF15//PON1//ENPP2//XCL1//SOSTDC1//F5//F12//PON3//AQP1//SNCA//CLCF1//MDK//NTS//SCGN//SCGB1C1//RGD1563000 |
| GO:0005615 | extracellular space | Cellular component | 15 | 707 | 90 | 15288 | 3.6039603960396 | 1.47349839345749e-05 | 0.00263756212428891 | 4.8316503333213 | APOA2//PON1//CLCF1//C3//ACE//BMP4//GDF15//ENPP2//XCL1//SOSTDC1//C1QTNF3//F5//F12//PON3//WNT10A |
| GO:0031091 | platelet alpha granule | Cellular component | 2 | 14 | 90 | 15288 | 24.2666666666667 | 0.00297857755673348 | 0.241938603226129 | 2.52599108718076 | SNCA//F5 |
| GO:0043005 | neuron projection | Cellular component | 11 | 742 | 90 | 15288 | 2.51823899371069 | 0.0041277832953091 | 0.241938603226129 | 2.38428311082897 | SNCA//NTRK1//EPHA5//ROBO3//HDC//TRPV4//AQP1//LPAR1//LAMA2//S100A4//KLC3 |
| GO:0005578 | proteinaceous extracellular matrix | Cellular component | 6 | 262 | 90 | 15288 | 3.89007633587786 | 0.00448444355265891 | 0.241938603226129 | 2.34829143823912 | C1QTNF3//COL8A1//LAMA2//SNCA//BMP4//WNT10A |
| GO:0042995 | cell projection | Cellular component | 15 | 1211 | 90 | 15288 | 2.10404624277457 | 0.00452586564485958 | 0.241938603226129 | 2.34429834268402 | AQP1//FOLR1//TRPV4//CCDC40//KLC3//S100A4//SNCA//NTRK1//EPHA5//ROBO3//HDC//SLCO1A5//FSCN2//LPAR1//LAMA2 |
| GO:0031012 | extracellular matrix | Cellular component | 7 | 350 | 90 | 15288 | 3.39733333333333 | 0.00456567092199847 | 0.241938603226129 | 2.34049539418405 | C1QTNF3//COL8A1//BMP4//WNT10A//LAMA2//SNCA//MMP14 |
| GO:0071944 | cell periphery | Cellular component | 29 | 3081 | 90 | 15288 | 1.59887482419128 | 0.00464657143681153 | 0.241938603226129 | 2.33286738157985 | ACE//AQP1//SNCA//NTRK1//TRPV4//EPHA5//RPE65//SLC5A5//LPAR1//CDH3//KCNE2//VAV3//RHBDL2//BAIAP2L1//SCN4B//WNT10A//CDH19//EPS8L2//FOLR1//SLC6A6//SLCO1A5//SLC4A4//CLDN19//EPN3//SEMA7A//SLC16A8//SLC4A2//LAMA2//SSPN |
| GO:0016323 | basolateral plasma membrane | Cellular component | 6 | 273 | 90 | 15288 | 3.73333333333333 | 0.00546599624809844 | 0.241938603226129 | 2.2623306707463 | TRPV4//AQP1//SLC16A8//SLC4A2//SLC4A4//CLDN19 |
| GO:0034364 | high-density lipoprotein particle | Cellular component | 2 | 19 | 90 | 15288 | 17.880701754386 | 0.00549116715925888 | 0.241938603226129 | 2.26033533552232 | APOA2//PON1 |
| GO:0005903 | brush border | Cellular component | 3 | 61 | 90 | 15288 | 8.35409836065574 | 0.0055502060295325 | 0.241938603226129 | 2.25569089510646 | AQP1//SLCO1A5//FOLR1 |
| GO:0005886 | plasma membrane | Cellular component | 28 | 2986 | 90 | 15288 | 1.59285554811342 | 0.00585698667027873 | 0.241938603226129 | 2.23232576436729 | SCN4B//FOLR1//SLC6A6//NTRK1//EPHA5//SLCO1A5//SLC4A4//CLDN19//TRPV4//KCNE2//ACE//SEMA7A//AQP1//SLC16A8//SLC4A2//LAMA2//SSPN//SNCA//RPE65//SLC5A5//LPAR1//CDH3//VAV3//RHBDL2//BAIAP2L1//WNT10A//CDH19//EPS8L2 |
| GO:0031226 | intrinsic to plasma membrane | Cellular component | 8 | 480 | 90 | 15288 | 2.83111111111111 | 0.00728356372847288 | 0.274040498843142 | 2.13765607550633 | SCN4B//FOLR1//SLC6A6//NTRK1//EPHA5//SLCO1A5//SLC4A4//KCNE2 |
| GO:0031982 | vesicle | Cellular component | 11 | 807 | 90 | 15288 | 2.3154068566708 | 0.00765476253751792 | 0.274040498843142 | 2.11606827723812 | NTS//SSPN//SCGN//LPAR1//SNCA//EPN3//F5//NTRK1//BMP4//MMP14//AQP1 |
| GO:0031988 | membrane-bounded vesicle | Cellular component | 10 | 715 | 90 | 15288 | 2.37575757575758 | 0.0092131478760363 | 0.309216275589468 | 2.03559195811859 | NTS//SSPN//SCGN//LPAR1//SNCA//EPN3//F5//MMP14//AQP1//BMP4 |
| GO:0034358 | plasma lipoprotein particle | Cellular component | 2 | 27 | 90 | 15288 | 12.5827160493827 | 0.0109329072930527 | 0.345351248021724 | 1.96126433453372 | APOA2//PON1 |
| GO:0032994 | protein-lipid complex | Cellular component | 2 | 28 | 90 | 15288 | 12.1333333333333 | 0.0117292078581216 | 0.349921367767294 | 1.93073131733574 | APOA2//PON1 |
| GO:0044420 | extracellular matrix part | Cellular component | 4 | 154 | 90 | 15288 | 4.41212121212121 | 0.0129573006751425 | 0.366214234871133 | 1.8874854632796 | C1QTNF3//COL8A1//LAMA2//SNCA |
| GO:0005887 | integral to plasma membrane | Cellular component | 7 | 439 | 90 | 15288 | 2.7085801063022 | 0.0148602705891884 | 0.398998265319709 | 1.82797328247844 | SCN4B//KCNE2//SLC6A6//NTRK1//EPHA5//SLCO1A5//SLC4A4 |
| GO:0009925 | basal plasma membrane | Cellular component | 2 | 35 | 90 | 15288 | 9.70666666666667 | 0.0179784832430976 | 0.459735500073496 | 1.74524695036357 | AQP1//SLC16A8 |
| GO:0044459 | plasma membrane part | Cellular component | 15 | 1426 | 90 | 15288 | 1.78681626928471 | 0.0188935481141918 | 0.461174333514591 | 1.72368647607941 | SCN4B//FOLR1//SLC6A6//NTRK1//EPHA5//SLCO1A5//SLC4A4//CLDN19//TRPV4//KCNE2//ACE//SEMA7A//AQP1//SLC16A8//SLC4A2 |
| GO:0042383 | sarcolemma | Cellular component | 3 | 101 | 90 | 15288 | 5.04554455445545 | 0.0217290786610673 | 0.498835181175169 | 1.66295868789472 | AQP1//LAMA2//SSPN |
| GO:0030133 | transport vesicle | Cellular component | 3 | 102 | 90 | 15288 | 4.99607843137255 | 0.0222943097731919 | 0.498835181175169 | 1.65180596876556 | SCGN//NTS//SSPN |
| GO:0045178 | basal part of cell | Cellular component | 2 | 41 | 90 | 15288 | 8.28617886178862 | 0.0242207186610828 | 0.520261036840059 | 1.61581297490427 | AQP1//SLC16A8 |
| GO:0031526 | brush border membrane | Cellular component | 2 | 42 | 90 | 15288 | 8.08888888888889 | 0.0253358301540577 | 0.523282338181884 | 1.59626486104295 | AQP1//SLCO1A5 |
| GO:0031410 | cytoplasmic vesicle | Cellular component | 9 | 749 | 90 | 15288 | 2.0411214953271 | 0.0319540173383628 | 0.635529900396327 | 1.49547453349294 | NTS//SSPN//SCGN//LPAR1//SNCA//EPN3//F5//MMP14//NTRK1 |
| GO:0005581 | collagen | Cellular component | 2 | 55 | 90 | 15288 | 6.1769696969697 | 0.0416094489993555 | 0.798009789737639 | 1.38080803518335 | C1QTNF3//COL8A1 |
| GO:0016023 | cytoplasmic membrane-bounded vesicle | Cellular component | 8 | 680 | 90 | 15288 | 1.99843137254902 | 0.0467719144024714 | 0.818107720569469 | 1.33001485379442 | NTS//SSPN//SCGN//LPAR1//SNCA//EPN3//F5//MMP14 |
| GO:0030424 | axon | Cellular component | 5 | 334 | 90 | 15288 | 2.54291417165669 | 0.0471860817819452 | 0.818107720569469 | 1.32618608392276 | AQP1//SNCA//NTRK1//EPHA5//ROBO3 |
| GO:0005791 | rough endoplasmic reticulum | Cellular component | 2 | 59 | 90 | 15288 | 5.75819209039548 | 0.0472278199956304 | 0.818107720569469 | 1.32580210070149 | SNCA//EPHA5 |
