# Supplementary material for: Gene Expression in the Hippocampus in a Rat Model of Premenstrual Dysphoric Disorder After Treatment With Baixiangdan Capsules
Source: Front Psychol. 2018 Nov 13;9:2065. doi: 10.3389/fpsyg.2018.02065 (PMC6242977; doi:10.3389/fpsyg.2018.02065)
Supplement: Supplementary file 3 [file Data_Sheet_3.ZIP › Data Analysis Folder/GO Analysis Report/fluoxetine vs model (down)/MF_result(Rat).html]

| GO.ID | Term | Ontology | Count | Pop.Hits | List.Total | Pop.Total | Fold.Enrichment | Pvalue | FDR | Enrichment.Score | GENES |
| --- | --- | --- | --- | --- | --- | --- | --- | --- | --- | --- | --- |
| GO:0005102 | receptor binding | Molecular function | 19 | 1052 | 81 | 14392 | 3.20903159179458 | 4.29438945892972e-06 | 0.00385206734465996 | 5.36709857187131 | WNT10A//AQP1//NTRK1//BMP4//GDF15//CLCF1//VAV3//MMP14//SEMA7A//NTS//XCL1//MDK//MED1//NMB//ACE//APOA2//S100A4//EPHA5//LAMA2 |
| GO:0052689 | carboxylic ester hydrolase activity | Molecular function | 6 | 104 | 81 | 14392 | 10.2507122507122 | 2.5771553753216e-05 | 0.0115585418583174 | 4.58885939727733 | PON1//PON3//CES1D//RGN//ENPP2//RPE65 |
| GO:0005515 | protein binding | Molecular function | 43 | 4586 | 81 | 14392 | 1.66598288941653 | 5.88557298765769e-05 | 0.0141303626060293 | 4.23021125019278 | DNAJB13//WNT10A//LPAR1//MSX1//SNCA//ACE//TRPV4//WIPF3//BAIAP2L1//FSCN2//LAMA2//CLCF1//NTRK1//AQP1//BMP4//GDF15//VAV3//MMP14//SEMA7A//NTS//CAMKK2//XCL1//KLC3//MDK//FOXJ1//MDFIC//MED1//S100A4//MGST1//C1QTNF3//CRB3//UGT1A6//WFS1//NMB//APOA2//HDC//PRPS2//PON1//PON3//ROBO3//SCN4B//EPHA5//C3 |
| GO:0042803 | protein homodimerization activity | Molecular function | 12 | 558 | 81 | 14392 | 3.82105402893933 | 6.3011650417076e-05 | 0.0141303626060293 | 4.20057914508505 | HDC//S100A4//PRPS2//BMP4//APOA2//NTRK1//PON1//UGT1A6//MGST1//XCL1//PON3//ROBO3 |
| GO:0042802 | identical protein binding | Molecular function | 14 | 827 | 81 | 14392 | 3.00786719811307 | 0.000191088282939472 | 0.0342812379593413 | 3.71876594199125 | HDC//S100A4//PRPS2//BMP4//APOA2//NTRK1//PON1//UGT1A6//MGST1//XCL1//PON3//ROBO3//SNCA//C1QTNF3 |
| GO:0015291 | secondary active transmembrane transporter activity | Molecular function | 6 | 158 | 81 | 14392 | 6.74730426629161 | 0.000262072553316327 | 0.0391798467207909 | 3.5815784600432 | SLC6A6//SLC4A2//SLC4A4//SLC5A5//SLC16A8//SLCO1A5 |
| GO:0005509 | calcium ion binding | Molecular function | 10 | 531 | 81 | 14392 | 3.34612075980563 | 0.000766850772886506 | 0.0862413270674826 | 3.11528914037607 | S100A4//RGN//SNCA//MMP14//CAMKK2//PON1//ENPP2//SCGN//CDH19//CALML4 |
| GO:0046983 | protein dimerization activity | Molecular function | 13 | 839 | 81 | 14392 | 2.7530717049986 | 0.000769153418662052 | 0.0862413270674826 | 3.11398702530537 | HDC//S100A4//PRPS2//BMP4//APOA2//NTRK1//PON1//UGT1A6//MGST1//XCL1//PON3//ROBO3//CLCF1 |
| GO:0015077 | monovalent inorganic cation transmembrane transporter activity | Molecular function | 7 | 310 | 81 | 14392 | 4.0121067303863 | 0.00179008148320867 | 0.167722437467063 | 2.74712719980085 | COX8B//SCN4B//KCNE2//AQP1//SLC6A6//SLC5A5//SLC4A4 |
| GO:0005452 | inorganic anion exchanger activity | Molecular function | 2 | 12 | 81 | 14392 | 29.6131687242798 | 0.00199072842052896 | 0.167722437467063 | 2.70098798332291 | SLC4A2//SLC4A4 |
| GO:0005215 | transporter activity | Molecular function | 14 | 1052 | 81 | 14392 | 2.3645495939539 | 0.00205679689201527 | 0.167722437467063 | 2.68680859261842 | SLC6A6//COX8B//AQP1//SCN4B//KCNE2//TRPV4//APOA2//SLC4A2//SLC4A4//SLCO1A5//SLC5A5//FOLR1//SLC16A8//WFS1 |
| GO:0022857 | transmembrane transporter activity | Molecular function | 12 | 839 | 81 | 14392 | 2.54129695846025 | 0.00245056567470784 | 0.172317603209185 | 2.61073365398342 | SLC6A6//COX8B//AQP1//SCN4B//KCNE2//TRPV4//SLC4A2//SLC4A4//SLCO1A5//SLC5A5//FOLR1//SLC16A8 |
| GO:0022890 | inorganic cation transmembrane transporter activity | Molecular function | 8 | 429 | 81 | 14392 | 3.31336153558376 | 0.00280630455461432 | 0.172317603209185 | 2.55186519887749 | COX8B//SCN4B//KCNE2//TRPV4//AQP1//SLC6A6//SLC5A5//SLC4A4 |
| GO:0046873 | metal ion transmembrane transporter activity | Molecular function | 7 | 337 | 81 | 14392 | 3.69066197750669 | 0.00286168807453509 | 0.172317603209185 | 2.54337770631731 | SCN4B//KCNE2//TRPV4//AQP1//SLC6A6//SLC5A5//SLC4A4 |
| GO:0015293 | symporter activity | Molecular function | 4 | 106 | 81 | 14392 | 6.70486839040298 | 0.0029944790000419 | 0.172317603209185 | 2.52367872822943 | SLC6A6//SLC5A5//SLC4A4//SLC16A8 |
| GO:0015301 | anion:anion antiporter activity | Molecular function | 2 | 15 | 81 | 14392 | 23.6905349794239 | 0.00313257658721654 | 0.172317603209185 | 2.50409830229744 | SLC4A2//SLC4A4 |
| GO:0015075 | ion transmembrane transporter activity | Molecular function | 10 | 648 | 81 | 14392 | 2.74196006706295 | 0.00335447902177714 | 0.172317603209185 | 2.47437491976811 | COX8B//AQP1//SCN4B//KCNE2//TRPV4//SLC6A6//SLC4A2//SLC4A4//SLC5A5//SLCO1A5 |
| GO:0022892 | substrate-specific transporter activity | Molecular function | 12 | 880 | 81 | 14392 | 2.42289562289562 | 0.00361898303839856 | 0.172317603209185 | 2.44141345235662 | SLC6A6//COX8B//AQP1//SCN4B//KCNE2//TRPV4//APOA2//SLC4A2//SLC4A4//SLCO1A5//SLC5A5//SLC16A8 |
| GO:0015081 | sodium ion transmembrane transporter activity | Molecular function | 4 | 112 | 81 | 14392 | 6.34567901234568 | 0.00364998267667169 | 0.172317603209185 | 2.43770919676099 | SCN4B//SLC6A6//SLC5A5//SLC4A4 |
| GO:0022891 | substrate-specific transmembrane transporter activity | Molecular function | 11 | 772 | 81 | 14392 | 2.53169577176486 | 0.00385626444327305 | 0.172953460280796 | 2.41383319201081 | SLC6A6//COX8B//AQP1//SCN4B//KCNE2//TRPV4//SLC4A2//SLC4A4//SLCO1A5//SLC5A5//SLC16A8 |
| GO:0019894 | kinesin binding | Molecular function | 2 | 19 | 81 | 14392 | 18.7030539311241 | 0.00502778634796131 | 0.214758302577205 | 2.29862318561474 | SNCA//KLC3 |
| GO:0008509 | anion transmembrane transporter activity | Molecular function | 4 | 127 | 81 | 14392 | 5.59618936521824 | 0.00570055459496219 | 0.232427157803686 | 2.24408289067573 | SLC4A2//SLC4A4//SLC5A5//SLCO1A5 |
| GO:0005125 | cytokine activity | Molecular function | 4 | 130 | 81 | 14392 | 5.4670465337132 | 0.00618673395634727 | 0.24104786174138 | 2.20853855923045 | XCL1//BMP4//GDF15//CLCF1 |
| GO:0022804 | active transmembrane transporter activity | Molecular function | 6 | 297 | 81 | 14392 | 3.5894749968824 | 0.00653146919684111 | 0.24104786174138 | 2.1849891169926 | SLC6A6//SLC4A2//SLC4A4//SLC5A5//SLC16A8//SLCO1A5 |
| GO:0043014 | alpha-tubulin binding | Molecular function | 2 | 22 | 81 | 14392 | 16.1526374859708 | 0.00671816783002732 | 0.24104786174138 | 2.17274915102363 | SNCA//TRPV4 |
| GO:0008028 | monocarboxylic acid transmembrane transporter activity | Molecular function | 2 | 23 | 81 | 14392 | 15.4503488996243 | 0.00733128501180012 | 0.252246141227116 | 2.13481989648818 | SLCO1A5//SLC16A8 |
| GO:0008324 | cation transmembrane transporter activity | Molecular function | 8 | 507 | 81 | 14392 | 2.80361360703241 | 0.00759269321419413 | 0.252246141227116 | 2.11960414760981 | COX8B//AQP1//SCN4B//KCNE2//TRPV4//SLC6A6//SLC5A5//SLC4A4 |
| GO:0015294 | solute:cation symporter activity | Molecular function | 3 | 73 | 81 | 14392 | 7.30187721968544 | 0.00805009676891459 | 0.2578906000613 | 2.09419889901685 | SLC6A6//SLC5A5//SLC4A4 |
| GO:0015296 | anion:cation symporter activity | Molecular function | 2 | 26 | 81 | 14392 | 13.667616334283 | 0.00931556214722896 | 0.278535308202146 | 2.03079093249365 | SLC5A5//SLC4A4 |
| GO:0048487 | beta-tubulin binding | Molecular function | 2 | 26 | 81 | 14392 | 13.667616334283 | 0.00931556214722896 | 0.278535308202146 | 2.03079093249365 | SNCA//TRPV4 |
| GO:0046875 | ephrin receptor binding | Molecular function | 2 | 31 | 81 | 14392 | 11.463162086818 | 0.0130888710654534 | 0.378732817603603 | 1.88309781037725 | AQP1//NTRK1 |
| GO:0019904 | protein domain specific binding | Molecular function | 8 | 564 | 81 | 14392 | 2.52026967866211 | 0.013859554036071 | 0.388500624073615 | 1.85825074395145 | WIPF3//CRB3//BAIAP2L1//LPAR1//TRPV4//MED1//SNCA//FOXJ1 |
| GO:0008017 | microtubule binding | Molecular function | 3 | 94 | 81 | 14392 | 5.67060677698976 | 0.015951969610559 | 0.401094008242998 | 1.79718568638818 | SNCA//TRPV4//KLC3 |
| GO:0015103 | inorganic anion transmembrane transporter activity | Molecular function | 3 | 94 | 81 | 14392 | 5.67060677698976 | 0.015951969610559 | 0.401094008242998 | 1.79718568638818 | SLC4A4//SLC4A2//SLC5A5 |
| GO:0016597 | amino acid binding | Molecular function | 3 | 94 | 81 | 14392 | 5.67060677698976 | 0.015951969610559 | 0.401094008242998 | 1.79718568638818 | FOLR1//MGST1//HDC |
| GO:0017124 | SH3 domain binding | Molecular function | 3 | 95 | 81 | 14392 | 5.61091617933723 | 0.0164065810796988 | 0.401094008242998 | 1.78498191089807 | WIPF3//CRB3//BAIAP2L1 |
| GO:0003779 | actin binding | Molecular function | 5 | 264 | 81 | 14392 | 3.36513280957725 | 0.0165445689018851 | 0.401094008242998 | 1.78134454467044 | TRPV4//FSCN2//ACE//WIPF3//BAIAP2L1 |
| GO:0046943 | carboxylic acid transmembrane transporter activity | Molecular function | 3 | 97 | 81 | 14392 | 5.49522718594883 | 0.0173375432500168 | 0.406386619413795 | 1.76101244253177 | SLC6A6//SLCO1A5//SLC16A8 |
| GO:0031406 | carboxylic acid binding | Molecular function | 4 | 177 | 81 | 14392 | 4.01534491176676 | 0.0176689834527737 | 0.406386619413795 | 1.75278843597997 | FOLR1//HDC//MGST1//SNCA |
| GO:0005342 | organic acid transmembrane transporter activity | Molecular function | 3 | 99 | 81 | 14392 | 5.38421249532361 | 0.0182975541316371 | 0.410322651401962 | 1.73760695934745 | SLC6A6//SLCO1A5//SLC16A8 |
| GO:0043176 | amine binding | Molecular function | 2 | 39 | 81 | 14392 | 9.11174422285533 | 0.0202628218288168 | 0.443311004401187 | 1.69330007430904 | SLC6A6//APOA2 |
| GO:0001664 | G-protein coupled receptor binding | Molecular function | 4 | 195 | 81 | 14392 | 3.64469768914213 | 0.0242189482385152 | 0.517247537379718 | 1.61584472098352 | XCL1//NMB//ACE//WNT10A |
| GO:0015297 | antiporter activity | Molecular function | 2 | 45 | 81 | 14392 | 7.89684499314129 | 0.0264922954961341 | 0.552641606047263 | 1.57688040945994 | SLC4A2//SLC4A4 |
| GO:0008092 | cytoskeletal protein binding | Molecular function | 7 | 529 | 81 | 14392 | 2.35114004994282 | 0.0292229064333365 | 0.595228775614374 | 1.53427659246784 | ACE//TRPV4//WIPF3//BAIAP2L1//FSCN2//SNCA//KLC3 |
| GO:0015370 | solute:sodium symporter activity | Molecular function | 2 | 48 | 81 | 14392 | 7.40329218106996 | 0.0298609753652696 | 0.595228775614374 | 1.52489601078258 | SLC6A6//SLC5A5 |
| GO:0004714 | transmembrane receptor protein tyrosine kinase activity | Molecular function | 2 | 55 | 81 | 14392 | 6.46105499438833 | 0.038335666900802 | 0.745739739549439 | 1.41639697720183 | EPHA5//NTRK1 |
| GO:0008083 | growth factor activity | Molecular function | 3 | 133 | 81 | 14392 | 4.00779727095517 | 0.0390744345137387 | 0.745739739549439 | 1.40810729838465 | BMP4//GDF15//MDK |
| GO:0005178 | integrin binding | Molecular function | 2 | 60 | 81 | 14392 | 5.92263374485597 | 0.0448819367314777 | 0.758840228115256 | 1.34792841080613 | MMP14//SEMA7A |
| GO:0004175 | endopeptidase activity | Molecular function | 5 | 346 | 81 | 14392 | 2.56761578534218 | 0.0454067819046659 | 0.758840228115256 | 1.34287927657449 | RHBDL2//F5//F12//MMP14//ACE |
| GO:0046982 | protein heterodimerization activity | Molecular function | 5 | 347 | 81 | 14392 | 2.56021631622016 | 0.0458743357439749 | 0.758840228115256 | 1.33843021123718 | S100A4//APOA2//UGT1A6//ROBO3//CLCF1 |
