# Supplementary material for: Gene Expression in the Hippocampus in a Rat Model of Premenstrual Dysphoric Disorder After Treatment With Baixiangdan Capsules
Source: Front Psychol. 2018 Nov 13;9:2065. doi: 10.3389/fpsyg.2018.02065 (PMC6242977; doi:10.3389/fpsyg.2018.02065)
Supplement: Supplementary file 3 [file Data_Sheet_3.ZIP › Data Analysis Folder/GO Analysis Report/fluoxetine vs model (up)/BP_result(Rat).html]

| GO.ID | Term | Ontology | Count | Pop.Hits | List.Total | Pop.Total | Fold.Enrichment | Pvalue | FDR | Enrichment.Score | GENES |
| --- | --- | --- | --- | --- | --- | --- | --- | --- | --- | --- | --- |
| GO:0009582 | detection of abiotic stimulus | Biological process | 2 | 69 | 7 | 13692 | 56.695652173913 | 0.000517111013268678 | 1 | 3.28641621266135 | TULP1//CALCA |
| GO:0009581 | detection of external stimulus | Biological process | 2 | 82 | 7 | 13692 | 47.7073170731707 | 0.000729705083535811 | 1 | 3.1368526281973 | TULP1//CALCA |
| GO:0009605 | response to external stimulus | Biological process | 4 | 1128 | 7 | 13692 | 6.93617021276596 | 0.00130932762594054 | 1 | 2.88295166870144 | CALCA//TULP1//RXRG//ABCG2 |
| GO:2000021 | regulation of ion homeostasis | Biological process | 2 | 129 | 7 | 13692 | 30.3255813953488 | 0.00179335052459084 | 1 | 2.74633481583813 | CALCA//RXRG |
| GO:0007565 | female pregnancy | Biological process | 2 | 158 | 7 | 13692 | 24.7594936708861 | 0.0026751296589516 | 1 | 2.5726551636231 | CALCA//ABCG2 |
| GO:0033273 | response to vitamin | Biological process | 2 | 212 | 7 | 13692 | 18.4528301886792 | 0.00476069571585254 | 1 | 2.32232957596075 | RXRG//ABCG2 |
| GO:0016048 | detection of temperature stimulus | Biological process | 1 | 10 | 7 | 13692 | 195.6 | 0.00510240194927647 | 1 | 2.29222533218707 | CALCA |
| GO:0046415 | urate metabolic process | Biological process | 1 | 10 | 7 | 13692 | 195.6 | 0.00510240194927647 | 1 | 2.29222533218707 | ABCG2 |
| GO:0002031 | G-protein coupled receptor internalization | Biological process | 1 | 11 | 7 | 13692 | 177.818181818182 | 0.0056114125607627 | 1 | 2.2509278000408 | CALCA |
| GO:0045986 | negative regulation of smooth muscle contraction | Biological process | 1 | 11 | 7 | 13692 | 177.818181818182 | 0.0056114125607627 | 1 | 2.2509278000408 | CALCA |
| GO:0046851 | negative regulation of bone remodeling | Biological process | 1 | 11 | 7 | 13692 | 177.818181818182 | 0.0056114125607627 | 1 | 2.2509278000408 | CALCA |
| GO:0006855 | drug transmembrane transport | Biological process | 1 | 12 | 7 | 13692 | 163 | 0.00612019993829882 | 1 | 2.21323438983458 | ABCG2 |
| GO:0045649 | regulation of macrophage differentiation | Biological process | 1 | 12 | 7 | 13692 | 163 | 0.00612019993829882 | 1 | 2.21323438983458 | CALCA |
| GO:0050951 | sensory perception of temperature stimulus | Biological process | 1 | 12 | 7 | 13692 | 163 | 0.00612019993829882 | 1 | 2.21323438983458 | CALCA |
| GO:0032844 | regulation of homeostatic process | Biological process | 2 | 247 | 7 | 13692 | 15.8380566801619 | 0.00641154206693006 | 1 | 2.19303750392666 | CALCA//RXRG |
| GO:0003085 | negative regulation of systemic arterial blood pressure | Biological process | 1 | 14 | 7 | 13692 | 139.714285714286 | 0.00713710531786382 | 1 | 2.14647789457982 | CALCA |
| GO:0034104 | negative regulation of tissue remodeling | Biological process | 1 | 14 | 7 | 13692 | 139.714285714286 | 0.00713710531786382 | 1 | 2.14647789457982 | CALCA |
| GO:0051593 | response to folic acid | Biological process | 1 | 14 | 7 | 13692 | 139.714285714286 | 0.00713710531786382 | 1 | 2.14647789457982 | ABCG2 |
| GO:0045671 | negative regulation of osteoclast differentiation | Biological process | 1 | 15 | 7 | 13692 | 130.4 | 0.00764522348300256 | 1 | 2.1166098148931 | CALCA |
| GO:0045762 | positive regulation of adenylate cyclase activity | Biological process | 1 | 15 | 7 | 13692 | 130.4 | 0.00764522348300256 | 1 | 2.1166098148931 | CALCA |
| GO:0031644 | regulation of neurological system process | Biological process | 2 | 279 | 7 | 13692 | 14.0215053763441 | 0.00812033775804157 | 1 | 2.09042590630033 | RXRG//CALCA |
| GO:0045932 | negative regulation of muscle contraction | Biological process | 1 | 17 | 7 | 13692 | 115.058823529412 | 0.00866079117159435 | 1 | 2.06244243295228 | CALCA |
| GO:0051482 | elevation of cytosolic calcium ion concentration involved in phospholipase C-activating G-protein coupled signaling pathway | Biological process | 1 | 17 | 7 | 13692 | 115.058823529412 | 0.00866079117159435 | 1 | 2.06244243295228 | CALCA |
| GO:0015893 | drug transport | Biological process | 1 | 18 | 7 | 13692 | 108.666666666667 | 0.00916824085801515 | 1 | 2.03771398591629 | ABCG2 |
| GO:0032732 | positive regulation of interleukin-1 production | Biological process | 1 | 18 | 7 | 13692 | 108.666666666667 | 0.00916824085801515 | 1 | 2.03771398591629 | CALCA |
| GO:0001976 | neurological system process involved in regulation of systemic arterial blood pressure | Biological process | 1 | 19 | 7 | 13692 | 102.947368421053 | 0.00967546788112383 | 1 | 2.01432802441994 | CALCA |
| GO:0002029 | desensitization of G-protein coupled receptor protein signaling pathway | Biological process | 1 | 19 | 7 | 13692 | 102.947368421053 | 0.00967546788112383 | 1 | 2.01432802441994 | CALCA |
| GO:0022401 | negative adaptation of signaling pathway | Biological process | 1 | 19 | 7 | 13692 | 102.947368421053 | 0.00967546788112383 | 1 | 2.01432802441994 | CALCA |
| GO:0032757 | positive regulation of interleukin-8 production | Biological process | 1 | 19 | 7 | 13692 | 102.947368421053 | 0.00967546788112383 | 1 | 2.01432802441994 | CALCA |
| GO:0006897 | endocytosis | Biological process | 2 | 308 | 7 | 13692 | 12.7012987012987 | 0.00982937499248846 | 1 | 2.00747409620072 | CALCA//TULP1 |
| GO:0030225 | macrophage differentiation | Biological process | 1 | 20 | 7 | 13692 | 97.8 | 0.0101824723223463 | 1 | 1.99214676172501 | CALCA |
| GO:0031281 | positive regulation of cyclase activity | Biological process | 1 | 20 | 7 | 13692 | 97.8 | 0.0101824723223463 | 1 | 1.99214676172501 | CALCA |
| GO:0045494 | photoreceptor cell maintenance | Biological process | 1 | 20 | 7 | 13692 | 97.8 | 0.0101824723223463 | 1 | 1.99214676172501 | TULP1 |
| GO:0002548 | monocyte chemotaxis | Biological process | 1 | 21 | 7 | 13692 | 93.1428571428571 | 0.010689254263082 | 1 | 1.97105259233337 | CALCA |
| GO:0023058 | adaptation of signaling pathway | Biological process | 1 | 21 | 7 | 13692 | 93.1428571428571 | 0.010689254263082 | 1 | 1.97105259233337 | CALCA |
| GO:0031641 | regulation of myelination | Biological process | 1 | 21 | 7 | 13692 | 93.1428571428571 | 0.010689254263082 | 1 | 1.97105259233337 | RXRG |
| GO:0007584 | response to nutrient | Biological process | 2 | 327 | 7 | 13692 | 11.9633027522936 | 0.0110300550830799 | 1 | 1.95742231872772 | RXRG//ABCG2 |
| GO:0051349 | positive regulation of lyase activity | Biological process | 1 | 22 | 7 | 13692 | 88.9090909090909 | 0.0111958137847087 | 1 | 1.9509443335844 | CALCA |
| GO:0007602 | phototransduction | Biological process | 1 | 23 | 7 | 13692 | 85.0434782608696 | 0.011702150968579 | 1 | 1.93173430338836 | TULP1 |
| GO:0032845 | negative regulation of homeostatic process | Biological process | 1 | 23 | 7 | 13692 | 85.0434782608696 | 0.011702150968579 | 1 | 1.93173430338836 | CALCA |
| GO:0045909 | positive regulation of vasodilation | Biological process | 1 | 23 | 7 | 13692 | 85.0434782608696 | 0.011702150968579 | 1 | 1.93173430338836 | CALCA |
| GO:0045761 | regulation of adenylate cyclase activity | Biological process | 1 | 24 | 7 | 13692 | 81.5 | 0.0122082658960221 | 1 | 1.91334602035269 | CALCA |
| GO:0045124 | regulation of bone resorption | Biological process | 1 | 25 | 7 | 13692 | 78.24 | 0.0127141586483436 | 1 | 1.89571237370859 | CALCA |
| GO:0048384 | retinoic acid receptor signaling pathway | Biological process | 1 | 26 | 7 | 13692 | 75.2307692307692 | 0.0132198293068247 | 1 | 1.87877415238311 | RXRG |
| GO:0045935 | positive regulation of nucleobase-containing compound metabolic process | Biological process | 3 | 1077 | 7 | 13692 | 5.44846796657382 | 0.0133512905036701 | 1 | 1.8744767544091 | CALCA//PLAGL1//RXRG |
| GO:0001578 | microtubule bundle formation | Biological process | 1 | 27 | 7 | 13692 | 72.4444444444444 | 0.0137252779527232 | 1 | 1.8624788518242 | KIF20A |
| GO:0051173 | positive regulation of nitrogen compound metabolic process | Biological process | 3 | 1096 | 7 | 13692 | 5.35401459854015 | 0.014009810577595 | 1 | 1.85356773663866 | CALCA//PLAGL1//RXRG |
| GO:0002762 | negative regulation of myeloid leukocyte differentiation | Biological process | 1 | 28 | 7 | 13692 | 69.8571428571428 | 0.0142305046672742 | 1 | 1.84677969792513 | CALCA |
| GO:0042462 | eye photoreceptor cell development | Biological process | 1 | 28 | 7 | 13692 | 69.8571428571428 | 0.0142305046672742 | 1 | 1.84677969792513 | TULP1 |
| GO:0051926 | negative regulation of calcium ion transport | Biological process | 1 | 28 | 7 | 13692 | 69.8571428571428 | 0.0142305046672742 | 1 | 1.84677969792513 | CALCA |
| GO:0030279 | negative regulation of ossification | Biological process | 1 | 29 | 7 | 13692 | 67.448275862069 | 0.0147355095316858 | 1 | 1.83163484230652 | CALCA |
| GO:0009583 | detection of light stimulus | Biological process | 1 | 30 | 7 | 13692 | 65.2 | 0.0152402926271468 | 1 | 1.8170066940768 | TULP1 |
| GO:0010039 | response to iron ion | Biological process | 1 | 30 | 7 | 13692 | 65.2 | 0.0152402926271468 | 1 | 1.8170066940768 | ABCG2 |
| GO:0046850 | regulation of bone remodeling | Biological process | 1 | 30 | 7 | 13692 | 65.2 | 0.0152402926271468 | 1 | 1.8170066940768 | CALCA |
| GO:0031279 | regulation of cyclase activity | Biological process | 1 | 31 | 7 | 13692 | 63.0967741935484 | 0.0157448540348184 | 1 | 1.80286136120507 | CALCA |
| GO:0042312 | regulation of vasodilation | Biological process | 1 | 31 | 7 | 13692 | 63.0967741935484 | 0.0157448540348184 | 1 | 1.80286136120507 | CALCA |
| GO:0043954 | cellular component maintenance | Biological process | 1 | 31 | 7 | 13692 | 63.0967741935484 | 0.0157448540348184 | 1 | 1.80286136120507 | TULP1 |
| GO:0031328 | positive regulation of cellular biosynthetic process | Biological process | 3 | 1153 | 7 | 13692 | 5.08933217692975 | 0.016100830942707 | 1 | 1.7931517100228 | CALCA//PLAGL1//RXRG |
| GO:0032652 | regulation of interleukin-1 production | Biological process | 1 | 32 | 7 | 13692 | 61.125 | 0.0162491938358399 | 1 | 1.78916818061317 | CALCA |
| GO:0048265 | response to pain | Biological process | 1 | 32 | 7 | 13692 | 61.125 | 0.0162491938358399 | 1 | 1.78916818061317 | CALCA |
| GO:0042461 | photoreceptor cell development | Biological process | 1 | 33 | 7 | 13692 | 59.2727272727273 | 0.0167533121113269 | 1 | 1.77589932059877 | TULP1 |
| GO:0009891 | positive regulation of biosynthetic process | Biological process | 3 | 1178 | 7 | 13692 | 4.98132427843803 | 0.0170732487270664 | 1 | 1.7676838327227 | CALCA//PLAGL1//RXRG |
| GO:0050766 | positive regulation of phagocytosis | Biological process | 1 | 34 | 7 | 13692 | 57.5294117647059 | 0.0172572089423702 | 1 | 1.76302944262681 | TULP1 |
| GO:0051339 | regulation of lyase activity | Biological process | 1 | 34 | 7 | 13692 | 57.5294117647059 | 0.0172572089423702 | 1 | 1.76302944262681 | CALCA |
| GO:0007159 | leukocyte cell-cell adhesion | Biological process | 1 | 35 | 7 | 13692 | 55.8857142857143 | 0.0177608844100375 | 1 | 1.75053541215901 | CALCA |
| GO:0001754 | eye photoreceptor cell differentiation | Biological process | 1 | 36 | 7 | 13692 | 54.3333333333333 | 0.0182643385953729 | 1 | 1.738396050229 | TULP1 |
| GO:0032677 | regulation of interleukin-8 production | Biological process | 1 | 37 | 7 | 13692 | 52.8648648648649 | 0.0187675715793976 | 1 | 1.72659191906205 | CALCA |
| GO:0007566 | embryo implantation | Biological process | 1 | 38 | 7 | 13692 | 51.4736842105263 | 0.0192705834431059 | 1 | 1.71510513628977 | CALCA |
| GO:0032612 | interleukin-1 production | Biological process | 1 | 39 | 7 | 13692 | 50.1538461538462 | 0.0197733742674723 | 1 | 1.70391921330134 | CALCA |
| GO:0032637 | interleukin-8 production | Biological process | 1 | 39 | 7 | 13692 | 50.1538461538462 | 0.0197733742674723 | 1 | 1.70391921330134 | CALCA |
| GO:0045670 | regulation of osteoclast differentiation | Biological process | 1 | 39 | 7 | 13692 | 50.1538461538462 | 0.0197733742674723 | 1 | 1.70391921330134 | CALCA |
| GO:0002763 | positive regulation of myeloid leukocyte differentiation | Biological process | 1 | 40 | 7 | 13692 | 48.9 | 0.0202759441334451 | 1 | 1.69301891406384 | CALCA |
| GO:0034103 | regulation of tissue remodeling | Biological process | 1 | 40 | 7 | 13692 | 48.9 | 0.0202759441334451 | 1 | 1.69301891406384 | CALCA |
| GO:0007189 | adenylate cyclase-activating G-protein coupled receptor signaling pathway | Biological process | 1 | 41 | 7 | 13692 | 47.7073170731707 | 0.0207782931219501 | 1 | 1.68239013137773 | CALCA |
| GO:0045778 | positive regulation of ossification | Biological process | 1 | 42 | 7 | 13692 | 46.5714285714286 | 0.0212804213138885 | 1 | 1.6720197780459 | CALCA |
| GO:0045776 | negative regulation of blood pressure | Biological process | 1 | 43 | 7 | 13692 | 45.4883720930233 | 0.0217823287901376 | 1 | 1.66189569085021 | CALCA |
| GO:0010522 | regulation of calcium ion transport into cytosol | Biological process | 1 | 45 | 7 | 13692 | 43.4666666666667 | 0.0227854819189625 | 1 | 1.64234178154136 | CALCA |
| GO:0046530 | photoreceptor cell differentiation | Biological process | 1 | 45 | 7 | 13692 | 43.4666666666667 | 0.0227854819189625 | 1 | 1.64234178154136 | TULP1 |
| GO:0002027 | regulation of heart rate | Biological process | 1 | 46 | 7 | 13692 | 42.5217391304348 | 0.0232867277331757 | 1 | 1.63289153453399 | CALCA |
| GO:0006940 | regulation of smooth muscle contraction | Biological process | 1 | 46 | 7 | 13692 | 42.5217391304348 | 0.0232867277331757 | 1 | 1.63289153453399 | CALCA |
| GO:0050764 | regulation of phagocytosis | Biological process | 1 | 46 | 7 | 13692 | 42.5217391304348 | 0.0232867277331757 | 1 | 1.63289153453399 | TULP1 |
| GO:0031645 | negative regulation of neurological system process | Biological process | 1 | 47 | 7 | 13692 | 41.6170212765957 | 0.0237877531549745 | 1 | 1.62364657681429 | CALCA |
| GO:0045453 | bone resorption | Biological process | 1 | 47 | 7 | 13692 | 41.6170212765957 | 0.0237877531549745 | 1 | 1.62364657681429 | CALCA |
| GO:0031667 | response to nutrient levels | Biological process | 2 | 490 | 7 | 13692 | 7.98367346938775 | 0.0238184291586701 | 1 | 1.62308688392029 | RXRG//ABCG2 |
| GO:0044057 | regulation of system process | Biological process | 2 | 495 | 7 | 13692 | 7.9030303030303 | 0.0242775825723387 | 1 | 1.61479456009111 | CALCA//RXRG |
| GO:0007200 | phospholipase C-activating G-protein coupled receptor signaling pathway | Biological process | 1 | 48 | 7 | 13692 | 40.75 | 0.0242885582651189 | 1 | 1.61459826354342 | CALCA |
| GO:0030819 | positive regulation of cAMP biosynthetic process | Biological process | 1 | 50 | 7 | 13692 | 39.12 | 0.0252895078733613 | 1 | 1.59705962182061 | CALCA |
| GO:0043271 | negative regulation of ion transport | Biological process | 1 | 50 | 7 | 13692 | 39.12 | 0.0252895078733613 | 1 | 1.59705962182061 | CALCA |
| GO:0030816 | positive regulation of cAMP metabolic process | Biological process | 1 | 51 | 7 | 13692 | 38.3529411764706 | 0.0257896525328605 | 1 | 1.58855450912526 | CALCA |
| GO:0009991 | response to extracellular stimulus | Biological process | 2 | 515 | 7 | 13692 | 7.59611650485437 | 0.0261519153960782 | 1 | 1.58249649740917 | RXRG//ABCG2 |
| GO:0045638 | negative regulation of myeloid cell differentiation | Biological process | 1 | 52 | 7 | 13692 | 37.6153846153846 | 0.0262895772035054 | 1 | 1.58021639828546 | CALCA |
| GO:0031623 | receptor internalization | Biological process | 1 | 53 | 7 | 13692 | 36.9056603773585 | 0.026789281965938 | 1 | 1.57203892664599 | CALCA |
| GO:0030316 | osteoclast differentiation | Biological process | 1 | 55 | 7 | 13692 | 35.5636363636364 | 0.0277880320886099 | 1 | 1.5561422082899 | CALCA |
| GO:0042311 | vasodilation | Biological process | 1 | 55 | 7 | 13692 | 35.5636363636364 | 0.0277880320886099 | 1 | 1.5561422082899 | CALCA |
| GO:0045744 | negative regulation of G-protein coupled receptor protein signaling pathway | Biological process | 1 | 57 | 7 | 13692 | 34.3157894736842 | 0.028785903545532 | 1 | 1.54082013415465 | CALCA |
| GO:0030804 | positive regulation of cyclic nucleotide biosynthetic process | Biological process | 1 | 60 | 7 | 13692 | 32.6 | 0.0302810646418877 | 1 | 1.51882885968819 | CALCA |
| GO:0007422 | peripheral nervous system development | Biological process | 1 | 61 | 7 | 13692 | 32.0655737704918 | 0.0307790130388583 | 1 | 1.5117453103866 | RXRG |
| GO:0030810 | positive regulation of nucleotide biosynthetic process | Biological process | 1 | 61 | 7 | 13692 | 32.0655737704918 | 0.0307790130388583 | 1 | 1.5117453103866 | CALCA |
| GO:1900373 | positive regulation of purine nucleotide biosynthetic process | Biological process | 1 | 61 | 7 | 13692 | 32.0655737704918 | 0.0307790130388583 | 1 | 1.5117453103866 | CALCA |
| GO:0045639 | positive regulation of myeloid cell differentiation | Biological process | 1 | 62 | 7 | 13692 | 31.5483870967742 | 0.0312767422523226 | 1 | 1.50477848884493 | CALCA |
| GO:0006873 | cellular ion homeostasis | Biological process | 2 | 568 | 7 | 13692 | 6.88732394366197 | 0.031404261008943 | 1 | 1.50301142176363 | CALCA//RXRG |
| GO:0030801 | positive regulation of cyclic nucleotide metabolic process | Biological process | 1 | 63 | 7 | 13692 | 31.047619047619 | 0.0317742523626842 | 1 | 1.4979246594547 | CALCA |
| GO:0045981 | positive regulation of nucleotide metabolic process | Biological process | 1 | 65 | 7 | 13692 | 30.0923076923077 | 0.0327686155956035 | 1 | 1.48454190624931 | CALCA |
| GO:1900544 | positive regulation of purine nucleotide metabolic process | Biological process | 1 | 65 | 7 | 13692 | 30.0923076923077 | 0.0327686155956035 | 1 | 1.48454190624931 | CALCA |
| GO:0033555 | multicellular organismal response to stress | Biological process | 1 | 67 | 7 | 13692 | 29.1940298507463 | 0.0337621033803822 | 1 | 1.47157050464005 | CALCA |
| GO:0046849 | bone remodeling | Biological process | 1 | 67 | 7 | 13692 | 29.1940298507463 | 0.0337621033803822 | 1 | 1.47157050464005 | CALCA |
| GO:0030817 | regulation of cAMP biosynthetic process | Biological process | 1 | 70 | 7 | 13692 | 27.9428571428571 | 0.0352506949974105 | 1 | 1.45283231610337 | CALCA |
| GO:0060402 | calcium ion transport into cytosol | Biological process | 1 | 71 | 7 | 13692 | 27.5492957746479 | 0.0357464551746989 | 1 | 1.44676701888102 | CALCA |
| GO:0007218 | neuropeptide signaling pathway | Biological process | 1 | 72 | 7 | 13692 | 27.1666666666667 | 0.0362419969714668 | 1 | 1.44078788026139 | CALCA |
| GO:0060401 | cytosolic calcium ion transport | Biological process | 1 | 72 | 7 | 13692 | 27.1666666666667 | 0.0362419969714668 | 1 | 1.44078788026139 | CALCA |
| GO:0048522 | positive regulation of cellular process | Biological process | 4 | 2813 | 7 | 13692 | 2.78137220049769 | 0.0365255121183517 | 1 | 1.43740368617765 | CALCA//PLAGL1//RXRG//TULP1 |
| GO:0003073 | regulation of systemic arterial blood pressure | Biological process | 1 | 73 | 7 | 13692 | 26.7945205479452 | 0.0367373204678838 | 1 | 1.43489252327382 | CALCA |
| GO:0030814 | regulation of cAMP metabolic process | Biological process | 1 | 73 | 7 | 13692 | 26.7945205479452 | 0.0367373204678838 | 1 | 1.43489252327382 | CALCA |
| GO:0055082 | cellular chemical homeostasis | Biological process | 2 | 624 | 7 | 13692 | 6.26923076923077 | 0.0373874077889543 | 1 | 1.42727464508861 | CALCA//RXRG |
| GO:0050801 | ion homeostasis | Biological process | 2 | 626 | 7 | 13692 | 6.24920127795527 | 0.0376090607768258 | 1 | 1.42470751221799 | CALCA//RXRG |
| GO:0002761 | regulation of myeloid leukocyte differentiation | Biological process | 1 | 75 | 7 | 13692 | 26.08 | 0.0377273128802235 | 1 | 1.4233441262289 | CALCA |
| GO:0045807 | positive regulation of endocytosis | Biological process | 1 | 75 | 7 | 13692 | 26.08 | 0.0377273128802235 | 1 | 1.4233441262289 | TULP1 |
| GO:0006939 | smooth muscle contraction | Biological process | 1 | 77 | 7 | 13692 | 25.4025974025974 | 0.0387164330525991 | 1 | 1.41210466109843 | CALCA |
| GO:0010604 | positive regulation of macromolecule metabolic process | Biological process | 3 | 1599 | 7 | 13692 | 3.66979362101313 | 0.0387378117799737 | 1 | 1.41186491533408 | CALCA//PLAGL1//RXRG |
| GO:0000910 | cytokinesis | Biological process | 1 | 79 | 7 | 13692 | 24.7594936708861 | 0.039704681625516 | 1 | 1.4011582820466 | KIF20A |
| GO:0031325 | positive regulation of cellular metabolic process | Biological process | 3 | 1621 | 7 | 13692 | 3.61998766193708 | 0.0401516183643172 | 1 | 1.39629694521649 | CALCA//PLAGL1//RXRG |
| GO:0006171 | cAMP biosynthetic process | Biological process | 1 | 81 | 7 | 13692 | 24.1481481481481 | 0.0406920592391034 | 1 | 1.39049033193215 | CALCA |
| GO:0006909 | phagocytosis | Biological process | 1 | 85 | 7 | 13692 | 23.0117647058824 | 0.0426642041469228 | 1 | 1.36993635123428 | TULP1 |
| GO:0009408 | response to heat | Biological process | 1 | 85 | 7 | 13692 | 23.0117647058824 | 0.0426642041469228 | 1 | 1.36993635123428 | CALCA |
| GO:0030802 | regulation of cyclic nucleotide biosynthetic process | Biological process | 1 | 85 | 7 | 13692 | 23.0117647058824 | 0.0426642041469228 | 1 | 1.36993635123428 | CALCA |
| GO:1900371 | regulation of purine nucleotide biosynthetic process | Biological process | 1 | 85 | 7 | 13692 | 23.0117647058824 | 0.0426642041469228 | 1 | 1.36993635123428 | CALCA |
| GO:0045944 | positive regulation of transcription from RNA polymerase II promoter | Biological process | 2 | 674 | 7 | 13692 | 5.80415430267062 | 0.043088397932828 | 1 | 1.36563965308886 | PLAGL1//RXRG |
| GO:0001935 | endothelial cell proliferation | Biological process | 1 | 86 | 7 | 13692 | 22.7441860465116 | 0.0431566970234549 | 1 | 1.36495180123146 | CALCA |
| GO:0006937 | regulation of muscle contraction | Biological process | 1 | 86 | 7 | 13692 | 22.7441860465116 | 0.0431566970234549 | 1 | 1.36495180123146 | CALCA |
| GO:0030808 | regulation of nucleotide biosynthetic process | Biological process | 1 | 86 | 7 | 13692 | 22.7441860465116 | 0.0431566970234549 | 1 | 1.36495180123146 | CALCA |
| GO:0042552 | myelination | Biological process | 1 | 89 | 7 | 13692 | 21.9775280898876 | 0.0446328728895681 | 1 | 1.35034515797393 | RXRG |
| GO:0007188 | adenylate cyclase-modulating G-protein coupled receptor signaling pathway | Biological process | 1 | 90 | 7 | 13692 | 21.7333333333333 | 0.0451244975230886 | 1 | 1.34558762107886 | CALCA |
| GO:0019725 | cellular homeostasis | Biological process | 2 | 696 | 7 | 13692 | 5.62068965517241 | 0.0456999752548394 | 1 | 1.34008403508747 | CALCA//RXRG |
| GO:0007272 | ensheathment of neurons | Biological process | 1 | 92 | 7 | 13692 | 21.2608695652174 | 0.046107096285863 | 1 | 1.33623222774232 | RXRG |
| GO:0008366 | axon ensheathment | Biological process | 1 | 92 | 7 | 13692 | 21.2608695652174 | 0.046107096285863 | 1 | 1.33623222774232 | RXRG |
| GO:0072358 | cardiovascular system development | Biological process | 2 | 701 | 7 | 13692 | 5.58059914407989 | 0.0463020981745501 | 1 | 1.33439932852885 | CALCA//RXRG |
| GO:0072359 | circulatory system development | Biological process | 2 | 701 | 7 | 13692 | 5.58059914407989 | 0.0463020981745501 | 1 | 1.33439932852885 | CALCA//RXRG |
| GO:0030799 | regulation of cyclic nucleotide metabolic process | Biological process | 1 | 93 | 7 | 13692 | 21.0322580645161 | 0.0465980705745397 | 1 | 1.33163206520223 | CALCA |
| GO:0043542 | endothelial cell migration | Biological process | 1 | 94 | 7 | 13692 | 20.8085106382979 | 0.0470888282409841 | 1 | 1.32708211641237 | CALCA |
| GO:0007631 | feeding behavior | Biological process | 1 | 95 | 7 | 13692 | 20.5894736842105 | 0.0475793693648485 | 1 | 1.3225813185657 | CALCA |
| GO:0009893 | positive regulation of metabolic process | Biological process | 3 | 1731 | 7 | 13692 | 3.38994800693241 | 0.0476464217790843 | 1 | 1.32196970908546 | CALCA//PLAGL1//RXRG |
| GO:0008277 | regulation of G-protein coupled receptor protein signaling pathway | Biological process | 1 | 96 | 7 | 13692 | 20.375 | 0.0480696940257628 | 1 | 1.31812864224301 | CALCA |
| GO:0046058 | cAMP metabolic process | Biological process | 1 | 97 | 7 | 13692 | 20.1649484536082 | 0.0485598023033313 | 1 | 1.31372309002891 | CALCA |
| GO:0016192 | vesicle-mediated transport | Biological process | 2 | 723 | 7 | 13692 | 5.41078838174274 | 0.0489886275311371 | 1 | 1.30990472759824 | CALCA//TULP1 |
| GO:0007187 | G-protein coupled receptor signaling pathway, coupled to cyclic nucleotide second messenger | Biological process | 1 | 98 | 7 | 13692 | 19.9591836734694 | 0.0490496942771357 | 1 | 1.30936369519875 | CALCA |
| GO:0008016 | regulation of heart contraction | Biological process | 1 | 99 | 7 | 13692 | 19.7575757575758 | 0.0495393700267356 | 1 | 1.30504952047227 | CALCA |
