# Supplementary material for: Gene Expression in the Hippocampus in a Rat Model of Premenstrual Dysphoric Disorder After Treatment With Baixiangdan Capsules
Source: Front Psychol. 2018 Nov 13;9:2065. doi: 10.3389/fpsyg.2018.02065 (PMC6242977; doi:10.3389/fpsyg.2018.02065)
Supplement: Supplementary file 1 [file Data_Sheet_1.ZIP › File 1. Summary & QC/Agilent Gene Expression Profiling Service Report Instruction.doc]

**Agilent Gene Expression** **Profiling Service Report**

Monday, 30 November 2026

***Project:*	Gene** **expression profiling for 12 Rat samples**

***Customer:*	孙鹏**

***Institute:* 山东中医药大学**

***Project Code:*	H1306079**

**Contents of This Service Report:**

- **Summary:** An abstract of the experiment and the data analysis.
- **Experiment Workflow:** A workflow of the experiment and the data analysis.
- **Project Description:** Sample and experiment information.
- **Array Information:** Rat 4x44K gene expression array information.
- **Summary Table of Files for Data Delivery:** Contains summary table of files for data delivery and the recommended software programs for viewing the data.
- **Data Analysis:**

1. **Raw gene data normalization and low intensity filtering:** Raw signal intensities were normalized in quantile method by GeneSpring GX v11.5, and low intensity genes were filtered (Genes that at least 12 out of 12 samples have flags in Detected were chosen for further analysis).
2. **Quality assessment of gene data after filtering:** Contains Box Plot and Scatter Plot for genes after filtering.
3. **Differentially expressed genes screening:** Contains differentially expressed genes with statistical significance between two groups that passed Volcano Plot filtering (Fold Change>=2.0, P-value<=0.05).
4. **Heat map and hierarchical clustering:** Hierarchical Clustering of Differentially Expressed Genes.
5. **Pathway analysis:** Pathway analysis of the differentially expressed genes.
6. **GO analysis:** GO term analysis of the differentially expressed genes.

- **Sample RNA Quality Control:** Sample quality control data file from NanoDrop ND-1000 spectrophotometer and standard denaturing agarose gel electrophoresis.
- **Methods:** A brief introduction of methods for sample preparation, microarray design, experiment, and data analysis.
- **FAQ:** Frequently Asked Questions.

**Summary**

Dear Sun,

We have completed the gene microarray analysis of the 12 samples that you submitted. Total RNA from each sample was quantified by the NanoDrop ND-1000 and RNA integrity was assessed by standard denaturing agarose gel electrophoresis. For microarray analysis, Agilent Array platform was employed. The sample preparation and microarray hybridization were performed based on the manufacturer’s standard protocols. Briefly, total RNA from each sample was amplified and transcribed into fluorescent cRNA with using the manufacturer’s Agilent’s Quick Amp Labeling protocol (version 5.7, Agilent Technologies). The labeled cRNAs were hybridized onto the Whole Genome Oligo Array (4x44K, Agilent Technologies). After having washed the slides, the arrays were scanned by the Agilent Scanner G2505C.

Agilent Feature Extraction software (version 11.0.1.1) was used to analyze acquired array images. Quantile normalization and subsequent data processing were performed using the GeneSpring GX v11.5 software package (Agilent Technologies). After quantile normalization of the raw data, genes that at least 12 out of 12 samples have flags in Detected (“All Targets Value”) were chosen for further data analysis. Differentially expressed genes with statistical significance between two groups were identified through Volcano Plot filtering. Pathway analysis and GO analysis were applied to determine the roles of these differentially expressed genes played in these biological pathways or GO terms. Finally, Hierarchical Clustering was performed to show the distinguishable gene expression pattern among samples.

Respectfully,

KangChen Services

KangChen Inc.

**Experiment Workflow**

**RNA Extraction and RNA QC**

Integrity and concentration of RNA were assessed after RNA extraction and prior to sample labeling. Results were provided in the *RNA_QC.pdf* file.

**Labeling and Hybridization**

Agilent Quick Amp Labeling Kit was used for sample labeling. Hybridization was performed in Agilent’s SureHyb Hybridization Chambers.

**Data Collection and Normalization**

After washing, slides were scanned with the Agilent DNA microarray Scanner. Data was extracted and normalized using Agilent Feature Extraction Software (version 11.0.1.1). Normalization and subsequent data processing were performed using the Agilent GeneSpring GX v11.5.

**Further Data Analysis**

Further Data analysis was performed using Agilent GeneSpring GX v11.5 software. Results were provided in the *Gene Expression Profiling Data.xls* file and *Data Analysis Folder*.

**Project Description**

**(Project Title/Code: H1306079)**

**Sample Description**

| **Organism** | **Rat** |
| --- | --- |
| **Sample Type** | **Tissue** |

| **Sample ID** | **Sample Name** | **Group Name** |
| --- | --- | --- |
| **1** | **3_1(3 1 背)** | **blank(空白组)** |
| **2** | **6_25(6 25 空)** |  |
| **3** | **21_20(21 20 头)** |  |
| **4** | **15_11(15 11 全)** | **model(模型组)** |
| **5** | **17_4(17 4 颈)** |  |
| **6** | **17_11(17 11 空)** |  |
| **7** | **2_26(2 26 尾)** | **fluoxetine(氟西汀)** |
| **8** | **18_8(18 8 全)** |  |
| **9** | **24_14(24 14 空)** |  |
| **10** | **4_4(4 4 空)** | **BXD(白香丹)** |
| **11** | **14_21(14 21 背)** |  |
| **12** | **24_22(24 22 空)** |  |

**Sample Tracking (Hybridizations to be performed)**

|  | **Array1_1** | | **Array1_2** | | **Array1_3** | | **Array1_4** | |
| --- | --- | --- | --- | --- | --- | --- | --- | --- |
| **Dye** | **Cy3** | **Cy5** | **Cy3** | **Cy5** | **Cy3** | **Cy5** | **Cy3** | **Cy5** |
| **Slide1** | **/** | **/** | **/** | **/** | **3** | **/** | **4** | **/** |

|  | **Array1_1** | | **Array1_2** | | **Array1_3** | | **Array1_4** | |
| --- | --- | --- | --- | --- | --- | --- | --- | --- |
| **Dye** | **Cy3** | **Cy5** | **Cy3** | **Cy5** | **Cy3** | **Cy5** | **Cy3** | **Cy5** |
| **Slide2** | **1** | **/** | **2** | **/** | **5** | **/** | **7** | **/** |

|  | **Array1_1** | | **Array1_2** | | **Array1_3** | | **Array1_4** | |
| --- | --- | --- | --- | --- | --- | --- | --- | --- |
| **Dye** | **Cy3** | **Cy5** | **Cy3** | **Cy5** | **Cy3** | **Cy5** | **Cy3** | **Cy5** |
| **Slide3** | **/** | **/** | **6** | **/** | **/** | **/** | **8** | **/** |

|  | **Array1_1** | | **Array1_2** | | **Array1_3** | | **Array1_4** | |
| --- | --- | --- | --- | --- | --- | --- | --- | --- |
| **Dye** | **Cy3** | **Cy5** | **Cy3** | **Cy5** | **Cy3** | **Cy5** | **Cy3** | **Cy5** |
| **Slide4** | **9** | **/** | **10** | **/** | **11** | **/** | **12** | **/** |

**Experiment Status**

|  | **Status** |
| --- | --- |
| **Number of Slides** | **4** |
| **Final Submission Form Received** | **OK** |
| **RNA Isolation** | **OK** |
| **RNA QC** | **OK** please see the “RNA_QC.pdf” file |
| **Labeling and Hybridization** | **OK** |
| **Scanning and Data Analysis** | **OK** |

**Array Information**

**Agilent Rat 4x44K Gene Expression Microarrays**

The Whole Rat Genome Oligo Microarray Kit is a tool for modeling human biology in the rat model organism. It is a broad view that represents well-known rat genes and transcripts, including homologues to human and mouse genes. Coupled with Agilent's probe selection and robust validation processes, this design delivers increased data quality and less gene coverage redundancy.

- Updated content providing full coverage of rat genes and transcripts
- Content sourced from RefSeq, Ensembl, Unigene, and GenBank databases
- Empirically validated probes of the highest quality printed using Agilent's 60-mer SurePrint technology

**Summary Table of Files for Data Delivery**

The following table lists all the files containing data analysis results, the recommended software programs for viewing the corresponding data file were also shown in the table.

| **Data Folder** | **Files** | **Description** | **Viewing Software** |
| --- | --- | --- | --- |
| Summary & QC | RNA QC.pdf  Labeling Report.pdf | RNA QC report and labeling QC report | Adobe Reader |
| Graph | *.jpg | Array image of each sample | Windows Picture and Fax Viewer |
| Data Analysis Folder | Differentially Expressed Genes (Pass Volcano Plot).xls | Contains significant differentially expressed genes that passed Volcano Plot filtering | Excel |
| GO Analysis Report | summary_GO.doc  model vs blank (up)  …  BXD vs fluoxetine (down) | GO analysis of the differentially expressed genes | Word  Excel |
| Pathway Analysis Report | summary_PATHWAY.doc  model vs blank (up)  …  BXD vs fluoxetine (down) | Pathway analysis of the differentially expressed genes | Word  Excel |
| Data | Raw Intensity.xls | Raw intensity extracted by Feature Extraction software | Excel |
| Data | Gene Expression Profiling Data.xls | Gene intensities after filtering (All Targets Value), quality control (Box Plot, Scatter Plot) and hierarchical clustering maps | Excel |

**Data Analysis**

**Raw gene data normalization and low intensity filtering**

Quantile normalization and subsequent data processing were performed using the GeneSpring GX v11.5 software package (Agilent Technologies). After Quantile normalization of the raw data, genes that at least 12 out of 12 samples have flags in Detected (“All Targets Value”) were chosen for differentially expressed genes screening (All these genes can be found from the spread sheet “All Targets Value” in *Gene Expression Profiling Data.xls* file)

The following list only shows part of the whole results. The whole results can be found from the spread sheet “All Targets Value” in [*Gene Expression Profiling Data.xls*](../../File%202.%20Data/Gene%20Expression%20Profiling%20Data.xls) file.

| **ProbeName** | **[14_21](raw)** | **...** | **[6_25](raw)** | **[14_21](normalized)** | **...** | **[6_25](normalized)** | **GenbankAccession** | **...** |
| --- | --- | --- | --- | --- | --- | --- | --- | --- |
| A_64_P002176 | 609.7896 | ... | 456.72357 | 9.08703 | ... | 9.633996 | NM_001170551 | ... |
| A_42_P664913 | 796.6073 | ... | 334.0386 | 9.455837 | ... | 9.158989 | NM_001108400 | ... |
| A_43_P11804 | 18914.623 | ... | 10602.032 | 13.772478 | ... | 14.295478 | NM_017066 | ... |
| A_64_P142111 | 828.5772 | ... | 706.9314 | 9.512397 | ... | 10.288062 | NM_001100887 | ... |
| A_64_P095642 | 165.23126 | ... | 112.14598 | 7.255642 | ... | 7.4701304 |  | ... |
| A_42_P735279 | 30046.56 | ... | 11730.855 | 14.419001 | ... | 14.448448 | NM_001004446 | ... |
| A_42_P610788 | 11607.844 | ... | 2710.0454 | 13.064085 | ... | 12.309357 | NM_031544 | ... |
| A_44_P242429 | 3473.6313 | ... | 1186.767 | 11.437127 | ... | 11.060878 | NM_001134842 | ... |
| A_64_P020571 | 408.3537 | ... | 225.18883 | 8.523636 | ... | 8.558477 | NM_001031659 | ... |
| A_42_P518462 | 9018.054 | ... | 3665.117 | 12.7299595 | ... | 12.744089 | NM_001007020 | ... |
| ... | ... | ... | ... | ... | ... | ... | ... | ... |

**ProbeName:** Probe name.

**[14_21] ~ [6_25] (raw):** Raw intensities of each sample.

**[14_21] ~ [6_25] (normalized):** Intensities after quantile normalization (log2 transformed).

**GenbankAccession:** GenBank accession number.

**Quality assessment of gene data after filtering**

**Box-Plot**

The Box Plot is a convenient way to quickly visualize the distributions of a dataset. It is commonly used for comparing the distributions of the intensities from all samples. After normalization, the distributions of log2-ratios among all samples are nearly the same.

**Note:** The Box-Plot can be found from the sheet “Box Plot” in [*Gene Expression Profiling Data.xls*](../../File%202.%20Data/Gene%20Expression%20Profiling%20Data.xls) file.

**Scatter-Plot**

The Scatter-Plot is a visualization method used for assessing the gene expression variation (or reproducibility) between arrays. The values of X and Y axes in the Scatter-Plot are the normalized signal values of the compared samples or averaged normalized signal values of the compared groups. The green lines are Fold Change Lines (The default fold change value given is 2.0). The genes above the top green line and below the bottom green line indicated more than 2.0 fold change of genes between two samples or groups.

There are 6 Scatter-Plots provided in the data report.

**Scatter-Plot for model vs blank**

**…**

**Scatter-Plot for BXD vs fluoxetine**

The following Scatter-Plot is for **model vs blank**.

**Note:** All Scatter-Plots can be found from the sheet “Scatter Plot” in [*Gene Expression Profiling Data.xls*](../../File%202.%20Data/Gene%20Expression%20Profiling%20Data.xls) file.

**Differentially expressed genes screening**

To identify differentially expressed genes with statistical significance, we performed a Volcano Plot filtering between two groups. The threshold is Fold Change>=2.0, P-value<=0.05.

There are 6 comparison pairs provided in the data report.

**Differentially expressed genes in model vs blank**

**…**

**Differentially expressed genes in BXD vs fluoxetine**

The following list only shows part of **differentially expressed genes in model vs blank** results.

**Note:** The whole differentially expressed gene results can be found from [*Differentially Expressed Genes (Pass Volcano Plot).xls*](../../File%202.%20Data/Data%20Analysis%20Folder/Differentially%20Expressed%20Genes%20(Pass%20Volcano%20Plot).xls) file in Data Analysis Folder.

| **model vs blank 2 fold up regulated genes** | | | | | | | | | | |
| --- | --- | --- | --- | --- | --- | --- | --- | --- | --- | --- |
| **ProbeName** | **p** | | **FC(abs)** | | **Regulation** | **GenBank Accession** | **…** | **model(normalized)** | **blank(normalized)** | **…** |
| A_64_P144869 | 0.0046107 | | 2.0352097 | | up | NM_017131 | … | 8.8334683 | 7.8082918 | … |
| A_42_P571433 | 6.46E-04 | | 3.5419128 | | up | NM_031059 | … | 13.11772 | 11.29319 | … |
| A_42_P575104 | 1.29E-04 | | 147.71477 | | up | NM_153737 | … | 14.431889 | 7.2252194 | … |
| A_44_P991940 | 0.0299501 | | 2.305092 | | up | XM_225667 | … | 7.5957395 | 6.390915 | … |
| A_44_P596424 | 0.0029549 | | 2.141268 | | up |  | … | 8.8481768 | 7.7497117 | … |
| A_42_P521242 | 2.56E-02 | | 6.287422 | | up | XM_213312 | … | 11.109527 | 8.4570583 | … |
| A_64_P005288 | 0.0297912 | | 4.0046763 | | up | XM_575125 | … | 8.0570265 | 6.0553414 | … |
| … | … | | … | | … | … | … | … | … | … |
|  | | | | | | | | | | |
| **model vs blank 2 fold down regulated genes** | | | | | | | | | | |
| **ProbeName** | **p** | **FC(abs)** | | **Regulation** | | **GenBank Accession** | **…** | **model(normalized)** | **blank(normalized)** | **…** |
| A_44_P852695 | 0.0344493 | 2.4773567 | | down | |  | … | 10.16054 | 11.469343 | … |
| A_43_P12808 | 0.0276143 | 2.000254 | | down | | NM_053457 | … | 11.423376 | 12.423559 | … |
| A_64_P102158 | 0.0070401 | 2.0407999 | | down | | XM_002726376 | … | 11.838153 | 12.867288 | … |
| A_43_P19742 | 0.0207635 | 3.4026117 | | down | | NM_001134614 | … | 9.2632267 | 11.029869 | … |
| A_44_P370355 | 0.0359904 | 2.0728855 | | down | |  | … | 10.688304 | 11.739945 | … |
| A_64_P103537 | 0.0288134 | 2.4374952 | | down | | NM_031506 | … | 7.132065 | 8.4174637 | … |
| A_64_P074340 | 0.0439919 | 2.0583696 | | down | | XM_002726502 | … | 11.551345 | 12.592847 | … |
| … | … | … | | … | | … | … | … | … | … |

**ProbeName:** Probe name.

**p:** P-value calculated from t-test

**FC(abs):** Absolute fold change between two compared groups.

**Regulation:** “up” indicates up-regulation; “down” indicates down-regulation.

**GenbankAccession:** GenBank Accession.

**[model] ~ [blank] (normalized):** Averaged normalized intensities of the groups (log2 transformed).

**Heat map and hierarchical clustering**

Hierarchical Clustering is one of the most widely used clustering methods for analyzing gene expression data. Cluster analysis arranges samples into groups based on their expression levels, which allows us to hypothesize the relationships among samples. The dendrogram shows the relationships among gene expression patterns of samples.

There are 6 clusters provided in data report.

**Cluster for Differentially Expressed Genes in model vs blank**

**…**

**Cluster for Differentially Expressed Genes in BXD vs fluoxetine**

The following Hierarchical Clustering was performed based on “Differentially Expressed Genes in model vs blank”. The result of Hierarchical Clustering shows a distinguishable gene expression profiling among samples.

**Note:** All heat maps can be found in [*Gene Expression Profiling Data.xls*](../../File%202.%20Data/Gene%20Expression%20Profiling%20Data.xls) file.

Hierarchical Clustering for “Differentially Expressed Genes in model vs blank”.

“Red” indicates high relative expression, and “blue” indicates low relative expression.

**Pathway analysis**

Base on the latest KEGG (Kyoto Encyclopedia of Genes and Genomes) database, we provide pathway analysis by differentially expressed genes. This analysis allows users to determine the biological pathway that there is a significant enrichment of differentially expressed genes. The P-value denotes the significance of the Pathway. The less the P-value is, the more significant of the Pathway is (The p-value cut-off is 0.05).

There are 12 Pathway analysis folders provided in the data report.

**Pathway analysis of differentially expressed genes in model vs blank (up)**

**…**

**Pathway analysis of differentially expressed genes in BXD vs fluoxetine (down)**

The following list only shows part of **pathway analysis of differentially expressed genes in model vs blank (up)** results.

**Note:** The whole pathway analysis results can be found from [*Pathway Analysis Report*](../../File%202.%20Data/Data%20Analysis%20Folder/Pathway%20Analysis%20Report) folder in the *Data Analysis Folder*.

| **Pathway analysis for model vs blank (up) differentially expressed gene list** | | | | | | |
| --- | --- | --- | --- | --- | --- | --- |
| **PathwayID** | **Definition** | **...** | **Fisher-Pvalue** | **...** | **Enrichment_Score** | **Genes** |
| rno04670 | Leukocyte transendothelial migration - Rattus norvegicus (rat) | ... | 0.001900687 | ... | 2.721089 | CLDN1//CLDN19//CLDN3//MMP2 |
| rno04530 | Tight junction - Rattus norvegicus (rat) | ... | 0.003116352 | ... | 2.506353 | CLDN1//CLDN19//CLDN3//CRB3 |
| rno04976 | Bile secretion - Rattus norvegicus (rat) | ... | 0.004248878 | ... | 2.371726 | AQP1//SLC4A2//SLCO1A5 |
| rno04514 | Cell adhesion molecules (CAMs) - Rattus norvegicus (rat) | ... | 0.006538567 | ... | 2.184517 | CDH3//CLDN1//CLDN19//CLDN3 |
| rno05160 | Hepatitis C - Rattus norvegicus (rat) | ... | 0.01879833 | ... | 1.725881 | CLDN1//CLDN19//CLDN3 |

**PathwayID:** KEGG pathway ID.

**Definition:** Name of the pathway.

**Fisher-Pvalue:** P-value is calculated by Fisher’s exact test (Cut-off is P-value <= 0.05).

**Enrichment_Score:** It equals “-log10 (P-value)”.

**Genes:** Differentially expressed gene list in the pathway.

**GO analysis**

GO analysis is a functional analysis associating differentially expressed genes with GO categories. The GO categories are derived from Gene Ontology ([www.geneontology.org](http://www.geneontology.org/)), which comprises three structured networks of defined terms that describe gene product attributes. The P-value denotes the significance of GO Term enrichment in the differentially expressed gene list. The less the P-value is, the more significant of the GO Term is (P-value <= 0.05 is recommended).

There are 12 GO analysis folders provided in the data report.

**GO analysis of differentially expressed genes in model vs blank (up)**

**…**

**GO analysis of differentially expressed genes in BXD vs fluoxetine (down)**

The following list only shows part of **GO analysis of differentially expressed genes in model vs blank (up)** results.

**Note**: The whole GO analysis results can be found from [*GO Analysis Report*](../../File%202.%20Data/Data%20Analysis%20Folder/GO%20Analysis%20Report) folder in the *Data Analysis Folder*.

| **GO analysis for model vs blank (up) differentially expressed gene list** | | | | | | |
| --- | --- | --- | --- | --- | --- | --- |
| **Biological_Process** | | | | | | |
| **GO.ID** | **Term** | **...** | **Pvalue** | **FDR** | **...** | **GENES** |
| GO:0009887 | organ morphogenesis | ... | 1.76318E-06 | 0.007729765 | ... | COL11A1//FOXJ1… |
| GO:0051179 | localization | ... | 1.50378E-05 | 0.027802355 | ... | NR2F2//XCL1… |
| GO:0003007 | heart morphogenesis | ... | 1.90253E-05 | 0.027802355 | ... | FOXJ1//MESP1… |
| GO:0007204 | elevation of cytosolic calcium ion concentration | ... | 2.69128E-05 | 0.029496389 | ... | DHRS7C//CASQ2… |
| ... | ... | ... | ... | ... | ... | ... |
|  | | | | | | |
| **Cellular_Component** | | | | | | |
| **GO.ID** | **Term** | **...** | **Pvalue** | **FDR** | **...** | **GENES** |
| GO:0005903 | brush border | ... | 0.000275602 | 0.079210002 | ... | AQP1//LRP2… |
| GO:0030286 | dynein complex | ... | 0.00039639 | 0.079210002 | ... | DYNLRB2//DNAH1… |
| GO:0005875 | microtubule associated complex | ... | 0.000442514 | 0.079210002 | ... | DYNLRB2//DNAH1… |
| GO:0031526 | brush border membrane | ... | 0.001318708 | 0.157335107 | ... | AQP1//LRP2… |
| ... | ... | ... | ... | ... | ... | ... |
|  | | | | | | |
| **Molecular_Function** | | | | | | |
| **GO.ID** | **Term** | **...** | **Pvalue** | **FDR** | **...** | **GENES** |
| GO:0008028 | monocarboxylic acid transmembrane transporter activity | ... | 0.000197877 | 0.177495548 | ... | SLCO1A5//SLC16A8… |
| GO:0000981 | sequence-specific DNA binding RNA polymerase II transcription factor activity | ... | 0.000784799 | 0.346363518 | ... | MSX2//MSX1… |
| GO:0015291 | secondary active transmembrane transporter activity | ... | 0.001158406 | 0.346363518 | ... | SLC4A2//SLC5A5… |
| GO:0042802 | identical protein binding | ... | 0.007837529 | 1 | ... | TRHR//CASQ2… |
| ... | ... | ... | ... | ... | ... | ... |

**GO.ID:** Gene Ontology ID.

**Term:** The name of Gene Ontology terms.

**Pvalue:** P-value is calculated by Fisher’s exact test (Cut-off is P-value <= 0.05, Bioconductor package *topGO*).

**FDR:** The False Discovery Rate of the listed GO terms.

**GENES:** The differentially expressed gene list in the GO term.

**Sample RNA Quality Control**

## 1. Sample RNA Integrity

The integrity of RNA can be assessed by electrophoresis on a denaturing agarose gel. Intact total RNA run on a denaturing gel will have sharp 28S and 18S rRNA bands (eukaryotic samples). The 28S rRNA band should be approximately twice as intense as the 18S rRNA band. This 2:1 intensity ratio indicates that the RNA is intact.

## 2. Sample RNA Purity and Concentration

The NanoDrop ND-1000 was used for accurately measuring RNA concentrations (OD260), protein contamination (ratio of OD260/OD280) and organic compound contamination (ratio of OD260/OD230). We typically ask our customers to provide total RNA with OD260/OD280 ratio higher than 1.8.

**Note:** The sample quality control data can be found in *RNA_QC.pdf*.

**Methods**

Samples

RNA quantity and quality were measured by NanoDrop ND-1000. RNA integrity was assessed by standard denaturing agarose gel electrophoresis.

DNA microarray

The Rat 4x44K Gene Expression Array was manufactured by Agilent. Updated content provided full coverage of rat genes and transcripts.

RNA labeling and array hybridization

Sample labeling and array hybridization were performed according to the Agilent One-Color Microarray-Based Gene Expression Analysis protocol (Agilent Technology). Briefly, total RNA from each sample was linearly amplified and labeled with Cy3-UTP. The labeled cRNAs were purified by RNeasy Mini Kit (Qiagen). The concentration and specific activity of the labeled cRNAs (pmol Cy3/μg cRNA) were measured by NanoDrop ND-1000. 1 μg of each labeled cRNA was fragmented by adding 11 μl 10 × Blocking Agent and 2.2 μl of 25 × Fragmentation Buffer, then heated the mixture at 60 °C for 30 min, finally 55 μl 2 × GE Hybridization buffer was added to dilute the labeled cRNA. 100 μl of hybridization solution was dispensed into the gasket slide and assembled to the gene expression microarray slide. The slides were incubated for 17 hours at 65°C in an Agilent Hybridization Oven. The hybridized arrays were washed, fixed and scanned with using the Agilent DNA Microarray Scanner (part number G2505C).

Data analysis

Agilent Feature Extraction software (version 11.0.1.1) was used to analyze acquired array images. Quantile normalization and subsequent data processing were performed with using the GeneSpring GX v11.5 software package (Agilent Technologies). After quantile normalization of the raw data, genes that at least 12 out of 12 samples have flags in Detected (“All Targets Value”) were chosen for further data analysis. Differentially expressed genes with statistical significance between two groups were identified through Volcano Plot filtering. Hierarchical Clustering was performed using the Agilent GeneSpring GX software (version 12.0). GO analysis and Pathway analysis were performed in the standard enrichment computation method.

**Frequently Asked Questions**

**How are the normalized values calculated from the initially loaded raw data?**

In GeneSpring GX v11.5, a normalized value is a relative number that comes from the ratio of the comparison between the raw data value of the listed probe and that of the controls. Normalized values were calculated based on the type of normalization methods applied to your data. Please read the tech notes on control values to know how the control value was calculated.

For the 1-color data, there are 4 normalization methods (median, scale, quantile, normalized to control genes).

For the 2-color data, each raw intensity value corresponding to the control channel is adjusted by using a locally-weighted regression method called Lowess. Each value in the signal channel was divided by the adjusted control value, which resulted in the final normalized value.
