# Supplementary material for: Gene Expression in the Hippocampus in a Rat Model of Premenstrual Dysphoric Disorder After Treatment With Baixiangdan Capsules
Source: Front Psychol. 2018 Nov 13;9:2065. doi: 10.3389/fpsyg.2018.02065 (PMC6242977; doi:10.3389/fpsyg.2018.02065)
Supplement: Supplementary file 3 [file Data_Sheet_3.ZIP › Data Analysis Folder/GO Analysis Report/fluoxetine vs model (up)/CC_Pvalue_tree.pdf]

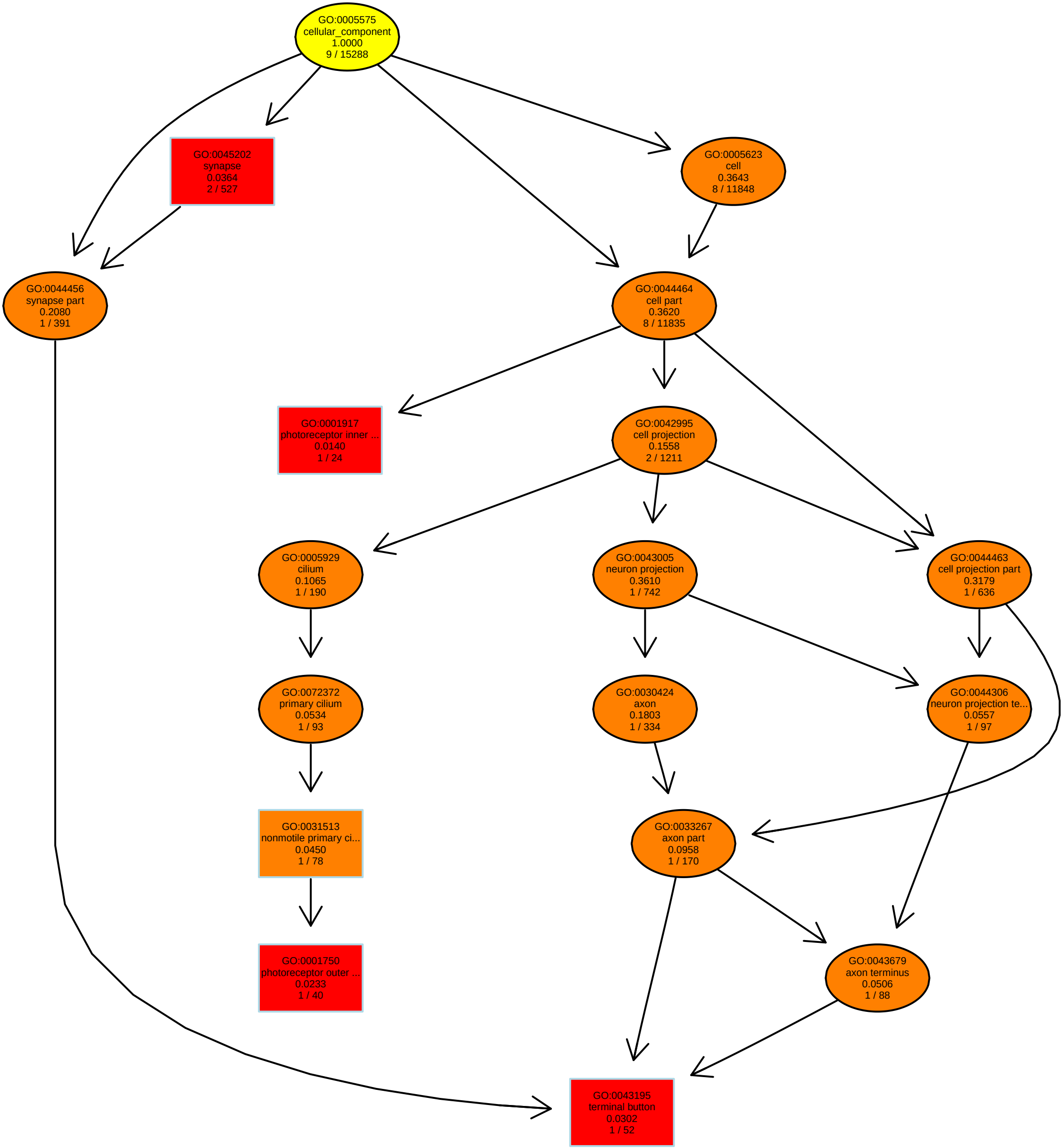
GO:0005575
cellular_component
1.0000
9 / 15288
GO:0045202
synapse
0.0364
2 / 527
GO:0005623
cell
0.3643
8 / 11848
GO:0044456
synapse part
0.2080
1 / 391
GO:0044464
cell part
0.3620
8 / 11835
GO:0001917
photoreceptor inner ...
0.0140
1 / 24
GO:0042995
cell projection
0.1558
2 / 1211
GO:0005929
cilium
0.1065
1 / 190
GO:0043005
neuron projection
0.3610
1 / 742
GO:0044463
cell projection part
0.3179
1 / 636
GO:0072372
primary cilium
0.0534
1 / 93
GO:0030424
axon
0.1803
1 / 334
GO:0044306
neuron projection te...
0.0557
1 / 97
GO:0031513
nonmotile primary ci...
0.0450
1 / 78
GO:0033267
axon part
0.0958
1 / 170
GO:0001750
photoreceptor outer ...
0.0233
1 / 40
GO:0043679
axon terminus
0.0506
1 / 88
GO:0043195
terminal button
0.0302
1 / 52
