# Supplementary material for: Gene Expression in the Hippocampus in a Rat Model of Premenstrual Dysphoric Disorder After Treatment With Baixiangdan Capsules
Source: Front Psychol. 2018 Nov 13;9:2065. doi: 10.3389/fpsyg.2018.02065 (PMC6242977; doi:10.3389/fpsyg.2018.02065)
Supplement: Supplementary file 3 [file Data_Sheet_3.ZIP › Data Analysis Folder/GO Analysis Report/fluoxetine vs model (up)/CC_result(Rat).html]

| GO.ID | Term | Ontology | Count | Pop.Hits | List.Total | Pop.Total | Fold.Enrichment | Pvalue | FDR | Enrichment.Score | GENES |
| --- | --- | --- | --- | --- | --- | --- | --- | --- | --- | --- | --- |
| GO:0001917 | photoreceptor inner segment | Cellular component | 1 | 24 | 9 | 15288 | 70.7777777777778 | 0.0140439840646746 | 1 | 1.8525096719885 | TULP1 |
| GO:0001750 | photoreceptor outer segment | Cellular component | 1 | 40 | 9 | 15288 | 42.4666666666667 | 0.023308969291347 | 1 | 1.63247693029655 | TULP1 |
| GO:0043195 | terminal button | Cellular component | 1 | 52 | 9 | 15288 | 32.6666666666667 | 0.0302068374424255 | 1 | 1.51989474156552 | CALCA |
| GO:0045202 | synapse | Cellular component | 2 | 527 | 9 | 15288 | 6.44655281467426 | 0.0363604767861719 | 1 | 1.43937043061382 | CALCA//TULP1 |
| GO:0031513 | nonmotile primary cilium | Cellular component | 1 | 78 | 9 | 15288 | 21.7777777777778 | 0.0450038656652123 | 1 | 1.34675018033653 | TULP1 |
