# Supplementary material for: Gene Expression in the Hippocampus in a Rat Model of Premenstrual Dysphoric Disorder After Treatment With Baixiangdan Capsules
Source: Front Psychol. 2018 Nov 13;9:2065. doi: 10.3389/fpsyg.2018.02065 (PMC6242977; doi:10.3389/fpsyg.2018.02065)
Supplement: Supplementary file 3 [file Data_Sheet_3.ZIP › Data Analysis Folder/GO Analysis Report/fluoxetine vs model (up)/MF_Pvalue_tree.pdf]

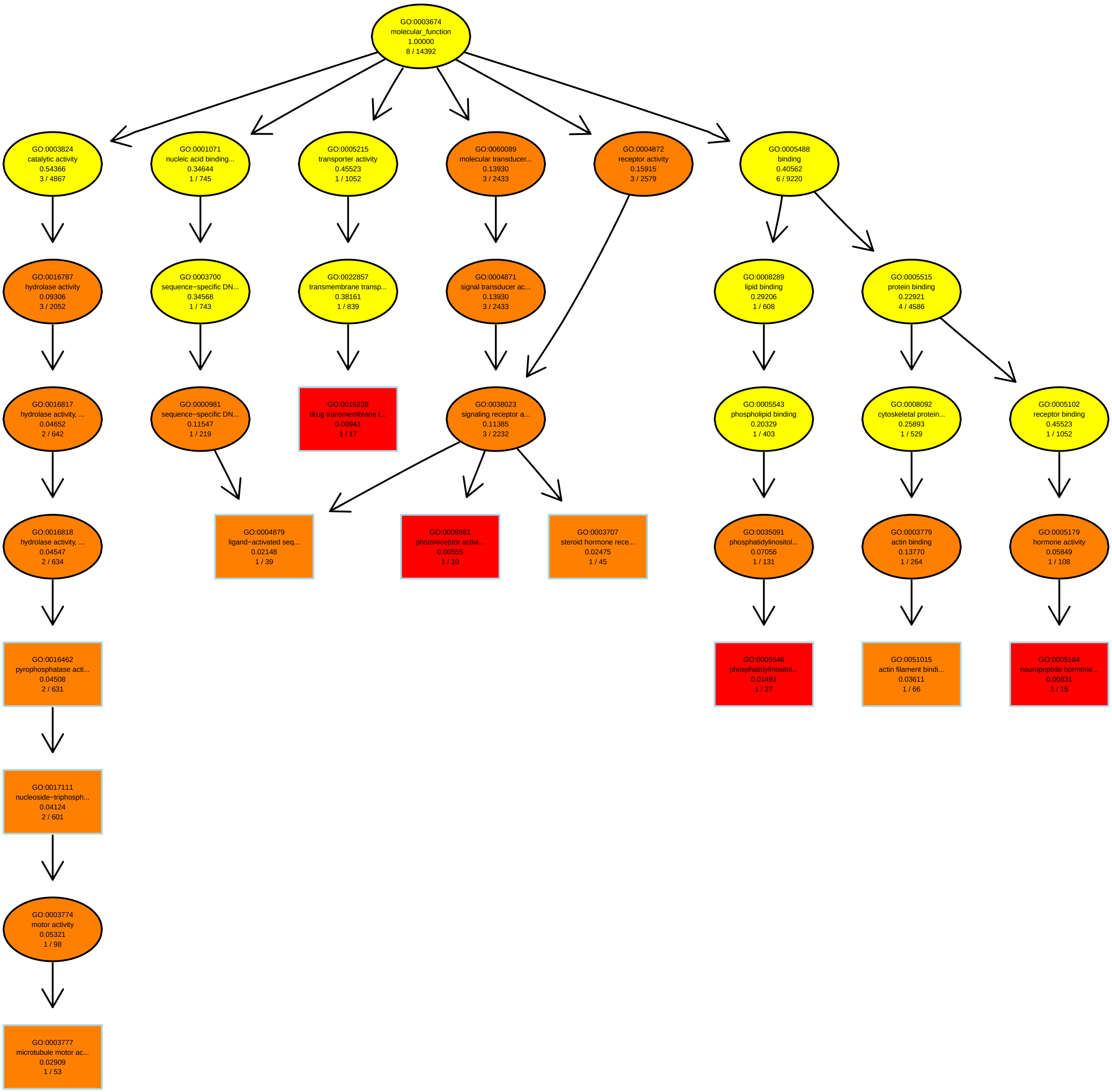
GO:0003674
molecular_function
1.00000
8 / 14392
GO:0003824
catalytic activity
0.54366
3 / 4867
GO:0001071
nucleic acid binding...
0.34644
1 / 745
GO:0005215
transporter activity
0.45523
1 / 1052
GO:0060089
molecular transducer...
0.13930
3 / 2433
GO:0004872
receptor activity
0.15915
3 / 2579
GO:0005488
binding
0.40562
6 / 9220
GO:0016787
hydrolase activity
0.09306
3 / 2052
GO:0003700
sequence−specific DN...
0.34568
1 / 743
GO:0022857
transmembrane transp...
0.38161
1 / 839
GO:0004871
signal transducer ac...
0.13930
3 / 2433
GO:0008289
lipid binding
0.29206
1 / 608
GO:0005515
protein binding
0.22921
4 / 4586
GO:0016817
hydrolase activity, ...
0.04652
2 / 642
GO:0000981
sequence−specific DN...
0.11547
1 / 219
GO:0015238
drug transmembrane t...
0.00941
1 / 17
GO:0038023
signaling receptor a...
0.11385
3 / 2232
GO:0005543
phospholipid binding
0.20329
1 / 403
GO:0008092
cytoskeletal protein...
0.25893
1 / 529
GO:0005102
receptor binding
0.45523
1 / 1052
GO:0016818
hydrolase activity, ...
0.04547
2 / 634
GO:0004879
ligand−activated seq...
0.02148
1 / 39
GO:0009881
photoreceptor activi...
0.00555
1 / 10
GO:0003707
steroid hormone rece...
0.02475
1 / 45
GO:0035091
phosphatidylinositol...
0.07056
1 / 131
GO:0003779
actin binding
0.13770
1 / 264
GO:0005179
hormone activity
0.05849
1 / 108
GO:0016462
pyrophosphatase acti...
0.04508
2 / 631
GO:0005546
phosphatidylinositol...
0.01491
1 / 27
GO:0051015
actin filament bindi...
0.03611
1 / 66
GO:0005184
neuropeptide hormone...
0.00831
1 / 15
GO:0017111
nucleoside−triphosph...
0.04124
2 / 601
GO:0003774
motor activity
0.05321
1 / 98
GO:0003777
microtubule motor ac...
0.02909
1 / 53
