# Supplementary material for: Gene Expression in the Hippocampus in a Rat Model of Premenstrual Dysphoric Disorder After Treatment With Baixiangdan Capsules
Source: Front Psychol. 2018 Nov 13;9:2065. doi: 10.3389/fpsyg.2018.02065 (PMC6242977; doi:10.3389/fpsyg.2018.02065)
Supplement: Supplementary file 3 [file Data_Sheet_3.ZIP › Data Analysis Folder/GO Analysis Report/fluoxetine vs model (up)/MF_result(Rat).html]

| GO.ID | Term | Ontology | Count | Pop.Hits | List.Total | Pop.Total | Fold.Enrichment | Pvalue | FDR | Enrichment.Score | GENES |
| --- | --- | --- | --- | --- | --- | --- | --- | --- | --- | --- | --- |
| GO:0009881 | photoreceptor activity | Molecular function | 1 | 10 | 8 | 14392 | 179.9 | 0.00554649007361685 | 1 | 2.25598175991784 | TULP1 |
| GO:0005184 | neuropeptide hormone activity | Molecular function | 1 | 15 | 8 | 14392 | 119.933333333333 | 0.00830962679070879 | 1 | 2.08041848119489 | CALCA |
| GO:0015238 | drug transmembrane transporter activity | Molecular function | 1 | 17 | 8 | 14392 | 105.823529411765 | 0.00941299904912996 | 1 | 2.02627198518296 | ABCG2 |
| GO:0005546 | phosphatidylinositol-4,5-bisphosphate binding | Molecular function | 1 | 27 | 8 | 14392 | 66.6296296296296 | 0.0149137633617655 | 1 | 1.82641275218641 | TULP1 |
| GO:0004879 | ligand-activated sequence-specific DNA binding RNA polymerase II transcription factor activity | Molecular function | 1 | 39 | 8 | 14392 | 46.1282051282051 | 0.0214793852838665 | 1 | 1.66797815182001 | RXRG |
| GO:0003707 | steroid hormone receptor activity | Molecular function | 1 | 45 | 8 | 14392 | 39.9777777777778 | 0.0247478134975222 | 1 | 1.60646316553478 | RXRG |
| GO:0003777 | microtubule motor activity | Molecular function | 1 | 53 | 8 | 14392 | 33.9433962264151 | 0.0290908563087143 | 1 | 1.53624349481771 | KIF20A |
| GO:0051015 | actin filament binding | Molecular function | 1 | 66 | 8 | 14392 | 27.2575757575758 | 0.0361122121779354 | 1 | 1.44234590655398 | TULP1 |
| GO:0017111 | nucleoside-triphosphatase activity | Molecular function | 2 | 601 | 8 | 14392 | 5.98668885191348 | 0.0412378582587156 | 1 | 1.38470389844612 | KIF20A//ABCG2 |
| GO:0016462 | pyrophosphatase activity | Molecular function | 2 | 631 | 8 | 14392 | 5.70206022187005 | 0.0450799171992356 | 1 | 1.34601689031777 | KIF20A//ABCG2 |
| GO:0016818 | hydrolase activity, acting on acid anhydrides, in phosphorus-containing anhydrides | Molecular function | 2 | 634 | 8 | 14392 | 5.67507886435331 | 0.0454716137552903 | 1 | 1.34225963271855 | KIF20A//ABCG2 |
| GO:0016817 | hydrolase activity, acting on acid anhydrides | Molecular function | 2 | 642 | 8 | 14392 | 5.60436137071651 | 0.0465227000438603 | 1 | 1.33233508800805 | KIF20A//ABCG2 |
| GO:0016820 | hydrolase activity, acting on acid anhydrides, catalyzing transmembrane movement of substances | Molecular function | 1 | 87 | 8 | 14392 | 20.6781609195402 | 0.0473605681684445 | 1 | 1.32458309617238 | ABCG2 |
| GO:0042626 | ATPase activity, coupled to transmembrane movement of substances | Molecular function | 1 | 87 | 8 | 14392 | 20.6781609195402 | 0.0473605681684445 | 1 | 1.32458309617238 | ABCG2 |
| GO:0043492 | ATPase activity, coupled to movement of substances | Molecular function | 1 | 89 | 8 | 14392 | 20.2134831460674 | 0.0484258251650604 | 1 | 1.31492297026648 | ABCG2 |
