# Supplementary material for: Gene Expression in the Hippocampus in a Rat Model of Premenstrual Dysphoric Disorder After Treatment With Baixiangdan Capsules
Source: Front Psychol. 2018 Nov 13;9:2065. doi: 10.3389/fpsyg.2018.02065 (PMC6242977; doi:10.3389/fpsyg.2018.02065)
Supplement: Supplementary file 3 [file Data_Sheet_3.ZIP › Data Analysis Folder/GO Analysis Report/model vs blank (down)/BP_Pvalue_tree.pdf]

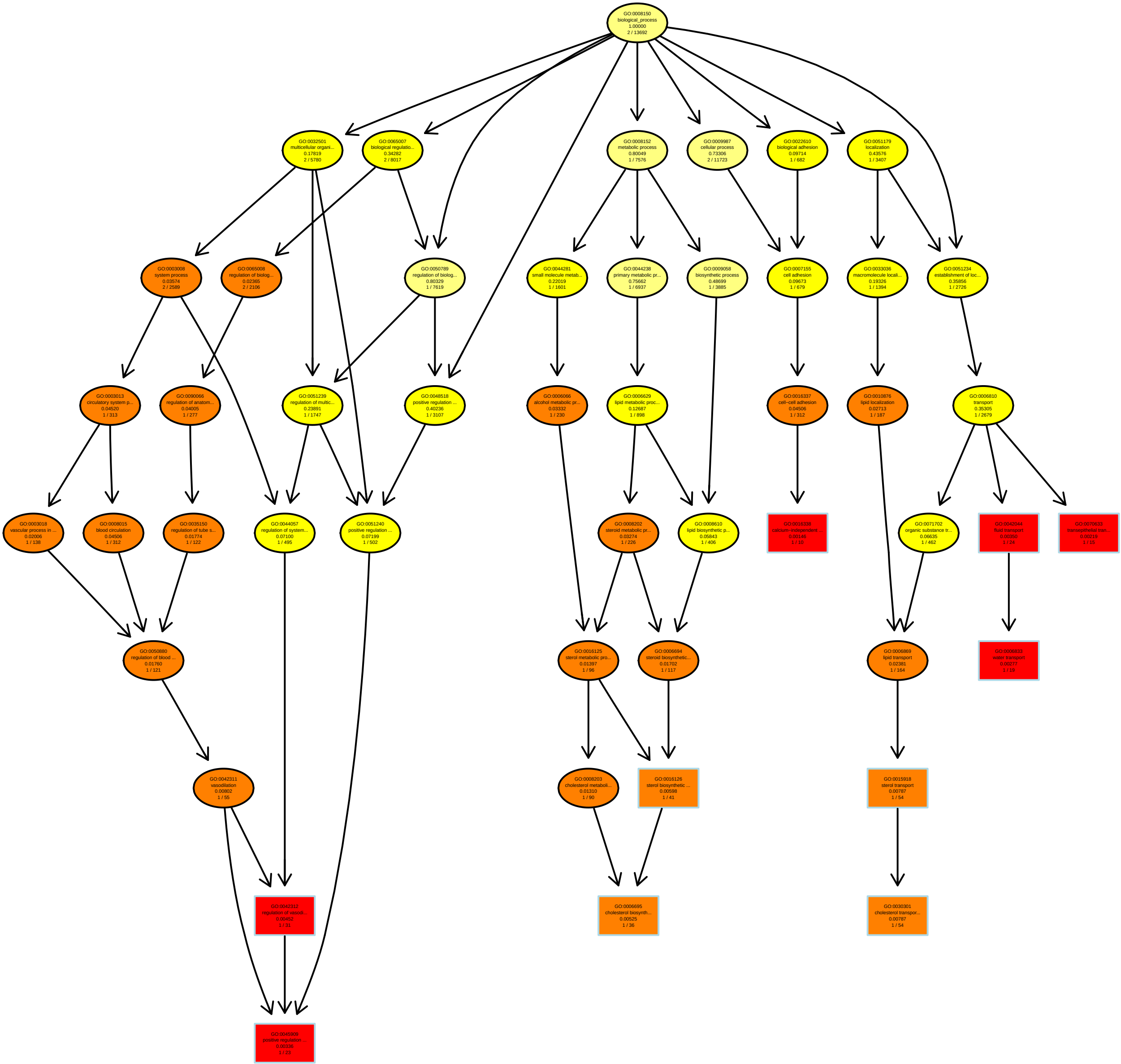

GO:0008150 biological_process 1.00000 2 / 13692
GO:0032501 multicellular organi... 0.17819 2 / 5780
GO:0065007 biological regulatio... 0.34282 2 / 8017
GO:0008152 metabolic process 0.80049 1 / 7576
GO:0009987 cellular process 0.73306 2 / 11723
GO:0022610 biological adhesion 0.09714 1 / 682
GO:0051179 localization 0.43576 1 / 3407
GO:0003008 system process 0.03574 2 / 2589
GO:0065008 regulation of biolog... 0.02365 2 / 2106
GO:0050789 regulation of biolog... 0.80329 1 / 7619
GO:0044281 small molecule metab... 0.22019 1 / 1601
GO:0044238 primary metabolic pr... 0.75662 1 / 6937
GO:0009058 biosynthetic process 0.48699 1 / 3885
GO:0007155 cell adhesion 0.09673 1 / 679
GO:0033036 macromolecule locali... 0.19326 1 / 1394
GO:0051234 establishment of loc... 0.35856 1 / 2726
GO:0003013 circulatory system p... 0.04520 1 / 313
GO:0090066 regulation of anatom... 0.04005 1 / 277
GO:0051239 regulation of multic... 0.23891 1 / 1747
GO:0048518 positive regulation ... 0.40236 1 / 3107
GO:0006066 alcohol metabolic pr... 0.03332 1 / 230
GO:0006629 lipid metabolic proc... 0.12687 1 / 898
GO:0016337 cell−cell adhesion 0.04506 1 / 312
GO:0010876 lipid localization 0.02713 1 / 187
GO:0006810 transport 0.35305 1 / 2679
GO:0003018 vascular process in ... 0.02006 1 / 138
GO:0008015 blood circulation 0.04506 1 / 312
GO:0035150 regulation of tube s... 0.01774 1 / 122
GO:0044057 regulation of system... 0.07100 1 / 495
GO:0051240 positive regulation ... 0.07199 1 / 502
GO:0008202 steroid metabolic pr... 0.03274 1 / 226
GO:0008610 lipid biosynthetic p... 0.05843 1 / 406
GO:0016338 calcium−independent ... 0.00146 1 / 10
GO:0071702 organic substance tr... 0.06635 1 / 462
GO:0042044 fluid transport 0.00350 1 / 24
GO:0070633 transepithelial tran... 0.00219 1 / 15
GO:0050880 regulation of blood ... 0.01760 1 / 121
GO:0016125 sterol metabolic pro... 0.01397 1 / 96
GO:0006694 steroid biosynthetic... 0.01702 1 / 117
GO:0006869 lipid transport 0.02381 1 / 164
GO:0006833 water transport 0.00277 1 / 19
GO:0042311 vasodilation 0.00802 1 / 55
GO:0008203 cholesterol metaboli... 0.01310 1 / 90
GO:0016126 sterol biosynthetic ... 0.00598 1 / 41
GO:0015918 sterol transport 0.00787 1 / 54
GO:0042312 regulation of vasodi... 0.00452 1 / 31
GO:0006695 cholesterol biosynth... 0.00525 1 / 36
GO:0030301 cholesterol transpor... 0.00787 1 / 54
GO:0045909 positive regulation ... 0.00336 1 / 23
