# Supplementary material for: Gene Expression in the Hippocampus in a Rat Model of Premenstrual Dysphoric Disorder After Treatment With Baixiangdan Capsules
Source: Front Psychol. 2018 Nov 13;9:2065. doi: 10.3389/fpsyg.2018.02065 (PMC6242977; doi:10.3389/fpsyg.2018.02065)
Supplement: Supplementary file 3 [file Data_Sheet_3.ZIP › Data Analysis Folder/GO Analysis Report/model vs blank (down)/BP_result(Rat).html]

| GO.ID | Term | Ontology | Count | Pop.Hits | List.Total | Pop.Total | Fold.Enrichment | Pvalue | FDR | Enrichment.Score | GENES |
| --- | --- | --- | --- | --- | --- | --- | --- | --- | --- | --- | --- |
| GO:0016338 | calcium-independent cell-cell adhesion | Biological process | 1 | 10 | 2 | 13692 | 684.6 | 0.00146022687251457 | 1 | 2.8355796635124 | CLDN11 |
| GO:0070633 | transepithelial transport | Biological process | 1 | 15 | 2 | 13692 | 456.4 | 0.00218994021738472 | 1 | 2.65956774069153 | CFTR |
| GO:0006833 | water transport | Biological process | 1 | 19 | 2 | 13692 | 360.315789473684 | 0.00277351884941501 | 1 | 2.55696887821885 | CFTR |
| GO:0045909 | positive regulation of vasodilation | Biological process | 1 | 23 | 2 | 13692 | 297.652173913043 | 0.00335692677578647 | 1 | 2.47405813185854 | CFTR |
| GO:0042044 | fluid transport | Biological process | 1 | 24 | 2 | 13692 | 285.25 | 0.00350275208462014 | 1 | 2.45559059979096 | CFTR |
| GO:0042312 | regulation of vasodilation | Biological process | 1 | 31 | 2 | 13692 | 220.838709677419 | 0.00452323051155379 | 1 | 2.34455127930142 | CFTR |
| GO:0006695 | cholesterol biosynthetic process | Biological process | 1 | 36 | 2 | 13692 | 190.166666666667 | 0.00525182360053922 | 1 | 2.27968986952068 | CFTR |
| GO:0016126 | sterol biosynthetic process | Biological process | 1 | 41 | 2 | 13692 | 166.975609756098 | 0.00598014996193386 | 1 | 2.22328792523838 | CFTR |
| GO:0015918 | sterol transport | Biological process | 1 | 54 | 2 | 13692 | 126.777777777778 | 0.00787255021643052 | 1 | 2.10388456045971 | CFTR |
| GO:0030301 | cholesterol transport | Biological process | 1 | 54 | 2 | 13692 | 126.777777777778 | 0.00787255021643052 | 1 | 2.10388456045971 | CFTR |
| GO:0042311 | vasodilation | Biological process | 1 | 55 | 2 | 13692 | 124.472727272727 | 0.00801804478305035 | 1 | 2.09593152242108 | CFTR |
| GO:0006821 | chloride transport | Biological process | 1 | 56 | 2 | 13692 | 122.25 | 0.00816352868056702 | 1 | 2.08812207712326 | CFTR |
| GO:0015711 | organic anion transport | Biological process | 1 | 62 | 2 | 13692 | 110.41935483871 | 0.00903620801449023 | 1 | 2.04401378013044 | CFTR |
| GO:0015698 | inorganic anion transport | Biological process | 1 | 78 | 2 | 13692 | 87.7692307692308 | 0.0113614751427059 | 1 | 1.94456527736219 | CFTR |
| GO:0008203 | cholesterol metabolic process | Biological process | 1 | 90 | 2 | 13692 | 76.0666666666667 | 0.0131036330794533 | 1 | 1.88260827628266 | CFTR |
| GO:0007589 | body fluid secretion | Biological process | 1 | 91 | 2 | 13692 | 75.2307692307692 | 0.0132487435583419 | 1 | 1.87782530599526 | CFTR |
| GO:0007272 | ensheathment of neurons | Biological process | 1 | 92 | 2 | 13692 | 74.4130434782609 | 0.0133938433681268 | 1 | 1.87309478414823 | CLDN11 |
| GO:0008366 | axon ensheathment | Biological process | 1 | 92 | 2 | 13692 | 74.4130434782609 | 0.0133938433681268 | 1 | 1.87309478414823 | CLDN11 |
| GO:0016125 | sterol metabolic process | Biological process | 1 | 96 | 2 | 13692 | 71.3125 | 0.0139741359162295 | 1 | 1.85467503699541 | CFTR |
| GO:0019228 | regulation of action potential in neuron | Biological process | 1 | 111 | 2 | 13692 | 61.6756756756757 | 0.016148712624343 | 1 | 1.79186209391672 | CLDN11 |
| GO:0006694 | steroid biosynthetic process | Biological process | 1 | 117 | 2 | 13692 | 58.5128205128205 | 0.0170178711540576 | 1 | 1.7690947688032 | CFTR |
| GO:0050880 | regulation of blood vessel size | Biological process | 1 | 121 | 2 | 13692 | 56.5785123966942 | 0.0175970967917944 | 1 | 1.75455897714645 | CFTR |
| GO:0035150 | regulation of tube size | Biological process | 1 | 122 | 2 | 13692 | 56.1147540983607 | 0.017741876528469 | 1 | 1.7510004474773 | CFTR |
| GO:0003018 | vascular process in circulatory system | Biological process | 1 | 138 | 2 | 13692 | 49.6086956521739 | 0.020056901317171 | 1 | 1.69773616228286 | CFTR |
| GO:0006820 | anion transport | Biological process | 1 | 138 | 2 | 13692 | 49.6086956521739 | 0.020056901317171 | 1 | 1.69773616228286 | CFTR |
| GO:0001508 | regulation of action potential | Biological process | 1 | 145 | 2 | 13692 | 47.2137931034483 | 0.0210688657993836 | 1 | 1.67635884314497 | CLDN11 |
| GO:0065008 | regulation of biological quality | Biological process | 2 | 2106 | 2 | 13692 | 6.5014245014245 | 0.0236487617555651 | 1 | 1.62619159390307 | CFTR//CLDN11 |
| GO:0006869 | lipid transport | Biological process | 1 | 164 | 2 | 13692 | 41.7439024390244 | 0.0238129912682133 | 1 | 1.62318604727959 | CFTR |
| GO:0030324 | lung development | Biological process | 1 | 173 | 2 | 13692 | 39.5722543352601 | 0.0251114958674399 | 1 | 1.60012741601567 | CFTR |
| GO:0030323 | respiratory tube development | Biological process | 1 | 175 | 2 | 13692 | 39.12 | 0.0253999350849057 | 1 | 1.59516739331326 | CFTR |
| GO:0010876 | lipid localization | Biological process | 1 | 187 | 2 | 13692 | 36.6096256684492 | 0.0271296741849922 | 1 | 1.56655542188565 | CFTR |
| GO:0060541 | respiratory system development | Biological process | 1 | 195 | 2 | 13692 | 35.1076923076923 | 0.028281980056757 | 1 | 1.54849018831147 | CFTR |
| GO:0008202 | steroid metabolic process | Biological process | 1 | 226 | 2 | 13692 | 30.2920353982301 | 0.0327407158366818 | 1 | 1.48491182948968 | CFTR |
| GO:0006066 | alcohol metabolic process | Biological process | 1 | 230 | 2 | 13692 | 29.7652173913044 | 0.0333152897452224 | 1 | 1.47735640529232 | CFTR |
| GO:0043627 | response to estrogen stimulus | Biological process | 1 | 237 | 2 | 13692 | 28.8860759493671 | 0.0343203833246775 | 1 | 1.46444787015717 | CFTR |
| GO:0050878 | regulation of body fluid levels | Biological process | 1 | 241 | 2 | 13692 | 28.4066390041494 | 0.0348944877926568 | 1 | 1.45724317218992 | CFTR |
| GO:0003008 | system process | Biological process | 2 | 2589 | 2 | 13692 | 5.28852838933951 | 0.0357432683343799 | 1 | 1.44680573849269 | CLDN11//CFTR |
| GO:0042391 | regulation of membrane potential | Biological process | 1 | 262 | 2 | 13692 | 26.1297709923664 | 0.0379057356098383 | 1 | 1.42129507089635 | CLDN11 |
| GO:0090066 | regulation of anatomical structure size | Biological process | 1 | 277 | 2 | 13692 | 24.7148014440433 | 0.040053746249837 | 1 | 1.39735685786829 | CFTR |
| GO:0008015 | blood circulation | Biological process | 1 | 312 | 2 | 13692 | 21.9423076923077 | 0.0450564356107963 | 1 | 1.34624316804475 | CFTR |
| GO:0016337 | cell-cell adhesion | Biological process | 1 | 312 | 2 | 13692 | 21.9423076923077 | 0.0450564356107963 | 1 | 1.34624316804475 | CLDN11 |
| GO:0003013 | circulatory system process | Biological process | 1 | 313 | 2 | 13692 | 21.8722044728434 | 0.0451991775486725 | 1 | 1.34486946760727 | CFTR |
| GO:0032870 | cellular response to hormone stimulus | Biological process | 1 | 326 | 2 | 13692 | 21 | 0.047053851852625 | 1 | 1.32740481921082 | CFTR |
| GO:0043434 | response to peptide hormone stimulus | Biological process | 1 | 334 | 2 | 13692 | 20.497005988024 | 0.048194293681119 | 1 | 1.31700438021571 | CFTR |
| GO:0007283 | spermatogenesis | Biological process | 1 | 339 | 2 | 13692 | 20.1946902654867 | 0.0489067230780588 | 1 | 1.31063143545706 | CLDN11 |
| GO:0048232 | male gamete generation | Biological process | 1 | 339 | 2 | 13692 | 20.1946902654867 | 0.0489067230780588 | 1 | 1.31063143545706 | CLDN11 |
