# Supplementary material for: Gene Expression in the Hippocampus in a Rat Model of Premenstrual Dysphoric Disorder After Treatment With Baixiangdan Capsules
Source: Front Psychol. 2018 Nov 13;9:2065. doi: 10.3389/fpsyg.2018.02065 (PMC6242977; doi:10.3389/fpsyg.2018.02065)
Supplement: Supplementary file 3 [file Data_Sheet_3.ZIP › Data Analysis Folder/GO Analysis Report/model vs blank (down)/CC_Pvalue_tree.pdf]

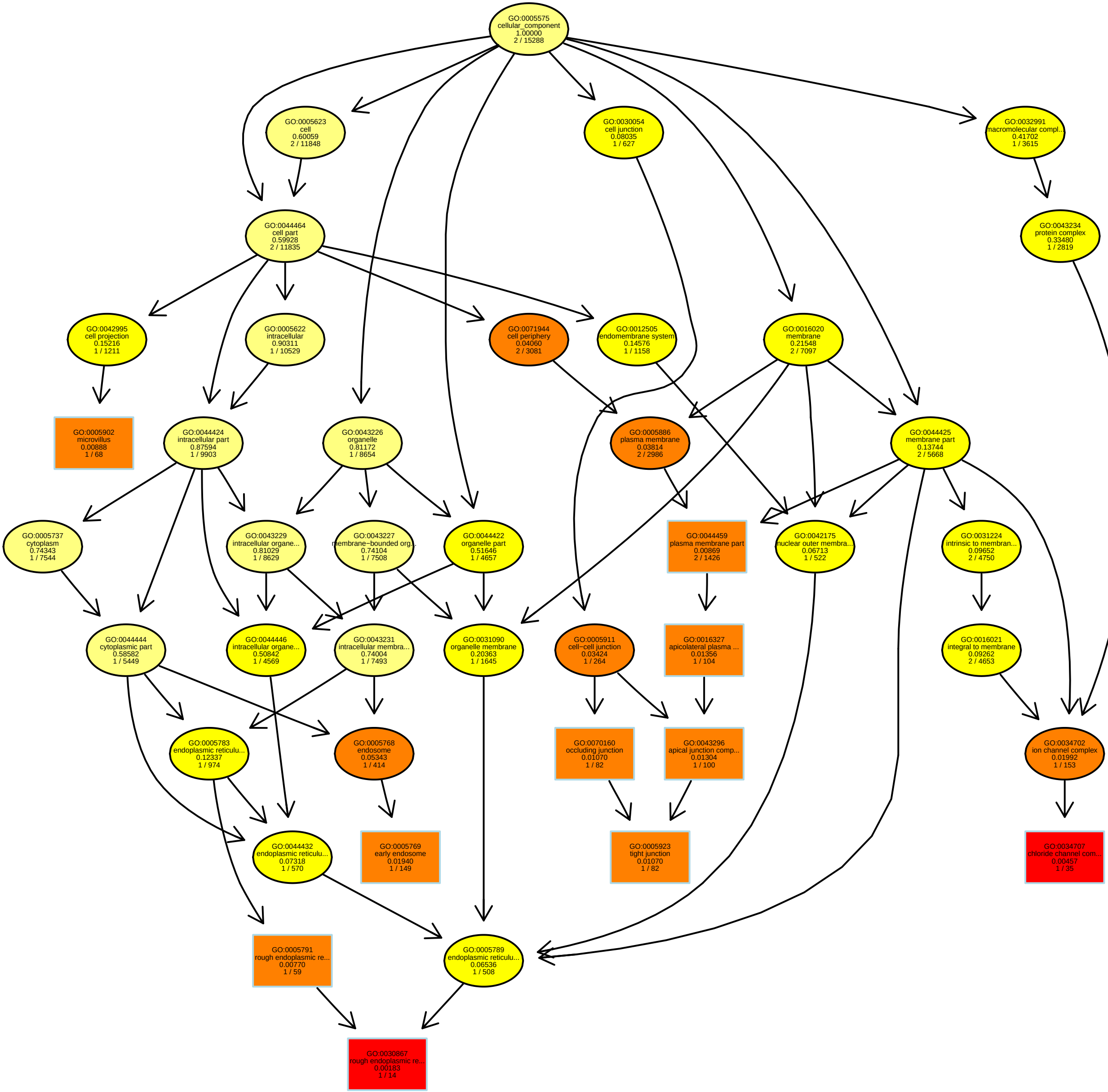
GO:0005575 cellular_component 1.00000 2 / 15288
GO:0005623 cell 0.60059 2 / 11848
GO:0030054 cell junction 0.08035 1 / 627
GO:0032991 macromolecular compl... 0.41702 1 / 3615
GO:0044464 cell part 0.59928 2 / 11835
GO:0043234 protein complex 0.33480 1 / 2819
GO:0042995 cell projection 0.15216 1 / 1211
GO:0005622 intracellular 0.90311 1 / 10529
GO:0071944 cell periphery 0.04060 2 / 3081
GO:0012505 endomembrane system 0.14576 1 / 1158
GO:0016020 membrane 0.21548 2 / 7097
GO:0005902 microvillus 0.00888 1 / 68
GO:0044424 intracellular part 0.87594 1 / 9903
GO:0043226 organelle 0.81172 1 / 8654
GO:0005886 plasma membrane 0.03814 2 / 2986
GO:0044425 membrane part 0.13744 2 / 5668
GO:0005737 cytoplasm 0.74343 1 / 7544
GO:0043229 intracellular organe... 0.81029 1 / 8629
GO:0043227 membrane−bounded org... 0.74104 1 / 7508
GO:0044422 organelle part 0.51646 1 / 4657
GO:0044459 plasma membrane part 0.00869 2 / 1426
GO:0042175 nuclear outer membra... 0.06713 1 / 522
GO:0031224 intrinsic to membran... 0.09652 2 / 4750
GO:0044444 cytoplasmic part 0.58582 1 / 5449
GO:0044446 intracellular organe... 0.50842 1 / 4569
GO:0043231 intracellular membra... 0.74004 1 / 7493
GO:0031090 organelle membrane 0.20363 1 / 1645
GO:0005911 cell−cell junction 0.03424 1 / 264
GO:0016327 apicolateral plasma ... 0.01356 1 / 104
GO:0016021 integral to membrane 0.09262 2 / 4653
GO:0005783 endoplasmic reticulu... 0.12337 1 / 974
GO:0005768 endosome 0.05343 1 / 414
GO:0070160 occluding junction 0.01070 1 / 82
GO:0043296 apical junction comp... 0.01304 1 / 100
GO:0034702 ion channel complex 0.01992 1 / 153
GO:0044432 endoplasmic reticulu... 0.07318 1 / 570
GO:0005769 early endosome 0.01940 1 / 149
GO:0005923 tight junction 0.01070 1 / 82
GO:0034707 chloride channel com... 0.00457 1 / 35
GO:0005791 rough endoplasmic re... 0.00770 1 / 59
GO:0005789 endoplasmic reticulu... 0.06536 1 / 508
GO:0030867 rough endoplasmic re... 0.00183 1 / 14
