# Supplementary material for: Gene Expression in the Hippocampus in a Rat Model of Premenstrual Dysphoric Disorder After Treatment With Baixiangdan Capsules
Source: Front Psychol. 2018 Nov 13;9:2065. doi: 10.3389/fpsyg.2018.02065 (PMC6242977; doi:10.3389/fpsyg.2018.02065)
Supplement: Supplementary file 3 [file Data_Sheet_3.ZIP › Data Analysis Folder/GO Analysis Report/model vs blank (down)/CC_result(Rat).html]

| GO.ID | Term | Ontology | Count | Pop.Hits | List.Total | Pop.Total | Fold.Enrichment | Pvalue | FDR | Enrichment.Score | GENES |
| --- | --- | --- | --- | --- | --- | --- | --- | --- | --- | --- | --- |
| GO:0030867 | rough endoplasmic reticulum membrane | Cellular component | 1 | 14 | 2 | 15288 | 546 | 0.00183072308080456 | 0.809056559105615 | 2.73737734306793 | CFTR |
| GO:0034707 | chloride channel complex | Cellular component | 1 | 35 | 2 | 15288 | 218.4 | 0.00457366274727455 | 0.809056559105615 | 2.33973586255341 | CFTR |
| GO:0005791 | rough endoplasmic reticulum | Cellular component | 1 | 59 | 2 | 15288 | 129.559322033898 | 0.00770382977954343 | 0.809056559105615 | 2.11329332175861 | CFTR |
| GO:0044459 | plasma membrane part | Cellular component | 2 | 1426 | 2 | 15288 | 10.7208976157083 | 0.00869483710880229 | 0.809056559105615 | 2.06073854976374 | CLDN11//CFTR |
| GO:0005902 | microvillus | Cellular component | 1 | 68 | 2 | 15288 | 112.411764705882 | 0.00887637159819865 | 0.809056559105615 | 2.05176452486397 | CFTR |
| GO:0005923 | tight junction | Cellular component | 1 | 82 | 2 | 15288 | 93.219512195122 | 0.0106989477486351 | 0.809056559105615 | 1.97065893347677 | CLDN11 |
| GO:0070160 | occluding junction | Cellular component | 1 | 82 | 2 | 15288 | 93.219512195122 | 0.0106989477486351 | 0.809056559105615 | 1.97065893347677 | CLDN11 |
| GO:0043296 | apical junction complex | Cellular component | 1 | 100 | 2 | 15288 | 76.44 | 0.0130397953244629 | 0.809056559105615 | 1.8847292253337 | CLDN11 |
| GO:0016327 | apicolateral plasma membrane | Cellular component | 1 | 104 | 2 | 15288 | 73.5 | 0.0135596071358483 | 0.809056559105615 | 1.86775289315368 | CLDN11 |
| GO:0005769 | early endosome | Cellular component | 1 | 149 | 2 | 15288 | 51.3020134228188 | 0.0193980551497209 | 0.972272167552485 | 1.71224181028032 | CFTR |
| GO:0034702 | ion channel complex | Cellular component | 1 | 153 | 2 | 15288 | 49.9607843137255 | 0.0199161896519131 | 0.972272167552485 | 1.70079374680775 | CFTR |
| GO:0030659 | cytoplasmic vesicle membrane | Cellular component | 1 | 214 | 2 | 15288 | 35.7196261682243 | 0.0278007751701553 | 1 | 1.55594309446223 | CFTR |
| GO:0044433 | cytoplasmic vesicle part | Cellular component | 1 | 222 | 2 | 15288 | 34.4324324324324 | 0.0288324572473567 | 1 | 1.54011834326938 | CFTR |
| GO:0016324 | apical plasma membrane | Cellular component | 1 | 230 | 2 | 15288 | 33.2347826086957 | 0.0298635916317604 | 1 | 1.52485796178004 | CFTR |
| GO:0012506 | vesicle membrane | Cellular component | 1 | 234 | 2 | 15288 | 32.6666666666667 | 0.0303789534391633 | 1 | 1.51742719174464 | CFTR |
| GO:0005911 | cell-cell junction | Cellular component | 1 | 264 | 2 | 15288 | 28.9545454545455 | 0.0342398025677 | 1 | 1.46546874820066 | CLDN11 |
| GO:0016323 | basolateral plasma membrane | Cellular component | 1 | 273 | 2 | 15288 | 28 | 0.0353965554299174 | 1 | 1.45103899872465 | CFTR |
| GO:0005886 | plasma membrane | Cellular component | 2 | 2986 | 2 | 15288 | 5.1198928332217 | 0.0381382884606912 | 1 | 1.41863880076972 | CLDN11//CFTR |
| GO:0045177 | apical part of cell | Cellular component | 1 | 306 | 2 | 15288 | 24.9803921568627 | 0.039632052105302 | 1 | 1.40195343977948 | CFTR |
| GO:0071944 | cell periphery | Cellular component | 2 | 3081 | 2 | 15288 | 4.9620253164557 | 0.0406040613406349 | 1 | 1.39143052480673 | CLDN11//CFTR |
