# Supplementary material for: Gene Expression in the Hippocampus in a Rat Model of Premenstrual Dysphoric Disorder After Treatment With Baixiangdan Capsules
Source: Front Psychol. 2018 Nov 13;9:2065. doi: 10.3389/fpsyg.2018.02065 (PMC6242977; doi:10.3389/fpsyg.2018.02065)
Supplement: Supplementary file 3 [file Data_Sheet_3.ZIP › Data Analysis Folder/GO Analysis Report/model vs blank (down)/MF_Pvalue_tree.pdf]

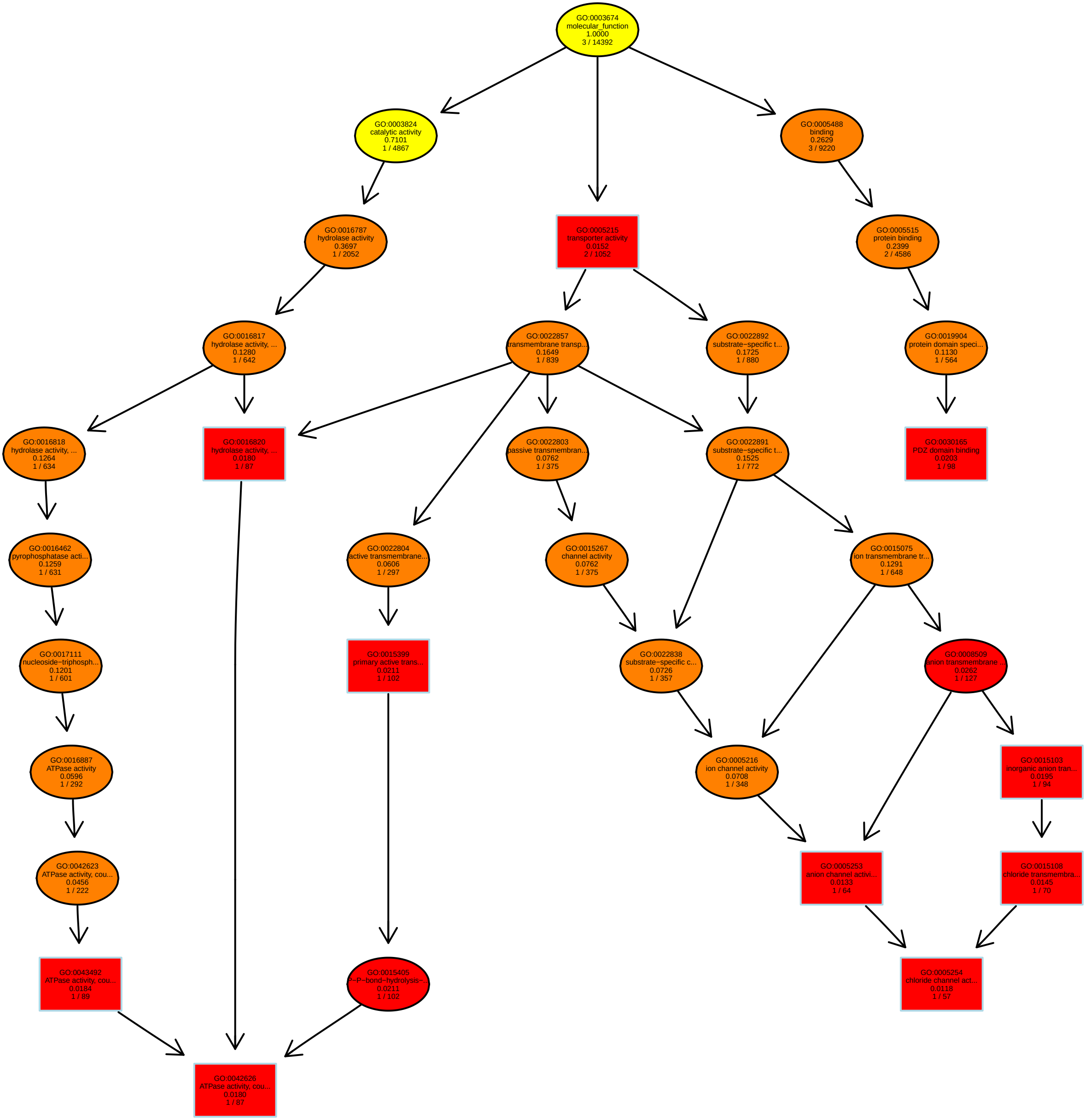

GO:0003674 molecular_function 1.0000 3 / 14392
GO:0003824 catalytic activity 0.7101 1 / 4867
GO:0005215 transporter activity 0.0152 2 / 1052
GO:0005488 binding 0.2629 3 / 9220
GO:0016787 hydrolase activity 0.3697 1 / 2052
GO:0005515 protein binding 0.2399 2 / 4586
GO:0016817 hydrolase activity, ... 0.1280 1 / 642
GO:0022857 transmembrane transp... 0.1649 1 / 839
GO:0022892 substrate−specific t... 0.1725 1 / 880
GO:0019904 protein domain speci... 0.1130 1 / 564
GO:0016818 hydrolase activity, ... 0.1264 1 / 634
GO:0016820 hydrolase activity, ... 0.0180 1 / 87
GO:0022803 passive transmembran... 0.0762 1 / 375
GO:0022891 substrate−specific t... 0.1525 1 / 772
GO:0030165 PDZ domain binding 0.0203 1 / 98
GO:0016462 pyrophosphatase acti... 0.1259 1 / 631
GO:0022804 active transmembrane... 0.0606 1 / 297
GO:0015267 channel activity 0.0762 1 / 375
GO:0015075 ion transmembrane tr... 0.1291 1 / 648
GO:0017111 nucleoside−triphosph... 0.1201 1 / 601
GO:0015399 primary active trans... 0.0211 1 / 102
GO:0022838 substrate−specific c... 0.0726 1 / 357
GO:0008509 anion transmembrane ... 0.0262 1 / 127
GO:0016887 ATPase activity 0.0596 1 / 292
GO:0005216 ion channel activity 0.0708 1 / 348
GO:0015103 inorganic anion tran... 0.0195 1 / 94
GO:0042623 ATPase activity, cou... 0.0456 1 / 222
GO:0005253 anion channel activi... 0.0133 1 / 64
GO:0015108 chloride transmembra... 0.0145 1 / 70
GO:0043492 ATPase activity, cou... 0.0184 1 / 89
GO:0015405 P−P−bond−hydrolysis−... 0.0211 1 / 102
GO:0005254 chloride channel act... 0.0118 1 / 57
GO:0042626 ATPase activity, cou... 0.0180 1 / 87
