# Supplementary material for: Gene Expression in the Hippocampus in a Rat Model of Premenstrual Dysphoric Disorder After Treatment With Baixiangdan Capsules
Source: Front Psychol. 2018 Nov 13;9:2065. doi: 10.3389/fpsyg.2018.02065 (PMC6242977; doi:10.3389/fpsyg.2018.02065)
Supplement: Supplementary file 3 [file Data_Sheet_3.ZIP › Data Analysis Folder/GO Analysis Report/model vs blank (down)/MF_result(Rat).html]

| GO.ID | Term | Ontology | Count | Pop.Hits | List.Total | Pop.Total | Fold.Enrichment | Pvalue | FDR | Enrichment.Score | GENES |
| --- | --- | --- | --- | --- | --- | --- | --- | --- | --- | --- | --- |
| GO:0005254 | chloride channel activity | Molecular function | 1 | 57 | 3 | 14392 | 84.1637426900585 | 0.0118354246718175 | 1 | 1.92681615435357 | CFTR |
| GO:0005253 | anion channel activity | Molecular function | 1 | 64 | 3 | 14392 | 74.9583333333333 | 0.0132824264743929 | 1 | 1.87672257947272 | CFTR |
| GO:0015108 | chloride transmembrane transporter activity | Molecular function | 1 | 70 | 3 | 14392 | 68.5333333333333 | 0.0145215888482787 | 1 | 1.83798586364352 | CFTR |
| GO:0005215 | transporter activity | Molecular function | 2 | 1052 | 3 | 14392 | 9.12040557667934 | 0.0152359746444075 | 1 | 1.81712975876005 | CFTR//FABP12 |
| GO:0016820 | hydrolase activity, acting on acid anhydrides, catalyzing transmembrane movement of substances | Molecular function | 1 | 87 | 3 | 14392 | 55.1417624521073 | 0.0180269139944179 | 1 | 1.74407861326126 | CFTR |
| GO:0042626 | ATPase activity, coupled to transmembrane movement of substances | Molecular function | 1 | 87 | 3 | 14392 | 55.1417624521073 | 0.0180269139944179 | 1 | 1.74407861326126 | CFTR |
| GO:0043492 | ATPase activity, coupled to movement of substances | Molecular function | 1 | 89 | 3 | 14392 | 53.9026217228464 | 0.0184387578682083 | 1 | 1.73426833865943 | CFTR |
| GO:0015103 | inorganic anion transmembrane transporter activity | Molecular function | 1 | 94 | 3 | 14392 | 51.0354609929078 | 0.0194678637241177 | 1 | 1.71068170249792 | CFTR |
| GO:0030165 | PDZ domain binding | Molecular function | 1 | 98 | 3 | 14392 | 48.9523809523809 | 0.0202906303180439 | 1 | 1.69270446162134 | CFTR |
| GO:0015399 | primary active transmembrane transporter activity | Molecular function | 1 | 102 | 3 | 14392 | 47.0326797385621 | 0.0211129365263943 | 1 | 1.67545135795169 | CFTR |
| GO:0015405 | P-P-bond-hydrolysis-driven transmembrane transporter activity | Molecular function | 1 | 102 | 3 | 14392 | 47.0326797385621 | 0.0211129365263943 | 1 | 1.67545135795169 | CFTR |
| GO:0008509 | anion transmembrane transporter activity | Molecular function | 1 | 127 | 3 | 14392 | 37.7742782152231 | 0.0262419277453365 | 1 | 1.58100426463134 | CFTR |
| GO:0042623 | ATPase activity, coupled | Molecular function | 1 | 222 | 3 | 14392 | 21.6096096096096 | 0.0455686825412938 | 1 | 1.34133352733849 | CFTR |
