# Supplementary material for: Gene Expression in the Hippocampus in a Rat Model of Premenstrual Dysphoric Disorder After Treatment With Baixiangdan Capsules
Source: Front Psychol. 2018 Nov 13;9:2065. doi: 10.3389/fpsyg.2018.02065 (PMC6242977; doi:10.3389/fpsyg.2018.02065)
Supplement: Supplementary file 3 [file Data_Sheet_3.ZIP › Data Analysis Folder/GO Analysis Report/model vs blank (up)/BP_Pvalue_tree.pdf]

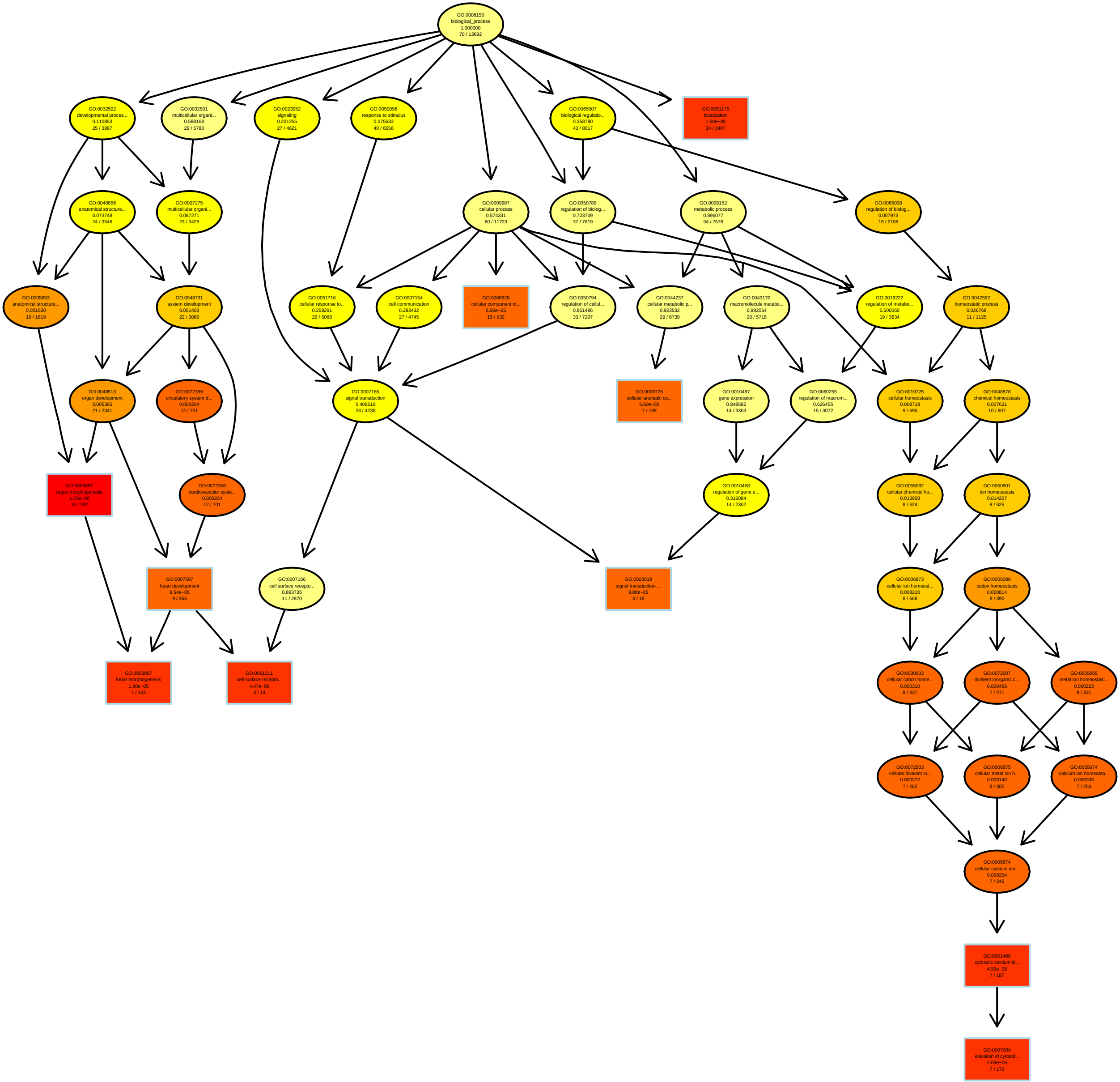
GO:0008150 biological_process 1.000000 70 / 13692
GO:0032502 developmental proces... 0.110853 25 / 3887
GO:0032501 multicellular organi... 0.598168 29 / 5780
GO:0023052 signaling 0.231255 27 / 4621
GO:0050896 response to stimulus 0.075633 40 / 6556
GO:0065007 biological regulatio... 0.358780 43 / 8017
GO:0051179 localization 1.50e−05 34 / 3407
GO:0048856 anatomical structure... 0.073748 24 / 3546
GO:0007275 multicellular organi... 0.087271 23 / 3429
GO:0009987 cellular process 0.574331 60 / 11723
GO:0050789 regulation of biolog... 0.723709 37 / 7619
GO:0008152 metabolic process 0.896077 34 / 7576
GO:0065008 regulation of biolog... 0.007973 19 / 2106
GO:0009653 anatomical structure... 0.001520 19 / 1819
GO:0048731 system development 0.051403 22 / 3069
GO:0051716 cellular response to... 0.258291 29 / 5068
GO:0007154 cell communication 0.283432 27 / 4745
GO:0006928 cellular component m... 5.83e−05 15 / 932
GO:0050794 regulation of cellul... 0.851486 33 / 7207
GO:0044237 cellular metabolic p... 0.923532 29 / 6739
GO:0043170 macromolecule metabo... 0.992054 20 / 5718
GO:0019222 regulation of metabo... 0.500065 19 / 3634
GO:0042592 homeostatic process 0.026768 11 / 1125
GO:0048513 organ development 0.005365 21 / 2341
GO:0072359 circulatory system d... 0.000204 12 / 701
GO:0007165 signal transduction 0.408519 23 / 4238
GO:0006725 cellular aromatic co... 6.60e−05 7 / 198
GO:0010467 gene expression 0.848582 14 / 3363
GO:0060255 regulation of macrom... 0.626455 15 / 3072
GO:0019725 cellular homeostasis 0.008718 9 / 696
GO:0048878 chemical homeostasis 0.007631 10 / 807
GO:0009887 organ morphogenesis 1.76e−06 16 / 792
GO:0072358 cardiovascular syste... 0.000204 12 / 701
GO:0010468 regulation of gene e... 0.316084 14 / 2362
GO:0055082 cellular chemical ho... 0.013958 8 / 624
GO:0050801 ion homeostasis 0.014207 8 / 626
GO:0007507 heart development 9.54e−05 9 / 365
GO:0007166 cell surface recepto... 0.893735 11 / 2870
GO:0023019 signal transduction ... 9.88e−05 3 / 18
GO:0006873 cellular ion homeost... 0.008210 8 / 568
GO:0055080 cation homeostasis 0.000814 8 / 390
GO:0003007 heart morphogenesis 1.90e−05 7 / 163
GO:0061311 cell surface recepto... 4.47e−05 3 / 14
GO:0030003 cellular cation home... 0.000310 8 / 337
GO:0072507 divalent inorganic c... 0.000456 7 / 271
GO:0055065 metal ion homeostasi... 0.000223 8 / 321
GO:0072503 cellular divalent in... 0.000372 7 / 262
GO:0006875 cellular metal ion h... 0.000140 8 / 300
GO:0055074 calcium ion homeosta... 0.000308 7 / 254
GO:0006874 cellular calcium ion... 0.000254 7 / 246
GO:0051480 cytosolic calcium io... 4.59e−05 7 / 187
GO:0007204 elevation of cytosol... 2.69e−05 7 / 172
