# Supplementary material for: Gene Expression in the Hippocampus in a Rat Model of Premenstrual Dysphoric Disorder After Treatment With Baixiangdan Capsules
Source: Front Psychol. 2018 Nov 13;9:2065. doi: 10.3389/fpsyg.2018.02065 (PMC6242977; doi:10.3389/fpsyg.2018.02065)
Supplement: Supplementary file 3 [file Data_Sheet_3.ZIP › Data Analysis Folder/GO Analysis Report/model vs blank (up)/BP_result(Rat).html]

| GO.ID | Term | Ontology | Count | Pop.Hits | List.Total | Pop.Total | Fold.Enrichment | Pvalue | FDR | Enrichment.Score | GENES |
| --- | --- | --- | --- | --- | --- | --- | --- | --- | --- | --- | --- |
| GO:0009887 | organ morphogenesis | Biological process | 16 | 792 | 70 | 13692 | 3.95151515151515 | 1.76317633539152e-06 | 0.00772976505435642 | 5.7537042517064 | COL11A1//FOXJ1//MESP1//SCX//MSX1//TEAD2//MSX2//HES1//HTR2C//LRP2//SLCO1A5//FSCN2//ITGA8//SOSTDC1//AQP1//MMP2 |
| GO:0051179 | localization | Biological process | 34 | 3407 | 70 | 13692 | 1.95198121514529 | 1.50378206072977e-05 | 0.027802355116817 | 4.82281510036725 | NR2F2//XCL1//AQP1//MESP1//CASQ2//TTR//SLC5A5//TRPV4//SLC4A2//GULP1//SCARA5//LRP2//HES1//SLC2A12//DHRS7C//HTR2C//ACE//RAB19//SLC16A8//SLC16A3//SLCO1A5//FOLR1//MDK//MMP2//ENPP2//MSX1//MSX2//MDFIC//KRT18//ITGA8//FOXJ1//SLC24A4//CCDC40//CRB3 |
| GO:0003007 | heart morphogenesis | Biological process | 7 | 163 | 70 | 13692 | 8.4 | 1.90253342496467e-05 | 0.027802355116817 | 4.72066770451786 | FOXJ1//MESP1//TEAD2//MSX2//HES1//MSX1//COL11A1 |
| GO:0007204 | elevation of cytosolic calcium ion concentration | Biological process | 7 | 172 | 70 | 13692 | 7.96046511627907 | 2.69127636047176e-05 | 0.0294963889107705 | 4.5700417033268 | DHRS7C//CASQ2//HTR2C//XCL1//EPOR//TRHR//TRPV4 |
| GO:0061311 | cell surface receptor signaling pathway involved in heart development | Biological process | 3 | 14 | 70 | 13692 | 41.9142857142857 | 4.47403899838168e-05 | 0.0335703395788186 | 4.3493002346542 | MESP1//MSX2//MSX1 |
| GO:0051480 | cytosolic calcium ion homeostasis | Biological process | 7 | 187 | 70 | 13692 | 7.32192513368984 | 4.59448078177262e-05 | 0.0335703395788186 | 4.33776356074185 | EPOR//TRHR//TRPV4//DHRS7C//CASQ2//HTR2C//XCL1 |
| GO:0006928 | cellular component movement | Biological process | 15 | 932 | 70 | 13692 | 3.1480686695279 | 5.83469513503048e-05 | 0.0361651325239864 | 4.23398183107241 | NR2F2//XCL1//MESP1//RSPH9//DNAH1//AQP1//ACE//HES1//MDK//MMP2//ENPP2//MSX2//FOXJ1//CCDC40//CASQ2 |
| GO:0006725 | cellular aromatic compound metabolic process | Biological process | 7 | 198 | 70 | 13692 | 6.91515151515152 | 6.59947673795372e-05 | 0.0361651325239864 | 4.18049049761392 | GDA//SLC5A5//PON3//TTR//DIO3//HTR2C//FOLR1 |
| GO:0007507 | heart development | Biological process | 9 | 365 | 70 | 13692 | 4.82301369863014 | 9.54020804998458e-05 | 0.0433296889436287 | 4.02044215422934 | FOXJ1//MESP1//COL11A1//MSX1//TEAD2//MSX2//HES1//SCX//EPOR |
| GO:0023019 | signal transduction involved in regulation of gene expression | Biological process | 3 | 18 | 70 | 13692 | 32.6 | 9.88359693057223e-05 | 0.0433296889436287 | 4.005084974156 | MSX2//MSX1//MESP1 |
| GO:0030509 | BMP signaling pathway | Biological process | 5 | 95 | 70 | 13692 | 10.2947368421053 | 0.000122581233715476 | 0.0461661824527548 | 3.91157601201697 | MSX2//HES1//MSX1//SOSTDC1//SCX |
| GO:0006875 | cellular metal ion homeostasis | Biological process | 8 | 300 | 70 | 13692 | 5.216 | 0.000140497236877529 | 0.0461661824527548 | 3.85233221683109 | CASQ2//TRPV4//SCARA5//EPOR//TRHR//DHRS7C//HTR2C//XCL1 |
| GO:0040011 | locomotion | Biological process | 14 | 906 | 70 | 13692 | 3.02251655629139 | 0.000165442228695147 | 0.0461661824527548 | 3.78135362810571 | NR2F2//XCL1//MESP1//ENPP2//LMX1A//AQP1//ACE//HES1//MDK//MMP2//MSX2//HTR2C//FOXJ1//CCDC40 |
| GO:0051209 | release of sequestered calcium ion into cytosol | Biological process | 4 | 55 | 70 | 13692 | 14.2254545454545 | 0.000175508089641434 | 0.0461661824527548 | 3.75570286093226 | DHRS7C//CASQ2//XCL1//HTR2C |
| GO:0051282 | regulation of sequestering of calcium ion | Biological process | 4 | 55 | 70 | 13692 | 14.2254545454545 | 0.000175508089641434 | 0.0461661824527548 | 3.75570286093226 | DHRS7C//CASQ2//HTR2C//XCL1 |
| GO:0051283 | negative regulation of sequestering of calcium ion | Biological process | 4 | 55 | 70 | 13692 | 14.2254545454545 | 0.000175508089641434 | 0.0461661824527548 | 3.75570286093226 | DHRS7C//CASQ2//HTR2C//XCL1 |
| GO:0051208 | sequestering of calcium ion | Biological process | 4 | 56 | 70 | 13692 | 13.9714285714286 | 0.000188283535240265 | 0.0461661824527548 | 3.72518765590971 | HTR2C//XCL1//CASQ2 |
| GO:0072358 | cardiovascular system development | Biological process | 12 | 701 | 70 | 13692 | 3.34835948644793 | 0.000203630066364506 | 0.0461661824527548 | 3.69115809719896 | MMP2//TEAD2//FOXJ1//MESP1//COL11A1//MSX1//MSX2//HES1//SCX//EPOR//AQP1//NR2F2 |
| GO:0072359 | circulatory system development | Biological process | 12 | 701 | 70 | 13692 | 3.34835948644793 | 0.000203630066364506 | 0.0461661824527548 | 3.69115809719896 | MMP2//TEAD2//FOXJ1//MESP1//COL11A1//MSX1//MSX2//HES1//SCX//EPOR//AQP1//NR2F2 |
| GO:0042403 | thyroid hormone metabolic process | Biological process | 3 | 23 | 70 | 13692 | 25.5130434782609 | 0.000210612146226071 | 0.0461661824527548 | 3.67651658620444 | SLC5A5//DIO3//TTR |
| GO:0055065 | metal ion homeostasis | Biological process | 8 | 321 | 70 | 13692 | 4.87476635514019 | 0.000223119975944739 | 0.0465789511686541 | 3.65146154556465 | CASQ2//TRPV4//SCARA5//EPOR//TRHR//DHRS7C//HTR2C//XCL1 |
| GO:0030510 | regulation of BMP signaling pathway | Biological process | 4 | 60 | 70 | 13692 | 13.04 | 0.000246162585305996 | 0.0483816106098895 | 3.60877795558806 | MSX2//HES1//MSX1//SOSTDC1 |
| GO:0006874 | cellular calcium ion homeostasis | Biological process | 7 | 246 | 70 | 13692 | 5.56585365853658 | 0.000253826880480716 | 0.0483816106098895 | 3.59546238765393 | CASQ2//EPOR//TRHR//TRPV4//DHRS7C//HTR2C//XCL1 |
| GO:0051238 | sequestering of metal ion | Biological process | 4 | 63 | 70 | 13692 | 12.4190476190476 | 0.000297251570760759 | 0.0512851546797747 | 3.52687584176923 | CASQ2//HTR2C//XCL1 |
| GO:0055074 | calcium ion homeostasis | Biological process | 7 | 254 | 70 | 13692 | 5.39055118110236 | 0.000308330671901417 | 0.0512851546797747 | 3.51098327068393 | CASQ2//TRPV4//EPOR//TRHR//DHRS7C//HTR2C//XCL1 |
| GO:0030003 | cellular cation homeostasis | Biological process | 8 | 337 | 70 | 13692 | 4.6433234421365 | 0.000309922900080423 | 0.0512851546797747 | 3.50874633272773 | CASQ2//TRPV4//SCARA5//EPOR//TRHR//DHRS7C//HTR2C//XCL1 |
| GO:0090100 | positive regulation of transmembrane receptor protein serine/threonine kinase signaling pathway | Biological process | 4 | 64 | 70 | 13692 | 12.225 | 0.000315852914314306 | 0.0512851546797747 | 3.50051511159918 | ITGA8//MSX2//HES1//MSX1 |
| GO:0030513 | positive regulation of BMP signaling pathway | Biological process | 3 | 27 | 70 | 13692 | 21.7333333333333 | 0.000342790081922162 | 0.0518886979784153 | 3.46497175217704 | MSX2//HES1//MSX1 |
| GO:0006811 | ion transport | Biological process | 12 | 742 | 70 | 13692 | 3.1633423180593 | 0.000343241843379116 | 0.0518886979784153 | 3.46439977431708 | AQP1//SLC5A5//TRPV4//SLC4A2//DHRS7C//CASQ2//SLC16A8//SLC16A3//SLCO1A5//SCARA5//HTR2C//XCL1 |
| GO:0072503 | cellular divalent inorganic cation homeostasis | Biological process | 7 | 262 | 70 | 13692 | 5.22595419847328 | 0.000371883749751977 | 0.0543446119637556 | 3.42959279864355 | CASQ2//TRPV4//EPOR//TRHR//DHRS7C//HTR2C//XCL1 |
| GO:0018958 | phenol-containing compound metabolic process | Biological process | 4 | 68 | 70 | 13692 | 11.5058823529412 | 0.000398670175754433 | 0.0563796790486269 | 3.39938625239817 | SLC5A5//TTR//DIO3//HTR2C |
| GO:0010817 | regulation of hormone levels | Biological process | 8 | 354 | 70 | 13692 | 4.42033898305085 | 0.000430658127314939 | 0.0590001634421466 | 3.3658673524308 | SLC5A5//TRPV4//TTR//DIO3//ACE//LRP2//AQP1//SLCO1A5 |
| GO:0072507 | divalent inorganic cation homeostasis | Biological process | 7 | 271 | 70 | 13692 | 5.05239852398524 | 0.000455526035544423 | 0.0605159436311137 | 3.34148679592448 | CASQ2//TRPV4//EPOR//TRHR//DHRS7C//HTR2C//XCL1 |
| GO:0060402 | calcium ion transport into cytosol | Biological process | 4 | 71 | 70 | 13692 | 11.0197183098592 | 0.000470204230851913 | 0.0606286867074937 | 3.32771346745401 | DHRS7C//CASQ2//HTR2C//XCL1 |
| GO:0060401 | cytosolic calcium ion transport | Biological process | 4 | 72 | 70 | 13692 | 10.8666666666667 | 0.000495956547076031 | 0.0621221000680377 | 3.30455637228355 | DHRS7C//CASQ2//HTR2C//XCL1 |
| GO:0048870 | cell motility | Biological process | 12 | 779 | 70 | 13692 | 3.01309370988447 | 0.000532383762006432 | 0.0626859196287588 | 3.27377519921267 | NR2F2//XCL1//MESP1//AQP1//ACE//HES1//MDK//MMP2//ENPP2//MSX2//FOXJ1//CCDC40 |
| GO:0051674 | localization of cell | Biological process | 12 | 779 | 70 | 13692 | 3.01309370988447 | 0.000532383762006432 | 0.0626859196287588 | 3.27377519921267 | NR2F2//XCL1//MESP1//AQP1//ACE//HES1//MDK//MMP2//ENPP2//MSX2//FOXJ1//CCDC40 |
| GO:0007178 | transmembrane receptor protein serine/threonine kinase signaling pathway | Biological process | 6 | 200 | 70 | 13692 | 5.868 | 0.000543354230358767 | 0.0626859196287588 | 3.26491694733108 | MSX2//MSX1//SCX//ITGA8//HES1//SOSTDC1 |
| GO:0051279 | regulation of release of sequestered calcium ion into cytosol | Biological process | 3 | 32 | 70 | 13692 | 18.3375 | 0.00057073949335984 | 0.0641569727920394 | 3.24356207461275 | DHRS7C//CASQ2//XCL1 |
| GO:0090092 | regulation of transmembrane receptor protein serine/threonine kinase signaling pathway | Biological process | 5 | 134 | 70 | 13692 | 7.29850746268657 | 0.000605710615194205 | 0.0663858834252849 | 3.21773481517627 | ITGA8//MSX2//HES1//MSX1//SOSTDC1 |
| GO:0006820 | anion transport | Biological process | 5 | 138 | 70 | 13692 | 7.08695652173913 | 0.000692248295103238 | 0.0730924371850036 | 3.15973810517887 | SLC4A2//SLC5A5//SLC16A8//SLC16A3//SLCO1A5 |
| GO:0048598 | embryonic morphogenesis | Biological process | 9 | 478 | 70 | 13692 | 3.68284518828452 | 0.000700246889089907 | 0.0730924371850036 | 3.1547488118926 | SCX//TEAD2//FOXJ1//MESP1//HES1//MSX2//MSX1//COL11A1//ITGA8 |
| GO:0030901 | midbrain development | Biological process | 3 | 35 | 70 | 13692 | 16.7657142857143 | 0.000744906833283098 | 0.075945850165421 | 3.12789804177688 | HES1//MSX1//LMX1A |
| GO:0007389 | pattern specification process | Biological process | 8 | 387 | 70 | 13692 | 4.04341085271318 | 0.000774091277577615 | 0.0771276400204606 | 3.11120782612458 | FOXJ1//MESP1//MSX2//MSX1//NR2F2//HES1//CCDC40//SOSTDC1 |
| GO:0055080 | cation homeostasis | Biological process | 8 | 390 | 70 | 13692 | 4.01230769230769 | 0.000813947548564595 | 0.079296578953493 | 3.08940358049521 | CASQ2//TRPV4//SCARA5//EPOR//TRHR//DHRS7C//HTR2C//XCL1 |
| GO:0030900 | forebrain development | Biological process | 7 | 306 | 70 | 13692 | 4.47450980392157 | 0.000934284855310105 | 0.0890414088191196 | 3.02952069097742 | HES1//MDK//LMX1A//AQP1//MSX1//LRP2//NR2F2 |
| GO:0016477 | cell migration | Biological process | 11 | 725 | 70 | 13692 | 2.96772413793103 | 0.00105404433908276 | 0.0983176677135919 | 2.97714111985027 | NR2F2//XCL1//MESP1//AQP1//ACE//ENPP2//MSX2//FOXJ1//HES1//MDK//MMP2 |
| GO:0016338 | calcium-independent cell-cell adhesion | Biological process | 2 | 10 | 70 | 13692 | 39.12 | 0.00112914064840245 | 0.0990030520519268 | 2.94725195796821 | CLDN1//CLDN3 |
| GO:0042447 | hormone catabolic process | Biological process | 2 | 10 | 70 | 13692 | 39.12 | 0.00112914064840245 | 0.0990030520519268 | 2.94725195796821 | DIO3//ACE |
| GO:0048368 | lateral mesoderm development | Biological process | 2 | 10 | 70 | 13692 | 39.12 | 0.00112914064840245 | 0.0990030520519268 | 2.94725195796821 | TEAD2//MESP1 |
| GO:0003143 | embryonic heart tube morphogenesis | Biological process | 3 | 42 | 70 | 13692 | 13.9714285714286 | 0.0012735890087903 | 0.109478710088954 | 2.89497069759818 | FOXJ1//MESP1//TEAD2 |
| GO:0051145 | smooth muscle cell differentiation | Biological process | 3 | 43 | 70 | 13692 | 13.646511627907 | 0.00136412011657157 | 0.113777543719483 | 2.8651473865216 | MESP1//HES1//ITGA8 |
| GO:0035886 | vascular smooth muscle cell differentiation | Biological process | 2 | 11 | 70 | 13692 | 35.5636363636364 | 0.00137550406412696 | 0.113777543719483 | 2.86153812208614 | MESP1//HES1 |
| GO:0009653 | anatomical structure morphogenesis | Biological process | 19 | 1819 | 70 | 13692 | 2.04310060472787 | 0.00152042673456571 | 0.122243518793008 | 2.81803450254415 | MSX1//COL11A1//MMP2//TEAD2//SCX//FOXJ1//MESP1//MSX2//HES1//LMX1A//HTR2C//LRP2//SLCO1A5//RSPH9//FSCN2//ITGA8//SOSTDC1//AQP1//NR2F2 |
| GO:0010522 | regulation of calcium ion transport into cytosol | Biological process | 3 | 45 | 70 | 13692 | 13.04 | 0.00155709567953959 | 0.122243518793008 | 2.80768470032887 | DHRS7C//CASQ2//XCL1 |
| GO:0014808 | release of sequestered calcium ion into cytosol by sarcoplasmic reticulum | Biological process | 2 | 12 | 70 | 13692 | 32.6 | 0.00164515684507013 | 0.122243518793008 | 2.78379269120865 | DHRS7C//CASQ2 |
| GO:0042538 | hyperosmotic salinity response | Biological process | 2 | 12 | 70 | 13692 | 32.6 | 0.00164515684507013 | 0.122243518793008 | 2.78379269120865 | AQP1//TRPV4 |
| GO:0070296 | sarcoplasmic reticulum calcium ion transport | Biological process | 2 | 12 | 70 | 13692 | 32.6 | 0.00164515684507013 | 0.122243518793008 | 2.78379269120865 | DHRS7C//CASQ2 |
| GO:0071470 | cellular response to osmotic stress | Biological process | 2 | 12 | 70 | 13692 | 32.6 | 0.00164515684507013 | 0.122243518793008 | 2.78379269120865 | TRPV4//AQP1 |
| GO:0003205 | cardiac chamber development | Biological process | 4 | 100 | 70 | 13692 | 7.824 | 0.00169806798889247 | 0.124072167721743 | 2.77004492504042 | MSX2//MESP1//HES1//COL11A1 |
| GO:0007420 | brain development | Biological process | 9 | 549 | 70 | 13692 | 3.20655737704918 | 0.00184433725213545 | 0.13255040185839 | 2.73415966172637 | HES1//MDK//LMX1A//AQP1//MSX1//LRP2//NR2F2//EPOR//ITGA8 |
| GO:0003203 | endocardial cushion morphogenesis | Biological process | 2 | 13 | 70 | 13692 | 30.0923076923077 | 0.00193786128320397 | 0.13485053754867 | 2.71267731402169 | MSX2//MSX1 |
| GO:0021542 | dentate gyrus development | Biological process | 2 | 13 | 70 | 13692 | 30.0923076923077 | 0.00193786128320397 | 0.13485053754867 | 2.71267731402169 | MDK//LMX1A |
| GO:0048729 | tissue morphogenesis | Biological process | 8 | 449 | 70 | 13692 | 3.48507795100223 | 0.00199792052925044 | 0.136857556253655 | 2.69942179058172 | SCX//TEAD2//FOXJ1//MESP1//MSX2//MSX1//COL11A1//MMP2 |
| GO:0035094 | response to nicotine | Biological process | 3 | 50 | 70 | 13692 | 11.736 | 0.00211188925967205 | 0.142438807913881 | 2.67532885847453 | HTR2C//MSX1//MMP2 |
| GO:0044092 | negative regulation of molecular function | Biological process | 9 | 562 | 70 | 13692 | 3.13238434163701 | 0.00216317959161098 | 0.143687565600341 | 2.66490742304455 | LRP2//SERPINB1A//FOXJ1//MSX2//AQP1//MSX1//XCL1//NR2F2//CASQ2 |
| GO:0071392 | cellular response to estradiol stimulus | Biological process | 2 | 14 | 70 | 13692 | 27.9428571428571 | 0.002253381432074 | 0.147445137286752 | 2.64716528860286 | MSX2//MMP2 |
| GO:0048705 | skeletal system morphogenesis | Biological process | 5 | 181 | 70 | 13692 | 5.40331491712707 | 0.00230952634858733 | 0.147891367850316 | 2.63647707867363 | COL11A1//SCX//MSX2//MMP2//MSX1 |
| GO:0042476 | odontogenesis | Biological process | 4 | 109 | 70 | 13692 | 7.17798165137615 | 0.00232766979508937 | 0.147891367850316 | 2.63307862898003 | MSX1//SOSTDC1//MSX2//AQP1 |
| GO:0003341 | cilium movement | Biological process | 2 | 15 | 70 | 13692 | 26.08 | 0.00259148309541713 | 0.160014956201531 | 2.58645161977487 | CCDC40//RSPH9 |
| GO:2000677 | regulation of transcription regulatory region DNA binding | Biological process | 2 | 15 | 70 | 13692 | 26.08 | 0.00259148309541713 | 0.160014956201531 | 2.58645161977487 | MSX2//MSX1 |
| GO:0035050 | embryonic heart tube development | Biological process | 3 | 55 | 70 | 13692 | 10.6690909090909 | 0.00277578796473505 | 0.167855053472085 | 2.55661371158265 | FOXJ1//MESP1//TEAD2 |
| GO:0030001 | metal ion transport | Biological process | 8 | 474 | 70 | 13692 | 3.30126582278481 | 0.00279503168418389 | 0.167855053472085 | 2.55361326463329 | AQP1//SLC5A5//TRPV4//DHRS7C//CASQ2//SCARA5//HTR2C//XCL1 |
| GO:0002063 | chondrocyte development | Biological process | 2 | 16 | 70 | 13692 | 24.45 | 0.00295193381584741 | 0.170279971693093 | 2.52989338388642 | MSX2//COL11A1 |
| GO:0002823 | negative regulation of adaptive immune response based on somatic recombination of immune receptors built from immunoglobulin superfamily domains | Biological process | 2 | 16 | 70 | 13692 | 24.45 | 0.00295193381584741 | 0.170279971693093 | 2.52989338388642 | FOXJ1//XCL1 |
| GO:0060317 | cardiac epithelial to mesenchymal transition | Biological process | 2 | 16 | 70 | 13692 | 24.45 | 0.00295193381584741 | 0.170279971693093 | 2.52989338388642 | MSX2//MSX1 |
| GO:0045747 | positive regulation of Notch signaling pathway | Biological process | 2 | 17 | 70 | 13692 | 23.0117647058823 | 0.00333450286360915 | 0.187122469550771 | 2.47696890528113 | MESP1//HES1 |
| GO:0072132 | mesenchyme morphogenesis | Biological process | 2 | 17 | 70 | 13692 | 23.0117647058823 | 0.00333450286360915 | 0.187122469550771 | 2.47696890528113 | MSX2//MSX1 |
| GO:0051234 | establishment of localization | Biological process | 24 | 2726 | 70 | 13692 | 1.72208363903155 | 0.00337196055987932 | 0.187122469550771 | 2.47211751374093 | AQP1//TTR//SLC5A5//TRPV4//SLC4A2//GULP1//SCARA5//LRP2//HES1//SLC2A12//DHRS7C//CASQ2//HTR2C//RAB19//SLC16A8//SLC16A3//SLCO1A5//FOLR1//MDFIC//KRT18//ITGA8//ACE//XCL1//SLC24A4 |
| GO:0003002 | regionalization | Biological process | 6 | 289 | 70 | 13692 | 4.06089965397924 | 0.00355123661256744 | 0.194607766368696 | 2.44962039046691 | MESP1//MSX2//MSX1//NR2F2//HES1//FOXJ1 |
| GO:0002820 | negative regulation of adaptive immune response | Biological process | 2 | 18 | 70 | 13692 | 21.7333333333333 | 0.00373896122539708 | 0.197489229061937 | 2.42724903863915 | FOXJ1//XCL1 |
| GO:0060914 | heart formation | Biological process | 2 | 18 | 70 | 13692 | 21.7333333333333 | 0.00373896122539708 | 0.197489229061937 | 2.42724903863915 | MESP1//HES1 |
| GO:2000826 | regulation of heart morphogenesis | Biological process | 2 | 18 | 70 | 13692 | 21.7333333333333 | 0.00373896122539708 | 0.197489229061937 | 2.42724903863915 | HES1//MESP1 |
| GO:0015711 | organic anion transport | Biological process | 3 | 62 | 70 | 13692 | 9.46451612903226 | 0.00390091484426896 | 0.203590603300894 | 2.40883353009522 | SLC16A8//SLC16A3//SLCO1A5 |
| GO:0042310 | vasoconstriction | Biological process | 3 | 63 | 70 | 13692 | 9.31428571428571 | 0.00408109800794437 | 0.210488631374448 | 2.38922297547953 | MMP2//HTR2C//ACE |
| GO:2000021 | regulation of ion homeostasis | Biological process | 4 | 129 | 70 | 13692 | 6.06511627906977 | 0.00426788608701136 | 0.217562937272765 | 2.36978718072896 | DHRS7C//CASQ2//HTR2C//XCL1 |
| GO:0051101 | regulation of DNA binding | Biological process | 3 | 65 | 70 | 13692 | 9.02769230769231 | 0.00445648364620321 | 0.224565796608677 | 2.35100768285248 | HES1//MSX1//MSX2 |
| GO:0048646 | anatomical structure formation involved in morphogenesis | Biological process | 10 | 750 | 70 | 13692 | 2.608 | 0.00460429297393516 | 0.224686739351228 | 2.33683704974355 | MMP2//SCX//TEAD2//MESP1//MSX2//MSX1//HES1//RSPH9//FOXJ1//AQP1 |
| GO:0003197 | endocardial cushion development | Biological process | 2 | 20 | 70 | 13692 | 19.56 | 0.00461263835346956 | 0.224686739351228 | 2.33605059422101 | MSX2//MSX1 |
| GO:0071391 | cellular response to estrogen stimulus | Biological process | 2 | 20 | 70 | 13692 | 19.56 | 0.00461263835346956 | 0.224686739351228 | 2.33605059422101 | MSX2//MMP2 |
| GO:0006575 | cellular modified amino acid metabolic process | Biological process | 4 | 134 | 70 | 13692 | 5.83880597014925 | 0.00488391138101966 | 0.227315212366361 | 2.31123222496535 | SLC5A5//TTR//DIO3//FOLR1 |
| GO:0043279 | response to alkaloid | Biological process | 4 | 134 | 70 | 13692 | 5.83880597014925 | 0.00488391138101966 | 0.227315212366361 | 2.31123222496535 | MMP2//HTR2C//CASQ2//MSX1 |
| GO:0009888 | tissue development | Biological process | 13 | 1149 | 70 | 13692 | 2.21305483028721 | 0.00500074639240499 | 0.227315212366361 | 2.30096516968186 | COL11A1//SCX//MSX1//TEAD2//FOXJ1//MESP1//MSX2//NR2F2//EPOR//ITGA8//MMP2//HES1//AQP1 |
| GO:0048736 | appendage development | Biological process | 4 | 135 | 70 | 13692 | 5.79555555555556 | 0.00501390755179969 | 0.227315212366361 | 2.2998236779792 | MSX2//MSX1//SCX//NR2F2 |
| GO:0060173 | limb development | Biological process | 4 | 135 | 70 | 13692 | 5.79555555555556 | 0.00501390755179969 | 0.227315212366361 | 2.2998236779792 | MSX2//MSX1//SCX//NR2F2 |
| GO:0002062 | chondrocyte differentiation | Biological process | 3 | 68 | 70 | 13692 | 8.62941176470588 | 0.00505770812267898 | 0.227315212366361 | 2.29604623714414 | MSX2//COL11A1//SCX |
| GO:0002704 | negative regulation of leukocyte mediated immunity | Biological process | 2 | 21 | 70 | 13692 | 18.6285714285714 | 0.00508140757570788 | 0.227315212366361 | 2.29401596927355 | XCL1//FOXJ1 |
| GO:0002707 | negative regulation of lymphocyte mediated immunity | Biological process | 2 | 21 | 70 | 13692 | 18.6285714285714 | 0.00508140757570788 | 0.227315212366361 | 2.29401596927355 | XCL1//FOXJ1 |
| GO:0045216 | cell-cell junction organization | Biological process | 3 | 69 | 70 | 13692 | 8.50434782608696 | 0.00526844111207755 | 0.233301473084323 | 2.27831786990596 | TRPV4//CLDN19//CLDN1 |
| GO:0048513 | organ development | Biological process | 21 | 2341 | 70 | 13692 | 1.75463477146519 | 0.00536517018676965 | 0.235209060987981 | 2.27041649736948 | COL11A1//MMP2//ITGA8//ACE//MSX1//HES1//FOXJ1//MESP1//SCX//MSX2//TEAD2//EPOR//LMX1A//NR2F2//MDK//AQP1//LRP2//HTR2C//SLCO1A5//FSCN2//SOSTDC1 |
| GO:0007368 | determination of left/right symmetry | Biological process | 3 | 70 | 70 | 13692 | 8.38285714285714 | 0.00548439728353891 | 0.235721545990535 | 2.26087109294173 | FOXJ1//MESP1//CCDC40 |
| GO:0060349 | bone morphogenesis | Biological process | 3 | 70 | 70 | 13692 | 8.38285714285714 | 0.00548439728353891 | 0.235721545990535 | 2.26087109294173 | SCX//MSX2//MSX1 |
| GO:0006810 | transport | Biological process | 23 | 2679 | 70 | 13692 | 1.67928331466965 | 0.0058680797709227 | 0.249763705977914 | 2.23150399097121 | AQP1//SLC5A5//TRPV4//SLC4A2//GULP1//SCARA5//LRP2//HES1//SLC2A12//DHRS7C//CASQ2//HTR2C//RAB19//SLC16A8//SLC16A3//SLCO1A5//FOLR1//MDFIC//KRT18//ACE//XCL1//SLC24A4//TTR |
| GO:0006972 | hyperosmotic response | Biological process | 2 | 23 | 70 | 13692 | 17.0086956521739 | 0.00608169603586511 | 0.251529768124836 | 2.21597528975673 | AQP1//TRPV4 |
| GO:0060343 | trabecula formation | Biological process | 2 | 23 | 70 | 13692 | 17.0086956521739 | 0.00608169603586511 | 0.251529768124836 | 2.21597528975673 | MSX2//MMP2 |
| GO:0071312 | cellular response to alkaloid | Biological process | 2 | 23 | 70 | 13692 | 17.0086956521739 | 0.00608169603586511 | 0.251529768124836 | 2.21597528975673 | CASQ2//MSX1 |
| GO:0060429 | epithelium development | Biological process | 8 | 545 | 70 | 13692 | 2.87119266055046 | 0.0064584000538536 | 0.264613325570974 | 2.18987505690326 | TEAD2//FOXJ1//MESP1//MSX2//MMP2//NR2F2//MSX1//AQP1 |
| GO:0042475 | odontogenesis of dentin-containing tooth | Biological process | 3 | 75 | 70 | 13692 | 7.824 | 0.00664372020944571 | 0.269685827761204 | 2.1775886655121 | MSX2//MSX1//SOSTDC1 |
| GO:0006766 | vitamin metabolic process | Biological process | 3 | 76 | 70 | 13692 | 7.72105263157895 | 0.00689171931279708 | 0.277186215296352 | 2.1616724188888 | TTR//FOLR1//LRP2 |
| GO:0006816 | calcium ion transport | Biological process | 5 | 236 | 70 | 13692 | 4.14406779661017 | 0.00711016001464507 | 0.281304923017317 | 2.14812062533277 | DHRS7C//CASQ2//HTR2C//XCL1//TRPV4 |
| GO:0009855 | determination of bilateral symmetry | Biological process | 3 | 77 | 70 | 13692 | 7.62077922077922 | 0.00714516778825859 | 0.281304923017317 | 2.14598756832028 | FOXJ1//MESP1//CCDC40 |
| GO:0043627 | response to estrogen stimulus | Biological process | 5 | 237 | 70 | 13692 | 4.12658227848101 | 0.0072350879598471 | 0.281304923017317 | 2.14055618462179 | NR2F2//FOXJ1//MSX2//MMP2//AQP1 |
| GO:0051216 | cartilage development | Biological process | 4 | 150 | 70 | 13692 | 5.216 | 0.00725078838981678 | 0.281304923017317 | 2.13961476932806 | COL11A1//SCX//MSX2//MSX1 |
| GO:0009799 | specification of symmetry | Biological process | 3 | 78 | 70 | 13692 | 7.52307692307692 | 0.00740409439940564 | 0.282857057067109 | 2.13052805279331 | FOXJ1//MESP1//CCDC40 |
| GO:0032355 | response to estradiol stimulus | Biological process | 4 | 151 | 70 | 13692 | 5.18145695364238 | 0.00741983612288264 | 0.282857057067109 | 2.12960568659614 | MSX2//MMP2//NR2F2//FOXJ1 |
| GO:0045893 | positive regulation of transcription, DNA-dependent | Biological process | 11 | 936 | 70 | 13692 | 2.29871794871795 | 0.00756277195694193 | 0.284986507209162 | 2.12131899484849 | PLAGL1//HES1//MSX1//FOXJ1//TEAD2//MESP1//SCX//ITGA8//MDK//NR2F2//MDFIC |
| GO:0048878 | chemical homeostasis | Biological process | 10 | 807 | 70 | 13692 | 2.42379182156134 | 0.00763115056044658 | 0.284986507209162 | 2.11740997785348 | AQP1//CASQ2//TRPV4//SCARA5//EPOR//TRHR//DHRS7C//PYGL//HTR2C//XCL1 |
| GO:0019439 | aromatic compound catabolic process | Biological process | 2 | 26 | 70 | 13692 | 15.0461538461538 | 0.00773571951594212 | 0.284986507209162 | 2.11149928542026 | PON3//DIO3 |
| GO:0035115 | embryonic forelimb morphogenesis | Biological process | 2 | 26 | 70 | 13692 | 15.0461538461538 | 0.00773571951594212 | 0.284986507209162 | 2.11149928542026 | MSX2//MSX1 |
| GO:0065008 | regulation of biological quality | Biological process | 19 | 2106 | 70 | 13692 | 1.76467236467236 | 0.00797290767167045 | 0.289732083880871 | 2.09838326511976 | SCX//CASQ2//ACE//AQP1//SLC5A5//TRPV4//SCARA5//EPOR//TRHR//DHRS7C//HTR2C//MSX1//TTR//DIO3//PYGL//MMP2//LRP2//XCL1//SLCO1A5 |
| GO:0007399 | nervous system development | Biological process | 15 | 1503 | 70 | 13692 | 1.95209580838323 | 0.00803532241284008 | 0.289732083880871 | 2.09499669272663 | NR2F2//TEAD2//LMX1A//EPOR//ITGA8//TRPV4//MMP2//HES1//MDK//AQP1//MSX1//MESP1//LRP2//FSCN2//ACE |
| GO:0006873 | cellular ion homeostasis | Biological process | 8 | 568 | 70 | 13692 | 2.75492957746479 | 0.00820958394346194 | 0.289732083880871 | 2.08567885209337 | CASQ2//TRPV4//SCARA5//EPOR//TRHR//DHRS7C//HTR2C//XCL1 |
| GO:0003231 | cardiac ventricle development | Biological process | 3 | 81 | 70 | 13692 | 7.24444444444444 | 0.00821401788024029 | 0.289732083880871 | 2.0854443561109 | MESP1//HES1//COL11A1 |
| GO:0006182 | cGMP biosynthetic process | Biological process | 2 | 27 | 70 | 13692 | 14.4888888888889 | 0.00832715387066373 | 0.289732083880871 | 2.07950341029513 | HTR2C//AQP1 |
| GO:0010470 | regulation of gastrulation | Biological process | 2 | 27 | 70 | 13692 | 14.4888888888889 | 0.00832715387066373 | 0.289732083880871 | 2.07950341029513 | MESP1//SCX |
| GO:0060325 | face morphogenesis | Biological process | 2 | 27 | 70 | 13692 | 14.4888888888889 | 0.00832715387066373 | 0.289732083880871 | 2.07950341029513 | MMP2//MSX1 |
| GO:0042445 | hormone metabolic process | Biological process | 4 | 157 | 70 | 13692 | 4.98343949044586 | 0.00848899235062473 | 0.293037342245188 | 2.07114385775782 | SLC5A5//TTR//DIO3//ACE |
| GO:0019725 | cellular homeostasis | Biological process | 9 | 696 | 70 | 13692 | 2.52931034482759 | 0.00871814138394002 | 0.297048713923076 | 2.05957609221589 | CASQ2//TRPV4//SCARA5//AQP1//EPOR//TRHR//DHRS7C//HTR2C//XCL1 |
| GO:0006812 | cation transport | Biological process | 8 | 575 | 70 | 13692 | 2.72139130434783 | 0.00880782957216481 | 0.297048713923076 | 2.05513109737242 | AQP1//SLC5A5//TRPV4//DHRS7C//CASQ2//SCARA5//HTR2C//XCL1 |
| GO:0051254 | positive regulation of RNA metabolic process | Biological process | 11 | 956 | 70 | 13692 | 2.25062761506276 | 0.00880847007527369 | 0.297048713923076 | 2.05509951673726 | HES1//MDK//NR2F2//MESP1//MDFIC//SCX//PLAGL1//MSX1//FOXJ1//TEAD2//ITGA8 |
| GO:0051172 | negative regulation of nitrogen compound metabolic process | Biological process | 10 | 828 | 70 | 13692 | 2.36231884057971 | 0.00907244730604888 | 0.303533198850152 | 2.04227554555878 | MSX2//HES1//MSX1//NR2F2//FOXJ1//XCL1//MESP1//MDFIC//SCX//HTR2C |
| GO:0071702 | organic substance transport | Biological process | 7 | 462 | 70 | 13692 | 2.96363636363636 | 0.00913922952742245 | 0.303533198850152 | 2.03909041543608 | GULP1//SLC2A12//HTR2C//SLCO1A5//AQP1//FOLR1//ACE |
| GO:0009651 | response to salt stress | Biological process | 2 | 29 | 70 | 13692 | 13.4896551724138 | 0.00956888524875417 | 0.315413480680739 | 2.0191386534994 | AQP1//TRPV4 |
| GO:0007417 | central nervous system development | Biological process | 9 | 712 | 70 | 13692 | 2.47247191011236 | 0.0100370519563225 | 0.328376386391924 | 1.99839382773964 | EPOR//LMX1A//ITGA8//HES1//MDK//AQP1//MSX1//LRP2//NR2F2 |
| GO:0007264 | small GTPase mediated signal transduction | Biological process | 7 | 474 | 70 | 13692 | 2.88860759493671 | 0.0104347530635254 | 0.334780952153101 | 1.9815178239744 | CTNNAL1//RGL3//RHOD//RAB19//RAB20 |
| GO:0003206 | cardiac chamber morphogenesis | Biological process | 3 | 89 | 70 | 13692 | 6.59325842696629 | 0.0106212315002696 | 0.334780952153101 | 1.97382512517883 | MSX2//MESP1//COL11A1 |
| GO:0045892 | negative regulation of transcription, DNA-dependent | Biological process | 9 | 720 | 70 | 13692 | 2.445 | 0.0107508816068205 | 0.334780952153101 | 1.96855592074574 | MSX2//HES1//MSX1//NR2F2//FOXJ1//XCL1//MESP1//MDFIC//SCX |
| GO:0035116 | embryonic hindlimb morphogenesis | Biological process | 2 | 31 | 70 | 13692 | 12.6193548387097 | 0.0108877024354673 | 0.334780952153101 | 1.96306375705532 | MSX2//MSX1 |
| GO:0043392 | negative regulation of DNA binding | Biological process | 2 | 31 | 70 | 13692 | 12.6193548387097 | 0.0108877024354673 | 0.334780952153101 | 1.96306375705532 | MSX2//MSX1 |
| GO:0060323 | head morphogenesis | Biological process | 2 | 31 | 70 | 13692 | 12.6193548387097 | 0.0108877024354673 | 0.334780952153101 | 1.96306375705532 | MMP2//MSX1 |
| GO:0061383 | trabecula morphogenesis | Biological process | 2 | 31 | 70 | 13692 | 12.6193548387097 | 0.0108877024354673 | 0.334780952153101 | 1.96306375705532 | MSX2//MMP2 |
| GO:0071320 | cellular response to cAMP | Biological process | 2 | 31 | 70 | 13692 | 12.6193548387097 | 0.0108877024354673 | 0.334780952153101 | 1.96306375705532 | AQP1//SLC5A5 |
| GO:0051098 | regulation of binding | Biological process | 4 | 169 | 70 | 13692 | 4.62958579881657 | 0.010920090364483 | 0.334780952153101 | 1.96177376780033 | HES1//MSX1//TRHR//MSX2 |
| GO:0051235 | maintenance of location | Biological process | 4 | 170 | 70 | 13692 | 4.60235294117647 | 0.0111409103405771 | 0.339178825924236 | 1.95307932085562 | CASQ2//HTR2C//XCL1 |
| GO:0007498 | mesoderm development | Biological process | 3 | 91 | 70 | 13692 | 6.44835164835165 | 0.0112802196347401 | 0.341051606060004 | 1.94768244421553 | SCX//MESP1//TEAD2 |
| GO:0070838 | divalent metal ion transport | Biological process | 5 | 266 | 70 | 13692 | 3.67669172932331 | 0.0115614716047237 | 0.341846006815016 | 1.93698688311837 | TRPV4//DHRS7C//CASQ2//HTR2C//XCL1 |
| GO:0003156 | regulation of organ formation | Biological process | 2 | 32 | 70 | 13692 | 12.225 | 0.0115754999649411 | 0.341846006815016 | 1.93646024200972 | HES1//MESP1 |
| GO:0030199 | collagen fibril organization | Biological process | 2 | 32 | 70 | 13692 | 12.225 | 0.0115754999649411 | 0.341846006815016 | 1.93646024200972 | COL11A1//SCX |
| GO:0031100 | organ regeneration | Biological process | 3 | 92 | 70 | 13692 | 6.37826086956522 | 0.0116183975856381 | 0.341846006815016 | 1.93485376589609 | HTR2C//LRP2//SLCO1A5 |
| GO:0072511 | divalent inorganic cation transport | Biological process | 5 | 269 | 70 | 13692 | 3.63568773234201 | 0.0120913898704804 | 0.350498115959403 | 1.91752377536858 | TRPV4//DHRS7C//CASQ2//HTR2C//XCL1 |
| GO:0002712 | regulation of B cell mediated immunity | Biological process | 2 | 33 | 70 | 13692 | 11.8545454545455 | 0.0122819501813947 | 0.350498115959403 | 1.91073266855532 | FOXJ1//XCL1 |
| GO:0002889 | regulation of immunoglobulin mediated immune response | Biological process | 2 | 33 | 70 | 13692 | 11.8545454545455 | 0.0122819501813947 | 0.350498115959403 | 1.91073266855532 | FOXJ1//XCL1 |
| GO:0035136 | forelimb morphogenesis | Biological process | 2 | 33 | 70 | 13692 | 11.8545454545455 | 0.0122819501813947 | 0.350498115959403 | 1.91073266855532 | MSX2//MSX1 |
| GO:0071383 | cellular response to steroid hormone stimulus | Biological process | 3 | 94 | 70 | 13692 | 6.24255319148936 | 0.0123122057157272 | 0.350498115959403 | 1.90966413680403 | MSX2//MMP2//AQP1 |
| GO:0071310 | cellular response to organic substance | Biological process | 10 | 877 | 70 | 13692 | 2.230330672748 | 0.0132519929735784 | 0.374742130166883 | 1.87771880305999 | MMP2//KRT18//EPOR//CASQ2//MSX1//AQP1//SLC5A5//XCL1//MSX2//FOXJ1 |
| GO:0031099 | regeneration | Biological process | 4 | 180 | 70 | 13692 | 4.34666666666667 | 0.0135091018286165 | 0.374742130166883 | 1.86937352468948 | MMP2//HTR2C//LRP2//SLCO1A5 |
| GO:0051239 | regulation of multicellular organismal process | Biological process | 16 | 1747 | 70 | 13692 | 1.79141385231826 | 0.0135644645576942 | 0.374742130166883 | 1.86759734486061 | CASQ2//XCL1//FOXJ1//HES1//AQP1//TRPV4//HTR2C//MEFV//SCX//MESP1//DIO3//MSX1//MSX2//LMX1A//MMP2//ACE |
| GO:0051253 | negative regulation of RNA metabolic process | Biological process | 9 | 749 | 70 | 13692 | 2.35033377837116 | 0.0136647239175092 | 0.374742130166883 | 1.86439913838139 | MSX2//HES1//MSX1//NR2F2//FOXJ1//XCL1//MESP1//MDFIC//SCX |
| GO:0008593 | regulation of Notch signaling pathway | Biological process | 2 | 35 | 70 | 13692 | 11.1771428571429 | 0.0137499981423305 | 0.374742130166883 | 1.86169736050831 | MESP1//HES1 |
| GO:0060324 | face development | Biological process | 2 | 35 | 70 | 13692 | 11.1771428571429 | 0.0137499981423305 | 0.374742130166883 | 1.86169736050831 | MMP2//MSX1 |
| GO:0021537 | telencephalon development | Biological process | 4 | 181 | 70 | 13692 | 4.32265193370166 | 0.0137621995795776 | 0.374742130166883 | 1.86131214830035 | MDK//LMX1A//AQP1//HES1 |
| GO:0055082 | cellular chemical homeostasis | Biological process | 8 | 624 | 70 | 13692 | 2.50769230769231 | 0.0139580546784177 | 0.375859365417381 | 1.85517510472851 | CASQ2//TRPV4//SCARA5//EPOR//TRHR//DHRS7C//HTR2C//XCL1 |
| GO:0051240 | positive regulation of multicellular organismal process | Biological process | 7 | 502 | 70 | 13692 | 2.72749003984064 | 0.0139746981211298 | 0.375859365417381 | 1.85465756489009 | XCL1//AQP1//SCX//DIO3//HTR2C//MSX2//MESP1 |
| GO:0050801 | ion homeostasis | Biological process | 8 | 626 | 70 | 13692 | 2.49968051118211 | 0.0142070290650451 | 0.377169017765683 | 1.84749673104899 | CASQ2//TRPV4//SCARA5//EPOR//TRHR//DHRS7C//HTR2C//XCL1 |
| GO:0042493 | response to drug | Biological process | 7 | 505 | 70 | 13692 | 2.71128712871287 | 0.0143995874017941 | 0.377169017765683 | 1.8416499517722 | HTR2C//AQP1//COX8B//LRP2//MDK//MMP2//CDH3 |
| GO:0001709 | cell fate determination | Biological process | 2 | 36 | 70 | 13692 | 10.8666666666667 | 0.014511194451048 | 0.377169017765683 | 1.83829683827564 | HES1//MESP1 |
| GO:0045168 | cell-cell signaling involved in cell fate commitment | Biological process | 2 | 36 | 70 | 13692 | 10.8666666666667 | 0.014511194451048 | 0.377169017765683 | 1.83829683827564 | MESP1//HES1 |
| GO:0090130 | tissue migration | Biological process | 2 | 36 | 70 | 13692 | 10.8666666666667 | 0.014511194451048 | 0.377169017765683 | 1.83829683827564 | MESP1//AQP1 |
| GO:0061448 | connective tissue development | Biological process | 4 | 184 | 70 | 13692 | 4.25217391304348 | 0.0145395903290147 | 0.377169017765683 | 1.8374478300901 | COL11A1//SCX//MSX2//MSX1 |
| GO:0046942 | carboxylic acid transport | Biological process | 4 | 185 | 70 | 13692 | 4.22918918918919 | 0.0148047914477307 | 0.377919699846716 | 1.82959770605501 | HTR2C//SLCO1A5//FOLR1//ACE |
| GO:0006865 | amino acid transport | Biological process | 3 | 101 | 70 | 13692 | 5.80990099009901 | 0.0149253333617323 | 0.377919699846716 | 1.82607595999464 | HTR2C//FOLR1//SLCO1A5 |
| GO:0010628 | positive regulation of gene expression | Biological process | 11 | 1031 | 70 | 13692 | 2.08690591658584 | 0.0149630619947197 | 0.377919699846716 | 1.82497952469768 | HES1//MDK//NR2F2//MESP1//MDFIC//SCX//PLAGL1//MSX1//FOXJ1//TEAD2//ITGA8 |
| GO:0009790 | embryo development | Biological process | 10 | 894 | 70 | 13692 | 2.18791946308725 | 0.0149998967507033 | 0.377919699846716 | 1.82391173032793 | HES1//MSX1//SCX//TEAD2//FOXJ1//MESP1//MSX2//COL11A1//ITGA8//EPOR |
| GO:0045596 | negative regulation of cell differentiation | Biological process | 6 | 393 | 70 | 13692 | 2.98625954198473 | 0.0150711379024974 | 0.377919699846716 | 1.82185395630387 | FOXJ1//HES1//MESP1//MSX2//LMX1A//MSX1 |
| GO:0007043 | cell-cell junction assembly | Biological process | 2 | 37 | 70 | 13692 | 10.572972972973 | 0.0152902405552047 | 0.377919699846716 | 1.81558568195353 | CLDN19//TRPV4 |
| GO:0043433 | negative regulation of sequence-specific DNA binding transcription factor activity | Biological process | 3 | 102 | 70 | 13692 | 5.75294117647059 | 0.0153222756574224 | 0.377919699846716 | 1.81467672869387 | FOXJ1//MSX2//XCL1 |
| GO:0060348 | bone development | Biological process | 3 | 102 | 70 | 13692 | 5.75294117647059 | 0.0153222756574224 | 0.377919699846716 | 1.81467672869387 | SCX//MSX2//MSX1 |
| GO:0015849 | organic acid transport | Biological process | 4 | 187 | 70 | 13692 | 4.18395721925134 | 0.0153443673751632 | 0.377919699846716 | 1.81405101216141 | HTR2C//SLCO1A5//FOLR1//ACE |
| GO:0032526 | response to retinoic acid | Biological process | 3 | 103 | 70 | 13692 | 5.69708737864078 | 0.0157251606962677 | 0.382995024957987 | 1.80340490778122 | AQP1//LRP2//MMP2 |
| GO:0042129 | regulation of T cell proliferation | Biological process | 3 | 103 | 70 | 13692 | 5.69708737864078 | 0.0157251606962677 | 0.382995024957987 | 1.80340490778122 | HES1//XCL1//FOXJ1 |
| GO:0001947 | heart looping | Biological process | 2 | 38 | 70 | 13692 | 10.2947368421053 | 0.0160869395226883 | 0.386510638549762 | 1.79352657086884 | FOXJ1//MESP1 |
| GO:0007566 | embryo implantation | Biological process | 2 | 38 | 70 | 13692 | 10.2947368421053 | 0.0160869395226883 | 0.386510638549762 | 1.79352657086884 | MMP2//SOSTDC1 |
| GO:0019233 | sensory perception of pain | Biological process | 3 | 104 | 70 | 13692 | 5.64230769230769 | 0.0161339979139157 | 0.386510638549762 | 1.79225800354621 | HTR2C//ACE//AQP1 |
| GO:0001666 | response to hypoxia | Biological process | 5 | 291 | 70 | 13692 | 3.36082474226804 | 0.0164908842694009 | 0.392913242592682 | 1.78275605611868 | AQP1//ACE//DIO3//CLDN3//MMP2 |
| GO:0010171 | body morphogenesis | Biological process | 2 | 39 | 70 | 13692 | 10.0307692307692 | 0.0169010959178933 | 0.398357013462603 | 1.77208513351282 | MMP2//MSX1 |
| GO:0035137 | hindlimb morphogenesis | Biological process | 2 | 39 | 70 | 13692 | 10.0307692307692 | 0.0169010959178933 | 0.398357013462603 | 1.77208513351282 | MSX2//MSX1 |
| GO:0036293 | response to decreased oxygen levels | Biological process | 5 | 294 | 70 | 13692 | 3.3265306122449 | 0.017163533249975 | 0.402379303571606 | 1.76539330430695 | ACE//DIO3//CLDN3//MMP2//AQP1 |
| GO:0043583 | ear development | Biological process | 4 | 194 | 70 | 13692 | 4.03298969072165 | 0.0173303314057418 | 0.404128579163681 | 1.76119313224807 | COL11A1//ITGA8//MSX1//HES1 |
| GO:0009952 | anterior/posterior pattern specification | Biological process | 4 | 195 | 70 | 13692 | 4.01230769230769 | 0.0176265518458427 | 0.408861393080288 | 1.75383263726322 | HES1//MSX2//MSX1//NR2F2 |
| GO:0061061 | muscle structure development | Biological process | 6 | 408 | 70 | 13692 | 2.87647058823529 | 0.0178074904146654 | 0.409227703190059 | 1.74939728075338 | MSX1//NR2F2//MESP1//COL11A1//HES1//ITGA8 |
| GO:0042471 | ear morphogenesis | Biological process | 3 | 108 | 70 | 13692 | 5.43333333333333 | 0.0178290354263917 | 0.409227703190059 | 1.74887215207574 | COL11A1//ITGA8//MSX1 |
| GO:0042130 | negative regulation of T cell proliferation | Biological process | 2 | 41 | 70 | 13692 | 9.54146341463415 | 0.0185810066728577 | 0.417739144891324 | 1.73093076070707 | XCL1//FOXJ1 |
| GO:0046068 | cGMP metabolic process | Biological process | 2 | 41 | 70 | 13692 | 9.54146341463415 | 0.0185810066728577 | 0.417739144891324 | 1.73093076070707 | HTR2C//AQP1 |
| GO:0061371 | determination of heart left/right asymmetry | Biological process | 2 | 41 | 70 | 13692 | 9.54146341463415 | 0.0185810066728577 | 0.417739144891324 | 1.73093076070707 | FOXJ1//MESP1 |
| GO:0071695 | anatomical structure maturation | Biological process | 2 | 41 | 70 | 13692 | 9.54146341463415 | 0.0185810066728577 | 0.417739144891324 | 1.73093076070707 | MMP2//MSX2 |
| GO:0010557 | positive regulation of macromolecule biosynthetic process | Biological process | 11 | 1068 | 70 | 13692 | 2.01460674157303 | 0.0190046859422568 | 0.425084403932928 | 1.72113930283919 | HES1//MDK//NR2F2//MESP1//MDFIC//SCX//PLAGL1//MSX1//FOXJ1//TEAD2//ITGA8 |
| GO:0048732 | gland development | Biological process | 5 | 303 | 70 | 13692 | 3.22772277227723 | 0.0192906300736317 | 0.429289960623357 | 1.71465358712892 | HES1//MSX1//MDK//MSX2//MMP2 |
| GO:0014031 | mesenchymal cell development | Biological process | 3 | 112 | 70 | 13692 | 5.23928571428571 | 0.0196199022962129 | 0.434412382154532 | 1.70730315966358 | MSX1//MSX2//HES1 |
| GO:0040012 | regulation of locomotion | Biological process | 6 | 418 | 70 | 13692 | 2.80765550239234 | 0.0198117163529955 | 0.436455097947398 | 1.70307789849751 | XCL1//NR2F2//AQP1//ACE//ENPP2//HTR2C |
| GO:0045935 | positive regulation of nucleobase-containing compound metabolic process | Biological process | 11 | 1077 | 70 | 13692 | 1.99777158774373 | 0.0201013807196625 | 0.439014168048309 | 1.6967741108215 | HES1//MDK//NR2F2//MESP1//MDFIC//SCX//PLAGL1//MSX1//FOXJ1//TEAD2//ITGA8 |
| GO:2000113 | negative regulation of cellular macromolecule biosynthetic process | Biological process | 9 | 800 | 70 | 13692 | 2.2005 | 0.0201697661234104 | 0.439014168048309 | 1.69529913757807 | MSX2//HES1//MSX1//NR2F2//FOXJ1//XCL1//MESP1//MDFIC//SCX |
| GO:0003151 | outflow tract morphogenesis | Biological process | 2 | 43 | 70 | 13692 | 9.09767441860465 | 0.0203284388945727 | 0.439014168048309 | 1.69189597136218 | MSX2//HES1 |
| GO:0042733 | embryonic digit morphogenesis | Biological process | 2 | 43 | 70 | 13692 | 9.09767441860465 | 0.0203284388945727 | 0.439014168048309 | 1.69189597136218 | MSX2//MSX1 |
| GO:0034330 | cell junction organization | Biological process | 3 | 114 | 70 | 13692 | 5.14736842105263 | 0.0205513757838239 | 0.441653095275902 | 1.68715909956293 | TRPV4//CLDN19//CLDN1 |
| GO:0007018 | microtubule-based movement | Biological process | 3 | 115 | 70 | 13692 | 5.10260869565217 | 0.0210261352442734 | 0.447399900733327 | 1.67724054644499 | RSPH9//CCDC40//DNAH1 |
| GO:0045944 | positive regulation of transcription from RNA polymerase II promoter | Biological process | 8 | 674 | 70 | 13692 | 2.32166172106825 | 0.0212116824899553 | 0.447399900733327 | 1.67342488230269 | ITGA8//PLAGL1//HES1//MSX1//FOXJ1//TEAD2//MESP1//SCX |
| GO:0010629 | negative regulation of gene expression | Biological process | 9 | 807 | 70 | 13692 | 2.1814126394052 | 0.0212135887333637 | 0.447399900733327 | 1.67338585504511 | MSX2//HES1//MSX1//NR2F2//FOXJ1//XCL1//MESP1//MDFIC//SCX |
| GO:0060350 | endochondral bone morphogenesis | Biological process | 2 | 44 | 70 | 13692 | 8.89090909090909 | 0.0212270025895374 | 0.447399900733327 | 1.67311132711184 | SCX//MSX2 |
| GO:0010951 | negative regulation of endopeptidase activity | Biological process | 3 | 116 | 70 | 13692 | 5.05862068965517 | 0.0215069132099554 | 0.45113065795428 | 1.66742191746386 | AQP1//LRP2//SERPINB1A |
| GO:0009914 | hormone transport | Biological process | 4 | 208 | 70 | 13692 | 3.76153846153846 | 0.021768001255798 | 0.454432940501993 | 1.66218144617381 | TRPV4//LRP2//AQP1//SLCO1A5 |
| GO:0007155 | cell adhesion | Biological process | 8 | 679 | 70 | 13692 | 2.30456553755523 | 0.0220619085021218 | 0.457877408506553 | 1.6563569209036 | COL11A1//MMP2//CDH3//CLDN1//CLDN3//HES1//EMB//CTNNAL1 |
| GO:0045446 | endothelial cell differentiation | Biological process | 2 | 45 | 70 | 13692 | 8.69333333333333 | 0.0221418819807001 | 0.457877408506553 | 1.65478546841227 | NR2F2//MESP1 |
| GO:0045934 | negative regulation of nucleobase-containing compound metabolic process | Biological process | 9 | 815 | 70 | 13692 | 2.16 | 0.0224541134872271 | 0.460495027446162 | 1.64870408668034 | MSX2//HES1//MSX1//NR2F2//FOXJ1//XCL1//MESP1//MDFIC//SCX |
| GO:0051173 | positive regulation of nitrogen compound metabolic process | Biological process | 11 | 1096 | 70 | 13692 | 1.96313868613139 | 0.0225715872991447 | 0.460495027446162 | 1.64643789900729 | HES1//MDK//NR2F2//MESP1//MDFIC//SCX//PLAGL1//MSX1//FOXJ1//TEAD2//ITGA8 |
| GO:0022610 | biological adhesion | Biological process | 8 | 682 | 70 | 13692 | 2.29442815249267 | 0.0225835836909044 | 0.460495027446162 | 1.64620714063555 | COL11A1//HES1//EMB//CTNNAL1//MMP2//CDH3//CLDN1//CLDN3 |
| GO:0070482 | response to oxygen levels | Biological process | 5 | 317 | 70 | 13692 | 3.08517350157729 | 0.0229340663317812 | 0.465476605548744 | 1.63951893572424 | ACE//DIO3//CLDN3//MMP2//AQP1 |
| GO:0009611 | response to wounding | Biological process | 8 | 688 | 70 | 13692 | 2.27441860465116 | 0.0236532333186125 | 0.473349644941658 | 1.62610948425168 | MEFV//MMP2//MSX2//AQP1//CDH3//XCL1//ACE//MDK |
| GO:0050880 | regulation of blood vessel size | Biological process | 3 | 121 | 70 | 13692 | 4.8495867768595 | 0.0240011266182244 | 0.473349644941658 | 1.6197683719303 | ACE//MMP2//HTR2C |
| GO:0003279 | cardiac septum development | Biological process | 2 | 47 | 70 | 13692 | 8.32340425531915 | 0.0240198483469638 | 0.473349644941658 | 1.61942973890999 | MSX2//HES1 |
| GO:0021983 | pituitary gland development | Biological process | 2 | 47 | 70 | 13692 | 8.32340425531915 | 0.0240198483469638 | 0.473349644941658 | 1.61942973890999 | HES1//MSX1 |
| GO:0032963 | collagen metabolic process | Biological process | 2 | 47 | 70 | 13692 | 8.32340425531915 | 0.0240198483469638 | 0.473349644941658 | 1.61942973890999 | MMP2//SCX |
| GO:0060322 | head development | Biological process | 2 | 47 | 70 | 13692 | 8.32340425531915 | 0.0240198483469638 | 0.473349644941658 | 1.61942973890999 | MMP2//MSX1 |
| GO:0010558 | negative regulation of macromolecule biosynthetic process | Biological process | 9 | 825 | 70 | 13692 | 2.13381818181818 | 0.0240777761911473 | 0.473349644941658 | 1.6183836267378 | MSX2//HES1//MSX1//NR2F2//FOXJ1//XCL1//MESP1//MDFIC//SCX |
| GO:0035295 | tube development | Biological process | 6 | 438 | 70 | 13692 | 2.67945205479452 | 0.0242736061218635 | 0.475069148385043 | 1.61486569946171 | TEAD2//FOXJ1//MESP1//HES1//MSX2//AQP1 |
| GO:0035150 | regulation of tube size | Biological process | 3 | 122 | 70 | 13692 | 4.80983606557377 | 0.0245180359039569 | 0.475606501782952 | 1.61051432331506 | ACE//MMP2//HTR2C |
| GO:0048762 | mesenchymal cell differentiation | Biological process | 3 | 122 | 70 | 13692 | 4.80983606557377 | 0.0245180359039569 | 0.475606501782952 | 1.61051432331506 | MSX1//MSX2//HES1 |
| GO:0001568 | blood vessel development | Biological process | 6 | 443 | 70 | 13692 | 2.64920993227991 | 0.0254867904111434 | 0.487384797079781 | 1.59368485242772 | MMP2//TEAD2//AQP1//NR2F2//HES1//MESP1 |
| GO:0010466 | negative regulation of peptidase activity | Biological process | 3 | 124 | 70 | 13692 | 4.73225806451613 | 0.0255699140803717 | 0.487384797079781 | 1.59227073128079 | LRP2//SERPINB1A//AQP1 |
| GO:0033189 | response to vitamin A | Biological process | 3 | 124 | 70 | 13692 | 4.73225806451613 | 0.0255699140803717 | 0.487384797079781 | 1.59227073128079 | LRP2//MMP2//AQP1 |
| GO:0035270 | endocrine system development | Biological process | 3 | 124 | 70 | 13692 | 4.73225806451613 | 0.0255699140803717 | 0.487384797079781 | 1.59227073128079 | HES1//MSX1//MDK |
| GO:0003158 | endothelium development | Biological process | 2 | 49 | 70 | 13692 | 7.98367346938775 | 0.025960873125687 | 0.490994251947592 | 1.58568070527339 | MESP1//NR2F2 |
| GO:0008285 | negative regulation of cell proliferation | Biological process | 6 | 445 | 70 | 13692 | 2.63730337078652 | 0.0259832724570806 | 0.490994251947592 | 1.58530615270288 | NR2F2//FOXJ1//HES1//XCL1//MSX2//MSX1 |
| GO:0030902 | hindbrain development | Biological process | 3 | 125 | 70 | 13692 | 4.6944 | 0.0261048789921187 | 0.491175062237976 | 1.58327831559929 | LMX1A//HES1//MDK |
| GO:0042592 | homeostatic process | Biological process | 11 | 1125 | 70 | 13692 | 1.91253333333333 | 0.0267675733741102 | 0.49860287835107 | 1.57239099818183 | SCX//AQP1//CASQ2//TRPV4//SCARA5//EPOR//TRHR//DHRS7C//PYGL//HTR2C//XCL1 |
| GO:0019229 | regulation of vasoconstriction | Biological process | 2 | 50 | 70 | 13692 | 7.824 | 0.0269545807867709 | 0.49860287835107 | 1.56936741818177 | MMP2//HTR2C |
| GO:0044259 | multicellular organismal macromolecule metabolic process | Biological process | 2 | 50 | 70 | 13692 | 7.824 | 0.0269545807867709 | 0.49860287835107 | 1.56936741818177 | MMP2//SCX |
| GO:0048645 | organ formation | Biological process | 2 | 50 | 70 | 13692 | 7.824 | 0.0269545807867709 | 0.49860287835107 | 1.56936741818177 | MESP1//HES1 |
| GO:0001501 | skeletal system development | Biological process | 5 | 333 | 70 | 13692 | 2.93693693693694 | 0.0276154399130814 | 0.508681044449365 | 1.55884803408789 | COL11A1//SCX//MSX2//MMP2//MSX1 |
| GO:0048863 | stem cell differentiation | Biological process | 3 | 128 | 70 | 13692 | 4.584375 | 0.0277458430751008 | 0.508944669628627 | 1.55680207433508 | HES1//MSX2//MSX1 |
| GO:0042098 | T cell proliferation | Biological process | 3 | 129 | 70 | 13692 | 4.54883720930233 | 0.0283048420510077 | 0.517035114798407 | 1.54813926425818 | HES1//XCL1//FOXJ1 |
| GO:0007423 | sensory organ development | Biological process | 6 | 456 | 70 | 13692 | 2.57368421052632 | 0.0288300941461486 | 0.522967820369736 | 1.54015393939861 | FSCN2//COL11A1//ITGA8//MSX1//HES1//AQP1 |
| GO:0032945 | negative regulation of mononuclear cell proliferation | Biological process | 2 | 52 | 70 | 13692 | 7.52307692307692 | 0.0289874955177568 | 0.522967820369736 | 1.53778930550751 | FOXJ1//XCL1 |
| GO:0050672 | negative regulation of lymphocyte proliferation | Biological process | 2 | 52 | 70 | 13692 | 7.52307692307692 | 0.0289874955177568 | 0.522967820369736 | 1.53778930550751 | FOXJ1//XCL1 |
| GO:0060562 | epithelial tube morphogenesis | Biological process | 4 | 230 | 70 | 13692 | 3.40173913043478 | 0.0300412215060586 | 0.539757029026889 | 1.52228241246163 | TEAD2//FOXJ1//MESP1//MSX2 |
| GO:0003208 | cardiac ventricle morphogenesis | Biological process | 2 | 54 | 70 | 13692 | 7.24444444444444 | 0.0310799039313289 | 0.553266021476964 | 1.50752033228291 | MESP1//COL11A1 |
| GO:0070664 | negative regulation of leukocyte proliferation | Biological process | 2 | 54 | 70 | 13692 | 7.24444444444444 | 0.0310799039313289 | 0.553266021476964 | 1.50752033228291 | FOXJ1//XCL1 |
| GO:0001944 | vasculature development | Biological process | 6 | 465 | 70 | 13692 | 2.52387096774194 | 0.0313080003230662 | 0.553266021476964 | 1.50434467037068 | MMP2//TEAD2//AQP1//NR2F2//HES1//MESP1 |
| GO:0031328 | positive regulation of cellular biosynthetic process | Biological process | 11 | 1153 | 70 | 13692 | 1.86608846487424 | 0.0313369708888107 | 0.553266021476964 | 1.50394298584616 | HES1//MDK//NR2F2//MESP1//MDFIC//SCX//PLAGL1//MSX1//FOXJ1//TEAD2//ITGA8 |
| GO:0031327 | negative regulation of cellular biosynthetic process | Biological process | 9 | 865 | 70 | 13692 | 2.03514450867052 | 0.031424096566552 | 0.553266021476964 | 1.50273719929996 | MSX2//HES1//MSX1//NR2F2//FOXJ1//XCL1//MESP1//MDFIC//SCX |
| GO:0032321 | positive regulation of Rho GTPase activity | Biological process | 2 | 55 | 70 | 13692 | 7.11272727272727 | 0.0321479837791249 | 0.563747043550734 | 1.49284625947563 | FOXJ1//ARHGAP8 |
| GO:2000027 | regulation of organ morphogenesis | Biological process | 3 | 136 | 70 | 13692 | 4.31470588235294 | 0.0323854742305122 | 0.565649079787113 | 1.48964973905515 | HES1//MESP1//MSX1 |
| GO:0002009 | morphogenesis of an epithelium | Biological process | 5 | 348 | 70 | 13692 | 2.81034482758621 | 0.0325234778150705 | 0.565805264846306 | 1.48780302042311 | TEAD2//FOXJ1//MESP1//MSX2//MMP2 |
| GO:0009628 | response to abiotic stimulus | Biological process | 9 | 873 | 70 | 13692 | 2.01649484536082 | 0.0330643133498896 | 0.570859248375901 | 1.48064049190603 | ACE//DIO3//CLDN3//MMP2//TRPV4//AQP1//LRP2//SCARA5//COL11A1 |
| GO:0040007 | growth | Biological process | 8 | 734 | 70 | 13692 | 2.13188010899183 | 0.033074418131268 | 0.570859248375901 | 1.48050778749125 | MMP2//LMX1A//IGFBPL1//MESP1//MSX2//MSX1//SOCS6//DIO3 |
| GO:0003018 | vascular process in circulatory system | Biological process | 3 | 138 | 70 | 13692 | 4.25217391304348 | 0.0336050606590477 | 0.577743474232412 | 1.47359531633623 | ACE//MMP2//HTR2C |
| GO:0001707 | mesoderm formation | Biological process | 2 | 57 | 70 | 13692 | 6.86315789473684 | 0.0343270375132396 | 0.587850517414228 | 1.46436367537322 | MESP1//SCX |
| GO:0048545 | response to steroid hormone stimulus | Biological process | 6 | 477 | 70 | 13692 | 2.46037735849057 | 0.0348246968876083 | 0.592101165849746 | 1.45811265496465 | NR2F2//FOXJ1//AQP1//MMP2//MDK//MSX2 |
| GO:0030198 | extracellular matrix organization | Biological process | 3 | 140 | 70 | 13692 | 4.19142857142857 | 0.0348483975085494 | 0.592101165849746 | 1.45781718798765 | COL11A1//SCX//ITGA8 |
| GO:0009890 | negative regulation of biosynthetic process | Biological process | 9 | 882 | 70 | 13692 | 1.99591836734694 | 0.0349804292780758 | 0.592101165849746 | 1.45617486518451 | MSX2//HES1//MSX1//NR2F2//FOXJ1//XCL1//MESP1//MDFIC//SCX |
| GO:0006357 | regulation of transcription from RNA polymerase II promoter | Biological process | 10 | 1027 | 70 | 13692 | 1.90457643622201 | 0.035206296968406 | 0.593632330421123 | 1.4533796520318 | MSX2//HES1//MSX1//NR2F2//FOXJ1//PLAGL1//TEAD2//MESP1//SCX//ITGA8 |
| GO:0045665 | negative regulation of neuron differentiation | Biological process | 2 | 58 | 70 | 13692 | 6.7448275862069 | 0.0354376724835964 | 0.593663043843361 | 1.45053480994314 | HES1//LMX1A |
| GO:0043062 | extracellular structure organization | Biological process | 3 | 141 | 70 | 13692 | 4.16170212765957 | 0.0354789501566972 | 0.593663043843361 | 1.45002923968301 | ITGA8//COL11A1//SCX |
| GO:0009891 | positive regulation of biosynthetic process | Biological process | 11 | 1178 | 70 | 13692 | 1.82648556876061 | 0.0358726786776163 | 0.597968909972129 | 1.44523619256094 | HES1//MDK//NR2F2//MESP1//MDFIC//SCX//PLAGL1//MSX1//FOXJ1//TEAD2//ITGA8 |
| GO:0014070 | response to organic cyclic compound | Biological process | 5 | 358 | 70 | 13692 | 2.73184357541899 | 0.036082005731612 | 0.599179973967375 | 1.4427093288363 | MMP2//HTR2C//CASQ2//MSX1//FOXJ1 |
| GO:0022600 | digestive system process | Biological process | 2 | 59 | 70 | 13692 | 6.63050847457627 | 0.0365621557702918 | 0.60129138308823 | 1.43696820540155 | AQP1//SLCO1A5 |
| GO:0050777 | negative regulation of immune response | Biological process | 2 | 59 | 70 | 13692 | 6.63050847457627 | 0.0365621557702918 | 0.60129138308823 | 1.43696820540155 | FOXJ1//XCL1 |
| GO:0070887 | cellular response to chemical stimulus | Biological process | 11 | 1182 | 70 | 13692 | 1.82030456852792 | 0.0366397991845411 | 0.60129138308823 | 1.43604691527942 | XCL1//PON3//MMP2//KRT18//EPOR//AQP1//CASQ2//MSX1//SLC5A5//MSX2//FOXJ1 |
| GO:0060485 | mesenchyme development | Biological process | 3 | 143 | 70 | 13692 | 4.1034965034965 | 0.0367577761559411 | 0.60129138308823 | 1.43465077129078 | MSX1//MSX2//HES1 |
| GO:0032844 | regulation of homeostatic process | Biological process | 4 | 247 | 70 | 13692 | 3.16761133603239 | 0.0375548814793898 | 0.609882686696021 | 1.42533360428498 | DHRS7C//CASQ2//HTR2C//XCL1 |
| GO:0065009 | regulation of molecular function | Biological process | 13 | 1493 | 70 | 13692 | 1.70314802411253 | 0.0376982803783048 | 0.609882686696021 | 1.42367845985379 | MDFIC//LRP2//SERPINB1A//FOXJ1//ARHGAP8//MSX2//AQP1//HES1//MSX1//XCL1//NR2F2//CASQ2//TRHR |
| GO:0002698 | negative regulation of immune effector process | Biological process | 2 | 60 | 70 | 13692 | 6.52 | 0.0377003211894666 | 0.609882686696021 | 1.4236549497884 | XCL1//FOXJ1 |
| GO:0050670 | regulation of lymphocyte proliferation | Biological process | 3 | 145 | 70 | 13692 | 4.04689655172414 | 0.0380601617173029 | 0.613440253561235 | 1.41952937073152 | HES1//XCL1//FOXJ1 |
| GO:0032944 | regulation of mononuclear cell proliferation | Biological process | 3 | 146 | 70 | 13692 | 4.01917808219178 | 0.0387201624419405 | 0.617127481464534 | 1.41206282937267 | HES1//XCL1//FOXJ1 |
| GO:0045598 | regulation of fat cell differentiation | Biological process | 2 | 61 | 70 | 13692 | 6.41311475409836 | 0.0388520038513256 | 0.617127481464534 | 1.41058657688545 | MSX2//HES1 |
| GO:0048332 | mesoderm morphogenesis | Biological process | 2 | 61 | 70 | 13692 | 6.41311475409836 | 0.0388520038513256 | 0.617127481464534 | 1.41058657688545 | SCX//MESP1 |
| GO:0051153 | regulation of striated muscle cell differentiation | Biological process | 2 | 61 | 70 | 13692 | 6.41311475409836 | 0.0388520038513256 | 0.617127481464534 | 1.41058657688545 | MSX1//MESP1 |
| GO:0051093 | negative regulation of developmental process | Biological process | 6 | 493 | 70 | 13692 | 2.38052738336714 | 0.0399004956995415 | 0.62879822230983 | 1.39902170886868 | FOXJ1//HES1//MESP1//MSX2//LMX1A//MSX1 |
| GO:0021766 | hippocampus development | Biological process | 2 | 62 | 70 | 13692 | 6.30967741935484 | 0.0400170401515608 | 0.62879822230983 | 1.39775503697372 | MDK//LMX1A |
| GO:0030856 | regulation of epithelial cell differentiation | Biological process | 2 | 62 | 70 | 13692 | 6.30967741935484 | 0.0400170401515608 | 0.62879822230983 | 1.39775503697372 | MSX2//FOXJ1 |
| GO:0051924 | regulation of calcium ion transport | Biological process | 3 | 149 | 70 | 13692 | 3.93825503355705 | 0.0407352587354031 | 0.637797765342883 | 1.39002952086553 | DHRS7C//CASQ2//XCL1 |
| GO:0006970 | response to osmotic stress | Biological process | 2 | 63 | 70 | 13692 | 6.20952380952381 | 0.0411952677628269 | 0.640425722951181 | 1.3851526699506 | TRPV4//AQP1 |
| GO:0044236 | multicellular organismal metabolic process | Biological process | 2 | 63 | 70 | 13692 | 6.20952380952381 | 0.0411952677628269 | 0.640425722951181 | 1.3851526699506 | MMP2//SCX |
| GO:0070663 | regulation of leukocyte proliferation | Biological process | 3 | 151 | 70 | 13692 | 3.88609271523179 | 0.0421077797153016 | 0.652298608734566 | 1.37563765772042 | HES1//XCL1//FOXJ1 |
| GO:0050868 | negative regulation of T cell activation | Biological process | 2 | 64 | 70 | 13692 | 6.1125 | 0.0423865256262679 | 0.652801941221406 | 1.37277218058157 | FOXJ1//XCL1 |
| GO:0001655 | urogenital system development | Biological process | 4 | 257 | 70 | 13692 | 3.0443579766537 | 0.0424380824014828 | 0.652801941221406 | 1.37224424826904 | ITGA8//ACE//MMP2//AQP1 |
| GO:0030334 | regulation of cell migration | Biological process | 5 | 375 | 70 | 13692 | 2.608 | 0.0426683485720925 | 0.654049091398789 | 1.36989416566908 | XCL1//NR2F2//AQP1//ACE//ENPP2 |
| GO:0030855 | epithelial cell differentiation | Biological process | 4 | 259 | 70 | 13692 | 3.02084942084942 | 0.0434560651785365 | 0.661250612064119 | 1.36194960034898 | FOXJ1//MESP1//MSX2//NR2F2 |
| GO:0042102 | positive regulation of T cell proliferation | Biological process | 2 | 65 | 70 | 13692 | 6.01846153846154 | 0.0435906539430954 | 0.661250612064119 | 1.36060661568643 | XCL1//HES1 |
| GO:0071407 | cellular response to organic cyclic compound | Biological process | 2 | 65 | 70 | 13692 | 6.01846153846154 | 0.0435906539430954 | 0.661250612064119 | 1.36060661568643 | CASQ2//MSX1 |
| GO:0035556 | intracellular signal transduction | Biological process | 13 | 1527 | 70 | 13692 | 1.66522593320236 | 0.044020572322381 | 0.665469617452822 | 1.35634431537567 | MDFIC//HES1//RHOD//RGL3//RAB19//RAB20//CTNNAL1//CASQ2//HTR2C//TEAD2//ARHGAP8//SOCS6 |
| GO:0071345 | cellular response to cytokine stimulus | Biological process | 4 | 261 | 70 | 13692 | 2.99770114942529 | 0.0444878328221116 | 0.670222196192911 | 1.35175874993464 | KRT18//EPOR//MMP2//XCL1 |
| GO:0048514 | blood vessel morphogenesis | Biological process | 5 | 383 | 70 | 13692 | 2.55352480417755 | 0.0460047184029321 | 0.672130494303553 | 1.33719762329086 | MMP2//TEAD2//AQP1//HES1//NR2F2 |
| GO:0001837 | epithelial to mesenchymal transition | Biological process | 2 | 67 | 70 | 13692 | 5.83880597014925 | 0.0460368889919196 | 0.672130494303553 | 1.33689403211637 | MSX2//MSX1 |
| GO:0042384 | cilium assembly | Biological process | 2 | 67 | 70 | 13692 | 5.83880597014925 | 0.0460368889919196 | 0.672130494303553 | 1.33689403211637 | RSPH9//FOXJ1 |
| GO:0006366 | transcription from RNA polymerase II promoter | Biological process | 10 | 1077 | 70 | 13692 | 1.81615598885794 | 0.0463240551361792 | 0.672130494303553 | 1.33419343014891 | MSX2//HES1//MSX1//NR2F2//FOXJ1//PLAGL1//TEAD2//MESP1//SCX//ITGA8 |
| GO:2000026 | regulation of multicellular organismal development | Biological process | 10 | 1078 | 70 | 13692 | 1.81447124304267 | 0.0465684179921361 | 0.672130494303553 | 1.33190851553422 | FOXJ1//HES1//TRPV4//MESP1//MSX1//MSX2//LMX1A//AQP1//ACE//SCX |
| GO:0007565 | female pregnancy | Biological process | 3 | 158 | 70 | 13692 | 3.71392405063291 | 0.0470934124261907 | 0.672130494303553 | 1.32703983909285 | MMP2//SOSTDC1//EPOR |
| GO:0042692 | muscle cell differentiation | Biological process | 4 | 267 | 70 | 13692 | 2.93033707865169 | 0.0476658076414586 | 0.672130494303553 | 1.32179304396655 | MSX1//MESP1//HES1//ITGA8 |
| GO:0051100 | negative regulation of binding | Biological process | 2 | 69 | 70 | 13692 | 5.6695652173913 | 0.0485327194035193 | 0.672130494303553 | 1.31396537345178 | MSX1//MSX2 |
| GO:0061180 | mammary gland epithelium development | Biological process | 2 | 69 | 70 | 13692 | 5.6695652173913 | 0.0485327194035193 | 0.672130494303553 | 1.31396537345178 | MSX2//MSX1 |
| GO:2000145 | regulation of cell motility | Biological process | 5 | 389 | 70 | 13692 | 2.51413881748072 | 0.0486073287573366 | 0.672130494303553 | 1.31329824516337 | XCL1//NR2F2//AQP1//ACE//ENPP2 |
| GO:0010033 | response to organic substance | Biological process | 14 | 1714 | 70 | 13692 | 1.59766627771295 | 0.0496192734694531 | 0.672130494303553 | 1.30434959900209 | AQP1//MDK//FOXJ1//MMP2//NR2F2//ACE//KRT18//HTR2C//EPOR//CASQ2//MSX1//SLC5A5//XCL1//MSX2 |
| GO:0002643 | regulation of tolerance induction | Biological process | 1 | 10 | 70 | 13692 | 19.56 | 0.0499805066475726 | 0.672130494303553 | 1.301199345786 | FOXJ1 |
| GO:0002710 | negative regulation of T cell mediated immunity | Biological process | 1 | 10 | 70 | 13692 | 19.56 | 0.0499805066475726 | 0.672130494303553 | 1.301199345786 | XCL1 |
| GO:0002719 | negative regulation of cytokine production involved in immune response | Biological process | 1 | 10 | 70 | 13692 | 19.56 | 0.0499805066475726 | 0.672130494303553 | 1.301199345786 | XCL1 |
| GO:0002921 | negative regulation of humoral immune response | Biological process | 1 | 10 | 70 | 13692 | 19.56 | 0.0499805066475726 | 0.672130494303553 | 1.301199345786 | FOXJ1 |
| GO:0002923 | regulation of humoral immune response mediated by circulating immunoglobulin | Biological process | 1 | 10 | 70 | 13692 | 19.56 | 0.0499805066475726 | 0.672130494303553 | 1.301199345786 | FOXJ1 |
| GO:0003148 | outflow tract septum morphogenesis | Biological process | 1 | 10 | 70 | 13692 | 19.56 | 0.0499805066475726 | 0.672130494303553 | 1.301199345786 | MSX2 |
| GO:0003211 | cardiac ventricle formation | Biological process | 1 | 10 | 70 | 13692 | 19.56 | 0.0499805066475726 | 0.672130494303553 | 1.301199345786 | MESP1 |
| GO:0007501 | mesodermal cell fate specification | Biological process | 1 | 10 | 70 | 13692 | 19.56 | 0.0499805066475726 | 0.672130494303553 | 1.301199345786 | MESP1 |
| GO:0015669 | gas transport | Biological process | 1 | 10 | 70 | 13692 | 19.56 | 0.0499805066475726 | 0.672130494303553 | 1.301199345786 | AQP1 |
| GO:0015870 | acetylcholine transport | Biological process | 1 | 10 | 70 | 13692 | 19.56 | 0.0499805066475726 | 0.672130494303553 | 1.301199345786 | HTR2C |
| GO:0021681 | cerebellar granular layer development | Biological process | 1 | 10 | 70 | 13692 | 19.56 | 0.0499805066475726 | 0.672130494303553 | 1.301199345786 | MDK |
| GO:0032692 | negative regulation of interleukin-1 production | Biological process | 1 | 10 | 70 | 13692 | 19.56 | 0.0499805066475726 | 0.672130494303553 | 1.301199345786 | MEFV |
| GO:0032703 | negative regulation of interleukin-2 production | Biological process | 1 | 10 | 70 | 13692 | 19.56 | 0.0499805066475726 | 0.672130494303553 | 1.301199345786 | XCL1 |
| GO:0036037 | CD8-positive, alpha-beta T cell activation | Biological process | 1 | 10 | 70 | 13692 | 19.56 | 0.0499805066475726 | 0.672130494303553 | 1.301199345786 | XCL1 |
| GO:0040034 | regulation of development, heterochronic | Biological process | 1 | 10 | 70 | 13692 | 19.56 | 0.0499805066475726 | 0.672130494303553 | 1.301199345786 | HES1 |
| GO:0046541 | saliva secretion | Biological process | 1 | 10 | 70 | 13692 | 19.56 | 0.0499805066475726 | 0.672130494303553 | 1.301199345786 | AQP1 |
| GO:0048711 | positive regulation of astrocyte differentiation | Biological process | 1 | 10 | 70 | 13692 | 19.56 | 0.0499805066475726 | 0.672130494303553 | 1.301199345786 | HES1 |
| GO:0051458 | corticotropin secretion | Biological process | 1 | 10 | 70 | 13692 | 19.56 | 0.0499805066475726 | 0.672130494303553 | 1.301199345786 | AQP1 |
| GO:0060456 | positive regulation of digestive system process | Biological process | 1 | 10 | 70 | 13692 | 19.56 | 0.0499805066475726 | 0.672130494303553 | 1.301199345786 | AQP1 |
| GO:0060536 | cartilage morphogenesis | Biological process | 1 | 10 | 70 | 13692 | 19.56 | 0.0499805066475726 | 0.672130494303553 | 1.301199345786 | MSX1 |
| GO:0060850 | regulation of transcription involved in cell fate commitment | Biological process | 1 | 10 | 70 | 13692 | 19.56 | 0.0499805066475726 | 0.672130494303553 | 1.301199345786 | NR2F2 |
| GO:0071353 | cellular response to interleukin-4 | Biological process | 1 | 10 | 70 | 13692 | 19.56 | 0.0499805066475726 | 0.672130494303553 | 1.301199345786 | XCL1 |
| GO:0071731 | response to nitric oxide | Biological process | 1 | 10 | 70 | 13692 | 19.56 | 0.0499805066475726 | 0.672130494303553 | 1.301199345786 | AQP1 |
| GO:2000380 | regulation of mesoderm development | Biological process | 1 | 10 | 70 | 13692 | 19.56 | 0.0499805066475726 | 0.672130494303553 | 1.301199345786 | MESP1 |
