# Supplementary material for: Gene Expression in the Hippocampus in a Rat Model of Premenstrual Dysphoric Disorder After Treatment With Baixiangdan Capsules
Source: Front Psychol. 2018 Nov 13;9:2065. doi: 10.3389/fpsyg.2018.02065 (PMC6242977; doi:10.3389/fpsyg.2018.02065)
Supplement: Supplementary file 3 [file Data_Sheet_3.ZIP › Data Analysis Folder/GO Analysis Report/model vs blank (up)/CC_Pvalue_tree.pdf]

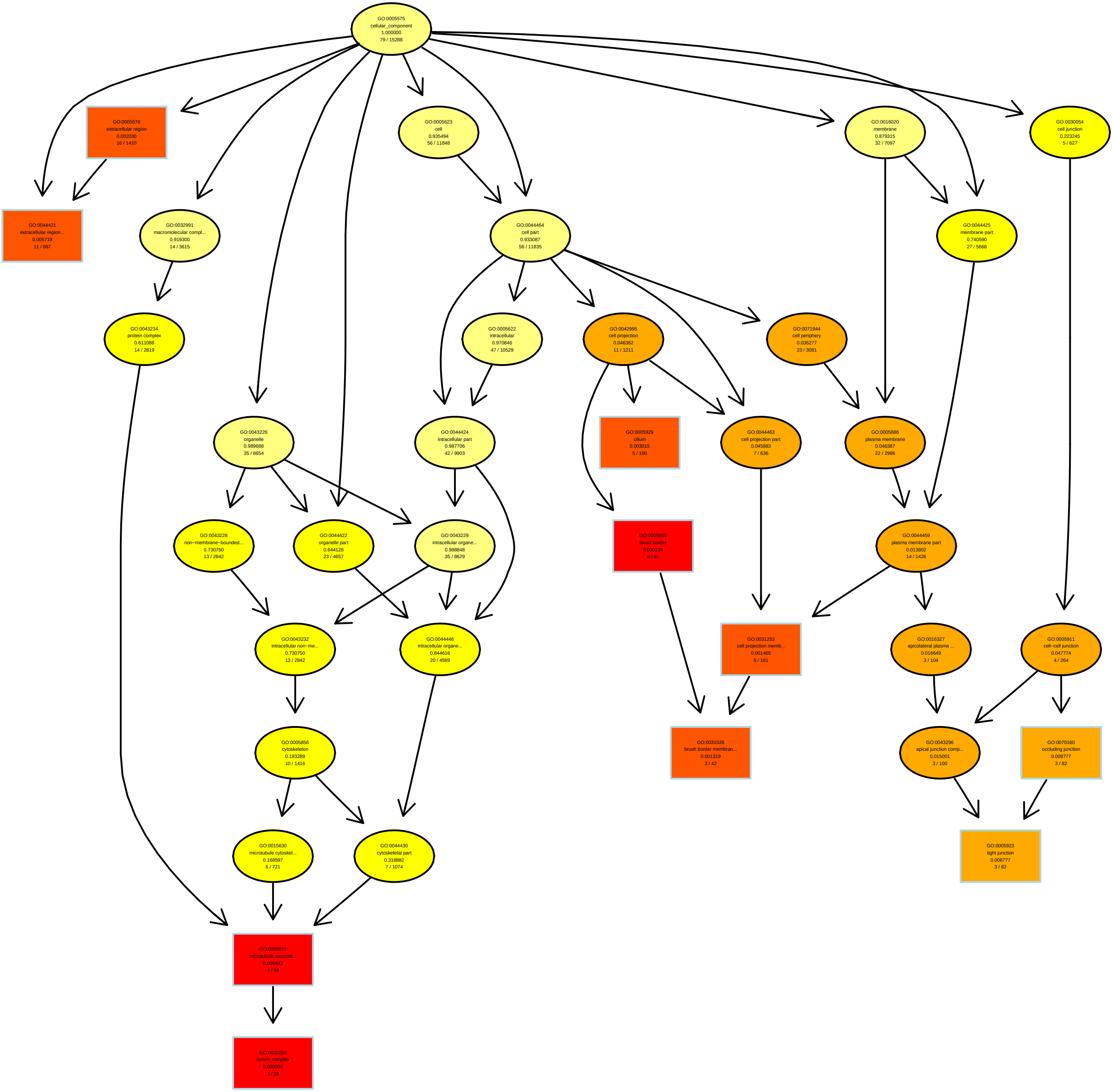
GO:0005575 cellular_component 1.000000 79 / 15288
GO:0005576 extracellular region 0.002030 16 / 1410
GO:0005623 cell 0.935494 56 / 11848
GO:0016020 membrane 0.879315 32 / 7097
GO:0030054 cell junction 0.223245 5 / 627
GO:0044421 extracellular region... 0.005719 11 / 887
GO:0032991 macromolecular compl... 0.919300 14 / 3615
GO:0044464 cell part 0.933087 56 / 11835
GO:0044425 membrane part 0.740590 27 / 5668
GO:0043234 protein complex 0.611088 14 / 2819
GO:0005622 intracellular 0.970846 47 / 10529
GO:0042995 cell projection 0.046382 11 / 1211
GO:0071944 cell periphery 0.036277 23 / 3081
GO:0043226 organelle 0.989688 35 / 8654
GO:0044424 intracellular part 0.987706 42 / 9903
GO:0005929 cilium 0.003015 5 / 190
GO:0044463 cell projection part 0.045883 7 / 636
GO:0005886 plasma membrane 0.046387 22 / 2986
GO:0043228 non−membrane−bounded... 0.730750 13 / 2842
GO:0044422 organelle part 0.644128 23 / 4657
GO:0043229 intracellular organe... 0.988848 35 / 8629
GO:0005903 brush border 0.000276 4 / 61
GO:0044459 plasma membrane part 0.013802 14 / 1426
GO:0043232 intracellular non−me... 0.730750 13 / 2842
GO:0044446 intracellular organe... 0.844616 20 / 4569
GO:0031253 cell projection memb... 0.001465 5 / 161
GO:0016327 apicolateral plasma ... 0.016649 3 / 104
GO:0005911 cell−cell junction 0.047774 4 / 264
GO:0005856 cytoskeleton 0.193289 10 / 1416
GO:0031526 brush border membran... 0.001319 3 / 42
GO:0043296 apical junction comp... 0.015001 3 / 100
GO:0070160 occluding junction 0.008777 3 / 82
GO:0015630 microtubule cytoskel... 0.168597 6 / 721
GO:0044430 cytoskeletal part 0.318882 7 / 1074
GO:0005923 tight junction 0.008777 3 / 82
GO:0005875 microtubule associat... 0.000443 4 / 69
GO:0030286 dynein complex 0.000396 3 / 28
