# Supplementary material for: Gene Expression in the Hippocampus in a Rat Model of Premenstrual Dysphoric Disorder After Treatment With Baixiangdan Capsules
Source: Front Psychol. 2018 Nov 13;9:2065. doi: 10.3389/fpsyg.2018.02065 (PMC6242977; doi:10.3389/fpsyg.2018.02065)
Supplement: Supplementary file 3 [file Data_Sheet_3.ZIP › Data Analysis Folder/GO Analysis Report/model vs blank (up)/CC_result(Rat).html]

| GO.ID | Term | Ontology | Count | Pop.Hits | List.Total | Pop.Total | Fold.Enrichment | Pvalue | FDR | Enrichment.Score | GENES |
| --- | --- | --- | --- | --- | --- | --- | --- | --- | --- | --- | --- |
| GO:0005903 | brush border | Cellular component | 4 | 61 | 79 | 15288 | 12.6897696617556 | 0.000275601757594191 | 0.0792100019004052 | 3.5597180171308 | AQP1//LRP2//SLCO1A5//FOLR1 |
| GO:0030286 | dynein complex | Cellular component | 3 | 28 | 79 | 15288 | 20.7341772151899 | 0.000396389686149614 | 0.0792100019004052 | 3.40187765415292 | DYNLRB2//DNAH1//DNALI1 |
| GO:0005875 | microtubule associated complex | Cellular component | 4 | 69 | 79 | 15288 | 11.2184920198129 | 0.000442513977097236 | 0.0792100019004052 | 3.35407300727048 | DYNLRB2//DNAH1//DNALI1//MEFV |
| GO:0031526 | brush border membrane | Cellular component | 3 | 42 | 79 | 15288 | 13.8227848101266 | 0.00131870813960467 | 0.157335106854641 | 2.87985131316088 | AQP1//LRP2//SLCO1A5 |
| GO:0031253 | cell projection membrane | Cellular component | 5 | 161 | 79 | 15288 | 6.00990643918547 | 0.00146494512900038 | 0.157335106854641 | 2.83417864194375 | AQP1//LRP2//SLCO1A5//TRPV4//ITGA8 |
| GO:0005576 | extracellular region | Cellular component | 16 | 1410 | 79 | 15288 | 2.19596014004848 | 0.00203039764218794 | 0.181720588975821 | 2.69241889957628 | COL11A1//MMP2//ACE//TTR//LRP2//ENPP2//XCL1//SOSTDC1//SERPINB1A//PON3//AQP1//MDK//WFDC2//SCGB1C1//IGFBPL1//RGD1563000 |
| GO:0005929 | cilium | Cellular component | 5 | 190 | 79 | 15288 | 5.09260493004664 | 0.00301536628879128 | 0.23132167101156 | 2.52065992480457 | DNAH1//TRPV4//CCDC40//EFHC1//PACRG |
| GO:0044421 | extracellular region part | Cellular component | 11 | 887 | 79 | 15288 | 2.3998972500107 | 0.00571942975347569 | 0.383916722202056 | 2.24264726967978 | COL11A1//MMP2//ACE//TTR//LRP2//ENPP2//XCL1//SOSTDC1//SERPINB1A//PON3//AQP1 |
| GO:0005923 | tight junction | Cellular component | 3 | 82 | 79 | 15288 | 7.07996295152825 | 0.00877652032193947 | 0.47129914128815 | 2.05667763722818 | CLDN1//CLDN3//CLDN19 |
| GO:0070160 | occluding junction | Cellular component | 3 | 82 | 79 | 15288 | 7.07996295152825 | 0.00877652032193947 | 0.47129914128815 | 2.05667763722818 | CLDN1//CLDN3//CLDN19 |
| GO:0005615 | extracellular space | Cellular component | 9 | 707 | 79 | 15288 | 2.46346659982454 | 0.0105122106540093 | 0.502955608713206 | 1.97830594487741 | ACE//TTR//LRP2//MMP2//ENPP2//XCL1//SOSTDC1//SERPINB1A//PON3 |
| GO:0005667 | transcription factor complex | Cellular component | 5 | 261 | 79 | 15288 | 3.70726029390368 | 0.0112835498508523 | 0.502955608713206 | 1.94755424803869 | MSX2//MSX1//FOXJ1//TEAD2//SCX |
| GO:0016323 | basolateral plasma membrane | Cellular component | 5 | 273 | 79 | 15288 | 3.54430379746835 | 0.0134942834840125 | 0.502955608713206 | 1.86985017055177 | TRPV4//AQP1//SLC16A8//SLC4A2//CLDN19 |
| GO:0044459 | plasma membrane part | Cellular component | 14 | 1426 | 79 | 15288 | 1.89990590658121 | 0.0138016415459764 | 0.502955608713206 | 1.86006925621027 | FOLR1//SLCO1A5//SCARA5//CLDN1//CLDN3//CLDN19//TRPV4//ACE//HTR2C//AQP1//SLC16A8//SLC4A2//LRP2//ITGA8 |
| GO:0009925 | basal plasma membrane | Cellular component | 2 | 35 | 79 | 15288 | 11.0582278481013 | 0.0140490393495309 | 0.502955608713206 | 1.85235337109423 | AQP1//SLC16A8 |
| GO:0043296 | apical junction complex | Cellular component | 3 | 100 | 79 | 15288 | 5.80556962025316 | 0.0150011124114757 | 0.503474835310153 | 1.8238765345275 | CLDN1//CLDN3//CLDN19 |
| GO:0016327 | apicolateral plasma membrane | Cellular component | 3 | 104 | 79 | 15288 | 5.58227848101266 | 0.0166485881825086 | 0.525899520823948 | 1.77862258921729 | CLDN1//CLDN3//CLDN19 |
| GO:0045178 | basal part of cell | Cellular component | 2 | 41 | 79 | 15288 | 9.43995060203767 | 0.0189799973762663 | 0.561392853003122 | 1.72170385194419 | AQP1//SLC16A8 |
| GO:0016529 | sarcoplasmic reticulum | Cellular component | 2 | 42 | 79 | 15288 | 9.21518987341772 | 0.0198630618380993 | 0.561392853003122 | 1.70195380534226 | DHRS7C//CASQ2 |
| GO:0005930 | axoneme | Cellular component | 2 | 47 | 79 | 15288 | 8.23485052518179 | 0.024529063815657 | 0.627243203286086 | 1.6103190269038 | DNAH1//EFHC1 |
| GO:0016528 | sarcoplasm | Cellular component | 2 | 47 | 79 | 15288 | 8.23485052518179 | 0.024529063815657 | 0.627243203286086 | 1.6103190269038 | DHRS7C//CASQ2 |
| GO:0019861 | flagellum | Cellular component | 2 | 56 | 79 | 15288 | 6.91139240506329 | 0.0339214091272394 | 0.827990759151253 | 1.46952611503935 | PACRG//EFHC1 |
| GO:0071944 | cell periphery | Cellular component | 23 | 3081 | 79 | 15288 | 1.44464028200609 | 0.036277013010283 | 0.846989390718347 | 1.44036847923477 | ACE//HTR2C//AQP1//TRHR//DIO3//PYGL//CLDN1//TRPV4//SLC16A3//MMP2//SLC5A5//CDH3//RAB19//FOLR1//SLCO1A5//SCARA5//CLDN3//CLDN19//EPN3//SLC16A8//SLC4A2//LRP2//ITGA8 |
| GO:0044463 | cell projection part | Cellular component | 7 | 636 | 79 | 15288 | 2.12992596130881 | 0.0458825958619141 | 0.925108381457731 | 1.33835201935374 | EFHC1//ITGA8//TRPV4//AQP1//LRP2//SLCO1A5//DNAH1 |
| GO:0042995 | cell projection | Cellular component | 11 | 1211 | 79 | 15288 | 1.7578107851028 | 0.0463818041782831 | 0.925108381457731 | 1.33365236200607 | EFHC1//AQP1//LRP2//FOLR1//TRPV4//CCDC40//PACRG//ITGA8//SLCO1A5//FSCN2//DNAH1 |
| GO:0005886 | plasma membrane | Cellular component | 22 | 2986 | 79 | 15288 | 1.42579294089718 | 0.0463865002410584 | 0.925108381457731 | 1.33360839280421 | FOLR1//SLCO1A5//SCARA5//CLDN1//CLDN3//CLDN19//TRPV4//ACE//HTR2C//AQP1//SLC16A8//SLC4A2//LRP2//ITGA8//TRHR//DIO3//PYGL//SLC16A3//MMP2//SLC5A5//CDH3//RAB19 |
| GO:0005911 | cell-cell junction | Cellular component | 4 | 264 | 79 | 15288 | 2.93210586881473 | 0.0477740953662583 | 0.925108381457731 | 1.32080752784515 | CDH3//CLDN1//CLDN3//CLDN19 |
| GO:0005902 | microvillus | Cellular component | 2 | 68 | 79 | 15288 | 5.69173492181683 | 0.0482365636514273 | 0.925108381457731 | 1.31662363868673 | FSCN2//AQP1 |
