# Supplementary material for: Gene Expression in the Hippocampus in a Rat Model of Premenstrual Dysphoric Disorder After Treatment With Baixiangdan Capsules
Source: Front Psychol. 2018 Nov 13;9:2065. doi: 10.3389/fpsyg.2018.02065 (PMC6242977; doi:10.3389/fpsyg.2018.02065)
Supplement: Supplementary file 3 [file Data_Sheet_3.ZIP › Data Analysis Folder/GO Analysis Report/model vs blank (up)/MF_Pvalue_tree.pdf]

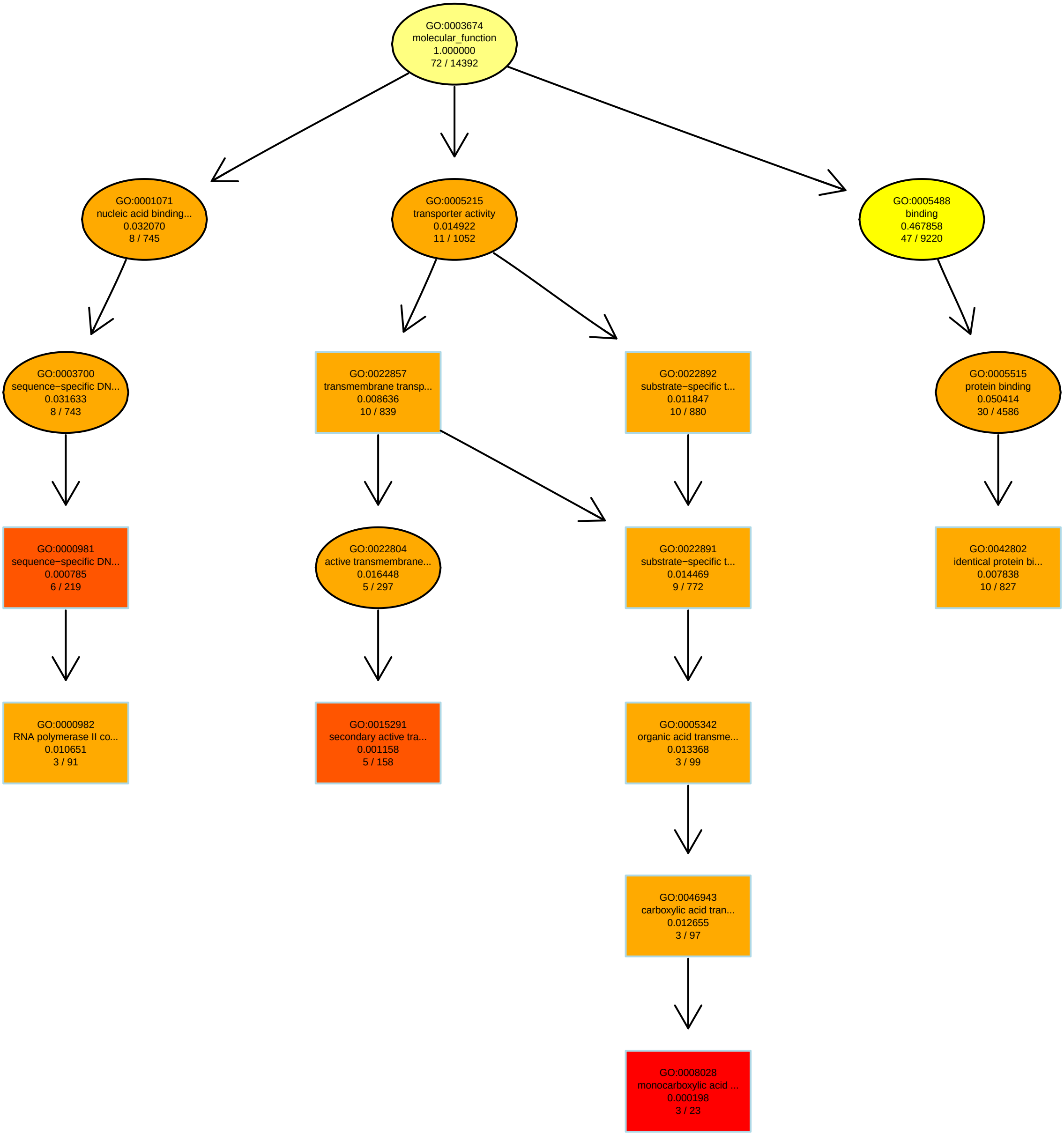
GO:0003674
molecular_function
1.000000
72 / 14392
GO:0001071
nucleic acid binding...
0.032070
8 / 745
GO:0005215
transporter activity
0.014922
11 / 1052
GO:0005488
binding
0.467858
47 / 9220
GO:0003700
sequence−specific DN...
0.031633
8 / 743
GO:0022857
transmembrane transp...
0.008636
10 / 839
GO:0022892
substrate−specific t...
0.011847
10 / 880
GO:0005515
protein binding
0.050414
30 / 4586
GO:0000981
sequence−specific DN...
0.000785
6 / 219
GO:0022804
active transmembrane...
0.016448
5 / 297
GO:0022891
substrate−specific t...
0.014469
9 / 772
GO:0042802
identical protein bi...
0.007838
10 / 827
GO:0000982
RNA polymerase II co...
0.010651
3 / 91
GO:0015291
secondary active tra...
0.001158
5 / 158
GO:0005342
organic acid transme...
0.013368
3 / 99
GO:0046943
carboxylic acid tran...
0.012655
3 / 97
GO:0008028
monocarboxylic acid ...
0.000198
3 / 23
