# Supplementary material for: Gene Expression in the Hippocampus in a Rat Model of Premenstrual Dysphoric Disorder After Treatment With Baixiangdan Capsules
Source: Front Psychol. 2018 Nov 13;9:2065. doi: 10.3389/fpsyg.2018.02065 (PMC6242977; doi:10.3389/fpsyg.2018.02065)
Supplement: Supplementary file 3 [file Data_Sheet_3.ZIP › Data Analysis Folder/GO Analysis Report/model vs blank (up)/MF_result(Rat).html]

| GO.ID | Term | Ontology | Count | Pop.Hits | List.Total | Pop.Total | Fold.Enrichment | Pvalue | FDR | Enrichment.Score | GENES |
| --- | --- | --- | --- | --- | --- | --- | --- | --- | --- | --- | --- |
| GO:0008028 | monocarboxylic acid transmembrane transporter activity | Molecular function | 3 | 23 | 72 | 14392 | 26.0724637681159 | 0.00019787686548575 | 0.177495548340718 | 3.70360497779555 | SLCO1A5//SLC16A8//SLC16A3 |
| GO:0000981 | sequence-specific DNA binding RNA polymerase II transcription factor activity | Molecular function | 6 | 219 | 72 | 14392 | 5.47640791476408 | 0.000784798838814172 | 0.346363518186818 | 3.10524164820835 | MSX2//MSX1//HES1//FOXJ1//TEAD2//NR2F2 |
| GO:0015291 | secondary active transmembrane transporter activity | Molecular function | 5 | 158 | 72 | 14392 | 6.32559774964838 | 0.00115840641534053 | 0.346363518186818 | 2.93613904598526 | SLC4A2//SLC5A5//SLC16A8//SLC16A3//SLCO1A5 |
| GO:0042802 | identical protein binding | Molecular function | 10 | 827 | 72 | 14392 | 2.41703614134086 | 0.00783752857114462 | 1 | 2.10582086296916 | TRHR//CASQ2//HES1//PYGL//NR2F2//XCL1//PON3//EPOR//CLDN1//CLDN3 |
| GO:0022857 | transmembrane transporter activity | Molecular function | 10 | 839 | 72 | 14392 | 2.38246589855648 | 0.00863587841066386 | 1 | 2.06369348096836 | COX8B//AQP1//TRPV4//SLC4A2//SLCO1A5//SLC5A5//FOLR1//SLC16A8//SLC16A3//SLC2A12 |
| GO:0000982 | RNA polymerase II core promoter proximal region sequence-specific DNA binding transcription factor activity | Molecular function | 3 | 91 | 72 | 14392 | 6.58974358974359 | 0.0106511101549477 | 1 | 1.97260512377071 | HES1//MSX2//MSX1 |
| GO:0022892 | substrate-specific transporter activity | Molecular function | 10 | 880 | 72 | 14392 | 2.27146464646465 | 0.0118471697423598 | 1 | 1.92638538907042 | COX8B//AQP1//TRPV4//SLC4A2//SLCO1A5//SLC5A5//SLC16A8//SLC16A3//SLC2A12//LRP2 |
| GO:0046943 | carboxylic acid transmembrane transporter activity | Molecular function | 3 | 97 | 72 | 14392 | 6.18213058419244 | 0.0126551218354129 | 1 | 1.89773366937631 | SLCO1A5//SLC16A8//SLC16A3 |
| GO:0005342 | organic acid transmembrane transporter activity | Molecular function | 3 | 99 | 72 | 14392 | 6.05723905723906 | 0.0133678524551302 | 1 | 1.87393835652254 | SLCO1A5//SLC16A8//SLC16A3 |
| GO:0022891 | substrate-specific transmembrane transporter activity | Molecular function | 9 | 772 | 72 | 14392 | 2.33031088082902 | 0.0144685499779905 | 1 | 1.83957499121075 | COX8B//AQP1//TRPV4//SLC4A2//SLCO1A5//SLC5A5//SLC16A8//SLC16A3//SLC2A12 |
| GO:0005215 | transporter activity | Molecular function | 11 | 1052 | 72 | 14392 | 2.09009294465568 | 0.0149220925864035 | 1 | 1.82617026969159 | COX8B//AQP1//TRPV4//SLC4A2//SLCO1A5//SLC5A5//FOLR1//SLC16A8//SLC16A3//SLC2A12//LRP2 |
| GO:0015293 | symporter activity | Molecular function | 3 | 106 | 72 | 14392 | 5.65723270440252 | 0.0160405256725405 | 1 | 1.79478140332475 | SLC5A5//SLC16A8//SLC16A3 |
| GO:0022804 | active transmembrane transporter activity | Molecular function | 5 | 297 | 72 | 14392 | 3.36513280957725 | 0.0164483215941642 | 1 | 1.78387841136639 | SLC4A2//SLC5A5//SLC16A8//SLC16A3//SLCO1A5 |
| GO:0005080 | protein kinase C binding | Molecular function | 2 | 41 | 72 | 14392 | 9.75067750677507 | 0.0178464204180767 | 1 | 1.74844888031225 | CASQ2//TRPV4 |
| GO:0008144 | drug binding | Molecular function | 3 | 112 | 72 | 14392 | 5.35416666666667 | 0.0185537727151505 | 1 | 1.7315677678399 | ACE//HTR2C//PYGL |
| GO:0042803 | protein homodimerization activity | Molecular function | 7 | 558 | 72 | 14392 | 2.50756670649144 | 0.0212575352792654 | 1 | 1.67248709149038 | TRHR//CASQ2//HES1//PYGL//NR2F2//XCL1//PON3 |
| GO:0046983 | protein dimerization activity | Molecular function | 9 | 839 | 72 | 14392 | 2.14421930870083 | 0.0235378870423766 | 1 | 1.62823252564819 | TRHR//CASQ2//HES1//PYGL//NR2F2//XCL1//PON3//TTR//SCX |
| GO:0008509 | anion transmembrane transporter activity | Molecular function | 3 | 127 | 72 | 14392 | 4.72178477690289 | 0.0257428567119382 | 1 | 1.58934326064102 | SLC4A2//SLC5A5//SLCO1A5 |
| GO:0038024 | cargo receptor activity | Molecular function | 2 | 50 | 72 | 14392 | 7.99555555555556 | 0.0259044914792967 | 1 | 1.58662492875432 | ENPP2//SCARA5 |
| GO:0033293 | monocarboxylic acid binding | Molecular function | 2 | 53 | 72 | 14392 | 7.54297693920335 | 0.0288623394693727 | 1 | 1.53966846958831 | NR2F2//PYGL |
| GO:0003700 | sequence-specific DNA binding transcription factor activity | Molecular function | 8 | 743 | 72 | 14392 | 2.15223568117242 | 0.0316330949816696 | 1 | 1.49985831463807 | HES1//MSX2//MSX1//FOXJ1//TEAD2//NR2F2//LMX1A//MESP1 |
| GO:0001071 | nucleic acid binding transcription factor activity | Molecular function | 8 | 745 | 72 | 14392 | 2.14645786726324 | 0.0320701376175406 | 1 | 1.49389917644792 | HES1//MSX2//MSX1//NR2F2//LMX1A//TEAD2//MESP1//FOXJ1 |
| GO:0043565 | sequence-specific DNA binding | Molecular function | 7 | 618 | 72 | 14392 | 2.2641136281913 | 0.0344865946764823 | 1 | 1.46234968722805 | MSX2//HES1//MSX1//NR2F2//FOXJ1//LMX1A//SCX |
| GO:0051015 | actin filament binding | Molecular function | 2 | 66 | 72 | 14392 | 6.05723905723906 | 0.043107442228235 | 1 | 1.36544774516092 | TRPV4//FSCN2 |
| GO:0003779 | actin binding | Molecular function | 4 | 264 | 72 | 14392 | 3.02861952861953 | 0.0431715590542422 | 1 | 1.36480226738662 | TRPV4//FSCN2//ACE//MEFV |
| GO:0004622 | lysophospholipase activity | Molecular function | 1 | 10 | 72 | 14392 | 19.9888888888889 | 0.0489313949380137 | 1 | 1.31041240318045 | ENPP2 |
| GO:0015250 | water channel activity | Molecular function | 1 | 10 | 72 | 14392 | 19.9888888888889 | 0.0489313949380137 | 1 | 1.31041240318045 | AQP1 |
| GO:0051184 | cofactor transporter activity | Molecular function | 1 | 10 | 72 | 14392 | 19.9888888888889 | 0.0489313949380137 | 1 | 1.31041240318045 | FOLR1 |
| GO:0051378 | serotonin binding | Molecular function | 1 | 10 | 72 | 14392 | 19.9888888888889 | 0.0489313949380137 | 1 | 1.31041240318045 | HTR2C |
| GO:0072349 | modified amino acid transmembrane transporter activity | Molecular function | 1 | 10 | 72 | 14392 | 19.9888888888889 | 0.0489313949380137 | 1 | 1.31041240318045 | SLCO1A5 |
| GO:0005509 | calcium ion binding | Molecular function | 6 | 531 | 72 | 14392 | 2.2586315128688 | 0.0494546286669961 | 1 | 1.30579305471472 | CASQ2//LRP2//ENPP2//CAPSL//EFHC1//CALML4 |
