# Supplementary material for: Gene Expression in the Hippocampus in a Rat Model of Premenstrual Dysphoric Disorder After Treatment With Baixiangdan Capsules
Source: Front Psychol. 2018 Nov 13;9:2065. doi: 10.3389/fpsyg.2018.02065 (PMC6242977; doi:10.3389/fpsyg.2018.02065)
Supplement: Supplementary file 3 [file Data_Sheet_3.ZIP › Data Analysis Folder/GO Analysis Report/summary_GO.doc]

- **GO analysis**

The Gene Ontology project provides a controlled vocabulary to describe gene and gene product attributes in any organism (http://www.geneontology.org). The ontology covers three domains: Biological Process, Cellular Component and Molecular Function. Fisher’s exact test is used to find if there is more overlap between the DE list and the GO annotation list than would be expected by chance. The p-value denotes the significance of GO terms enrichment in the DE genes. The lower the p-value, the more significant the GO Term (p-value<=0.05 is recommended).

- **Items explanation:**
- **GOID:** The ID of gene ontology term used in [Gene Ontology Project](http://www.geneontology.org/).
- **Term:** The name of gene ontology term.
- **Ontology:** The ontology of GOID belongs to, namely "biological process", "cellular component" or "molecular function".
- **Count:** The number of DE genes associated with the listed GOID.
- **Pop.Hits:** The number of background population genes associated with the listed GOID.
- **List.Total:** The total number of DE genes.
- **Pop.Total:** The total number of background population genes.
- **Fold.Enrichment:** The GOID ‘s Fold Enrichment value, it equals ( Count/Pop.Hits )/( List.Total/Pop.Total ).
- **Pvalue:** The significance testing value of the GOID, result from the topGO of bioconductor. If the Pvalue is lower than 1e-299, the Pvalue will be adjusted as the value of 1e-299.
- **FDR:** The false discovery rate of the GOID, using Benjamini & Hochberg (1995) method.
- **Enrichment.Score:** The GOID ‘s Enrichment Score value, it equals ( -log10(Pvalue)).
- **Genes:** The DE genes associated with the GOID.
- **GO Directed Acyclic Graph (DAG) explanation:**
- In the GO DAG, annotation moves from more general to more specific as one moves from parent nodes to child nodes. A gene is annotated to a specific node and then also considered to be annotated at the parent nodes. Top ten terms with the lowest Pvalue and their parents are shown in GO DAG, the terms with pane marks are significant enrichment, more red more significantly. If the Pvalue is lower than “1e-20”, it will be shown as “<1e-20” in the GO DAG.
- **Pie chart explanation:**
- The chart shows the top ten counts of the significant enrichment terms.
- **Bar plot explanation(Fold Enrichment):**
- The bar plot shows the top ten Fold Enrichment value of the significant enrichment terms.
- **Bar plot explanation(Enrichment Score):**
- The bar plot shows the top ten Enrichment Score value of the significant enrichment terms.
