# Supplementary material for: Gene Expression in the Hippocampus in a Rat Model of Premenstrual Dysphoric Disorder After Treatment With Baixiangdan Capsules
Source: Front Psychol. 2018 Nov 13;9:2065. doi: 10.3389/fpsyg.2018.02065 (PMC6242977; doi:10.3389/fpsyg.2018.02065)
Supplement: Supplementary file 3 [file Data_Sheet_3.ZIP › Data Analysis Folder/Pathway Analysis Report/BXD vs blank (down)/rno_pathwayResult.html]

| PathwayID | Definition | OriginalWebSite | Fisher-Pvalue | SelectionCounts | SelectionSize | Count | Size | FDR | Enrichment\_Score | Genes |
| --- | --- | --- | --- | --- | --- | --- | --- | --- | --- | --- |
| rno04141 | Protein processing in endoplasmic reticulum - Rattus norvegicus (rat) | http://www.genome.jp/kegg-bin/show\_pathway?rno04141+64202+29144+689593+301252+24468+288444+25272+50658+29468+116598+685144+294912 | 2.340368e-004 | 12 | 159 | 162 | 7182 | 6.061552e-002 | 3.630716e+000 | CALR//CANX//DNAJB2//HSP90AB1//HSPA8//HSPH1//MAPK10//MAPK9//PDIA3//PDIA4//SEC24C//SEC62 |
| rno04360 | Axon guidance - Rattus norvegicus (rat) | http://www.genome.jp/kegg-bin/show\_pathway?rno04360+24511+641521+246331+29432+24674+24675+58946+296787+315711 | 2.052447e-003 | 9 | 159 | 128 | 7182 | 2.657919e-001 | 2.687728e+000 | ITGB1//LRRC4//NRP1//PAK2//PPP3CA//PPP3CB//ROBO1//SEMA3C//SEMA7A |
| rno04010 | MAPK signaling pathway - Rattus norvegicus (rat) | http://www.genome.jp/kegg-bin/show\_pathway?rno04010+306243+60350+25112+24468+308106+25272+50658+29432+24667+24674+24675+114513 | 1.246064e-002 | 12 | 159 | 260 | 7182 | 5.589978e-001 | 1.904460e+000 | CACNA2D3//CD14//GADD45A//HSPA8//MAP3K4//MAPK10//MAPK9//PAK2//PPM1B//PPP3CA//PPP3CB//RASGRF2 |
| rno04310 | Wnt signaling pathway - Rattus norvegicus (rat) | http://www.genome.jp/kegg-bin/show\_pathway?rno04310+25272+50658+24654+24674+24675+312563+78973+679869 | 1.381001e-002 | 8 | 159 | 143 | 7182 | 5.589978e-001 | 1.859806e+000 | MAPK10//MAPK9//PLCB1//PPP3CA//PPP3CB//PRICKLE2//SENP2//TCF7L2 |
| rno00062 | Fatty acid elongation - Rattus norvegicus (rat) | http://www.genome.jp/kegg-bin/show\_pathway?rno00062+361895+170670+84013 | 1.713296e-002 | 3 | 159 | 25 | 7182 | 5.589978e-001 | 1.766168e+000 | ELOVL7//HADHA//HSD17B12 |
| rno01040 | Biosynthesis of unsaturated fatty acids - Rattus norvegicus (rat) | http://www.genome.jp/kegg-bin/show\_pathway?rno01040+84575+170670+84013 | 1.905929e-002 | 3 | 159 | 26 | 7182 | 5.589978e-001 | 1.719893e+000 | FADS1//HADHA//HSD17B12 |
| rno05412 | Arrhythmogenic right ventricular cardiomyopathy (ARVC) - Rattus norvegicus (rat) | http://www.genome.jp/kegg-bin/show\_pathway?rno05412+81822+287876+306243+24511+679869 | 2.125843e-002 | 5 | 159 | 72 | 7182 | 5.589978e-001 | 1.672469e+000 | ACTB//ACTG1//CACNA2D3//ITGB1//TCF7L2 |
| rno04930 | Type II diabetes mellitus - Rattus norvegicus (rat) | http://www.genome.jp/kegg-bin/show\_pathway?rno04930+25272+50658+85243+25630 | 2.420333e-002 | 4 | 159 | 50 | 7182 | 5.589978e-001 | 1.616125e+000 | MAPK10//MAPK9//PIK3CB//PKM2 |
| rno04728 | Dopaminergic synapse - Rattus norvegicus (rat) | http://www.genome.jp/kegg-bin/show\_pathway?rno04728+25262+25272+50658+24654+691318+24674+24675 | 2.497719e-002 | 7 | 159 | 130 | 7182 | 5.589978e-001 | 1.602456e+000 | ITPR1//MAPK10//MAPK9//PLCB1//PPP2R5C//PPP3CA//PPP3CB |
| rno04723 | Retrograde endocannabinoid signaling - Rattus norvegicus (rat) | http://www.genome.jp/kegg-bin/show\_pathway?rno04723+29347+25262+25272+50658+29254+24654 | 2.634581e-002 | 6 | 159 | 103 | 7182 | 5.589978e-001 | 1.579288e+000 | FAAH//ITPR1//MAPK10//MAPK9//MGLL//PLCB1 |
| rno03013 | RNA transport - Rattus norvegicus (rat) | http://www.genome.jp/kegg-bin/show\_pathway?rno03013+362952+298573+56783+308306+24948+24917+78973+361271 | 2.884061e-002 | 8 | 159 | 164 | 7182 | 5.589978e-001 | 1.539996e+000 | EIF3D//EIF4G3//EIF5//EIF5B//FMR1//KPNB1//SENP2//UPF2 |
| rno04530 | Tight junction - Rattus norvegicus (rat) | http://www.genome.jp/kegg-bin/show\_pathway?rno04530+81822+287876+84588+116654+500261+29365+84006 | 2.886646e-002 | 7 | 159 | 134 | 7182 | 5.589978e-001 | 1.539606e+000 | ACTB//ACTG1//CLDN11//EXOC4//MAGI1//MPDZ//PRKCI |
| rno05132 | Salmonella infection - Rattus norvegicus (rat) | http://www.genome.jp/kegg-bin/show\_pathway?rno05132+81822+287876+60350+25272+50658 | 2.889014e-002 | 5 | 159 | 78 | 7182 | 5.589978e-001 | 1.539250e+000 | ACTB//ACTG1//CD14//MAPK10//MAPK9 |
| rno03040 | Spliceosome - Rattus norvegicus (rat) | http://www.genome.jp/kegg-bin/show\_pathway?rno03040+361859+245957+29578+362152+117282+24468+679898 | 3.095718e-002 | 7 | 159 | 136 | 7182 | 5.589978e-001 | 1.509239e+000 | CDC40//DDX46//HNRNPA1//HNRNPA3//HNRNPK//HSPA8//ZMAT2 |
| rno04130 | SNARE interactions in vesicular transport - Rattus norvegicus (rat) | http://www.genome.jp/kegg-bin/show\_pathway?rno04130+94189+25012+25624 | 3.860482e-002 | 3 | 159 | 34 | 7182 | 5.589978e-001 | 1.413359e+000 | GOSR1//SNAP25//VAMP1 |
| rno05134 | Legionellosis - Rattus norvegicus (rat) | http://www.genome.jp/kegg-bin/show\_pathway?rno05134+78963+64310+60350+24468 | 4.111468e-002 | 4 | 159 | 59 | 7182 | 5.589978e-001 | 1.386003e+000 | APAF1//ARF1//CD14//HSPA8 |
| rno04670 | Leukocyte transendothelial migration - Rattus norvegicus (rat) | http://www.genome.jp/kegg-bin/show\_pathway?rno04670+81822+287876+84588+24511+85243+252857 | 4.494420e-002 | 6 | 159 | 117 | 7182 | 5.589978e-001 | 1.347326e+000 | ACTB//ACTG1//CLDN11//ITGB1//PIK3CB//RAPGEF4 |
| rno05152 | Tuberculosis - Rattus norvegicus (rat) | http://www.genome.jp/kegg-bin/show\_pathway?rno05152+78963+60350+291671+25328+25272+50658+24674+24675 | 4.605835e-002 | 8 | 159 | 180 | 7182 | 5.589978e-001 | 1.336692e+000 | APAF1//CD14//HSPA9//LAMP1//MAPK10//MAPK9//PPP3CA//PPP3CB |
| rno04912 | GnRH signaling pathway - Rattus norvegicus (rat) | http://www.genome.jp/kegg-bin/show\_pathway?rno04912+25262+308106+25272+50658+24654 | 4.693701e-002 | 5 | 159 | 89 | 7182 | 5.589978e-001 | 1.328485e+000 | ITPR1//MAP3K4//MAPK10//MAPK9//PLCB1 |
| rno05014 | Amyotrophic lateral sclerosis (ALS) - Rattus norvegicus (rat) | http://www.genome.jp/kegg-bin/show\_pathway?rno05014+78963+24248+24674+24675 | 4.792722e-002 | 4 | 159 | 62 | 7182 | 5.589978e-001 | 1.319418e+000 | APAF1//CAT//PPP3CA//PPP3CB |
| rno04724 | Glutamatergic synapse - Rattus norvegicus (rat) | http://www.genome.jp/kegg-bin/show\_pathway?rno04724+24957+25262+24654+24674+24675+29483 | 4.977878e-002 | 6 | 159 | 120 | 7182 | 5.589978e-001 | 1.302956e+000 | GLUL//ITPR1//PLCB1//PPP3CA//PPP3CB//SLC1A3 |
