# Supplementary material for: Gene Expression in the Hippocampus in a Rat Model of Premenstrual Dysphoric Disorder After Treatment With Baixiangdan Capsules
Source: Front Psychol. 2018 Nov 13;9:2065. doi: 10.3389/fpsyg.2018.02065 (PMC6242977; doi:10.3389/fpsyg.2018.02065)
Supplement: Supplementary file 3 [file Data_Sheet_3.ZIP › Data Analysis Folder/Pathway Analysis Report/BXD vs blank (up)/rno_pathwayResult.html]

| PathwayID | Definition | OriginalWebSite | Fisher-Pvalue | SelectionCounts | SelectionSize | Count | Size | FDR | Enrichment\_Score | Genes |
| --- | --- | --- | --- | --- | --- | --- | --- | --- | --- | --- |
| rno04080 | Neuroactive ligand-receptor interaction - Rattus norvegicus (rat) | http://www.genome.jp/kegg-bin/show\_pathway?rno04080+25302+29422+24318+171101+85268+25187+25264+24604+366274+114115+24664+25033+294126+24806+24807+25570 | 4.860649e-007 | 16 | 80 | 329 | 7182 | 1.258908e-004 | 6.313306e+000 | CHRNA7//CHRNE//DRD2//GRP//HRH3//HTR2C//NMBR//NPY//NTSR1//P2RX2//POMC//SSTR1//TAAR7B//TAC1//TACR1//TRHR |
| rno04020 | Calcium signaling pathway - Rattus norvegicus (rat) | http://www.genome.jp/kegg-bin/show\_pathway?rno04020+25302+25187+366274+114115+24807+25570 | 1.757698e-002 | 6 | 80 | 187 | 7182 | 1.000000e+000 | 1.755056e+000 | CHRNA7//HTR2C//NTSR1//P2RX2//TACR1//TRHR |
| rno04966 | Collecting duct acid secretion - Rattus norvegicus (rat) | http://www.genome.jp/kegg-bin/show\_pathway?rno04966+24217+79430 | 3.592985e-002 | 2 | 80 | 27 | 7182 | 1.000000e+000 | 1.444545e+000 | ATP4B//CLCNKB |
