# Supplementary material for: Gene Expression in the Hippocampus in a Rat Model of Premenstrual Dysphoric Disorder After Treatment With Baixiangdan Capsules
Source: Front Psychol. 2018 Nov 13;9:2065. doi: 10.3389/fpsyg.2018.02065 (PMC6242977; doi:10.3389/fpsyg.2018.02065)
Supplement: Supplementary file 3 [file Data_Sheet_3.ZIP › Data Analysis Folder/Pathway Analysis Report/BXD vs fluoxetine (down)/rno_pathwayResult.html]

| PathwayID | Definition | OriginalWebSite | Fisher-Pvalue | SelectionCounts | SelectionSize | Count | Size | FDR | Enrichment\_Score | Genes |
| --- | --- | --- | --- | --- | --- | --- | --- | --- | --- | --- |
| rno04512 | ECM-receptor interaction - Rattus norvegicus (rat) | http://www.genome.jp/kegg-bin/show\_pathway?rno04512+29393+84032+25353 | 4.914371e-004 | 3 | 15 | 77 | 7182 | 1.272822e-001 | 3.308532e+000 | COL1A1//COL3A1//SPP1 |
| rno05146 | Amoebiasis - Rattus norvegicus (rat) | http://www.genome.jp/kegg-bin/show\_pathway?rno05146+29393+84032+291091 | 1.182580e-003 | 3 | 15 | 104 | 7182 | 1.531441e-001 | 2.927169e+000 | COL1A1//COL3A1//SERPINB1A |
| rno04510 | Focal adhesion - Rattus norvegicus (rat) | http://www.genome.jp/kegg-bin/show\_pathway?rno04510+29393+84032+25353 | 6.750522e-003 | 3 | 15 | 192 | 7182 | 5.827950e-001 | 2.170663e+000 | COL1A1//COL3A1//SPP1 |
| rno04610 | Complement and coagulation cascades - Rattus norvegicus (rat) | http://www.genome.jp/kegg-bin/show\_pathway?rno04610+54249+304929 | 9.059326e-003 | 2 | 15 | 70 | 7182 | 5.865914e-001 | 2.042904e+000 | CFD//F5 |
| rno04974 | Protein digestion and absorption - Rattus norvegicus (rat) | http://www.genome.jp/kegg-bin/show\_pathway?rno04974+29393+84032 | 1.285849e-002 | 2 | 15 | 84 | 7182 | 6.660698e-001 | 1.890810e+000 | COL1A1//COL3A1 |
| rno04151 | PI3K-Akt signaling pathway - Rattus norvegicus (rat) | http://www.genome.jp/kegg-bin/show\_pathway?rno04151+29393+84032+25353 | 2.764042e-002 | 3 | 15 | 324 | 7182 | 1.000000e+000 | 1.558455e+000 | COL1A1//COL3A1//SPP1 |
