# Supplementary material for: Gene Expression in the Hippocampus in a Rat Model of Premenstrual Dysphoric Disorder After Treatment With Baixiangdan Capsules
Source: Front Psychol. 2018 Nov 13;9:2065. doi: 10.3389/fpsyg.2018.02065 (PMC6242977; doi:10.3389/fpsyg.2018.02065)
Supplement: Supplementary file 3 [file Data_Sheet_3.ZIP › Data Analysis Folder/Pathway Analysis Report/BXD vs fluoxetine (up)/rno_pathwayResult.html]

| PathwayID | Definition | OriginalWebSite | Fisher-Pvalue | SelectionCounts | SelectionSize | Count | Size | FDR | Enrichment\_Score | Genes |
| --- | --- | --- | --- | --- | --- | --- | --- | --- | --- | --- |
| rno04610 | Complement and coagulation cascades - Rattus norvegicus (rat) | http://www.genome.jp/kegg-bin/show\_pathway?rno04610+24232+54249+304929 | 1.365591e-003 | 3 | 23 | 70 | 7182 | 1.993092e-001 | 2.864679e+000 | C3//CFD//F5 |
| rno04512 | ECM-receptor interaction - Rattus norvegicus (rat) | http://www.genome.jp/kegg-bin/show\_pathway?rno04512+29393+84032+25353 | 1.798398e-003 | 3 | 23 | 77 | 7182 | 1.993092e-001 | 2.745114e+000 | COL1A1//COL3A1//SPP1 |
| rno04974 | Protein digestion and absorption - Rattus norvegicus (rat) | http://www.genome.jp/kegg-bin/show\_pathway?rno04974+29393+84032+170566 | 2.308601e-003 | 3 | 23 | 84 | 7182 | 1.993092e-001 | 2.636651e+000 | COL1A1//COL3A1//SLC16A10 |
| rno05146 | Amoebiasis - Rattus norvegicus (rat) | http://www.genome.jp/kegg-bin/show\_pathway?rno05146+29393+84032+291091 | 4.231907e-003 | 3 | 23 | 104 | 7182 | 2.740160e-001 | 2.373464e+000 | COL1A1//COL3A1//SERPINB1A |
| rno05150 | Staphylococcus aureus infection - Rattus norvegicus (rat) | http://www.genome.jp/kegg-bin/show\_pathway?rno05150+24232+54249 | 1.094728e-002 | 2 | 23 | 50 | 7182 | 5.670691e-001 | 1.960694e+000 | C3//CFD |
| rno04510 | Focal adhesion - Rattus norvegicus (rat) | http://www.genome.jp/kegg-bin/show\_pathway?rno04510+29393+84032+25353 | 2.247227e-002 | 3 | 23 | 192 | 7182 | 9.700528e-001 | 1.648353e+000 | COL1A1//COL3A1//SPP1 |
