# Supplementary material for: Gene Expression in the Hippocampus in a Rat Model of Premenstrual Dysphoric Disorder After Treatment With Baixiangdan Capsules
Source: Front Psychol. 2018 Nov 13;9:2065. doi: 10.3389/fpsyg.2018.02065 (PMC6242977; doi:10.3389/fpsyg.2018.02065)
Supplement: Supplementary file 3 [file Data_Sheet_3.ZIP › Data Analysis Folder/Pathway Analysis Report/BXD vs model (down)/rno_pathwayResult.html]

| PathwayID | Definition | OriginalWebSite | Fisher-Pvalue | SelectionCounts | SelectionSize | Count | Size | FDR | Enrichment\_Score | Genes |
| --- | --- | --- | --- | --- | --- | --- | --- | --- | --- | --- |
| rno05310 | Asthma - Rattus norvegicus (rat) | http://www.genome.jp/kegg-bin/show\_pathway?rno05310+688090+309622+294269+294270 | 3.110103e-004 | 4 | 89 | 27 | 7182 | 8.055167e-002 | 3.507225e+000 | RT1-BB//RT1-BB//RT1-DA//RT1-DB1 |
| rno05140 | Leishmaniasis - Rattus norvegicus (rat) | http://www.genome.jp/kegg-bin/show\_pathway?rno05140+29527+688090+309622+294269+294270 | 1.377453e-003 | 5 | 89 | 67 | 7182 | 1.769284e-001 | 2.860923e+000 | PTGS2//RT1-BB//RT1-BB//RT1-DA//RT1-DB1 |
| rno04672 | Intestinal immune network for IgA production - Rattus norvegicus (rat) | http://www.genome.jp/kegg-bin/show\_pathway?rno04672+688090+309622+294269+294270 | 2.049364e-003 | 4 | 89 | 44 | 7182 | 1.769284e-001 | 2.688381e+000 | RT1-BB//RT1-BB//RT1-DA//RT1-DB1 |
| rno05150 | Staphylococcus aureus infection - Rattus norvegicus (rat) | http://www.genome.jp/kegg-bin/show\_pathway?rno05150+688090+309622+294269+294270 | 3.286165e-003 | 4 | 89 | 50 | 7182 | 1.783455e-001 | 2.483311e+000 | RT1-BB//RT1-BB//RT1-DA//RT1-DB1 |
| rno05322 | Systemic lupus erythematosus - Rattus norvegicus (rat) | http://www.genome.jp/kegg-bin/show\_pathway?rno05322+24237+117517+688090+309622+294269+294270 | 3.759748e-003 | 6 | 89 | 121 | 7182 | 1.783455e-001 | 2.424841e+000 | C6//C7//RT1-BB//RT1-BB//RT1-DA//RT1-DB1 |
| rno04514 | Cell adhesion molecules (CAMs) - Rattus norvegicus (rat) | http://www.genome.jp/kegg-bin/show\_pathway?rno04514+116777+65129+298487+688090+309622+294269+294270 | 4.223003e-003 | 7 | 89 | 165 | 7182 | 1.783455e-001 | 2.374379e+000 | CDH3//CLDN1//CLDN19//RT1-BB//RT1-BB//RT1-DA//RT1-DB1 |
| rno00982 | Drug metabolism - cytochrome P450 - Rattus norvegicus (rat) | http://www.genome.jp/kegg-bin/show\_pathway?rno00982+246245+84493+24424+171341+113992 | 5.259798e-003 | 5 | 89 | 91 | 7182 | 1.783455e-001 | 2.279031e+000 | FMO2//FMO3//GSTM2//MGST1//UGT1A6 |
| rno04612 | Antigen processing and presentation - Rattus norvegicus (rat) | http://www.genome.jp/kegg-bin/show\_pathway?rno04612+25599+688090+309622+294269+294270 | 5.508741e-003 | 5 | 89 | 92 | 7182 | 1.783455e-001 | 2.258948e+000 | CD74//RT1-BB//RT1-BB//RT1-DA//RT1-DB1 |
| rno05332 | Graft-versus-host disease - Rattus norvegicus (rat) | http://www.genome.jp/kegg-bin/show\_pathway?rno05332+688090+309622+294269+294270 | 7.525359e-003 | 4 | 89 | 63 | 7182 | 2.084644e-001 | 2.123473e+000 | RT1-BB//RT1-BB//RT1-DA//RT1-DB1 |
| rno05416 | Viral myocarditis - Rattus norvegicus (rat) | http://www.genome.jp/kegg-bin/show\_pathway?rno05416+309368+688090+309622+294269+294270 | 8.474074e-003 | 5 | 89 | 102 | 7182 | 2.084644e-001 | 2.071908e+000 | LAMA2//RT1-BB//RT1-BB//RT1-DA//RT1-DB1 |
| rno05330 | Allograft rejection - Rattus norvegicus (rat) | http://www.genome.jp/kegg-bin/show\_pathway?rno05330+688090+309622+294269+294270 | 8.853702e-003 | 4 | 89 | 66 | 7182 | 2.084644e-001 | 2.052875e+000 | RT1-BB//RT1-BB//RT1-DA//RT1-DB1 |
| rno04610 | Complement and coagulation cascades - Rattus norvegicus (rat) | http://www.genome.jp/kegg-bin/show\_pathway?rno04610+24237+117517+304929+295703 | 1.085115e-002 | 4 | 89 | 70 | 7182 | 2.162863e-001 | 1.964524e+000 | C6//C7//F5//SERPING1 |
| rno04940 | Type I diabetes mellitus - Rattus norvegicus (rat) | http://www.genome.jp/kegg-bin/show\_pathway?rno04940+688090+309622+294269+294270 | 1.195063e-002 | 4 | 89 | 72 | 7182 | 2.162863e-001 | 1.922609e+000 | RT1-BB//RT1-BB//RT1-DA//RT1-DB1 |
| rno04976 | Bile secretion - Rattus norvegicus (rat) | http://www.genome.jp/kegg-bin/show\_pathway?rno04976+25240+83500+24780+80900 | 1.195063e-002 | 4 | 89 | 72 | 7182 | 2.162863e-001 | 1.922609e+000 | AQP1//SLC22A8//SLC4A2//SLCO1A5 |
| rno05320 | Autoimmune thyroid disease - Rattus norvegicus (rat) | http://www.genome.jp/kegg-bin/show\_pathway?rno05320+688090+309622+294269+294270 | 1.252623e-002 | 4 | 89 | 73 | 7182 | 2.162863e-001 | 1.902180e+000 | RT1-BB//RT1-BB//RT1-DA//RT1-DB1 |
| rno05145 | Toxoplasmosis - Rattus norvegicus (rat) | http://www.genome.jp/kegg-bin/show\_pathway?rno05145+309368+688090+309622+294269+294270 | 1.426657e-002 | 5 | 89 | 116 | 7182 | 2.309402e-001 | 1.845680e+000 | LAMA2//RT1-BB//RT1-BB//RT1-DA//RT1-DB1 |
| rno04640 | Hematopoietic cell lineage - Rattus norvegicus (rat) | http://www.genome.jp/kegg-bin/show\_pathway?rno04640+60350+24936+294269+294270 | 1.705475e-002 | 4 | 89 | 80 | 7182 | 2.598341e-001 | 1.768155e+000 | CD14//CD9//RT1-DA//RT1-DB1 |
| rno05323 | Rheumatoid arthritis - Rattus norvegicus (rat) | http://www.genome.jp/kegg-bin/show\_pathway?rno05323+688090+309622+294269+294270 | 1.927164e-002 | 4 | 89 | 83 | 7182 | 2.772975e-001 | 1.715081e+000 | RT1-BB//RT1-BB//RT1-DA//RT1-DB1 |
| rno05204 | Chemical carcinogenesis - Rattus norvegicus (rat) | http://www.genome.jp/kegg-bin/show\_pathway?rno05204+24424+171341+29527+113992 | 2.249403e-002 | 4 | 89 | 87 | 7182 | 3.066292e-001 | 1.647933e+000 | GSTM2//MGST1//PTGS2//UGT1A6 |
| rno05152 | Tuberculosis - Rattus norvegicus (rat) | http://www.genome.jp/kegg-bin/show\_pathway?rno05152+60350+25599+688090+309622+294269+294270 | 2.385535e-002 | 6 | 89 | 180 | 7182 | 3.089268e-001 | 1.622414e+000 | CD14//CD74//RT1-BB//RT1-BB//RT1-DA//RT1-DB1 |
| rno05166 | HTLV-I infection - Rattus norvegicus (rat) | http://www.genome.jp/kegg-bin/show\_pathway?rno05166+81710+25483+688090+309622+294269+294270+24230+116466 | 2.773831e-002 | 8 | 89 | 292 | 7182 | 3.421058e-001 | 1.556920e+000 | MSX1//MSX2//RT1-BB//RT1-BB//RT1-DA//RT1-DB1//TSPO//WNT2B |
| rno00062 | Fatty acid elongation - Rattus norvegicus (rat) | http://www.genome.jp/kegg-bin/show\_pathway?rno00062+361895+170670 | 3.787718e-002 | 2 | 89 | 25 | 7182 | 4.459177e-001 | 1.421622e+000 | ELOVL7//HADHA |
| rno04390 | Hippo signaling pathway - Rattus norvegicus (rat) | http://www.genome.jp/kegg-bin/show\_pathway?rno04390+25644+85272+360661+679869+116466 | 4.186000e-002 | 5 | 89 | 154 | 7182 | 4.633616e-001 | 1.378201e+000 | BMP6//BMP7//LLGL2//TCF7L2//WNT2B |
| rno05168 | Herpes simplex infection - Rattus norvegicus (rat) | http://www.genome.jp/kegg-bin/show\_pathway?rno05168+25599+688090+309622+294269+294270+364594 | 4.293698e-002 | 6 | 89 | 207 | 7182 | 4.633616e-001 | 1.367169e+000 | CD74//RT1-BB//RT1-BB//RT1-DA//RT1-DB1//TLR3 |
| rno00053 | Ascorbate and aldarate metabolism - Rattus norvegicus (rat) | http://www.genome.jp/kegg-bin/show\_pathway?rno00053+25106+113992 | 4.660362e-002 | 2 | 89 | 28 | 7182 | 4.642437e-001 | 1.331580e+000 | RGN//UGT1A6 |
| rno00650 | Butanoate metabolism - Rattus norvegicus (rat) | http://www.genome.jp/kegg-bin/show\_pathway?rno00650+295458+170670 | 4.660362e-002 | 2 | 89 | 28 | 7182 | 4.642437e-001 | 1.331580e+000 | BDH2//HADHA |
| rno05216 | Thyroid cancer - Rattus norvegicus (rat) | http://www.genome.jp/kegg-bin/show\_pathway?rno05216+59109+679869 | 4.966127e-002 | 2 | 89 | 29 | 7182 | 4.763803e-001 | 1.303982e+000 | NTRK1//TCF7L2 |
