# Supplementary material for: Gene Expression in the Hippocampus in a Rat Model of Premenstrual Dysphoric Disorder After Treatment With Baixiangdan Capsules
Source: Front Psychol. 2018 Nov 13;9:2065. doi: 10.3389/fpsyg.2018.02065 (PMC6242977; doi:10.3389/fpsyg.2018.02065)
Supplement: Supplementary file 3 [file Data_Sheet_3.ZIP › Data Analysis Folder/Pathway Analysis Report/BXD vs model (up)/rno_pathwayResult.html]

| PathwayID | Definition | OriginalWebSite | Fisher-Pvalue | SelectionCounts | SelectionSize | Count | Size | FDR | Enrichment\_Score | Genes |
| --- | --- | --- | --- | --- | --- | --- | --- | --- | --- | --- |
| rno04080 | Neuroactive ligand-receptor interaction - Rattus norvegicus (rat) | http://www.genome.jp/kegg-bin/show\_pathway?rno04080+25706+25590+25302+29422+60590+171101+24664+25033+294126 | 7.598604e-005 | 9 | 41 | 329 | 7182 | 1.968038e-002 | 4.119266e+000 | CCKBR//CHRNA4//CHRNA7//CHRNE//GRM8//GRP//POMC//SSTR1//TAAR7B |
| rno05033 | Nicotine addiction - Rattus norvegicus (rat) | http://www.genome.jp/kegg-bin/show\_pathway?rno05033+25590+25302+84487 | 1.473582e-003 | 3 | 41 | 40 | 7182 | 1.908289e-001 | 2.831626e+000 | CHRNA4//CHRNA7//SLC17A6 |
| rno04974 | Protein digestion and absorption - Rattus norvegicus (rat) | http://www.genome.jp/kegg-bin/show\_pathway?rno04974+25412+117184+170566 | 1.194985e-002 | 3 | 41 | 84 | 7182 | 1.000000e+000 | 1.922638e+000 | COL2A1//CTRL//SLC16A10 |
| rno04670 | Leukocyte transendothelial migration - Rattus norvegicus (rat) | http://www.genome.jp/kegg-bin/show\_pathway?rno04670+290789+304407+54355 | 2.867331e-002 | 3 | 41 | 117 | 7182 | 1.000000e+000 | 1.542522e+000 | CLDN23//CLDN4//RASSF5 |
| rno05223 | Non-small cell lung cancer - Rattus norvegicus (rat) | http://www.genome.jp/kegg-bin/show\_pathway?rno05223+54355+83574 | 3.522693e-002 | 2 | 41 | 52 | 7182 | 1.000000e+000 | 1.453125e+000 | RASSF5//RXRG |
| rno04360 | Axon guidance - Rattus norvegicus (rat) | http://www.genome.jp/kegg-bin/show\_pathway?rno04360+24553+290562+314903 | 3.605094e-002 | 3 | 41 | 128 | 7182 | 1.000000e+000 | 1.443083e+000 | MET//SEMA3G//SRGAP1 |
| rno04530 | Tight junction - Rattus norvegicus (rat) | http://www.genome.jp/kegg-bin/show\_pathway?rno04530+290789+304407+24583 | 4.045134e-002 | 3 | 41 | 134 | 7182 | 1.000000e+000 | 1.393067e+000 | CLDN23//CLDN4//MYH3 |
| rno04060 | Cytokine-cytokine receptor interaction - Rattus norvegicus (rat) | http://www.genome.jp/kegg-bin/show\_pathway?rno04060+25542+252834+171334+24553 | 4.629156e-002 | 4 | 41 | 239 | 7182 | 1.000000e+000 | 1.334498e+000 | CCL3//GDF6//IL12RB2//MET |
