# Supplementary material for: Gene Expression in the Hippocampus in a Rat Model of Premenstrual Dysphoric Disorder After Treatment With Baixiangdan Capsules
Source: Front Psychol. 2018 Nov 13;9:2065. doi: 10.3389/fpsyg.2018.02065 (PMC6242977; doi:10.3389/fpsyg.2018.02065)
Supplement: Supplementary file 3 [file Data_Sheet_3.ZIP › Data Analysis Folder/Pathway Analysis Report/fluoxetine vs blank (down)/rno_pathwayResult.html]

| PathwayID | Definition | OriginalWebSite | Fisher-Pvalue | SelectionCounts | SelectionSize | Count | Size | FDR | Enrichment\_Score | Genes |
| --- | --- | --- | --- | --- | --- | --- | --- | --- | --- | --- |
| rno04977 | Vitamin digestion and absorption - Rattus norvegicus (rat) | http://www.genome.jp/kegg-bin/show\_pathway?rno04977+246046+25702 | 5.953774e-003 | 2 | 35 | 24 | 7182 | 1.000000e+000 | 2.225208e+000 | LMBRD1//PNLIP |
| rno04080 | Neuroactive ligand-receptor interaction - Rattus norvegicus (rat) | http://www.genome.jp/kegg-bin/show\_pathway?rno04080+24953+116744+299757+29597+60399 | 2.050805e-002 | 5 | 35 | 329 | 7182 | 1.000000e+000 | 1.688076e+000 | GCGR//LPAR1//NTS//P2RY2//S1PR5 |
| rno04340 | Hedgehog signaling pathway - Rattus norvegicus (rat) | http://www.genome.jp/kegg-bin/show\_pathway?rno04340+25296+316527 | 2.262112e-002 | 2 | 35 | 48 | 7182 | 1.000000e+000 | 1.645486e+000 | BMP4//WNT10A |
| rno05217 | Basal cell carcinoma - Rattus norvegicus (rat) | http://www.genome.jp/kegg-bin/show\_pathway?rno05217+25296+316527 | 2.721969e-002 | 2 | 35 | 53 | 7182 | 1.000000e+000 | 1.565117e+000 | BMP4//WNT10A |
