# Supplementary material for: Gene Expression in the Hippocampus in a Rat Model of Premenstrual Dysphoric Disorder After Treatment With Baixiangdan Capsules
Source: Front Psychol. 2018 Nov 13;9:2065. doi: 10.3389/fpsyg.2018.02065 (PMC6242977; doi:10.3389/fpsyg.2018.02065)
Supplement: Supplementary file 3 [file Data_Sheet_3.ZIP › Data Analysis Folder/Pathway Analysis Report/fluoxetine vs blank (up)/rno_pathwayResult.html]

| PathwayID | Definition | OriginalWebSite | Fisher-Pvalue | SelectionCounts | SelectionSize | Count | Size | FDR | Enrichment\_Score | Genes |
| --- | --- | --- | --- | --- | --- | --- | --- | --- | --- | --- |
| rno04080 | Neuroactive ligand-receptor interaction - Rattus norvegicus (rat) | http://www.genome.jp/kegg-bin/show\_pathway?rno04080+25083+29422+24318+85268+29595+25187+25635+114115+24797+25033+24806+24807+24808+25570 | 1.784508e-011 | 14 | 31 | 329 | 7182 | 4.621874e-009 | 1.074848e+001 | ADRA2A//CHRNE//DRD2//HRH3//HTR2A//HTR2C//MC4R//P2RX2//SST//SSTR1//TAC1//TACR1//TACR3//TRHR |
| rno04020 | Calcium signaling pathway - Rattus norvegicus (rat) | http://www.genome.jp/kegg-bin/show\_pathway?rno04020+29595+25187+114115+24807+24808+25570 | 1.230444e-004 | 6 | 31 | 187 | 7182 | 1.593425e-002 | 3.909938e+000 | HTR2A//HTR2C//P2RX2//TACR1//TACR3//TRHR |
| rno04540 | Gap junction - Rattus norvegicus (rat) | http://www.genome.jp/kegg-bin/show\_pathway?rno04540+24318+29595+25187 | 6.041460e-003 | 3 | 31 | 87 | 7182 | 5.215794e-001 | 2.218858e+000 | DRD2//HTR2A//HTR2C |
| rno04726 | Serotonergic synapse - Rattus norvegicus (rat) | http://www.genome.jp/kegg-bin/show\_pathway?rno04726+29595+25187+79246 | 1.553809e-002 | 3 | 31 | 123 | 7182 | 8.949025e-001 | 1.808602e+000 | HTR2A//HTR2C//HTR3A |
| rno04360 | Axon guidance - Rattus norvegicus (rat) | http://www.genome.jp/kegg-bin/show\_pathway?rno04360+25186+295382+360272 | 1.727611e-002 | 3 | 31 | 128 | 7182 | 8.949025e-001 | 1.762554e+000 | EFNB1//NTNG1//SLIT2 |
| rno04971 | Gastric acid secretion - Rattus norvegicus (rat) | http://www.genome.jp/kegg-bin/show\_pathway?rno04971+29719+24797 | 3.822308e-002 | 2 | 31 | 72 | 7182 | 1.000000e+000 | 1.417674e+000 | KCNJ16//SST |
