# Supplementary material for: Gene Expression in the Hippocampus in a Rat Model of Premenstrual Dysphoric Disorder After Treatment With Baixiangdan Capsules
Source: Front Psychol. 2018 Nov 13;9:2065. doi: 10.3389/fpsyg.2018.02065 (PMC6242977; doi:10.3389/fpsyg.2018.02065)
Supplement: Supplementary file 3 [file Data_Sheet_3.ZIP › Data Analysis Folder/Pathway Analysis Report/fluoxetine vs model (down)/rno_pathwayResult.html]

| PathwayID | Definition | OriginalWebSite | Fisher-Pvalue | SelectionCounts | SelectionSize | Count | Size | FDR | Enrichment\_Score | Genes |
| --- | --- | --- | --- | --- | --- | --- | --- | --- | --- | --- |
| rno04976 | Bile secretion - Rattus norvegicus (rat) | http://www.genome.jp/kegg-bin/show\_pathway?rno04976+25240+24780+84484+80900 | 9.312176e-004 | 4 | 44 | 72 | 7182 | 2.411854e-001 | 3.030949e+000 | AQP1//SLC4A2//SLC4A4//SLCO1A5 |
| rno04964 | Proximal tubule bicarbonate reclamation - Rattus norvegicus (rat) | http://www.genome.jp/kegg-bin/show\_pathway?rno04964+25240+84484 | 6.498312e-003 | 2 | 44 | 20 | 7182 | 6.492432e-001 | 2.187199e+000 | AQP1//SLC4A4 |
| rno04610 | Complement and coagulation cascades - Rattus norvegicus (rat) | http://www.genome.jp/kegg-bin/show\_pathway?rno04610+24232+306761+304929 | 8.826317e-003 | 3 | 44 | 70 | 7182 | 6.492432e-001 | 2.054220e+000 | C3//F12//F5 |
| rno00030 | Pentose phosphate pathway - Rattus norvegicus (rat) | http://www.genome.jp/kegg-bin/show\_pathway?rno00030+24689+25106 | 1.168351e-002 | 2 | 44 | 27 | 7182 | 6.492432e-001 | 1.932427e+000 | PRPS2//RGN |
| rno00053 | Ascorbate and aldarate metabolism - Rattus norvegicus (rat) | http://www.genome.jp/kegg-bin/show\_pathway?rno00053+25106+113992 | 1.253365e-002 | 2 | 44 | 28 | 7182 | 6.492432e-001 | 1.901922e+000 | RGN//UGT1A6 |
| rno04340 | Hedgehog signaling pathway - Rattus norvegicus (rat) | http://www.genome.jp/kegg-bin/show\_pathway?rno04340+25296+316527 | 3.462852e-002 | 2 | 44 | 48 | 7182 | 1.000000e+000 | 1.460566e+000 | BMP4//WNT10A |
| rno05217 | Basal cell carcinoma - Rattus norvegicus (rat) | http://www.genome.jp/kegg-bin/show\_pathway?rno05217+25296+316527 | 4.150040e-002 | 2 | 44 | 53 | 7182 | 1.000000e+000 | 1.381948e+000 | BMP4//WNT10A |
| rno04360 | Axon guidance - Rattus norvegicus (rat) | http://www.genome.jp/kegg-bin/show\_pathway?rno04360+79208+315564+315711 | 4.311316e-002 | 3 | 44 | 128 | 7182 | 1.000000e+000 | 1.365390e+000 | EPHA5//ROBO3//SEMA7A |
| rno00983 | Drug metabolism - other enzymes - Rattus norvegicus (rat) | http://www.genome.jp/kegg-bin/show\_pathway?rno00983+113902+113992 | 4.883956e-002 | 2 | 44 | 58 | 7182 | 1.000000e+000 | 1.311228e+000 | CES1D//UGT1A6 |
