# Supplementary material for: Gene Expression in the Hippocampus in a Rat Model of Premenstrual Dysphoric Disorder After Treatment With Baixiangdan Capsules
Source: Front Psychol. 2018 Nov 13;9:2065. doi: 10.3389/fpsyg.2018.02065 (PMC6242977; doi:10.3389/fpsyg.2018.02065)
Supplement: Supplementary file 3 [file Data_Sheet_3.ZIP › Data Analysis Folder/Pathway Analysis Report/model vs blank (up)/rno_pathwayResult.html]

| PathwayID | Definition | OriginalWebSite | Fisher-Pvalue | SelectionCounts | SelectionSize | Count | Size | FDR | Enrichment\_Score | Genes |
| --- | --- | --- | --- | --- | --- | --- | --- | --- | --- | --- |
| rno04670 | Leukocyte transendothelial migration - Rattus norvegicus (rat) | http://www.genome.jp/kegg-bin/show\_pathway?rno04670+65129+298487+65130+81686 | 1.900687e-003 | 4 | 33 | 117 | 7182 | 3.668198e-001 | 2.721089e+000 | CLDN1//CLDN19//CLDN3//MMP2 |
| rno04530 | Tight junction - Rattus norvegicus (rat) | http://www.genome.jp/kegg-bin/show\_pathway?rno04530+65129+298487+65130+301112 | 3.116352e-003 | 4 | 33 | 134 | 7182 | 3.668198e-001 | 2.506353e+000 | CLDN1//CLDN19//CLDN3//CRB3 |
| rno04976 | Bile secretion - Rattus norvegicus (rat) | http://www.genome.jp/kegg-bin/show\_pathway?rno04976+25240+24780+80900 | 4.248878e-003 | 3 | 33 | 72 | 7182 | 3.668198e-001 | 2.371726e+000 | AQP1//SLC4A2//SLCO1A5 |
| rno04514 | Cell adhesion molecules (CAMs) - Rattus norvegicus (rat) | http://www.genome.jp/kegg-bin/show\_pathway?rno04514+116777+65129+298487+65130 | 6.538567e-003 | 4 | 33 | 165 | 7182 | 4.233722e-001 | 2.184517e+000 | CDH3//CLDN1//CLDN19//CLDN3 |
| rno05160 | Hepatitis C - Rattus norvegicus (rat) | http://www.genome.jp/kegg-bin/show\_pathway?rno05160+65129+298487+65130 | 1.879833e-002 | 3 | 33 | 124 | 7182 | 9.737534e-001 | 1.725881e+000 | CLDN1//CLDN19//CLDN3 |
